# Divergent ring opening and functionalization of N-alkyl aziridines via boryl radical fragmentation

**DOI:** 10.1038/s41557-026-02247-x

**Published:** 2026-09-23

**Authors:** Pan Peng, Baptiste Roure, Thomas Lulli, Chiara Stavagna, Giovanni Lonardi, Daniele Leonori

**Affiliations:** Institute of Organic Chemistry, RWTH Aachen University, Aachen, Germany

**Keywords:** Photocatalysis, Catalytic mechanisms

## Abstract

Aziridine ring opening is a powerful platform for molecular diversification. Unactivated N-alkyl aziridines have long resisted ring opening and cross-coupling, so the installation and removal of N-activating groups followed by N-alkylation remains the most applicable route for aziridine diversification. Here we report a general radical activation mode that directly engages N-alkyl aziridines in ring-opening and divergent functionalization. Coordination of BH_3_ generates stable aziridine-ligated borane complexes that are selectively converted into aziridine-ligated boryl radicals. These species undergo regioselective β-scission to form β-aminoalkyl radicals that can be intercepted in metal-catalysed cross-coupling reactions. Two complementary platforms were developed: an oxidative copper catalytic system engaging aryl boronic acids, as well as nitrile and sulfide nucleophiles, and a dual photo–nickel catalytic system for cross-coupling with electrophilic aryl bromides. Together, these results establish a general strategy to directly engage N-alkyl aziridines in divergent ring opening and functionalization, thus streamlining access to β-functionalized amines.

**Figure:**
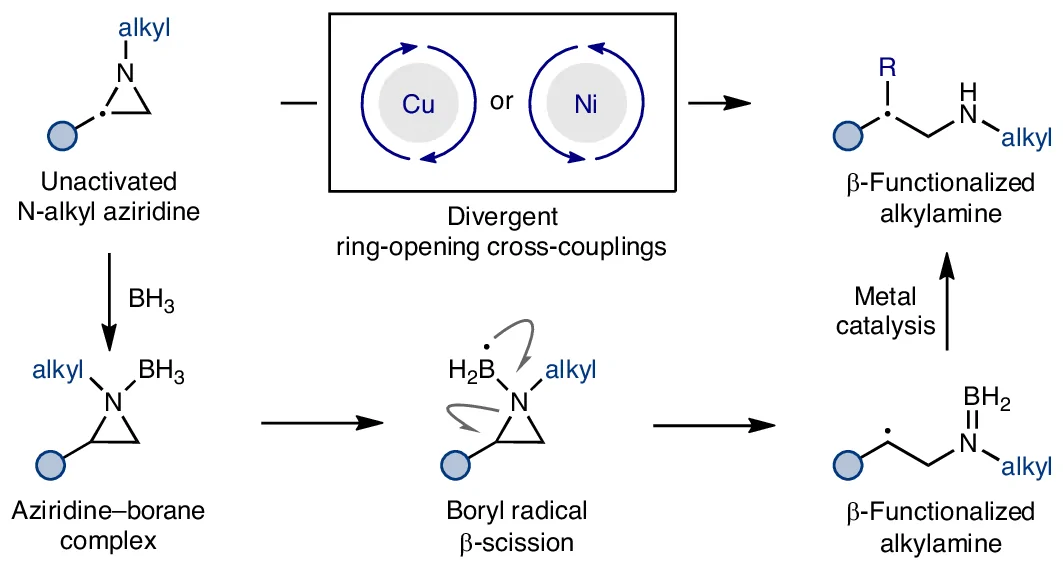

## Abstract (web summary)

Aziridine ring opening is a powerful strategy for molecular diversification, but direct functionalization of unactivated N-alkyl aziridines has remained a long-standing challenge. Now it has been shown that aziridine-ligated boryl radicals enable direct ring opening and divergent functionalization of N-alkyl aziridines, providing streamlined access to diverse β-functionalized amines.

## Main

**Fig. 1:**
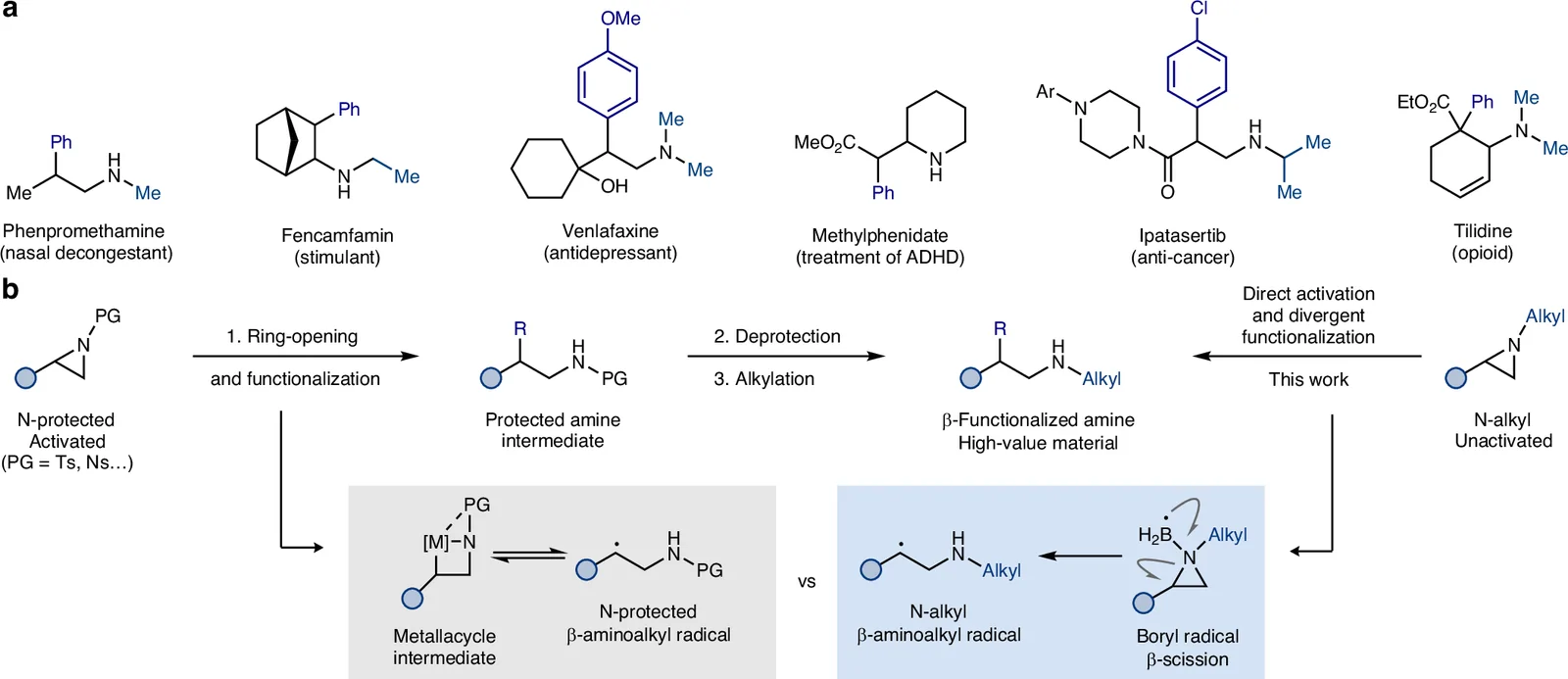
Ring opening of N-alkyl aziridines is a useful approach for the synthesis of high-value molecules with biological activity. **a**, Structures of biologically active β-functionalized N-alkylamines. **b**, Aziridine ring opening and functionalization often require the presence of N-activating groups (for example, Ts) to activate the C(*sp*^3^)–N bonds towards metal insertion or single-electron transfer (SET). However, these groups need to be removed, and further N-alkylation performed. Our work demonstrates the regioselective ring opening of unactivated N-alkyl aziridines via boryl radical β-scission. ADHD, attention deficit hyperactivity disorder.

Aziridines are versatile intermediates in organic synthesis^1–3^. Their ring-opening reactions are particularly powerful when coupled with concomitant functionalization, as they provide access to β-substituted amines, which are structural motifs ubiquitous in bioactive molecules^4,5^ (Fig. 1a). Despite this synthetic potential, aziridine ring opening remains constrained by limitations in substrate activation, nucleophile scope and overall synthetic economy^6,7^.

Direct nucleophilic ring opening is feasible under Lewis or Brønsted acid activation but restricted by the types of nucleophiles that can be engaged and the often forcing conditions required^8,9^. In practice, these transformations are typically limited to highly activated aziridines and strongly nucleophilic partners, with poor generality across different substitution patterns. Furthermore, the nature of the aziridine β-substituent typically dictates the regioselectivity of ring opening. Specifically, benzylic substrates favour cleavage at the more substituted position and lead to branched products, whereas β-alkyl-substituted aziridines preferentially undergo opening at the less substituted position, thus yielding linear products. Methods able to override this intrinsic substrate-controlled reactivity are rare, and this is a fundamental limitation, as it prevents the development of general strategies that deliver a single regioisomer across different substrate classes^10–12^. To overcome these intrinsic limitations, three main catalytic activation strategies based on transition metals have been developed^3^. One approach relies on direct metal insertion into the strained C(*sp*^3^)–N bond to form cyclometallated four-membered intermediates^13^ (Fig. 1b, left). Alternatively, single-electron reduction can generate β-aminoalkyl radicals that either react directly or recombine with the metal centre to access related cyclometallated species^14,15^. A third strategy exploits nucleophilic ring opening with halides (typically, iodide or bromide) to generate β-haloamines that subsequently participate in reductive cross-coupling chemistry^16^. Collectively, these approaches highlight β-aminoalkyl radicals, either as discrete intermediates or as precursors to organometallic species, as the most versatile intermediates for aziridine ring opening and downstream functionalization.

However, all these strategies remain fundamentally dependent on the presence of strongly electron-withdrawing N-activating groups (for example, tosyl or nosyl) to weaken the C(*sp*^3^)–N bond and enable activation (Fig. 1b, left). While effective, these groups must be then removed, and the desired N-substitution constructed, typically via alkylation or reductive amination^17^. A catalytic strategy capable of directly engaging N-alkyl aziridines in ring opening and β-aminoalkyl radical formation, while also overriding their intrinsic substrate-controlled regioselectivity, would expand the reactivity landscape of these strained heterocycles and streamline synthetic access to β-functionalized amines. To date, however, N-alkyl aziridines have largely resisted such activation and remain essentially inert under established transition metal catalytic manifolds, leaving their direct conversion into β-functionalized *N*-alkylamines an unresolved synthetic and mechanistic challenge^18–20^.

In this Article, we establish a radical-based platform for the divergent ring opening and functionalization of unactivated N-alkyl aziridines (Fig. 1b, right). Our strategy exploits in situ Lewis acid–Lewis base coordination between the aziridines and borane (BH_3_) to allow the formation of aziridine-ligated boryl radicals. These species undergo regioselective β-scission across the more substituted C(*sp*^3^)–N bond, bypassing the issues of unselective ring opening under standard Lewis acid activation and unveiling previously inaccessible β-aminoalkyl radicals. By integrating this activation mode into two complementary catalytic manifolds based on copper and nickel catalysis, we enable divergent ring-opening functionalization with both nucleophilic and electrophilic partners from a single mechanistic blueprint. Notably, this activation mode overrides the intrinsic substrate-controlled regioselectivity of aziridine ring opening, enabling selective cleavage of the more substituted C(*sp*^3^)–N bond across both benzylic and alkyl-substituted systems. Overall, this approach eliminates the historical dependence on electron-withdrawing *N*-activating groups and establishes aziridine-ligated boryl radicals as a class of synthetically useful intermediates, delivering streamlined access to β-functionalized N-alkylamines and expanding the reactivity logic of aziridine chemistry.

## Results and discussion

At the outset of this work, we recognized that converting N-alkyl aziridines into β-aminoalkyl radicals could open the door to divergent functionalization strategies. Realizing this concept, however, is non-trivial given the challenging redox properties of these aziridines (Supplementary Figs. 3 and 4). A potential entry point was suggested by Roberts’ pioneering studies on amine-ligated boryl radicals^21–24^. In a series of low-temperature electron paramagnetic resonance (EPR) experiments, Roberts demonstrated that NH-aziridine–BH_3_ complexes participated in H-atom abstraction (HAT) by *t*-BuO• to generate NH-aziridine-ligated boryl radicals. These species rapidly underwent C(*sp*^3^)–N β-scission to give β-boraneylidene-azene radicals^21–24^. Despite this important precedent, this reactivity mode has never been exploited synthetically, and its potential for N-alkyl aziridine ring opening and β-functionalized N-alkylamine preparation remains unexplored.

**Fig. 2:**
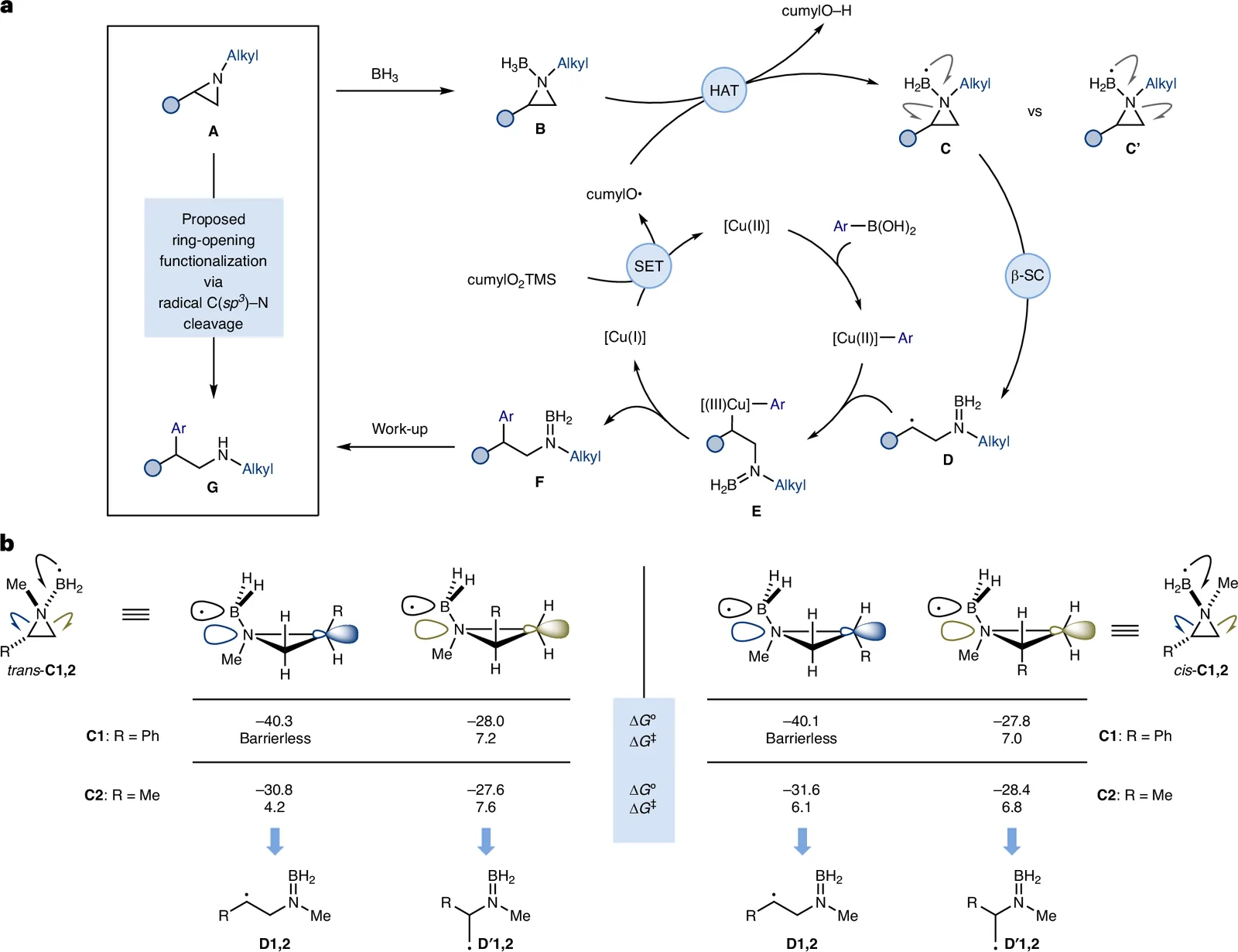
Boryl radical β-scission enables the regioselective ring opening and arylation of N-alkyl aziridines using copper catalysis and aryl boronic acids. **a**, The proposed mechanism for the catalytic β-arylation of N-alkyl aziridines. **b**, A computational evaluation of the regioselectivity of the β-scission ring-opening step ((UM06-2X/def2-QZVP(SMD, EtOAc)//UM06-2X/def2-TVP)). Δ*G*º, Gibbs free energy change of the ring opening, kcal mol^−^^1^; Δ*G*^‡^, Gibbs free energy change at the transition state, kcal mol^−1^.

We therefore hypothesized that amine–boryl radical chemistry could provide a practical activation strategy to achieve general N-alkyl aziridine ring opening and functionalization. Our proposed approach begins with coordination of BH_3_ to N-alkyl aziridine **A**, forming the ligated borane **B**, which we expected to participate in a redox [Cu(I/II/III)] catalytic manifold under oxidative conditions (Fig. 2a). Specifically, ground-state single-electron transfer (SET) between a [Cu(I)] catalyst and cumylO_2_TMS (TMS, trimethylsilyl) should generate the electrophilic cumylO• radical alongside [Cu(II)]^25^. The cumylO• radical would then perform a polarity-matched HAT on **B**^26^, giving the key aziridine-ligated boryl radical **C**, primed for β-scission. Strain release should accelerate this step, affording the β-boraneylidene-azene radical **D**. For productive functionalization, a nucleophile such as an aryl-B(OH)_2_ must transmetalate with the [Cu(II)] species to produce a [Cu(II)–Ar] complex. At this point, radical capture^27^, either through a discrete [Cu(III)] intermediate **E** followed by reductive elimination^28^, or by direct C(*sp*^3^)–C(*sp*^2^) bond formation^29^, would furnish the β-arylated boraneylidene-azene **F**. Finally, hydrolytic work-up would deliver the desired β-functionalized N-alkylamine **G**. The formation of **F** before work-up is highly advantageous, as it deactivates the α-N alkyl positions towards HAT by the electrophilic cumylO• radical^30,31^. This effectively insulates the product from any undesired α-N functionalization that would otherwise compete under the oxidative conditions.

However, a closer analysis of Roberts’ data highlighted a critical issue arising from the stereochemical nature of the aziridine-ligated boryl radical, which can compromise regioselectivity during β-scission^32^. Because the aziridine nitrogen is pyramidalized in complex **B**^33^, two diastereomeric adducts (*trans* and *cis*) may form. Roberts reported that such diastereomeric boryl radicals underwent β-scission selectively but across different C(*sp*^3^)–N bonds, giving either secondary (for example, via **C**) or primary (for example, via **C′**) radicals. From a synthetic standpoint, this could be problematic as uncontrolled BH_3_ coordination could yield diastereomeric mixtures and consequently mixtures of regioisomeric β-functionalized N-alkylamines. Furthermore, if aziridine substitution perturbs the coordination geometry, the predictability and generality of the transformation could be compromised.

To better understand these aspects, before attempting any reaction development, we performed computational studies on the β-scission of representative *trans*- and *cis*-*N*-Me-aziridine-ligated boryl radicals bearing β-Ph (**C1**) and β-methyl (**C2**) substituents (Fig. 2b). In contrast to Roberts’ conclusions, our calculations indicate that, with a β-Ph substituent (**C1**), β-scission should be completely regioselective with both *trans* and *cis* diastereomers, furnishing the stabilized secondary benzylic radical **D1**, as the processes are highly exothermic and essentially barrierless. In the case of β-Me-aziridine-ligated boryl radicals (**C2**), the situation is more nuanced. The *trans* diastereomer is predicted to selectively generate the desired secondary radical **D2**, whereas the *cis* diastereomer might undergo promiscuous β-scission. In this case, the cleavages leading to either a secondary (**D2**) or a primary (**D′2**) radical are both strongly exothermic and proceed with nearly identical activation barriers.

We attribute this divergence to conformational differences between the *trans* and *cis* diastereomers. In the *trans* isomer, favourable orbital overlap between the boryl radical singly occupied molecular orbital (SOMO) and the more substituted C(*sp*^3^)–N σ* orbital weakens that bond and enables significant 1,2-strain release between BH_2_ and the β-Me group, a feature not accessible during cleavage of the less substituted C(*sp*^3^)–N bond. This effect is diminished in the *cis* isomer, where steric repulsion between the β-Me substituent and the N-Me group disfavours differentiation between the two C(*sp*^3^)–N bonds. In the β-Ph series, we propose that the enthalpic preference for forming a stabilized benzylic radical overrides these steric factors entirely. Overall, these insights show that controlling the diastereomer formed upon BH_3_ coordination is essential for β-alkyl aziridines to achieve high efficiency and regioselectivity in the desired functionalization process.

We began by evaluating the feasibility of the N-alkyl aziridine ring opening–arylation sequence using β-aryl-substituted aziridines, as these substrates minimize potential complications associated with β-scission regioselectivity (Fig. 2b). Gratifyingly, we found that BH_3_ coordination proceeded in high yield and furnished the *trans* diastereomer as the exclusive or major isomer in most cases. This complex could be cleanly engaged in ring-opening arylation with aryl boronic acids under oxidative conditions using cumylO_2_TMS as the oxidant and [Cu(CH_3_CN)_4_](PF_6_)/dtbpy (dtbpy, 4,4′-di-tert-butyl-2,2′-bipyridine) as the optimal catalyst–ligand system at 10°C (Table 1). Interestingly, we found a significant solvent effect across the scope of these reactions, and various media were evaluated to maximize the reaction productivity (see footnotes in Table 1).

**Table 1:**
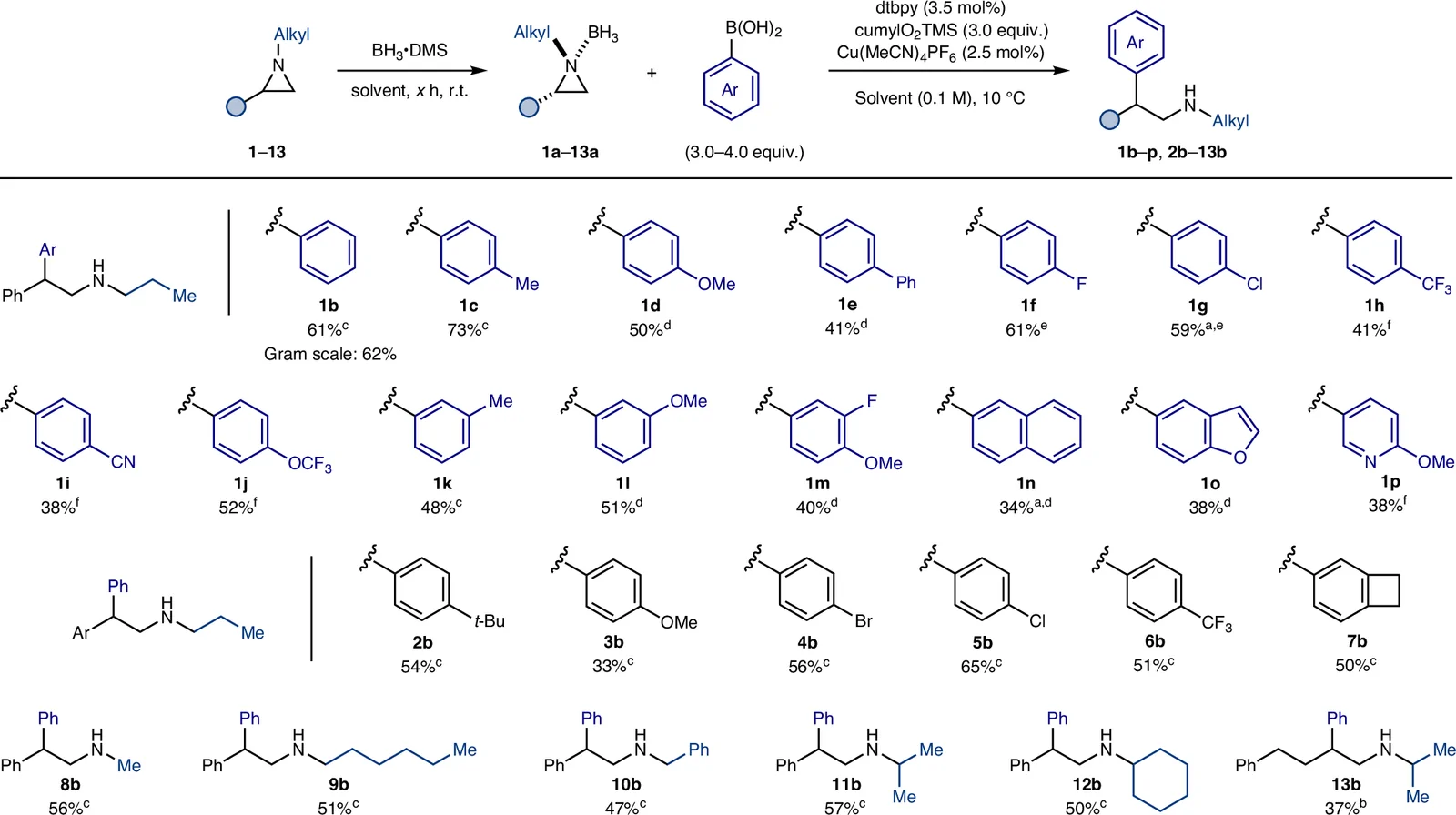
Substrate scope for ring opening–arylation of N-alkyl aziridines

^a^NaHCO_3_ (2.0 equiv.) was added to the reaction.

^b^This reaction was run using Cu(CH_3_CN)_4_PF_6_ (5 mol%), bathophen (6 mol%), cumylO_2_TMS (3.0 equiv.), NaHCO_3_ (2.0 equiv.) and CH_3_CN–C_6_H_5_F (0.1 M, 7:3) at r.t. for 1.5 h.

^c^EtOAc solvent.

^d^EtOAc–acetone (2:1) solvent.

^e^EtOAc–C_6_H_5_F (7:3) solvent.

^f^EtOAc–CH_3_CN (7:3) solvent.

Using *N*-Pr aziridine **1** and Ph–B(OH)_2_ as our initial substrates, we obtained the β-phenyl product **1b** in good yield, including on gram scale. We then explored the scope of compatible aryl boronic acids and observed consistently good reactivity with a series of *para*-substituted derivatives spanning electron-rich (**1c** and **1d**) and electron-poor (**1e**–**j**) functionalities, as well as *meta*-substituted analogues (**1k** and **1l**) and a disubstituted reagent (**1m**). The coupling also tolerated a 2-naphthyl boronic acid (**1n**), as well as electron-rich benzofuran (**1o**) and electron-poor pyridyl (**1p**) boronic acids.

Next, using Ph-B(OH)_2_ as a standard coupling partner, we examined the influence of the β-aryl substituent on the aziridine. Both electron-rich (**2b** and **3b**) and electron-poor (**4b**–**6b**) β-aryl groups were well tolerated, including a cyclobutane-annulated system (**7b**). We then assessed the scope of the N-alkyl substituent and found that the reaction accommodated N-Me (**8b**), N-hexyl (**9b**) and HAT-activated Bn (**10b**) groups. Importantly, secondary alkyl groups such as *i*-Pr (**11b**) and cyclohexyl (**12b**) were also compatible.

Having established the reactivity profile of β-aryl N-alkyl aziridines, we next turned our attention to β-alkyl analogues, which represent a significantly more demanding and mechanistically distinct class of substrates. In general, β-alkyl aziridine ring-opening reactions typically proceed with opposite regioselectivity, favouring cleavage at the less substituted C(*sp*^3^)–N bond and leading to linear products^10–12^. Using **13** as the model aziridine, BH_3_ coordination occurred selectively to form the *trans* diastereomer **13a**, thereby mitigating concerns regarding β-scission regioselectivity. Although the desired β-arylated product **13b** was obtained selectively, conversions were lower. At present, β-alkyl N-alkyl aziridines participate less efficiently in this copper-catalysed ring-opening cross-coupling. However, as discussed below, we have identified a complementary strategy that enables the efficient synthesis of this valuable class of products in substantially higher yields. Despite the reduced efficiency, the preservation of regioselectivity across both β-aryl and β-alkyl substrates suggests that this platform can promote a consistent selectivity outcome across different substrate classes.

A synthetically valuable aspect of the redox [Cu] catalytic manifold discussed above is its potential for divergence. Specifically, by employing different nucleophiles, it should be possible to access a variety of ring-opening functionalization processes beyond arylation. A key challenge in this context is that each nucleophile may interact differently with the Cu catalyst, thereby altering its binding efficiency as well as the electronic properties of the intermediated species that can in turn affect the efficiency of the individual steps in the catalytic cycle. To explore this possibility, we evaluated a series of nucleophiles using aziridine **1** under the previously optimized arylation conditions (Table 2). Gratifyingly, TMS–CN afforded the corresponding β-nitrile amine **1q** in 65% yield (48% on gram scale). This result is noteworthy as the nitrile can be readily hydrolysed to deliver valuable β-amino acids. High reactivity was also observed with (PhS)_2_, which furnished the β-thioether derivative **1r** in 75% yield. Additional nucleophiles were examined, and although the desired products were obtained in lower yields, successful proof-of-concept was demonstrated for β-azidation, and β-phthalimidooxylation (Supplementary Fig. 1). These preliminary results indicate that additional reactivity might be identified but that dedicated optimization campaigns that are beyond the scope of the present work will probably be required to enhance the efficiency of these ring-opening functionalizations.

**Table 2:**
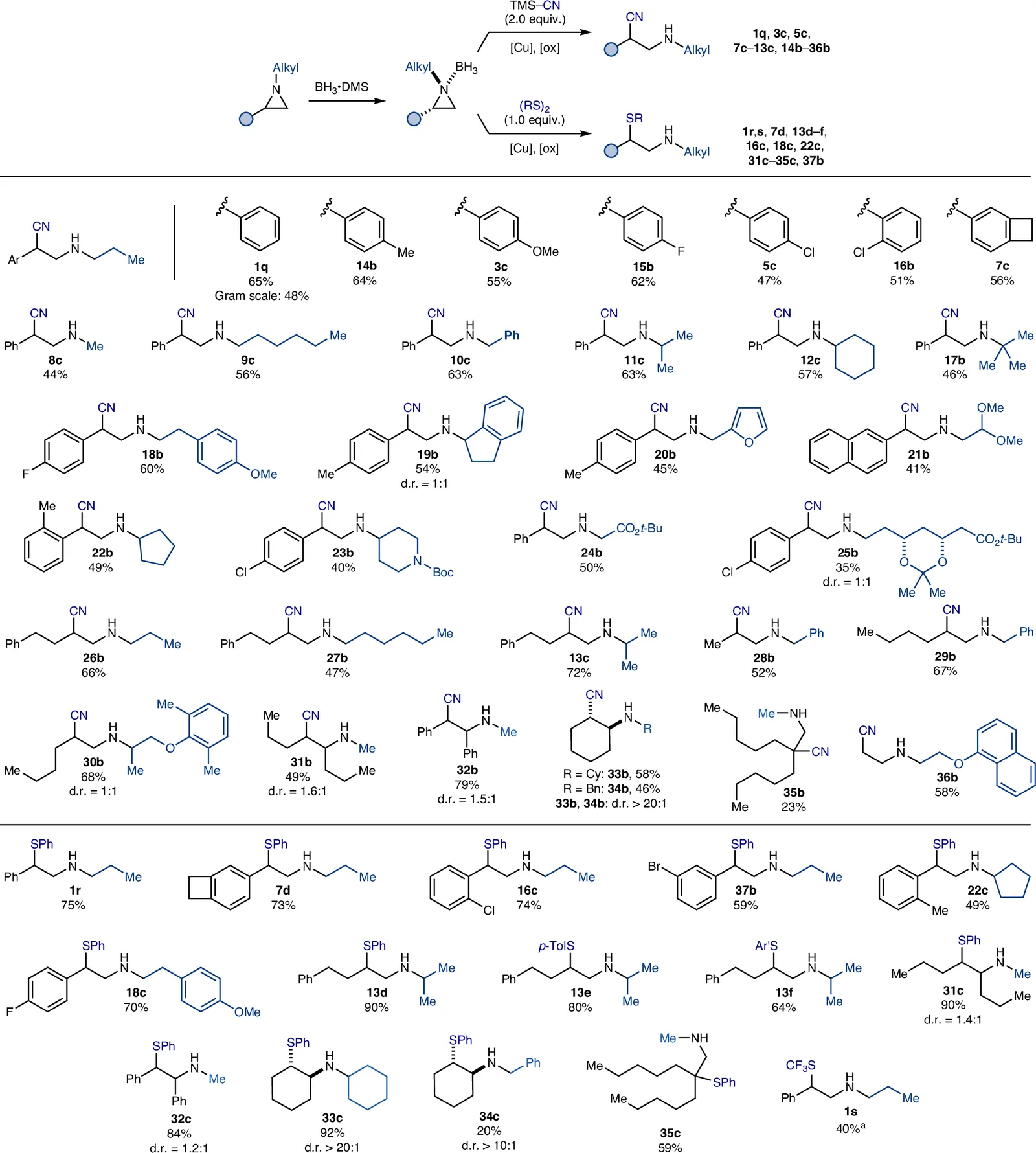
Scope for N-alkyl aziridine ring-opening cyanation and thioetherification

General reaction conditions for cyanation: aziridine–borane (1.0 equiv.), TMS–CN (2.0 equiv.), Cu(CH_3_CN)_4_PF_6_ (2.5 mol%), dtbpy (3.5 mol%), cumylO_2_TMS (3.0 equiv.) and CH_3_CN (0.1 M), r.t., 1-4.2 h. General reaction conditions for thioetherification: (RS)_2_ (1.0 equiv.), aziridine–borane (2.0 equiv.), Cu(CH_3_CN)_4_PF_6_ (2.5 mol%), dtbpy (3.5 mol%), cumylO_2_TMS (3.0 equiv.) and PhCl (0.1 M), r.t., 2 h. Ar′ = *p*-NO_2_-C_6_H_4_. d.r., diastereomeric ratio.

^a^AgSCF_3_ was used instead of (RS)_2_.

We next evaluated the generality of the ring-opening cyanation and thioetherification processes. The cyanation proved broadly compatible with a wide range of aziridine derivatives. Varying the β-aryl substituent, we observed good reactivity for electron-rich (**14b** and **3c**) and electron-poor (**15b** and **5c**) *para*-substituted systems. The transformation was also successful with an aziridine bearing an *ortho*-chloro group (**16b**) and a cyclobutane-annulated aromatic unit (**7c**). Turning to the N-substituent, the reaction tolerated a diverse set of moieties, including Me (**8c**), Hex (**9c**), Bn (**10c**), *i*-Pr (**11c**), cyclohexyl (**12c**) and the sterically hindered *t*-Bu (**17b**). Additional substrates containing HAT-labile C(*sp*^3^)–H bonds, such as benzylic (**18b**–**20b**) and α-oxy (**21b**) positions, also performed well, indicating that potential undesired abstractions by cumylO• do not compete significantly^34,35^. Secondary N-alkyl groups such as cyclopentyl could also be present in the case of a β-*ortho*-functionalized aromatic (**22b**). More complex motifs were also compatible, including a 4-*N*-Boc-piperidinyl substituent of medicinal relevance (**23b**), a glycine derivative (**24b**) and an atorvastatin intermediate (**25b**), all of which afforded the corresponding β-cyano amines in moderate to good yields. Importantly, this ring-opening–cyanation reactivity also extended to aziridines that generate non-benzylic alkyl radicals. Substrates with varied *N*-substituents and different alkyl chains delivered the desired products (**26b**–**36b** and **13c**) in good yields. Notably, successful applications included aziridine based on the antiarrhythmic drug mexiletine (**30b**), several disubstituted derivatives over acyclic and cyclic scaffolds and a *gem*-disubstituted system enabling access to α,β-difunctionalized amines (**31b**–**34b**) and a tertiary nitrile (**35b**). Furthermore, the direct cleavage of an unsubstituted aziridine via a primary radical intermediate was also demonstrated feasible (**36b**). Importantly, all substrates examined delivered the branched products exclusively, further demonstrating that this activation mode consistently overrides the inherent regioselectivity bias of classical aziridine ring opening.

In terms of the thioetherification process, we also demonstrated feasibility with a broad range of derivatives, enabling functionalization at both benzylic and alkyl positions in generally good yield (**1r**, **7d**, **13d**–**f**, **16c**, **18c**, **22c**, **31c**–**35c** and **37b**). At present the reactivity is high yielding for the use of (ArS)_2_ reagents, but we obtained a proof of principle where the use of silver(I) trifluoromethanethiolate (AgSCF_3_) enabled efficient ring opening and trifluoromethylthiolation (**1s**).

As outlined above, we sought to broaden the utility of aziridine-ligated boryl-radical β-scission as a general blueprint for generating β-aminoalkyl radicals en route to β-arylated N-alkylamines. To push this concept further, we examined whether the key reactivity steps, that is, HAT activation of the ligated borane by an electrophilic O-centred radical followed by boryl radical β-scission, could be embedded within a dual photo–nickel catalytic manifold^36^. In this design, diaryl ketones (**DK**) would serve as HAT photocatalysts, thereby enabling cross-coupling with aryl bromides, which are a widely accessible and versatile class of coupling partners. This strategy would constitute an integration of boryl-radical β-scission into both photocatalysis and nickel catalysis, and by engaging aryl bromides rather than nucleophilic aryl boron reagents, it would represent an umpolung variant of our earlier copper-catalysed arylation using aryl boronic acids.

**Fig. 3:**
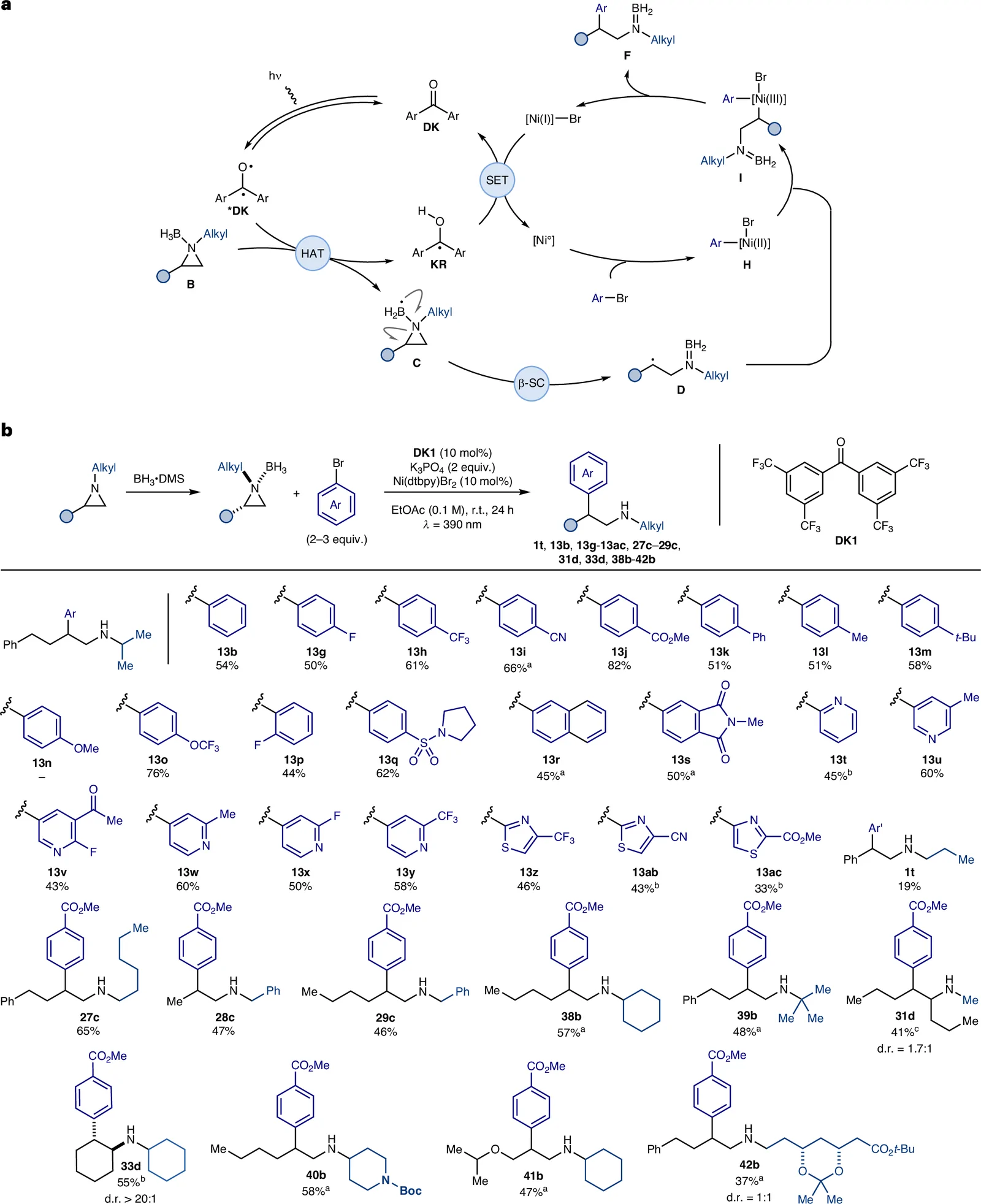
The boryl radical β-scission can be integrated with dual photocatalysis with diaryl ketones and nickel to enable ring opening and arylation with aryl bromides. **a**, The proposed mechanism for the dual catalytic approach enabling N-alkyl aziridine ring opening and arylation with aryl bromides. **b**, Substrate scope. Ar′ = *p*-CO_2_Me-C_6_H_4_. ^a^Yield of two steps from aziridine. ^b^**DK1** (20 mol%). ^c^**DK1** (20 mol%), 16 h. SC, scission; DMS, dimethyl sulfide; d.r., diastereomeric ratio.

Mechanistically, this approach requires a photocatalytic cycle in which triplet-excited diaryl ketone (*DK) abstracts a H-atom from complex **B** to generate the boryl radical **C**^37^ (Fig. 3a). Subsequent β-scission would provide the alkyl radical **D**, which is intercepted by a [Ni(II)]–Ar complex **H** formed via oxidative addition of Ar–Br to Ni(0). Radical capture would afford the alkyl–[Ni(III)]–Ar intermediate **I**, from which reductive elimination delivers the cross-coupled product **F** (after work-up). The resulting [Ni(I)] species regenerates [Ni(0)] through SET with the ketyl radical (**KR**) originating from the initial HAT step, closing both catalytic cycles.

We initiated optimization using **13** as the N-alkyl aziridine. Under irradiation with purple light-emitting diodes (LEDs), employing **DK1** as the diaryl ketone, Ni(dtbpy)Br_2_ as the nickel catalyst and K_3_PO_4_ as the base, we obtained the desired product **13b** in improved, 54% yield (Fig. 3b). With these conditions in hand, the method proved broadly effective with a panel of aryl bromides, including *para*-F (**13g**), –CF_3_ (**13h**), –CN (**13i**), –ester (**13j**) and –Ph (**13k**), as well as moderately electron-rich substrates such as –Me (**13l**) and –*t*-Bu (**13m**). In contrast, strongly electron-donating substituents (for example, –OMe) were not compatible, whereas oxygen-based groups could be accommodated when a sufficiently electron-withdrawing group was incorporated or introduced (for example, –OCF_3_, **13o**). The protocol also tolerated an *ortho*-F (**13p**) and a *para*-N-sulfonamide substituent (**13q**) and extended smoothly to polyaromatic systems such as 2-naphthyl (**13r**) and 2-methyl-isoindolinone (**13s**). Notably, the dual manifold enabled efficient coupling with heteroaryl bromides, including 2-pyridyl (**13t**), 3-pyridyl (**13u** and **13v**), 4-pyridyl (**13w**–**y**) and electron-deficient thiazole systems (**13z**–**ac**). As observed in the copper-catalysed system, the nickel manifold also enforces selective cleavage at the more substituted position, reinforcing the generality of this regioselectivity across distinct catalytic regimes.

A key limitation emerged with β-aryl-substituted aziridines, which underwent productive coupling but with low efficiency (for example, **1t**). We attribute this to the increased stabilization of the resulting benzylic radicals, which probably slows their engagement with the [Ni(II)] species and promotes unproductive side pathways. This limitation underscores the complementarity of our two strategies based on either oxidative copper catalysis or dual photo–nickel catalysis. Although both approaches deliver the same family of β-functionalized amines, their effectiveness depends critically on the nature of the intermediate radical (benzylic versus alkyl) and the type of coupling partner (nucleophiles, Ar-B(OH)_2_ versus electrophiles, Ar–Br).

Further scope exploration demonstrated that the method tolerates a range of N-substituents and alkyl chains varying in length and steric demand (**27c**, **28c**, **29c**, **31d**, **33d**, **38b**, **39b**, **40b**, **41** and **42b**), including substrates bearing N-Boc-piperidine (**40b**) as well as ether and acetal functionalities (**41b** and **42b**).

## Conclusion

Aziridines are versatile synthetic intermediates, and their ring-opening functionalization provides direct access to valuable β-substituted amines. Traditionally, such transformations have relied on the use of strongly electron-withdrawing N-substituents to enable C(*sp*^3^)–N bond activation. The work presented here addresses this limitation by demonstrating that simple borane coordination can be used for N-alkyl aziridine activation. The resulting aziridine-ligated boryl radicals undergo regioselective β-scission to generate the most substituted β-aminoalkyl radicals, which can be engaged in divergent copper and nickel-catalysed cross-couplings with both nucleophilic and electrophilic partners. Notably, this approach allows access to consistent regioselectivity across both β-aryl and β-alkyl substrates, mitigating the substrate-dependent behaviour typically observed in aziridine ring opening. We anticipate that the generality of this activation mode and the scope of the functionalizations developed will facilitate the synthesis of biologically relevant molecules and inspire broader exploration of amine-ligated boryl radicals in synthesis.

## Online content

Any methods, additional references, Nature Portfolio reporting summaries, source data, extended data, supplementary information, acknowledgements, peer review information; details of author contributions and competing interests; and statements of data and code availability are available at https://doi.org/10.1038/s41557-026-02247-x.

## Supplementary information

Supplementary Information Supplementary Discussion, Figs. S1–S18, Tables S1–S26, synthetic procedures, characterization of compounds, copies of NMR spectra, computational analysis and mechanistic studies.

Supplementary Data 1 The atomic coordinates of optimized structures.

## Data Availability

The authors declare that the data supporting the findings of this study are available within the paper and its Supplementary Information.
