# Supplementary material for: Divergent ring opening and functionalization of N-alkyl aziridines via boryl radical fragmentation
Source: Nat Chem. 2026 Sep 23;18(10):1755–62. doi: 10.1038/s41557-026-02247-x (PMC13623657; doi:10.1038/s41557-026-02247-x)
Supplement: Supplementary file 1 [file 41557_2026_2247_MOESM1_ESM.pdf]

nature chemistry

Supplementary information https://doi.org/10.1038/s41557-026-02247-x

Divergent ring opening and functionalization of N-alkyl aziridines via boryl radical fragmentation

In the format provided by the authors and unedited

Table of content

1 General Experimental Details ... 3

2 General Procedures ... 4

3 Starting Material Synthesis ... 10

3.1 Peroxides ... 10

3.2 Sulfonium Bromide Salts ... 11

3.3 Aziridines ... 15

3.4 Aziridine–Borane Complexes ... 30

4 Reaction Optimization ... 61

4.1 Optimization of the Arylation with Ph–B$(OH)_2$ ... 61

4.2 Control Reactions ... 70

4.3 Optimization of the Arylation with Electron-Deficient Boronic Acids ... 71

4.4 Optimization of the Arylation with Electron-Rich Boronic Acids ... 72

4.5 Optimization of the Cyanation with TMSCN ... 73

4.6 Control Reactions for Cyanation and Thioetherification ... 76

4.7 Optimization of Photo–Nickel Dual Catalysis ... 77

5 Substrate scope ... 81

5.1 Ring-Opening–Arylation ... 81

5.2 Ring-Opening–Cyanation ... 90

5.3 Ring-Opening–Thioetherification ... 106

5.4 Ring-Opening–Arylation via Dual Photo–Ni-Catalysis ... 117

6 Scope of Other Nucleophiles ... 134

7 Comparison of Arylation of *cis* and *trans* Aziridine Boranes ... 135

8 Scale-up Experiments ... 136

9 Cyclic Voltammetry Studies ... 137

10 Laser Flash Photolysis Studies ... 140

11 Radical Trapping Experiment ... 142

12 Computational Studies ... 143

12.1 General Information ... 143

12.2 Thermodynamics ... 144

12.3 Chemical Properties Across the Potential Energy Surface ... 146

12.4 Visual Representation of Orbital Overlap at the Transition State 148

13 Literature Review of Ring-Opening Reactions of *N*-alkyl aziridines 150

13.1 Background 150

13.2 Nucleophilic Ring Opening 150

13.3 Metal-Catalysed Ring Opening 153

14 NMR Spectra 155

15 References 406

1 General Experimental Details

All required fine chemicals were used directly without purification unless stated otherwise. All air and moisture sensitive reactions were carried out under Argon atmosphere using standard Schlenk manifold technique. All solvents were bought from Acros as 99.8% purity and degassed by Ar bubbling. ^{1}H and ^{13}C Nuclear Magnetic Resonance (NMR) spectra were acquired at various field strengths as indicated and were referenced to $CHCl_3$ (7.26 and 77.2 ppm for ^{1}H and ^{13}C respectively) or DMSO (2.50 and 39.5 ppm for ^{1}H and ^{13}C respectively). ^{1}H NMR coupling constants are reported in Hertz and refer to apparent multiplicities and not true coupling constants. Data are reported as follows: chemical shift, integration, multiplicity (s = singlet, br s = broad singlet, d = doublet, t = triplet, q = quartet, p = pentet, sx = sextet, hp = heptet, n = nonet, m = multiplet, dd = doublet of doublets, etc.), proton and carbon assignment (determined by 2D NOESY NMR experiments) where possible. ^{11}B NMR and ^{19}F NMR spectra were recorded and reported unreferenced. High-resolution mass spectra were obtained using a JEOL JMS-700 spectrometer or a Fissions VG Trio 2000 quadrupole mass spectrometer. Spectra were obtained using electron impact ionization (EI) and chemical ionization (CI) techniques, or positive electrospray (ESI). Analytical TLC: aluminum backed plates pre-coated (0.25 mm) with Merck Silica Gel 60 F254. Compounds were visualized by exposure to UV-light or by dipping the plates in ninhydrin stain followed by heating. Flash column chromatography was performed using Merck Silica Gel 60 (40–63 µm). All mixed solvent eluents are reported as v/v solutions. All the reactions were conducted in CEM 9 mL glass microwave tubes capped with a LABSOLUTE crimp seal with septum (PTFE/butyl) purchased from Th. Geyer. The LEDs used are Kessil PR160L-390 nm.

2 General Procedures

General Procedure for the Preparation of the Sulfonium Bromides – GP1a[1]

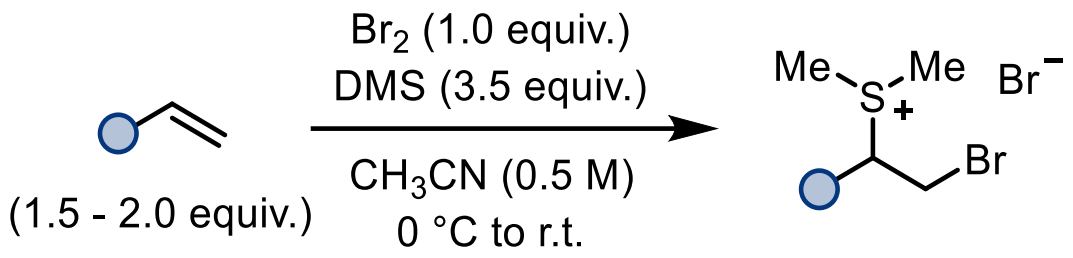

To a round bottom flask equipped with a stirring bar was added Br_2 (1 equiv.) and CH_3CN (0.5 M). The resulting mixture was cooled down at 0 °C with an ice-water bath and dimethyl sulfide (3.5 equiv.) was added dropwise over 5 min via syringe, leading to the formation of a yellow precipitate. The reaction mixture was stirred for 10 min before adding the corresponding alkene (1.5 or 2.0 equiv.) dropwise over 5 min via syringe. The reaction mixture was then warmed up to room temperature generating a homogeneous solution, followed by the precipitation of the corresponding sulfonium bromide. The reaction mixture was stirred for 30 min before being diluted with Et_2O to ensure full precipitation. The solid precipitate was collected by filtration and washed with Et_2O (x 2) to give the pure sulfonium bromides as solids that were used without any further purification.

General Procedure for the Preparation of the Sulfonium Bromides – GP1b[2]

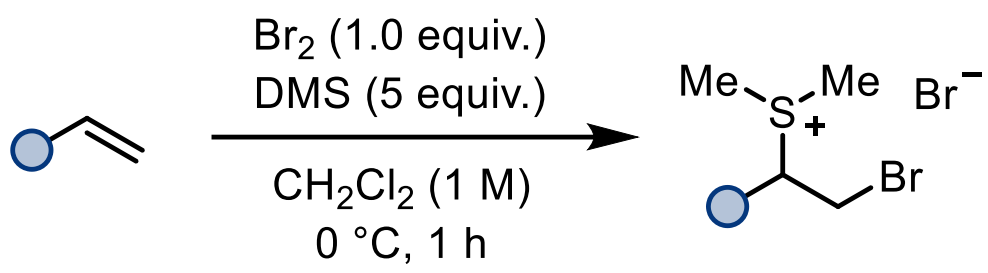

To a round bottom flask equipped with a stirring bar was added the corresponding alkene (1.0 equiv.), dimethyl sulfide (5 equiv.) and CH_2Cl_2 (1 M). The resulting mixture was cooled down at 0 °C with an ice-water bath and a solution of bromine (Br_2, 1.0 equiv.) in CH_2Cl_2 (4 M) was slowly added via syringe. The mixture was stirred for 1 h at 0 °C. A fading out of the reaction mixture was observed and a solid precipitated, which was collected by filtration and washed with Et_2O (x 2) to give the pure sulfonium bromides as solids that were used without any further purification.

Note: The stability of the sulfonium bromides greatly depends on the substituents. Some decomposes readily under air and need to be used quickly or stored under inert atmosphere in the fridge or the freezer. Specifically, the sulfonium bromides containing fluoro and/or ortho substituents decompose under air. The sulfonium bromides can be analysed by NMR but they decompose relatively fast (overnight) in D_2O or DMSO.

General Procedure for the Preparation of the *N*-Alkyl Aryl Aziridines – GP2[3]

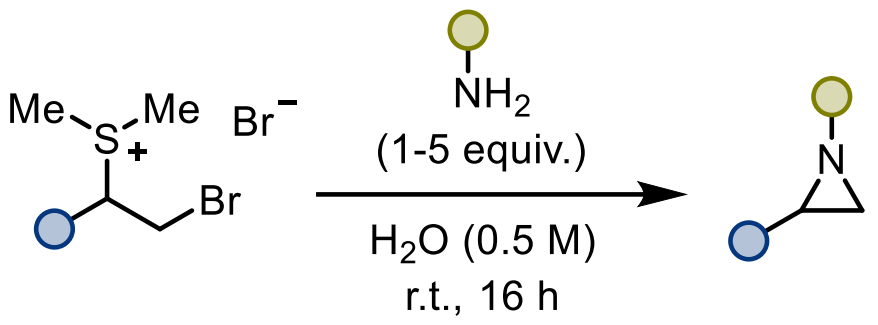

A round bottom flask equipped with a stirring bar was charged with the corresponding sulfonium bromide (1.0 equiv.), H_2O (0.5 M) and the corresponding amine (1-5 equiv.) were added, and the reaction mixture was stirred for 16 h at room temperature. After complete consumption of starting material, the aqueous phase was extracted with CH_2Cl_2 (x 3). The combined organic layers were dried ($MgSO_4$) and filtered. Pure *N*-alkyl aziridines were directly obtained by evaporating the solvent and the excess of amine, or via column chromatography on silica gel.

Note: If the excess amine is not volatile, it can be removed by aqueous workup or purified by column chromatography on silica gel.

General Procedure for the Preparation of the *N*-Alkyl Alkyl Aziridines – GP3a[4]

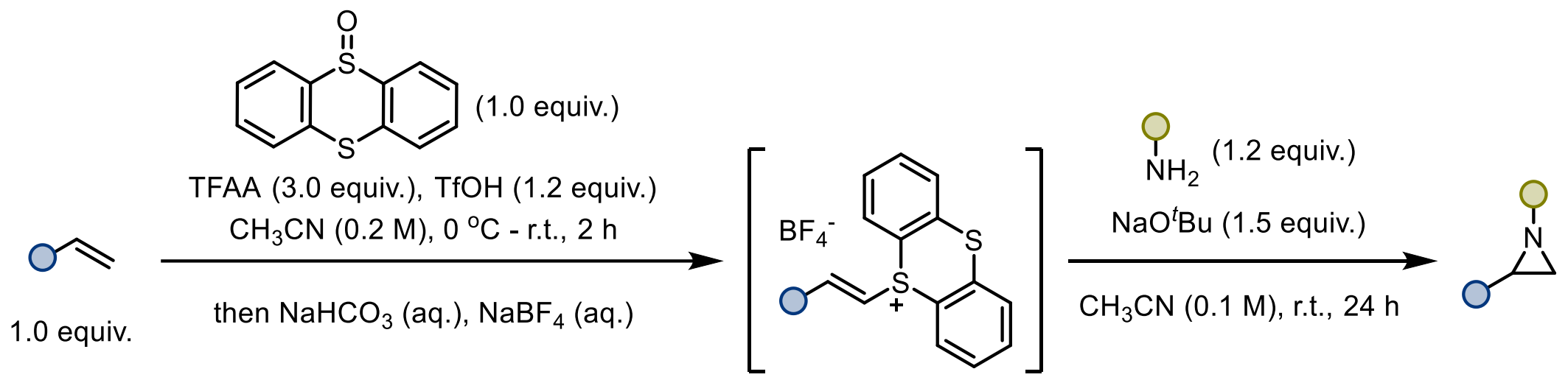

Step 1: A Schlenk tube equipped with a magnetic stir bar was charged with the corresponding alkene (1.0 equiv.), thianthrene 5-oxide (1.0 equiv.), and dry CH_3CN (0.2 M). The reaction mixture was cooled down to 0 °C and TFAA (3.0 equiv.) was added dropwise over 2 minutes via syringe, followed by dropwise addition of TfOH (1.2 equiv.) over 2 minutes via syringe. After stirring the lilac mixture at 0 °C for 1 h and then 1 h at room temperature, the mixture was concentrated under reduced pressure, diluted with CH_2Cl_2, and washed with saturated aqueous $NaHCO_3$ solution, aqueous $NaBF_4$ solution (x 3, 5 % w/w). The organic layer was dried over $MgSO_4$, filtered, and the solvent was removed under reduced pressure. The crude thianthrene was directly used for the next step without further purification.

Step 2: To a round bottom flask equipped with a stirring bar was added the corresponding amine (1.2 equiv.), NaO*t*-Bu (1.5 equiv.), thianthrene (1.0 equiv.) and dry CH_3CN (0.1 M). The reaction mixture was stirred for 24 h at room temperature. After complete consumption of starting material, the reaction mixture was evaporated, and the residue was purified directly by column chromatography on silica gel to afford the product.

General Procedure for the Preparation of the *N*-Alkyl Alkyl Aziridines – GP3b[5,6]

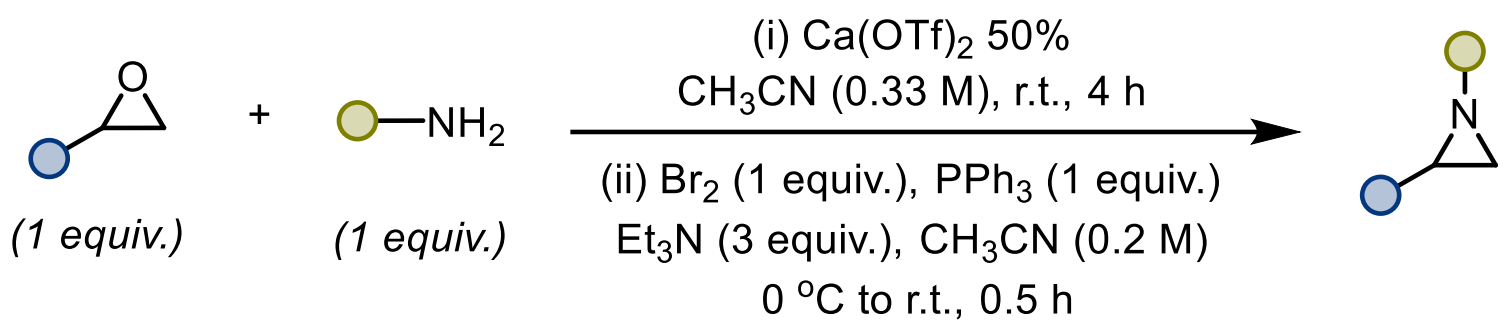

Step 1: A round bottom flask equipped with a stirring bar was charged with the corresponding amine (1.0 equiv.), CH_3CN (0.33 M), $Ca(OTf)_2$ (50 mol%) and the epoxide (1.0 equiv.) in sequence. The resulting mixture was stirred for 4 h at room temperature. Upon completion, water was added, the aqueous layer was extracted and separated with CH_2Cl_2 (x 3). The organic layer was dried ($MgSO_4$), filtered, and the solvent was removed under reduced pressure. The crude amino alcohol was directly used in the next step without further purification.

Step 2: Br_2 (1.0 equiv.) was added dropwise to an ice-cold solution of PPh_3 (1.0 equiv.) in dry CH_3CN (0.2 M). To this mixture was slowly added the crude amino alcohol (1.0 equiv., the amount depending on first step), followed by the dropwise addition of Et_3N (3.0 equiv.) at 0 ºC. The mixture was then stirred at room temperature for 30 min. Et_3N•HBr was filtered off and the solution was concentrated by rotary evaporation and the residue was purified directly by column chromatography on silica gel to give desired aziridine.

General Procedure for the Preparation of the *N*-Alkyl Aziridine Boranes – GP4

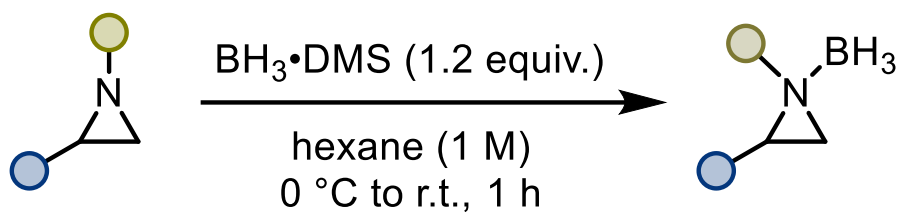

A round bottom flask equipped with a stirring bar was charged with the corresponding alkyl aziridine (1.0 equiv.). *n*-Hexane (1 M) was added, and the reaction mixture was cooled down to 0 °C with an ice bath and BH_3•DMS (1.2 equiv.) was slowly added via syringe. The resulting mixture was slowly warmed up to room temperature and stirred for 1 h at this temperature (a precipitate formed over the course of the reaction). The precipitate was collected by filtration and washed with *n*-hexane (x 2) to give the pure alkyl aziridine boranes as solids that were used without any further purification. If there are no precipitate, the amine boranes were isolated by column chromatography on silica gel.

<u>Note</u>: The filtration is enough in most cases to obtain the pure alkyl aziridine boranes. However, it can also be purified by column chromatography on silica gel or triturated with a mixture of Et_2O/pentane (50% to 100%).

The diasteroselectivity of the BH_3 coordination is discussed in **Section 3.4**.

General Procedure for the Ring Opening-Arylation of Aziridine Boranes – GP5

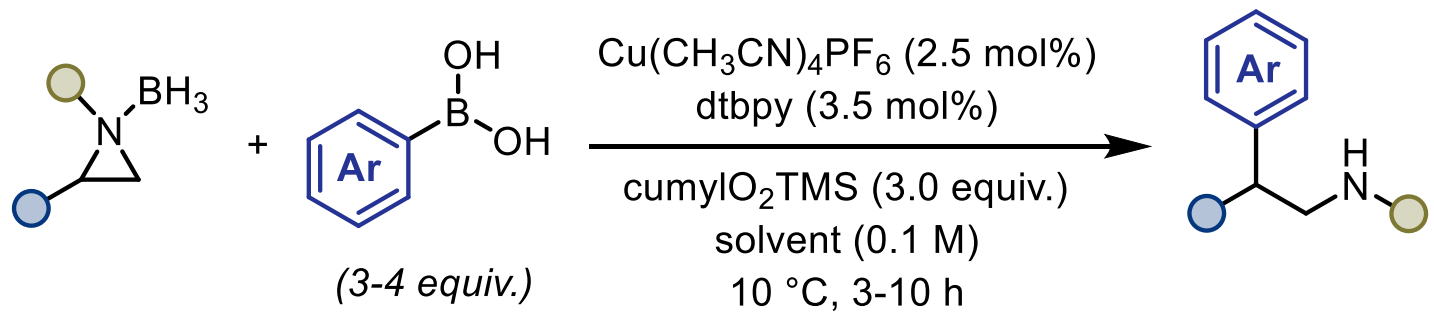

An oven-dried vial equipped with a stirring bar was charged with the corresponding aziridine borane (0.1 mmol, 1.0 equiv.) and the corresponding boronic acid (see **Table S1** for the equivalents). The tube was capped with a Supelco aluminium crimp seal with septum (PTFE/butyl), evacuated and refilled with Ar (x 3). The peroxide (0.3 mmol, 3.0 equiv.) and dry and degassed solvent (0.5 mL) was then added to the vial and stirred for around **1 min**. The resulting mixture was cooled down to 10 °C (chiller) and the catalyst solution (0.5 mL of the corresponding dry and degassed solvent, containing $Cu(CH_3CN)_4PF_6$ (1 mg, 2.5 mol%) and dtbpy (1 mg, 3.5 mol%), pre-stirred for 10 min until fully dissolved) was then added under stirring. The resulting mixture was stirred at 10 ºC for 10 h. Upon completion, aqueous NaOH (2 mL, 1 M) was added, the aqueous layer was separated and extracted with EtOAc (2 x 3 mL). The combined organic layers were dried ($MgSO_4$) and filtered. The solvent was evaporated and the residue was purified by column chromatography on silica gel to afford the desired products.

Table S1 shows the three conditions that were predominantly used during the Ring Opening-Arylation reactions.

Table S1. Variations used for Ring Opening-Arylation of Aziridine Boranes

General Procedure	solvent	$ArB(OH)_2$ equiv.
**GP5a**	EtOAc	3.0
**GP5b**	EtOAc:acetone (2:1)	4.0
**GP5c**	EtOAc:CH_3CN (7:3)	4.0
**GP5d**	EtOAc:PhF (7:3)	4.0

General Procedure for the Ring Opening-Cyanation of Aziridine Boranes – GP6

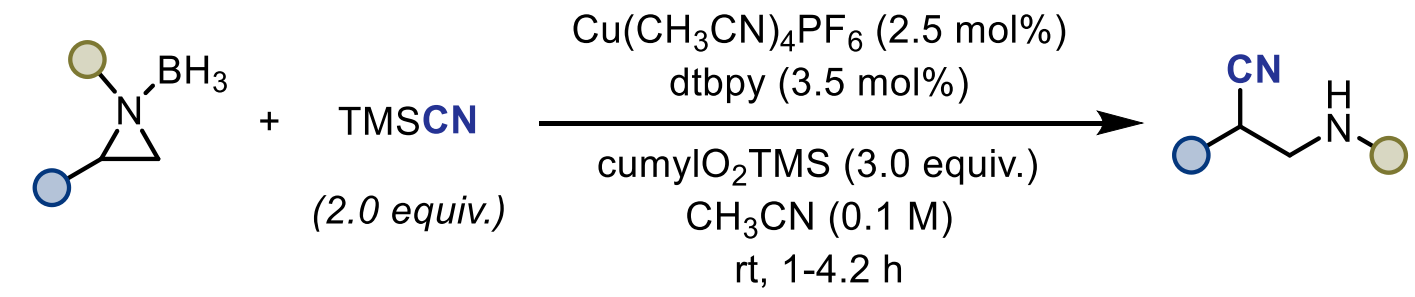

An oven-dried vial equipped with a stirring bar was charged with the corresponding aziridine borane (0.1 mmol, 1.0 equiv.). The tube was capped with a Supelco aluminium crimp seal with septum (PTFE/butyl), evacuated and refilled with Ar (x 3). The peroxide (0.3 mmol, 3.0 equiv.), TMSCN (0.2 mmol, 2.0 equiv.) and dry and degassed CH_3CN (0.5 mL) was then added to the

vial and stirred for around **1 min**. The catalyst solution (0.5 mL of dry and degassed CH_3CN, containing $Cu(CH_3CN)_4PF_6$ (1 mg, 2.5 mol%) and dtbpy (1 mg, 3.5 mol%), pre-stirred for 10 min until fully dissolved) was then added under stirring. The resulting mixture was stirred at room temperature for 2 h. Upon completion, an aqueous solution of NaOH (2 mL, 1 M) was added, the aqueous layer was separated and extracted with EtOAc (2 x 3 mL). The combined organic layers were dried ($MgSO_4$) and filtered. The solvent was evaporated, and the residue was purified by column chromatography on silica gel to afford the desired products.

General Procedure for the Ring Opening-Thiolation of Aziridine Boranes– GP7

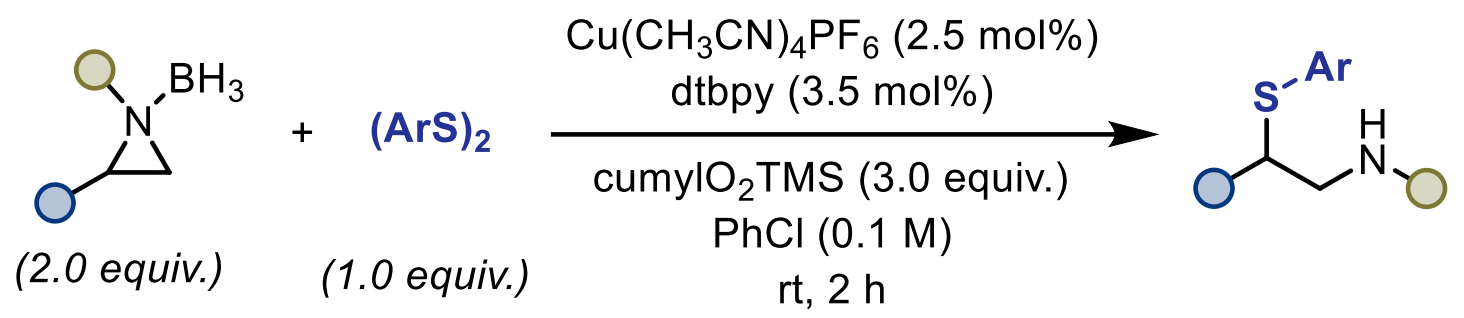

An oven-dried vial equipped with a stirring bar was charged with the corresponding aziridine borane (0.2 mmol, 1.0 equiv.) and diaryl disulfide ($(ArS)_2$, 0.1 mmol, 1.0 equiv.). The tube was capped with a Supelco aluminium crimp seal with septum (PTFE/butyl), evacuated and refilled with Ar (x 3). The peroxide (0.3 mmol, 3.0 equiv.) and dry and degassed PhCl (0.5 mL) was then added to the vial and stirred for around **1 min**. The catalyst solution (0.5 mL of dry and degassed PhCl, containing $Cu(CH_3CN)_4PF_6$ (1 mg, 2.5 mol%) and dtbpy (1 mg, 3.5 mol%), pre-stirred for 10 min until fully dissolved) was then added under stirring. The resulting mixture was stirred at room temperature for 2 h. Upon completion, aqueous NaOH (2 mL, 1 M) was added, the aqueous layer was separated and extracted with EtOAc (2 x 3 mL). The combined organic layers were dried ($MgSO_4$) and filtered. The solvent was evaporated and the residue was purified by column chromatography on silica gel to afford the desired products.

General Procedure for the Nickel Catalyzed Ring Opening-Arylation of Aziridine Boranes – GP8a

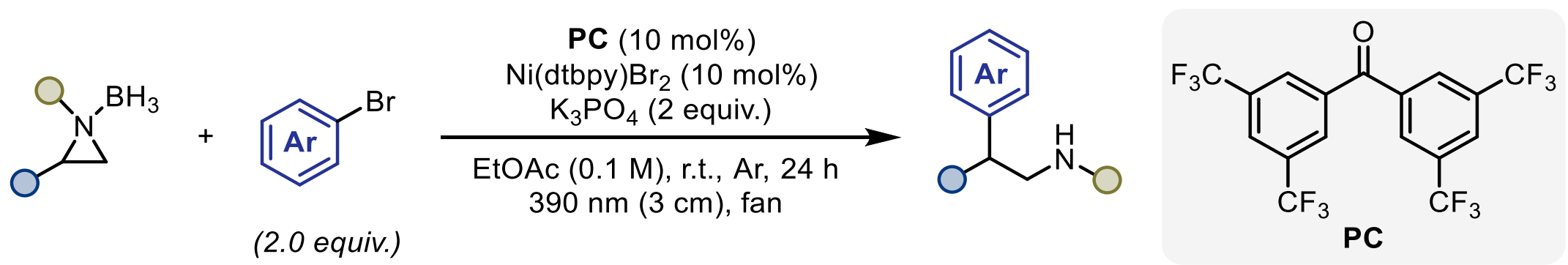

An oven-dried microwave vial was charged with aziridine borane (0.1 mmol, 1.0 equiv.), aryl bromides (0.2 mmol, 2.0 equiv.), 3,3',5,5'-tetrakis(trifluoromethyl)benzophenone (5 mg, 10 mol%), $Ni(dtbpy)Br_2$ (5 mg, 10 mol%) and K_3PO_4 (42 mg, 2.0 equiv.). The tube was capped with a Supelco aluminium crimp seal with septum (PTFE/butyl), evacuated and refilled with Ar (x 3). Dry and degassed EtOAc (1.0 mL) was then added to the vial and the mixture was stirred for 1

min at room temperature and then was placed 3 cm from the 390 nm purple LEDs (*the reaction temperature was around 35 °C, it's worth noting that distance and temperature have an influence on the reaction yield*). The LEDs were switched on (kessil, 100 W) and the mixture was stirred under irradiation with a fan for 24 hours. Upon completion, aqueous NaOH (2 mL, 1 M) was added, the aqueous layer was separated and extracted with EtOAc (2 x 3 mL). The combined organic layers were dried ($MgSO_4$) and filtered. The solvent was evaporated, and the residue was purified by column chromatography on silica gel to afford the desired products.

General Procedure for the Nickel Catalyzed Ring Opening-Arylation of Aziridine Boranes – GP8b

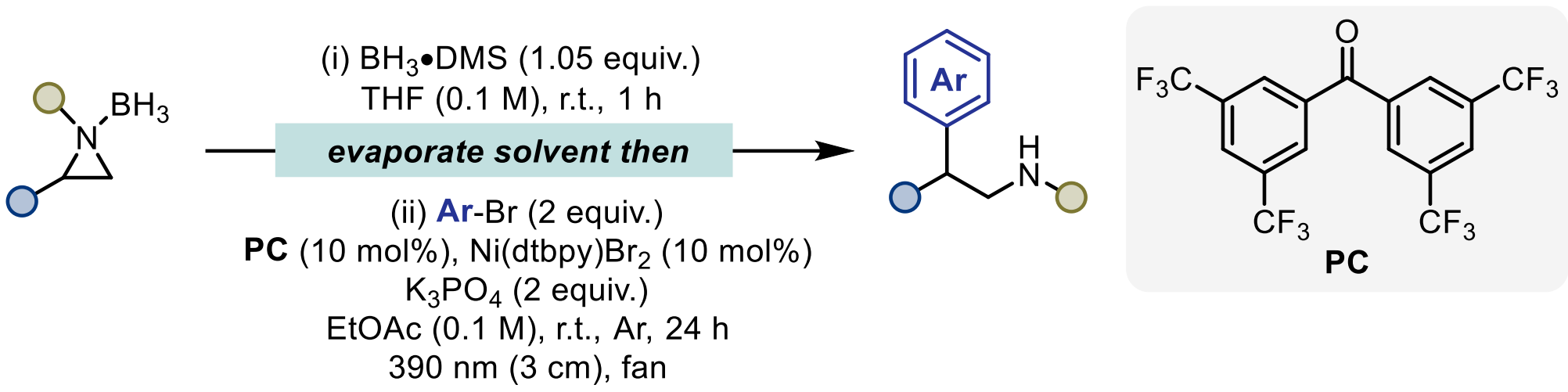

An oven-dried microwave vial equipped with a stirring bar was charged with the aziridine (0.1 mmol, 1.0 equiv.), dry THF (1 mL, 0.1 M) was added, followed by BH_3•DMS (52.5 uL, 1.05 equiv., 2 M in THF). The mixture was stirred for 1 h at r.t. The volatiles were evaporated, and the vial was charged with the aryl bromide (2.0 equiv.), 3,3',5,5'-tetrakis(trifluoromethyl)benzophenone (5 mg, 10 mol%), Ni(dtbpy)Br_2 (5 mg, 10 mol%) and K_3PO_4 (42 mg, 2.0 equiv.). The tube was capped with a Supelco aluminium crimp seal with septum (PTFE/butyl), evacuated and refilled with Ar (x 3). Dry and degassed EtOAc (1.0 mL) was then added to the vial and the mixture was stirred for **1 min** at room temperature and then was placed 3 cm from the 390 nm purple LEDs (*the reaction temperature was around 35 °C*). The LEDs were switched on (kessil, 100 W) and the mixture was stirred under irradiation with a fan for 24 hours. Upon completion, aqueous NaOH (2 mL, 1 M) was added, the aqueous layer was separated and extracted with EtOAc (2 x 3 mL). The combined organic layers were dried ($MgSO_4$) and filtered. The solvent was evaporated, and the residue was purified by column chromatography on silica gel to afford the desired products.

3 Starting Material Synthesis

Please note that the starting material synthesis is not optimised.

3.1 Peroxides

Trimethyl((2-phenylpropan-2-yl)peroxy)silane

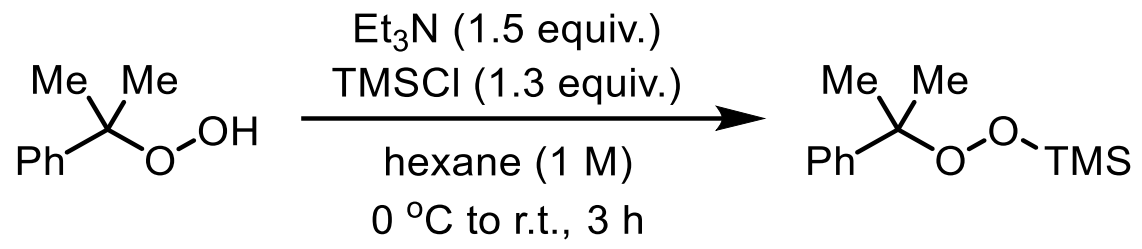

A round bottom flask equipped with a stirring bar was charged with Et_3N (10.5 mL, 75.0 mmol, 1.50 equiv.) and hexane (100 mL). CumylO_2H (9.3 mL, 80% technical solution, 50 mmol, 1.0 equiv.) was slowly added. The reaction media was then cooled in an ice-water bath and TMSCl (8.25 mL, 65.0 mmol, 1.30 equiv.) was slowly added. When the addition was completed, the ice-water bath was removed and the reaction was let stirring at room temperature. After 3 h, H_2O (100 mL) was added. The layers were separated and the organic phase was washed with H_2O (100 mL x 4) and brine (100 mL), dried ($MgSO_4$), filtered and evaporated to give cumylO_2TMS as an oil (10.97 g, 98%) with approx density of 0.94 g/mL. ^{1}H NMR (400 MHz, $CDCl_3$) δ 7.45 (2H, d, *J* = 8.4 Hz), 7.33 (2H, t, *J* = 7.7 Hz), 7.24 (1H, t, *J* = 7.9 Hz), 1.58 (6H, s), 0.18 (9H, s); ^{13}C NMR (101 MHz, $CDCl_3$) δ 145.4, 127.9, 127.0, 125.6, 83.4, 26.5, –1.1. Data in accordance with the literature.[7]

3.2 Sulfonium Bromide Salts

(2-Bromo-1-phenylethyl)dimethylsulfonium Bromide (S1a)

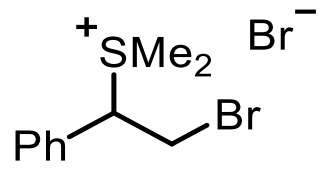

Following **GP1a**, styrene (23 mL, 200 mmol, 2.0 equiv.) gave **S1a** (24.52 g, 75%) as a solid. ^{1}H NMR (600 MHz, DMSO-d_6) 7.62 – 7.56 (2H, m), 7.56 – 7.50 (3H, m), 5.52 –5.44 (1H, dd, *J*=10.1, 5.5 Hz), 4.46 – 4.35 (2H, m), 3.00 (3H, s), 2.71 (3H, s); ^{13}C NMR (151 MHz, DMSO-d_6) δ 130.4, 130.1, 129.6, 129.3, 58.2, 29.3, 23.9, 22.7. Data in accordance with the literature.[8]

(2-Bromo-1-(4-(*tert*-butyl)phenyl)ethyl)dimethylsulfonium Bromide (S2a)

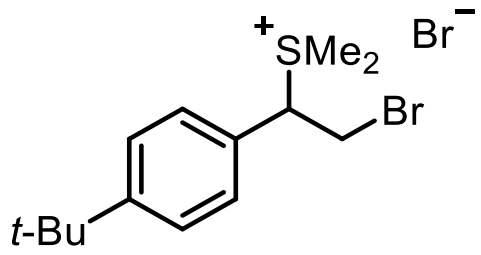

Following **GP1a**, 1-(*tert*-butyl)-4-vinylbenzene (5.49 mL, 30 mmol, 1.5 equiv.) gave **S2a** (4.36 g, 57%) as a solid. ^{1}H NMR (600 MHz, DMSO-d_6) 7.55 (2H, d, *J* = 8.0 Hz), 7.49 (2H, d, *J* = 8.0 Hz), 5.36 (1H, dd, *J* = 10.0, 5.6 Hz), 4.44 – 4.31 (2H, m), 2.94 (3H, s), 2.67 (3H, s), 1.30 (9H, s); ^{13}C NMR (151 MHz, DMSO-d_6): 153.0, 129.3, 127.0, 126.2, 58.3, 34.6, 30.9, 29.4, 23.8, 22.6.

(2-Bromo-1-(4-methoxyphenyl)ethyl)dimethylsulfonium Bromide (S3a)

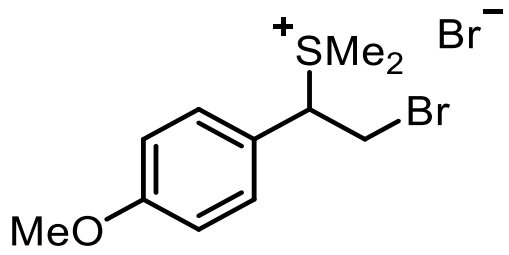

Following **GP1a**, 1-methoxy-4-vinylbenzene (4.03 g, 30 mmol, 1.5 equiv.) gave **S3a** (4.27 g, 60%) as a solid. ^{1}H NMR (600 MHz, DMSO-d_6) δ 7.29 (2H, d, *J* = 8.4 Hz), 6.87 (2H, d, *J* = 8.6 Hz), 4.71 (1H, dd, *J* = 7.1, 4.7 Hz), 3.72 (3H, s), 3.59 (1H, dd, *J* = 10.2, 4.7 Hz), 3.52 (1H, dd, *J* = 10.1, 7.1 Hz), 2.54 (6H, s); ^{13}C NMR (151 MHz, DMSO-d_6) δ 158.7, 134.7, 127.5, 113.5, 71.8, 55.1, 40.4 (2C), 40.4.

(2-Bromo-1-(4-bromophenyl)ethyl)dimethylsulfonium Bromide (S4a)

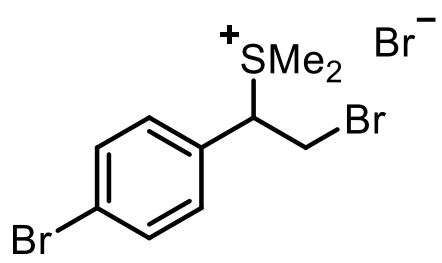

Following **GP1b**, 1-bromo-4-vinylbenzene (2.6 mL, 20 mmol, 1.0 equiv.) gave **S4a** (2.90 g, 36%) as a solid. ^{1}H NMR (400 MHz, DMSO-d_6) δ 7.77 (2H, d, *J* = 8.3 Hz), 7.51 (2H, d, *J* = 8.5 Hz), 5.32 (1H, dd, *J* = 9.9, 5.9 Hz), 4.44 – 4.29 (2H, m), 2.92 (3H, s), 2.68 (3H, s); ^{13}C NMR (101

MHz, DMSO-d_6) δ 132.4, 131.7, 129.4, 124.2, 57.8, 28.9, 23.9, 22.6. Data in accordance with the literature.[1]

(2-Bromo-1-(4-chlorophenyl)ethyl)dimethylsulfonium Bromide (S5a)

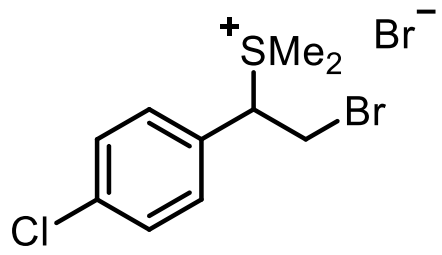

Following **GP1b**, 1-chloro-4-vinylbenzene (3.6 mL, 30 mmol, 1.0 equiv.) gave **S5a** (4.40 g, 41%) as a solid. ^{1}H NMR (600 MHz, DMSO-d_6) δ 7.64 (2H, d, J = 8.6 Hz), 7.59 (2H, d, J = 8.6 Hz), 5.36 (1H, dd, J = 10.1, 5.6 Hz), 4.40 (1H, dd, J = 10.8, 5.7 Hz), 4.36 (1H, t, J = 10.5 Hz), 2.94 (3H, s), 2.69 (3H, s); ^{13}C NMR (151 MHz, DMSO-d_6) δ 135.3, 131.5, 129.5, 129.0, 57.7, 29.0, 23.9, 22.6.

(2-Bromo-1-(4-(trifluoromethyl)phenyl)ethyl)dimethylsulfonium Bromide (S6a)

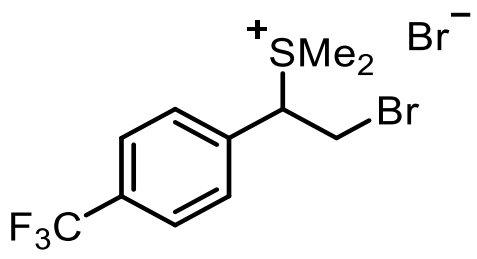

Following **GP1a**, 1-(trifluoromethyl)-4-vinylbenzene (5.16 g, 30 mmol, 1.5 equiv.) gave **S6a** (2.34 g, 30%) as a solid. *<u>Note</u>: This sulfonium bromide salt could not be characterized as it was quickly decomposing. Hence, it was directly used in the second step (see below in **section 3.3**).*

(1-(Bicyclo[4.2.0]octa-1(6),2,4-trien-3-yl)-2-bromoethyl)dimethylsulfonium Bromide (S7a)

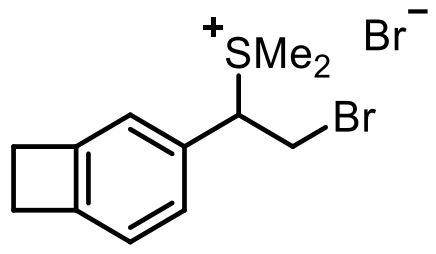

Following **GP1b**, 3-vinylbicyclo[4.2.0]octa-1(6),2,4-triene (0.5 mL, 3.7 mmol, 1.0 equiv.) gave **S7a** (800 mg, 61%) as a solid. ^{1}H NMR (600 MHz, DMSO-d_6) δ 7.32 (1H, d, J = 7.4 Hz), 7.28 (1H, s), 7.24 (1H, d, J = 7.6 Hz), 5.30 – 5.17 (1H, m), 4.38 (1H, dd, J = 10.7, 5.6 Hz), 4.32 (1H, t, J = 10.4 Hz), 3.19 (4H, s), 2.91 (3H, s), 2.63 (3H, s); ^{13}C NMR (151 MHz, DMSO-d_6) δ 148.1, 146.5, 128.8, 128.4, 123.5, 123.4, 59.6, 29.5, 29.3, 29.1, 24.0, 22.7.

(2-Bromo-1-(*p*-tolyl)ethyl)dimethylsulfonium Bromide (S8a)

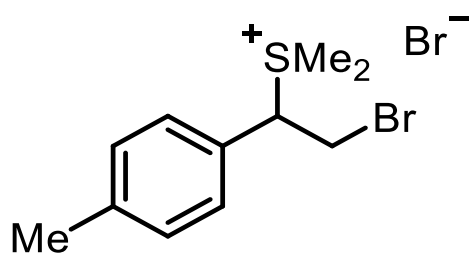

Following **GP1b**, 1-methyl-4-vinylbenzene (5.27 mL, 40 mmol, 1.0 equiv.) gave **S8a** (9.0 g, 66%) as a solid. ^{1}H NMR (600 MHz, DMSO-d_6) δ 7.47 (2H, d, J = 7.5 Hz), 7.36 (2H, d, J = 7.8 Hz),

5.58 (1H, dd, *J* = 9.2, 5.6 Hz), 4.38 (1H, dd, *J* = 11.0, 5.9 Hz), 4.25 (1H, t, *J* = 10.1 Hz), 3.11 (3H, s), 2.78 (3H, s), 2.45 (3H, s); ^{13}C NMR (151 MHz, DMSO-*d*$_6$) δ 140.5, 129.7, 129.0, 126.1, 59.1, 28.7, 24.0, 22.2, 20.8.

(2-Bromo-1-(4-fluorophenyl)ethyl)dimethylsulfonium Bromide (S9a)

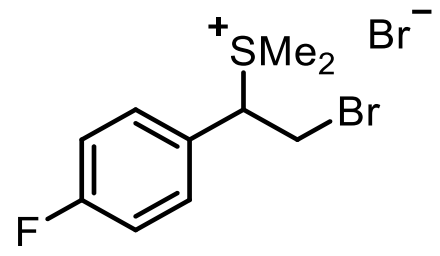

Following **GP1b**, 1-fluoro-4-vinylbenzene (4.77 mL, 40 mmol, 1.0 equiv.) gave **S9a** (9.75 g, 71%) as a solid. ^{1}H NMR (400 MHz, DMSO-*d*$_6$) δ 7.66 – 7.57 (2H, m), 7.47 – 7.36 (2H, m), 5.45 – 5.25 (1H, m), 4.54 – 4.26 (2H, m), 2.93 (3H, s), 2.67 (3H, s); ^{13}C NMR (101 MHz, DMSO-*d*$_6$) δ 132.0 (d, *J* = 8.9 Hz), 126.3 (d, *J* = 3.3 Hz), 116.5 (d, *J* = 21.7 Hz), 57.8, 29.2, 23.9, 22.5; ^{19}F NMR (565 MHz, DMSO-*d*$_6$) δ –80.42 (d, *J* = 6.2 Hz).

(2-Bromo-1-(2-chlorophenyl)ethyl)dimethylsulfonium Bromide (S10a)

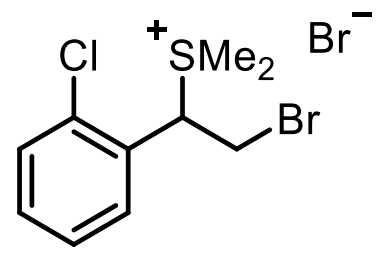

Following **GP1b**, 1-chloro-2-vinylbenzene (5.1 mL, 40 mmol, 1.0 equiv.) gave **S10a** (*considered as 60-70%*) as a solid. *Note: This sulfonium bromide salt could not be characterized as it was quickly decomposing. Hence, it was directly used in the second step (see below in* ***section 3.3****).*

(2-Bromo-1-(naphthalen-2-yl)ethyl)dimethylsulfonium Bromide (S11a)

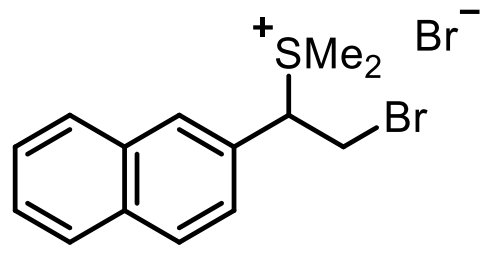

Following **GP1b**, 2-vinylnaphthalene (3.08 g, 20 mmol, 1.0 equiv.) gave **S11a** (4.6 g, 61%) as a solid. ^{1}H NMR (600 MHz, DMSO-*d*$_6$) δ 8.17 – 8.12 (1H, m), 8.10 (1H, d, *J* = 8.5 Hz), 8.06 – 7.99 (2H, m), 7.68 – 7.59 (3H, m), 5.47 (1H, dd, *J* = 8.8, 7.0 Hz), 4.51 – 4.47 (2H, m), 2.98 (3H, s), 2.70 (3H, s); ^{13}C NMR (151 MHz, DMSO-*d*$_6$) δ 133.5, 133.6, 130.0, 129.3, 128.3, 127.8, 127.7, 127.4, 127.2, 125.8, 58.9, 29.3, 24.0, 22.8.

(2-Bromo-1-(o-tolyl)ethyl)dimethylsulfonium Bromide (S12a)

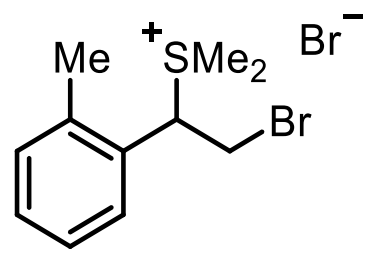

Following **GP1a**, 1-methyl-2-vinylbenzene (1.94 mL, 15 mmol, 1.5 equiv.) gave **S12a** (2.15 g, 83%) as a solid. ^{1}H NMR (400 MHz, DMSO-d_6) δ 7.65 – 7.55 (1H, m), 7.44 – 7.32 (3H, m), 5.60 – 5.47 (1H, m), 4.41 – 4.24 (2H, m), 3.14 (3H, s), 2.78 (3H, s), 2.43 (3H, s); ^{13}C NMR (101 MHz, DMSO-d_6): 138.5, 131.3, 130.0, 129.6, 127.8 (br s), 127.1, 54.8 (br s), 30.6, 23.5, 23.3, 19.6.

(2-Bromo-1-(3-bromophenyl)ethyl)dimethylsulfonium Bromide (S13a)

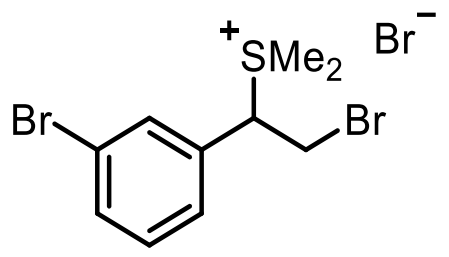

Following **GP1b**, 1-bromo-3-vinylbenzene (1.95 mL, 15 mmol, 1.0 equiv.) gave **S13a** (*considered as 60-70%*) as a solid. <u>Note</u>*: This sulfonium bromide salt could not be characterized as it was quickly decomposing under air. Hence, it was directly used in the second step (see below in* ***section 3.3****).*

3.3 Aziridines

2-Phenyl-1-propylaziridine (1)

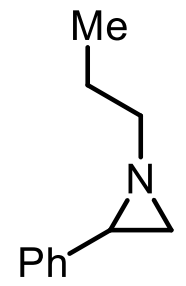

Following **GP2**, **S1a** (1.63 g, 5 mmol, 1.0 equiv.) and propylamine (1.48 g, 25.0 mmol, 5.0 equiv.) gave after purification via column chromatography **1** (734 mg, 91%) as an oil. R*f* 0.58 [*n*-pentane:EtOAc (10:1)]. ^{1}H NMR (400 MHz, $CDCl_3$) δ 7.35 – 7.19 (5H, m), 2.50 (1H, dt, *J* = 11.5, 7.3 Hz), 2.37 – 2.26 (2H, m), 1.91 (1H, d, *J* = 3.4 Hz), 1.73 – 1.61 (3H, m), 0.99 (3H, t, *J* = 7.4 Hz). Data in accordance with the literature.[9]

2-(4-(*tert*-Butyl)phenyl)-1-propylaziridine (2)

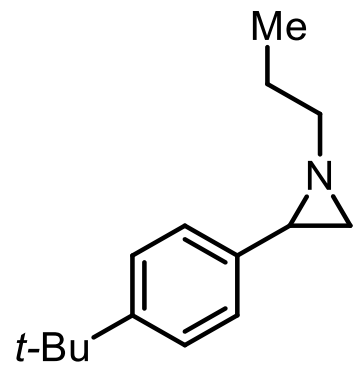

Following **GP2**, **S2a** (4.36 g, 11.4 mmol, 1.0 equiv.) and propylamine (3.37 g, 57.0 mmol, 5.0 equiv.) gave after purification via column chromatography **2** (2.28 g, 92%) as an oil. R_f 0.23 [*n*-pentane:EtOAc (30:1)]. ^{1}H NMR (400 MHz, $CDCl_3$) δ 7.35 (2H, d, *J* = 8.3 Hz), 7.22 (2H, d, *J* = 8.4 Hz), 2.50 (1H, dt, *J* = 11.6, 7.2 Hz), 2.36 – 2.25 (2H, m), 1.91 (1H, d, *J* = 3.4 Hz), 1.73 – 1.61 (3H, m), 1.34 (9H, s), 0.99 (3H, t, *J* = 7.4 Hz); ^{13}C NMR (151 MHz, $CDCl_3$) δ 149.8, 137.6, 126.0, 125.3, 63.9, 41.1, 37.7, 34.6, 31.5, 23.2, 12.2. HRMS (ESI) found MH^+ 218.1896, $C_{15}H_{24}N$ requires 218.1903.

2-(4-Methoxyphenyl)-1-propylaziridine (3)

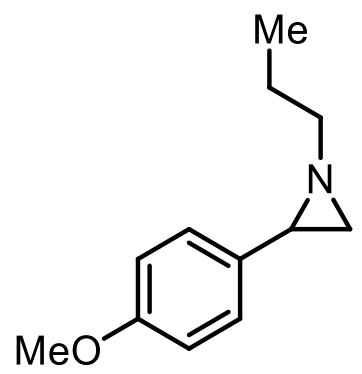

Following **GP2**, **S3a** (4.84 g, 13.6 mmol, 1.0 equiv.) and propylamine (4.02 g, 68.0 mmol, 5.0 equiv.) gave after purification via column chromatography **3** (1.46 g, 56%) as an oil. R_f 0.4 [*n*-pentane:EtOAc (6:1)]. ^{1}H NMR (600 MHz, $CDCl_3$) δ 7.16 (2H, d, *J* = 8.6 Hz), 6.83 – 6.81 (2H, d, *J* = 8.6 Hz), 3.78 (3H, s), 2.46 (1H, dt, *J* = 11.5, 7.2 Hz), 2.30 – 2.22 (2H, m), 1.85 (1H, d, *J* = 3.3 Hz), 1.66 – 1.59 (3H, m), 0.95 (3H, t, *J* = 7.4 Hz); ^{13}C NMR (151 MHz, $CDCl_3$) δ 158.8, 132.8, 127.4, 113.9, 63.8, 55.4, 40.9, 37.5, 23.2, 12.1. HRMS (ESI) found MH^+ 192.1375, $C_{12}H_{18}NO$ requires 192.1383.

2-(4-Bromophenyl)-1-propylaziridine (4)

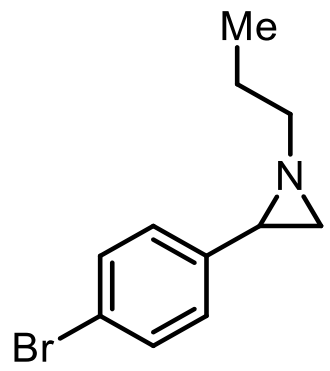

Following **GP2**, **S4a** (2.90 g, 7.2 mmol, 1.0 equiv.) and propylamine (3.0 mL, 36 mmol, 5.0 equiv.) gave **4** (1.57 g, 91%) as a yellow oil. ^{1}H NMR (400 MHz, $CDCl_3$) δ 7.40 (2H, d, *J* = 8.4 Hz), 7.12 (2H, d, *J* = 8.4 Hz), 2.46 (1H, dt, *J* = 11.5, 7.2 Hz), 2.36 – 2.19 (2H, m), 1.83 (1H, d, *J* = 3.3 Hz), 1.72 – 1.55 (3H, m), 0.95 (3H, t, *J* = 7.4 Hz); ^{13}C NMR (101 MHz, $CDCl_3$) δ 139.9, 131.4, 128.0, 120.6, 63.6, 40.7, 38.0, 23.2, 12.1. HRMS (ESI) found MH^+ 240.0384, $C_{11}H_{15}BrN$ requires 240.0382.

2-(4-Chlorophenyl)-1-propylaziridine (5)

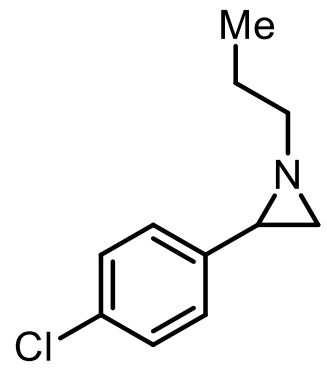

Following **GP2**, **S5a** (4.40 g, 12.3 mmol, 1.0 equiv.) and propylamine (5.1 mL, 62 mmol, 5.0 equiv.) gave **5** (2.17 g, 90%) as a yellow oil. ^{1}H NMR (600 MHz, $CDCl_3$) δ 7.25 (2H, d, *J* = 8.4 Hz), 7.17 (2H, d, *J* = 8.5 Hz), 2.46 (1H, dt, *J* = 11.5, 7.2 Hz), 2.35 – 2.22 (2H, m), 1.83 (1H, d, *J* = 3.2 Hz), 1.70 – 1.55 (3H, m), 0.95 (3H, t, *J* = 7.4 Hz); ^{13}C NMR (151 MHz, $CDCl_3$) δ 139.3, 132.5, 128.5, 127.7, 63.6, 40.7, 38.0, 23.2, 12.1. HRMS (ESI) found MH^+ 196.0888, $C_{11}H_{15}NCl$ requires 196.0888.

1-Propyl-2-(4-(trifluoromethyl)phenyl)aziridine (6)

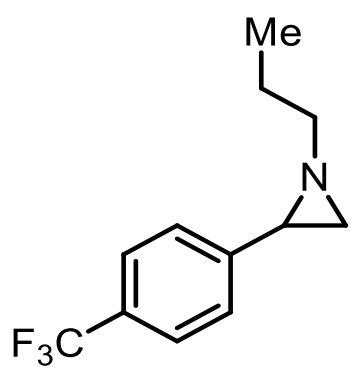

Following **GP2**, **S6a** (1.89 g, 6.0 mmol, 1.0 equiv.) and propylamine (1.77 g, 30.0 mmol, 5.0 equiv.) gave after purification via column chromatography **6** (1.11 g, 81%) as an oil. R_f 0.55 [*n*-pentane:EtOAc (10:1)]. ^{1}H NMR (600 MHz, $CDCl_3$) δ 7.53 (2H, d, *J* = 8.1 Hz), 7.35 (2H, d, *J* = 8.0 Hz), 2.49 (1H, dt, *J* = 11.5, 7.2 Hz), 2.36 – 2.27 (2H, m), 1.87 (1H, dd, *J* = 3.2, 0.8 Hz), 1.72 (1H, d, *J* = 6.5 Hz), 1.63 (2H, sx, *J* = 7.3 Hz), 0.96 (3H, t, *J* = 7.4 Hz); ^{13}C NMR (151 MHz, $CDCl_3$) δ 145.03, 129.10 (q, *J* = 32.3 Hz), 126.57, 125.31 (q, *J* = 3.8 Hz), 124.5 (q, *J* = 271.9 Hz),

63.5, 40.8, 38.4, 23.1, 12.1; ^{19}F NMR (565 MHz, $CDCl_3$) δ –62.37. HRMS (ESI) found MH^+ 230.1145, $C_{12}H_{15}NF_3$ requires 230.1151.

2-(Bicyclo[4.2.0]octa-1(6),2,4-trien-3-yl)-1-propylaziridine (7)

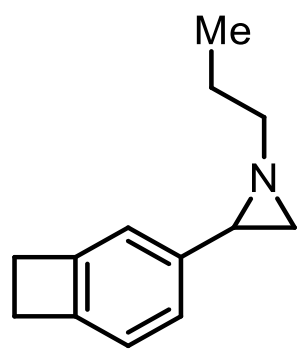

Following **GP2**, **S7a** (800 mg, 2.26 mmol, 1.0 equiv.) and propylamine (900 µL, 11.25 mmol, 5.0 equiv.) gave **7** (360 mg, 95%) as yellow oil. ^{1}H NMR (400 MHz, $CDCl_3$) δ 7.10 (1H, dd, *J* = 7.4, 1.5 Hz), 6.96 (1H, d, *J* = 7.6 Hz), 6.94 (1H, s), 3.13 (4H, s), 2.48 (1H, dt, *J* = 11.5, 7.2 Hz), 2.32 – 2.19 (2H, m), 1.85 (1H, d, *J* = 3.3 Hz), 1.71 – 1.57 (3H, m), 0.96 (3H, t, *J* = 7.4 Hz); ^{13}C NMR (101 MHz, $CDCl_3$) δ 146.0, 144.4, 139.3, 125.1, 122.4, 120.4, 63.8, 42.0, 37.9, 29.5, 29.4, 23.2, 12.2. HRMS (ESI) found MH^+ 188.1433, $C_{13}H_{18}N$ requires 188.1434.

1-Methyl-2-phenylaziridine (8)

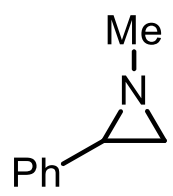

Following **GP2**, **S1a** (3.26 g, 10 mmol, 1 equiv.) and 40% methylamine aqueous solution (4.6 mL, 50 mmol, 5.0 equiv.) gave after purification via column chromatography **8** (821 mg, 54%) as an oil. R_f 0.21 [*n*-pentane:EtOAc (10:1)]. ^{1}H NMR (600 MHz, $CDCl_3$) δ 7.33 – 7.27 (2H, m), 7.25 – 7.19 (3H, m), 2.50 (3H, s), 2.27 (1H, dd, *J* = 6.5, 3.3 Hz), 1.91 (1H, d, *J* = 3.4 Hz), 1.63 (1H, d, *J* = 6.5 Hz). Data in accordance with the literature.[10]

1-Hexyl-2-phenylaziridine (9)

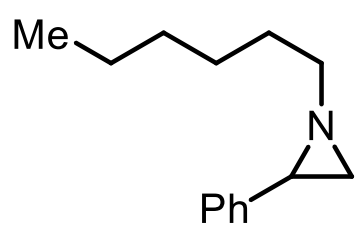

Following **GP2**, **S1a** (4.89 g, 15 mmol, 1.0 equiv.) and *n*-hexylamine (7.59 g, 75.0 mmol, 5.0 equiv.) gave after purification via column chromatography **9** (2.65 g, 87%) as an oil. R_f 0.72 [*n*-pentane:EtOAc (10:1)]. ^{1}H NMR (600 MHz, $CDCl_3$) δ 7.31 – 7.18 (5H, m), 2.52 – 2.44 (1H, m), 2.36 – 2.27 (2H, m), 1.89 (1H, d, *J* = 3.3 Hz), 1.67 – 1.57 (3H, m), 1.40 – 1.33 (2H, m), 1.33 – 1.24 (4H, m), 0.88 (3H, t, *J* = 6.9 Hz). Data in accordance with the literature.[9]

1-Benzyl-2-phenylaziridine (10)

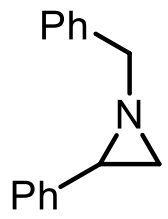

Following **GP2**, **S1a** (4.89 g, 15 mmol, 1.0 equiv.) and benzylamine (8.04 g, 75.0 mmol, 5.0 equiv.) gave after purification via column chromatography **10** (2.64 g, 84%) as an oil. R_f 0.55 [*n*-pentane:EtOAc (10:1)]. ^{1}H NMR (600 MHz, $CDCl_3$) δ 7.43 – 7.39 (2H, m), 7.37 – 7.22 (8H, m), 3.73 (1H, d, *J* = 13.7 Hz), 3.65 (1H, d, *J* = 13.7 Hz), 2.54 (1H, dd, *J* = 6.5, 3.4 Hz), 2.02 (1H, d, *J* = 3.3 Hz), 1.88 (1H, d, *J* = 6.5 Hz). Data in accordance with the literature.[10]

1-Isopropyl-2-phenylaziridine (11)

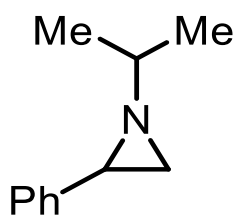

Following **GP2**, **S1a** (1.63 g, 5 mmol, 1.0 equiv.) and isopropylamine (1.48 g, 25.0 mmol, 5.0 equiv.) gave after purification via column chromatography **11** (613 mg, 76%) as an oil. R*f* 0.55 [*n*-pentane:EtOAc (10:1)]. ^{1}H NMR (600 MHz, $CDCl_3$) δ 7.34 – 7.27 (4H, m), 7.27 – 7.21 (1H, m), 2.37 (1H, dd, *J* = 6.6, 3.4 Hz), 1.92 (1H, d, *J* = 3.3 Hz), 1.69 (1H, d, *J* = 6.6 Hz), 1.64 (1H, hp, *J* = 6.3 Hz), 1.21 (6H, d, *J* = 6.3 Hz). Data in accordance with the literature.[9]

1-Cyclohexyl-2-phenylaziridine (12)

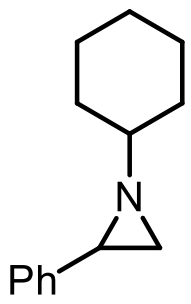

Following **GP2**, **S1a** (4.89 g, 15 mmol, 1.0 equiv.) and cyclohexylamine (7.44 g, 75.0 mmol, 5.0 equiv.) gave after purification via column chromatography **12** (2.63 g, 87%) as an oil. R_f 0.6 [*n*-pentane:EtOAc (10:1)]. ^{1}H NMR (600 MHz, $CDCl_3$) δ 7.32 – 7.27 (4H, m), 7.26 – 7.20 (1H, m), 2.37 (1H, dd, *J* = 6.6, 3.3 Hz), 1.93 – 1.86 (3H, m), 1.85 – 1.75 (2H, m), 1.69 (1H, d, *J* = 6.0 Hz), 1.66 – 1.61 (1H, m), 1.50 – 1.41 (2H, m), 1.36 – 1.27 (1H, m), 1.27 – 1.17 (3H, m). Data in accordance with the literature.[9]

1-Isopropyl-2-phenethylaziridine (13)

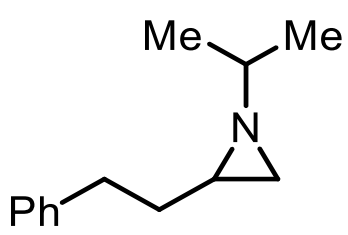

Following **GP3a**, 4-phenyl-1-butene (1.32 g, 10.0 mmol, 1.0 equiv.) and isopropylamine (709 mg, 12.0 mmol, 1.2 equiv.) gave **13** (571 mg, 30% *over two steps*) as an oil. R_f 0.26 [*n*-pentane: EtOAc

(1:1)]. ^{1}H NMR (600 MHz, $CDCl_3$) δ 7.29 (2H, t, *J* = 7.6 Hz), 7.25 – 7.21 (2H, m), 7.21 – 7.17 (1H, m), 2.86 (1H, ddd, *J* = 13.8, 10.1, 5.8 Hz), 2.73 (1H, ddd, *J* = 13.8, 10.0, 6.2 Hz), 1.81 (1H, dddd, *J* = 13.7, 10.0, 6.2, 5.0 Hz), 1.63 (1H, dddd, *J* = 13.6, 10.0, 7.6, 5.8 Hz), 1.53 (1H, d, *J* = 3.4 Hz), 1.42 – 1.33 (2H, m), 1.25 (1H, d, *J* = 6.4 Hz), 1.17 (3H, d, *J* = 6.2 Hz), 1.14 (3H, d, *J* = 6.3 Hz); ^{13}C NMR (151 MHz, $CDCl_3$) δ 142.1, 128.4, 128.4, 125.8, 60.9, 38.7, 35.2, 34.1, 33.1, 22.5, 21.9. Data in accordance with the literature.[4]

1-Propyl-2-(*p*-tolyl)aziridine (14)

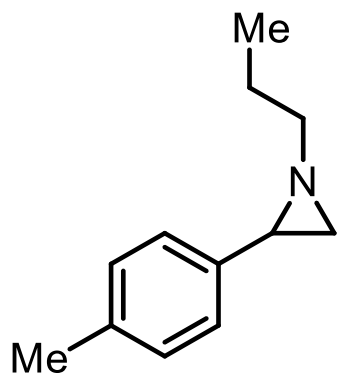

Following **GP2**, **S8a** (9.00 g, 26.4 mmol, 1.0 equiv.) and propylamine (6.5 mL, 135 mmol, 5.0 equiv.) gave **14** (3.1 g, 90%) as a yellow oil. ^{1}H NMR (600 MHz, $CDCl_3$) δ 7.14 (2H, d, *J* = 8.2 Hz), 7.10 (2H, d, *J* = 7.9 Hz), 2.49 (1H, dt, *J* = 11.5, 7.2 Hz), 2.32 (3H, s), 2.29 – 2.21 (2H, m), 1.87 (1H, dd, *J* = 3.4, 0.7 Hz), 1.68 – 1.58 (3H, m), 0.96 (3H, t, *J* = 7.4 Hz); ^{13}C NMR (151 MHz, $CDCl_3$) δ 137.7, 136.5, 129.1, 126.2, 63.8, 41.2, 37.7, 23.2, 21.2, 12.1. HRMS (ESI) found MH^+ 176.1434, $C_{12}H_{18}N$ requires 176.1434.

2-(4-Fluorophenyl)-1-propylaziridine (15)

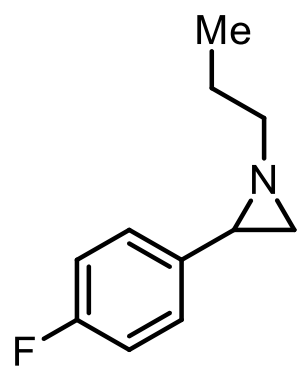

Following **GP2**, **S9a** (9.75 g, 28 mmol, 1.0 equiv.) and propylamine (6.5 mL, 140 mmol, 5.0 equiv.) gave **15** (4.77 g, 95%) as an oil. ^{1}H NMR (600 MHz, $CDCl_3$) δ 7.20 (2H, dd, *J* = 8.6, 5.6 Hz), 6.97 (2H, dd, *J* = 8.7, 8.3 Hz), 2.45 (1H, dt, *J* = 11.4, 7.2 Hz), 2.34 – 2.25 (2H, m), 1.84 (1H, d, *J* = 3.2 Hz), 1.68 – 1.58 (3H, m), 0.96 (3H, t, *J* = 7.4 Hz); ^{13}C NMR (151 MHz, $CDCl_3$) δ 162.3 (d, *J* = 244.3 Hz), 136.6 (d, *J* = 3.0 Hz), 128.0 (d, *J* = 7.9 Hz), 115.4 (d, *J* = 21.3 Hz), 63.9, 40.9, 38.0, 23.4, 12.3; ^{19}F NMR ($CDCl_3$, 565 MHz) δ –116.39 (tt, *J* = 8.6, 4.4 Hz). HRMS (ESI) found MH^+ 180.1184, $C_{11}H_{15}FN_2$ requires 180.1183.

2-(2-Chlorophenyl)-1-propylaziridine (16)

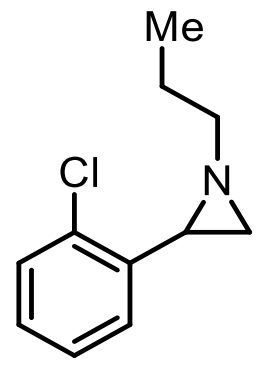

Following **GP2**, **S10a** (8.65 g, 24 mmol, 1.0 equiv.) and propylamine (7.5 mL, 125 mmol, 5.0 equiv.) gave **16** (2.11 g, 27% *over two steps*) as a light-yellow oil. ^{1}H NMR (400 MHz, $CDCl_3$) δ 7.36 – 7.27 (2H, m), 7.24 – 7.10 (2H, m), 2.65 (1H, dd, J = 6.7, 3.2 Hz), 2.49 (1H, dt, J = 11.8, 7.2 Hz), 2.38 (1H, dt, J = 11.8, 7.0 Hz), 1.81 (1H, d, J = 3.3 Hz), 1.73 (1H, d, J = 6.5 Hz), 1.65 (2H, sx, J = 7.3 Hz), 1.01 – 0.94 (3H, t, J = 7.4 Hz); ^{13}C NMR (101 MHz, $CDCl_3$) δ 138.0, 133.8, 128.9, 127.7, 127.5, 126.8, 63.3, 38.8, 37.2, 23.1, 12.0. HRMS (ESI) found MH^+ 196.0889, $C_{11}H_{15}ClN$ requires 196.0888.

1-(*tert*-Butyl)-2-phenylaziridine (17)

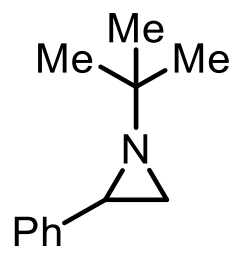

Following **GP2**, **S1a** (4.89 g, 15 mmol, 1.0 equiv.) and *tert*-butylamine (5.49 g, 75.0 mmol, 5.0 equiv.) gave after purification via column chromatography **17** (2.31 g, 88%) as an oil. R_f 0.2 [*n*-pentane:EtOAc (50:1)]. ^{1}H NMR (600 MHz, $CDCl_3$): 7.33 – 7.26 (4H, m), 7.23 – 7.17 (1H, m), 2.63 (1H, dd, J = 6.4, 3.0 Hz), 1.89 (1H, dd, J = 6.4, 0.9 Hz), 1.64 (1H, dd, J = 3.1, 0.9 Hz), 1.05 (9H, s). Data in accordance with the literature.[10]

2-(4-Fluorophenyl)-1-(4-methoxyphenethyl)aziridine (18)

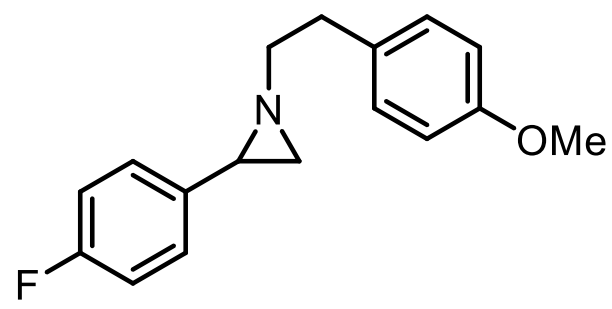

Following **GP2**, **S9a** (4.7 g, 13.65 mmol, 1.0 equiv.) and 2-(4-methoxyphenyl)ethan-1-amine (5.1 mL, 34.5 mmol, 2.5 equiv.) gave after purification via column chromatography **18** (1.84 g, 95%) as a light-yellow oil. R_f 0.39 [*n*-pentane:EtOAc (8:2)]. ^{1}H NMR (600 MHz, $CDCl_3$) δ 7.18 – 7.12 (2H, m), 7.09 (2H, d, J = 8.3 Hz), 6.97 (2H, t, J = 8.7 Hz), 6.83 – 6.75 (2H, m), 3.78 (3H, s), 2.86 (2H, t, J = 7.4 Hz), 2.73 (1H, dt, J = 11.5, 7.4 Hz), 2.54 (1H, dt, J = 11.5, 7.4 Hz), 2.23 (1H, dd, J = 6.6, 3.4 Hz), 1.84 (1H, d, J = 3.3 Hz), 1.64 (1H, d, J = 6.5 Hz); ^{13}C NMR (151 MHz, $CDCl_3$) δ 162.1 (d, J = 244.4 Hz), 158.1, 136.2 (d, J = 2.9 Hz), 132.2, 129.9, 127.8 (d, J = 8.1 Hz), 115.2 (d, J = 21.4 Hz), 113.9, 63.6, 55.4, 40.9, 37.9, 35.6; ^{19}F NMR (565 MHz, $CDCl_3$) δ –116.29. HRMS (ESI) found MH^+ 272.1443, $C_{17}H_{19}FNO$ requires 272.1445.

1-(2,3-dihydro-1*H*-inden-1-yl)-2-(p-tolyl)aziridine (**19**)

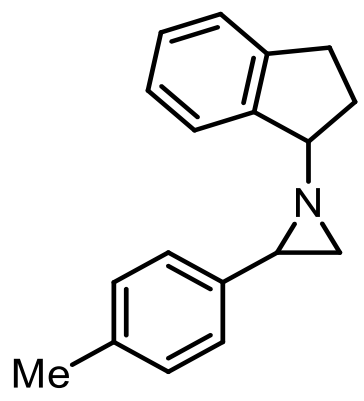

Following **GP2**, **S8a** (1.02 g, 3 mmol, 1.0 equiv.) and 2,3-dihydro-1*H*-inden-1-amine (768 µL, 6 mmol, 2.0 equiv.) gave after purification via column chromatography **19** as mixture of diastereomers (770 mg, 99% yield, the mixture was used directly in the next step) as an oil. d.r. 1:1. R_f 0.74 and 0.87 [*n*-pentane:EtOAc (8:2)]. ^{1}H NMR (400 MHz, $CDCl_3$), s*ignals corresponding to both diastereomers*: δ 7.48 – 7.42 (0.5H, m), 7.31 – 7.14 (5H, m), 7.10 (2.5H, d, *J* = 8.1 Hz), 3.32 (0.5H, t, *J* = 7.0 Hz), 3.25 (0.5H, dd, *J* = 7.0, 4.9 Hz), 3.18 – 3.01 (1H, m), 2.89 – 2.77 (1H, m), 2.71 (0.5H, dd, *J* = 6.6, 3.3 Hz), 2.57 (0.5H, dd, *J* = 6.6, 3.4 Hz), 2.42 – 2.26 (4H, m), 2.22 – 2.11 (1H, m), 2.09 (0.5H, d, *J* = 6.6 Hz), 2.02 (0.5H, d, *J* = 3.4 Hz), 1.92 (0.5H, d, *J* = 3.3 Hz), 1.87 (0.5H, d, *J* = 6.6 Hz); ^{13}C NMR (101 MHz, $CDCl_3$) δ 144.5, 144.2, 144.1, 144.1, 137.4, 137.3, 136.6, 136.5, 129.2, 129.1, 128.0, 127.7, 126.5 (2C), 126.4 (2C), 125.1, 124.8, 124.1 (2C), 74.9, 74.9, 41.2, 39.4, 37.4, 35.2, 33.0, 32.6, 30.8, 30.6, 21.2, 21.2. HRMS (ESI) found MNa^+ 272.1411, $C_{18}H_{19}NNa$ requires 272.1410.

1-(Furan-2-ylmethyl)-2-(p-tolyl)aziridine (20)

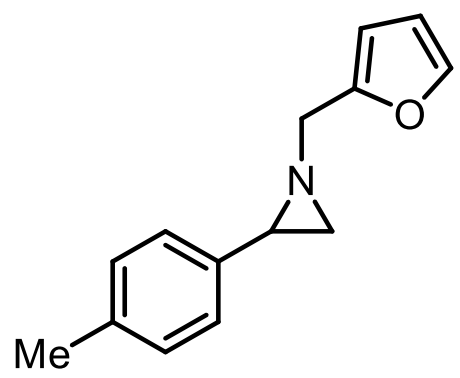

Following **GP2**, **S8a** (2.00 g, 5.81 mmol, 1.0 equiv.) and furan-2-ylmethanamine (2.7 mL, 34.5 mmol, 2.5 equiv.) gave **20** (1.30 g, 95%) as a light-yellow oil. ^{1}H NMR (600 MHz, $CDCl_3$) δ 7.36 (1H, s), 7.16 – 7.12 (2H, m), 7.10 (2H, d, *J* = 7.8 Hz), 6.32 – 6.29 (1H, m), 6.23 (1H, d, *J* = 3.2 Hz), 3.61 (2H, s), 2.51 (1H, dd, *J* = 6.7, 3.5 Hz), 2.31 (3H, s), 1.99 – 1.93 (1H, m), 1.86 (1H, d, *J* = 6.2 Hz); ^{13}C NMR (151 MHz, $CDCl_3$) δ 152.8, 142.1, 136.9, 136.7, 129.1, 126.3, 110.3, 107.5, 57.0, 41.1, 37.3, 21.2. HRMS (ESI) found MH^+ 214.1225, $C_{14}H_{16}NO$ requires 214.1226.

1-(2,2-Dimethoxyethyl)-2-(naphthalen-2-yl)aziridine (21)

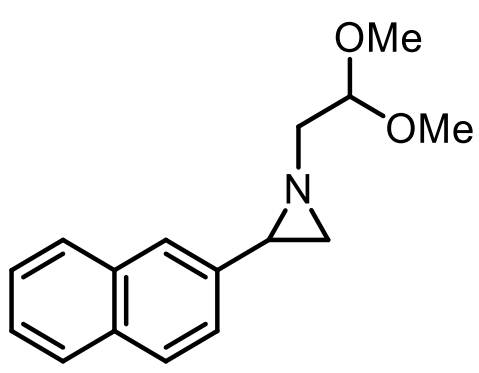

Following **GP2**, **S11a** (1.53 g, 4.1 mmol, 1.0 equiv.) and 2,2-dimethoxyethan-1-amine (2.25 mL, 20 mmol, 5.0 equiv.) gave **21** (1.01 g, 95%) as a light-yellow oil. ^{1}H NMR (600 MHz, $CDCl_3$) δ 7.83 – 7.75 (3H, m), 7.74 (1H, t, *J* = 1.1 Hz), 7.47 – 7.40 (2H, m), 7.36 (1H, dd, *J* = 8.5, 1.7 Hz), 4.61 (1H, t, *J* = 5.3 Hz), 3.44 (3H, s), 3.37 (3H, s), 2.76 (1H, dd, *J* = 12.2, 5.2 Hz), 2.62 (1H, dd, *J* = 6.5, 3.5 Hz), 2.55 (1H, dd, *J* = 12.2, 5.4 Hz), 2.05 (1H, d, *J* = 3.4 Hz), 1.84 (1H, d, *J* = 6.5 Hz); ^{13}C NMR (151 MHz, $CDCl_3$) δ 137.8, 133.5, 132.8, 128.1, 127.8, 127.8, 126.1, 125.6, 125.1, 124.6, 104.9, 63.1, 54.8, 41.6, 37.6. HRMS (ESI) found MH^+ 258.1488, $C_{16}H_{20}NO_2$ requires 258.1489.

1-Cyclopentyl-2-(*o*-tolyl)aziridine (22)

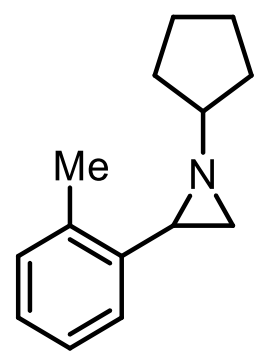

Following **GP2**, **S12a** (1.61 g, 4.75 mmol, 1.0 equiv.) and cyclopentylamine (2.35 mL, 140 mmol, 5.0 equiv.) gave after purification via column chromatography **22** (840 mg, 88%) as a yellow oil. R_f 0.64 [*n*-pentane:EtOAc (9:1)]. ^{1}H NMR (400 MHz, $CDCl_3$) δ 7.38 – 7.30 (1H, m), 7.20 – 7.04 (3H, m), 2.42 (1H, dd, *J* = 6.6, 3.4 Hz), 2.39 (3H, s), 2.11 (1H, p, *J* = 5.5 Hz), 1.96 – 1.82 (2H, m), 1.82 – 1.66 (6H, m), 1.64 – 1.51 (2H, m); ^{13}C NMR (101 MHz, $CDCl_3$) δ 138.7, 136.2, 129.5, 126.6, 126.5, 126.1, 72.5, 39.3, 36.1, 33.3, 32.4, 24.6, 24.4, 19.2. HRMS (ESI) found MH^+ 202.1591, $C_{14}H_{20}N$ requires 202.1590.

***tert*-Butyl 4-(2-(4-Chlorophenyl)aziridin-1-yl)piperidine-1-carboxylate (23)**

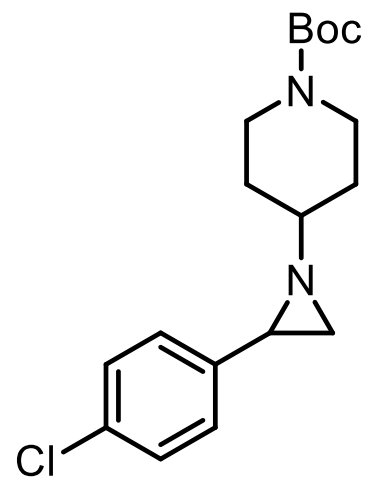

Following **GP2**, **S5a** (1.73 g, 4.8 mmol, 1.0 equiv.) and *tert*-butyl 4-aminopiperidine-1-carboxylate (4.80 g, 24 mmol, 5.0 equiv.) gave after purification via column chromatography **23** (1.44 g, 90%) as a light-yellow oil. R_f 0.65 [*n*-pentane:EtOAc (6:4)]. ^{1}H NMR (600 MHz, $CDCl_3$) δ 7.25 (2H, d, *J* = 8.4 Hz), 7.19 (2H, d, *J* = 8.5 Hz), 4.04 – 3.79 (2H, br m), 3.03 – 2.92 (2H, m), 2.36 (1H, dd, *J* = 6.5, 3.2 Hz), 1.83 (1H, d, *J* = 3.2 Hz), 1.77 (2H, br m), 1.71 (1H, d, *J* = 6.5 Hz), 1.62 – 1.52 (3H, m), 1.45 (9H, s); ^{13}C NMR (151 MHz, $CDCl_3$) δ 154.9, 138.9, 132.5, 128.4, 127.6, 79.4, 66.5, 39.4, 36.6, 31.8 (br s), 31.2 (br s), 28.4. HRMS (ESI) found MH^+ 337.1679, $C_{18}H_{26}ClN_2O_2$ requires 337.1677.

tert-Butyl 2-(2-phenylaziridin-1-yl)acetate (24)

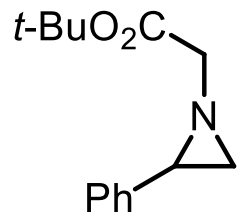

Following **GP2**, **S1a** (1.96 g, 6 mmol, 1.0 equiv.) and *tert*-butyl glycinate (3.15 g, 24.0 mmol, 4.0 equiv.) gave after purification via column chromatography **24** (602 mg, 43%) as an oil. R_f 0.74 [*n*-pentane:EtOAc (1:1)]. ^{1}H NMR (600 MHz, $CDCl_3$) δ 7.32 – 7.26 (4H, m), 7.24 – 7.18 (1H, m), 3.20 (1H, d, *J* = 16.0 Hz), 3.11 (1H, d, *J* = 15.9 Hz), 2.44 (1H, dd, *J* = 6.6, 3.5 Hz), 2.04 (1H, d, *J* = 3.6 Hz), 1.76 (1H, d, *J* = 6.6 Hz), 1.43 (9H, s); ^{13}C NMR (151 MHz, $CDCl_3$) δ 169.4, 139.6, 128.3, 127.0, 126.4, 81.3, 62.7, 41.4, 37.6, 28.1. Data in accordance with the literature.[11]

tert-Butyl 2-((4*R*,6*R*)-6-(2-(2-(4-chlorophenyl)aziridin-1-yl)ethyl)-2,2-dimethyl-1,3-dioxan-4-yl)acetate (25)

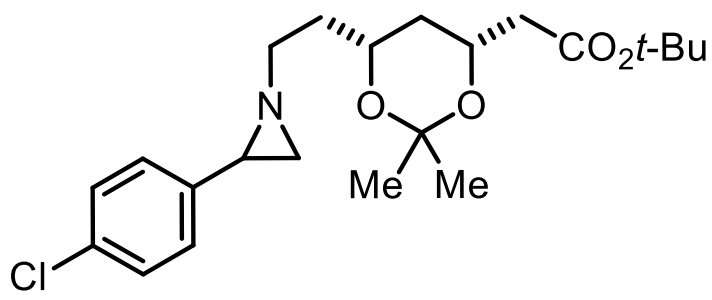

Following **GP2**, **S5a** (1.08 g, 3.00 mmol, 1.0 equiv.) and *tert*-butyl 2-((4*R*,6*R*)-6-(2-aminoethyl)-2,2-dimethyl-1,3-dioxan-4-yl)acetate (1.6 mL, 6 mmol, 2.0 equiv.) gave after purification via column chromatography **25** as mixture of diastereomers (1.16 g, 94% yield, the mixture was used directly in the next step) as an oil. d.r. 1:1. R_f 0.45 [*n*-pentane:EtOAc (8:2)]. ^{1}H NMR (400 MHz, $CDCl_3$), s*ignals corresponding to both diastereomers*: δ 7.25 (2H, br d, *J* = 8.9 Hz), 7.20 – 7.11 (2H, m), 4.29 – 4.15 (1H, m), 4.08 – 3.94 (1H, m), 2.60 – 2.36 (3H, m), 2.34 – 2.23 (2H, m), 1.83 (0.5H, d, *J* = 3.2 Hz), 1.79 – 1.63 (3.5H, m), 1.61 – 1.54 (1H, m), 1.48 – 1.41 (10.5H, m), 1.38 (1.5H, s), 1.35 (1.5H, s), 1.34 (1.5H, s), 1.23 – 1.12 (1H, m); ^{13}C NMR (101 MHz, $CDCl_3$, diastereomers) δ 170.4 (2C), 139.2, 139.2, 132.6, 132.6, 128.5, 128.5, 127.6, 127.6, 98.8, 98.8, 80.7 (2C), 66.9, 66.8, 66.4, 66.3, 57.1, 56.9, 42.9, 42.9, 41.0, 40.3, 38.7, 38.0, 36.9, 36.8, 36.7, 36.7, 30.3, 28.3, 20.0, 19.9. HRMS (ESI) found MH^+ 410.2092, $C_{22}H_{33}ClNO_4$ requires 410.2093.

2-Phenethyl-1-propylaziridine (26)

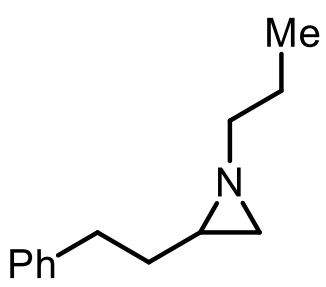

Following **GP3a**, 4-phenyl-1-butene (1.32 g, 10.0 mmol, 1.0 equiv.) and propylamine (709 mg, 12.0 mmol, 1.2 equiv.) gave **26** (291 mg, 15% *over two steps*) as an oil. R_f 0.29 [*n*-pentane:EtOAc (1:1)]. ^{1}H NMR (600 MHz, $CDCl_3$) δ 7.23 – 7.17 (2H, m), 7.15 – 7.08 (3H, m), 2.72 (1H, ddd, *J* = 13.7, 9.5, 6.1 Hz), 2.64 (1H, ddd, *J* = 13.8, 9.4, 6.5 Hz), 2.16 (1H, ddd, *J* = 11.6, 8.5, 6.6 Hz),

2.04 (1H, ddd, J = 11.5, 8.3, 5.9 Hz), 1.70 – 1.55 (2H, m), 1.55 – 1.47 (2H, m), 1.45 (1H, d, J = 3.5 Hz), 1.25 – 1.18 (1H, m), 1.13 (1H, d, J = 6.3 Hz), 0.86 (3H, t, J = 7.4 Hz); ^{13}C NMR (151 MHz, $CDCl_3$) δ 142.1, 128.6, 128.5, 125.9, 63.6, 39.1, 35.0, 34.01, 33.99, 23.2, 12.1. HRMS (ESI) found MH^+ 190.1584, $C_{13}H_{20}N$ requires 190.1590.

1-Hexyl-2-phenethylaziridine (27)

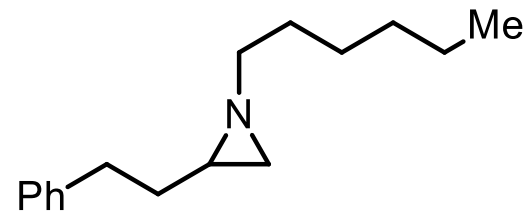

Following **GP3a**, 4-phenyl-1-butene (1.32 g, 10.0 mmol, 1.0 equiv.) and hexylamine (1.21 g, 12.0 mmol, 1.2 equiv.) gave **27** (1.20 g, 52% *over two steps*) as an oil. R_f 0.6 [*n*-pentane: EtOAc (1:1)]. ^{1}H NMR (400 MHz, $CDCl_3$) δ 7.31 – 7.12 (5H, m), 2.84 – 2.63 (2H, m), 2.30 – 1.19 (1H, m), 2.16 – 2.04 (1H, m), 1.79 – 1.60 (2H, m), 1.60 – 1.48 (3H, m), 1.40 – 1.20 (7H, m), 1.18 (1H, d, J = 6.3 Hz), 0.87 (3H, t, J = 6.7 Hz). Data in accordance with the literature.[4]

1-Benzyl-2-methylaziridine (28)

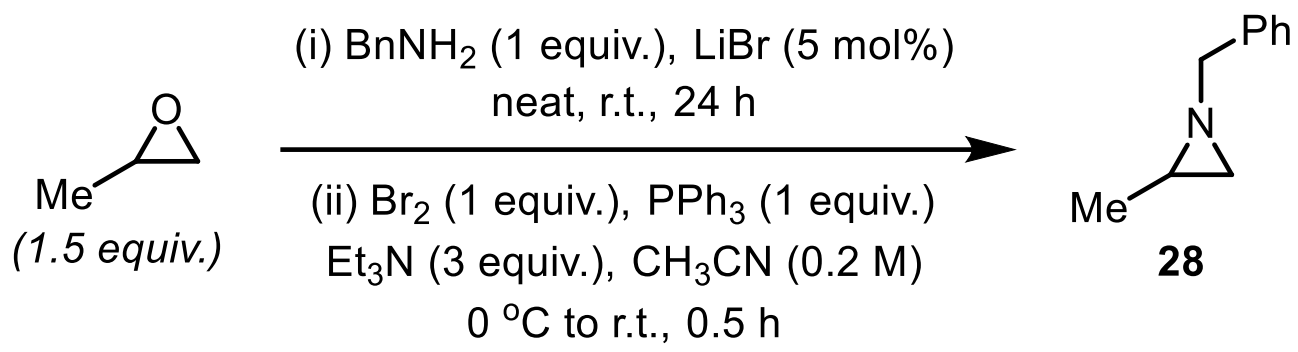

Step 1: To a round bottom flask equipped with a stirring bar was added propylene oxide (1.05 mL, 15 mmol, 1.5 equiv.), benzylamine (1.1 mL, 10 mmol, 1.0 equiv.) and LiBr (41 mg, 0.5 mmol, 5 mol%). The mixture was stirred for 24 h at room temperature. The solvent was evaporated to afford crude amino alcohol, that was used directly in the next step.

Step 2: An ice-cold solution of bromine (0.51 mL, 10 mmol, 1.0 equiv.) in CH_3CN (6 mL) was added dropwise to an ice-cold solution of triphenylphosphine (2.63 g, 10 mmol, 1.0 equiv.) in CH_3CN (15 mL). To this mixture was slowly added the crude amino alcohol (10 mmol, 1.0 equiv.), followed by the dropwise addition of triethylamine (4.16 mL, 30 mmol, 3.0 equiv.) at 0 °C. The mixture was then stirred at room temperature for 30 min. Triethylamine hydrobromide was filtered off and the solution was concentrated by rotary evaporation and purified directly by column chromatography on silica gel to give **28** (258 mg, 17% *over two steps*) as an oil. R_f 0.38 [*n*-pentane: EtOAc (1:1)]. ^{1}H NMR (600 MHz, $CDCl_3$) δ 7.30 – 7.23 (4H, m), 7.21 – 7.15 (1H, m), 3.39 (1H, d, J = 13.6 Hz), 3.33 (1H, d, J = 13.6 Hz), 1.50 (1H, d, J = 3.5 Hz), 1.47 – 1.42 (1H, m), 1.31 (1H, d, J = 6.3 Hz), 1.14 (3H, d, J = 5.5 Hz). Data in accordance with the literature.[12]

1-Benzyl-2-butylaziridine (29)

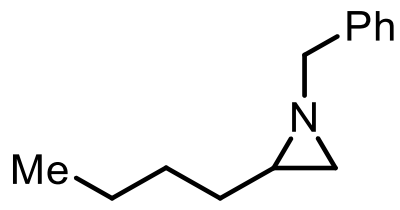

Following **GP3a**, 1-hexene (1.25 mL, 10 mmol, 1.0 equiv.) and benzylamine (1.30 mL, 12.0 mmol, 1.2 equiv.) gave **29** (700 mg, 37%) as an oil. R_f 0.61 [*n*-pentane: EtOAc (1:1)]. ^{1}H NMR (600 MHz, $CDCl_3$) δ 7.39 – 7.32 (4H, m), 7.31 – 7.25 (1H, m), 3.51 (1H, d, *J* = 13.2 Hz), 3.36 (1H, d, *J* = 13.3 Hz), 1.63 (1H, d, *J* = 3.2 Hz), 1.51 – 1.25 (8H, m), 0.87 (3H, t, *J* = 7.0 Hz). Data in accordance with the literature.[12]

2-Butyl-1-(1-(2,6-dimethylphenoxy)propan-2-yl)aziridine (30)

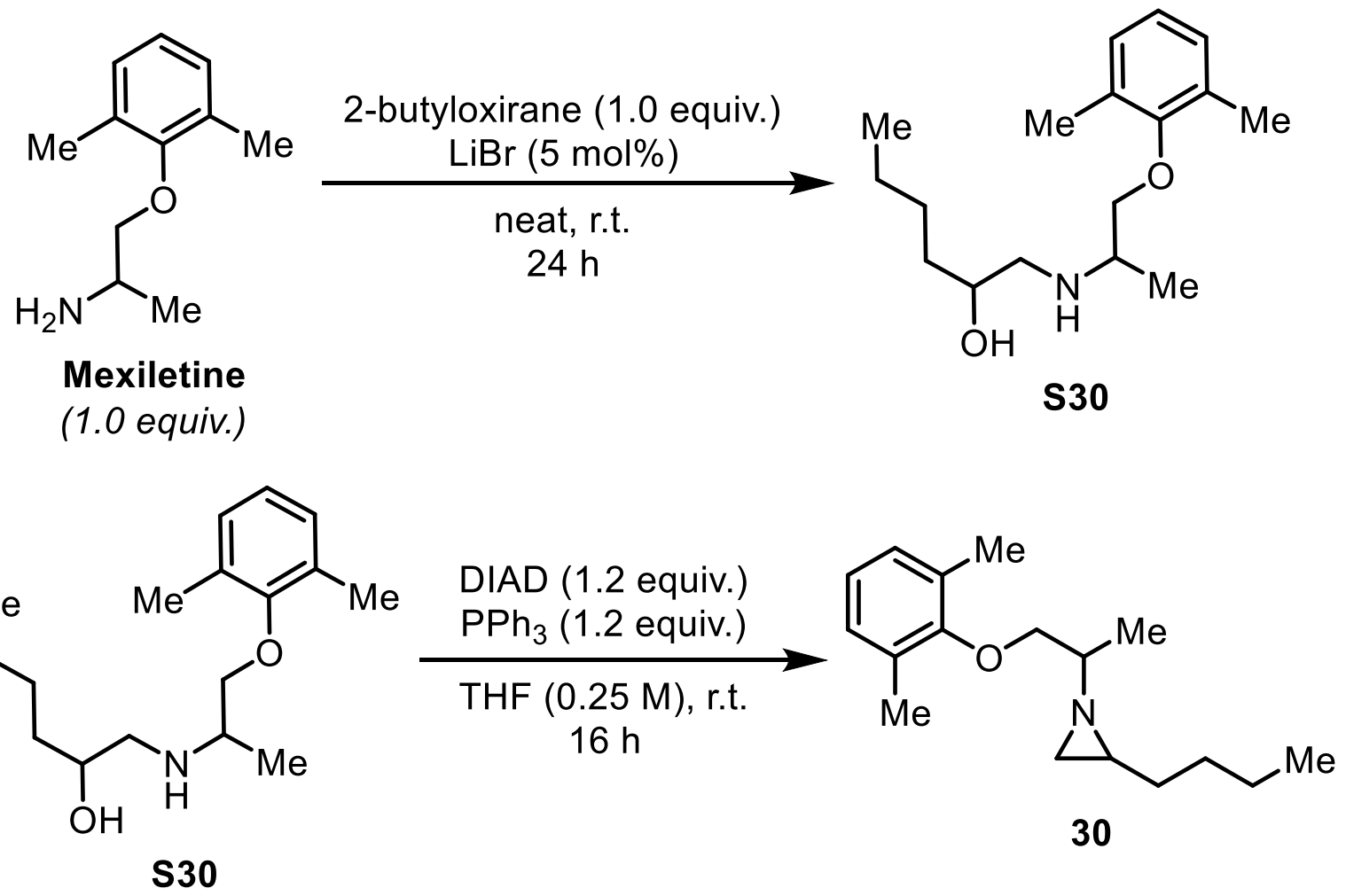

Step 1: A round bottom flask equipped with a stirring bar was charged with Mexiletine (950 mg, 5.3 mmol, 1.0 equiv.), 2-butyloxirane (0.64 mL, 5.3 mmol, 1.0 equiv.) and LiBr (23 mg, 0.26 mmol, 5 mol%) The resulting reaction mixture was stirred at room temperature for 24 h. Upon completion, the mixture was concentrated by rotary evaporation and the residue was purified by column chromatography on silica gel to afford the desired amino alcohol intermediate **S30** as mixture of diastereomers (578 mg, 39%, the mixture was used directly in the next step) as an oil. d.r. 1:1. R_f 0.41 [EtOAc]. ^{1}H NMR (600 MHz, $CDCl_3$), *signals corresponding to both diastereomers*: δ 7.00 (2H, d, *J* = 7.4 Hz), 6.94 – 6.88 (1H, m), 3.72 – 3.64 (2H, m), 3.64 – 3.57 (1H, m), 3.14 – 3.06 (1H, m), 2.92 – 2.83 (1H, m), 2.54 – 2.47 (1H, m), 2.28 (6H, s), 1.52 – 1.41 (3H, m), 1.39 – 1.30 (3H, m), 1.21 (1.5H, d, *J*=6.5 Hz), 1.18 (1.5H, d, *J*=6.4 Hz), 0.94 – 0.88 (3H, m); ^{13}C NMR (151 MHz, $CDCl_3$) δ 155.6, 155.5, 130.9, 130.9, 129.1, 129.1, 124.0, 124.0, 76.0, 75.7, 70.3, 70.1, 53.7, 53.2, 53.0, 52.8, 35.1, 34.8, 28.0, 28.0, 23.0, 22.9, 18.0, 17.5, 16.4, 16.4, 14.2; HRMS (ESI) found MH^+ 280.2262, $C_{17}H_{30}NO_2$ requires 280.2271.

Step 2: A round bottom flask equipped with a stirring bar was charged with the amino alcohol (549 mg, 2.1 mmol, 1.0 equiv.) and PPh_3 (0.65 g, 2.52 mmol, 1.2 equiv.). Dry THF (8.4 mL, 0.25

M) was added followed by the dropwise addition of diisopropylazodicarboxylate (DIAD) (0.5 mL, 2.52 mmol, 1.2 equiv.) via syringe. The resulting reaction mixture was stirred at room temperature for 16 h. Upon completion, the mixture was concentrated by rotary evaporation and the residue was purified by column chromatography on silica gel to afford **30** as a single diastereomer (159 mg, 29%) as an oil. R_f 0.69 [*n*-pentane:EtOAc (1:1)]. ^{1}H NMR (600 MHz, $CDCl_3$) δ 7.01 (2H, d, *J*=7.4 Hz), 6.92 (1H, t, *J*=7.5 Hz), 3.86 (1H, dd, *J*=9.0, 5.5 Hz), 3.67 (1H, dd, *J*=9.0, 6.6 Hz), 2.28 (6H, s), 1.81 (1H, sx, *J*=6.4 Hz), 1.60 – 1.47 (3H, m), 1.42 – 1.22 (9H, m), 0.87 (3H, t, *J*=7.1 Hz); ^{13}C NMR (151 MHz, $CDCl_3$) δ 156.0, 131.0, 129.0, 123.9, 76.2, 65.2, 40.3, 33.1, 31.9, 29.7, 22.7, 17.7, 16.5, 14.1; HRMS (ESI) found MH^+ 262.2158, $C_{17}H_{28}NO$ requires 262.2165.

7-cyclohexyl-7-azabicyclo[4.1.0]heptane (33)

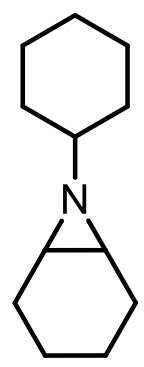

Following **GP3b**, 7-oxabicyclo[4.1.0]heptane (491 mg, 5 mmol, 1.0 equiv.) and cyclohexanamine (496 mg, 5.0 mmol, 1.0 equiv.) gave **33** (448 mg, 50% *over two steps*) as an oil. R*f* 0.62 [*n*-pentane:EtOAc (5:1)]. ^{1}H NMR (600 MHz, $CDCl_3$) δ 1.80 – 1.68 (8H, m), 1.59 – 1.52 (1H, m), 1.47 – 1.39 (2H, m), 1.37 – 1.26 (4H, m), 1.20 – 1.05 (5H, m), 0.98 (1H, tt, *J*=10.8, 3.9 Hz); ^{13}C NMR (151 MHz, $CDCl_3$) δ 69.8, 36.9, 32.5, 26.3, 25.2, 25.0, 20.8. Data in accordance with the literature.[13]

7-Benzyl-7-azabicyclo[4.1.0]heptane (34)

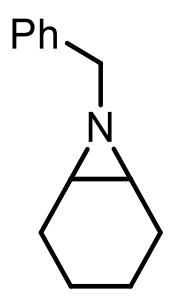

A round bottom flask equipped with a stirring bar was charged with (1*R*,2*R*)-2-(benzylamino)cyclohexan-1-ol (0.5 g, 2.4 mmol, 1.0 equiv.) and PPh_3 (0.75 g, 2.9 mmol, 1.2 equiv.). Dry THF (9.6 mL, 0.25 M) was added followed by the dropwise addition of diisopropylazodicarboxylate (DIAD) (0.57 mL, 2.9 mmol, 1.2 equiv.) via syringe. The resulting reaction mixture was stirred at room temperature for 16 h. Upon completion, the mixture was concentrated by rotary evaporation and the residue was purified by column chromatography on silica gel to afford **34** (371 mg, 83%) as an oil. R_f 0.35 [*n*-pentane: EtOAc (10:1)]. ^{1}H NMR (600 MHz, $CDCl_3$) δ 7.38 – 7.34 (2H, m), 7.34 – 7.28 (2H, m), 7.26 – 7.20 (1H, m), 3.47 (2H, s), 1.91

– 1.84 (2H, m), 1.81 – 1.73 (2H, m), 1.65 – 1.60 (2H, m), 1.45 – 1.36 (2H, m), 1.25 – 1.15 (2H, m). Data in accordance with the literature.[14]

1-(2-(Naphthalen-1-yloxy)ethyl)aziridine (36)

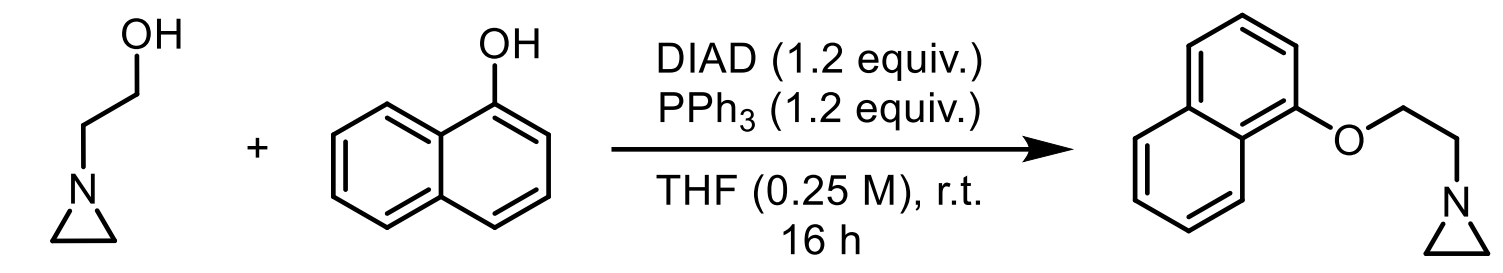

A round bottom flask equipped with a stirring bar was charged with 2-(aziridin-1-yl)ethan-1-ol (261 mg, 3.0 mmol, 1.0 equiv.) and PPh_3 (0.78 g, 3.6 mmol, 1.2 equiv.). Dry THF (12 mL, 0.25 M) was added followed by the dropwise addition of diisopropylazodicarboxylate (0.71 mL, 3.6 mmol, 1.2 equiv.) via syringe. The mixture was stirred at room temperature for 15 min, before adding 1-naphthol (519 mg, 5.0 mmol, 1.2 equiv.) in one portion. The resulting mixture was stirred at room temperature for 16 h. Upon completion, the mixture was concentrated by rotary evaporation and the residue was purified by column chromatography on silica gel to deliver **36** (333 mg, 52%) as an oil. R_f 0.16 [EtOAc]. ^{1}H NMR (600 MHz, $CDCl_3$) δ 8.32 – 8.27 (1H, m), 7.82 – 7.78 (1H, m), 7.52 – 7.45 (2H, m), 7.47 – 7.41 (1H, m), 7.37 (1H, t, *J* = 7.9 Hz), 6.84 (1H, dd, *J* = 7.5, 1.0 Hz), 4.35 (2H, t, *J* = 5.5 Hz), 2.78 (2H, t, *J* = 5.5 Hz), 1.88 – 1.85 (2H, m), 1.38 – 1.33 (2H, m); ^{13}C NMR (151 MHz, $CDCl_3$) δ 154.8, 134.7, 127.6, 126.5, 126.0, 125.8, 125.3, 122.2, 120.5, 104.8, 68.2, 60.6, 27.5. HRMS (ESI) found MNa^+ 236.1043, $C_{14}H_{15}NONa$ requires 236.1046.

2-(3-Bromophenyl)-1-propylaziridine (37)

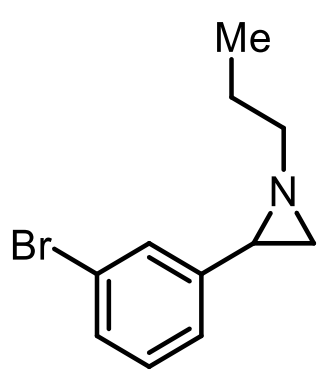

Following **GP2**, **S13a** (3.64 g, 9 mmol, 1.0 equiv.) and propylamine (3.2 mL, 45 mmol, 5.0 equiv.) gave **37** (560 mg, 16% *over two steps*) as yellow oil. ^{1}H NMR (600 MHz, $CDCl_3$) δ 7.41 (1H, t, *J* = 1.8 Hz), 7.35 (1H, ddd, *J* = 7.7, 2.1, 1.3 Hz), 7.20 (1H, dt, *J* = 7.7, 1.5 Hz), 7.16 (1H, t, *J* = 7.7 Hz), 2.47 (1H, dt, *J* = 11.5, 7.2 Hz), 2.32 (1H, dt, *J* = 11.5, 7.1 Hz), 2.25 (1H, dd, *J* = 6.5, 3.2 Hz), 1.87 (1H, dd, *J* = 3.3, 0.7 Hz), 1.69 (1H, d, *J* = 6.6 Hz), 1.65 (2H, sx, *J* = 7.3 Hz), 0.98 (3H, t, *J* = 7.4 Hz); ^{13}C NMR (151 MHz, $CDCl_3$) δ 143.3, 130.0, 129.9, 129.3, 125.1, 122.6, 63.6, 40.7, 38.1, 23.1, 12.1. HRMS (ESI) found MH^+ 240.0383, $C_{11}H_{15}BrN$ requires 240.0382.

2-Butyl-1-cyclohexylaziridine (38)

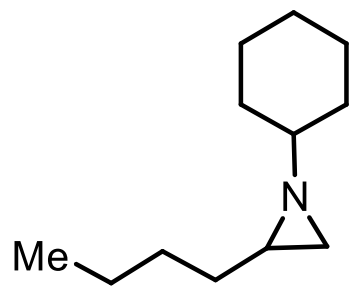

Following **GP3b**, 2-butyloxirane (501 mg, 5 mmol, 1.0 equiv.) and cyclohexanamine (496 mg, 5.0 mmol, 1.0 equiv.) gave **38** (326 mg, 38% *over two steps*) as an oil. R*f* 0.48 [*n*-pentane:EtOAc (1:1)]. ^{1}H NMR (600 MHz, $CDCl_3$) δ 1.84 – 1.77 (2H, m), 1.77 – 1.69 (2H, m), 1.63 – 1.56 (1H, m), 1.50 – 1.40 (3H, m), 1.40 – 1.08 (11H, m), 0.99 (1H, tt, *J*=10.8, 3.8 Hz), 0.89 (3H, t, *J*=7.2 Hz); ^{13}C NMR (151 MHz, $CDCl_3$) δ 69.2, 38.5, 33.3, 33.2, 32.6, 32.6, 30.2, 26.3, 25.2, 25.2, 22.8, 14.2; HRMS (ESI) found MH^+ 182.1905, $C_{12}H_{24}N$ requires 182.1903.

1-(*tert*-Butyl)-2-phenethylaziridine (39)

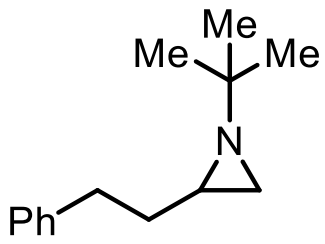

Following **GP3b**, 2-phenethyloxirane (741 mg, 5 mmol, 1.0 equiv.) and tert-butylamine (366 mg, 5.0 mmol, 1.0 equiv.) gave **39** (478 mg, 47% *over two steps*) as an oil. R*f* 0.36 [*n*-pentane:EtOAc (1:1)]. ^{1}H NMR (600 MHz, $CDCl_3$) δ 7.29 (2H, t, *J*=7.6 Hz), 7.23 (2H, d, *J*=6.8 Hz), 7.20 (1H, t, *J*=7.3 Hz), 2.85 (1H, ddd, *J*=14.7, 10.0, 5.0 Hz), 2.70 (1H, ddd, *J*=13.8, 9.7, 6.4 Hz), 1.86 – 1.77 (1H, m), 1.69 – 1.59 (2H, m), 1.51 (1H, d, *J*=6.2 Hz), 1.31 (1H, d, *J*=2.8 Hz), 1.01 (9H, s); ^{13}C NMR (151 MHz, $CDCl_3$) δ 142.2, 128.5, 128.4, 125.8, 52.6, 35.6, 34.4, 32.0, 26.8, 26.8; HRMS (ESI) found MH^+ 204.1742, $C_{12}H_{22}N$ requires 204.1747.

***tert*-Butyl 4-(2-butylaziridin-1-yl)piperidine-1-carboxylate (40)**

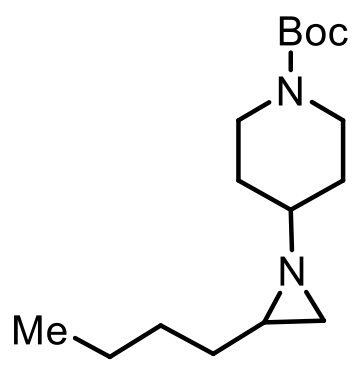

Following **GP3b**, 2-butyloxirane (501 mg, 5 mmol, 1.0 equiv.) and tert-butyl 4-aminopiperidine-1-carboxylate (1.00 g, 5.0 mmol, 1.0 equiv.) gave **40** (353 mg, 25% *over two steps*) as an oil. R*f* 0.5 [*n*-pentane:EtOAc (1:1)]. ^{1}H NMR (600 MHz, $CDCl_3$) δ 4.00 (2H, br s), 2.72 (2H, br s), 1.72 (2H, d, *J*=12.8 Hz), 1.59 – 1.39 (14H, m), 1.38 – 1.28 (4H, m), 1.27 – 1.16 (3H, m), 0.88 (3H, t, *J*=6.9 Hz); ^{13}C NMR (151 MHz, $CDCl_3$) δ 155.0, 79.5, 66.9, 38.7, 33.0, 32.7, 32.1, 31.6, 30.1, 28.6, 22.7, 14.2; HRMS (ESI) found MH^+ 305.2203, $C_{16}H_{30}N_2O_2Na$ requires 305.2200.

1-Cyclohexyl-2-(isopropoxymethyl)aziridine (41)

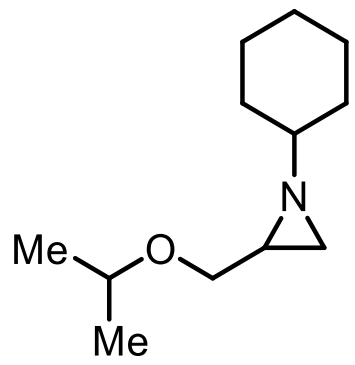

Following **GP3b**, 2-(isopropoxymethyl)oxirane (581 mg, 5 mmol, 1.0 equiv.) and cyclohexanamine (496 mg, 5.0 mmol, 1.0 equiv.) gave **41** (326 mg, 33% *over two steps*) as an oil. R*f* 0.37 [*n*-pentane:EtOAc (1:1)]. ^{1}H NMR (600 MHz, $CDCl_3$) δ 3.62 (1H, hept, *J*=6.1 Hz), 3.39 (1H, dd, *J*=10.5, 5.6 Hz), 3.30 (1H, dd, *J*=10.3, 5.5 Hz), 1.89 – 1.84 (1H, m), 1.83 – 1.77 (1H, m), 1.76 – 1.70 (2H, m), 1.61 – 1.55 (3H, m), 1.37 – 1.26 (3H, m), 1.09 – 1.04 (10H, m); ^{13}C NMR (151 MHz, $CDCl_3$) δ 71.7, 70.9, 69.0, 37.7, 33.0, 32.6, 30.8, 26.2, 25.1, 25.0, 22.5, 22.0; HRMS (ESI) found MH^+ 198.1851, $C_{12}H_{24}NO$ requires 198.1852.

***tert*-Butyl 2-((4*R*,6*R*)-2,2-dimethyl-6-(2-(2-phenethylaziridin-1-yl)ethyl)-1,3-dioxan-4-yl)acetate (42)**

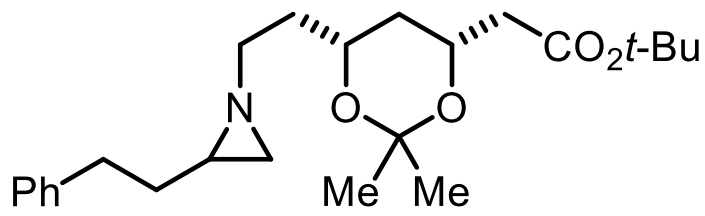

Following **GP3a**, 4-phenyl-1-butene (582 mg, 4.4 mmol, 1.0 equiv.) and *tert*-butyl 2-((4*R*,6*R*)-6-(2-aminoethyl)-2,2-dimethyl-1,3-dioxan-4-yl)acetate (2.41 g, 8.8 mmol, 2 equiv.) gave **42** as mixture of diastereomers (604 mg, 34% *over two steps*, the mixture was used directly in the next step) as an oil. d.r. 1:1. R_f 0.26 [*n*-pentane:EtOAc (1:1)]. ^{1}H NMR (600 MHz, $CDCl_3$), s*ignals corresponding to both diastereomers*: δ 7.24 – 7.18 (2H, m), 7.15 – 7.08 (3H, m), 4.22 – 4.13 (1H, m), 3.97 – 3.87 (1H, m), 2.75 – 2.60 (2H, m), 2.38 – 2.09 (4H, m), 1.69 – 1.56 (4H, m), 1.54 – 1.42 (2H, m), 1.39 – 1.34 (12H, m), 1.30 – 1.23 (4H, m), 1.18 – 1.07 (2H, m); ^{13}C NMR (151 MHz, $CDCl_3$) δ 170.4, 142.0, 128.5, 128.5, 128.5, 125.9, 125.9, 98.8, 67.0, 67.0, 66.4, 57.0, 57.0, 42.9, 39.5, 38.8, 37.0, 36.9, 36.7, 35.0, 34.6, 34.0, 33.9, 33.9, 30.3, 30.3, 28.2, 19.9; HRMS (ESI) found MNa^+ 426.2617, $C_{23}H_{28}N$ requires 426.2615.

Note: Most chemical shift between the two diastereomers in ^{1}H NMR and ^{13}C NMR are overlapped, the proton and carbon atoms close to the stereocenter are splitting, indicating the presence of a mixture of diastereomers.

3.4 Aziridine–Borane Complexes

In most cases, *N*-alkyl β-aryl and β-alkyl aziridines coordinate with BH_3 to give almost exclusively the *trans*-diastereomer. In some cases, the *cis*-diastereomer was obtained and it is mentioned in the individual product characterization.

trans-2-Phenyl-1-propylaziridine Borane (1a)

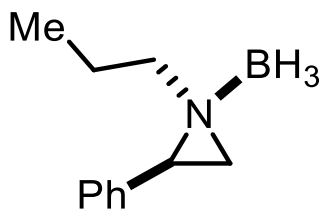

Following **GP4**, but adding BH_3•DMS at -78 °C, **1** (3.22 g, 20.0 mmol, 1.0 equiv.) gave after precipitation and filtration **1a** (2.94 g, 84%) as a solid. ^{1}H (600 MHz, $CDCl_3$) δ 7.41 – 7.31 (5H, m), 3.35 (1H, dd, J = 7.6, 6.2 Hz), 3.10 (1H, td, J = 11.5, 4.9 Hz), 2.80 (1H, dd, J = 6.2, 1.9 Hz), 2.45 – 2.34 (2H, m), 2.15 – 2.04 (1H, m), 1.94 – 1.82 (1H, m), 1.57 – 0.92 (6H, m); ^{13}C (101 MHz, DMSO-d_6) δ 131.9, 130.3, 128.2, 127.7, 65.3, 48.2, 40.6, 19.6, 11.1; ^{11}B (128 MHz, DMSO-d_6) δ –15.72. HRMS (ESI) found MNa^+ 198.1425, $C_{11}H_{18}BNNa$ requires 198.1425.

The relative configuration was determined by a 2D ^{1}H-^{1}H NOESY (600 MHz, $CDCl_3$) experiment:

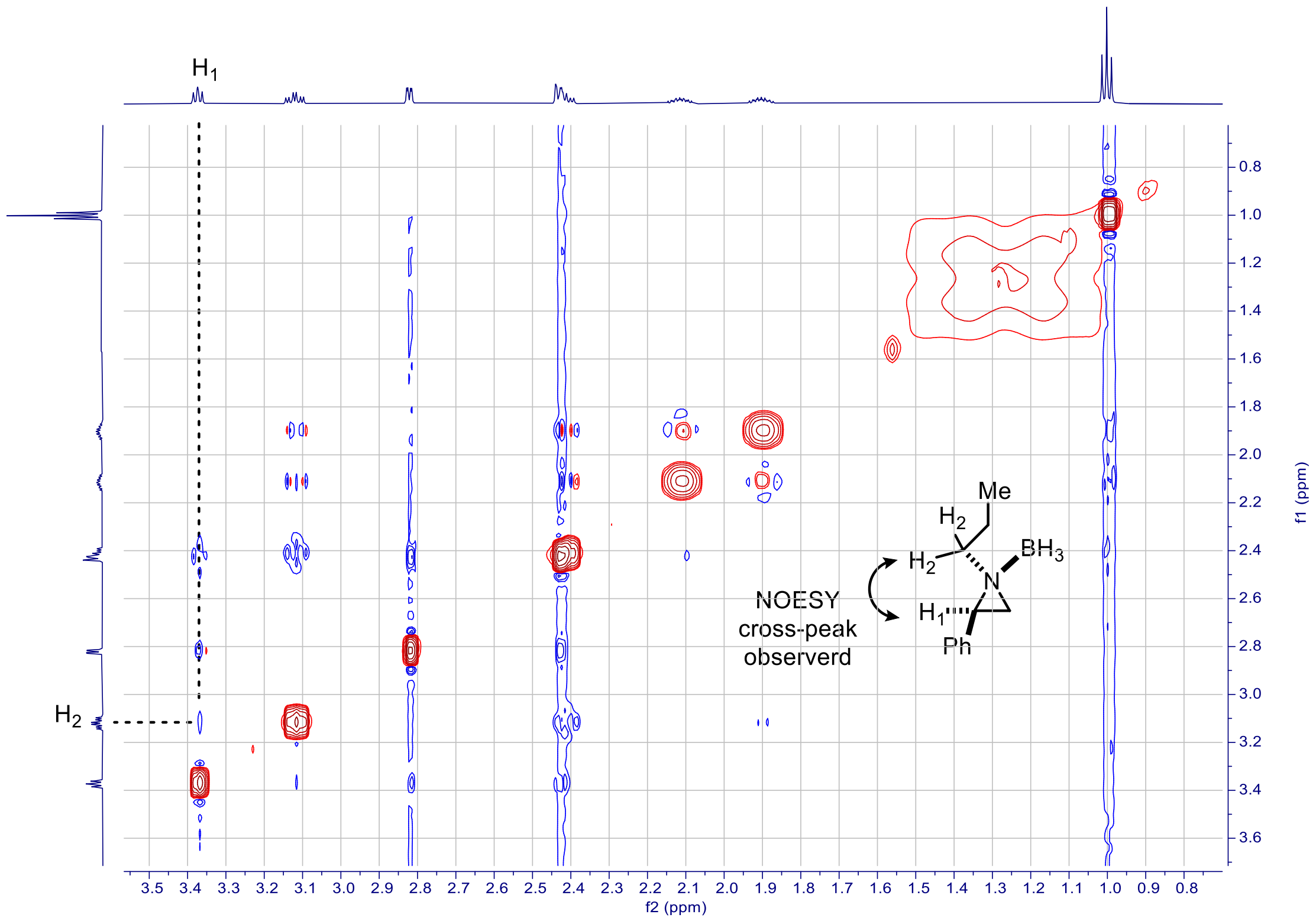

***cis*-2-(4-(*tert*-Butyl)phenyl)-1-propylaziridine Borane (2a) and *trans*-2-(4-(*tert*-Butyl)phenyl)-1-propylaziridine Borane (2a')**

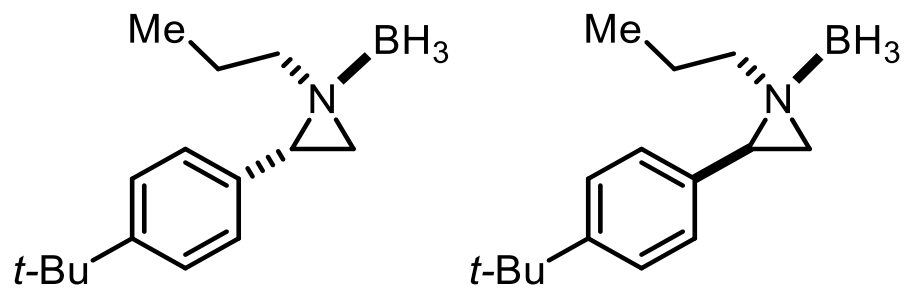

Following **GP4**, but adding BH_3•DMS at -78 ºC, **2** (1.09 g, 5.0 mmol, 1.0 equiv.) gave after purification via column chromatography **2a** (518 mg, 45%) and **2a'** (379 mg, 33%) as separable mixture of diastereomers. **2a**: **2a'** = 58:42.

Data for **2a** (solid): R_f 0.43 [pentane:EtOAc (10:1)]. ^{1}H (600 MHz, $CDCl_3$) δ 7.38 – 7.32 (2H, m), 7.22 – 7.18 (2H, m), 3.67 (1H, t, J = 6.5 Hz), 2.55 (1H, dd, J = 7.0, 1.9 Hz), 2.39 (1H, dd, J = 6.2, 1.9 Hz), 2.31 (1H, ddd, J = 12.3, 10.7, 4.5 Hz), 2.09 – 1.38 (6H, m), 1.26 (9H, s), 0.63 (3H, t, J = 7.5 Hz); ^{13}C (151 MHz, $CDCl_3$) δ 152.9, 130.1, 126.9, 125.9, 56.7, 51.0, 39.3, 34.9, 31.3, 19.8, 11.4; ^{11}B (193 MHz, $CDCl_3$) δ –11.99. HRMS (ESI) found MNa^+ 254.2043, $C_{15}H_{26}BNNa$ requires 254.2051.

The relative configuration was determined by a 2D ^{1}H-^{1}H NOESY (600 MHz, $CDCl_3$) experiment:

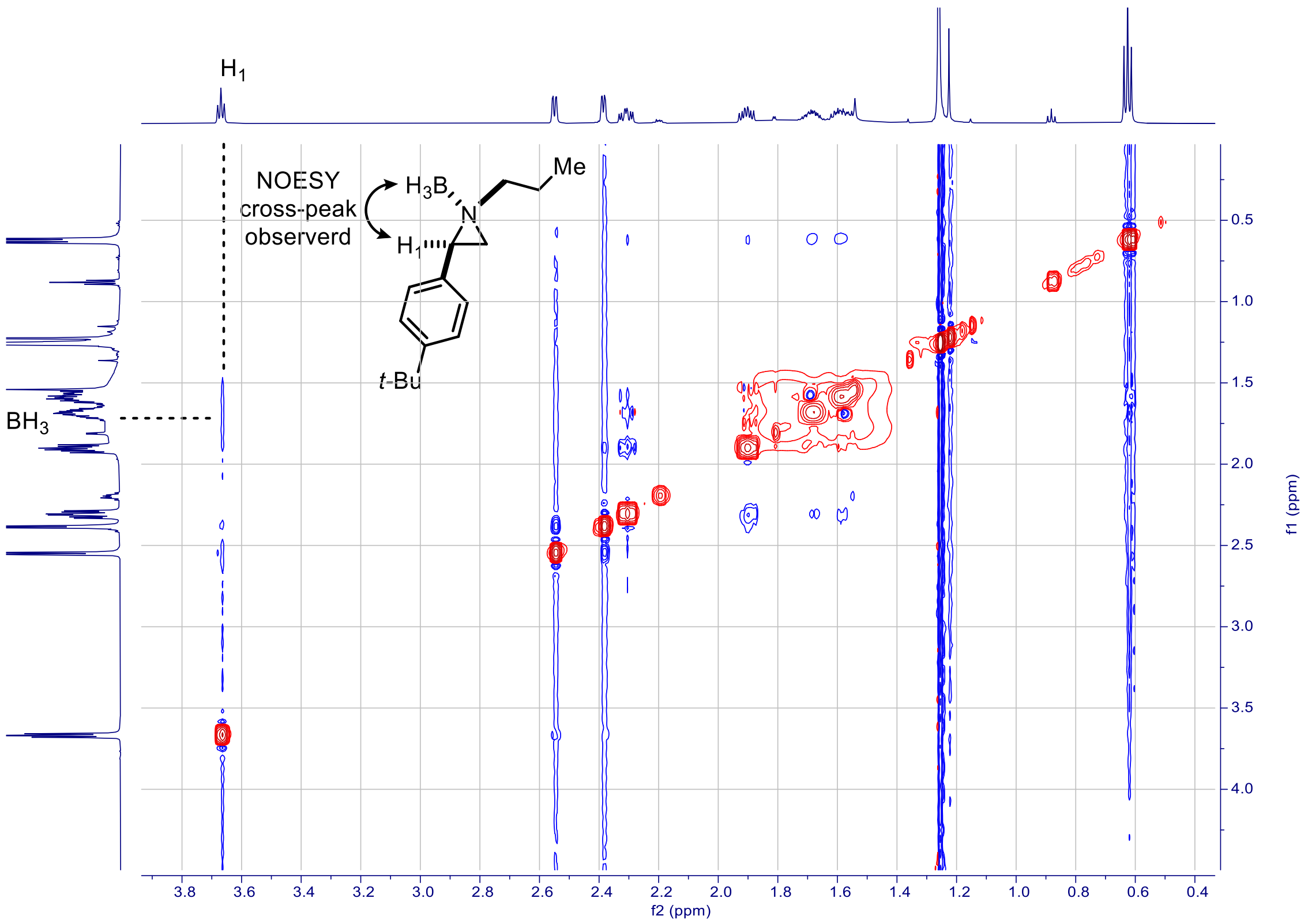

Data for **2a'** (solid): R_f 0.21 [pentane:EtOAc (10:1)]. ^{1}H (600 MHz, $CDCl_3$) δ 7.34 – 7.30 (2H, m), 7.22 – 7.17 (2H, m), 3.24 (1H, t, J = 7.0 Hz), 3.02 (1H, td, J = 11.4, 4.9 Hz), 2.72 (1H, dd, J = 6.3, 1.9 Hz), 2.35 – 2.26 (2H, m), 2.07 – 1.95 (1H, m), 1.87 – 1.75 (1H, m), 1.25 (12H, m), 0.90 (3H, t, J = 7.4 Hz); ^{13}C (151 MHz, $CDCl_3$) δ 152.0, 129.9, 128.0, 125.2, 67.1, 49.5, 41.8, 34.8,

31.4, 20.3, 11.5; ^{11}B (193 MHz, $CDCl_3$) δ –16.43. HRMS (ESI) found MNa^+ 254.2044, $C_{15}H_{26}BNNa$ requires 254.2051.

The relative configuration was determined by a 2D ^1H-^1H NOESY (600 MHz, $CDCl_3$) experiment:

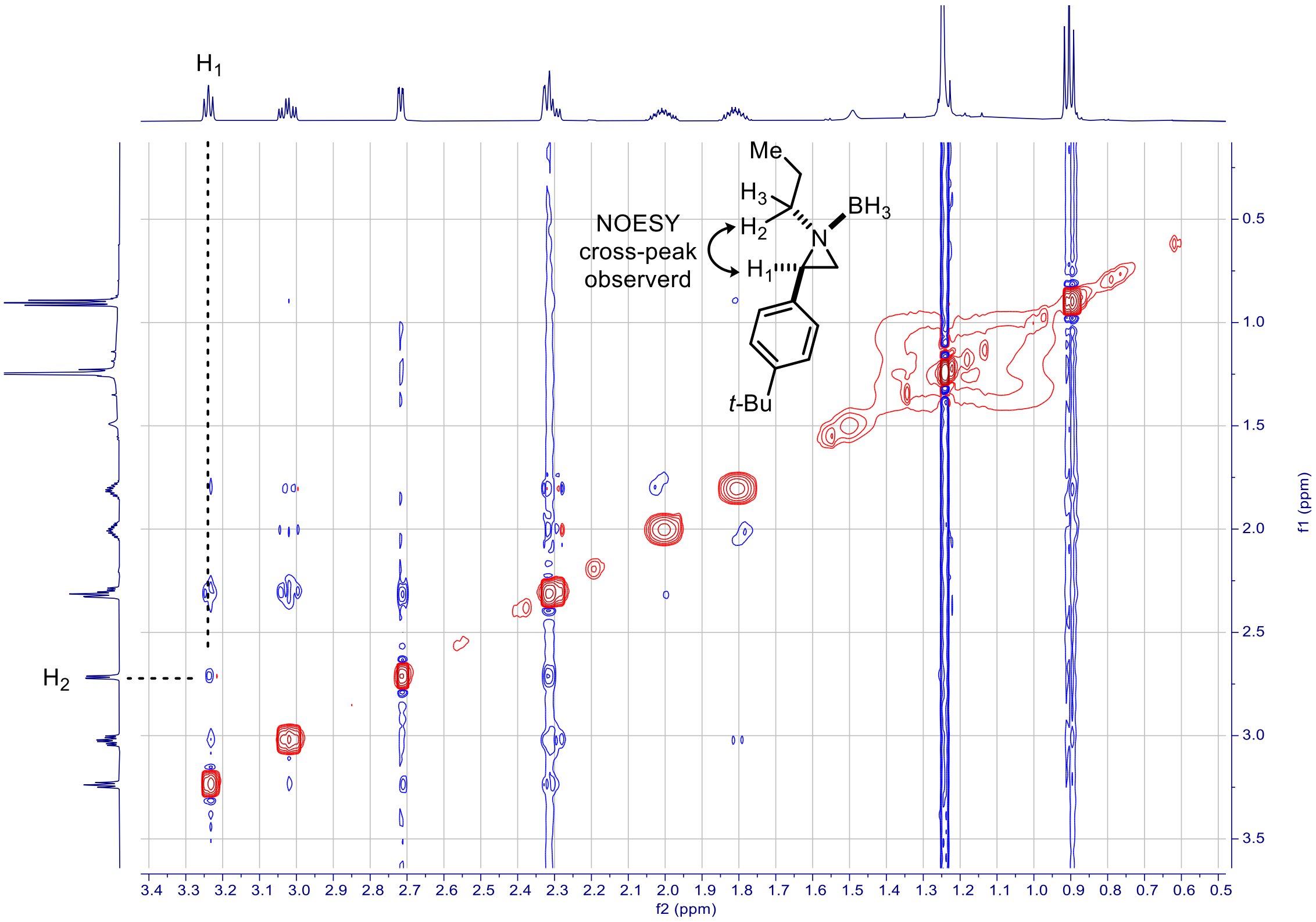

cis-2-(4-(tert-Butyl)phenyl)-1-propylaziridine Borane (3a) and trans-2-(4-(tert-Butyl)phenyl)-1-propylaziridine Borane (3a')

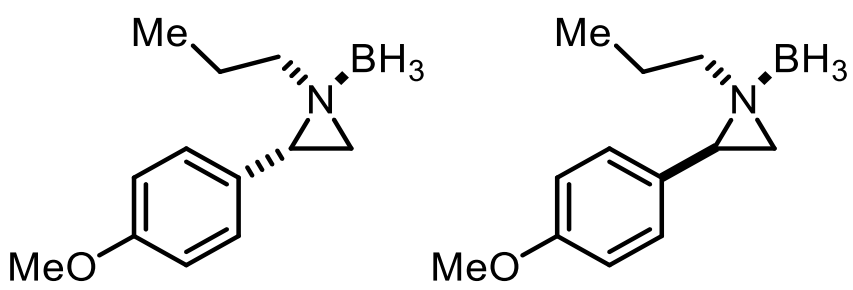

Following **GP4**, but adding BH_3•DMS at -78 ºC, **3** (0.96 g, 5.0 mmol, 1.0 equiv.) gave after purification via column chromatography **3a** (297 mg, 29%) and **3a'** (626 mg, 61%) as separable mixture of diastereomers. **3a**: **3a'** = 32:68.

<u>Data for **3a** (oil)</u>: R_f 0.6 [*n*-pentane:EtOAc (3:1)]. ^1H (600 MHz, $CDCl_3$) δ 7.22 – 7.17 (2H, m), 6.88 – 6.83 (2H, m), 3.76 (3H, s), 3.65 (1H, t, J = 6.5 Hz), 2.54 (1H, dd, J = 7.0, 2.0 Hz), 2.35 (1H, dd, J = 6.2, 1.9 Hz), 2.26 (1H, ddd, J = 12.3, 10.8, 4.7 Hz), 1.91 (1H, ddd, J = 12.3, 10.8, 5.8 Hz), 1.84 – 1.39 (5H, m), 0.62 (3H, t, J = 7.5 Hz); ^{13}C (151 MHz, $CDCl_3$) δ 160.6, 131.7, 121.9, 114.4, 56.6, 55.5, 50.9, 39.4, 19.7, 11.5; ^{11}B (193 MHz, $CDCl_3$) δ –12.00 (q, J = 103.3 Hz); HRMS (ESI) found MNa^+ 228.1523, $C_{12}H_{20}BNONa$ requires 228.1530.

The relative configuration was determined by a 2D ^1H-^1H NOESY (600 MHz, $CDCl_3$) experiment:

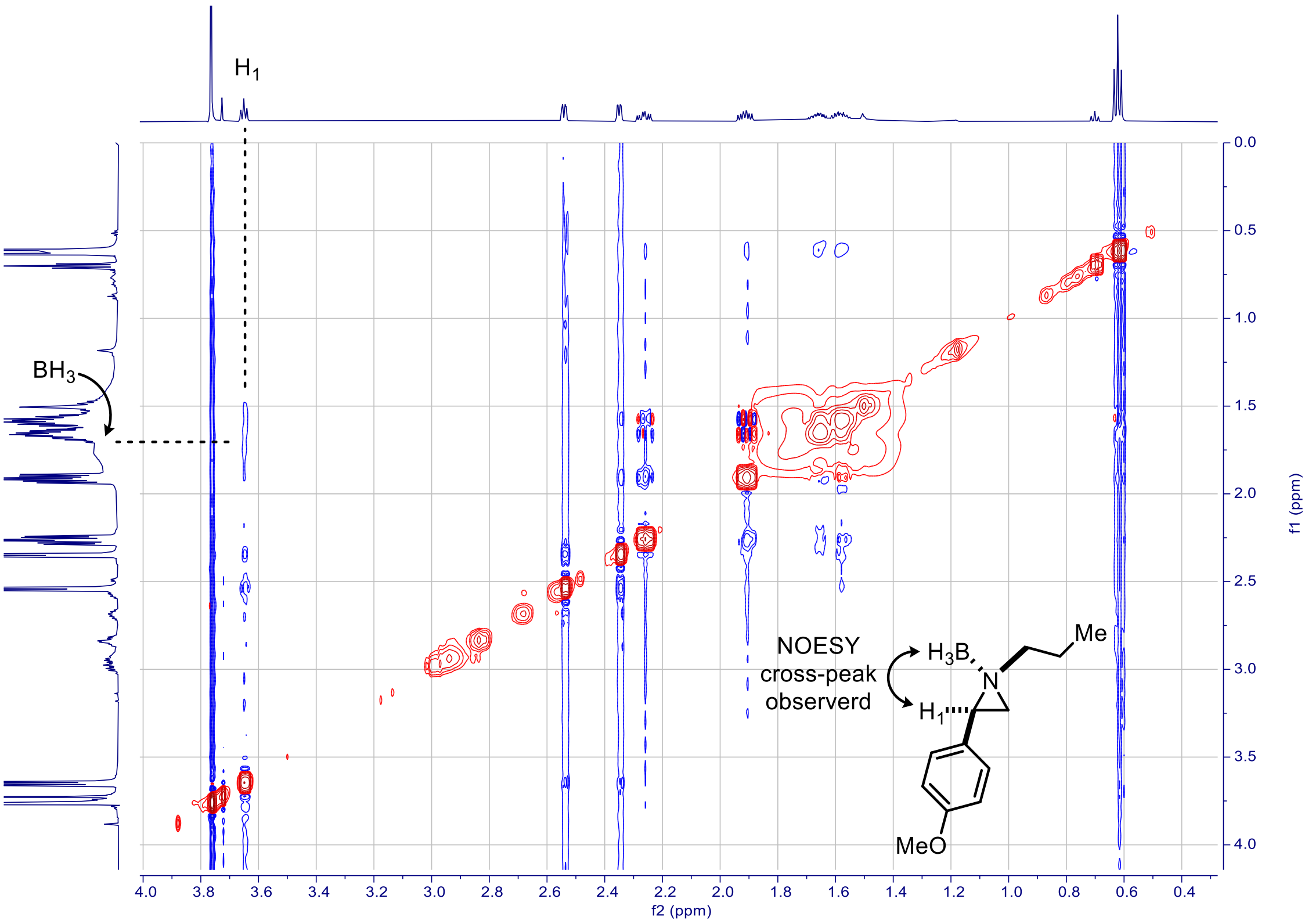

Data for **3a'** (oil): R_f 0.45 [*n*-pentane:EtOAc (3:1)]. ^{1}H (600 MHz, $CDCl_3$) δ 7.30 – 7.23 (2H, m), 6.94 – 6.90 (2H, m), 3.83 (3H, s), 3.32 (1H, dd, *J* = 7.7, 6.3 Hz), 3.10 (1H, td, *J* = 11.5, 4.9 Hz), 2.79 (1H, dd, *J* = 6.3, 2.0 Hz), 2.44 – 2.34 (2H, m), 2.15 – 2.02 (1H, m), 1.95 – 1.83 (1H, m), 0.99 (6H, m); ^{13}C (151 MHz, $CDCl_3$) δ 160.17, 131.51, 122.99, 113.71, 66.97, 55.37, 49.32, 41.46, 20.34, 11.45; ^{11}B (193 MHz, $CDCl_3$) δ –16.49 (q, *J* = 97.8 Hz); HRMS (ESI) found MNa^+ 228.1529, $C_{12}H_{20}BNONa$ requires 228.1530.

The relative configuration was determined by a 2D ^{1}H-^{1}H NOESY (600 MHz, $CDCl_3$) experiment:

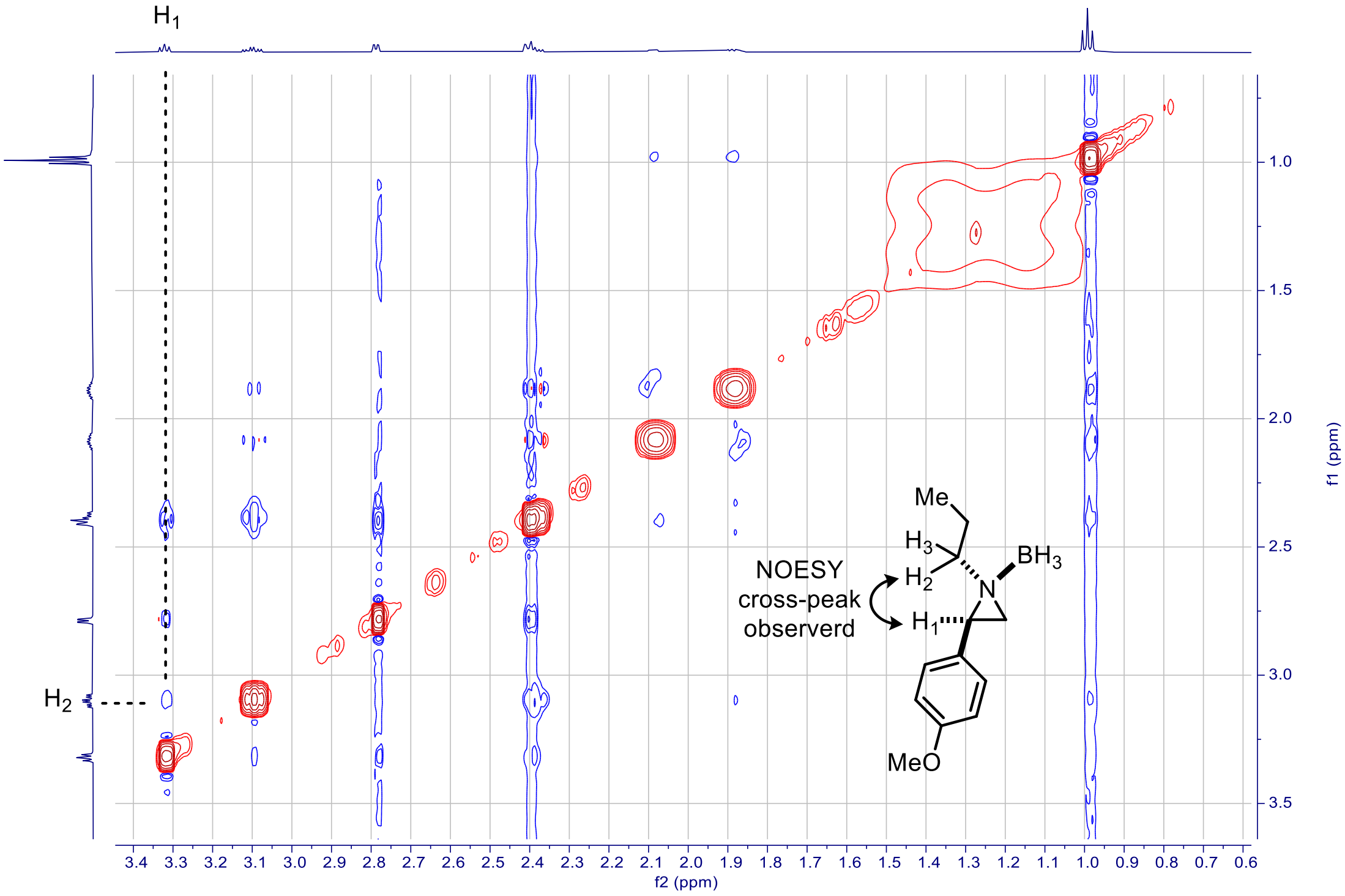

***trans*-2-(4-Bromophenyl)-1-propylaziridine Borane (4a)**

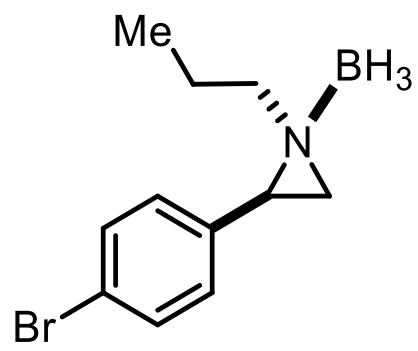

Following **GP4**, but adding BH_3•DMS at -78 °C and react at r.t. for 4 h, **4** (1.57 g, 6.56 mmol, 1.0 equiv.) gave after precipitation and filtration **4a** (1.45 g, 87%) as a solid. ^{1}H NMR (400 MHz, $CDCl_3$) δ 7.50 (2H, d, J = 7.9 Hz), 7.21 (2H, d, J = 8.0 Hz), 3.29 (1H, t, J = 7.0 Hz), 3.10 (1H, td, J = 11.6, 4.9 Hz), 2.77 (1H, d, J = 6.2 Hz), 2.48 – 2.33 (2H, m), 2.17 – 2.01 (1H, m), 1.94 – 1.79 (1H, m), 1.64 – 0.75 (6H, m); ^{13}C NMR (101 MHz, $CDCl_3$) δ 131.8, 131.5, 130.1, 123.5, 67.1, 48.9, 41.5, 20.4, 11.4; ^{11}B NMR (128 MHz, $CDCl_3$) δ –16.21; HRMS (ESI) found MNa$^+$ 276.0533, $C_{11}H_{17}NBBrNa$ requires 276.0530.

The relative configuration was determined by ^{1}H NMR and ^{11}B NMR shift in analogy with the shift differences observed for **3a** and **3a'**.

trans-2-(4-Chlorophenyl)-1-propylaziridine Borane (5a)

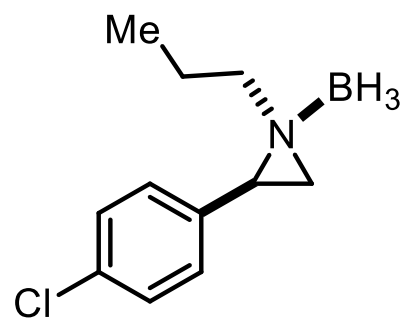

Following **GP4**, but adding BH_3•DMS at -78 °C and react at r.t. for 4 h, **5** (2.17 g, 11.1 mmol, 1.0 equiv.) gave after precipitation and filtration **5a** (2.08, 87%) as a solid. ^{1}H NMR (400 MHz, $CDCl_3$) δ 7.39 – 7.31 (2H, m), 7.27 (2H, dd, J = 7.2, 1.7 Hz), 3.31 (1H, t, J = 7.0 Hz), 3.10 (1H, td, J = 11.3, 4.8 Hz), 2.84 – 2.73 (1H, m), 2.50 – 2.33 (2H, m), 2.18 – 2.00 (1H, m), 1.95 – 1.78 (1H, m), 1.03 – 0.92 (3H, m); ^{13}C NMR (101 MHz, $CDCl_3$) δ 135.2, 131.6, 129.6, 128.6, 67.1, 48.8, 41.5, 20.4, 11.4; ^{11}B NMR (128 MHz, $CDCl_3$) δ –16.22; HRMS (ESI) found MNa^+ 232.1037, $C_{11}H_{17}NBClNa$ requires 232.1035.

The relative configuration was determined by ^{1}H NMR and ^{11}B NMR shift in analogy with the shift differences observed for **3a** and **3a'**.

cis-1-Propyl-2-(4-(trifluoromethyl)phenyl)aziridine Borane (6a) and _trans_-1-Propyl-2-(4-(trifluoromethyl)phenyl)aziridine Borane (6a')

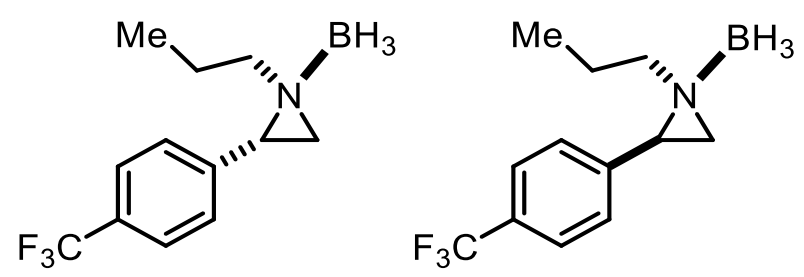

Following **GP4**, but adding BH_3•DMS at -78 °C, **6** (917 mg, 4.0 mmol, 1.0 equiv.) gave after purification via column chromatography **6a** (101 mg, 10%) and **6a'** (745 mg, 77%) as separable mixture of diastereomers. **6a**: **6a'** = 11:89.

<u>Data for **6a** (solid)</u>: ^{1}H (600 MHz, $CDCl_3$) δ 7.70 (2H, d, J = 7.9 Hz), 7.50 (2H, d, J = 8.0 Hz), 3.82 (1H, t, J = 6.5 Hz), 2.72 (1H, dd, J = 6.8, 2.0 Hz), 2.51 (1H, dd, J = 6.2, 2.0 Hz), 2.42 (1H, td, J = 11.5, 4.4 Hz), 2.09 – 1.48 (6H, m), 0.71 (3H, t, J = 7.4 Hz); ^{13}C (151 MHz, $CDCl_3$) δ 134.1, 132.0 (q, J = 32.8 Hz), 130.8, 126.03 (q, J = 3.8 Hz), 123.8 (d, J = 272.4 Hz), 57.4, 50.5, 39.4, 19.8, 11.3; ^{11}B (193 MHz, $CDCl_3$) δ –11.46 (q, J = 100.7 Hz); ^{19}F (565 MHz, $CDCl_3$) δ –62.88; HRMS (ESI) found MNa^+ 266.1298, $C_{12}H_{17}BNF_3Na$ requires 266.1298.

The relative configuration was determined by a 2D ^{1}H-^{1}H NOESY (600 MHz, $CDCl_3$) experiment:

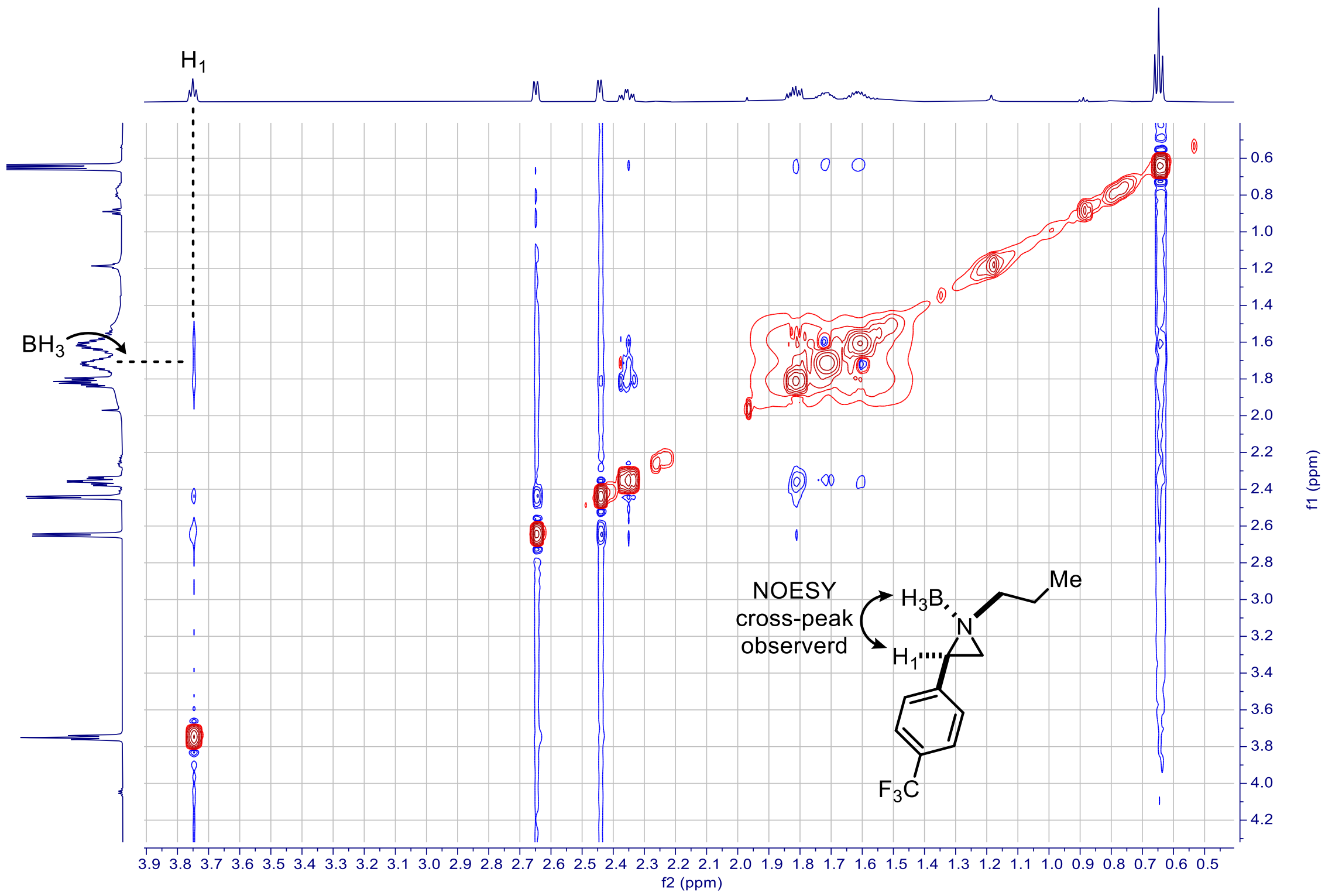

<u>Data for **6a'** (solid)</u>: R_f 0.3 [*n*-pentane:EtOAc (6:1)]. ^{1}H (600 MHz, $CDCl_3$) δ 7.63 (2H, d, *J* = 8.1 Hz), 7.46 (2H, d, *J* = 8.0 Hz), 3.38 (1H, t, *J* = 6.9 Hz), 3.11 (1H, td, *J* = 11.5, 4.9 Hz), 2.82 (1H, dd, *J* = 6.2, 1.9 Hz), 2.50 – 2.40 (2H, m), 2.17 – 2.05 (1H, m), 1.93 – 1.82 (1H, m), 1.52 – 0.90 (6H, m); ^{13}C (151 MHz, $CDCl_3$) δ 135.0, 131.1 (q, *J* = 32.6 Hz), 130.6, 125.3 (q, *J* = 3.7 Hz), 124.1 (d, *J* = 272.3 Hz), 67.1, 48.7, 41.5, 20.4, 11.4; ^{11}B (193 MHz, $CDCl_3$) δ –15.88 (q, *J* = 103.7 Hz); ^{19}F (565 MHz, $CDCl_3$) δ –62.74; HRMS (ESI) found MNa^+ 266.1294, $C_{12}H_{17}BNF_3Na$ requires 266.1298.

The relative configuration was determined by a 2D ^{1}H-^{1}H NOESY (600 MHz, $CDCl_3$) experiment:

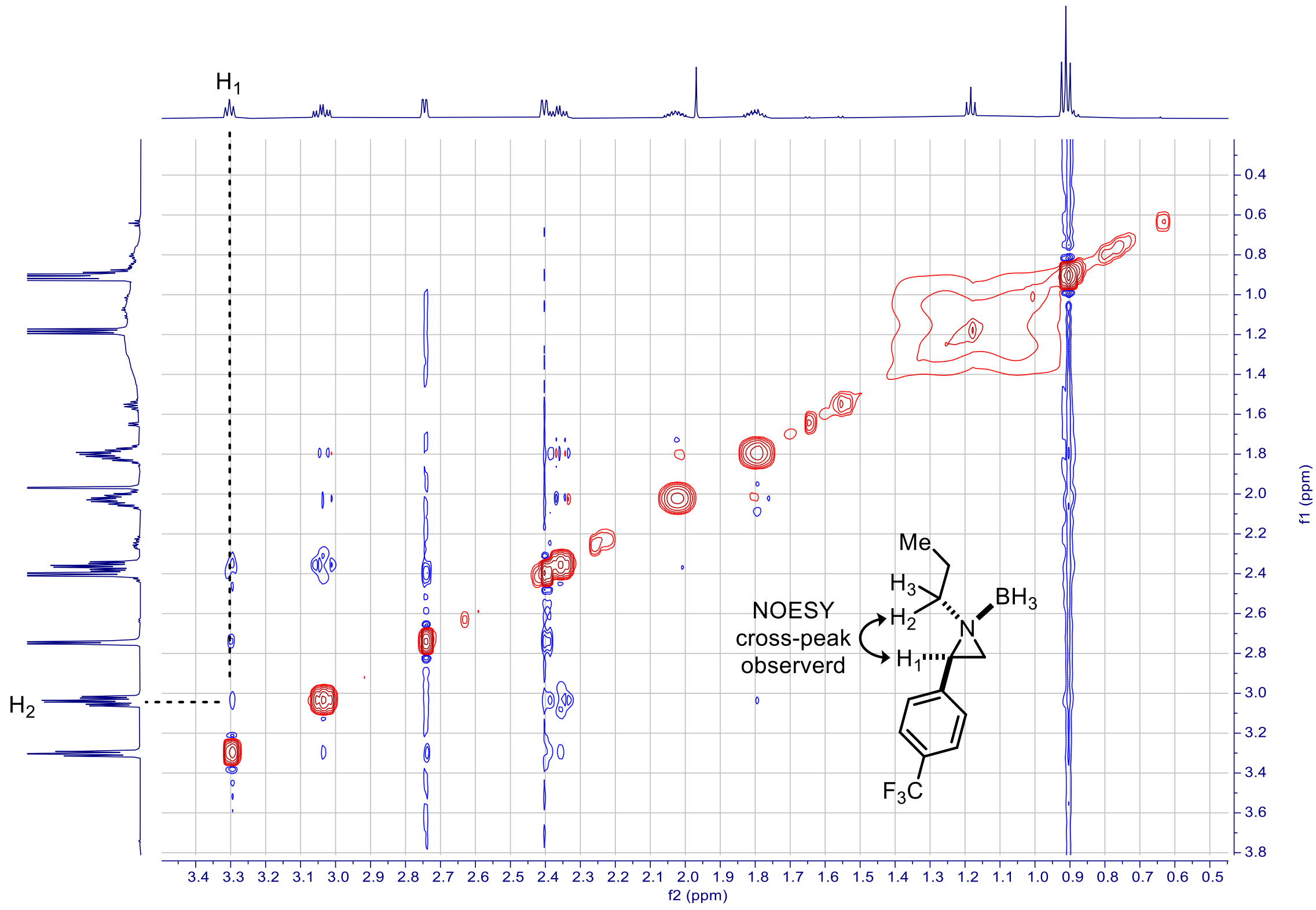

trans-2-(Bicyclo[4.2.0]octa-1(6),2,4-trien-3-yl)-1-propylaziridine Borane (7a)

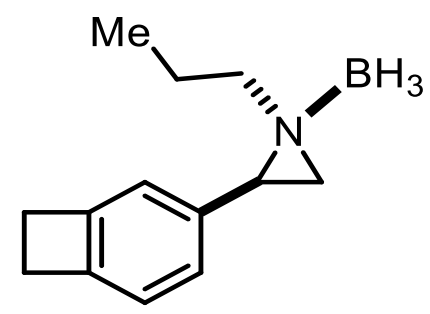

Following **GP4**, but adding BH_3•DMS at -78 °C and react at r.t. for 4 h, **7** (360 mg, 1.92 mmol, 1.0 equiv.) gave after precipitation and filtration **7a** (300 mg, 77%) as a solid. ^{1}H NMR (600 MHz, $CDCl_3$) δ 7.15 (1H, d, *J* = 7.6 Hz), 7.05 (1H, d, *J* = 7.6 Hz), 7.02 (1H, s), 3.34 (1H, t, *J* = 7.0 Hz), 3.17 (4H, s), 3.09 (1H, td, *J* = 11.6, 4.8 Hz), 2.79 (1H, dd, *J* = 6.0, 2.1 Hz), 2.43 – 2.33 (2H, m), 2.15 – 2.02 (1H, m), 1.95 – 1.81 (1H, m), 1.45 – 0.90 (6H, m); ^{13}C NMR (151 MHz, $CDCl_3$) δ 146.9, 145.7, 129.4, 129.0, 124.3, 122.4, 67.1, 50.3, 41.5, 29.7, 29.5, 20.4, 11.5; ^{11}B NMR (192 MHz, $CDCl_3$) δ –16.45; HRMS (ESI) found MNa^+ 224.1581, $C_{13}H_{20}NBNa$ requires 224.1581. The relative configuration was determined by ^{1}H NMR and ^{11}B NMR shift in analogy with the shift differences observed for **3a** and **3a’**.

trans-1-Methyl-2-phenylaziridine Borane (8a)

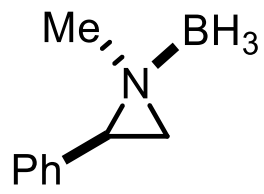

Following **GP4**, but adding BH_3•DMS at -78 °C, **8** (666 mg, 5.0 mmol, 1.0 equiv.) gave after precipitation and filtration **8a** (436 mg, 59%) as a white solid. ^{1}H NMR (600 MHz, $CDCl_3$) δ 7.41 – 7.37 (3H, m), 7.36 – 7.32 (2H, m), 3.41 (1H, dd, J = 7.6, 6.2 Hz), 2.87 (3H, s), 2.78 (1H, dd, J = 6.3, 1.9 Hz), 2.42 (1H, dd, J = 7.7, 1.9 Hz), 1.30 (3H, q, J = 110.5 Hz); ^{13}C NMR (151 MHz, $CDCl_3$) δ 131.0, 130.2, 129.2, 128.3, 52.6, 50.5, 40.9; ^{11}B (193 MHz, $CDCl_3$) δ –14.85 (q, J = 97.4 Hz). HRMS (ESI) found MNa^+170.1107, $C_9H_{14}BNNa$ requires 170.1112.

The relative configuration was determined by a 2D ^{1}H/^{1}H NOESY (600 MHz, $CDCl_3$) experiment:

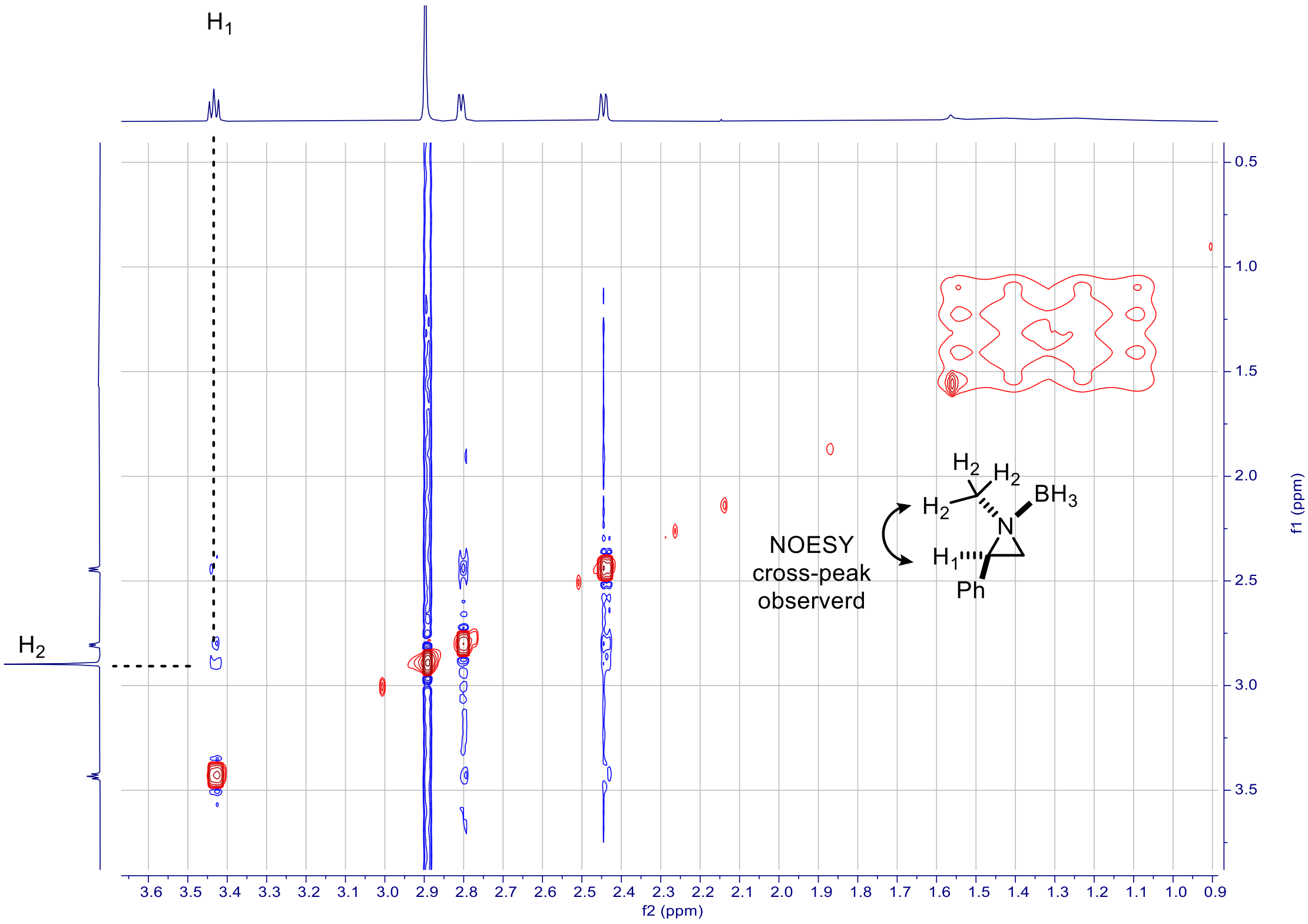

cis-1-Hexyl-2-phenylaziridine Borane (9a) and _trans_-1-Hexyl-2-phenylaziridine Borane (9a')

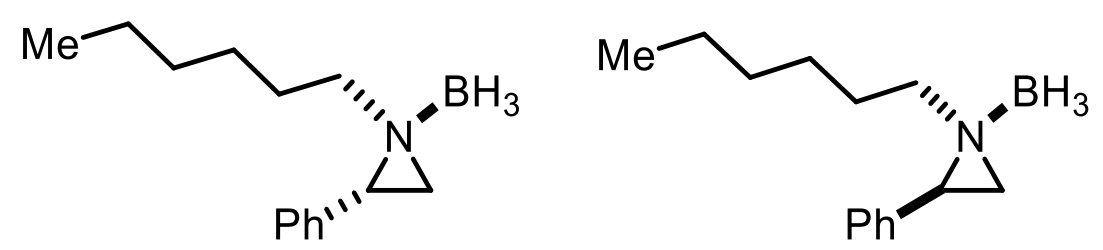

Following **GP4**, but adding BH_3•DMS at -78 ºC, **9** (1.02 g, 5.0 mmol, 1.0 equiv.) gave after purification via column chromatography **9a** (597 mg, 55%) and **9a'** (445 mg, 41%) as separable mixture of diasteromers. **9a**: **9a'** = 57:43.

Data for **9a** (colorless oil): R_f 0.5 [pentane:EtOAc (10:1)]. ^{1}H (400 MHz, $CDCl_3$) δ 7.45 – 7.39 (3H, m), 7.39 – 7.32 (2H, m), 3.78 (1H, t, J = 6.5 Hz), 2.64 (1H, dd, J = 7.0, 1.9 Hz), 2.47 (1H, dd, J = 6.1, 2.0 Hz), 2.37 (1H, ddd, J = 12.2, 10.4, 5.2 Hz), 2.18 – 1.44 (6H, m), 1.23 – 0.91 (6H, m), 0.79 (3H, t, J = 7.1 Hz); ^{13}C (151 MHz, $CDCl_3$) δ 130.4, 130.0, 129.7, 129.0, 55.3, 51.3, 39.3, 31.4, 26.6, 26.3, 22.5, 14.0; ^{11}B (193 MHz, $CDCl_3$) δ –11.75 (q, J = 95.9 Hz); HRMS (ESI) found MNa^+ 240.1889, $C_{14}H_{24}BNNa$ requires 240.1894.

The relative configuration was determined by a 2D ^{1}H/^{1}H NOESY (600 MHz, $CDCl_3$) experiment:

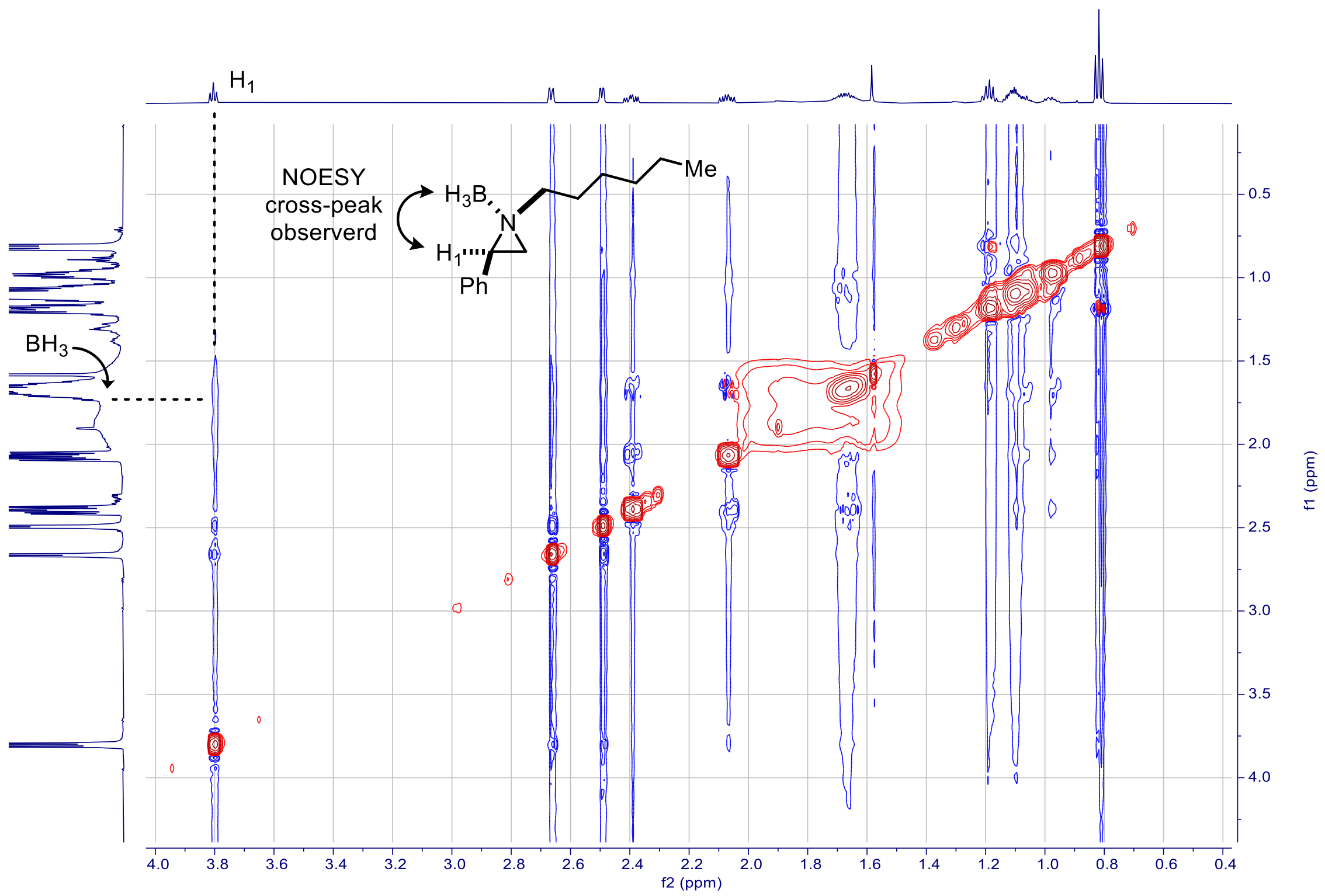

Data for **9a'** (white solid): R_f 0.35 [pentane:EtOAc (10:1)]. ^{1}H (600 MHz, $CDCl_3$) δ 7.40 – 7.31 (5H, m), 3.35 (1H, dd, J = 7.6, 6.2 Hz), 3.13 (1H, td, J = 11.6, 4.9 Hz), 2.81 (1H, dd, J = 6.2, 1.9 Hz), 2.46 – 2.38 (2H, m), 2.11 – 2.04 (1H, m), 1.88 – 1.80 (1H, m), 1.67 – 1.01 (9H, m), 0.94 – 0.89 (3H, m); ^{13}C (151 MHz, $CDCl_3$) δ 131.1, 130.2, 129.1, 128.3, 65.6, 49.5, 41.5, 31.7, 27.0,

26.8, 22.7, 14.1; ^{11}B (193 MHz, $CDCl_3$) δ –16.19 (q, J = 104.3 Hz); HRMS (ESI) found MNa^+ 240.1889, $C_{14}H_{24}BNNa$ requires 240.1894.

The relative configuration was determined by a 2D ^{1}H-^{1}H NOESY (600 MHz, $CDCl_3$) experiment:

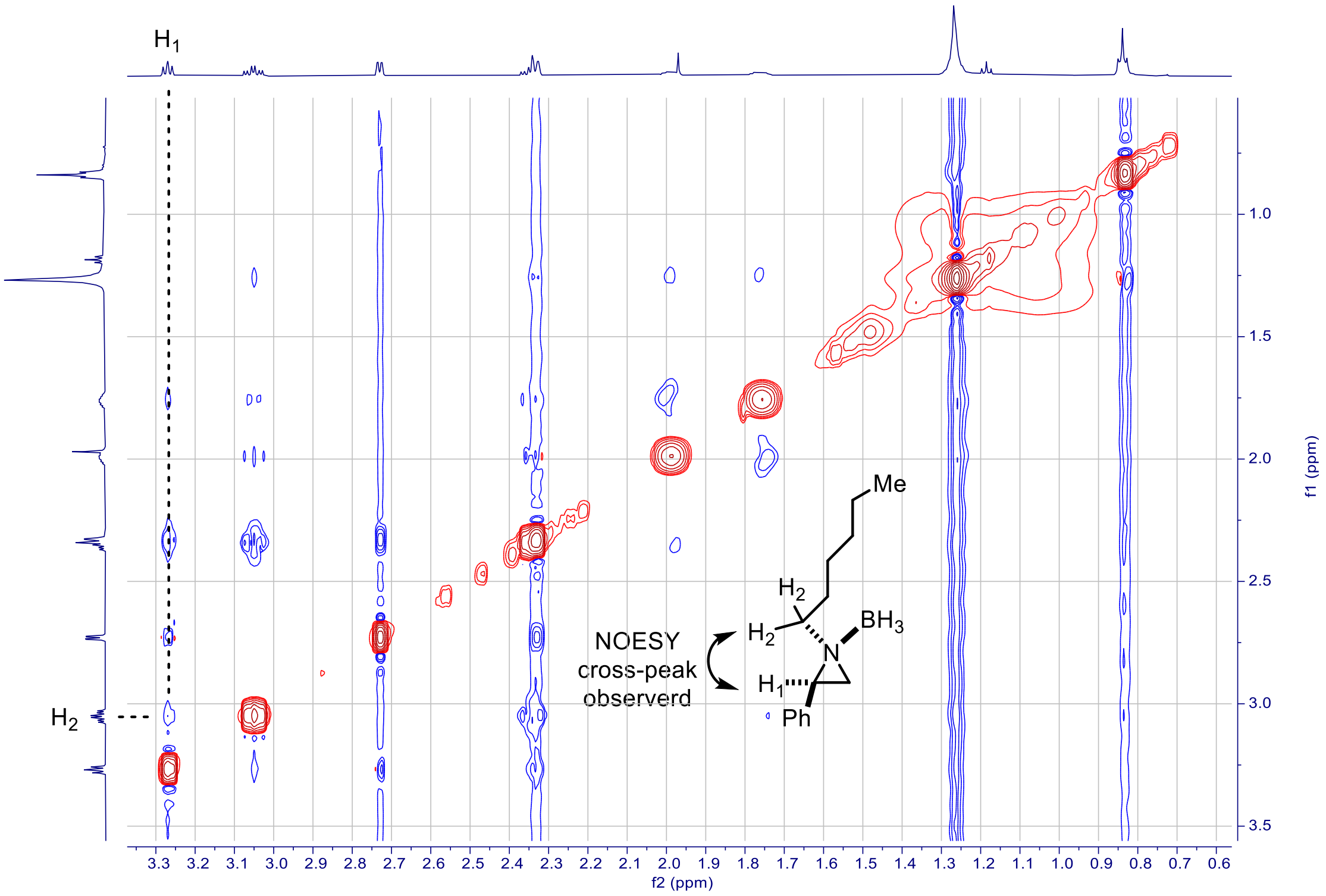

cis-1-Benzyl-2-phenylaziridine Borane (10a) and trans-1-Benzyl-2-phenylaziridine Borane (10a')

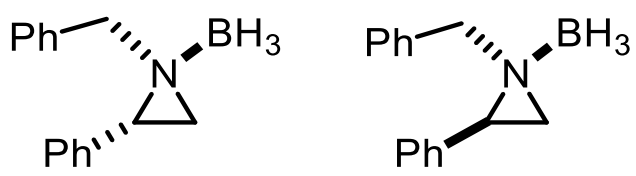

Following **GP4**, but adding BH_3•DMS at -78 °C, **10** (1.05 g, 5.0 mmol, 1.0 equiv.) gave after purification via column chromatography **10a** (63 mg, 6%) and **10a'** (725 mg, 65%) as separable mixture of diastereomers. **10a**: **10a'** = 8:92.

Data for **10a** (white solid): R_f 0.5 [pentane:EtOAc (10:1)]. ^{1}H (600 MHz, $CDCl_3$) δ 7.51 – 7.46 (3H, m), 7.43 – 7.38 (2H, m), 7.32 – 7.27 (3H, m), 7.22 – 7.17 (2H, m), 3.96 (1H, t, J = 6.6 Hz), 3.89 (1H, d, J = 13.6 Hz), 3.05 (1H, d, J = 13.6 Hz), 2.70 (1H, dd, J = 6.4, 2.3 Hz), 2.63 (1H, dd, J = 6.9, 2.3 Hz), 1.87 (3H, q, J = 116.0 Hz); ^{13}C (151 MHz, $CDCl_3$) δ 134.0, 130.6, 130.4, 130.1, 130.0, 129.3, 128.5, 128.4, 57.4, 53.0, 38.1; ^{11}B (193 MHz, $CDCl_3$) δ –10.18 (q, J = 95.9 Hz); HRMS (ESI) found MNa^+ 246.1424, $C_{15}H_{18}BNNa$ requires 246.1425.

The relative configuration was determined by a 2D ^{1}H-^{1}H NOESY (600 MHz, $CDCl_3$) experiment:

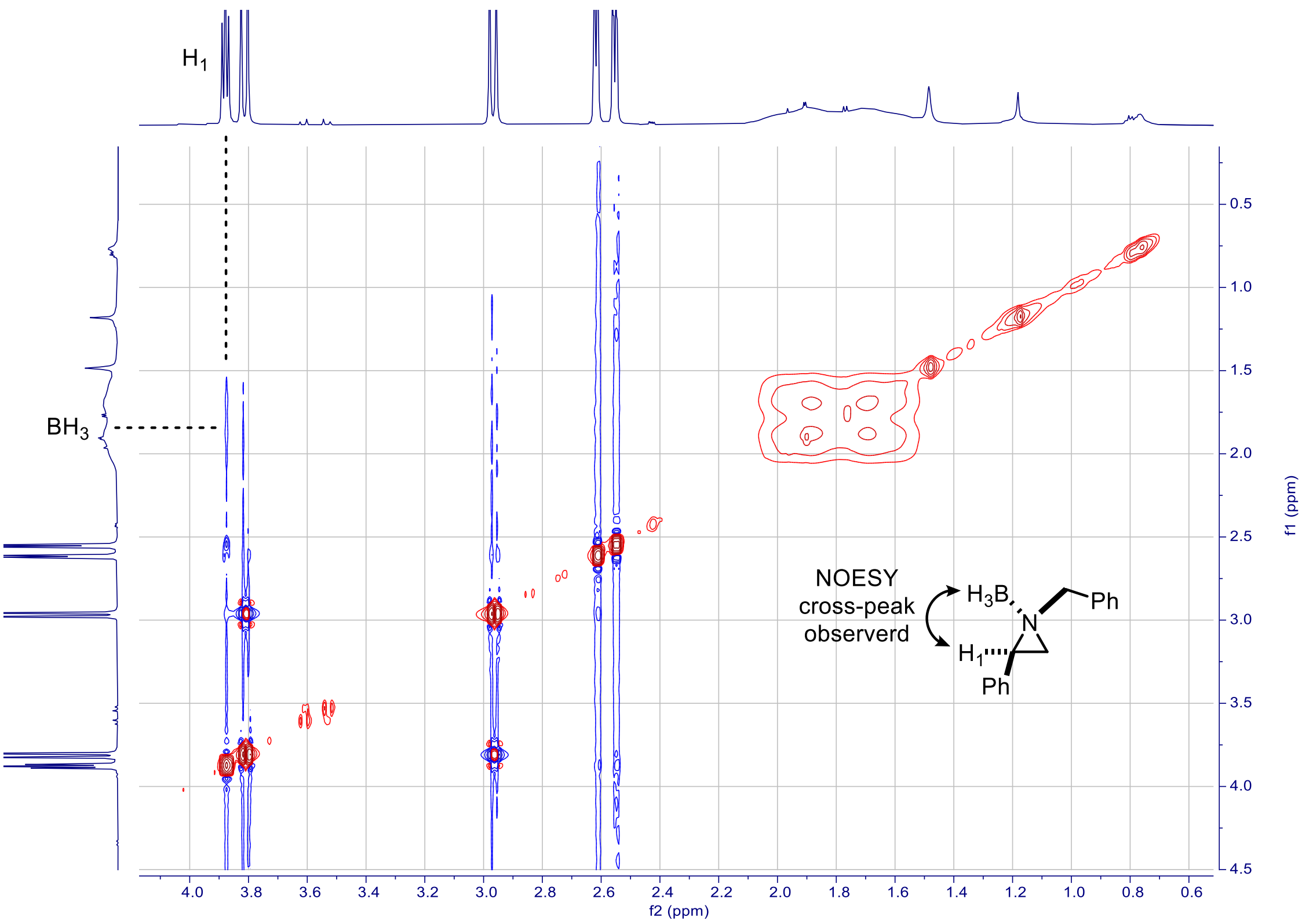

<u>Data for **10a’** (white solid)</u>: ^{1}H (600 MHz, $CDCl_3$) δ 7.48 – 7.42 (5H, m), 7.36 – 7.30 (3H, m), 7.22 –7.17 (2H, m), 4.41 (1H, d, *J* = 13.1 Hz), 3.94 (1H, d, *J* = 13.1 Hz), 3.48 (1H, t, *J* = 7.0 Hz), 2.81 (1H, dd, *J* = 6.3, 1.9 Hz), 2.52 (1H, dd, *J* = 7.8, 1.9 Hz), 1.40 (3H, q, *J* = 116.0 Hz); ^{13}C (151 MHz, $CDCl_3$) δ 132.6, 131.5, 131.0, 130.1, 129.3, 128.9, 128.9, 128.2, 66.1, 46.5 (d, *J* = 2.1 Hz), 39.1 (d, *J* = 2.6 Hz); ^{11}B (193 MHz, $CDCl_3$) δ –14.50 (q, *J* = 97.5 Hz); HRMS (ESI) found MNa^+ 246.1418, $C_{15}H_{18}BNNa$ requires 246.1425.

The relative configuration was determined by a 2D ^{1}H-^{1}H NOESY (600 MHz, $CDCl_3$) experiment:

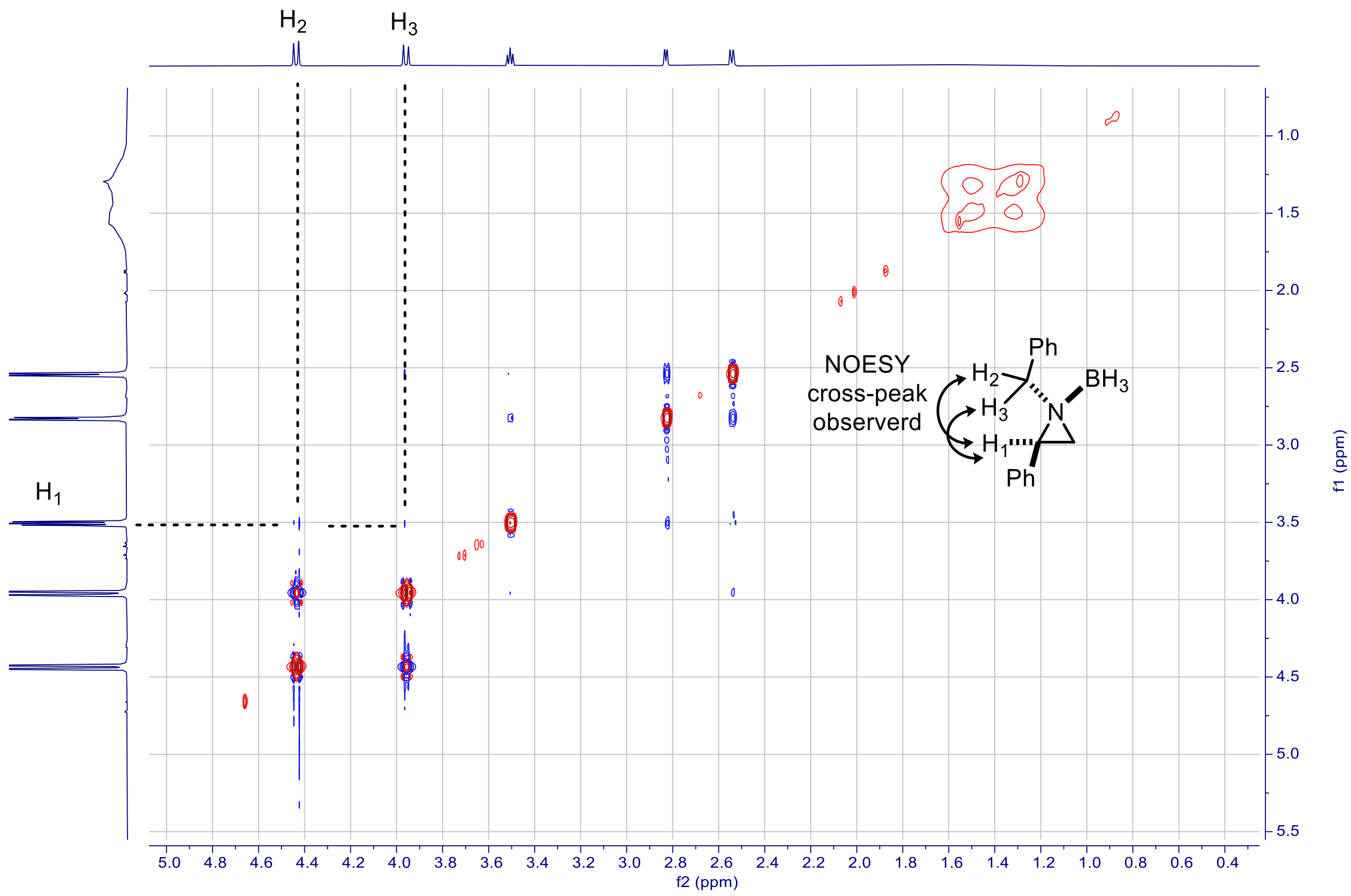

cis-1-Isopropyl-2-phenylaziridine Borane (11a)

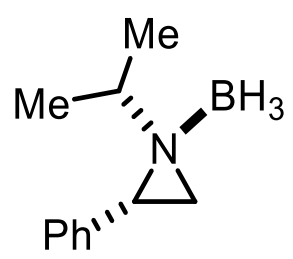

Following **GP4**, but adding BH_3•DMS at -78 °C, **11** (806 mg, 5.0 mmol, 1.0 equiv.) gave after purification via column chromatography **11a** (446 mg, 51%) as a white solid. R_f 0.59 [pentane:EtOAc (10:1)] ^{1}H (600 MHz, $CDCl_3$) δ 7.44 – 7.38 (5H, m), 3.82 (1H, t, J = 6.6 Hz), 2.66 (1H, dd, J = 6.9, 1.9 Hz), 2.47 (1H, dd, J = 6.3, 1.9 Hz), 2.12 (1H, sx, J = 6.5 Hz), 1.62 (1H, q, J = 111.4 Hz), 1.26 (3H, d, J = 6.6 Hz), 0.95 (3H, d, J = 6.5 Hz); ^{13}C (151 MHz, $CDCl_3$) δ 131.0, 129.7, 129.5, 128.8, 54.4, 52.6, 39.8, 19.5, 18.7; ^{11}B (193 MHz, $CDCl_3$) δ –15.23 (q, J = 98.4, 98.0 Hz); HRMS (ESI) found MNa^+ 198.1422, $C_{11}H_{18}BNNa$ requires 198.1425.

The relative configuration was determined by a 2D ^{1}H-^{1}H NOESY (600 MHz, $CDCl_3$) experiment:

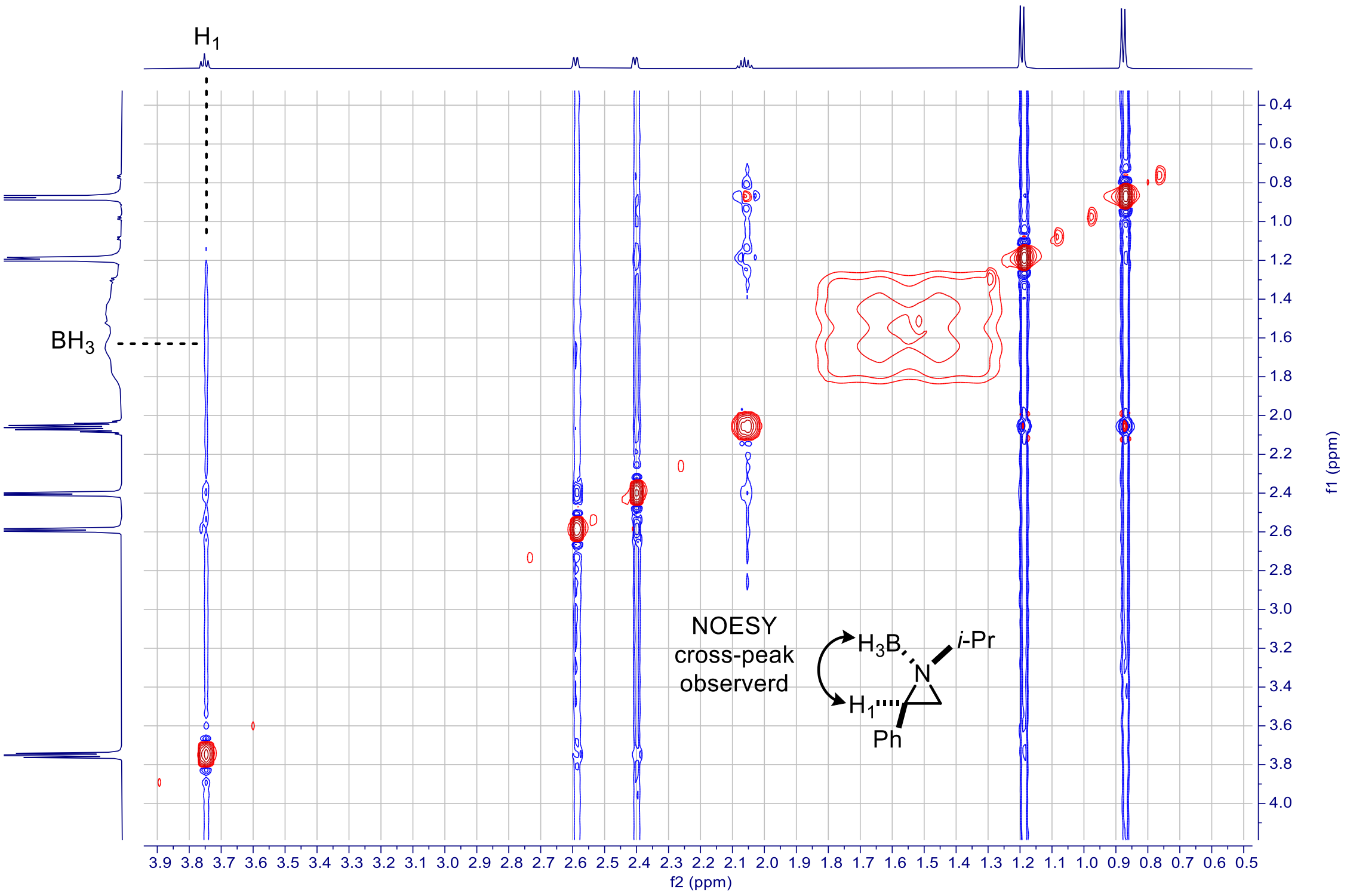

cis-1-Cyclohexyl-2-phenylaziridine Borane (12a) and _trans_-1-Cyclohexyl-2-phenylaziridine Borane (12a')

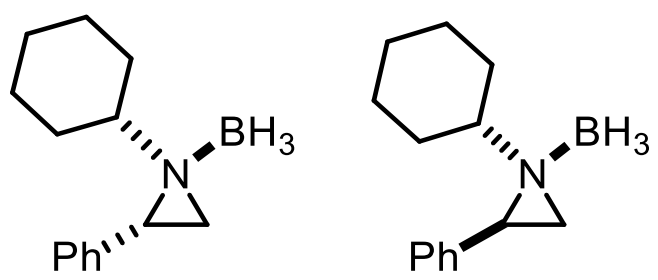

Following **GP4**, but adding BH_3•DMS at -78 ºC, **12** (1.07 g, 5.0 mmol, 1.0 equiv.) gave after purification via column chromatography **12a** (194 mg, 18%) and **12a'** (839 mg, 78%) as separable mixture of diastereomers. **12a**: **12a'** = 19:81.

<u>Data for **12a** (white solid)</u>: R_f 0.5 [*n*-pentane:EtOAc (10:1)]. ^{1}H (600 MHz, $CDCl_3$) δ 7.40 (5H, d, *J* = 3.3 Hz), 3.81 (1H, t, *J* = 6.6 Hz), 2.64 (1H, dd, *J* = 6.9, 1.9 Hz), 2.48 (1H, dd, *J* = 6.3, 1.9 Hz), 2.01 (1H, qd, *J* = 11.8, 3.1 Hz), 1.92 – 1.29 (10H, m), 1.11 – 0.94 (2H, m), 0.50 (1H, qt, *J* = 12.8, 3.5 Hz); ^{13}C (151 MHz, $CDCl_3$) δ 130.9, 129.7, 129.5, 128.7, 62.6, 52.3, 39.5, 29.4, 29.3, 25.7, 25.4, 25.1; ^{11}B (193 MHz, $CDCl_3$) δ –14.26 (q, *J* = 94.9 Hz); HRMS (ESI) found MNa^+ 238.1738, $C_{14}H_{24}BNNa$ requires 238.1738.

The relative configuration was determined by a 2D ^{1}H-^{1}H NOESY (600 MHz, $CDCl_3$) experiment:

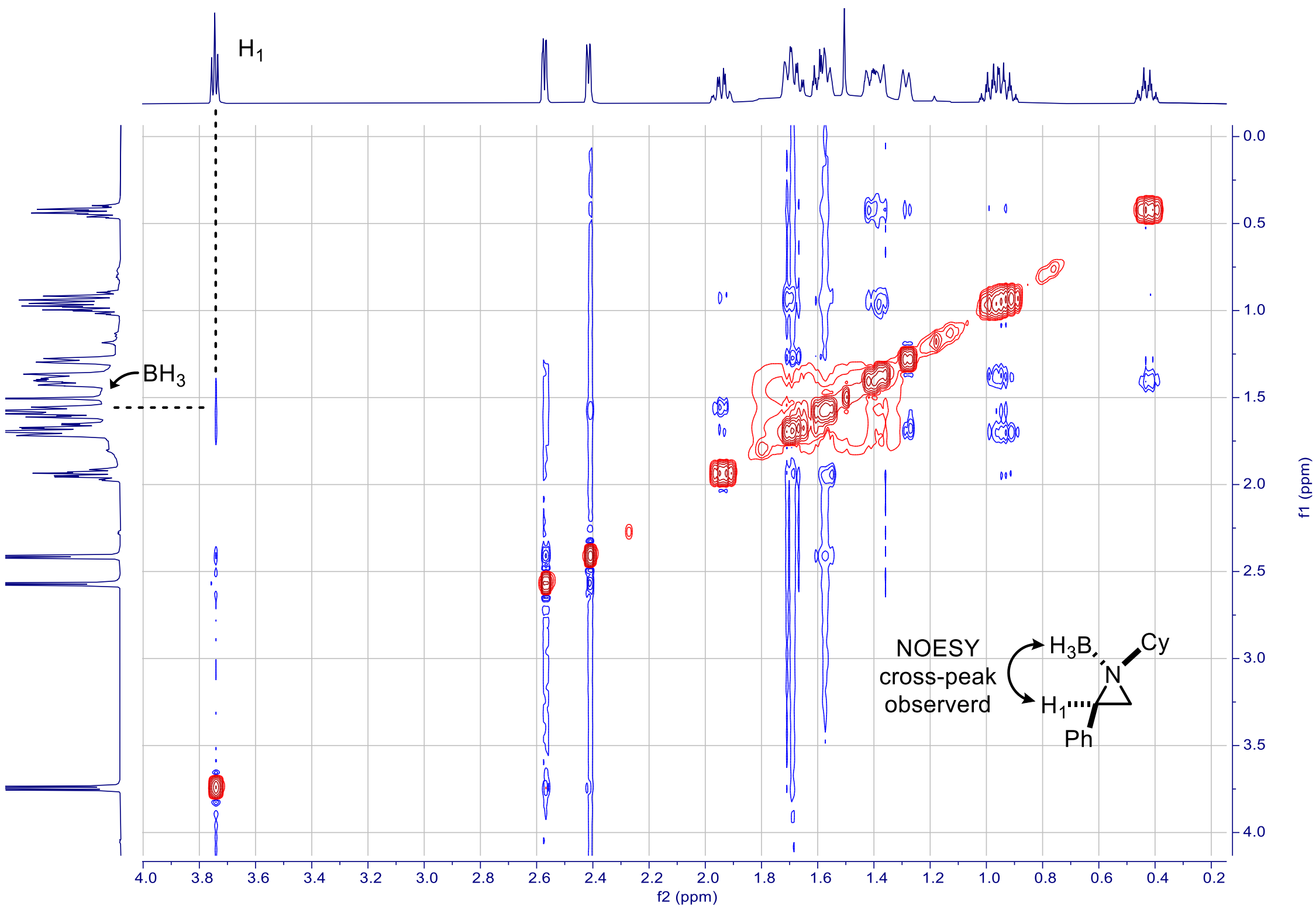

<u>Data for **12a’**</u> <u>(white solid)</u>: R_f 0.35 [*n*-pentane:EtOAc (10:1)]. ^{1}H (600 MHz, $CDCl_3$) δ 7.40 – 7.30 (5H, m), 3.29 (1H, dd, *J* = 7.7, 6.2 Hz), 2.76 (1H, dd, *J* = 6.2, 1.9 Hz), 2.39 (1H, dd, *J* = 7.8, 1.9 Hz), 2.13 – 1.96 (3H, m), 1.96 – 1.81 (4H, m), 1.69 – 1.64 (1H, m), 1.38 – 0.79 (6H, m); ^{13}C (151 MHz, $CDCl_3$) δ 131.18, 130.34, 128.88, 128.17, 73.79, 50.70, 40.37, 29.97, 29.32, 25.73(2C), 25.55; ^{11}B (193 MHz, $CDCl_3$) δ –18.87 (q, *J* = 92.5 Hz). HRMS (ESI) found MNa^+ 238.1731, $C_{14}H_{24}BNNa$ requires 238.1738.

The relative configuration was determined by a 2D ^{1}H-^{1}H NOESY (600 MHz, $CDCl_3$) experiment:

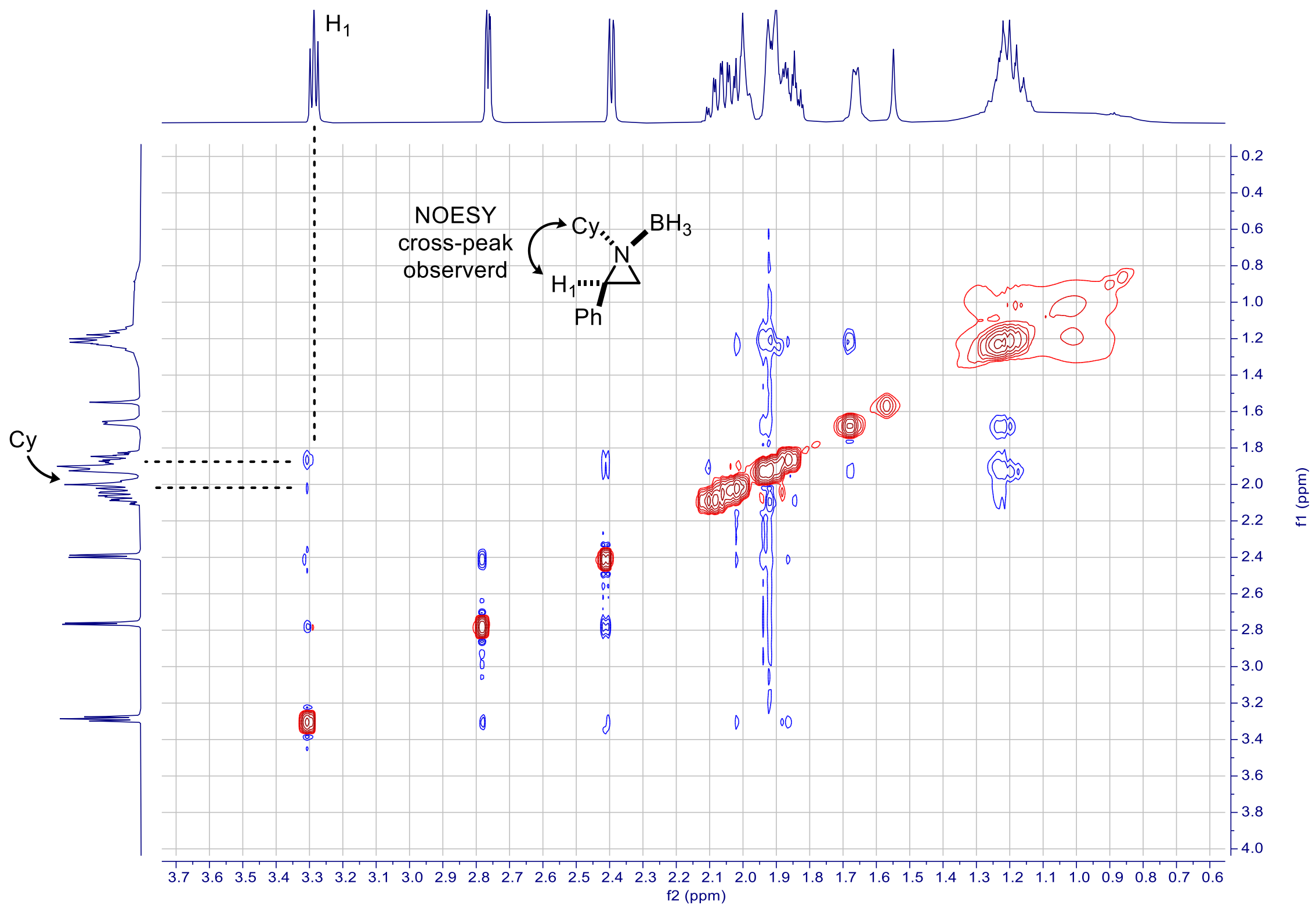

***trans*-1-Isopropyl-2-phenethylaziridine Borane (13a)**

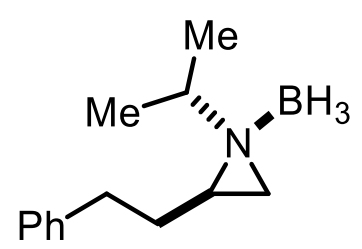

Following **GP4**, **13** (511 mg, 2.7 mmol, 1.0 equiv.) gave after purification via column chromatography **13a** (383 mg, 70%) as a solid. R_f 0.54 [*n*-pentane:EtOAc (10:1)]. ^{1}H (600 MHz, $CDCl_3$) δ 7.21 – 7.15 (2H, m), 7.15 – 7.12 (2H, m), 7.11 – 7.06 (1H, m), 2.86 (1H, ddd, *J* = 14.6, 9.6, 5.6 Hz), 2.63 (1H, ddd, *J* = 13.7, 9.4, 6.8 Hz), 2.32 – 2.23 (1H, m), 2.07 – 1.94 (1H, m), 1.93 – 1.86 (3H, m), 1.82 (1H, hp, *J* = 6.6 Hz), 1.18 (9H, m); ^{13}C (151 MHz, $CDCl_3$) δ 140.8, 128.4, 128.3, 126.0, 65.2, 48.1, 42.1, 33.8, 28.8, 19.0, 18.7; ^{11}B (193 MHz, $CDCl_3$) δ –20.37 (q, *J* = 99.4 Hz); HRMS (ESI) found MNa^+ 226.1731, $C_{17}H_{22}BNNa$ requires 226.1738.

The relative configuration was determined by ^{1}H NMR and ^{11}B NMR shift in analogy with the shift differences observed for **12a** and **12a’**.

***trans*-1-Propyl-2-(*p*-tolyl)aziridine Borane (14a)**

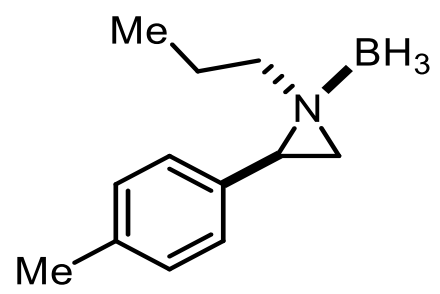

Following **GP4**, but adding BH_3•DMS at -78 °C and react at r.t. for 4 h, **14** (3.1 g, 14.00 mmol, 1.0 equiv.) gave after precipitation and filtration **14a** (2.31 g, 87%) as a solid. ^{1}H NMR (600 MHz, $CDCl_3$) δ 7.22 (2H, d, *J* = 8.0 Hz), 7.18 (2H, d, *J* = 7.9 Hz), 3.32 (1H, t, *J* = 6.9 Hz), 3.10 (1H, td, *J* = 11.5, 4.9 Hz), 2.80 (1H, dd, *J* = 6.3, 1.9 Hz), 2.42 – 2.37 (2H, m), 2.36 (3H, s), 2.14 – 2.03 (1H, m), 1.94 – 1.82 (1H, m), 0.97 (3H, t, *J* = 7.4 Hz); ^{13}C NMR (151 MHz, $CDCl_3$) δ 139.0, 130.1, 129.0, 127.9, 67.1, 49.5, 41.4, 21.5, 20.4, 11.5; ^{11}B NMR (192 MHz, $CDCl_3$) δ –16.55; HRMS (ESI) found MNa^+ 212.1586, $C_{12}H_{20}NBNa$ requires 212.1581.

The relative configuration was determined by ^{1}H NMR and ^{11}B NMR shift in analogy with the shift differences observed for **3a** and **3a'**.

***cis*-2-(4-Fluorophenyl)-1-propylaziridine Borane (15a) and *trans*-2-(4-Fluorophenyl)-1-propylaziridine Borane (15a')**

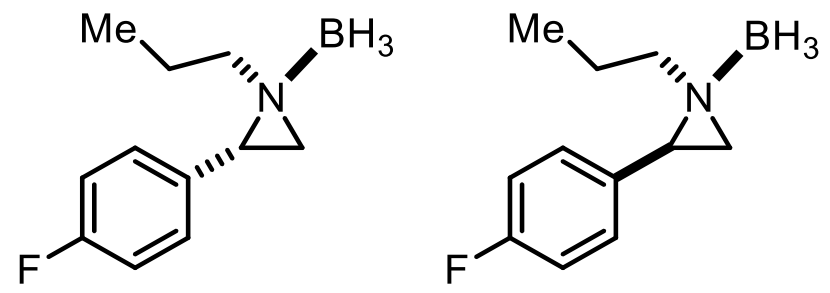

Following **GP4**, but adding BH_3•DMS at -78 °C and react at r.t. for 4 h, **15** (2.46 g, 13 mmol, 1.0 equiv.) gave after precipitation and filtration **15a** (12%) and **15a'** (77%) as mixture of diastereomers as a solid (2.23 g, 89% total yield; the mixture was used directly in the next step). Mixture of 2 diastereomers, **15a** and **15a'** (Ratio 13:87): ^{1}H NMR (600 MHz, $CDCl_3$) δ 7.36 – 7.33 (0.3H, m), 7.33 – 7.28 (2H, m), 7.12 (0.3H, t, *J* = 8.5 Hz), 7.06 (2H, t, *J* = 8.6 Hz), 3.75 (0.15H, t, *J* = 6.5 Hz), 3.32 (1H, t, *J* = 6.9 Hz), 3.10 (1H, td, *J* = 11.5, 4.9 Hz), 2.78 (1H, dd, *J* = 6.2, 2.0 Hz), 2.66 (0.15H, dd, *J* = 7.0, 2.0 Hz), 2.45 – 2.32 (2.3H, m), 2.14 – 2.03 (1H, m), 1.98 – 1.91 (0.15H, m), 1.90 – 1.81 (1H, m), 1.79 – 1.70 (0.15H, m), 1.70 – 1.60 (0.15H, m), 0.98 (3H, t, *J* = 7.5 Hz), 0.70 (0.45H, t, *J* = 7.5 Hz); ^{13}C NMR (151 MHz, $CDCl_3$) δ 163.2 (d, *J* = 248.4 Hz) (2C), 132.3 (d, *J* = 8.3 Hz) (**15a**), 132.0 (d, *J* = 8.3 Hz) (**15a'**), 126.9 (d, *J* = 3.5 Hz) (**15a'**), 126.0 (d, *J* = 3.2 Hz) (**15a**), 116.2 (d, *J* = 21.6 Hz) (**15a**), 115.4 (d, *J* = 21.5 Hz) (**15a'**), 67.1 (**15a'**), 57.0 (**15a**), 50.5 (**15a**), 48.8 (**15a'**), 41.6 (**15a'**), 39.5 (**15**), 20.4 (**15a'**), 19.8 (**15a**), 11.4 (2C); ^{19}F NMR (564 MHz, $CDCl_3$) δ –110.91 (ddd, *J* = 13.6, 8.5, 5.1 Hz) (**15a**), -112.34 (ddd, *J* = 13.7, 8.7, 5.1 Hz) (**15a'**); ^{11}B NMR (192 MHz, $CDCl_3$) δ –11.79 (**15a**), -16.27 (**15a'**); HRMS (ESI) found MNa^+ 216.1335, $C_{11}H_{17}NBFNa$ requires 216.1330.

The relative configuration was determined by ^{1}H NMR and ^{11}B NMR shift in analogy with the shift differences observed for **3a** and **3a'**.

trans-2-(2-Chlorophenyl)-1-propylaziridine Borane (16a)

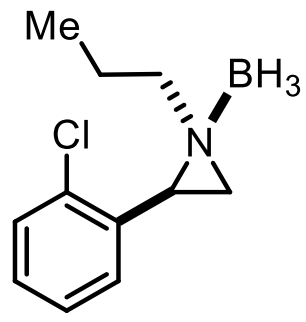

Following **GP4**, but adding BH_3•DMS at -78 ºC and react at r.t. for 4 h, **16** (2.11 g, 10.79 mmol, 1.0 equiv.) gave after precipitation and filtration **16a** (1.65 g, 73%) as a solid. ^{1}H NMR (400 MHz, $CDCl_3$) δ 7.45 (1H, d, J = 7.9 Hz), 7.39 – 7.14 (3H, m), 3.47 (1H, t, J = 7.2 Hz), 3.07 (1H, dt, J = 11.6, 7.4 Hz), 2.78 (1H, d, J = 6.2 Hz), 2.67 (1H, dt, J = 12.0, 8.2 Hz)), 2.48 (1H, d, J = 7.7 Hz), 2.19 – 2.01 (1H, m), 2.01 – 1.83 (1H, m), 0.96 (3H, t, J = 7.5 Hz); ^{13}C NMR (101 MHz, $CDCl_3$) δ 136.8, 131.2, 130.4, 129.7, 129.5, 126.4, 66.6, 48.8, 39.7, 19.8, 11.3; ^{11}B NMR ($CDCl_3$, 128 MHz) δ –16.55; HRMS (ESI) found MNa^+ 232.1031, $C_{11}H_{17}NBClNa$ requires 232.1035.

The relative configuration was determined by ^{1}H NMR and ^{11}B NMR shift in analogy with the shift differences observed for **3a** and **3a'**.

trans-1-(_tert_-Butyl)-2-phenylaziridine Borane (17a)

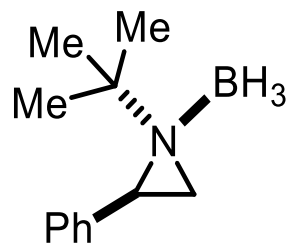

Following **GP4**, but adding BH_3•DMS at -78 ºC, **17** (876 mg, 5.0 mmol, 1.0 equiv.) gave after precipitation **17a** (520 mg, 55%) as a white solid. ^{1}H (600 MHz, $CDCl_3$) δ 7.40 – 7.27 (5H, m), 3.69 (1H, t, J = 7.2 Hz), 2.67 – 2.60 (2H, m), 1.56 – 0.91 (12H, m); ^{13}C (151 MHz, $CDCl_3$) δ 132.2, 130.2, 128.5, 128.1, 60.9, 44.6, 36.1, 26.4; ^{11}B (193 MHz, $CDCl_3$) δ –16.61 (q, J = 99.2 Hz). Data in accordance with the literature.[15]

The relative configuration was determined by a 2D ^{1}H-^{1}H NOESY (600 MHz, $CDCl_3$) experiment:

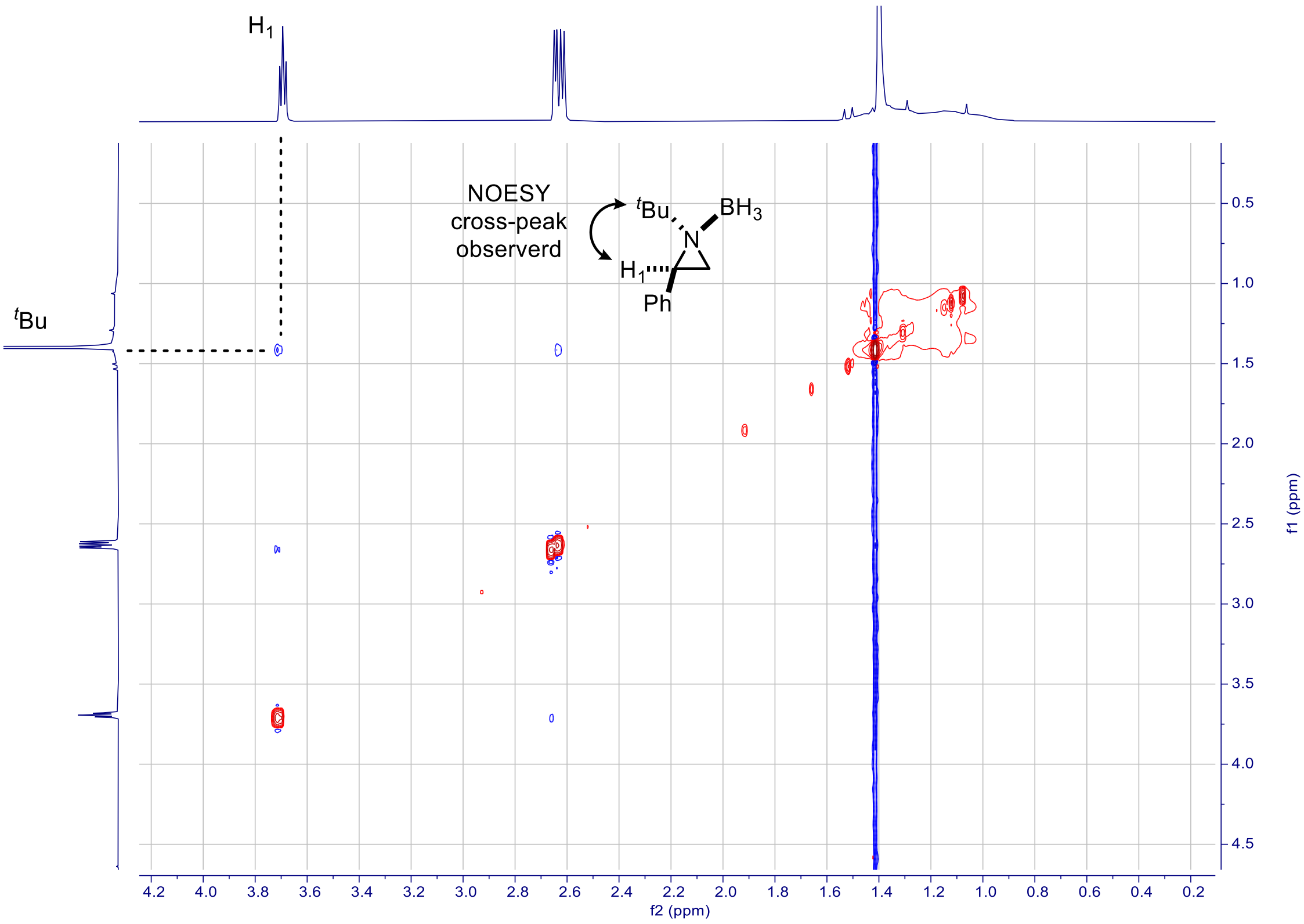

***cis*-2-(4-Fluorophenyl)-1-(4-methoxyphenethyl)aziridine Borane (18a) and *trans*-2-(4-Fluorophenyl)-1-(4-methoxyphenethyl)aziridine Borane (18a')**

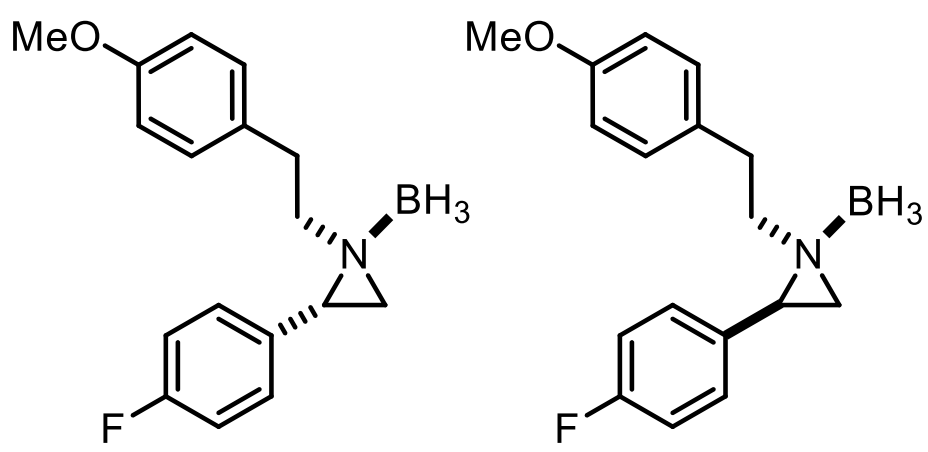

Following **GP4**, but adding BH_3•DMS at -78 °C and react at r.t. for 4 h, **18** (1.84 g, 6.5 mmol, 1.0 equiv.) gave after precipitation and filtration **18a** (12%) and **18a**' (68%) as mixture of diastereomers as a solid (1.48 g, 80% total yield; the mixture was used directly in the next step). Mixture of 2 diastereomers, **18a** and **18a'** (Ratio 15:85): ^{1}H NMR (600 MHz, $CDCl_3$) δ 7.34 (0.3H, dd, J = 8.5, 5.2 Hz), 7.19 (2H, d, J = 8.6 Hz), 7.17 – 7.11 (2.3H, m), 7.03 (2H, t, J = 8.6 Hz), 6.88 (2H, d, J = 8.6 Hz), 6.83 (0.3H, d, J = 8.6 Hz), 6.75 (0.3H, d, J = 8.6 Hz), 3.81 (3H, s), 3.75 (0.45H, s), 3.34 (1H, ddd, J = 11.9, 9.6, 5.3 Hz), 3.30 – 3.23 (2H, m), 3.12 (1H, ddd, J = 13.8, 9.6, 6.5 Hz), 3.02 (0.15H, ddd, J = 13.6, 10.2, 6.9 Hz), 2.76 (1H, dd, J = 6.2, 2.1 Hz), 2.71 (1H, ddd, J = 11.8, 9.5, 6.5 Hz), 2.62 (0.15H, dd, J = 7.0, 2.2 Hz), 2.54 (0.15H, ddd, J = 12.2, 10.2, 4.4 Hz), 2.37 (1H, dd, J = 7.7, 2.1 Hz), 2.35 – 2.29 (0.15H, m); ^{13}C NMR (151 MHz, $CDCl_3$) δ 163.2 (d, J = 248.9 Hz) (**18a'**), 158.7 (**18a'**), 132.3 (d, J = 8.8 Hz) (**18a**), 132.1 (d, J = 8.5 Hz) (**18a'**), 130.1 (**18a'**), 130.0 (**18a**), 129.8 (**18a'**), 126.6 (d, J = 2.5 Hz) (**18a'**), 116.3 (d, J = 21.8 Hz) (**18a**), 115.3

(d, J = 21.5 Hz) (**18a'**), 114.4 (**18a'**), 114.1 (**18a**), 66.8 (**18a'**), 64.0 (**18a**), 56.9 (**18a'**), 55.5 (**18a**), 50.2 (**18a'**), 49.4 (**18a**), 41.4 (**18a**), 39.8 (**18a'**), 32.4 (**18a**), 31.8 (**18a'**); ^{11}B NMR (192 MHz, $CDCl_3$) δ –11.72 (**18a**), -16.26 (**18a'**); ^{19}F NMR (564 MHz, $CDCl_3$) δ –110.62 (tt, J = 8.4, 5.1 Hz) (**18a**), -112.21 (tt, J = 8.4, 5.1 Hz) (**18a'**); HRMS (ESI) found MNa^+ 308.1599, $C_{17}H_{21}NOBFNa$ requires 308.1592.

The relative configuration was determined by ^{1}H NMR and ^{11}B NMR shift in analogy with the shift differences observed for **3a** and **3a'**.

<u>Note</u>: *some peaks belonging to the minor diastereomer* **18a** *are missing in ^{1}H and ^{13}C because either they are overlapping with the peaks of the major diastereomer* **18a'**, *or their intensity is low.*

trans **1-(2,3-Dihydro-1*H*-inden-1-yl)-2-(*p*-tolyl)aziridine (19a)**

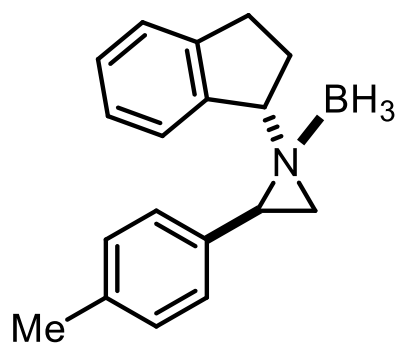

Following **GP4**, but adding BH_3•DMS at -78 °C and react at r.t. for 4 h, **19** (770 mg, 3.0 mmol, 1.0 equiv.) gave after precipitation and filtration **19a** as mixture of diastereomers (680 mg, 86% total yield, the mixture was used directly in the next step) as a solid. d.r. 1:1. ^{1}H NMR (400 MHz, $CDCl_3$), s*ignals corresponding to both diastereomers*: δ 7.52 – 7.40 (1H, m), 7.40 – 7.23 (4H, m), 7.23 – 7.11 (3H, m), 4.49 (0.5H, d, J = 8.3 Hz), 4.17 (0.5H, dd, J = 8.4, 3.9 Hz), 3.50 (0.5H, t, J = 7.1 Hz), 3.34 (0.5H, dt, J = 17.8, 8.8 Hz), 3.25 (0.5H, dd, J = 15.9, 8.0 Hz), 3.14 (0.5H, t, J = 7.2 Hz), 3.00 – 2.83 (2H, m), 2.83 – 2.70 (1H, m), 2.65 (0.5H, d, J = 7.8 Hz), 2.57 (0.5H, d, J = 7.8 Hz), 2.47 (1H, dq, J = 16.6, 7.8 Hz), 2.35 (1.5H, s), 2.35 (1.5H, s); ^{13}C NMR (151 MHz, $CDCl_3$) δ 147.0, 145.8, 139.0, 138.8, 137.6, 131.0, 130.3, 130.1, 129.9, 129.8, 129.3, 129.1, 128.1, 128.0, 126.8, 126.6, 126.6, 125.8, 125.6, 125.4, 76.6, 74.4, 48.0, 45.1, 39.2, 37.6, 31.2, 31.1, 30.1, 29.6, 21.5, 21.4; ^{11}B NMR (192 MHz, $CDCl_3$) δ –15.94, -17.69; HRMS (ESI) found MNa^+ 286.1738, $C_{18}H_{22}NBNa$ requires 286.1738.

The relative configuration was determined by ^{1}H NMR and ^{11}B NMR shift in analogy with the shift differences observed for **12a** and **12a'**.

cis-1-(Furan-2-ylmethyl)-2-(_p_-tolyl)aziridine Borane (20a) and _trans_-1-(Furan-2-ylmethyl)-2-(_p_-tolyl)aziridine Borane (20a')

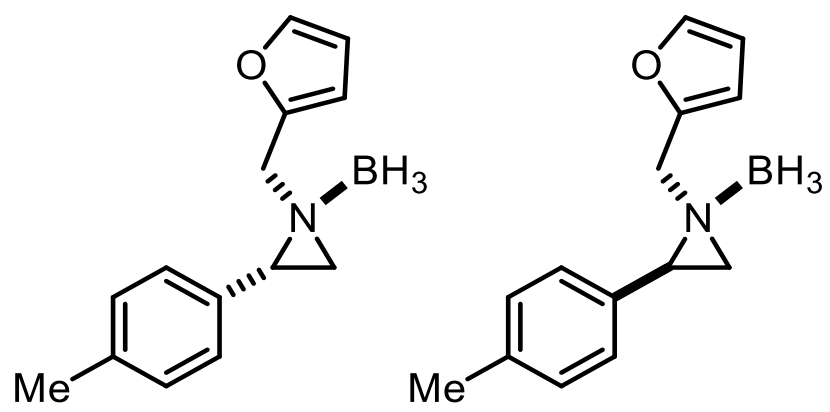

Following **GP4**, but adding BH_3•DMS at -78 °C and react at r.t. for 4 h, **20** (1.3 g, 5.6 mmol, 1.0 equiv.) gave after precipitation and filtration **20a** (44%) and **20a'** (53%) as mixture of diastereomers as an oil (1.23 g, 97% total yield; the mixture was used directly in the next step). Mixture of 2 diastereomers, **20a** and **20a'** (Ratio 55:45): ^{1}H NMR (600 MHz, $CDCl_3$) δ 7.61 (1H, dd, *J* = 1.9, 0.8 Hz), 7.48 (1H, dd, *J* = 1.9, 0.9 Hz), 7.41 (2H, d, *J* = 8.1 Hz), 7.37 (2H, d, *J* = 7.0 Hz), 7.26 (4H, s), 6.63 (1H, d, *J* = 3.3 Hz), 6.54 (1H, dd, *J* = 3.3, 1.9 Hz), 6.44 (1H, dd, *J* = 3.2, 1.8 Hz), 6.41 (1H, d, *J* = 3.3 Hz), 4.45 (1H, d, *J* = 14.3 Hz), 4.10 (1H, d, *J* = 14.3 Hz), 3.99 (1H, t, *J* = 6.7 Hz), 3.81 (1H, d, *J* = 14.4 Hz), 3.66 (1H, dd, *J* = 7.8, 6.4 Hz), 3.17 (1H, d, *J* = 14.4 Hz), 2.97 (1H, dd, *J* = 6.4, 2.4 Hz), 2.86 (1H, dd, *J* = 6.4, 2.0 Hz), 2.73 (1H, dd, *J* = 7.0, 2.4 Hz), 2.67 (1H, dd, *J* = 7.8, 2.0 Hz), 2.50 (3H, s), 2.45 (3H, s); ^{13}C NMR (151 MHz, $CDCl_3$) δ 148.2, 147.4, 143.8, 142.9, 140.2, 138.9, 130.4, 130.0, 130.0, 129.7, 129.0, 128.7, 127.9, 126.7, 113.0, 111.6, 111.1, 110.7, 57.7, 51.8, 50.1, 46.9, 38.5, 38.4, 21.4, 21.4; ^{11}B NMR (192 MHz, $CDCl_3$) δ –11.12 (**20**), -15.20 (**20a'**); HRMS (ESI) found MNa^+ 250.1378, $C_{14}H_{18}NOBNa$ requires 250.1374.
The relative configuration was determined by ^{1}H NMR and ^{11}B NMR shift in analogy with the shift differences observed for **10a** and **10a'**.

trans-1-(2,2-Dimethoxyethyl)-2-(naphthalen-2-yl)aziridine Borane (21a)

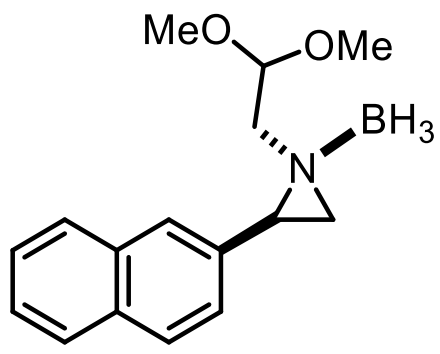

Following **GP4**, but adding BH_3•DMS at -78 °C and react at r.t. for 4 h, **21** (1.01 g, 3.93 mmol, 1.0 equiv.) gave after precipitation and filtration **21a** (900 mg, 84%) as a solid. ^{1}H NMR (400 MHz, $CDCl_3$) δ 7.93 – 7.81 (3H, m), 7.79 (1H, s), 7.59 – 7.44 (3H, m), 5.14 (1H, dd, *J* = 7.3, 3.6 Hz), 3.81 (1H, t, *J* = 6.9 Hz), 3.65 (3H, s), 3.52 (3H, s), 3.34 (1H, dd, *J* = 12.4, 3.5 Hz), 2.94 (1H, dd, *J* = 6.6, 2.2 Hz), 2.58 (1H, dd, *J* = 7.7, 2.1 Hz), 2.51 (1H, dd, *J* = 12.4, 7.3 Hz); ^{13}C NMR (101 MHz, $CDCl_3$) δ 133.6, 132.9, 130.0, 128.7, 128.3, 127.9, 127.8, 127.5, 126.8, 126.4, 102.8, 65.7, 56.2, 55.9, 50.5, 41.0; ^{11}B NMR (192 MHz, $CDCl_3$) δ –15.71; HRMS (ESI) found $[M-BH_3]H^+$ 258.1488, $C_{16}H_{20}NO_2$ requires 258.1489.

The relative configuration was determined by ^{1}H NMR and ^{11}B NMR shift in analogy with the shift differences observed for **3a** and **3a'**.

trans-1-Cyclopentyl-2-(_o_-tolyl)aziridine Borane (22a)

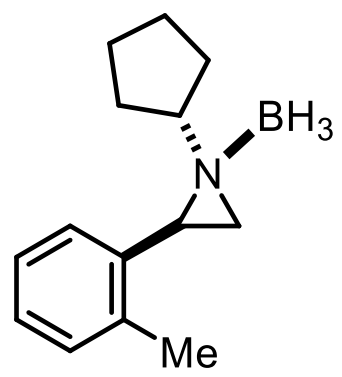

Following **GP4**, but adding BH_3•DMS at -78 °C and react at r.t. for 4 h, **22** (840 mg, 4.17 mmol, 1.0 equiv.) gave after precipitation and filtration **22a** (690 mg, 77%) as a solid. ^{1}H NMR (400 MHz, $CDCl_3$) δ 7.34 – 7.21 (2H, m), 7.19 (1H, t, *J* = 7.4 Hz), 7.11 (1H, d, *J* = 7.6 Hz), 3.36 (1H, t, *J* = 6.9 Hz), 2.98 (1H, p, *J* = 8.1 Hz), 2.82 (1H, dd, *J* = 6.3, 1.8 Hz), 2.53 – 2.37 (4H, m), 2.28 – 1.99 (3H, m), 1.93 – 1.77 (3H, m), 1.65 – 0.72 (5H, m); ^{13}C NMR (101 MHz, $CDCl_3$) δ 139.5, 130.4, 130.2, 129.8, 129.2, 125.7, 74.2, 50.0, 38.8, 29.1, 28.7, 24.9, 24.2, 20.4; ^{11}B NMR (128 MHz, $CDCl_3$) δ –18.56; HRMS (ESI) found MNa^+ 238.1736, $C_{14}H_{22}NBNa$ requires 238.1738.
The relative configuration was determined by ^{1}H NMR and ^{11}B NMR shift in analogy with the shift differences observed for **12a** and **12a'**.

tert-Butyl _trans_-4-(2-(4-Chlorophenyl)aziridin-1-yl)piperidine-1-carboxylate Borane (23a)

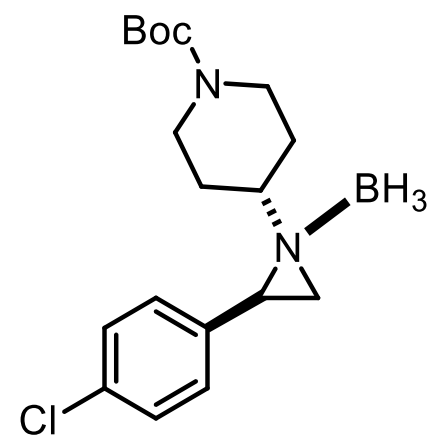

Following **GP4**, but adding BH_3•DMS at -78 °C and react at r.t. for 4 h, **23** (1.44 g, 4.27 mmol, 1.0 equiv.) gave after precipitation and filtration **23a** (1.20 g, 80%) as a solid. ^{1}H NMR (600 MHz, $CDCl_3$) δ 7.35 (2H, d, *J* = 8.4 Hz), 7.27 – 7.24 (2H, m), 4.53 – 4.04 (2H, br m), 3.31 (1H, t, *J* = 7.0 Hz), 2.80 (1H, dd, *J* = 6.2, 2.1 Hz), 2.65 (2H, br s), 2.48 (1H, dd, *J* = 7.8, 2.0 Hz), 2.30 – 2.15 (2H, m), 2.05 (1H, tt, *J* = 11.8, 3.7 Hz), 1.91 – 1.83 (2H, br m), 1.46 (9H, s); ^{13}C NMR (151 MHz, $CDCl_3$) δ 154.5, 135.3, 131.6, 129.2, 128.6, 80.2, 71.6, 65.1, 49.9, 40.4, 28.6; ^{11}B NMR (192 MHz, $CDCl_3$) δ –18.51; HRMS (ESI) found MNa^+ 373.1822, $C_{18}H_{28}N_2O_2BClNa$ requires 373.1825.
The relative configuration was determined by ^{1}H NMR and ^{11}B NMR shift in analogy with the shift differences observed for **12a** and **12a'**.

***tert*-Butyl *cis*-2-(2-Phenylaziridin-1-yl)acetate Borane (24a) and *tert*-Butyl *trans*-2-(2-Phenylaziridin-1-yl)acetate Borane (24a')**

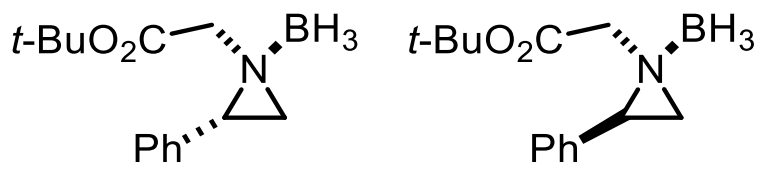

Following **GP4**, **24** (607 mg, 2.6 mmol, 1.0 equiv.) gave after purification via column chromatography **24a** (122 mg, 19%) and **24a'** (341 mg, 53%) as separable mixture of diastereomers. **24a**: **24a'** = 26:74.

Data for **24a** (solid): ^{1}H (600 MHz, $CDCl_3$) δ 7.46 – 7.41 (3H, m), 7.39 – 7.34 (2H, m), 3.80 (1H, t, J = 6.7 Hz), 3.21 (1H, d, J = 15.2 Hz), 3.01 (1H, dd, J = 6.7, 3.0 Hz), 2.93 (1H, dd, J = 7.0, 2.9 Hz), 2.36 (1H, d, J = 15.0 Hz), 1.46 (9H, s); ^{13}C (151 MHz, $CDCl_3$) δ 166.5, 130.3, 130.2, 129.8, 129.3, 82.9, 55.7, 50.1, 40.3, 28.1; ^{11}B (193 MHz, $CDCl_3$) δ –10.74 (q, J = 103.1 Hz); HRMS (ESI) found MNa^+ 270.1636, $C_{14}H_{22}BNO_2Na$ requires 270.1636.

The relative configuration was determined by a 2D ^{1}H-^{1}H NOESY (600 MHz, $CDCl_3$) experiment:

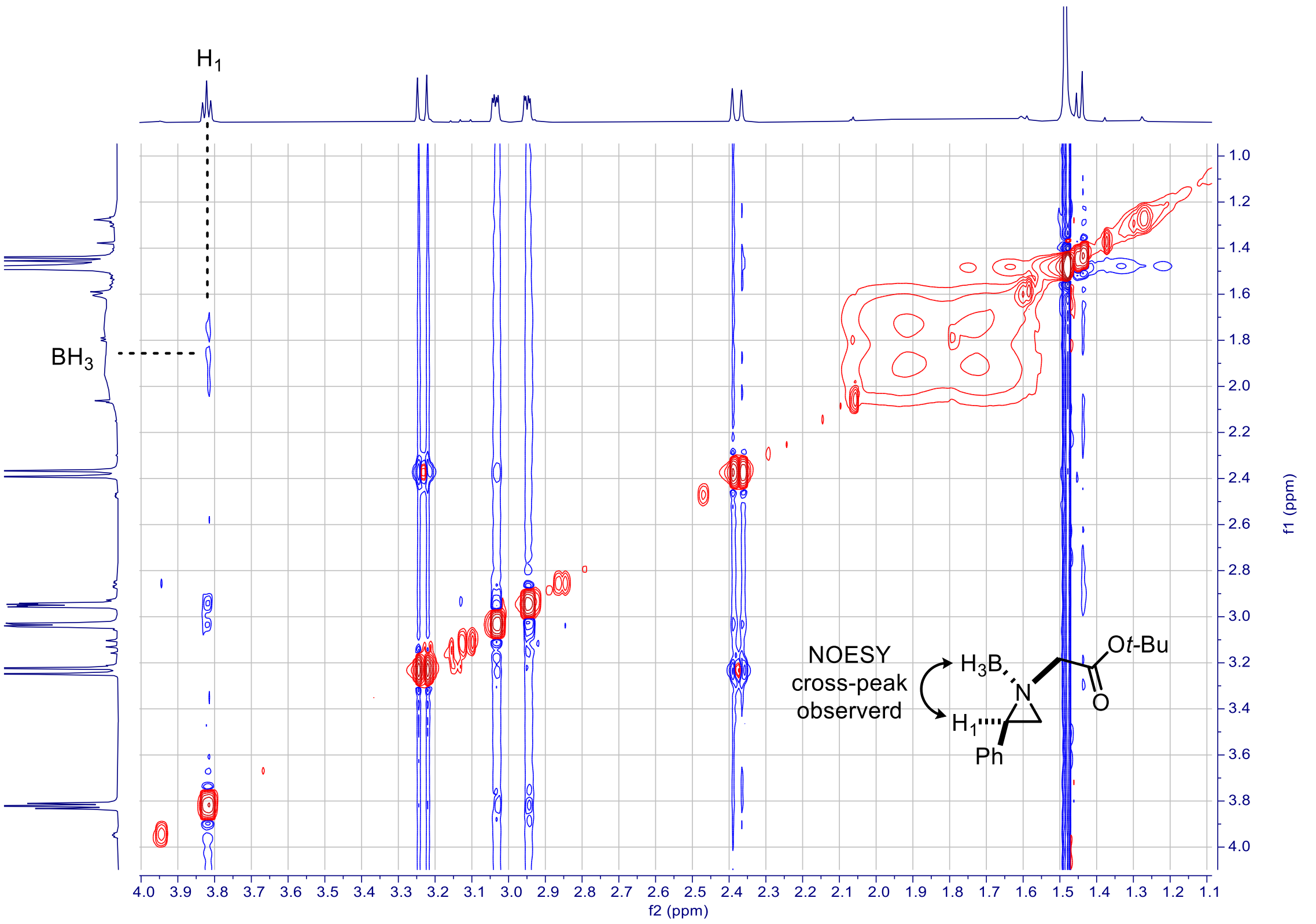

Data for **24a'** (solid): ^{1}H (600 MHz, $CDCl_3$) δ 7.53 – 7.48 (2H, m), 7.41 –7.36 (3H, m), 3.79 – 3.71 (2H, m), 3.16 (1H, d, J = 14.6 Hz), 2.84 (1H, dd, J = 6.7, 2.4 Hz), 2.64 (1H, dd, J = 7.8, 2.5 Hz), 1.54 (12H, m); ^{13}C (151 MHz, $CDCl_3$) δ 165.9, 130.9, 130.5, 129.2, 128.2, 83.0, 64.5, 50.0, 40.7, 28.2; ^{11}B (193 MHz, $CDCl_3$) δ –15.28 (q, J = 87.0 Hz); HRMS (ESI) found MNa^+ 270.1634, $C_{14}H_{22}BNO_2Na$ requires 270.1636.

The relative configuration was determined by a 2D ^{1}H-^{1}H NOESY (600 MHz, $CDCl_3$) experiment:

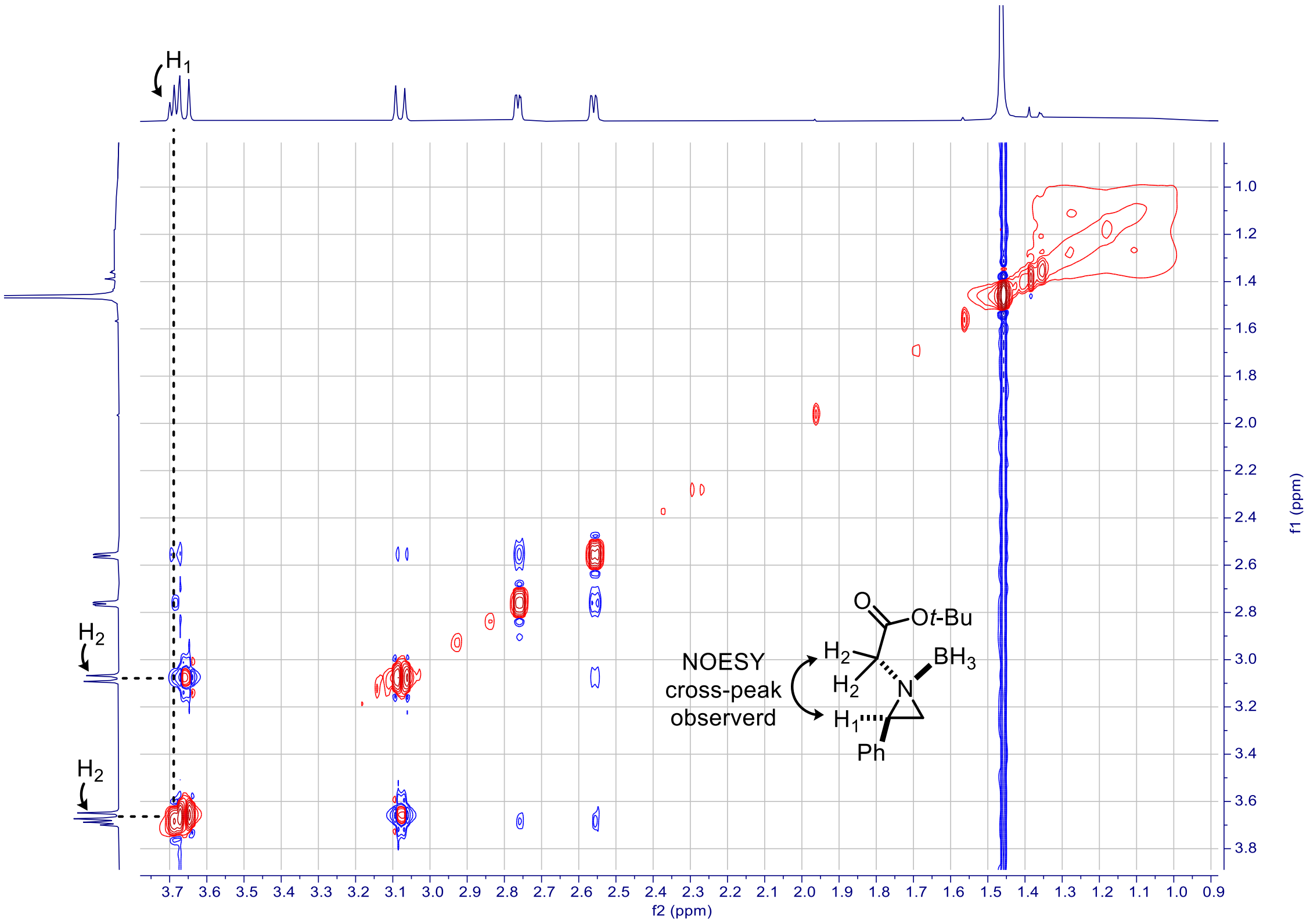

tert-Butyl *trans*-2-((4*R*,6*R*)-6-(2-(2-(4-Chlorophenyl)aziridin-1-yl)ethyl)-2,2-dimethyl-1,3-dioxan-4-yl)acetate (25a)

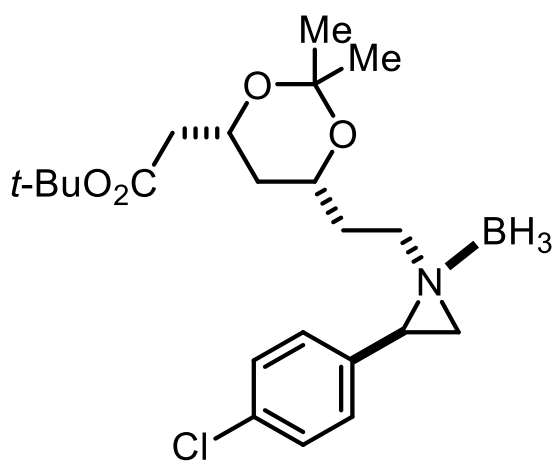

Following **GP4**, but adding BH_3•DMS at -78 °C and react at r.t. for 4 h, **25** (1.16 g, 2.82 mmol, 1.0 equiv.) gave after precipitation and filtration **25a** as mixture of diastereomers (950 mg, 80% total yield, the mixture was used directly in the next step) as a solid. d.r.1.3:1. ^{1}H NMR (600 MHz, $CDCl_3$), *signals corresponding to both diastereomers*: δ 7.37 – 7.33 (2.3H, m), 7.30 – 7.24 (2.3H, m), 4.30 – 4.22 (1.15H, m), 4.02 – 3.96 (0.65H, m), 3.96 – 3.89 (0.5H, m), 3.40 – 3.33 (1.15H, m), 3.17 (0.5H, td, *J* = 11.0, 6.1 Hz), 3.09 (0.65H, td, *J* = 11.2, 4.8 Hz), 2.79 (0.5H, dt, *J* = 6.2, 1.5 Hz), 2.77 – 2.69 (1.3H, m), 2.62 (0.5H, td, *J* = 11.2, 4.2 Hz), 2.50 (0.5H, dd, *J* = 7.6, 1.9 Hz), 2.48 – 2.41 (1.8H, m), 2.40 – 2.34 (0.5H, m), 2.34 – 2.31 (0.65H, m), 2.31 – 2.28 (0.5H, m), 2.22 – 2.16 (0.65H, m), 2.04 – 1.96 (0.65H, m), 1.83 – 1.74 (0.5H, m), 1.65 – 1.56 (1.15H, m), 1.45 (10.35H, s), 1.44 (1.5H, s), 1.42 (1.95H, s), 1.35 (1.5H, s), 1.34 (1.95H, s), 1.31 – 1.20 (1.15H, m); ^{13}C NMR (151 MHz, $CDCl_3$) δ 170.3, 170.2, 135.2, 131.5, 131.5, 129.7, 129.5, 128.6, 128.6, 99.0, 98.9, 80.9, 80.9, 66.6, 66.5, 66.2, 66.2, 61.3, 60.9, 49.7, 48.5, 42.8, 42.7, 42.2, 40.9, 36.7,

36.2, 33.9, 33.4, 30.2, 30.2, 28.2, 20.0, 19.9; ^{11}B NMR (192 MHz, $CDCl_3$) δ –16.31; HRMS (ESI) found MNa^+ 446.2234, $C_{22}H_{35}NO_4BClNa$ requires 446.2240.

The relative configuration was determined by ^{1}H NMR and ^{11}B NMR shift in analogy with the shift differences observed for **3a** and **3a'**.

***trans*-2-Phenethyl-1-propylaziridine Borane (26a)**

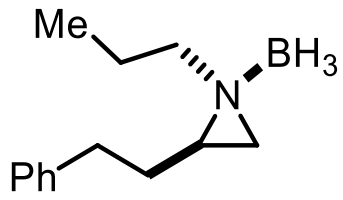

Following **GP4**, **26** (147 mg, 0.78 mmol, 1.0 equiv.) gave after purification via column chromatography **26a** (124 mg, 78%) as a solid. R_f 0.37 [*n*-pentane:EtOAc (10:1)]. ^{1}H (600 MHz, $CDCl_3$) δ 7.33 – 7.27 (2H, m), 7.25 – 7.18 (3H, m), 2.99 – 2.87 (2H, m), 2.76 (1H, ddd, *J* = 14.0, 9.1, 6.7 Hz), 2.38 – 2.28 (1H, m), 2.15 – 2.05 (4H, m), 2.04 – 2.00 (1H, m), 1.99 – 1.90 (1H, m), 1.86 – 1.33 (4H, m), 0.88 (3H, t, *J* = 7.4 Hz); ^{13}C (151 MHz, $CDCl_3$) δ 140.8, 128.6, 128.5, 126.3, 67.0, 46.7, 42.7, 33.8, 29.2, 19.9, 11.3; ^{11}B (193 MHz, $CDCl_3$) δ –16.71 (q, *J* = 98.0 Hz); HRMS (ESI) found MNa^+ 226.1734, $C_{13}H_{22}BNNa$ requires 226.1738.

The relative configuration was determined by ^{1}H NMR and ^{11}B NMR shift in analogy with the shift differences observed for **3a** and **3a'**.

***trans*-1-Hexyl-2-phenethylaziridine Borane (27a)**

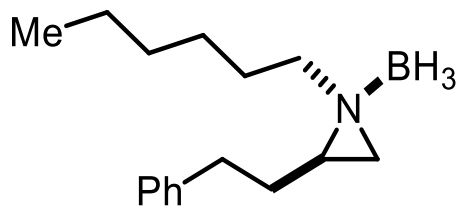

Following **GP4**, **27** (1.11 g, 4.8 mmol, 1.0 equiv.) gave after purification via column chromatography **27a** (671 mg, 57%) as an oil. R_f 0.78 [*n*-pentane:EtOAc (6:1)]. ^{1}H (600 MHz, $CDCl_3$) δ 7.35 – 7.21 (5H, m), 3.01 – 2.92 (2H, m), 2.82 – 2.74 (1H, m), 2.40 – 2.31 (1H, m), 2.19 – 2.01 (5H, m), 1.98 – 1.88 (1H, m), 1.83 – 1.42 (4H, m), 1.36 – 1.21 (6H, m), 0.91 (3H, t, *J* = 6.8 Hz); ^{13}C (151 MHz, $CDCl_3$) δ 140.9, 128.7, 128.6, 126.3, 65.5, 46.6, 42.8, 33.8, 31.6, 29.2, 26.7, 26.6, 22.6, 14.1; ^{11}B (193 MHz, $CDCl_3$) δ –16.71 (d, *J*=103.6 Hz); HRMS (ESI) found MNa^+ 268.2204, $C_{16}H_{28}BNNa$ requires 268.2207.

The relative configuration was determined by ^{1}H NMR and ^{11}B NMR shift in analogy with the shift differences observed for **3a** and **3a'**.

trans-1-Benzyl-2-methylaziridine Borane (28a)

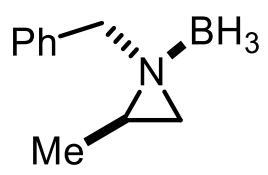

Following **GP4**, **28** (250 mg, 1.7 mmol, 1.0 equiv.) gave after purification via column chromatography **28a** (134 mg, 49%) as a solid. R_f 0.34 [*n*-pentane:EtOAc (3:1)]. ^{1}H (600 MHz, $CDCl_3$) δ 7.42 – 7.35 (3H, m), 7.34 – 7.29 (2H, m), 4.16 (1H, d, *J* = 13.3 Hz), 3.80 (1H, d, *J* = 13.3 Hz), 2.37 – 2.28 (1H, m), 2.17 (1H, dd, *J* = 7.7, 1.6 Hz), 2.09 (1H, dd, *J* = 6.1, 1.6 Hz), 1.51 (6H, m); ^{13}C (151 MHz, $CDCl_3$) δ 132.7, 130.9, 128.9, 128.7, 65.8, 40.7, 40.2, 12.6; ^{11}B (193 MHz, $CDCl_3$) δ –15.40 (q, *J* = 97.8 Hz); HRMS (ESI) found MNa^+ 184.1270, $C_{10}H_{16}BNNa$ requires 184.1268.

The relative configuration was determined by a 2D ^{1}H-^{1}H NOESY (600 MHz, $CDCl_3$) experiment:

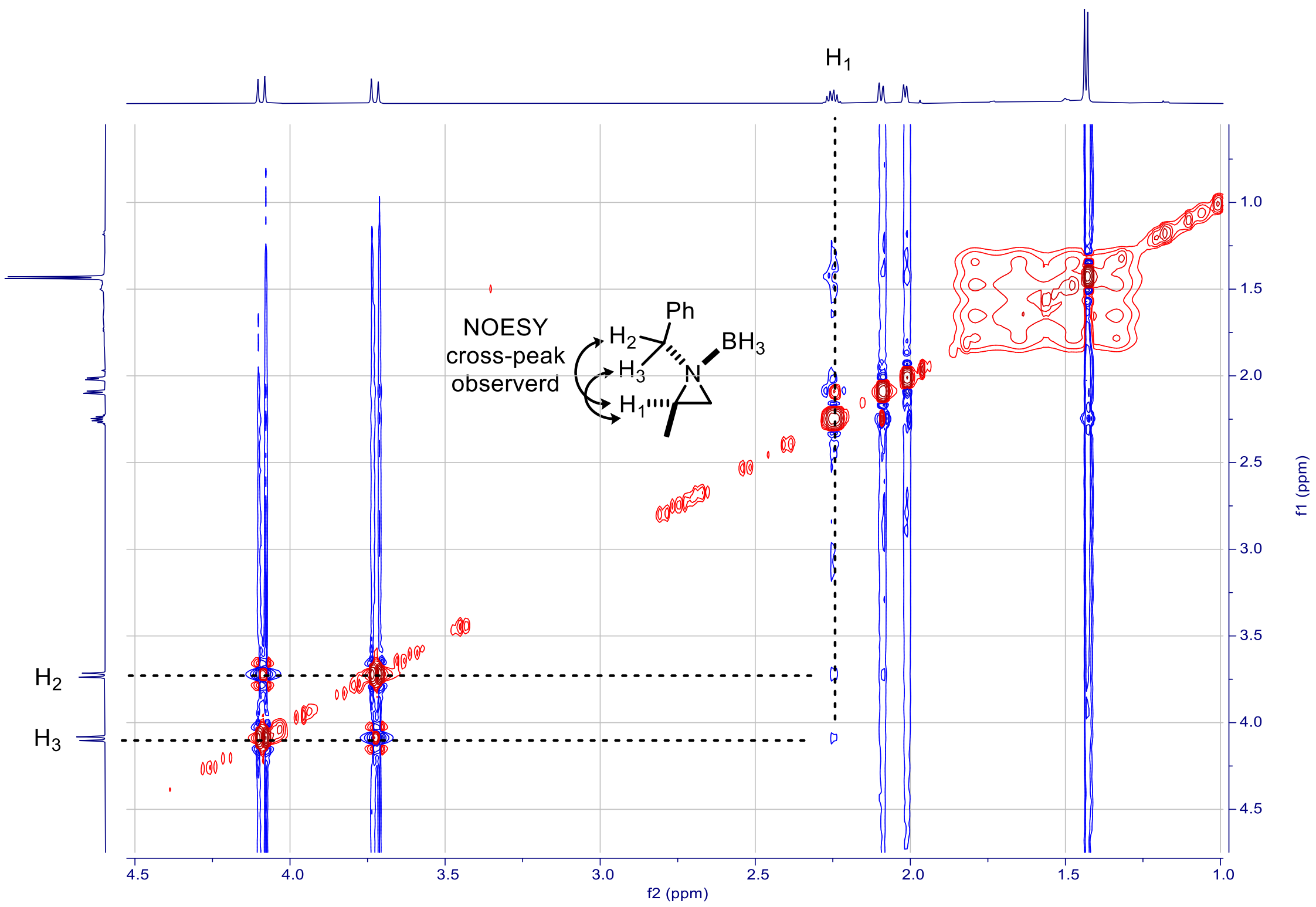

trans-1-Benzyl-2-butylaziridine Borane (29a)

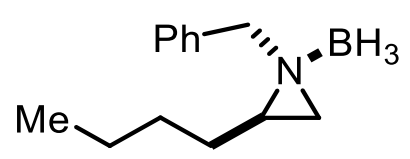

Following **GP4**, **29** (833 mg, 4.4 mmol, 1.0 equiv.) gave after purification via column chromatography **29a** (811 mg, 90%) as a solid. R_f 0.43 [*n*-pentane:EtOAc (10:1)]. ^{1}H NMR (600 MHz, $CDCl_3$) δ 7.40 – 7.35 (3H, m), 7.35 – 7.30 (2H, m), 4.19 (1H, d, *J* = 13.2 Hz), 3.74 (1H, d, *J* = 13.2 Hz), 2.20 (1H, p, *J* = 6.6 Hz), 2.16 (1H, d, *J* = 7.8 Hz), 2.11 (1H, d, *J* = 6.4 Hz), 2.04 – 1.95 (1H, m), 1.93 – 1.38 (5H, m), 1.36 – 1.24 (3H, m), 0.88 (3H, t, *J* = 7.1 Hz); ^{13}C NMR (151

MHz, $CDCl_3$) δ 132.8, 131.2, 129.0, 128.6, 66.2, 44.9, 40.5, 29.7, 26.8, 22.4, 13.9; ^{11}B (193 MHz, $CDCl_3$) δ –15.15 (q, J = 97.8 Hz); HRMS (ESI) found MNa^+ 226.1731, $C_{13}H_{22}BNNa$ requires 226.1738.

The relative configuration was determined by ^1H NMR and ^{11}B NMR shift in analogy with the shift differences observed for **10a** and **10a'**.

trans-2-Butyl-1-(1-(2,6-dimethylphenoxy)propan-2-yl)aziridine Borane (30a)

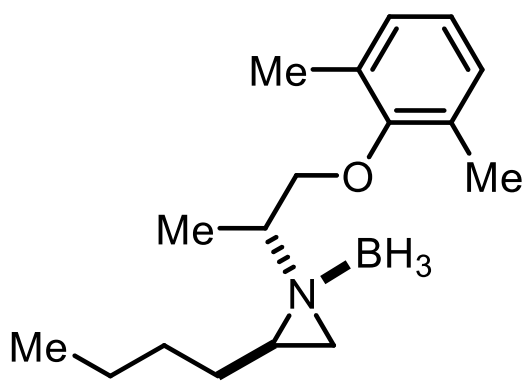

Following **GP4**, **30** (160 mg, 0.61 mmol, 1.0 equiv.) and BH_3•DMS (0.92 mmol, 1.5 equiv.) gave after purification via column chromatography **30a** as a single diastereomer (82 mg, 49%) as an oil. R_f 0.69 [*n*-pentane:EtOAc (1:1)]. ^1H NMR (600 MHz, $CDCl_3$) δ 7.02 (2H, d, J=7.4 Hz), 6.94 (1H, t, J=7.5 Hz), 4.37 (1H, dd, J=10.0, 7.2 Hz), 3.62 (1H, dd, J=10.0, 5.2 Hz), 2.45 (1H, p, J=6.8 Hz), 2.35 (1H, sx, J=6.7 Hz), 2.31 (6H, s), 2.22 – 2.14 (1H, m), 2.13 – 2.07 (2H, m), 1.87 – 1.78 (1H, m), 1.73 – 1.22 (10H, m), 0.93 – 0.87 (3H, d, J=7.2 Hz); ^{13}C NMR (151 MHz, $CDCl_3$) δ 155.6, 130.9, 129.1, 124.2, 72.9, 69.2, 50.8, 41.4, 29.7, 27.2, 22.5, 16.6, 14.9, 14.0; ^{11}B (193 MHz, $CDCl_3$) δ –20.01. HRMS could not be obtained for this compound.

The relative configuration was determined by a 2D ^1H-^1H NOESY (600 MHz, $CDCl_3$) experiment:

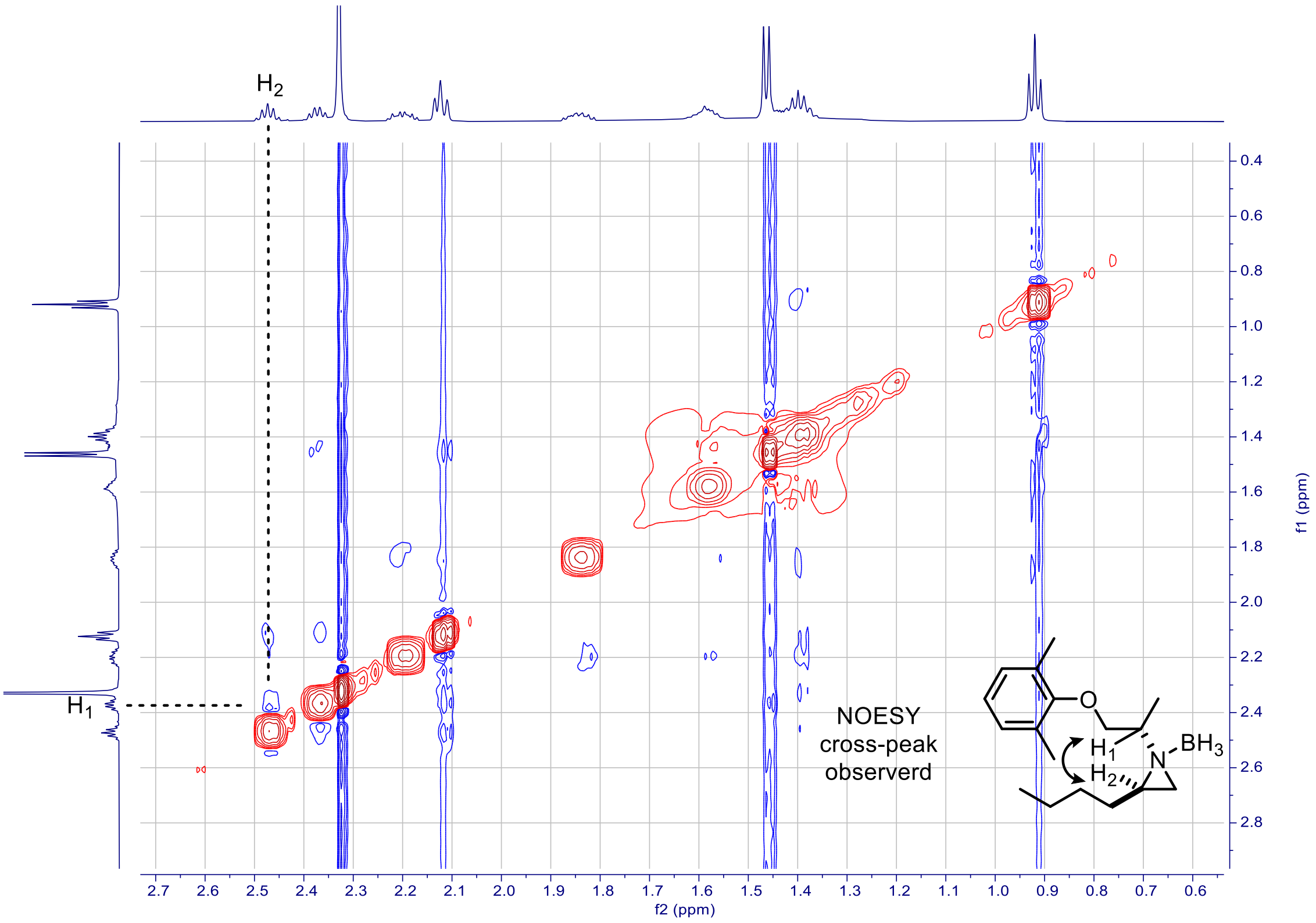

1-Benzyl-2,3-dipropylaziridine Borane (31a)

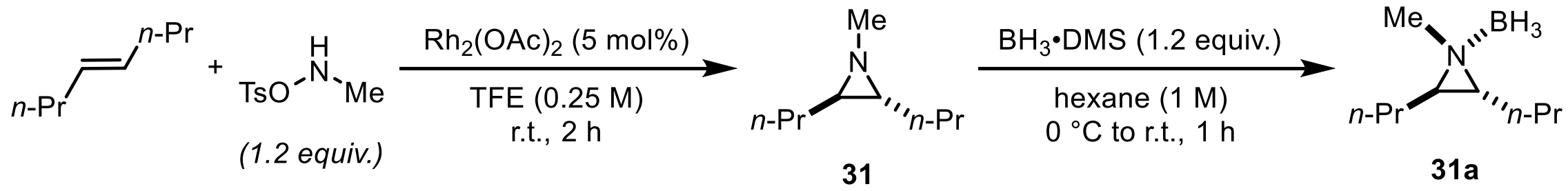

Step 1: To a round-bottom flask equipped with a magnetic stirring bar were added (*E*)-oct-4-ene (471 uL, 3.0 mmol, 1.0 equiv.), *N*-methyl-*O*-tosylhydroxylamine (723 mg, 3.6 mmol, 1.2 equiv.), and TFE (12 mL, 0.25 M). To this stirred solution was added $Rh_2(OAc)_2$ (66 mg, 5 mol %) in one portion. The reaction mixture was stirred at room temperature for 2 h. Upon completion, the reaction mixture was diluted with CH_2Cl_2 and washed with a saturated aqueous $NaHCO_3$ solution (x 2). The aqueous layer was extracted with CH_2Cl_2 (x 2), and the combined organic layers were dried over anhydrous Na_2SO_4. The solvent was evaporated and purified by column chromatography on silica gel to afford **31**. *Note: the product is volatile, so the crude was used without being completely dried, 47% ^{1}H NMR yield was obtained.* R_f 0.2 [*n*-pentane: EtOAc (1:1)].
Step 2: Following **GP4**, **31** (1.4 mmol, 1.0 equiv.) gave after purification via column chromatography **31a** (174 mg, 80%) as an oil. R_f 0.59 [*n*-pentane:EtOAc (5:1)]. ^{1}H NMR (600 MHz, $CDCl_3$) δ 2.58 (3H, s), 2.18 (1H, q, *J* = 6.6 Hz), 2.01 – 1.92 (1H, m), 1.86 – 1.28 (11H, m), 0.98 (3H, t, *J* = 7.3 Hz), 0.96 (3H, t, *J* = 7.3 Hz); ^{13}C NMR (151 MHz, $CDCl_3$) δ 53.8, 51.8, 44.5, 29.4, 29.0, 20.9, 20.8, 13.9, 13.9; ^{11}B (193 MHz, $CDCl_3$) δ –14.89 (q, *J* = 96.2 Hz); HRMS (ESI) found MNa^+ 178.1738, $C_9H_{22}BNNa$ requires 178.1738.

1-Methyl-2,3-diphenylaziridine Borane (32a)

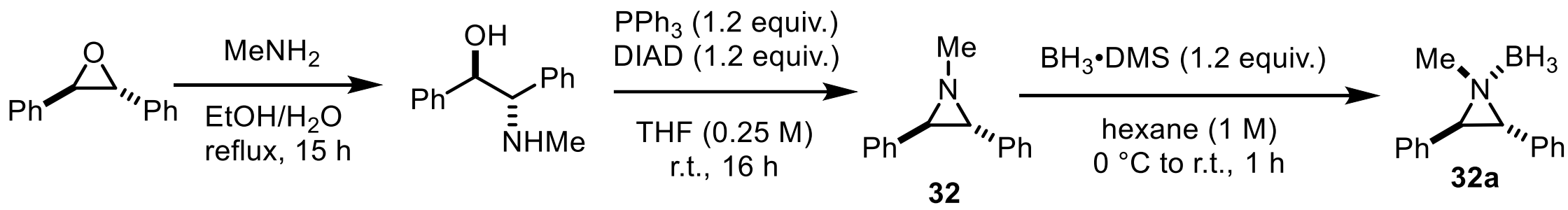

Step 1: Methylamine (33 wt% in EtOH, 20 mL) was added to a solution of *trans*-stilbene oxide (1.96 g, 10 mmol, 1.0 equiv.). Water (0.3 mL) was then added and the solution was stirred at reflux for 15 h. Upon completion, the excess methylamine and EtOH were removed under reduced pressure until crystallization began. The mixture was cooled at 0 °C for 12 h, filtered, and washed with pentane to give erythro-l,2-diphenyl-2-(*N*-methylamino)ethanol as a white solid (1.43 g, 63%). ^{1}H NMR (600 MHz, $CDCl_3$) δ 7.33 – 7.25 (6H, m), 7.19 – 7.14 (4H, m), 4.84 (1H, d, *J* = 5.9 Hz), 3.79 (1H, d, *J* = 5.9 Hz), 2.30 (3H, s). Data in accordance with the literature.[16]
Step 2: A round bottom flask equipped with a stirring bar was charged with erythro-l,2-diphenyl-2-(*N*-methylamino)ethanol (750 mg, 3.3 mmol, 1.0 equiv.) and PPh_3 (0.86 g, 3.9 mmol, 1.2 equiv.). Dry THF (13 mL, 0.25 M) was added followed by the dropwise addition of diisopropylazodicarboxylate (DIAD) (0.65 mL, 3.9 mmol, 1.2 equiv.) via syringe. The resulting

reaction mixture was stirred at room temperature for 16 h. Upon completion, the mixture was concentrated by rotary evaporation and the residue was purified by column chromatography to deliver the crude *trans*-1-methyl-2,3-diphenylaziridine **32** that was directly used in the next step. R_f 0.66 [*n*-pentane:EtOAc (1:1)]. *Note: It can't give pure aziridine by column isolation because of side product from DIAD.*

Step 3: Following **GP4** but in THF (1 M), crude *trans*-1-methyl-2,3-diphenylaziridine and BH_3•DMS (6.6 mmol, 2,0 equiv.) gave **32a** (545.1 mg, 74%, overall two steps) as a solid. ^{1}H NMR (400 MHz, $CDCl_3$) δ 7.50 – 7.40 (10H, m), 4.18 (1H, d, J = 6.5 Hz), 3.82 (1H, d, J = 6.5 Hz), 2.48 (3H, s), 1.48 (3H, q, J = 121.3 Hz); ^{13}C NMR (101 MHz, $CDCl_3$) δ 131.2, 130.2, 130.02, 129.98, 129.8, 129.19, 129.16, 128.3, 53.4, 53.2, 45.6; ^{11}B (128 MHz, $CDCl_3$) δ -9.57 – -17.62 (m); HRMS (ESI) found MNa^+ 246.1421, $C_{15}H_{18}BNNa$ requires 246.1425.

***trans*-7-Cyclohexyl-7-azabicyclo[4.1.0]heptane Borane (33a)**

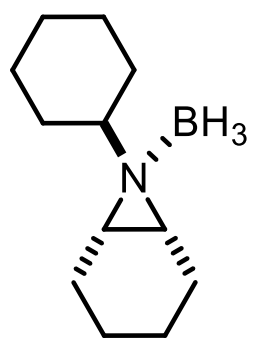

Following **GP4** but in (1 M), **33** (438 mg, 2.4 mmol, 1.0 equiv.) and BH_3•DMS (2.5 mmol, 1.05 equiv.) gave after purification via column chromatography **33a** (367 mg, 78%) as a solid. R_f 0.67 [*n*-pentane:EtOAc (5:1)]. ^{1}H NMR (400 MHz, $CDCl_3$) δ 2.21 – 2.10 (4H, m), 2.04 (2H, qd, J=12.4, 3.4 Hz), 1.94 – 0.70 (18H, m); ^{13}C NMR (151 MHz, $CDCl_3$) δ 76.8, 44.1, 29.0, 25.9, 25.7, 19.8, 18.1; ^{11}B (193 MHz, $CDCl_3$) δ -24.27 (q, J=96.3 Hz); HRMS (ESI) found MNa^+ 216.1895, $C_{12}H_{24}BNNa$ requires 216.1894.

***trans*-7-Benzyl-7-azabicyclo[4.1.0]heptane Borane (34a)**

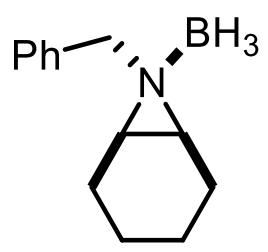

Following **GP4**, **34** (374.6 mg, 2 mmol, 1.0 equiv.) gave **34a** (305.7 mg, 76%) as a solid. R_f 0.22 [*n*-pentane:EtOAc (10:1)]. ^{1}H NMR (600 MHz, $CDCl_3$) δ 7.33 – 7.16 (5H, m), 3.80 (2H, s), 2.38 – 2.32 (2H, m), 2.08 – 1.99 (2H, m), 1.84 – 1.71 (2H, m), 1.65 – 1.02 (7H, m); ^{13}C NMR (151 MHz, $CDCl_3$) δ 133.8, 129.9, 128.2, 128.1, 68.8, 42.1, 19.4, 17.9; ^{11}B (193 MHz, $CDCl_3$) δ -19.69 (q, J = 97.4 Hz); HRMS (ESI) found MNa^+ 224.1574, $C_{13}H_{20}BNNa$ requires 224.1581.

The relative configuration was determined by a 2D ^{1}H-^{1}H NOESY (600 MHz, $CDCl_3$) experiment:

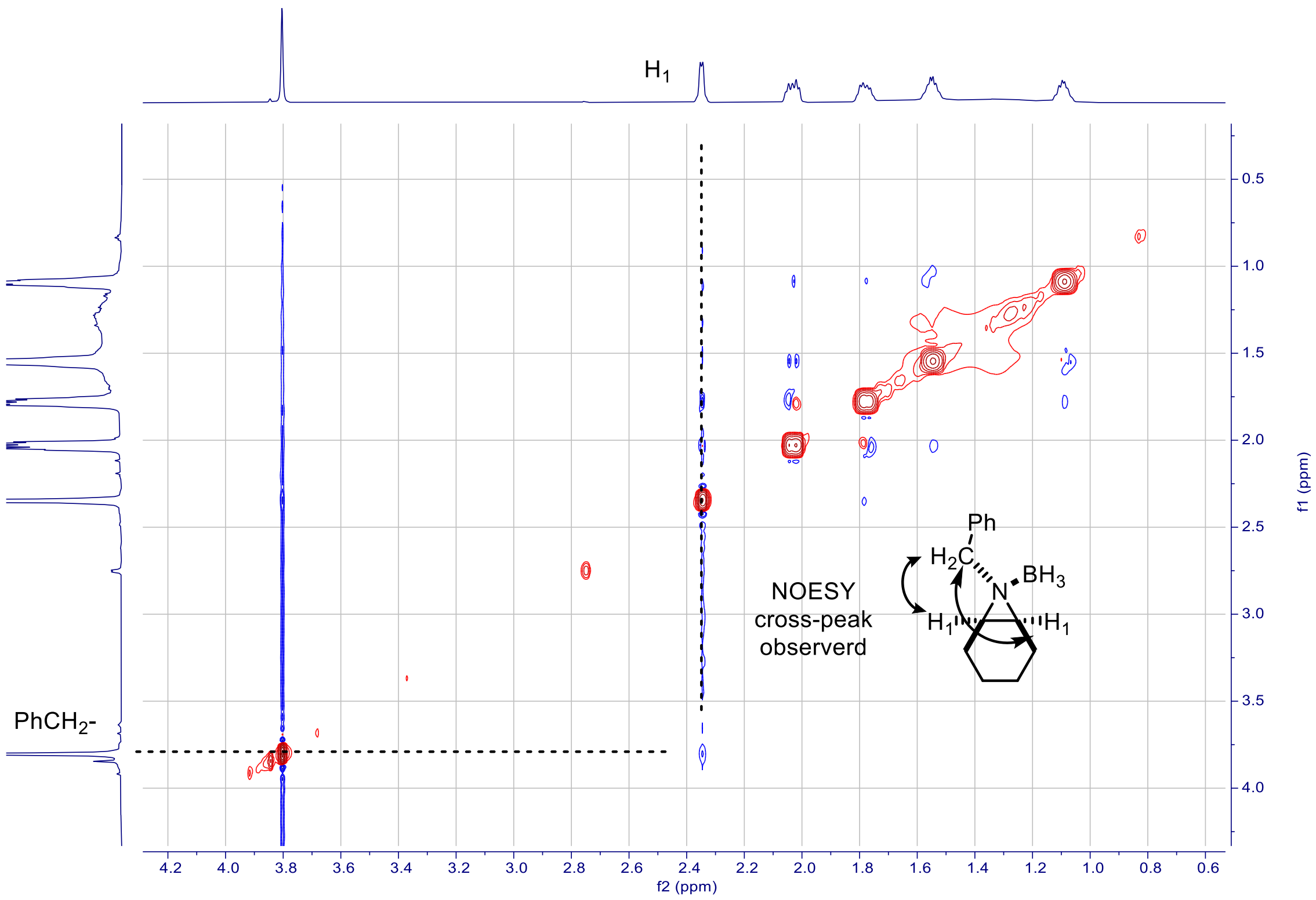

1-Methyl-2,2-dipentylaziridine borane (35a)

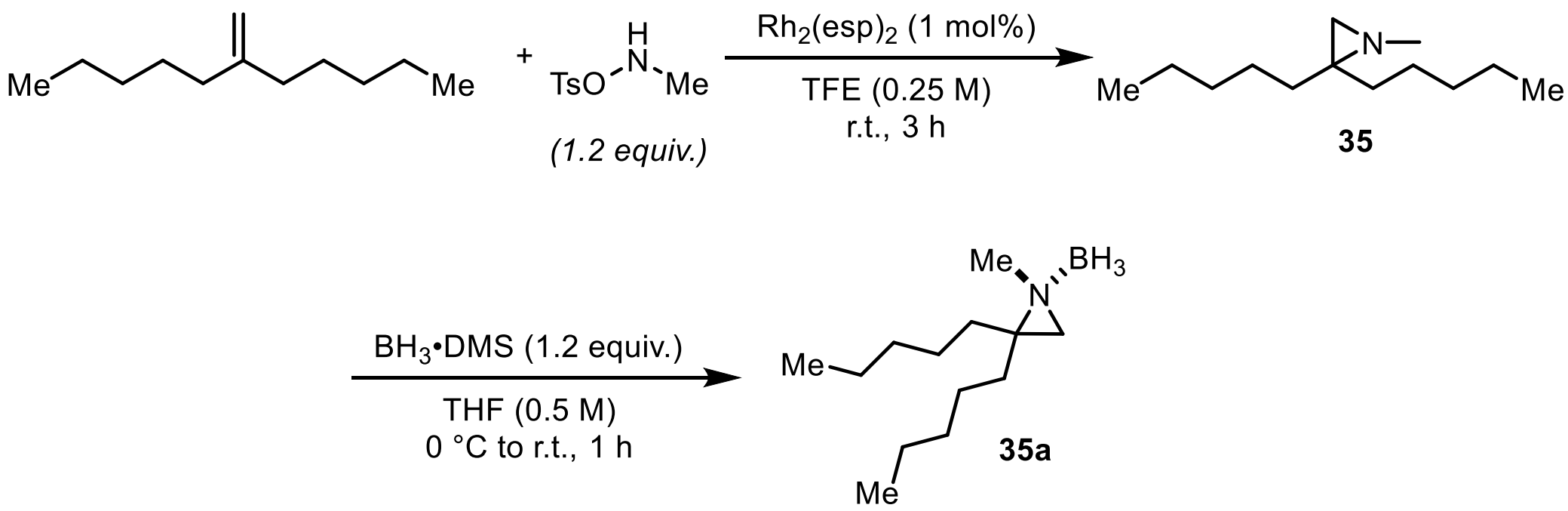

Step 1: A round bottom flask equipped with a stirring bar was charged with *N*-methyl-*O*-tosylhydroxylamine (723 mg, 3.6 mmol, 1.2 equiv.) The flask was evacuated and refilled with Ar (x 3). 6-methyleneundecane (505 mg, 3.0 mmol, 1.0 equiv.) and catalyst solution (12 mL of TFE containing $Rh_2(esp)_2$ (23 mg, 1 mol %), pre-stirred for 10 min) was then added under stirring. The reaction mixture was stirred at room temperature for 3 h. Upon completion, the reaction mixture was diluted with CH_2Cl_2 and washed with a saturated aqueous $NaHCO_3$ solution (x 2). The aqueous layer was extracted with CH_2Cl_2 (x 2), and the combined organic layers were dried over anhydrous Na_2SO_4. The solvent was evaporated to afford crude **35** directly for next step. *Note: the product is volatile, so the crude was used without being completely dried*.

Step 2: Following **GP4**, crude **35** and BH_3•DMS (3.6 mmol, 1.2 equiv.) in THF (6 mL) gave after purification via column chromatography **35a** (171 mg, 27% *over two steps*) as an oil. R_f 0.37 [*n*-

pentane:EtOAc (30:1)]. ^{1}H NMR (600 MHz, $CDCl_3$) δ 2.71 (3H, s), 2.19 (1H, d, *J*=1.7 Hz), 2.00 – 1.23 (21H, m), 0.94 – 0.86 (6H, m); ^{13}C (151 MHz, $CDCl_3$) δ 53.1, 48.5, 45.5, 32.0, 31.8, 30.4, 30.1, 25.8, 25.7, 22.6, 22.6, 14.1, 14.1; ^{11}B (193 MHz, $CDCl_3$) δ -13.47 (q, *J*=98.2 Hz); HRMS (ESI) found MNa^+ 234.2362, $C_{13}H_{30}BNNa$ requires 234.2364.

1-(2-(Naphthalen-1-yloxy)ethyl)aziridine Borane (36a)

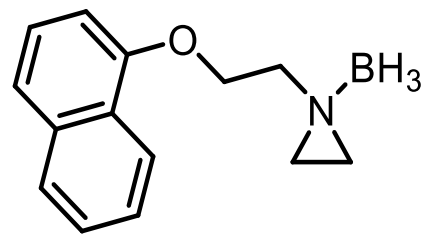

Following **GP4**, **36** (320 mg, 1.5 mmol, 1.0 equiv.) gave after purification via column chromatography **36a** (228 mg, 67%) as a solid. R_f 0.61 [*n*-pentane:EtOAc (1:1)]. ^{1}H NMR (600 MHz, $CDCl_3$) δ 8.18 – 8.14 (1H, m), 7.85 – 7.80 (1H, m), 7.54 – 7.45 (3H, m), 7.40 (1H, t, *J* = 7.9 Hz), 6.90 – 6.86 (1H, m), 4.63 (2H, t, *J* = 5.0 Hz), 3.17 (2H, t, *J* = 5.0 Hz), 2.45 (2H, d, *J* = 5.0 Hz), 2.38 (2H, d, *J* = 4.8 Hz), 1.67 (3H, q, *J* = 130.4 Hz); ^{13}C NMR (151 MHz, $CDCl_3$) δ 153.9, 134.7, 127.9, 126.6, 126.0, 125.5, 125.5, 121.6, 121.1, 105.2, 64.7, 60.9, 37.1; ^{11}B (193 MHz, $CDCl_3$) δ –12.81 (q, J = 99.4 Hz); HRMS (ESI) found MNa^+ 250.1371, $C_{14}H_{18}BNONa$ requires 250.1374.

***trans*-2-(3-Bromophenyl)-1-propylaziridine Borane (37a)**

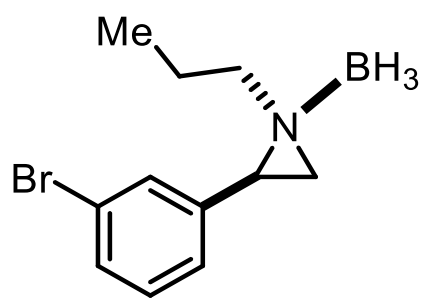

Following **GP4**, but adding BH_3•DMS at -78 °C and react at r.t. for 4 h, **37** (560 mg, 2.33 mmol, 1.0 equiv.) gave after precipitation and filtration **37a** (490 mg, 86%) as a solid. ^{1}H NMR (600 MHz, $CDCl_3$) δ 7.53 (1H, dt, *J* = 7.7, 1.6 Hz), 7.49 (1H, t, *J* = 1.8 Hz), 7.35 – 7.24 (2H, m), 3.33 (1H, t, *J* = 6.9 Hz), 3.12 (1H, td, *J* = 11.5, 4.9 Hz), 2.81 (1H, dd, *J* = 6.2, 2.0 Hz), 2.48 – 2.38 (2H, m), 2.18 – 2.06 (1H, m), 1.97 – 1.80 (1H, m), 1.48 – 0.96 (6H, m); ^{13}C NMR (151 MHz, $CDCl_3$) δ 133.3, 133.2, 132.2, 129.8, 128.9, 122.3, 67.1, 48.7, 41.4, 20.4, 11.4; ^{11}B NMR (192 MHz, $CDCl_3$) δ –16.05; HRMS (ESI) found MNa^+ 276.0526, $C_{11}H_{17}NBBrNa$ requires 276.0530.

The relative configuration was determined by ^{1}H NMR and ^{11}B NMR shift in analogy with the shift differences observed for **3a** and **3a'**.

4 Reaction Optimization

4.1 Optimization of the Arylation with Ph–B(OH)$_2$

An oven-dried vial equipped with a stirring bar was charged with *trans*-aziridine **1a** (0.1 mmol, 1.0 equiv.) and Ph–B(OH)$_2$ (with the corresponding equivalent stated in the Tables). The tube was capped with a Supelco aluminium crimp seal with septum (PTFE/butyl), evacuated and refilled with Ar (x 3). Dry and degassed solvent (0.5 mL) and the oxidant (with the corresponding equivalent stated in the Tables) were then added to the vial and stirred for around **1 min**. If necessary, the resulting mixture was cooled down to the corresponding temperature and the catalyst solution (0.5 mL of the corresponding dry and degassed solvent, containing copper catalyst and ligand with the corresponding loading stated in the Tables, pre-stirred for 10 min until fully dissolved) was then added under stirring. The resulting mixture was stirred at the corresponding temperature for the corresponding amount of time. Upon completion, 1,3,5-trimethoxybenzene (0.066 M in EtOAc, 0.5 mL, 0.033 mmol, 0.33 equiv.) and an aqueous solution of NaOH (2 mL, 1 M) was added, the aqueous layer was separated and extracted with EtOAc (2 x 3 mL). The combined organic layers were dried ($MgSO_4$) and filtered. The solvent was evaporated and analysed by ^{1}H NMR spectroscopy to determine the NMR yield.

Table S2. Screening of the ligand, ligand equivalent and base.

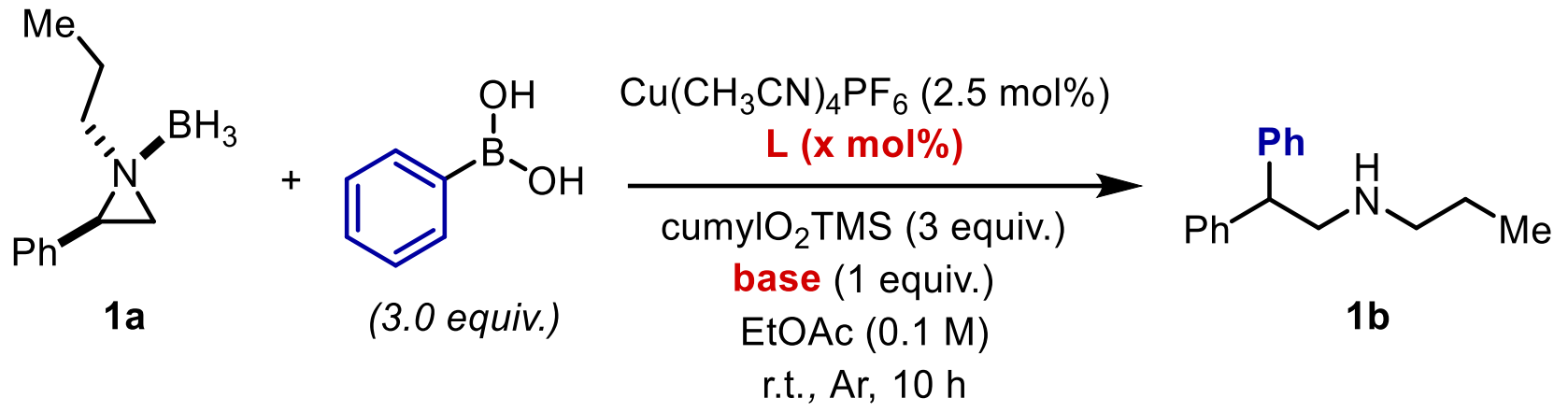

entry	ligand (x mol%)	base	1b (%)	1a (%)
**1**[a,b]	bathophen (2.7)	KH_2PO_4	36	0
**2**[a,b]	**L1** (2.7)	KH_2PO_4	26	n.d.
**3**[a,b]	**L2** (2.7)	KH_2PO_4	30	73
**4**[a,b]	**L3** (2.7)	KH_2PO_4	n.d.	58
**5**	bathophen (2.7)	$NaHCO_3$	47	n.d.
**6**	bpy (2.7)	$NaHCO_3$	31	n.d.
**7**	**L4** (2.7)	$NaHCO_3$	36	n.d.
**8**	**L5** (2.7)	$NaHCO_3$	32	n.d.
**9**	**L6** (2.7)	$NaHCO_3$	32	n.d.
**10**	dtbpy (2.7)	$NaHCO_3$	47	n.d.
**11**	**L7** (2.7)	$NaHCO_3$	18	n.d.
**12**	**L8** (2.7)	$NaHCO_3$	32	n.d.
**13**	**L9** (2.7)	$NaHCO_3$	17	n.d.
**14**[c]	dtbpy (3.5)	-	**61**	n.d.
**15**[c,d]	phen (3.5)	-	42	n.d.
**16**[c,d]	**L10** (3.5)	-	36	n.d.
**17**[c]	**L11** (3.5)	-	0	0
**18**[c]	**L12** (3.5)	-	0	0
**19**[c]	**L13** (3.5)	-	0	45
**20**[c]	**L14** (3.5)	-	0	64
**21**[c]	**L15** (3.5)	-	12	0
**22**[c]	**L16** (3.5)	-	0	51
**23**[c]	**L17** (3.5)	-	0	46
**24**[c]	**L18** (3.5)	-	0	72

[a]1.5 equivalents of $PhB(OH)_2$ was used. [b]8 h. [c]The reaction was run at 10 °C. [d]12 h.

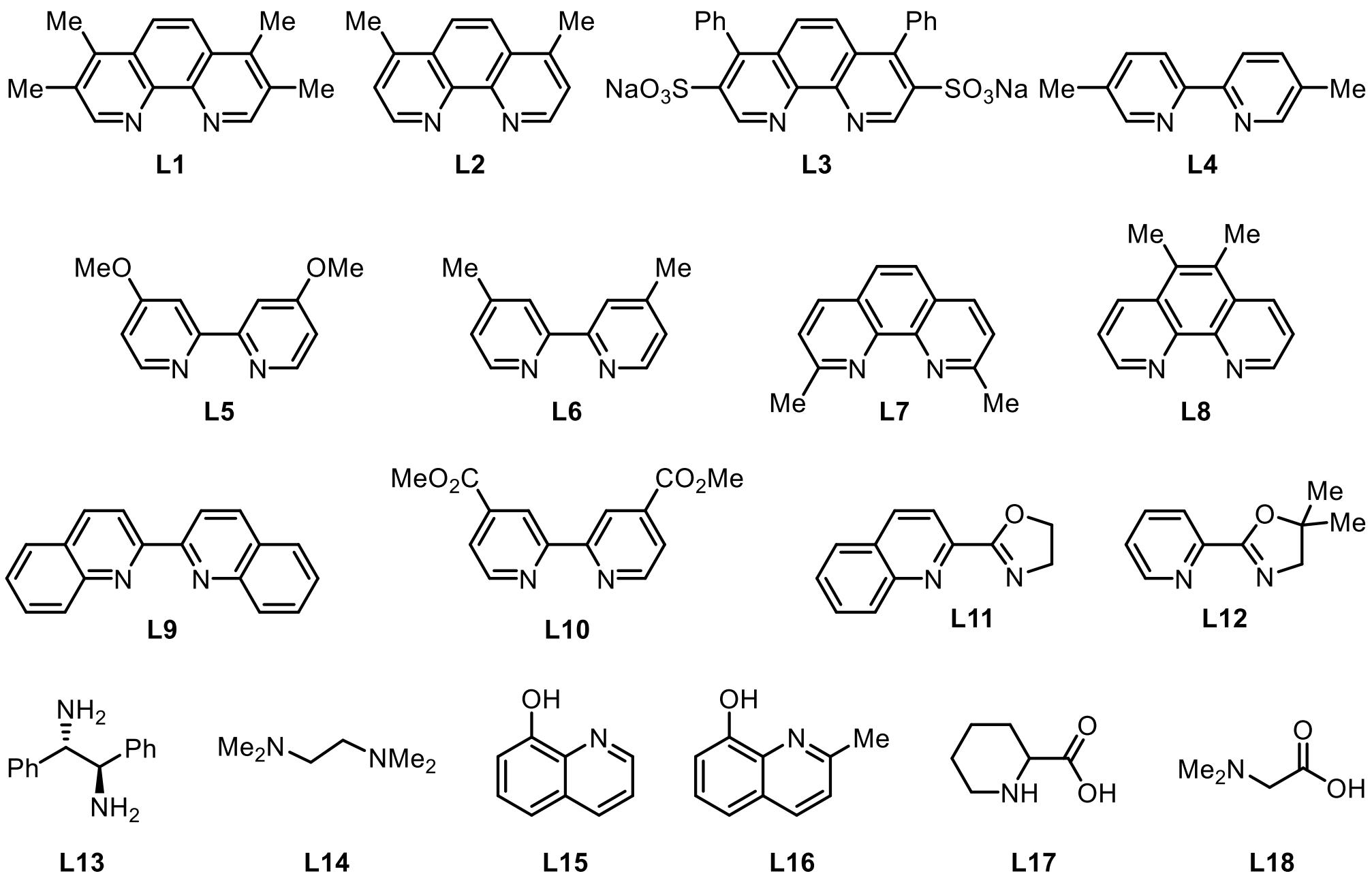

Table S3. Screening of the copper source.

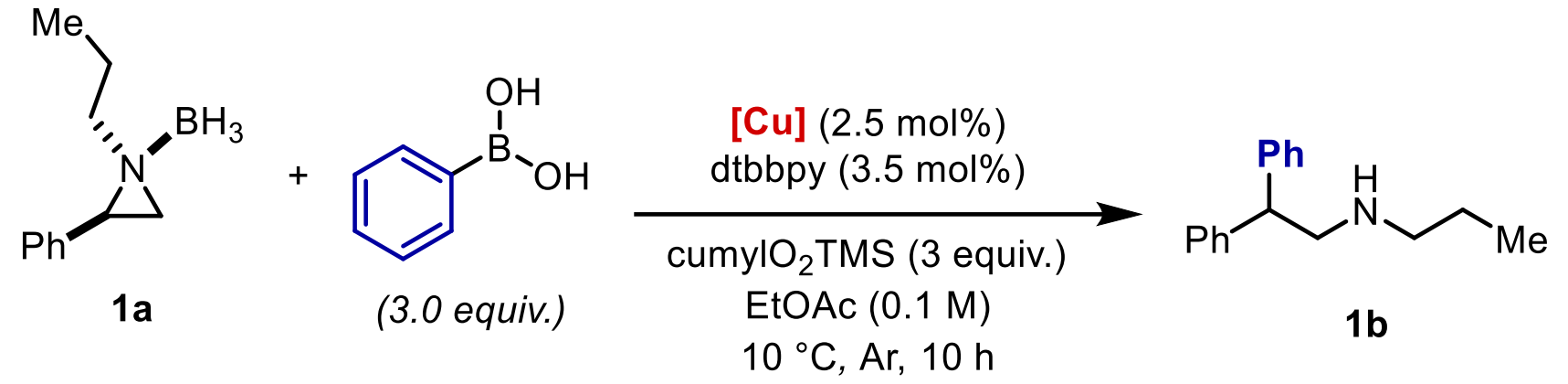

entry	[Cu]	1b (%)	1a (%)
**1**	CuCl	47	n.d.
**2**[a]	CuBr•DME	n.d.	22
**3**[a]	$Cu(CH_3CN)_4BF_4$	n.d.	25
**4**[a]	$Cu(CH_3CN)_4OTf$	n.d.	35
**5**	CuOAc	43	n.d.
**6**	$Cu(OTf)_2$	54	n.d.
**7**	$[Cu(OTf)]_2$•PhH	56	n.d.
**8**	CuBr	47	n.d.
**9**	$CuSO_4$	n.d.	56
**10**	CuCN	n.d.	28
**11**	$Cu(acac)_2$	53	n.d.
**12**[a]	**Cu-1**	**61**	n.d.
**13**[a]	**Cu-2**	46	n.d.
**14**[a]	**Cu-3**	53	n.d.
**15**[a]	**Cu-4**	45	n.d.
**16**	$Cu(CH_3CN)_4PF_6$	**61**	n.d.

[a]12 h.

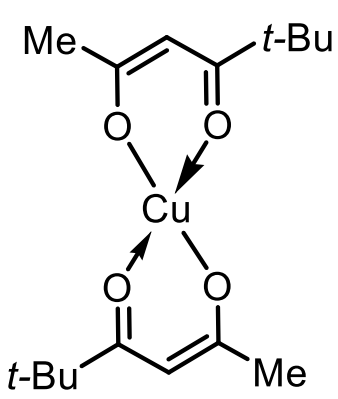

Cu-1

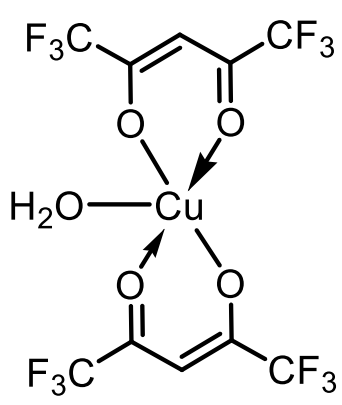

Cu-2

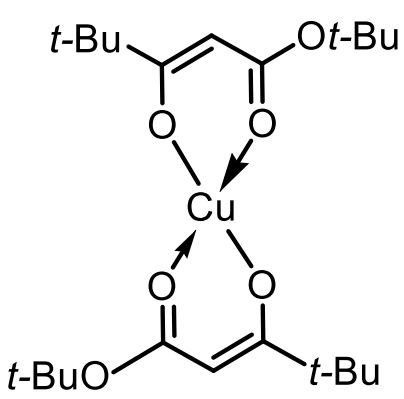

Cu-3

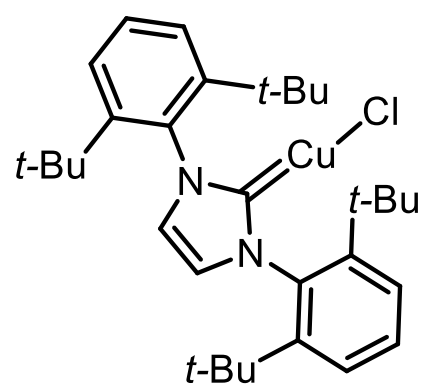

Cu-4

Table S4. Screening of the solvent.

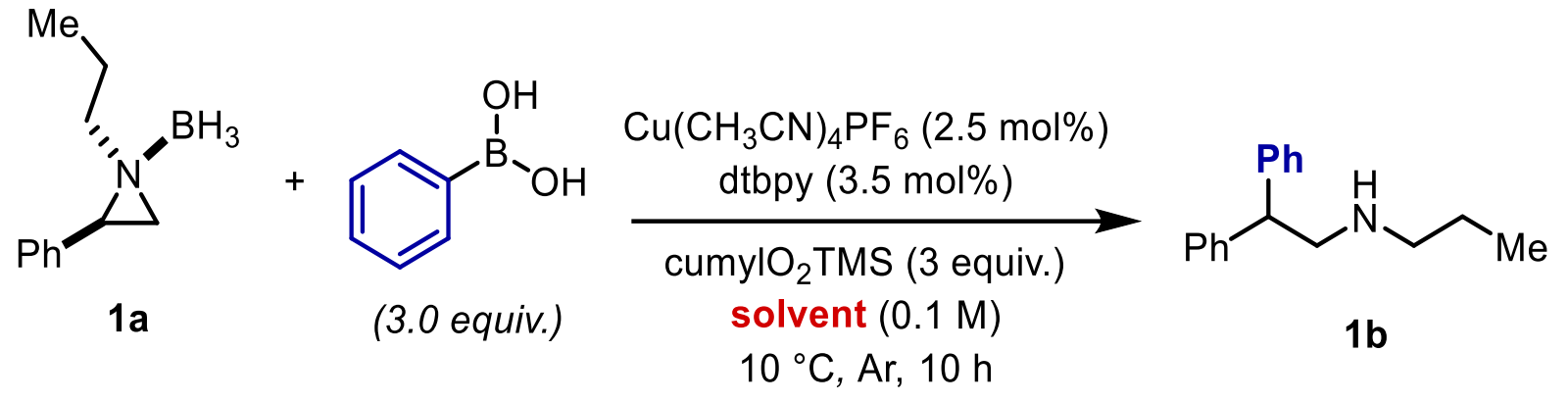

entry	solvent	1b (%)	1a (%)
**1**	EtOAc	**61**	n.d.
**2**	CH_2Cl_2	34	35
**3**	EtOAc/DMA (4:1)	14	39
**4**	PhH	5	30
**5**	EtOMe	58	n.d.
**6**	PhCl	20	42
**7**	PhF	16	46
**8**	EtOAc/CH_3CN (19:1)	51	n.d.
**9**	EtOAc/CH_3CN (9:1)	46	n.d.
**10**	acetone	21	n.d.
**11**	EtOAc/acetone (2:1)	**60**	n.d.
**12**	EtOAc/acetone (9:1)	49	n.d.
**13**	EtOAc/CH_2Cl_2 (7:3)	n.d.	27
**14**	EtOAc/$PhCF_3$ (7:3)	**60**	n.d.
**15**	EtOAc/$PhCF_3$ (9:1)	44	n.d.

Table S5. Screening of the base and the base equivalent.

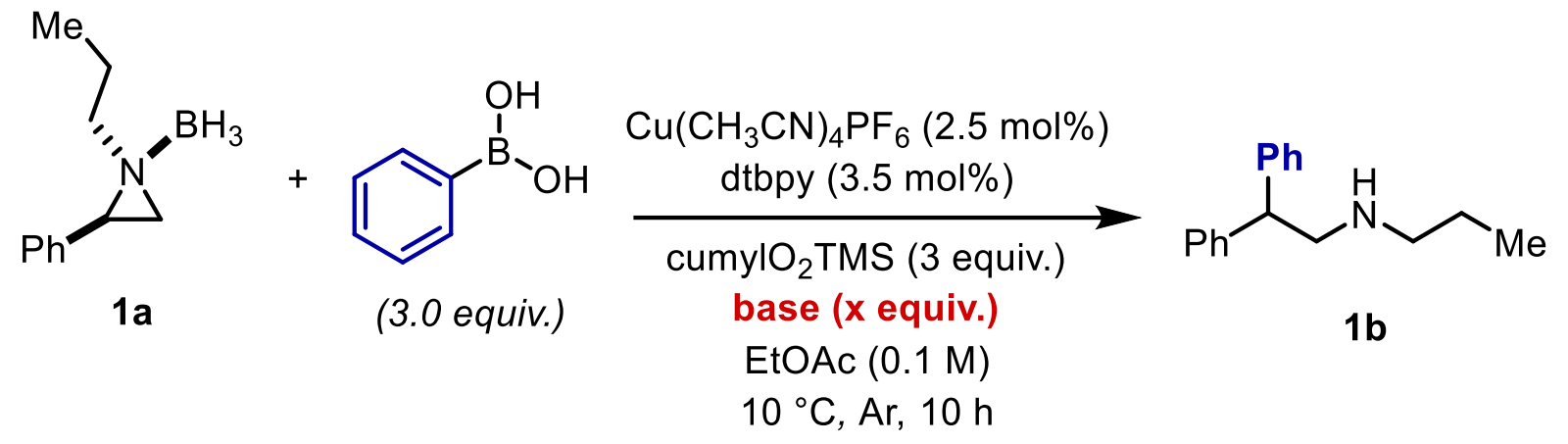

entry	base (equiv.)	1b (%)	1a (%)
**1**	-	**61**	n.d.
**2**	Li_2CO_3 (1.5)	57	n.d.
**3**	H_3BO_3 (1.5)	12	24
**4**	DBN (1.5)	6	56
**5**	pyridine (1.5)	14	11
**6**	BTMG (2)	11	58
**7**	Et_3N (1.5)	15	54
**8**	TMP (1.5)	14	20
**9**	*t*-BuOK (1.5)	33	11
**10**	LiF (1.5)	51	n.d.
**11**	LiF (3)	51	n.d.
**12**	BzOK (1.5)	38	12
**13**	BzOK (3)	36	10
**14**	LiBr (2)	11	n.d.
**15**	LiI (2)	11	n.d.
**16**	LiO*t*-Bu (2)	n.d.	n.d.
**17**	LiOH (2)	8	n.d.
**18**	LiTFA (2)	18	n.d.
**19**	$LiOAc·2H_2O$ (2)	12	10

BTMG = 2-*t*-Bu-1,1,3,3-tetramethylguanidine; TMP = 2,2,6,6-tetramethylpiperidine

Table S6. Screening of the temperature.

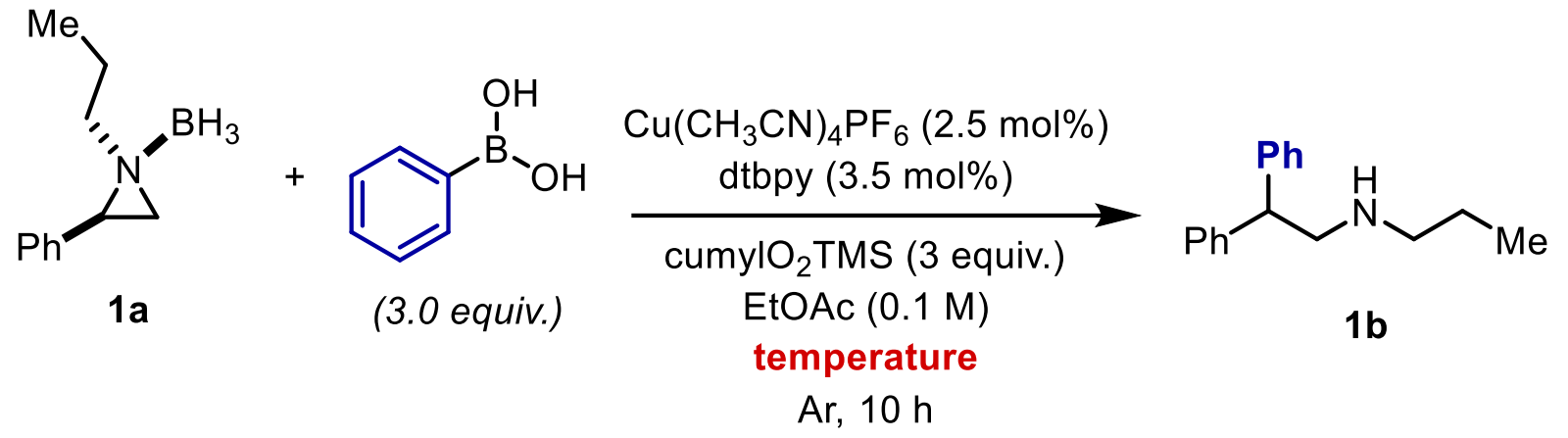

entry	temperature	1b (%)	1a (%)
**1**	−10 °C	52	n.d.
**2**	0 °C	57	n.d.
**3**	10 °C	**61**	n.d.
**4**	r.t.	47	n.d.
**5**	45 °C	43	n.d.

Table S7. Screening of the oxidant, oxidant equivalent, base and temperature.

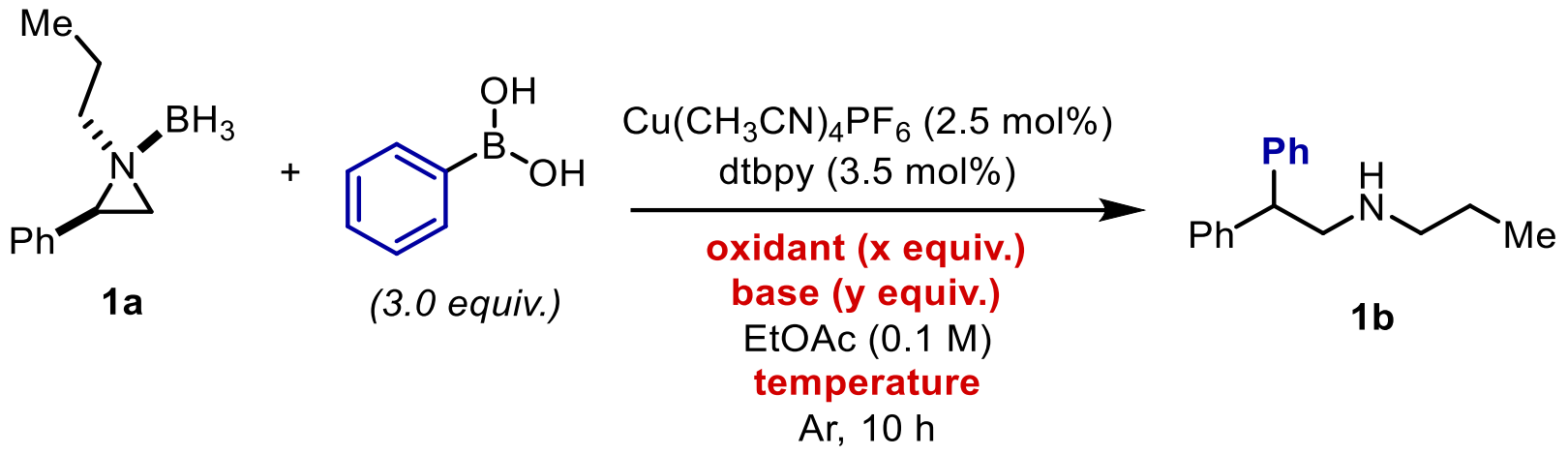

entry	oxidant (equiv.)	base (equiv.)	temperature	1b (%)	1a (%)
**1**	cumylO_2TMS (2)	KH_2PO_4 (1.0)	rt	35	28
**2**	cumylO_2TMS (3)	KH_2PO_4 (1.0)	rt	46	n.d.
**3**	cumylO_2TMS (4)	KH_2PO_4 (1.0)	rt	42	n.d.
**4**	NFSI (3)	KH_2PO_4 (1.0)	rt	n.d.	n.d.
**5**	(*t*-BuO)$_2$ (3)	KH_2PO_4 (1.0)	rt	n.d.	90
**6**	(cumylO)$_2$ (3)	KH_2PO_4 (1.0)	rt	n.d.	>95
**7**	cumylO_2TES (3)	KH_2PO_4 (1.0)	rt	44	n.d.
**8**	*t*-BuO_2TMS (3)	KH_2PO_4 (1.0)	rt	30	26
**9**	cumylO_2*i*-Pr (3)	-	10 °C	n.d.	90
**10**	cumylO_2TBS (3)	-	10 °C	n.d.	74
**11**	cumylO_2TMS (2.5)	-	10 °C	54	n.d.
**12**	cumylO_2TMS (3)	-	10 °C	**61**	n.d.
**13**	cumylO_2TMS (3.5)	-	10 °C	54	n.d.
**14**	cumylO_2TMS (4)	-	10 °C	49	n.d.
**15**[a]	BPO (3)	-	10 °C	<5	33
**16**[a]	LPO (3)	-	10 °C	10	36
**17**[a]	TBPB (3)	-	10 °C	n.d.	43

[a]The reaction was run for 16 h. NFSI = *N*-Fluorobenzenesulfonimide; BPO = Benzoyl peroxide; LPO = Lauroyl peroxide; TBPB = *tert*-Butyl peroxybenzoate

Table S8. Screening of phenylboronic acid equivalent and catalyst/ligand loading.

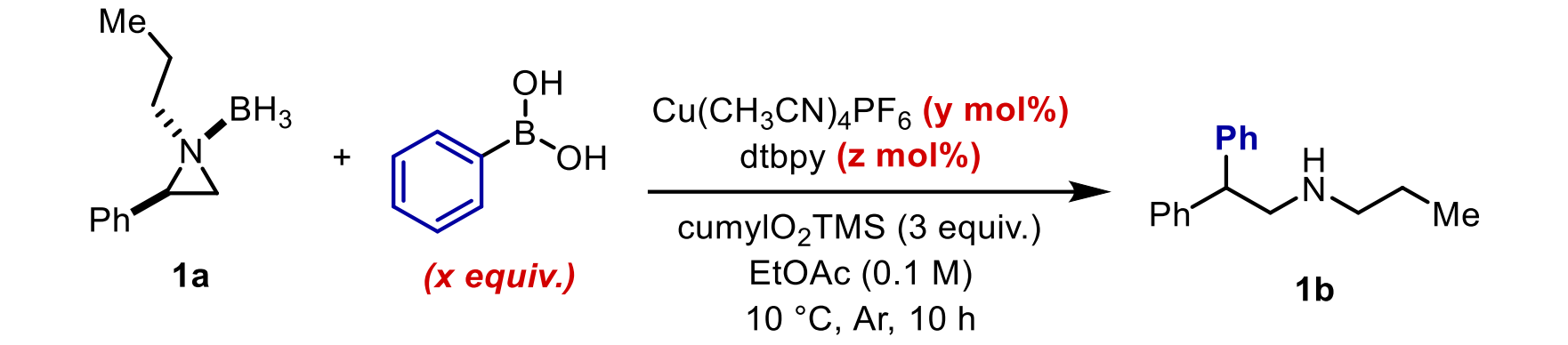

entry	x	y	z	1b (%)	1a (%)
**1**	2.5	2.5	3.5	47	n.d.
**2**	3	2.5	3.5	**61**	n.d.
**3**	3.5	2.5	3.5	56	n.d.
**4**	5	2.5	3.5	48	n.d.
**5**	3	5	7	56	n.d.

4.2 Control Reactions

Table S9. Influence of each reagent and time on the reaction outcome.

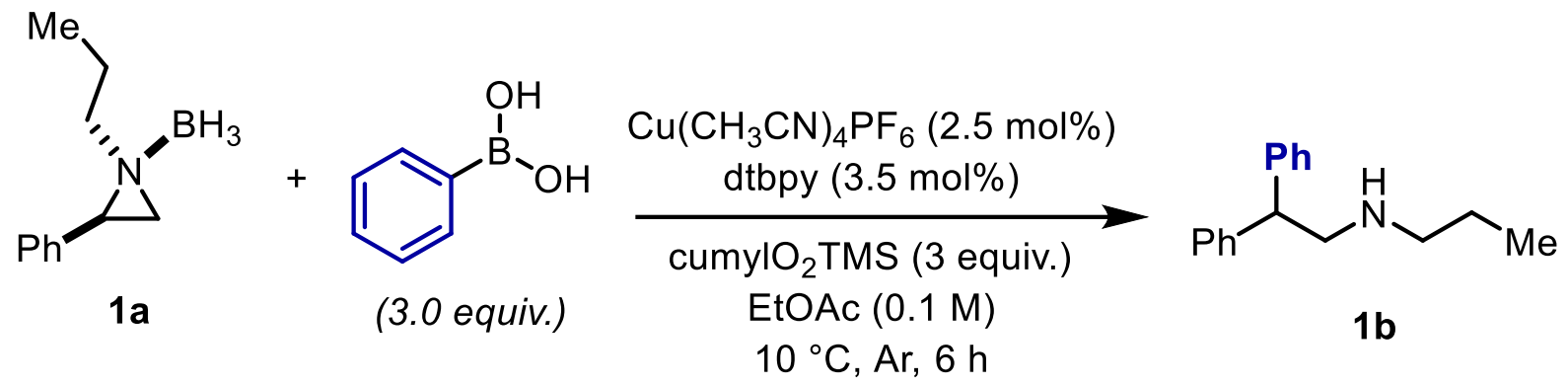

entry	modifications	1b (%)	1a (%)
**1**	-	55	n.d.
**2**	no cumylO_2TMS	n.d.	85
**3**[a]	no Ph–B$(OH)_2$	n.d.	12
**4**	no Cu/dtbpy	n.d.	84
**5**	no cumylO_2TMS, no Ph–B$(OH)_2$	n.d.	97
**6**	no cumylO_2TMS, no Cu/dtbpy	n.d.	76
**7**	no Ph–B$(OH)_2$, no Cu/dtbpy	n.d.	95
**8**	no Ph–B$(OH)_2$, no Cu/dtbpy, no cumylO_2TMS	n.d.	98
**9**	time = 1 h	15	21
**10**	time = 2 h	21	10
**11**	time = 4 h	53	n.d.

4.3 Optimization of the Arylation with Electron-Deficient Boronic Acids

Table S10. Screening of boronic acid equivalent and solvent in the case of boronic acids with electron withdrawing groups.

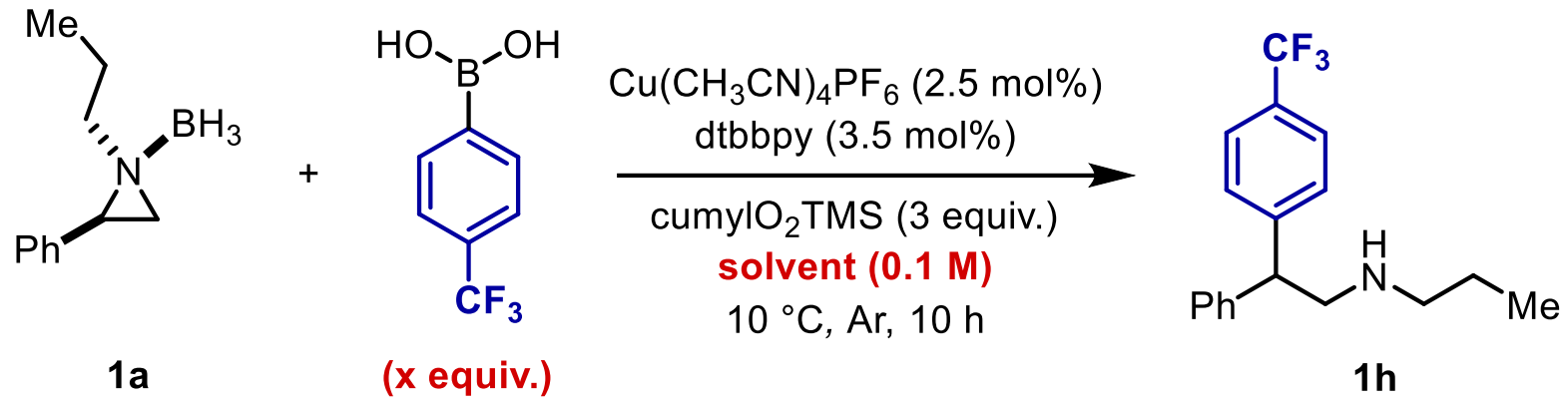

entry	x	solvent	1h (%)	1a (%)
**1**	3	EtOAc	19	n.d.
**2**	3	EtOAc:acetone (2:1)	22	n.d.
**3**	4	EtOAc:acetone (2:1)	30	n.d.
**4**	3	EtOAc:PhCF$_3$ (7:3)	20	n.d.
**5**	4	EtOAc:PhCF$_3$ (7:3)	23	n.d.
**6**	5	EtOAc:PhCF$_3$ (7:3)	20	n.d.
**7**	4	EtOAc:MTBE (2:1)	<5	n.d.
**8**	4	EtOAc:PhF (2:1)	35	n.d.
**9**	4	EtOAc:DCE (2:1)	17	n.d.
**10**	4	EtOAc:CH$_3$CN (7:3)	**41**	n.d.

4.4 Optimization of the Arylation with Electron-Rich Boronic Acids

Table S11. Screening of boronic acid equivalent and solvent in the case of boronic acids with electron rich groups.

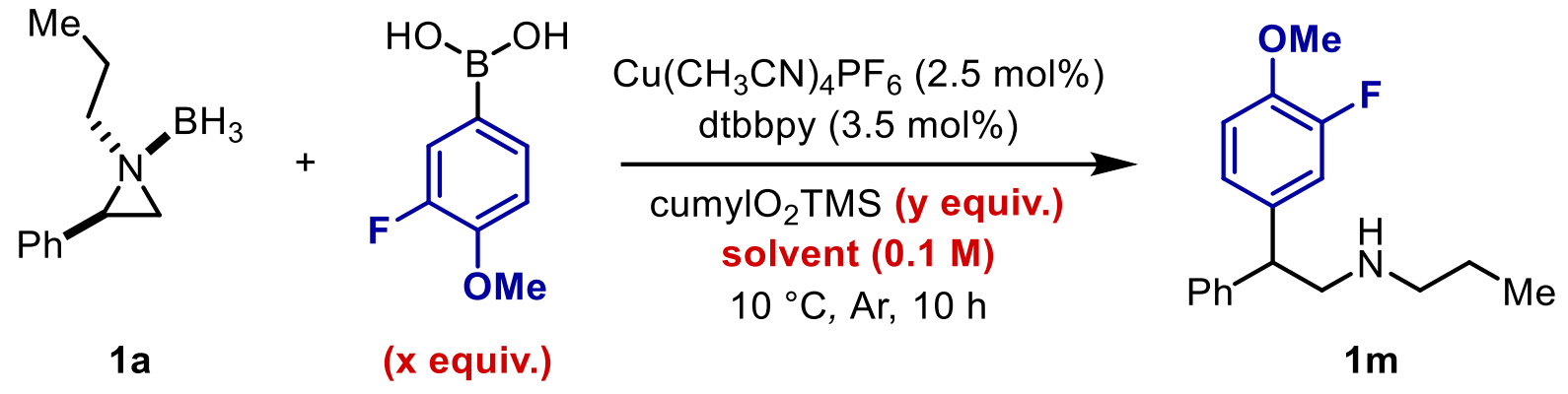

entry	x	y	solvent	1m (%)	1a (%)
**1**	3	3	EtOAc	30	n.d.
**2**	2	3	EtOAc:acetone (2:1)	20	n.d.
**3**	3	3	EtOAc:acetone (2:1)	35	n.d.
**4**[a]	3	3	EtOAc:acetone (2:1)	30	n.d.
**5**	4	3	EtOAc:acetone (2:1)	**40**	n.d.
**6**	5	3	EtOAc:acetone (2:1)	41	n.d.
**7**	4	4	EtOAc:acetone (2:1)	35	n.d.
**8**[b]	4	3	EtOAc:acetone (2:1)	30	n.d.

[a]$Cu(CH_3CN)_4PF_6$ (5 mol%), dtbbpy (7 mol%). [b]Bathophen was used instead of dtbpy.

4.5 Optimization of the Cyanation with TMSCN

Table S12. Screening of the solvent.

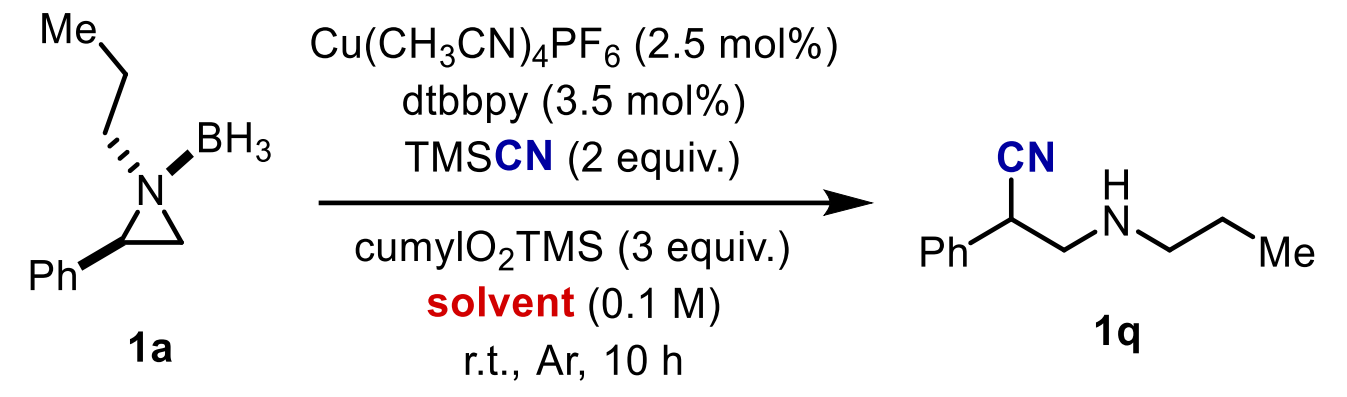

entry	solvent	1q (%)	1a (%)
**1**	EtOAc	48	n.d.
**2**	acetone	14	n.d.
**3**	CH_3CN	**56**	n.d.
**4**	CH_2Cl_2	56	n.d.
**5**	DCE	52	n.d.
**6**	PhF	49	n.d.
**7**	$PhCF_3$	51	n.d.
**8**	MTBE	<5	76

Table S13. Screening of the ligand and ligand equivalent.

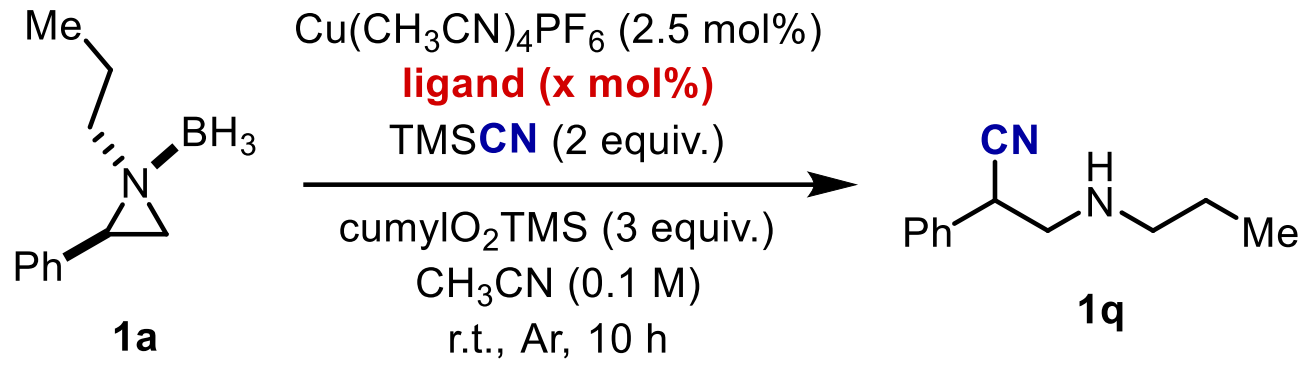

entry	ligand	1q (%)	1a (%)
**1**	bpy (3.5)	49	n.d.
**2**	**L1** (3.5)	53	n.d.
**3**	**L2** (3.5)	30	n.d.
**4**	dtbbpy (3.5)	**56**	n.d.
**5**	**L3** (3.5)	41	n.d.
**6**	**L4** (3.5)	31	29
**9**	**L5** (2.7)	n.d.	32
**10**	**L6** (2.7)	38	n.d.
**11**	phen (3.5)	56	n.d.
**12**	**L7** (3.5)	37	n.d.
**13**	bathophen (2.7)	54	n.d.
**14**	**L8** (3.5)	30	n.d.
**15**	**L9** (3.5)	50	n.d.
**16**	**L10** (3.5)	38	n.d.
**17**	**L11** (3.5)	10	70
**18**	**L12** (3.5)	16	61
**19**	**L13** (3.5)	n.d.	28

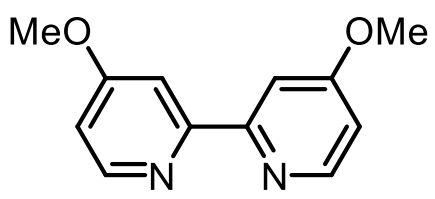

L1

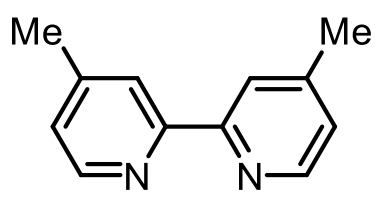

L2

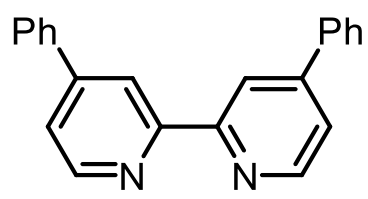

L3

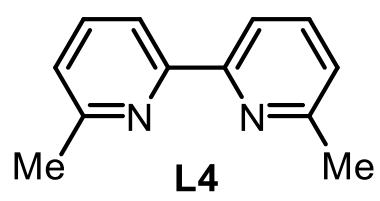

L4

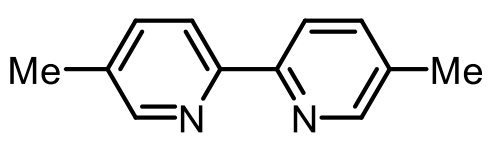

L5

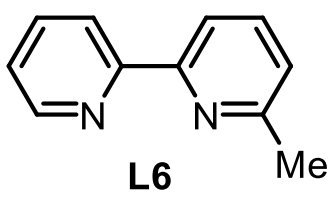

L6

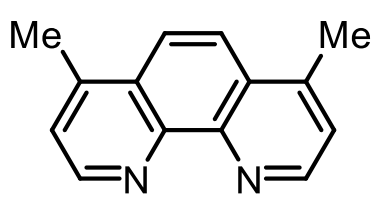

L7

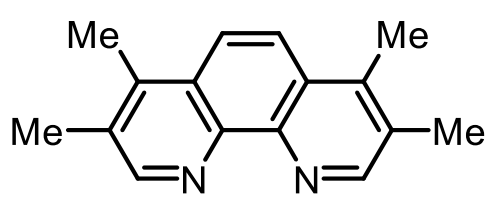

L8

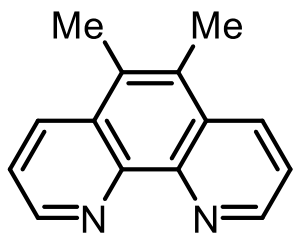

L9

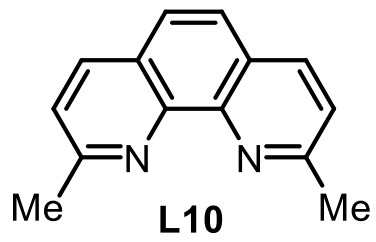

L10

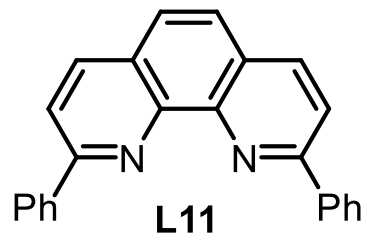

L11

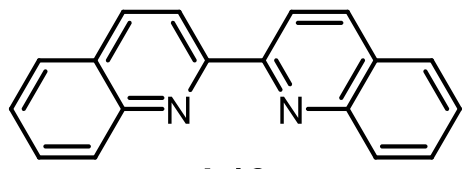

L12

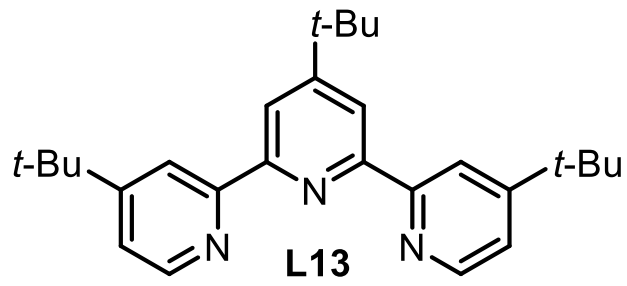

L13

Table S14. Screening of the time.

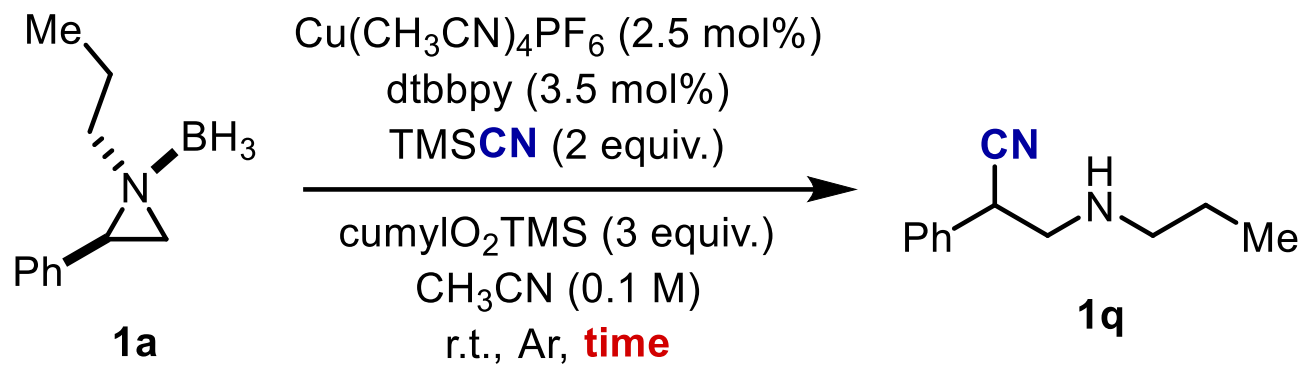

entry	time	1q (%)	1a (%)
**1**	1 h	11	56
**2**	2 h	65	n.d.
**3**	10 h	56	n.d.
**4**	20 h	50	n.d.

4.6 Control Reactions for Cyanation and Thioetherification

Table S15. Control reactions for Cyanation.

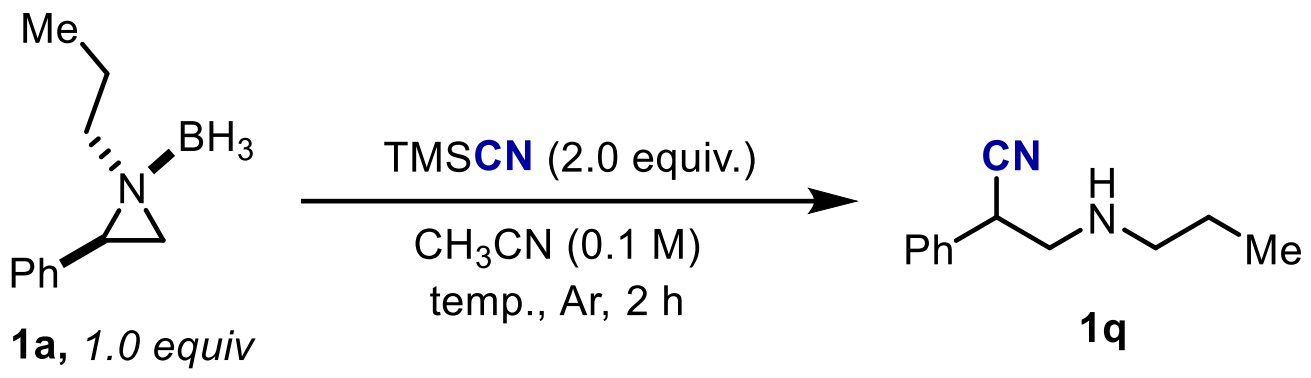

entry	T (ºC)	1q (%)	1a (%)
**1**	25	n.d.	96
**2**	80	n.d.	59

Table S16. Control reactions for thioetherification.

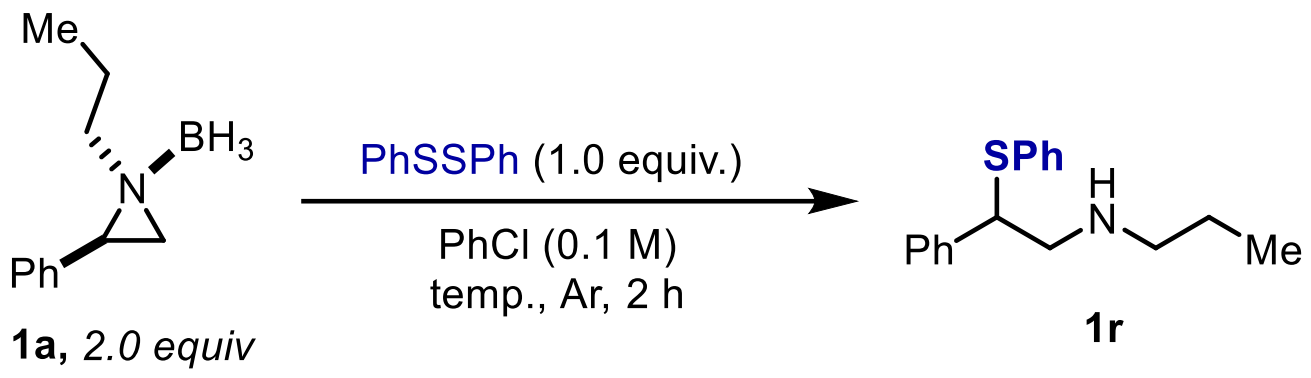

entry	T (ºC)	1r (%)	1a (%)
**1**	25	n.d.	98
**2**	100	n.d.	9

4.7 Optimization of Photo–Nickel Dual Catalysis

An oven-dried microwave vial was charged with *trans*-aziridine **1a** (0.1 mmol, 1.0 equiv.), methyl 4-bromobenzoate (0.2 mmol, 2.0 equiv.), photocatalyst (with the corresponding equivalent stated in the Tables), nickel catalyst and/or ligand (with the corresponding equivalent stated in the Tables) and K_3PO_4 (42 mg, 2.0 equiv.). The tube was capped with a Supelco aluminium crimp seal with septum (PTFE/butyl), evacuated and refilled with Ar (x 3). Dry and degassed EtOAc (1.0 mL) was then added to the vial and the mixture was stirred for **1 min** at room temperature and then was placed 4 cm from the 390 nm purple LEDs. The LEDs were switched on (kessil, 100 W) and the mixture was stirred under irradiation with a fan for 16 hours. Upon completion, aqueous NaOH (2 mL, 1 M) was added, the aqueous layer was separated and extracted with EtOAc (3 mL). The aliquot from organic phase was taken for GC anaylsis.

Table S17. Screening of the nickel catalyst and the ligand.

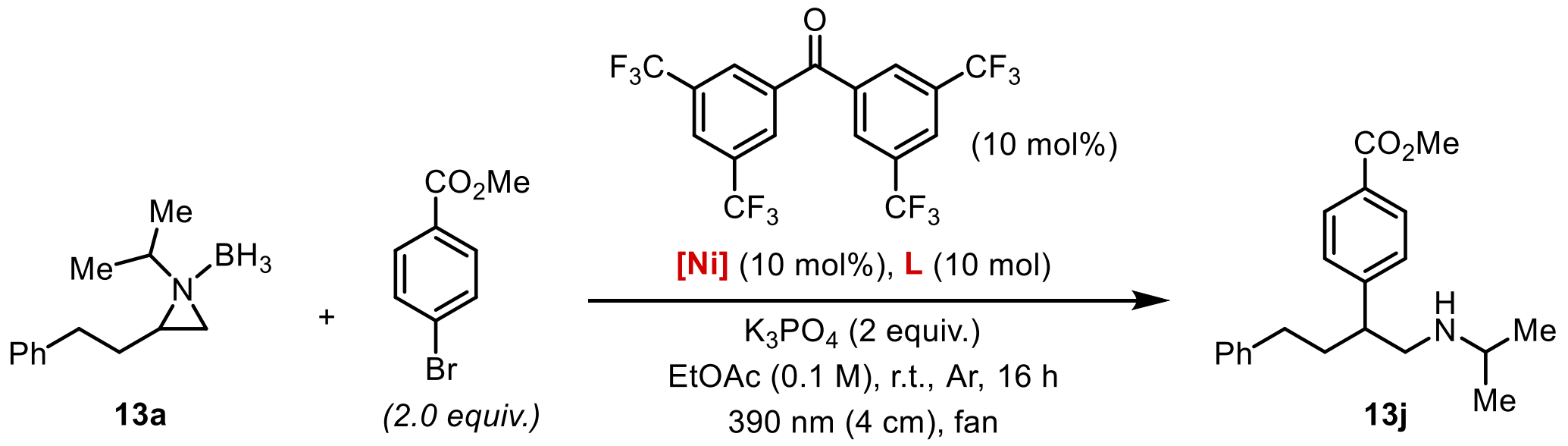

entry	[Ni]	L	13j (%)	13a (%)	ArBr (%)
1	$Ni(dtbpy)Br_2$		**82**	2	n.d.
2	$NiBr_2$•DME	bpy	3	81	89
3	$NiBr_2$•DME	4,4'-diMe-bpy	1	85	79
4	$NiBr_2$•DME	4,4'-diOMe-bpy	1	68	82
5	$NiBr_2$•DME	phen	n.d.	85	98
6	$NiBr_2$•DME	dtbpy	76	5	2
7	$Ni(OAc)_2$•$4H_2O$	dtbpy	2	77	57
8	$Ni(acac)_2$	dtbpy	n.d.	82	93
9	$NiCl_2$•DME	dtbpy	6	71	84
10	$Ni(NO_3)_2$•$6H_2O$	dtbpy	77	17	6
11	$NiCl_2$•$6H_2O$	dtbpy	1	82	89

Table S18. Screening of the photocatalyst.

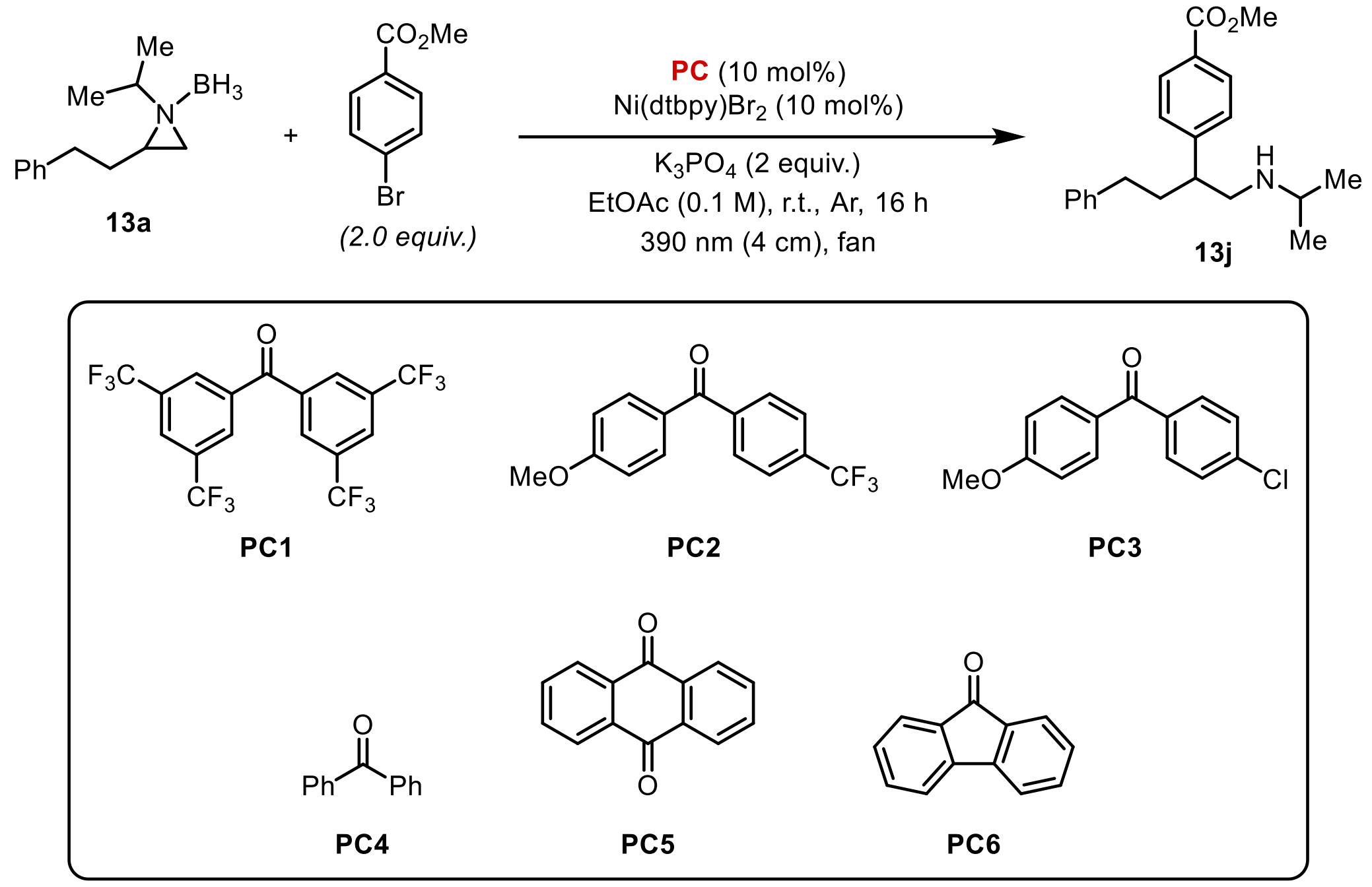

entry	PC	13j (%)	13a (%)	ArBr (%)
1	**PC1**	**82**	2	n.d.
2	**PC2**	19	57	39
3	**PC3**	9	63	16
4	**PC4**	25	53	55
5	**PC5**	30	41	33
6	**PC6**	1	85	52

Table S19. Screening of the base.

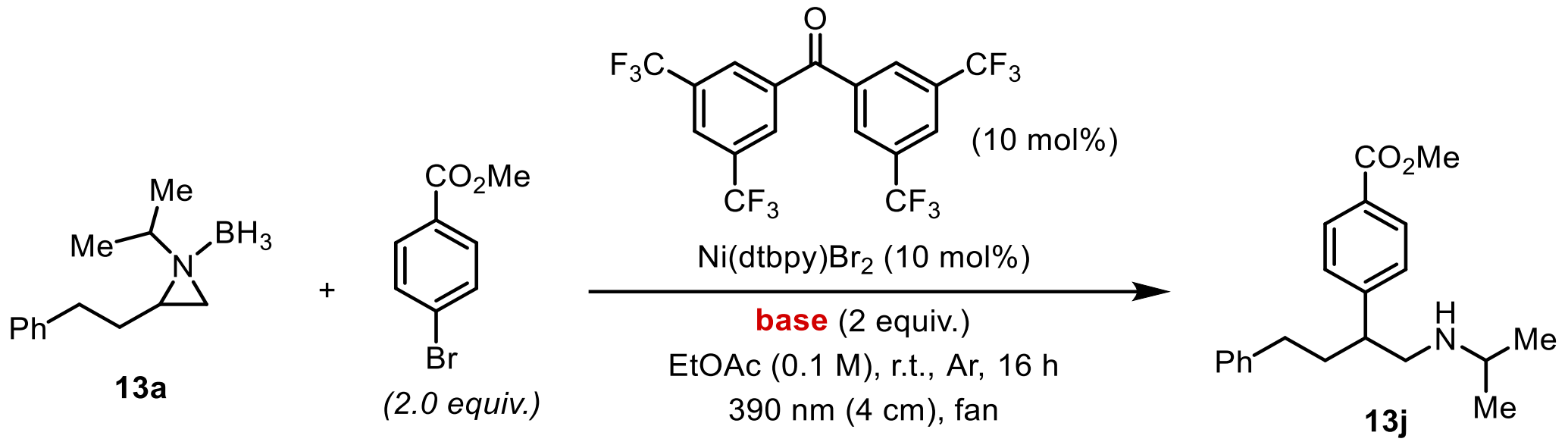

entry	base	13j (%)	13a (%)	ArBr (%)
1	K_3PO_4	**82**	2	n.d.
2	Na_2CO_3	n.d.	n.d.	n.d.
3	Cs_2CO_3	54	20	4

Table S20. Screening of the solvent.

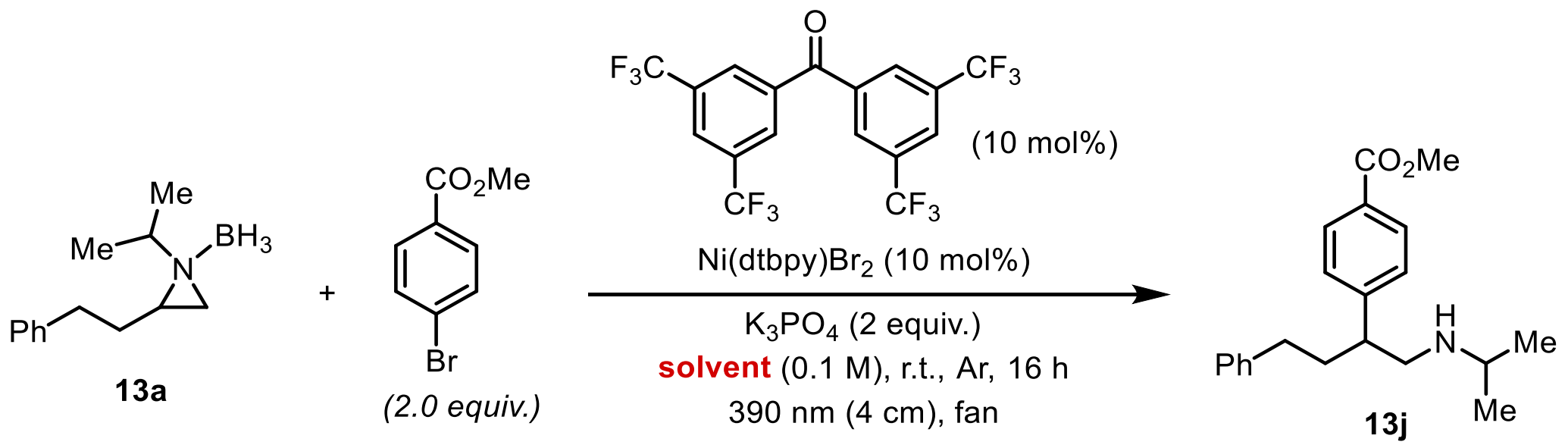

entry	solvent	13j (%)	13a (%)	ArBr (%)
1	EtOAc	**82**	2	n.d.
2	acetone	17	4	n.d.
3	CH_3CN	24	28	n.d.
4	DCE	17	47	66

Table S21. Screening of the amount aryl bromide, photocatalyst, nickel catalyst, base and solvent concentration.

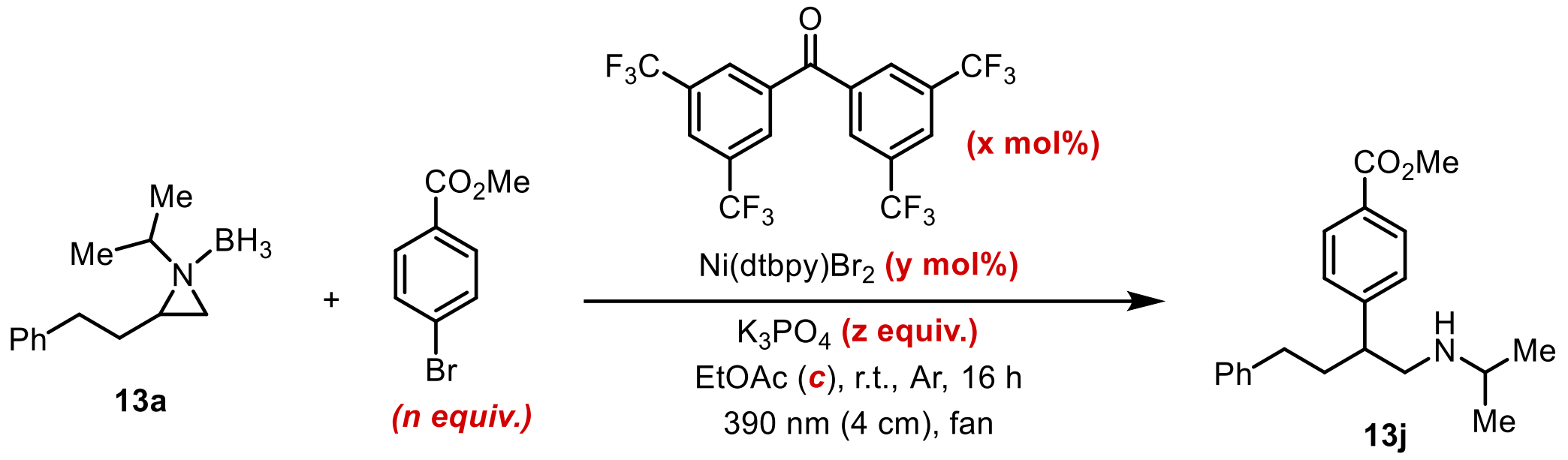

entry	ArBr (equiv.)	x	y	z (equiv.)	*c*	13j (%)	13a (%)	ArBr (%)
1	2.0	5%	10%	2.0	0.1 M	66	15	4
2	2.0	10%	10%	2.0	0.1 M	**82**	2	n.d.
3	2.0	15%	10%	2.0	0.1 M	69	1	1
4	2.0	20%	10%	2.0	0.1 M	66	13	n.d.
5	2.0	10%	5%	2.0	0.1 M	72	3	n.d.
6	2.0	10%	15%	2.0	0.1 M	54	3	n.d.
7	1.2	10%	10%	2.0	0.1 M	49	21	n.d.
8	1.5	10%	10%	2.0	0.1 M	70	12	n.d.
9	2.5	10%	10%	2.0	0.1 M	**84**	4	26
10	2.0	10%	10%	1.0	0.1 M	43	32	66
11	2.0	10%	10%	1.5	0.1 M	69	4	10
12	2.0	10%	10%	2.5	0.1 M	73	4	n.d.
13	2.0	10%	10%	1.0	0.2 M	72	5	5

5 Substrate scope

5.1 Ring-Opening–Arylation

N-(2,2-Diphenylethyl)propan-1-amine (1b)

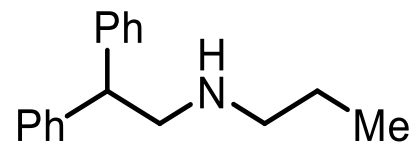

Following **GP5a**, *trans*-aziridine borane **1a** (18 mg, 0.1 mmol, 1.0 equiv.) and phenylboronic acid (37 mg, 0.3 mmol, 3.0 equiv.) gave **1b** (61%) as an oil. R_f 0.15 [EtOAc]. ^{1}H NMR (600 MHz, $CDCl_3$) δ 7.31 (4H, t, *J* = 7.5 Hz), 7.28 (4H, d, *J* = 6.8 Hz), 7.21 (2H, t, *J* = 7.1 Hz), 4.23 (1H, t, *J* = 7.7 Hz), 3.25 (2H, d, *J* = 7.7 Hz), 2.62 (2H, t, *J* = 7.3 Hz), 1.46 (2H, sx, *J* = 7.3 Hz), 1.34 (NH, br s), 0.86 (3H, t, *J* = 7.4 Hz); ^{13}C NMR (151 MHz, $CDCl_3$) δ 143.0, 128.7, 128.1, 126.6, 54.5, 51.8, 51.2, 23.0, 11.8. Data in accordance with the literature.[17]

N-(2-Phenyl-2-(*p*-tolyl)ethyl)propan-1-amine (1c)

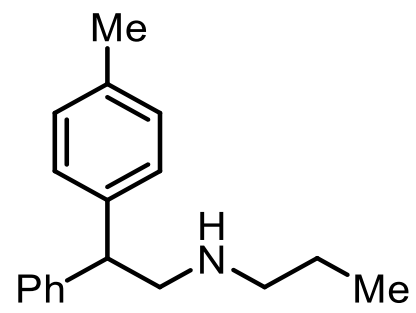

Following **GP5a**, *trans*-aziridine borane **1a** (18 mg, 0.1 mmol, 1.0 equiv.) and *p*-tolylboronic acid (41 mg, 0.3 mmol, 3.0 equiv.) gave **1c** (73%) as an oil. R_f 0.2 [EtOAc]. ^{1}H NMR (600 MHz, $CDCl_3$) δ 7.29 (2H, t, *J* = 7.5 Hz), 7.25 (2H, dd, *J* = 7.2,1.3 Hz), 7.21 – 7.17 (1H, tt, *J* = 7.2,1.5 Hz), 7.15 (2H, d, *J* = 8.1 Hz), 7.11 (2H, d, *J* = 7.9 Hz), 4.17 (1H, t, *J* = 7.7 Hz), 3.21 (2H, d, *J* = 7.7 Hz), 2.63 – 2.57 (2H, m), 2.31 (3H, s), 1.45 (2H, sx, *J* = 7.4 Hz), 0.85 (3H, t, *J* = 7.4 Hz); ^{13}C NMR (151 MHz, $CDCl_3$) δ 143.4, 140.1, 136.2, 129.5, 128.7, 128.1, 128.0, 126.6, 54.7, 51.9, 50.9, 23.2, 21.1, 11.9; HRMS (ESI) found MH^+ 254.1904, $C_{18}H_{24}N$ requires 254.1903.

N-(2-(4-Methoxyphenyl)-2-phenylethyl)propan-1-amine (1d/3b)

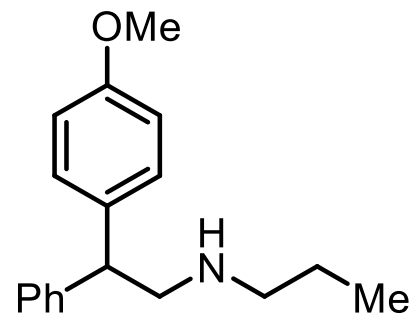

Following **GP5b**, *trans*-aziridine borane **1a** (18 mg, 0.1 mmol, 1.0 equiv.) and (4-methoxyphenyl)boronic acid (61 mg, 0.4 mmol, 4.0 equiv.) gave **1d** (50%) as an oil. R_f 0.13 [EtOAc]. ^{1}H NMR (600 MHz, $CDCl_3$) δ 7.29 (2H, t, *J* = 7.7 Hz), 7.25 – 7.22 (2H, d, *J* = 7.8 Hz), 7.22 – 7.19 (1H, t, *J* = 7.3 Hz), 7.17 (2H, d, *J* = 8.8 Hz), 6.85 (2H, d, *J* = 8.7 Hz), 4.17 (1H, t, *J* = 7.7 Hz), 3.78 (3H, s), 3.25 – 3.17 (2H, m), 2.61 (2H, t, *J* = 7.3 Hz), 1.78 (NH, br s), 1.45 (2H, sx, *J* = 7.4 Hz), 0.84 (3H, t, *J* = 7.4 Hz); ^{13}C NMR (151 MHz, $CDCl_3$) δ 158.4, 143.3, 134.9, 129.1,

128.8, 128.0, 126.6, 114.2, 55.4, 54.7, 51.8, 50.3, 23.0, 11.8; HRMS (ESI) found MH^+ 270.1854, $C_{18}H_{24}NO$ requires 270.1852.

Following **GP5a**, but running the reaction for 5 h, *trans*-aziridine **3a'** (21 mg, 0.1 mmol, 1.0 equiv.) and phenylboronic acid (37 mg, 0.3 mmol, 3.0 equiv.) gave **3b** (33%) as an oil.

***N-(2*-([1,1'-Biphenyl]-4-yl)-2-phenylethyl)propan-1-amine (1e)**

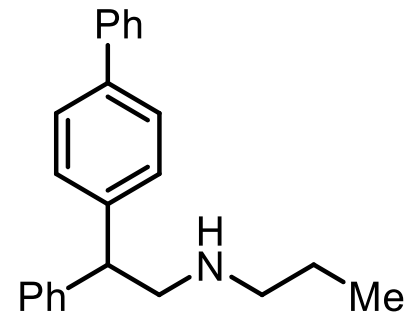

Following **GP5b**, but running the reaction for 6 h, *trans*-aziridine borane **1a** (18 mg, 0.1 mmol) and [1,1'-biphenyl]-4-ylboronic acid (79 mg, 0.4 mmol, 4.0 equiv.) gave **1e** (41%) as a yellow solid. R_f 0.28 [EtOAc]. ^{1}H NMR (600 MHz, $CDCl_3$) δ 7.57 (2H, d, *J* = 8.3 Hz), 7.54 (2H, d, *J* = 8.2 Hz), 7.42 (2H, t, *J* = 7.8 Hz), 7.36 – 7.28 (7H, m), 7.23 (1H, tt, *J* = 7.8, 1.8 Hz), 4.27 (1H, t, *J* = 7.7 Hz), 3.29 (2H, d, *J* = 7.7 Hz), 2.69 – 2.59 (2H, t, *J* = 7.4 Hz), 1.79 (NH, br s), 1.48 (2H, sx, *J* = 7.4 Hz), 0.86 (3H, t, *J* = 7.4 Hz); ^{13}C NMR (151 MHz, $CDCl_3$) δ 142.9, 142.1, 141.0, 139.6, 128.9, 128.9, 128.5, 128.2, 127.5, 127.3, 127.1, 126.8, 54.6, 51.9, 50.9, 23.0, 11.8; HRMS (ESI) found MH^+ 316.2054, $C_{23}H_{26}N$ requires 316.2060.

***N*-(2-(4-Fluorophenyl)-2-phenylethyl)propan-1-amine (1f)**

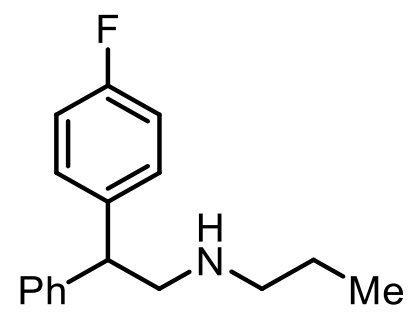

Following **GP5d**, *trans*-aziridine borane **1a** (18 mg, 0.1 mmol) and (4-fluorophenyl)boronic acid (56 mg, 0.4 mmol, 4.0 equiv.) gave **1f** (61%) as an oil. R_f 0.37 [EtOAc]. ^{1}H NMR (600 MHz, $CDCl_3$) δ 7.33 – 7.28 (2H, m), 7.25 – 7.18 (5H, m), 7.01 – 6.95 (2H, m), 4.18 (1H, t, *J* = 7.6 Hz), 3.26 – 3.15 (2H, m), 2.60 (2H, t, *J* = 7.3 Hz), 1.45 (3H, sx, *J* = 7.3 Hz), 0.85 (3H, t, *J* = 7.4 Hz); ^{13}C NMR (151 MHz, $CDCl_3$) δ 161.7 (d, *J* = 244.5 Hz), 142.9, 138.9 (d, *J* = 3.4 Hz), 129.57 (d, *J* = 7.9 Hz), 128.9, 128.1, 126.8, 115.6 (d, *J* = 21.2 Hz), 54.7, 51.9, 50.5, 23.1, 11.8; ^{19}F NMR (565 MHz, $CDCl_3$) δ -116.61 – -116.69 (m). HRMS (ESI) found MH^+ 258.1646, $C_{17}H_{21}FN$ requires 258.1653.

N-(2-(4-Chlorophenyl)-2-phenylethyl)propan-1-amine (1g/5b)

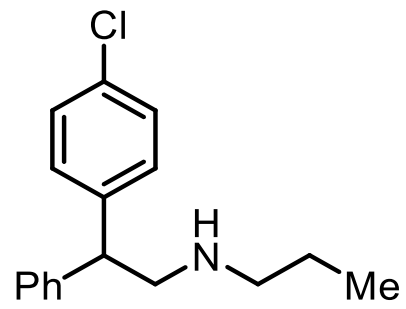

Following **GP5d**, but adding $NaHCO_3$ (17 mg, 0.2 mmol, 2 equiv.), *trans*-aziridine borane **1a** (18 mg, 0.1 mmol) and (4-chlorophenyl)boronic acid (63 mg, 0.4 mmol, 4.0 equiv.) gave **1g** (59%) as an oil. R_f 0.48 [EtOAc]. ^{1}H NMR (400 MHz, $CDCl_3$) δ 7.38 – 7.13 (9H, m), 4.19 (1H, t, *J* = 7.6 Hz), 3.33 – 3.10 (2H, m), 2.61 (2H, t, *J* = 7.1 Hz), 1.46 (2H, sx, *J* = 6.9 Hz), 0.86 (3H, t, *J* = 7.3 Hz); ^{13}C NMR (101 MHz, $CDCl_3$) δ 142.6, 141.7, 132.4, 129.5, 128.9, 128.1, 126.9, 54.5, 51.9, 50.7, 23.1, 11.8; HRMS (ESI) found MH^+ 274.1363, $C_{17}H_{21}NCl$ requires 274.1357.

Following **GP5a**, **5a** (42 mg, 0.2 mmol, 1.0 equiv.) and phenylboronic acid (73 mg, 0.6 mmol, 3.0 equiv.) gave **5b** (65%) as an oil.

N-(2-Phenyl-2-(4-(trifluoromethyl)phenyl)ethyl)propan-1-amine (1h/6b)

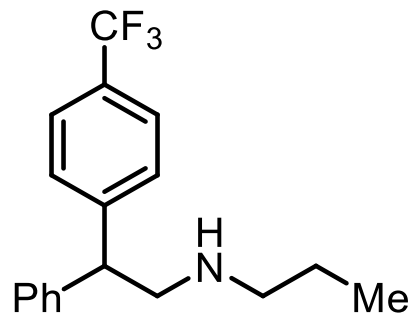

Following **GP5c**, *trans*-aziridine borane **1a** (18 mg, 0.1 mmol, 1.0 equiv.) and (4-(trifluoromethyl)phenyl)boronic acid (76 mg, 0.4 mmol, 4.0 equiv.) gave **1h** (41%) as an oil. R_f 0.16 [EtOAc]. ^{1}H NMR (600 MHz, $CDCl_3$) δ 7.55 (2H, d, *J* = 8.0 Hz), 7.38 (2H, d, *J* = 8.0 Hz), 7.32 (2H, dd, *J* = 8.4, 6.9 Hz), 7.26 – 7.21 (3H, m), 4.27 (1H, t, *J* = 7.6 Hz), 3.31 – 3.22 (2H, m), 2.62 (2H, t, *J* = 7.3 Hz), 1.49 – 1.38 (3H, m), 0.86 (3H, t, *J* = 7.4 Hz); ^{13}C NMR (151 MHz, $CDCl_3$) δ 147.3, 142.0, 128.99, 128.96 (q, $J_{C\text{-}F}$ = 32.5 Hz), 128.5, 128.1, 127.1, 125.7 (q, *J* = 3.8 Hz), 124.3 (q, *J* = 271.8 Hz), 54.3, 51.9, 51.2, 23.1, 11.8; ^{19}F NMR (565 MHz, $CDCl_3$) δ –62.4; HRMS (ESI) found MH^+ 308.1621, $C_{18}H_{21}F_3N$ requires 308.1621.

Following **GP5a**, but running the reaction for 6 h, *trans*-aziridine **6a’** (24 mg, 0.1 mmol, 1.0 equiv.) and phenylboronic acid (37 mg, 0.3 mmol, 3.0 equiv.) gave **6b** (51%) as an oil.

4-(1-Phenyl-2-(propylamino)ethyl)benzonitrile (1i)

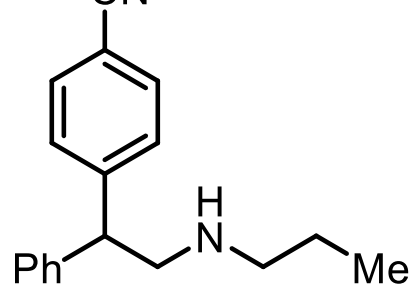

Following **GP5c**, *trans*-aziridine borane **1a** (18 mg, 0.1 mmol, 1.0 equiv.) and (4-cyanophenyl)boronic acid (60 mg, 0.4 mmol, 4.0 equiv.) gave **1i** (38%) as an oil. R_f 0.25 [EtOAc]. ^{1}H NMR (600 MHz, $CDCl_3$) δ 7.60 – 7.55 (2H, m), 7.39 – 7.35 (2H, m), 7.34 – 7.30 (2H, m),

7.26 – 7.20 (3H, m), 4.24 (1H, t, *J* = 7.5 Hz), 3.29 – 3.17 (2H, m), 2.64 – 2.57 (2H, m), 1.53 (NH, br s), 1.45 (2H, sx, *J* = 7.4 Hz), 0.85 (3H, t, *J* = 7.4 Hz); ^{13}C NMR (151 MHz, $CDCl_3$) δ 148.9, 141.5, 132.5, 129.1, 129.0, 128.1, 127.2, 119.0, 110.5, 54.1, 51.9, 51.5, 23.1, 11.8; HRMS (ESI) found MH^+ 265.1692, $C_{18}H_{21}N_2$ requires 265.1699.

_N_-(2-Phenyl-2-(4-(trifluoromethoxy)phenyl)ethyl)propan-1-amine (1j)

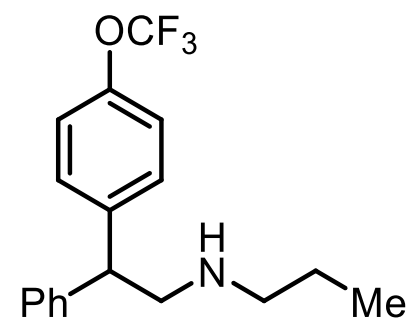

Following **GP5c**, *trans*-aziridine borane **1a** (18 mg, 0.1 mmol, 1.0 equiv.) and (4-(trifluoromethoxy)phenyl)boronic acid (82 mg, 0.4 mmol, 4.0 equiv.) gave **1j** (52%) as an oil. R_f 0.35 [EtOAc]. ^{1}H NMR (600 MHz, $CDCl_3$) δ 7.27 – 7.21 (2H, m), 7.21 – 7.17 (2H, m), 7.17 – 7.13 (3H, m), 7.08 – 7.04 (2H, m), 4.14 (1H, t, *J* = 7.6 Hz), 3.21 – 3.11 (2H, m), 2.57 – 2.52 (2H, m), 1.61 (NH, br s), 1.38 (2H, sx, *J* = 7.4 Hz), 0.76 (3H, t, *J* = 7.4 Hz); ^{13}C NMR (151 MHz, $CDCl_3$) δ 148.0, 142.2, 141.8, 129.4, 129.0, 128.1, 127.0, 121.2, 120.6 (q, *J* = 256.9 Hz), 54.4, 51.8, 50.5, 22.9, 11.7; ^{19}F (565 MHz, $CDCl_3$) δ –57.88; HRMS (EI) found MH^+ 324.1571, $C_{18}H_{21}F_3NO$ requires 324.1570.

_N_-(2-Phenyl-2-(_m_-tolyl)ethyl)propan-1-amine (1k)

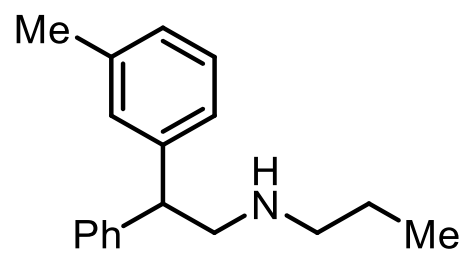

Following **GP5a**, *trans*-aziridine borane **1a** (18 mg, 0.1 mmol, 1.0 equiv.) and *m*-tolylboronic acid (41 mg, 0.3 mmol, 3.0 equiv.) gave **1k** (48%) as an oil. R_f 0.15 [EtOAc]. ^{1}H NMR (600 MHz, $CDCl_3$) δ 7.34 – 7.30 (2H, m), 7.30 – 7.27 (2H, m), 7.24 – 7.19 (2H, m), 7.11 – 7.07 (2H, m), 7.04 (1H, d, *J* = 7.5 Hz), 4.20 (1H, t, *J* = 7.7 Hz), 3.25 (2H, d, *J* = 7.7 Hz), 2.63 (2H, t, *J* = 7.3 Hz), 2.34 (3H, s), 1.53 – 1.43 (3H, m), 0.87 (3H, t, *J* = 7.4 Hz); ^{13}C NMR (151 MHz, $CDCl_3$) δ 143.2, 143.0, 138.3, 129.0, 128.7, 128.6, 128.1, 127.5, 126.6, 125.0, 54.6, 51.9, 51.2, 23.1, 21.6, 11.8; HRMS (EI) found MH^+ 254.1905, $C_{18}H_{24}N$ requires 254.1903.

_N_-(2-(3-methoxyphenyl)-2-phenylethyl)propan-1-amine (1l)

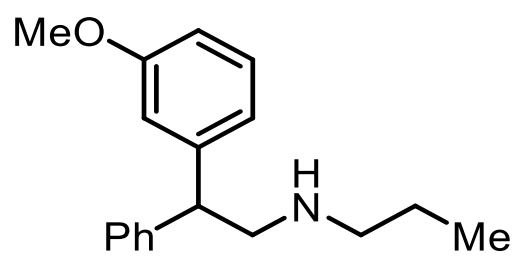

Following **GP5b**, *trans*-aziridine borane **1a** (18 mg, 0.1 mmol, 1.0 equiv.) and (3-methoxyphenyl)boronic acid (61 mg, 0.4 mmol, 4.0 equiv.) gave **1l** (51%) as an oil. R_f 0.26

[EtOAc]. ^{1}H NMR (400 MHz, $CDCl_3$) δ 7.32 – 7.16 (6H, m), 6.84 (1H, d, *J* = 7.7 Hz), 6.79 (1H, t, *J* = 2.2 Hz), 6.73 (1H, dd, *J* = 8.0, 2.6 Hz), 4.17 (1H, t, *J* = 7.7 Hz), 3.76 (3H, s), 3.21 (2H, d, *J* = 7.7 Hz), 2.59 (2H, t, *J* = 7.3 Hz), 1.58 (NH, br s), 1.44 (2H, sx, *J* = 7.3 Hz), 0.83 (3H, t, *J* = 7.4 Hz); ^{13}C NMR (101 MHz, $CDCl_3$) δ 159.9, 144.6, 142.9, 129.7, 128.8, 128.1, 126.7, 120.5, 114.3, 111.7, 55.3, 54.6, 51.9, 51.3, 23.1, 11.8. HRMS (ESI) found MH^+ 270.1849, $C_{18}H_{24}NO$ requires 270.1852.

***N*-(2-(3-fluoro-4-methoxyphenyl)-2-phenylethyl)propan-1-amine (1m)**

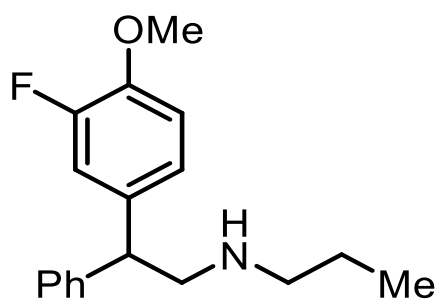

Following **GP5b**, *trans*-aziridine borane **1a** (18 mg, 0.1 mmol, 1.0 equiv.) and (3-fluoro-4-methoxyphenyl)boronic acid (68 mg, 0.4 mmol, 4.0 equiv.) gave **1m** (40%) as an oil. R_f 0.35 [EtOAc]. ^{1}H NMR (600 MHz, $CDCl_3$) δ 7.31 (2H, t, *J* = 7.5 Hz), 7.25 – 7.19 (3H, m), 7.01 – 6.94 (2H, m), 6.92 – 6.86 (1H, m), 4.13 (1H, t, *J* = 7.6 Hz), 3.85 (3H, s), 3.22 – 3.14 (2H, m), 2.60 (2H, t, *J* = 7.3 Hz), 1.45 (2H, sx, *J* = 7.3 Hz), 1.37 (NH, br s), 0.85 (3H, t, *J* = 7.4 Hz); ^{13}C NMR (101 MHz, $CDCl_3$) δ 152.6 (d, *J* = 245.8 Hz), 146.3 (d, *J* = 10.9 Hz), 142.8, 136.4 (d, *J* = 5.8 Hz), 128.9, 128.0, 126.8, 123.7 (d, *J* = 3.4 Hz), 115.8 (d, *J* = 18.4 Hz), 113.7 (d, *J* = 2.3 Hz), 56.5, 54.6, 51.9, 50.4, 23.1, 11.8; ^{19}F NMR ($CDCl_3$, 565 MHz) δ –134.80 (dd, *J* = 12.5, 8.7 Hz). HRMS (ESI) found MH^+ 288.1753, $C_{18}H_{23}FNO$ requires 288.1758.

***N*-(2-(Naphthalen-2-yl)-2-phenylethyl)propan-1-amine (1n)**

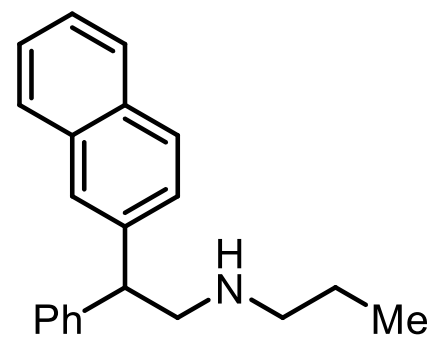

Following **GP5b**, but adding $NaHCO_3$ (17 mg, 0.2 mmol, 2.0 equiv.), *trans*-aziridine borane **1a** (18 mg, 0.1 mmol, 1.0 equiv.) and naphthalen-2-ylboronic acid (69 mg, 0.4 mmol, 4.0 equiv.) gave **1n** (34%) as a solid. R_f 0.13 [EtOAc]. ^{1}H NMR (600 MHz, $CDCl_3$) δ 7.83 – 7.75 (3H, m), 7.73 (1H, d, *J* = 1.8 Hz), 7.48 – 7.41 (2H, m), 7.37 (1H, dd, *J* = 8.5, 1.8 Hz), 7.32 – 7.29 (4H, m), 7.24 – 7.20 (1H, m), 4.39 (1H, t, *J* = 7.6 Hz), 3.43 – 3.31 (2H, m), 2.66 (2H, t, *J* = 7.3 Hz), 1.84 (NH, br s), 1.47 (2H, sx, *J* = 7.4 Hz), 0.85 (3H, t, *J* = 7.4 Hz); ^{13}C NMR (151 MHz, $CDCl_3$) δ 142.8, 140.3, 133.7, 132.5, 128.8, 128.5, 128.3, 127.9, 127.7, 126.8, 126.8, 126.4, 126.2, 125.8, 54.4, 51.9, 51.2, 23.0, 11.8; HRMS (ESI) found MH^+ 290.1913, $C_{21}H_{24}N$ requires 290.1903.

N-(2-(Benzofuran-5-yl)-2-phenylethyl)propan-1-amine (1o)

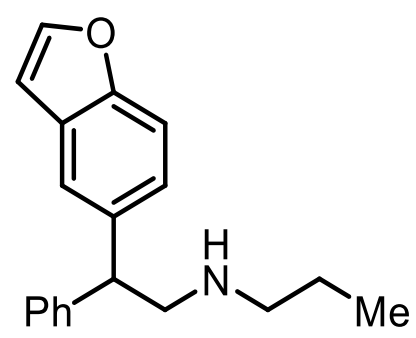

Following **GP5b**, *trans*-aziridine borane **1a** (18 mg, 0.1 mmol, 1.0 equiv.) and benzofuran-5-ylboronic acid (49 mg, 0.3 mmol, 3.0 equiv.) gave **1o** (38%) as an oil. R_f 0.25 [EtOAc]. ^{1}H NMR (600 MHz, $CDCl_3$) δ 7.59 (1H, d, *J* = 2.2 Hz), 7.49 (1H, d, *J* = 1.8 Hz), 7.43 (1H, d, *J* = 8.5 Hz), 7.32 – 7.27 (4H, m), 7.23 – 7.17 (2H, m), 6.72 (1H, d, *J* = 2.2 Hz), 4.32 (1H, t, *J* = 7.7 Hz), 3.29 (2H, d, *J* = 7.7 Hz), 2.63 (2H, t, *J* = 7.3 Hz), 1.46 (2H, sx, *J* = 7.3 Hz), 0.84 (3H, t, *J* = 7.4 Hz); ^{13}C NMR (151 MHz, $CDCl_3$) δ 154.0, 145.5, 143.4, 137.6, 128.8, 128.1, 127.9, 126.6, 124.7, 120.4, 111.6, 106.7, 54.9, 51.9, 51.0, 23.0, 11.8; HRMS (ESI) found MH^+ 280.1697, $C_{19}H_{22}NO$ requires 280.1696.

N-(2-(6-Methoxypyridin-3-yl)-2-phenylethyl)propan-1-amine (1p)

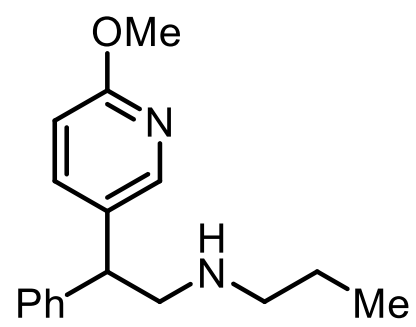

Following **GP5c**, but running the reaction for 6 h, *trans*-aziridine borane **1a** (18 mg, 0.1 mmol, 1.0 equiv.) and (6-methoxypyridin-3-yl)boronic acid (61 mg, 0.4 mmol, 4.0 equiv.) gave **1p** (38%) as an oil. R_f 0.2 [EtOAc]. ^{1}H NMR (600 MHz, $CDCl_3$) δ 8.09 (1H, d, *J* = 2.5 Hz), 7.43 (1H, dd, *J* = 8.6, 2.5 Hz), 7.31 (2H, t, *J* = 7.6 Hz), 7.25 – 7.19 (3H, m), 6.68 (1H, d, *J* = 8.6 Hz), 4.14 (1H, t, *J* = 7.6 Hz), 3.91 (3H, s), 3.25 – 3.14 (2H, m), 2.60 (2H, t, *J* = 7.3 Hz), 1.48 – 1.40 (2H, m), 0.85 (3H, t, *J* = 7.4 Hz); ^{13}C NMR (151 MHz, $CDCl_3$) δ 163.2, 146.1, 142.5, 138.5, 131.3, 128.9, 128.0, 126.9, 111.0, 54.4, 53.5, 51.9, 48.2, 23.2, 11.8. HRMS (ESI) found MH^+ 271.1808, $C_{17}H_{23}N_2O$ requires 271.1805.

N-(2-(4-(*tert*-Butyl)phenyl)-2-phenylethyl)propan-1-amine (2b)

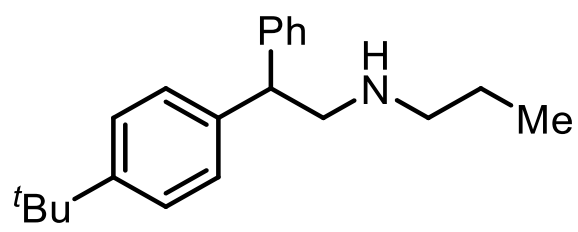

Following **GP5a**, *trans*-aziridine borane **2a’** (23 mg, 0.1 mmol, 1.0 equiv.) and phenylboronic acid (37 mg, 0.3 mmol, 3.0 equiv.) gave **2b** (54%) as an oil. R_f 0.13 [EtOAc]. ^{1}H NMR (600 MHz, $CDCl_3$) δ 7.35 – 7.27 (6H, m), 7.24 – 7.18 (3H, m), 4.20 (1H, t, *J* = 7.7 Hz), 3.24 (2H, d, *J* = 7.7 Hz), 2.65 – 2.60 (2H, t, *J* = 7.3 Hz), 1.47 (2H, sx, *J* = 7.4 Hz), 1.31 (9H, s), 0.87 (3H, t, *J* = 7.4

Hz); ^{13}C NMR (151 MHz, $CDCl_3$) δ 149.3, 143.4, 140.1, 128.7, 128.2, 127.7, 126.6, 125.6, 54.8, 52.0, 50.9, 34.5, 31.5, 23.2, 11.9; HRMS (ESI) found MH^+ 296.2382, $C_{21}H_{30}N$ requires 296.2373.

_N_-(2-(4-Bromophenyl)-2-phenylethyl)propan-1-amine (4b)

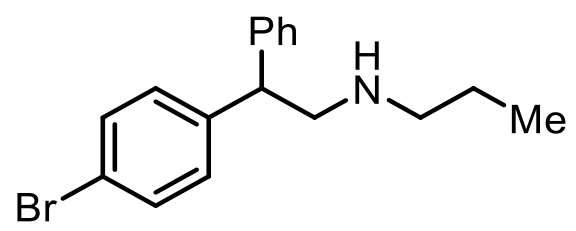

Following **GP5a**, *trans*-aziridine borane **4a** (51 mg, 0.2 mmol, 1.0 equiv.) and phenylboronic acid (73 mg, 0.6 mmol, 3.0 equiv.) gave **4b** (56%) as an oil. R_f 0.48 [EtOAc]. ^{1}H NMR (400 MHz, $CDCl_3$) δ 7.47 – 7.38 (2H, m), 7.35 – 7.27 (2H, m), 7.27 – 7.18 (3H, m), 7.18 – 7.09 (2H, m), 4.17 (1H, t, J = 7.6 Hz), 3.28 – 3.14 (2H, m), 2.61 (2H, t, J = 7.3 Hz), 1.45 (2H, sx, J = 7.4 Hz), 0.85 (3H, t, J = 7.4 Hz); ^{13}C NMR (101 MHz, $CDCl_3$) δ 142.5, 142.2, 131.8, 129.9, 128.9, 128.0, 126.9, 120.5, 54.4, 51.9, 50.7, 23.1, 11.8; HRMS (ESI) found MH^+ 318.0860, $C_{17}H_{21}NBr$ requires 318.0852.

_N_-(2-(Bicyclo[4.2.0]octa-1(6),2,4-trien-3-yl)-2-phenylethyl)propan-1-amine (7b)

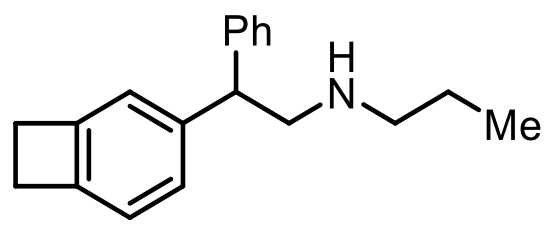

Following **GP5a**, but running the reaction for 5 h, *trans*-aziridine borane **7a** (20 mg, 0.1 mmol) and phenylboronic acid (37 mg, 0.3 mmol, 3.0 equiv.) gave **7b** (50%) as an oil. R_f 0.30 [EtOAc]. ^{1}H NMR (400 MHz, $CDCl_3$) δ 7.36 – 7.24 (4H, m), 7.24 – 7.15 (1H, m), 7.11 (1H, dd, J = 7.5, 1.6 Hz), 7.02 – 6.94 (2H, m), 4.19 (1H, t, J = 7.7 Hz), 3.23 (2H, dd, J = 7.7, 1.6 Hz), 3.13 (4H, s), 2.62 (2H, t, J = 7.3 Hz), 1.47 (2H, sx, J = 7.4 Hz), 0.86 (3H, t, J = 7.4 Hz); ^{13}C NMR (151 MHz, $CDCl_3$) δ 146.2, 144.1, 143.6, 141.8, 128.7, 128.1, 126.8, 126.5, 122.8, 122.3, 54.8, 51.9, 51.8, 29.5, 29.4, 23.1, 11.9; HRMS (ESI) found MH^+ 266.1894, $C_{19}H_{24}N$ requires 266.1903.

_N_-Methyl-2,2-diphenylethan-1-amine (**8b**)

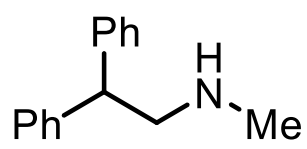

Following **GP5a**, but running the reaction for 5 h, *trans*-aziridine borane **8a** (15 mg, 0.1 mmol, 1.0 equiv.) and phenylboronic acid (37 mg, 0.3 mmol, 3.0 equiv.) gave **8b** (56%) as an oil. R_f 0.27 [CH_2Cl_2:MeOH (10:1)]. ^{1}H NMR (600 MHz, $CDCl_3$) δ 7.33 – 7.27 (8H, m), 7.24 – 7.19 (2H, m), 4.50 (1H, t, J=7.8 Hz), 3.42 (2H, d, J=7.8 Hz), 2.43 (3H, s); ^{13}C NMR (151 MHz, $CDCl_3$) δ 141.1, 129.1, 128.1, 127.4, 54.6, 49.1, 34.8. Data in accordance with the literature.[18]

N-(2,2-Diphenylethyl)hexan-1-amine (9b)

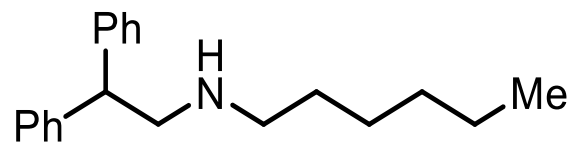

Following **GP5a**, but running the reaction for 6 h, *trans*-aziridine borane **9a'** (22 mg, 0.1 mmol, 1.0 equiv.) and phenylboronic acid (37 mg, 0.3 mmol, 3.0 equiv.) gave **9b** (51%) as an oil. R_f 0.35 [EtOAc]. ^{1}H NMR (400 MHz, $CDCl_3$) δ 7.37 – 7.19 (10H, m), 4.23 (1H, t, *J* = 7.7 Hz), 3.26 (2H, d, *J* = 7.7 Hz), 2.65 (2H, t, *J* = 7.3 Hz), 1.62 (NH, br s), 1.48 – 1.39 (2H, m), 1.31 – 1.20 (6H, m), 0.88 (3H, t, *J* = 6.8 Hz); ^{13}C NMR (101 MHz, $CDCl_3$) δ 143.1, 128.8, 128.2, 126.7, 54.7, 51.3, 50.0, 31.8, 30.0, 27.1, 22.7, 14.2; HRMS (ESI) found MH^+ 282.2216, $C_{20}H_{28}N$ requires 282.2216.

N-Benzyl-2,2-diphenylethan-1-amine (10b)

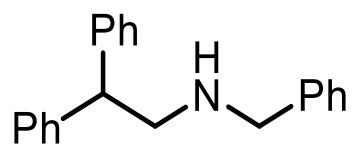

Following **GP5a**, but running the reaction for 5 h, *trans*-aziridine borane **10a'** (22 mg, 0.1 mmol, 1.0 equiv.) and phenylboronic acid (37 mg, 0.3 mmol, 3.0 equiv.) gave **10b** (47%) as an oil. R_f 0.56 [EtOAc]. ^{1}H NMR (400 MHz, $CDCl_3$) δ 7.35 – 7.20 (15H, m), 4.25 (1H, t, *J* = 7.7 Hz), 3.85 (2H, s), 3.27 (2H, d, *J* = 7.6 Hz), 1.47 (NH, br s); ^{13}C NMR (101 MHz, $CDCl_3$) δ 143.0, 140.3, 128.7, 128.5, 128.2, 128.2, 127.1, 126.7, 54.0, 53.9, 51.4; HRMS (ESI) found MH^+ 288.1748, $C_{21}H_{22}N$ requires 288.1747. Data in accordance with the literature.[19]

N-(2,2-Diphenylethyl)propan-2-amine (11b)

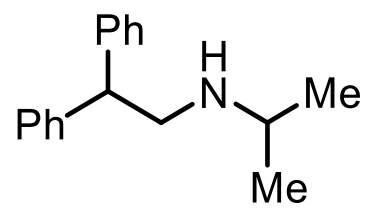

Following **GP5a**, but running the reaction for 3 h, *cis*-aziridine borane **11a** (18 mg, 0.1 mmol, 1.0 equiv.) and phenylboronic acid (37 mg, 0.3 mmol, 3.0 equiv.) gave **11b** (57%) as an oil. R_f 0.28 [EtOAc]. ^{1}H NMR (600 MHz, $CDCl_3$) δ 7.22 (4H, t, *J* = 7.5 Hz), 7.18 (4H, d, *J* = 7.2 Hz), 7.13 (2H, t, *J* = 7.1), 4.11 (1H, t, *J* = 7.7 Hz), 3.16 (2H, dd, *J* = 7.7), 2.77 (1H, hp, *J* = 6.3), 0.95 (6H, d, *J* = 6.2); ^{13}C NMR (151 MHz, $CDCl_3$) δ 143.0, 128.8, 128.1, 126.7, 52.2, 51.4, 48.8, 22.9; HRMS (ESI) found MH^+ 240.1748, $C_{17}H_{22}N$ requires 240.1747.

N-(2,2-Diphenylethyl)cyclohexanamine (12b)

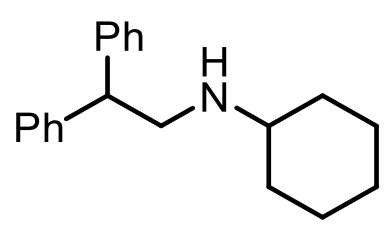

Following **GP5a**, *trans*-aziridine borane **12a'** (22 mg, 0.1 mmol, 1.0 equiv.) and phenylboronic acid (37 mg, 0.3 mmol, 3.0 equiv.) gave **12b** (50%) as a solid. R_f 0.58 [EtOAc]. ^{1}H NMR (600

MHz, $CDCl_3$) δ 7.30 (4H, t, *J* = 7.5 Hz), 7.28 – 7.25 (4H, m), 7.23 – 7.17 (2H, m), 4.18 (1H, t, *J* = 7.7 Hz), 3.26 (2H, d, *J* = 7.7 Hz), 2.45 (1H, tt, *J* = 10.5, 3.8 Hz), 1.83 (2H, dd, *J* = 12.5, 3.8 Hz), 1.69 (2H, dp, *J* = 10.9, 3.7 Hz), 1.63 – 1.56 (1H, m), 1.29 – 1.18 (3H, m), 1.14 (1H, tt, *J* = 12.4, 3.5 Hz), 1.03 (2H, tdd, *J* = 12.7, 10.7, 3.5 Hz); ^{13}C NMR (151 MHz, $CDCl_3$) δ 143.2, 128.7, 128.2, 126.6, 57.0, 51.8, 51.5, 33.6, 26.3, 25.2; HRMS (ESI) found MH^+ 280.2059, $C_{20}H_{26}N$ requires 280.2060.

***N*-Isopropyl-2,4-diphenylbutan-1-amine (13b)**

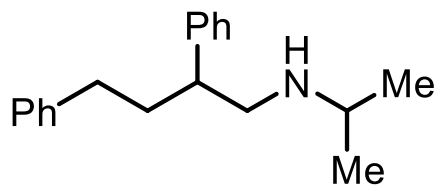

Following **GP5a,** but using $Cu(CH_3CN)_4PF_6$ (5 mol%) and bathophen (6 mol%) as the ligand, CH_3CN:PhF = 7:3 (0.1 M) as the solvent mixture and react at room temperature for 1.5 h, *trans*-aziridine borane **13a** (20 mg, 0.1 mmol, 1.0 equiv.) gave **13b** (37%) as an oil. R_f 0.19 [EtOAc]. ^1H NMR (600 MHz, $CDCl_3$) δ 7.30 –7.24 (2H, m), 7.20 – 7.12 (5H, m), 7.11 – 7.05 (1H, m), 7.05 – 7.01 (2H, m), 2.81 (1H, dd, *J* = 10.6, 4.7 Hz), 2.76 – 2.66 (2H, m), 2.62 (1H, hp, *J* = 6.2 Hz), 2.46 – 2.34 (2H, m), 1.93 (1H, dddd, *J* = 13.5, 9.7, 7.0, 4.7 Hz), 1.82 (1H, dtd, *J* = 13.5, 9.4, 5.7 Hz), 0.91 (3H, d, *J* = 6.3 Hz), 0.87 (3H, d, *J* = 6.3 Hz); ^{13}C NMR (151 MHz, $CDCl_3$) δ 143.7, 142.5, 128.8, 128.5, 128.4, 128.0, 126.7, 125.8, 53.6, 48.7, 45.9, 36.6, 33.8, 23.1, 22.9; HRMS (ESI) found MH^+ 268.2055, $C_{19}H_{26}N$ requires 268.2060.

Following **GP8a**, *trans*-aziridine **13a** (20 mg, 0.1 mmol, 1.0 equiv.) and bromobenzene (31 mg, 0.2 mmol, 2.0 equiv.) gave **13b** (54%) as an oil.

5.2 Ring-Opening–Cyanation

2-Phenyl-3-(propylamino)propanenitrile (1q)

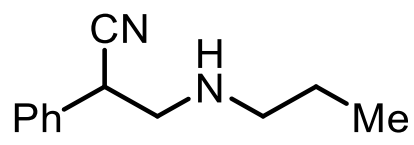

Following **GP6**, *trans*-aziridine borane **1a** (18 mg, 0.1 mmol, 1.0 equiv.) gave **1q** (65%) as a yellow oil. R_f 0.56 [*n*-pentane:EtOAc (1:1)]. ^{1}H NMR (600 MHz, $CDCl_3$) δ 7.39 (2H, d, *J* = 7.4 Hz), 7.36 – 7.31 (3H, m), 3.98 (1H, dd, *J* = 8.1, 6.3 Hz), 3.12 (1H, dd, *J* = 12.4, 8.1 Hz), 3.01 (1H, dd, *J* = 12.4, 6.3 Hz), 2.67 – 2.57 (2H, m), 1.48 (2H, sx, *J* = 7.3 Hz), 1.20 (NH, br s), 0.90 (3H, t, *J* = 7.4 Hz). ^{13}C NMR (151 MHz, $CDCl_3$) δ 134.0, 129.3, 128.4, 127.6, 120.3, 54.3, 51.1, 38.6, 23.2, 11.7; HRMS (ESI) found MH^+ 189.1387, $C_{12}H_{17}N_2$ requires 189.1386.

The structure of **1q** was further confirmed by 2D NMR spectroscopy (COSY, HSQC, and HMBC), which also established the regioselectivity of the reaction.

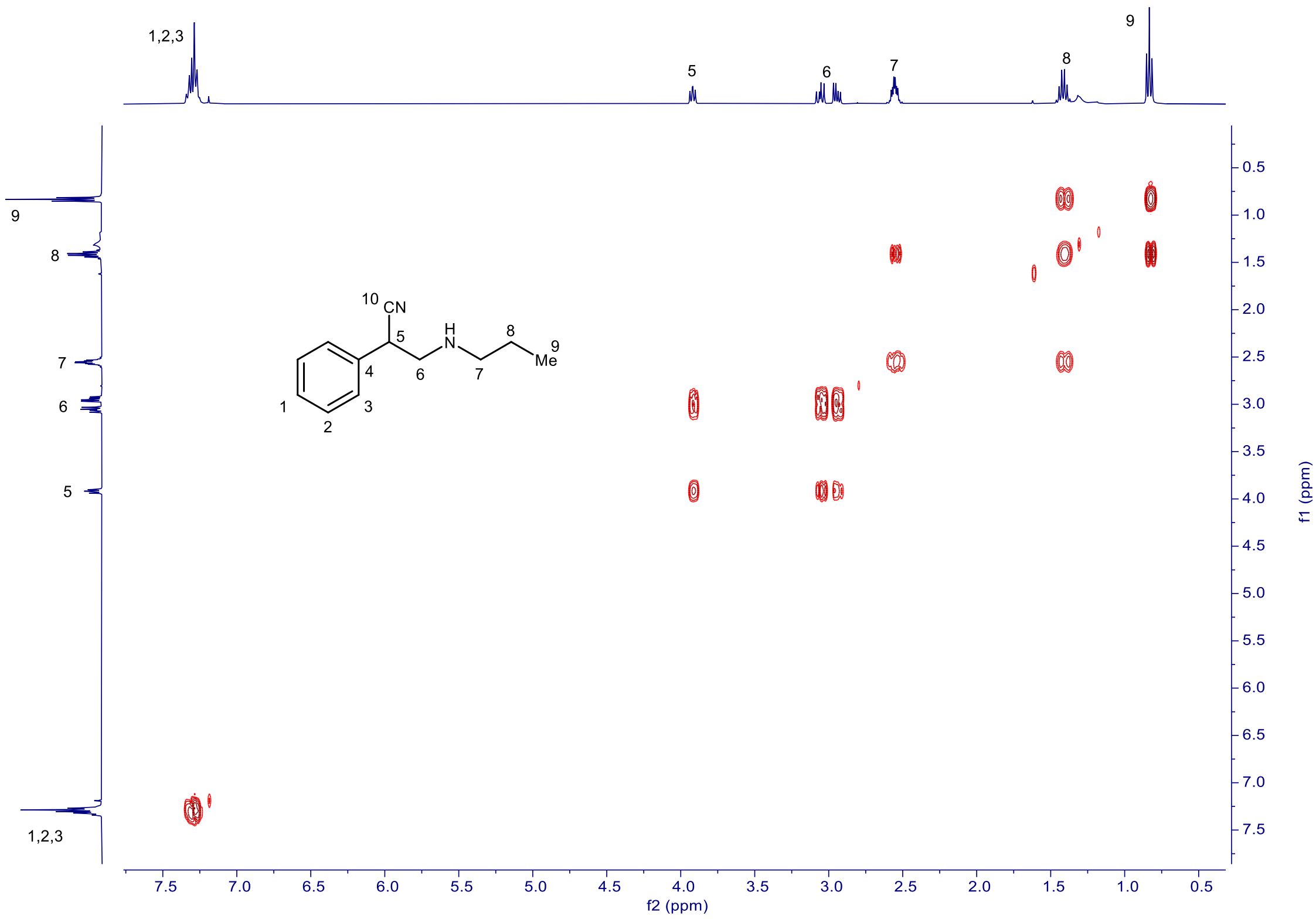

COSY NMR spectrum (600 MHz, $CDCl_3$, at rt) of **1q**

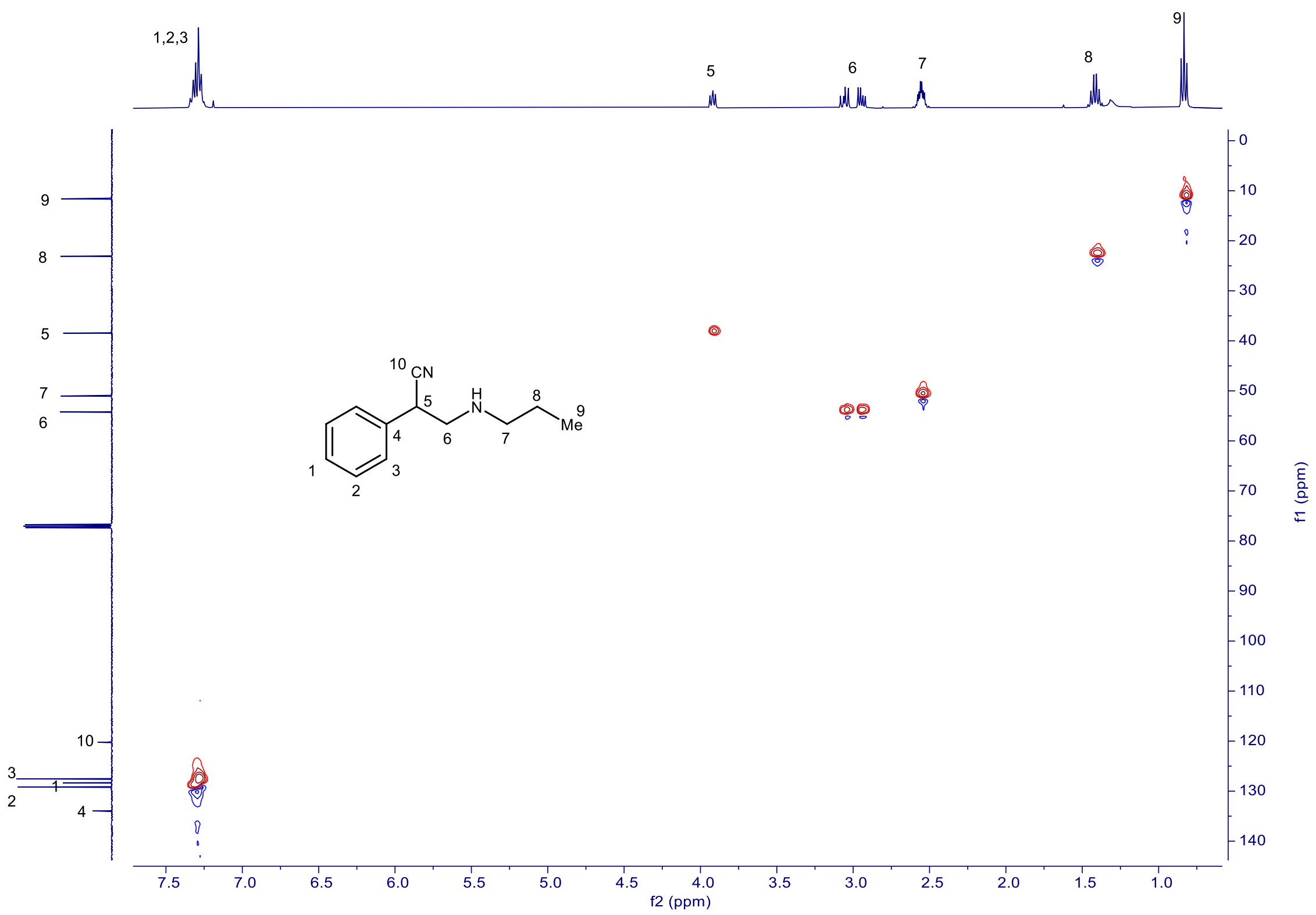

HSQC NMR spectrum (600 MHz, $CDCl_3$, at rt) of **1q**

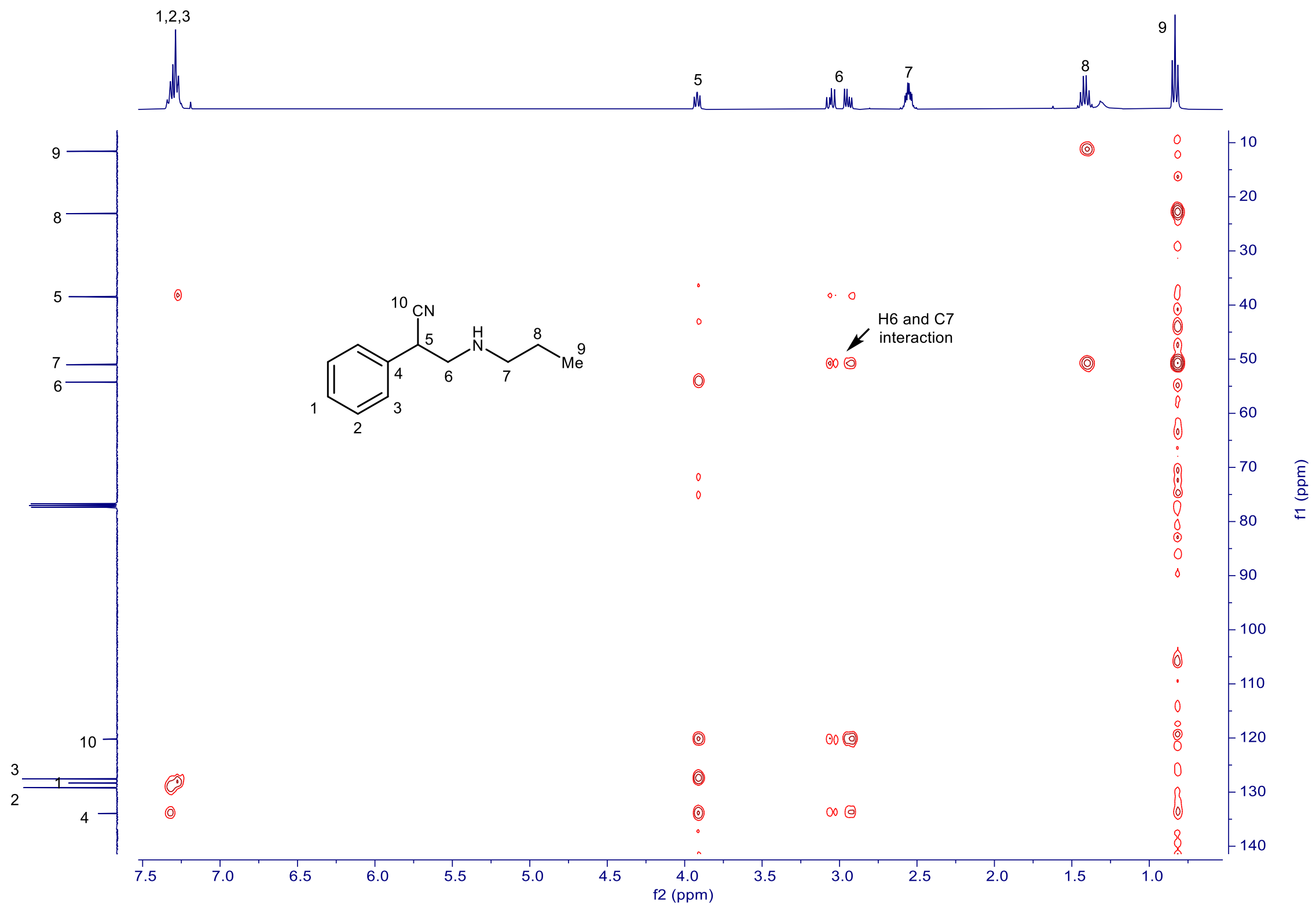

HMBC NMR spectrum (600 MHz, $CDCl_3$, at rt) of **1q**

3-(Propylamino)-2-(*p*-tolyl)propanenitrile (14b)

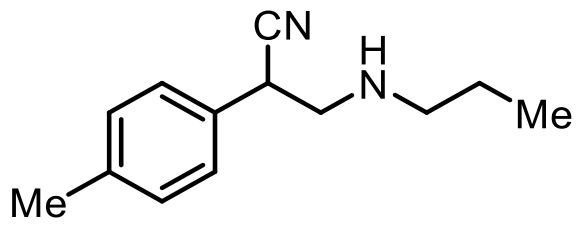

Following **GP6**, *trans*-aziridine borane **14a** (19 mg, 0.1 mmol, 1.0 equiv.) gave **14b** (64%) as a yellow oil. R_f 0.23 [*n*-pentane:EtOAc (8:2)]. ^{1}H NMR (600 MHz, $CDCl_3$) δ 7.23 (2H, d, *J* = 8.1 Hz), 7.19 (2H, d, *J* = 8.1 Hz), 3.95 (1H, dd, *J* = 8.1, 6.4 Hz), 3.15 – 3.06 (1H, m), 3.04 – 2.95 (1H, m), 2.68 – 2.56 (2H, m), 2.35 (3H, s), 1.48 (2H, sx, *J* = 7.4 Hz), 0.90 (3H, t, *J* = 7.4 Hz); ^{13}C NMR (151 MHz, $CDCl_3$) δ 138.3, 131.0, 130.0, 127.5, 120.5, 54.4, 51.1, 38.1, 23.2, 21.2, 11.8; HRMS (ESI) found MH^+ 203.1548, $C_{13}H_{19}N_2$ requires 203.1543.

2-(4-Methoxyphenyl)-3-(propylamino)propanenitrile (3c)

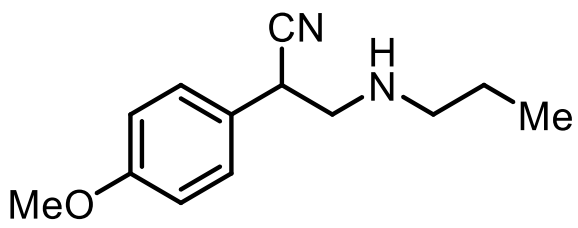

Following **GP6**, *trans*-aziridine borane **3a'** (21 mg, 0.1 mmol, 1.0 equiv.) gave **3c** (55%) as an oil. R_f 0.31 [*n*-pentane:EtOAc (1:1)]. ^{1}H (400 MHz, $CDCl_3$) δ 7.26 (2H, d, *J* = 8.4 Hz), 6.91 (2H, d, *J* = 8.7 Hz), 3.93 (1H, dd, *J* = 8.0, 6.5 Hz), 3.81 (3H, s), 3.09 (1H, dd, *J* = 12.3, 8.0 Hz), 2.97 (1H, dd, *J* = 12.3, 6.5 Hz), 2.69 – 2.54 (2H, m), 1.47 (2H, sx, *J* = 7.3 Hz), 1.38 (NH, br s), 0.90 (3H, t, *J* = 7.4 Hz); ^{13}C NMR (101 MHz, $CDCl_3$) δ 159.7, 128.8, 126.0, 120.6, 114.7, 55.5, 54.4, 51.2, 37.7, 23.3, 11.8; HRMS (ESI): found MH^+ 219.1490, $C_{13}H_{19}N_2O$ requires 219.1492.

2-(4-Fluorophenyl)-3-(propylamino)propanenitrile (15b)

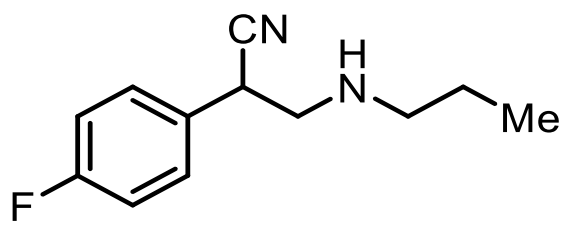

Following **GP6**, *cis*-aziridine borane **15a** and *trans*-aziridine borane **15a'** (Ratio 13:87, 19 mg, 0.1 mmol, 1.0 equiv.) gave **15b** (62%) as a yellow oil. R_f 0.43 [*n*-pentane:EtOAc (6:4)]. ^{1}H NMR (600 MHz, $CDCl_3$) δ 7.33 (2H, dd, *J* = 8.7, 5.1 Hz), 7.08 (2H, t, *J* = 8.6 Hz), 3.96 (1H, dd, *J* = 8.0, 6.3 Hz), 3.15 – 3.05 (1H, m), 3.04 – 2.96 (1H, m), 2.67 – 2.56 (2H, m), 1.48 (2H, sx, *J* = 7.3 Hz), 0.90 (3H, t, *J* = 7.3 Hz); ^{13}C NMR (151 MHz, $CDCl_3$) δ 162.7 (d, *J* = 247.3 Hz), 129.9 (d, *J* = 3.7 Hz), 129.4 (d, *J* = 8.4 Hz), 120.2, 116.3 (d, *J* = 21.5 Hz), 54.3, 51.2, 37.9, 23.2, 11.7; ^{19}F NMR (564 MHz, $CDCl_3$) δ –113.44 (tt, *J* = 8.2, 5.0 Hz); HRMS (ESI) found MH^+ 207.1300, $C_{12}H_{16}N_2F$ requires 207.1292.

2-(4-Chlorophenyl)-3-(propylamino)propanenitrile (5c)

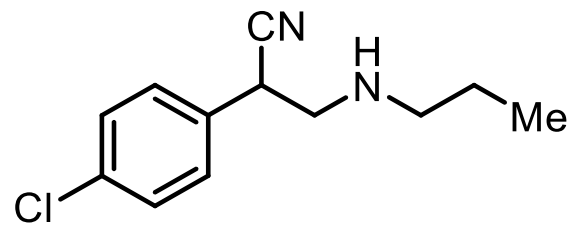

Following **GP6**, but running the reaction for 3 h, *trans*-aziridine borane **5a** (21 mg, 0.1 mmol, 1.0 equiv.) gave **5c** (47%, 9% rsm) as a yellow oil. R_f 0.23 [*n*-pentane:EtOAc (8:2)]. ^{1}H NMR (600 MHz, $CDCl_3$) δ 7.37 (2H, d, *J* = 8.5 Hz), 7.29 (2H, d, *J* = 8.5 Hz), 3.96 (1H, dd, *J* = 8.0, 6.3 Hz), 3.10 (1H, dd, *J* = 12.4, 8.0 Hz), 2.99 (1H, dd, *J* = 12.5, 6.3 Hz), 2.61 (2H, qt, *J* = 11.5, 7.1 Hz), 1.48 (2H, sx, *J* = 7.3 Hz), 0.90 (3H, t, *J* = 7.4 Hz); ^{13}C NMR (151 MHz, $CDCl_3$) δ 134.4, 132.5, 129.3, 128.9, 119.8, 54.1, 51.1, 38.0, 23.1, 11.6; HRMS (ESI) found MH^+ 223.0999, $C_{12}H_{16}N_2Cl$ requires 223.0997.

2-(2-Chlorophenyl)-3-(propylamino)propanenitrile (16b)

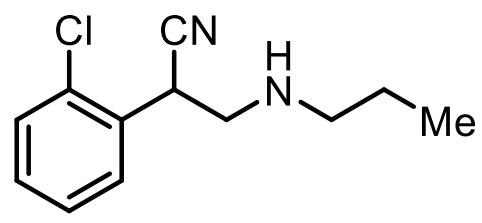

Following **GP6**, *trans*-aziridine borane **16a** (21 mg, 0.1 mmol, 1.0 equiv.) gave **16b** (51%) as a yellow oil. R_f 0.37 [*n*-pentane:EtOAc (8:2)]. ^{1}H NMR (400 MHz, $CDCl_3$) δ 7.59 (1H, dd, *J* = 7.5, 1.9 Hz), 7.41 (1H, dd, *J* = 7.6, 1.7 Hz), 7.37 – 7.28 (2H, m), 4.51 (1H, dd, *J* = 8.2, 5.6 Hz), 3.11 – 3.00 (2H, m), 2.72 (1H, dt, *J* = 11.4, 7.2 Hz), 2.62 (1H, dt, *J* = 11.4, 7.1 Hz), 1.51 (2H, sx, *J* = 7.3 Hz), 0.92 (3H, t, *J* = 7.4 Hz); ^{13}C NMR (101 MHz, $CDCl_3$) δ 132.8, 131.8, 130.0, 129.7, 129.3, 127.7, 119.9, 52.4, 50.8, 36.0, 23.2, 11.6; HRMS (ESI) found MH^+ 223.1000, $C_{12}H_{16}N_2Cl$ requires 223.0997.

2-(Bicyclo[4.2.0]octa-1(6),2,4-trien-3-yl)-3-(propylamino)propanenitrile (7c)

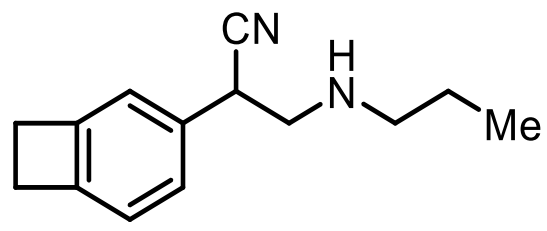

Following **GP6**, but running the reaction for 1 h, *trans*-aziridine borane **7a** (20 mg, 0.1 mmol, 1.0 equiv.) gave **7c** (56%, 13% rsm) as a yellow oil. R_f 0.33 [*n*-pentane:EtOAc (6:4)]. ^{1}H NMR (600 MHz, $CDCl_3$) δ 7.17 – 7.13 (1H, m), 7.07 – 7.03 (2H, m), 3.94 (1H, dd, *J* = 8.1, 6.5 Hz), 3.17 (4H, s), 3.10 (1H, dd, *J* = 12.3, 8.1 Hz), 2.98 (1H, dd, *J* = 12.3, 6.5 Hz), 2.67 – 2.57 (2H, m), 1.49 (2H, sx, *J* = 7.3 Hz), 0.91 (3H, t, *J* = 7.4 Hz); ^{13}C NMR (151 MHz, $CDCl_3$) δ 146.9, 146.1, 132.5, 126.4, 123.3, 121.9, 120.7, 54.7, 51.1, 38.9, 29.6, 29.5, 23.2, 11.8; HRMS (ESI) found MH^+ 215.1537, $C_{14}H_{19}N_2$ requires 215.1543.

3-(Methylamino)-2-phenylpropanenitrile (8c)

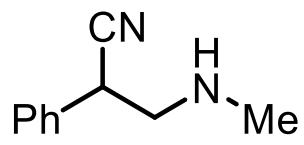

Following **GP6**, *trans*-aziridine borane **8a** (15 mg, 0.1 mmol, 1.0 equiv.) gave **8c** (44%) as a yellow oil. R_f 0.23 [EtOAc]. ^{1}H NMR (600 MHz, $CDCl_3$) δ 7.43 – 7.32 (5H, m), 4.01 (1H, dd, *J* = 7.9, 6.4 Hz), 3.11 (1H, dd, *J* = 12.4, 8.0 Hz), 2.99 (1H, dd, *J* = 12.3, 6.4 Hz), 2.48 (3H, s),1.33 (NH, br s); ^{13}C NMR (151 MHz, $CDCl_3$) δ 133.9, 129.3, 128.5, 127.7, 120.23, 56.3, 38.2, 36.0; HRMS (ESI) found MH^+ 161.1071, $C_{10}H_{13}N_2$ requires 161.1073.

3-(Hexylamino)-2-phenylpropanenitrile (9c)

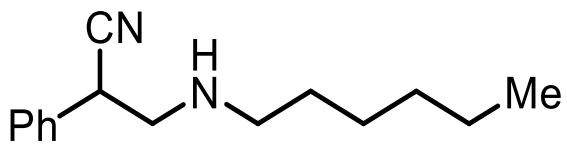

Following **GP6**, *trans*-aziridine borane **9a'** (22 mg, 0.1 mmol, 1.0 equiv.) gave **9c** (56%) as a yellow oil. R_f 0.56 [*n*-pentane:EtOAc (1:1)]. ^{1}H NMR (600 MHz, $CDCl_3$) δ 7.43 – 7.30 (5H, m), 3.99 (1H, dd, *J* = 8.1, 6.4 Hz), 3.13 (1H, dd, *J* = 12.4, 8.1 Hz), 3.01 (1H, dd, *J* = 12.4, 6.4 Hz), 2.72 – 2.57 (2H, m), 1.52 (NH, br s), 1.49 – 1.42 (2H, m), 1.31 – 1.23 (6H, m), 0.88 (3H, t, *J* = 6.7 Hz). ^{13}C NMR (151 MHz, $CDCl_3$) δ 134.1, 129.3, 128.5, 127.7, 120.4, 54.4, 49.4, 38.6, 31.8, 30.1, 27.0, 22.7, 14.2; HRMS (ESI) found MH^+ 231.1856, $C_{15}H_{23}N_2$ requires 231.1856.

3-(Benzylamino)-2-phenylpropanenitrile (10c)

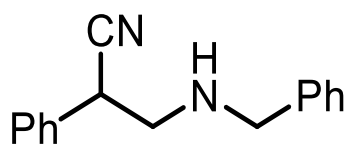

Following **GP6**, *trans*-aziridine borane **10a'** (22 mg, 0.1 mmol, 1.0 equiv.) gave **10c** (63%) as an oil. R_f 0.7 [*n*-pentane:EtOAc (1:1)]. ^{1}H NMR (400 MHz, $CDCl_3$) δ 7.39 – 7.11 (10H, m), 3.89 (1H, dd, *J* = 7.9, 6.3 Hz), 3.76 (2H, s), 3.04 (1H, dd, *J* = 12.4, 7.9 Hz), 2.95 (1H, dd, *J* = 12.4, 6.3 Hz), 1.62 (NH, s); ^{13}C NMR (151 MHz, $CDCl_3$) δ 139.6, 133.9, 129.3, 128.7, 128.5, 128.1, 127.7, 127.4, 120.3, 53.4, 53.3, 38.7; HRMS (ESI) found MH^+ 237.1385, $C_{16}H_{17}N_2$ requires 237.1386.

3-(Isopropylamino)-2-phenylpropanenitrile (11c)

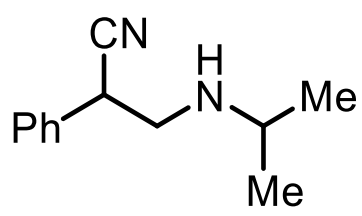

Following **GP6**, but running the reaction for 2.2 h, *cis*-aziridine borane **11a** (19 mg, 0.1 mmol, 1.0 equiv.) gave **11c** (63%) as an oil. R_f 0.47 [*n*-pentane:EtOAc (1:1)]. ^{1}H NMR (400 MHz, $CDCl_3$) δ 7.45 – 7.29 (5H, m), 3.96 (1H, dd, *J* = 8.2, 6.3 Hz), 3.10 (1H, dd, *J* = 12.3, 8.3 Hz), 3.01 (1H, dd, *J* = 12.3, 6.4 Hz), 2.87 (1H, hp, *J* = 6.3 Hz), 1.51 (NH, br s), 1.05 (6H, d, *J* = 6.3 Hz); ^{13}C

NMR (151 MHz, $CDCl_3$) δ 134.1, 129.3, 128.5, 127.7, 120.4, 51.9, 48.2, 38.9, 23.1, 22.9; HRMS (ESI) found MH^+ 189.1386, $C_{12}H_{17}N_2$ requires 189.1386.

3-(Cyclohexylamino)-2-phenylpropanenitrile (12c)

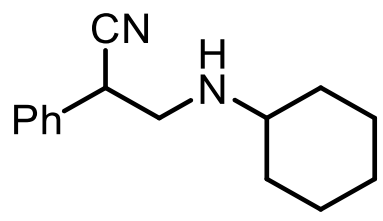

Following **GP6**, but running the reaction for 1.7 h, *trans*-aziridine borane **12a'** (43 mg, 0.2 mmol, 1.0 equiv.) gave **12c** (57%) as an oil. R_f 0.26 [*n*-pentane:EtOAc (3:1)]. ^{1}H NMR (600 MHz, $CDCl_3$) δ 7.41 – 7.31 (5H, m), 3.94 (1H, dd, *J* = 8.3, 6.3 Hz), 3.13 (1H, dd, *J* = 12.3, 8.3 Hz), 3.04 (1H, dd, *J* = 12.4, 6.3 Hz), 2.48 (1H, tt, *J* = 10.3, 3.7 Hz), 1.88 – 1.80 (2H, m), 1.75 – 1.69 (2H, m), 1.60 (1H, dt, *J* = 12.7, 3.9 Hz), 1.36 (NH, br s), 1.28 – 1.20 (2H, m), 1.16 (1H, tt, *J* = 12.2, 3.4 Hz), 1.10 – 1.02 (2H, m). ^{13}C NMR (151 MHz, $CDCl_3$) δ 134.2, 129.2, 128.4, 127.7, 120.4, 56.0, 51.5, 39.0, 33.7, 33.5, 26.1, 25.0, 24.9; HRMS (ESI) found MH^+ 229.1699, $C_{15}H_{21}N_2$ requires 229.1699.

3-(*tert*-Butylamino)-2-phenylpropanenitrile (17b)

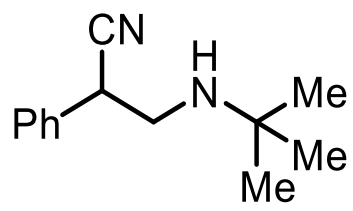

Following **GP6**, but running the reaction for 1.3 h, *trans*-aziridine borane **17a** (20 mg, 0.1 mmol, 1.0 equiv.) gave **17b** (46%) as an oil. R_f 0.55 [*n*-pentane:EtOAc (1:1)]. ^{1}H NMR (600 MHz, $CDCl_3$): 7.41 – 7.33 (5H, m), 3.88 (1H, dd, *J* = 8.3, 6.1 Hz), 3.04 (1H, dd, *J* = 11.6, 8.4 Hz), 2.96 (1H, dd, *J* = 11.6, 6.1 Hz), 1.08 (9H, s); ^{13}C NMR (151 MHz, $CDCl_3$): 134.3, 129.2, 128.5, 127.7, 120.5, 50.8, 48.1, 40.1, 29.1; HRMS (ESI) found MH+ 203.1534, $C_{13}H_{19}N_2$ requires 203.1543.

2-(4-Fluorophenyl)-3-((4-methoxyphenethyl)amino)propanenitrile (18b)

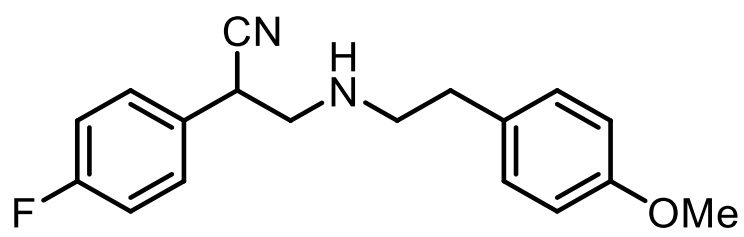

Following **GP6**, *cis*-aziridine borane **18a** and *trans*-aziridine borane **18a'** (Ratio 15:85, 57 mg, 0.2 mmol, 1.0 equiv.) gave **18b** (60%) as yellow oil. R_f 0.25 [*n*-pentane:EtOAc (6:4)]. ^{1}H NMR (600 MHz, $CDCl_3$) δ 7.50 – 7.43 (2H, m), 7.30 – 7.21 (4H, m), 7.02 (2H, d, *J* = 8.5 Hz), 4.12 (1H, dd, *J* = 7.6, 6.5 Hz), 3.99 (3H, s), 3.30 (1H, dd, *J* = 12.4, 7.7 Hz), 3.20 (1H, dd, *J* = 12.4, 6.5 Hz), 3.12 – 3.03 (2H, m), 2.91 (2H, t, *J* = 6.6 Hz); ^{13}C NMR (151 MHz, $CDCl_3$) δ 162.6 (d, *J* = 247.8 Hz), 158.3, 131.5, 129.7 (d, *J* = 3.7 Hz), 129.7, 129.4 (d, *J* = 8.3 Hz), 120.1, 116.2 (d, *J* = 22.0 Hz), 114.1, 55.3, 54.1, 50.7, 37.7, 35.4; ^{19}F NMR (564 MHz, $CDCl_3$) δ –113.41 (tt, *J* = 8.8, 5.1 Hz); HRMS (ESI) found MH^+ 299.1549, $C_{18}H_{20}FN_2O$ requires 299.1554.

3-((2,3-Dihydro-1*H*-inden-1-yl)amino)-2-(*p*-tolyl)propanenitrile (19b)

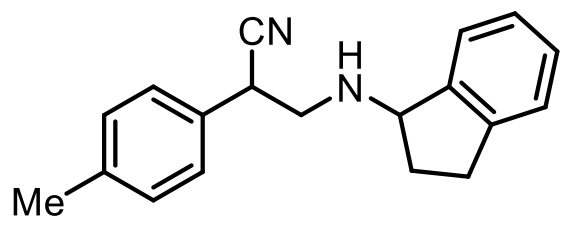

Following **GP6**, *trans*-aziridine borane **19a** (26 mg, 0.1 mmol, 1.0 equiv.) gave as mixture of diastereomers **19b** (54%) as a yellow oil. d.r. 1:1. R_f 0.42 [*n*-pentane:EtOAc (4:1)]. ^{1}H NMR (600 MHz, $CDCl_3$), *signals corresponding to both diastereomers*: δ 7.39 – 7.34 (1H, m), 7.34 – 7.29 (3H, m), 7.29 – 7.23 (4H, m), 4.36 (1H, t, *J* = 6.8 Hz), 4.02 (0.5H, dd, *J* = 8.2, 5.9 Hz), 3.99 (0.5H, t, *J* = 7.1 Hz), 3.28 – 3.21 (1H, m), 3.21 – 3.13 (1H, m), 3.07 – 3.00 (1H, m), 2.91 – 2.83 (1H, m), 2.50 – 2.43 (1H, m), 2.42 (3H, s), 1.88 – 1.79 (1H, m); ^{13}C NMR (151 MHz, $CDCl_3$) δ 144.6, 143.6, 138.4, 138.3, 131.0, 131.0, 130.0, 129.9, 129.9, 129.8, 127.8, 127.8, 127.7, 127.6, 126.5, 126.5, 125.0, 124.9, 124.3, 124.2, 120.6 (2C), 63.2, 63.1, 51.8, 51.7, 39.0, 39.0, 33.7, 33.7, 30.5, 30.4, 21.2 (2C); HRMS (ESI) found MH^+ 277.1698, $C_{19}H_{21}N_2$ requires 277.1699.

3-((Furan-2-ylmethyl)amino)-2-(*p*-tolyl)propanenitrile (20b)

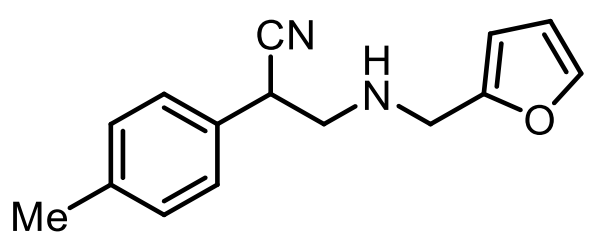

Following **GP6**, *cis*-aziridine borane **20a** and *trans*-aziridine borane **20a'** (Ratio 55:45, 45 mg, 0.2 mmol, 1.0 equiv.) gave **20b** (45%) as yellow oil. R_f 0.42 [*n*-pentane:EtOAc (6:4)]. ^{1}H NMR (600 MHz, $CDCl_3$) δ 7.38 – 7.33 (1H, m), 7.21 (2H, d, *J*=8.0 Hz), 7.18 (2H, d, *J* = 8.1 Hz), 6.31 (1H, dd, *J* = 3.2, 1.8 Hz), 6.17 (1H, d, *J* = 3.1 Hz), 3.90 (1H, dd, *J* = 8.0, 6.4 Hz), 3.83 (2H, s), 3.09 (1H, ddd, *J* = 12.3, 8.1, 0.9 Hz), 2.99 (1H, ddd, *J* = 12.3, 6.4, 0.9 Hz), 2.35 (3H, s), 2.35 (NH, br s); ^{13}C NMR (151 MHz, $CDCl_3$) δ 153.2, 142.2, 138.4, 130.8, 130.0, 127.6, 120.4, 110.3, 107.5, 53.3, 45.7, 38.2, 21.2; HRMS (ESI) found MH^+ 241.1328, $C_{15}H_{17}N_2O$ requires 241.1335.

3-((2,2-Dimethoxyethyl)amino)-2-(naphthalen-2-yl)propanenitrile (21b)

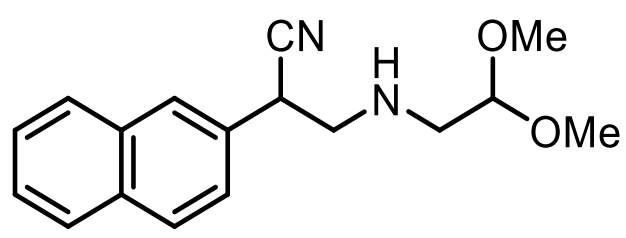

Following **GP6**, *trans*-aziridine borane **21a** (27 mg, 0.1 mmol, 1.0 equiv.) gave **21b** (41%) as a yellow oil. R_f 0.19 [*n*-pentane:EtOAc (6:4)]. ^{1}H NMR (600 MHz, $CDCl_3$) δ 7.91 – 7.81 (4H, m), 7.56 7.48 (2H, m), 7.42 (1H, dd, *J* = 8.5, 1.9 Hz), 4.42 (1H, t, *J* = 5.3 Hz), 4.14 (1H, dd, *J* = 8.0, 6.2 Hz), 3.40 – 3.32 (6H, m), 3.24 (1H, dd, *J* = 12.6, 8.0 Hz), 3.15 (1H, dd, *J* = 12.6, 6.2 Hz), 2.81 (2H, d, *J* = 5.3 Hz); ^{13}C NMR (151 MHz, $CDCl_3$) δ 133.4, 133.1, 131.2, 129.3, 128.0, 127.9, 127.0, 126.9, 126.8, 125.0, 120.3, 104.1, 54.4, 54.4, 54.3, 50.8, 39.0; HRMS (ESI) found MNa^+ 307.1416, $C_{17}H_{20}N_2O_2Na$ requires 307.1417.

3-(Cyclopentylamino)-2-(*o*-tolyl)propanenitrile (22b)

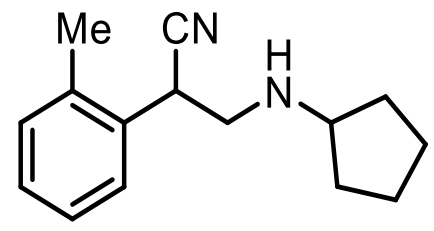

Following **GP6**, but running the reaction for 4.2 h, *trans*-aziridine borane **22a** (22 mg, 0.1 mmol, 1.0 equiv.) gave **22b** (49%) as a yellow oil. R_f 0.53 [*n*-pentane:EtOAc (6:4)]. ^{1}H NMR (600 MHz, $CDCl_3$) δ 7.44 (1H, dd, *J* = 7.0, 2.1 Hz), 7.26 – 7.23 (2H, m), 7.22 – 7.18 (1H, m), 4.18 (1H, dd, *J* = 9.1, 5.5 Hz), 3.19 – 3.13 (1H, m), 3.06 (1H, dd, *J* = 12.4, 9.2 Hz), 2.94 (1H, dd, *J* = 12.4, 5.5 Hz), 2.37 (3H, s), 1.89 – 1.78 (2H, m), 1.74 – 1.65 (2H, m), 1.66 – 1.58 (1H, m), 1.57 – 1.51 (2H, m), 1.38 – 1.29 (2H, m); ^{13}C NMR (151 MHz, $CDCl_3$) δ 135.3, 132.2, 131.0, 128.3, 127.7, 126.9, 120.6, 59.1, 51.7, 35.7, 33.3, 33.0, 24.0, 23.9, 19.2; HRMS (ESI) found MH^+ 229.1691, $C_{15}H_{21}N_2$ requires 229.1699.

***tert*-Butyl 4-((2-(4-Chlorophenyl)-2-cyanoethyl)amino)piperidine-1-carboxylate (23b)**

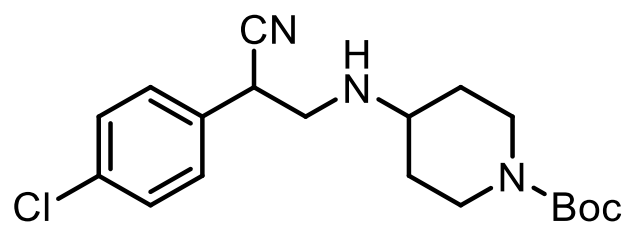

Following **GP6**, but running the reaction for 3 h, *trans*-aziridine borane **23a** (35 mg, 0.1 mmol, 1.0 equiv.) gave **23b** (40%, 7% rsm) as yellow oil. R_f 0.28 [*n*-pentane:EtOAc (6:4)]. ^{1}H NMR (600 MHz, $CDCl_3$) δ 7.37 (2H, d, *J* = 8.5 Hz), 7.29 (2H, d, *J* = 8.5 Hz), 3.98 (2H, br s), 3.90 (1H, dd, *J* = 7.7, 6.2 Hz), 3.10 (1H, dd, *J* = 12.3, 7.7 Hz), 3.04 (1H, dd, *J* = 12.4, 6.2 Hz), 2.84 – 2.78 (1H, br m), 2.65 (1H, tt, *J* = 10.0, 3.9 Hz), 1.82 – 1.74 (2H, br m), 1.48 – 1.45 (1H, m), 1.45 (9H, s), 1.22 (2H, br m); ^{13}C NMR (101 MHz, $CDCl_3$) δ 154.9, 134.6, 132.5, 129.5, 129.1, 119.9, 79.7, 54.4, 51.2, 42.4 (br s), 38.7, 32.6, 32.5, 28.6; HRMS (ESI) found MNa^+ 386.1604, $C_{19}H_{26}ClN_3O_2Na$ requires 386.1606.

***tert*-Butyl (2-cyano-2-phenylethyl)glycinate (24b)**

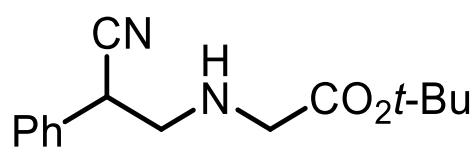

Following **GP6**, *trans*-aziridine borane **24a'** (49 mg, 0.2 mmol, 1.0 equiv.) gave **24b** (50%) as an oil. R_f 0.65 [*n*-pentane:EtOAc (1:1)]. ^{1}H NMR (600 MHz, $CDCl_3$) δ 7.42 – 7.31 (5H, m), 3.94 (1H, dd, *J* = 7.8, 6.2 Hz), 3.34 (2H, s), 3.13 (1H, dd, *J* = 12.2, 7.8 Hz), 3.06 (1H, dd, *J* = 12.2, 6.2 Hz), 1.76 (NH, br s), 1.45 (9H, s); ^{13}C NMR (151 MHz, $CDCl_3$) δ 171.3, 133.8, 129.3, 128.5, 127.7, 120.2, 81.7, 53.9, 51.5, 39.1, 28.2; HRMS (ESI) found MNa^+ 283.1417, $C_{15}H_{20}N_2O_2Na$ requires 283.1417.

tert-Butyl 2-((4_R_,6_R_)-6-(2-((2-(4-Chlorophenyl)-2-cyanoethyl)amino)ethyl)-2,2-dimethyl-1,3-dioxan-4-yl)acetate (25b)

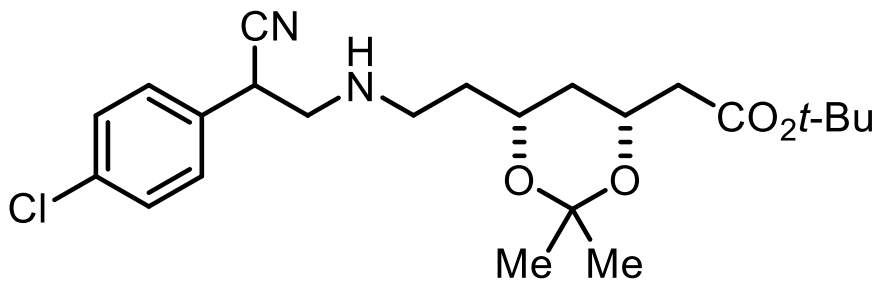

Following **GP6**, *trans*-aziridine borane **25a** (42 mg, 0.1 mmol, 1.0 equiv.) gave **25b** as mixture of diastereomers (35%) as a yellow oil. d.r. 1:1. R_f 0.13 [*n*-pentane:EtOAc (3:2)]. ^{1}H NMR (600 MHz, $CDCl_3$), *signals corresponding to both diastereomers*: δ 7.37 (2H, d, *J* = 8.5 Hz), 7.29 (2H, d, *J* = 8.6 Hz), 4.30 – 4.17 (1H, m), 3.94 – 3.90 (2H, m), 3.15 – 3.04 (1H, m), 2.98 (1H, dd, *J* = 12.4, 6.3 Hz), 2.81 – 2.67 (2H, m), 2.42 (1H, dd, *J* = 15.1, 7.0 Hz), 2.28 (1H, dd, *J* = 15.2, 6.1 Hz), 1.75 (NH, br s), 1.68 – 1.56 (2H, m), 1.44 (9H, s), 1.41 (3H, s), 1.32 (3H, s), 1.27 – 1.14 (2H, m); ^{13}C NMR (151 MHz, $CDCl_3$) δ 170.4, 134.6, 132.6, 129.5, 129.1, 129.1, 119.9, 98.8, 80.8, 67.9, 67.8, 66.3, 54.6, 54.3, 45.9, 42.8, 38.1, 38.1, 36.7, 36.6, 36.3, 36.3, 30.2, 28.3, 19.9, 19.8; HRMS (ESI) found MH^+ 437.2200, $C_{23}H_{34}ClN_2O_4$ requires 437.2202.

<u>Note</u>*: There is no difference in chemical shift between the two diastereomers in ^{1}H NMR but in ^{13}C NMR, the carbon atoms close to the stereocenter are splitting, indicating the presence of a mixture of diastereomers.*

4-Phenyl-2-((propylamino)methyl)butanenitrile (26b)

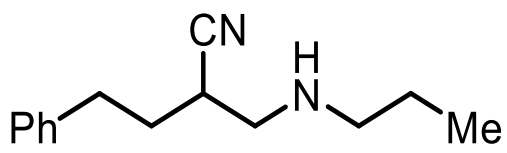

Following **GP6**, *trans*-aziridine borane **26a** (41 mg, 0.2 mmol, 1.0 equiv.) gave **26b** (66%) as an oil. R_f 0.39 [*n*-pentane:EtOAc (1:1)]. ^{1}H (600 MHz, $CDCl_3$) δ 7.30 (2H, t, *J* = 7.5 Hz), 7.25 – 7.18 (3H, m), 2.95 – 2.84 (2H, m), 2.80 – 2.66 (3H, m), 2.63 – 2.51 (2H, m), 2.01 – 1.85 (2H, m), 1.48 (2H, sx, *J* = 7.3 Hz), 1.24 (NH, br s), 0.91 (3H, t, *J* = 7.4 Hz); ^{13}C NMR (151 MHz, $CDCl_3$) δ 140.2, 128.8, 128.5, 126.6, 121.6, 51.5, 51.1, 33.3, 32.3, 31.8, 23.3, 11.7; HRMS (ESI) found MH^+ 217.1691, $C_{14}H_{21}N_2$ requires 217.1699.

2-((Hexylamino)methyl)-4-phenylbutanenitrile (27b)

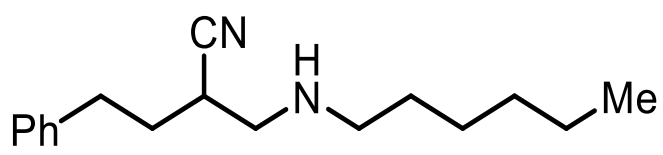

Following **GP6**, *trans*-aziridine borane **27a** (49 mg, 0.2 mmol, 1.0 equiv.) gave **27b** (47%) as an oil. R_f 0.56 [*n*-pentane:EtOAc (1:1)]. ^{1}H (400 MHz, $CDCl_3$) δ 7.30 (2H, t, *J* = 7.5 Hz), 7.25 – 7.19 (3H, m), 2.94 – 2.83 (2H, m), 2.80 – 2.65 (3H, m), 2.65 – 2.53 (2H, m), 2.04 – 1.82 (2H, m), 1.49 – 1.39 (2H, m), 1.33 – 1.25 (6H, m), 0.89 (3H, t, *J* = 6.6 Hz); ^{13}C NMR (101 MHz, $CDCl_3$) δ

140.2, 128.8, 128.5, 126.5, 121.6, 51.2, 49.7, 33.3, 32.3, 31.8(2C), 30.1, 27.0, 22.7, 14.1; HRMS (ESI): found MH+ 259.2168, $C_{17}H_{27}N_2$ requires 259.2169.

2-((Isopropylamino)methyl)-4-phenylbutanenitrile (13c)

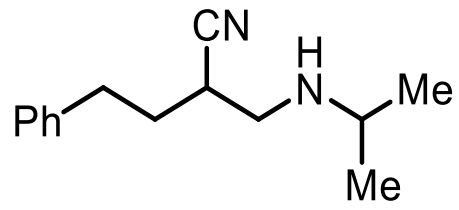

Following **GP6**, *trans*-aziridine borane **13a** (41 mg, 0.2 mmol, 1.0 equiv.) gave **13c** (72%) as an oil. R_f 0.33 [*n*-pentane:EtOAc (1:1)]. ^{1}H (600 MHz, $CDCl_3$) δ 7.33 – 7.28 (2H, t, *J* = 7.5 Hz), 7.25 – 7.18 (3H, m), 2.95 – 2.71 (5H, m), 2.66 (1H, ddt, *J* = 10.3, 8.1, 5.4 Hz), 2.02 – 1.93 (1H, m), 1.93 – 1.85 (1H, m), 1.04 (3H, d, *J*=6.2 Hz), 1.04 (3H, d, *J*=6.2 Hz); ^{13}C NMR (151 MHz, $CDCl_3$) δ 140.3, 128.8, 128.6, 126.6, 121.6, 48.7, 48.6, 33.4, 32.7, 31.9, 23.1, 23.0; HRMS (ESI) found MH+ 217.1692, $C_{14}H_{21}N_2$ requires 217.1699.

The structure of **13c** was further confirmed by 2D NMR spectroscopy (COSY, HSQC, and HMBC), which also established the regioselectivity of the reaction.

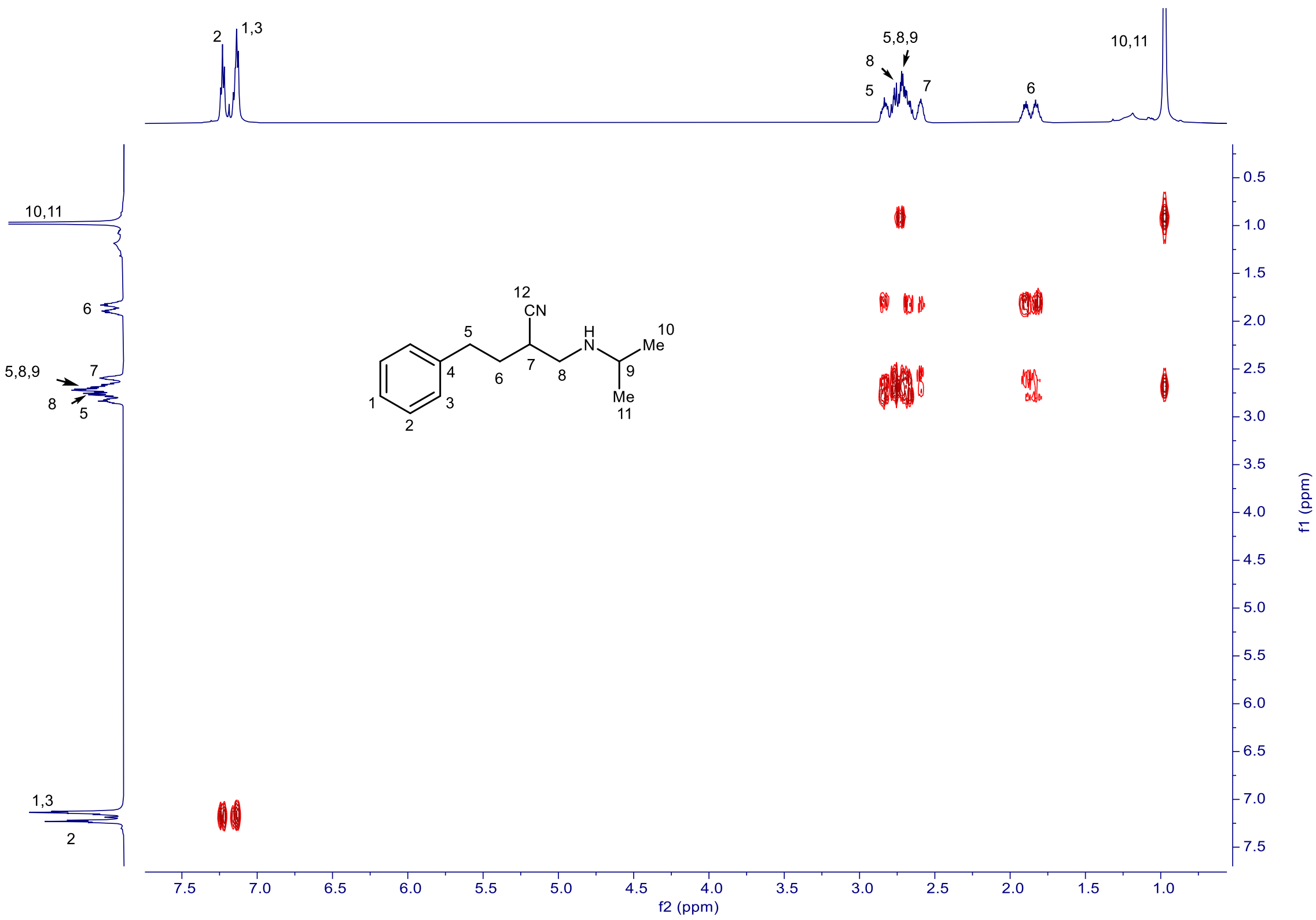

COSY NMR spectrum (600 MHz, $CDCl_3$, at rt) of **13c**

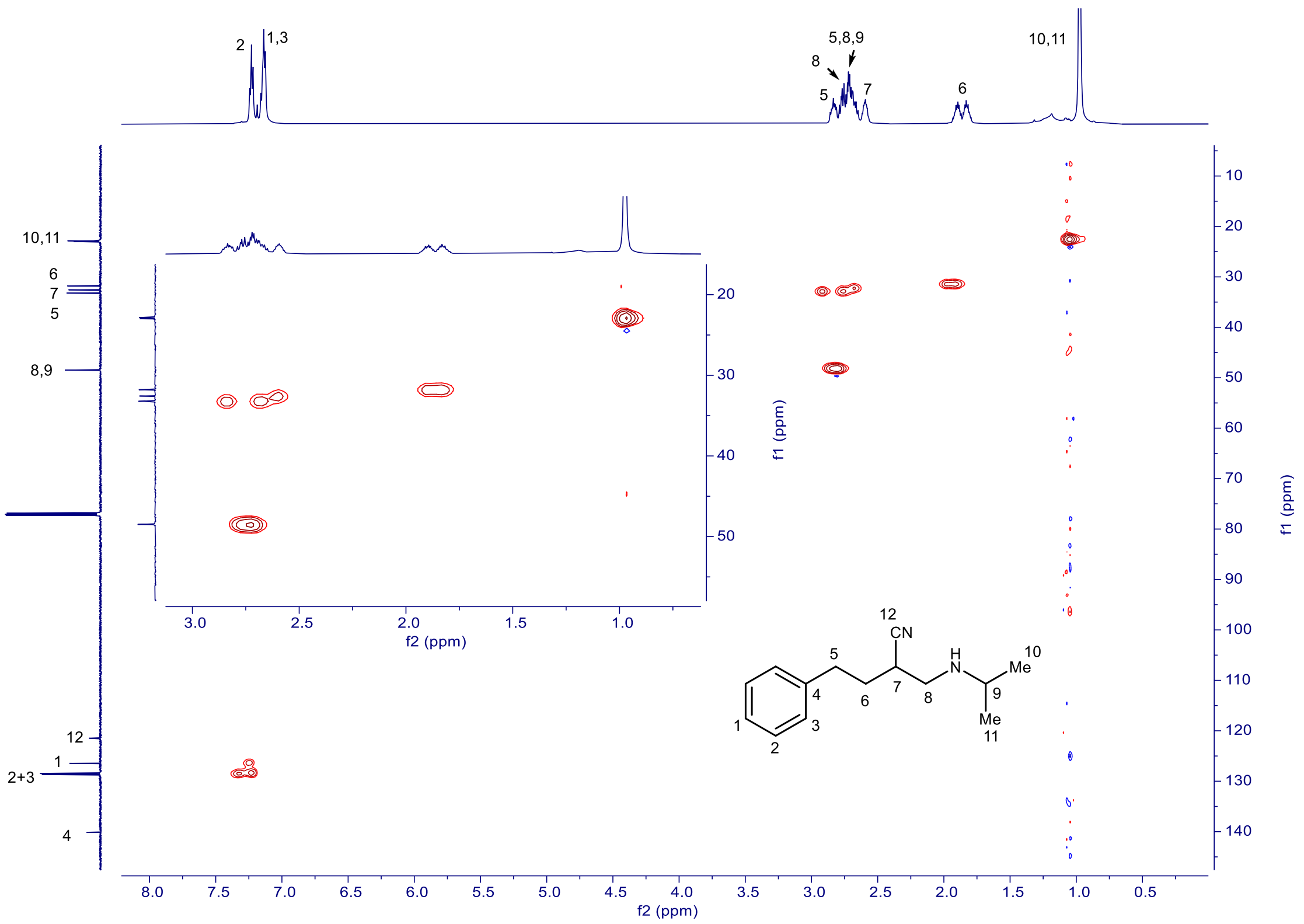

HSQC NMR spectrum (600 MHz, $CDCl_3$, at rt) of **13c**

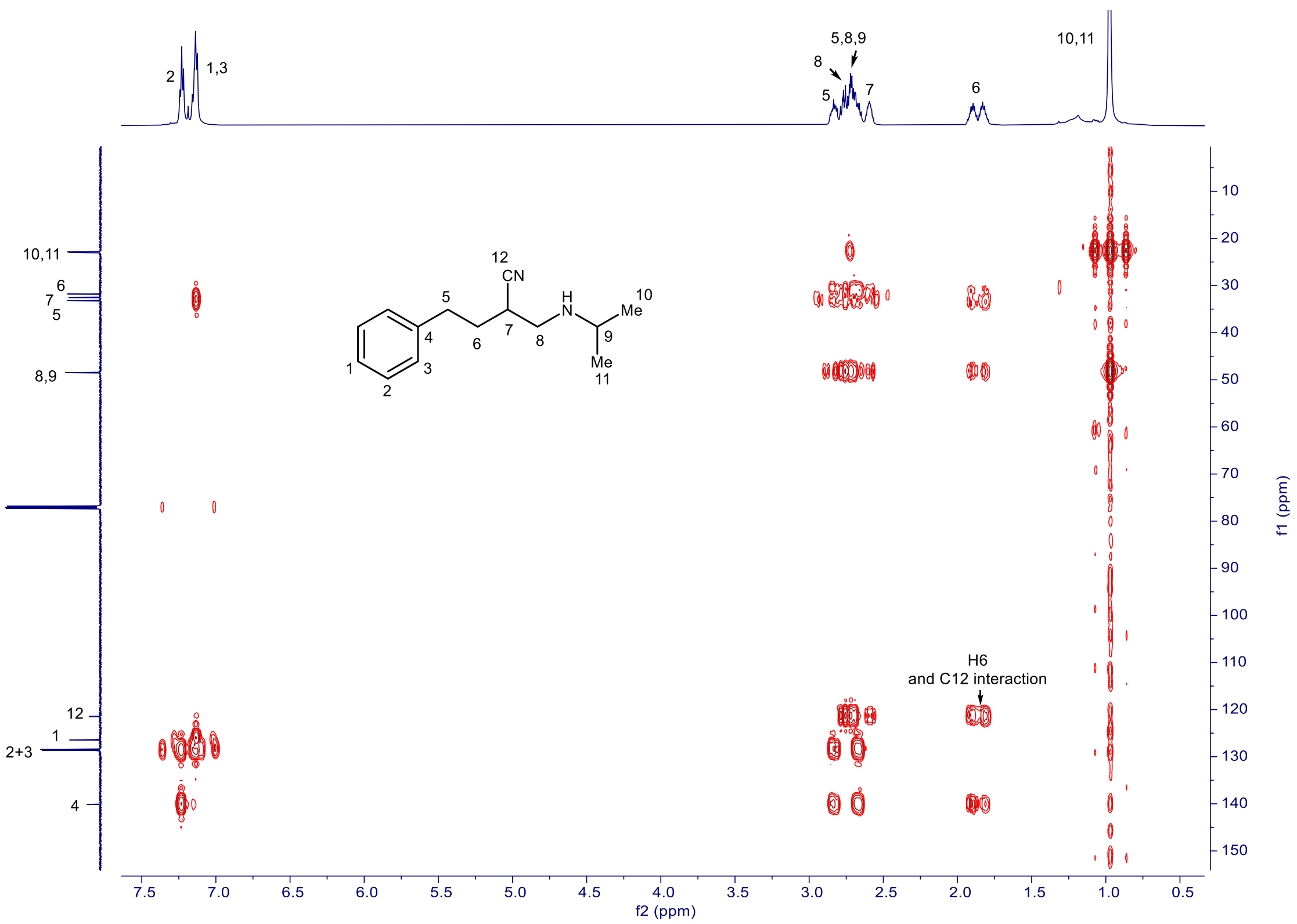

HMBC NMR spectrum (600 MHz, $CDCl_3$, at rt) of **13c**

3-(Benzylamino)-2-methylpropanenitrile (28b)

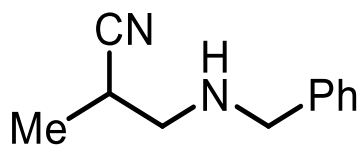

Following **GP6**, *trans*-aziridine borane **28a** (32 mg, 0.2 mmol, 1.0 equiv.) gave **28b** (52%) as an oil. R_f 0.29 [*n*-pentane:EtOAc (5:1)]. ^{1}H NMR (600 MHz, $CDCl_3$) δ 7.28 – 7.17 (5H, m), 3.81 – 3.73 (2H, m), 2.81 – 2.74 (1H, m), 2.74 – 2.66 (2H, m), 1.56 (NH, br s), 1.23 (3H, d, *J* = 6.8 Hz); ^{13}C NMR (151 MHz, $CDCl_3$) δ 139.8, 128.6, 128.2, 127.3, 122.4, 53.5, 51.9, 26.9, 15.8; HRMS (ESI) found MH^+ 175.1229, $C_{11}H_{15}N_2$ requires 175.1230.[20]

2-((Benzylamino)methyl)hexanenitrile (29b)

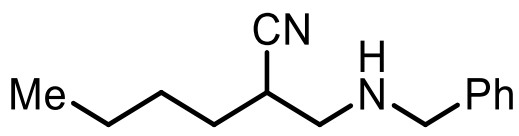

Following **GP6**, *trans*-aziridine borane **29a** (41 mg, 0.2 mmol, 1.0 equiv.) gave **29b** (67%) as an oil. R_f 0.29 [*n*-pentane:EtOAc (5:1)]. ^{1}H NMR (600 MHz, $CDCl_3$) 7.36 – 7.30 (4H, m), 7.29 – 7.25 (1H, m), 3.88 – 3.80 (2H, m), 2.86 (1H, dd, *J* = 12.1, 8.2 Hz), 2.79 (1H, dd, *J* = 12.2, 5.5 Hz), 2.70 (1H, tt, *J* = 8.5, 5.5 Hz), 1.67 – 1.55 (2H, m), 1.54 – 1.45 (1H, m), 1.45 – 1.28 (3H, m), 0.92 (3H, t, *J* = 7.2 Hz); ^{13}C NMR (151 MHz, $CDCl_3$) δ 139.9, 128.7, 128.2, 127.4, 121.8, 53.7, 50.5, 33.1, 29.9, 29.4, 22.4, 13.9; HRMS (ESI) found MH^+ 217.1693, $C_{14}H_{21}N_2$ requires 217.1699.

2-(((1-(2,6-Dimethylphenoxy)propan-2-yl)amino)methyl)hexanenitrile (30b)

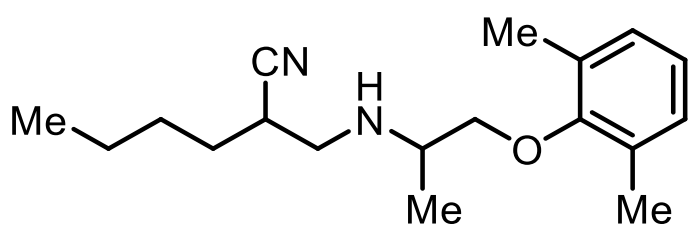

Following **GP6**, *trans*-aziridine borane **30a** (28 mg, 0.1 mmol, 1.0 equiv.) gave **30b** as mixture of diastereomers (68%) as an oil. d.r. 1:1. R_f 0.33 [*n*-pentane:EtOAc (5:1)]. ^{1}H NMR (600 MHz, $CDCl_3$), s*ignals corresponding to both diastereomers*: δ 7.01 (2H, d, *J*=7.4 Hz), 6.95 – 6.89 (1H, m), 3.70 – 3.63 (2H, m), 3.19 – 3.11 (1H, m), 3.01 – 2.88 (2H, m), 2.78 2.68 (1H, m), 2.28 (6H, s), 1.79 (1H, NH), 1.71 – 1.59 (2H, m), 1.59 – 1.49 (1H, m), 1.48 – 1.33 (3H, m), 1.20 – 1.14 (3H, m), 0.96 – 0.90 (3H, m); ^{13}C NMR (151 MHz, $CDCl_3$) δ 155.6, 155.5, 130.9, 129.1, 124.1, 124.0, 121.8, 121.8, 76.0, 75.9, 53.3, 53.2, 48.8, 48.8, 33.5, 33.4, 29.9, 29.8, 29.4, 29.4, 22.4, 22.4, 17.5, 17.4, 16.5, 16.5, 13.9; HRMS (ESI) found MH^+ 289.2269, $C_{18}H_{29}N_2O$ requires 289.2274.

3-(Methylamino)-2-propylhexanenitrile (31b)

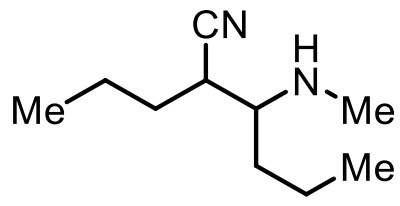

Following **GP6**, **31a** (31 mg, 0.2 mmol, 2.0 equiv.) gave **31b** (49%) as mixture of separable diastereomers. d.r. 1.6:1.

<u>Data for the major diasteromer</u>: (30%), obtained as an oil, R_f 0.47 [*n*-pentane:EtOAc (1:1)]; ^{1}H NMR (600 MHz, $CDCl_3$) δ 2.66 (1H, dt, *J* = 10.4, 4.4 Hz), 2.59 – 2.53 (1H, m), 2.45 (3H, s), 1.73 – 1.65 (1H, m), 1.64 – 1.49 (4H, m), 1.46 – 1.35 (3H, m), 0.97 (3H, dt, *J* = 7.1 Hz), 0.95 (3H, d, *J* = 7.3 Hz); ^{13}C NMR (151 MHz, $CDCl_3$) δ 60.4, 36.4, 34.7, 34.6, 30.6, 21.1, 19.6, 14.2, 13.8; HRMS (ESI) found MH^+ 169.1701, $C_{10}H_{21}N_2$ requires 169.1699.

<u>Data for the minor diasteromer</u>: (19%), obtained as an oil, R_f 0.37 [*n*-pentane:EtOAc (1:1)]; ^{1}H NMR (400 MHz, $CDCl_3$) δ 2.82 (1H, dt, *J* = 9.9, 4.5 Hz), 2.54 (1H, dt, *J* = 8.4, 4.1 Hz), 2.45 (3H, s), 1.71 – 1.28 (8H, m), 0.98 (3H, t, *J* = 7.1 Hz), 0.95 (3H, t, *J* = 6.8 Hz); ^{13}C NMR (101 MHz, $CDCl_3$) δ 60.5, 35.8, 34.0, 33.6, 31.1, 21.2, 19.5, 14.2, 13.8; HRMS (ESI) found MH^+ 169.1699, $C_{10}H_{21}N_2$ requires 169.1699.

3-(Methylamino)-2,3-diphenylpropanenitrile (32b)

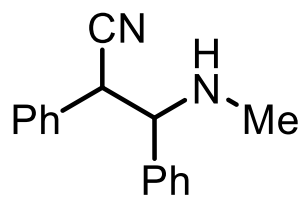

Following **GP6**, **32a** (44.6 mg, 0.2 mmol, 1.0 equiv.) gave **32b** (79%) as mixture of separable diastereomers. d.r. = 1.5:1.

<u>Data for the major diasteromer</u>: (47%), obtained as an oil, R_f 0.63 [*n*-pentane:EtOAc (1:1)]; ^{1}H NMR (600 MHz, $CDCl_3$) δ 7.32 – 7.26 (6H, m), 7.22 – 7.15 (4H, m), 4.01 (1H, d, *J* = 6.4 Hz), 3.90 (1H, d, *J* = 6.3 Hz), 2.27 (3H, s), 1.73 (1H, br s); ^{13}C NMR (151 MHz, $CDCl_3$) δ 138.7, 133.2, 128.9, 128.6, 128.4, 128.3(8), 128.3(5), 127.8, 119.6, 68.7, 46.3, 34.7; HRMS (ESI) found MH^+ 237.1384, $C_{16}H_{17}N_2$ requires 237.1386.

<u>Data for the minor diasteromer</u>: (32%), obtained as an oil, R_f 0.46 [*n*-pentane:EtOAc (1:1)]; ^{1}H NMR (600 MHz, $CDCl_3$) δ 7.36 – 7.30 (6H, m), 7.25 – 7.21 (2H, m), 7.21 – 7.16 (2H, m), 4.11 (1H, d, *J* = 7.2 Hz), 3.88 (1H, d, *J* = 7.2 Hz), 2.27 (3H, s), 1.70 (1H, br s). ^{13}C NMR (151 MHz, $CDCl_3$) δ 138.3, 133.1, 129.1, 128.6(3), 128.6(0), 128.5(8), 128.5(5), 128.0, 119.3, 68.6, 45.7, 34.6; HRMS (ESI) found MH^+ 237.1382, $C_{16}H_{17}N_2$ requires 237.1386.

trans-2-(Cyclohexylamino)cyclohexane-1-carbonitrile (33b)

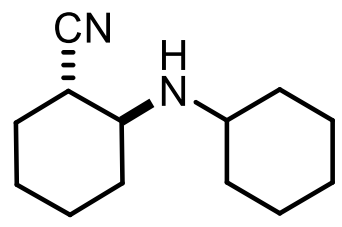

Following **GP6**, _trans_-aziridine borane **33a** (19 mg, 0.1 mmol, 1.0 equiv.) gave **33b** (58%) as an oil. R_f 0.53 [_n_-pentane:EtOAc (1:1)]. ^{1}H (400 MHz, $CDCl_3$) δ 2.74 (1H, td, _J_=9.8, 3.9 Hz), 2.58 (1H, tt, _J_=10.3, 3.8 Hz), 2.32 (1H, td, _J_=10.4, 3.7 Hz), 2.16 – 1.98 (2H, m), 1.98 – 1.88 (1H, m), 1.87 – 1.78 (1H, m), 1.78 – 1.66 (4H, m), 1.63 – 1.54 (2H, m), 1.33 – 1.01 (8H, m); ^{13}C (151 MHz, $CDCl_3$) δ 122.3, 55.0, 54.1, 36.8, 34.9, 33.9, 32.8, 29.0, 26.2, 25.3, 25.1, 24.5, 24.1; HRMS (ESI) found MH^+ 207.1851, $C_{13}H_{23}N_2$ requires 207.1856.

The _trans_ configuration was assigned based on the coupling constants observed for peaks at 2.74 ppm (1H, td, _J_=9.8, 3.9 Hz) and 2.32 (1H, td, _J_=10.4, 3.7 Hz).

trans-2-(Benzylamino)cyclohexane-1-carbonitrile (34b)

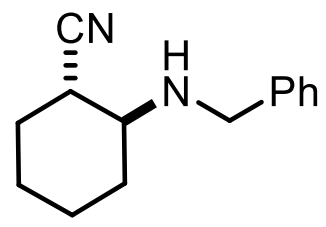

Following **GP6**, _trans_-aziridine borane **34a** (40.2 mg, 0.2 mmol, 1.0 equiv.) gave **34b** (46%) as oil. R_f 0.63 [_n_-pentane:EtOAc (5:1)]. ^{1}H NMR (400 MHz, $CDCl_3$) δ 7.33 – 7.13 (5H, m), 3.84 (1H, d, _J_ = 13.1 Hz), 3.75 (1H, d, _J_ = 13.1 Hz), 2.63 (1H, td, _J_ = 9.7, 3.8 Hz), 2.33 (1H, ddd, _J_ = 11.1, 9.4, 3.8 Hz), 2.06 – 1.95 (2H, m), 1.69 – 1.61 (2H, m), 1.57 – 1.43 (2H, m), 1.22 – 1.10 (2H, m); ^{13}C NMR (101 MHz, $CDCl_3$) δ 140.2, 128.6, 128.2, 127.3, 122.1, 57.2, 51.1, 35.9, 31.6, 28.8, 24.5, 23.8; HRMS (ESI) found MNa^+ 237.1356, $C_{14}H_{18}N_2Na$ requires 237.1362.

The _trans_ configuration was assigned based on the coupling constants observed for peaks at 2.63 (1H, td, _J_ = 9.7, 3.8 Hz) and 2.33 (1H, ddd, _J_ = 11.1, 9.4, 3.8 Hz).

2-((methylamino)methyl)-2-pentylheptanenitrile (35b)

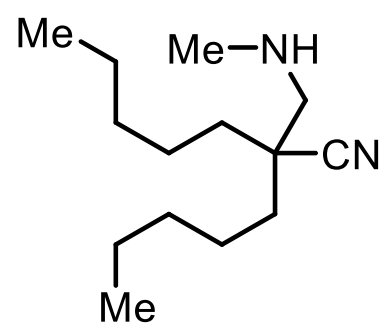

Following **GP6**, **35a** (21.1 mg, 0.1 mmol, 1.0 equiv.) gave **35b** (23%) as an oil. R_f 0.53 [_n_-pentane:EtOAc (1:1)]. ^{1}H (400 MHz, $CDCl_3$) δ 2.68 (2H, s), 2.49 (3H, s), 1.65 – 1.51 (4H, m), 1.45 – 1.28 (12H, m), 0.90 (8H, t, _J_=6.8 Hz); ^{13}C (101 MHz, $CDCl_3$) δ 123.8, 56.7, 42.3, 37.3, 34.8, 32.1, 24.2, 22.6, 14.1; HRMS (ESI) found MH^+ 225.2325, $C_{14}H_{29}N_2$ requires 225.2325.

The structure of **35b** was further confirmed by 2D NMR spectroscopy (COSY, HSQC, and HMBC), which also established the regioselectivity of the reaction.

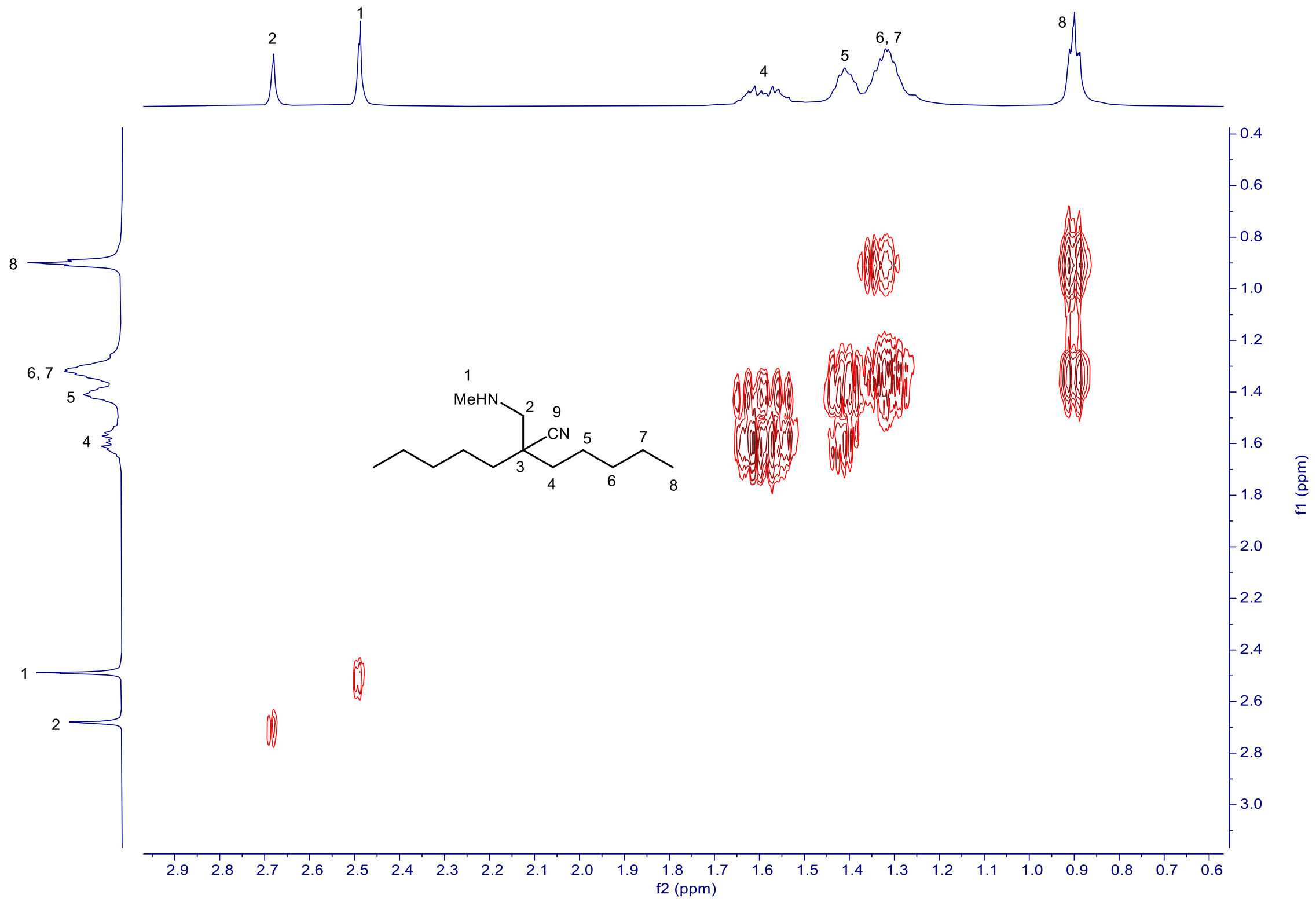

COSY NMR spectrum (600 MHz, $CDCl_3$, at rt) of **35b**

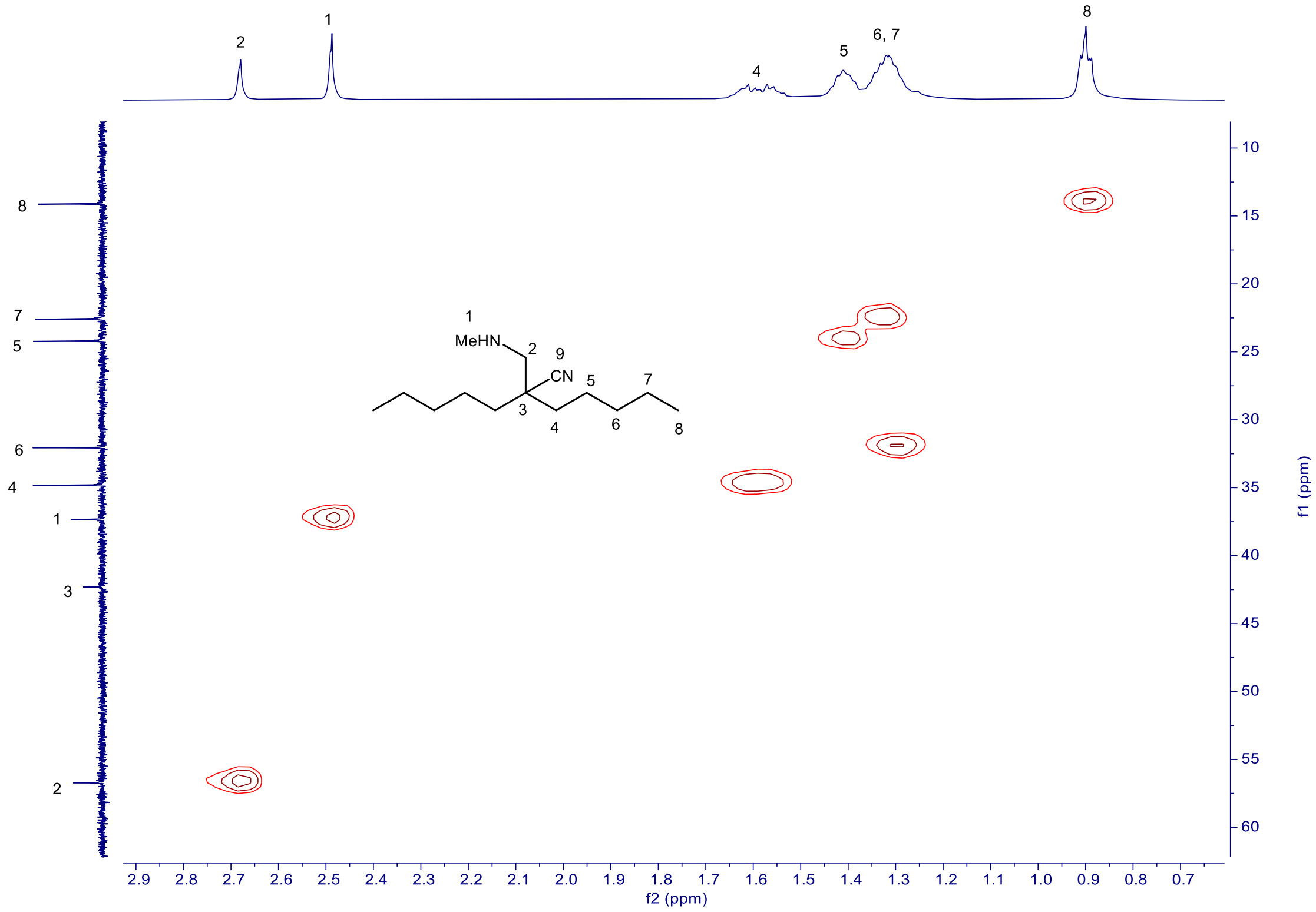

HSQC NMR spectrum (600 MHz, $CDCl_3$, at rt) of **35b**

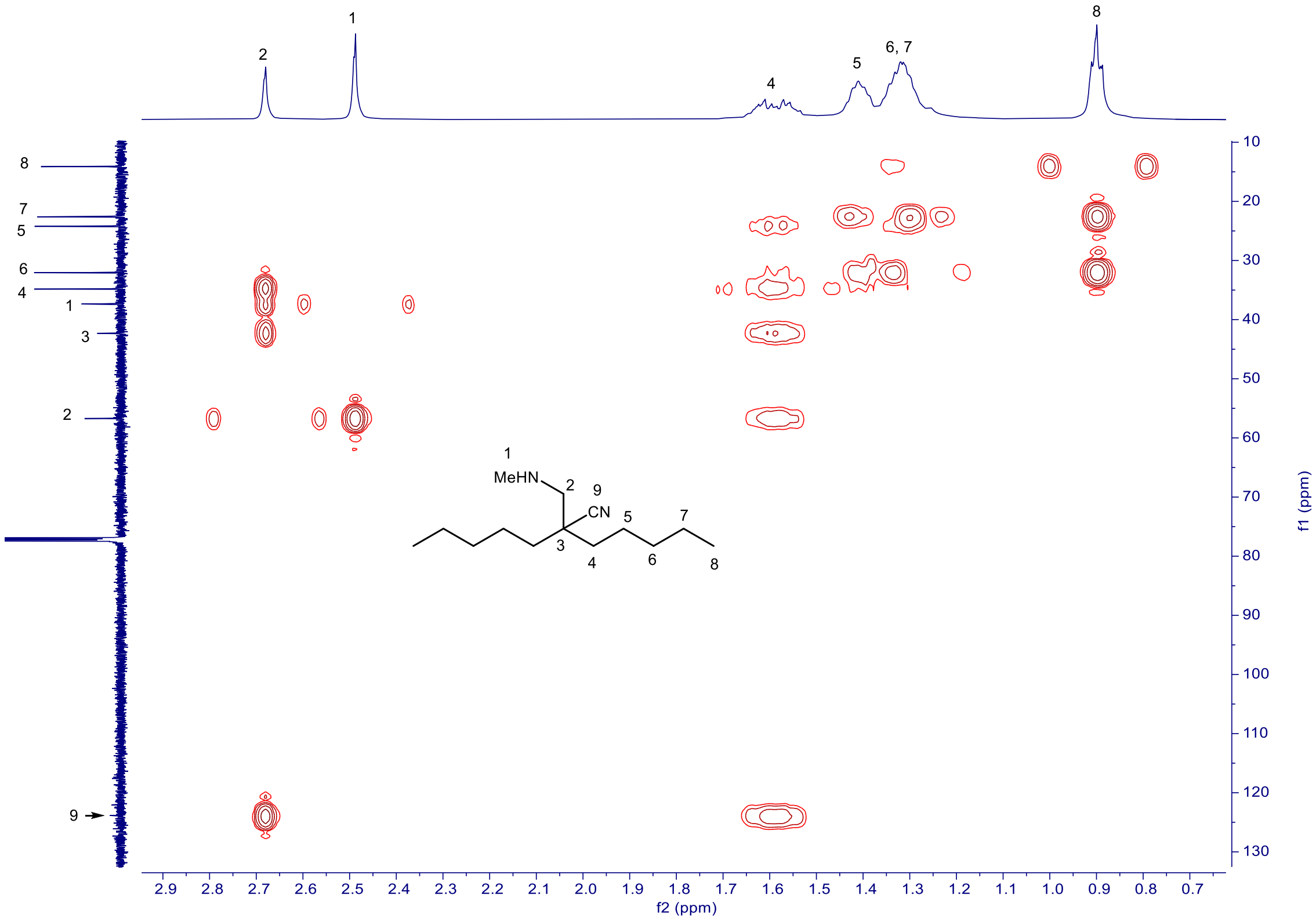

HMBC NMR spectrum (600 MHz, $CDCl_3$, at rt) of **35b**

3-((2-(Naphthalen-1-yloxy)ethyl)amino)propanenitrile (36b)

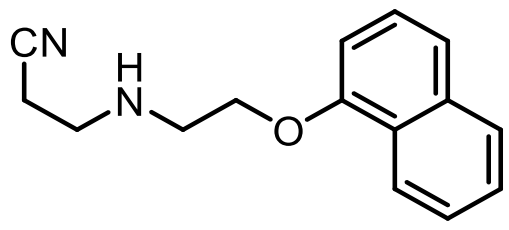

Following **GP6**, **36a** (45 mg, 0.2 mmol, 1.0 equiv.) gave **36b** (58%) as an oil. R_f 0.27 [EtOAc]. ^{1}H NMR (400 MHz, $CDCl_3$) δ 8.28 – 8.21 (1H, m), 7.84 – 7.77 (1H, m), 7.53 – 7.42 (3H, m), 7.37 (1H, t, J = 7.9 Hz), 6.83 (1H, d, J = 7.5 Hz), 4.26 (2H, t, J = 5.1 Hz), 3.19 (2H, t, J = 5.1 Hz), 3.07 (2H, t, J = 6.6 Hz), 2.56 (2H, t, J = 6.7 Hz), 2.01 (NH, br s); ^{13}C NMR (101 MHz, $CDCl_3$) δ 154.4, 134.6, 127.7, 126.6, 125.9, 125.7, 125.4, 121.9, 120.8, 118.7, 105.0, 67.8, 48.3, 45.2, 19.0; HRMS (ESI) found MNa^+ 263.1152, $C_{15}H_{16}N_2ONa$ requires 263.1155.

5.3 Ring-Opening–Thioetherification

_N_-(2-Phenyl-2-(phenylthio)ethyl)propan-1-amine (1r)

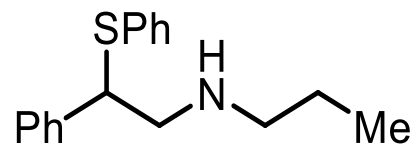

Following **GP7**, *trans*-aziridine borane **1a** (35 mg, 0.2 mmol) and diphenyl disulfide (22 mg, 0.1 mmol) gave **1r** (60%) as an oil. R_f 0.31 [*n*-pentane:EtOAc (6:4)]. ^{1}H NMR (600 MHz, $CDCl_3$) δ 7.32 – 7.27 (6H, m), 7.25 – 7.17 (4H, m), 4.38 (1H, t, *J* = 7.3 Hz), 3.15 – 3.03 (2H, m), 2.63 – 2.52 (2H, m), 1.46 (2H, sx, *J* = 7.4 Hz), 0.86 (3H, t, *J* = 7.4 Hz); ^{13}C NMR (151 MHz, $CDCl_3$) δ 140.6, 134.5, 132.4, 128.9, 128.7, 128.0, 127.6, 127.3, 54.5, 53.3, 51.4, 23.1, 11.8; HRMS (ESI) found MH^+ 272.14665, $C_{17}H_{22}NS$ requires 272.14675.

The structure of **1r** was further confirmed by 2D NMR spectroscopy (COSY, HSQC, and HMBC), which also established the regioselectivity of the reaction.

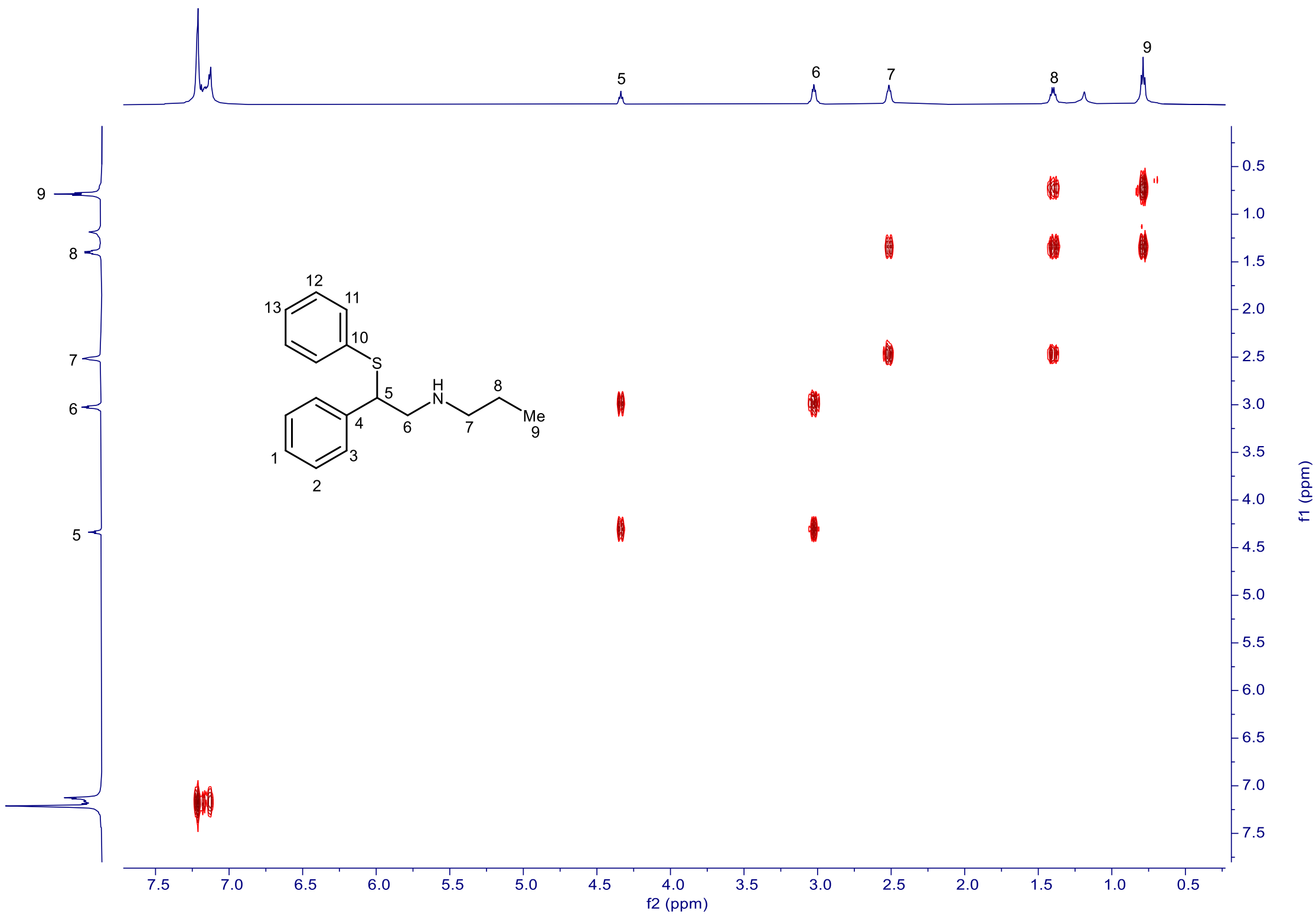

COSY NMR spectrum (600 MHz, $CDCl_3$, at rt) of **1r**

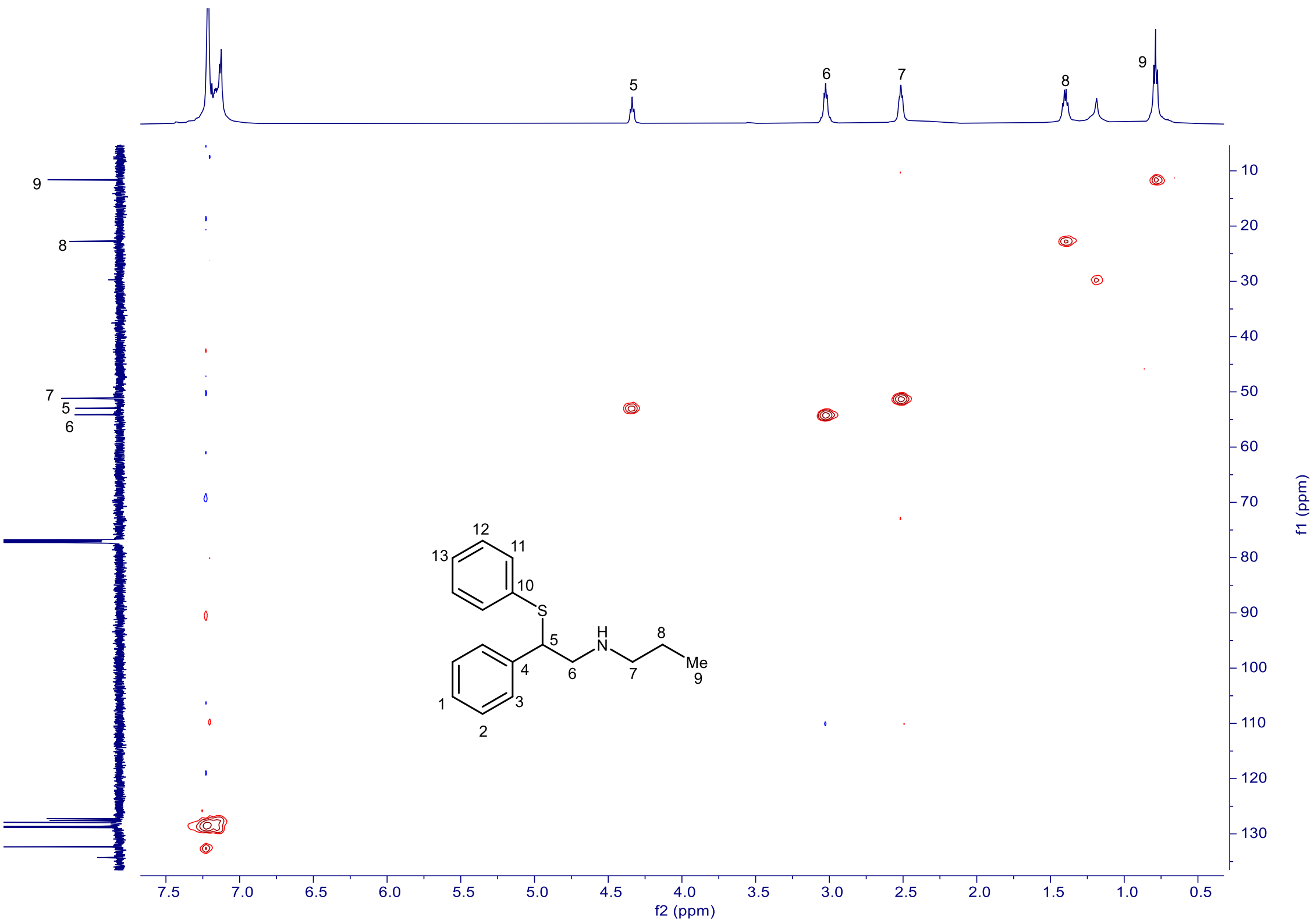

HSQC NMR spectrum (600 MHz, $CDCl_3$, at rt) of **1r**

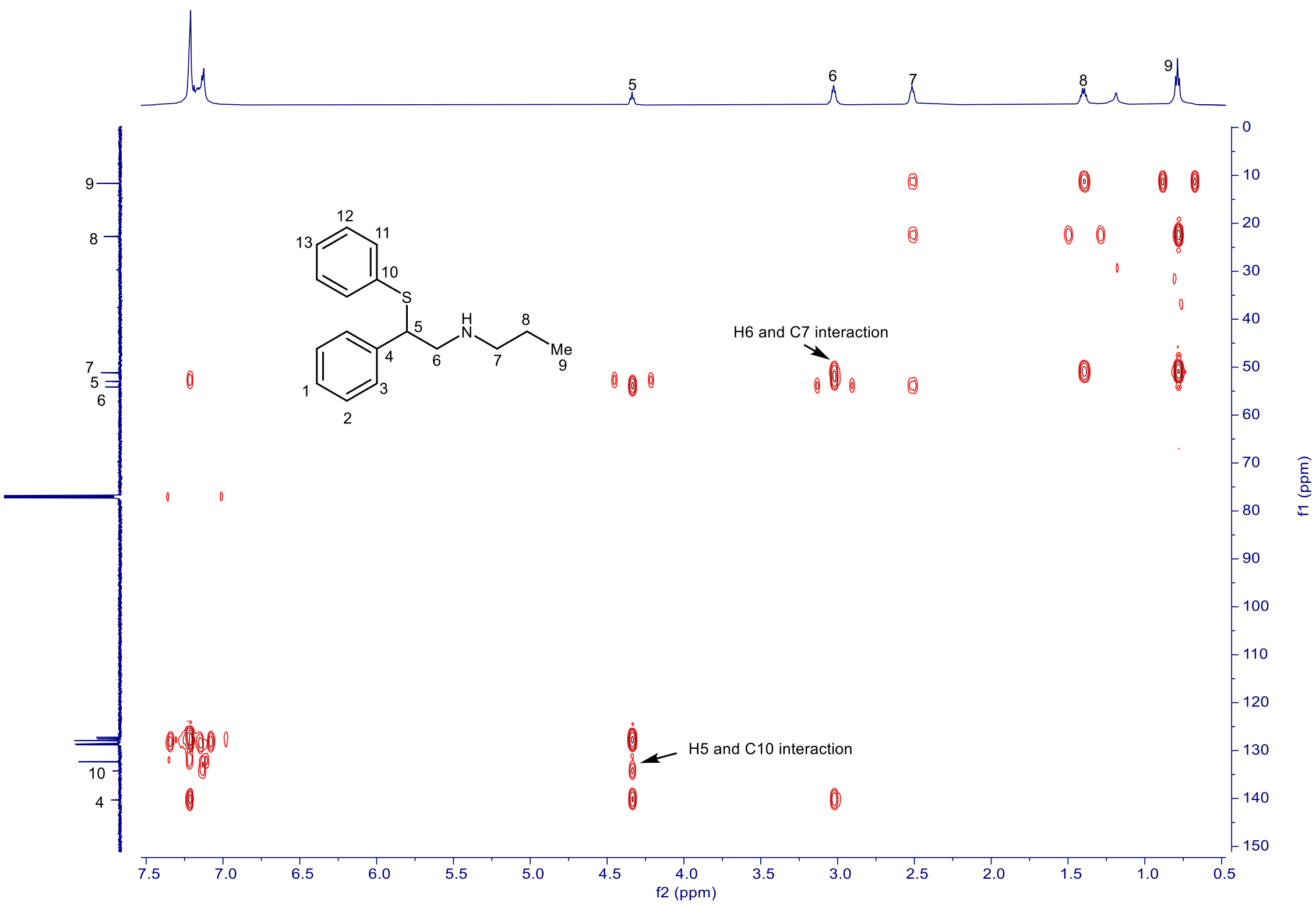

HMBC NMR spectrum (600 MHz, $CDCl_3$, at rt) of **1r**

N-(2-(Bicyclo[4.2.0]octa-1(6),2,4-trien-3-yl)-2-(phenylthio)ethyl)propan-1-amine (7d)

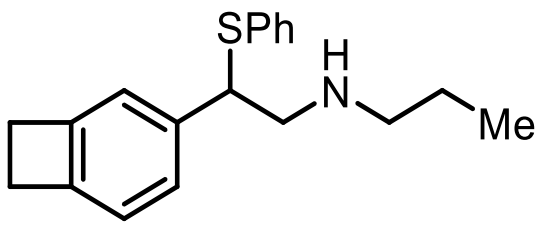

Following **GP7**, *trans*-aziridine borane **7a** (40 mg, 0.2 mmol) and diphenyl disulfide (22 mg, 0.1 mmol) gave **7d** (73%) as an oil. R_f 0.42 [*n*-pentane:EtOAc (6:4)]. ^{1}H NMR (400 MHz, $CDCl_3$) δ 7.29 – 7.21 (2H, m), 7.21 – 7.07 (3H, m), 7.04 (1H, dd, *J* = 7.6, 1.6 Hz), 6.96 (1H, d, *J* = 1.5 Hz), 6.94 – 6.84 (1H, dd, *J* = 7.7, 1.0 Hz), 4.27 (1H, t, *J* = 7.3 Hz), 3.06 (4H, s), 3.02 – 2.90 (2H, m), 2.54 – 2.38 (2H, m), 1.36 (2H, sx, *J* = 7.3 Hz), 0.78 (3H, t, *J* = 7.4 Hz); ^{13}C NMR (101 MHz, $CDCl_3$) δ 146.24, 145.22, 139.15, 135.15, 131.95, 128.88, 127.05, 126.75, 122.72, 122.04, 55.00, 53.89, 51.46, 29.49, 29.47, 23.20, 11.82; HRMS (ESI) found MH^+ 298.1615, $C_{19}H_{24}NS$ requires 298.1624.

N-(2-(2-Chlorophenyl)-2-(phenylthio)ethyl)propan-1-amine (16c)

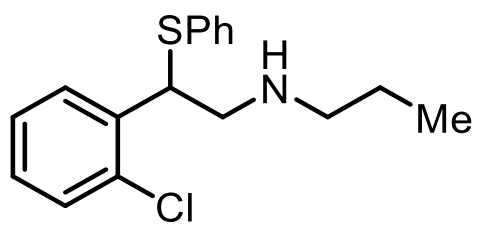

Following **GP7**, *trans*-aziridine borane **16a** (42 mg, 0.2 mmol) and diphenyl disulfide (22 mg, 0.1 mmol) gave **16c** (74%) as an oil. R_f 0.42 [*n*-pentane:EtOAc (6:4)]. ^{1}H NMR (400 MHz, $CDCl_3$) δ 7.44 (1H, dd, *J* = 7.7, 1.9 Hz), 7.35 (1H, dd, *J* = 7.8, 1.6 Hz), 7.33 – 7.27 (2H, m), 7.25 – 7.13 (5H, m), 4.99 (1H, t, *J* = 7.1 Hz), 3.18 – 2.95 (2H, m), 2.70 – 2.49 (2H, m), 1.46 (2H, sx, *J* = 7.3 Hz), 0.88 (3H, t, *J* = 7.4 Hz); ^{13}C NMR (101 MHz, $CDCl_3$) δ 138.2, 134.3, 134.0, 132.1, 129.8, 129.0, 128.9, 128.5, 127.3, 127.2, 53.8, 51.2, 49.0, 23.3, 11.8; HRMS (ESI) found MH^+ 306.1069, $C_{17}H_{21}ClNS$ requires 306.1078.

N-(2-(3-Bromophenyl)-2-(phenylthio)ethyl)propan-1-amine (37b)

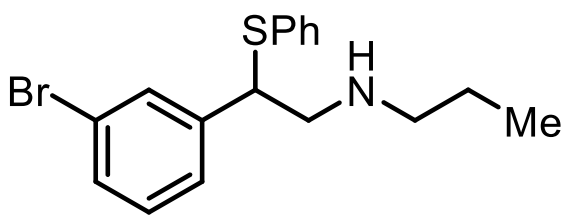

Following **GP7**, *trans*-aziridine borane **37a** (51 mg, 0.2 mmol) and diphenyl disulfide (22 mg, 0.1 mmol) gave **37b** (59%) as an oil. R_f 0.44 [*n*-pentane:EtOAc (6:4)]. ^{1}H NMR (600 MHz, $CDCl_3$) δ 7.42 (1H, t, *J* = 1.9 Hz), 7.36 (1H, ddd, *J* = 7.8, 2.0, 1.2 Hz), 7.29 – 7.25 (2H, m), 7.25 – 7.20 (3H, m), 7.19 (1H, dt, *J* = 7.8, 1.5 Hz), 7.14 (1H, t, *J* = 7.8 Hz), 4.29 (1H, t, *J* = 7.2 Hz), 3.04 (2H, d, *J* = 7.2 Hz), 2.57 (2H, t, *J* = 7.2 Hz), 1.46 (2H, sx, *J* = 7.4 Hz), 0.88 (3H, t, *J* = 7.4 Hz); ^{13}C NMR (151 MHz, $CDCl_3$) δ 143.3, 133.9, 132.7, 131.0, 130.7, 130.2, 129.0, 127.6, 126.7, 122.7, 54.3, 53.3, 51.5, 23.2, 11.8; HRMS (ESI) found MH^+ 350.0565, $C_{17}H_{21}BrNS$ requires 350.0573.

***N*-(2-(Phenylthio)-2-(*o*-tolyl)ethyl)cyclopentanamine (22c)**

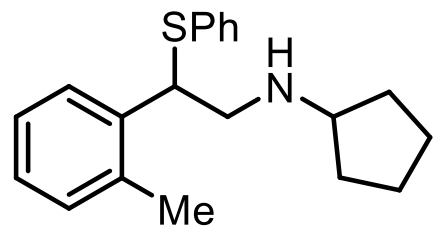

Following **GP7**, *trans*-aziridine borane **22a** (43 mg, 0.2 mmol) and diphenyl disulfide (22 mg, 0.1 mmol) gave **22c** (49%) as an oil. R_f 0.44 [*n*-pentane:EtOAc (6:4)]. ^{1}H NMR (600 MHz, $CDCl_3$) δ 7.30 – 7.26 (2H, m), 7.25 – 7.19 (4H, m), 7.17 – 7.12 (3H, m), 4.57 (1H, t, *J* = 7.3 Hz), 3.16 – 2.99 (3H, m), 2.36 (3H, s), 1.87 – 1.74 (2H, m), 1.70 – 1.60 (2H, m), 1.56 – 1.45 (2H, m), 1.34 – 1.20 (2H, m); ^{13}C NMR (151 MHz, $CDCl_3$) δ 138.3, 136.6, 134.3, 133.1, 130.7, 128.8, 127.6, 127.3, 126.7, 126.4, 59.6, 52.5, 49.1, 33.3, 24.2, 24.1, 19.6; HRMS (ESI) found MH^+ 312.1771, $C_{19}H_{26}NS$ requires 312.1781.

2-(4-Fluorophenyl)-*N*-(4-methoxyphenethyl)-2-(phenylthio)ethan-1-amine (18c)

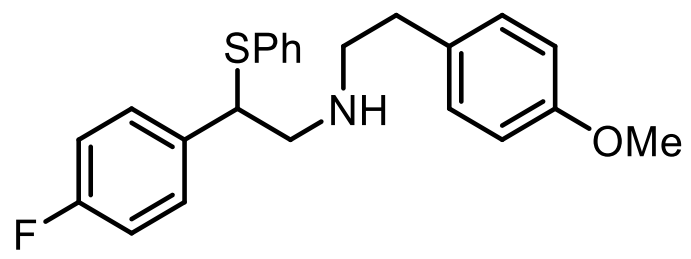

Following **GP7**, *cis*-aziridine borane **18a** and *trans*-aziridine borane **18a'** (Ratio 15:85, 57 mg, 0.2 mmol) and diphenyl disulfide (22 mg, 0.1 mmol) gave **18c** (70%) as an oil. R_f 0.31 [*n*-pentane:EtOAc (6:4)]. ^{1}H NMR (600 MHz, $CDCl_3$) δ 7.26 – 7.15 (7H, m), 7.08 – 7.01 (2H, m), 6.97 – 6.89 (2H, m), 6.85 – 6.76 (2H, m), 4.31 (1H, t, *J* = 7.3 Hz), 3.78 (3H, s), 3.10 – 3.00 (2H, m), 2.88 – 2.79 (2H, m), 2.69 (2H, t, *J* = 7.1 Hz); ^{13}C NMR (151 MHz, $CDCl_3$) δ 162.1 (d, *J* = 246.0 Hz), 158.2, 136.3 (d, *J* = 3.1 Hz), 134.1, 132.7, 131.8, 129.7, 129.5 (d, *J* = 7.9 Hz), 128.9, 127.5, 115.5 (d, *J* = 21.5 Hz), 114.0, 55.3, 54.3, 52.7, 51.0, 35.4; ^{19}F NMR (565 MHz, $CDCl_3$) δ –114.89 (tt, *J* = 8.6, 5.2 Hz); HRMS (ESI) found MH^+ 382.1637, $C_{23}H_{25}FNOS$ requires 382.1635.

***N*-Isopropyl-4-phenyl-2-(phenylthio)butan-1-amine (13d)**

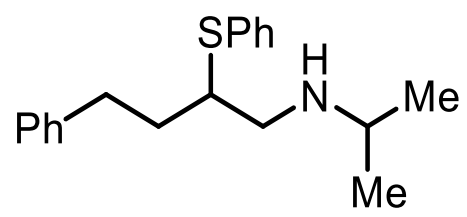

Following **GP7**, *trans*-aziridine borane **13a** (41 mg, 0.2 mmol, 2 equiv.) and diphenyl disulfide (22 mg, 0.1 mmol) gave **13d** (90%) as an oil. R_f 0.13 [*n*-pentane:EtOAc (1:1)]. ^{1}H NMR (400 MHz, $CDCl_3$) δ 7.46 – 7.39 (2H, m), 7.35 – 7.24 (5H, m), 7.24 – 7.15 (3H, m), 3.19 (1H, tt, *J* = 8.0, 5.2 Hz), 2.97 – 2.62 (5H, m), 2.03 – 1.82 (2H, m), 1.54 (NH, br s), 1.06 (3H, d, *J*=6.6 Hz), 1.04 (3H, t, *J* = 6.6 Hz); ^{13}C NMR (101 MHz, $CDCl_3$) δ 141.8, 134.2, 132.9, 129.0, 128.6, 128.5, 127.3, 126.1, 51.0, 49.5, 48.7, 35.1, 33.3, 23.2, 23.1; HRMS (ESI) found MH^+ 300.1774, $C_{19}H_{26}NS$ requires 300.1781.

The structure of **13d** was further confirmed by 2D NMR spectroscopy (COSY, HSQC, and HMBC), which also established the regioselectivity of the reaction.

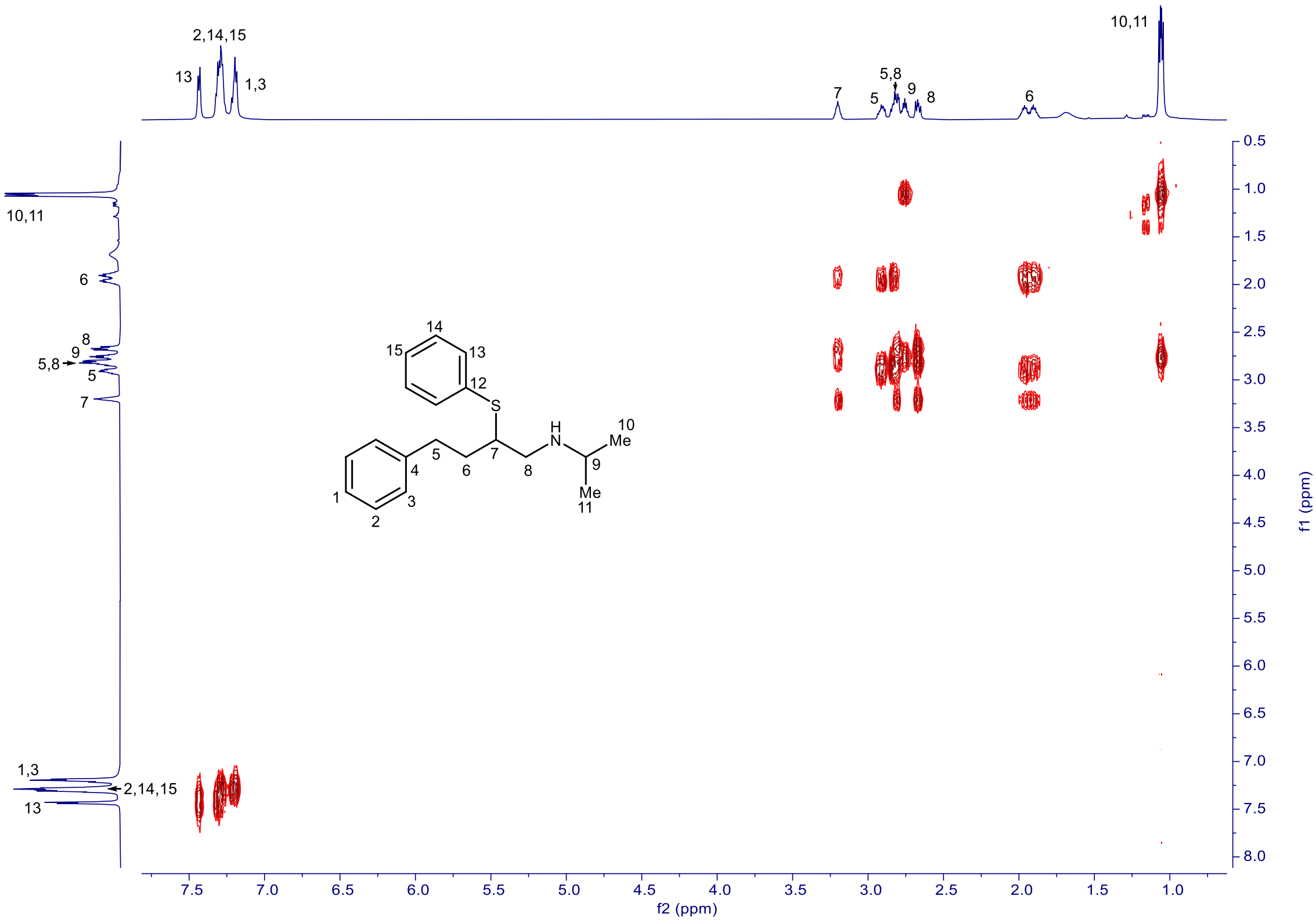

COSY NMR spectrum (600 MHz, $CDCl_3$, at rt) of **13d**

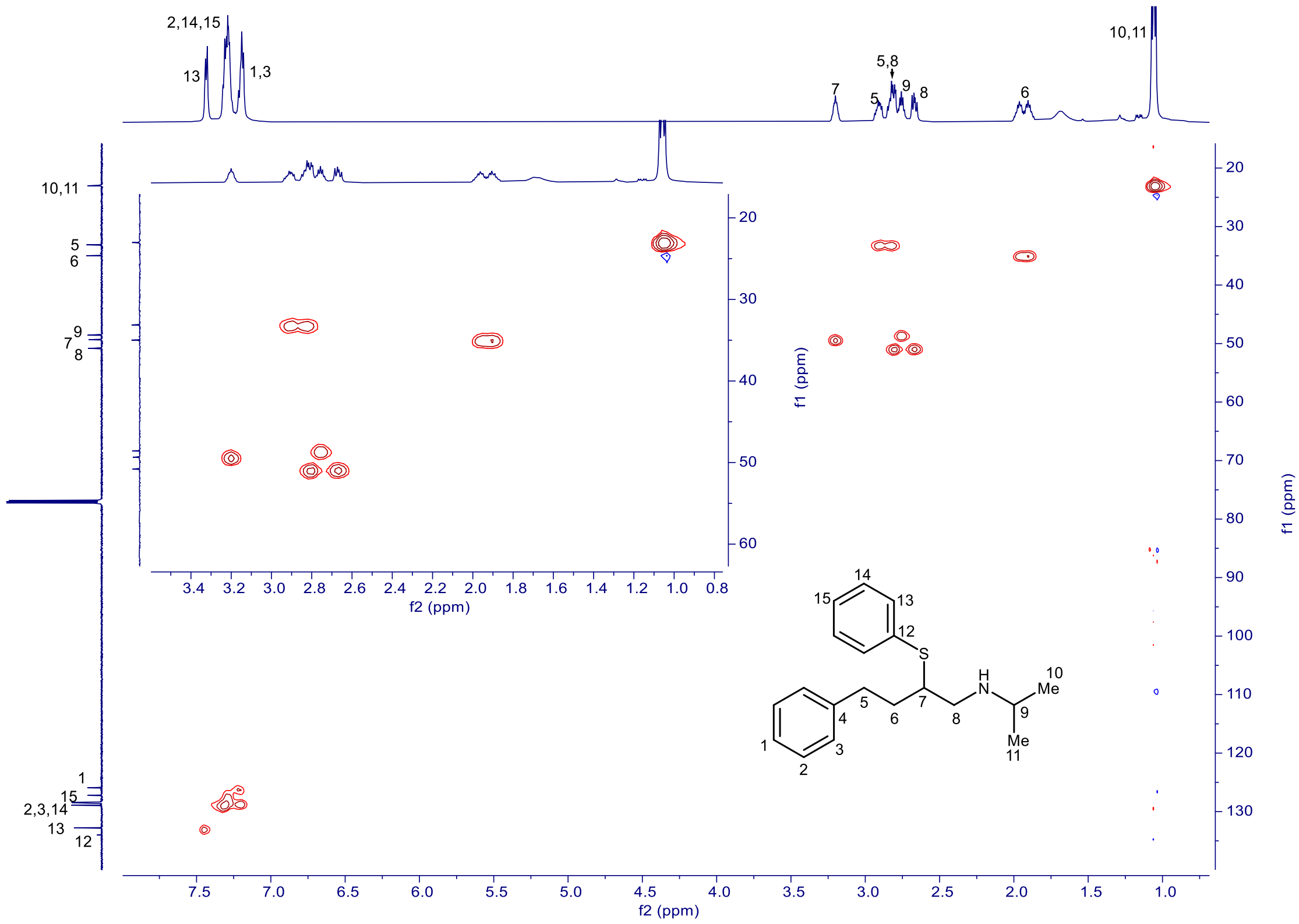

HSQC NMR spectrum (600 MHz, $CDCl_3$, at rt) of **13d**

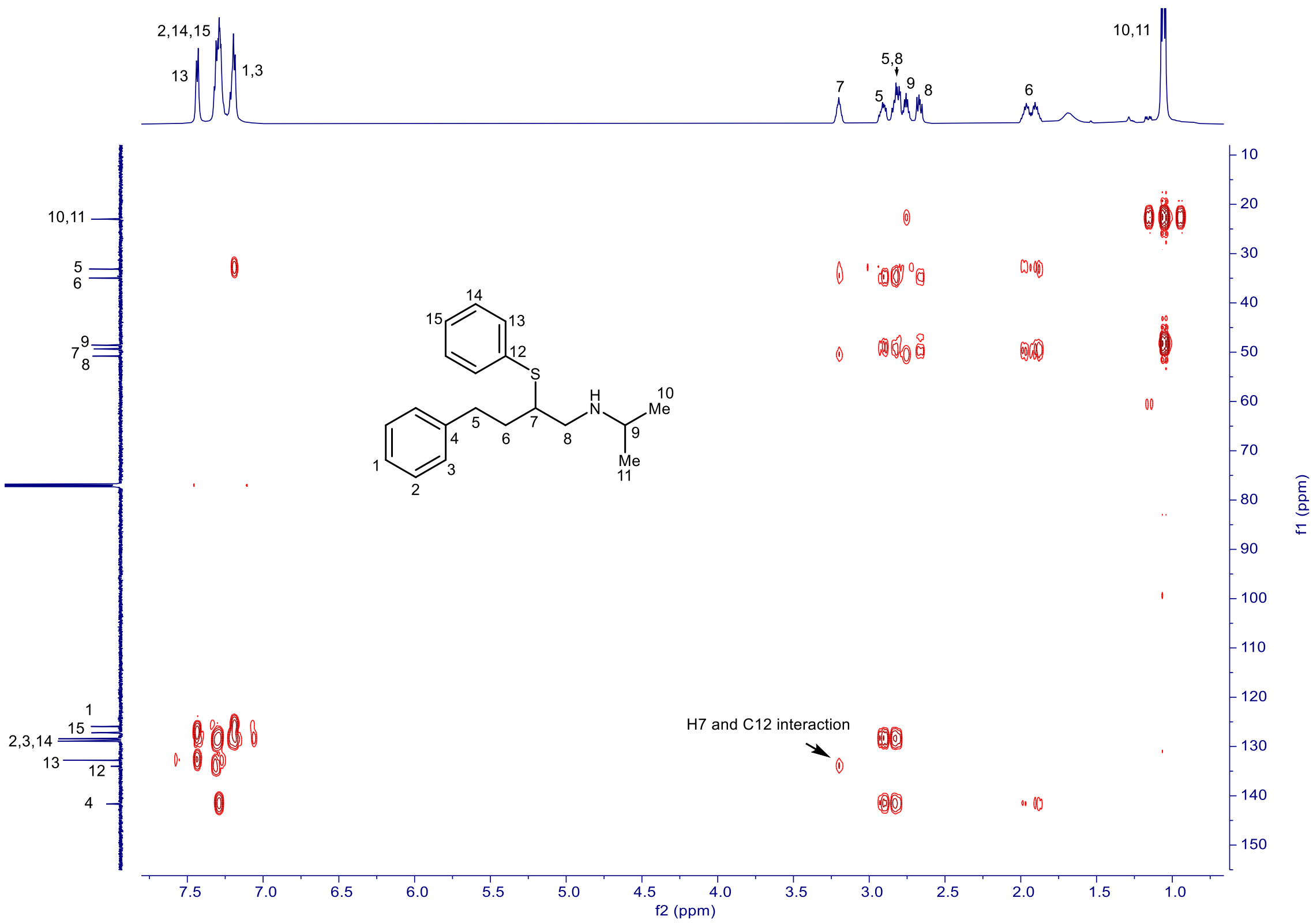

HMBC NMR spectrum (600 MHz, $CDCl_3$, at rt) of **13d**

_N_-Isopropyl-4-phenyl-2-(_p_-tolylthio)butan-1-amine (13e)

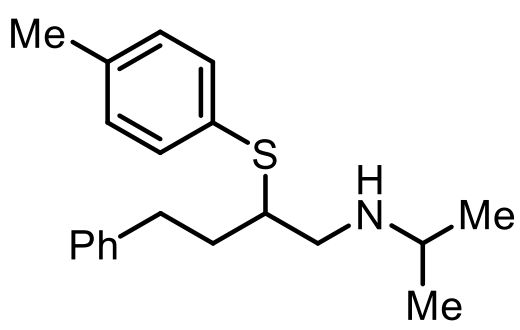

Following **GP7**, *trans*-aziridine borane **13a** (41 mg, 0.2 mmol, 2 equiv.) and *p*-tolyl disulfide (25 mg, 0.1 mmol) gave **13e** (80%) as an oil. R_f 0.32 [EtOAc]. ^1H NMR (400 MHz, $CDCl_3$) δ 7.31 (2H, d, *J*=8.1 Hz), 7.29 – 7.23 (2H, m), 7.20 – 7.14 (3H, m), 7.10 (2H, d, *J*=7.8 Hz), 3.08 (1H, tt, *J*=8.2, 5.2 Hz), 2.89 (1H, ddd, *J*=14.8, 9.2, 6.0 Hz), 2.84 – 2.69 (3H, m), 2.60 (1H, dd, *J*=12.2, 8.1 Hz), 2.33 (3H, s), 1.96 – 1.79 (2H, m), 1.65 (1H, br s), 1.04 (3H, d, *J*=6.6 Hz), 1.03 (3H, d, *J*=6.6 Hz); ^{13}C NMR (101 MHz, $CDCl_3$) δ 141.9, 137.7, 133.7, 129.9, 129.8, 128.6, 128.5, 126.0, 50.8, 49.8, 48.7, 35.0, 33.3, 23.2, 23.1, 21.2; HRMS (ESI) found MH^+ 314.1939, $C_{20}H_{28}NS$ requires 314.1937.

N-Isopropyl-2-((4-nitrophenyl)thio)-4-phenylbutan-1-amine (13f)

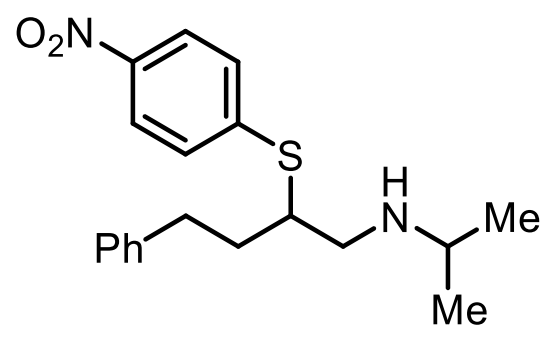

Following **GP7**, *trans*-aziridine borane **13a** (41 mg, 0.2 mmol, 2 equiv.) and 4-nitrophenyl disulfide (31 mg, 0.1 mmol) gave **13f** (64%) as an oil. R_f 0.32 [EtOAc]. ^{1}H NMR (600 MHz, $CDCl_3$) δ 8.07 (2H, d, J=8.9 Hz), 7.32 (2H, d, J=8.9 Hz), 7.29 (2H, t, J=7.5 Hz), 7.21 (1H, t, J=7.4 Hz), 7.16 (2H, d, J=7.0 Hz), 3.41 (1H, tt, J=7.3, 5.6 Hz), 2.91 (1H, dd, J=12.5, 5.2 Hz), 2.85 – 2.73 (4H, m), 2.10 (1H, ddt, J=14.7, 8.6, 6.5 Hz), 1.95 (1H, ddt, J=14.3, 8.4, 7.1 Hz), 1.44 (1H, br s), 1.04 (3H, d, J=6.1 Hz), 1.03 (3H, d, J=6.1 Hz); ^{13}C NMR (101 MHz, $CDCl_3$) δ 146.7, 145.6, 141.1, 128.7, 128.6, 128.4, 126.4, 124.0, 50.8, 48.9, 47.9, 35.0, 33.1, 23.2; HRMS (ESI) found MH^+ 345.1632, $C_{19}H_{25}N_2O_2S$ requires 345.1631.

N-methyl-5-(phenylthio)octan-4-amine (31c)

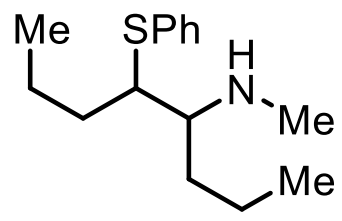

Following **GP7**, *trans*-aziridine borane **31a** (31 mg, 0.2 mmol, 2.0 equiv.) and diphenyl disulfide (22 mg, 0.1 mmol) gave **31c** (90%, dr = 1.4:1) as an oil.

<u>Data for the major diasteromer</u>: (53%), obtained as an oil, R_f 0.27 [n-pentane:EtOAc (2:3)]; ^{1}H NMR (400 MHz, $CDCl_3$) δ 7.42 (2H, d, J = 7.0 Hz), 7.32–7.26 (3H, m), 7.21 (1H, t, J = 7.3 Hz), 3.40–3.31 (1H, m), 2.49 (1H, td, J = 6.3, 2.9 Hz), 2.36 (3H, s), 1.71–1.49 (4H, m), 1.49–1.36 (3H, m), 1.39–1.25 (2H, m), 0.96–0.84 (6H, m); ^{13}C NMR (151 MHz, $CDCl_3$) δ 136.5, 131.9, 129.0, 126.8, 62.0, 53.4, 34.9, 33.4, 33.1, 21.4, 20.1, 14.4, 14.1; HRMS (ESI) found MH^+ 252.1781, $C_{15}H_{26}NS$ requires 252.1781.

<u>Data for the minor diasteromer</u>: (37%), obtained as an oil, R_f 0.17 [n-pentane:EtOAc (2:3)]; ^{1}H NMR (400 MHz, $CDCl_3$) δ 7.43–7.37 (2H, m), 7.31–7.23 (2H, m), 7.22–7.16 (1H, m), 3.22 (1H, dt, J = 8.8, 4.1 Hz), 2.53 (1H, dt, J = 6.7, 4.3 Hz), 2.39 (3H, s), 1.77–1.59 (3H, m), 1.45–1.29 (5H, m), 0.93–0.87 (6H, m); ^{13}C NMR (101 MHz, $CDCl_3$) δ 131.4, 129.0, 126.5, 62.7, 52.9, 35.0, 33.3, 33.1, 21.2, 20.0, 14.4, 14.2; HRMS (ESI) found MH^+ 252.1782, $C_{15}H_{26}NS$ requires 252.1781.

N-methyl-1,2-diphenyl-2-(phenylthio)ethan-1-amine (32c)

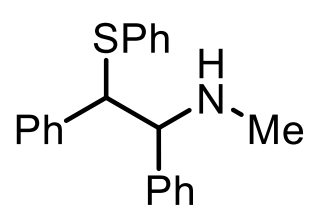

Following **GP7**, *trans*-aziridine borane **32a** (45 mg, 0.2 mmol, 2.0 equiv.) and diphenyl disulfide (22 mg, 0.1 mmol) gave **32c** (84%, dr = 1.15:1).

Data for the major diastereomer: (45%), obtained as an oil, R_f 0.28 [*n*-pentane:EtOAc (8:2)]; ^{1}H NMR (400 MHz, $CDCl_3$) δ 7.25–7.19 (2H, m), 7.21–7.04 (11H, m), 7.03–6.98 (2H, m), 4.35 (1H, d, *J* = 8.8 Hz), 3.89 (1H, d, *J* = 8.9 Hz), 2.30 (3H, s); ^{13}C NMR (101 MHz, $CDCl_3$) δ 140.2, 135.7, 134.9, 132.2, 128.9, 128.8, 128.4, 128.1, 128.0, 127.4, 127.2, 127.0, 69.8, 61.6, 34.7; HRMS (ESI) found MH^+ 320.1468, $C_{21}H_{22}NS$ requires 320.1468.

Data for the minor diastereomer: (39%), obtained as an oil, R_f 0.20 [*n*-pentane:EtOAc (8:2)]; ^{1}H NMR (400 MHz, $CDCl_3$) δ 7.31–7.27 (3H, m), 7.24–7.18 (7H, m), 7.15–7.08 (5H, m), 4.44 (1H, d, *J* = 7.2 Hz), 3.95 (1H, d, *J* = 7.3 Hz), 2.18 (3H, s); ^{13}C NMR (101 MHz, $CDCl_3$) δ 139.2, 136.1, 135.0, 132.3, 129.1, 128.7, 128.4, 128.4, 128.3, 127.9, 127.6, 127.1, 69.3, 60.7, 34.6; HRMS (ESI) found MH^+ 320.1470, $C_{21}H_{22}NS$ requires 320.1468.

trans-_N_-cyclohexyl-2-(phenylthio)cyclohexan-1-amine (33c)

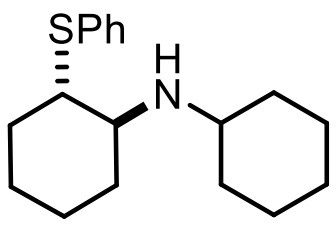

Following **GP7**, *trans*-aziridine borane **33a** (39 mg, 0.2 mmol, 2 equiv.) and diphenyl disulfide (22 mg, 0.1 mmol) gave **33c** (92%) as an oil. R_f 0.55 [EtOAc]. ^{1}H NMR (400 MHz, $CDCl_3$) δ 7.45 (2H, d, *J*=7.2 Hz), 7.34 – 7.22 (3H, m), 2.97 (1H, td, *J*=10.7, 4.0 Hz), 2.64 – 2.49 (2H, m), 2.15 – 2.06 (2H, m), 2.04 – 1.95 (1H, m), 1.93 – 1.84 (1H, m), 1.80 – 1.66 (4H, m), 1.65 – 1.58 (1H, m), 1.50 – 1.39 (1H, m), 1.34 – 1.13 (5H, m), 1.12 – 0.99 (1H, m); ^{13}C (151 MHz, $CDCl_3$) δ 134.4, 132.9, 129.0, 127.2, 57.0, 54.0, 53.7, 35.4, 33.8, 33.4, 33.1, 26.3, 26.2, 25.5, 25.3, 24.6; HRMS (ESI) found MH^+ 290.1938, $C_{10}H_{21}N_2$ requires 290.1937.

The *trans* configuration was assigned based on the coupling constants observed for peak at 2.97 (1H, td, *J* = 10.7, 4.0 Hz).

***N*-benzyl-2-(phenylthio)cyclohexan-1-amine (34c)**

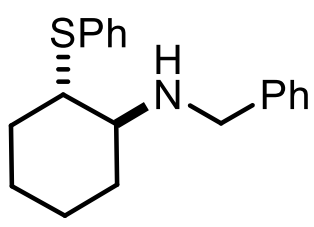

Following **GP7**, *trans*-aziridine borane **34a** (40 mg, 0.2 mmol, 2.0 equiv.) and diphenyl disulfide (22 mg, 0.1 mmol) gave **34c** (20%, dr > 10:1) as an oil, R_f 0.34 [*n*-pentane:EtOAc (3:2)]; ^{1}H NMR (400 MHz, $CDCl_3$) δ 7.31–7.26 (5H, m), 7.21–7.15 (5H, m), 3.85 (1H, d, *J* = 13.1 Hz), 3.65 (1H, d, *J* = 13.1 Hz), 2.87 (1H, ddd, *J* = 11.5, 9.9, 4.0 Hz), 2.36 (1H, td, *J* = 9.8, 4.1 Hz), 2.18–2.08 (1H, m), 2.05–1.96 (1H, m), 1.68–1.56 (2H, m), 1.39–1.26 (1H, m), 1.23–1.06 (3H, m); ^{13}C NMR (151 MHz, $CDCl_3$) δ 140.6, 133.9, 133.4, 128.9, 128.6, 128.4, 127.4, 127.1, 59.2, 53.9, 51.0, 33.6, 32.3, 26.4, 24.5; HRMS (ESI) found MH^+298.1624, $C_{10}H_{21}N_2$ requires 298.1619.

The *trans* configuration was assigned based on the coupling constants observed for peak at 2.87 (1H, ddd, J = 11.5, 9.9, 4.0 Hz).

2-Butyl-*N*-methyl-2-(phenylthio)heptan-1-amine (35c)

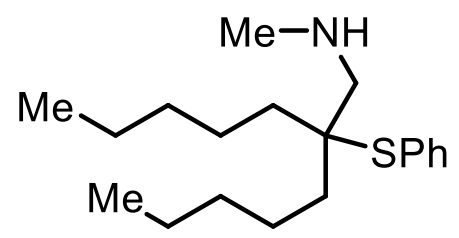

Following **GP7**, **35a** (42 mg, 0.2 mmol, 2 equiv.) and diphenyl disulfide (22 mg, 0.1 mmol) gave **33c** (59%) as an oil. R_f 0.55 [EtOAc]. ^{1}H (600 MHz, $CDCl_3$) δ 7.43 (2H, d, J=7.2 Hz), 7.38 – 7.28 (3H, m), 2.47 (3H, s), 2.37 (2H, s), 1.54 – 1.43 (4H, m), 1.39 – 1.27 (8H, m), 1.27 – 1.20 (4H, m), 0.89 (6H, t, J=7.1 Hz); ^{13}C (151 MHz, $CDCl_3$) δ 137.4, 131.2, 129.0, 128.8, 57.6, 57.1, 36.8, 34.8, 32.4, 23.3, 22.8, 14.3; HRMS (ESI) found MH^+ 308.2407, $C_{19}H_{34}NS$ requires 308.2407.

The structure of **1r** was further confirmed by 2D NMR spectroscopy (COSY, HSQC, and HMBC), which also established the regioselectivity of the reaction.

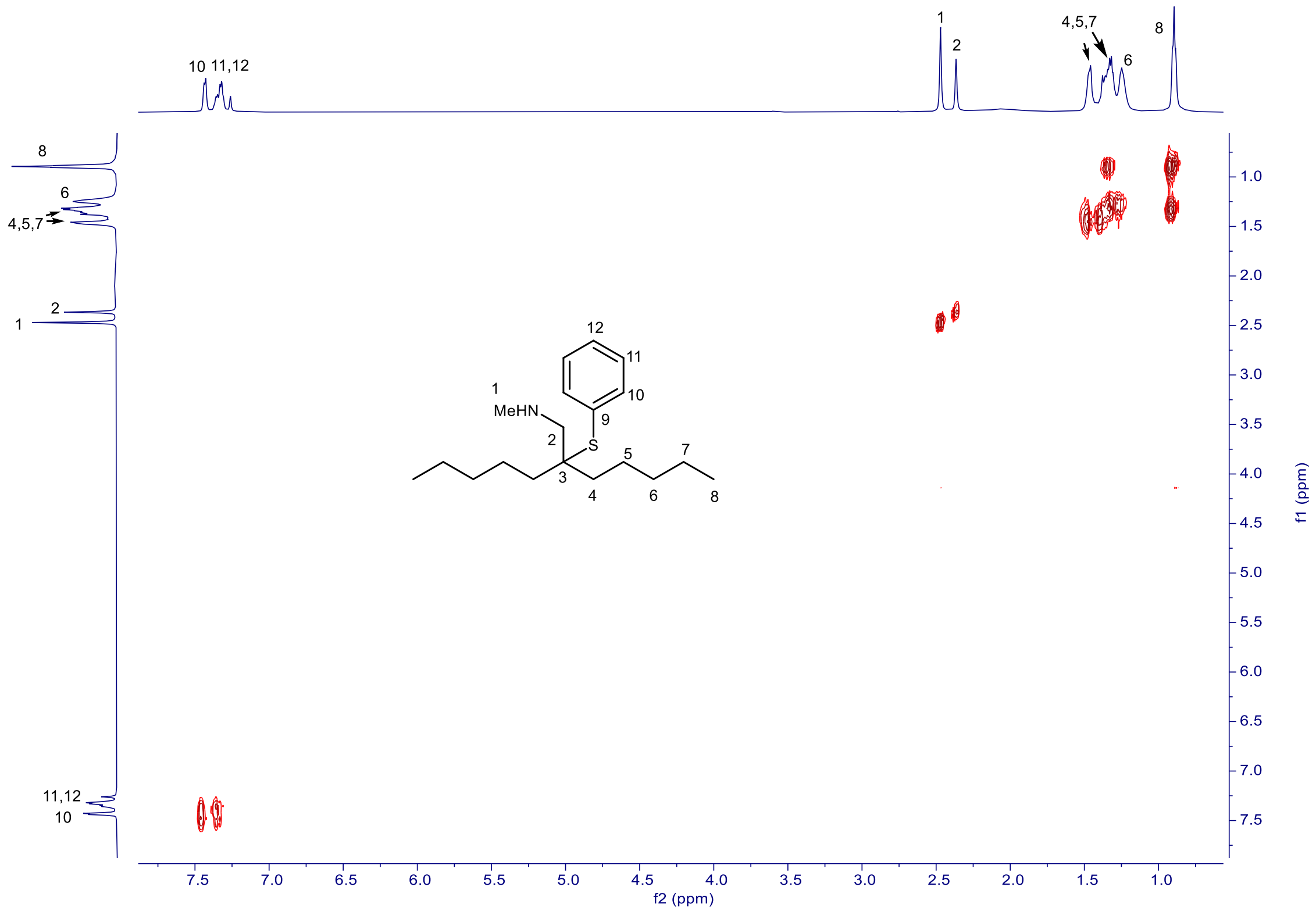

COSY NMR spectrum (600 MHz, $CDCl_3$, at rt) of **35c**

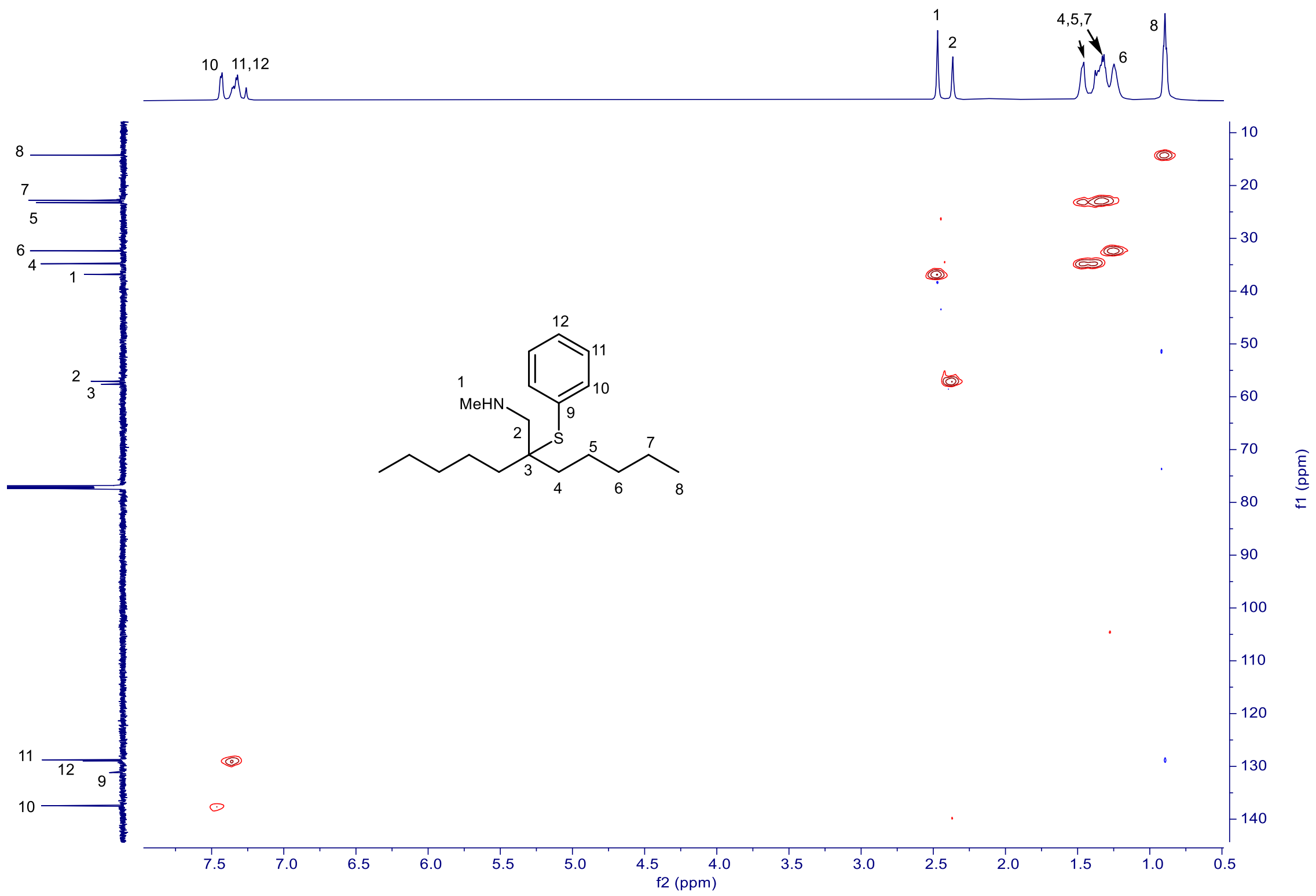

HSQC NMR spectrum (600 MHz, $CDCl_3$, at rt) of **35c**

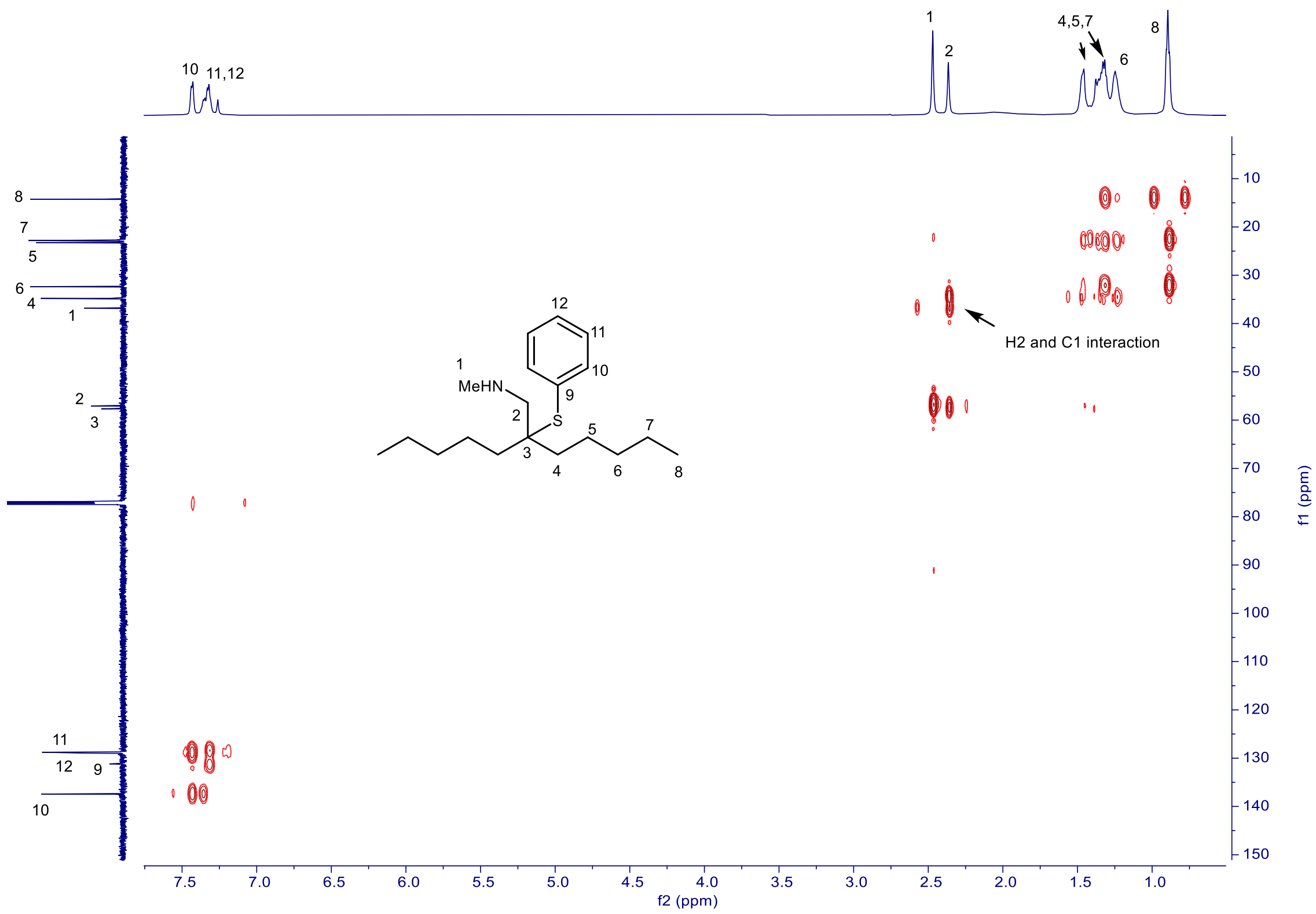

HMBC NMR spectrum (600 MHz, $CDCl_3$, at rt) of **35c**

***N*-(2-Phenyl-2-((trifluoromethyl)thio)ethyl)propan-1-amine (1s)**

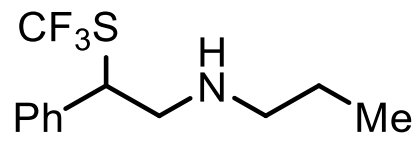

Following **GP7**, but using $AgSCF_3$ instead of diaryl disulfide and adding $NaHCO_3$ (17 mg, 0.2 mmol, 2 equiv.), *trans*-aziridine borane **1a** (35 mg, 0.2 mmol) and $AgSCF_3$ (21 mg, 0.1 mmol) gave **1s** (40%) as an oil. R_f 0.56 [*n*-pentane:EtOAc (1:1)]. ^{1}H NMR (400 MHz, $CDCl_3$) δ 7.41 – 7.26 (m, 5H), 4.52 (t, *J* = 7.1 Hz, 1H), 3.13 (d, *J* = 7.1 Hz, 2H), 2.58 (t, *J* = 7.2 Hz, 2H), 1.54 (br s, NH), 1.45 (sx, *J* = 7.4 Hz, 2H), 0.87 (t, *J* = 7.4 Hz, 3H); ^{13}C NMR (101 MHz, $CDCl_3$) δ 139.2, 130.8 (q, *J* = 308.5 Hz), 129.1, 128.3, 127.7, 54.6, 51.2, 49.4, 23.1, 11.7; ^{19}F NMR (376 MHz, $CDCl_3$) δ -39.72; HRMS (ESI) found MH^+ 264.1030, $C_{12}H_{17}F_3NS$ requires 264.1028.

5.4 Ring-Opening–Arylation via Dual Photo–Ni-Catalysis

2-(4-Fluorophenyl)-*N*-isopropyl-4-phenylbutan-1-amine (13g)

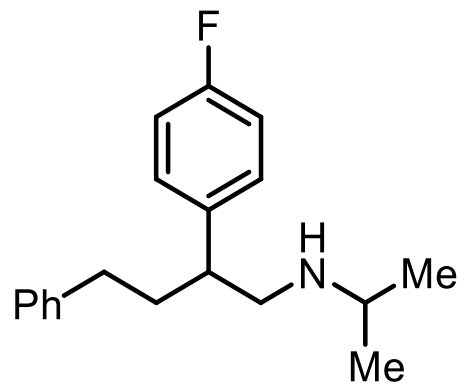

Following **GP8a**, *trans*-aziridine borane **13a** (20 mg, 0.1 mmol, 1.0 equiv.) and 1-bromo-4-fluorobenzene (22.0 μL, 0.2 mmol, 2.0 equiv.) gave **13g** (50%) as an oil. R_f 0.22 [CH_2Cl_2/MeOH/NH_3 15:1:0.01]. ^{1}H NMR (600 MHz, $CDCl_3$) δ 7.26 (2H, t, J = 7.4 Hz), 7.23 – 7.14 (3H, m), 7.11 (2H, d, J = 7.1 Hz), 7.05 (2H, t, J = 8.7 Hz), 2.90 (1H, dd, J = 11.2, 5.3 Hz), 2.84 (1H, hept, J = 6.0 Hz), 2.78 – 2.72 (2H, m), 2.52 – 2.43 (2H, m), 2.07 – 2.01 (1H, m), 1.90 – 1.83 (1H, m), 1.03 (3H, d, J = 6.3 Hz), 1.00 (3H, d, J = 6.3 Hz); ^{13}C NMR (151 MHz, $CDCl_3$) δ 161.8 (d, J = 243.7 Hz), 142.1, 139.0 (d, J = 9.5 Hz), 129.4 (d, J = 7.3 Hz), 128.4, 128.4, 125.9, 115.7 (d, J = 20.7 Hz), 53.3, 48.8, 44.9, 36.5, 33.6, 22.7, 22.6; ^{19}F NMR (564 MHz, $CDCl_3$) δ –116.4; HRMS (ESI) found MH^+ 286.1961, $C_{19}H_{25}FN$ requires 286.1965.

***N*-Isopropyl-4-phenyl-2-(4-(trifluoromethyl)phenyl)butan-1-amine (13h)**

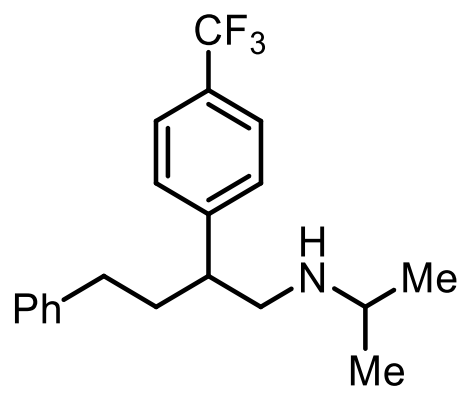

Following **GP8a**, *trans*-aziridine borane **13a** (20 mg, 0.1 mmol, 1.0 equiv.) and 1-bromo-4-(trifluoromethyl)benzene (28.0 μL, 0.2 mmol, 2.0 equiv.) gave **13h** (61%) as an oil. R_f 0.22 [CH_2Cl_2/MeOH/NH_3 15:1:0.01]. ^{1}H NMR (600 MHz, $CDCl_3$) δ 7.62 (2H, d, J = 8.2 Hz), 7.36 (2H, d, J = 8.2 Hz), 7.26 (2H, t, J = 6.0 Hz), 7.17 (1H, t, J = 7.4 Hz), 7.10 (2H, d, J = 6.9 Hz), 2.97 – 2.93 (2H, m), 2.85 – 2.81 (1H, m), 2.77 (1H, hept, J = 6.2 Hz), 2.47 (2H, t, J = 6.9 Hz), 2.14 – 2.09 (1H, m), 1.95 – 1.89 (1H, m), 1.05 (3H, d, J = 6.3 Hz), 1.03 (3H, d, J = 6.3 Hz); ^{13}C NMR (151 MHz, $CDCl_3$) δ 147.6, 141.8, 129.2 (q, J = 31.1 Hz), 128.5, 128.5, 128.4, 126.0, 125.8 (q, J = 3.7 Hz), 124.4 (q, J = 271.8 Hz), 52.7, 49.0, 45.5, 36.1, 33.6, 22.5, 22.3; ^{19}F NMR (564 MHz, $CDCl_3$) δ –62.4; HRMS (ESI) found MH^+ 336.1926, $C_{20}H_{25}F_3N$ requires 336.1934.

4-(1-(Isopropylamino)-4-phenylbutan-2-yl)benzonitrile (13i)

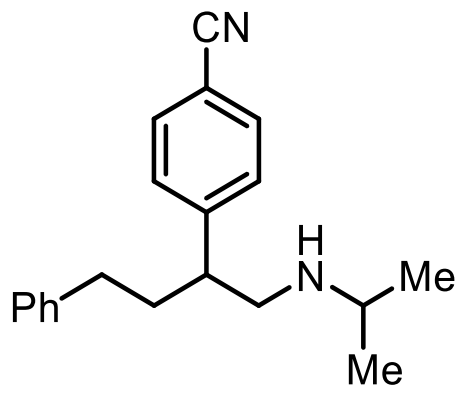

Following **GP8b**, **1331d** (19 mg, 0.1 mmol, 1.0 equiv.) and 4-bromobenzonitrile (36 mg, 0.2 mmol, 2.0 equiv.) gave **13i** (66% *over two steps*) as an oil. R_f 0.14 [EtOAc]. ^{1}H (600 MHz, $CDCl_3$) δ 7.65 – 7.61 (2H, m), 7.35 – 7.30 (2H, m), 7.27 – 7.22 (2H, m), 7.19 – 7.15 (1H, m), 7.09 – 7.05 (2H, m), 2.91 – 2.81 (2H, m), 2.80 – 2.73 (1H, m), 2.68 (1H, hp, *J*=6.3 Hz), 2.50 – 2.39 (2H, m), 2.10 – 2.01 (1H, m), 1.93 – 1.83 (1H, m), 0.98 (1H, d, *J*=6.2 Hz), 0.95 (1H, d, *J*=6.2 Hz); ^{13}C (151 MHz, $CDCl_3$) δ 149.7, 141.6, 132.6, 128.8, 128.5, 128.4, 126.1, 119.1, 110.6, 53.1, 48.7, 46.3, 36.0, 33.6, 23.0, 22.9. HRMS (ESI) found MH^+ 293.2013, $C_{20}H_{25}N_2$ requires 293.2012.

Methyl 4-(1-(Isopropylamino)-4-phenylbutan-2-yl)benzoate (13j)

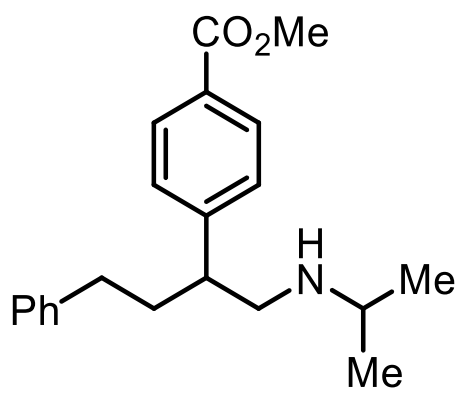

Following **GP8a**, *trans*-aziridine borane **13a** (20 mg, 0.1 mmol, 1.0 equiv.) and methyl 4-bromobenzoate (43 mg, 0.2 mmol, 2.0 equiv.) gave **13j** (82%) as an oil. R_f 0.18 [EtOAc]. ^{1}H NMR (600 MHz, $CDCl_3$) δ 8.04 (2H, dd, *J*=7.9, 2.8 Hz), 7.32 (2H, dd, *J*=7.9, 2.8 Hz), 7.27 (2H, t, *J*=8.0 Hz), 7.18 (1H, t, *J*=7.7 Hz), 7.10 (2H, d, *J*=7.5 Hz), 3.94 (3H, s), 2.94 – 2.85 (2H, m), 2.83 – 2.76 (1H, m), 2.74 – 2.68 (1H, m), 2.53 – 2.42 (2H, m), 2.09 – 2.02 (1H, m), 1.96 – 1.89 (1H, m), 1.02 – 0.92 (6H, m); ^{13}C NMR (151 MHz, $CDCl_3$) δ 167.2, 149.4, 142.0, 130.1, 128.8, 128.5, 128.1, 126.0, 53.3, 52.2, 48.7, 46.1, 36.3, 33.7, 23.0, 22.9; HRMS (ESI) found MH^+ 326.2102, $C_{21}H_{28}NO_2$ requires 326.2115.

The structure of **13j** was further confirmed by 2D NMR spectroscopy (COSY, HSQC, and HMBC), which also established the regioselectivity of the reaction.

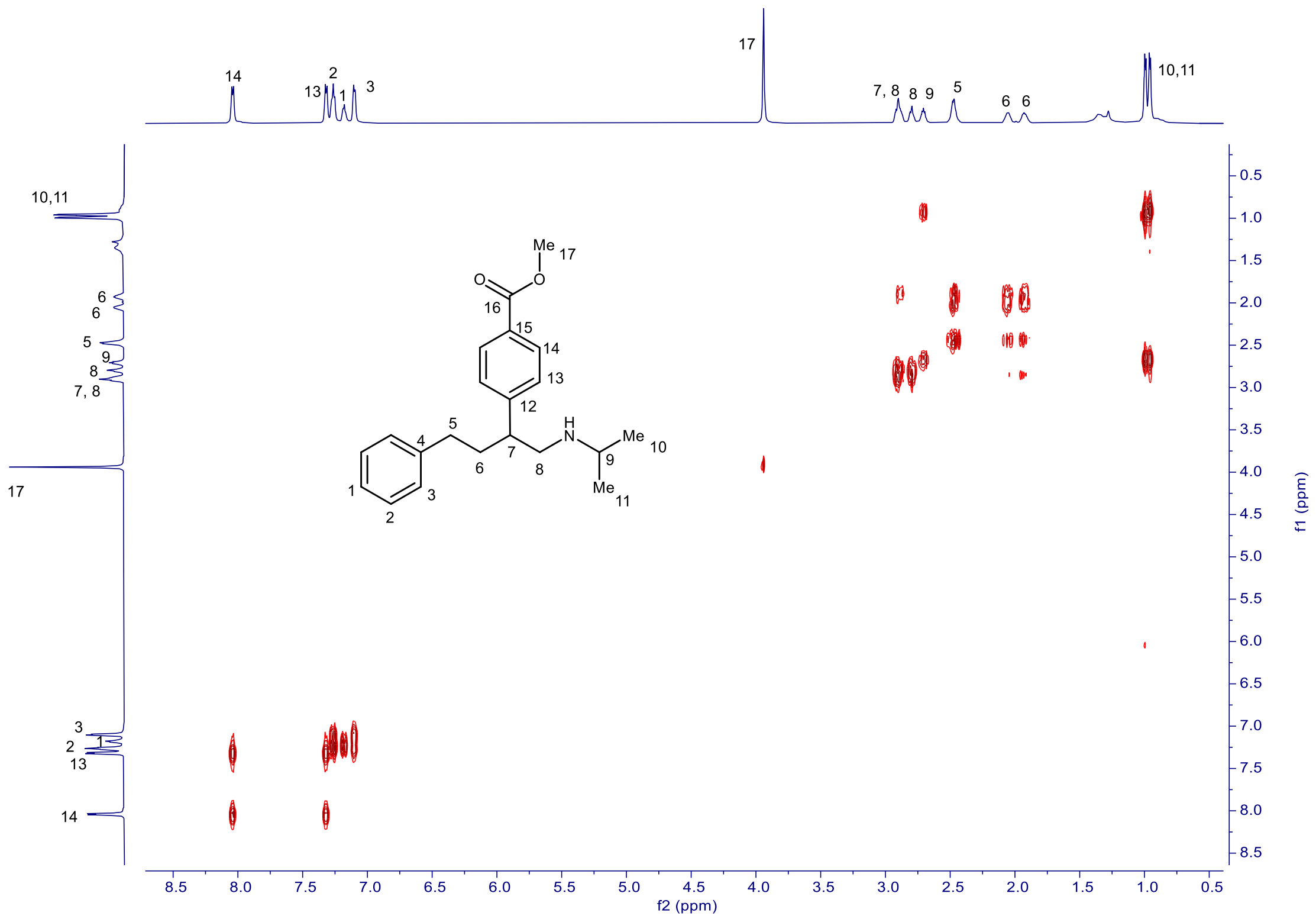

COSY NMR spectrum (600 MHz, $CDCl_3$, at rt) of **13j**

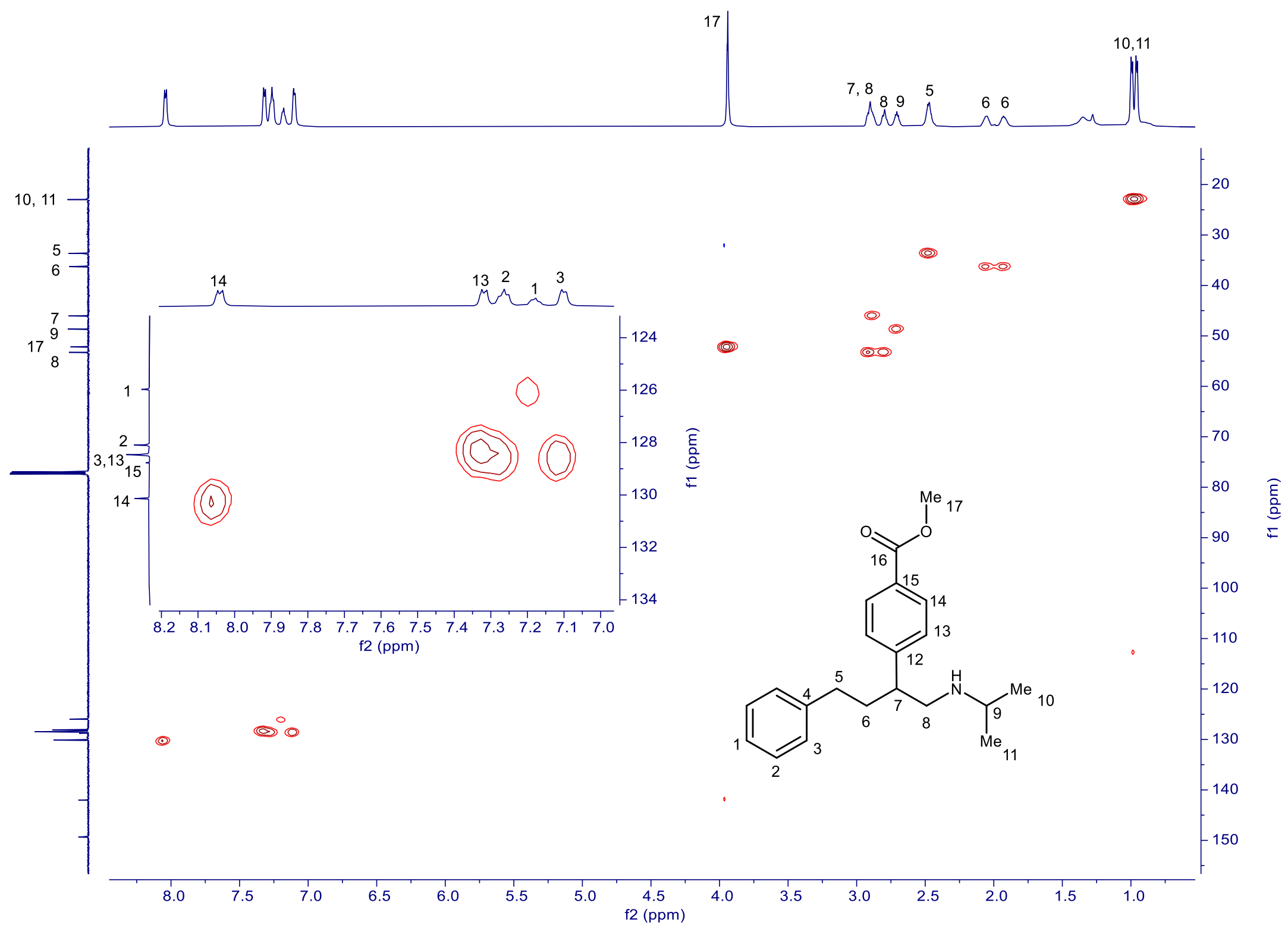

HSQC NMR spectrum (600 MHz, $CDCl_3$, at rt) of **13j**

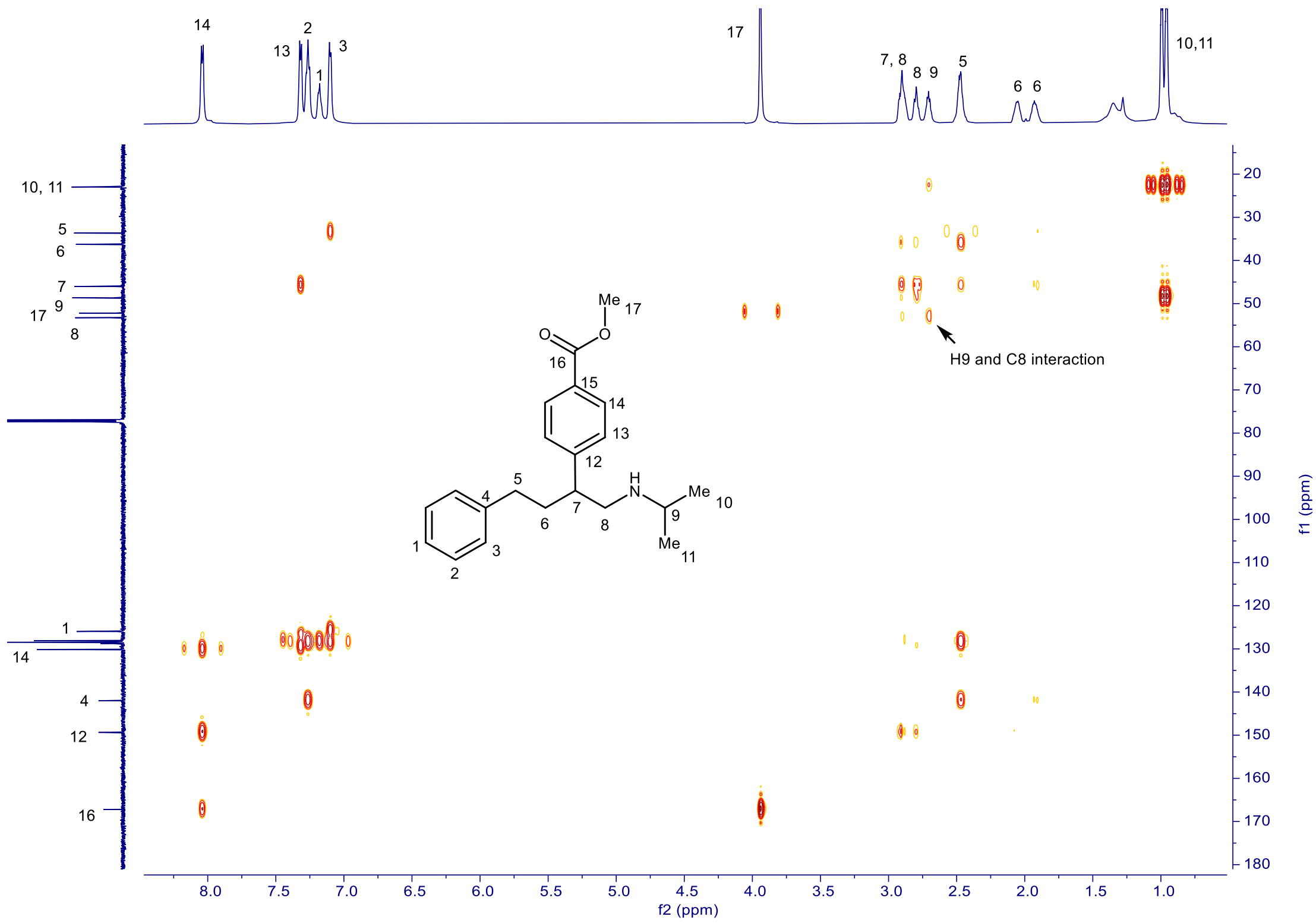

HMBC NMR spectrum (600 MHz, $CDCl_3$, at rt) of **13j**

2-([1,1'-biphenyl]-4-yl)-*N*-isopropyl-4-phenylbutan-1-amine (13k)

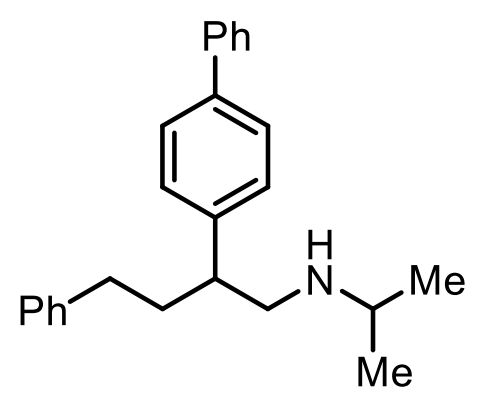

Following **GP8a**, *trans*-aziridine borane **13a** (20 mg, 0.1 mmol, 1.0 equiv.) and 4-bromo-1,1'-biphenyl (47 mg, 0.2 mmol, 2.0 equiv.) gave **13k** (51%) as an oil. R_f 0.32 [DCM:MeOH (15:1)]. ^{1}H NMR (600 MHz, $CDCl_3$) δ 7.58 – 7.47 (4H, m), 7.37 (2H, t, *J*=7.4 Hz), 7.31 – 7.14 (5H, m), 7.11 – 7.01 (3H, m), 2.96 – 2.81 (2H, m), 2.79 – 2.66 (2H, m), 2.50 – 2.38 (2H, m), 1.99 (1H, dtd, *J*=14.0, 10.2, 5.1 Hz), 1.91 – 1.82 (1H, m), 0.96 (3H, d, *J*=6.3 Hz), 0.94 (3H, d, *J*=6.3 Hz); ^{13}C NMR (151 MHz, $CDCl_3$) δ 142.3, 142.3, 141.0, 139.7, 128.9, 128.5, 128.4, 128.4, 127.6, 127.3, 127.1, 125.9, 53.1, 48.9, 45.2, 36.4, 33.7, 22.6, 22.4; HRMS (ESI) found MH^+ 344.2372, $C_{25}H_{30}N$ requires 344.2373.

N-isopropyl-4-phenyl-2-(p-tolyl)butan-1-amine (13l)

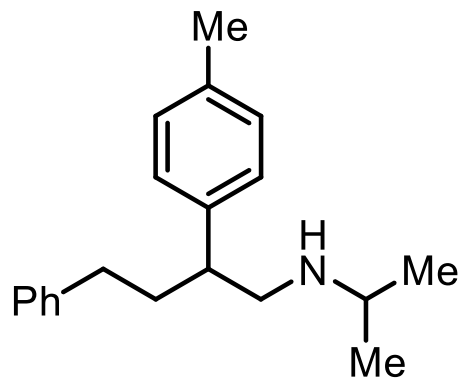

Following **GP8a**, *trans*-aziridine borane **13a** (20 mg, 0.1 mmol, 1.0 equiv.) and 1-bromo-4-methylbenzene (43 mg, 0.25 mmol, 2.5 equiv.) gave **13l** (51%) as an oil. R_f 0.31 [DCM:MeOH (15:1)]. ^{1}H NMR (400 MHz, $CDCl_3$) δ 7.26 (2H, t, 7.7 Hz), 7.21 – 7.05 (7H, m), 2.94 (1H, dd, *J*=10.6, 5.0 Hz), 2.86 (1H, tt, *J*=9.5, 4.6 Hz), 2.82 – 2.71 (2H, m), 2.56 – 2.43 (2H, m), 2.37 (3H, s), 2.12 – 1.99 (1H, m), 1.96 – 1.82 (1H, m), 1.11 – 0.97 (6H, m); ^{13}C NMR (101 MHz, $CDCl_3$) δ 142.4, 140.0, 136.4, 129.6, 128.5, 128.4, 127.9, 125.8, 53.0, 48.9, 45.0, 36.5, 33.7, 22.5, 22.3, 21.2; HRMS (ESI) found MH^+ 282.2214, $C_{20}H_{28}N$ requires 282.2216.

2-(4-(*tert*-butyl)phenyl)-N-isopropyl-4-phenylbutan-1-amine (13m)

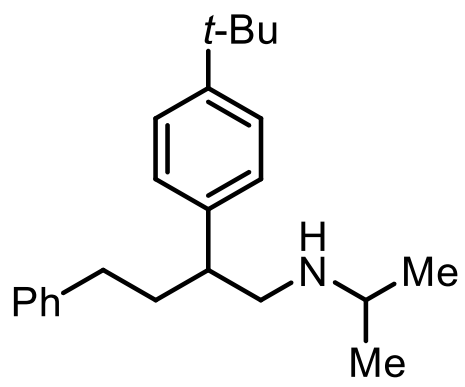

Following **GP8a**, *trans*-aziridine borane **13a** (20 mg, 0.1 mmol, 1.0 equiv.) and 1-bromo-4-(*tert*-butyl)benzene (35 mg, 0.2 mmol, 2.0 equiv.) gave **13m** (58%) as an oil. R_f 0.46 [DCM:MeOH (10:1)]. ^{1}H (600 MHz, $CDCl_3$) δ 7.3 (1H, d, *J*=8.4 Hz), 7.19 – 7.14 (2H, m), 7.11 – 6.97 (5H, m), 2.85 (1H, dd, *J*=11.2, 5.5 Hz), 2.78 (1H, tt, *J*=9.9, 5.1 Hz), 2.73 – 2.82 (2H, m), 2.47 – 2.35 (2H, m), 2.00 – 1.91 (1H, m), 1.82 (1H, dtd, *J*=13.2, 9.3, 6.1 Hz), 1.25 (9H, s), 0.96 (3H, d, *J*=6.3 Hz), 0.94 (3H, d, *J*=6.3 Hz); ^{13}C (151 MHz, $CDCl_3$) δ 149.6, 142.5, 139.9, 128.5, 128.4, 127.6, 125.8, 125.7, 52.9, 48.9, 44.8, 36.4, 34.6, 33.8, 31.5, 22.5, 22.2; HRMS (ESI) found MH^+ 324.2677, $C_{23}H_{34}N$ requires 324.2686.

N-isopropyl-4-phenyl-2-(4-(trifluoromethoxy)phenyl)butan-1-amine (13o)

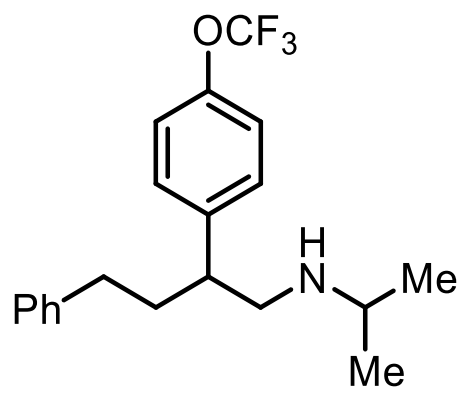

Following **GP8a**, *trans*-aziridine borane **13a** (20 mg, 0.1 mmol, 1.0 equiv.) and 1-bromo-4-(trifluoromethoxy)benzene (48 mg, 0.2 mmol, 2.0 equiv.) gave **13o** (76%) as an oil. R_f 0.32 [DCM:MeOH (15:1)]. ^{1}H NMR (600 MHz, $CDCl_3$) δ 7.29 – 7.22 (4H, m), 7.21 – 7.12 (3H, m),

7.09 (2H, d, *J*=7.4 Hz), 2.90 (1H, dd, *J*=11.1, 5.3 Hz), 2.88 – 2.81 (1H, m), 2.81 – 2.70 (2H, m), 2.54 – 2.41 (2H, m), 2.09 – 2.01 (1H, m), 1.87 (1H, dtd, *J*=14.7, 9.3, 5.7 Hz), 1.01 (3H, d, *J*=6.2 Hz), 0.98 (3H, d, *J*=6.3 Hz); ^{13}C NMR (151 MHz, $CDCl_3$) δ 148.1, 142.2, 142.0, 129.2, 128.5, 128.5, 126.0, 121.3, 120.7 (q, *J*=257.0 Hz), 53.2, 48.9, 45.1, 36.3, 33.6, 22.7, 22.6; ^{19}F NMR (564 MHz, $CDCl_3$) δ -57.84; HRMS (ESI) found MH^+ 352.1881, $C_{20}H_{25}F_3NO$ requires 352.1883.

2-(2-Fluorophenyl)-*N*-isopropyl-4-phenylbutan-1-amine (13p)

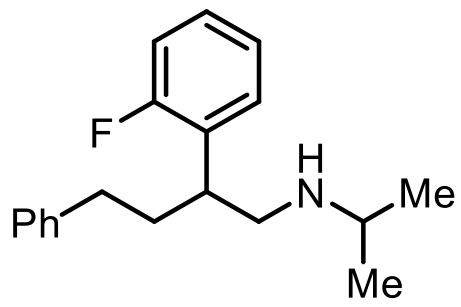

Following **GP8a**, *trans*-aziridine borane **13a** (20 mg, 0.1 mmol, 1.0 equiv.) and 1-bromo-2-fluorobenzene (21.8 μL, 0.2 mmol, 2.0 equiv.) gave **13p** (44%) as an oil. R_f 0.22 [CH_2Cl_2/MeOH/NH_3 15:1:0.01]. ^{1}H NMR (600 MHz, $CDCl_3$) δ 7.27 – 7.22 (4H, m), 7.18 – 7.12 (4H, m), 7.07 (1H, t, *J* = 9.0 Hz), 3.22 – 3.17 (1H, m), 2.92 – 2.85 (2H, m), 2.75 (1H, hept, *J* = 6.3 Hz), 2.52 (2H, t, *J* = 6.6 Hz), 2.09 – 2.03 (1H, m), 2.00 – 1.93 (1H, m), 1.00 (3H, d, *J* = 6.7 Hz), 0.99 (3H, d, *J* = 6.7 Hz); ^{13}C NMR (151 MHz, $CDCl_3$) δ 161.6 (d, *J* = 244.7 Hz), 142.2, 129.9 (d, *J* = 14.5 Hz), 129.2 (d, *J* = 5.0 Hz), 128.5, 128.4, 128.2 (d, *J* = 8.3 Hz), 125.9, 124.5 (d, *J* = 3.6 Hz), 115.8 (d, *J* = 23.1 Hz), 51.7, 48.7, 39.2, 35.2, 33.8, 22.6, 22.5; ^{19}F NMR (564 MHz, $CDCl_3$) δ –117.5; HRMS (ESI) found MH^+ 286.1961, $C_{19}H_{25}FN$ requires 286.1965.

***N*-Isopropyl-4-phenyl-2-(4-(pyrrolidin-1-ylsulfonyl)phenyl)butan-1-amine (13q)**

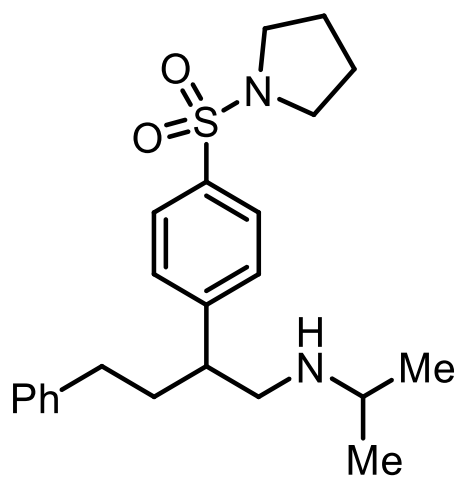

Following **GP8a**, *trans*-aziridine borane **13a** (20 mg, 0.1 mmol, 1.0 equiv.) and 1-((4-bromophenyl)sulfonyl)pyrrolidine (58 mg, 0.2 mmol, 2.0 equiv.) gave **13q** (62%) as a syrup. R_f 0.22 [CH_2Cl_2/MeOH/NH_3 15:1:0.01]. ^{1}H NMR (600 MHz, $CDCl_3$) δ 7.81 (2H, d, *J* = 7.9 Hz), 7.39 (2H, d, *J* = 7.9 Hz), 7.26 (2H, t, *J* = 7.5 Hz), 7.17 (2H, t, *J* = 7.4 Hz), 7.10 (2H, d, *J* = 7.5 Hz), 3.30 – 3.27 (4H, m), 2.95 – 2.91 (2H, m), 2.84 – 2.80 (1H, m), 2.73 (1H, hept, *J* = 6.2 Hz), 2.48 (2H, t, *J* = 7.9 Hz), 2.13 – 2.07 (1H, m), 1.96 – 1.89 (1H, m), 1.79 – 1.77 (4H, m), 1.02 (3H, d, *J* = 6.3 Hz), 0.99 (3H, d, *J* = 6.3 Hz); ^{13}C NMR (151 MHz, $CDCl_3$) δ 149.0, 141.7, 135.6, 128.7, 128.5, 128.4, 128.0, 126.1, 52.9, 48.9, 48.1, 45.8, 36.0, 33.7, 25.4, 22.8, 22.6; HRMS (ESI) found MH^+ 401.2247, $C_{23}H_{33}N_2O_2S$ requires 401.2257.

N-Isopropyl-2-(naphthalen-2-yl)-4-phenylbutan-1-amine (13r)

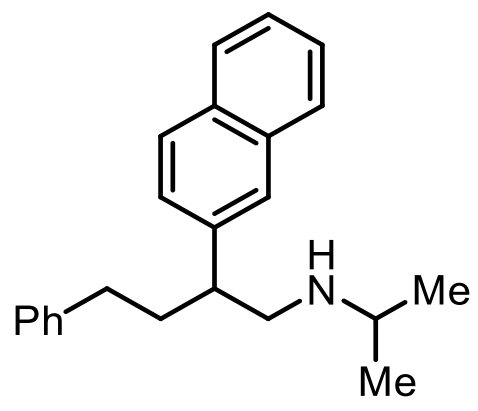

Following **GP8b**, **13** (19 mg, 0.1 mmol, 1.0 equiv.) and 2-bromonaphthalene (41 mg, 0.2 mmol, 2.0 equiv.) gave **13r** (45% *over two steps*) as an oil. R_f 0.14 [EtOAc]. ^{1}H (600 MHz, $CDCl_3$) δ 7.88 – 7.81 (3H, m), 7.68 (1H, d, *J*=1.7 Hz), 7.52 – 7.44 (2H, m), 7.39 (1H, dd, *J*=8.5, 1.7 Hz), 7.28 – 7.22 (2H, m), 7.19 – 7.13 (1H, m), 7.13 – 7.08 (2H, m), 3.03 – 2.93 (2H, m), 2.90 – 2.83 (1H, m), 2.71 (1H, hp, *J*=6.2 Hz), 2.54 – 2.45 (2H, m), 2.13 – 1.96 (2H, m), 0.98 (3H, d, *J*=6.2 Hz), 0.93 (3H, d, *J*=6.2 Hz); ^{13}C (151 MHz, $CDCl_3$) δ 142.4, 141.0, 133.7, 132.7, 128.7, 128.5, 128.4, 127.8, 127.3, 126.2, 125.9, 125.6, 125.6, 53.4, 48.7, 46.1, 36.5, 33.8, 23.0, 22.9. HRMS (ESI) found MH^+ 318.2212, $C_{23}H_{28}N$ requires 318.2216.

5-(1-(Isopropylamino)-4-phenylbutan-2-yl)-2-methylisoindoline-1,3-dione (13s)

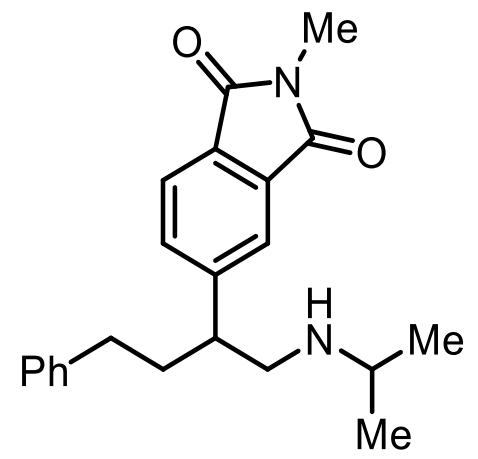

Following **GP8b**, **13** (19 mg, 0.1 mmol, 1.0 equiv.) and 5-bromo-2-methylisoindoline-1,3-dione (48 mg, 0.2 mmol, 2.0 equiv.) gave **13s** (50% *over two steps*) as an oil R_f 0.14 [EtOAc]. ^{1}H (600 MHz, $CDCl_3$) δ 7.73 (1H, d, *J*=7.6 Hz), 7.65 (1H, s), 7.46 (1H, dd, *J*=7.6, 1.5 Hz), 7.20 – 7.15 (2H, m), 7.09 (1H, t, *J*=7.4 Hz), 7.00 (2H, d, *J*=7.5 Hz), 3.11 (3H, s), 2.91 – 2.81 (2H, m), 2.74 (1H, dd, *J*=11.2, 8.3 Hz), 2.62 (1H, hp, *J*=6.2 Hz), 2.43 – 2.35 (2H, m), 2.07 – 1.98 (1H, m), 1.91 – 1.81 (1H, m), 0.90 (3H, d, *J*=6.2 Hz), 0.87 (3H, d, *J*=6.2 Hz); ^{13}C (151 MHz, $CDCl_3$) δ 168.8, 168.5, 151.5, 141.5, 134.1, 133.1, 130.8, 128.6, 128.4, 126.1, 123.6, 122.3, 53.2, 48.8, 46.6, 36.1, 33.6, 24.1, 23.0, 22.9. HRMS (ESI) found MH^+ 351.2064, $C_{22}H_{27}N_2O_2$ requires 351.2067.

N-Isopropyl-4-phenyl-2-(pyridin-2-yl)butan-1-amine (13t)

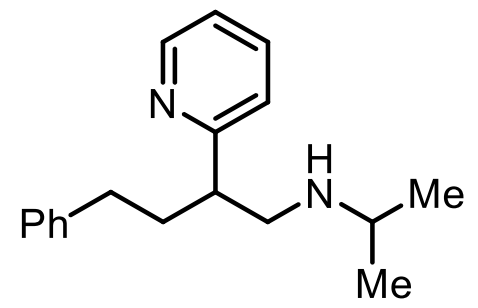

Following **GP8a**, but using 3,3',5,5'-tetrakis(trifluoromethyl)benzophenone (20 mol%) and heteroarylbromide (3 equiv.), *trans*-aziridine borane **13a** (20 mg, 0.1 mmol, 1.0 equiv.) and 2-

bromopyridine (28.7 μL, 0.3 mmol, 3.0 equiv.) gave **13t** (45%) as an oil. R_f 0.14 [CH_2Cl_2/MeOH/$NH_3$15:1:0.01]. ^{1}H NMR (600 MHz, $CDCl_3$) δ 8.60 (1H, d, *J* = 4.8 Hz), 7.63 (2H, t, *J* = 7.7 Hz), 7.24 (2H, t, *J* = 7.5 Hz), 7.16 – 7.11 (5H, m), 3.08 – 3.01 (2H, m), 2.96 – 2.91 (1H, m), 2.80 (1H, hept, *J* = 6.3 Hz), 2.49 (2H, t, *J* = 8.0 Hz), 2.14 – 2.00 (2H, m), 1.04 (3H, d, *J* = 6.3 Hz), 1.02 (3H, d, *J* = 6.3 Hz); ^{13}C NMR (151 MHz, $CDCl_3$) δ 163.1, 149.7, 142.2, 136.6, 128.5, 128.4, 125.9, 123.9, 121.8, 51.6, 49.0, 46.8, 35.3, 33.6, 22.5, 22.5; HRMS (ESI) found MH^+ 269.2009, $C_{18}H_{25}N_2$ requires 269.2012.

***N*-Isopropyl-2-(5-methylpyridin-3-yl)-4-phenylbutan-1-amine (13u)**

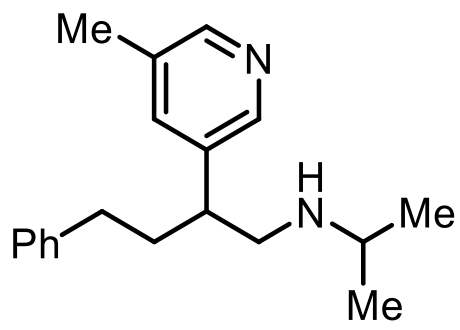

Following **GP8a**, *trans*-aziridine borane **13a** (20 mg, 0.1 mmol, 1.0 equiv.) and 3-bromo-5-methylpyridine (23.1 μL, 0.2 mmol, 2.0 equiv.) gave **13u** (60%) as an oil. R_f 0.21 [CH_2Cl_2/MeOH/NH_3 15:1:0.01]. ^{1}H NMR (600 MHz, $CDCl_3$) δ 8.24 (1H, s), 8.15 (1H, s), 7.26 (1H, s), 7.14 (2H, t, *J* = 7.5 Hz), 7.06 (1H, t, *J* = 7.4 Hz), 6.99 (2H, d, *J* = 7.6 Hz), 2.85 – 2.71 (4H, m), 2.37 (2H, t, *J* = 8.0 Hz), 2.24 (3H, s), 1.99 – 1.94 (1H, m), 1.83 – 1.77 (1H, m), 0.96 (3H, d, *J* = 6.2 Hz), 0.93 (3H, d, *J* = 6.2 Hz); ^{13}C NMR (151 MHz, $CDCl_3$) δ 148.8, 147.1, 141.7, 138.1, 136.0, 133.3, 128.5, 128.4, 126.0, 52.8, 49.1, 43.0, 36.0, 33.6, 29.8, 22.4, 18.6; HRMS (ESI) found MH^+ 283.2165, $C_{19}H_{27}N_2$ requires 283.2169.

1-(2-Fluoro-5-(1-(isopropylamino)-4-phenylbutan-2-yl)pyridin-3-yl)ethanone (13v)

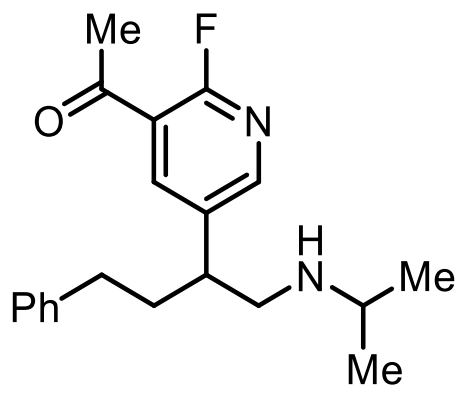

Following **GP8a**, *trans*-aziridine borane **13a** (20 mg, 0.1 mmol, 1.0 equiv.) and 3-acetyl-5-bromo-2-fluoropyridine (47 mg, 0.2 mmol, 2.0 equiv.) gave **13v** (43%) as an oil. R_f 0.22 [CH_2Cl_2/MeOH/NH_3 15:1:0.01]. ^{1}H NMR (600 MHz, $CDCl_3$) δ 8.22 (1H, s), 8.19 (1H, dd, *J* = 9.0, 2.6 Hz), 7.26 (2H, t, *J* = 8.4 Hz), 7.18 (1H, t, *J* = 7.3 Hz), 7.08 (2H, d, *J* = 7.5 Hz), 2.93 – 2.86 (2H, m), 2.81 (1H, dd, *J* = 10.8, 7.6 Hz), 2.75 – 2.68 (4H, m), 2.54 – 2.45 (2H, m), 2.16 – 2.10 (1H, m), 1.95 – 1.88 (1H, m), 1.01 (3H, d, *J* = 6.2 Hz), 0.99 (3H, d, *J* = 6.3 Hz); ^{13}C NMR (151 MHz, $CDCl_3$) δ 194.9, 160.9 (d, *J* = 243.2 Hz), 151.7 (d, *J* = 14.9 Hz), 141.3, 140.5 (d, *J* = 2.5 Hz), 138.0, 128.6, 128.4, 126.2, 119.8 (d, *J* = 28.7 Hz), 52.7, 48.9, 42.6, 35.6, 33.5, 31.2, 31.1,

22.8; ^{19}F NMR (564 MHz, $CDCl_3$) δ –65.4; HRMS (ESI) found MH^+ 329.2021, $C_{20}H_{26}FN_2O$ requires 329.2024.

_N_-Isopropyl-2-(2-methylpyridin-4-yl)-4-phenylbutan-1-amine (13w)

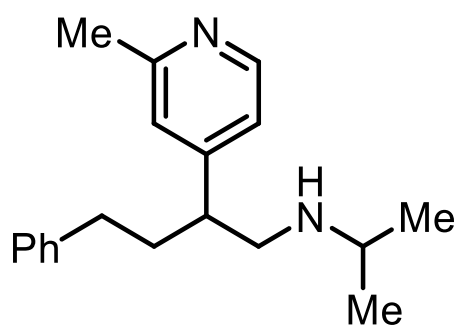

Following **GP8a**, *trans*-aziridine borane **13a** (20 mg, 0.1 mmol, 1.0 equiv.) and 4-bromo-2-methylpyridine (23.7 μL, 0.2 mmol, 2.0 equiv.) gave **13w** (60%) as an oil. R_f 0.13 [CH_2Cl_2/MeOH/NH_3 15:1:0.01]. ^{1}H NMR (600 MHz, $CDCl_3$) δ 8.44 (1H, d, *J* = 5.1 Hz), 7.26 (3H, t, *J* = 7.5 Hz), 7.17 (1H, t, *J* = 7.4 Hz), 7.10 (2H, d, *J* = 7.5 Hz), 7.03 (1H, s), 6.98 (1H, d, *J* = 5.1 Hz), 2.92 – 2.88 (1H, m), 2.83 – 2.73 (3H, m), 2.56 (3H, s), 2.48 (2H, t, *J* = 8.2 Hz), 2.08 – 2.02 (1H, m), 1.92 – 1.86 (1H, m), 1.03 (3H, d, *J* = 6.3 Hz), 1.01 (3H, d, *J* = 6.2 Hz); ^{13}C NMR (151 MHz, $CDCl_3$) δ 158.8, 153.1, 149.5, 141.7, 128.5, 128.4, 126.0, 123.1, 120.4, 52.6, 48.9, 45.3, 35.6, 33.6, 24.6, 22.7, 22.6; HRMS (ESI) found MH^+ 283.2165, $C_{19}H_{27}N_2$ requires 283.2169.

2-(2-Fluoropyridin-4-yl)-_N_-isopropyl-4-phenylbutan-1-amine (13x)

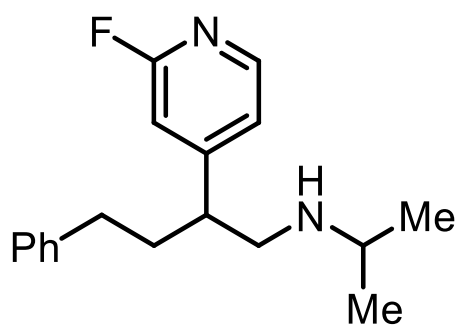

Following **GP8a**, *trans*-aziridine borane **13a** (20 mg, 0.1 mmol, 1.0 equiv.) and 4-bromo-2-fluoropyridine (20.5 μL, 0.2 mmol, 2.0 equiv.) gave **13x** (50%) as an oil. R_f 0.28 [CH_2Cl_2/MeOH/NH_3 15:1:0.01]. ^{1}H NMR (600 MHz, $CDCl_3$) δ 8.17 (1H, d, *J* = 5.1 Hz), 7.26 (2H, t, *J* = 7.5 Hz), 7.18 (1H, t, *J* = 7.4 Hz), 7.09 (2H, d, *J* = 7.5 Hz), 7.05 (1H, d, *J* = 5.1 Hz), 6.79 (1H, s), 2.89 – 2.77 (3H, m), 2.71 (1H, hept, *J* = 5.3 Hz), 2.53 – 2.44 (2H, m), 2.09 – 2.03 (1H, m), 1.92 – 1.86 (1H, m), 1.00 (3H, d, *J* = 6.3 Hz), 0.98 (3H, d, *J* = 6.2 Hz); ^{13}C NMR (151 MHz, $CDCl_3$) δ 164.4 (d, *J* = 239.0 Hz), 159.5 (d, *J* = 7.8 Hz), 147.9 (d, *J* = 15.6 Hz), 141.4, 128.6, 128.4, 126.2, 121.1 (d, *J* = 4.1 Hz), 108.9 (d, *J* = 37.2 Hz), 52.6, 48.8, 45.7, 35.5, 33.5, 22.9, 22.9; ^{19}F NMR (564 MHz, $CDCl_3$) δ –68.3; HRMS (ESI) found MH^+ 287.1914, $C_{18}H_{24}FN_2$ requires 287.1918.

N-Isopropyl-4-phenyl-2-(2-(trifluoromethyl)pyridin-4-yl)butan-1-amine (13y)

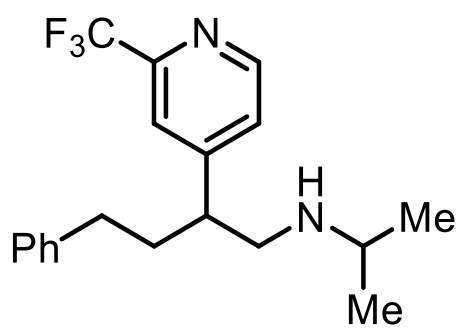

Following **GP8a**, *trans*-aziridine borane **13a** (20 mg, 0.1 mmol, 1.0 equiv.) and 4-bromo-2-(trifluoromethyl)pyridine (26.5 µL, 0.2 mmol, 2.0 equiv.) gave **13y** (58%) as an oil. R_f 0.22 [CH_2Cl_2/MeOH/NH_3 15:1:0.01]. ^{1}H NMR (600 MHz, $CDCl_3$) δ 8.66 (1H, d, *J* = 5.1 Hz), 7.53 (1H, s), 7.34 (1H, d, *J* = 3.5 Hz), 7.26 (2H, t, *J* = 7.6 Hz), 7.18 (1H, t, *J* = 7.4 Hz), 7.08 (2H, d, *J* = 7.1 Hz), 2.91 – 2.81 (3H, m), 2.71 (1H, hept, *J* = 6.2 Hz), 2.53 – 2.45 (2H, m), 2.14 – 2.09 (1H, m), 1.96 – 1.89 (1H, m), 1.00 (3H, d, *J* = 6.2 Hz), 0.98 (3H, d, *J* = 6.2 Hz); ^{13}C NMR (151 MHz, $CDCl_3$) δ 155.5, 150.2, 148.7 (q, *J* = 34.3 Hz), 141.2, 128.6, 128.4, 126.3, 126.2, 121.8 (q, *J* = 273.9 Hz), 120.9, 52.4, 48.8, 45.9, 35.3, 33.5, 22.9, 22.9; ^{19}F NMR (564 MHz, $CDCl_3$) δ –67.9; HRMS (ESI) found MH^+ 337.1881, $C_{19}H_{24}F_3N_2$ requires 337.1886.

N-Isopropyl-4-phenyl-2-(4-(trifluoromethyl)thiazol-2-yl)butan-1-amine (13z)

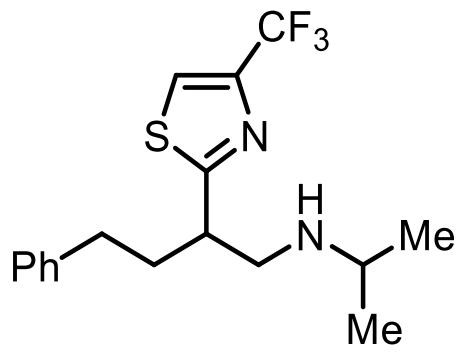

Following **GP8a**, *trans*-aziridine borane **13a** (20 mg, 0.1 mmol, 1.0 equiv.) and 2-bromo-4-(trifluoromethyl)thiazole (24.4 µL, 0.2 mmol, 2.0 equiv.) gave **13z** (46%) as an oil. R_f 0.30 [CH_2Cl_2/MeOH/NH_3 15:1:0.01]. ^{1}H NMR (600 MHz, $CDCl_3$) δ 7.65 (1H, s), 7.27 (2H, t, *J* = 7.5 Hz), 7.18 – 7.14 (3H, m), 3.31 (1H, p, *J* = 6.8 Hz), 3.01 – 2.93 (2H, m), 2.77 (1H, hept, *J* = 6.3 Hz), 2.63 (2H, t, *J* = 7.9 Hz), 2.16 – 2.06 (2H, m), 1.03 (3H, d, *J* = 6.0 Hz), 1.02 (3H, d, *J* = 6.1 Hz); ^{13}C NMR (151 MHz, $CDCl_3$) δ 176.3, 144.2 (q, *J* = 36.2 Hz), 141.4, 128.6, 128.5, 126.2, 120.7 (q, *J* = 270.5 Hz), 120.5 (q, *J* = 3.0 Hz), 51.3, 48.9, 44.4, 35.7, 33.4, 22.9, 22.8; ^{19}F NMR (564 MHz, $CDCl_3$) δ –63.8; HRMS (ESI) found MH^+ 343.1459, $C_{17}H_{22}F_3N_2S$ requires 343.1450.

2-(1-(Isopropylamino)-4-phenylbutan-2-yl)thiazole-4-carbonitrile (13ab)

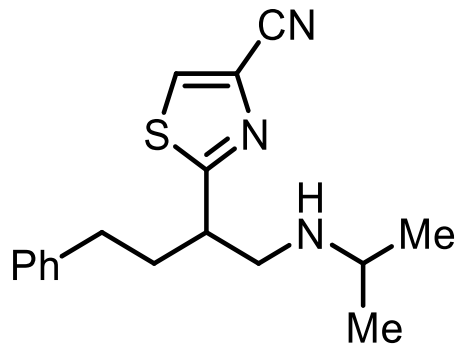

Following **GP8a,** but using 3,3',5,5'-tetrakis(trifluoromethyl)benzophenone (20 mol%) and heteroarylbromide (3 equiv.), *trans*-aziridine borane **13a** (20 mg, 0.1 mmol, 1.0 equiv.) and 2-bromo-4-cyanothiazole (57 mg, 0.3 mmol, 3.0 equiv.) gave **13ab** (43%) as an oil. R_f 0.29

[CH_2Cl_2/MeOH/NH_3 15:1:0.01]. ^{1}H NMR (600 MHz, $CDCl_3$) δ 7.89 (1H, s), 7.27-7.24 (2H, m), 7.18 (1H, t, *J* = 7.4 Hz), 7.14 (2H, d, *J* = 7.4 Hz), 3.28 – 3.23 (1H, m), 2.97 – 2.90 (2H, m), 2.74 (1H, hept, *J* = 6.0 Hz), 2.61 (2H, t, *J* = 7.9 Hz), 2.15 – 2.03 (2H, m), 1.08 (3H, d, *J* = 5.5 Hz), 1.01 (3H, d, *J* = 5.6 Hz); ^{13}C NMR (151 MHz, $CDCl_3$) δ 175.8, 141.2, 130.4, 128.6, 128.5, 126.3, 126.1, 114.5, 51.1, 49.0, 44.4, 35.5, 33.4, 23.0, 22.9; HRMS (ESI) found MH^+ 300.1535, $C_{17}H_{22}N_3S$ requires 300.1529.

Methyl 4-(1-(isopropylamino)-4-phenylbutan-2-yl)thiazole-2-carboxylate (13ac)

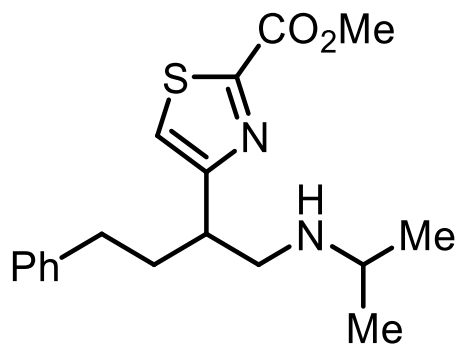

Following **GP8a**, but using 3,3',5,5'-tetrakis(trifluoromethyl)benzophenone (20 mol%) and heteroarylbromide (3 equiv.), *trans*-aziridine borane **13a** (20 mg, 0.1 mmol, 1.0 equiv.) and methyl 4-bromothiazole-2-carboxylate (67 mg, 0.3 mmol, 3.0 equiv.) gave **13ac** (33%) as an oil. R_f 0.39 [CH_2Cl_2/MeOH/NH_3 15:1:0.01; double run]. ^{1}H NMR (600 MHz, $CDCl_3$) δ 7.36 (1H, s), 7.24 (2H, t, *J* = 7.6 Hz), 7.15 (1H, t, *J* = 7.4 Hz), 7.11 (2H, d, *J* = 7.5 Hz), 4.01 (3H, s), 3.28 – 3.23 (1H, m), 3.06 (1H, t, *J* = 10.2 Hz), 3.00 (1H, dd, *J* = 12.1, 5.4 Hz), 2.83 (1H, hept, *J* = 6.0 Hz), 2.55 – 2.48 (2H, m), 2.17 – 2.05 (2H, m), 1.08 (3H, d, *J* = 6.3 Hz), 1.06 (3H, d, *J* = 6.2 Hz); ^{13}C NMR (151 MHz, $CDCl_3$) δ 161.0, 160.6, 157.9, 141.7, 128.5, 128.5, 126.0, 121.7, 53.3, 50.8, 49.3, 41.6, 34.6, 33.5, 22.2, 22.1; HRMS (ESI) found MH^+ 333.1639, $C_{18}H_{25}N_2O_2S$ requires 333.1631.

Methyl 4-(1-phenyl-2-(propylamino)ethyl)benzoate (1t)

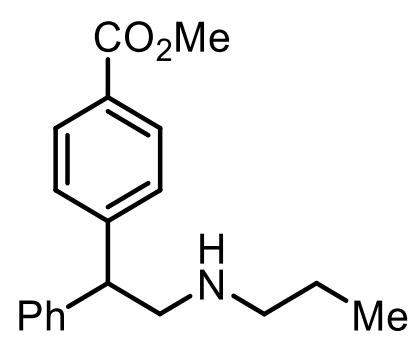

Following **GP8a**, *trans*-aziridine borane **1a** (18 mg, 0.1 mmol, 1.0 equiv.) and 4-bromobenzoate (43 mg, 0.2 mmol, 2.0 equiv.) gave **1t** (19%) as an oil. R_f 0.31 [EtOAc]. ^{1}H NMR (600 MHz, $CDCl_3$) δ 7.97 (2H, d, *J* = 8.3 Hz), 7.35 – 7.29 (4H, m), 7.25 – 7.20 (3H, m), 4.26 (1H, t, *J* = 7.6 Hz), 3.89 (3H, s), 3.29 – 3.22 (2H, m), 2.61 (2H, t, *J* = 7.3 Hz), 1.45 (2H, sx, *J* = 7.4 Hz), 0.84 (3H, t, *J* = 7.4 Hz); ^{13}C NMR (151 MHz, $CDCl_3$) δ 167.1, 148.5, 142.2, 130.1, 128.9, 128.6, 128.2, 128.2, 127.0, 54.3, 52.2, 51.9, 51.4, 23.1, 11.8; HRMS (ESI) found MH^+ 298.1795, $C_{19}H_{24}NO_2$ requires 298.1802.

Methyl 4-(1-(Hexylamino)-4-phenylbutan-2-yl)benzoate (27c)

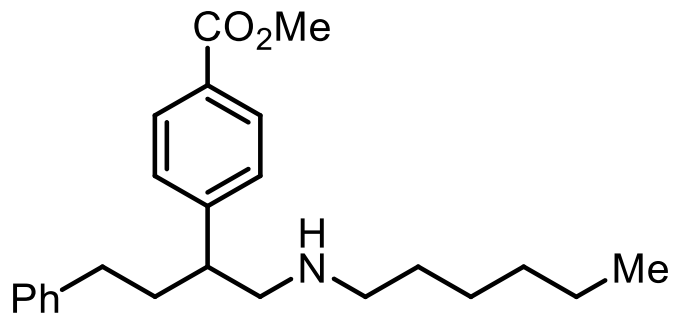

Following **GP8a**, *trans*-aziridine borane **27a** (25 mg, 0.1 mmol, 1.0 equiv.) and methyl 4-bromobenzoate (43 mg, 0.2 mmol, 2.0 equiv.) gave **27c** (65%) as an oil. R_f 0.49 [EtOAc]. ^{1}H NMR (600 MHz, $CDCl_3$) δ 8.02 (2H, d, *J*=8.3 Hz), 7.29 (2H, d, *J*=8.2 Hz), 7.24 (2H, t, *J*=7.6 Hz), 7.16 (1H, t, *J*=7.4 Hz), 7.08 (2H, d, *J*=6.8 Hz), 3.92 (3H, s), 2.93 – 2.84 (2H, m), 2.81 (1H, dd, *J*=11.4, 8.8 Hz), 2.57 – 2.41 (4H, m), 2.03 (1H, dddd, *J*=14.3, 9.7, 7.1, 4.7 Hz), 1.91 (1H, dtd, *J*=13.6, 9.4, 5.7 Hz), 1.41 – 1.30 (2H, m), 1.28 – 1.17 (6H, m), 0.85 (3H, t, *J*=7.1 Hz); ^{13}C NMR (151 MHz, $CDCl_3$) δ 167.2, 149.4, 142.0, 130.1, 128.7, 128.5, 128.1, 125.9, 55.8, 52.1, 50.1, 45.9, 36.1, 33.6, 31.8, 30.0, 27.0, 22.7, 14.1; HRMS (ESI) found MH^+ 368.2578, $C_{24}H_{34}NO_2$ requires 368.2584.

Methyl 4-(1-(Benzylamino)propan-2-yl)benzoate (28c)

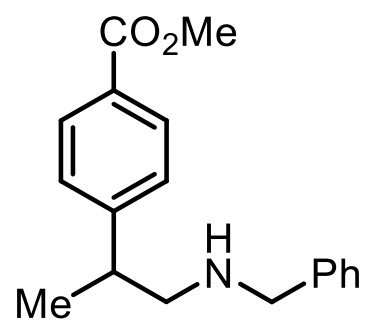

Following **GP8a**, *trans*-aziridine borane **28a** (16 mg, 0.1 mmol, 1.0 equiv.) and methyl 4-bromobenzoate (43 mg, 0.2 mmol, 2.0 equiv.) gave **28c** (47%) as an oil. R_f 0.29 [EtOAc]. ^{1}H NMR (600 MHz, $CDCl_3$) δ 7.90 (2H, d, *J*=8.3 Hz), 7.25 – 7.13 (7H, m), 3.83 (3H, s), 3.70 (1H, d, *J*=13.4 Hz), 3.65 (1H, d, *J*=13.4 Hz), 2.95 (1H, h, *J*=7.0 Hz), 2.75 – 2.72 (2H, dd, *J*=7.2, 1.3 Hz), 1.44 (1H, br s), 1.19 (3H, d, *J*=6.9 Hz); ^{13}C NMR (151 MHz, $CDCl_3$) δ 167.2, 151.1, 140.3, 130.0, 128.5, 128.1, 127.4, 127.1, 56.1, 53.9, 52.1, 40.4, 20.0; HRMS (ESI) found MH^+ 284.1642, $C_{18}H_{22}NO_2$ requires 284.1645.

Methyl 4-(1-(Benzylamino)hexan-2-yl)benzoate (29c)

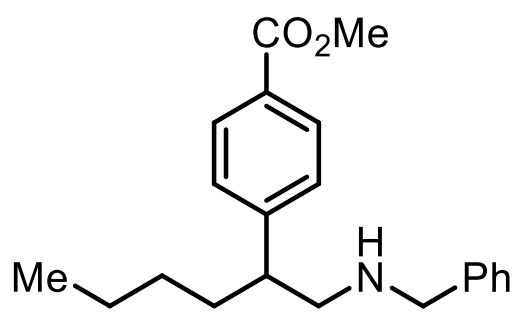

Following **GP8a**, *trans*-aziridine borane **29a** (20 mg, 0.1 mmol, 1.0 equiv.) and methyl 4-bromobenzoate (43 mg, 0.2 mmol, 2.0 equiv.) gave **29c** (46%) as an oil. R_f 0.20 [EtOAc]. ^{1}H NMR (600 MHz, $CDCl_3$) δ 7.98 (2H, d, *J*=8.3 Hz), 7.28 (2H, t, *J*=7.5 Hz), 7.26 – 7.17 (5H, m), 3.91 (3H, s), 3.76 (1H, d, *J*=13.4 Hz), 3.69 (1H, d, *J*=13.4 Hz), 2.90 – 2.77 (3H, m), 1.67 (1H, ddt, *J*=13.4, 10.4, 5.2 Hz), 1.57 – 1.51 (1H, m), 1.47 (1H, s), 1.32 – 1.05 (4H, m), 0.81 (3H, t,

J=7.3 Hz); ^{13}C NMR (151 MHz, $CDCl_3$) δ 167.2, 149.9, 140.3, 130.0, 128.5, 128.5, 128.1, 128.0, 127.0, 55.0, 54.0, 52.1, 46.5, 34.2, 29.7, 22.8, 14.1; HRMS (ESI) found MH^+ 326.2108, $C_{21}H_{28}NO_2$ requires 326.2115.

Methyl 4-(1-(Cyclohexylamino)hexan-2-yl)benzoate (38b)

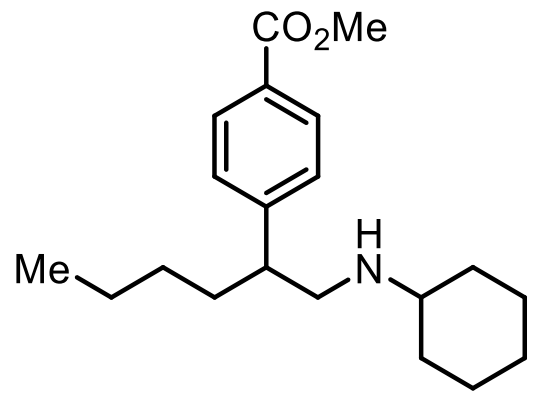

Following **GP8b**, **38** (18 mg, 0.1 mmol, 1.0 equiv.) and methyl 4-bromobenzoate (43 mg, 0.2 mmol, 2.0 equiv.) gave **38b** (57% *over two steps*) as an oil. R_f 0.19 [EtOAc]. ^{1}H NMR (600 MHz, $CDCl_3$) δ 8.00 (2H, d, J=8.0 Hz), 7.29 (2H, d, J=7.7 Hz), 3.93 (3H, s), 2.93 (1H, dd, J=10.3, 4.0 Hz), 2.88 – 2.77 (2H, m), 2.40 – 2.30 (1H, m), 1.83 (1H, d, J=12.8 Hz), 1.78 – 1.64 (4H, m), 1.62 – 1.53 (2H, m), 1.29 – 1.10 (7H, m), 1.04 – 0.93 (2H, m), 0.84 (3H, t, J=7.3 Hz); ^{13}C NMR (151 MHz, $CDCl_3$) δ 167.3, 150.1, 130.0, 128.5, 128.0, 56.9, 52.8, 52.1, 46.6, 34.5, 33.7, 33.6, 29.7, 26.3, 25.2, 25.1, 22.8, 14.1; HRMS (ESI) found MH^+ 318.2426, $C_{20}H_{32}NO_2$ requires 318.2428.

Methyl 4-(1-(*tert*-Butylamino)-4-phenylbutan-2-yl)benzoate (39b)

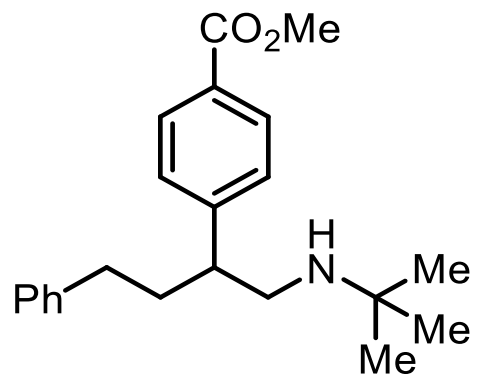

Following **GP8b**, **39** (20 mg, 0.1 mmol, 1.0 equiv.) and methyl 4-bromobenzoate (43 mg, 0.2 mmol, 2.0 equiv.) gave **39b** (48% *over two steps*) as an oil. R_f 0.14 [EtOAc]. ^{1}H NMR (600 MHz, $CDCl_3$) δ 8.02 (2H, d, J=8.3 Hz), 7.30 (2H, d, J=8.2 Hz), 7.24 (2H, t, J=7.7 Hz), 7.16 (1H, t, J=7.6 Hz), 7.08 (2H, d, J=7.2 Hz), 3.92 (3H, s), 2.86 – 2.75 (3H, m), 2.51 – 2.40 (2H, m), 2.10 – 2.02 (1H, m), 1.95 – 1.86 (1H, m), 0.99 (9H, s); ^{13}C NMR (151 MHz, $CDCl_3$) δ 167.2, 149.5, 142.1, 130.1, 128.8, 128.5, 128.1, 126.0, 52.2, 50.3, 48.5, 46.6, 36.5, 33.7, 29.0; HRMS (ESI) found MH^+ 340.2266, $C_{22}H_{30}NO_2$ requires 340.2271.

Methyl 4-(5-(Methylamino)octan-4-yl)benzoate (31d)

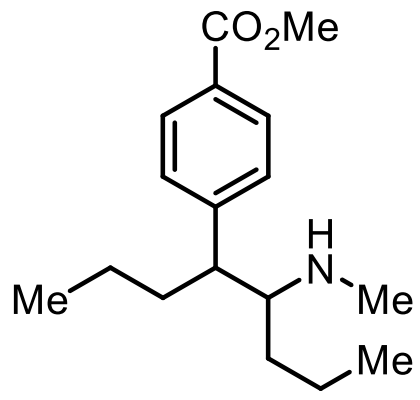

Following **GP8a**, but using 3,3',5,5'-tetrakis(trifluoromethyl)benzophenone (20 mol%) and running the reaction for 16 h, **31a** (16 mg, 0.1 mmol, 1.0 equiv.) and methyl 4-bromobenzoate (43 mg, 0.2 mmol, 2.0 equiv.) gave **31d** as mixture of diastereomers (41%) as an oil. d.r. 1.7:1. R_f 0.11 [EtOAc]. ^{1}H NMR (400 MHz, $CDCl_3$), s*ignals corresponding to both diastereomers*: δ 8.00 – 7.88 (2H, m), 7.31 – 7.14 (2H, m), 3.88 (3H, s), 2.82 – 2.67 (1H, m), 2.59 – 2.48 (1H, m), 2.39 (1.1H, s), 2.28 (1.9H, s), 1.83 – 1.54 (2H, m), 1.31 – 0.99 (6H, m), 0.92 – 0.72 (6H, m); ^{13}C NMR (151 MHz, $CDCl_3$) δ 167.3, 167.3, 149.4, 149.2, 129.7, 129.6, 128.9, 128.8, 128.4, 128.2, 64.0, 63.8, 52.1, 52.1, 48.9, 48.5, 34.5, 33.8, 33.8, 33.7, 33.3, 33.0, 21.1, 21.0, 19.3, 18.9, 14.5, 14.3, 14.3, 14.2; HRMS (ESI) found MH^+ 278.2108, $C_{17}H_{28}NO_2$ requires 278.2115.

***trans*-Methyl 4-(2-(cyclohexylamino)cyclohexyl)benzoate (33d)**

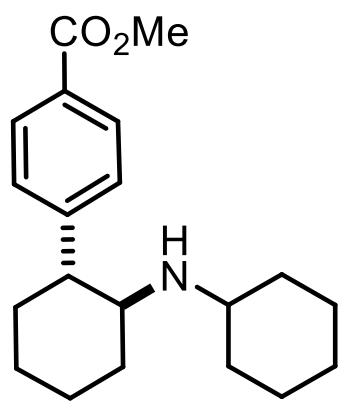

Following **GP8a** but using 3,3',5,5'-tetrakis(trifluoromethyl)benzophenone (20 mol%), *trans*-aziridine borane **33a** (19 mg, 0.1 mmol, 1.0 equiv.) and 4-bromobenzoate (43 mg, 0.2 mmol, 2.0 equiv.) gave **33d** (55%) as an oil. R_f 0.28 [DCM:MeOH (15:1)]. ^{1}H NMR (400 MHz, $CDCl_3$) δ 7.98 (2H, d, *J*=7.9 Hz), 7.31 (2H, d, *J*=7.9 Hz), 3.90 (3H, s), 2.84 (1H, td, *J*=10.5, 3.7 Hz), 2.63 – 2.51 (1H, m), 2.40 – 2.29 (1H, m), 2.18 – 2.08 (1H, m), 1.90 – 1.69 (5H, m), 1.62 – 1.43 (4H, m), 1.42 – 1.22 (4H, m), 1.20 – 0.79 (4H, m), 0.59 (1H, q, *J*=11.6 Hz); ^{13}C NMR (151 MHz, $CDCl_3$) δ 167.2, 149.8, 130.1, 128.8, 128.0, 57.9, 53.4, 52.2, 51.0, 34.7, 32.9, 32.4, 26.2, 25.9, 25.3, 25.2, 25.0; HRMS (ESI) found MH^+ 316.2265, $C_{20}H_{30}NO_2$ requires 316.2271.

The key ^{1}H and ^{13}C chemical shifts were further assigned using 2D NMR. The *trans* configuration was assigned based on the coupling constants observed for H-8 (^{1}H NMR: δ, 2.84, td, *J* = 10.5, 3.7 Hz, 1H).

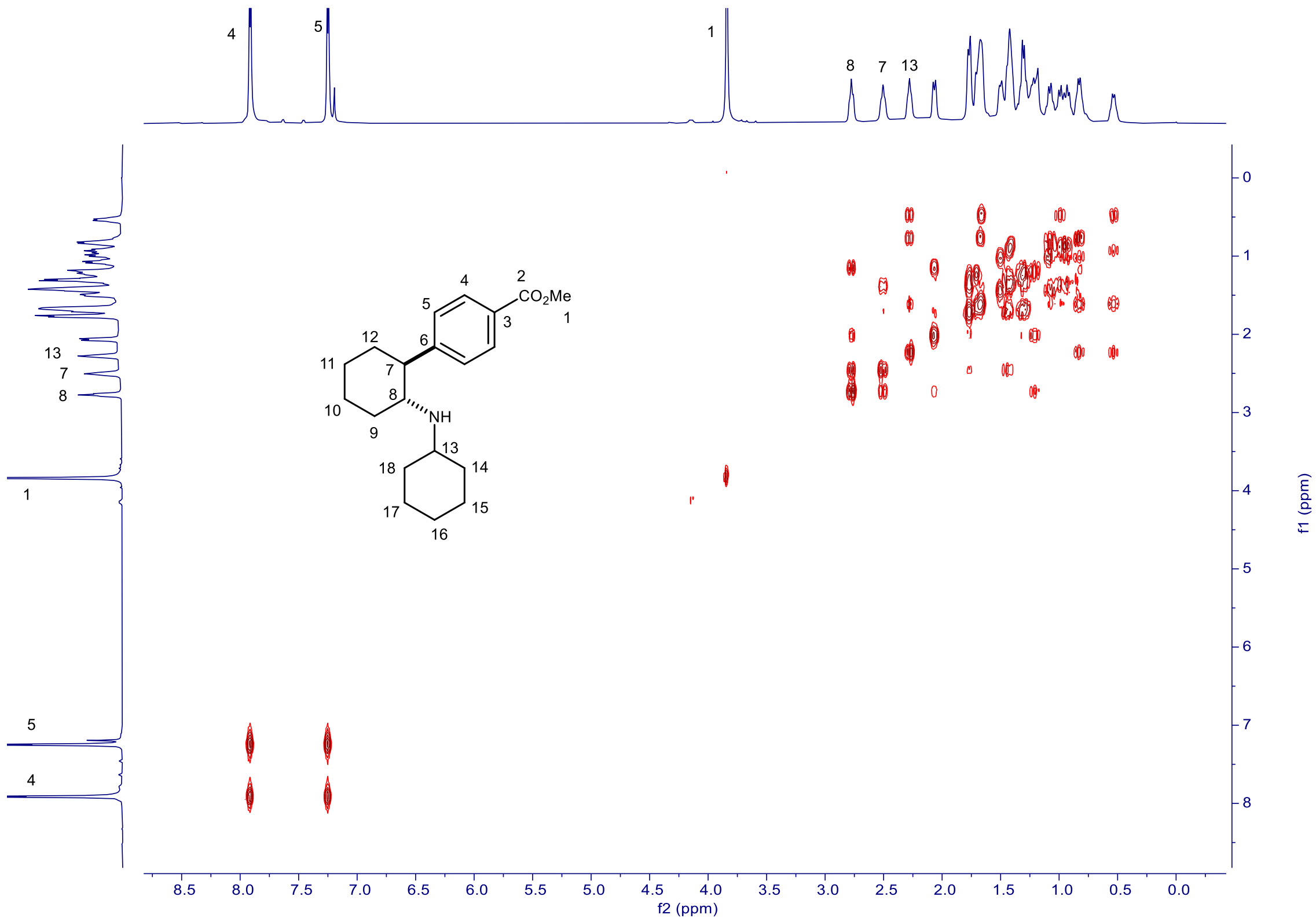

COSY NMR spectrum (600 MHz, $CDCl_3$, at rt) of **33d**

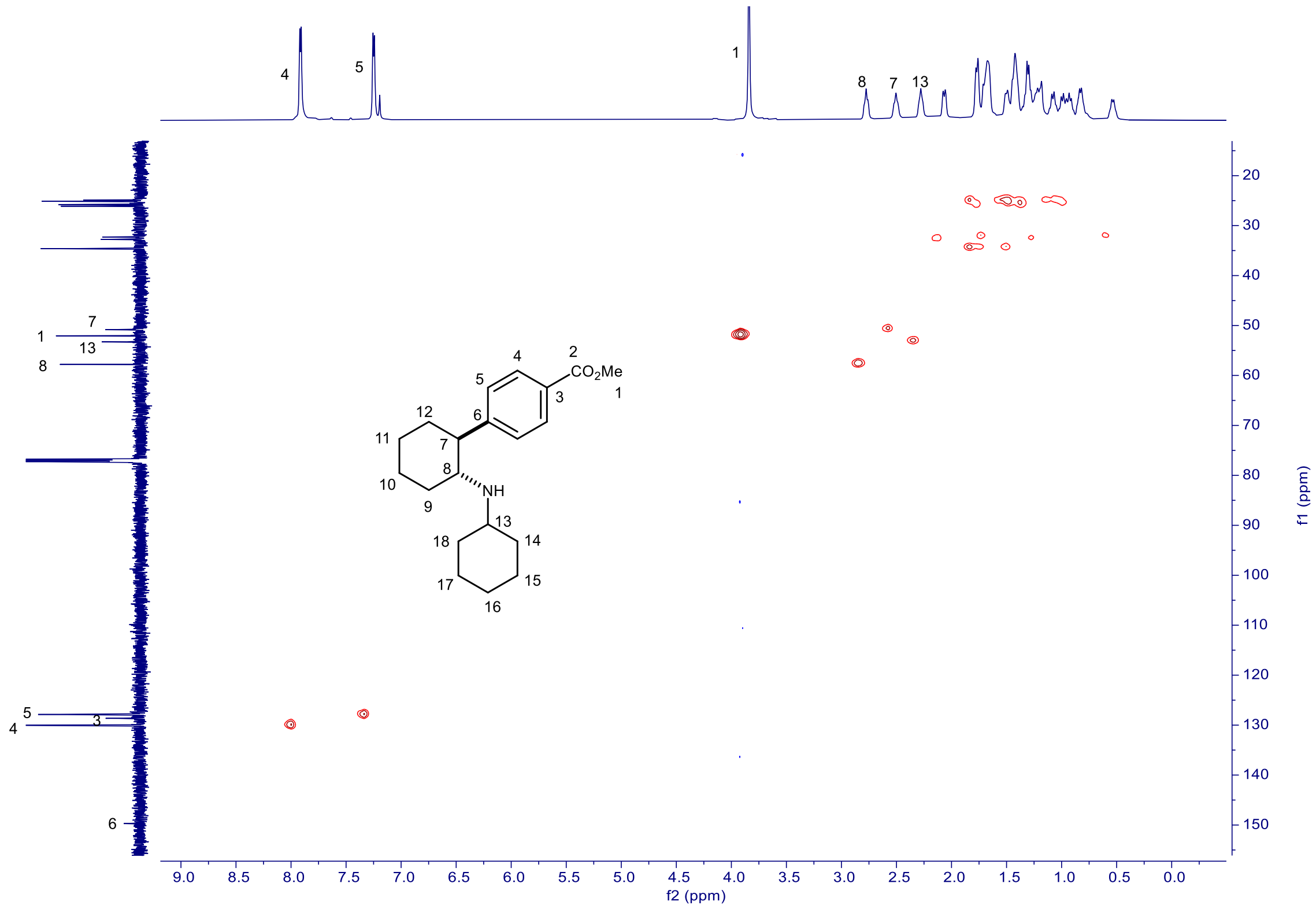

HSQC NMR spectrum (600 MHz, $CDCl_3$, at rt) of **33d**

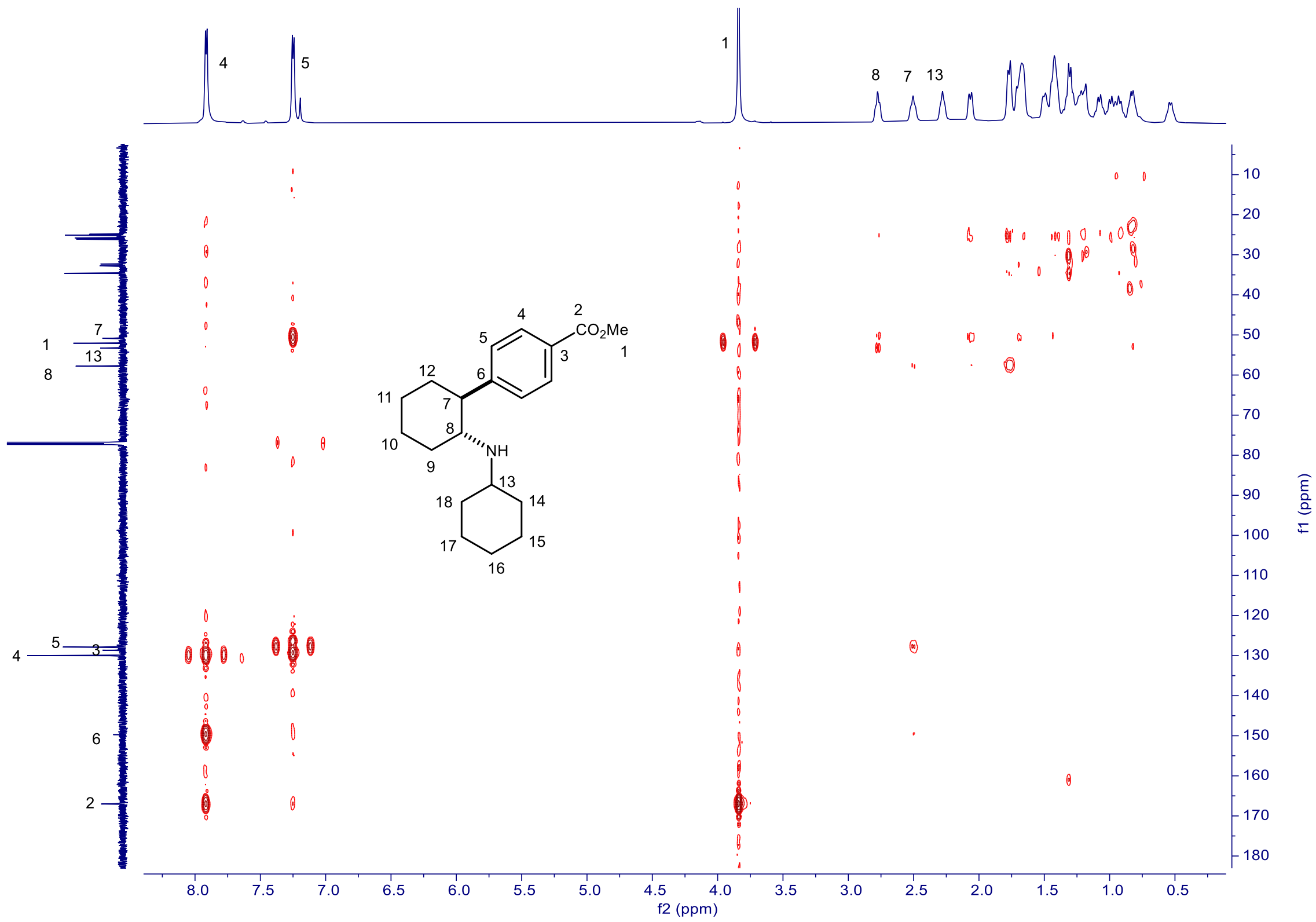

HMBC NMR spectrum (600 MHz, $CDCl_3$, at rt) of **33d**

tert-Butyl 4-((2-(4-(Methoxycarbonyl)phenyl)hexyl)amino)piperidine-1-carboxylate (40b)

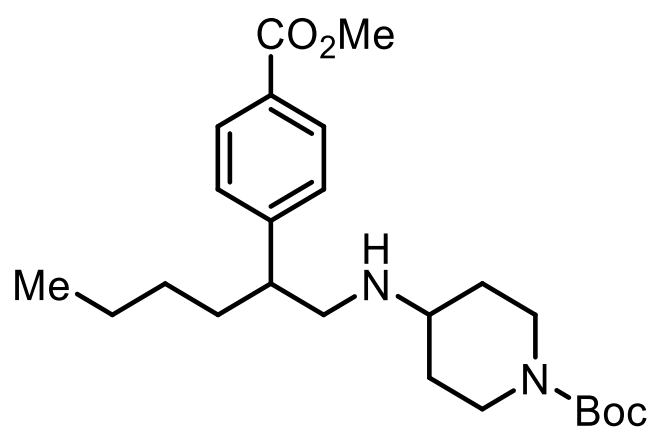

Following **GP8b**, **40** (28 mg, 0.1 mmol, 1.0 equiv.) and methyl 4-bromobenzoate (43 mg, 0.2 mmol, 2.0 equiv.) gave **40b** (58% *over two steps*) as an oil. R_f 0.25 [EtOAc]. ^{1}H NMR (600 MHz, $CDCl_3$) δ 7.98 (2H, d, *J*=8.3 Hz), 7.25 (2H, d, *J*=8.3 Hz), 4.06 – 3.82 (5H, m), 2.94 – 2.85 (1H, m), 2.83 – 2.67 (4H, m), 2.50 (1H, tt, *J*=10.2, 3.8 Hz), 1.79 – 1.63 (3H, m), 1.61 – 1.49 (1H, m), 1.42 (9H, s), 1.30 – 1.07 (6H, m), 0.81 (3H, t, *J*=7.3 Hz); ^{13}C NMR (151 MHz, $CDCl_3$) δ 167.2, 154.9, 149.8, 130.0, 128.6, 127.9, 79.5, 55.0, 52.6, 52.2, 46.7, 34.3, 29.7, 28.6, 22.8, 14.1; HRMS (ESI) found MH^+ 419.2896, $C_{24}H_{39}N_2O_4$ requires 419.2904.

Methyl 4-(1-(Cyclohexylamino)-3-isopropoxypropan-2-yl)benzoate (41b)

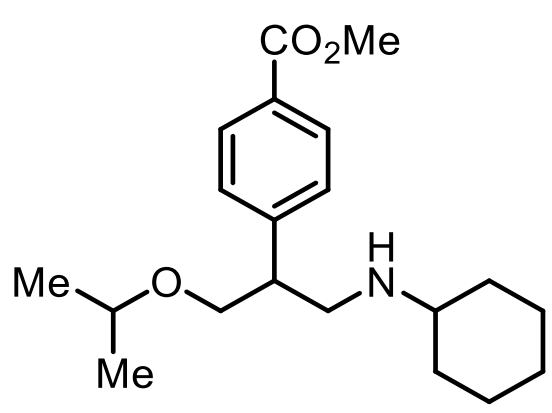

Following **GP8b**, **41** (20 mg, 0.1 mmol, 1.0 equiv.) and methyl 4-bromobenzoate (43 mg, 0.2 mmol, 2.0 equiv.) gave **41b** (47% *over two steps*) as an oil. R_f 0.16 [EtOAc]. ^{1}H NMR (600 MHz, $CDCl_3$) δ 7.97 (2H, d, *J*=8.3 Hz), 7.31 (2H, d, *J*=8.2 Hz), 3.90 (3H, s), 3.63 – 3.55 (2H, m), 3.50 (1H, hp, *J*=6.0 Hz), 3.14 – 3.03 (2H, m), 2.88 (1H, dd, *J*=11.3, 7.4 Hz), 2.37 (1H, tt, *J*=10.5, 3.7 Hz), 1.83 – 1.75 (2H, m), 1.71 – 1.64 (2H, m), 1.57 (1H, dt, *J*=13.3, 3.7 Hz), 1.49 (1H, br s), 1.27 – 1.15 (2H, m), 1.15 – 1.07 (7H, m), 1.06 – 0.93 (2H, m); ^{13}C NMR (151 MHz, $CDCl_3$) δ 167.2, 147.7, 129.9, 128.7, 128.2, 72.0, 71.5, 56.9, 52.1, 49.9, 46.7, 33.6, 33.5, 26.3, 25.2, 25.1, 22.1; HRMS (ESI) found MH^+ 334.2381, $C_{20}H_{32}NO_3$ requires 334.2377.

Methyl 4-(1-((2-((4*R*,6*R*)-6-(2-(*tert*-Butoxy)-2-oxoethyl)-2,2-dimethyl-1,3-dioxan-4-yl)ethyl)amino)-4-phenylbutan-2-yl)benzoate (42b)

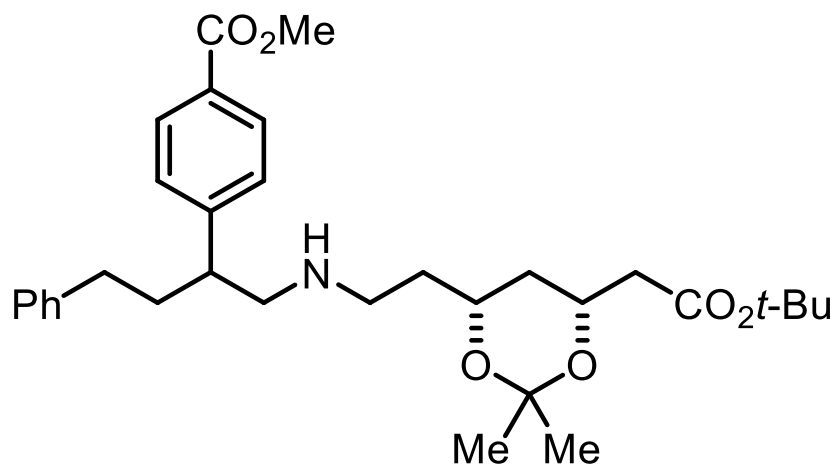

Following **GP8b**, **42** (40 mg, 0.1 mmol, 1.0 equiv.) and methyl 4-bromobenzoate (43 mg, 0.2 mmol, 2.0 equiv.) gave **42b** as mixture of diastereomers (37% *over two steps*) as an oil. d.r. 1:1. R_f 0.13 [EtOAc]. ^{1}H NMR (400 MHz, $CDCl_3$), s*ignals corresponding to both diastereomers*: δ 7.94 (2H, d, *J*=8.0 Hz), 7.26 – 7.13 (4H, m), 7.08 (1H, t, *J*=7.3 Hz), 7.00 (2H, d, *J*=7.4 Hz), 4.17 – 4.05 (1H, m), 3.85 (3H, s), 3.79 – 3.68 (1H, m), 2.86 – 2.71 (3H, m), 2.61 – 2.47 (2H, m), 2.43 – 2.27 (3H, m), 2.23 – 2.14 (1H, m), 2.01 – 1,89 (1H, m), 1.87 – 1.75 (1H, m), 1.53 – 1.33 (12H, s), 1.26 – 1.14 (6H, m), 1.13 – 1.00 (1H, m); ^{13}C NMR (101 MHz, $CDCl_3$) δ 170.4, 167.1, 149.4, 149.4, 142.0, 130.1, 128.7, 128.5, 128.5, 128.1, 126.0, 98.7, 98.7, 68.0, 67.5, 66.3, 55.9, 55.9, 52.2, 46.5, 46.0, 46.0, 42.8, 42.8, 36.7, 36.6, 36.5, 36.4, 36.2, 36.0, 33.6, 30.1, 30.1, 28.2, 19.7, 19.7; HRMS (ESI) found MH^+ 540.3312, $C_{32}H_{46}NO_6$ requires 540.3320.

6 Scope of Other Nucleophiles

We attempted to engage other nucleophiles under various reaction conditions. While we did not succeed in obtaining high yields, the results below demonstrate proof-of-principle results that additional ring-opening functionalisations could be further developed.

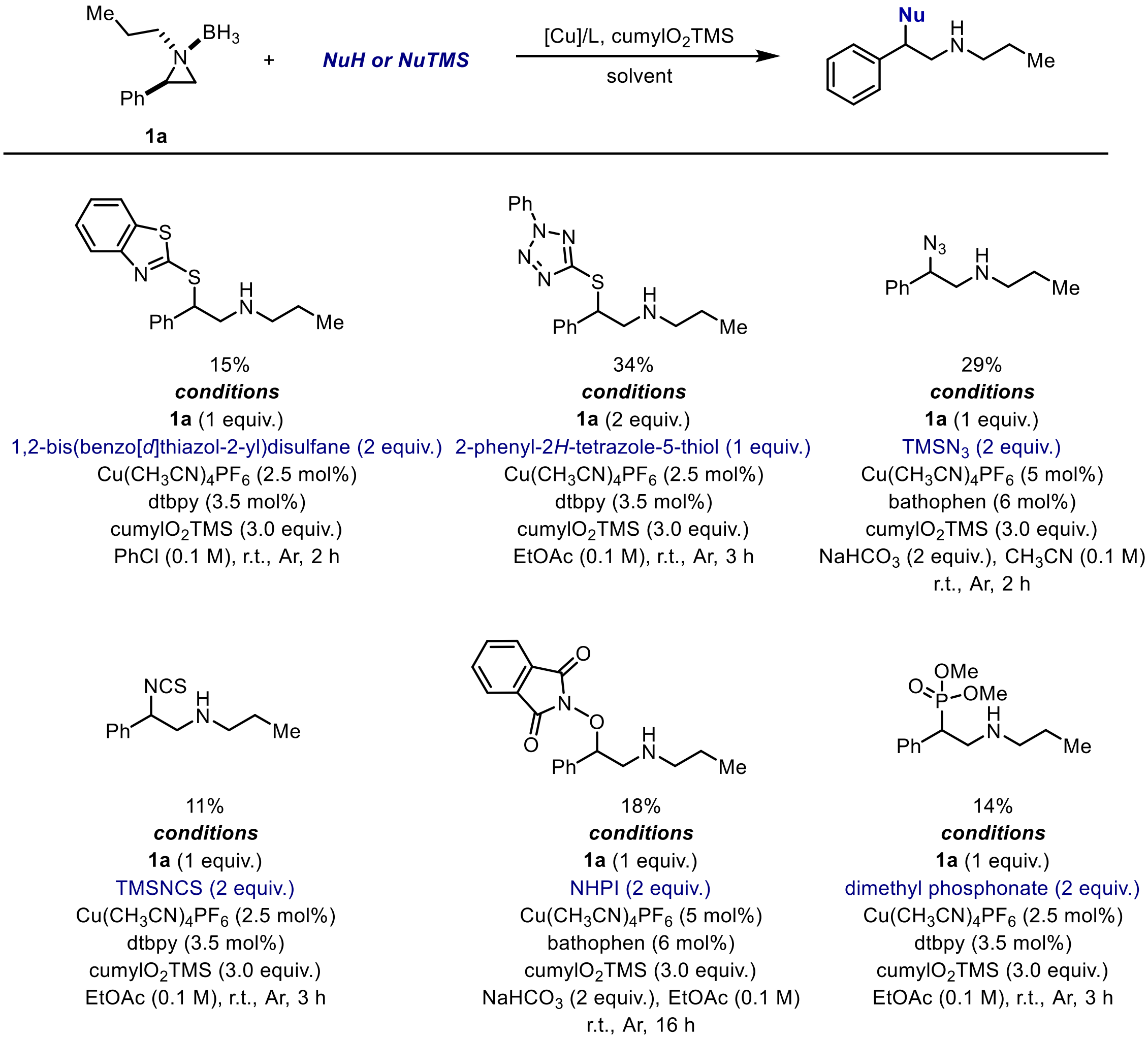

Figure S1. Scope of other nucleophiles. All reported yields are NMR yields determined by ^{1}H NMR using 1,3,5-trimethoxybenzene as internal standard.

7 Comparison of Arylation of *cis* and *trans* Aziridine Boranes

The *cis* and *trans* aziridine boranes **12a** and **12a'** were engaged separately under the optimised conditions (**GP5a**).

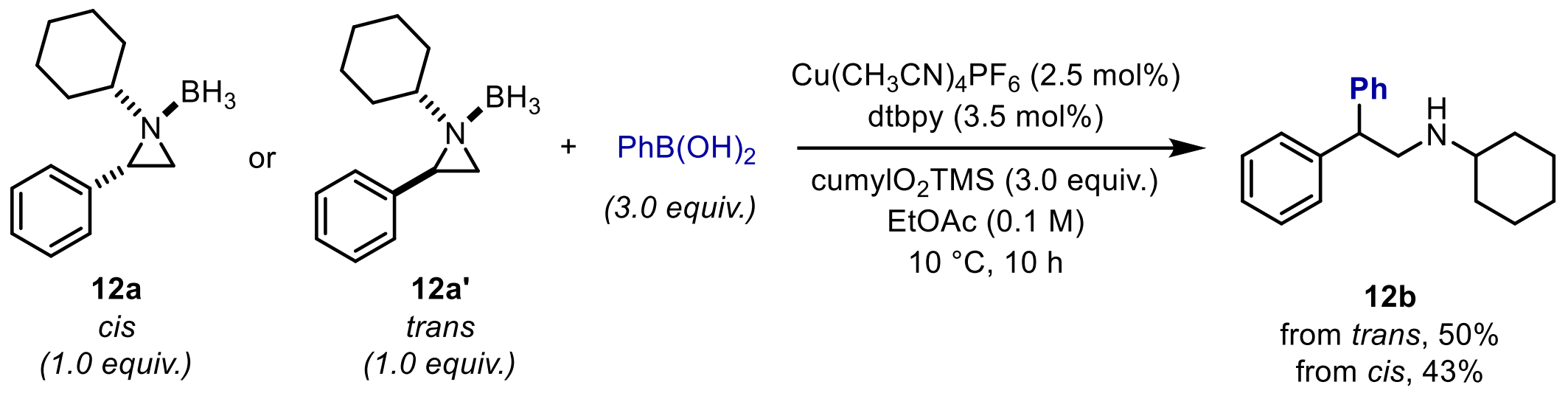

Figure S2.

8 Scale-up Experiments

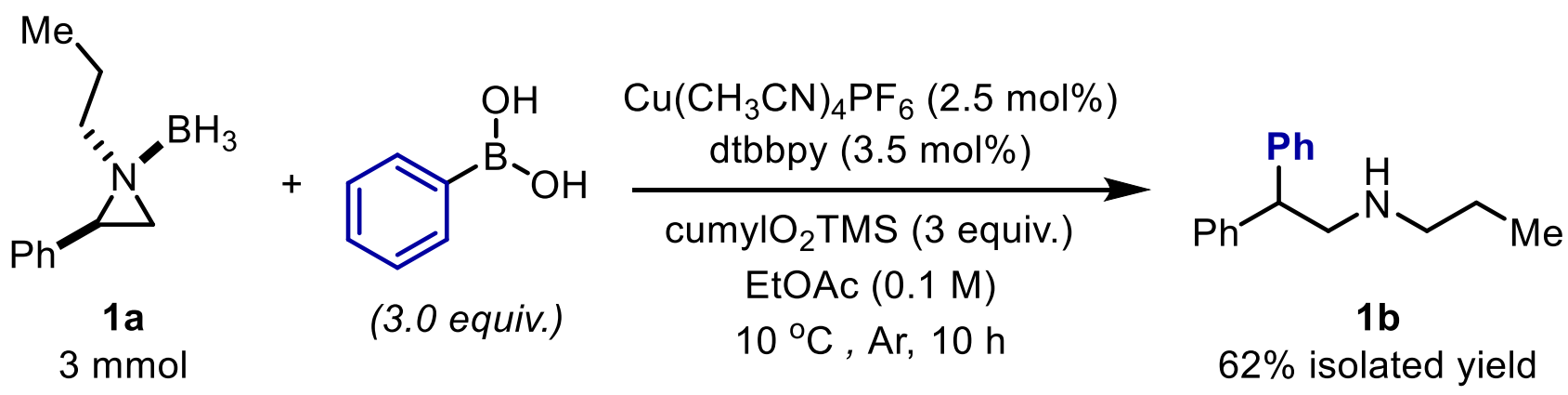

An oven-dried vial equipped with a stirring bar was charged with the *trans*-2-phenyl-1-propylaziridine borane **1a** (3 mmol, 1.0 equiv.) and phenylboronic acid (9 mmol, 3.0 equiv.). The tube was capped with a Supelco aluminium crimp seal with septum (PTFE/butyl), evacuated and refilled with Ar (x 3). The peroxide (9 mmol, 3.0 equiv.) and dry and degassed solvent (15 mL) was then added to the vial and stirred for around **1 min**. The resulting mixture was cooled down to 10 °C and the catalyst solution (15 mL of the corresponding dry and degassed solvent, containing $Cu(CH_3CN)_4PF_6$ (27 mg, 2.5 mol%) and dtbpy (30 mg, 3.5 mol%), pre-stirred for 10 min until fully dissolved) was then added under sitrring. The resulting mixture was stirred at 10 °C for 10 h. Upon completion, aqueous NaOH (1 M) was added, the aqueous layer was separated and extracted with EtOAc (2 x). The combined organic layers were dried ($MgSO_4$) and filtered. The solvent was evaporated and purified by column chromatography on silica gel to afford the desired product **1b** in 62% yield (1.86 mmol, 450 mg).

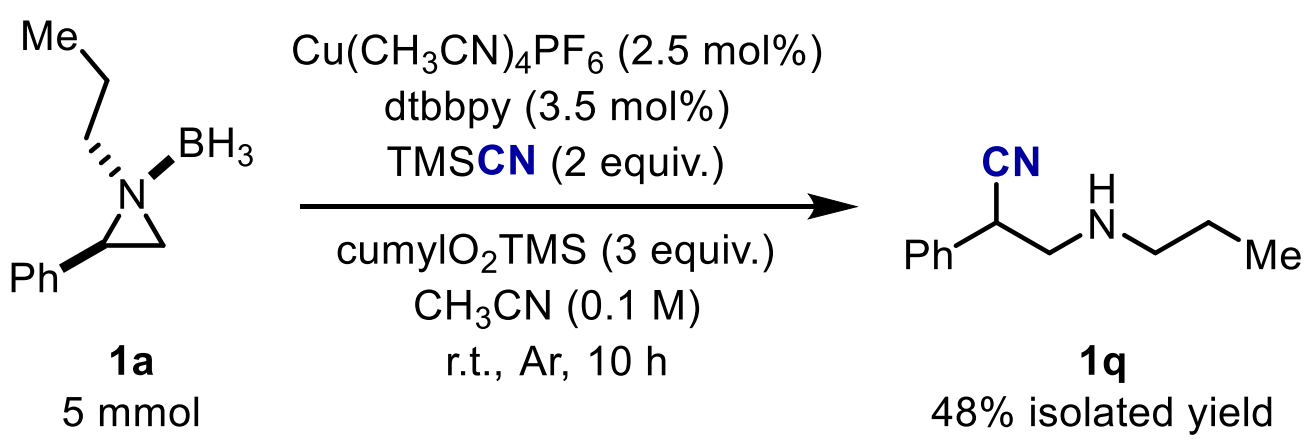

An oven-dried vial equipped with a stirring bar was charged with the *trans*-2-phenyl-1-propylaziridine borane **1a** (5 mmol, 1.0 equiv.). The tube was capped with a Supelco aluminium crimp seal with septum (PTFE/butyl), evacuated and refilled with Ar (x 3). The peroxide (9 mmol, 3.0 equiv.), TMSCN (10 mmol, 2.0 equiv.) and dry and degassed CH_3CN (25 mL) was then added to the vial and stirred for around **1 min**. The catalyst solution (25 mL of dry and degassed CH3CN, containing $Cu(CH_3CN)_4PF_6$ (45 mg, 2.5 mol%) and dtbpy (50 mg, 3.5 mol%), pre-stirred for 10 min until fully dissolved) was then added under sitrring. The resulting mixture was stirred at room temperature for 2 h. Upon completion, an aqueous solution of NaOH (1 M) was added, the aqueous layer was separated and extracted with EtOAc (2 x). The combined organic layers were dried ($MgSO_4$) and filtered. The solvent was evaporated and purified by column chromatography on silica gel to afford the desired product **1q** in 48% yield (2.4 mmol, 450 mg).

9 Cyclic Voltammetry Studies

Cyclic voltammetry (CV) experiments were conducted using a conventional three-electrode cell equipped with a platinum working electrode (1 mm diameter), an Ag/AgCl reference electrode (3.0 M KCl), and a platinum wire counter electrode. Measurements of the substrates (20 mM) were performed in CH_3CN containing *n*-Bu_4NPF_6 (0.1 M) as the supporting electrolyte at a scan rate of 100 mV s^{-1}. Ferrocene (Cp_2Fe, 20 mM) was dissolved in CH_3CN with *n*-Bu_4NPF_6 (0.1 M) and measured under identical conditions. The half-wave potential of the Fc^+/Fc redox couple was determined to be 0.43 V vs. Ag/AgCl (3.0 M KCl).

Cyclic voltammetry (CV) experiments were performed on *N*-alkyl β-aryl and β-alkyl aziridines (**1** and **13**), as well as their corresponding borane complexes (**1a** and **13a**). As shown in **Figure S3**, both *N*-alkyl β-aryl and β-alkyl aziridines exhibit relatively high oxidative potentials. The oxidation potential of the *N*-alkyl β-alkyl aziridine **13** (E_p = 1.82 V) is lower than that of the *N*-alkyl β-aryl aziridine **1** (E_p = 2.04 V). Upon coordination with borane, the oxidation potentials of both aziridines decrease significantly (E_p = 1.56 V for **1a** and 1.06 V for **13a**). In contrast, both the aziridines and their borane complexes display highly negative reduction potentials, and no obvious reductive peaks were observed within the accessible potential window.

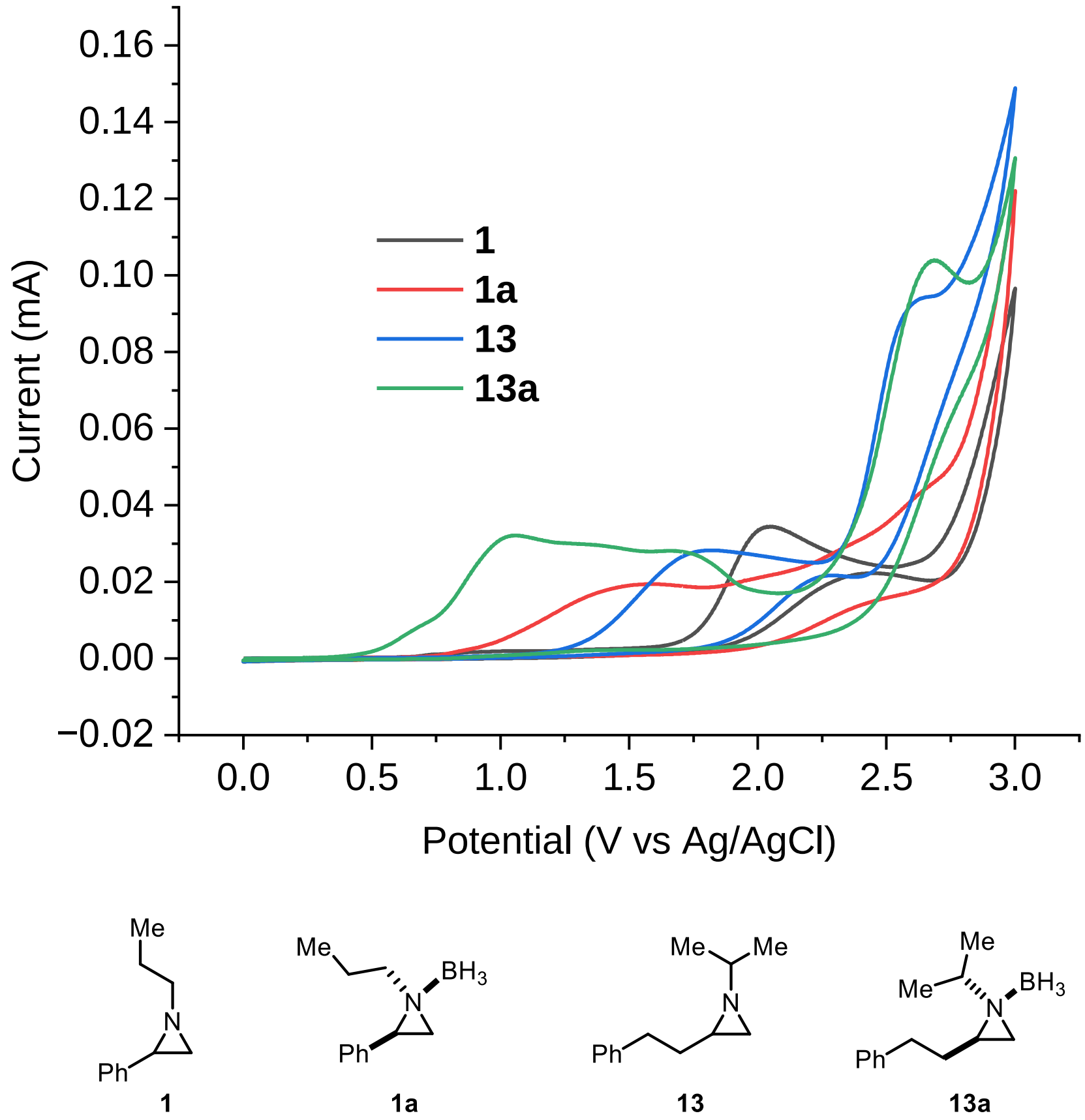

Figure S3. Cyclic voltammogram (positive current) of model aziridine and aziridine boranes in CH_3CN. A scan rate of 100 mV/s was used for all CV experiments.

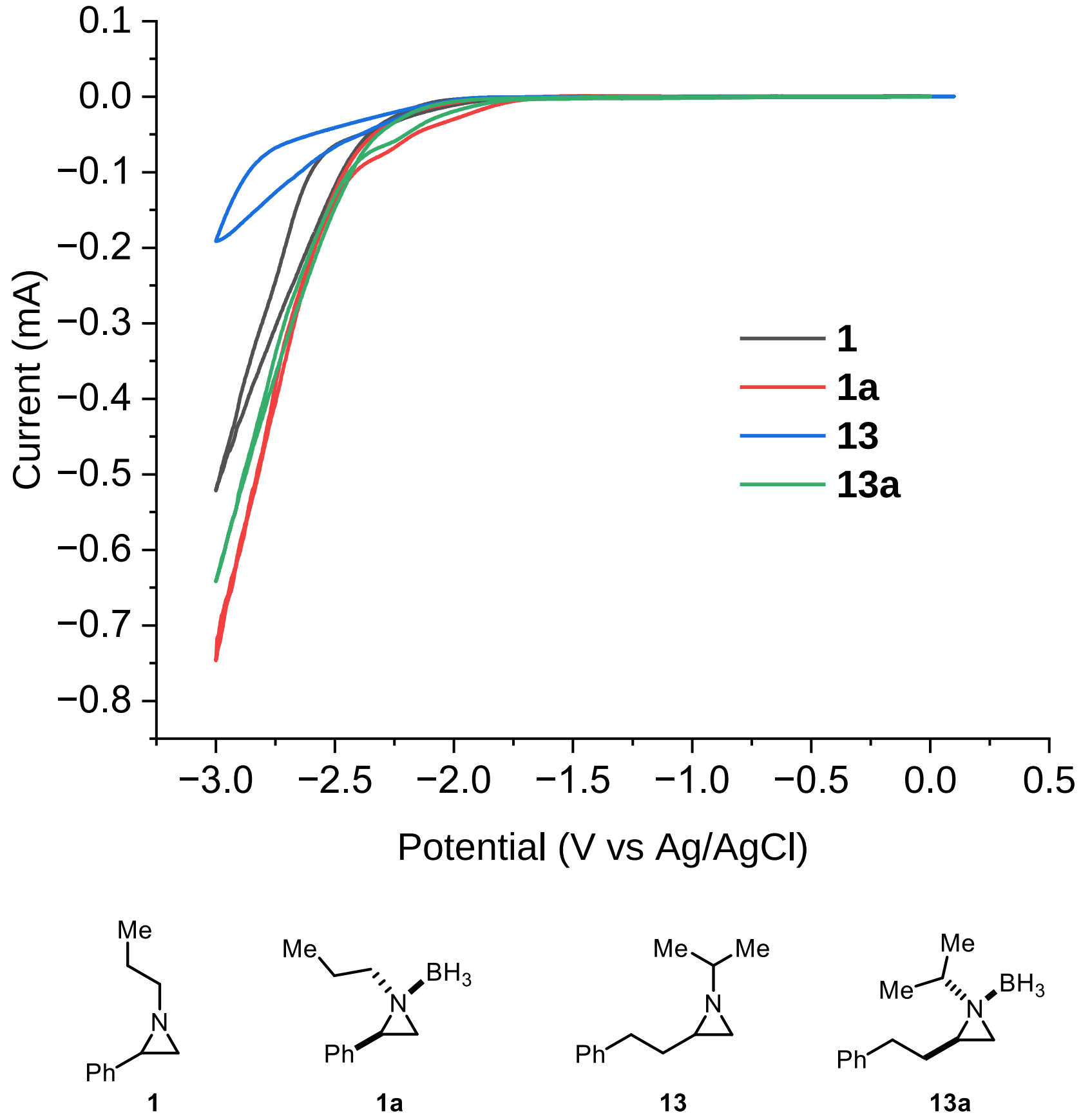

Figure S4. Cyclic voltammogram (negative current) of model aziridine and aziridine boranes in CH_3CN. A scan rate of 100 mV/s was used for all CV experiments.

10 Laser Flash Photolysis Studies

General Remarks

Laser Flash Photolysis (LFP) experiments were carried out with a laser spectrometer using the third harmonic (355 nm) of a Q-switched Nd:YAG laser, delivering 8 ns pulses. The laser energy was adjusted to 10 mJ/pulse. A 3.5 mL Suprasil quartz cell (10 mm × 10 mm) was used in all experiments. Argon saturated EtOAc–(*t*-BuO)$_2$ (3:1) or CH_3CN–(*t*-BuO)$_2$ (3:1) solutions were employed. Boryl-radicals have been generated by hydrogen atom transfer (HAT) from the pertinent amine borane precursor with *t*-BuO•.

Transient Absorption Spectroscopy Studies

To provide direct evidence regarding the formation of radical species we performed transient absorption spectroscopy (TAS) studies. The whole process can be described according to **Scheme 1**. According to our computational data, the ring-opening of the boryl radical arising from **1a** is barrierless. Indeed, when TAS studies using a solution of **1a** in EtOAc–(*t*-BuO)$_2$ (or CH_3CN) at 25 °C or –10 °C, provided only a signal at $\lambda \approx 300$ nm, which match the known absorption profile of benzylic radicals.[21] No boryl radical absorption was detected. Overall, these studies clearly confirms that the process goes via the formation of a benzylic radical and it is in line with the fast ring-opening of the aziridine-ligated boryl radical.

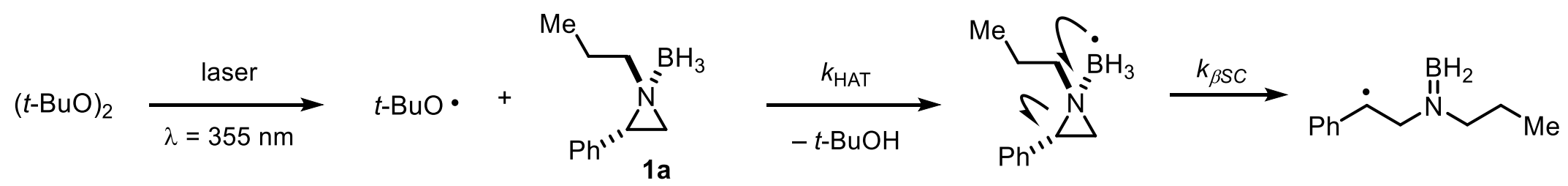

Scheme 1.

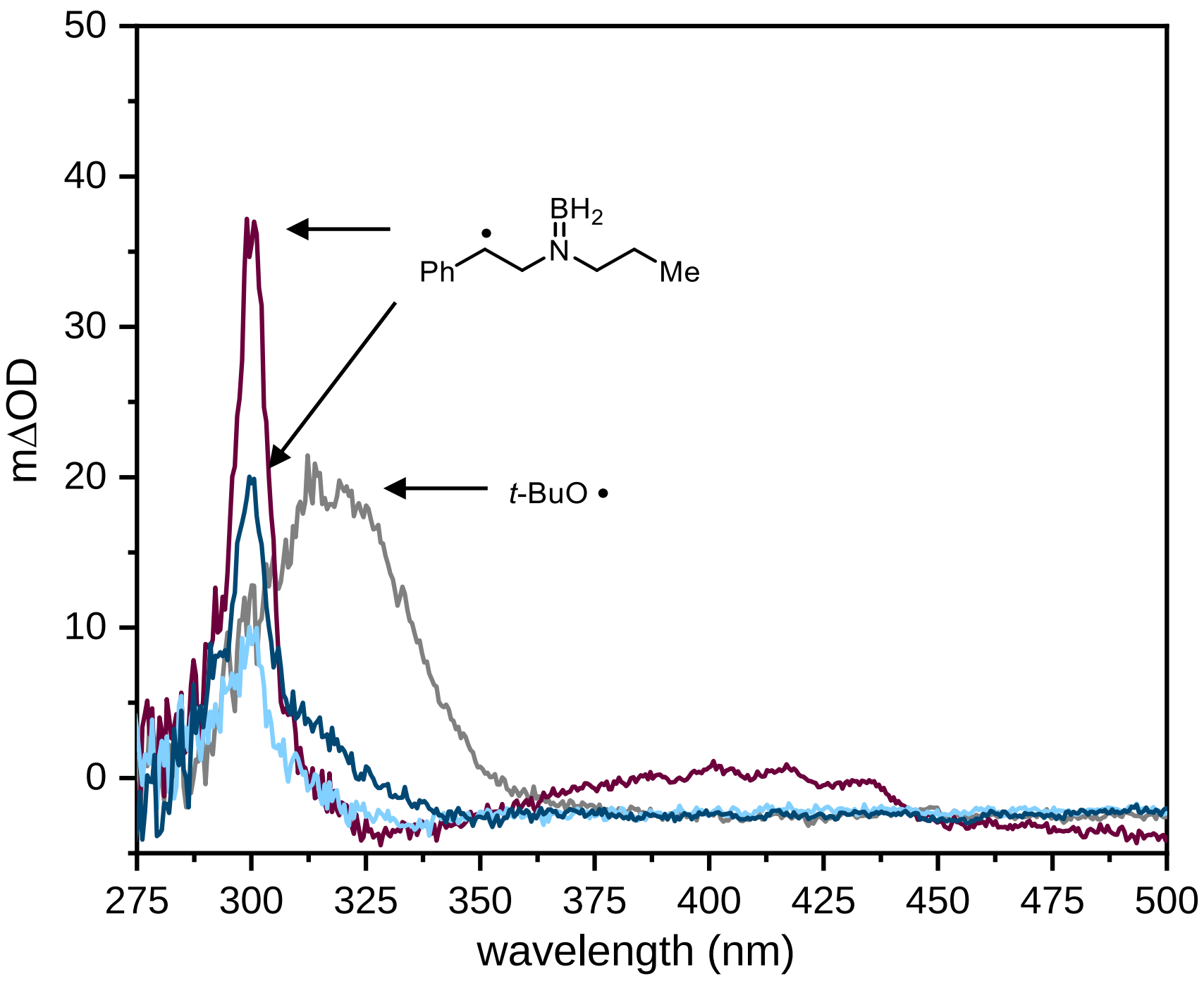

Figure S5. Transient Absorption Spectroscopy observed 200 ns after 355 nm LFP of **1a** in EtOAc–(t-BuO)$_2$ 3:1 at 25°C (red), CH_3CN–(t-BuO)$_2$ 3:1 at 25°C (dark blue), CH_3CN–(t-BuO)$_2$ 3:1 at –10°C (light blue) and of the blank solution EtOAc–(t-BuO)$_2$ 3:1 without **1a** at 25°C (grey).

11 Radical Trapping Experiment

N-(2-phenyl-2-((2,2,6,6-tetramethylpiperidin-1-yl)oxy)ethyl)propan-1-amine (S14)

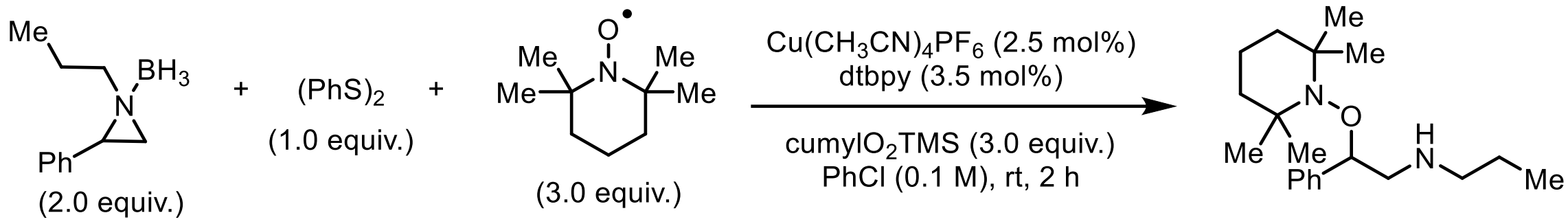

Following **GP7**, *trans*-aziridine **1a** (31 mg, 0.2 mmol, 2.0 equiv.), diphenyl disulfide (22 mg, 0.1 mmol) and TEMPO (47 mg, 0.3 mmol, 3.0 equiv.) gave **S14** as an oil (30%). R_f 0.26 [*n*-pentane:EtOAc (1:1)]; ^{1}H NMR (400 MHz, $CDCl_3$) δ 7.39–7.27 (5H, m), 5.03 (1H, br s), 3.28 (1H, dd, J = 12.1, 6.6 Hz), 2.98 (1H, dd, J = 11.2, 6.5 Hz), 2.59 (2H, t, J = 7.3 Hz), 1.61–1.47 (5H, m), 1.41–1.31 (6H, m), 1.19 (3H, br s), 1.04 (3H, br s), 0.88 (3H, t, J = 7.3 Hz), 0.81 (3H, br s); ^{13}C NMR (151 MHz, $CDCl_3$) δ 141.3, 128.4 127.9, 127.7, 84.0, 60.4, 54.9, 51.3, 49.6, 40.6, 34.4, 34.1, 29.8, 27.1, 22.7, 20.6, 17.3, 11.8; HRMS (ESI) found MH^+ 319.2742, $C_{20}H_{35}ON_2$ requires 319.2744.

Table S22. Control experiments for radical trapping.

entry	deviation	S14 (%)	1a	1
1	without Cu & dtbpy	–	99	–
2	**1** instead of **1a**	–	–	–*
3	without $(PhS)_2$	30	89	

*full decomposition of the starting amine **1**

12 Computational Studies

12.1 General Information

The computational work was carried out in the framework of density functional theory (DFT)[22,23] using the ORCA (version 5.0.4)[24] software package. For all critical points, geometry optimizations and harmonic vibrational frequencies, the Minnesota hybrid functional (U)M06-2X[25] was employed in combination with the def2-TZVP[26,27] basis set. This level of theory was applied to these studies as this functional and basis set have been used in literature to describe boryl radical processes effectively.[28-31] The self-consistent field (SCF) convergence was controlled by the 'TightSCF' keyword, and the integration grids were set with the 'defgrid3' keyword to increase numerical precision. Moreover, the default settings of this version of ORCA included the def2/J auxiliary basis set[27] and the RIJCOSX approximation in all calculations. All potential-energy minima were ensured to present only all-real vibrational frequencies. The transition state guess was determined by completing a scan of the breaking bond of interest and the optimized structure displayed only one imaginary frequency corresponding to the reaction normal mode. Intrinsic reaction coordinate (IRC) calculations were performed to confirm the connection between reactants and products through the corresponding transition states.

More accurate single-point electronic energies were calculated at the (U)M06-2X/def2-QZVP level of theory.[25-27] For the single point calculations (SPC), the solvent effects of EtOAc were included using the SMD solvation model.[32] The same level of theory was applied to determine the energy gap between orbitals and the Mayer bond order using *Gaussian 16*[33] and its implemented NBO 3.1 program.[34]

The thermodynamic data was obtained with the python script *OTHERM.py*,[35] where the thermodynamic contributions from the vibrational analysis were combined with the SPC calculations. The final thermodynamic values were corrected for the conditions at 298.15 K and with a concentration of 1.0 mol L^{-1}. All Cartesian coordinates of the geometries and their corresponding energies can be found in the Supplementary Material file.

12.2 Thermodynamics

Thermodynamic data and the SOMO-σ* orbital energy gap in EtOAc give an insight into the regioselectivity of the ring-opening step for the *N*-Me-aziridine-ligated boryl radical bearing either a β-Me or a β-Ph substituent (**Figure S6**). For the β-Me substituted species:

Trans. The secondary radical is the preferred thermodynamic and kinetic product, although showing a larger orbital gap between SOMO and the σ* of the breaking C–N bond. The energetics result in the reaction being regioselective thanks to the strain release between the substituents.

Cis. As in the *trans*-conformation, the SOMO-σ* energy gap is lower for the formation of the primary radical. Moreover, the primary and secondary radical products present a similar activation free energy ($\Delta\Delta G^{\ddagger}$ = 0.7 kcal mol^{-1}), with the more hindered position being favored thermodynamically ($\Delta\Delta G^0$ > 3 kcal mol^{-1}). Hence, due to the promiscuous parameters, a less regioselective reaction is likely to occur, leading to a mixture of both products.

The trends mentioned generally agree with the observation of Roberts[36] with *N*-H-aziridine-ligated boryl radical processes, with the *trans*-conformer leading to a more regioselective ring opening than the *cis*-species.

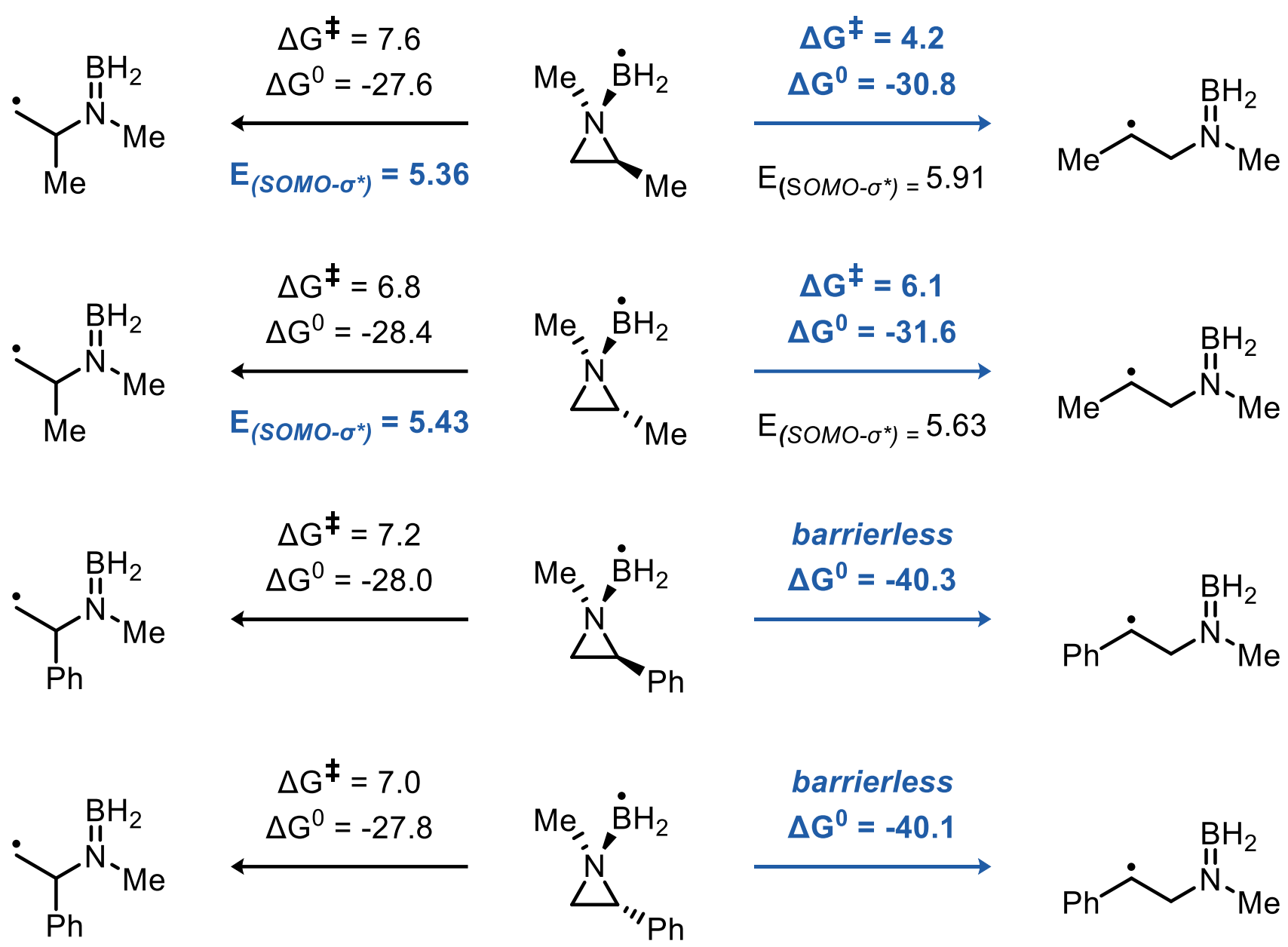

Figure S6. Gibbs free energy change at the transition state ($\Delta G^{\ddagger}$), Gibbs free energy change of the ring-opening (ΔG^0) in kcal mol^{-1}, followed by the energy gap of the SOMO and the σ* of the breaking C–N bond in eV. (U)M06-2X/def2-QZVP(SMD,EtOAc)//(U)M06-2X/def2-TZVP

The β-Ph *N*-Me-aziridine-ligated boryl radical favors the benzyl radical regardless of the conformation of the initial ring, suggesting that the stability of the radical product trumps the conformation of the initial species (**Figure S7**). Energy scans of the breaking C–N bond for all

species were employed to locate the transition state geometry guess. In the case of the β-Ph substituent with the ring-opening towards the formation of the benzyl radical, both *trans*- and *cis*-conformations resulted in a small electronic energy difference of ΔE ≈ 1 kcal mol^{-1} (**Figure S7**). However, transition state optimizations initiated from the scan maximum did not converge to a first-order saddle point and instead relaxed directly toward products, therefore deeming the reaction "barrierless".

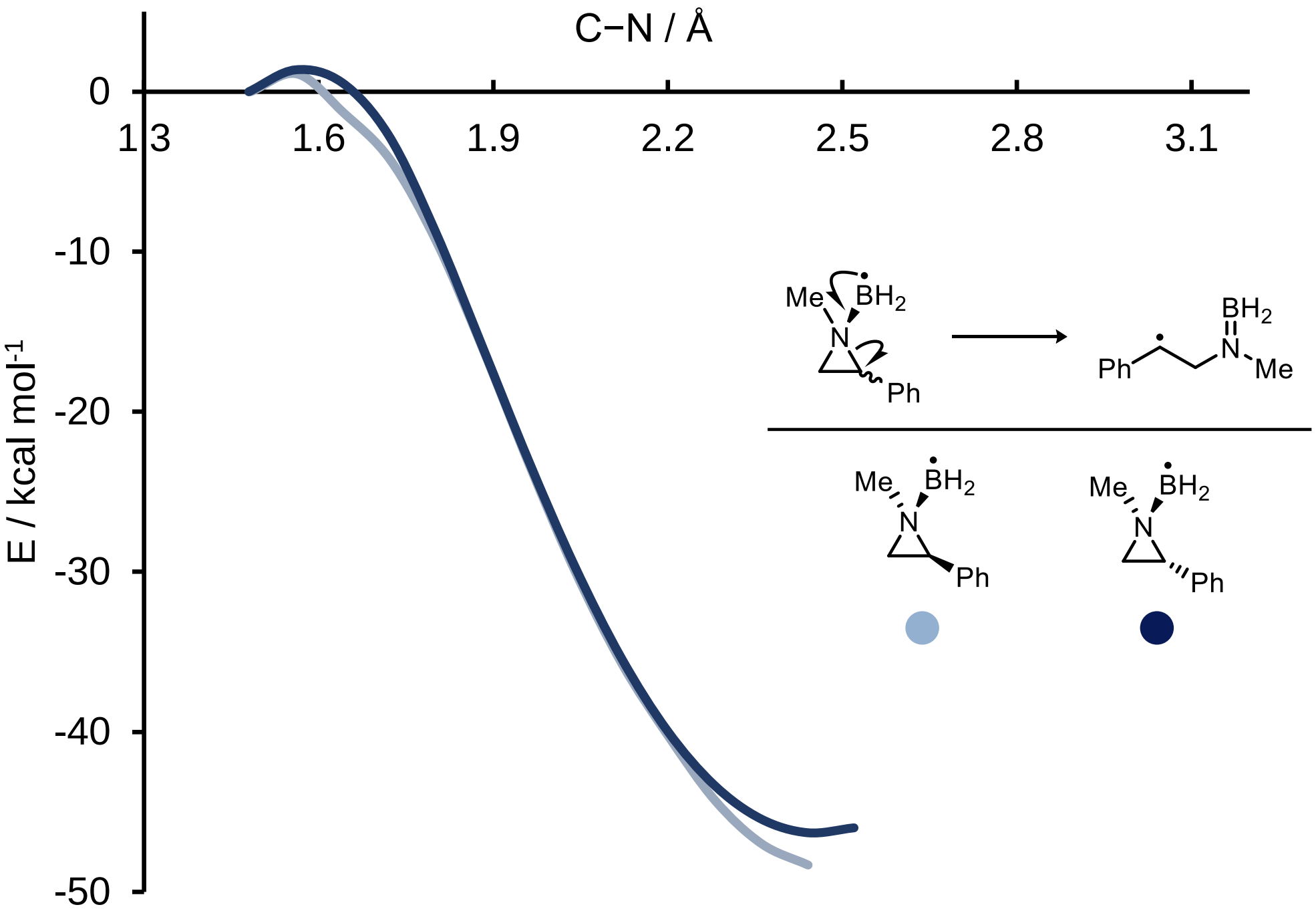

Figure S7. Energy scan of breaking the C−N bond for both conformations (*cis* and *trans*) of the *N*-Me-aziridine-ligated boryl radical, bearing a β-Ph substituent, ring opening step to yield the benzyl radical product. (U)M06-2X/def2-TZVP

12.3 Chemical Properties Across the Potential Energy Surface

Table S23. Data of the *trans*-*N*-Me-aziridine-ligated boryl radical, and its two possible radical products, bearing a β-methyl substituent in EtOAc.

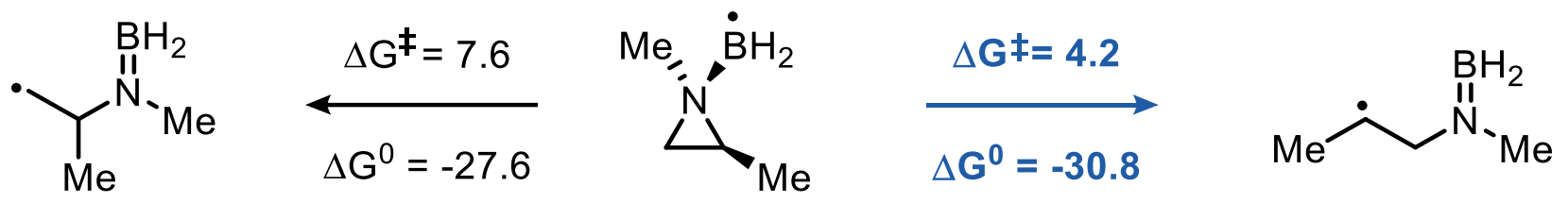

R	Species	B–N (Å)	Mayer BO B–N	C–N (Å)	Mayer BO C–N	Spin Density (C)	Spin Density (B)	$E_{SOMO\text{-}\sigma^*}$ (eV)
1°	SM	1.57	0.70	1.46	0.89	-0.05	1.01	-
Radical	TS	1.49	0.90	1.71	0.66	0.24	0.88	5.36
Formation	P	1.39	1.32	2.44	-	1.01	0.00	-
2°	SM	1.57	0.70	1.49	0.87	0.06	1.01	-
Radical	TS	1.50	0.87	1.68	0.67	0.22	0.89	5.91
Formation	P	1.39	1.31	2.47	-	1.02	0.00	-

Table S24. Data of the *cis*-*N*-Me-aziridine-ligated boryl radical, and its two possible radical products, bearing a β-methyl substituent in EtOAc.

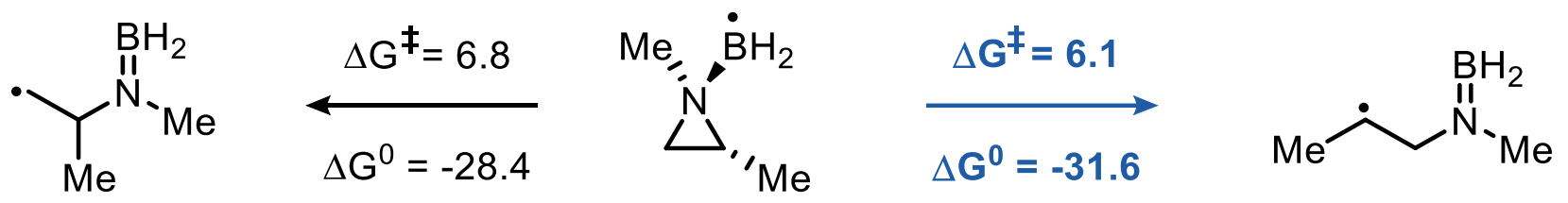

R	Species	B–N (Å)	Mayer BO B–N	C–N (Å)	Mayer BO C–N	Spin Density (C)	Spin Density (B)	$E_{SOMO\text{-}\sigma^*}$ (eV)
1°	SM	1.57	0.70	1.48	0.87	0.00	1.05	-
Radical	TS	1.49	0.90	1.70	0.67	0.23	0.90	5.43
Formation	P	1.39	1.32	2.44	-	1.01	0.00	-
2°	SM	1.57	0.70	1.47	0.88	-0.05	1.05	-
Radical	TS	1.49	0.86	1.72	0.90	0.20	0.92	5.63
Formation	P	1.39	1.31	2.47	-	1.02	0.00	-

Table S25. Data of the *trans*-*N*-Me-aziridine-ligated boryl radical, and its two possible radical products, bearing a β-Ph substituent in EtOAc.

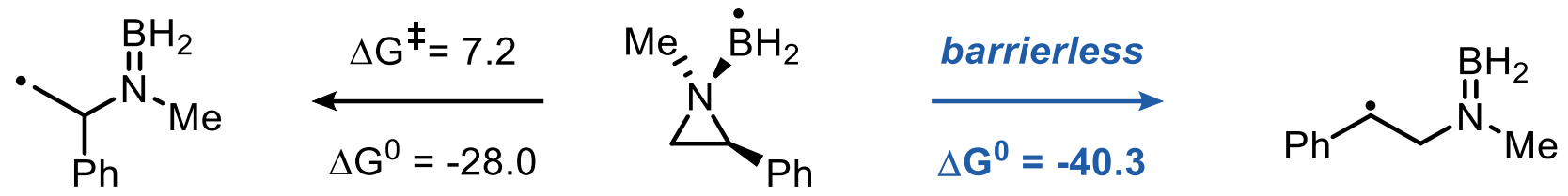

R	Species	B–N (Å)	Mayer BO B–N	C–N (Å)	Mayer BO C–N	Spin Density (C)	Spin Density (B)
1°	SM	1.57	0.69	1.48	0.87	-0.02	0.96
Radical	TS	1.49	0.89	1.71	0.66	0.21	0.85
Formation	P	1.39	1.32	2.45	-	0.97	0.00
2°	SM	1.57	0.69	1.48	0.86	0.04	0.96
Radical	TS	-	-	-	-	-	-
Formation	P	1.39	1.30	2.48	-	0.89	0.00

Table S26. Data of the *cis*-*N*-Me-aziridine-ligated boryl radical, and its two possible radical products, bearing a β-Ph substituent in EtOAc

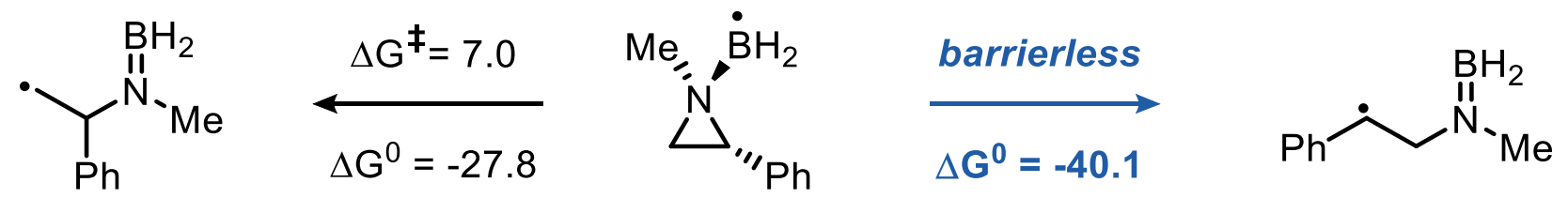

R	Species	B–N (Å)	Mayer BO B–N	C–N (Å)	Mayer BO C–N	Spin Density (C)	Spin Density (B)
1°	SM	1.57	0.72	1.48	0.88	-0.01	1.00
Radical	TS	1.48	0.91	1.71	0.65	0.23	0.84
Formation	P	1.39	1.32	2.45	-	0.97	0.00
2°	SM	1.57	0.72	1.48	0.86	0.01	1.00
Radical	TS	-	-	-	-	-	-
Formation	P	1.39	1.30	2.48	-	0.89	0.00

12.4 Visual Representation of Orbital Overlap at the Transition State

For the β-Me-substituted borylated aziridines, visual representations of the single occupied molecular orbital (SOMO) and the σ* of the breaking C–N bond at the transition state geometry are displayed below. The energies (eV) of the orbitals were used to determine the energy gap to predict the regioselectivity of the ring-opening step, as previously described by Roberts[36] using similar motifs.

a. b. c.

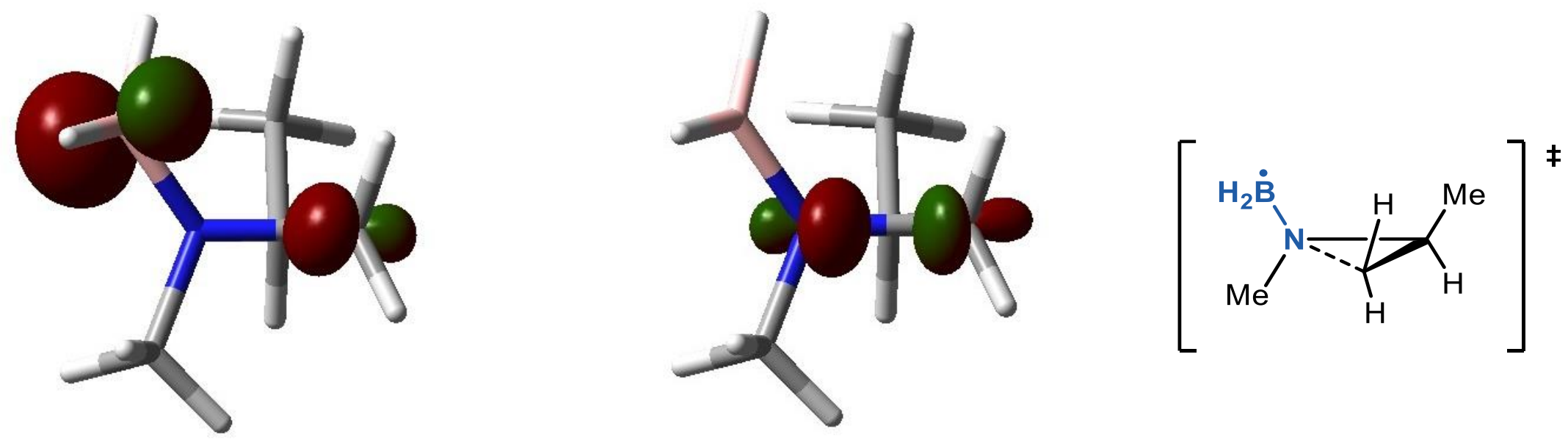

Figure S8. SOMO (a) and σ* orbital of the C–N bond (b) of the *trans*-*N*-Me-aziridine-ligated boryl radical at the transition state (c) to form the primary radical as product in EtOAc. Grey = carbon; white = hydrogen; blue = nitrogen; pink = boron.

a. b. c.

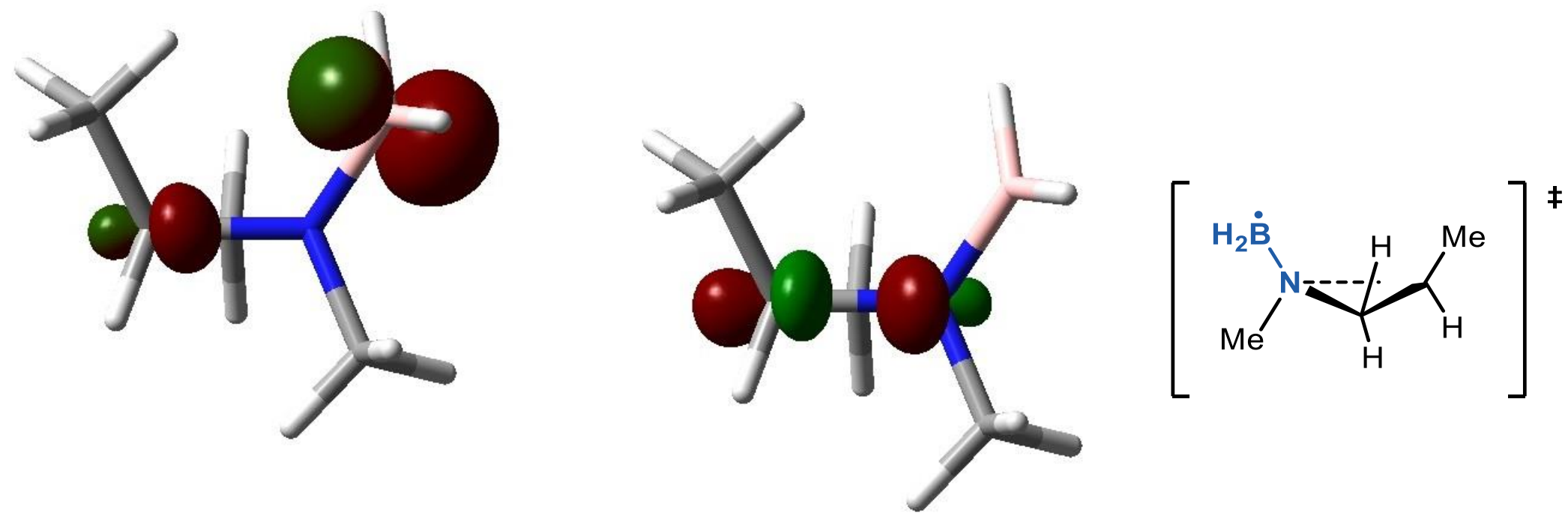

Figure S9. SOMO (a) and σ* orbital of the C–N bond (b) of the *trans*-*N*-Me-aziridine-ligated boryl radical at the transition state at the transition state (c) to form the secondary radical as product in EtOAc. Grey = carbon; white = hydrogen; blue = nitrogen; pink = boron.

a. b. c.

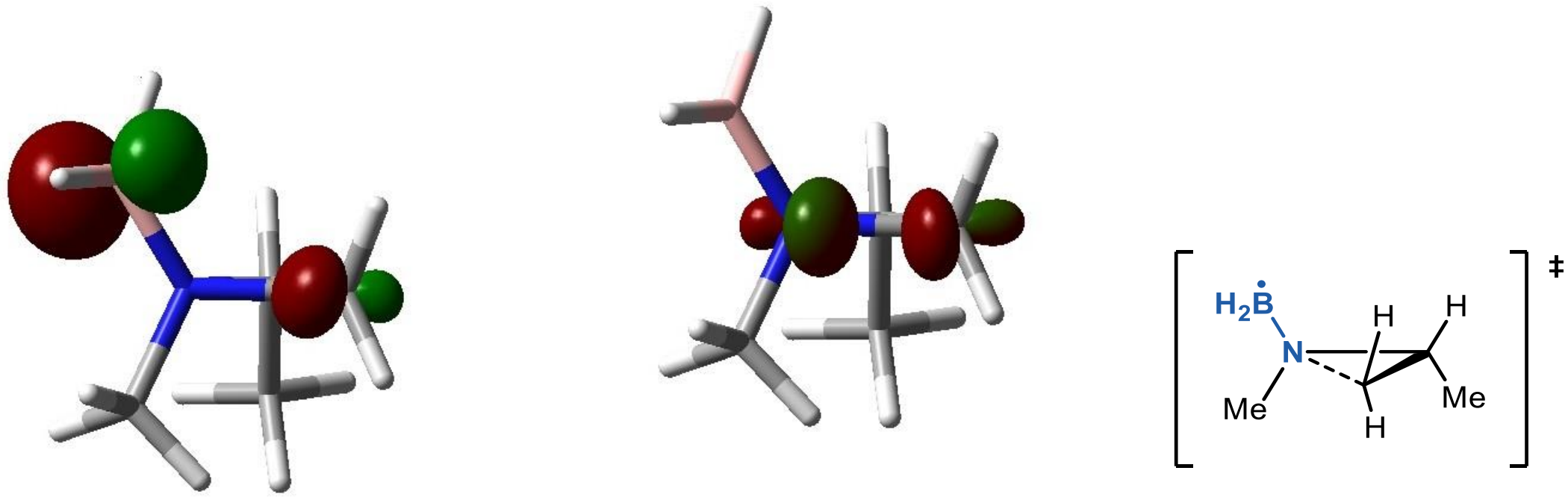

Figure S10. SOMO (a) and σ* orbital of the C–N bond (b) of the *cis*-*N*-Me-aziridine-ligated boryl radical at the transition state (c) to form the primary radical as product in EtOAc. Grey = carbon; white = hydrogen; blue = nitrogen; pink = boron.

a. b. c.

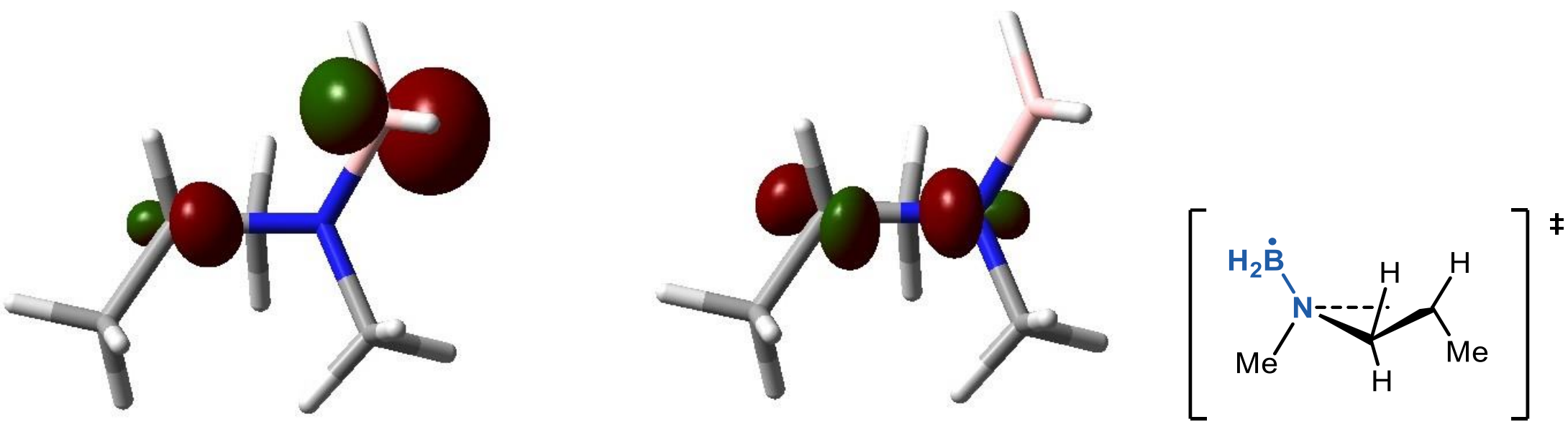

Figure S11. SOMO (a) and σ* orbital of the C–N bond (b) of the *cis*-*N*-Me-aziridine-ligated boryl radical at the transition state (c) to form the secondary radical as product in EtOAc. Grey = carbon; white = hydrogen; blue = nitrogen; pink = boron.

13 Literature Review of Ring-Opening Reactions of *N*-alkyl aziridines

13.1 Background

In the context of aziridine ring-opening chemistry, most studies rely on the use of electron-withdrawing *N*-activating protecting groups (mainly sulfonamide, see **Figure S12**) to activate the C–N bond.

While *N*-alkyl aziridines retain considerable ring strain, their inherent reactivity is low unless activated upon either protonation with strong Brønsted acids or coordination with Lewis acids (**Figure S12**). As a result, ring-opening reactions involving *N*-alkyl aziridines are less common and often restricted by the range of compatible nucleophiles. In particular, while methods for ring-opening and divergent cross-couplings have been developed for *N*-Ts-aziridines, to the best of our knowledge, *N*-alkyl aziridines cannot be engaged.

Below, we have summarized the state-of-the-art in ring-opening functionalization reactions of *N*-alkyl aziridines.

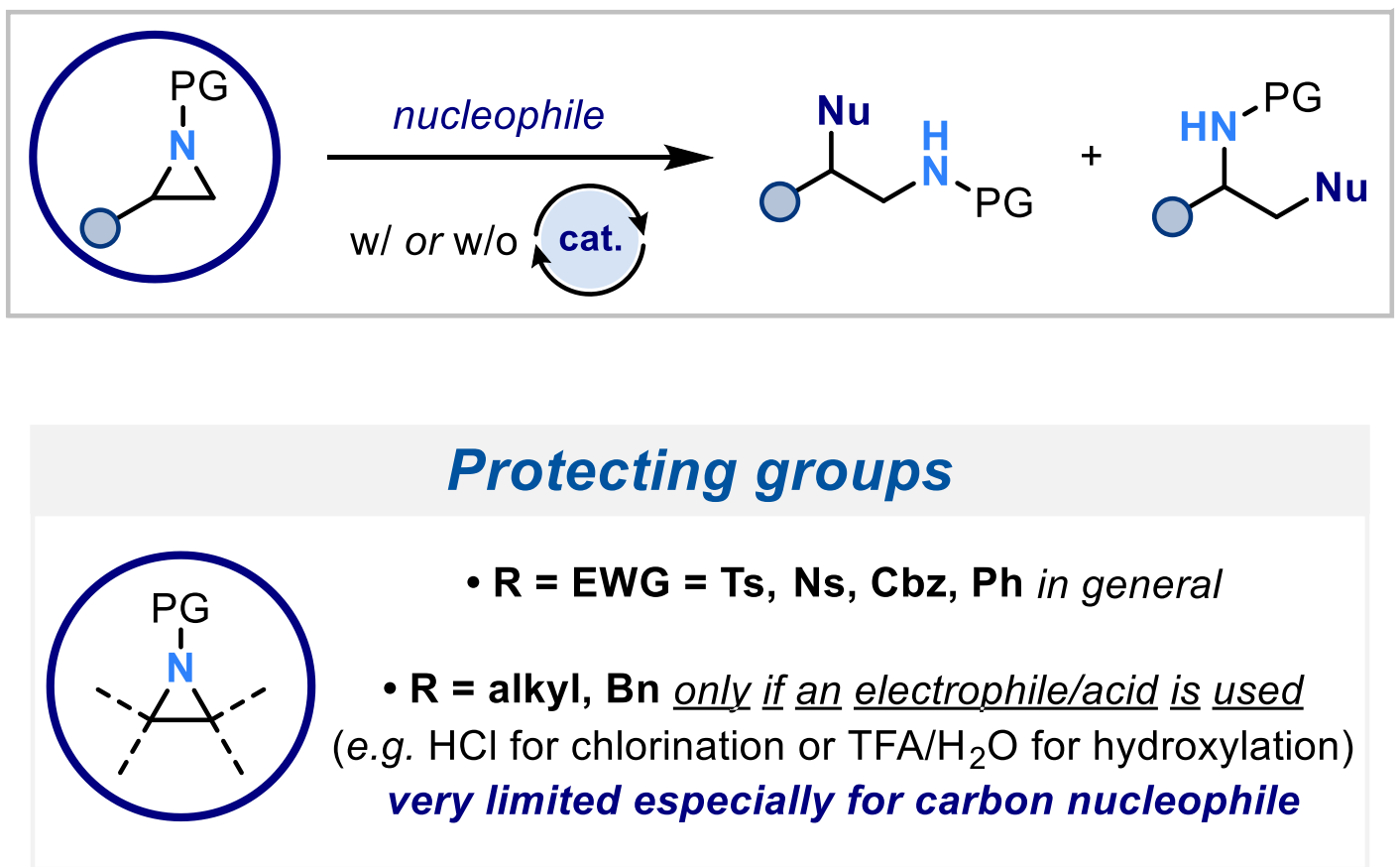

Figure S12. General aziridine ring-opening transformations scheme and commonly used protecting groups.

13.2 Nucleophilic Ring Opening[37-39]

Thiols can be used, and they lead often to nucleophilic attack at either the benzylic or at the least substituted position (**Figure S13**)[40-42]. These approaches are often rationalised on the basis of the basic aziridine N-atom undergoing deprotonation or H-bonding interaction with the thiol that synergistically activates the aziridine electrophile and the thiol nucleophile. The same reactivity featuring substitution at the least substituted site has been reported via the use of Bronsted/Lewis acids (**Figure S13**).[43,44] Please note that the latter is the opposite selectivity developed in our work.

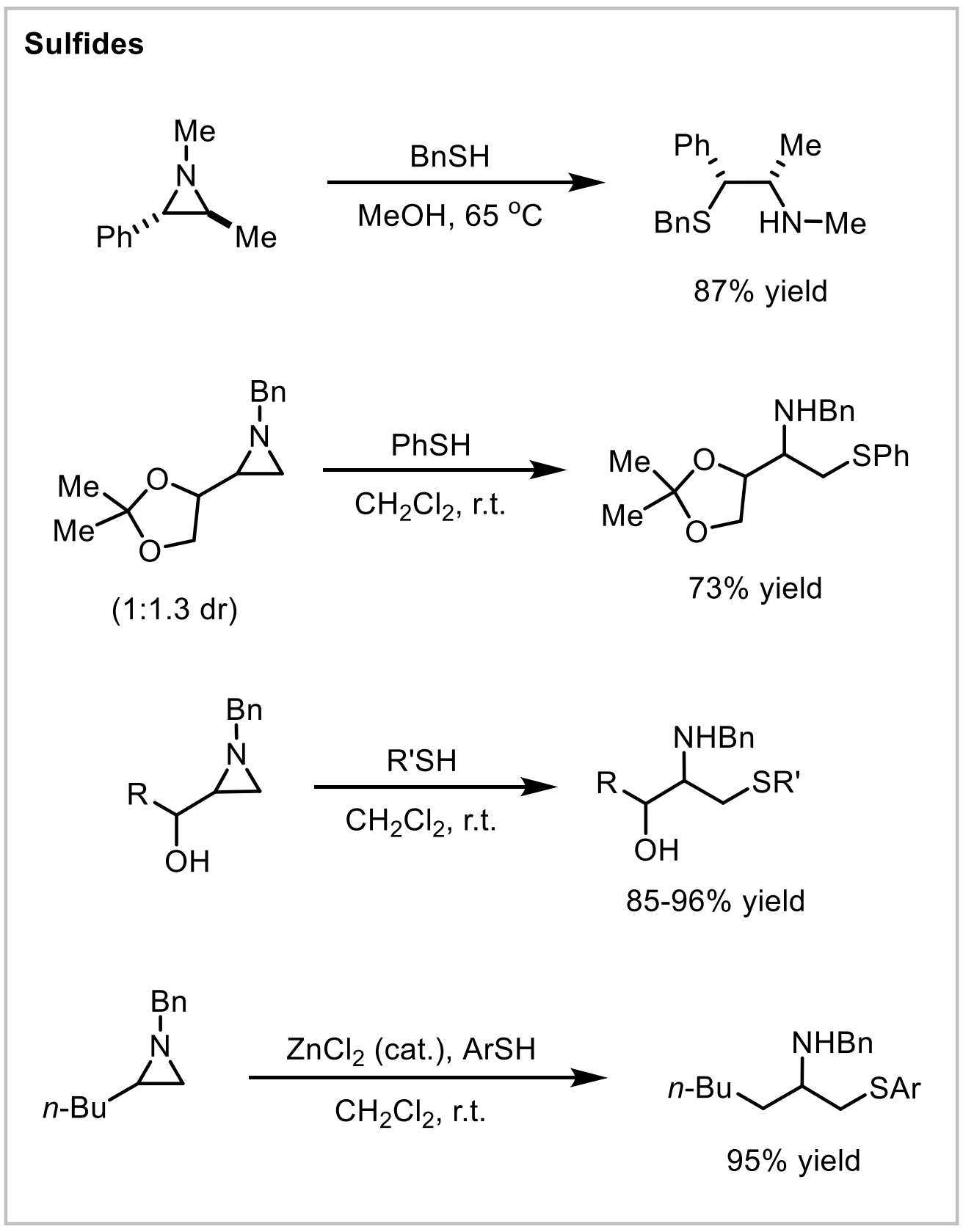

Figure S13. Summary of thioetherification ring-opening transformations involving *N*-alkyl aziridines.

Similarly to thiols, trimethylsilyl azide ($TMSN_3$) is able to directly open *N*-alkyl aziridines with various alkyl and aryl substituents (**Figure S14**), displaying a comparatively broad substrate scope and high yields.[45] Also in this case, β-alkyl aziridines undergo ring-opening at the least substituted side.

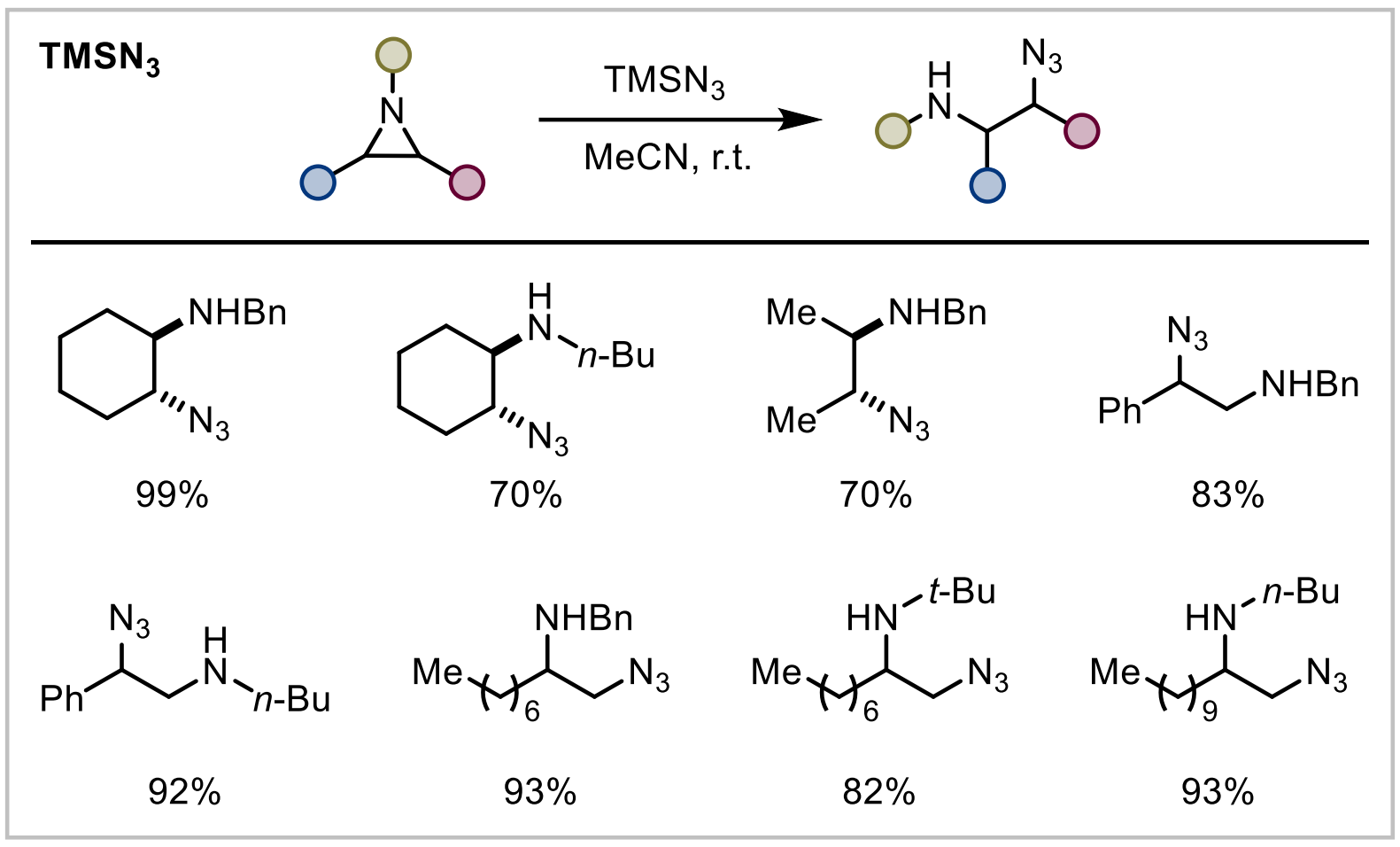

Figure S14. Summary of azidation ring-opening transformations involving *N*-alkyl aziridines.

HF-pyridine[46], TFA in a H_2O–acetone solvent mixture, and AcOH[47] can function as both Brønsted acids and nucleophiles to promote aziridine ring opening reactions (**Figure S15**). Similar methodologies have been reported with HBr and HI.[37,38] In this case, the ring-opening selectivity is less general and depends on both the nucleophile and the aziridine structure.

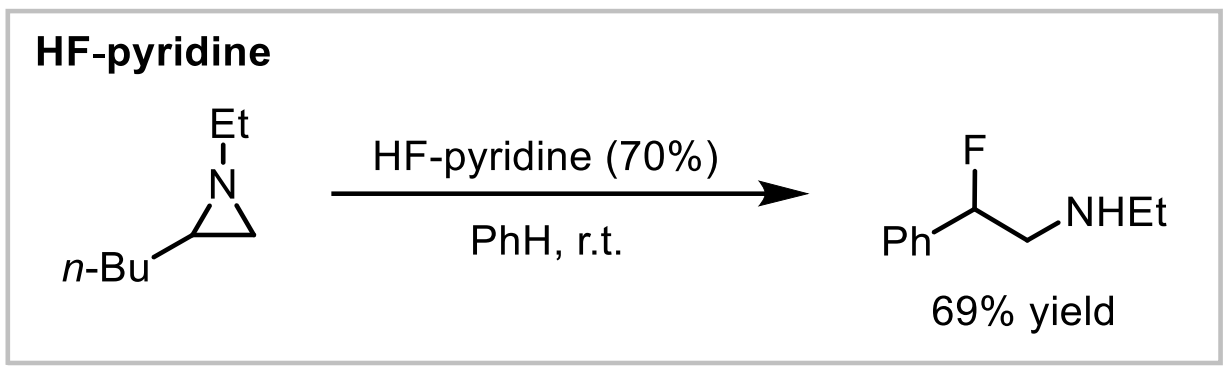

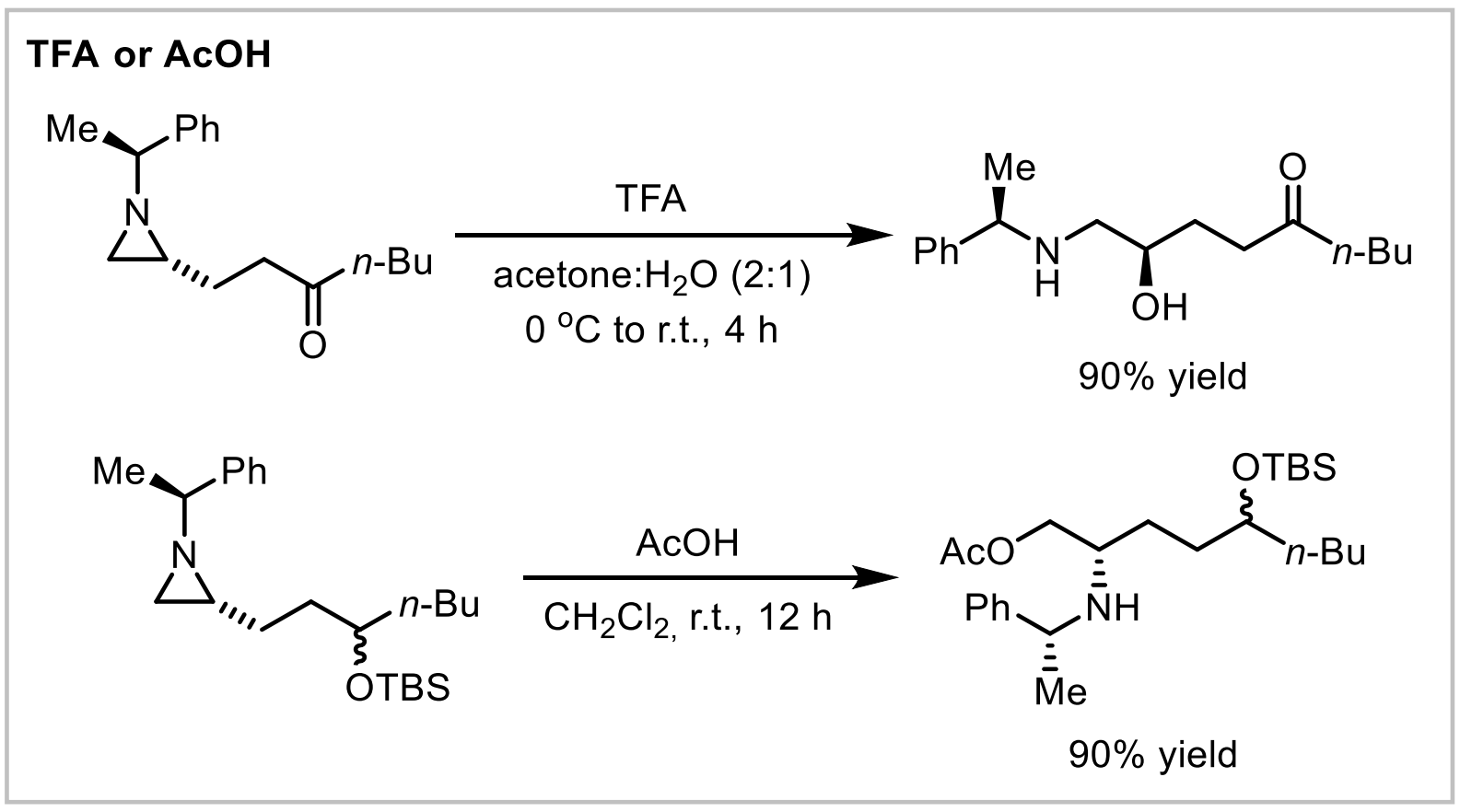

Figure S15. Summary of fluorination, hydroxylation and acetoxylation ring-opening transformations involving *N*-alkyl aziridines.

The use of cyanide as the nucleophile is well established for *N*-Ns-aziridines,[48-51] while the use of *N*-alkyl aziridine requires the use of strong Lewis acids (e.g. Et_2AlCN) or forcing conditions (microwave).[52-54]

Another mode of activation relies on N-quaternarization using either benzyl or methyl halides. These electrophiles can readily react with the nucleophilic nitrogen of *N*-alkyl aziridines to form highly electrophilic aziridinium intermediates, which can subsequently undergo efficient nucleophilic ring opening by a nucleophile present in the reaction medium (often the leaving group derived from the electrophile used) (**Figure S16**).[39,55-58] However, this strategy is limited to the formation of tertiary alkyl amines, and in the case of β-substituted *N*-alkyl aziridines, regioselectivity during the ring opening of the corresponding aziridinium intermediate can become a major issue. Overall, these two limitations curtail the broader applicability of this approach.

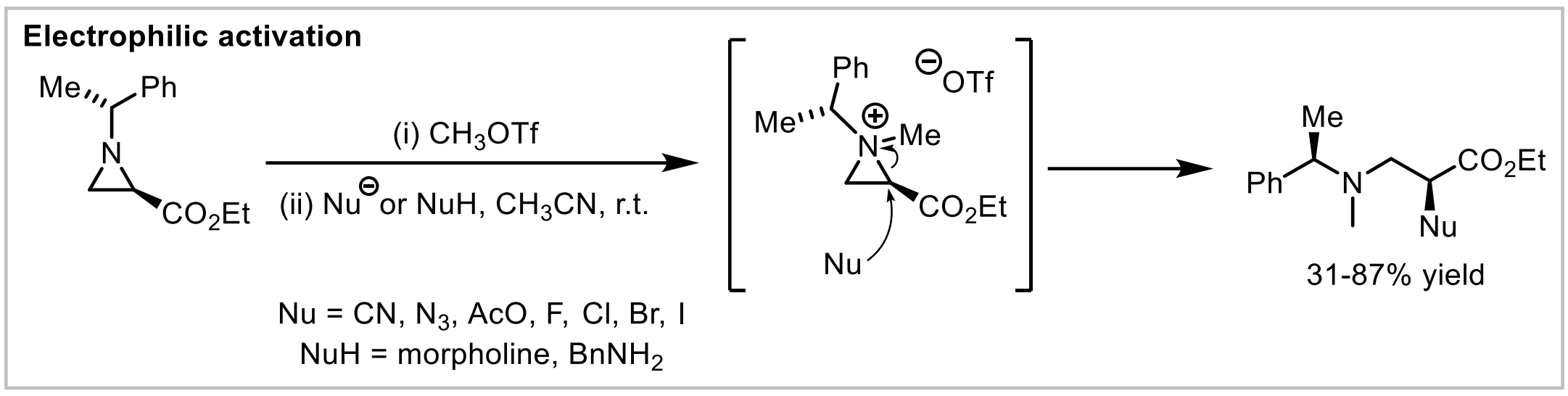

Figure S16. Summary of ring-opening transformations involving electrophilic activation.

The only example involving a carbon nucleophile was reported by Baldwin *et al.*[59] but required a β-ester substituent and the use of a higher order butylcuprate or *n*-BuMgCl in the presence of BF_3•Et_2O. However, the process led to a mixture of ring-opening products in low yield (**Figure S17**).

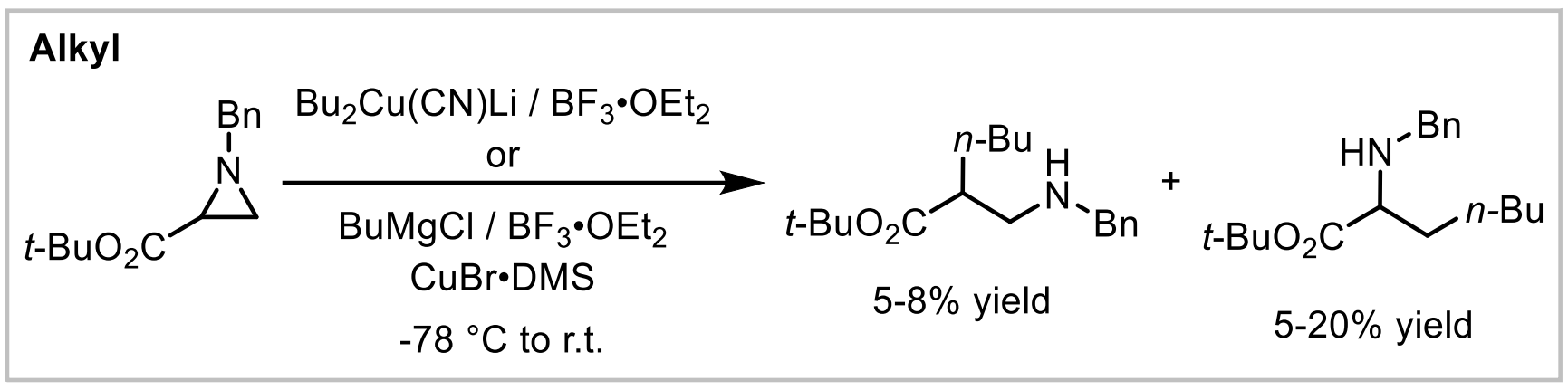

Figure S17. Summary of ring-opening transformations involving *N*-alkyl aziridines with cyanide anions.

Overall, reported ring-opening reactions of *N*-alkyl aziridines remain largely limited to strong nucleophiles and often suffer from poor generality. Consequently, general, mild, and broadly applicable catalytic systems for the ring opening of *N*-alkyl aziridines are still scarce, underscoring a clear opportunity for further methodological development.

13.3 Metal-Catalysed Ring Opening[60]

To our knowledge, only two documented approaches exist in the literature for the ring-opening of *N*-alkyl aziridines. The first method requires extremely forcing conditions and relies on a directing group to achieve the transformation (depicted in the top section of **Figure S18**). The second approach involves a specific reactivity pattern, where carbon monoxide is inserted into *N*-alkyl aziridines to produce a range of β-lactam products (illustrated in the bottom section of **Figure S18**).[61,62]

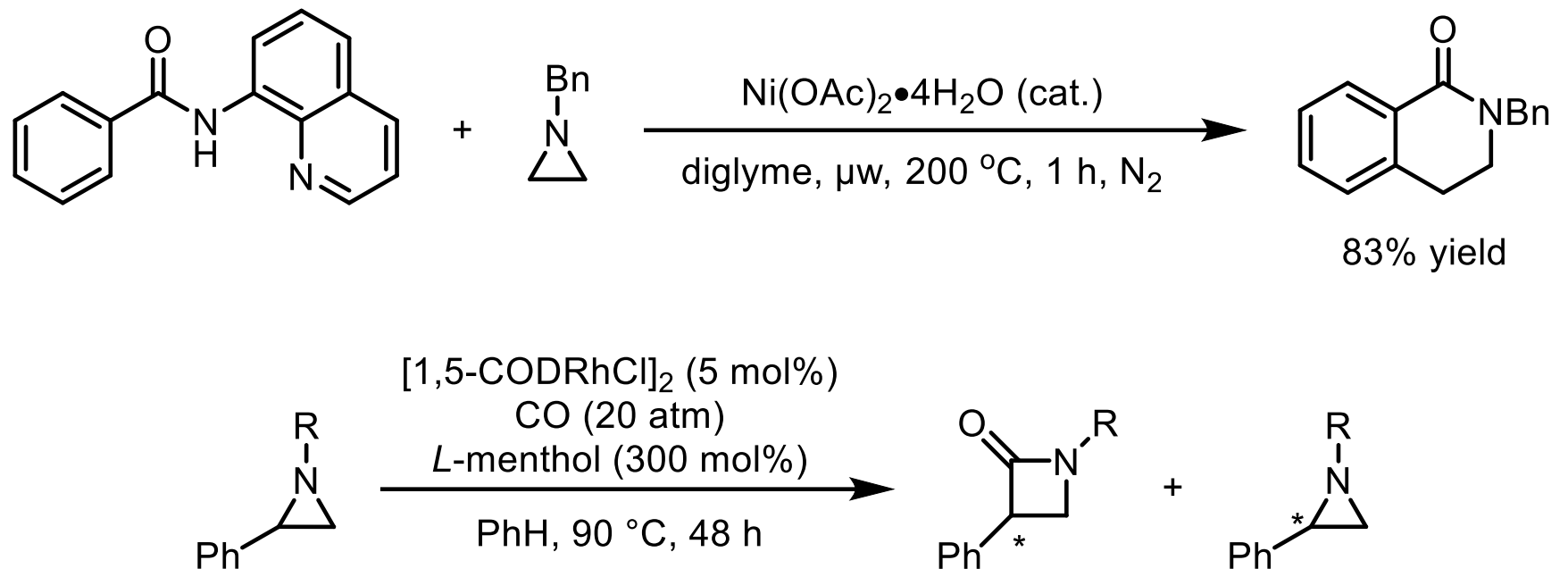

Figure S18. Summary of metal-catalysed ring opening reaction of *N*-alkyl aziridines.

14 NMR Spectra

S2a – ^{1}H NMR (600 MHz, $CDCl_3$)

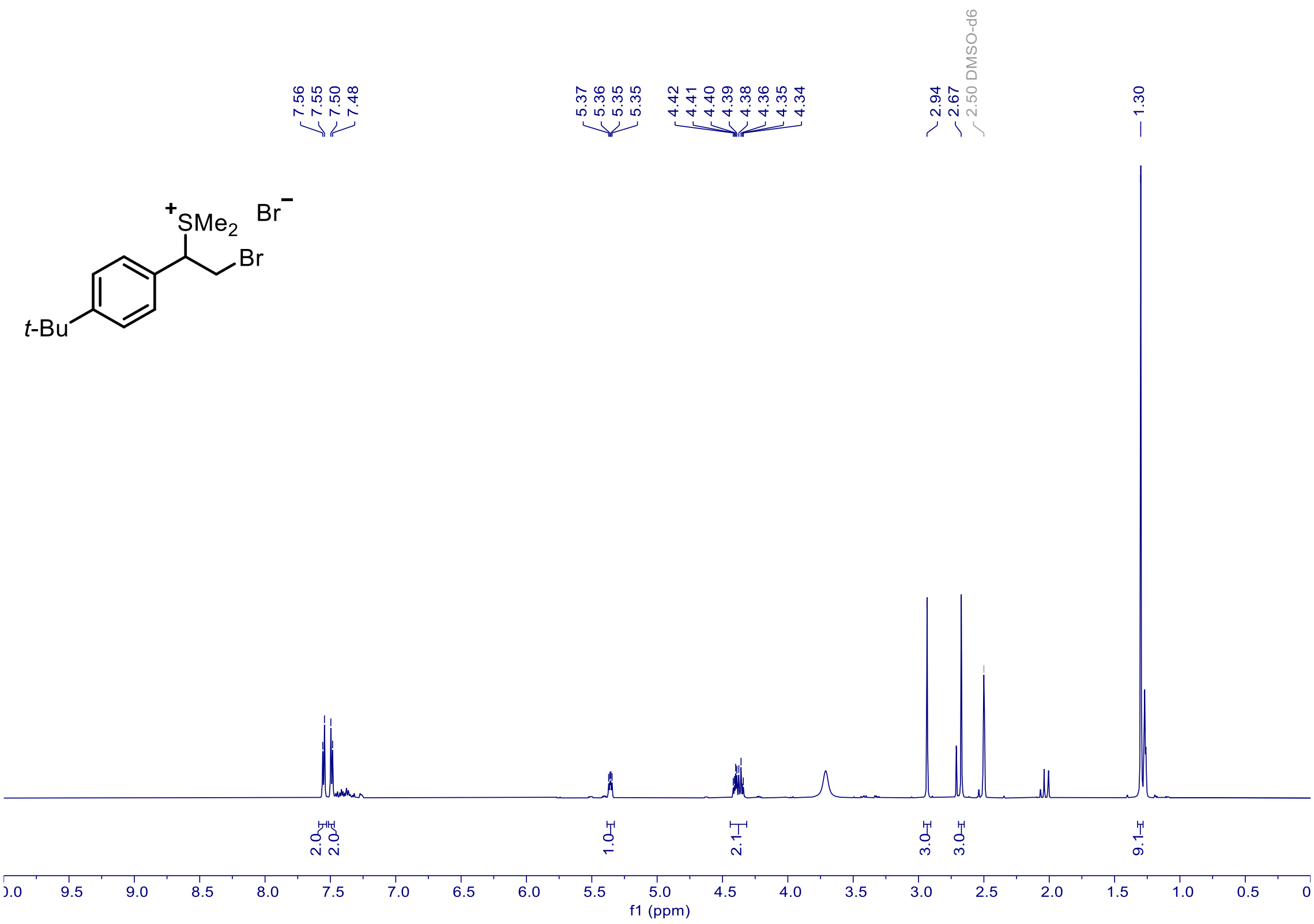

S2a – ^{13}C NMR (151 MHz, $CDCl_3$)

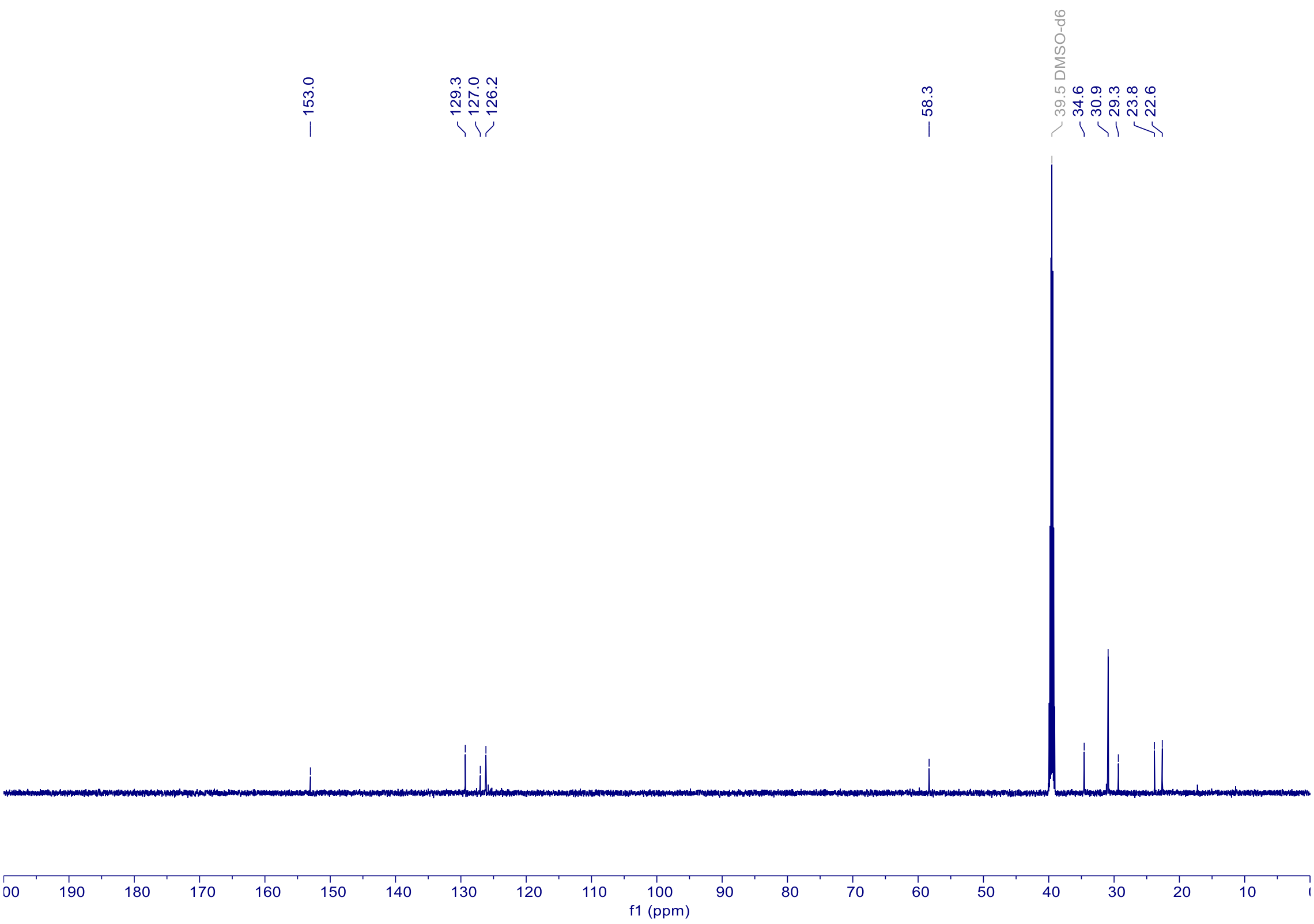

S3a – ^{1}H NMR (600 MHz, DMSO-d_6)

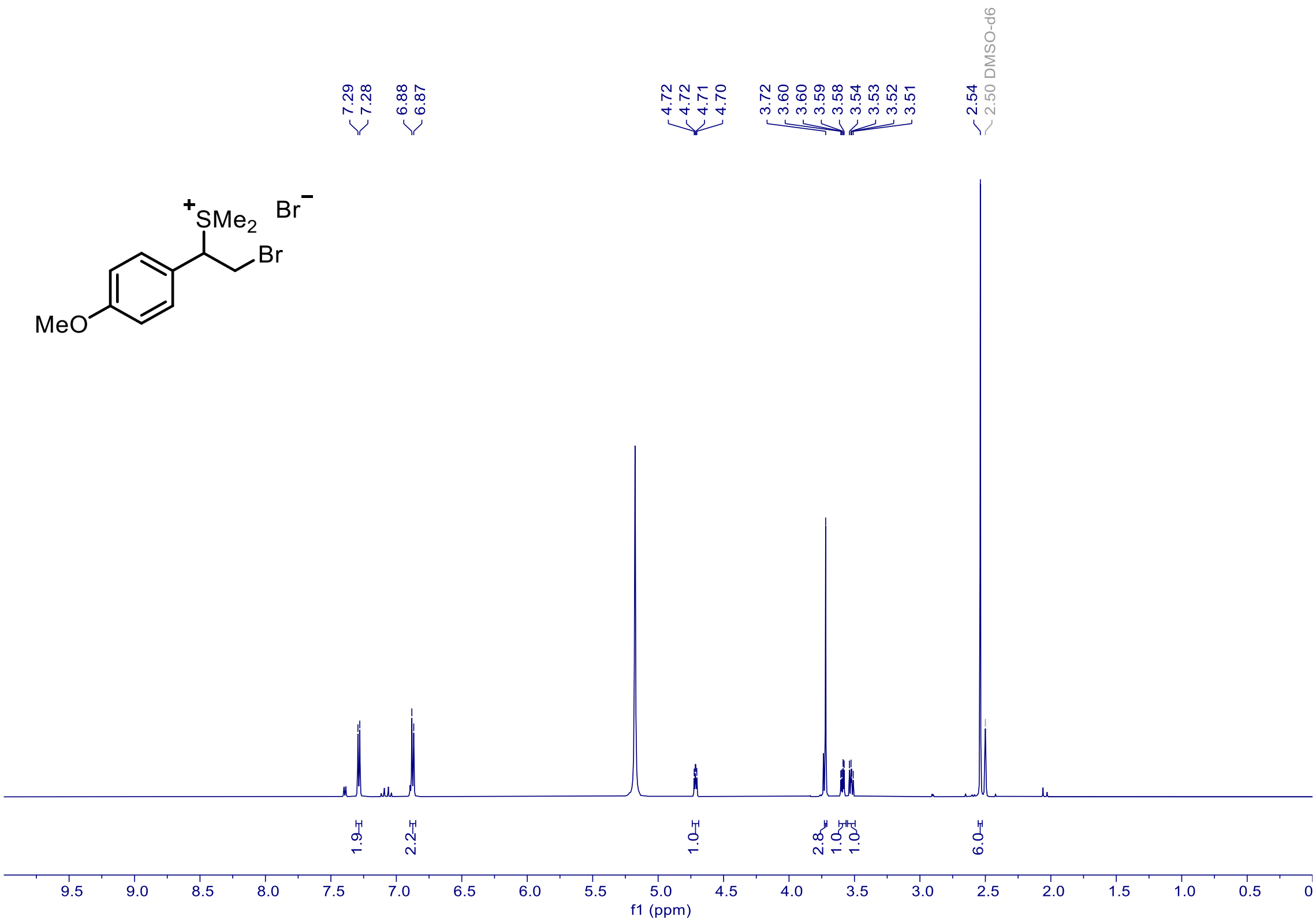

S3a – ^{13}C NMR (151 MHz, DMSO-d_6)

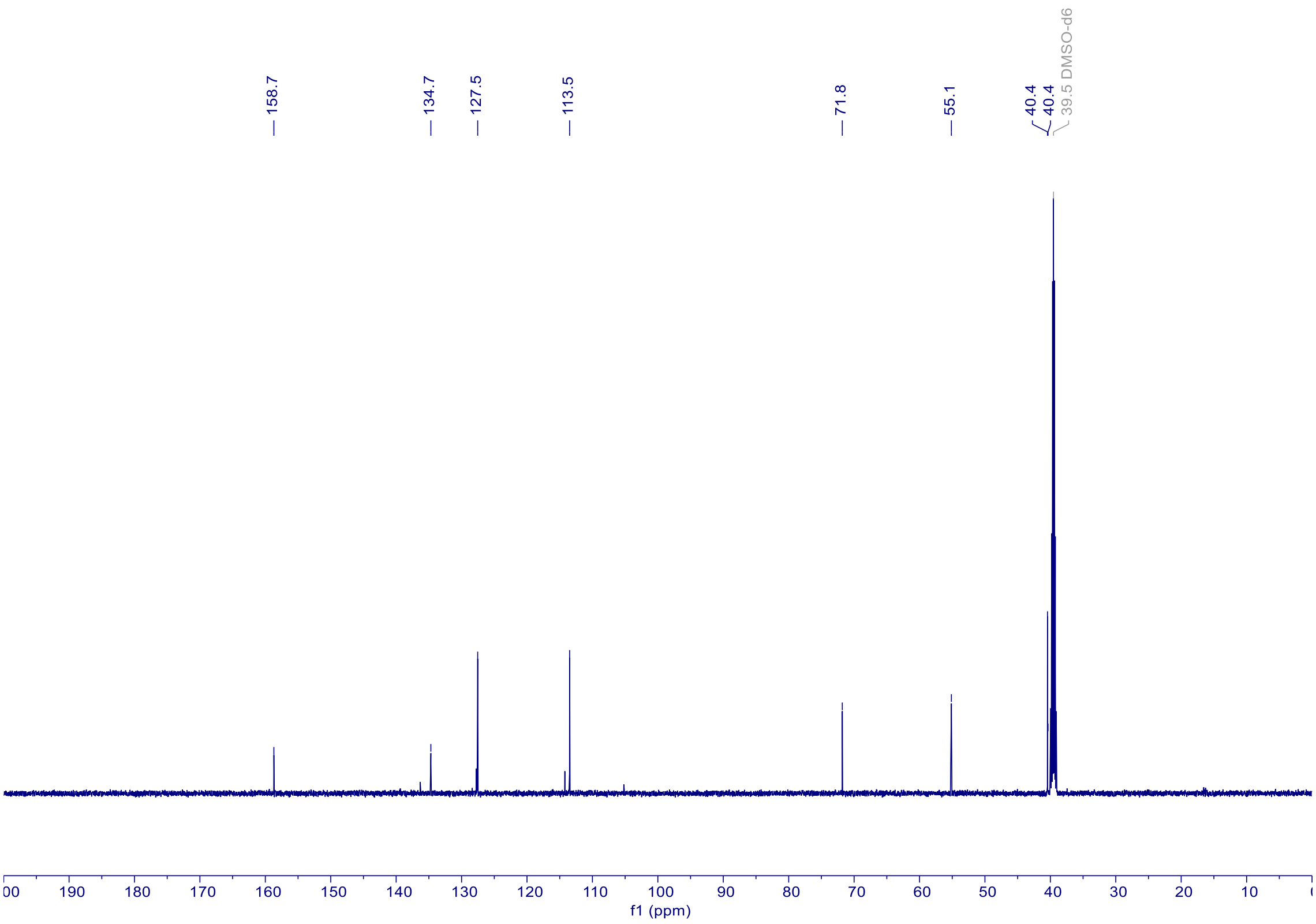

S5a – ^{1}H NMR (600 MHz, DMSO-d_6)

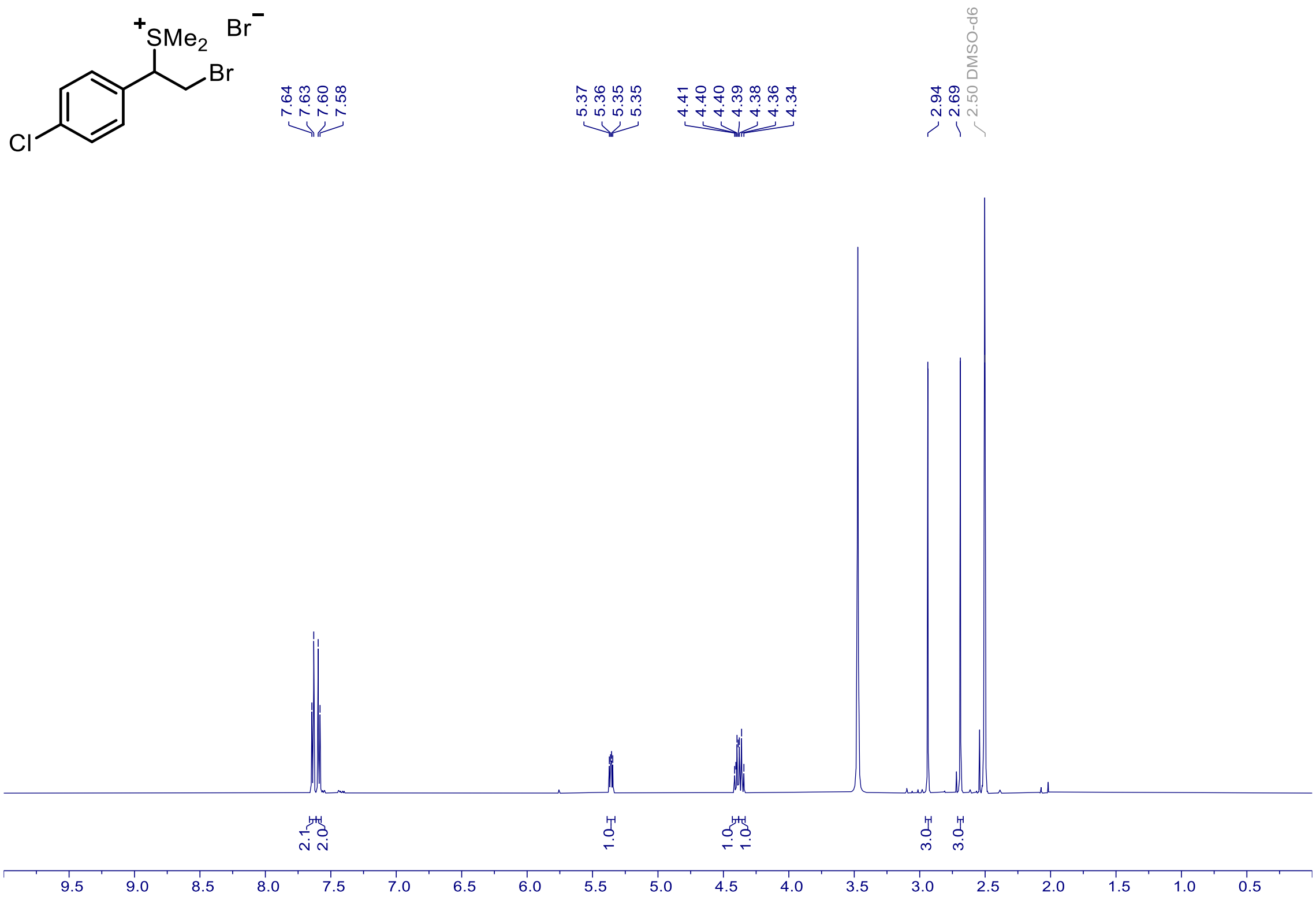

S5a – ^{13}C NMR (151 MHz, DMSO-d_6)

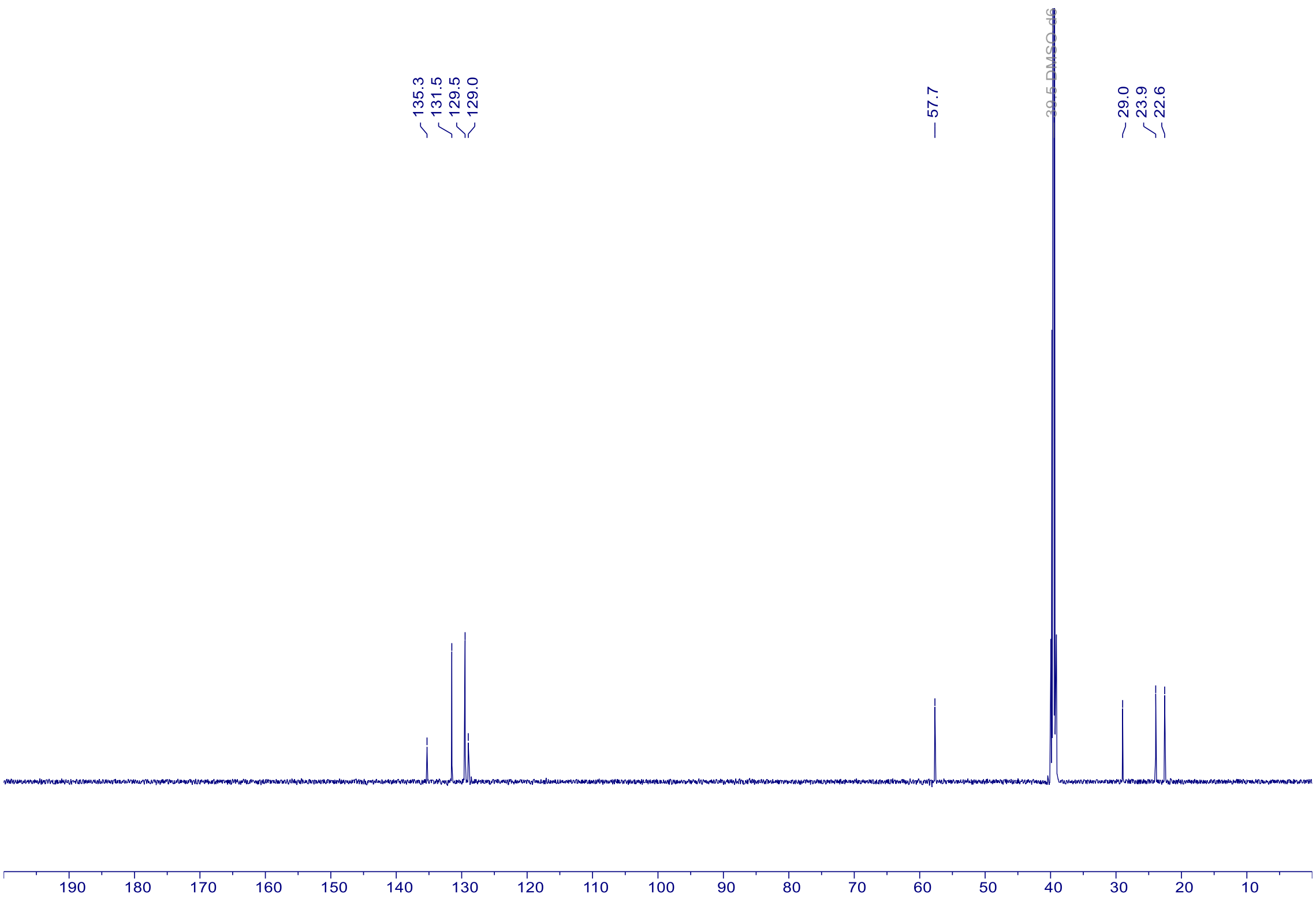

S7a – ^{1}H NMR (600 MHz, DMSO-d_6)

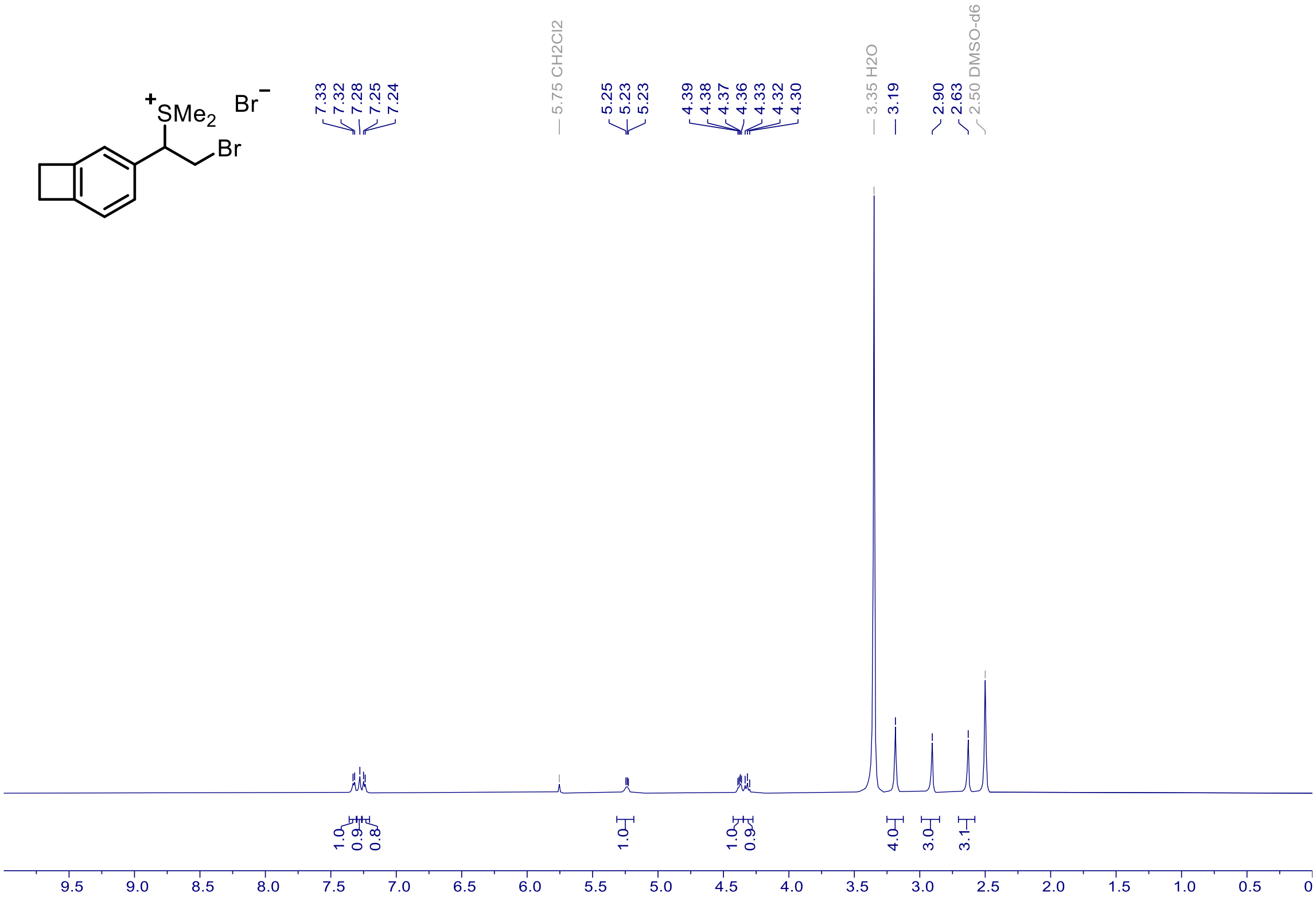

S7a – ^{13}C NMR (151 MHz, DMSO-d_6)

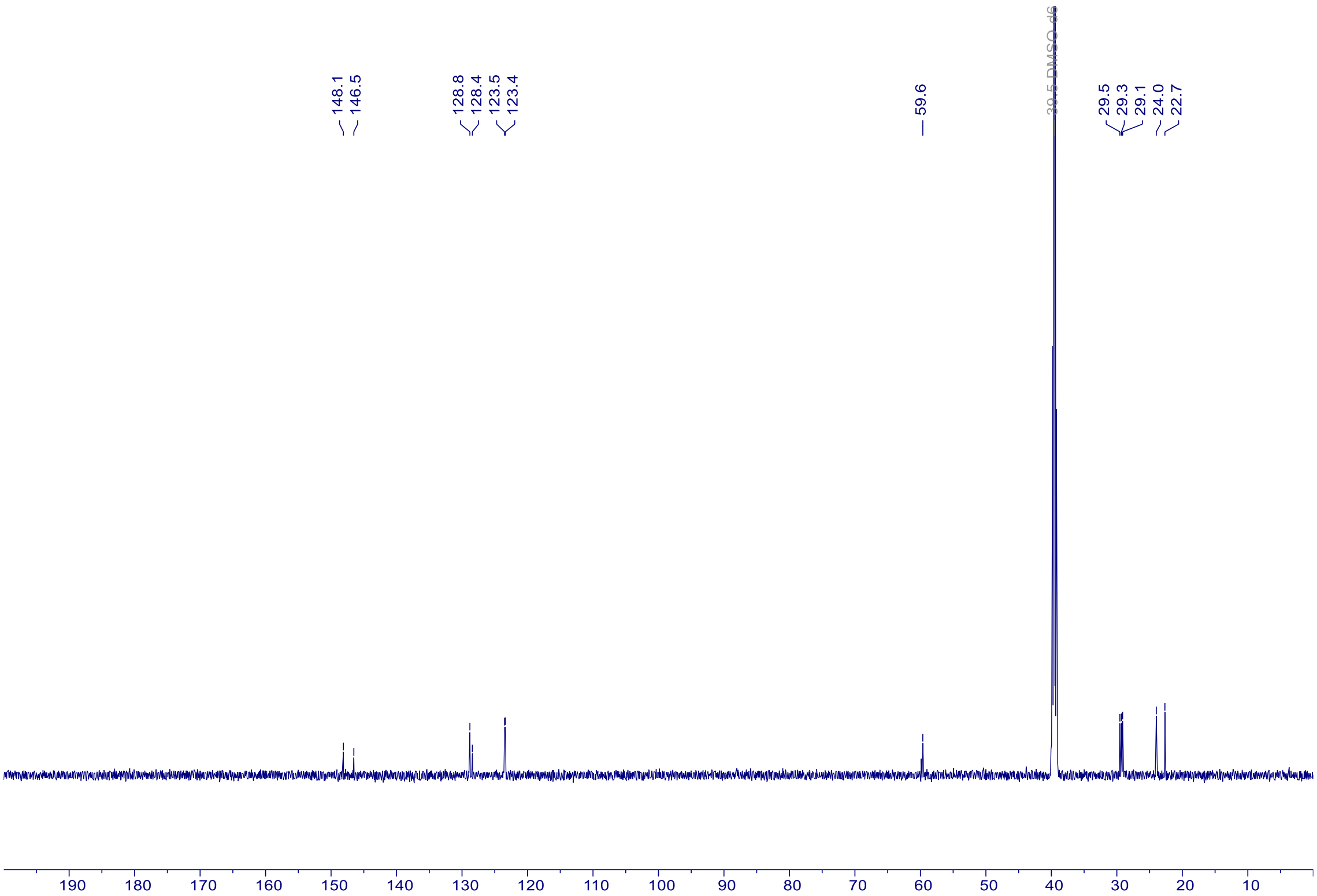

S8a – ^{1}H NMR (600 MHz, DMSO-d_6)

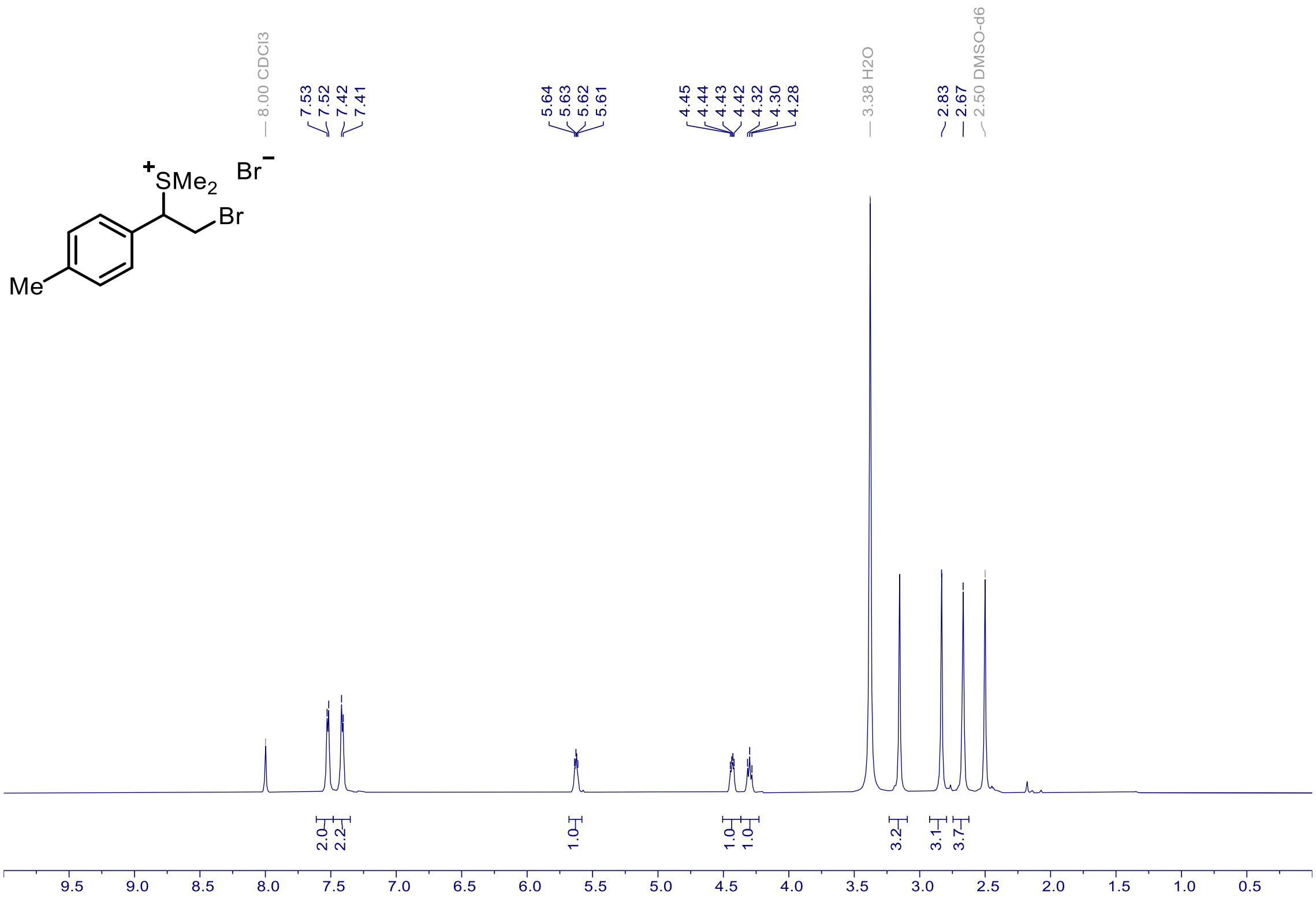

S8a – ^{13}C NMR (151 MHz, DMSO-d_6)

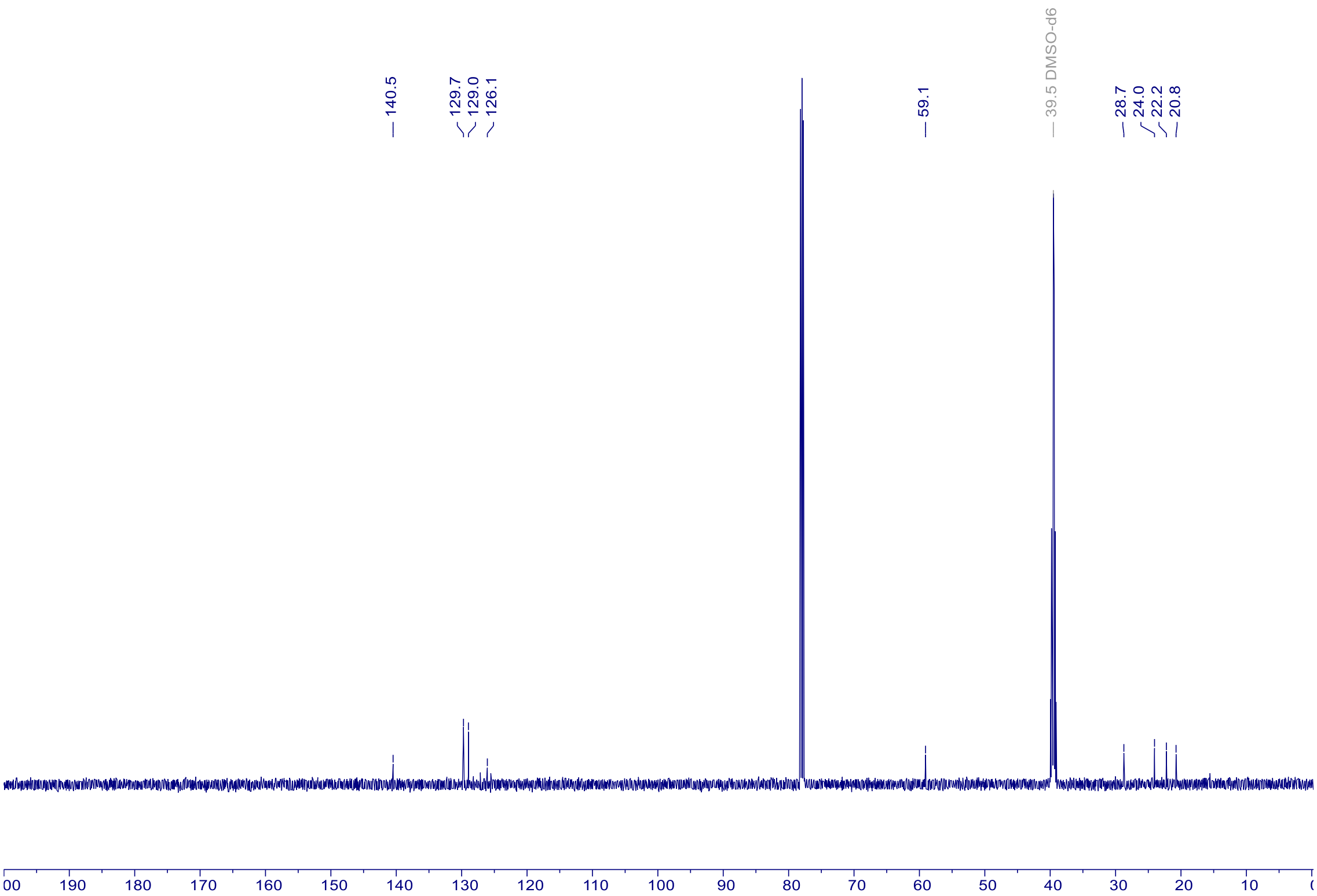

S9a – ^{1}H NMR (400 MHz, $CDCl_3$)

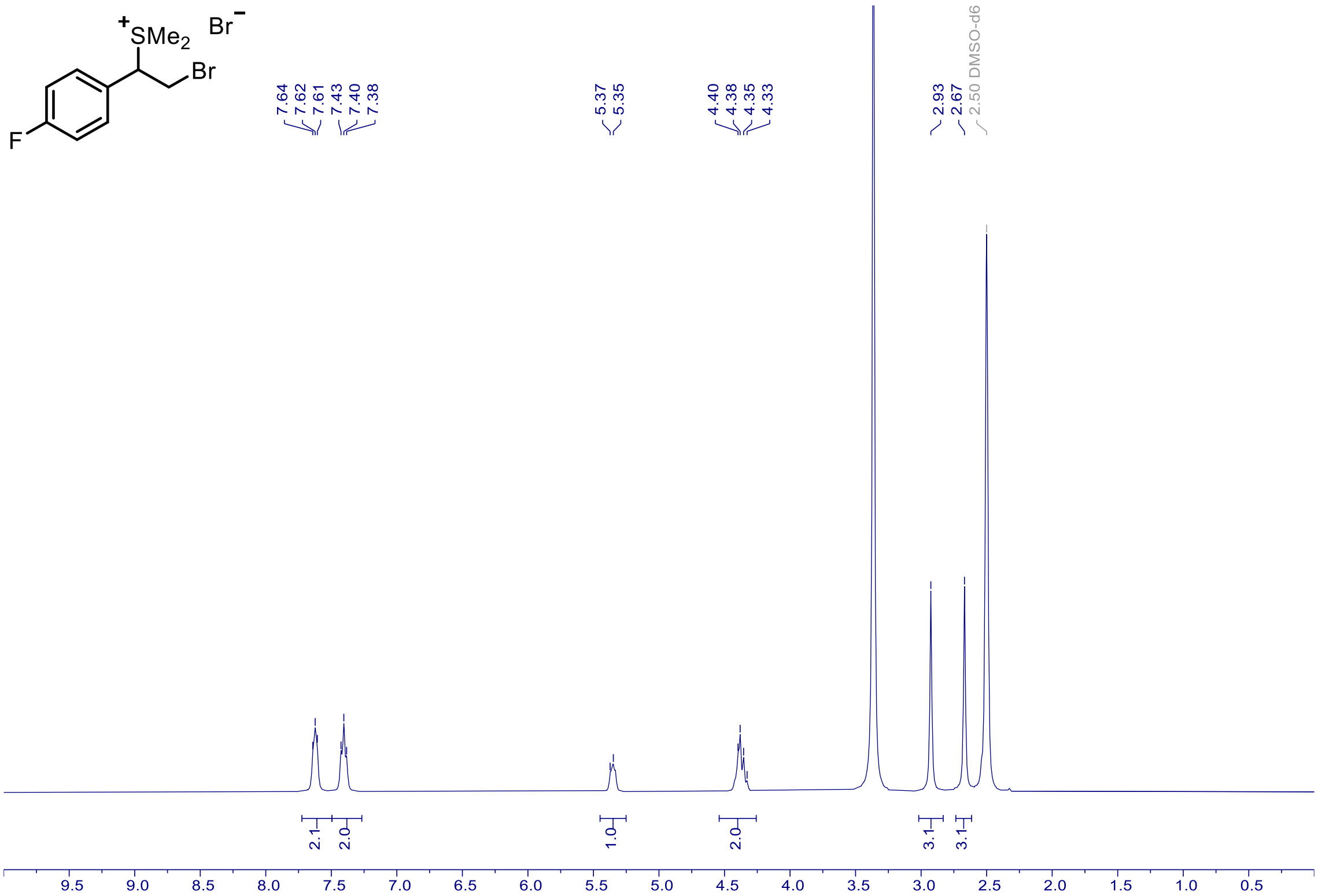

S9a – ^{13}C NMR (101 MHz, $CDCl_3$)

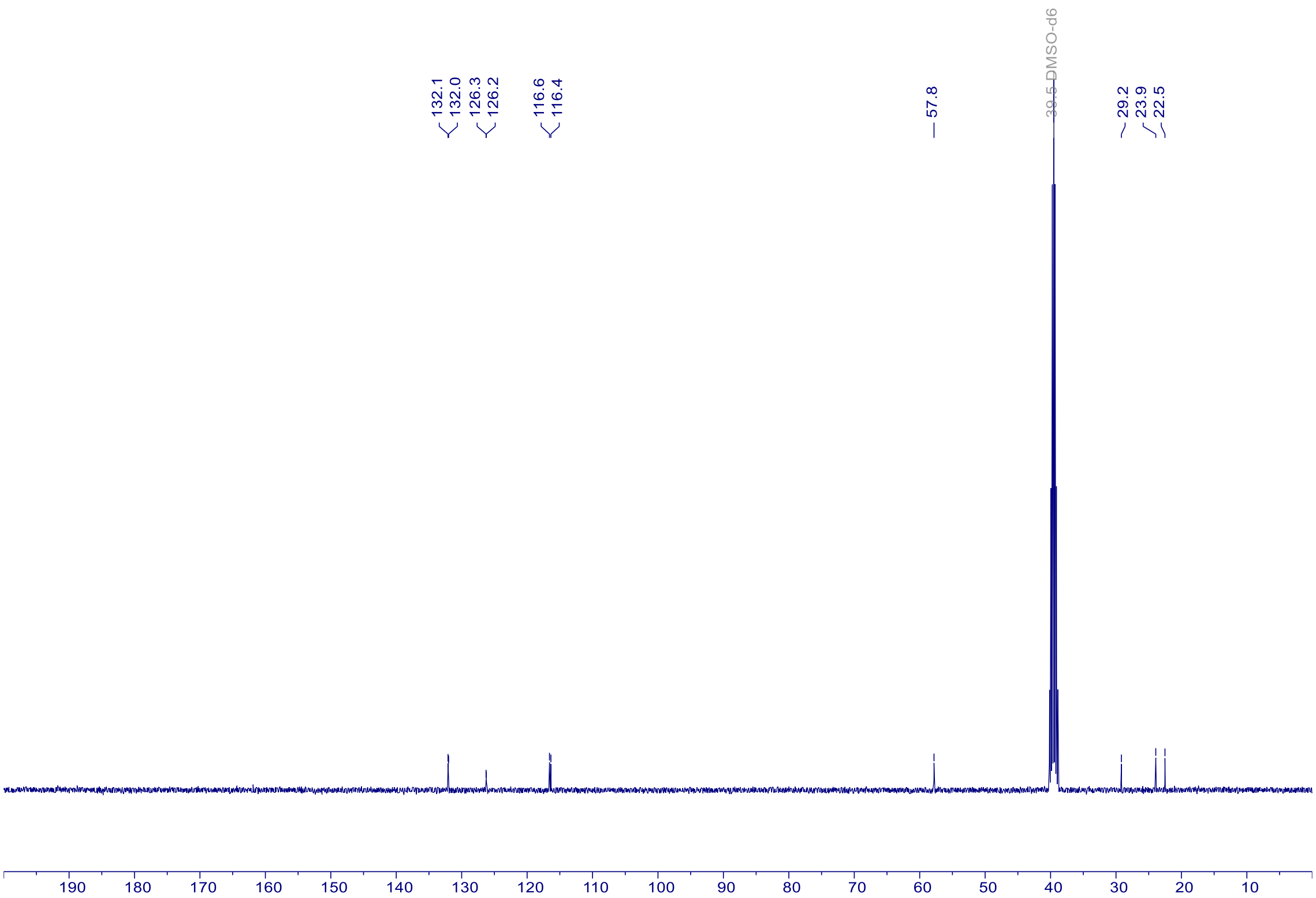

S9a – ^{19}F NMR (565 MHz, $CDCl_3$)

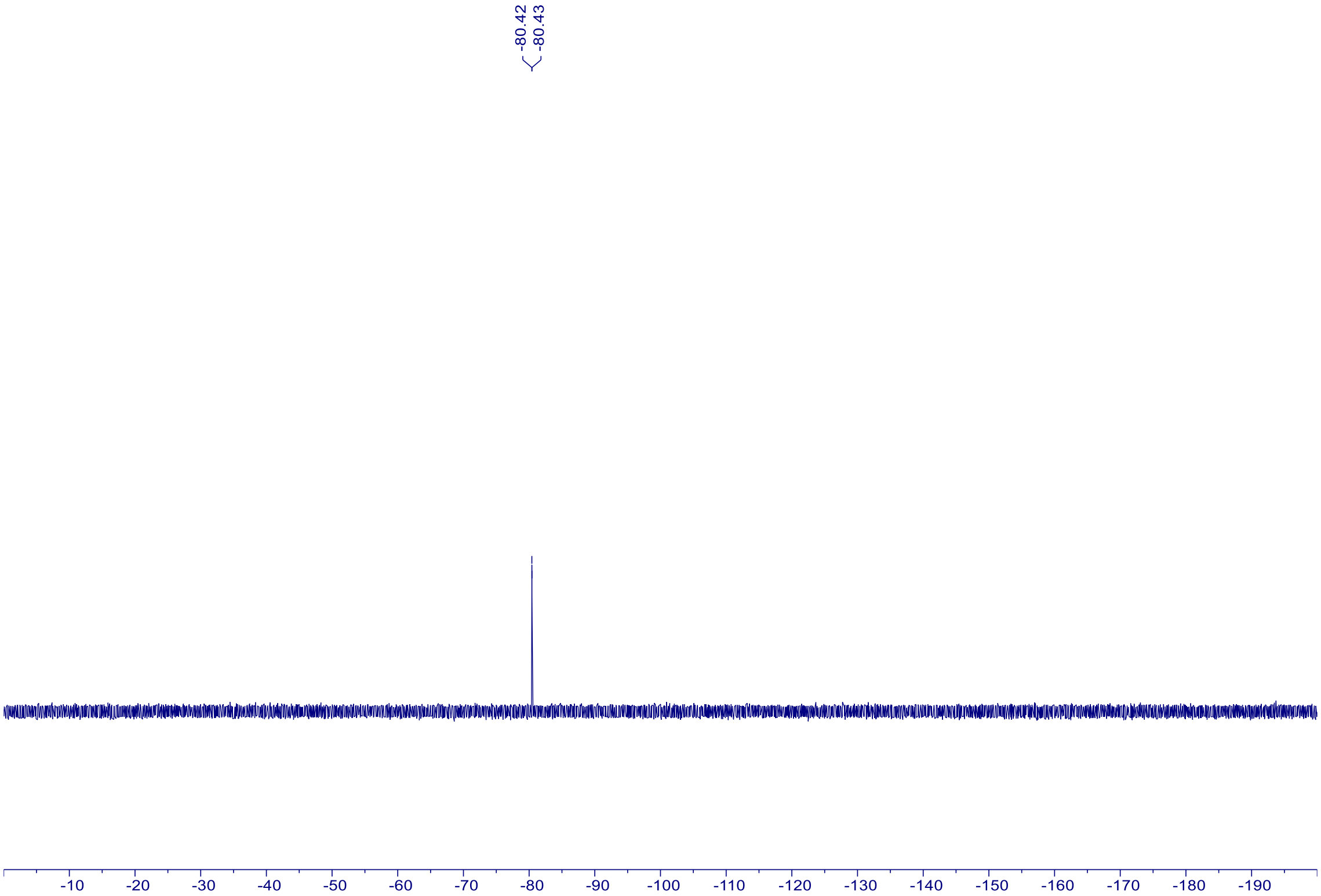

S11a – ^{1}H NMR (600 MHz, $CDCl_3$)

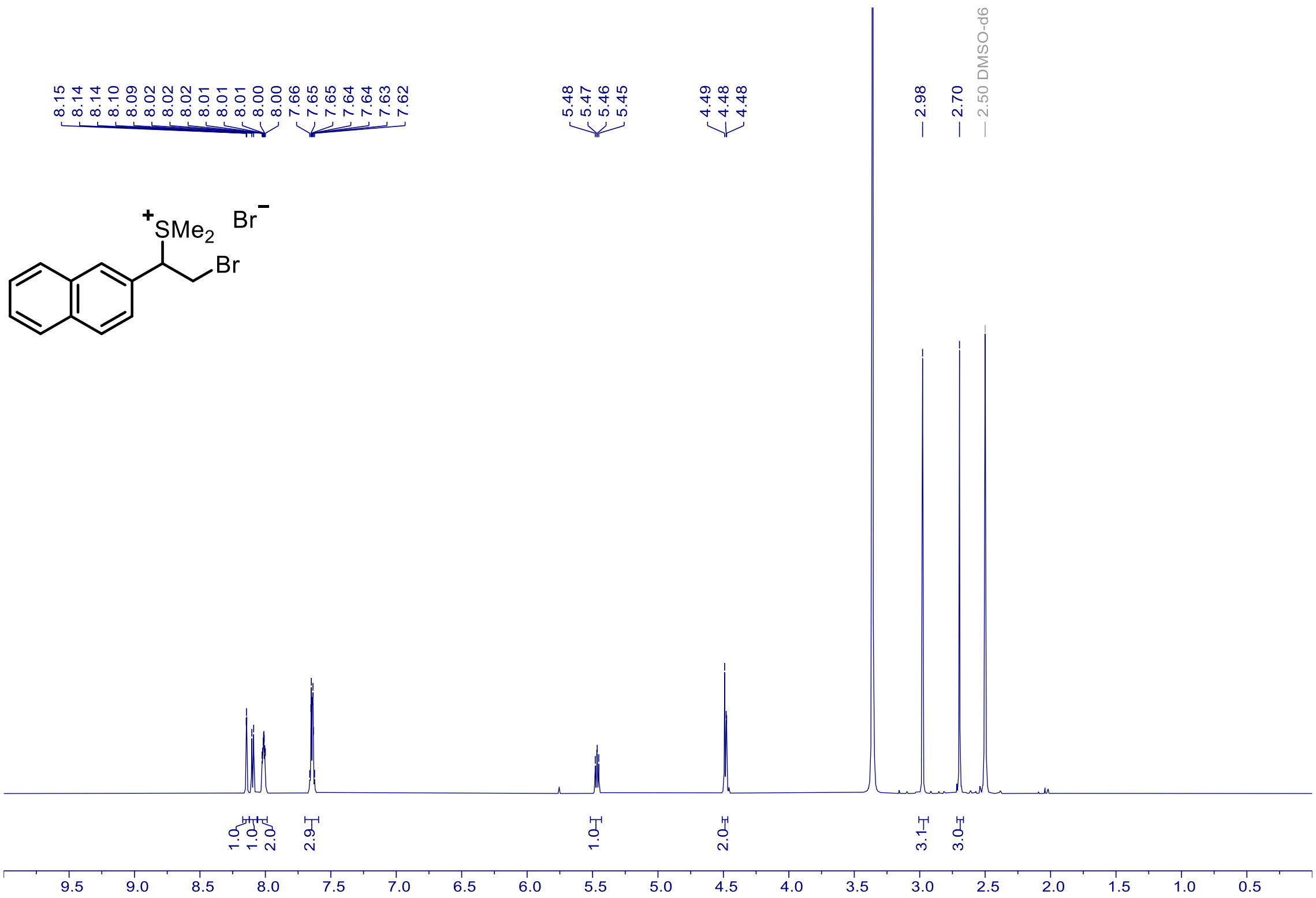

S11a – ^{13}C NMR (151 MHz, $CDCl_3$)

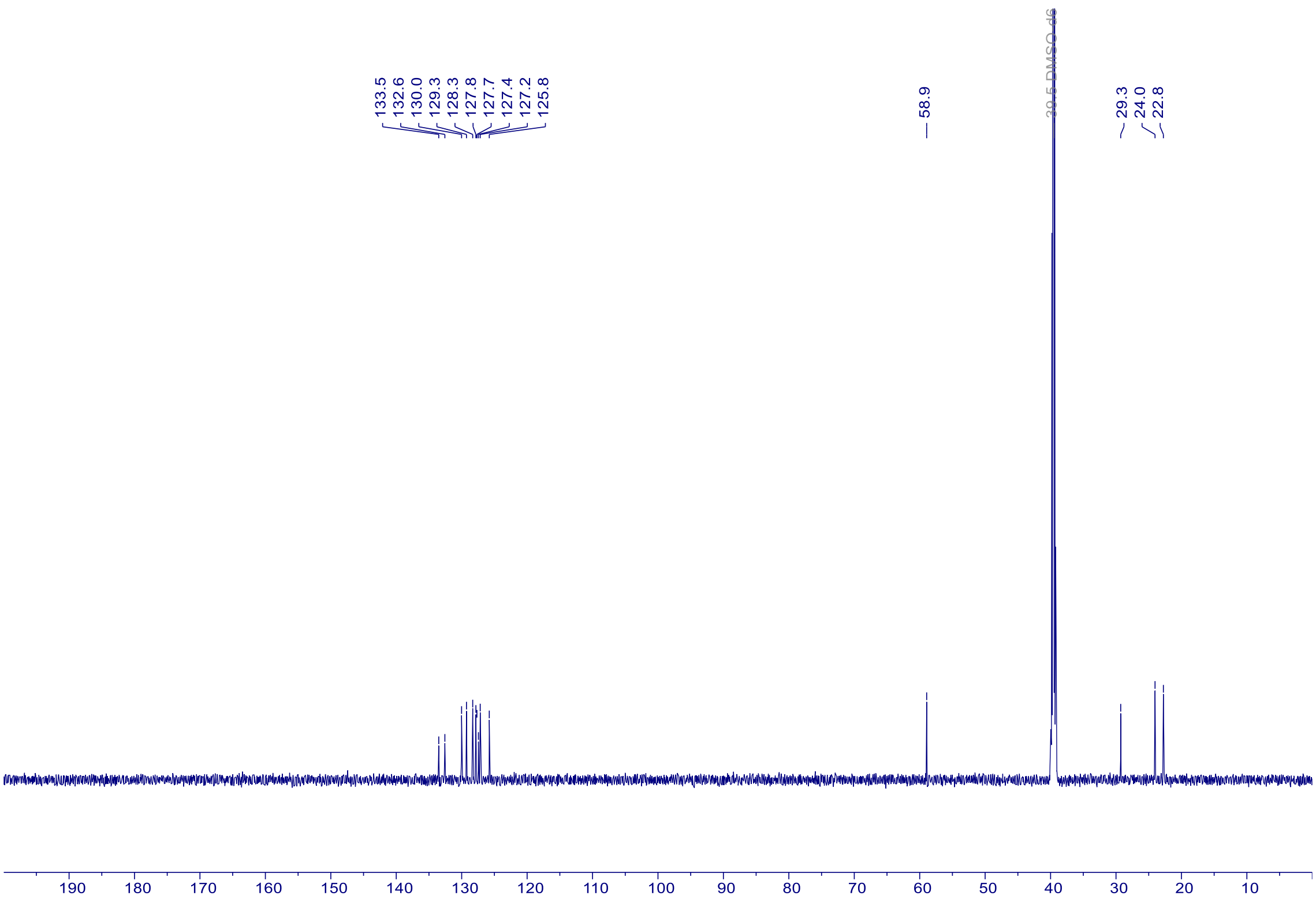

S12a – ^{1}H NMR (400 MHz, DMSO-d_6)

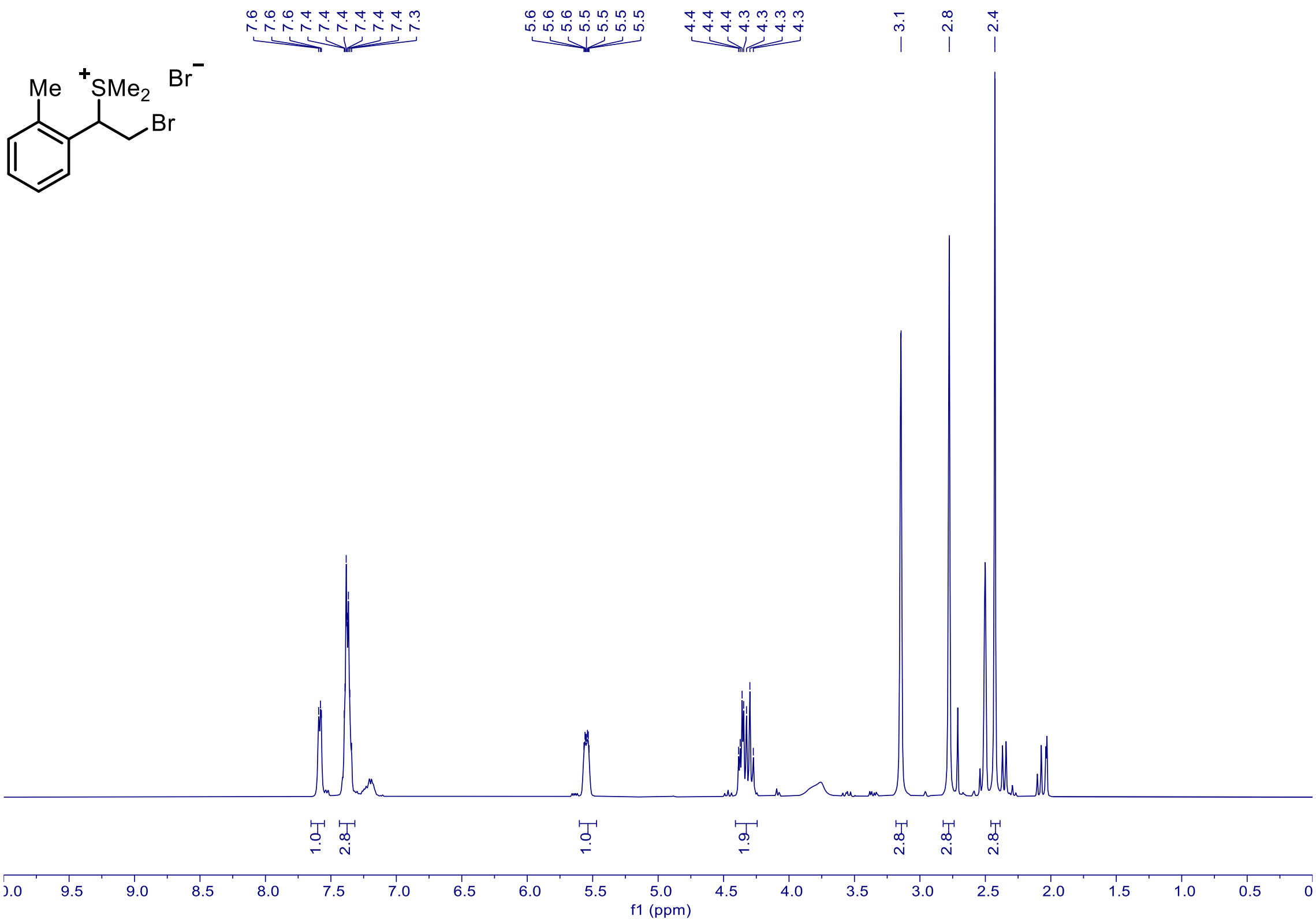

S12a – ^{13}C NMR (101 MHz, CDCl$_3$)

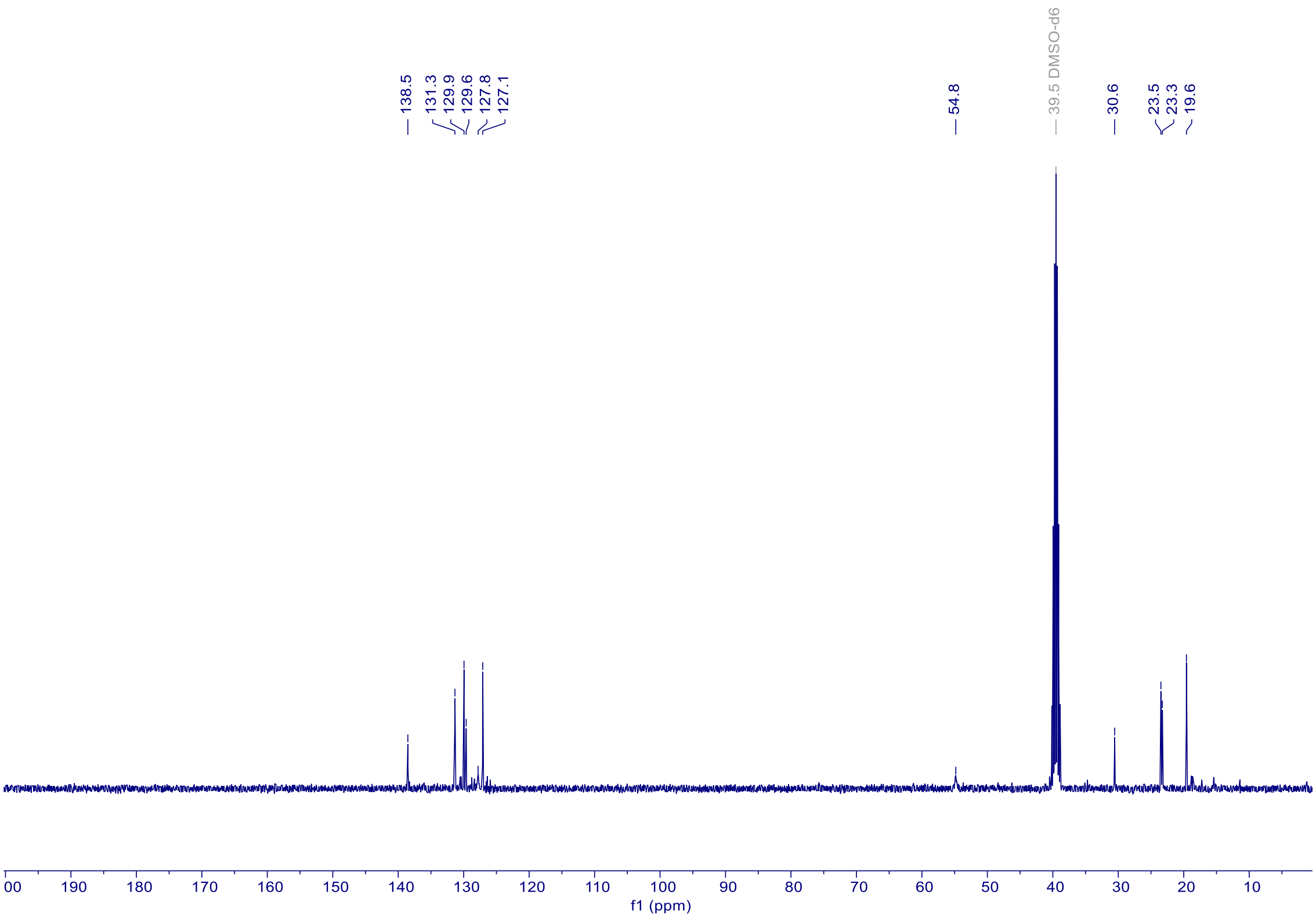

2 – ^{1}H NMR (400 MHz, $CDCl_3$)

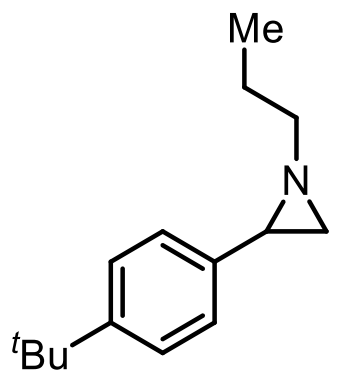

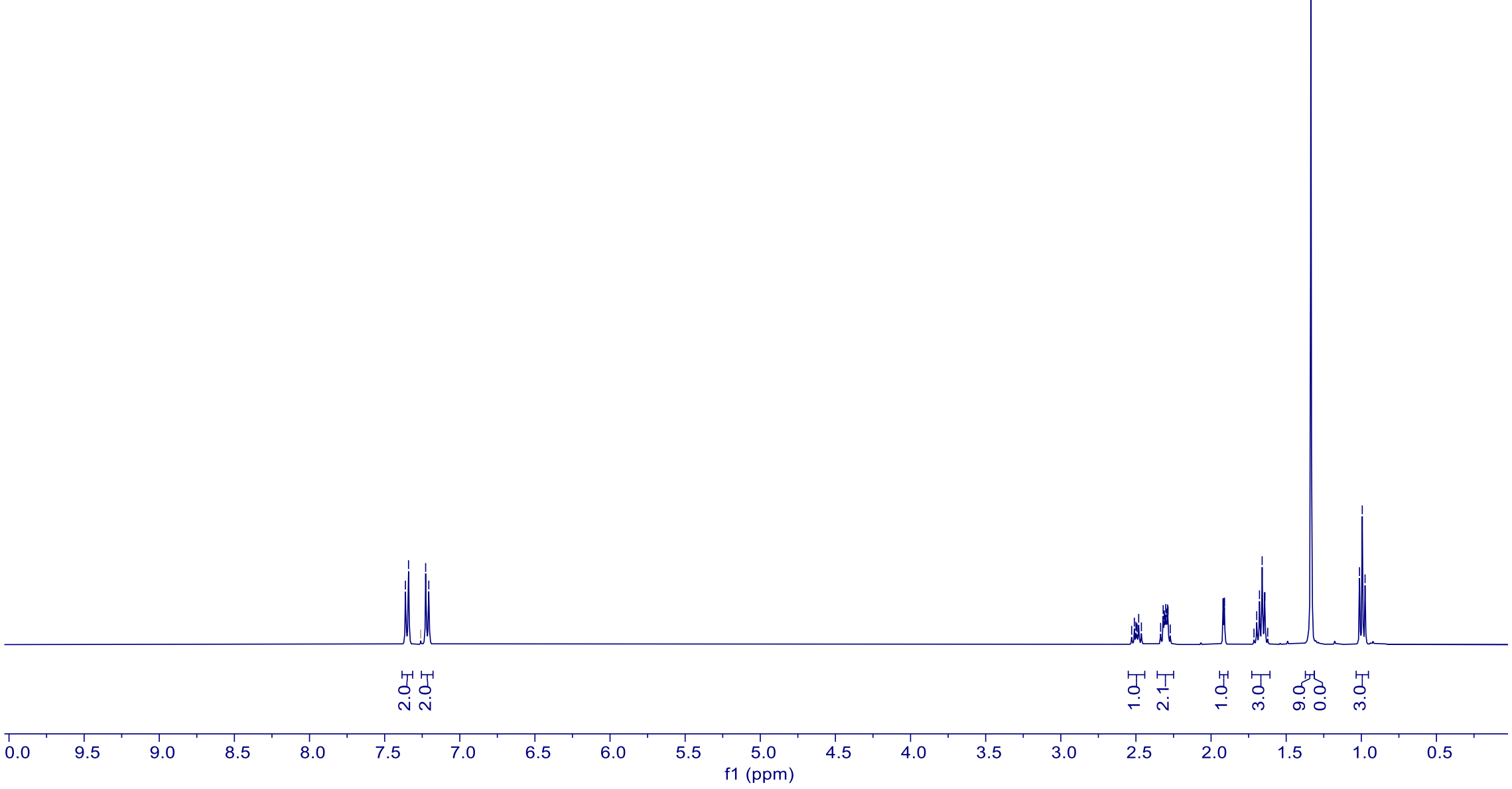

2 – ^{13}C NMR (151 MHz, $CDCl_3$)

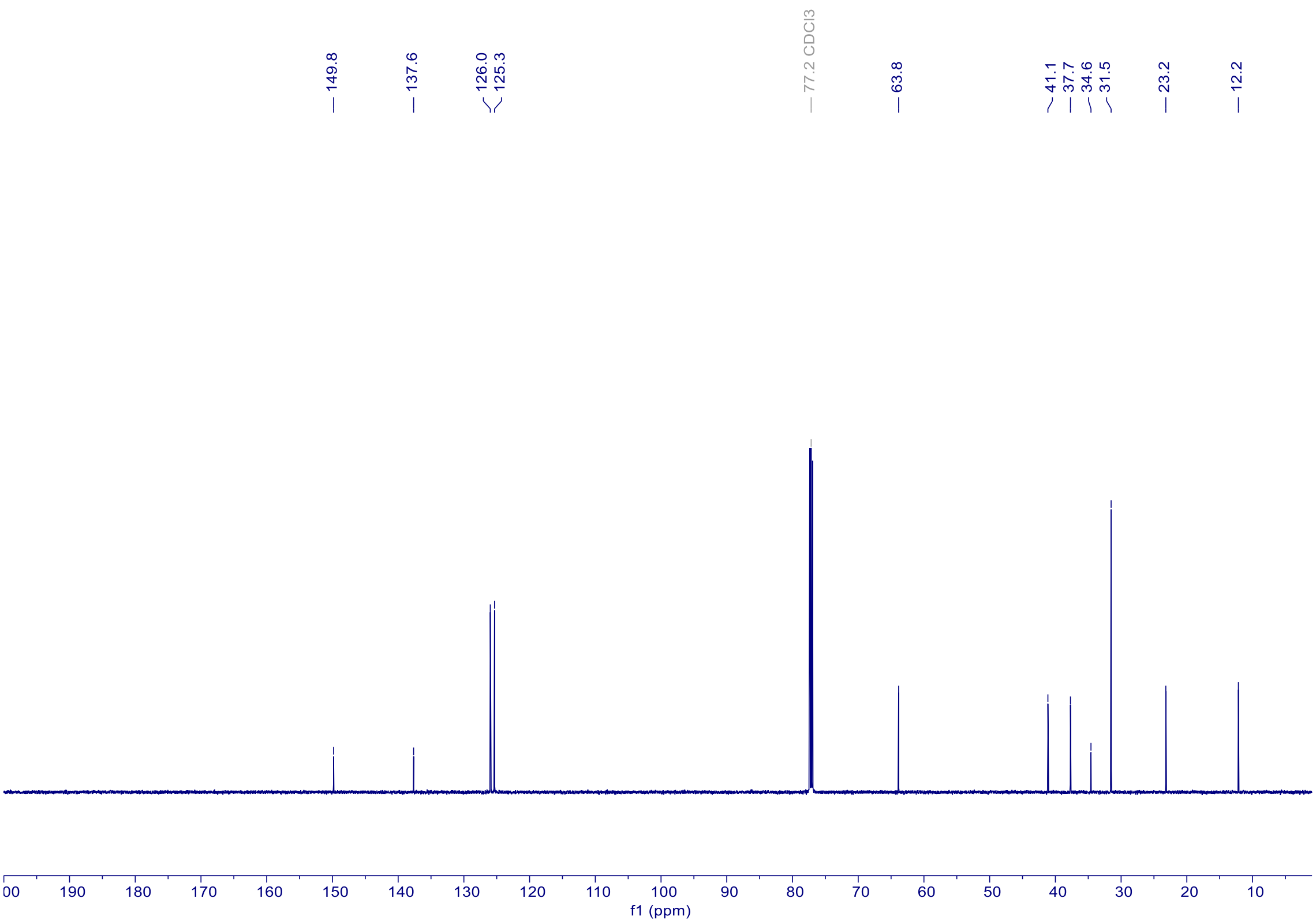

3 – ^{1}H NMR (600 MHz, $CDCl_3$)

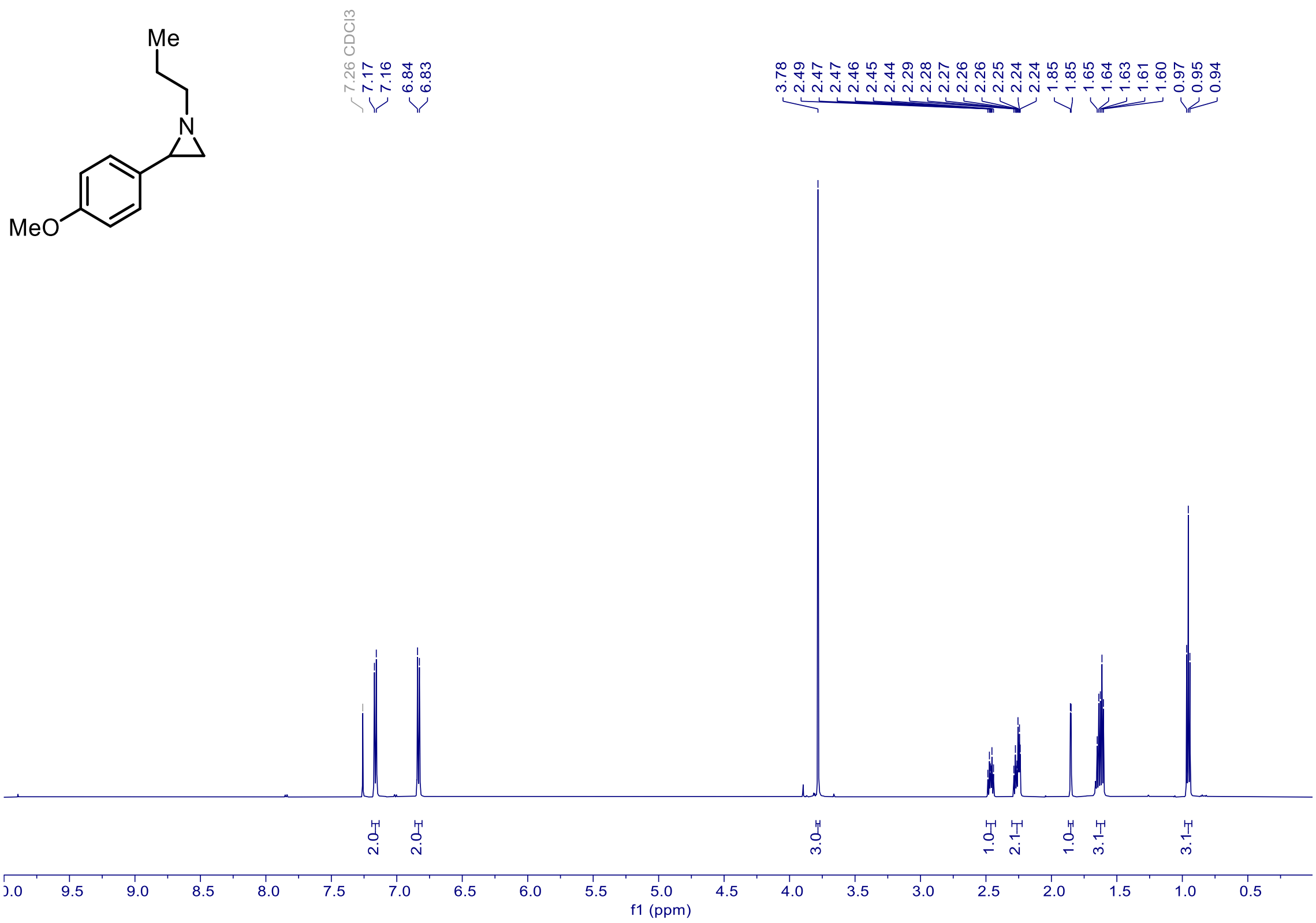

3 – ^{13}C NMR (151 MHz, $CDCl_3$)

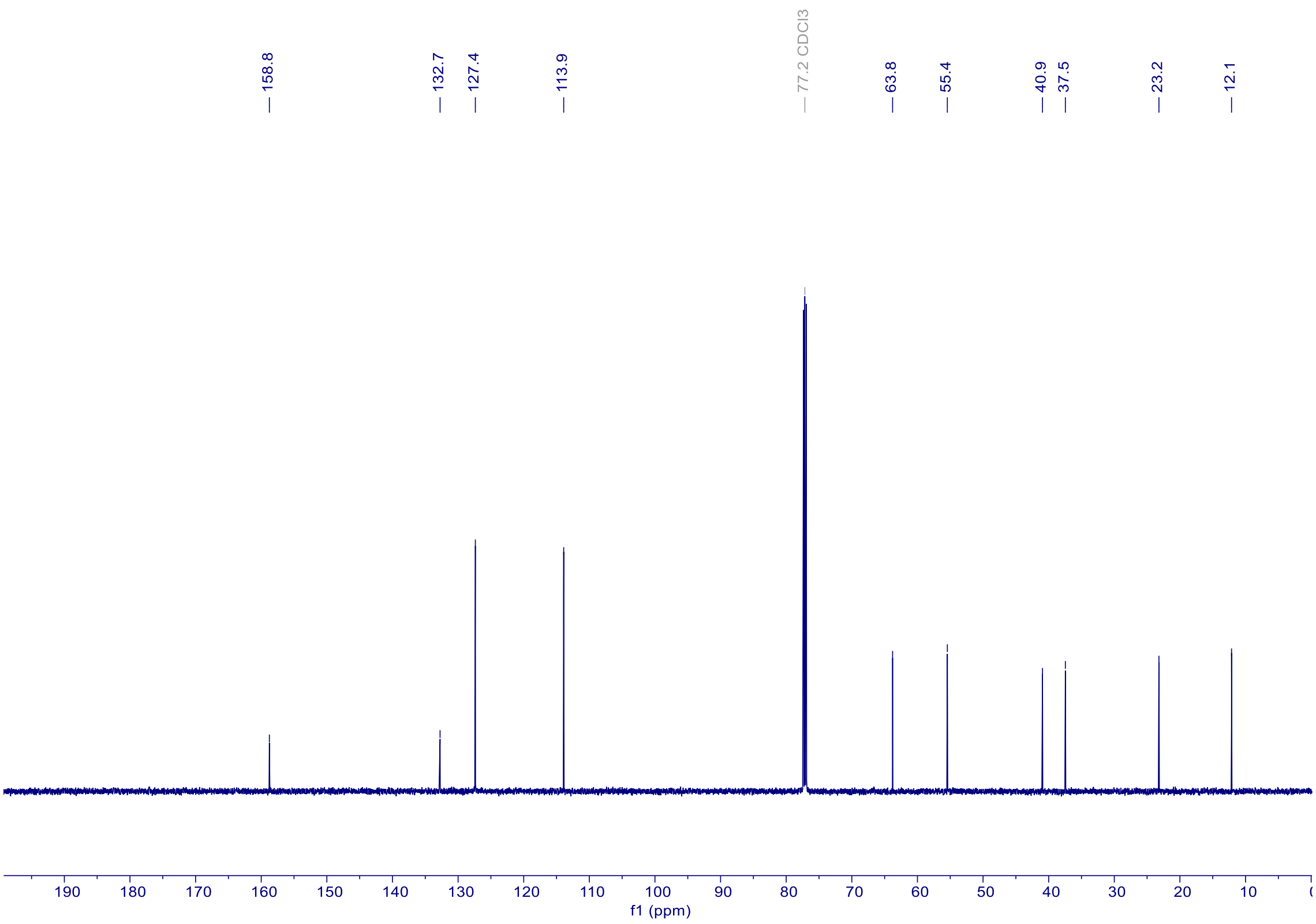

4 – ^{1}H NMR (400 MHz, $CDCl_3$)

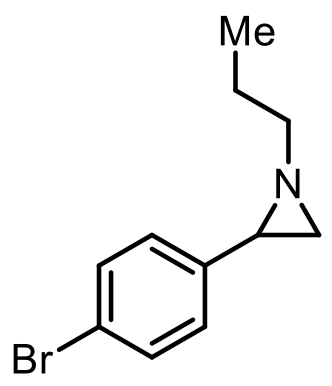

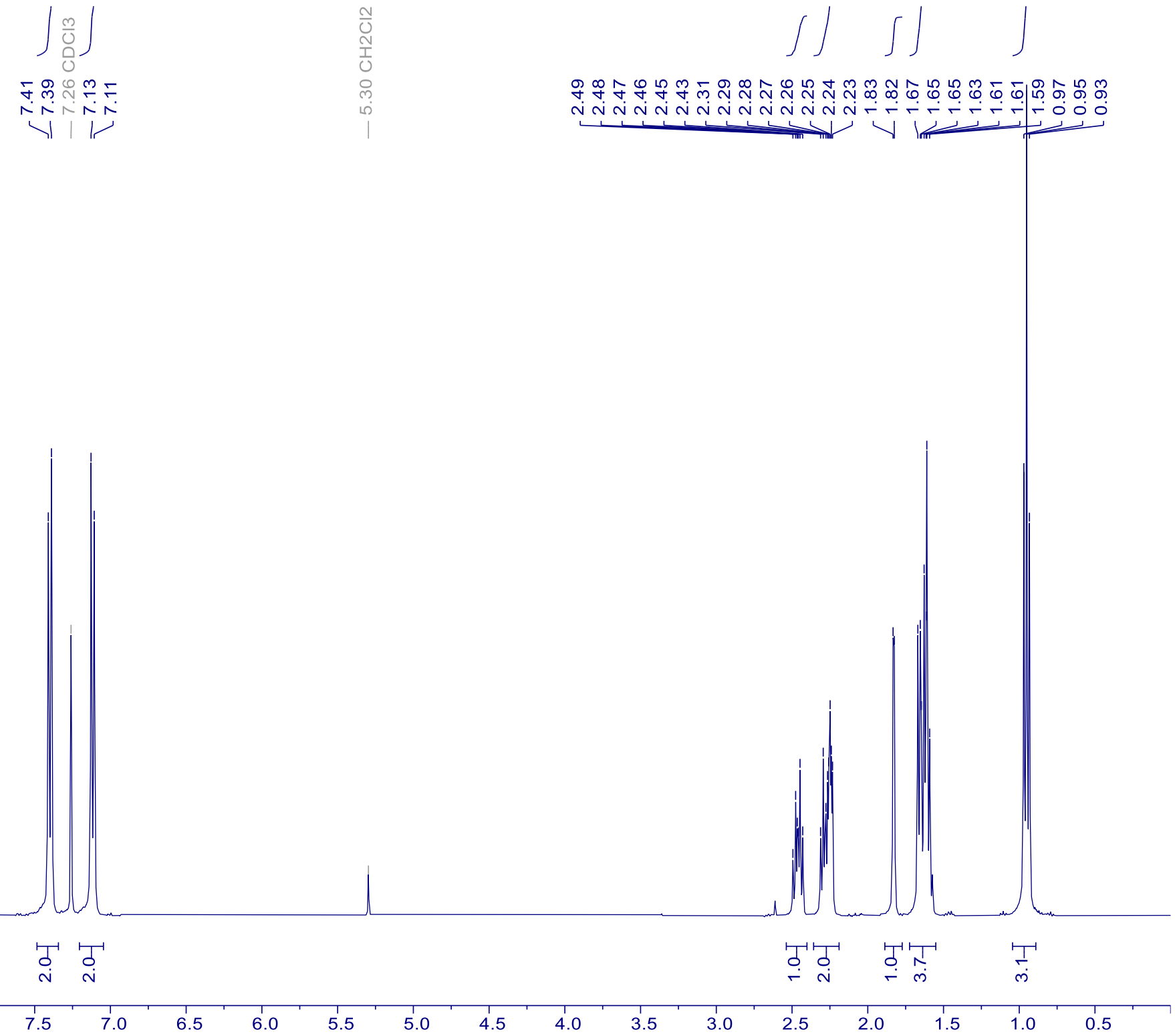

4 – ^{13}C NMR (151 MHz, $CDCl_3$)

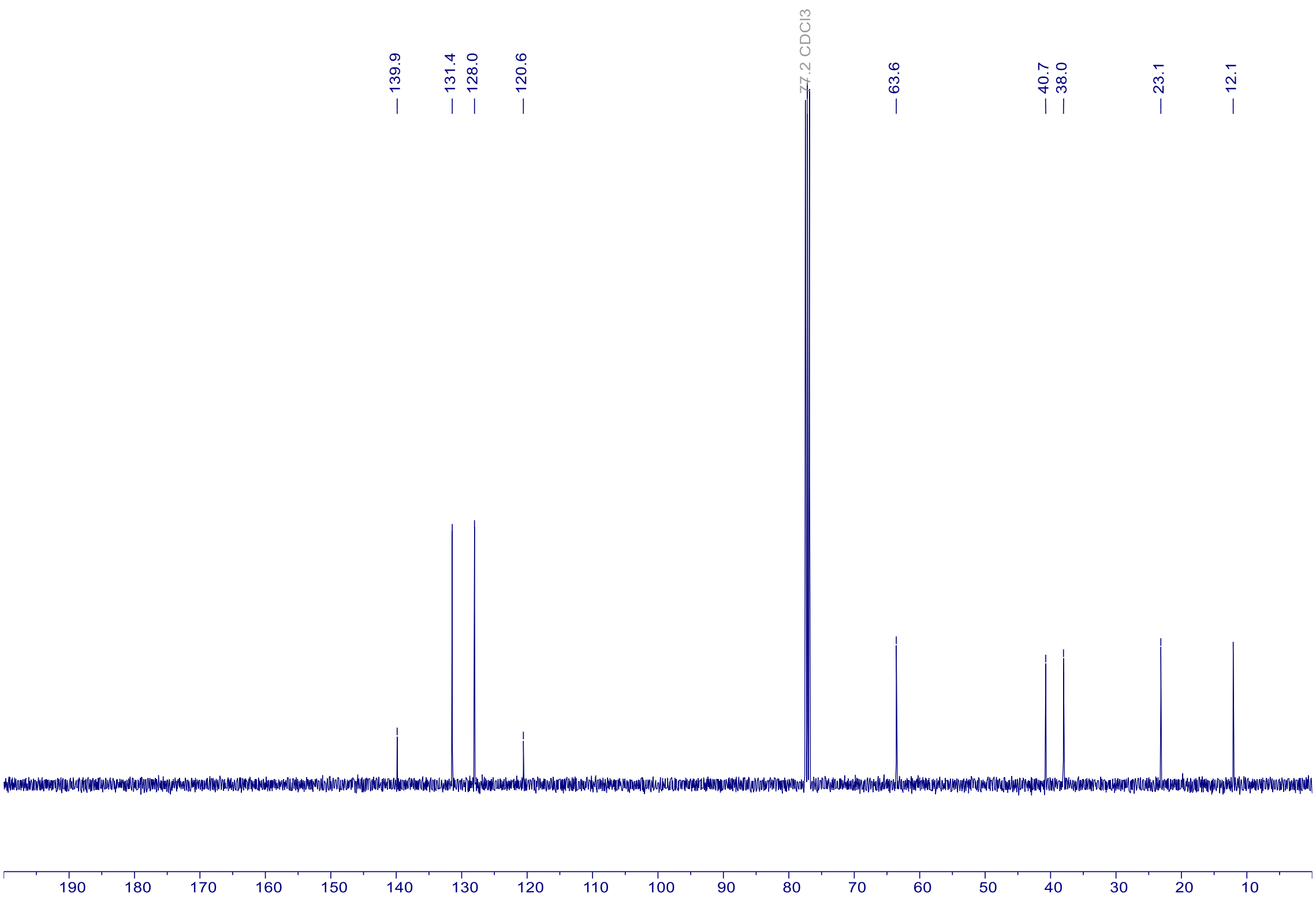

5 – ^{1}H NMR (600 MHz, $CDCl_3$)

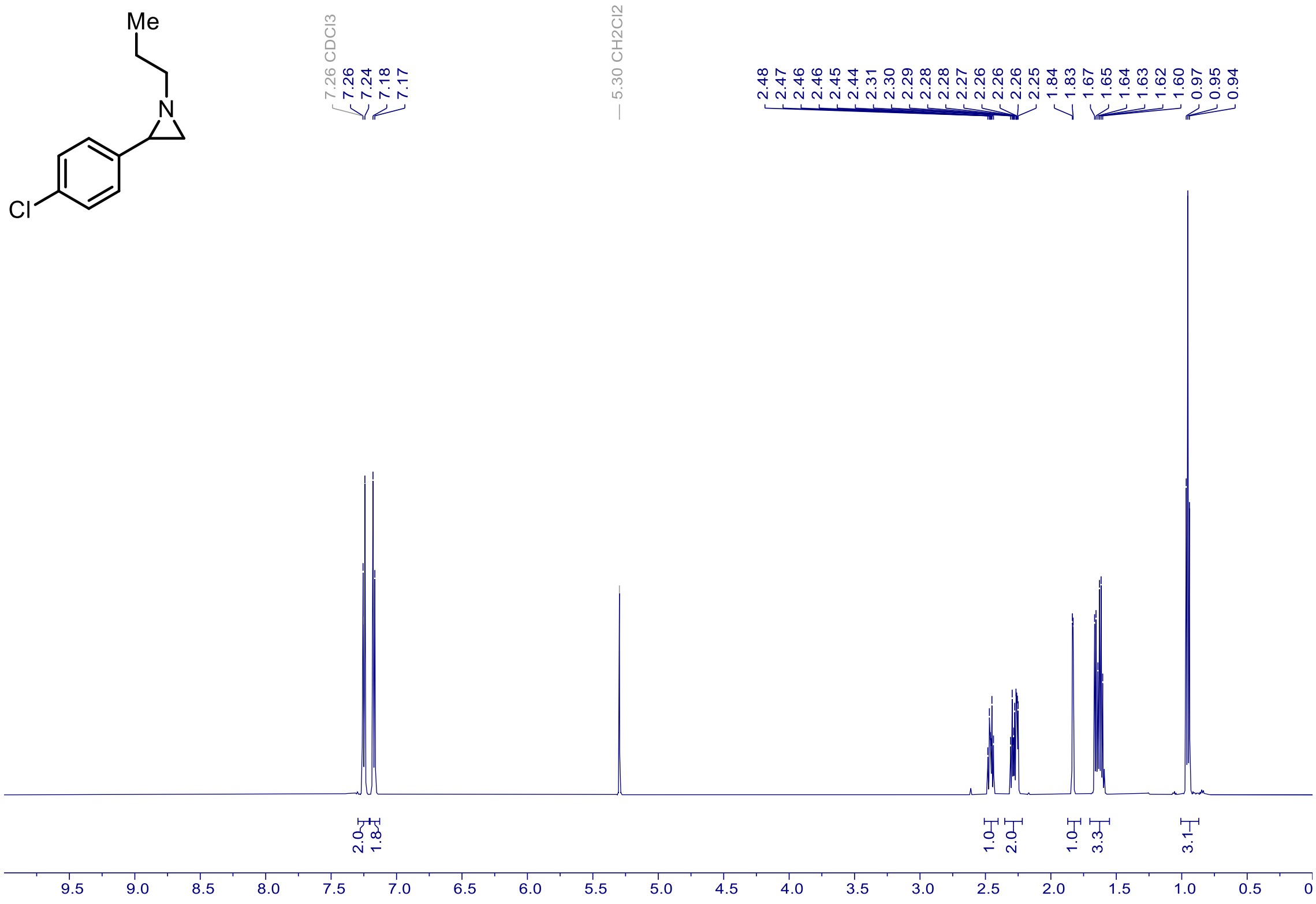

5 – ^{13}C NMR (151 MHz, $CDCl_3$)

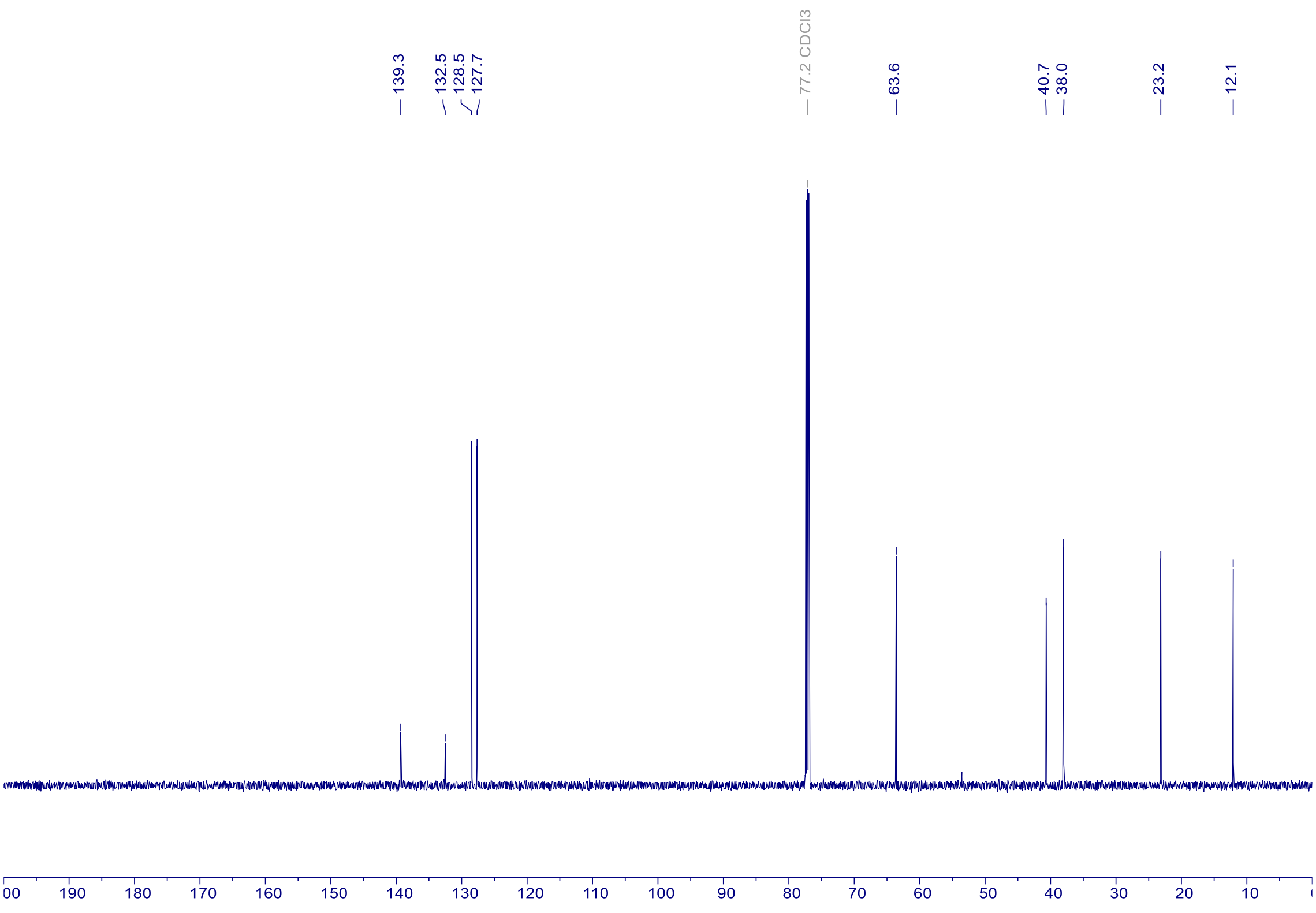

6 – ^{1}H NMR (600 MHz, $CDCl_3$)

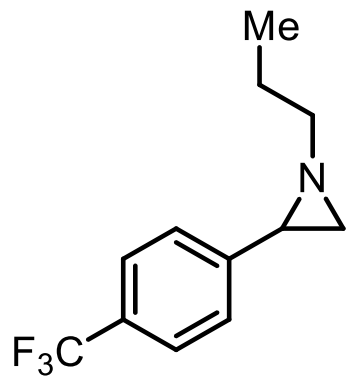

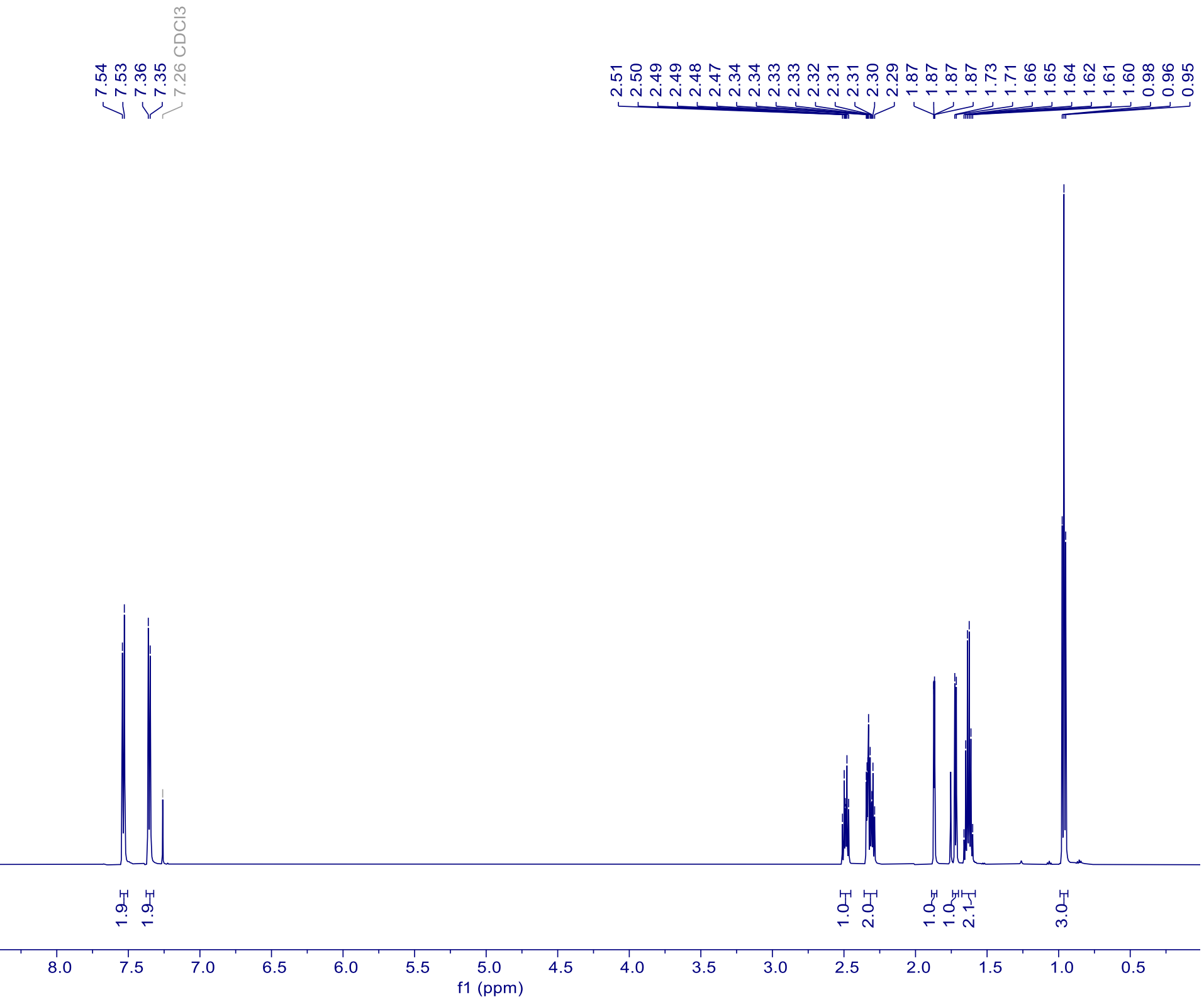

6 – ^{13}C NMR (151 MHz, $CDCl_3$)

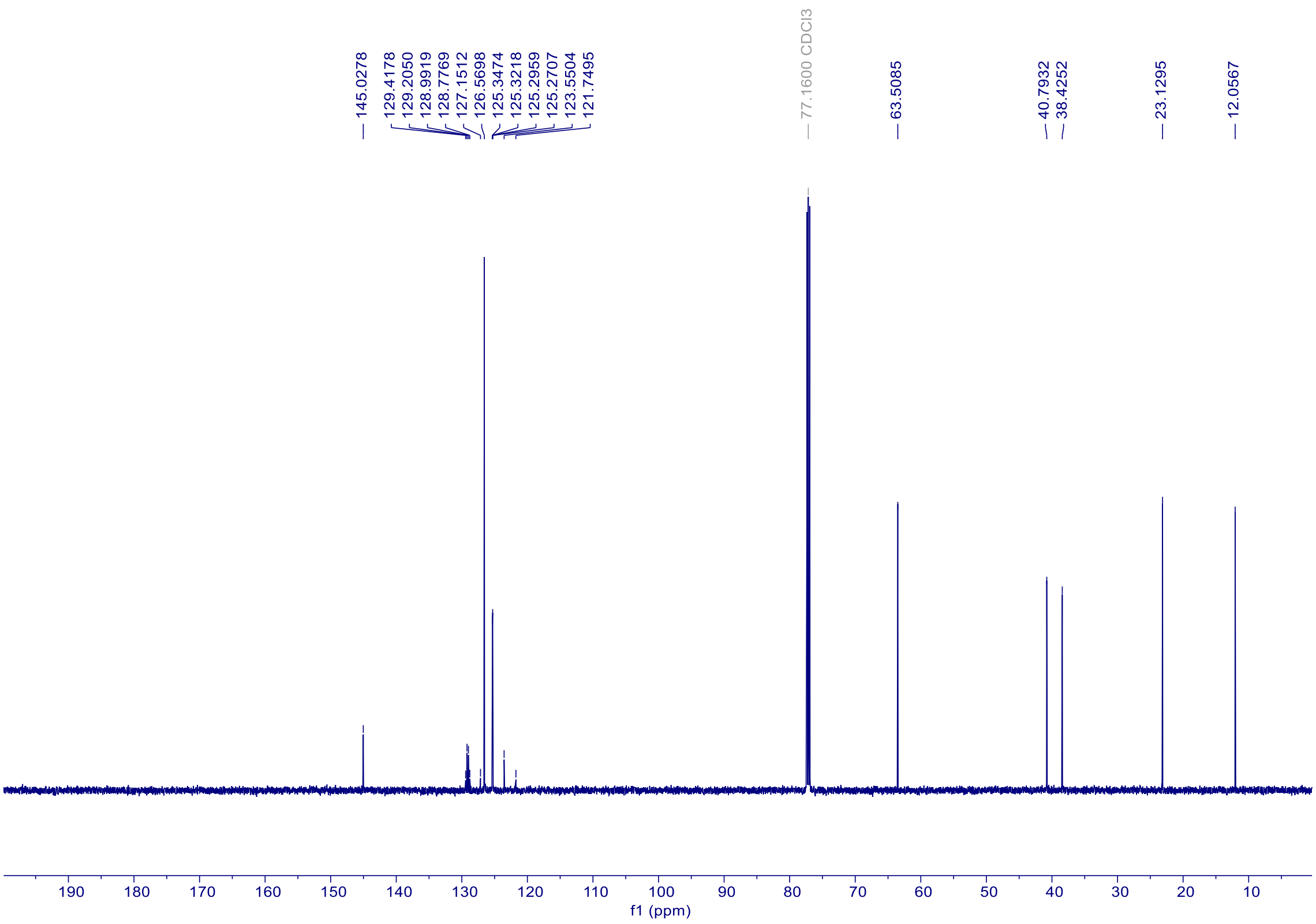

6 – ^{19}F NMR (565 MHz, $CDCl_3$)

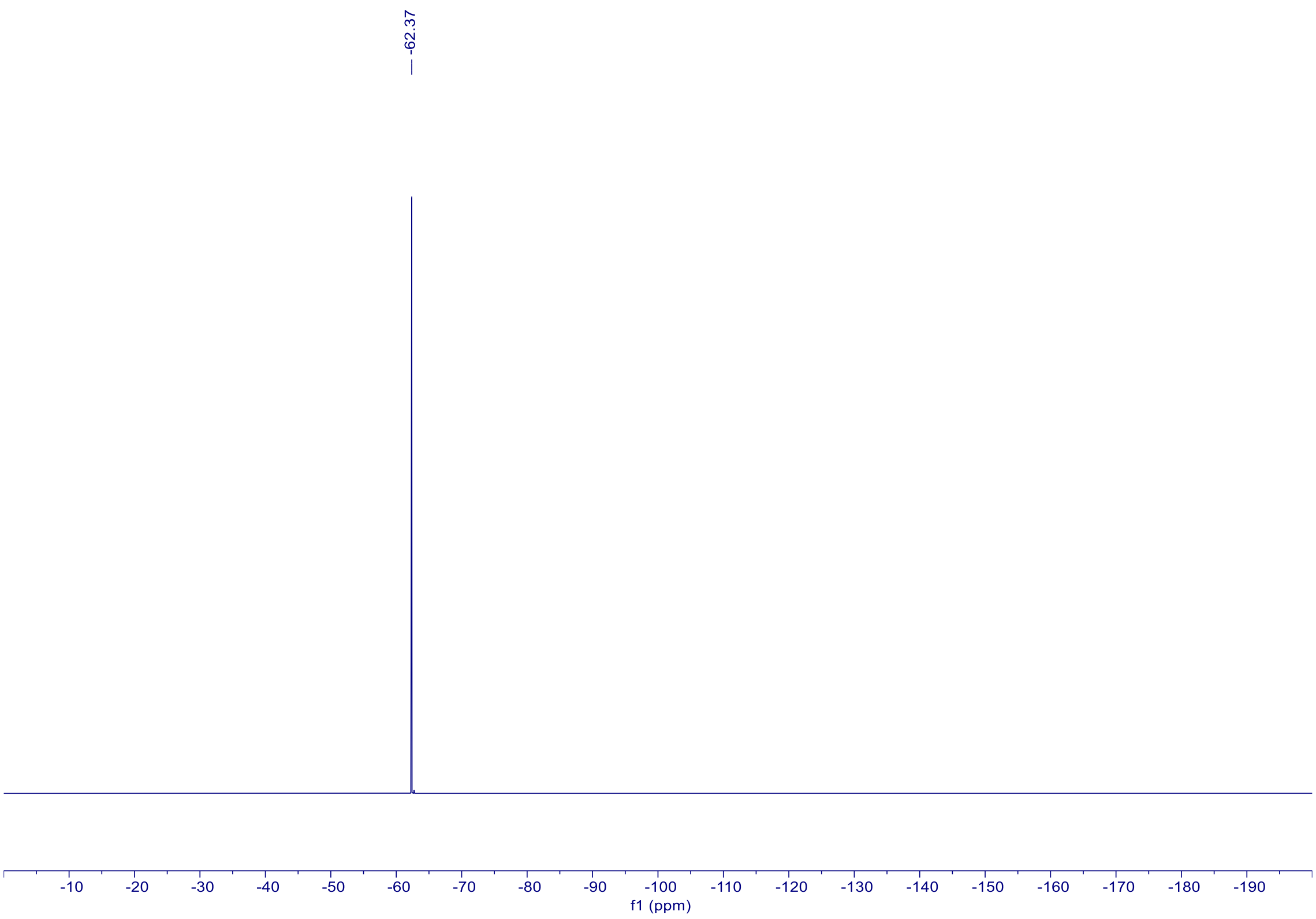

7 – ¹H NMR (400 MHz, $CDCl_3$)

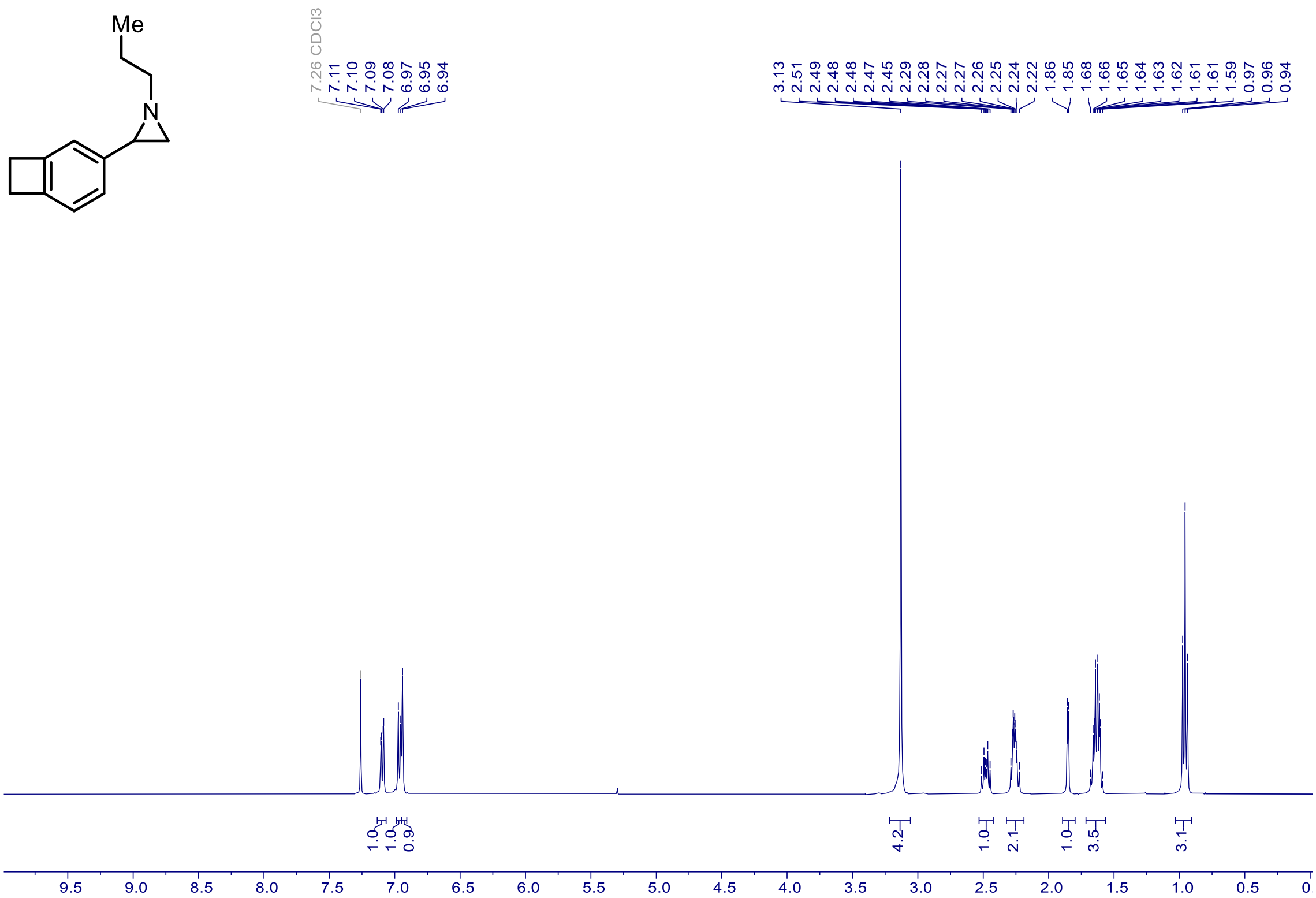

7 – ¹³C NMR (101 MHz, $CDCl_3$)

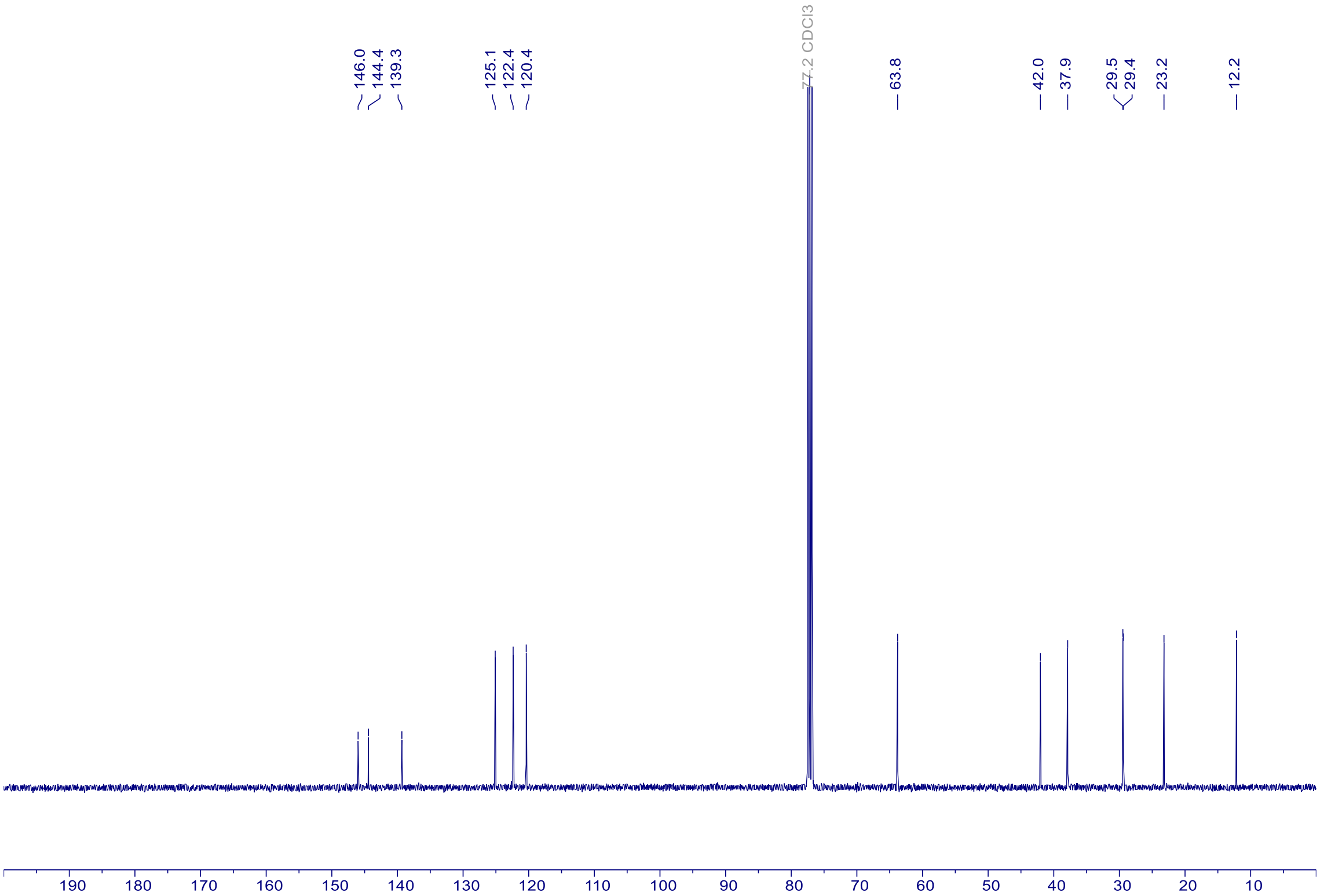

14 – ^{1}H NMR (600 MHz, $CDCl_3$)

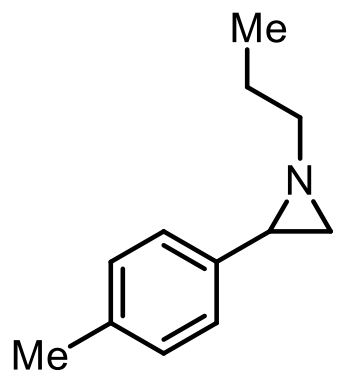

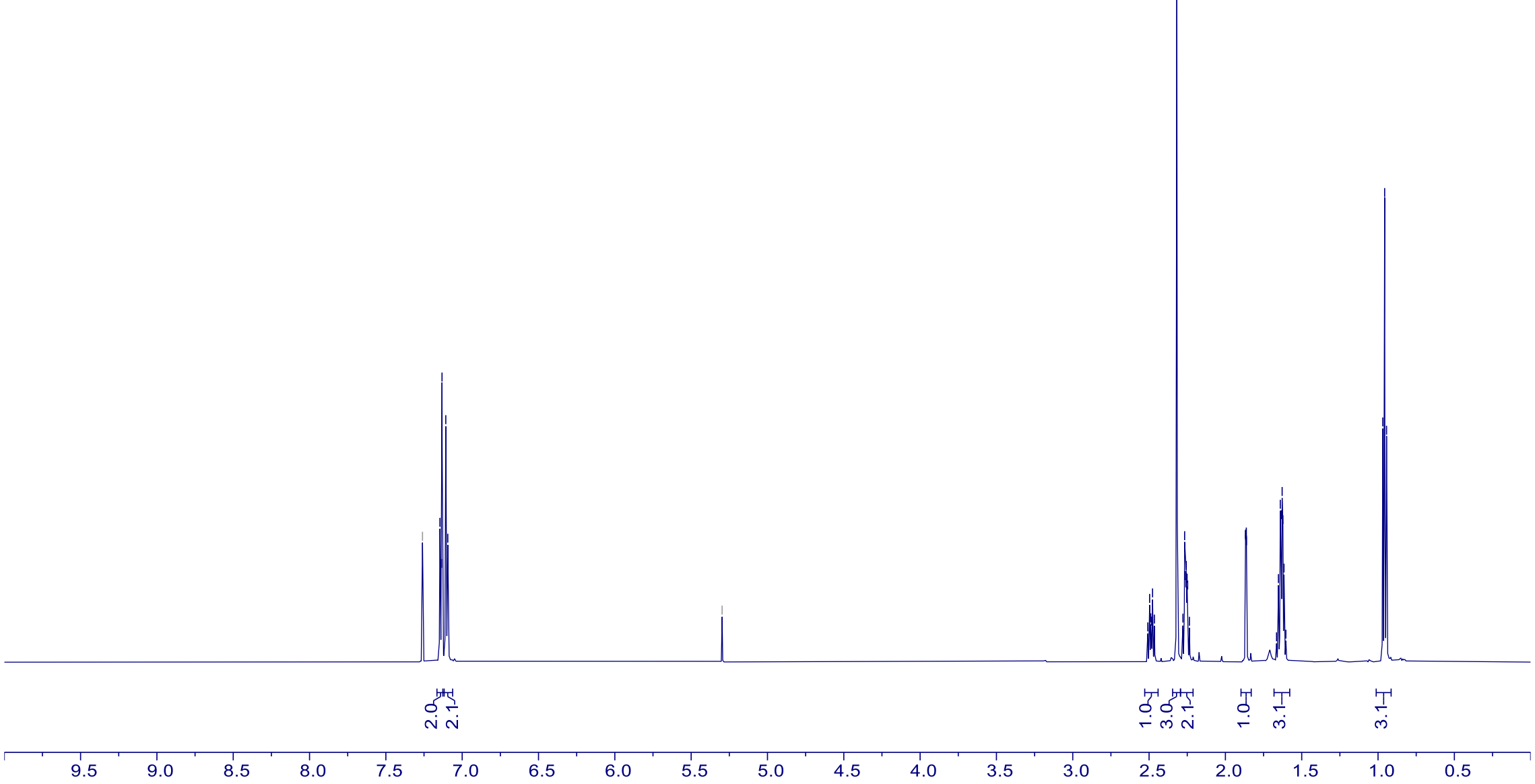

14 – ^{13}C NMR (151 MHz, $CDCl_3$)

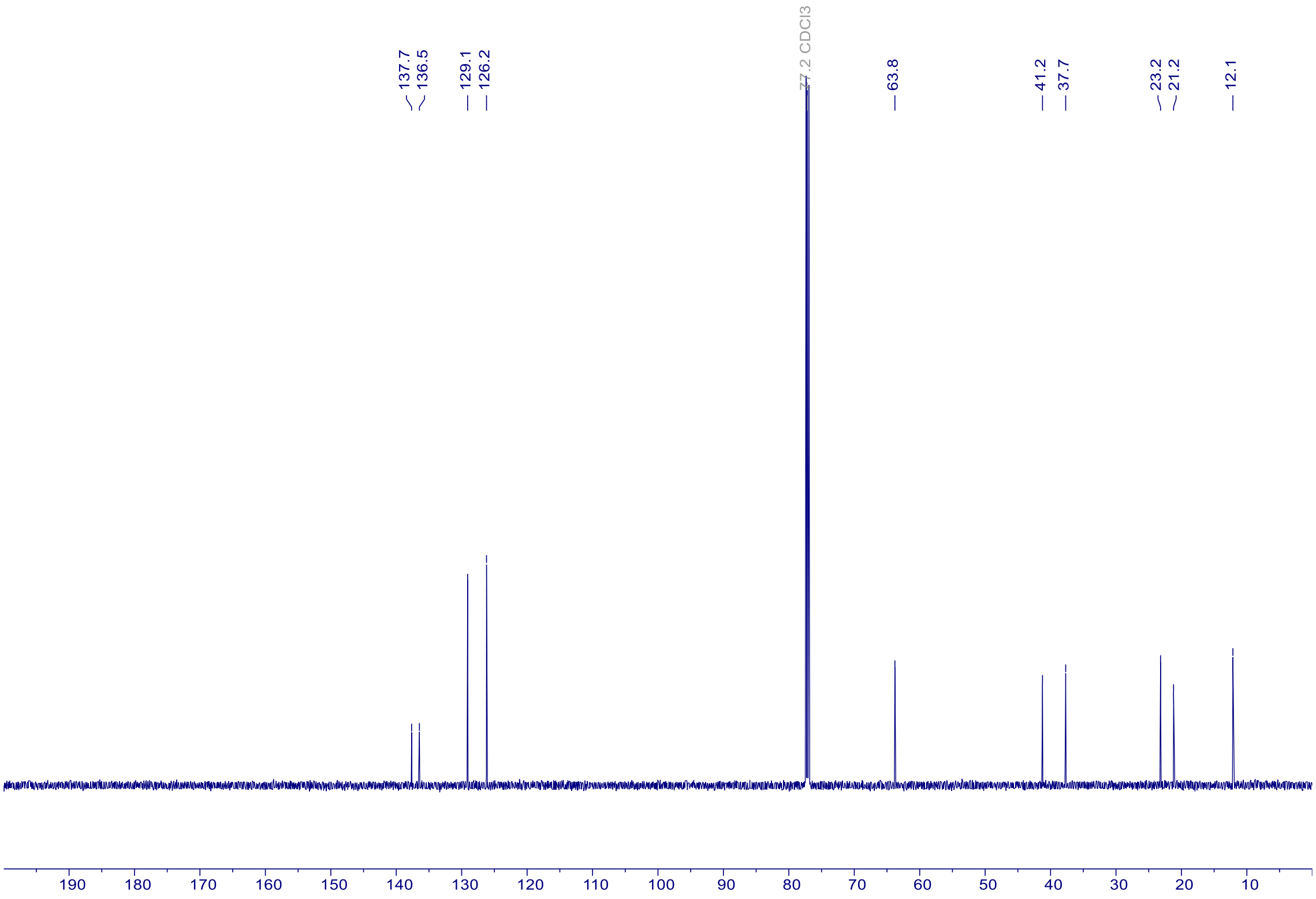

15 – ^{1}H NMR (600 MHz, $CDCl_3$)

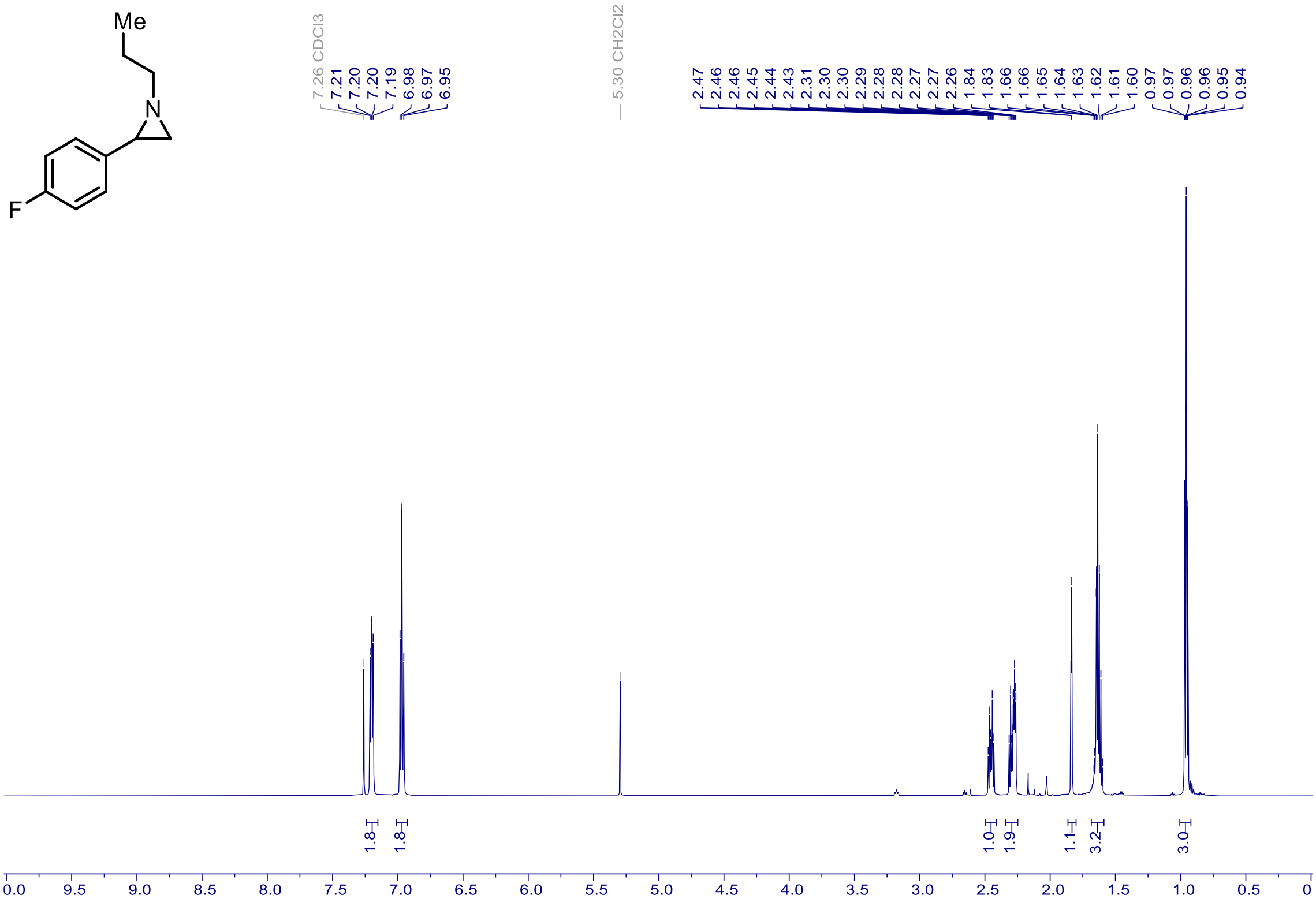

15 – ^{13}C NMR (151 MHz, $CDCl_3$)

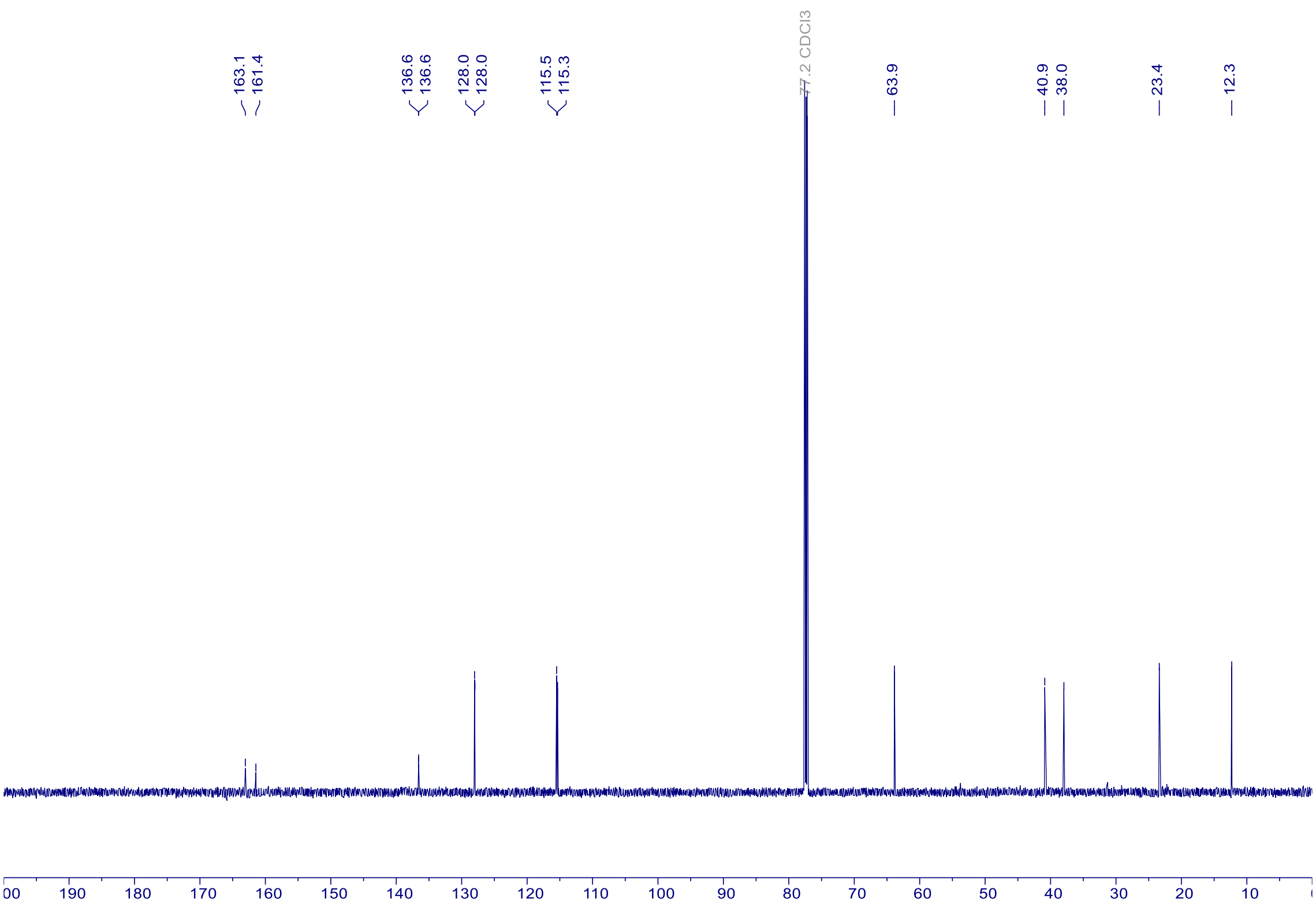

15 – ^{19}F NMR (565 MHz, $CDCl_3$)

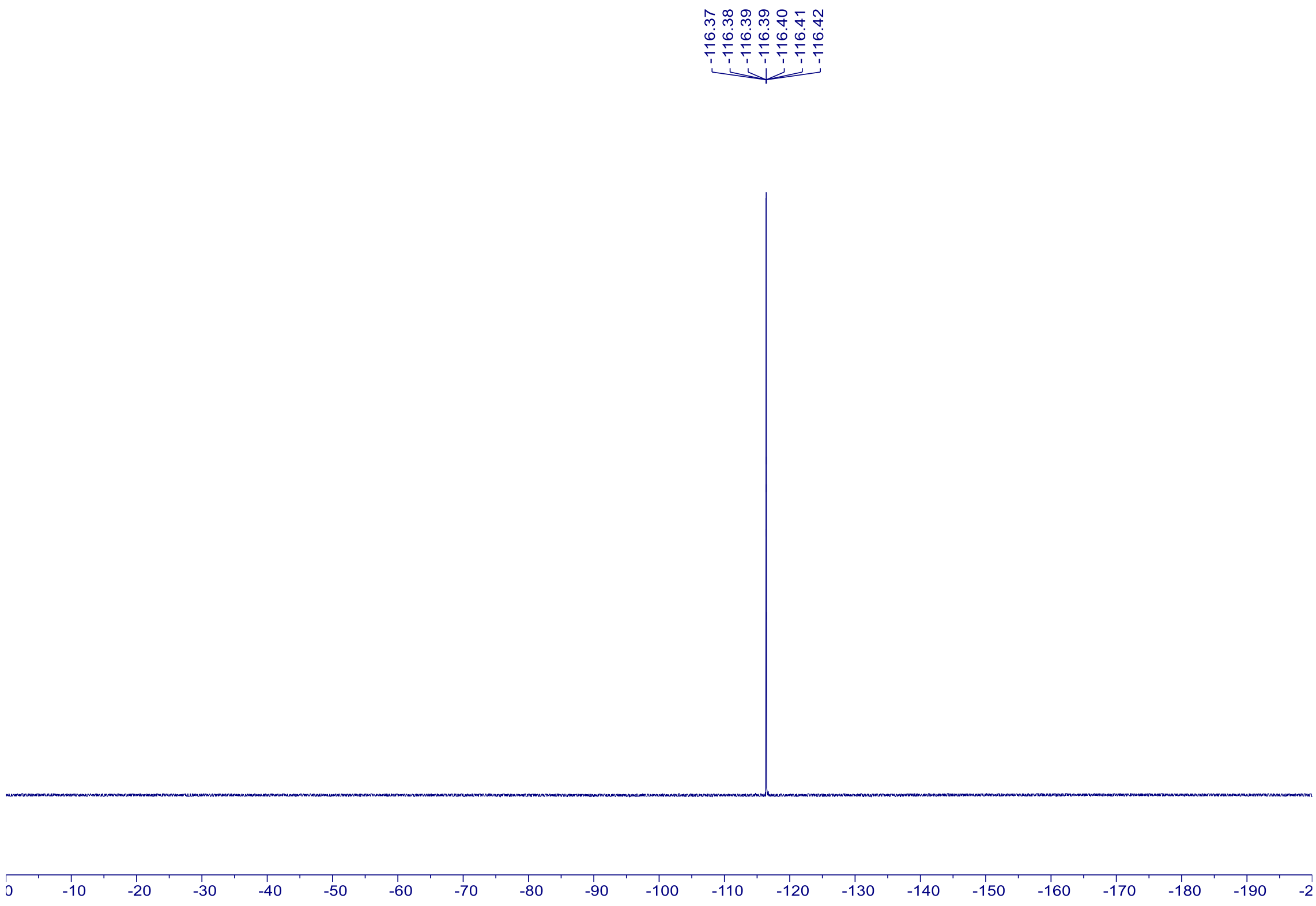

16 – ^{1}H NMR (400 MHz, $CDCl_3$)

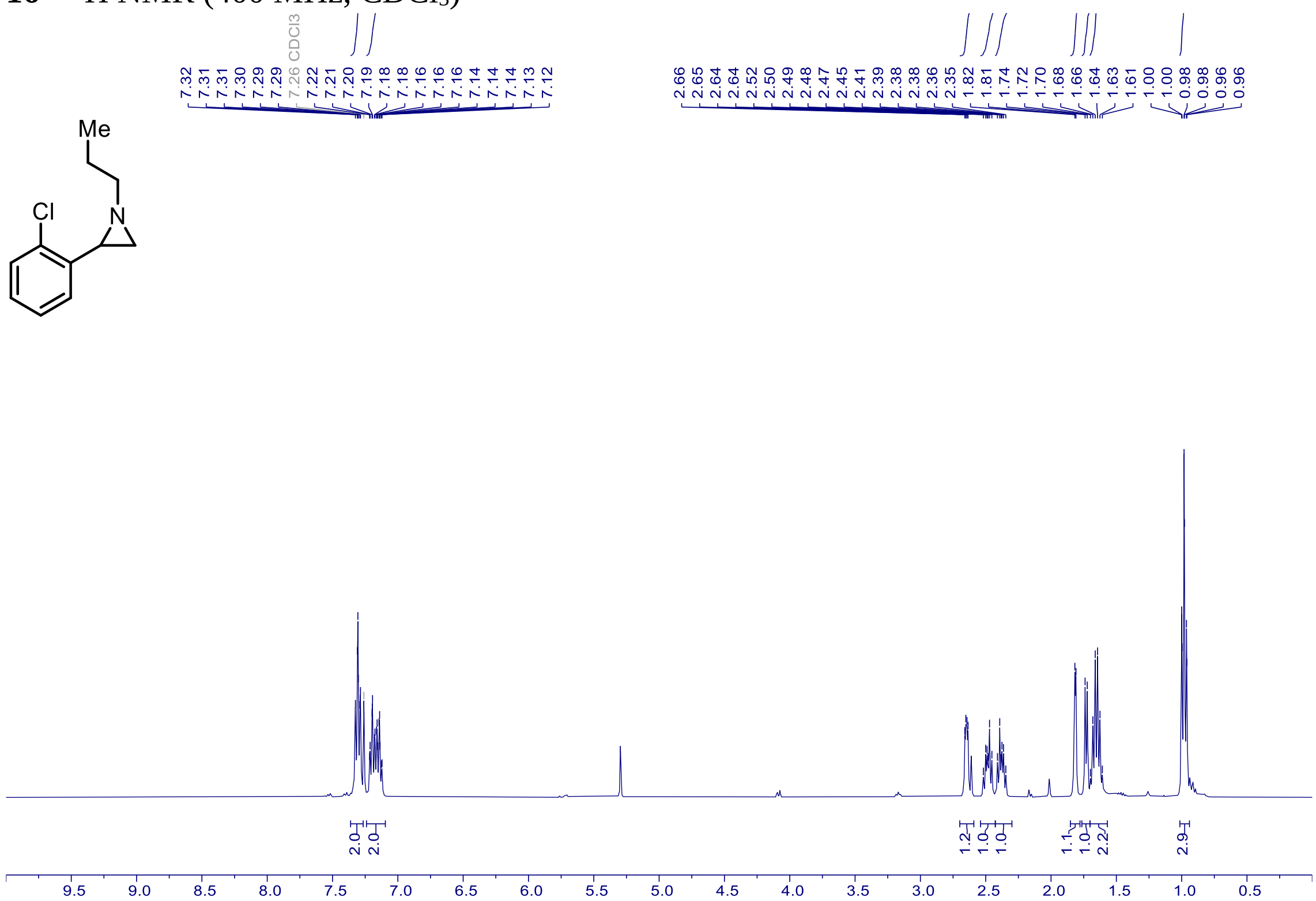

16 – ^{13}C NMR (101 MHz, $CDCl_3$)

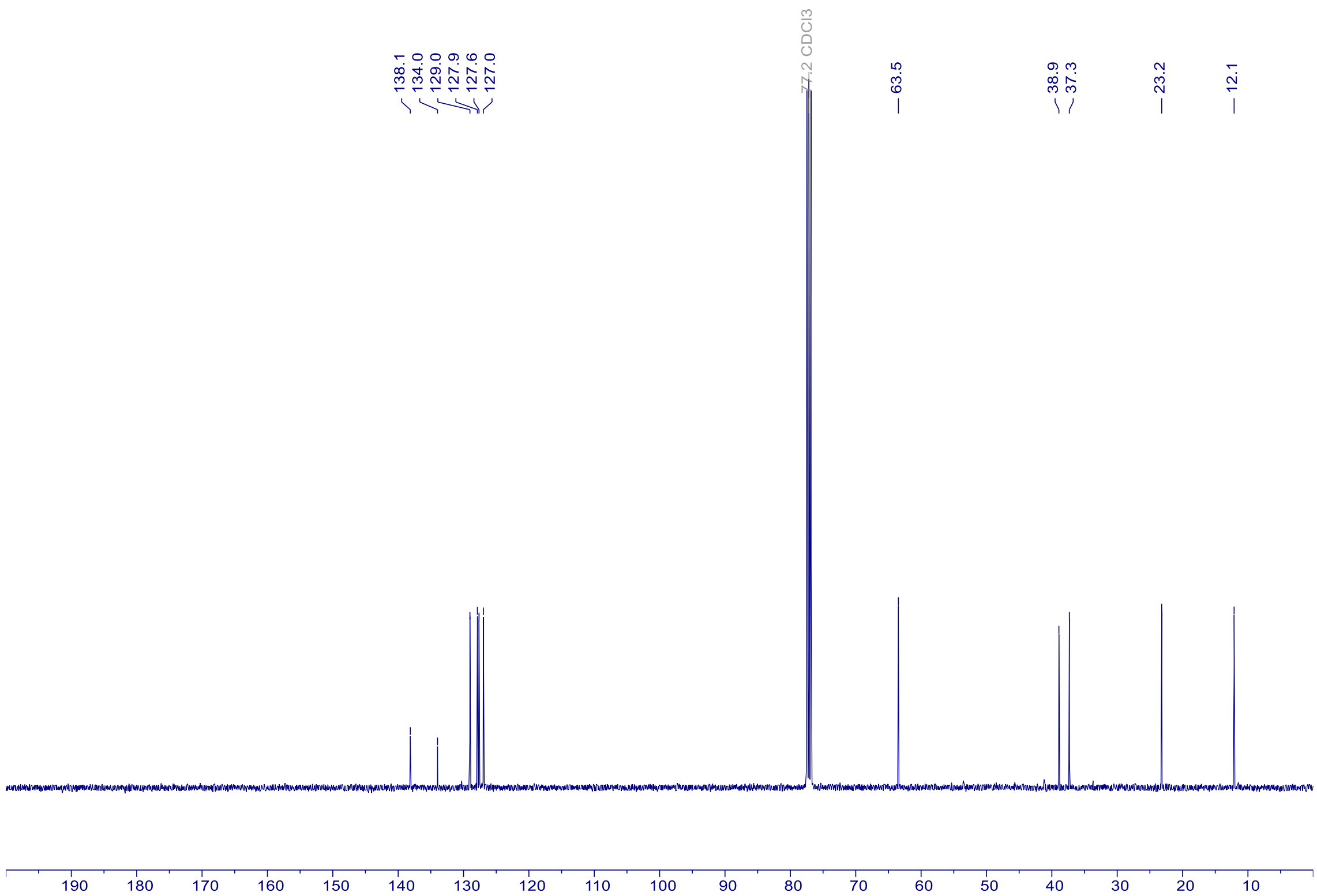

18 – ^{1}H NMR (600 MHz, $CDCl_3$)

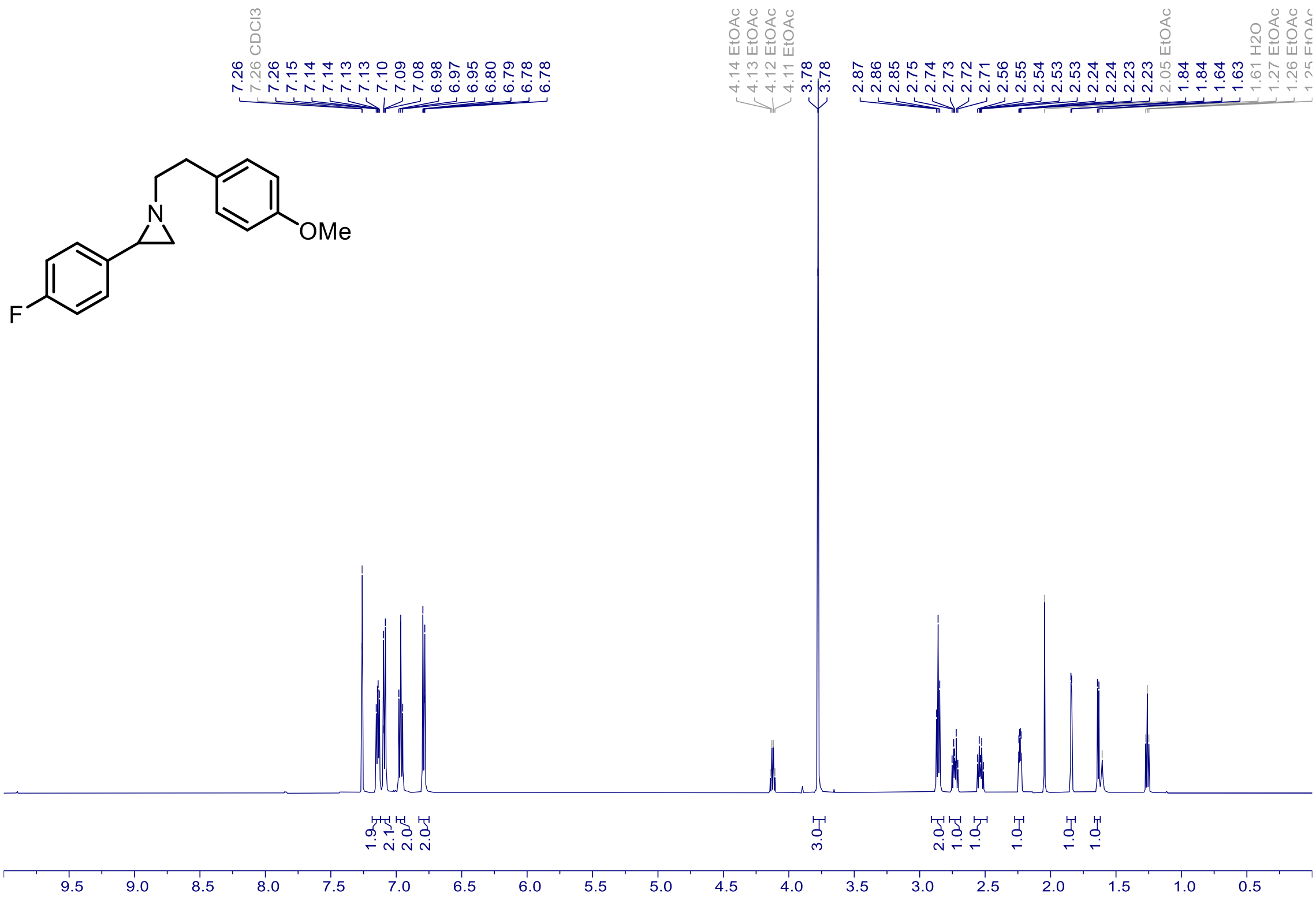

18 – ^{13}C NMR (151 MHz, $CDCl_3$)

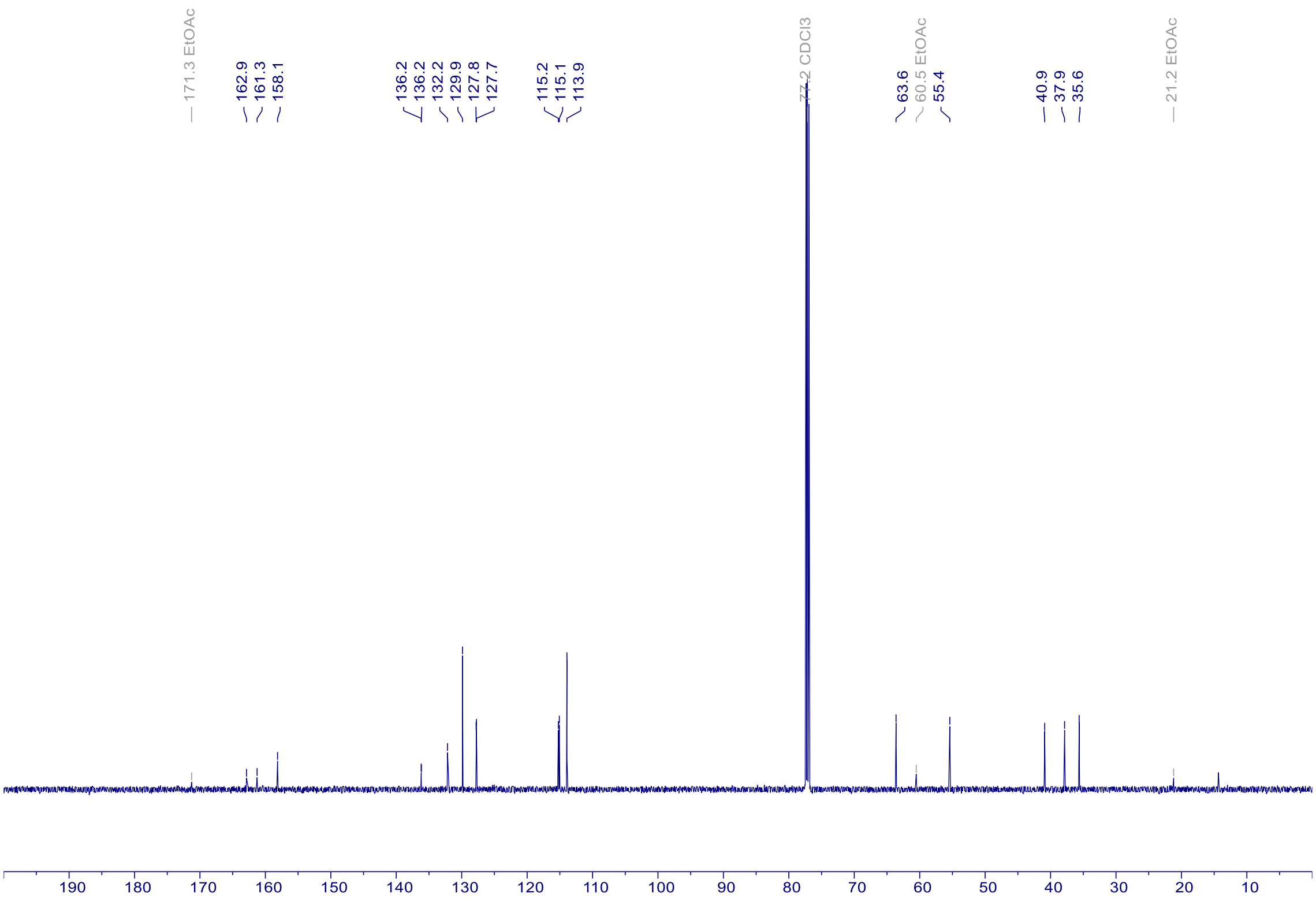

18 – ^{19}F NMR (565 MHz, $CDCl_3$)

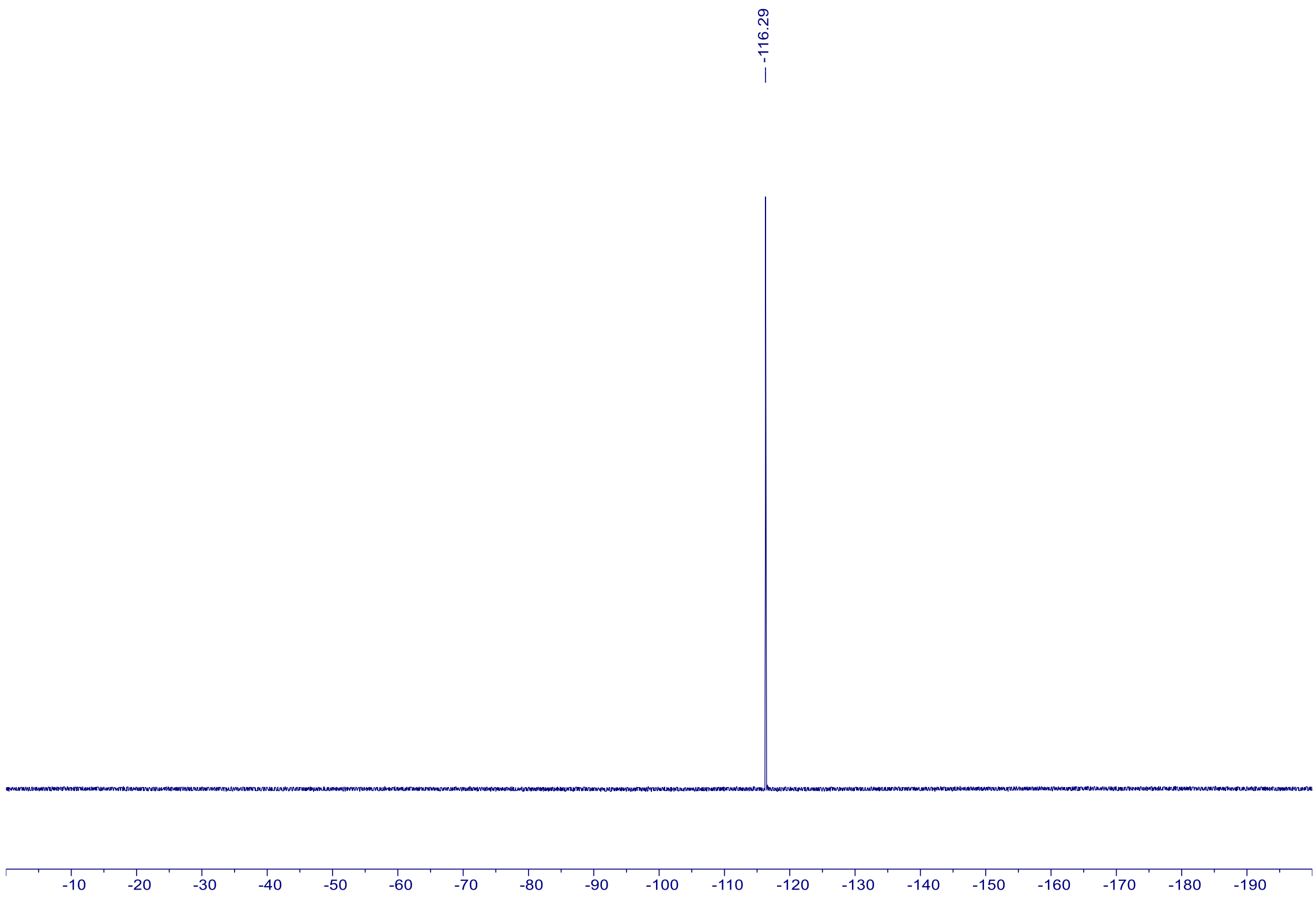

19 – ^{1}H NMR (400 MHz, $CDCl_3$)

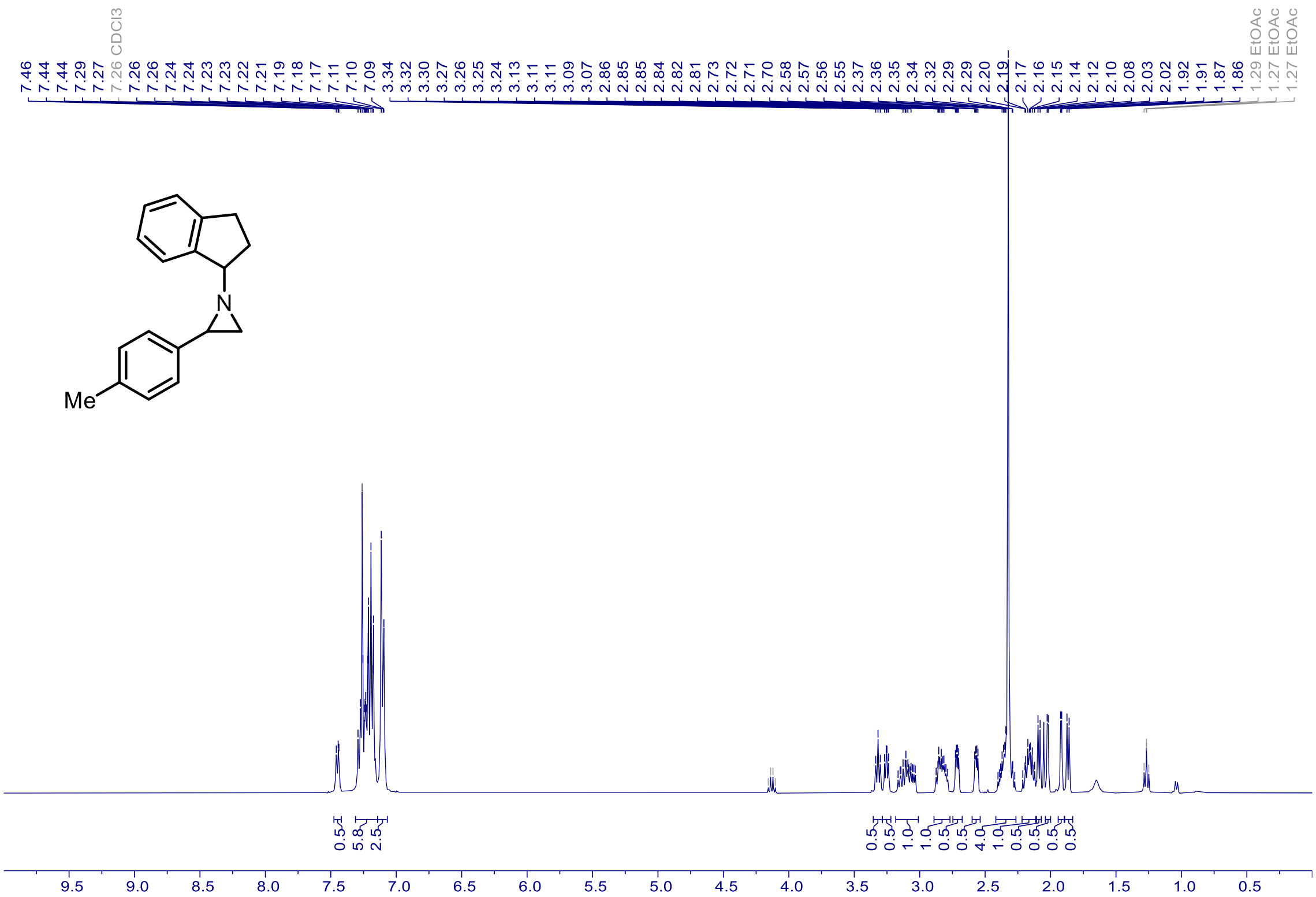

19 – ^{13}C NMR (101 MHz, $CDCl_3$)

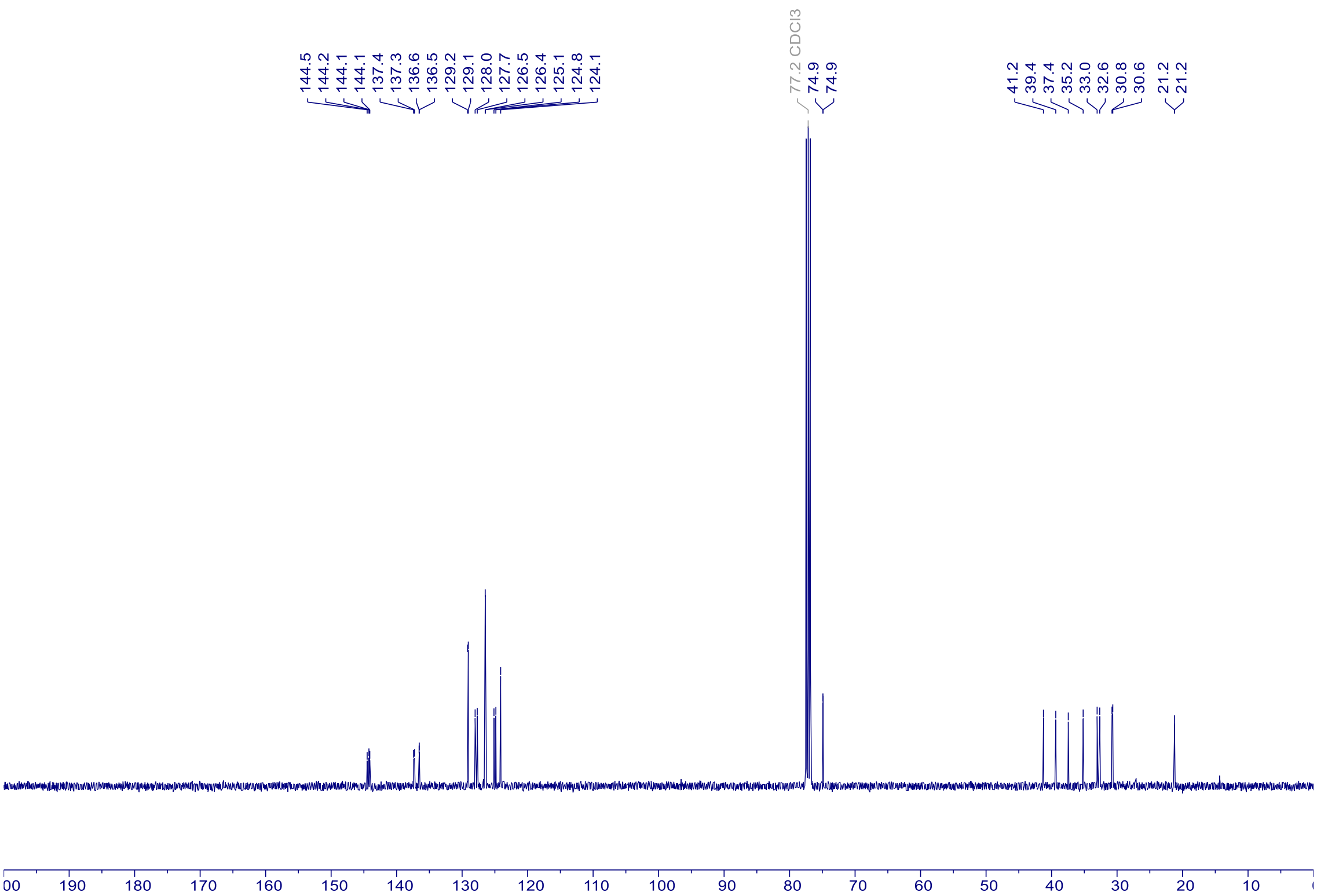

20 – ^{1}H NMR (600 MHz, $CDCl_3$)

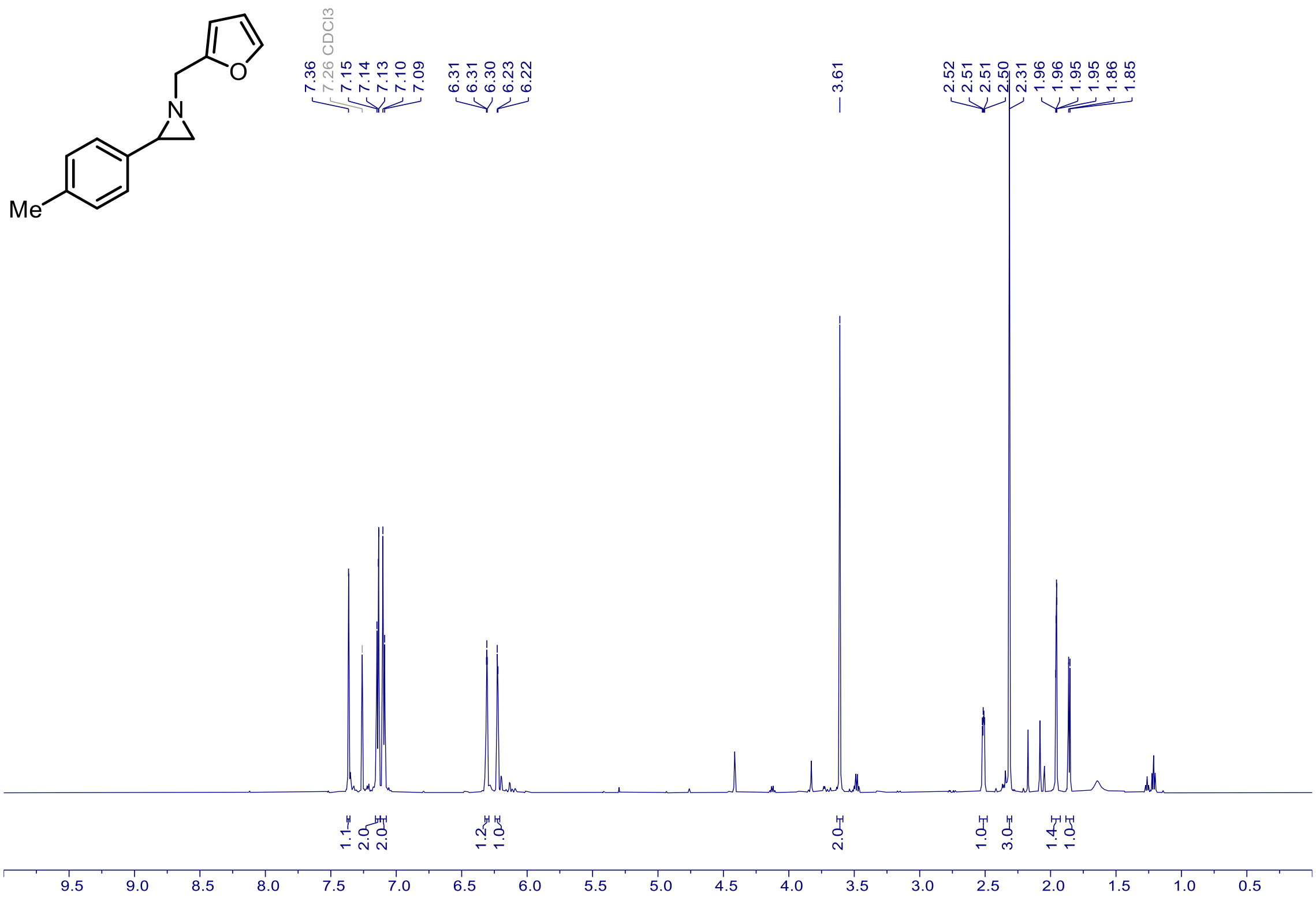

20 – ^{13}C NMR (151 MHz, $CDCl_3$)

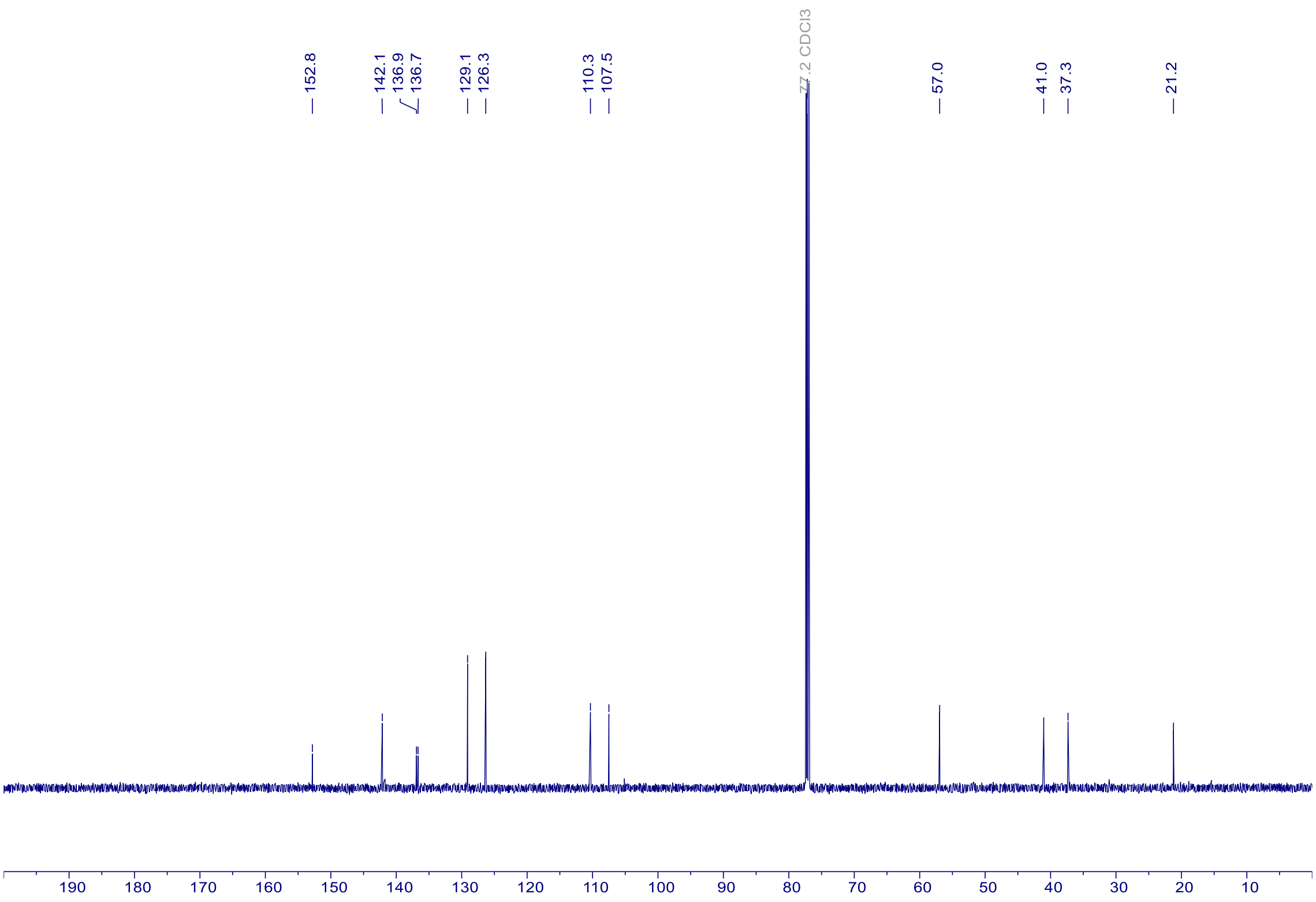

21 – ^{1}H NMR (600 MHz, CDCl$_3$)

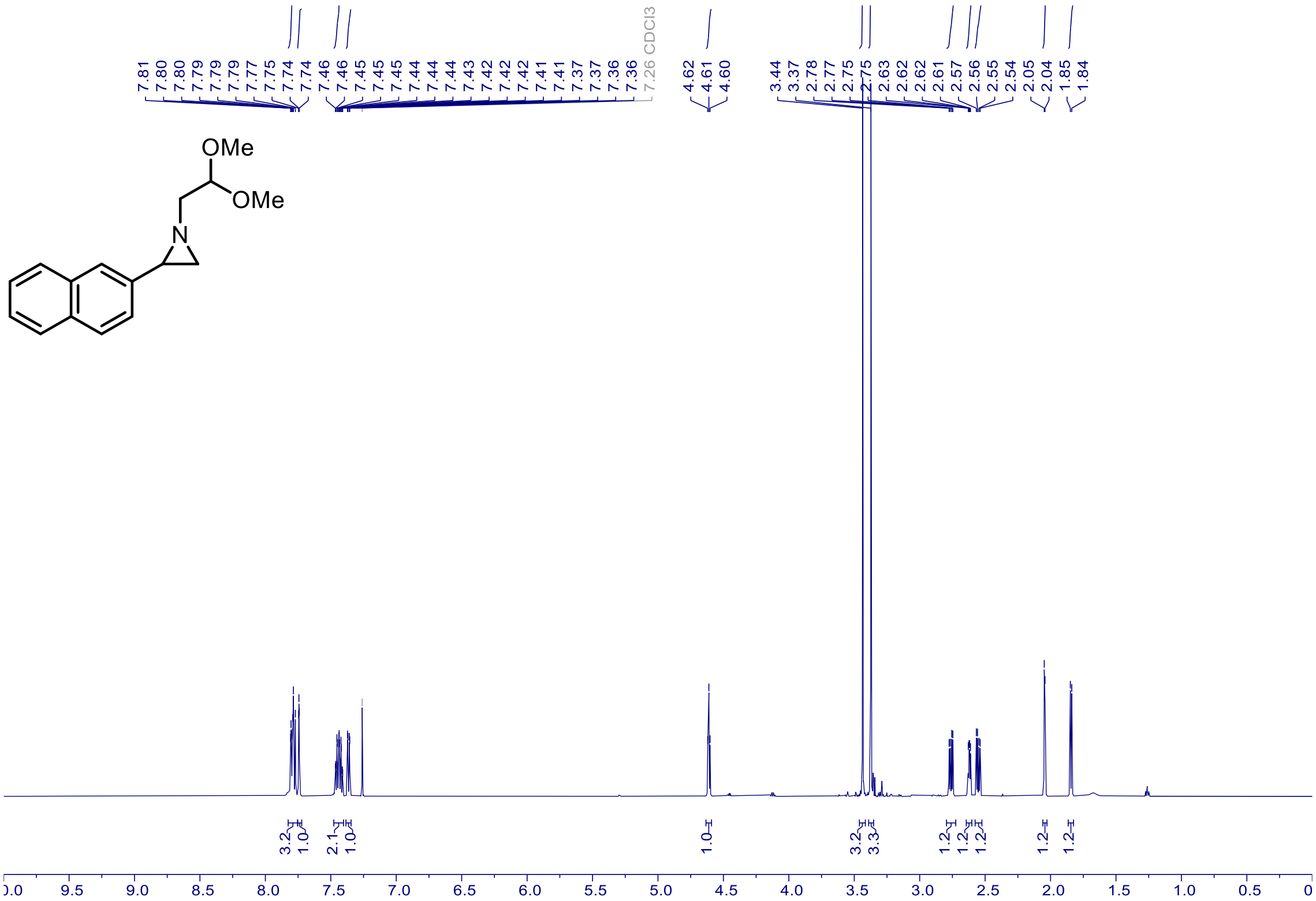

21 – ^{13}C NMR (151 MHz, CDCl$_3$)

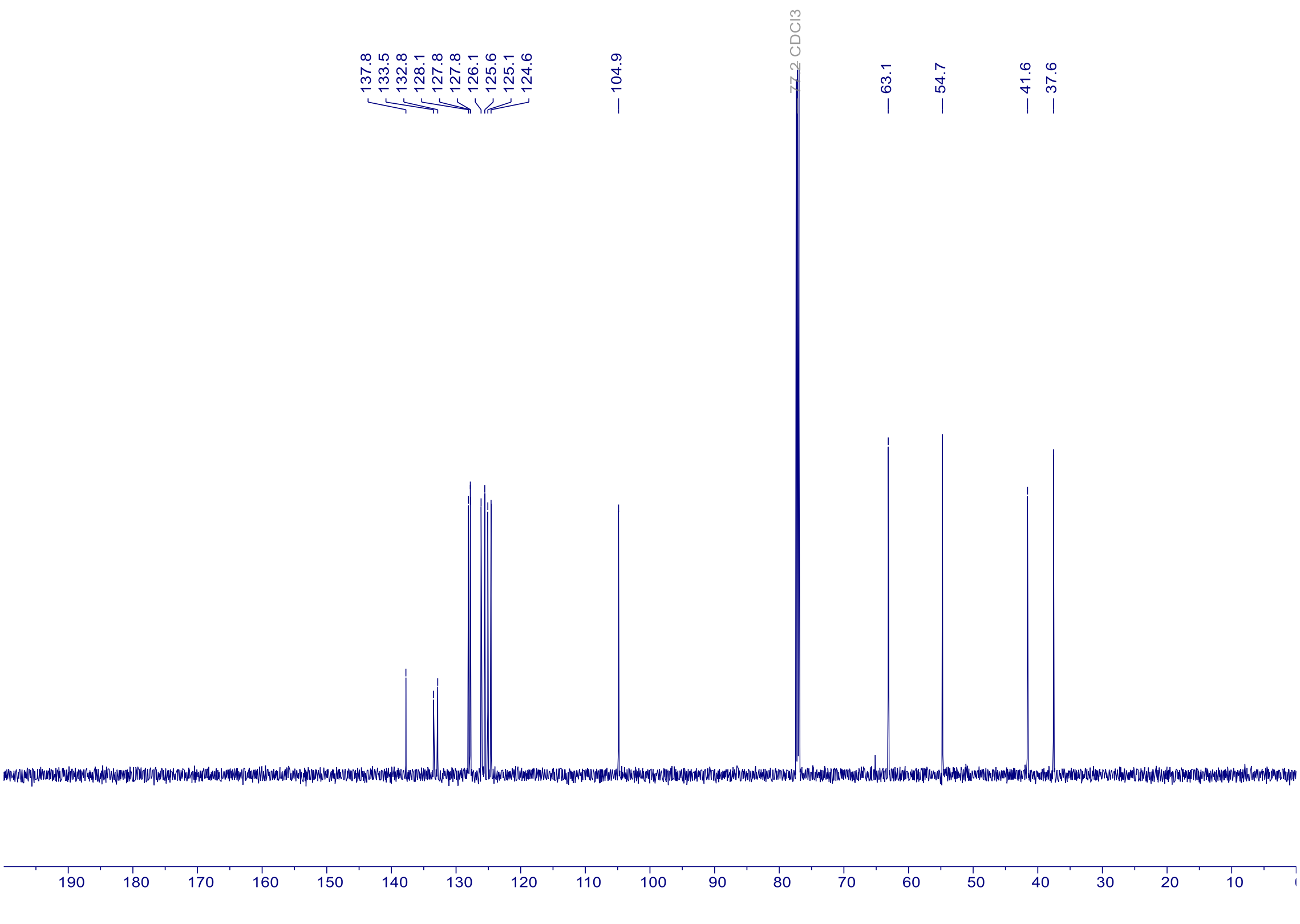

22 – ^{1}H NMR (400 MHz, $CDCl_3$)

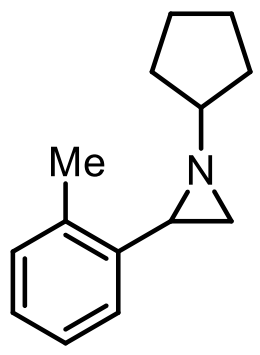

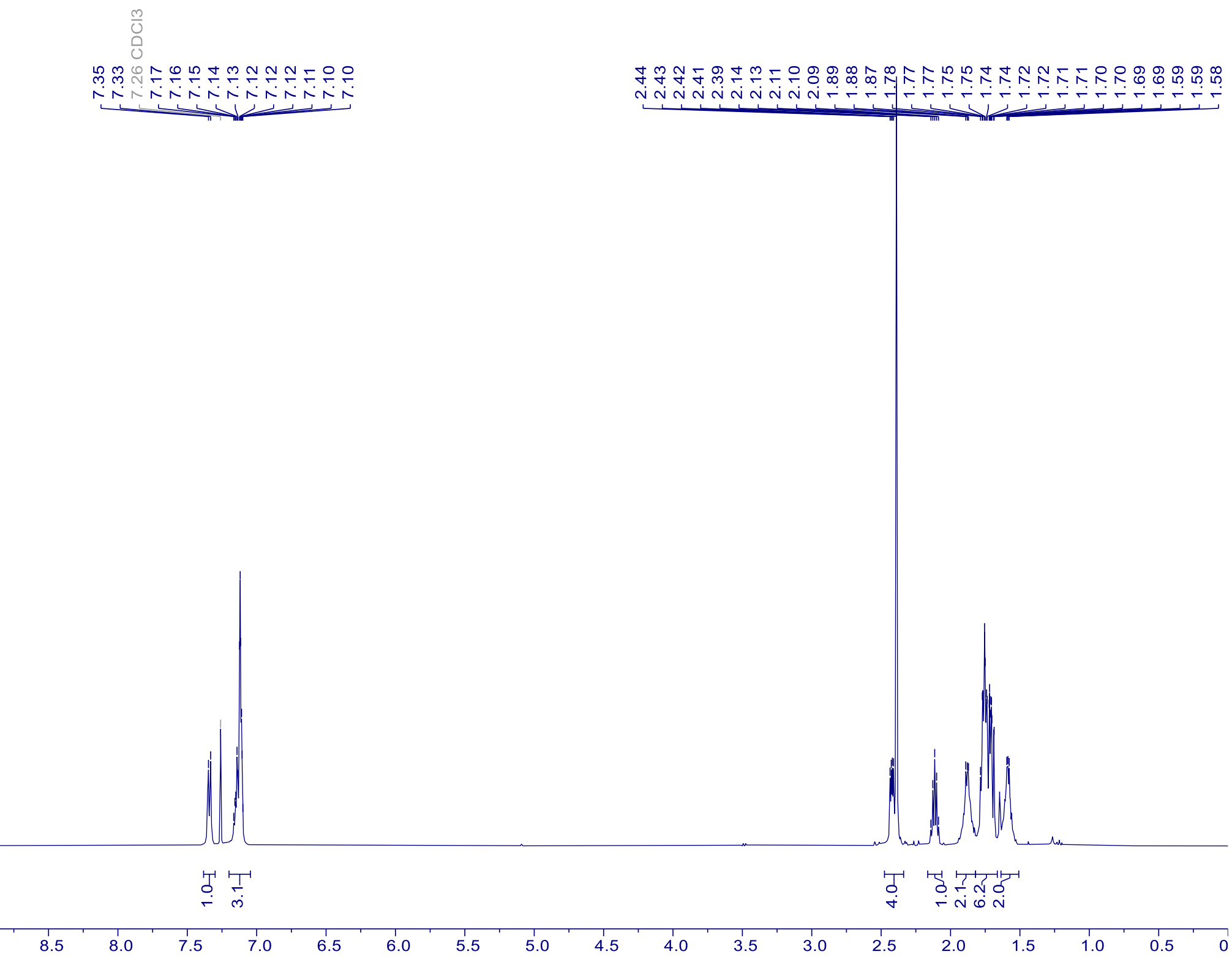

22 – ^{13}C NMR (101 MHz, $CDCl_3$)

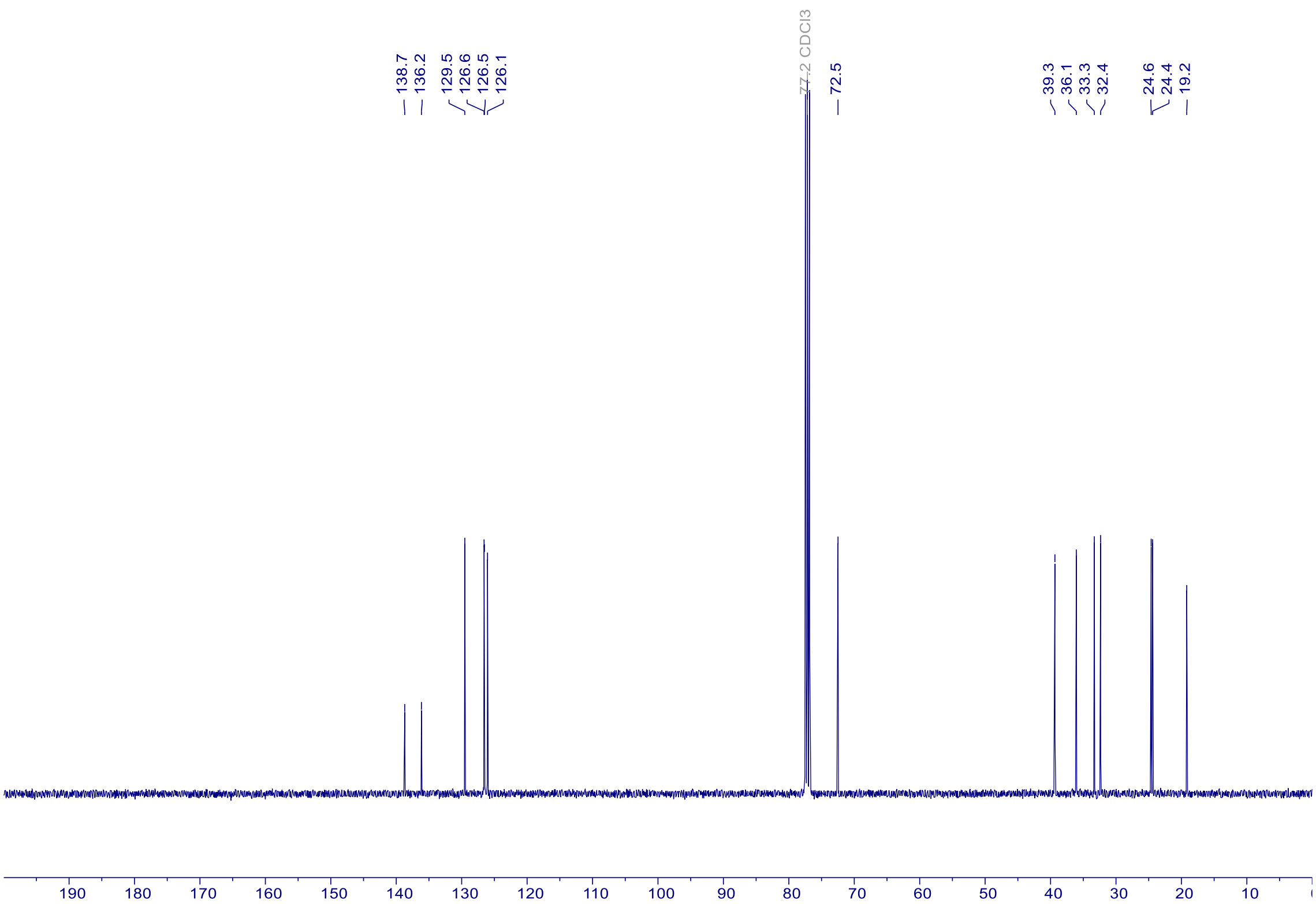

23 – ^{1}H NMR (600 MHz, $CDCl_3$)

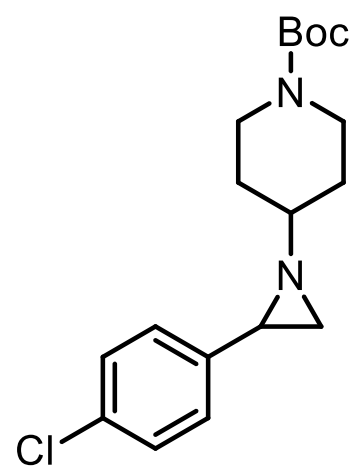

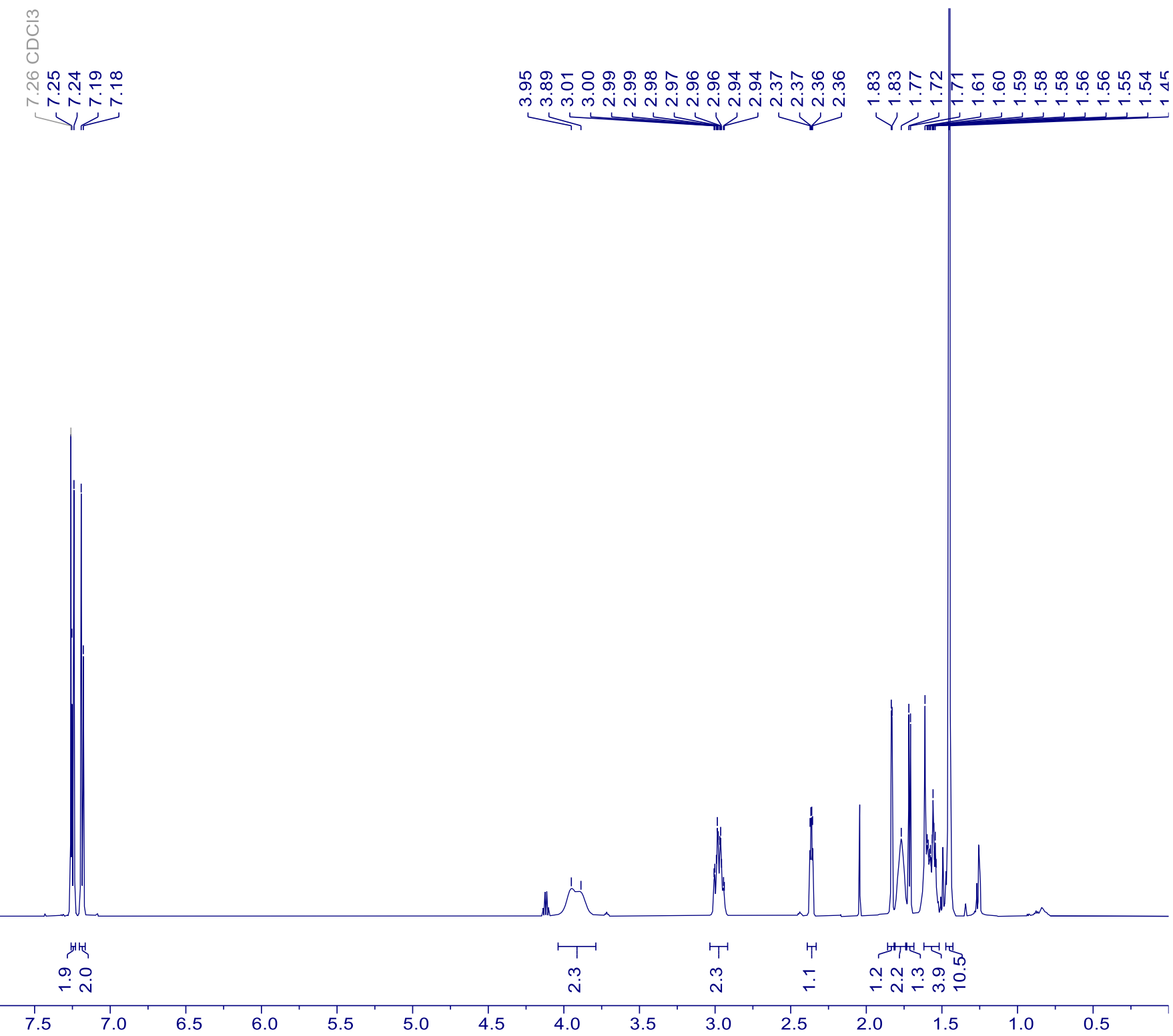

23 – ^{13}C NMR (151 MHz, $CDCl_3$)

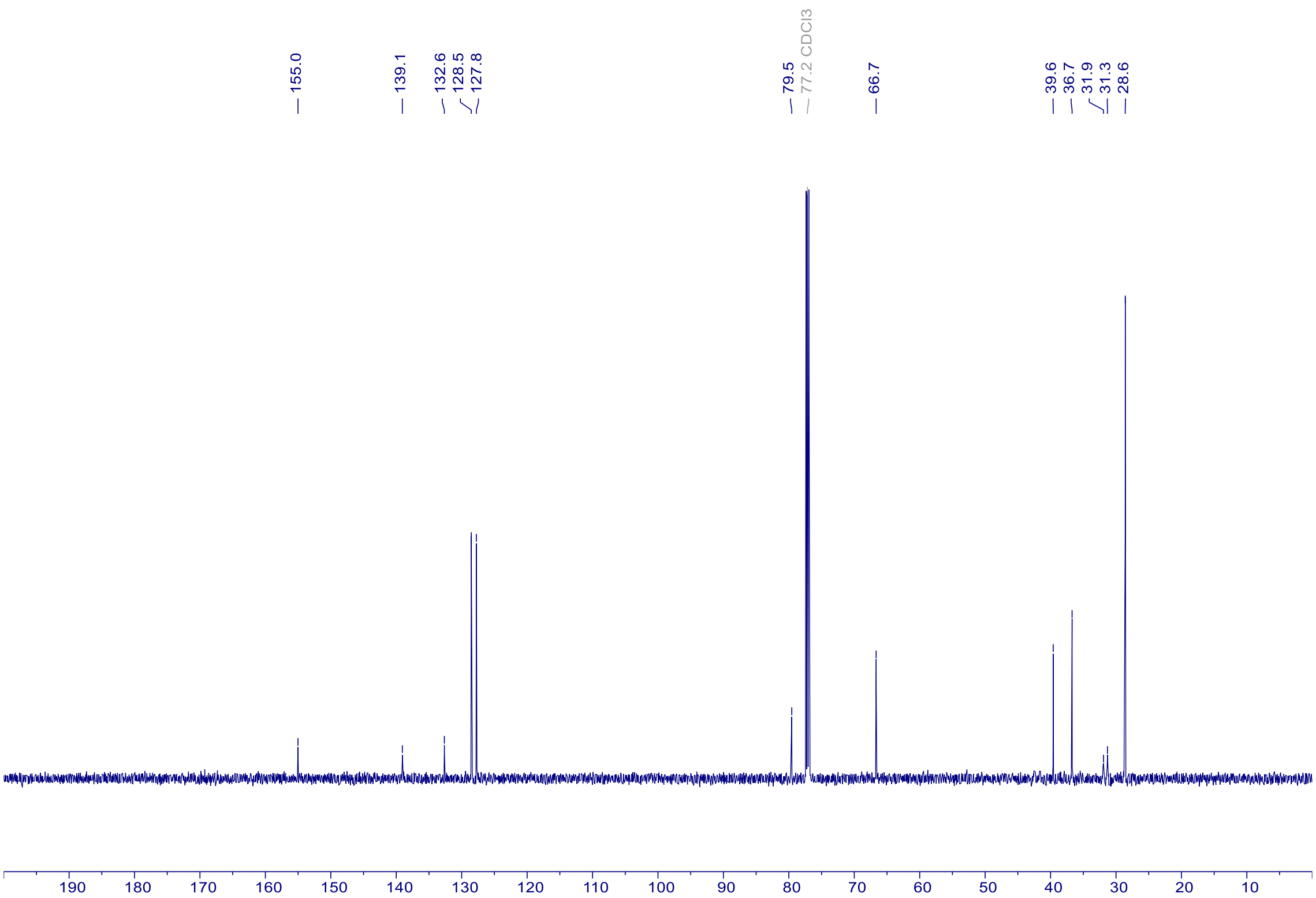

25 – ^{1}H NMR (400 MHz, $CDCl_3$)

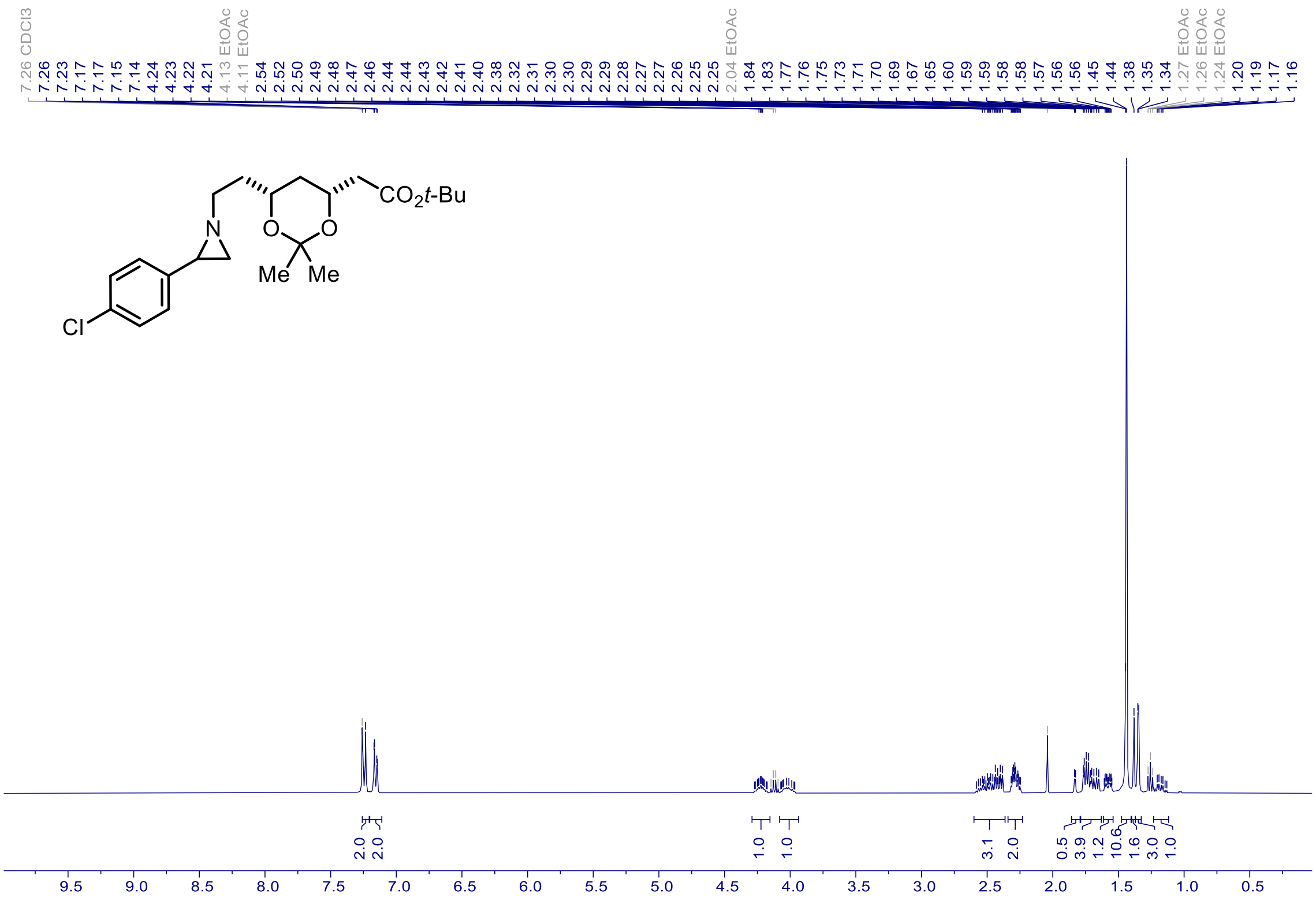

25 – ^{13}C NMR (101 MHz, $CDCl_3$)

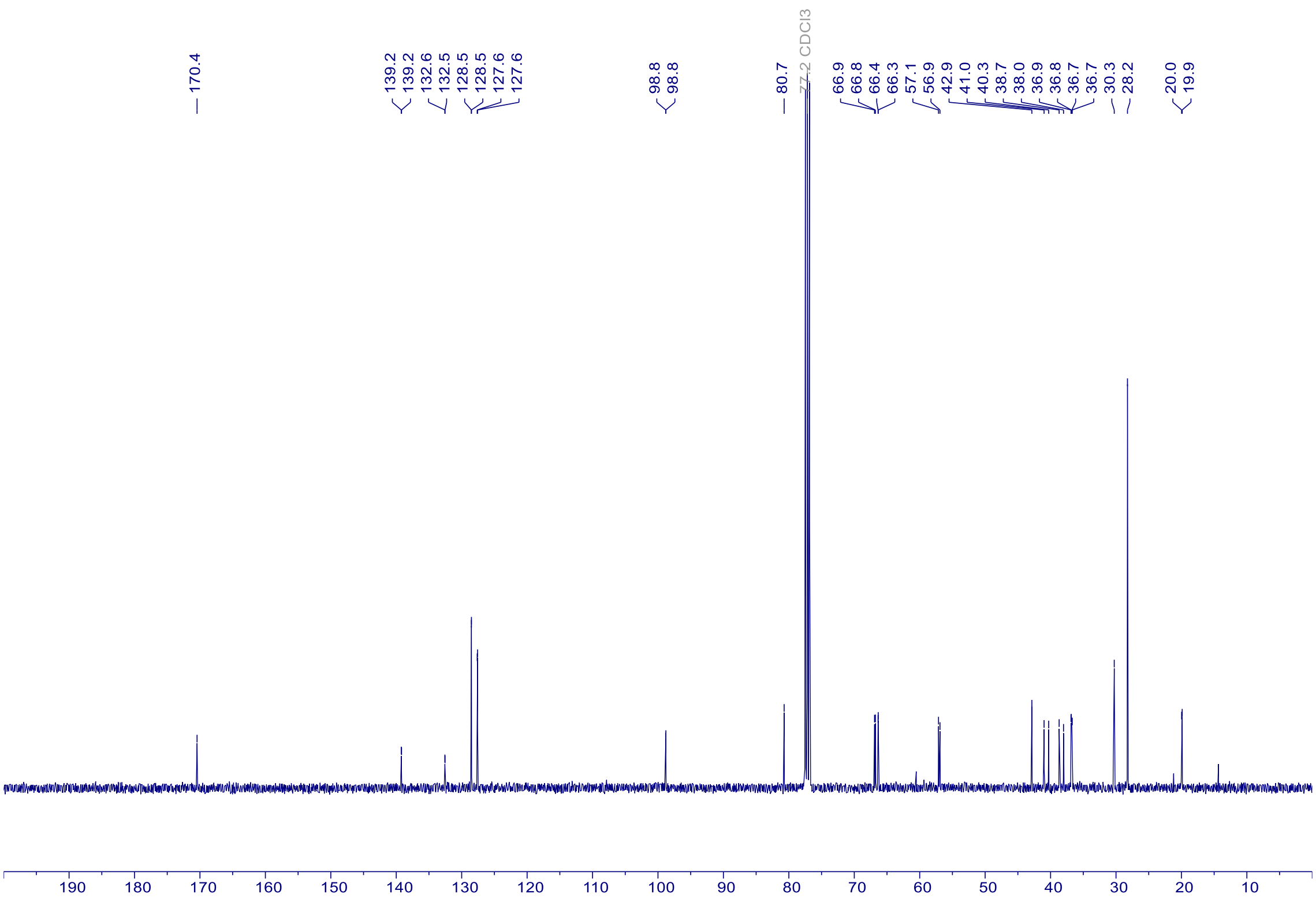

26 – ^{1}H NMR (600 MHz, $CDCl_3$)

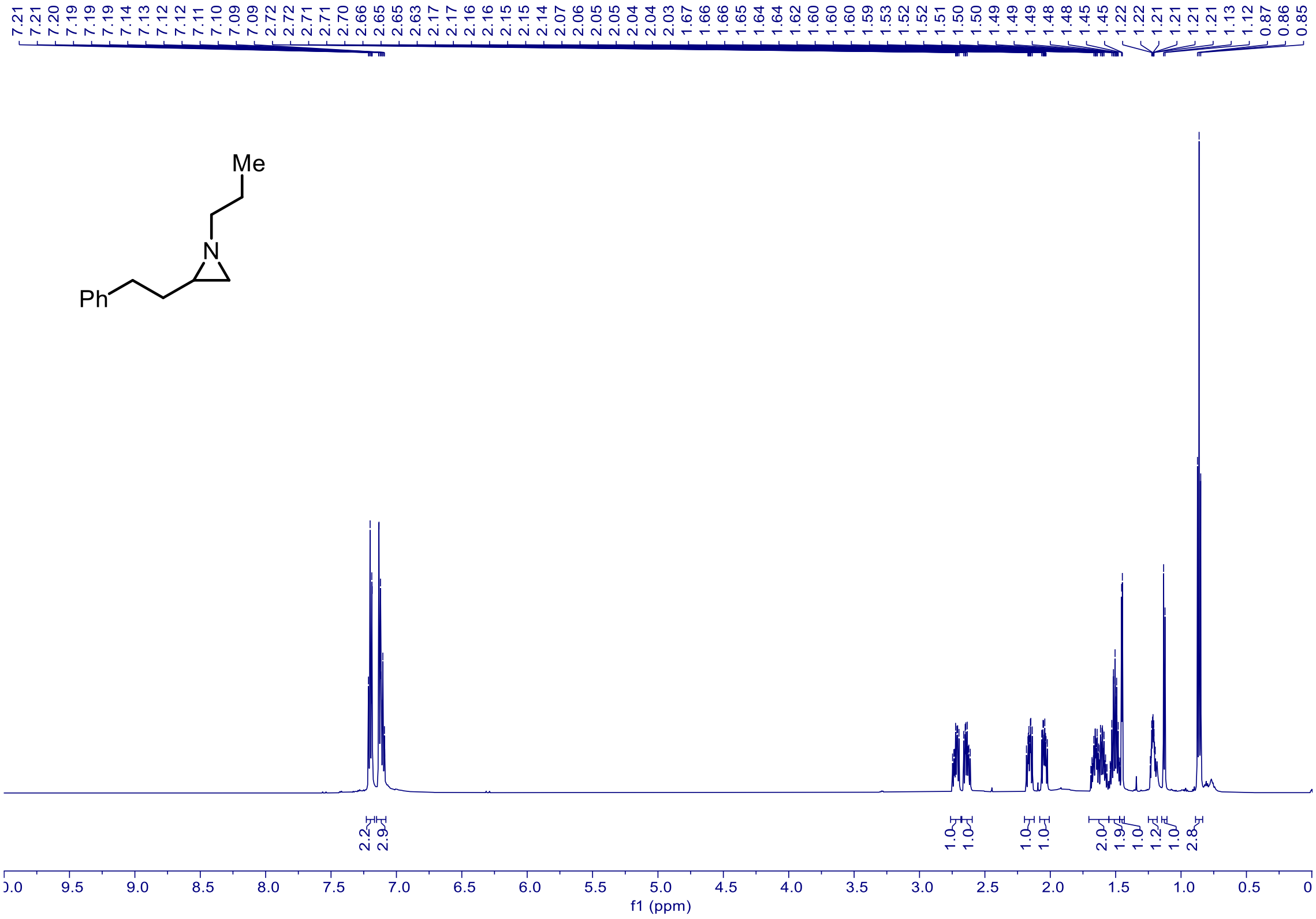

26 – ^{13}C NMR (151 MHz, $CDCl_3$)

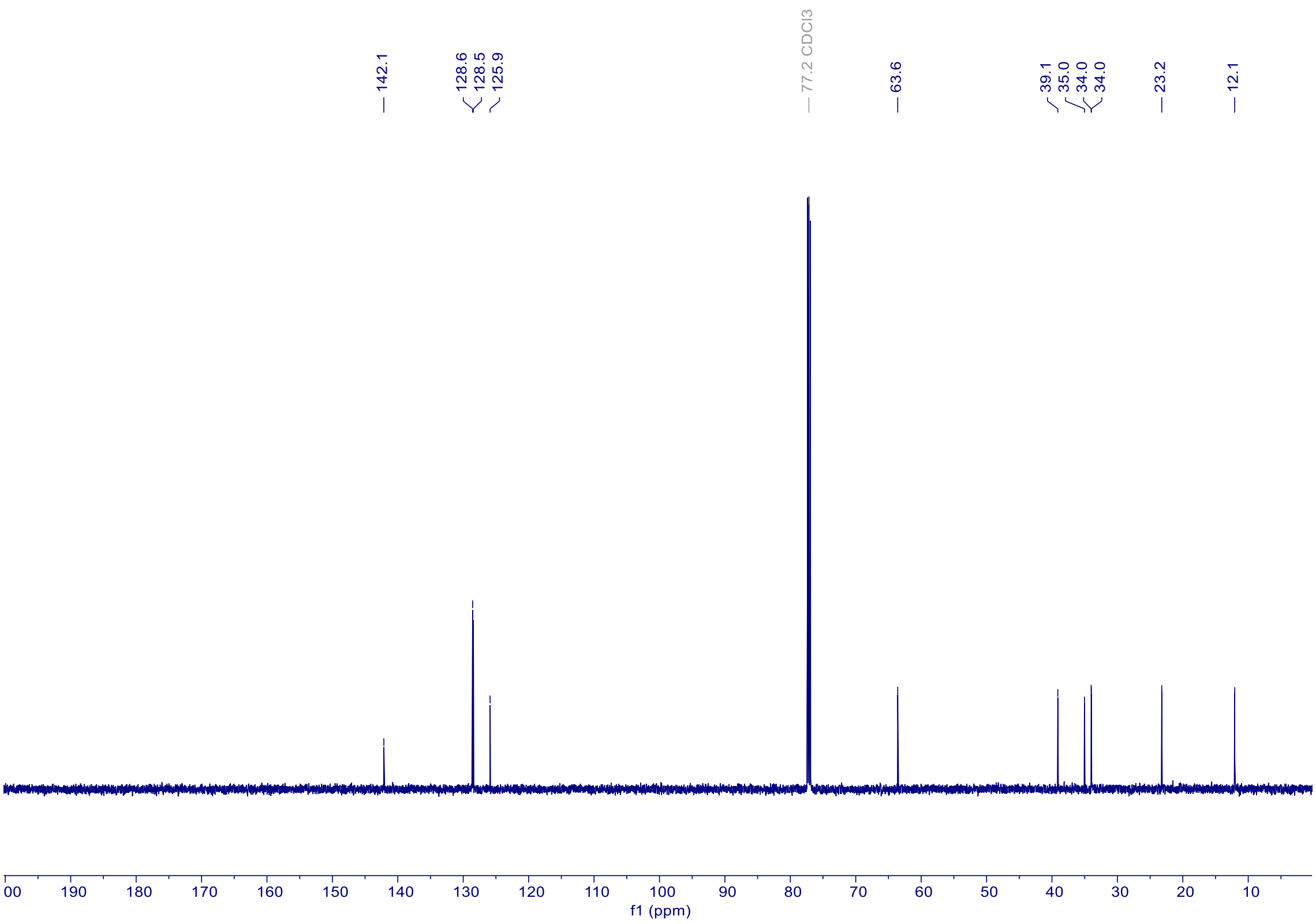

S30 – ^{1}H NMR (600 MHz, CDCl$_3$)

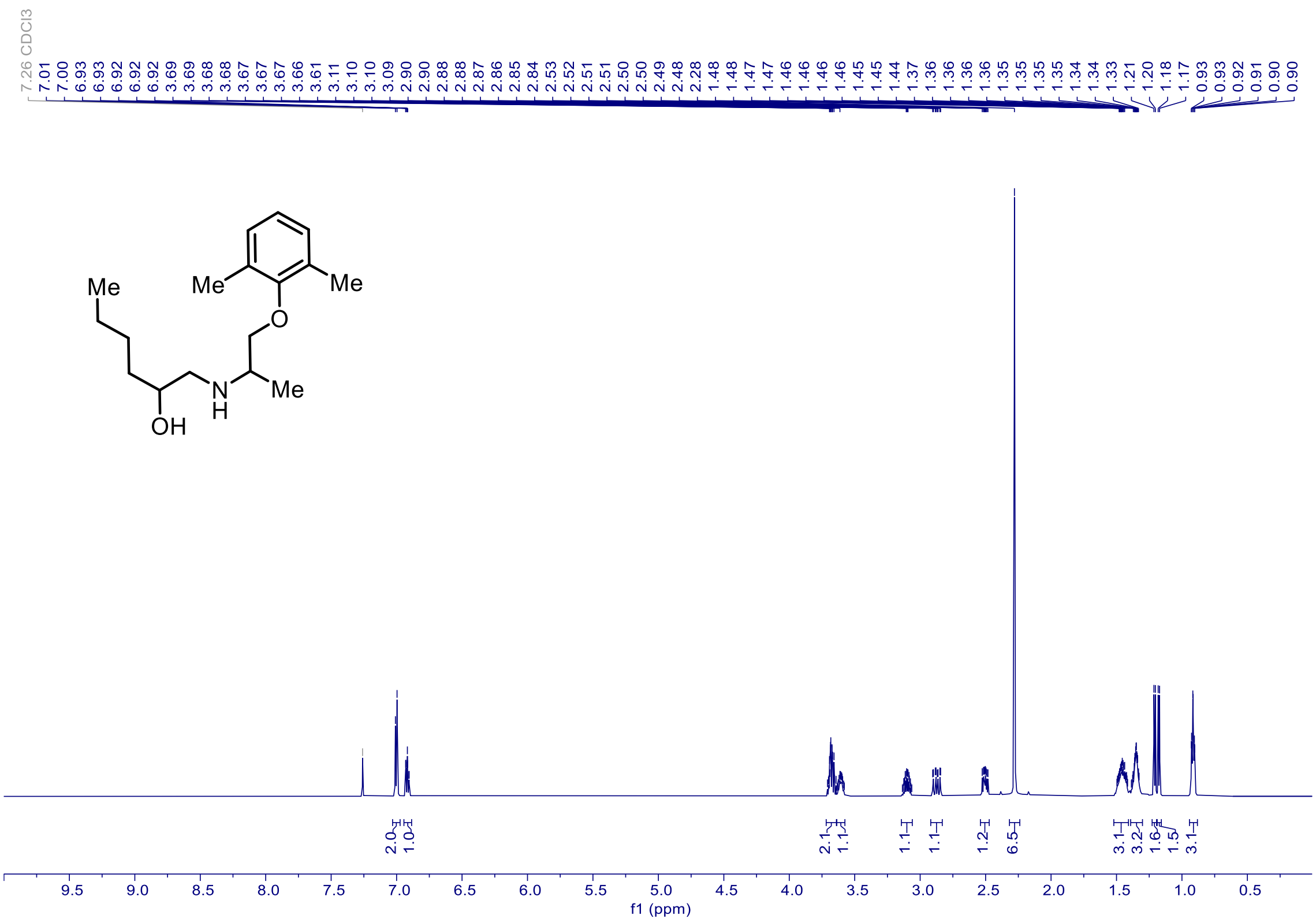

S30 – ^{13}C NMR (151 MHz, CDCl$_3$)

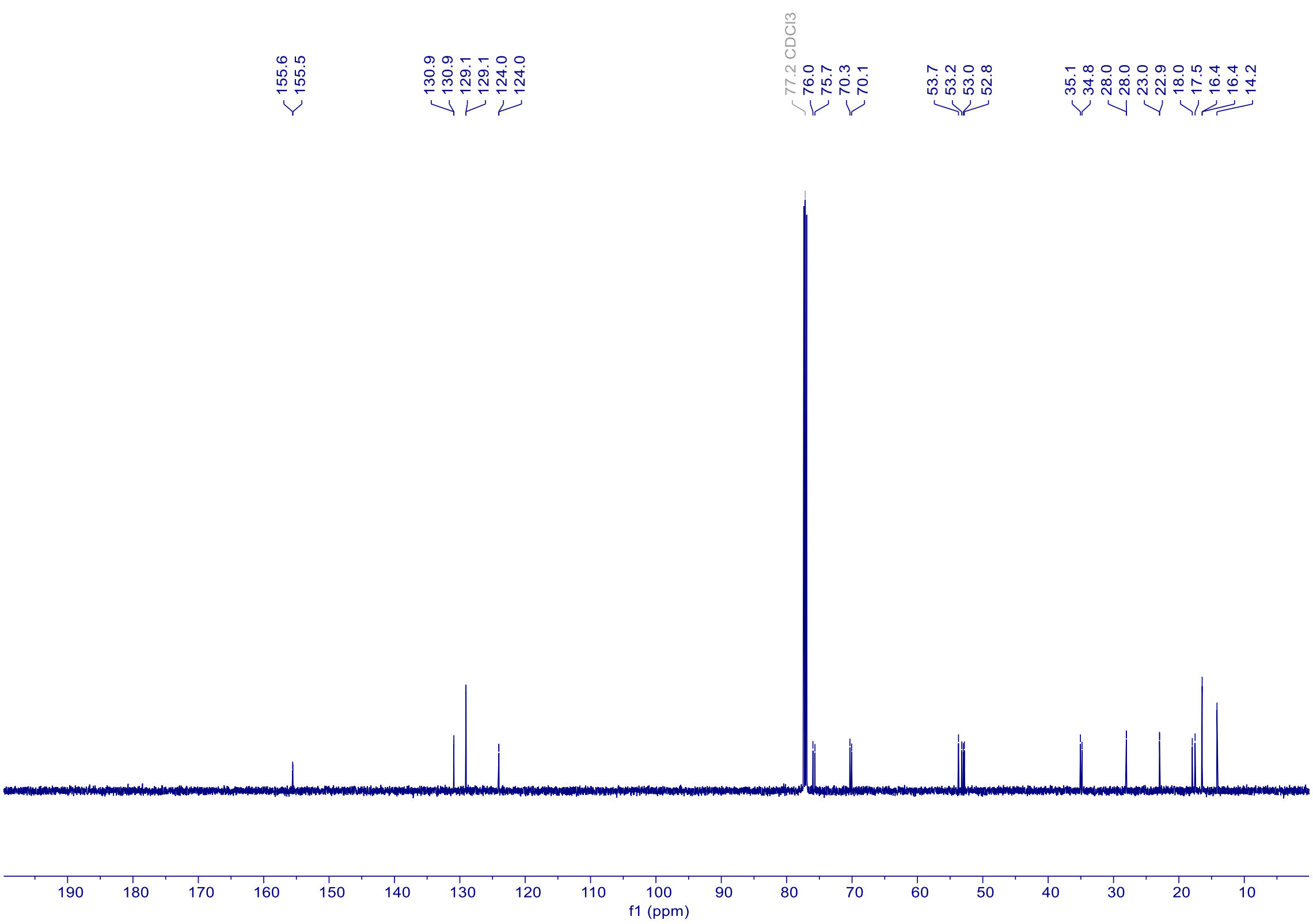

30 – ^{1}H NMR (600 MHz, $CDCl_3$)

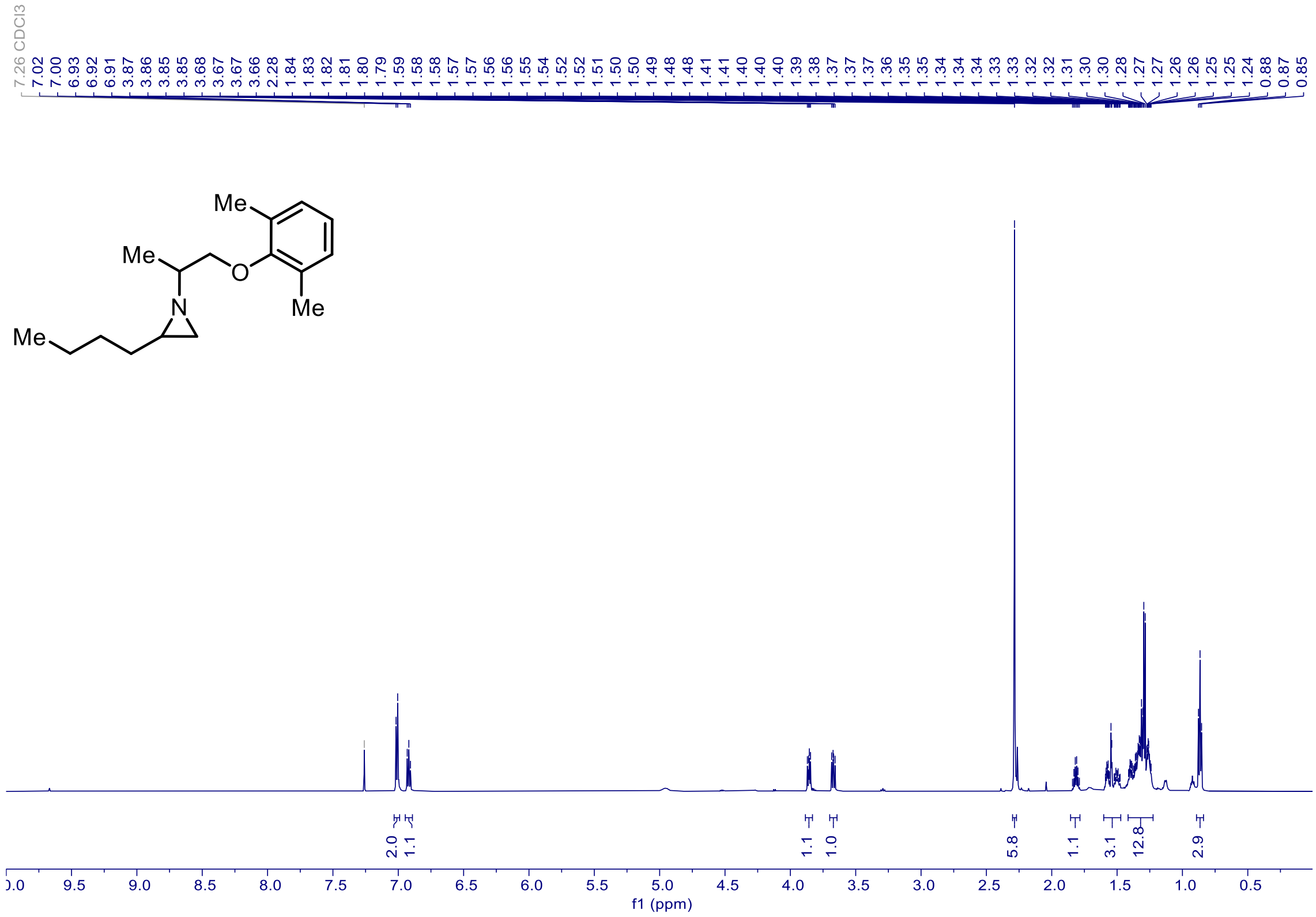

30 – ^{13}C NMR (151 MHz, $CDCl_3$)

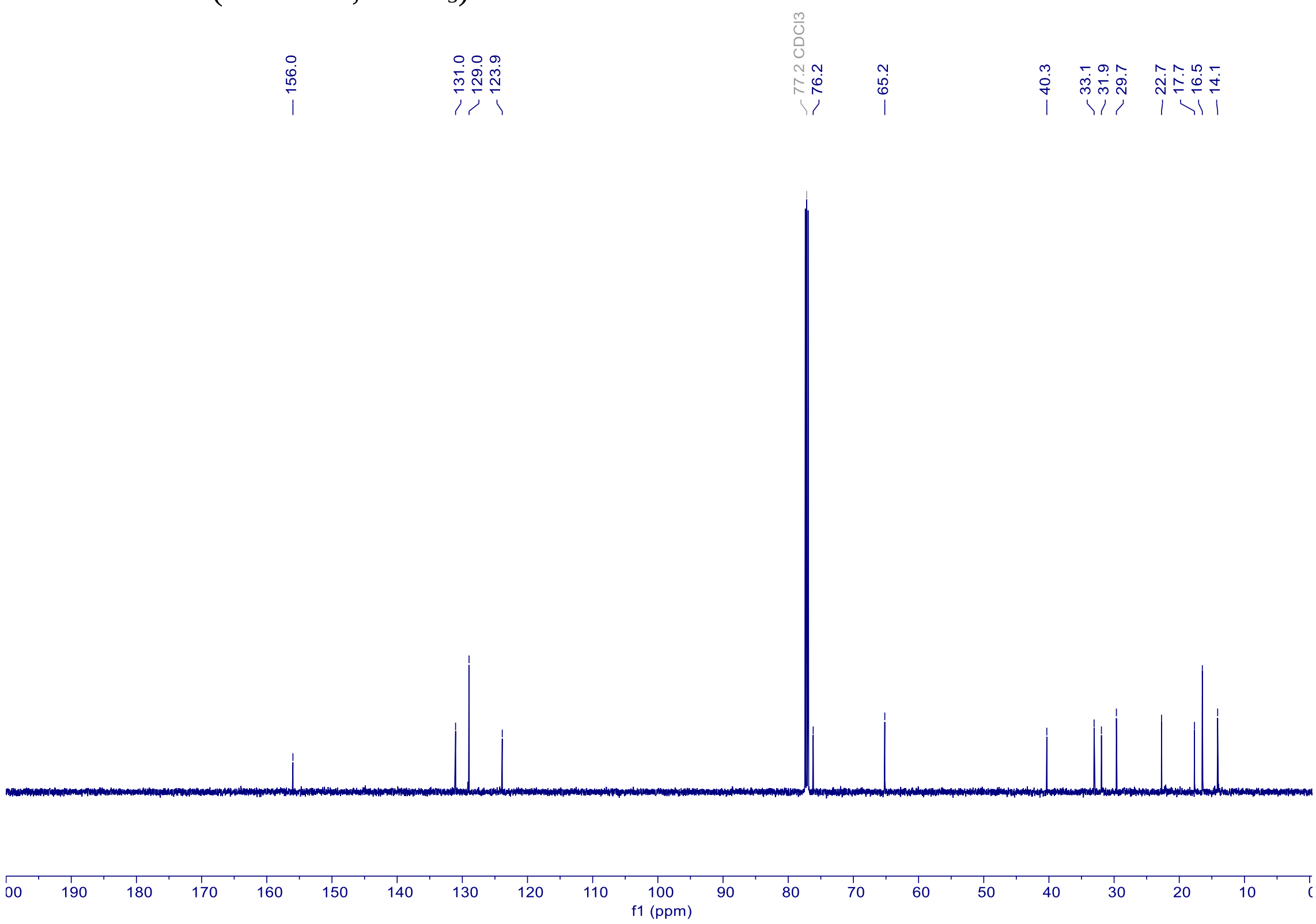

36 – ^{1}H NMR (600 MHz, $CDCl_3$)

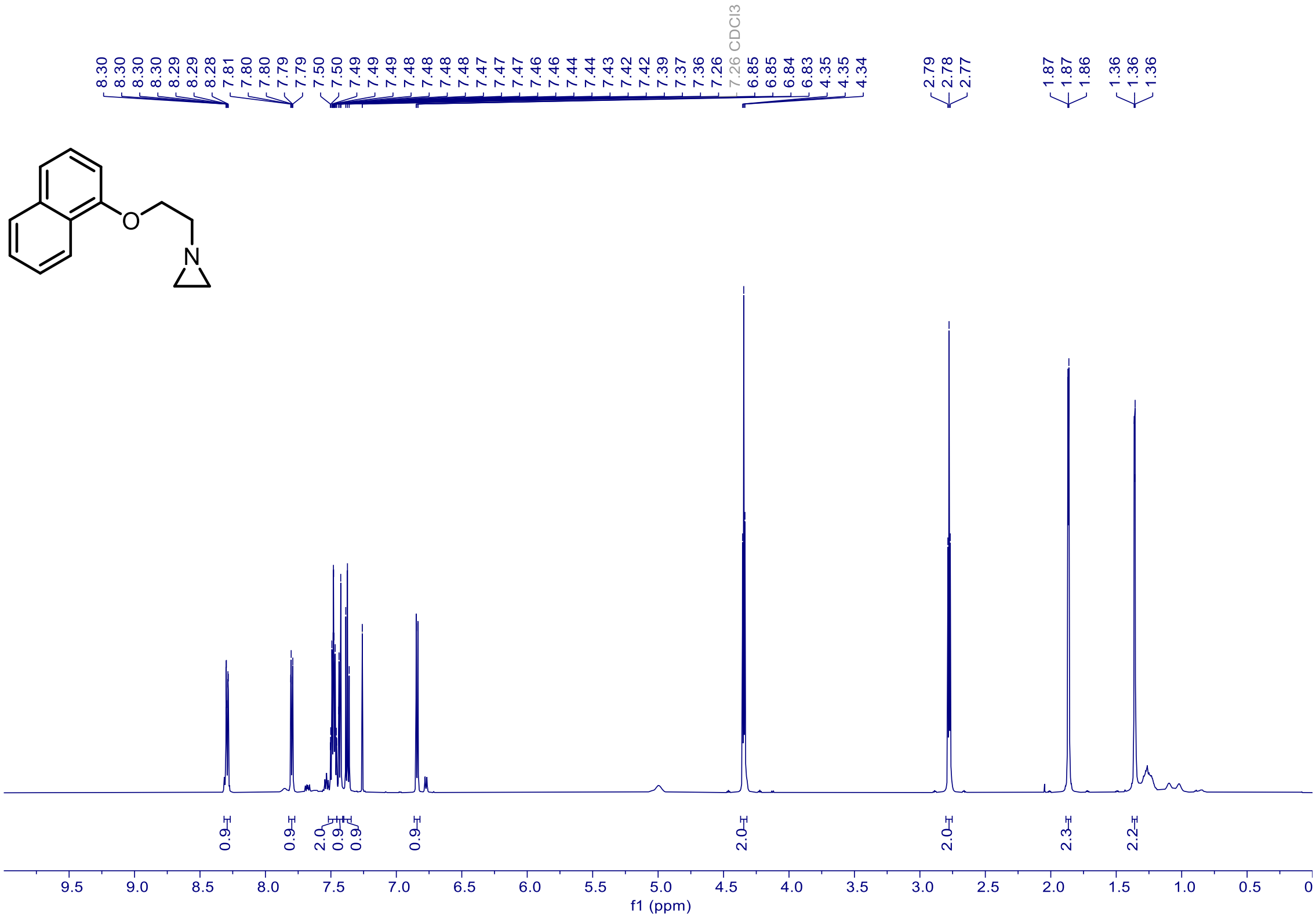

36 – ^{13}C NMR (151 MHz, $CDCl_3$)

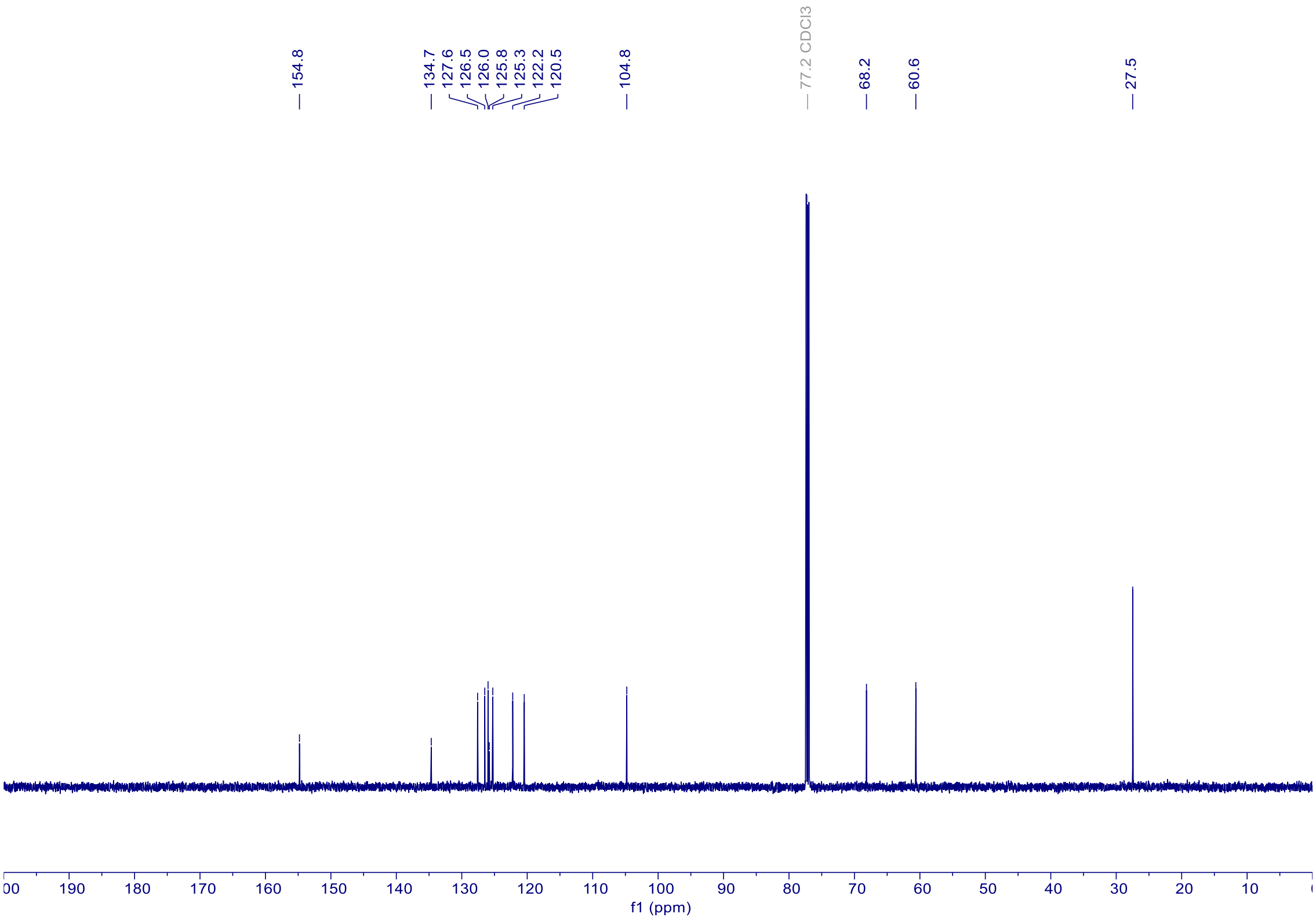

37 – ^{1}H NMR (600 MHz, $CDCl_3$)

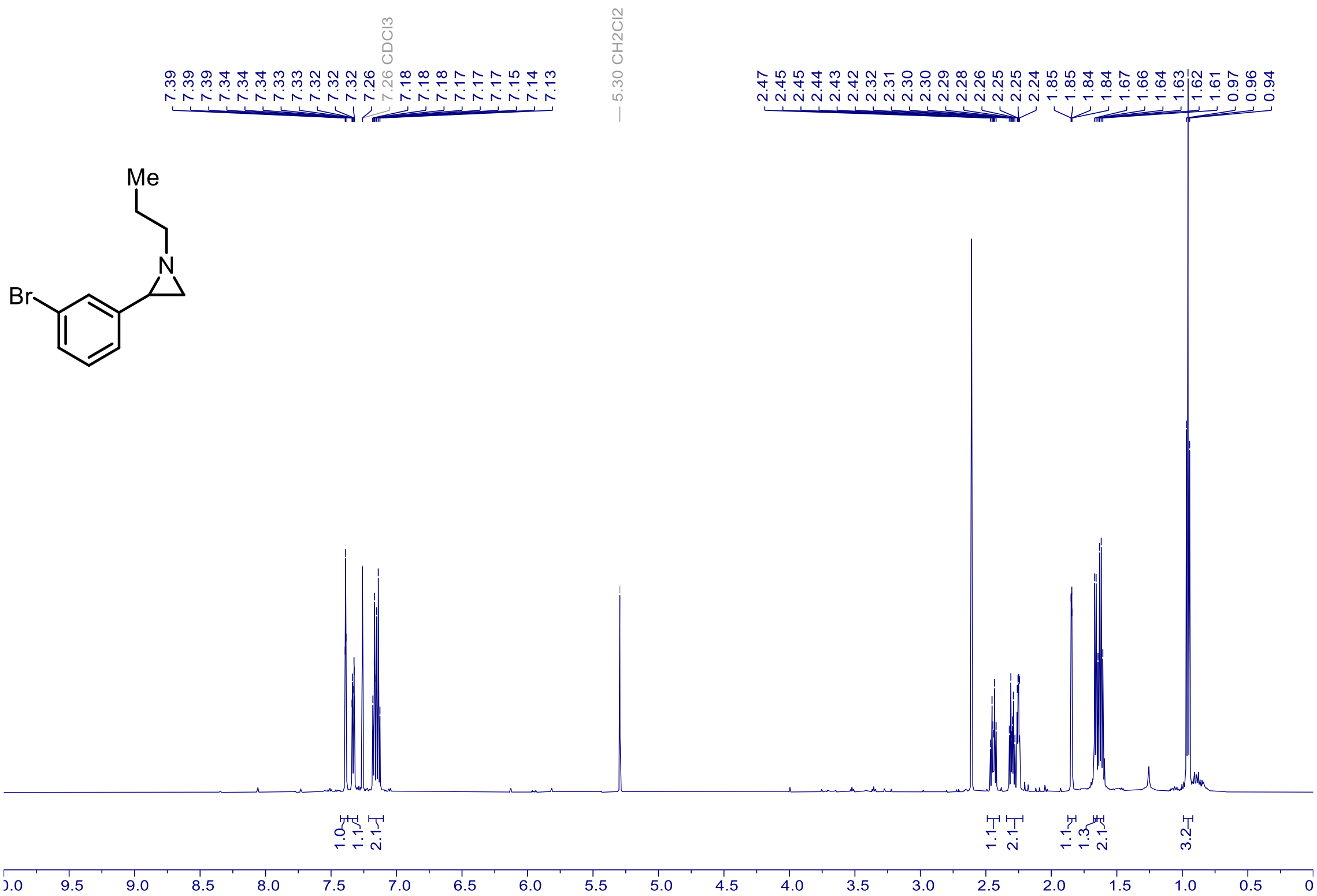

37 – ^{13}C NMR (151 MHz, $CDCl_3$)

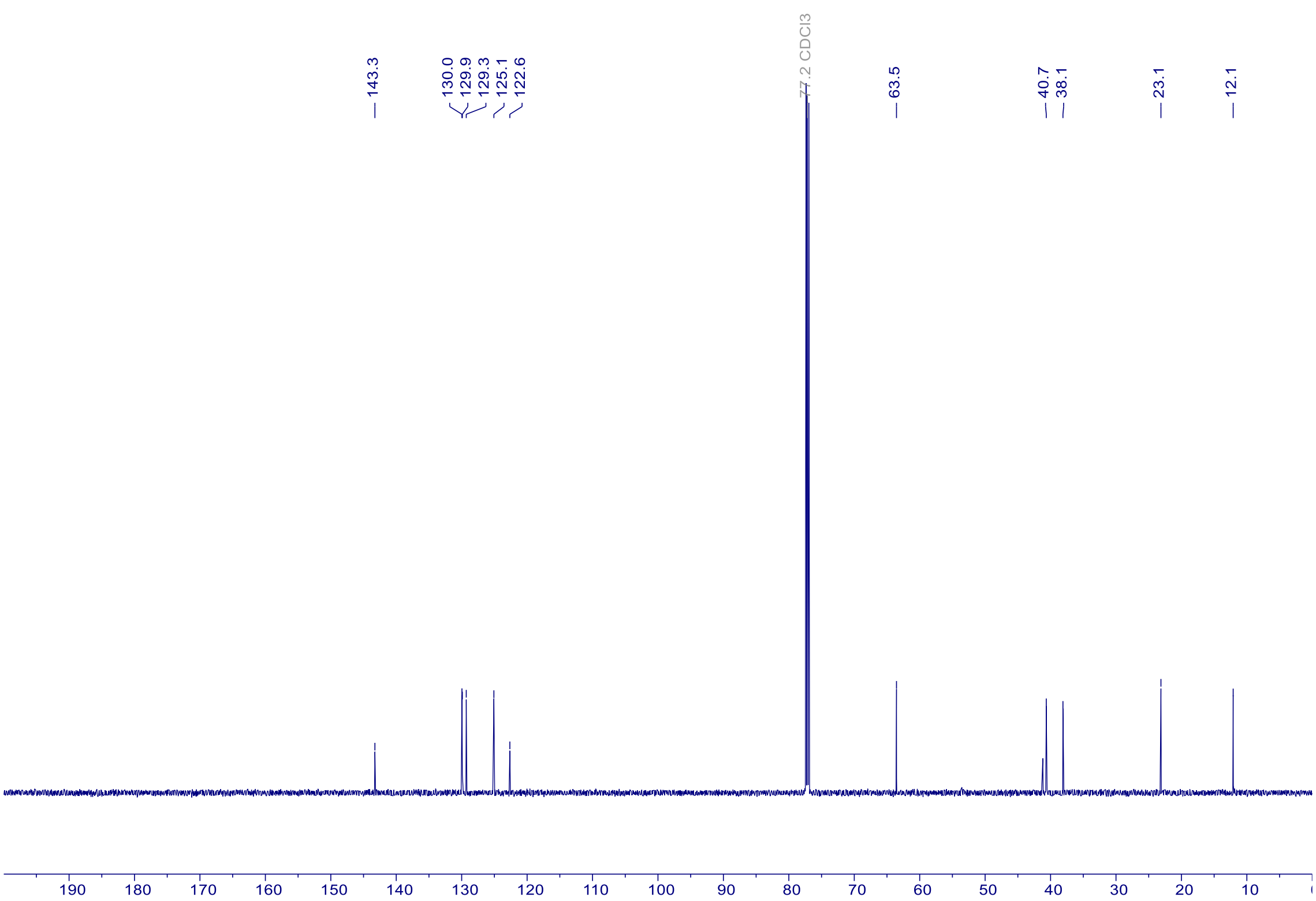

38 – ^{1}H NMR (600 MHz, $CDCl_3$)

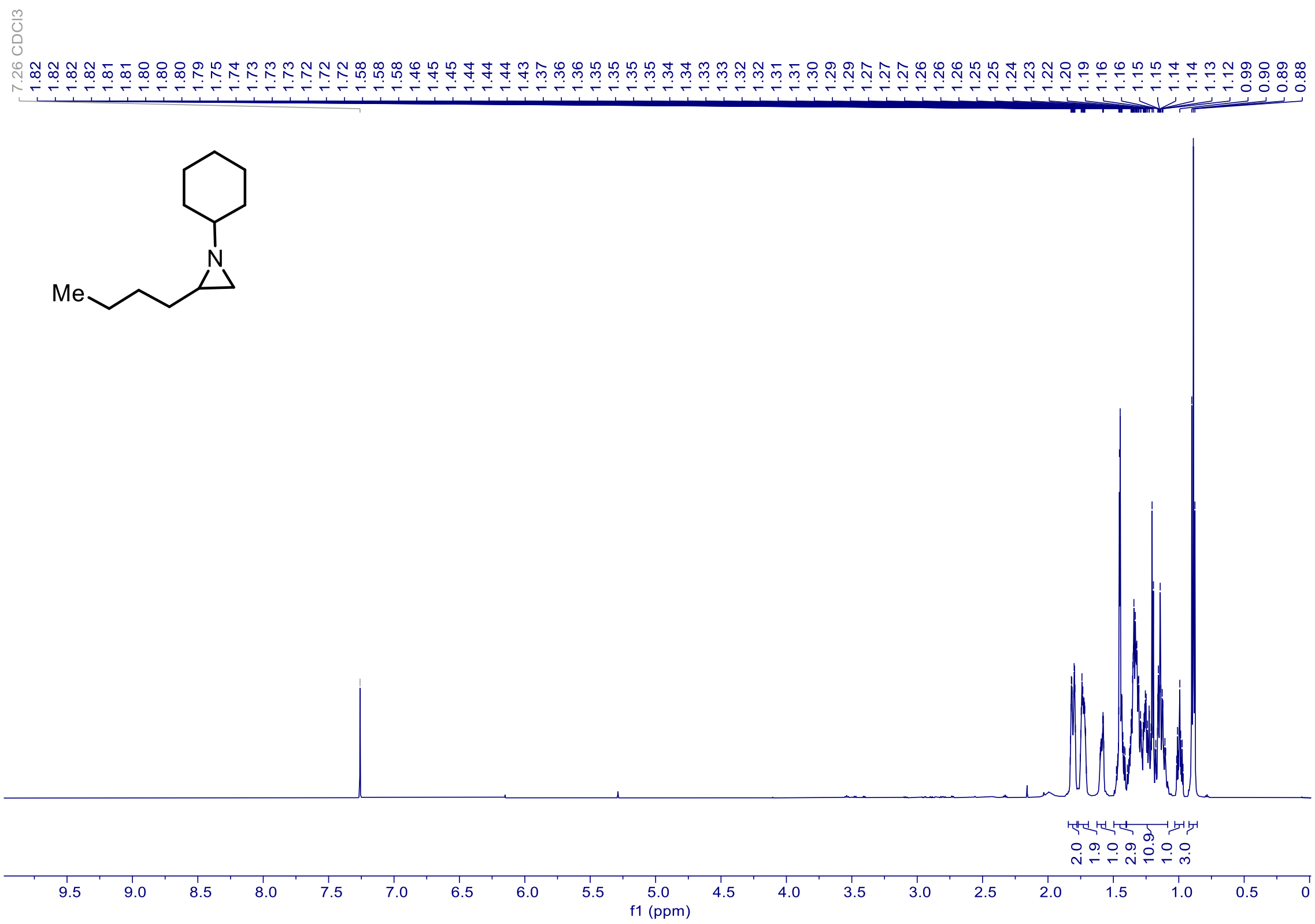

38 – ^{13}C NMR (151 MHz, $CDCl_3$)

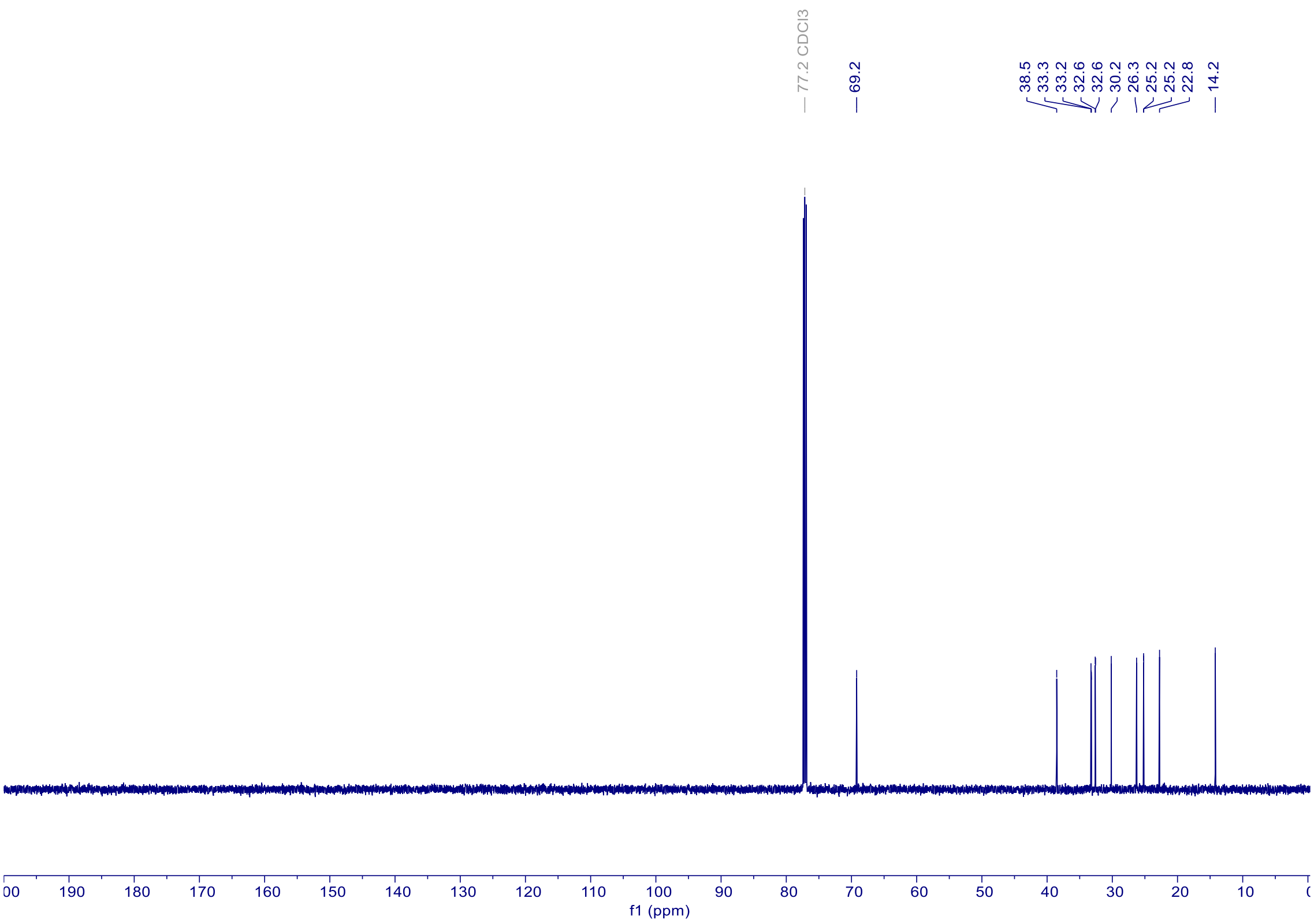

39 – ^{1}H NMR (600 MHz, $CDCl_3$)

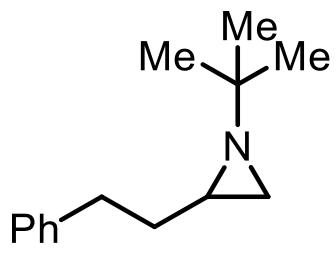

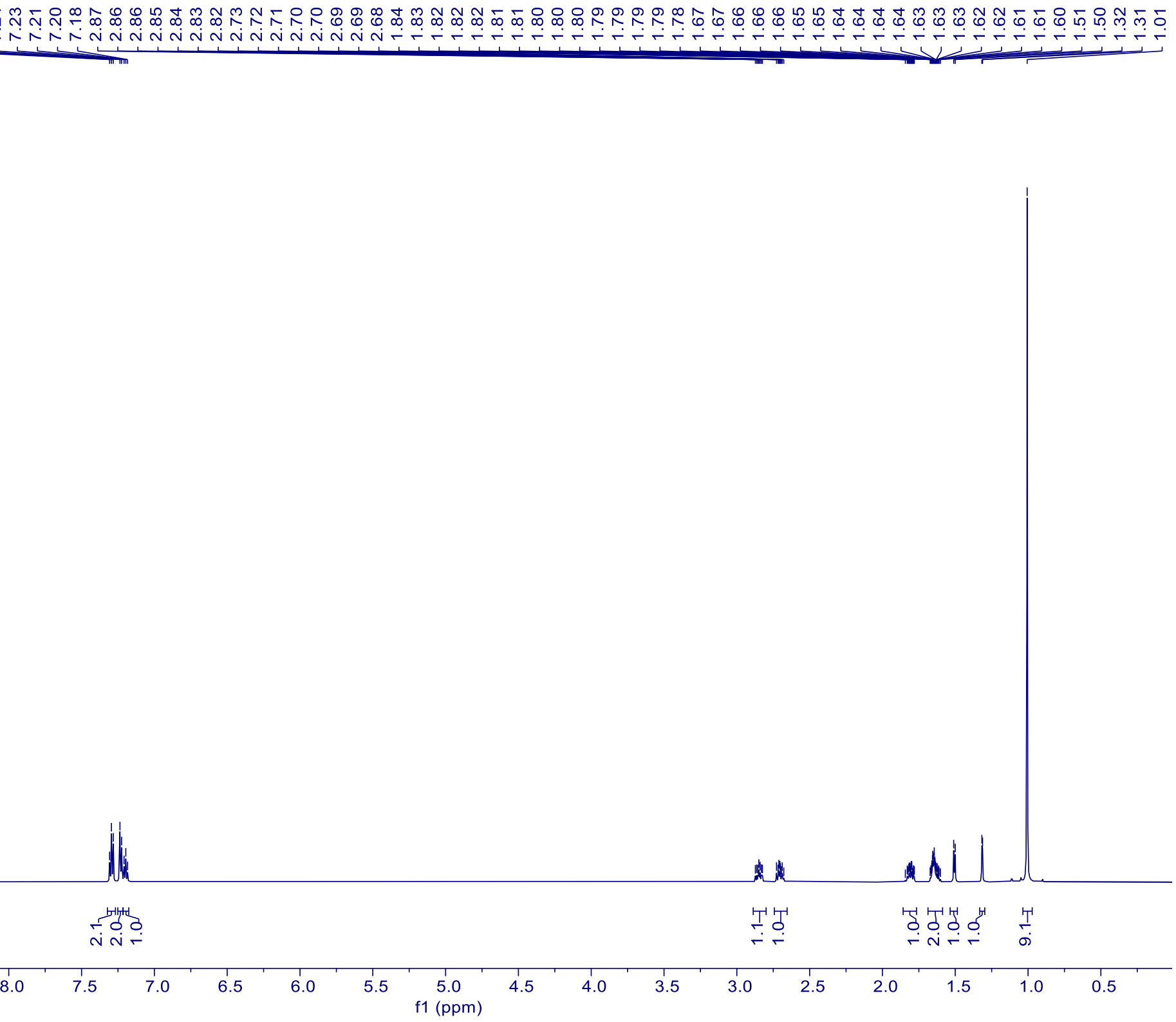

39 – ^{13}C NMR (151 MHz, $CDCl_3$)

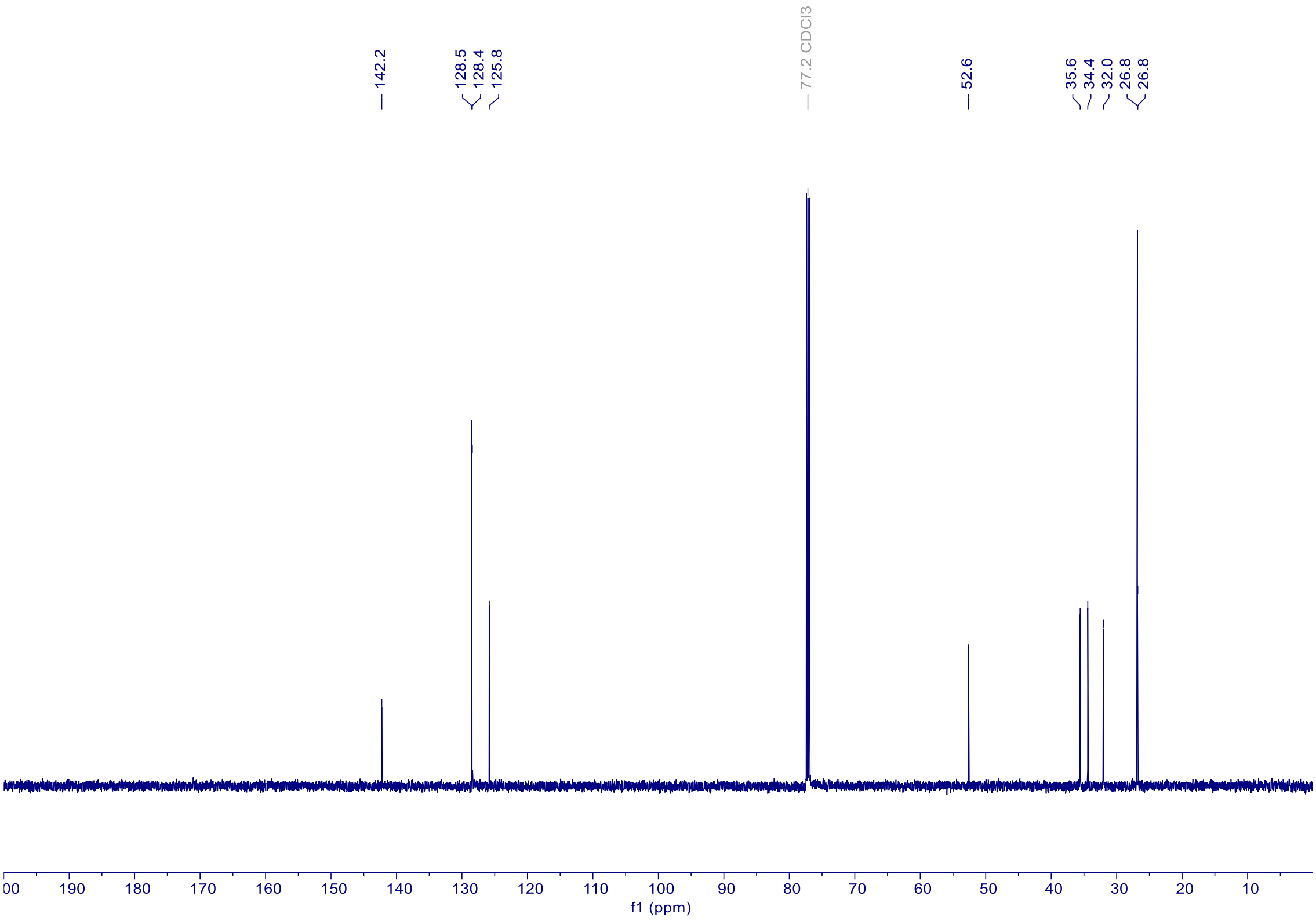

40 – ^{1}H NMR (600 MHz, $CDCl_3$)

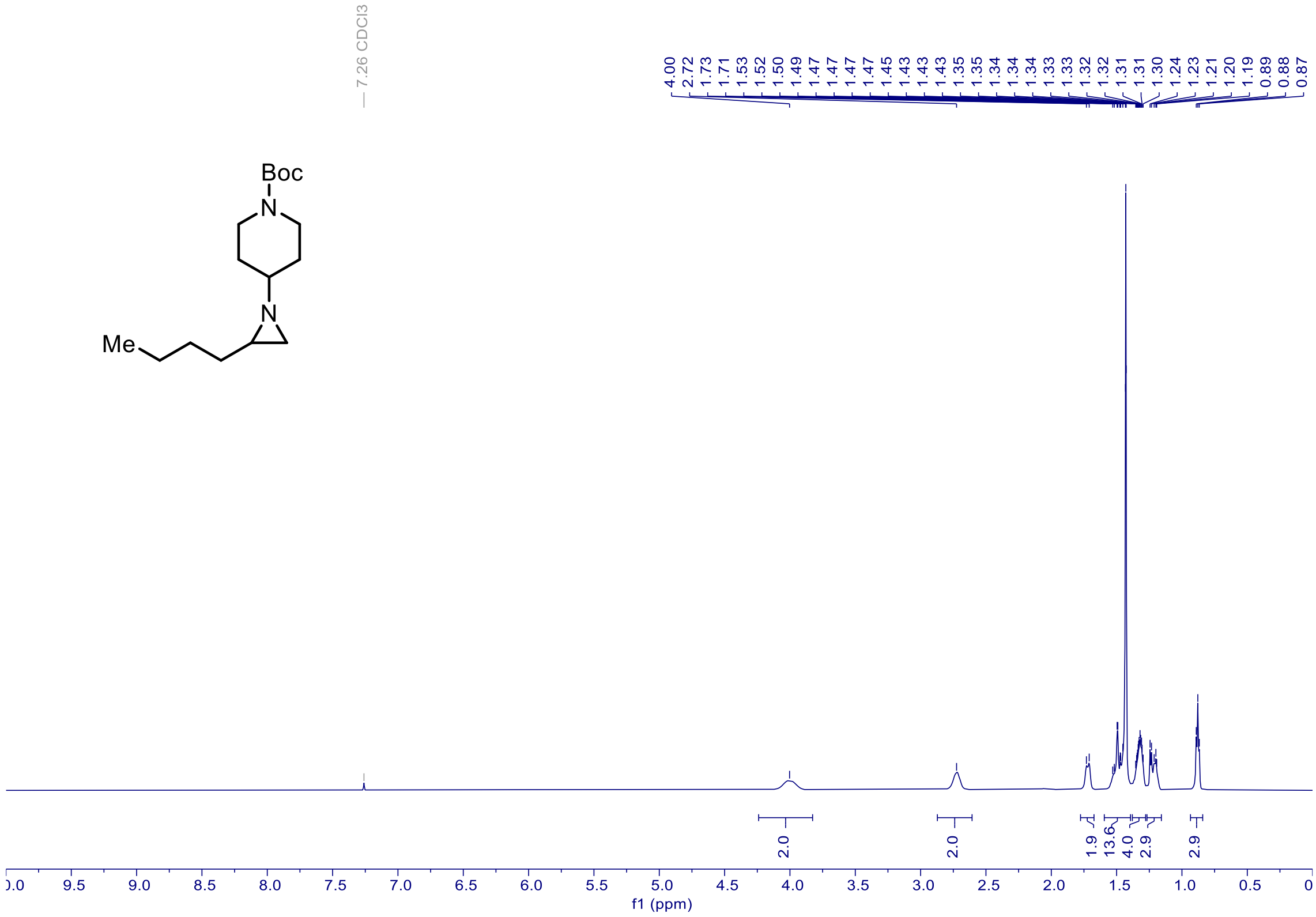

40 – ^{13}C NMR (151 MHz, $CDCl_3$)

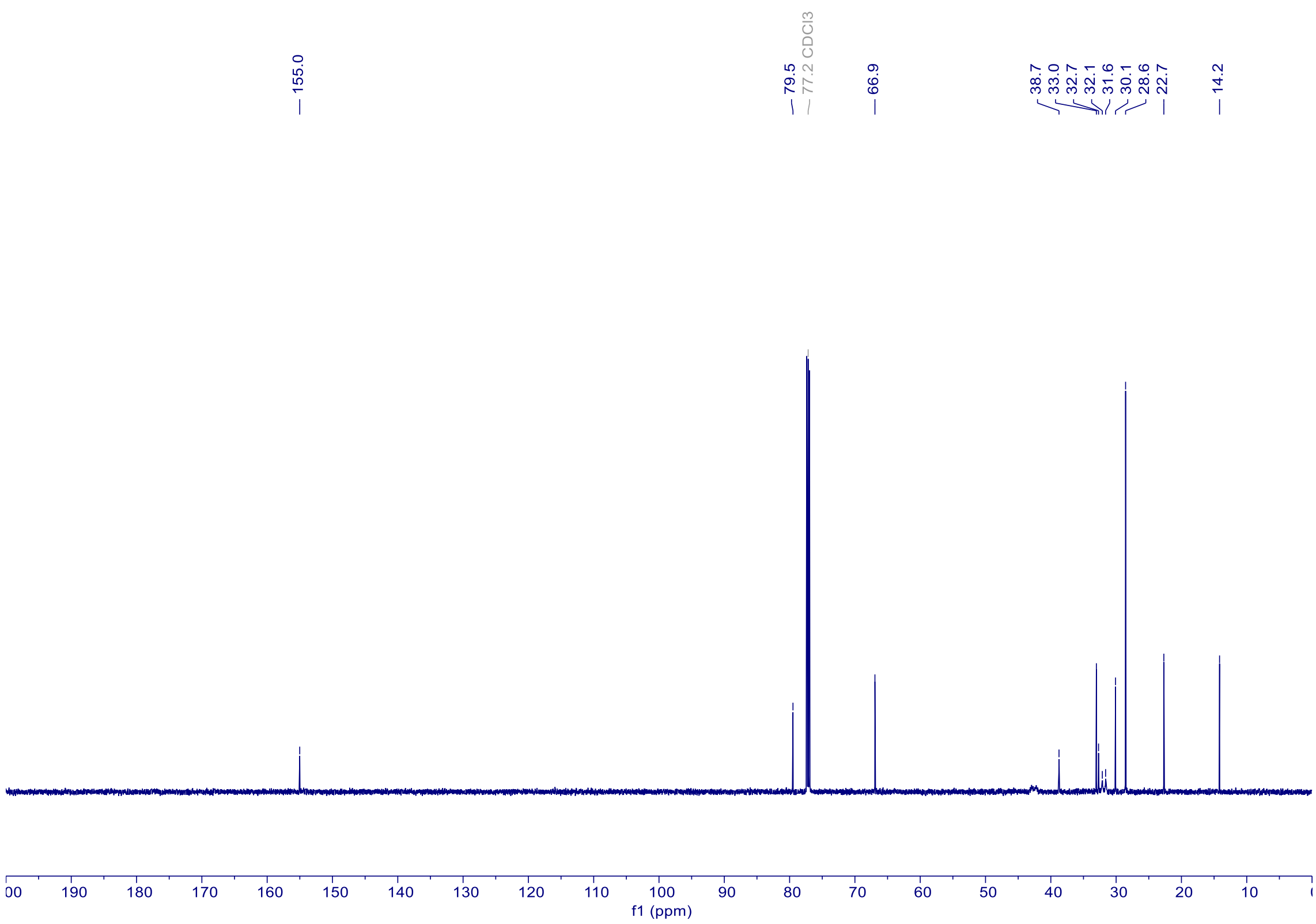

41 – ^{1}H NMR (600 MHz, $CDCl_3$)

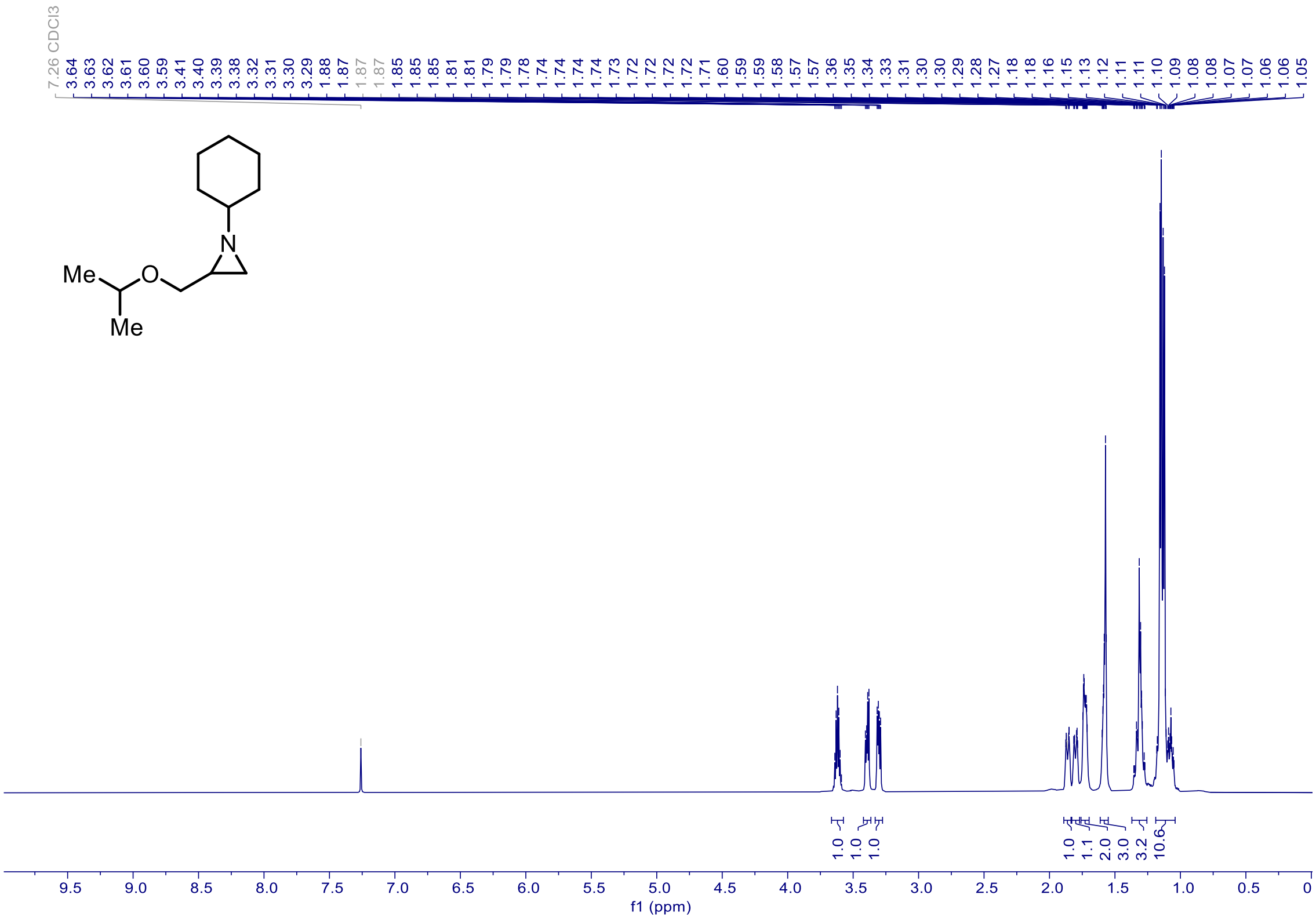

41 – ^{13}C NMR (151 MHz, $CDCl_3$)

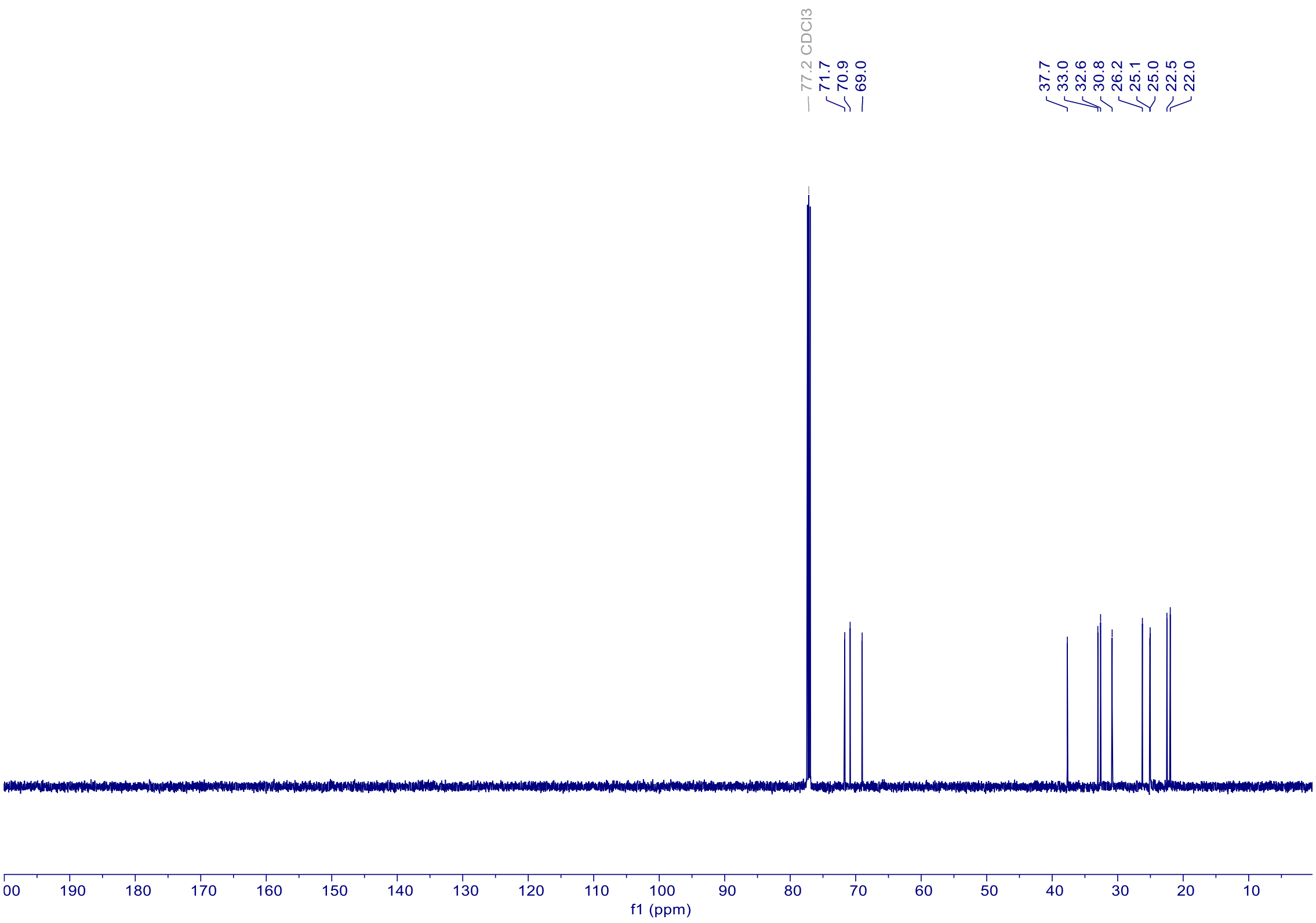

42 – ^{1}H NMR (600 MHz, $CDCl_3$)

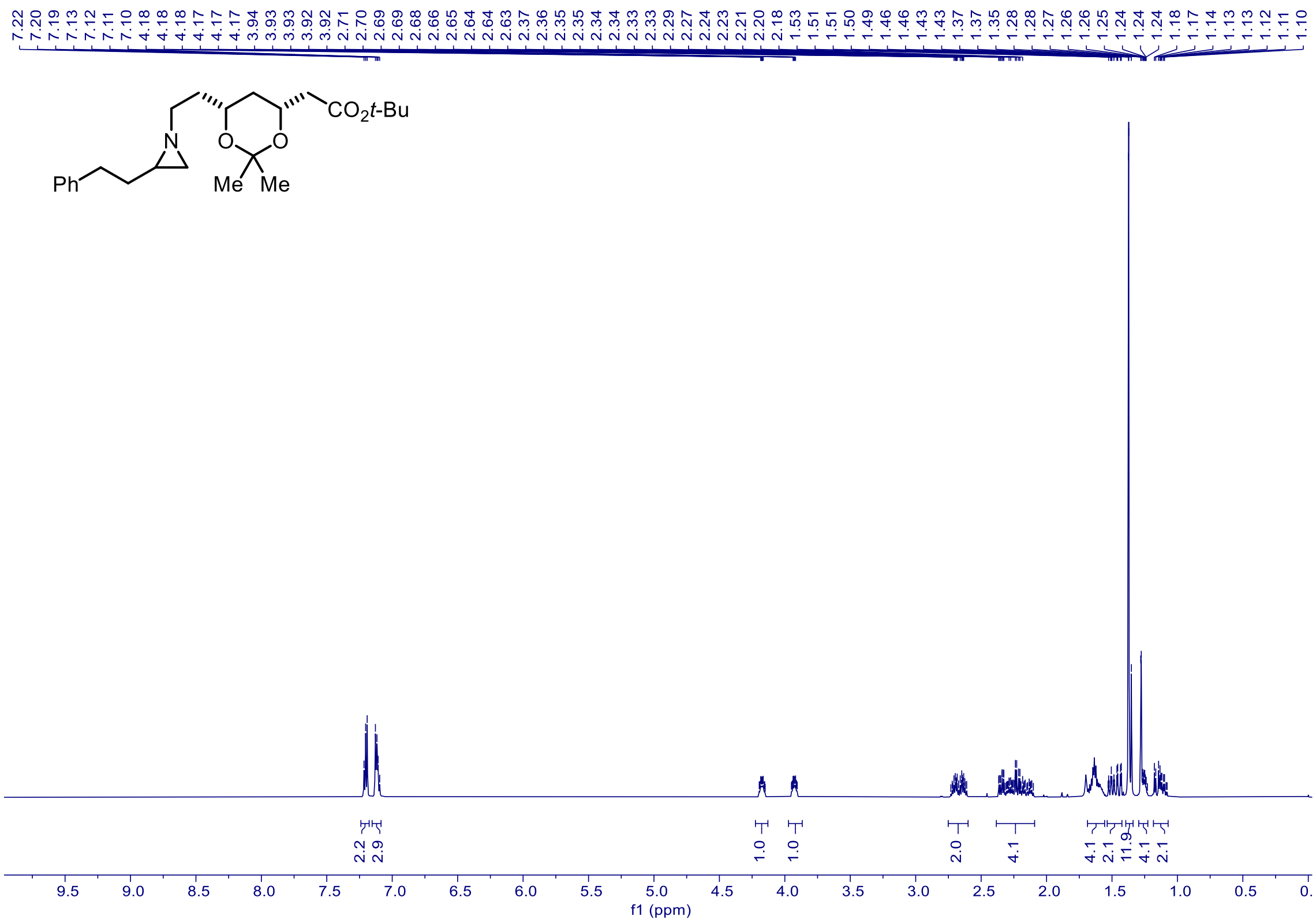

42 – ^{13}C NMR (151 MHz, $CDCl_3$)

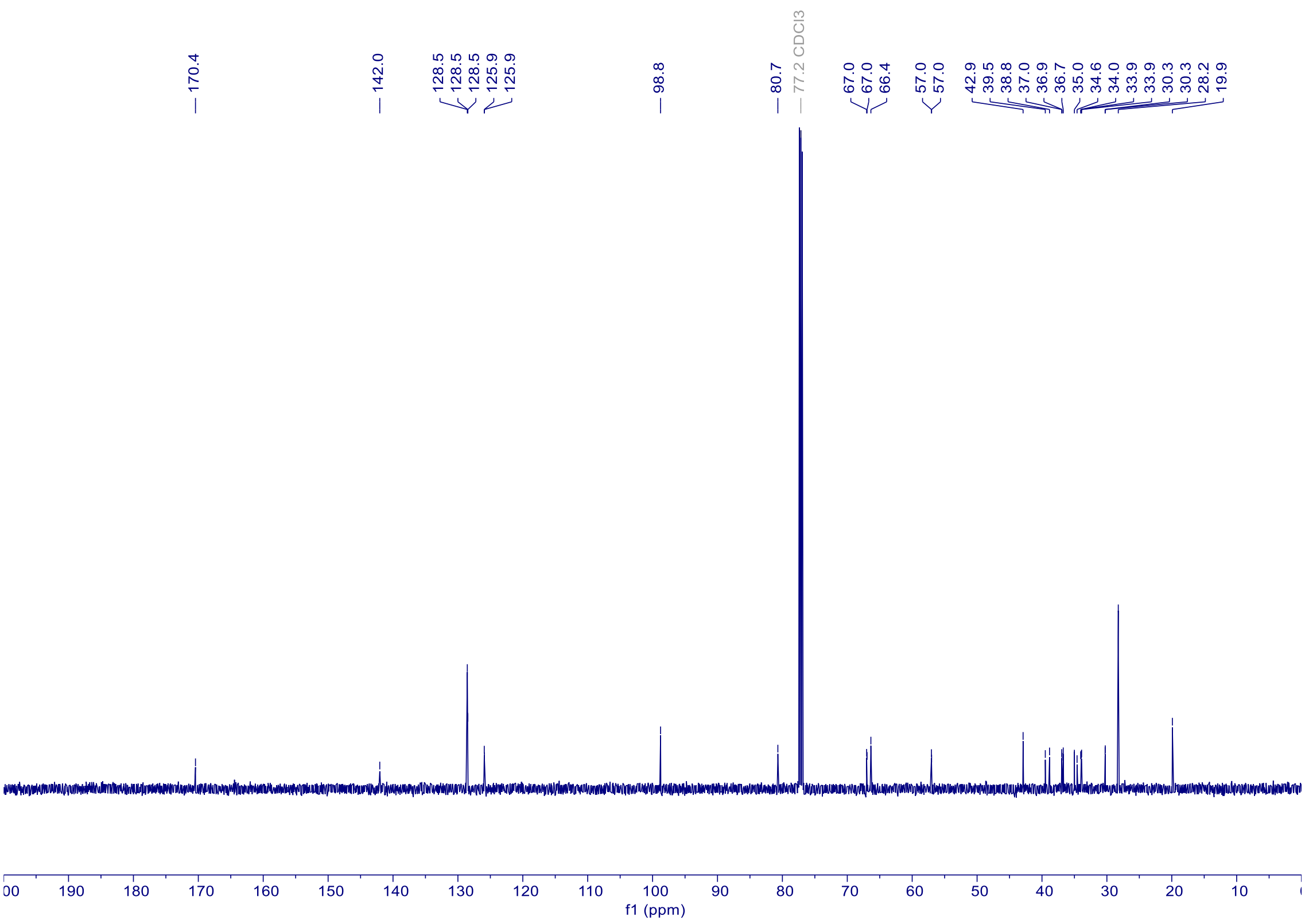

1a – ^{1}H NMR (600 MHz, $CDCl_3$)

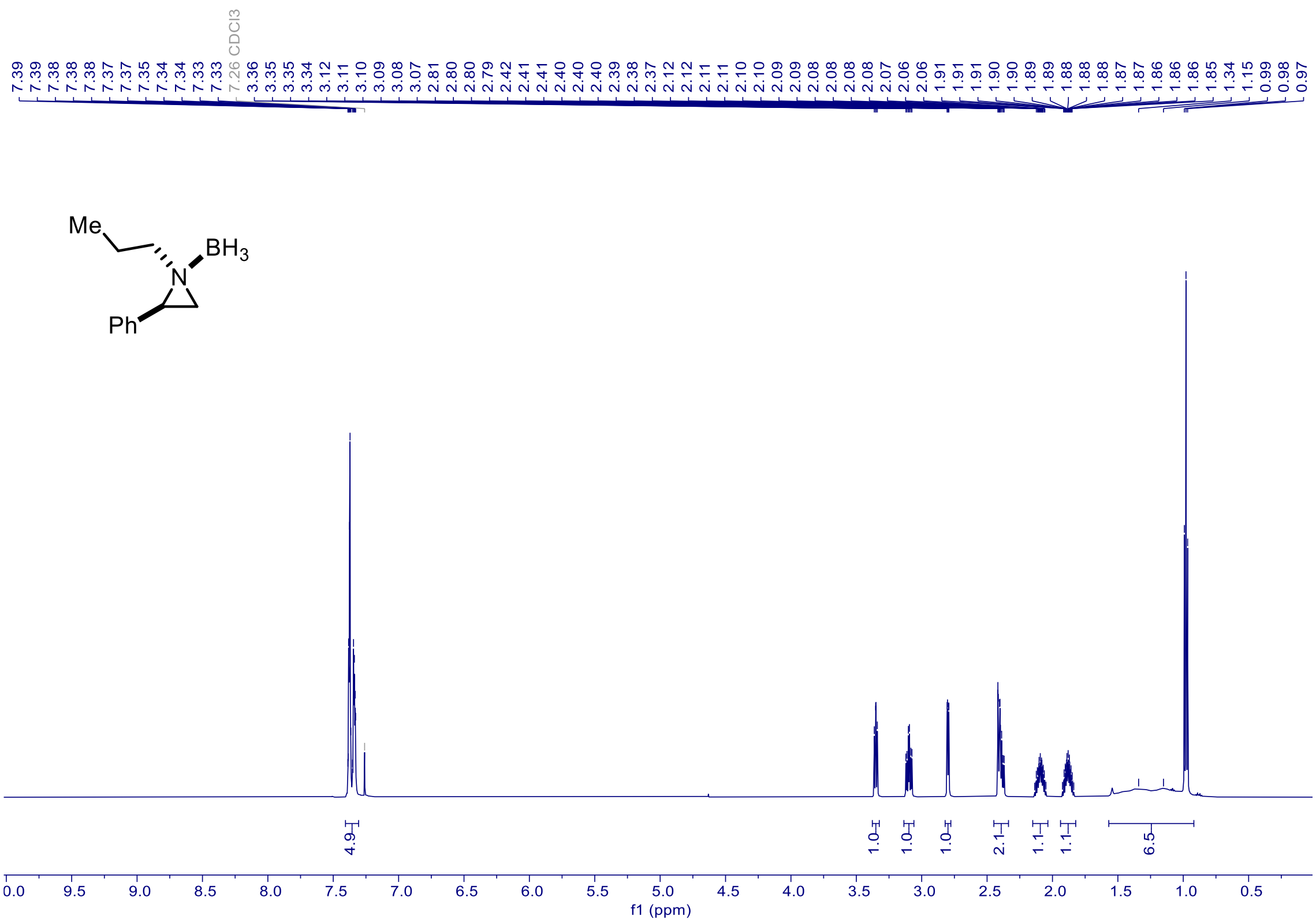

1a – ^{13}C NMR (101 MHz, DMSO-d_6)

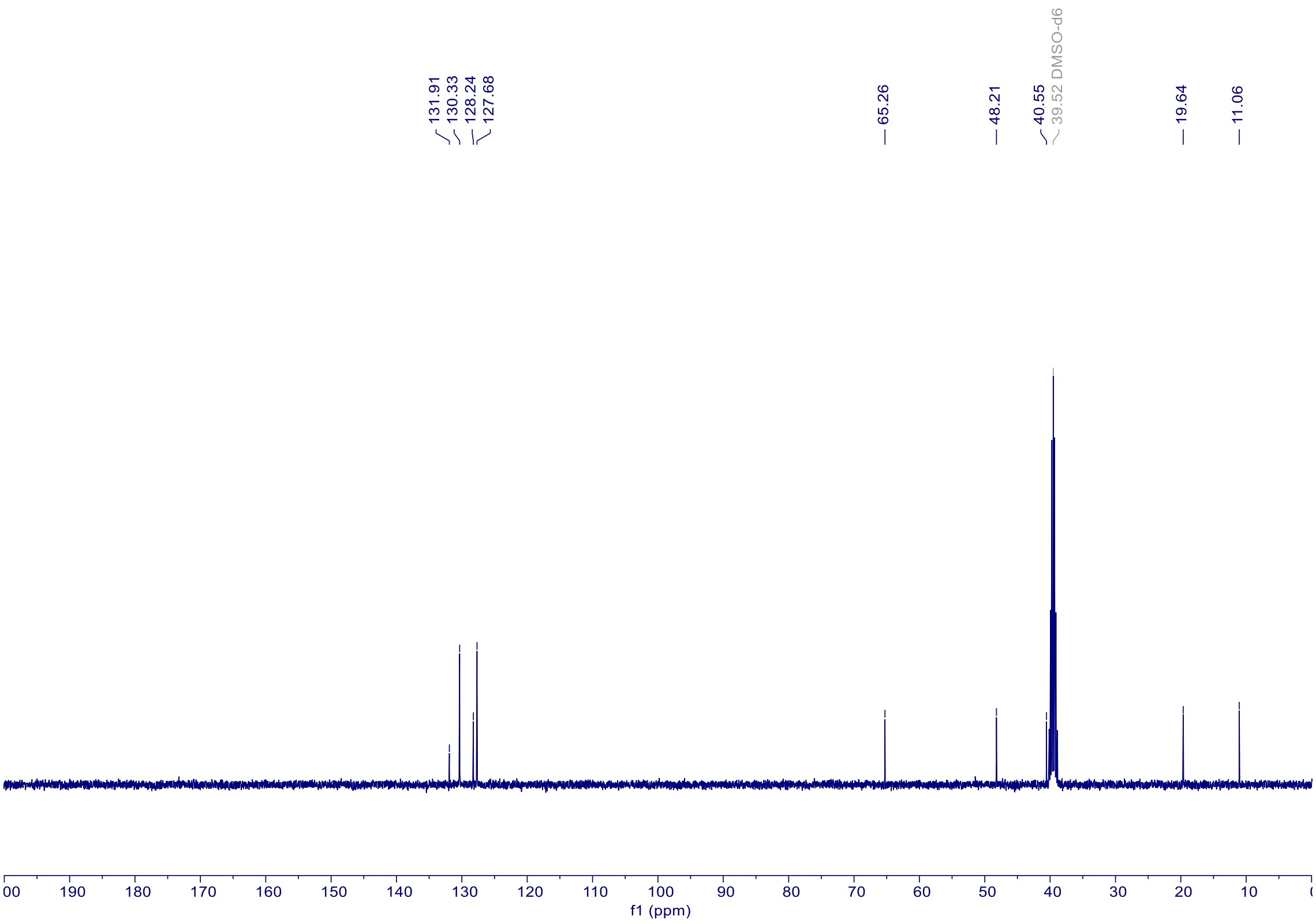

1a – ^{11}B NMR (128 MHz, DMSO-d_6)

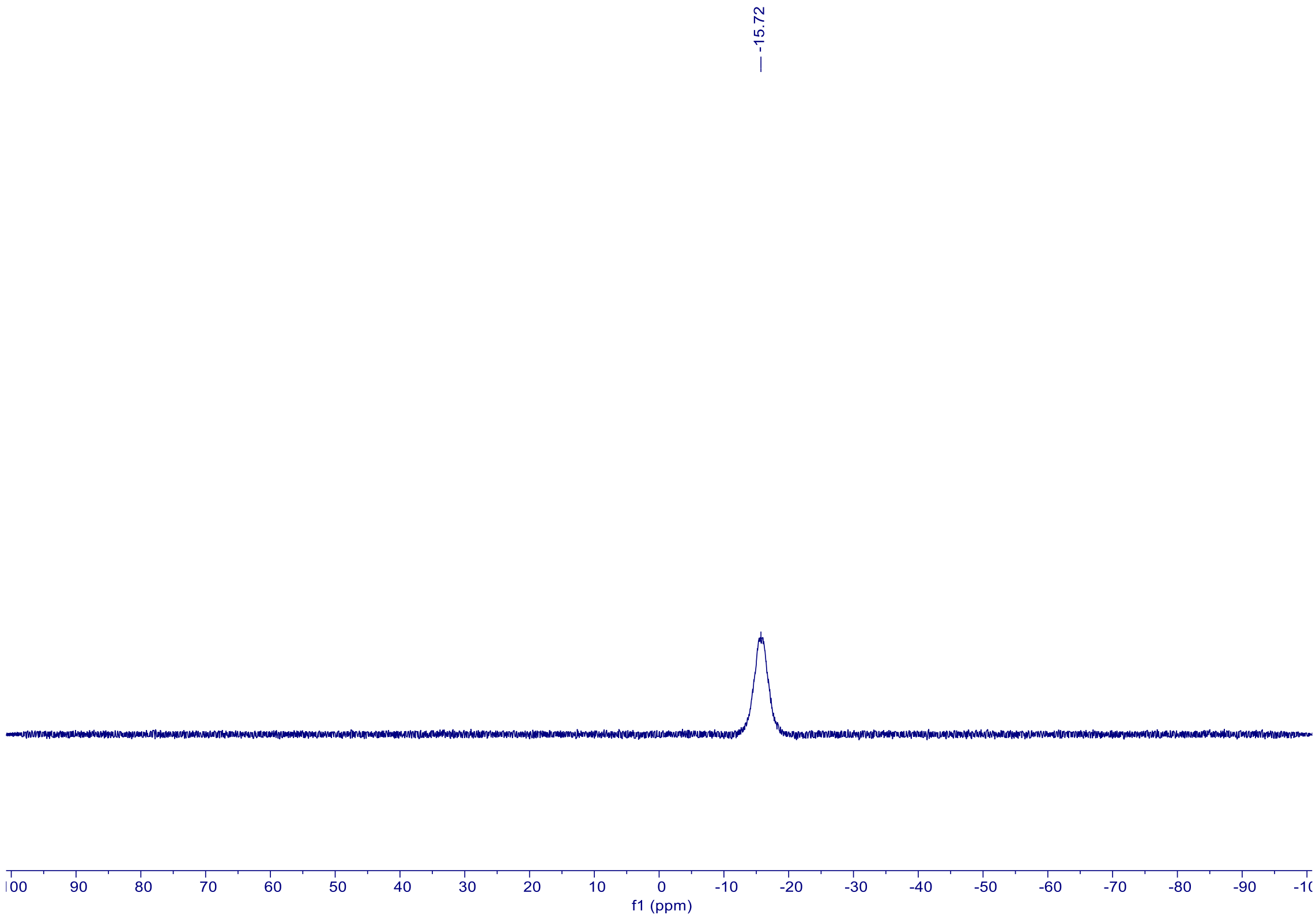

2a – ^{1}H NMR (600 MHz, $CDCl_3$)

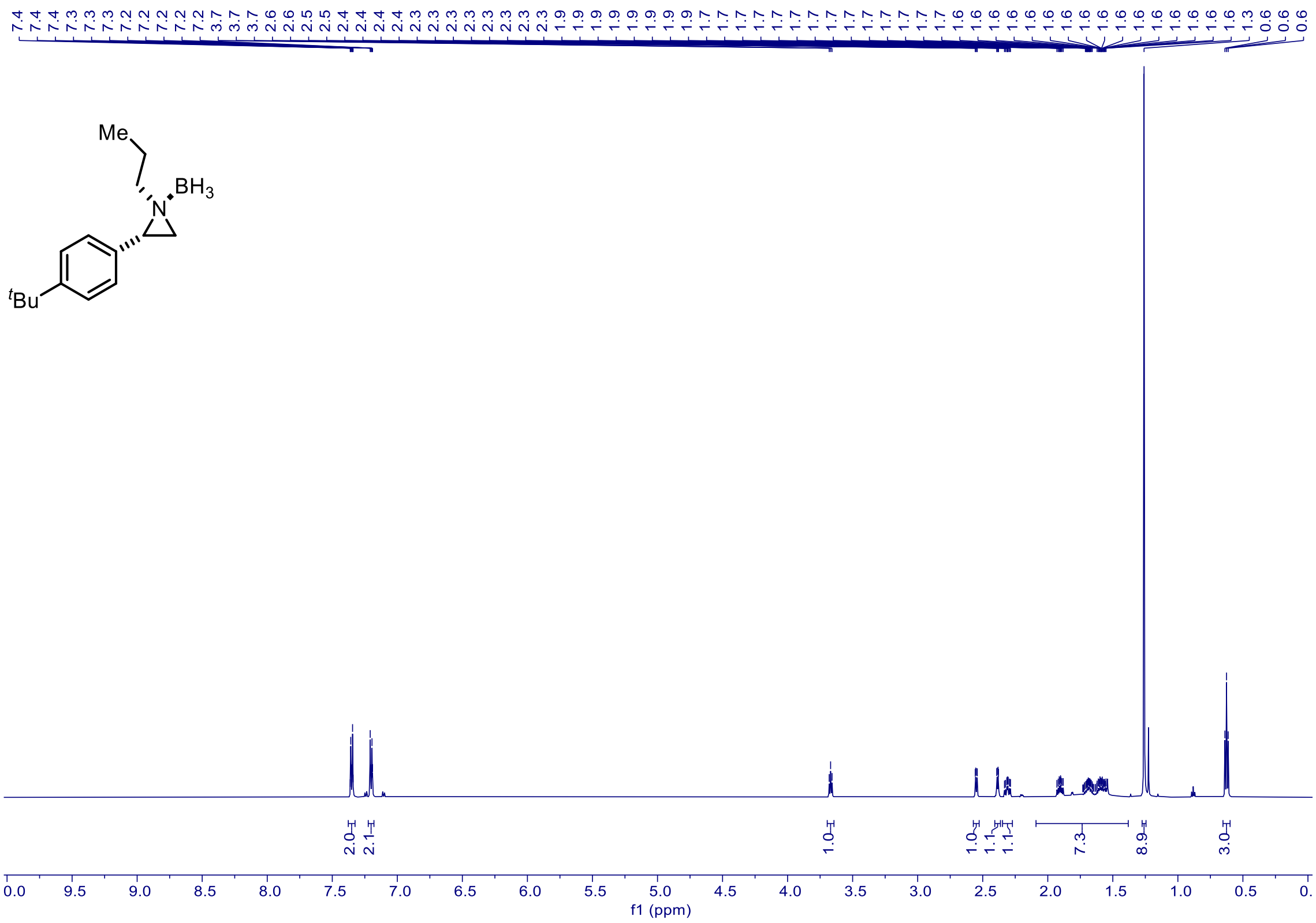

2a – ^{13}C NMR (151 MHz, $CDCl_3$)

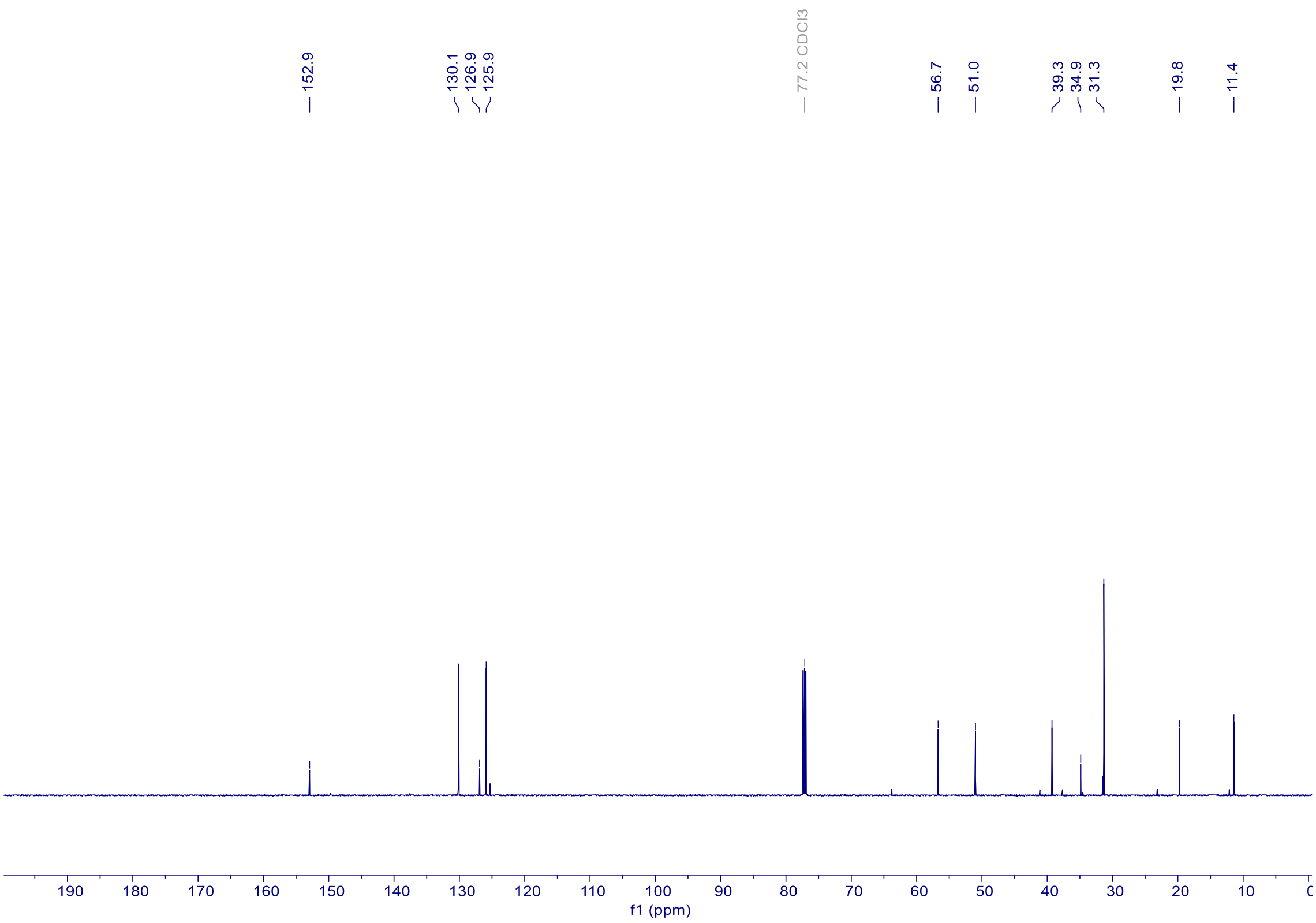

2a – ^{11}B NMR (193 MHz, $CDCl_3$)

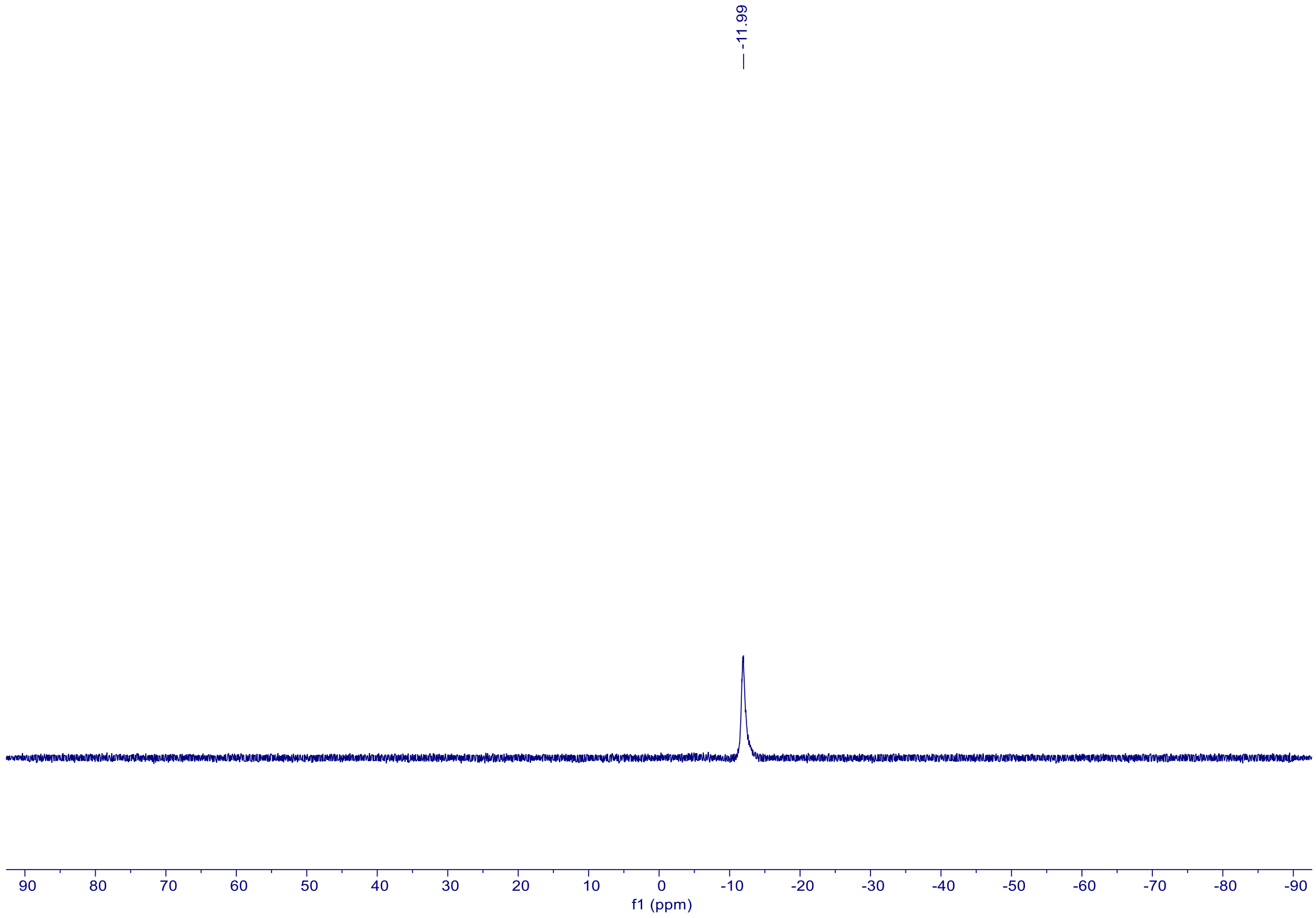

2a’ – ^{1}H NMR (600 MHz, $CDCl_3$)

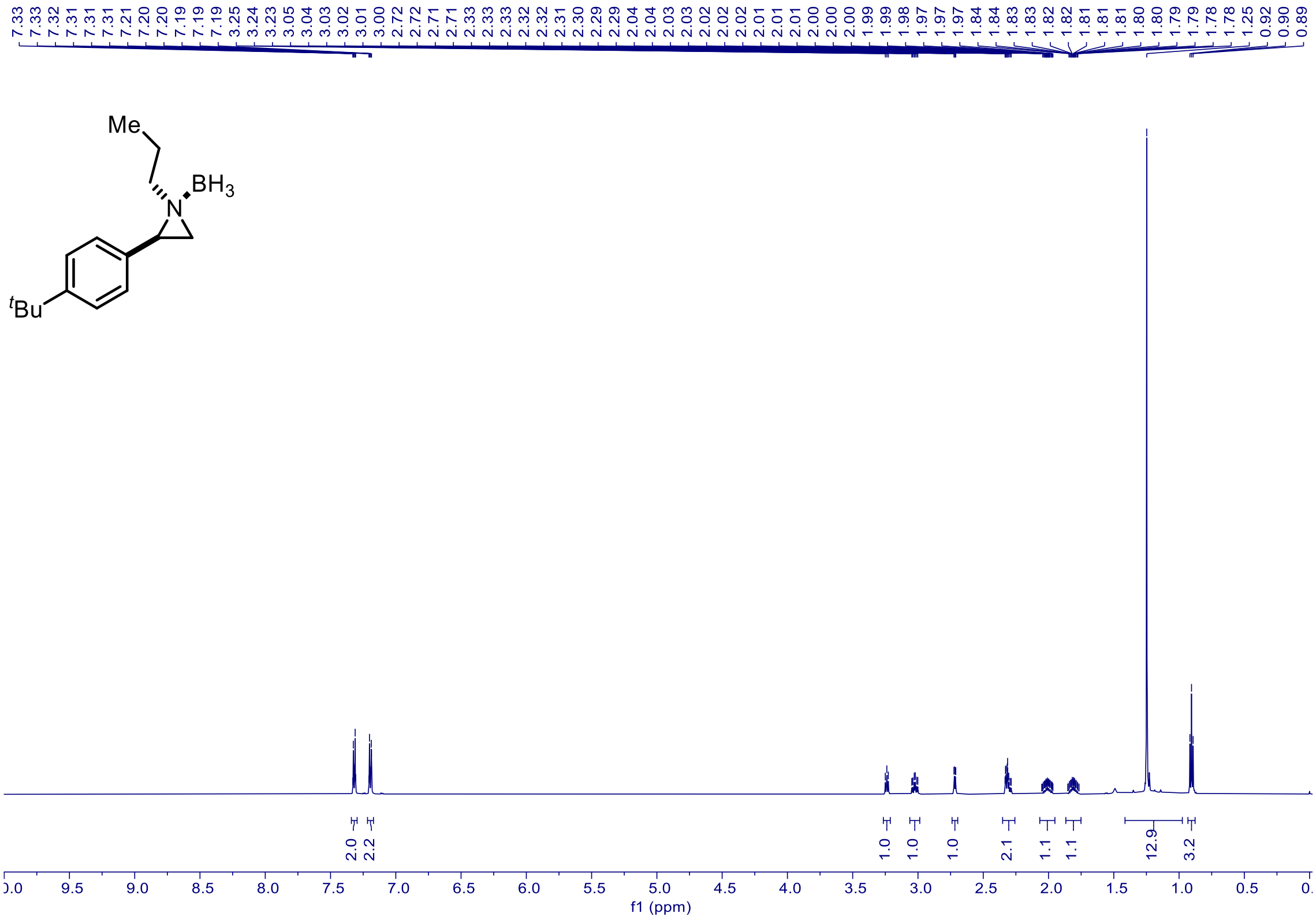

2a’ – ^{13}C NMR (151 MHz, $CDCl_3$)

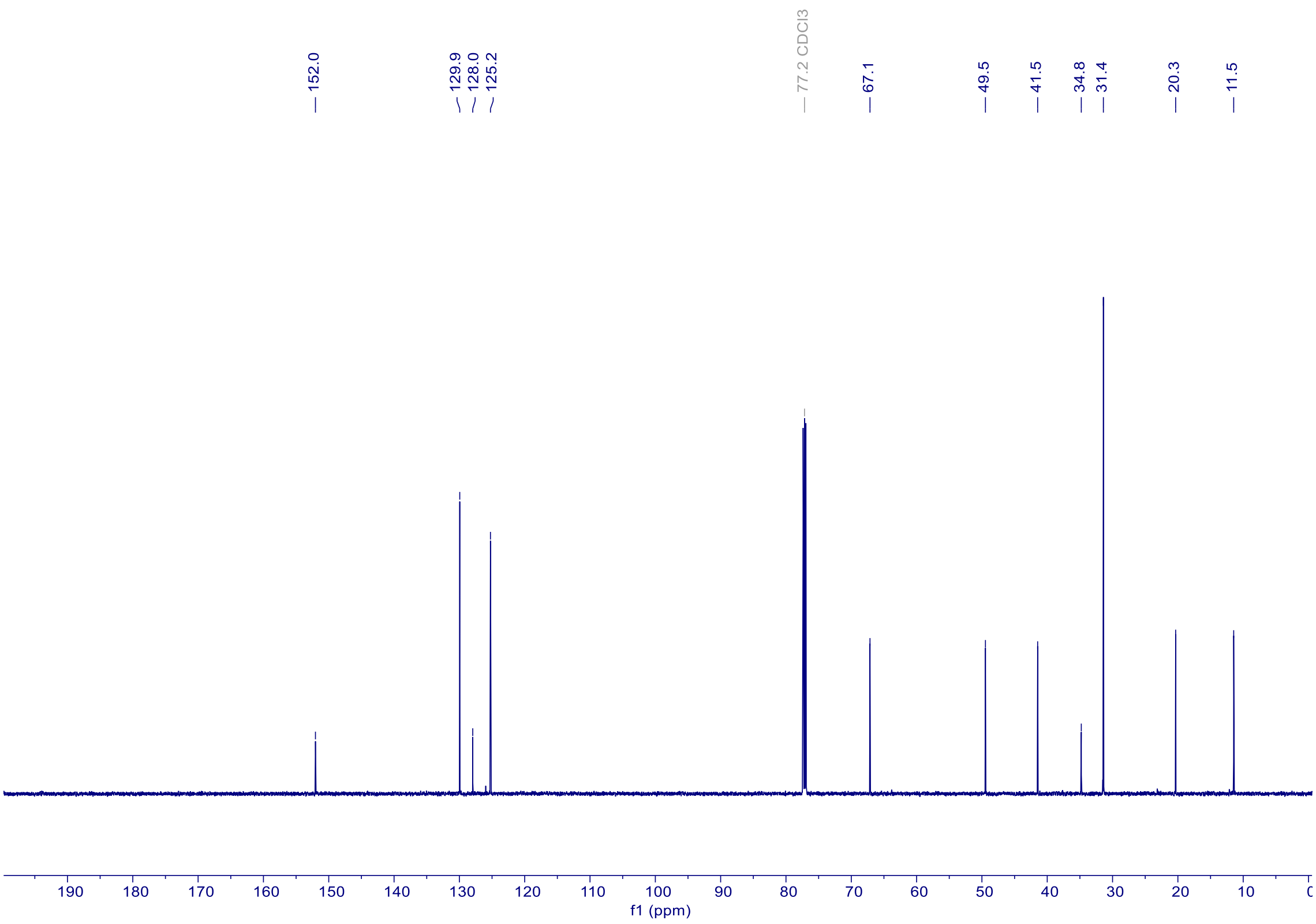

2a' – ^{11}B NMR (193 MHz, $CDCl_3$)

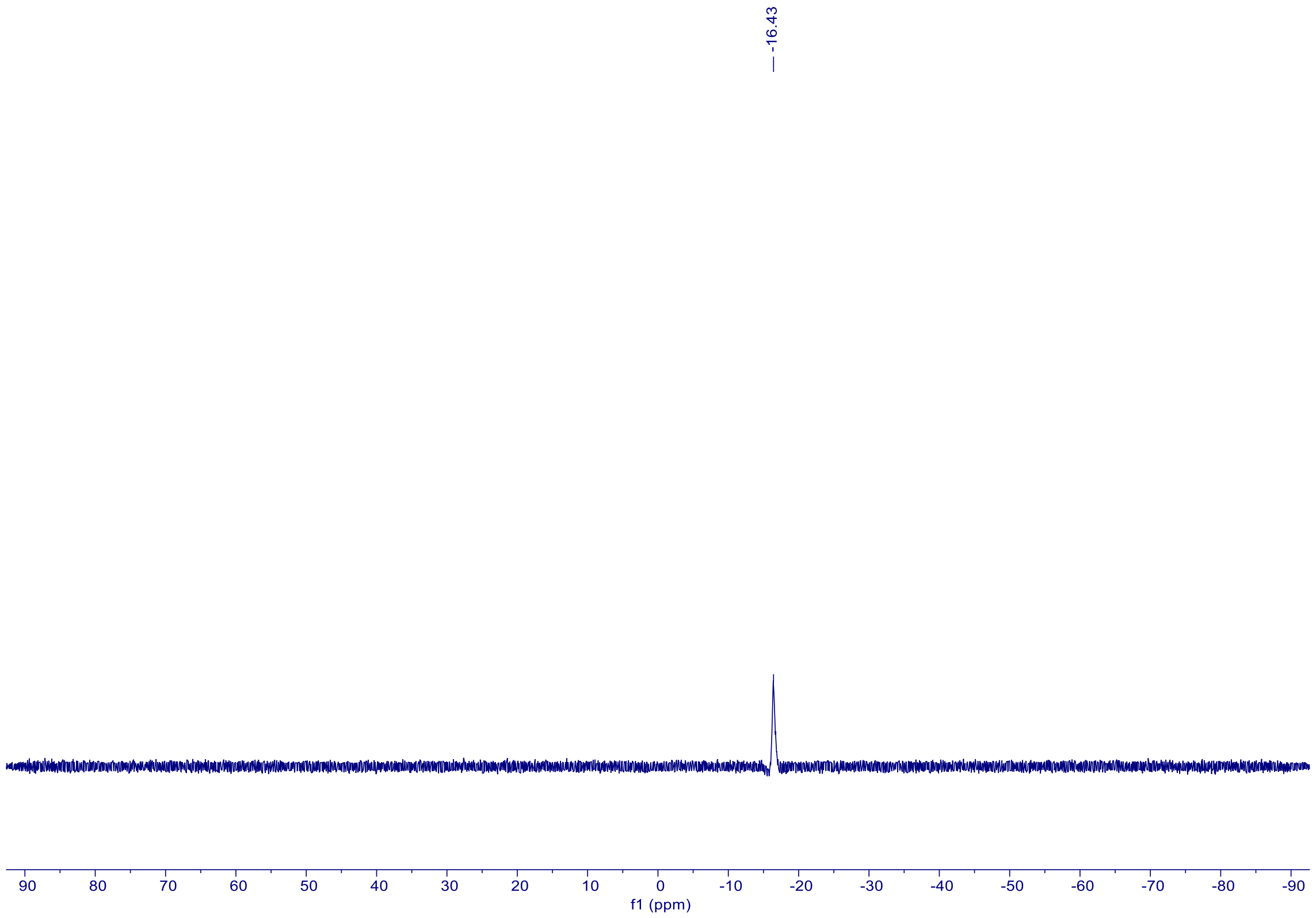

3a – ^{1}H NMR (600 MHz, $CDCl_3$)

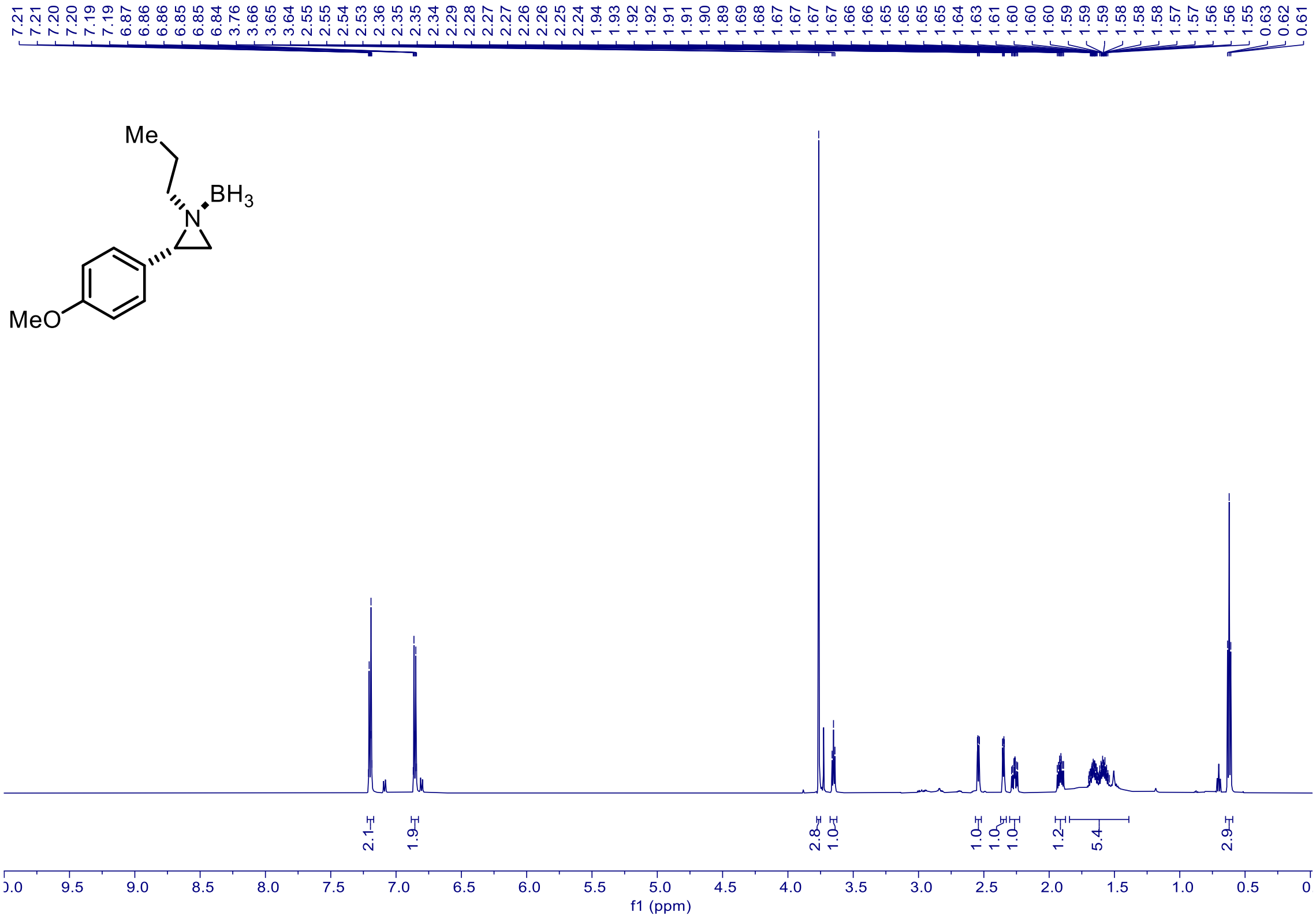

3a – ^{13}C NMR (151 MHz, $CDCl_3$)

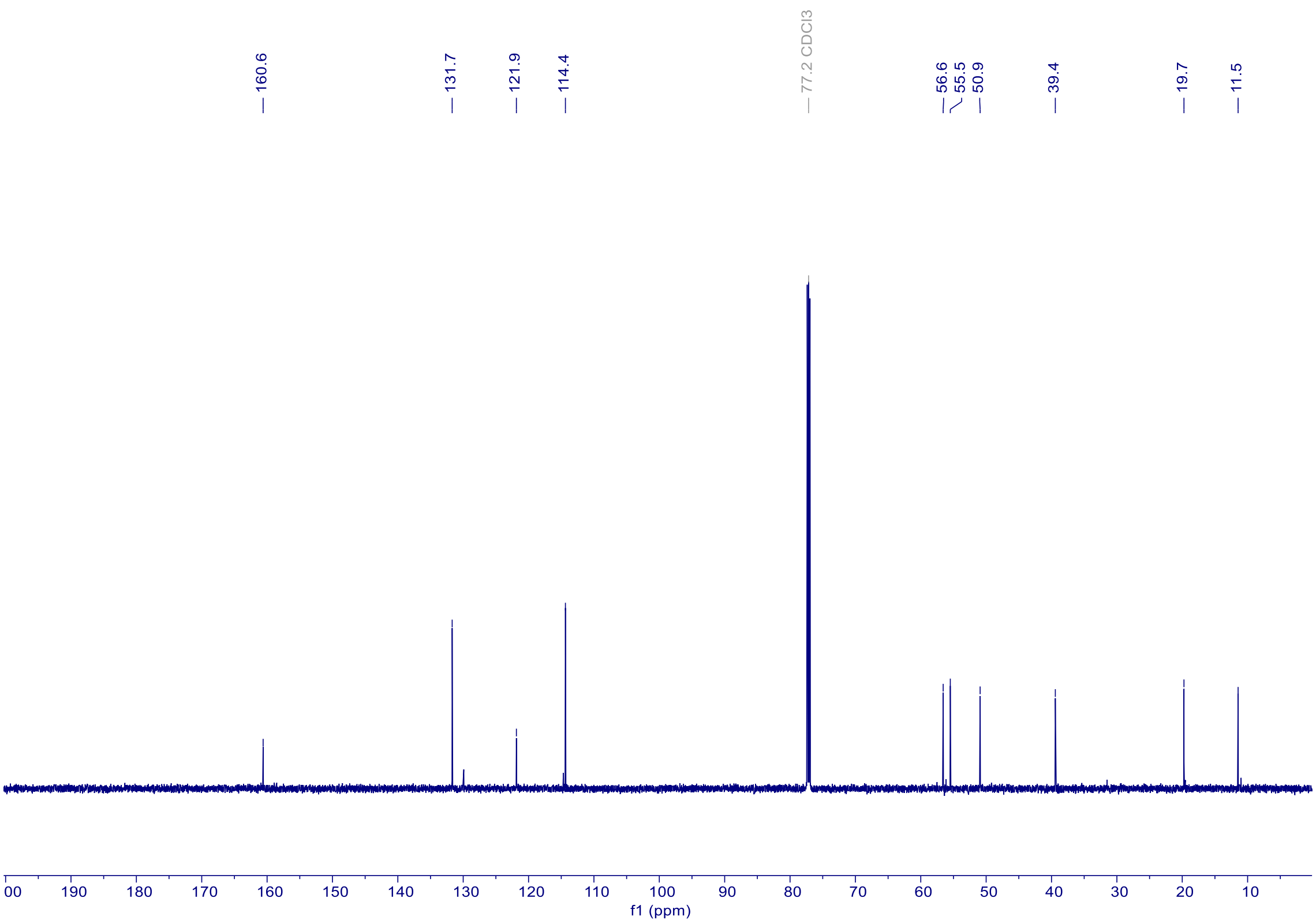

3a – ^{11}B NMR (193 MHz, $CDCl_3$)

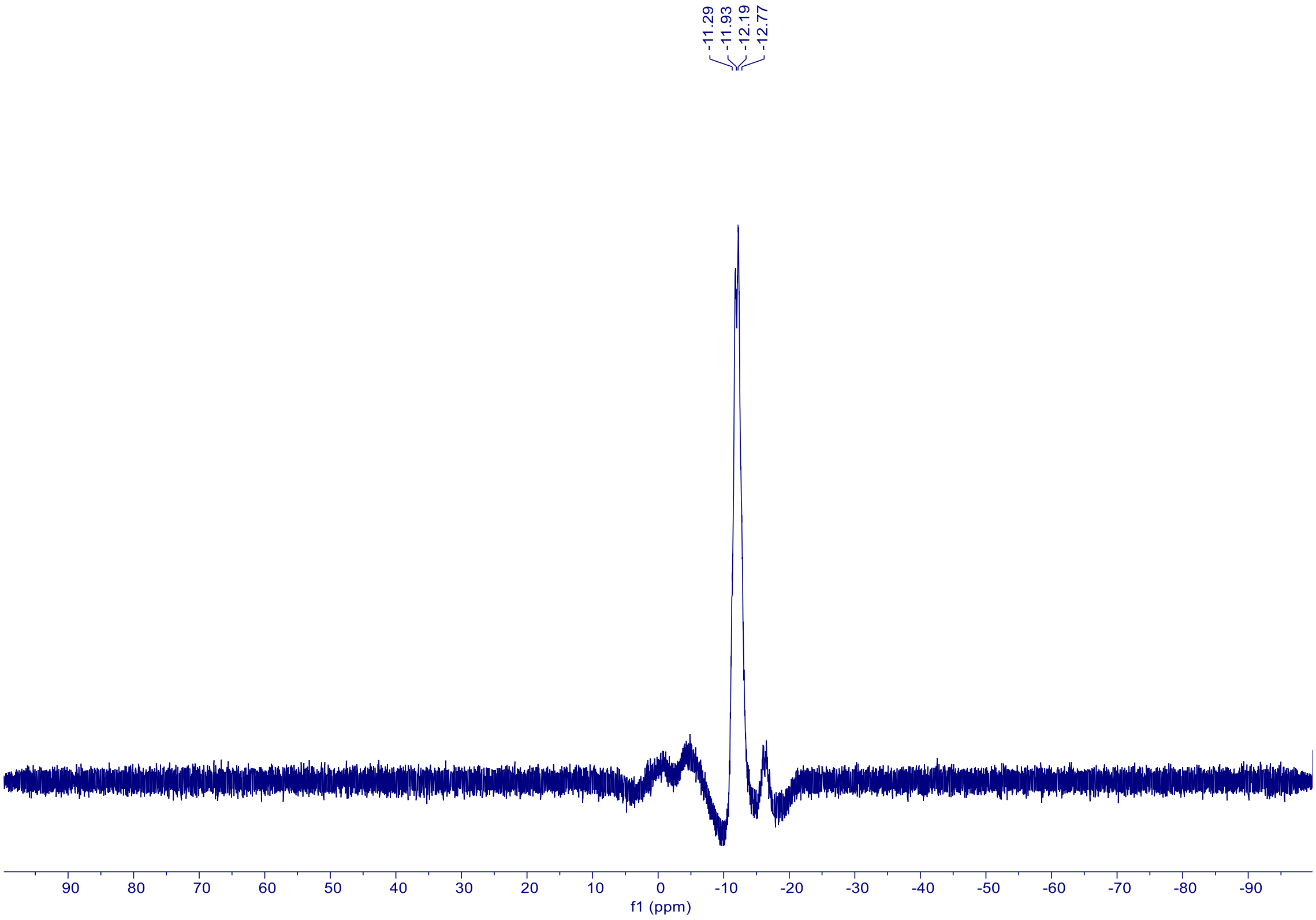

3a’ – ^{1}H NMR (600 MHz, $CDCl_3$)

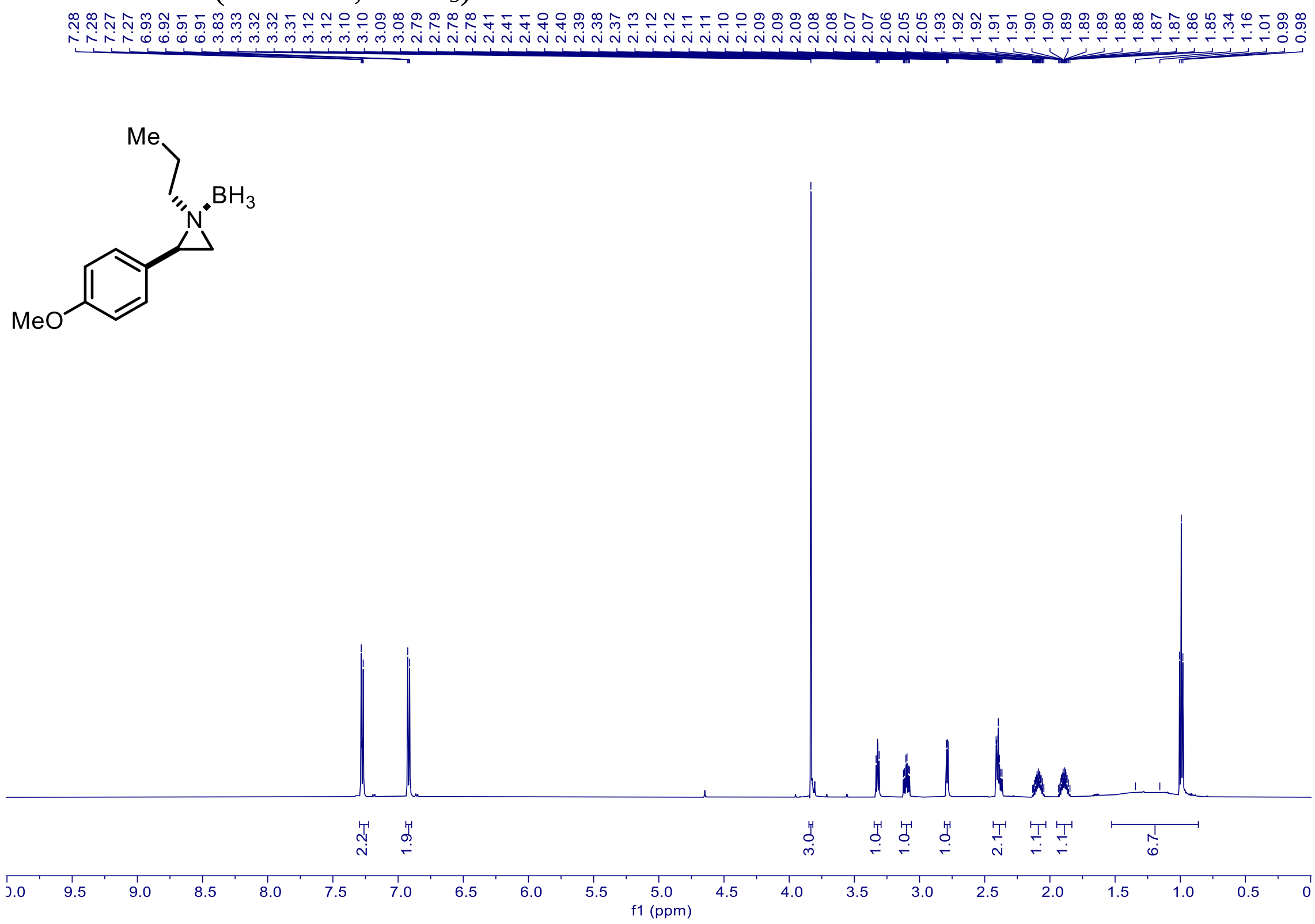

3a’ – ^{13}C NMR (151 MHz, $CDCl_3$)

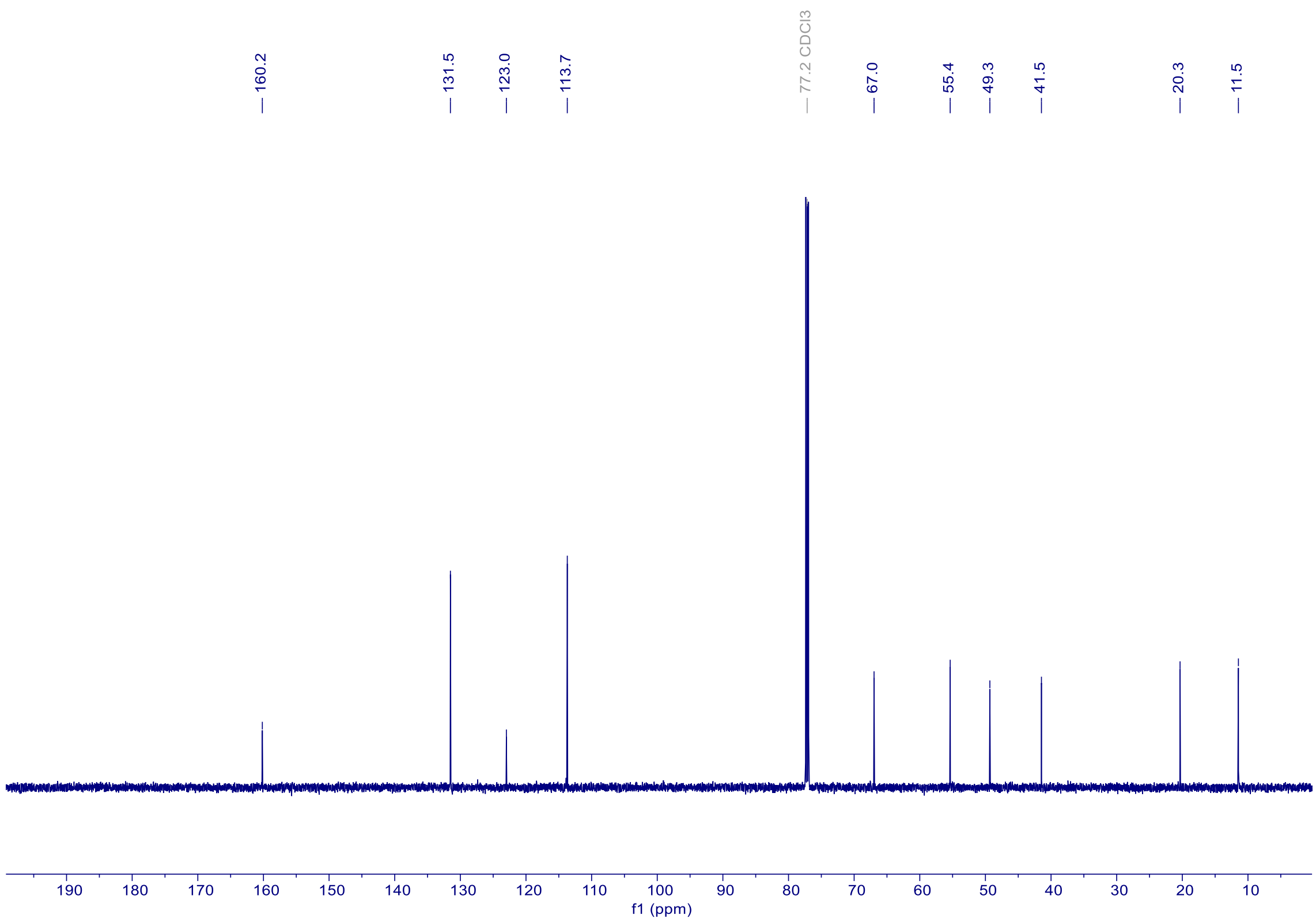

3a' – ^{11}B NMR (193 MHz, $CDCl_3$)

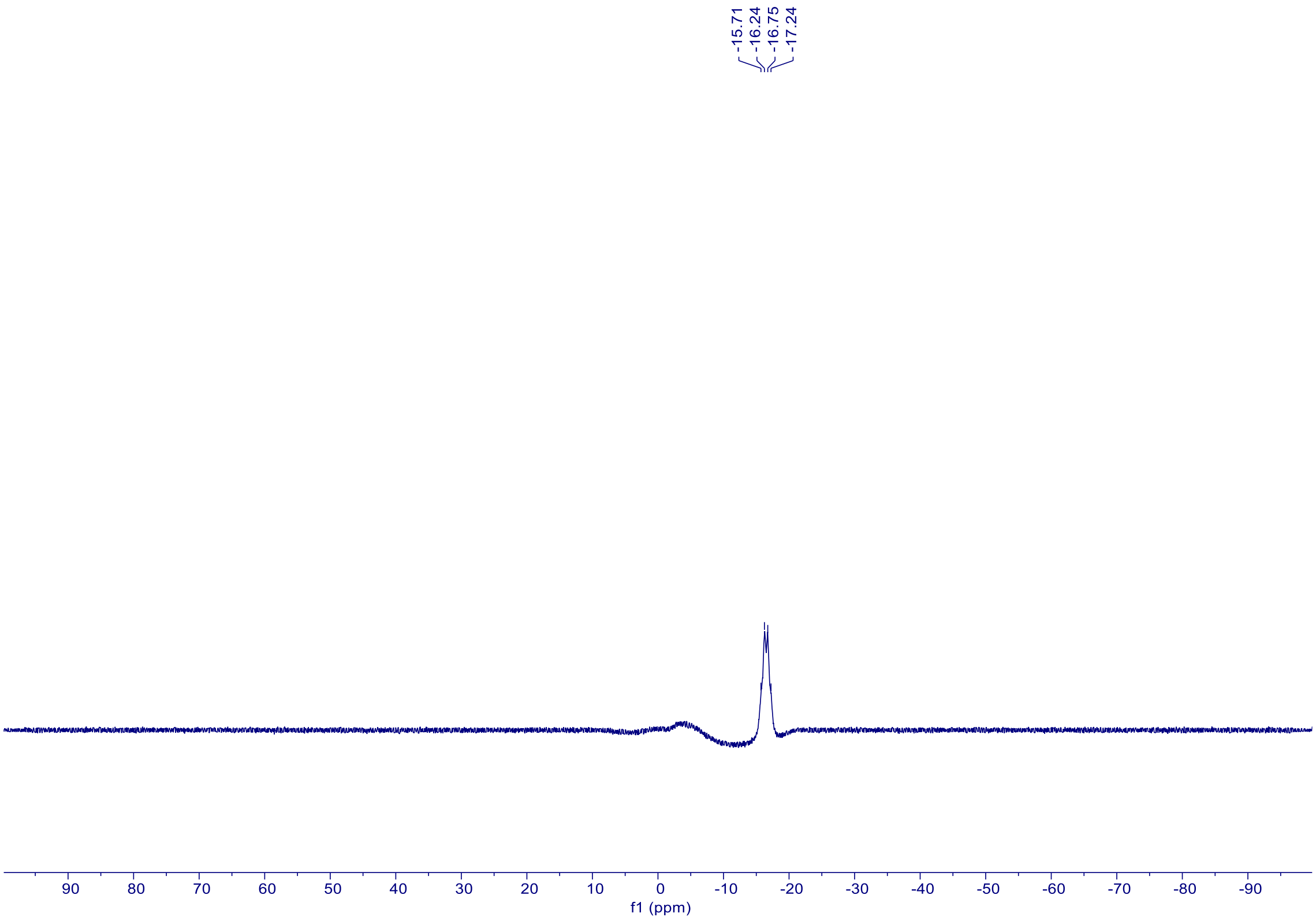

4a – ^{1}H NMR (400 MHz, $CDCl_3$)

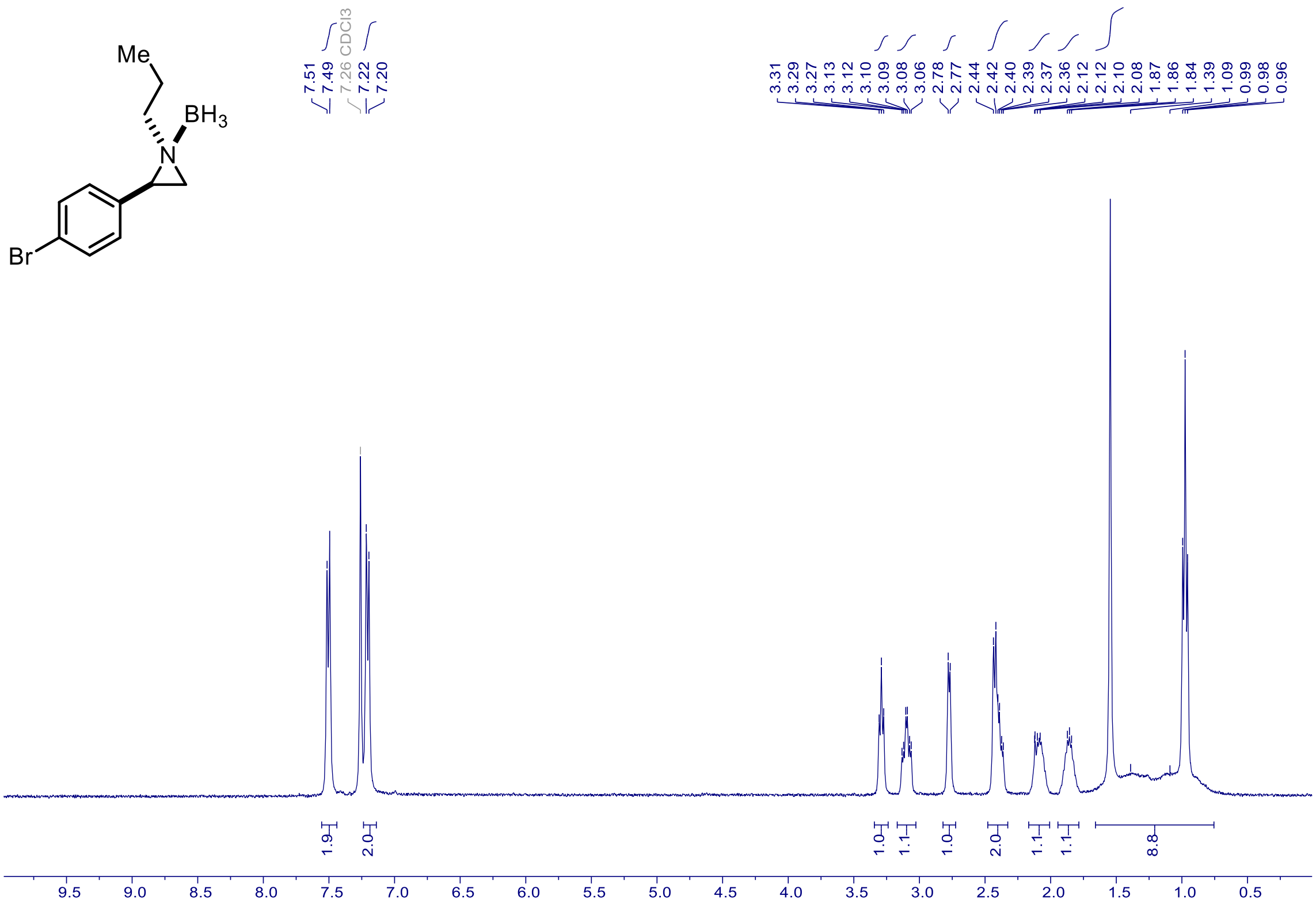

4a – ^{13}C NMR (101 MHz, $CDCl_3$)

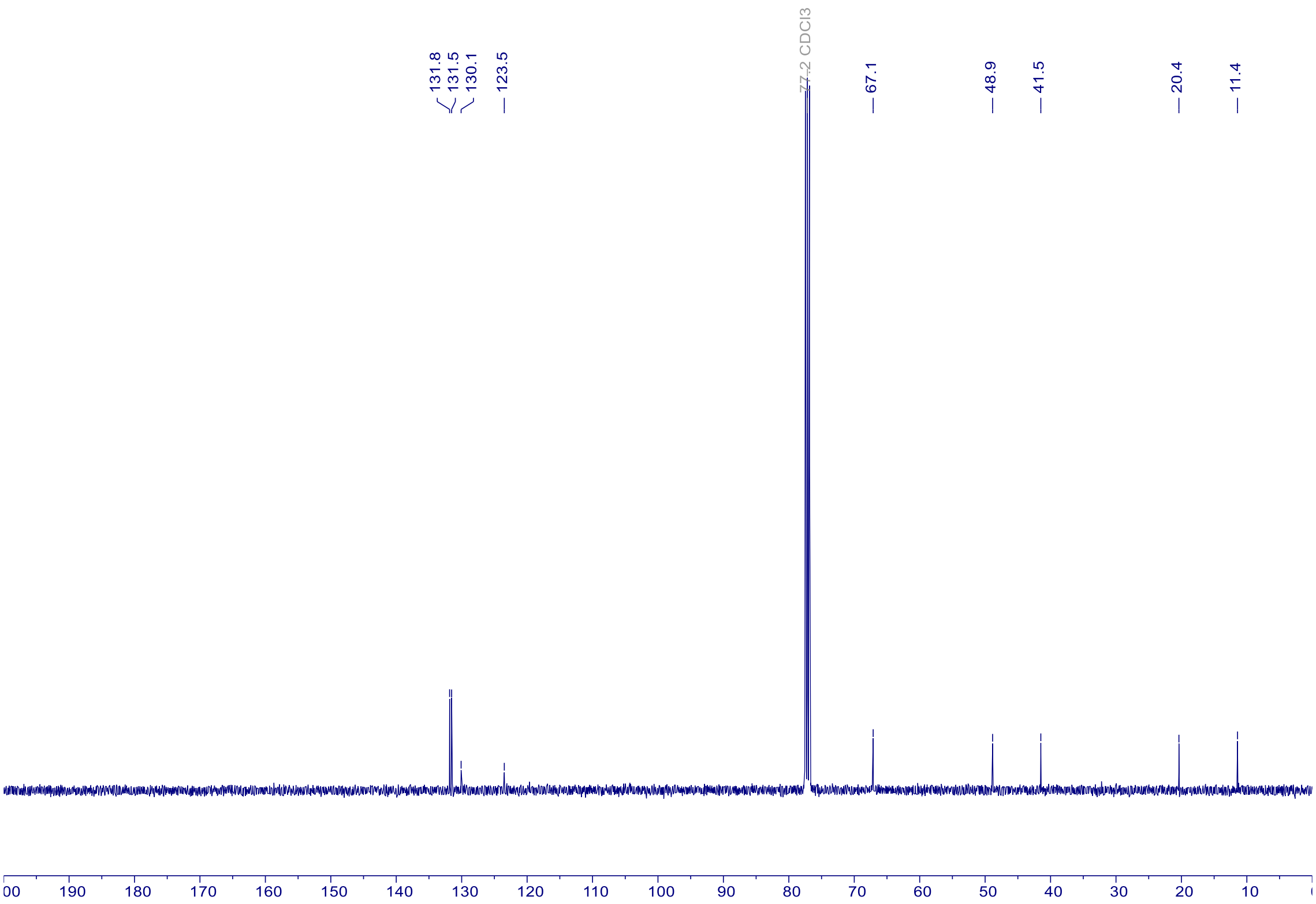

4a – ^{11}B NMR (128 MHz, $CDCl_3$)

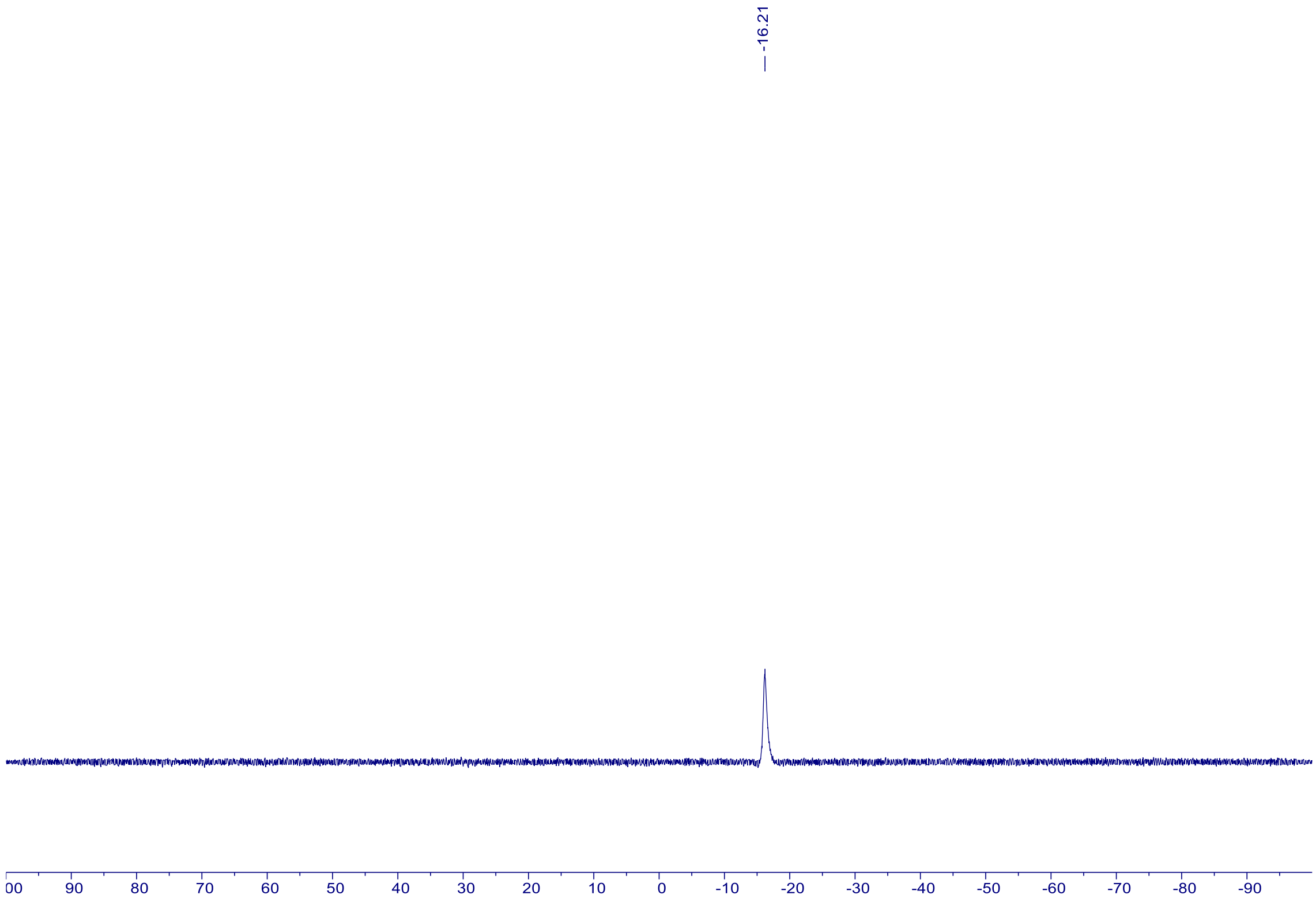
-16.21
00
90
80
70
60
50
40
30
20
10
0
-10
-20
-30
-40
-50
-60
-70
-80
-90

5a – ^{1}H NMR (400 MHz, $CDCl_3$)

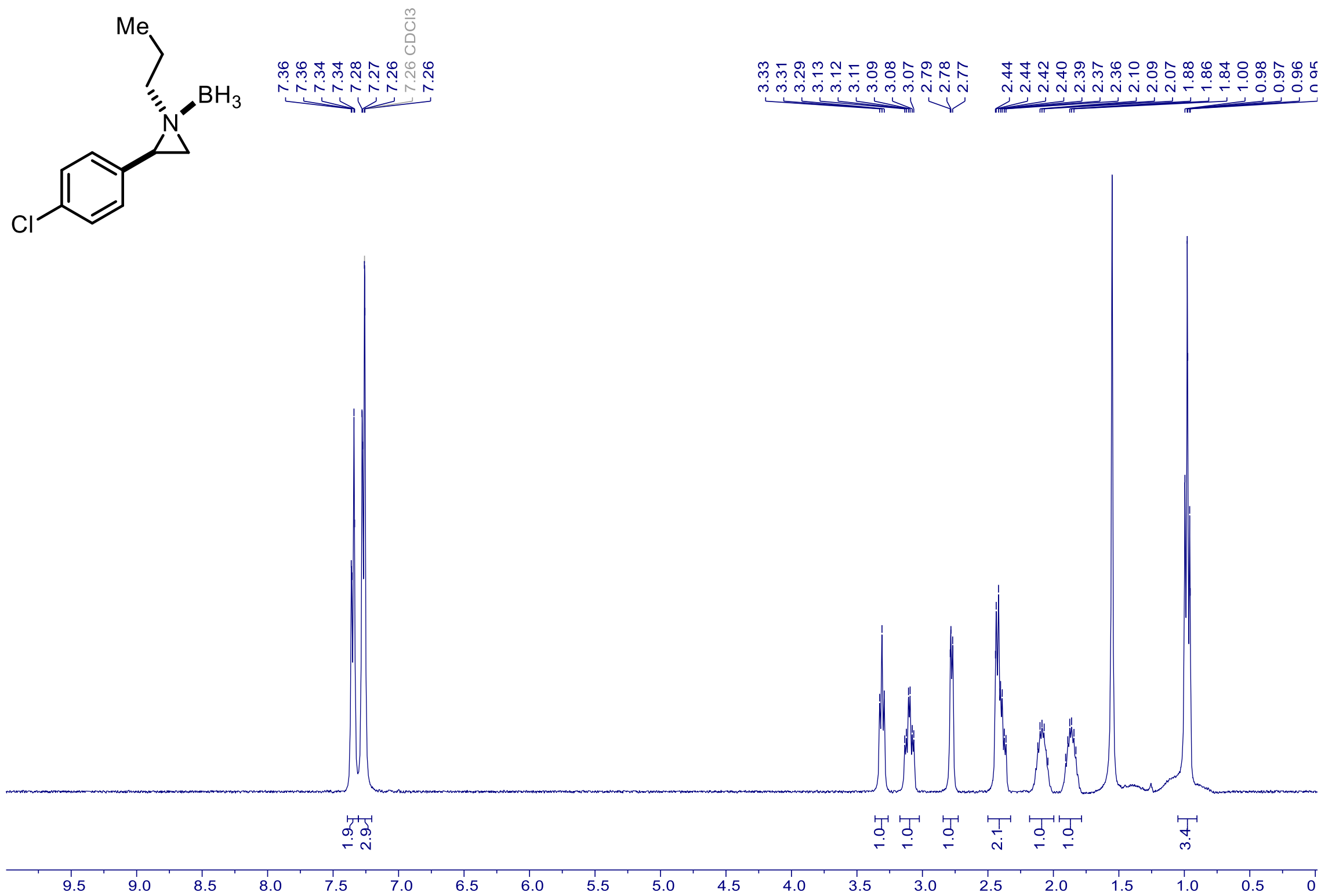

5a – ^{13}C NMR (101 MHz, $CDCl_3$)

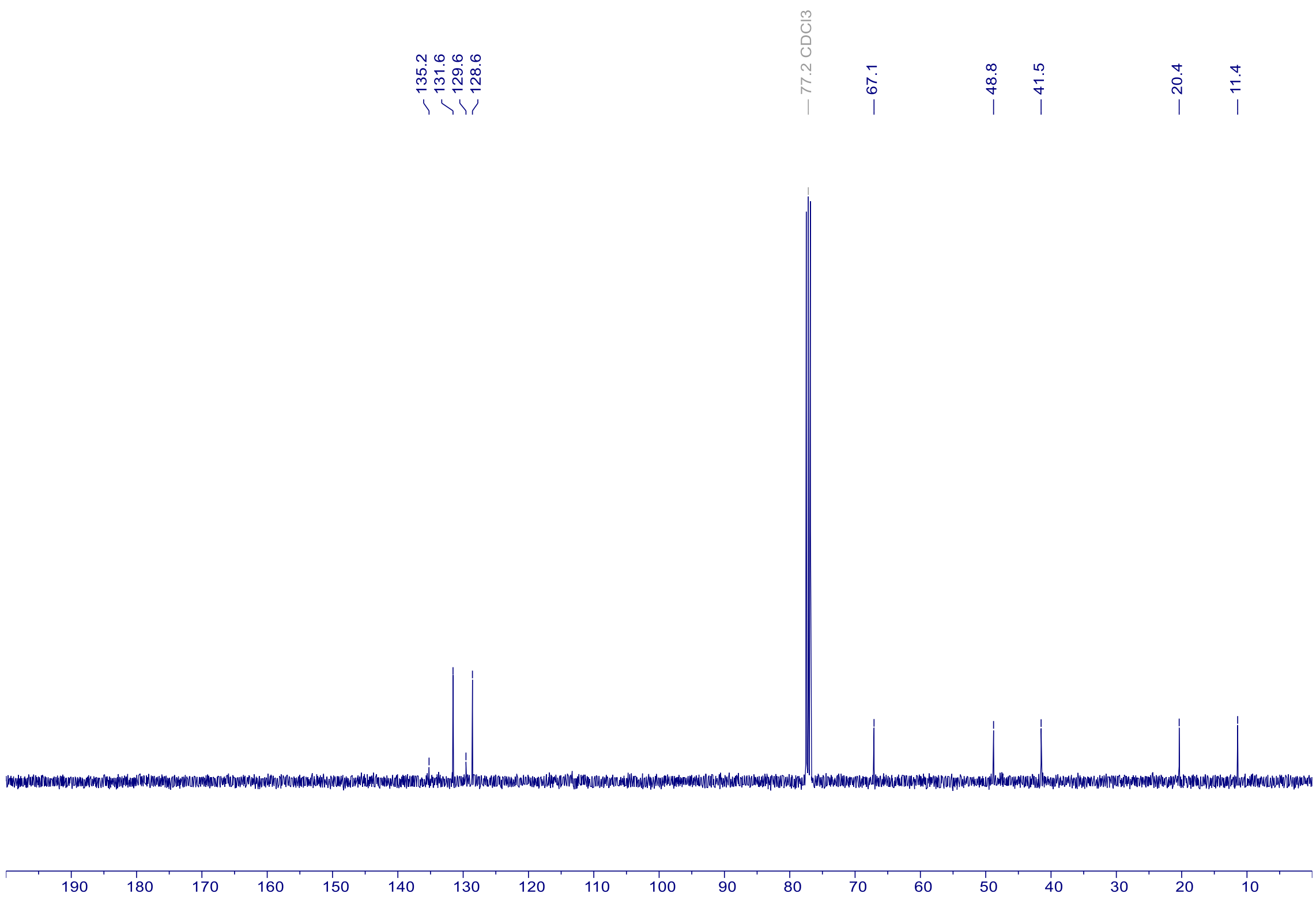

5a – ^{11}B NMR (128 MHz, $CDCl_3$)

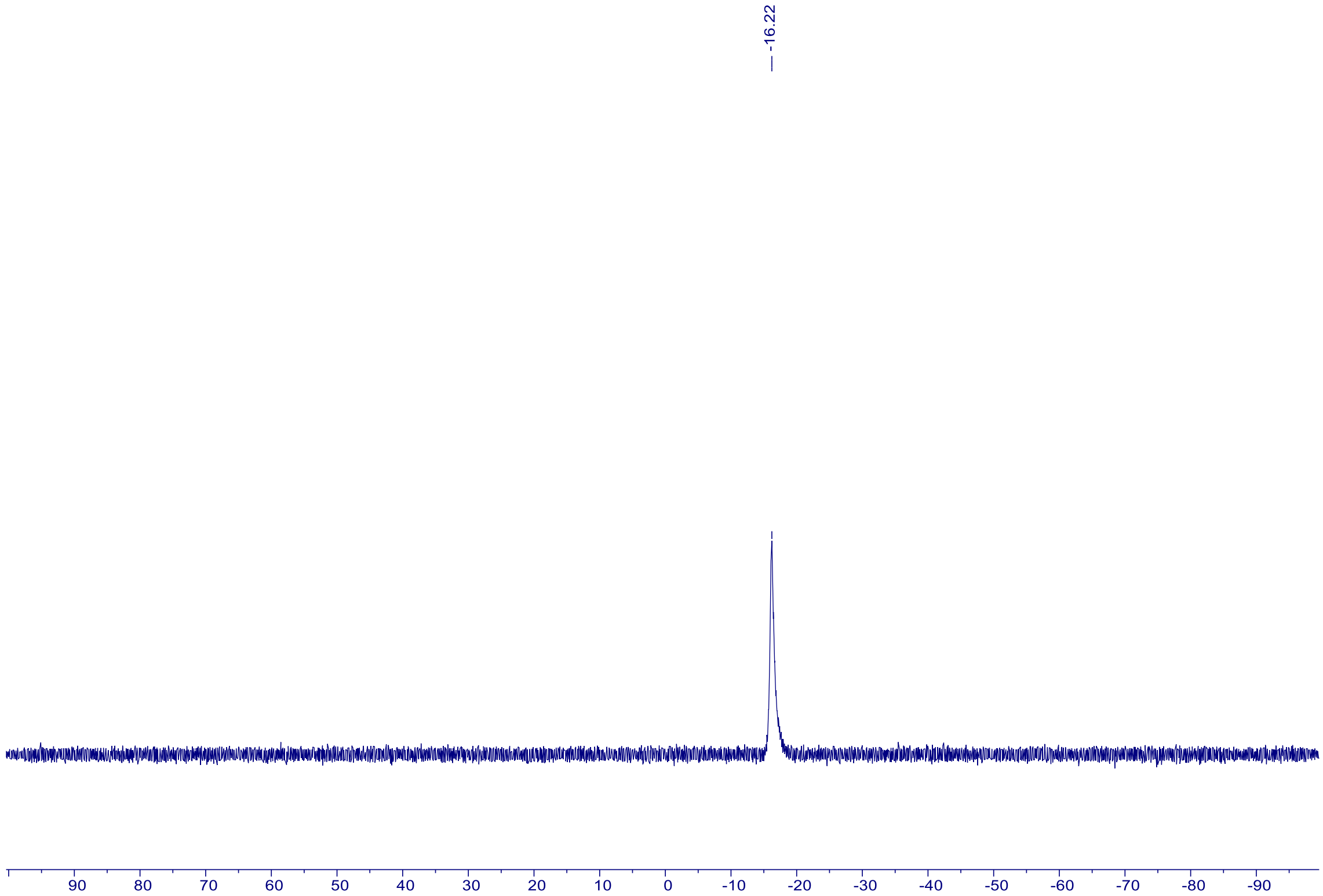

6a – ^{1}H NMR (600 MHz, $CDCl_3$)

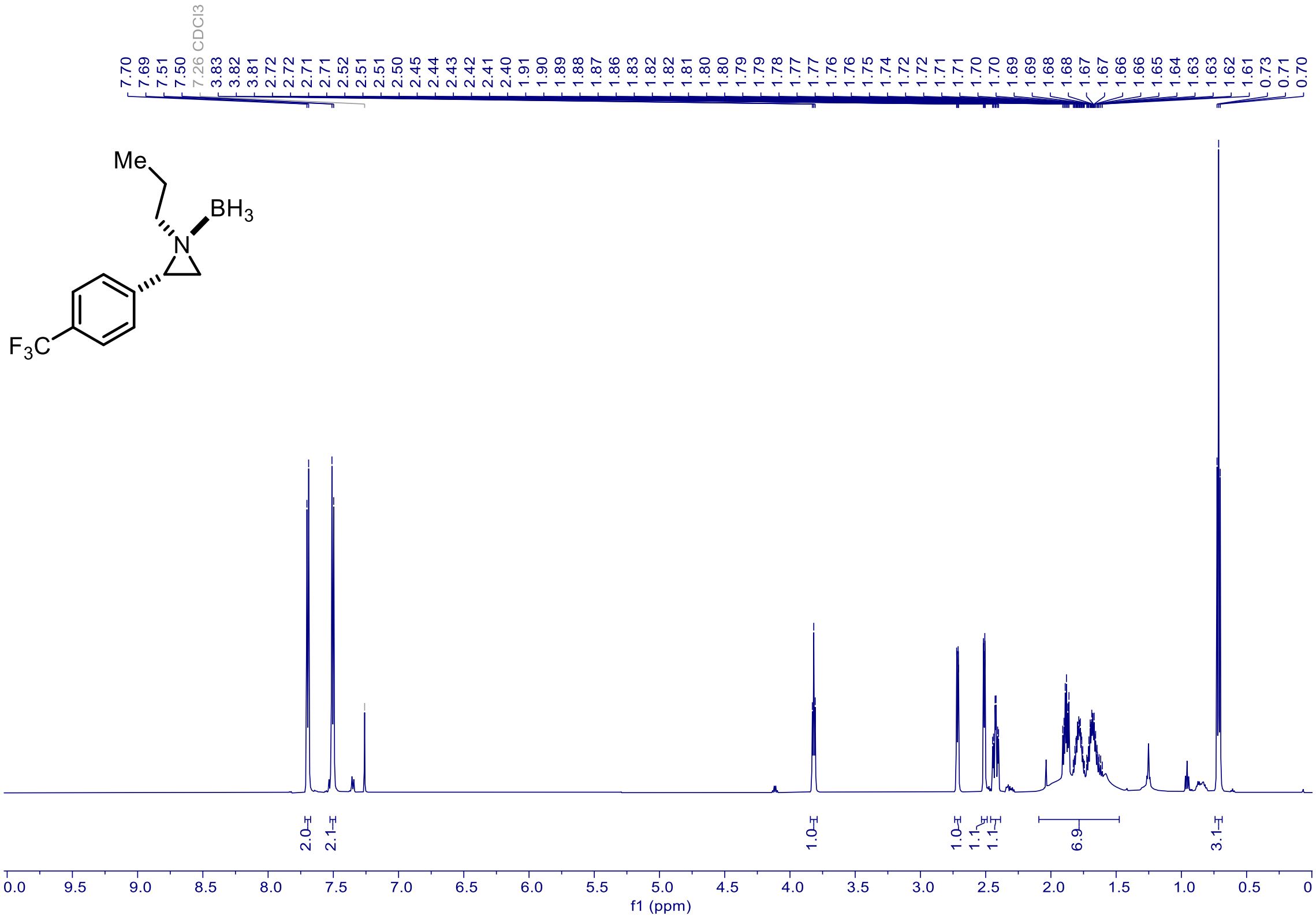

6a – ^{13}C NMR (151 MHz, $CDCl_3$)

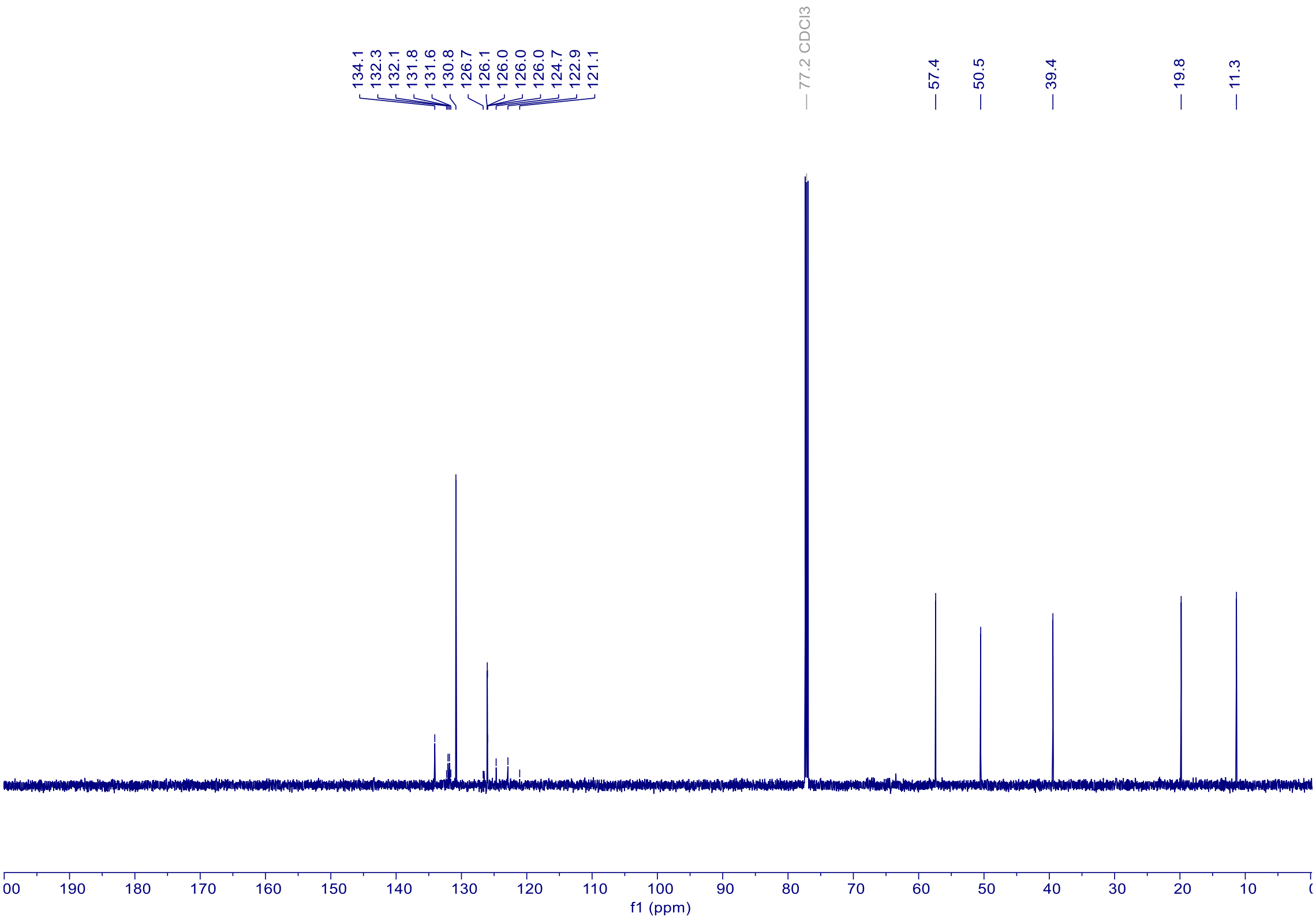

6a – ^{11}B NMR (193 MHz, $CDCl_3$)

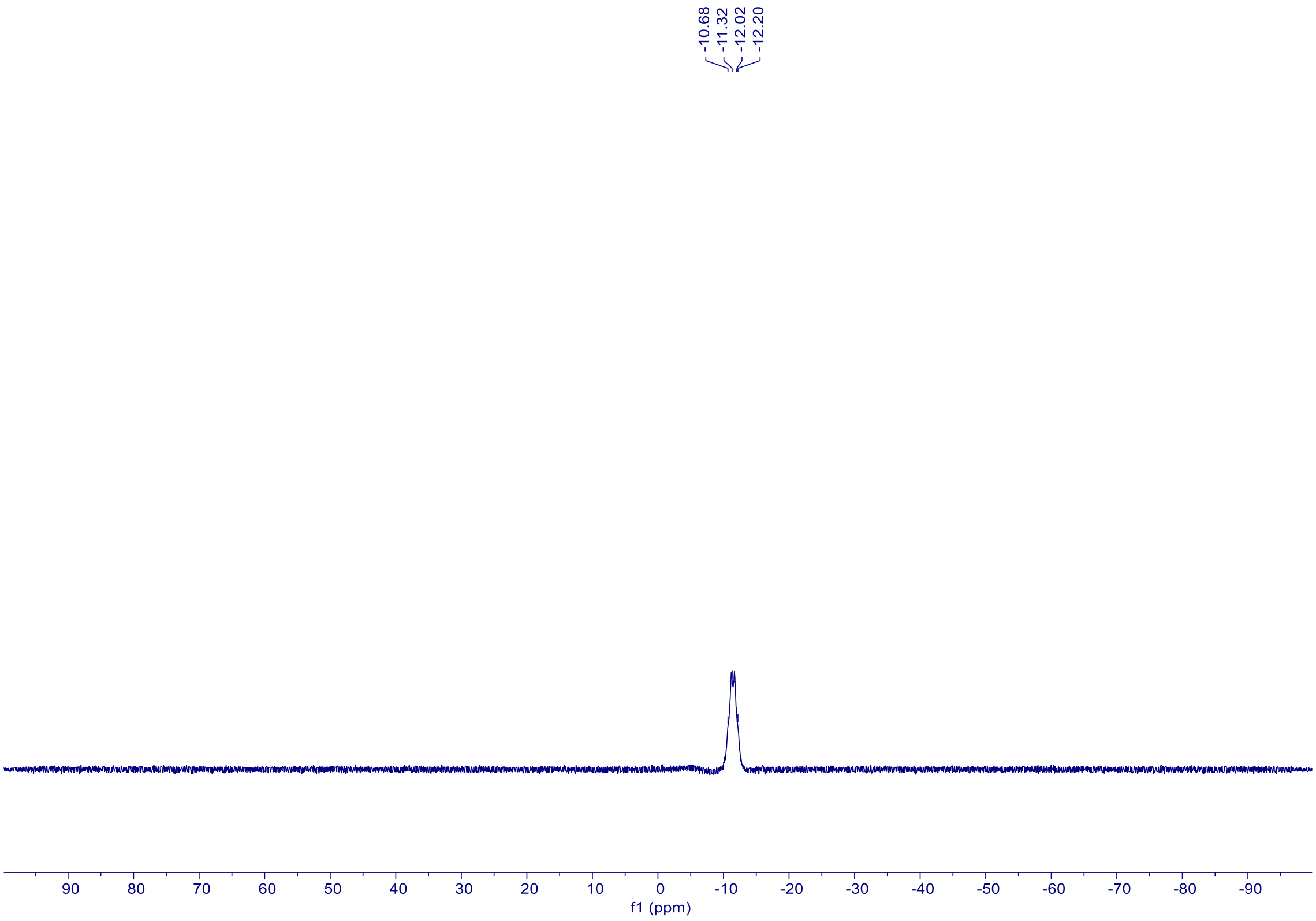

6a – ^{19}F NMR (565 MHz, $CDCl_3$)

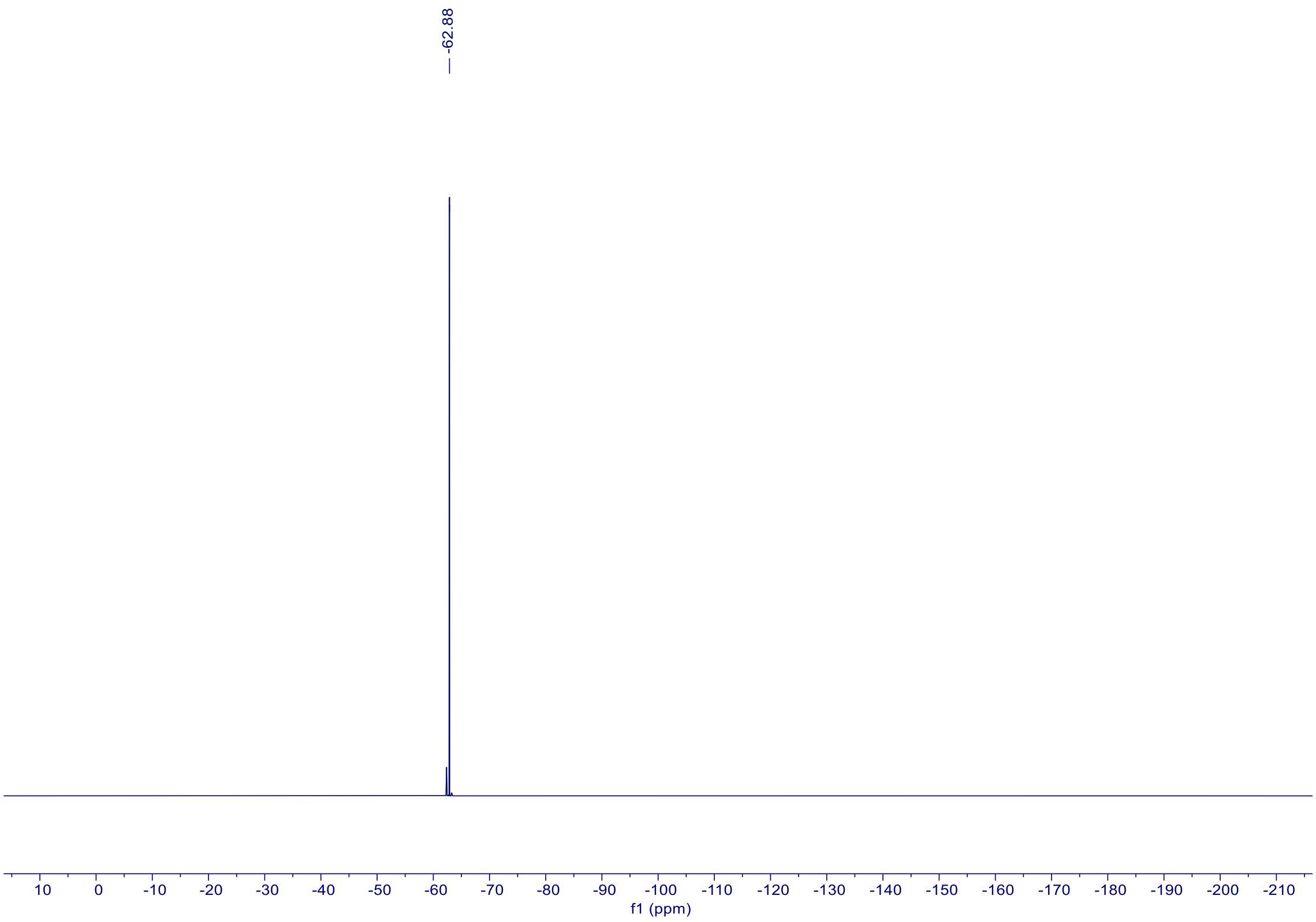

6a' – ^{1}H NMR (600 MHz, $CDCl_3$)

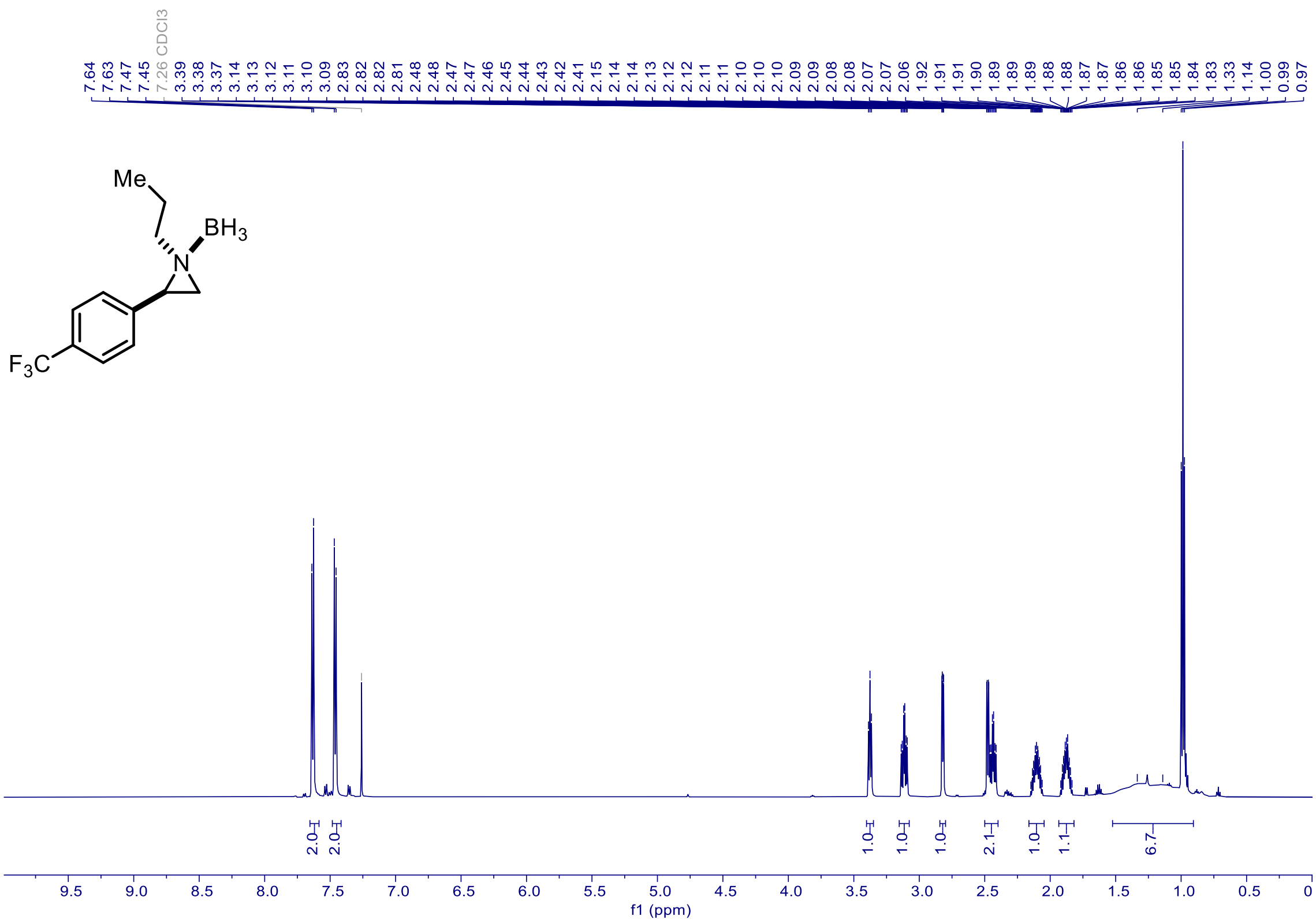

6a' – ^{13}C NMR (151 MHz, $CDCl_3$)

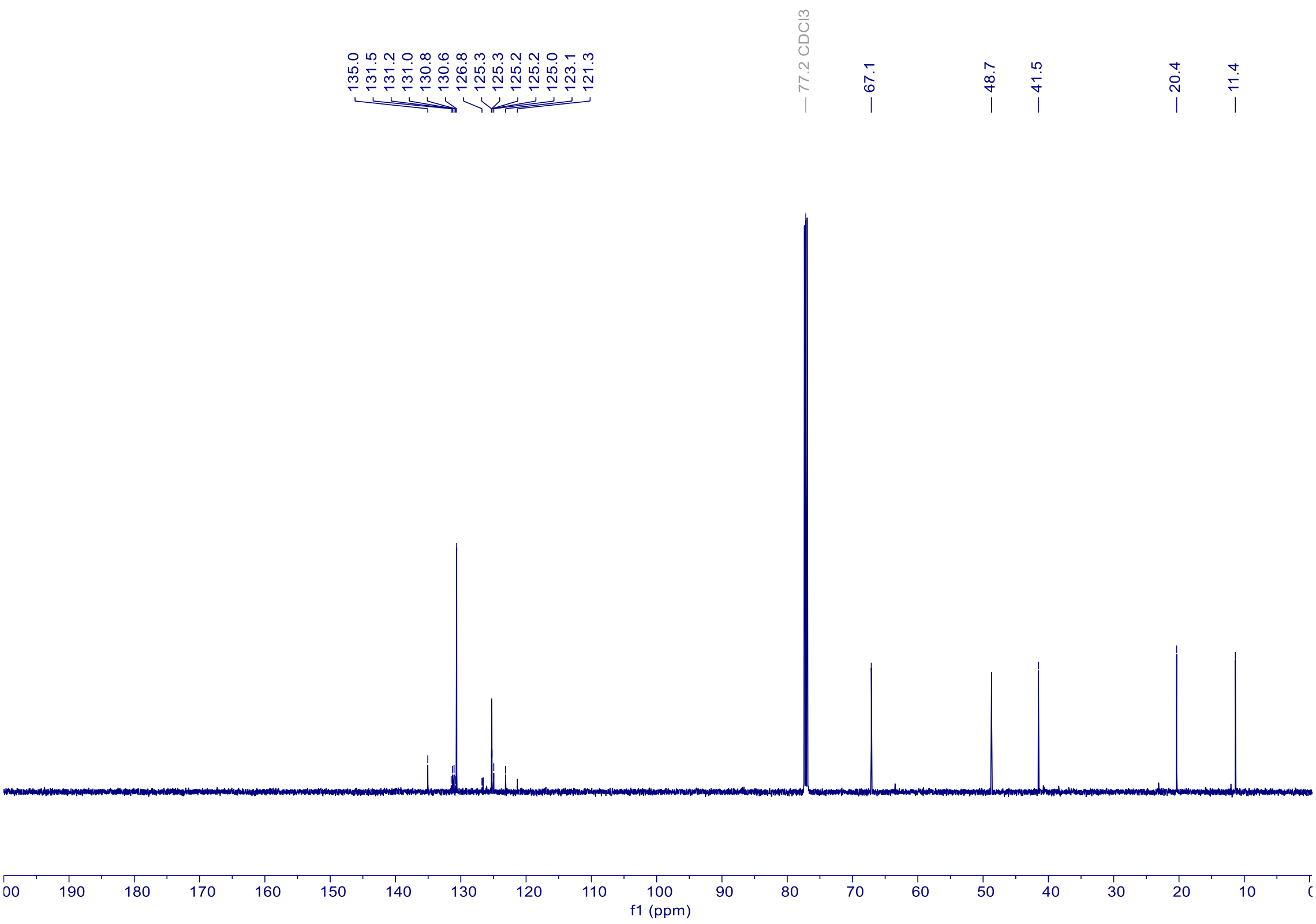

6a' – ^{11}B NMR (193 MHz, $CDCl_3$)

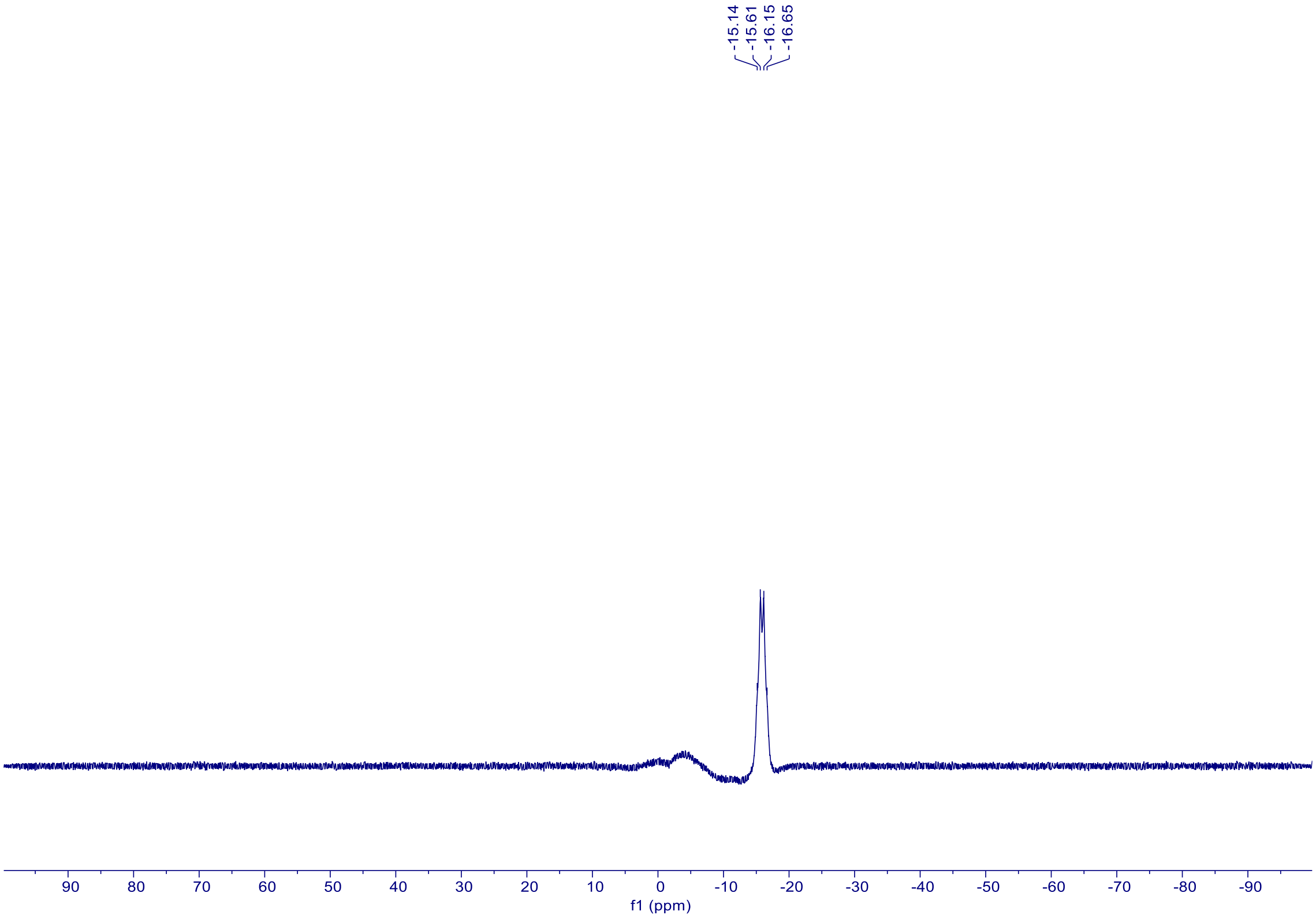

6a' – ^{19}F NMR (565 MHz, $CDCl_3$)

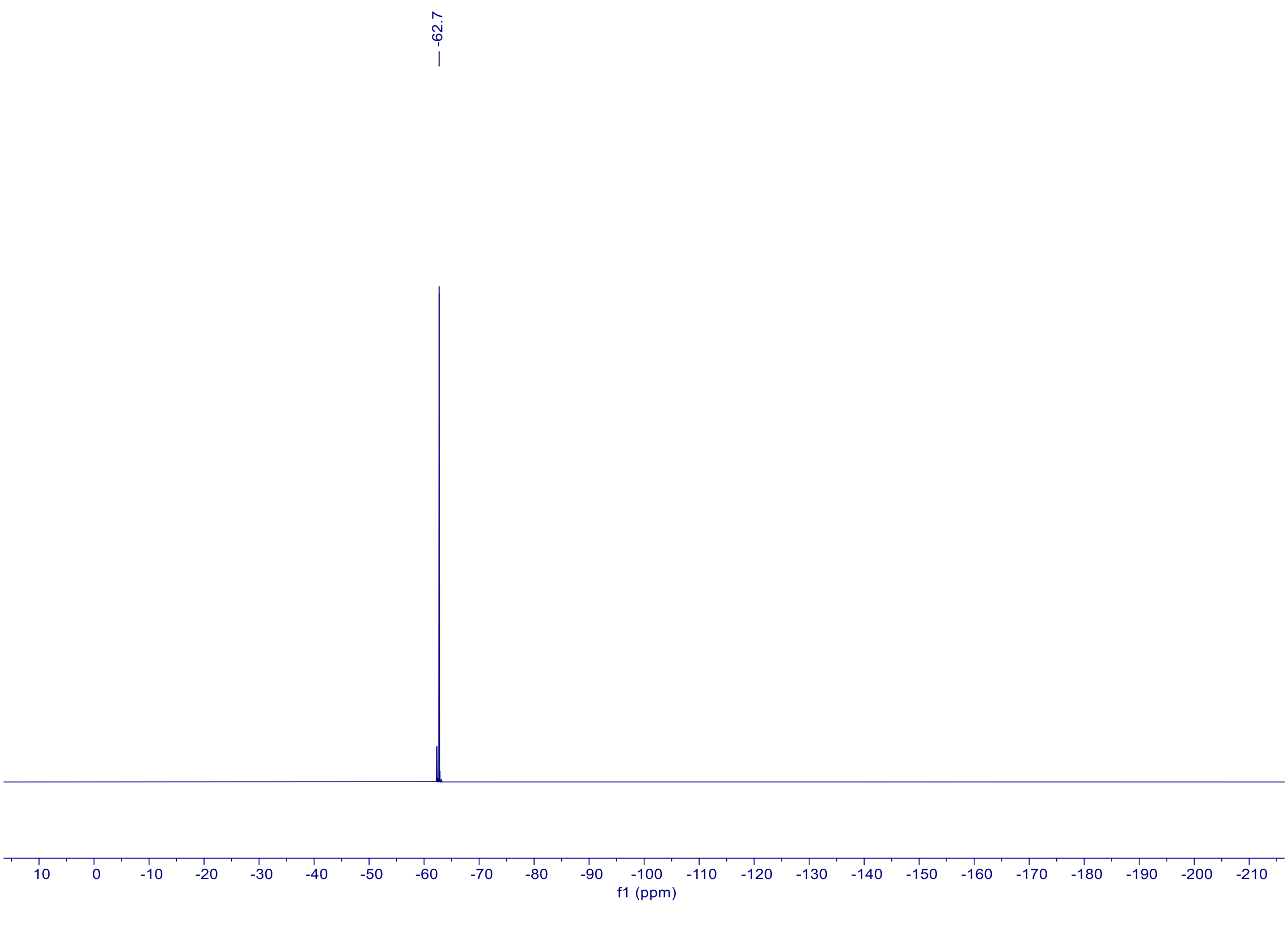

7a – ^{1}H NMR (600 MHz, $CDCl_3$)

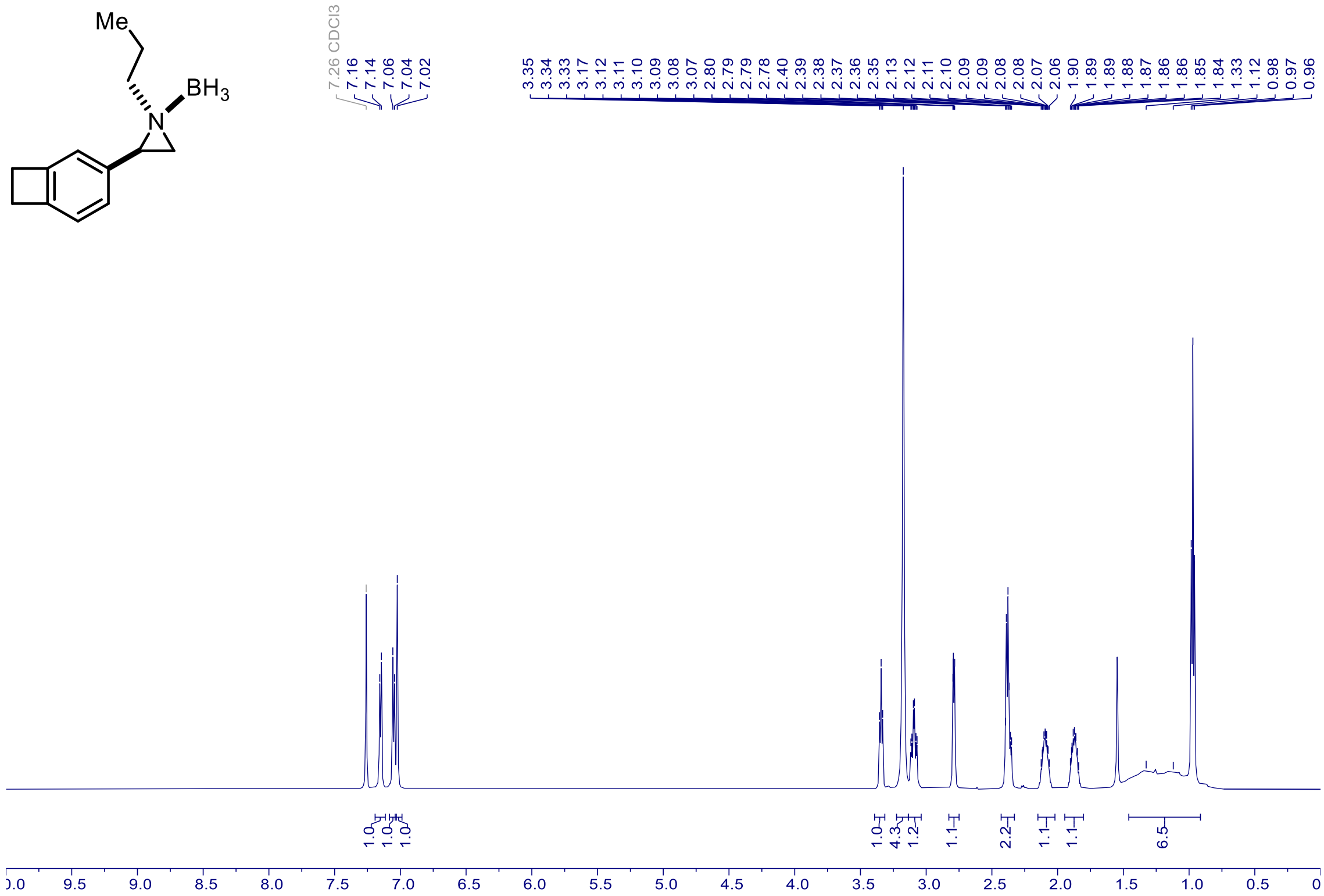

7a – ^{13}C NMR (151 MHz, $CDCl_3$)

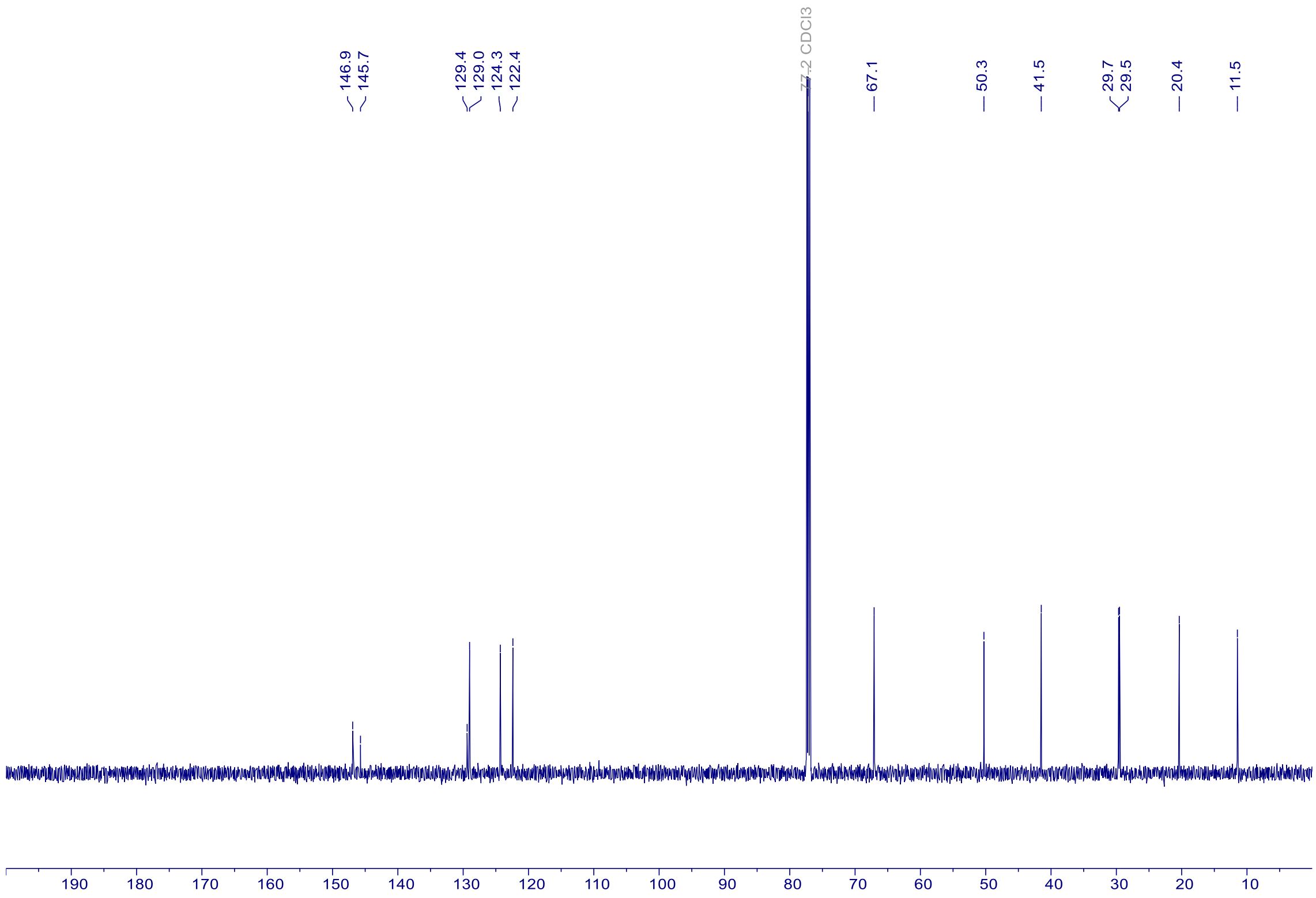

7a – ^{11}B NMR (192 MHz, $CDCl_3$)

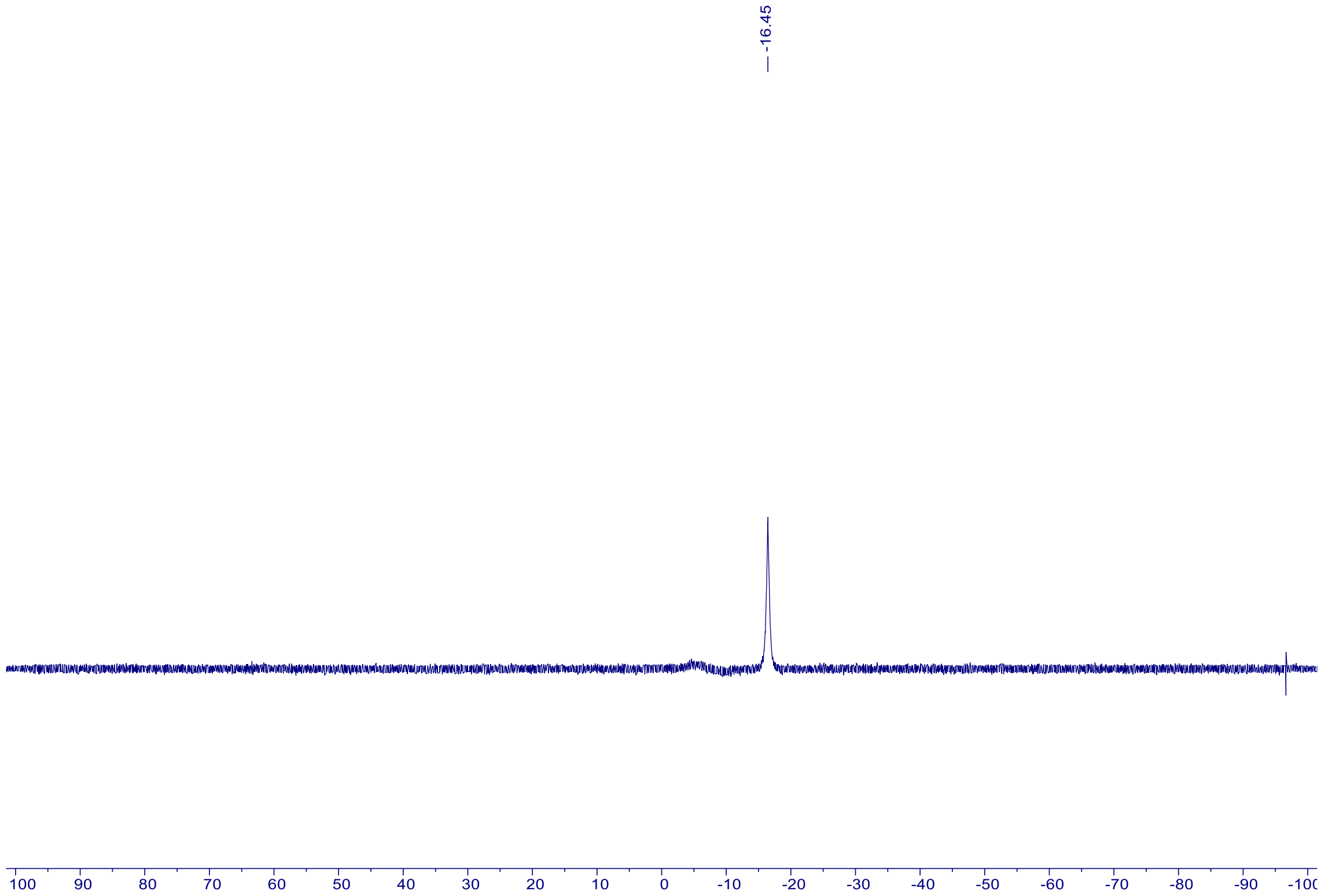

8a – ^{1}H NMR (600 MHz, $CDCl_3$)

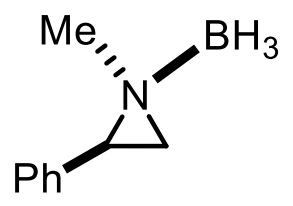

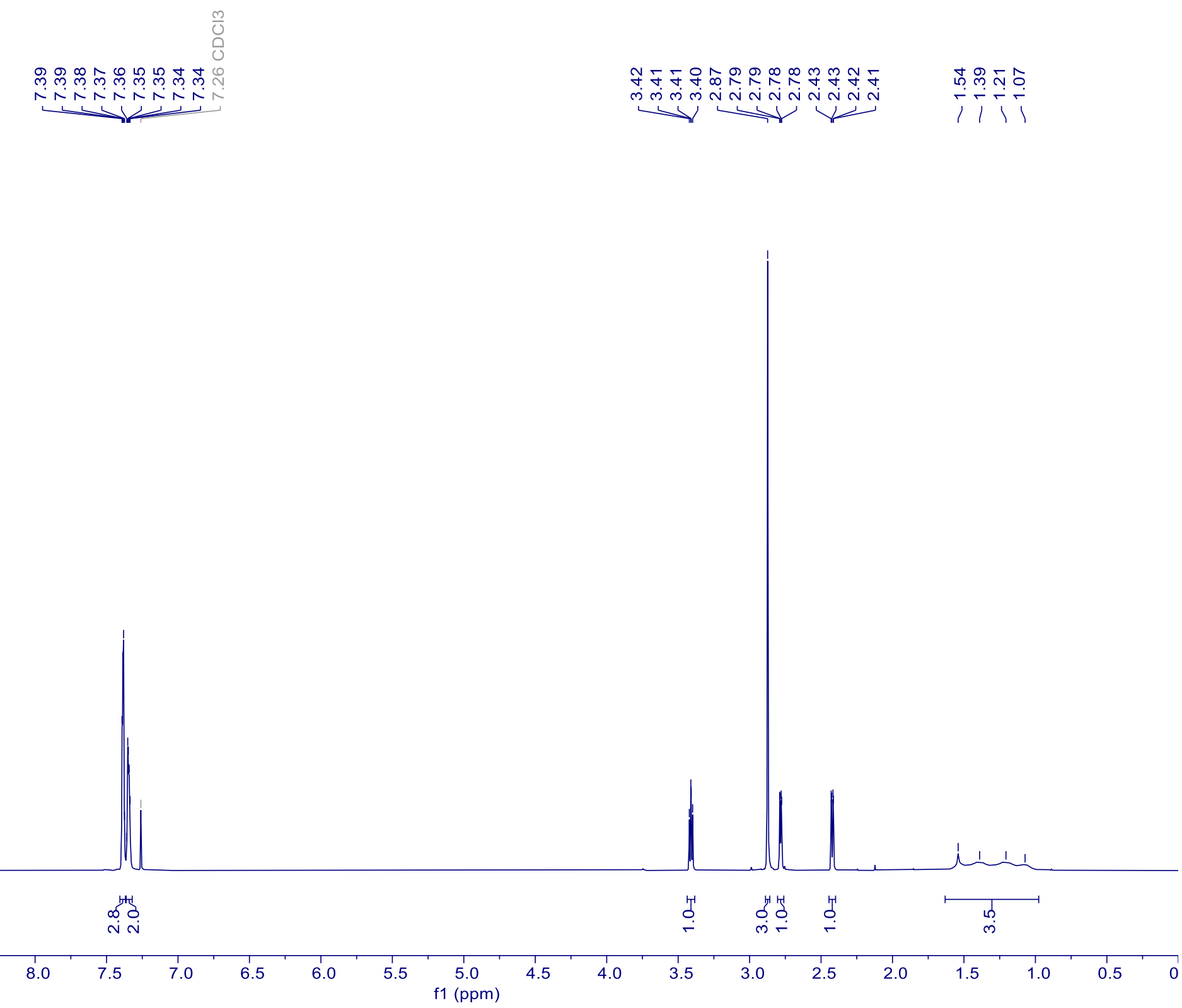

8a – ^{13}C NMR (151 MHz, $CDCl_3$)

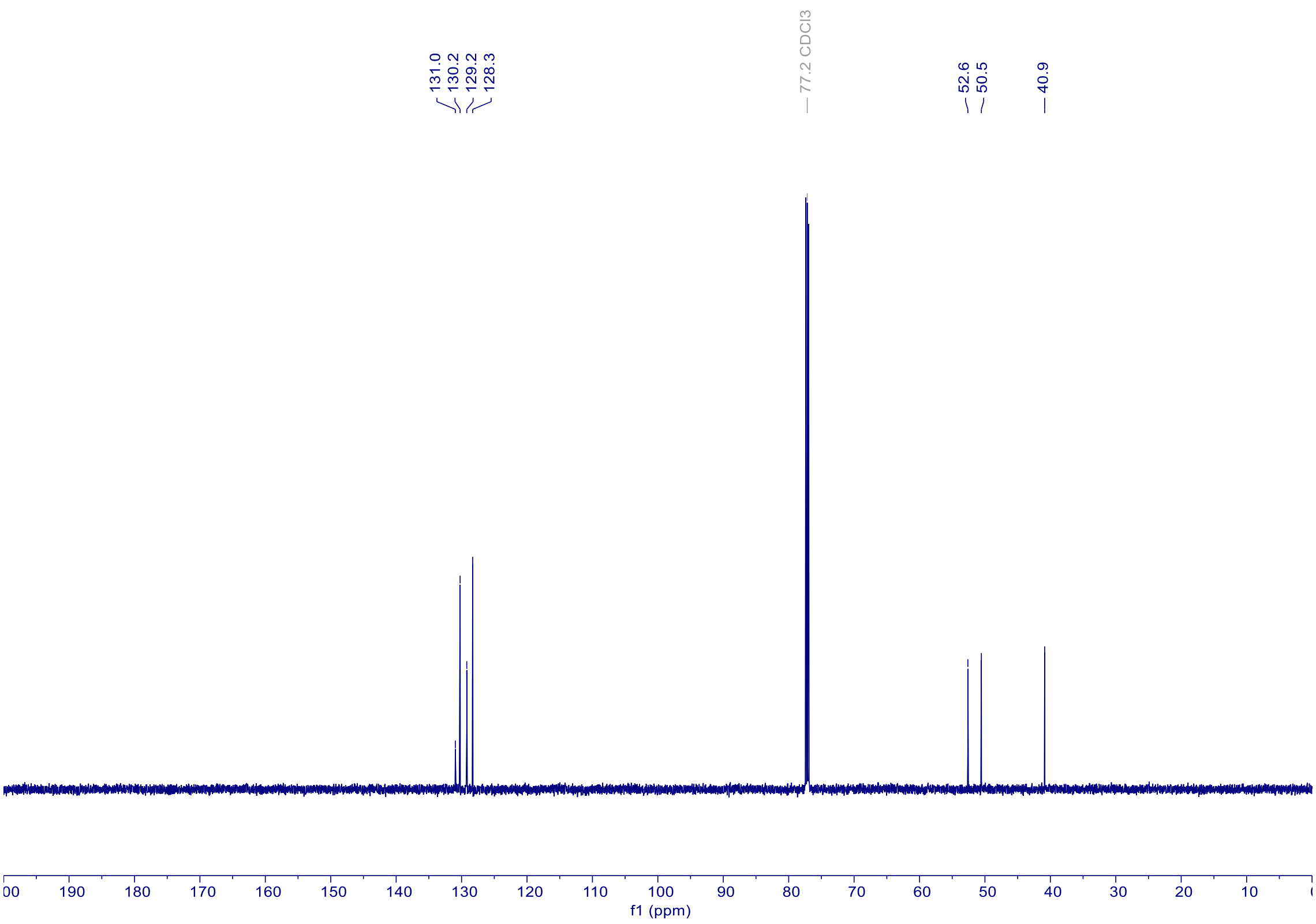

8a – ^{11}B NMR (193 MHz, $CDCl_3$)

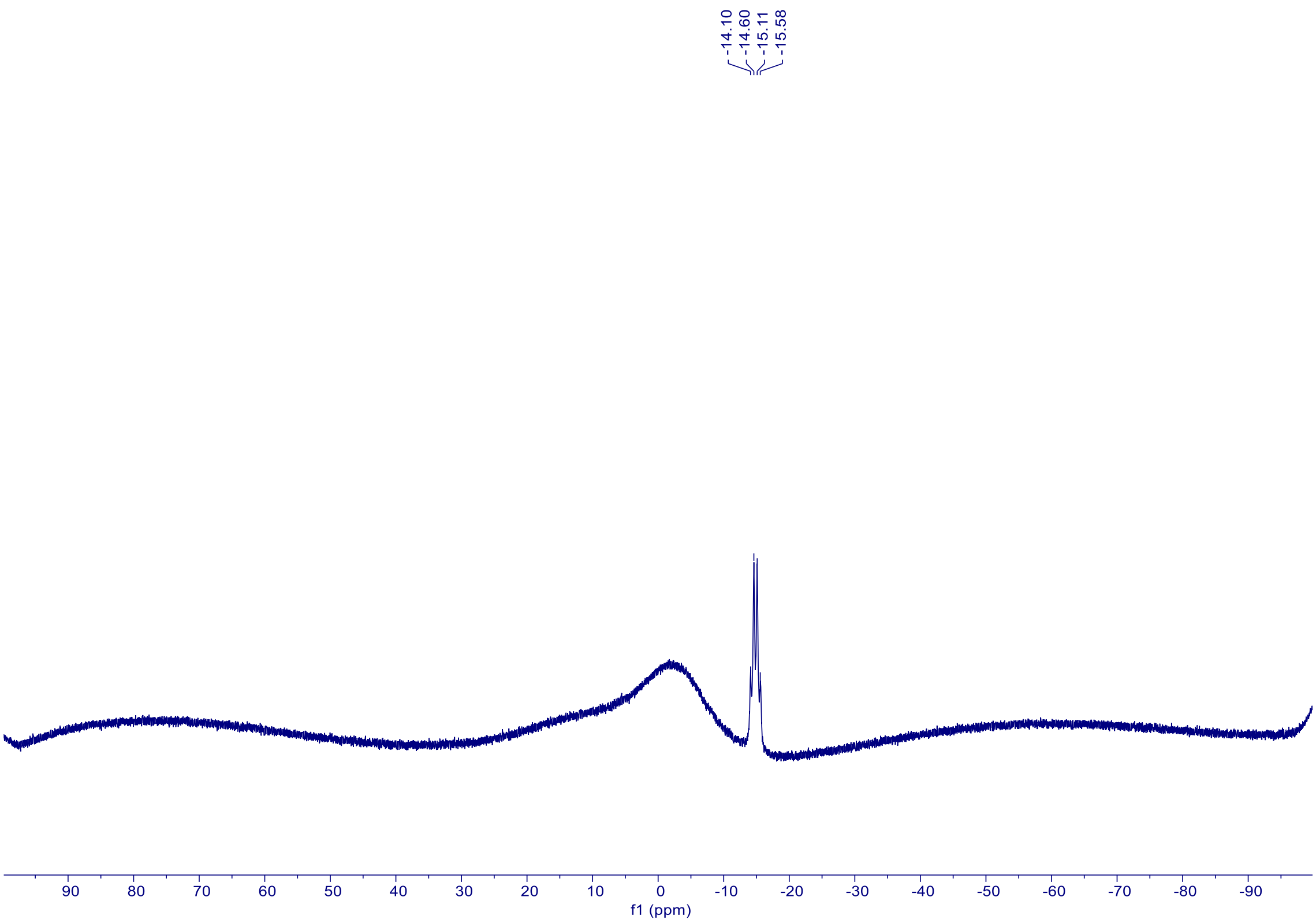

9a – ^{1}H NMR (400 MHz, $CDCl_3$)

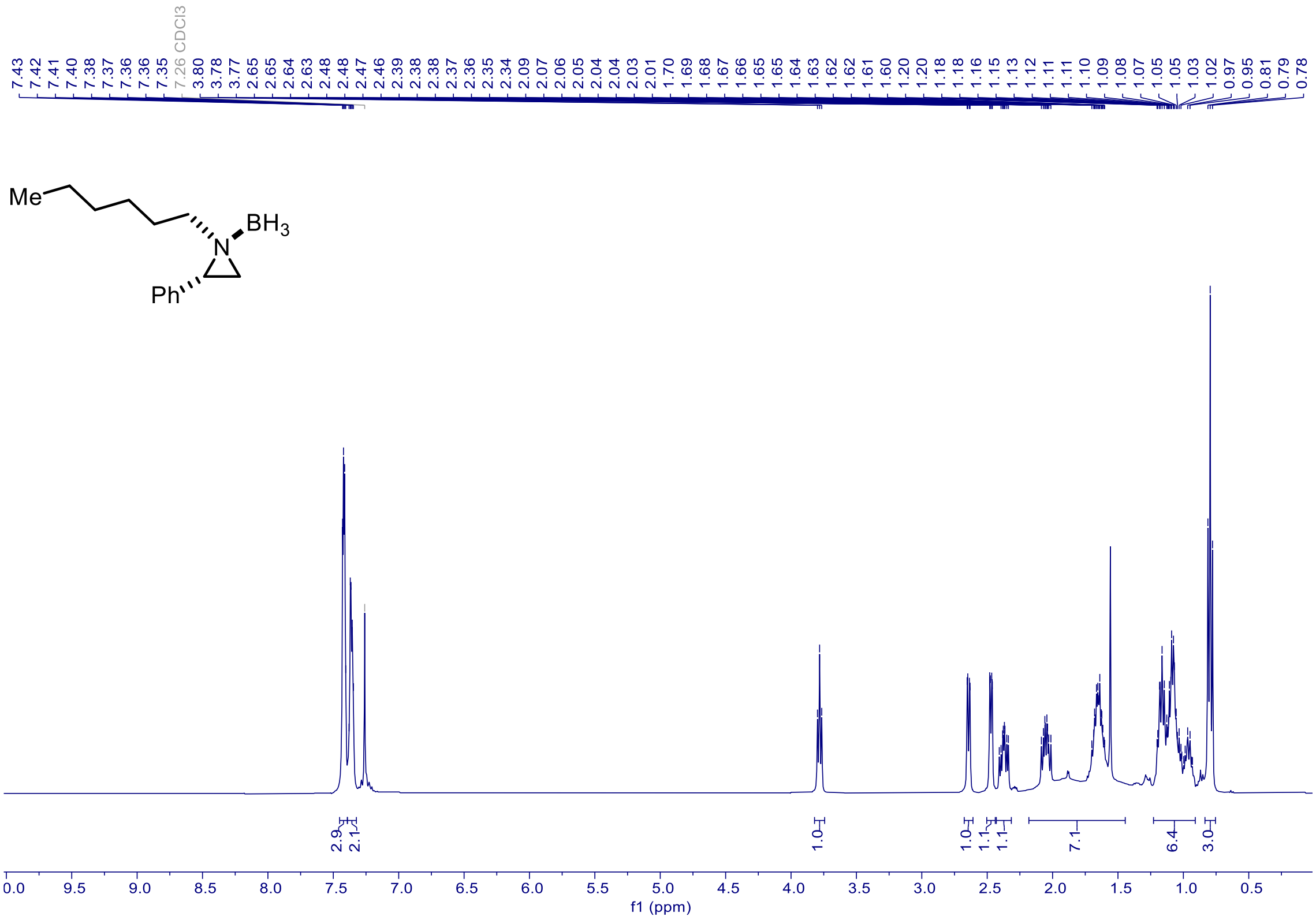

9a – ^{13}C NMR (151 MHz, $CDCl_3$)

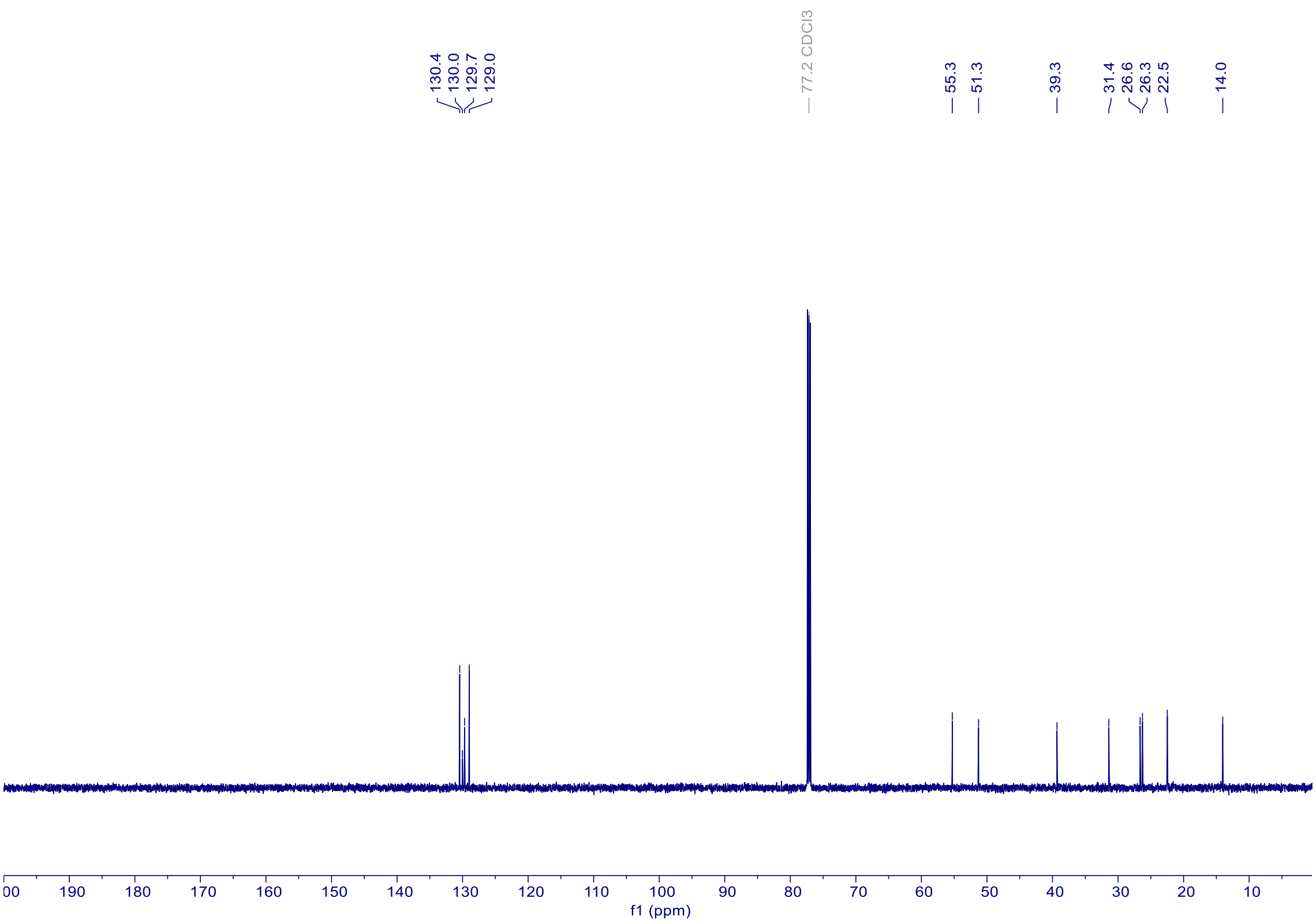

9a – ^{11}B NMR (193 MHz, $CDCl_3$)

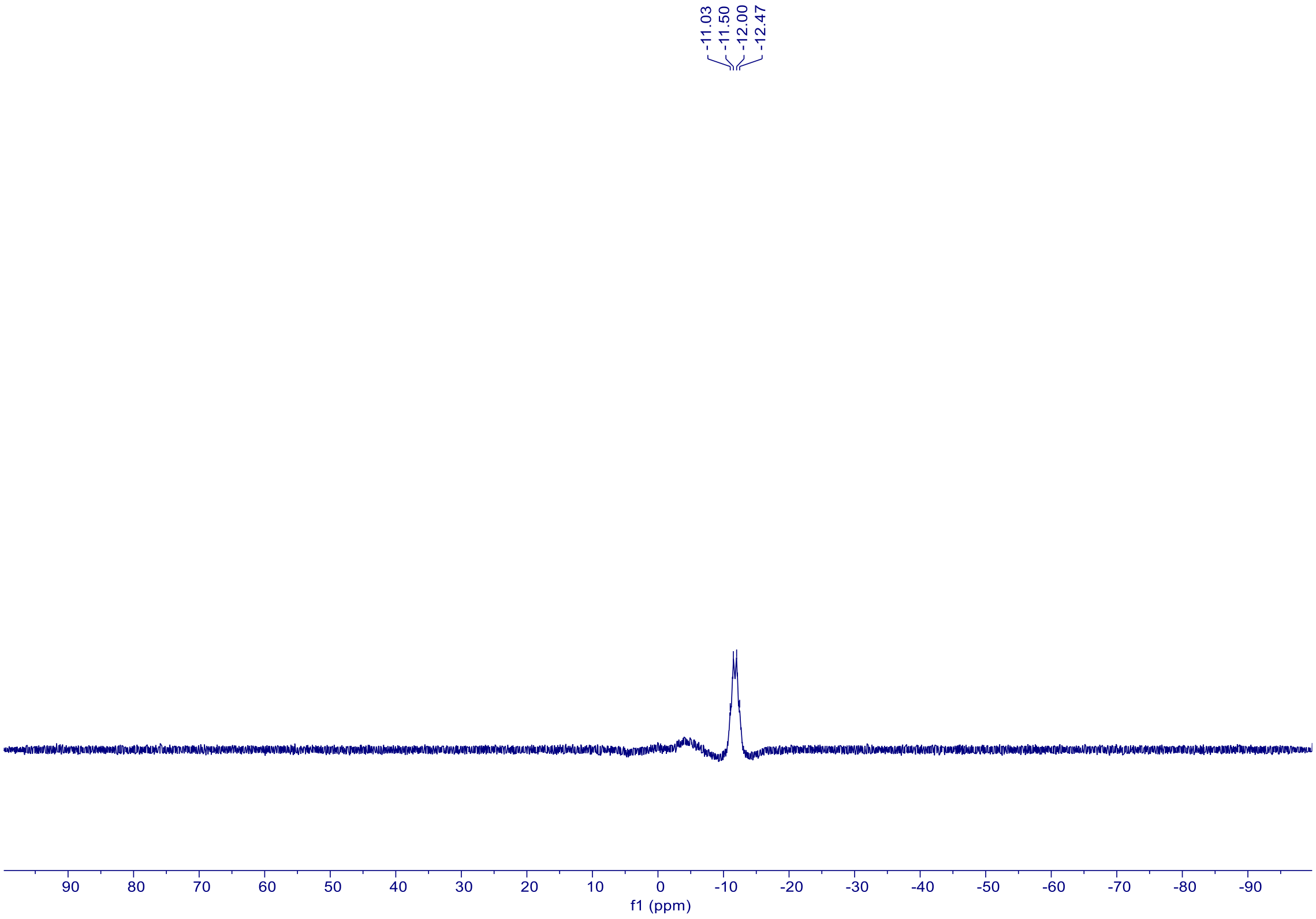

9a’ – ^{1}H NMR (600 MHz, CDCl$_3$)

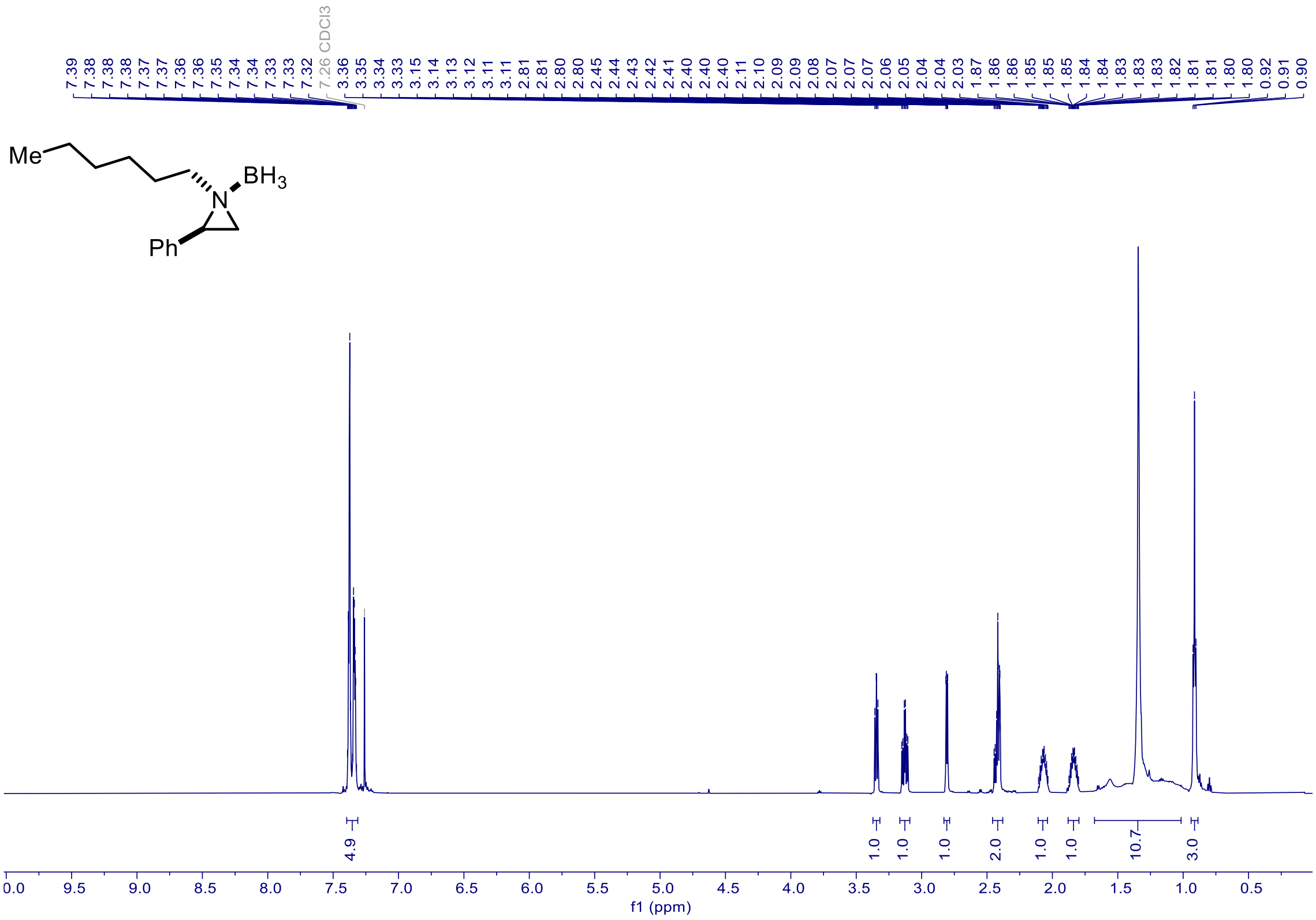

9a’ – ^{13}C NMR (151 MHz, CDCl$_3$)

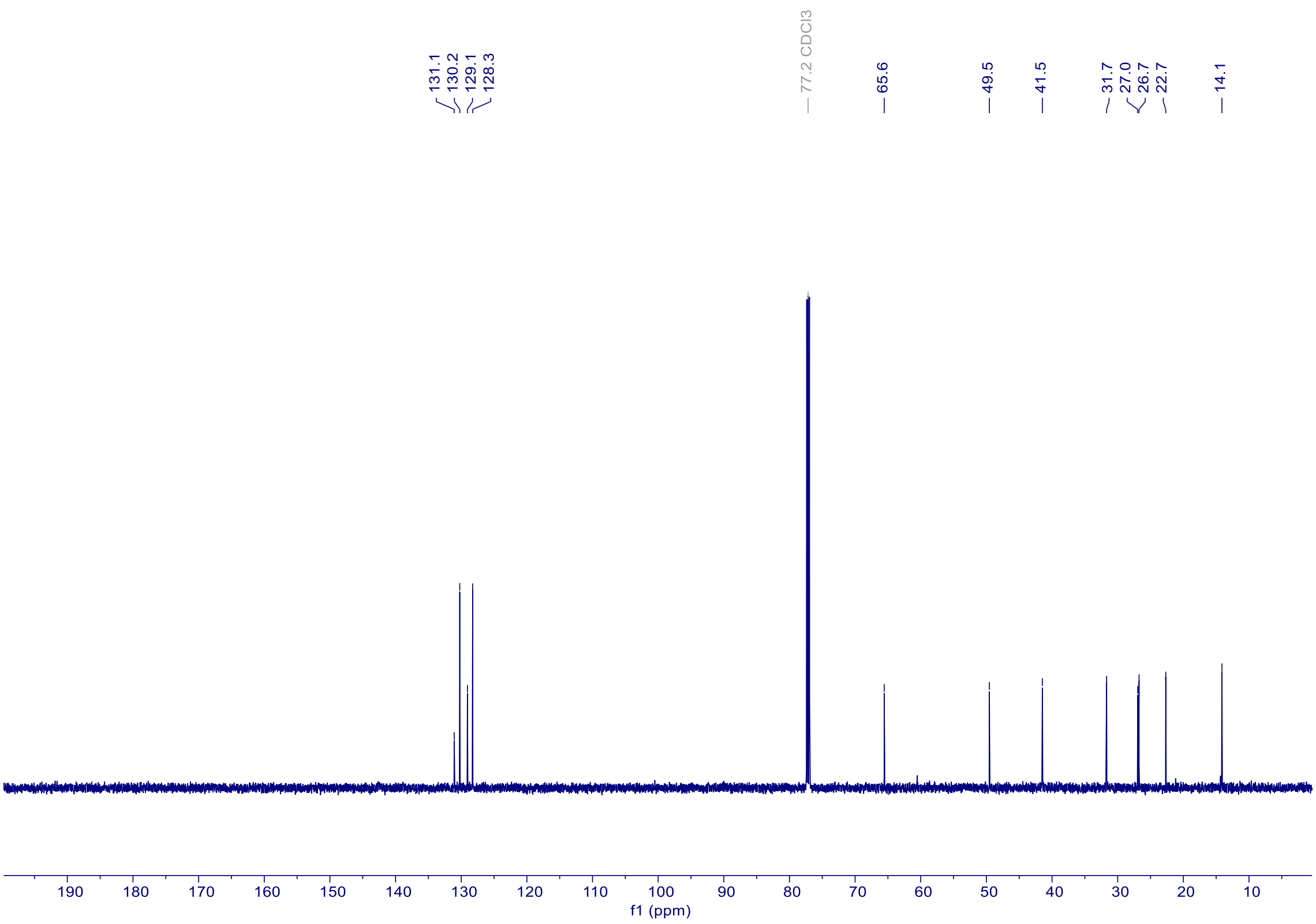

9a' – ^{11}B NMR (193 MHz, $CDCl_3$)

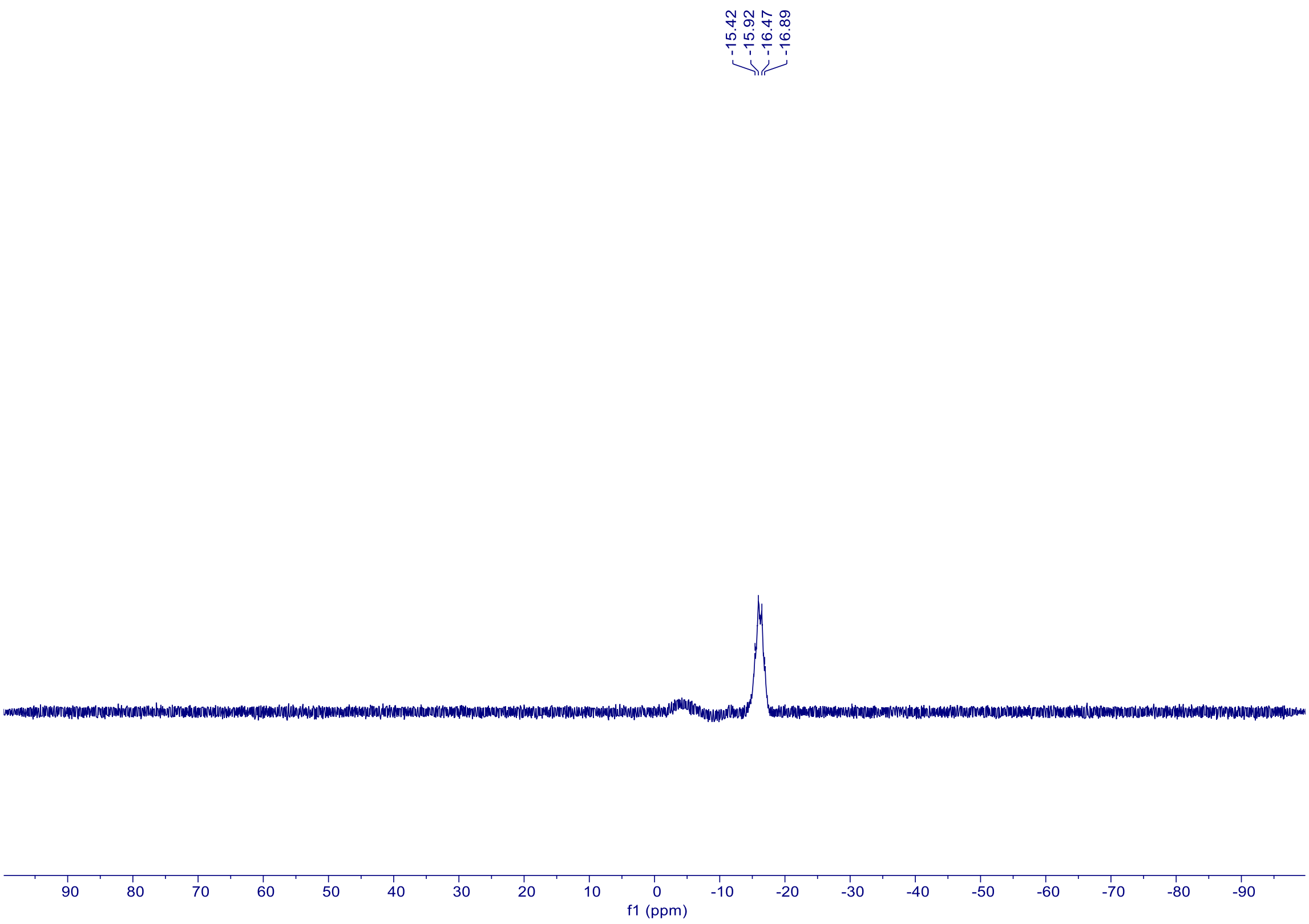

10a – ^{1}H NMR (600 MHz, CDCl$_3$)

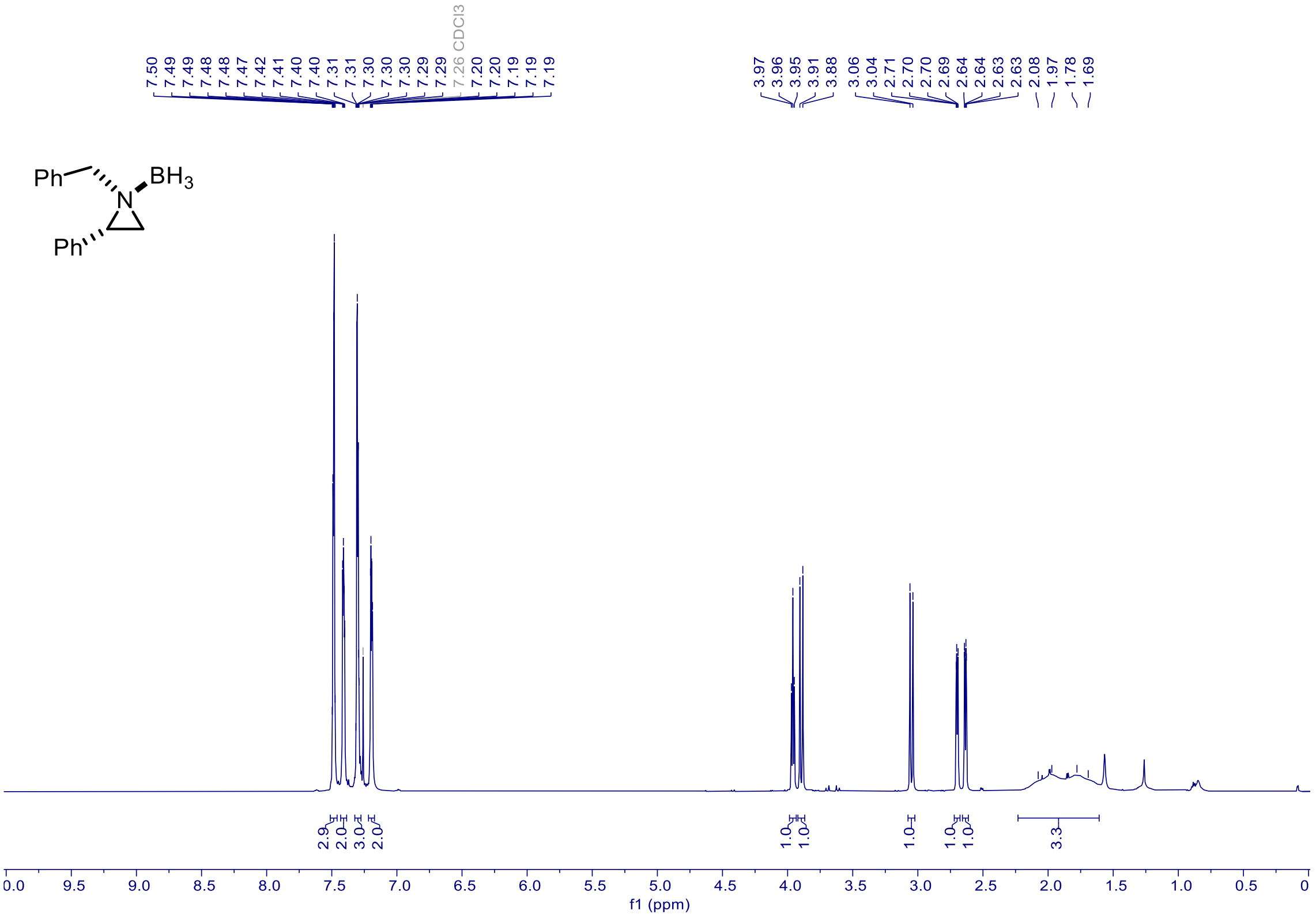

10a – ^{13}C NMR (151 MHz, CDCl$_3$)

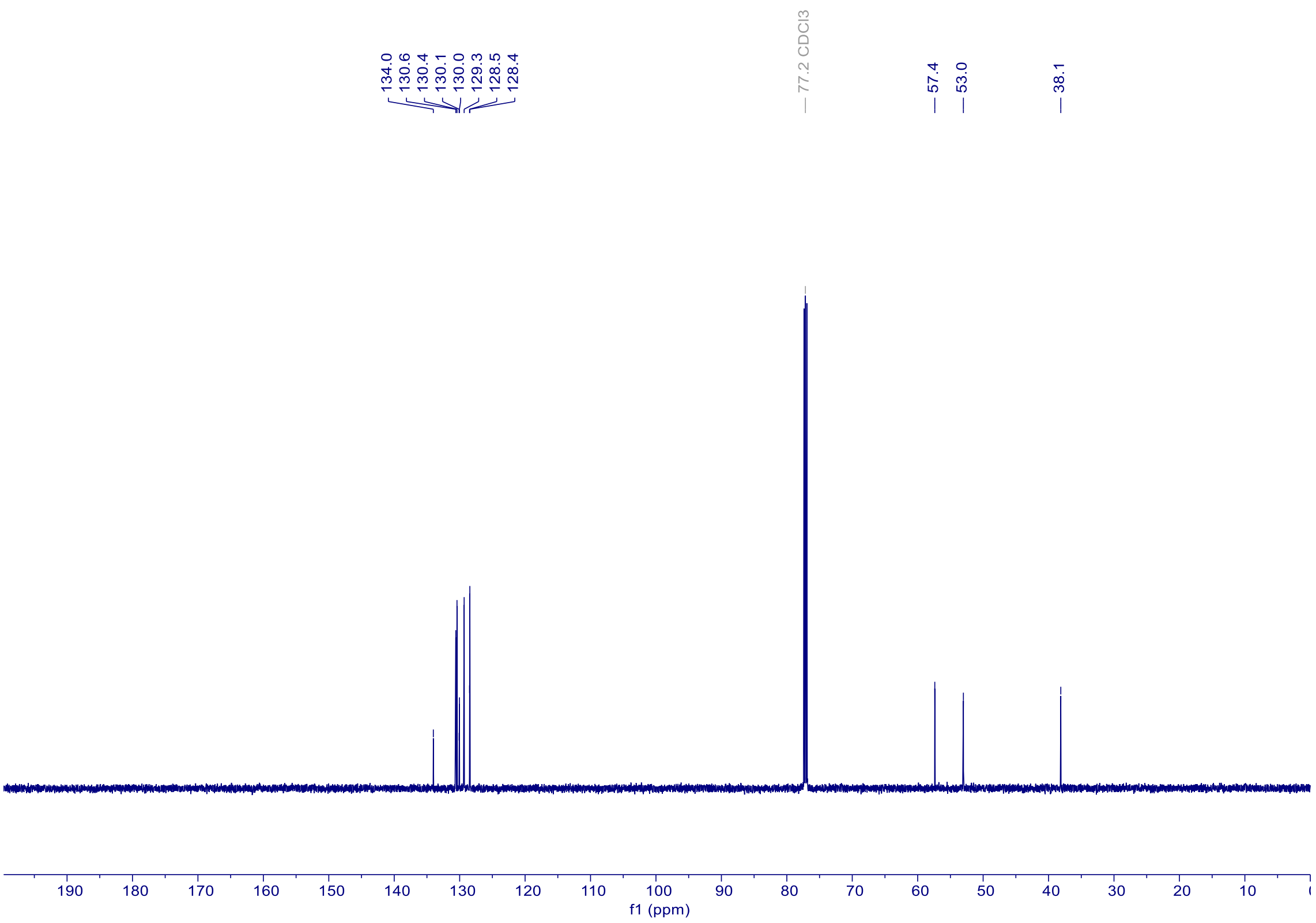

10a – ^{11}B NMR (193 MHz, $CDCl_3$)

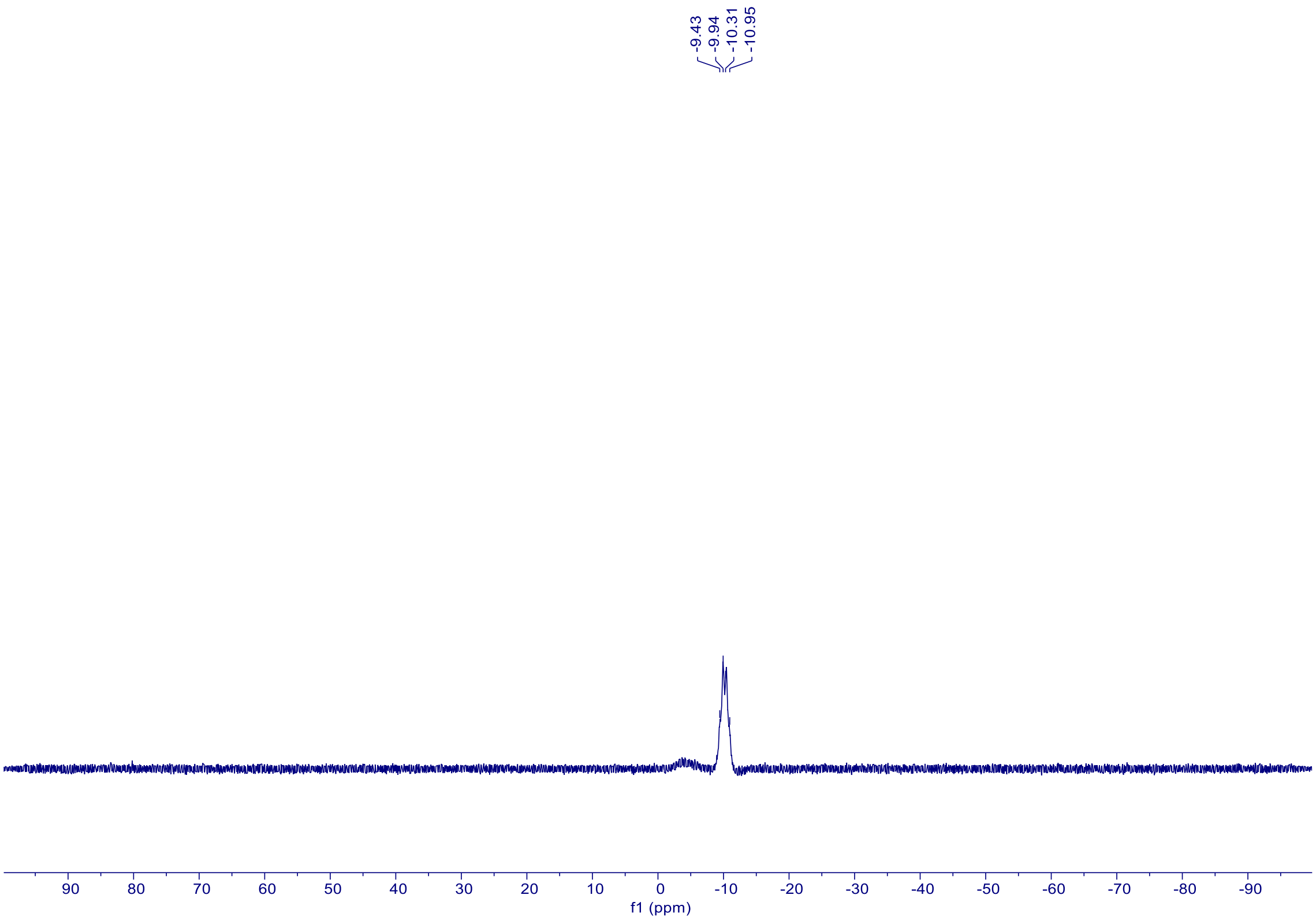

10a’ – ^{1}H NMR (600 MHz, $CDCl_3$)

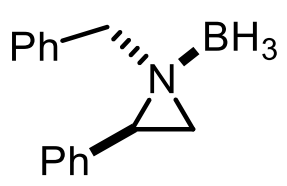

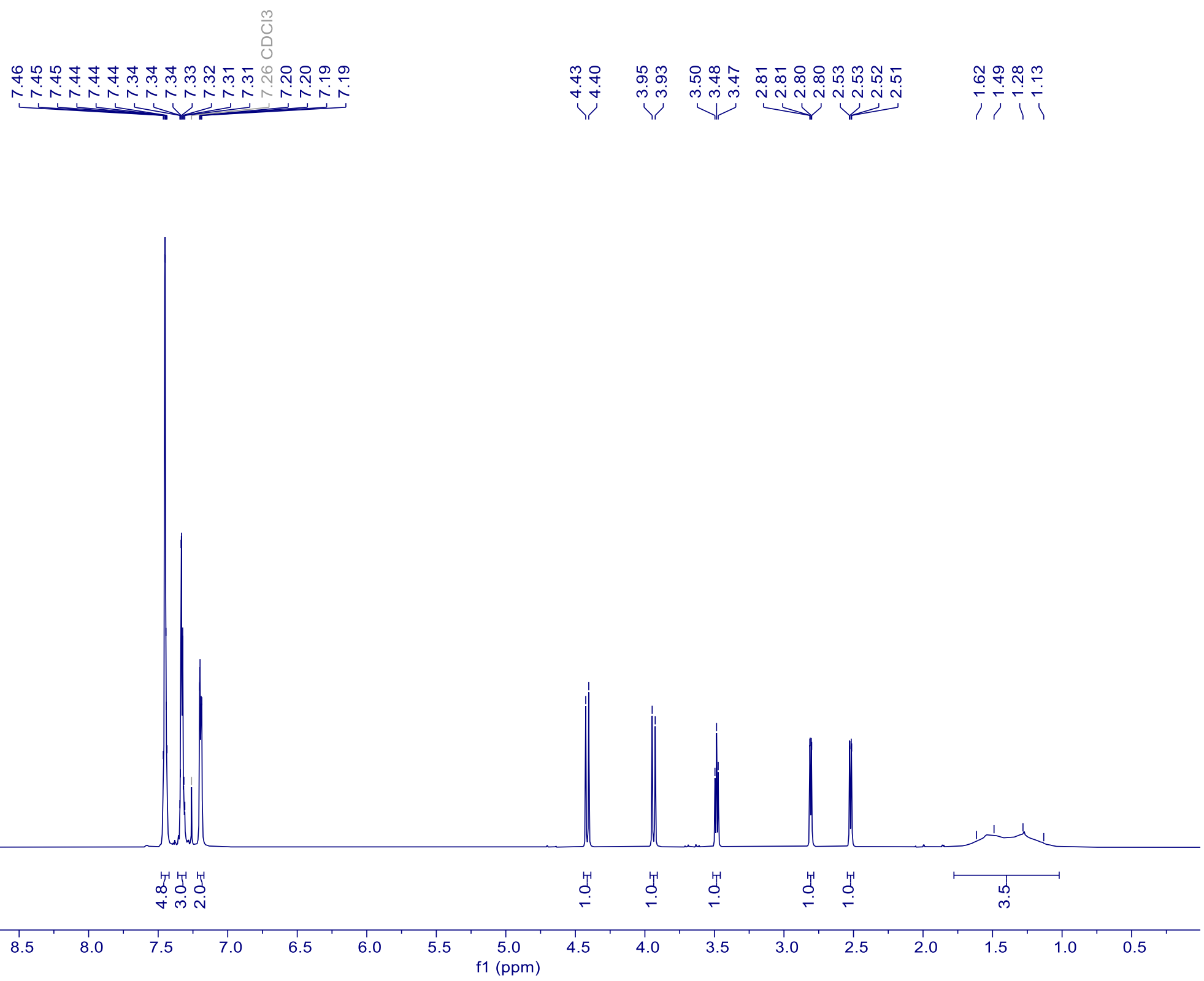

10a’ – ^{13}C NMR (151 MHz, $CDCl_3$)

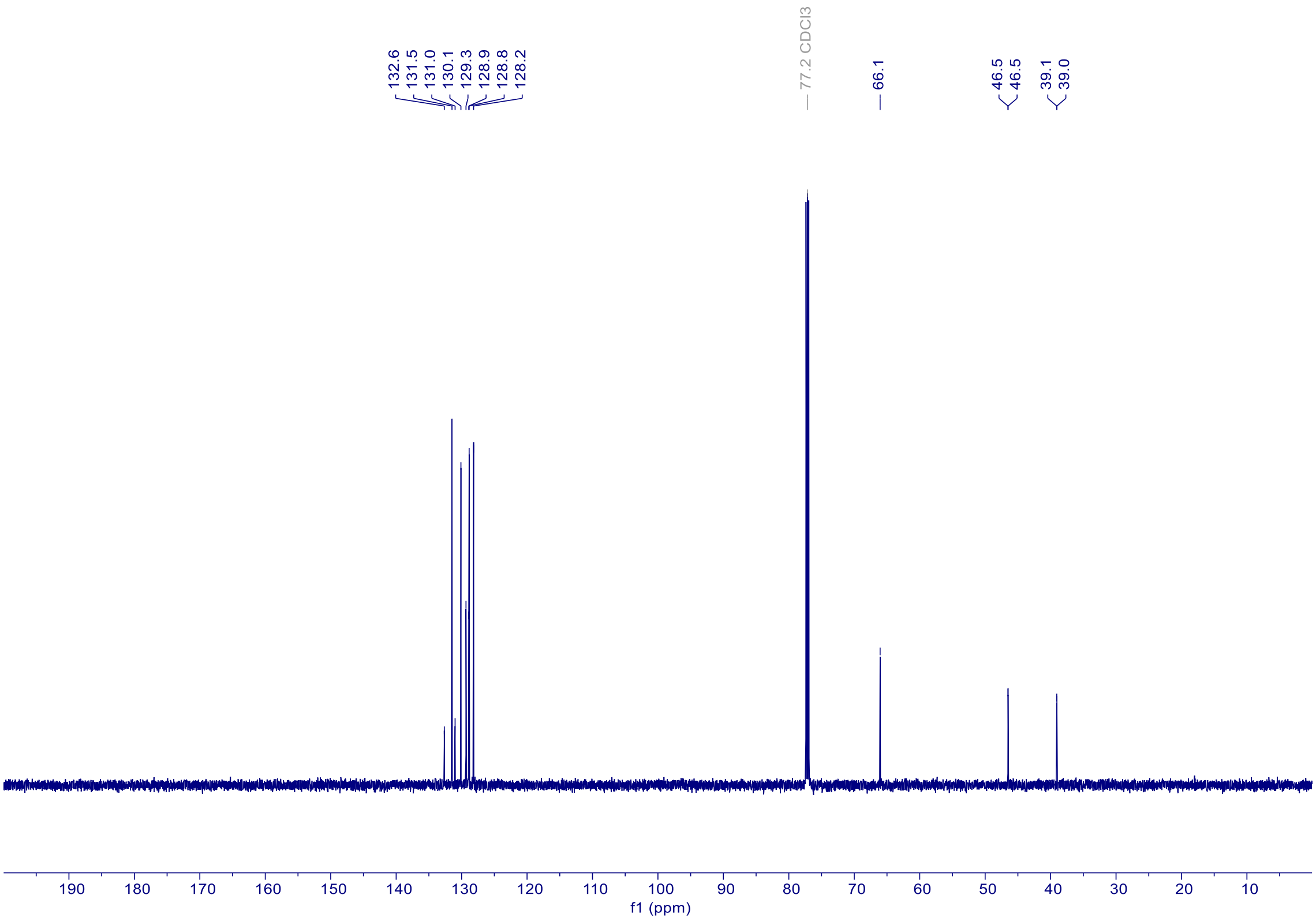

10a’ – ^{11}B NMR (193 MHz, $CDCl_3$)

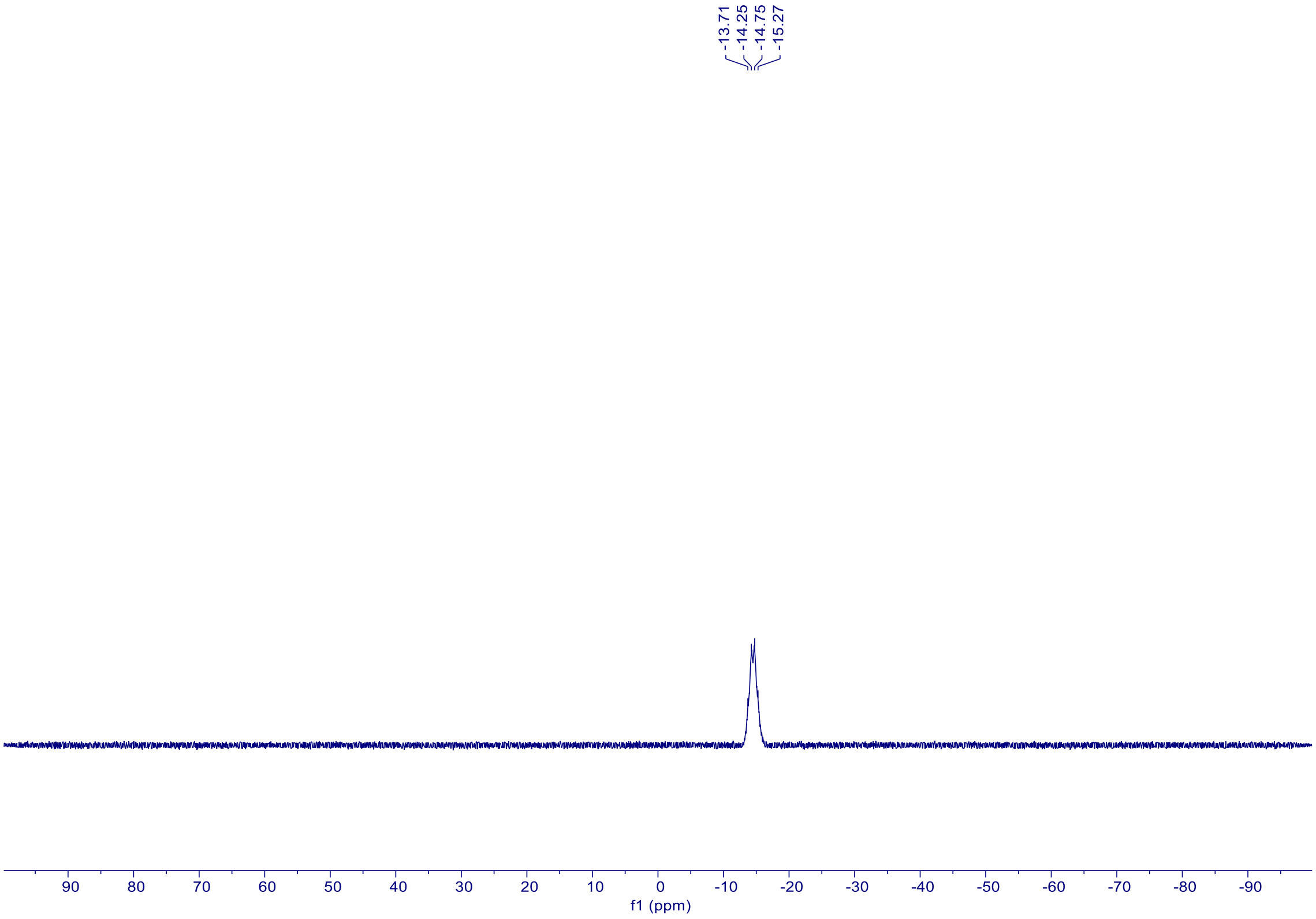

11a – ^{1}H NMR (600 MHz, $CDCl_3$)

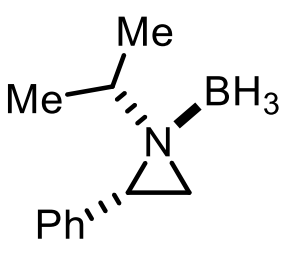

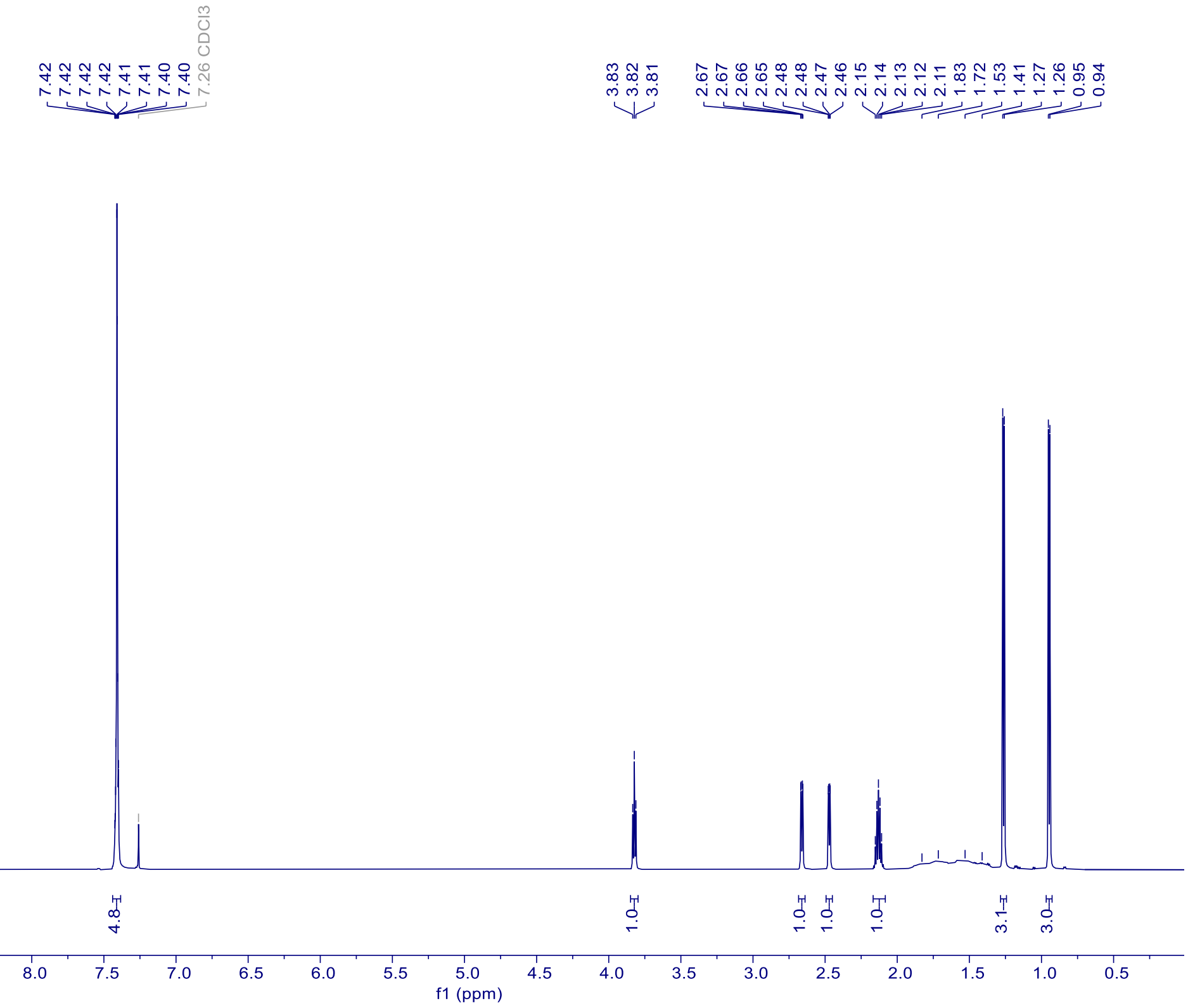

11a – ^{13}C NMR (151 MHz, $CDCl_3$)

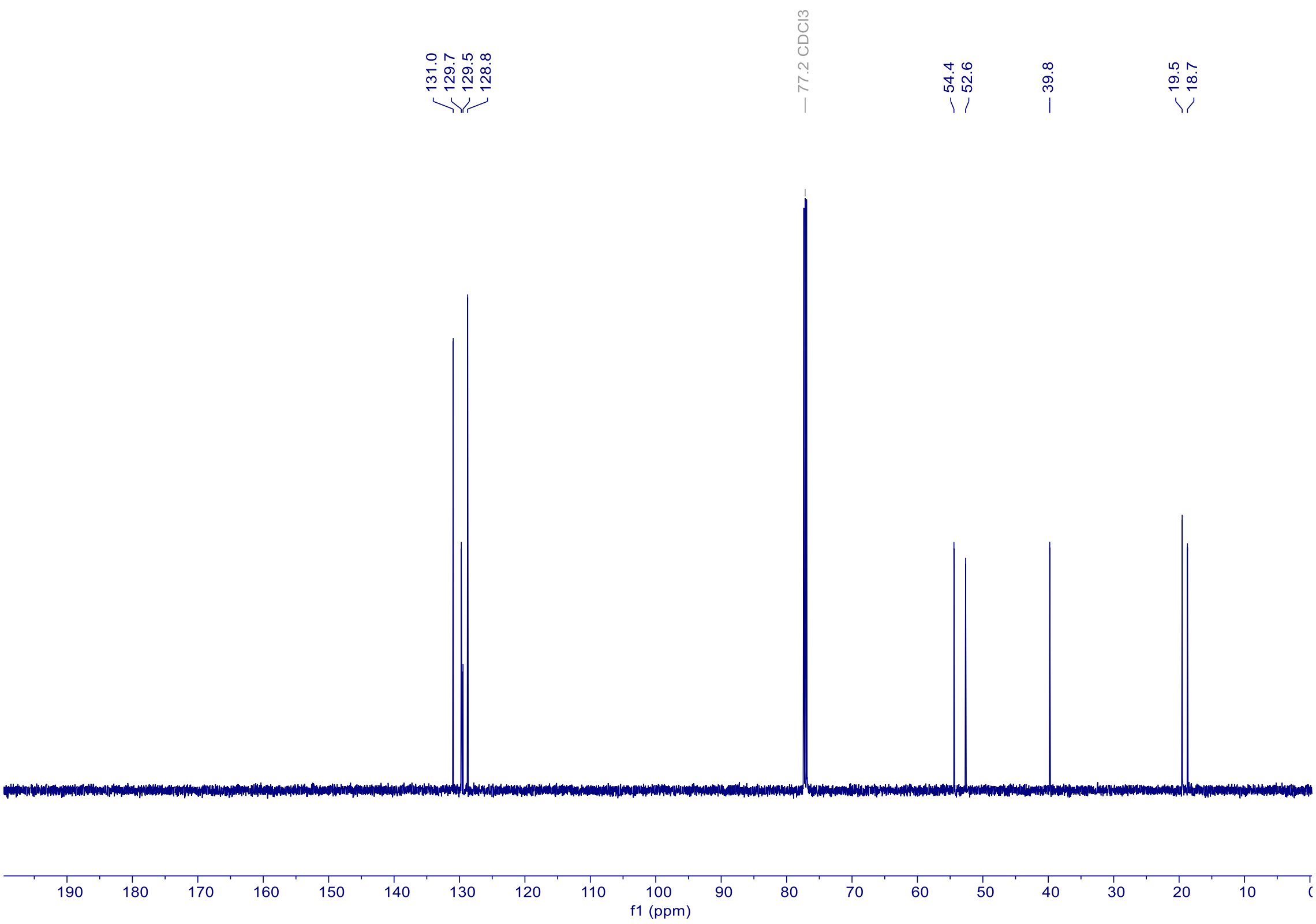

11a – ^{11}B NMR (193 MHz, $CDCl_3$)

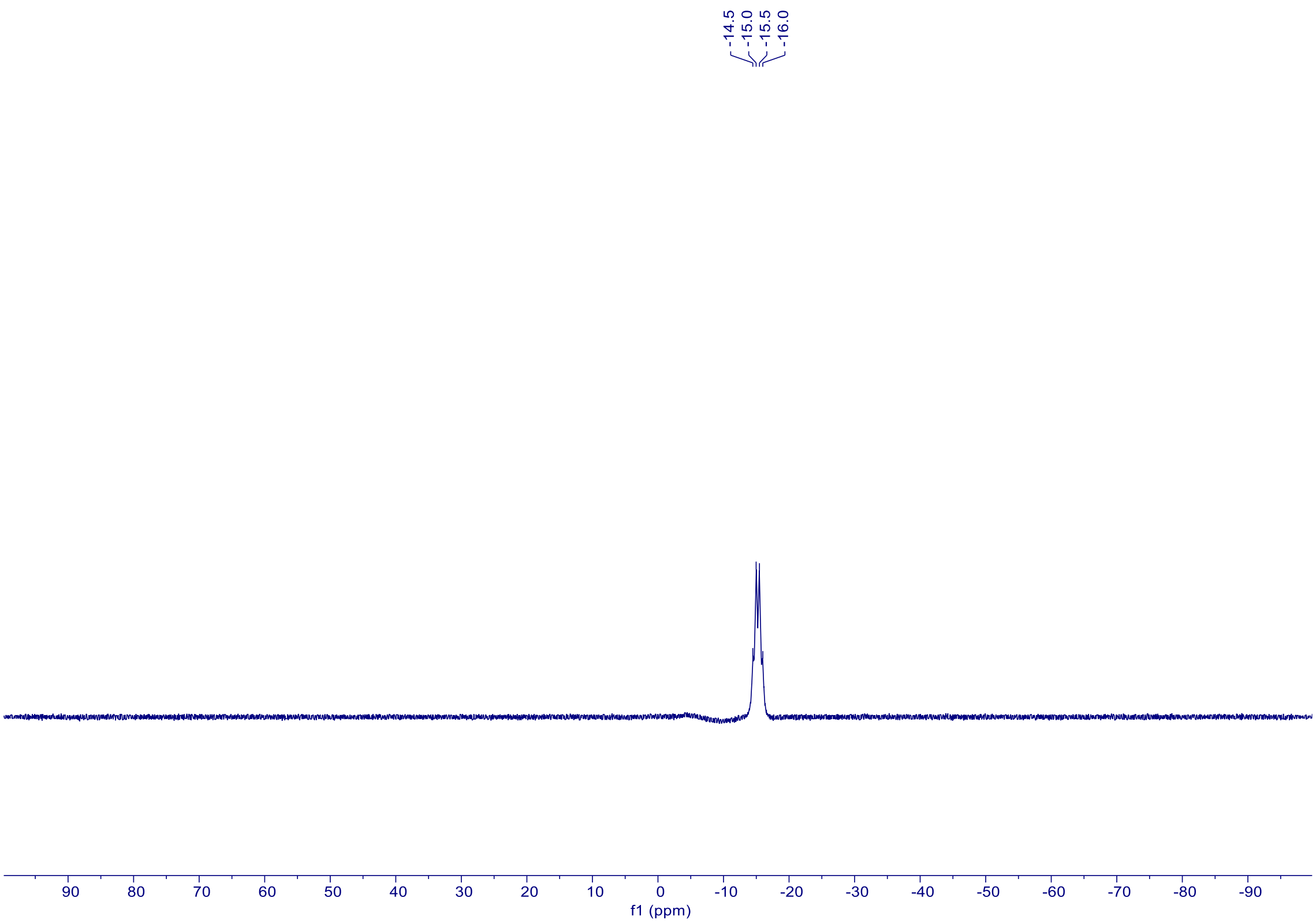

12a – ^{1}H NMR (600 MHz, $CDCl_3$)

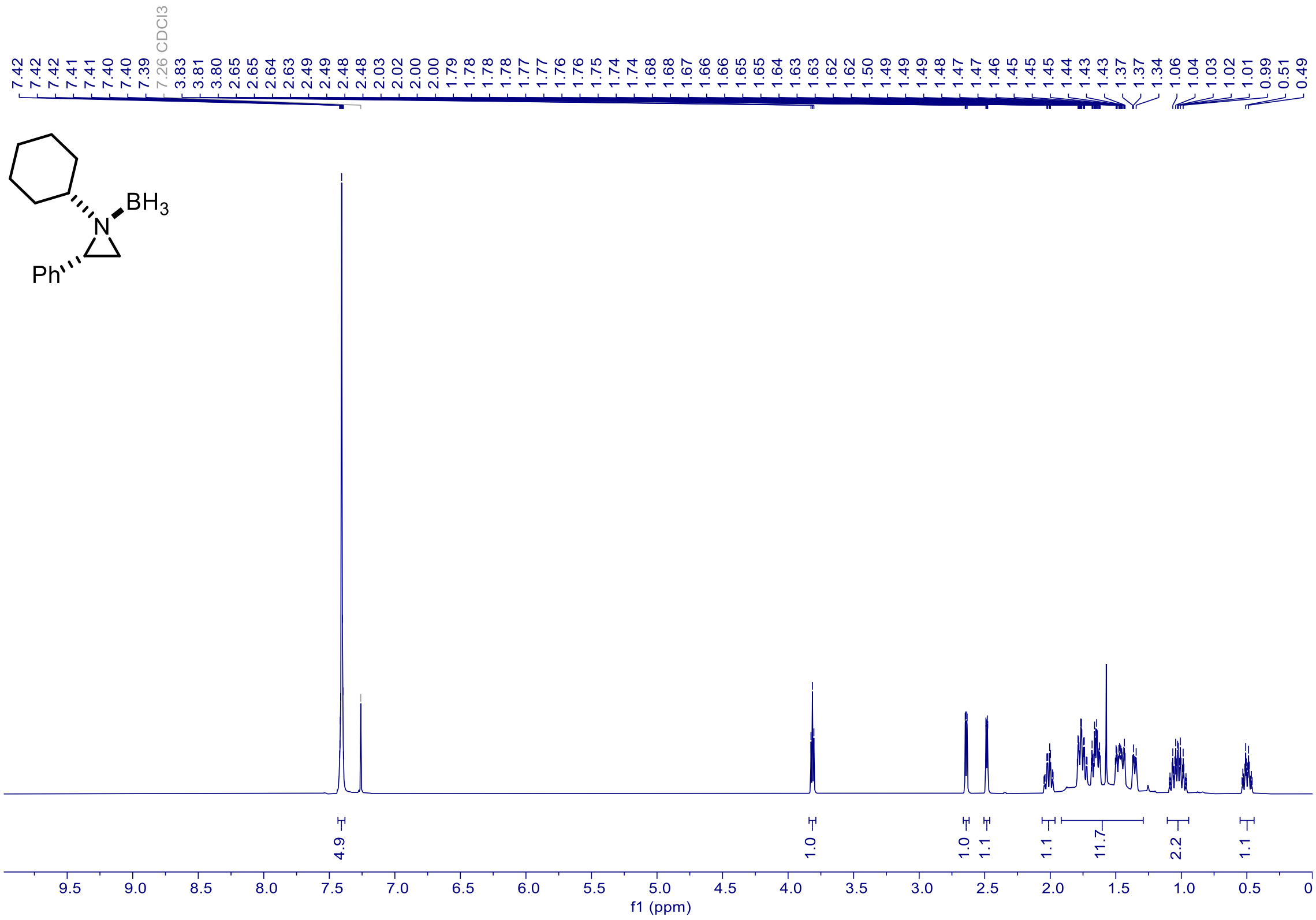

12a – ^{13}C NMR (151 MHz, $CDCl_3$)

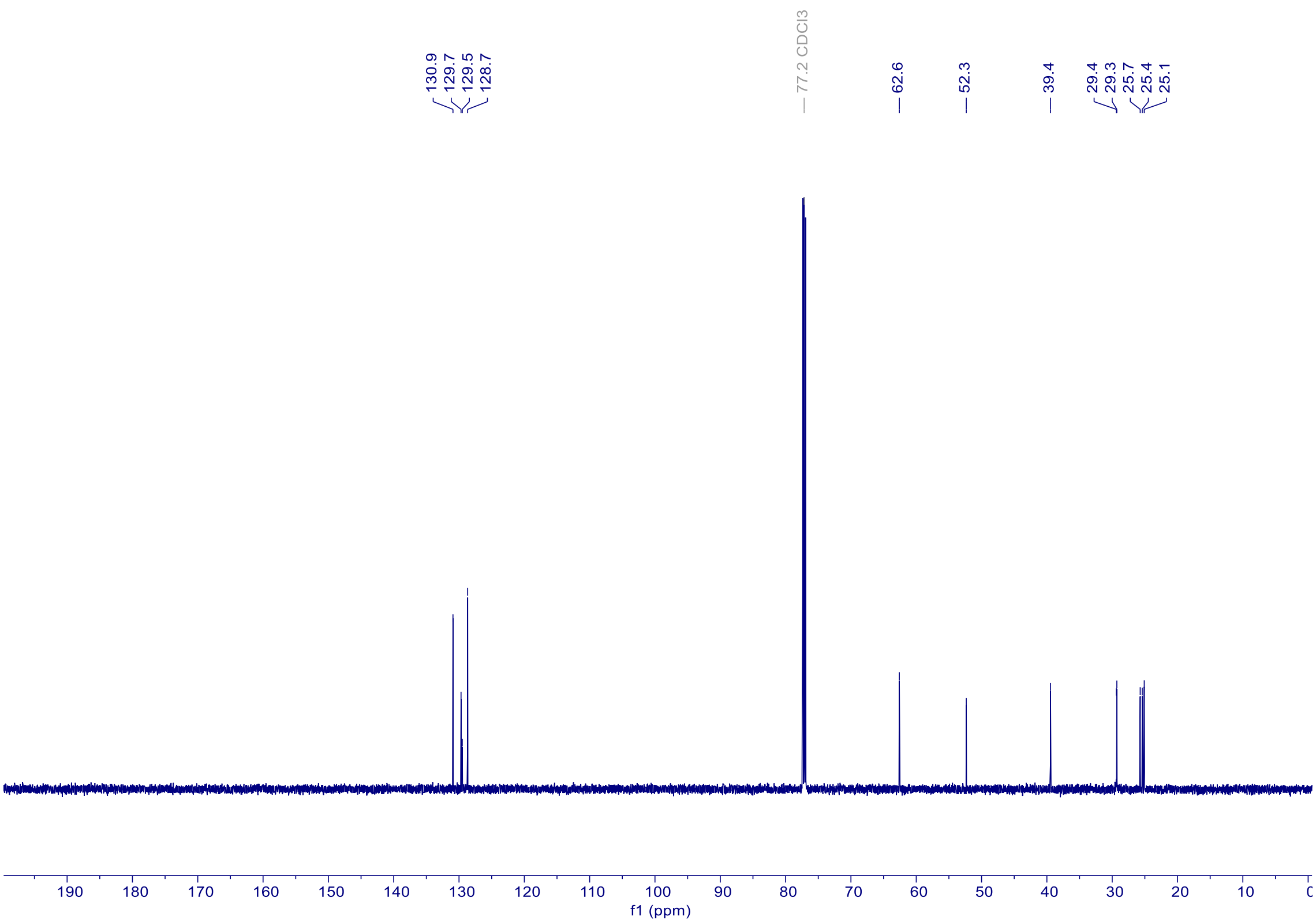

12a – ^{11}B NMR (193 MHz, $CDCl_3$)

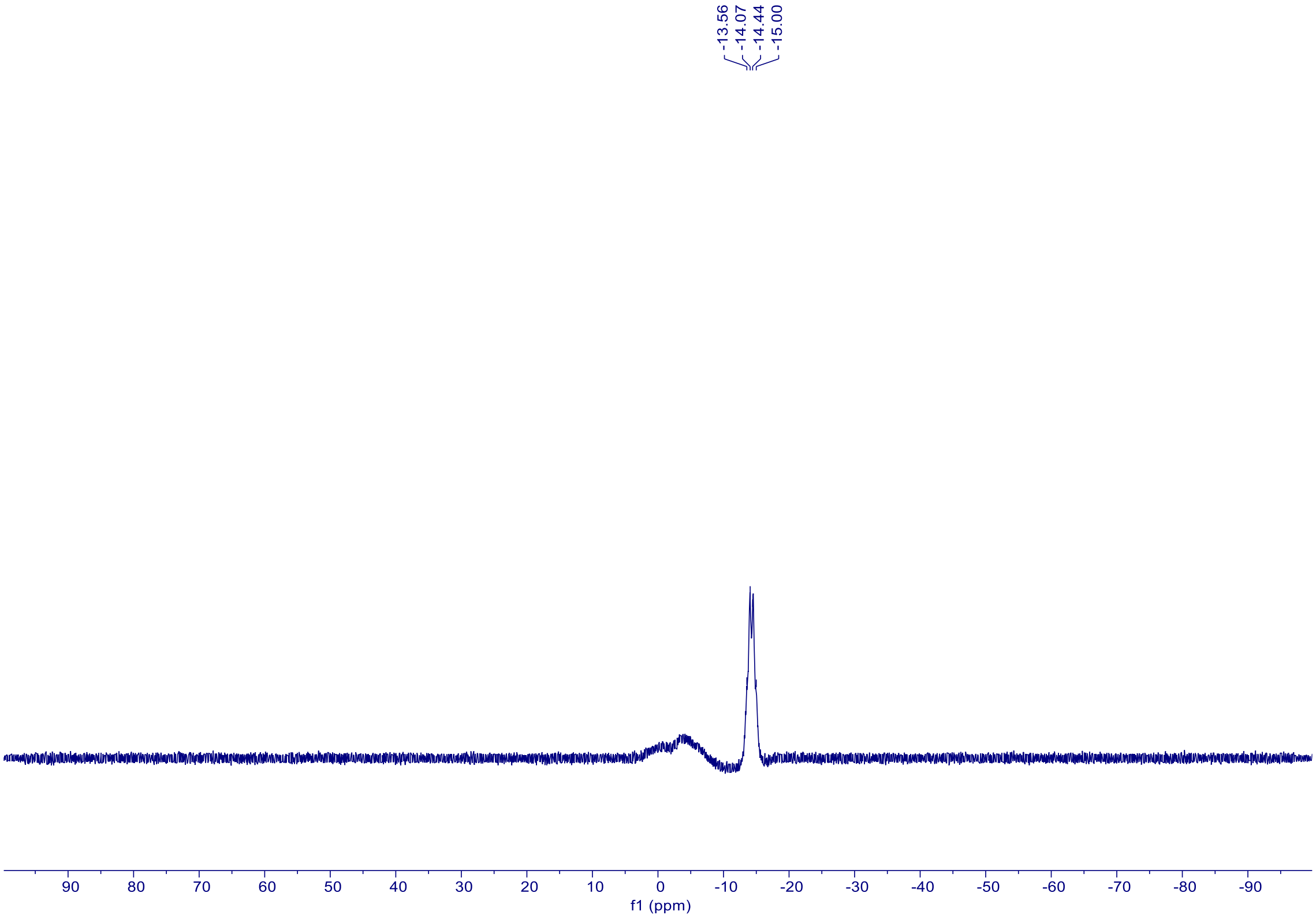

12a' – ^{1}H NMR (600 MHz, $CDCl_3$)

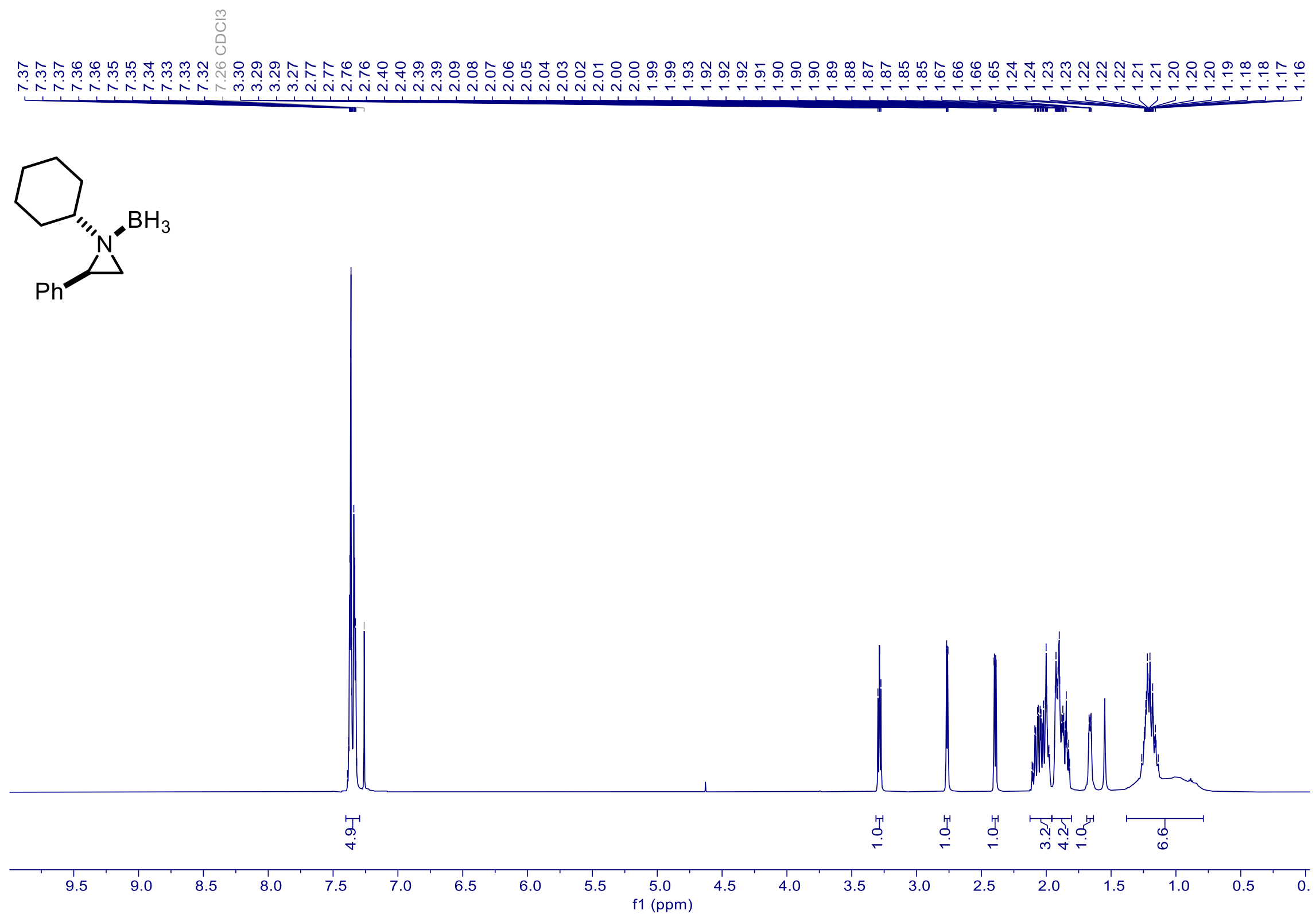

12a' – ^{13}C NMR (151 MHz, $CDCl_3$)

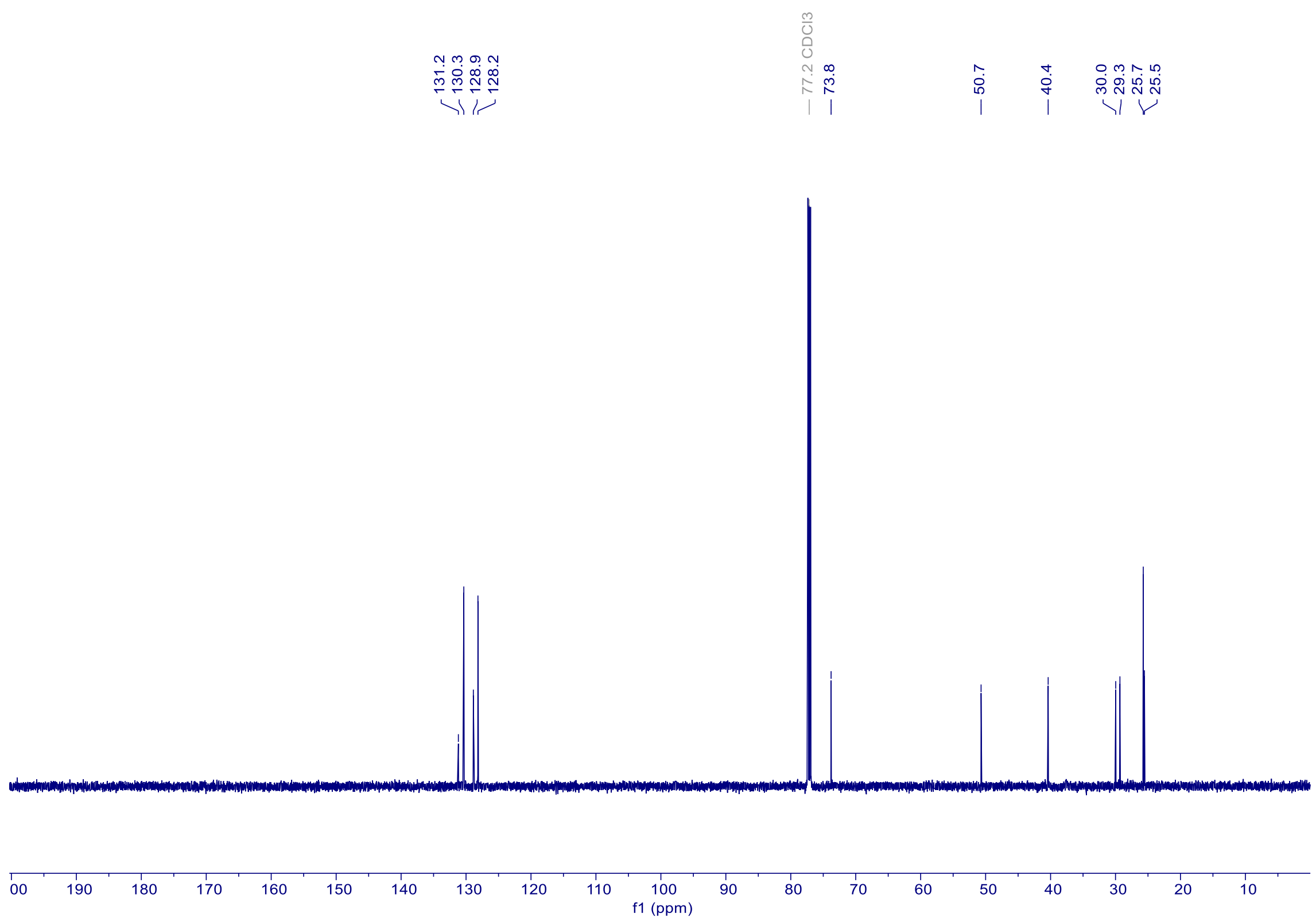

12a' – ^{11}B NMR (193 MHz, $CDCl_3$)

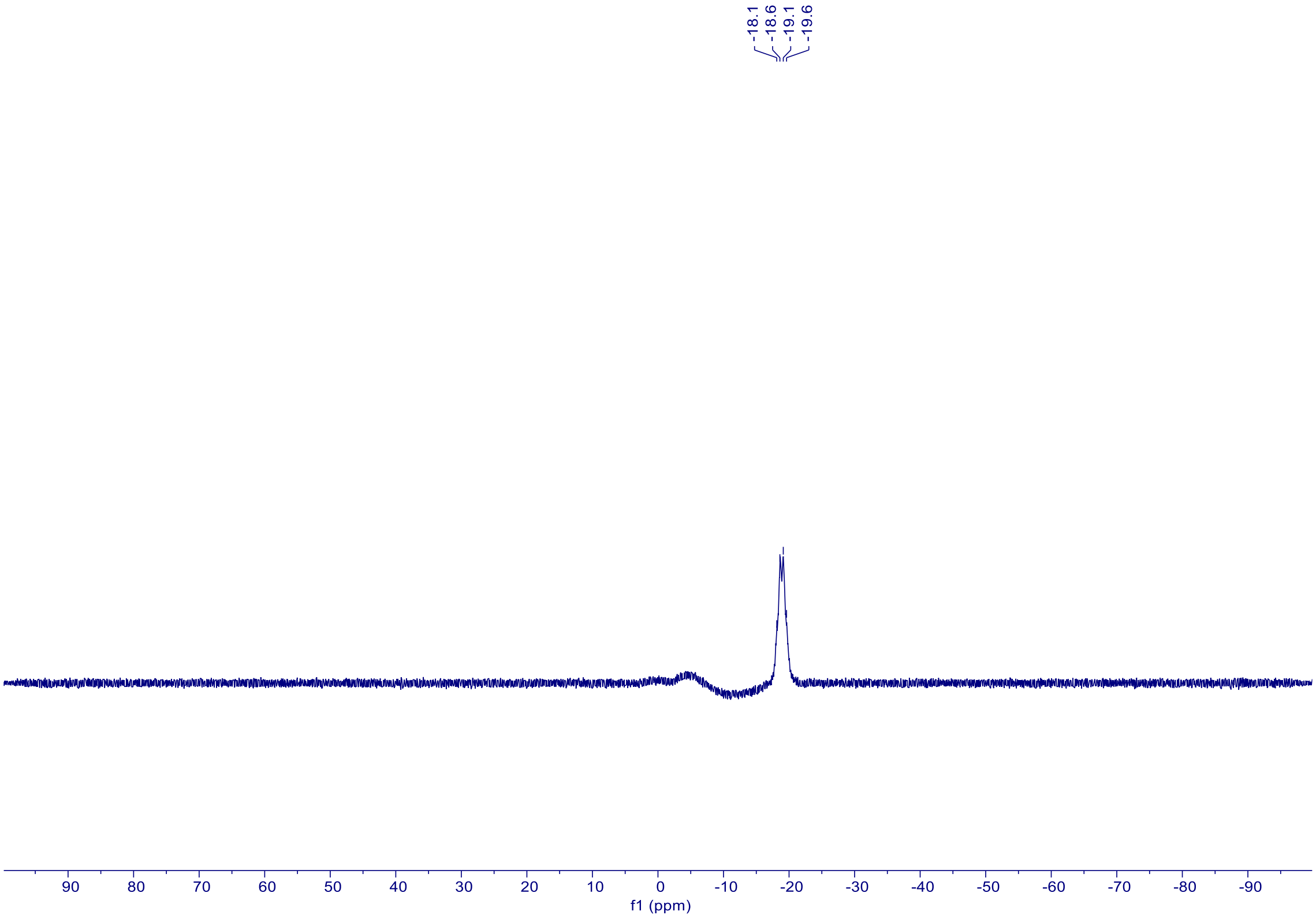

13a – ^{1}H NMR (600 MHz, $CDCl_3$)

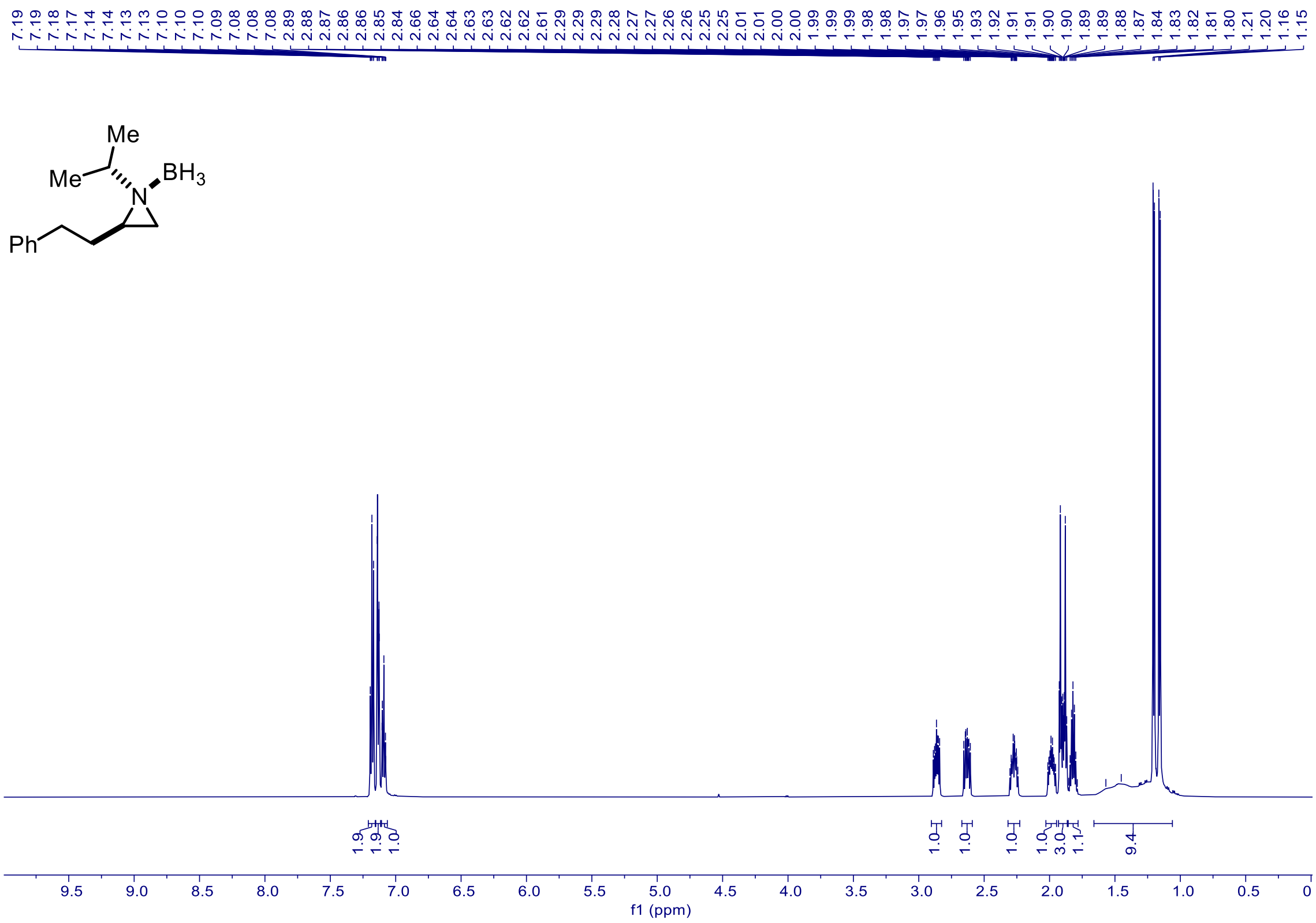

13a – ^{13}C NMR (151 MHz, $CDCl_3$)

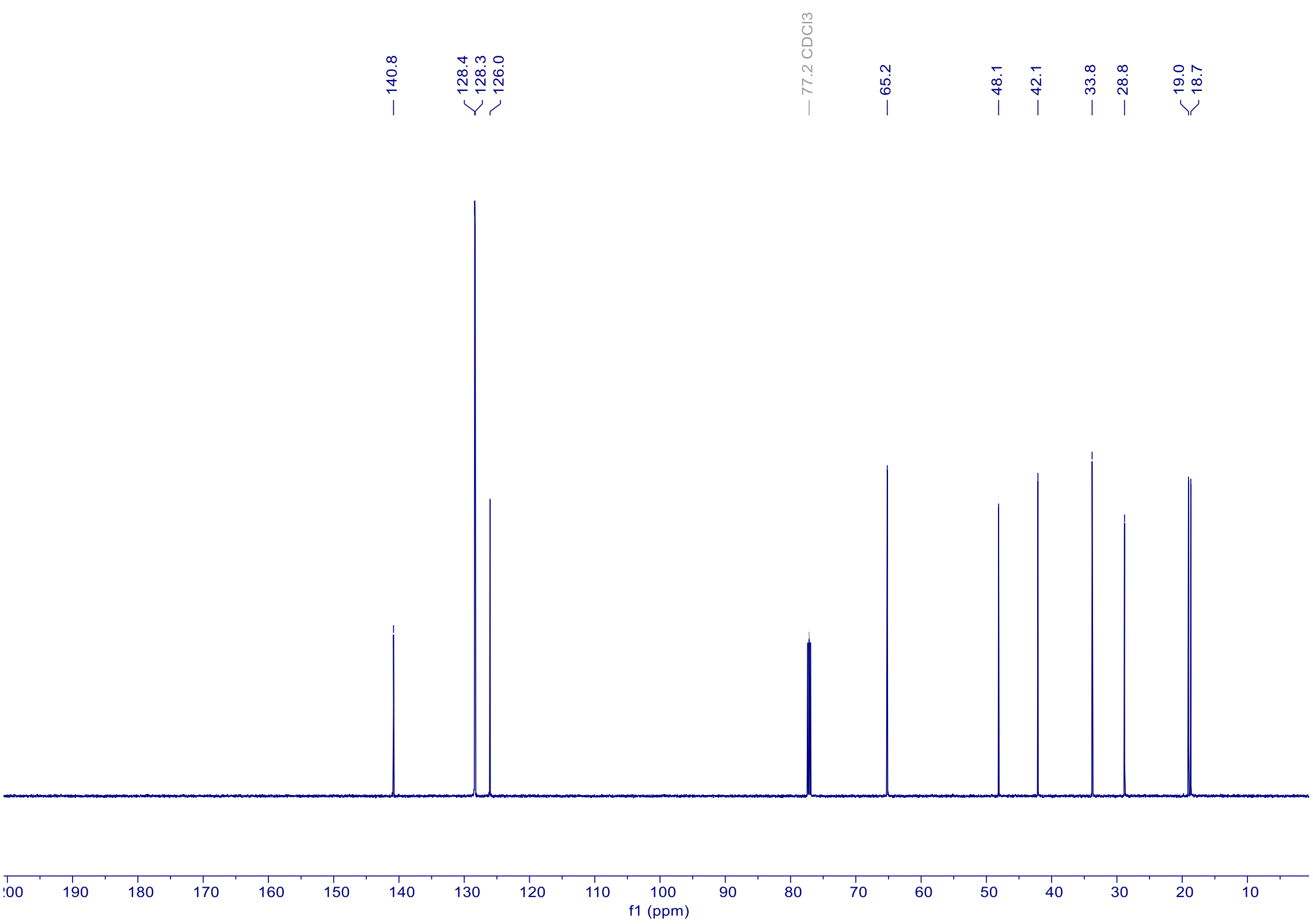

13a – ^{11}B NMR (193 MHz, $CDCl_3$)

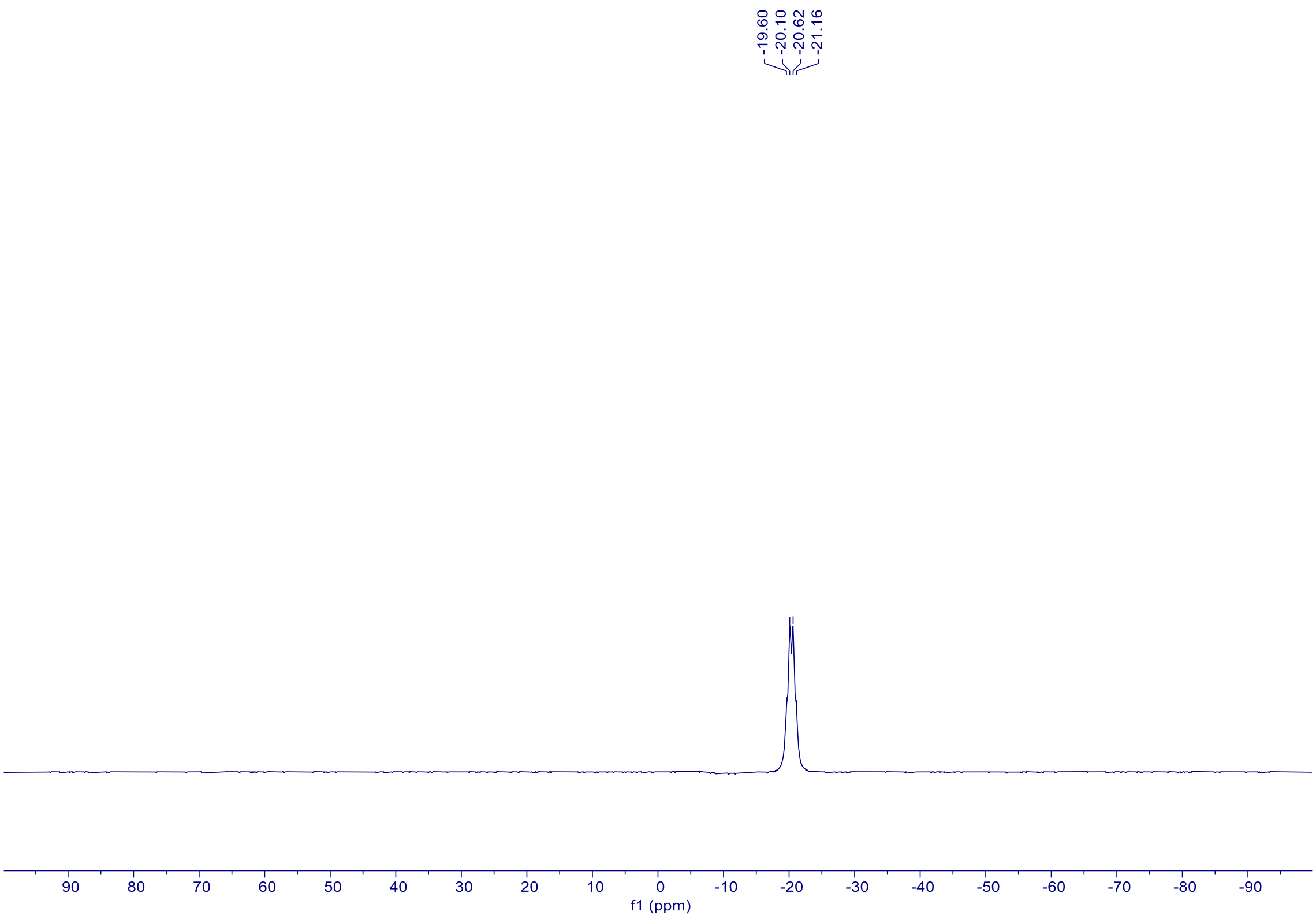

14a – ^{1}H NMR (600 MHz, $CDCl_3$)

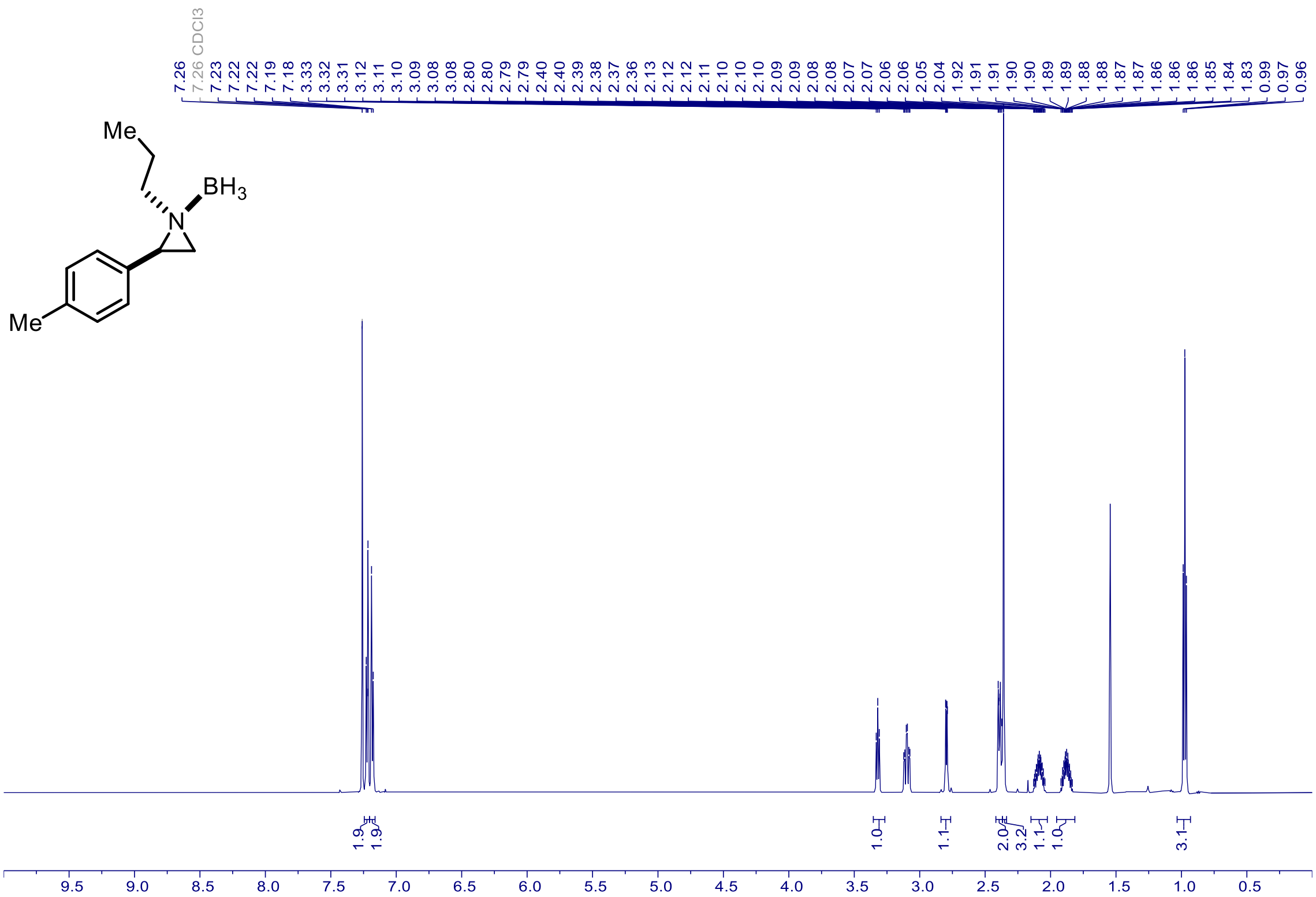

14a – ^{13}C NMR (151 MHz, $CDCl_3$)

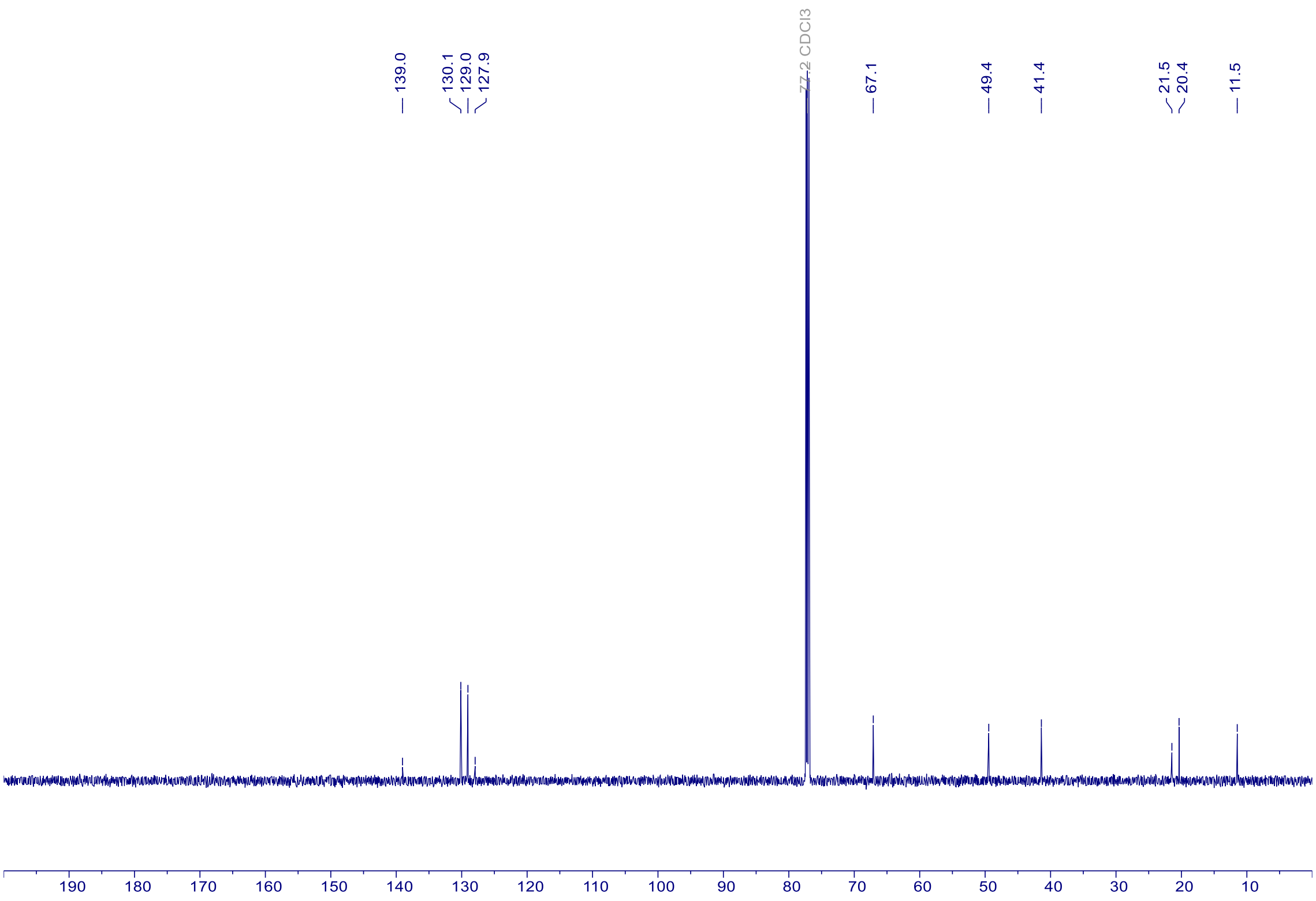

14a – ^{11}B NMR (192 MHz, $CDCl_3$)

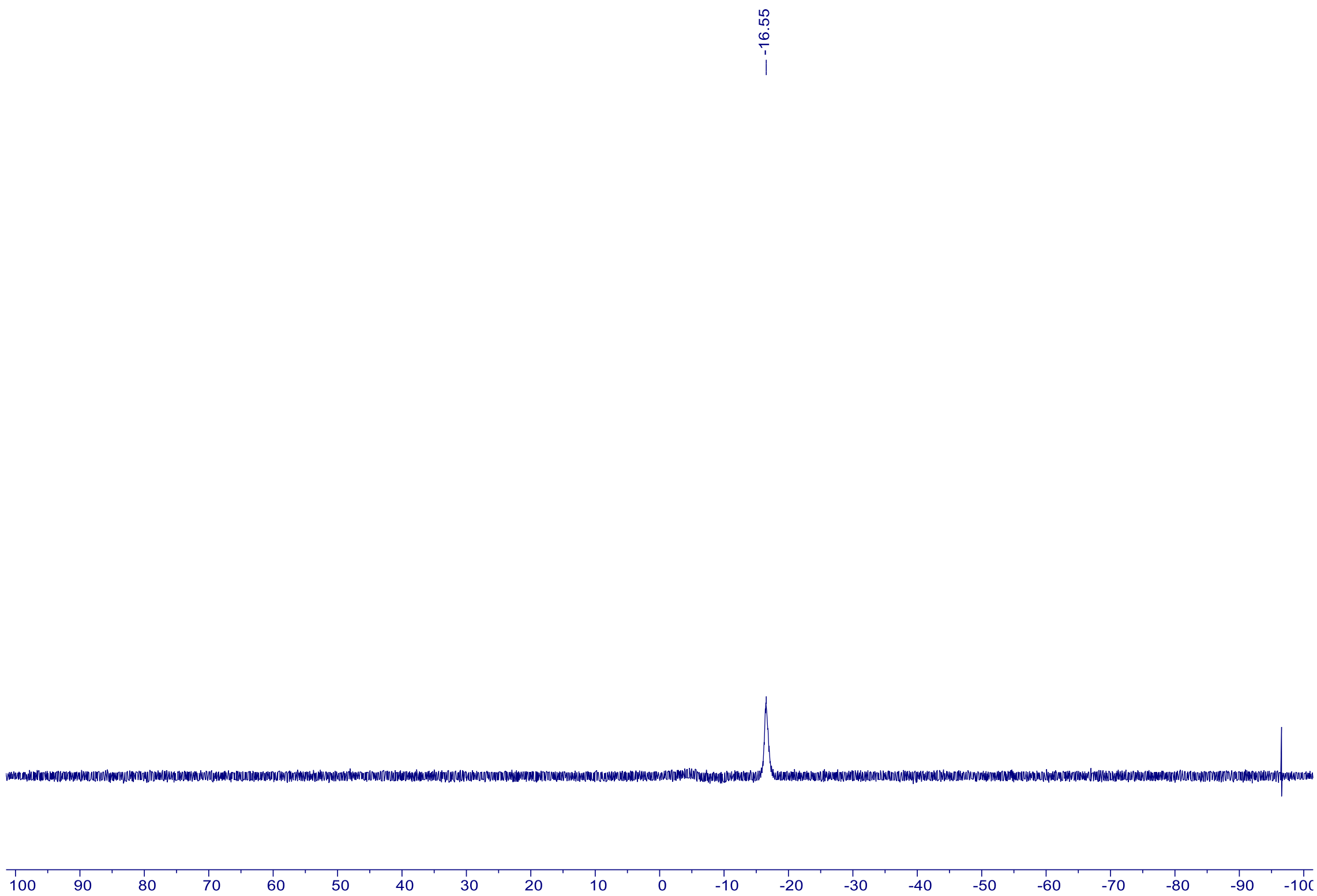

15a and 15a' – ^{1}H NMR (600 MHz, CDCl3)

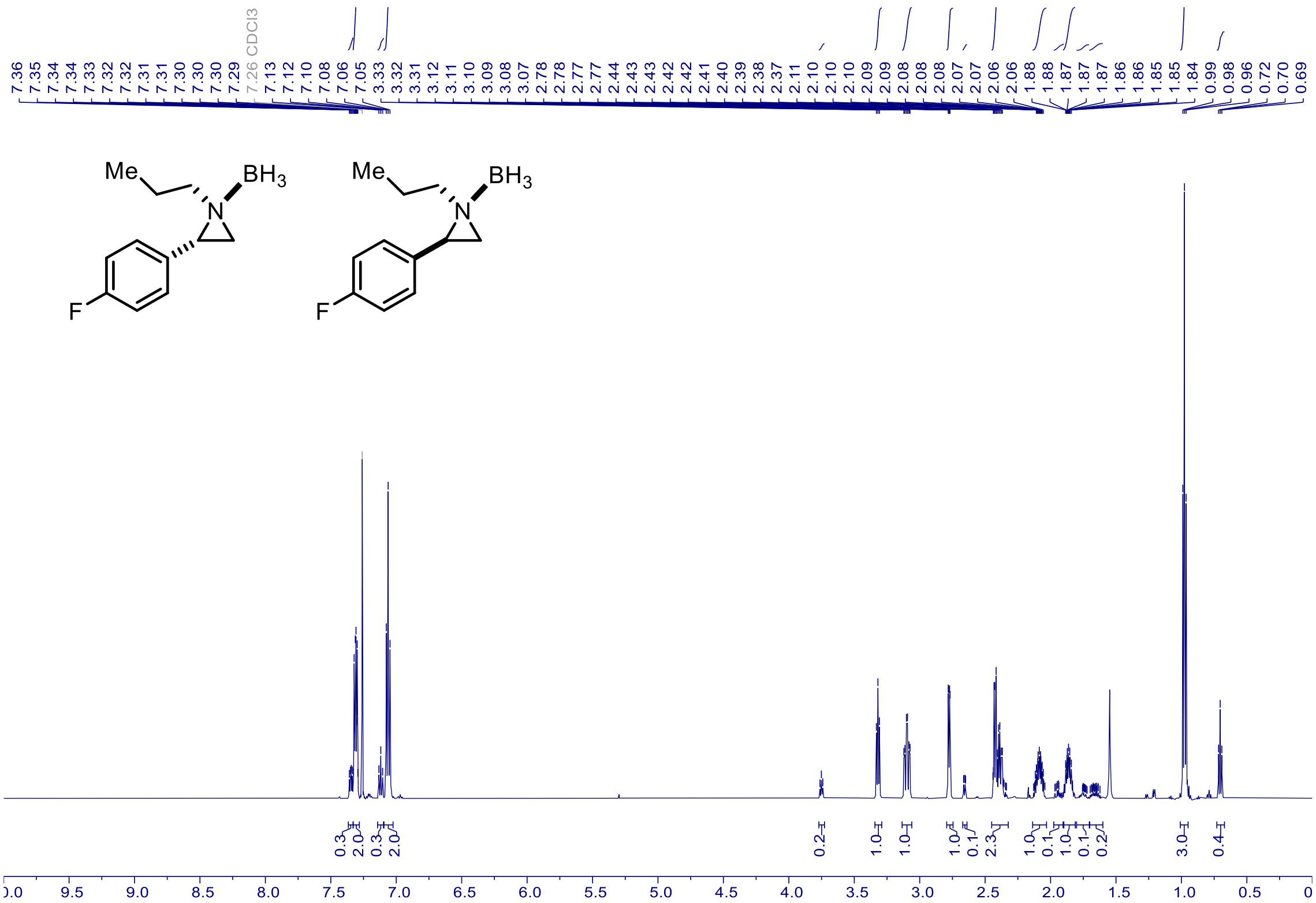

15a and 15a' – ^{13}C NMR (151 MHz, CDCl3)

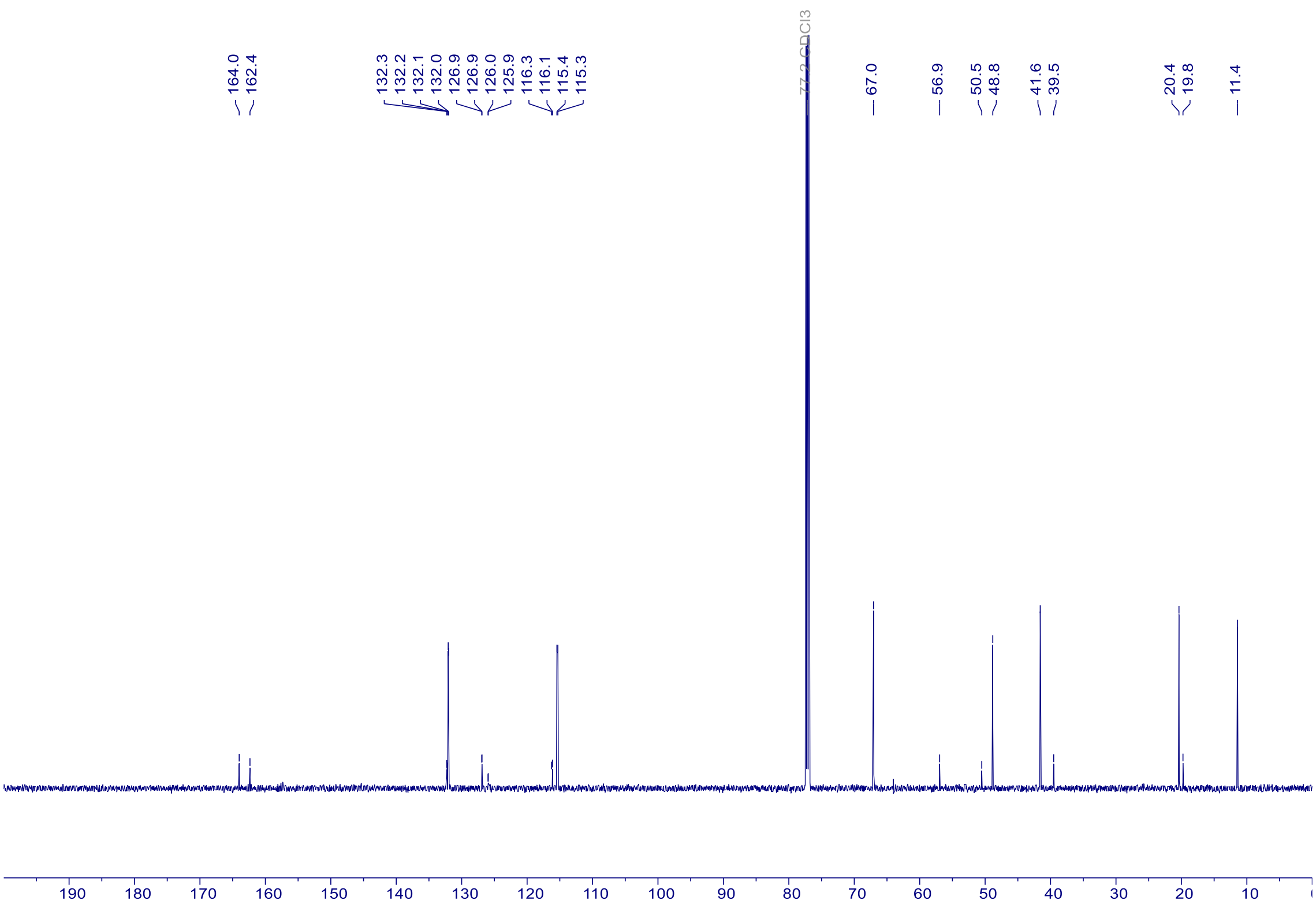

15a and 15a' – ^{11}B NMR (192 MHz, $CDCl_3$)

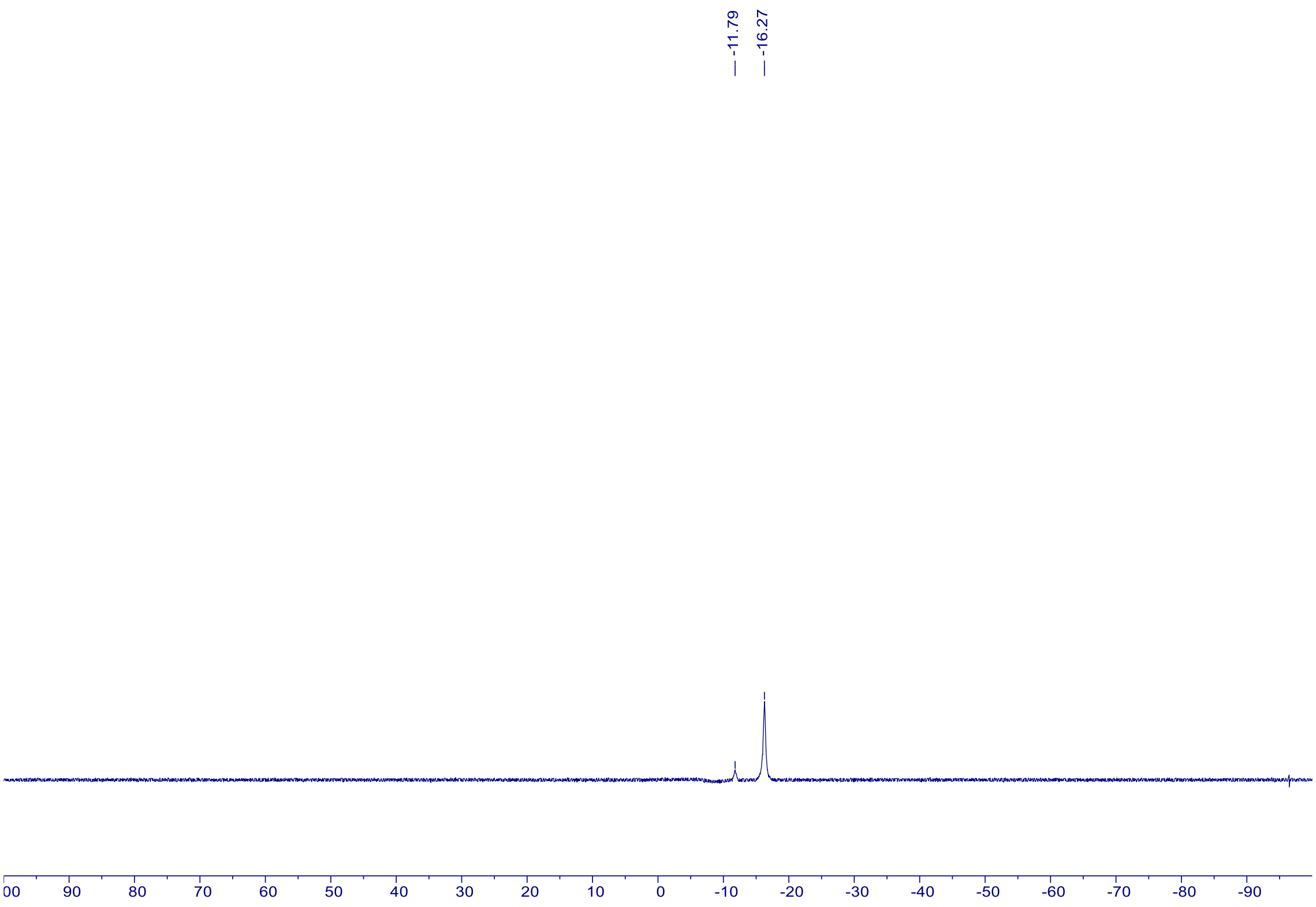

15a and 15a' – ^{19}F NMR (564 MHz, $CDCl_3$)

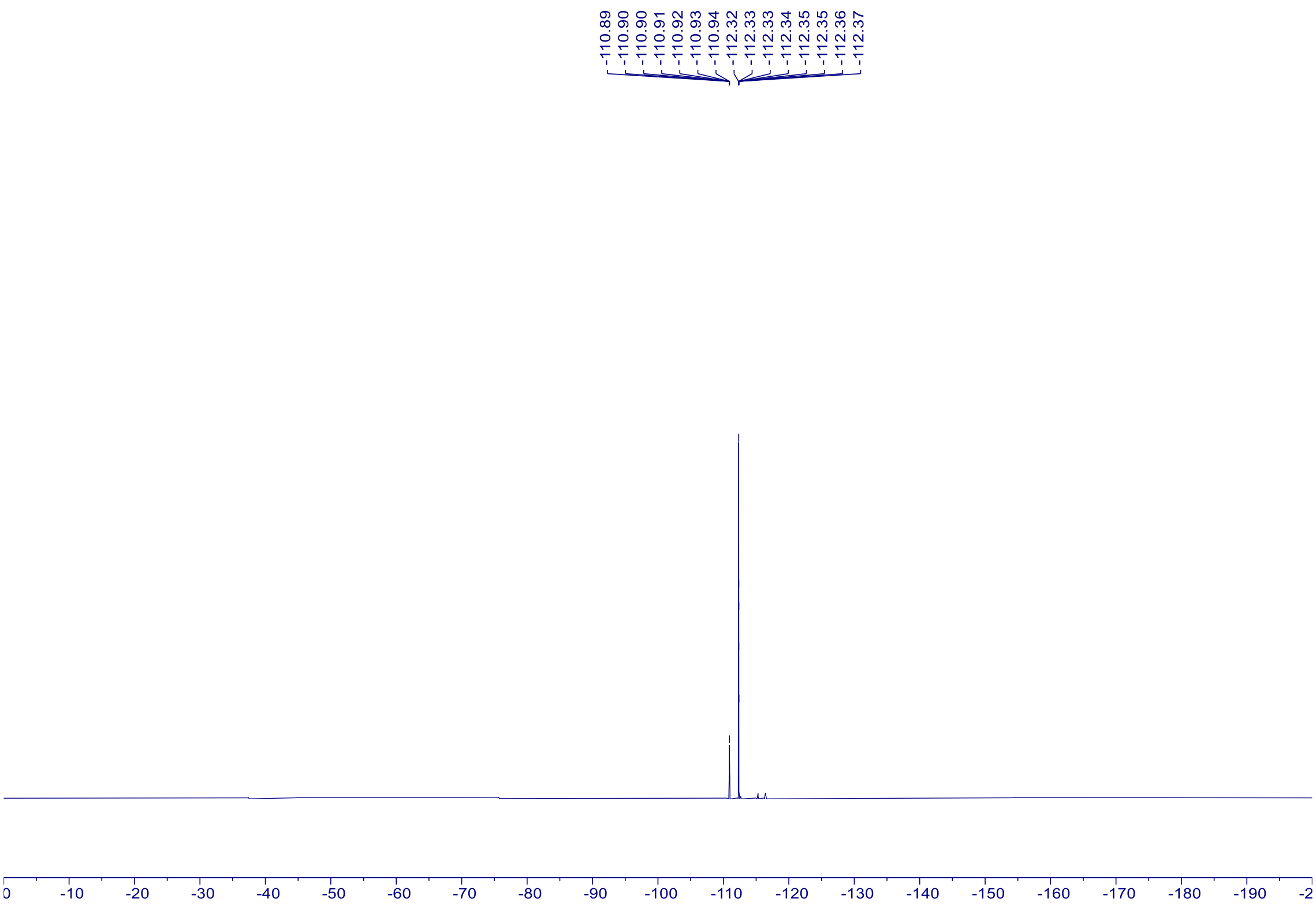

16a – ^{1}H NMR (400 MHz, $CDCl_3$)

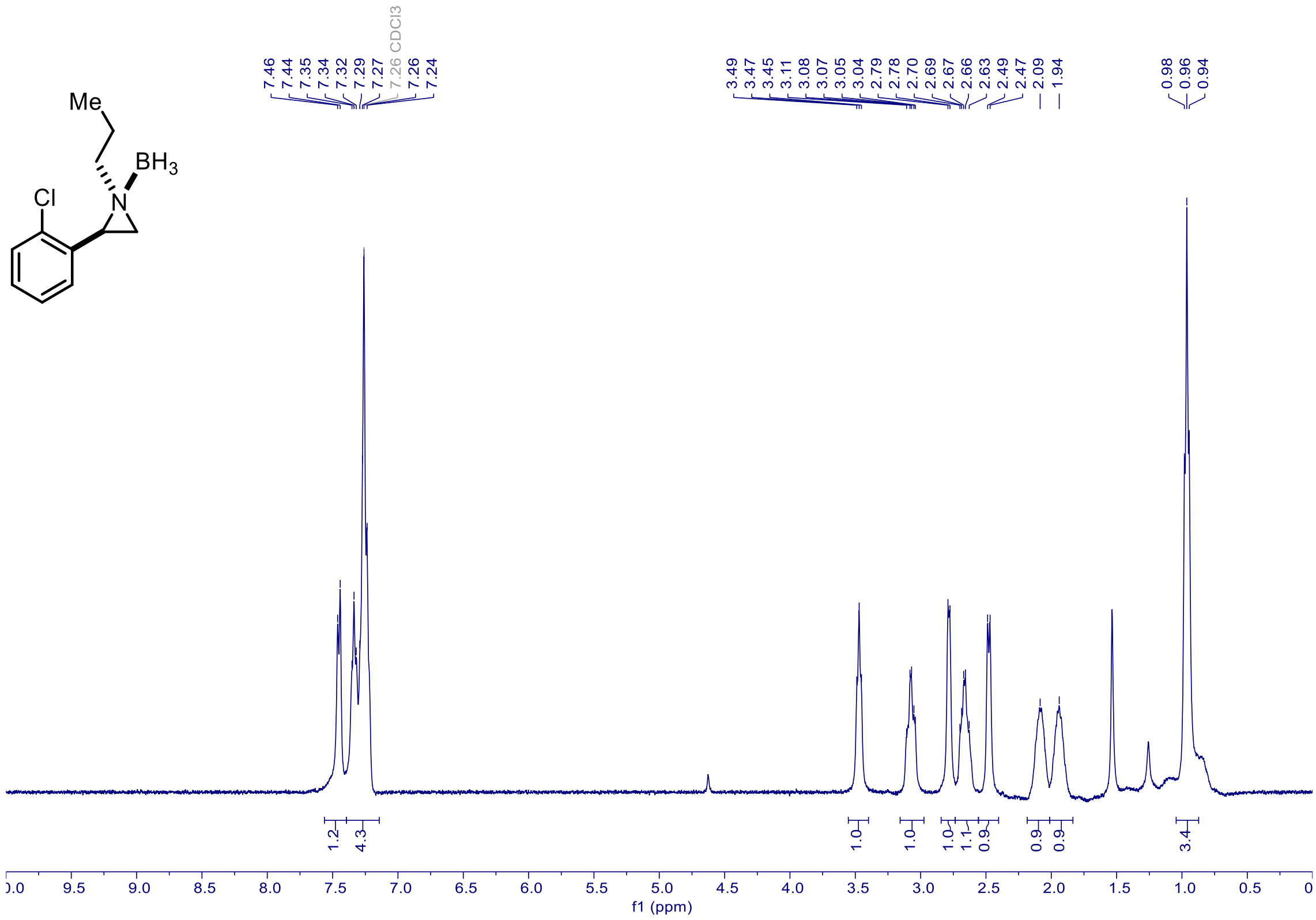

16a – ^{13}C NMR (101 MHz, $CDCl_3$)

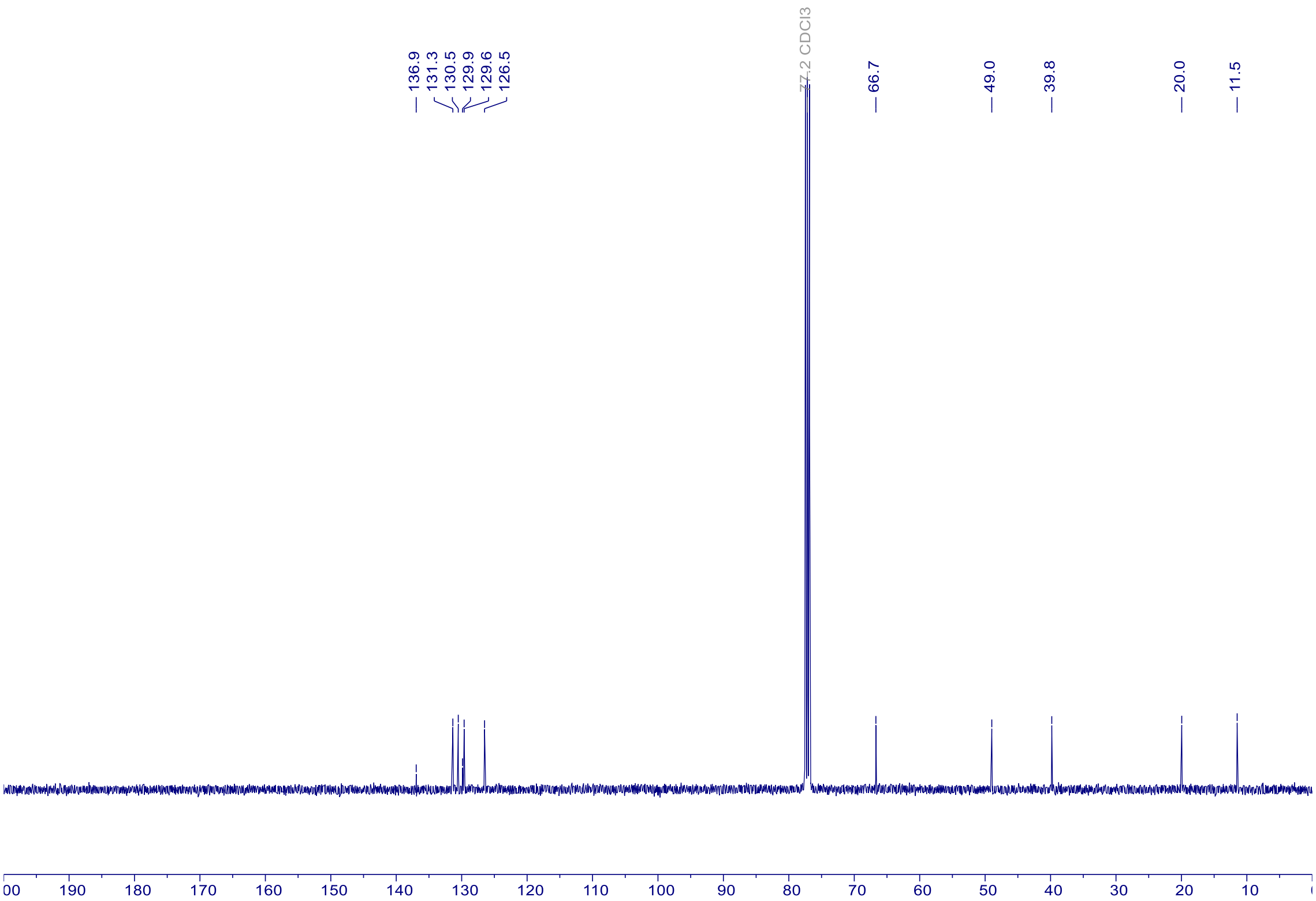

16a – ^{11}B NMR (128 MHz, $CDCl_3$)

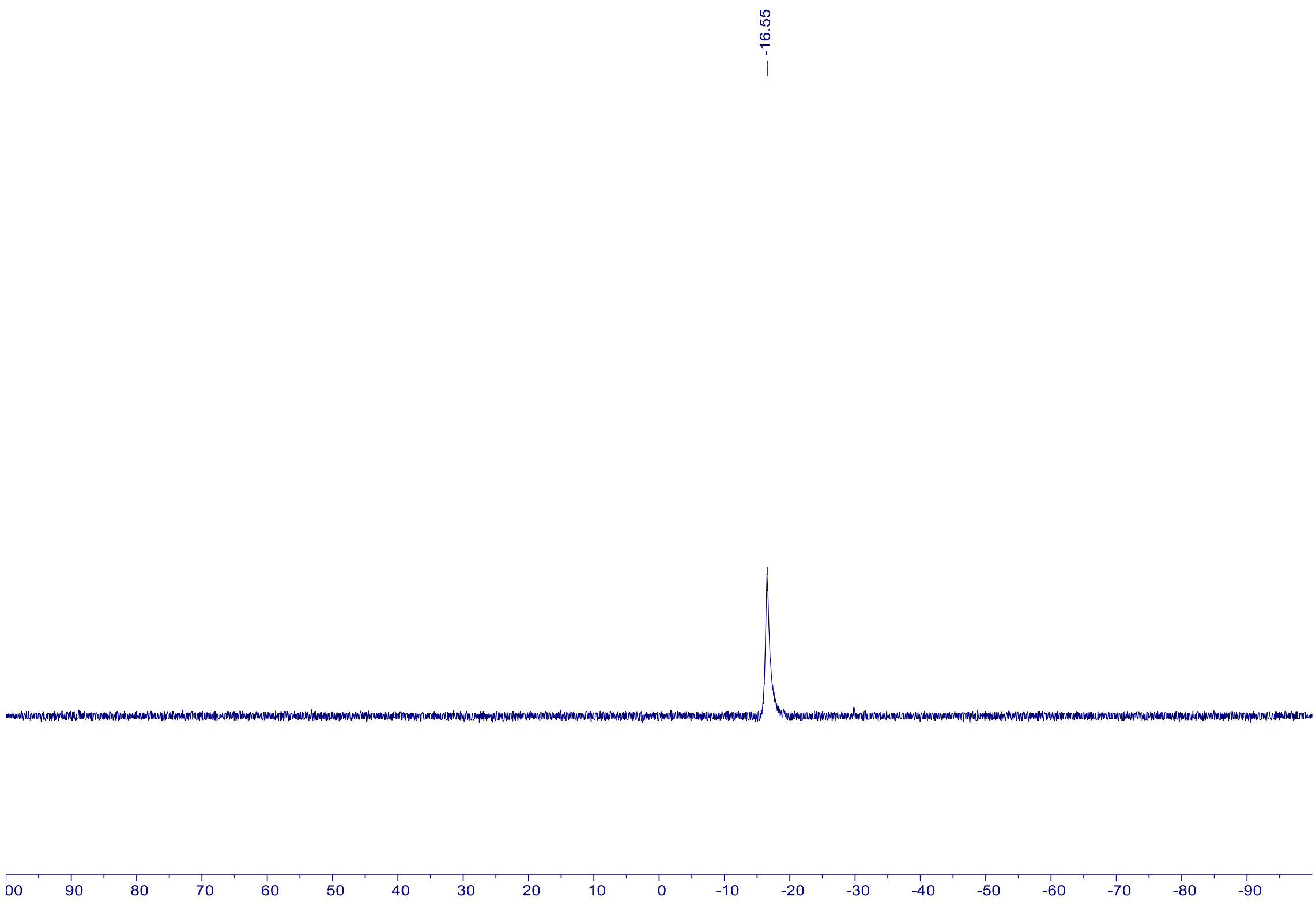

17a – ^{1}H NMR (600 MHz, $CDCl_3$)

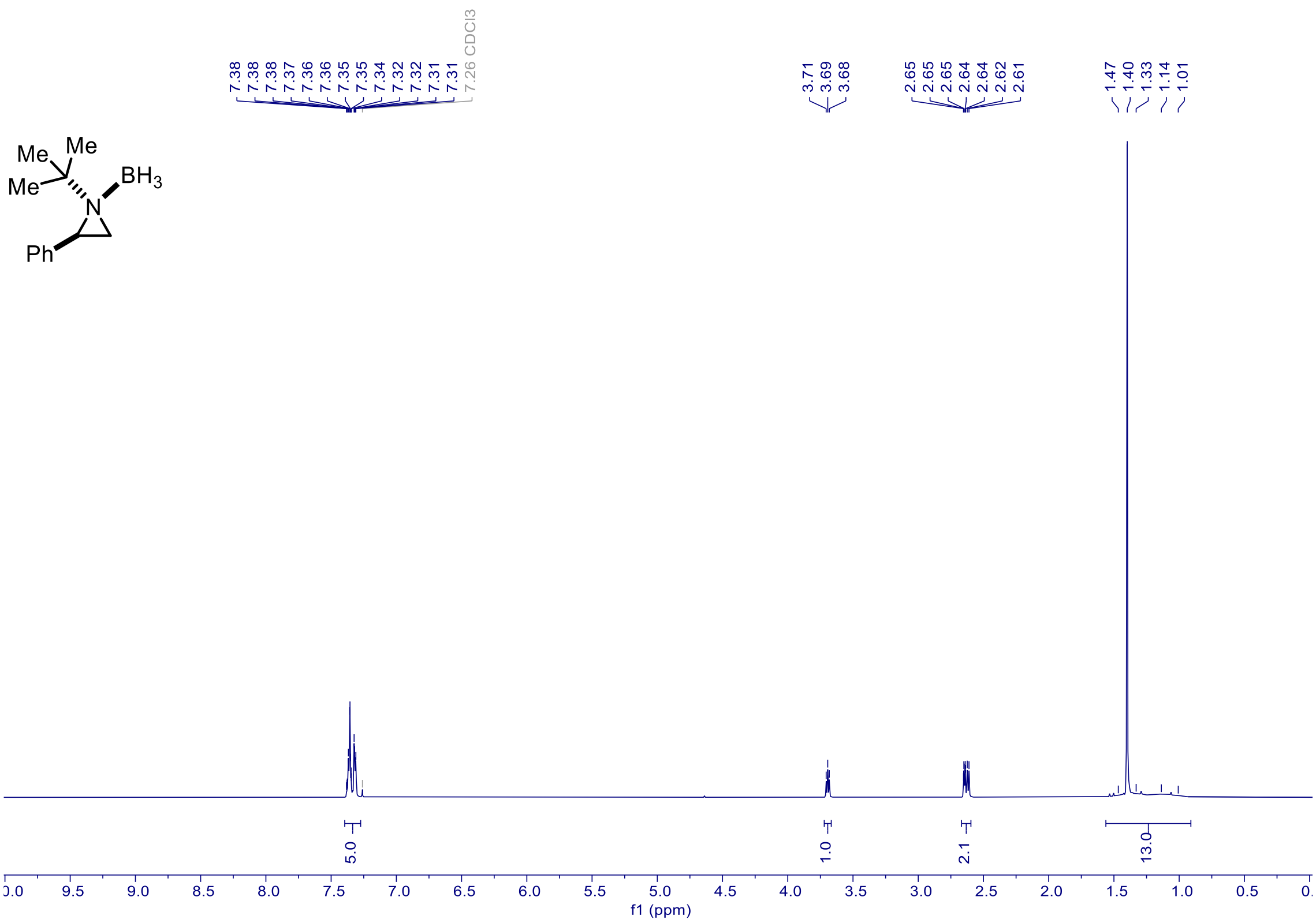

17a – ^{13}C NMR (151 MHz, $CDCl_3$)

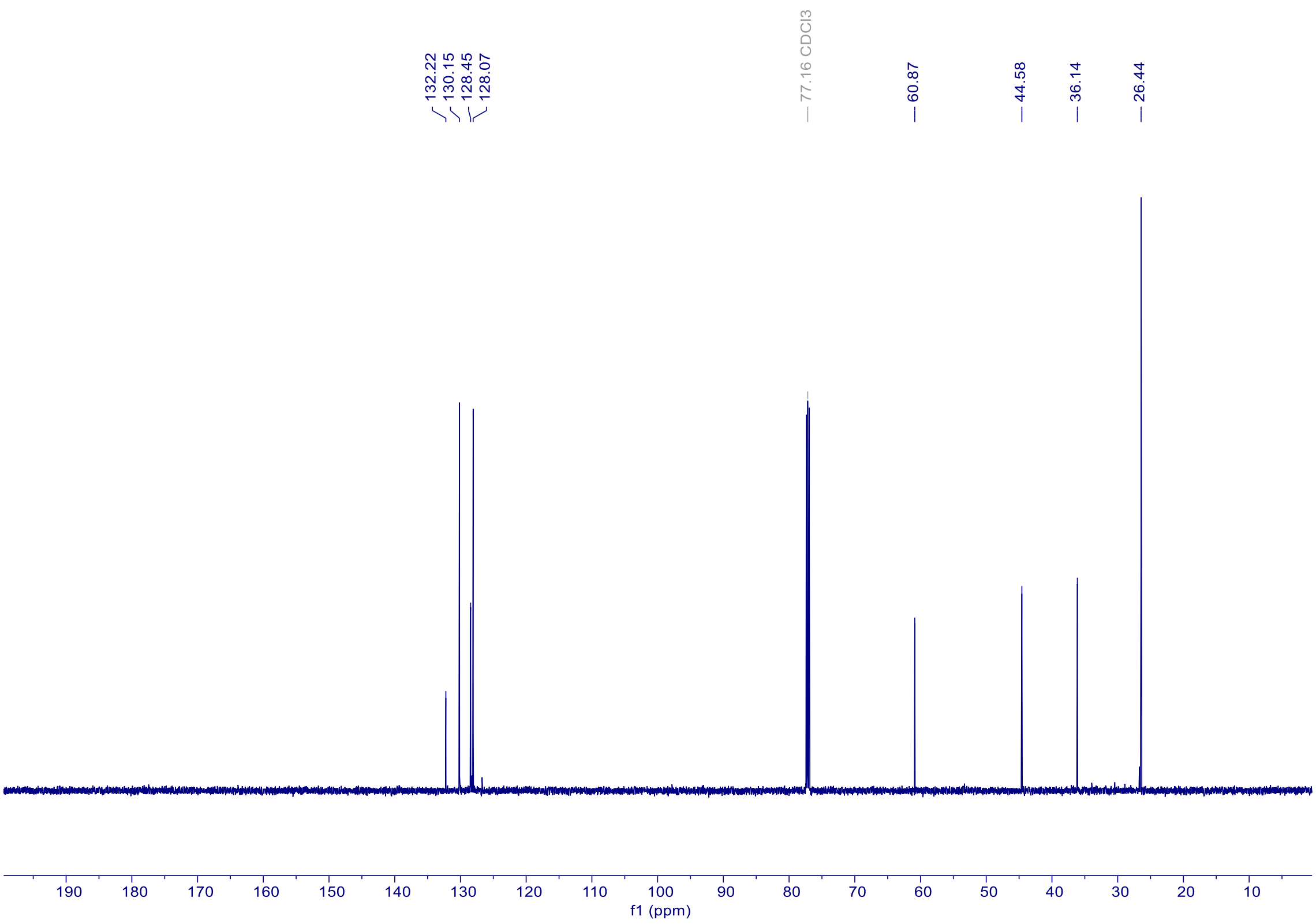

17a – ^{11}B NMR (193 MHz, $CDCl_3$)

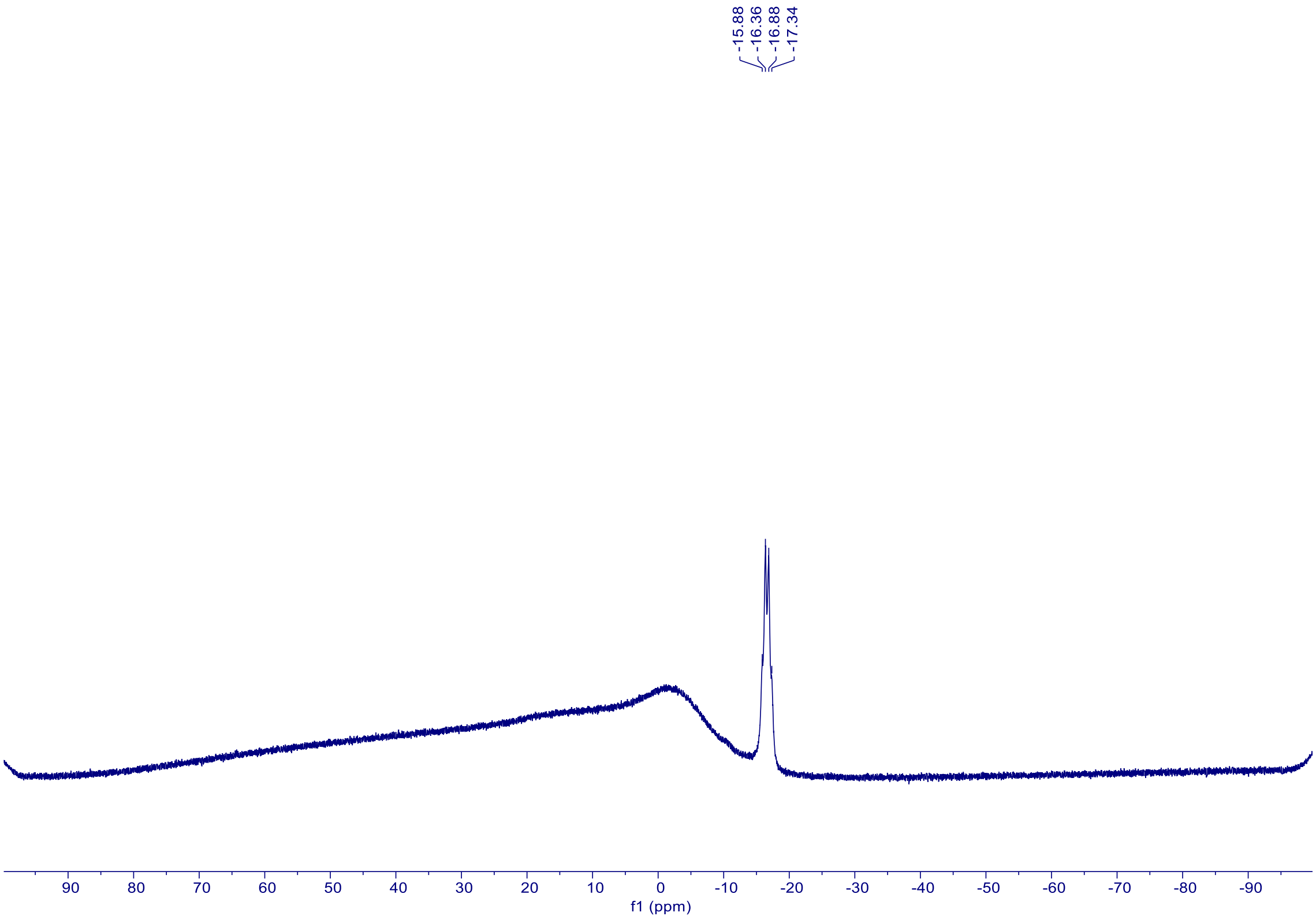

18a and 18a' – ^{1}H NMR (600 MHz, $CDCl_3$)

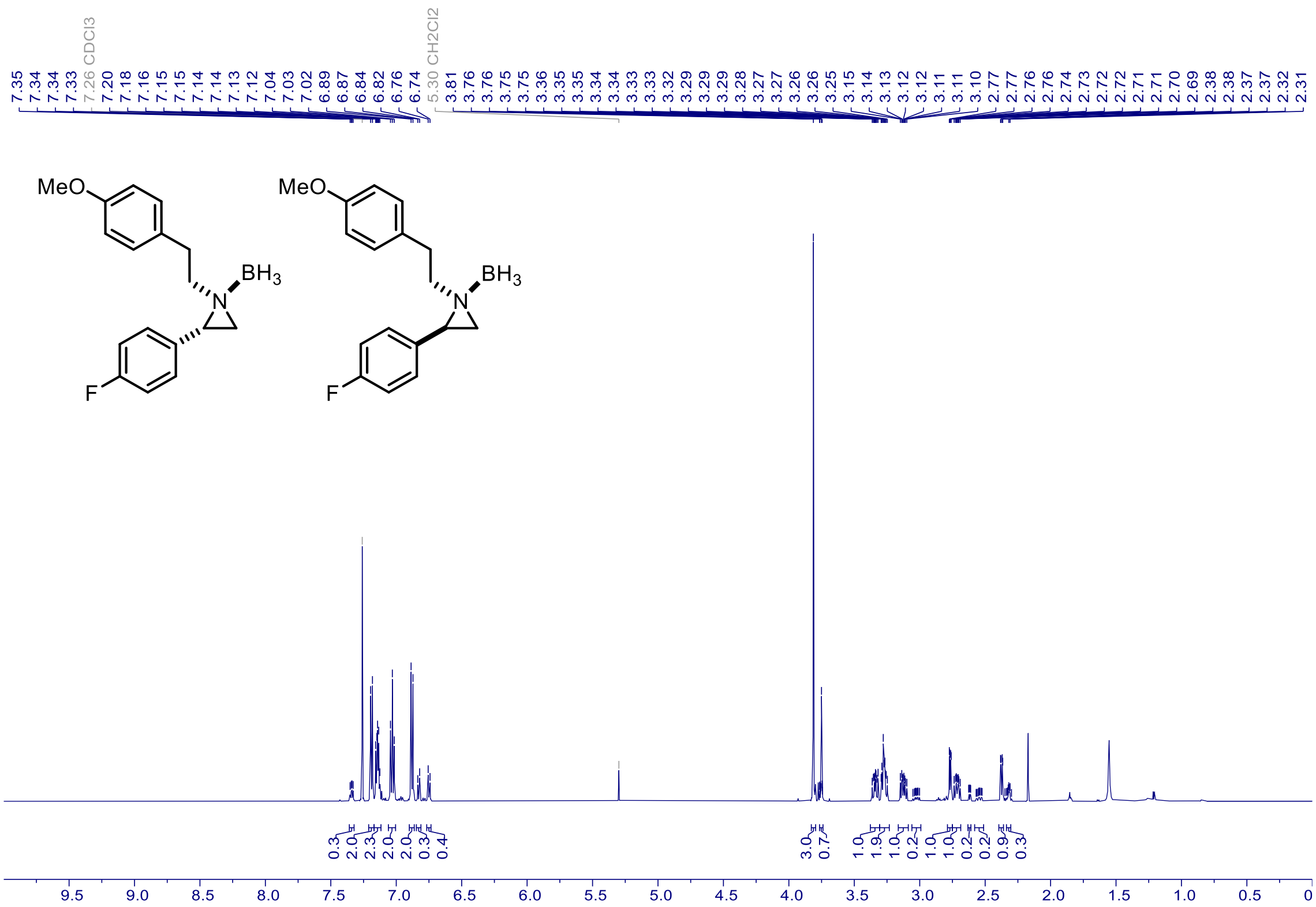

18a and 18a' – ^{13}C NMR (151 MHz, $CDCl_3$)

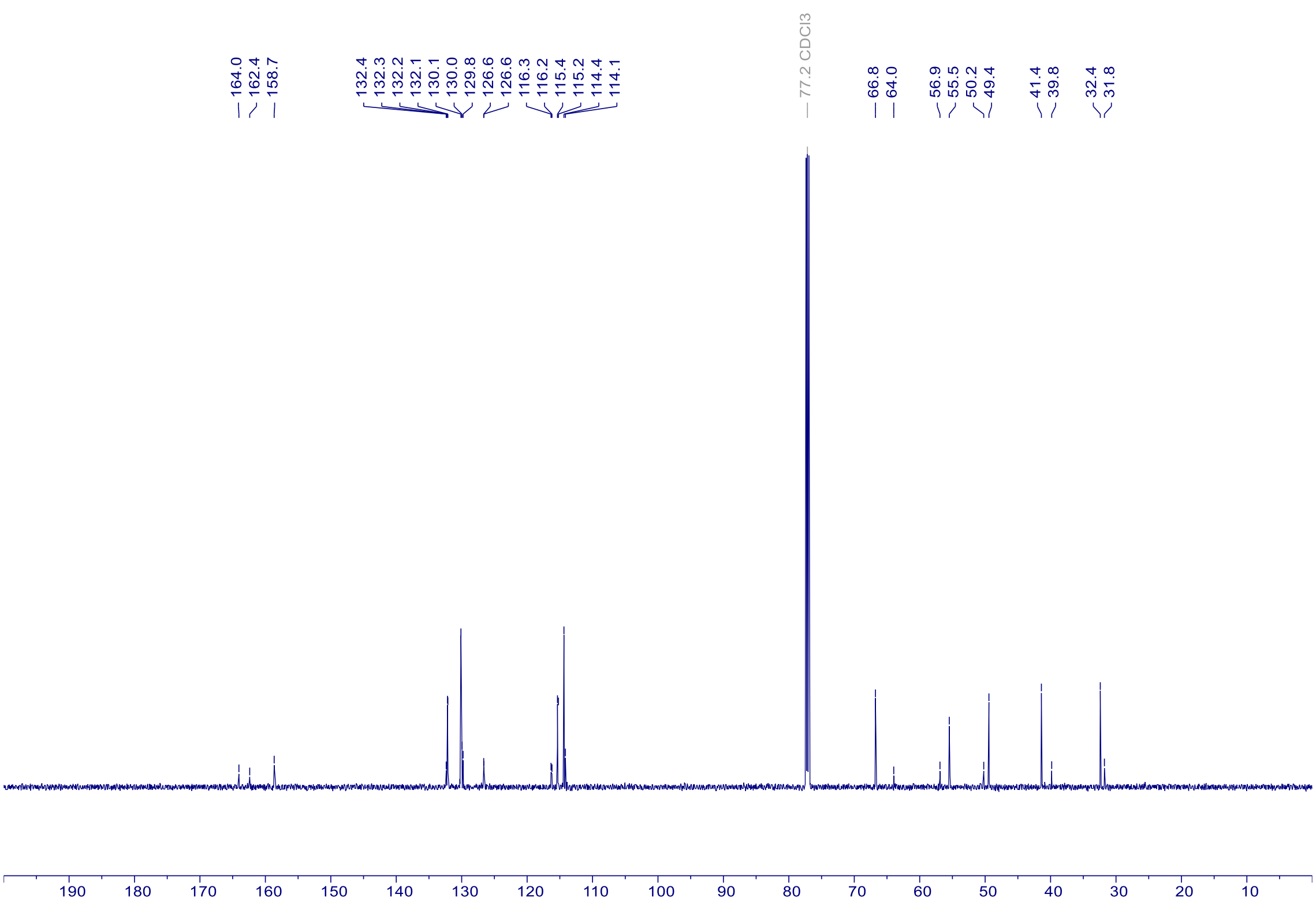

18a and 18a' – ^{11}B NMR (192 MHz, $CDCl_3$)

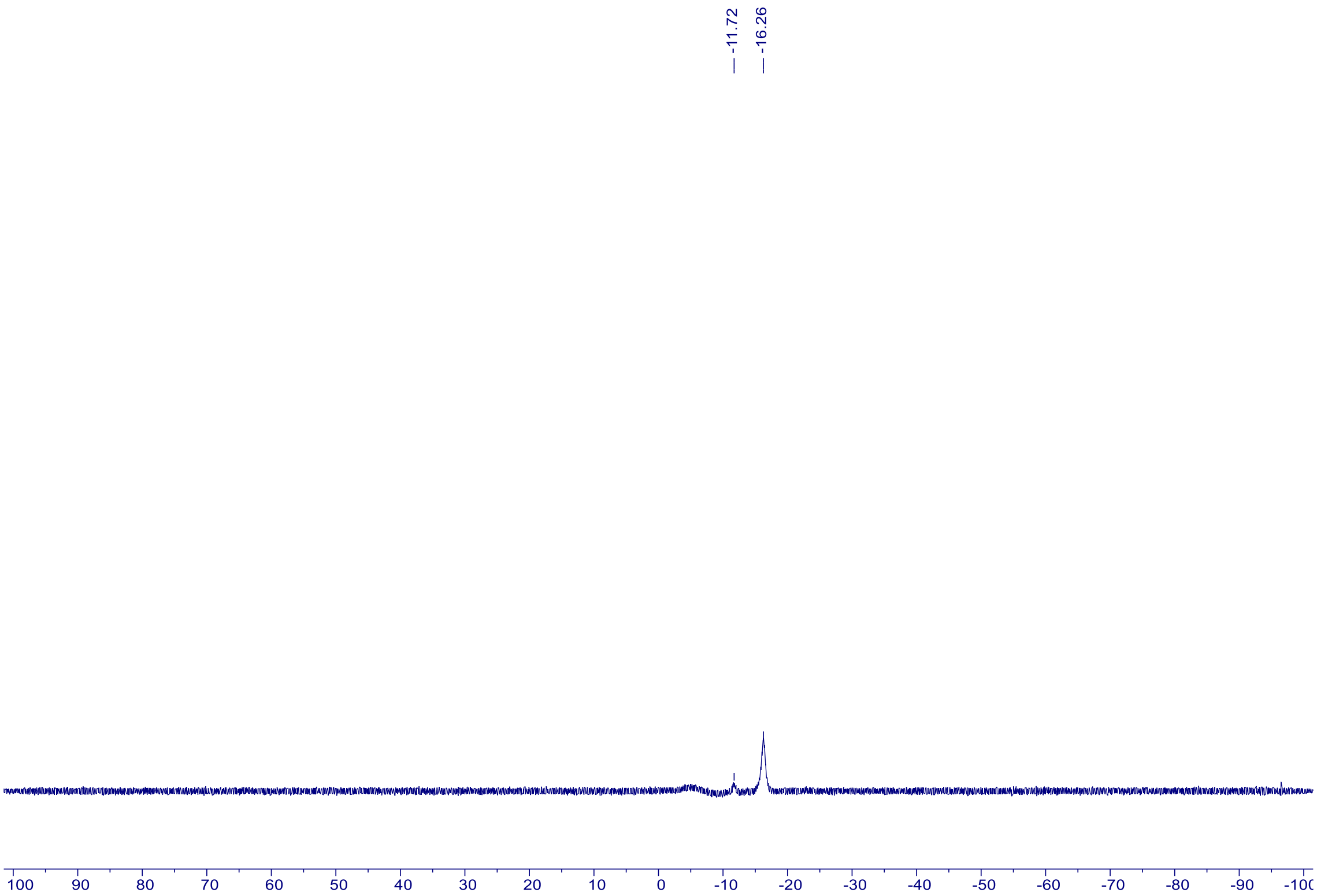

18a and 18a' – ^{19}F NMR (564 MHz, $CDCl_3$)

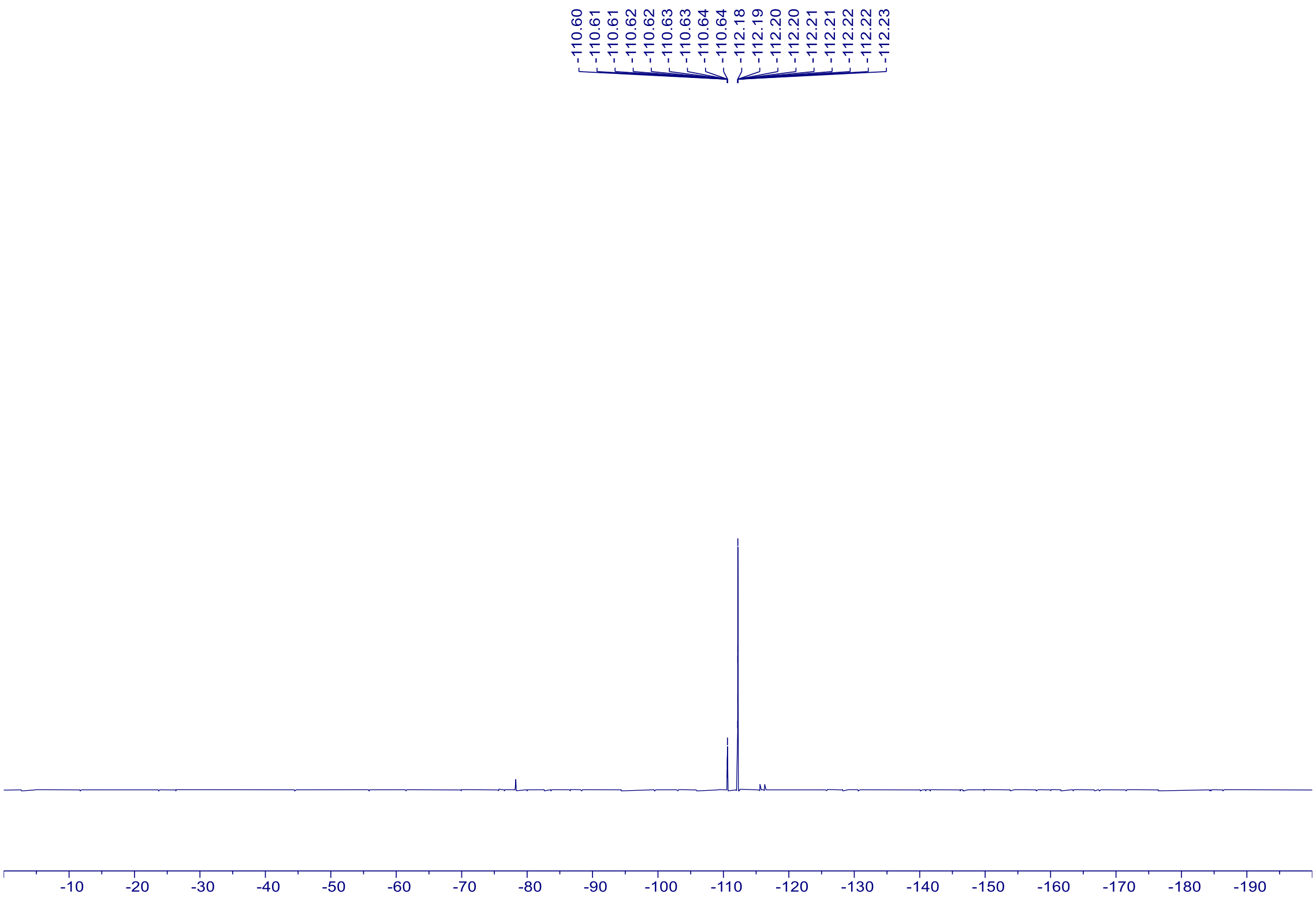

19a – ^{1}H NMR (400 MHz, $CDCl_3$)

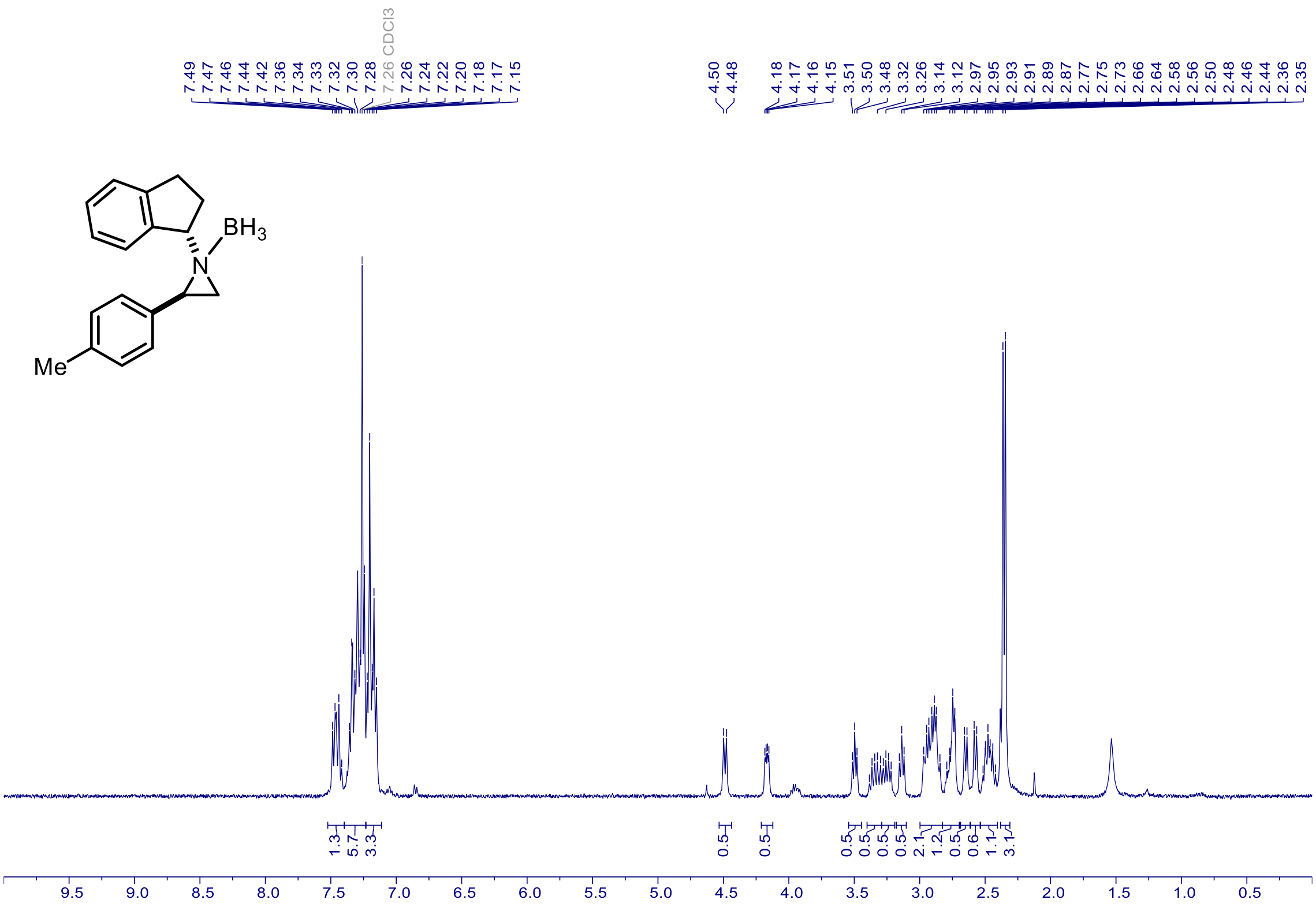

19a – ^{13}C NMR (151 MHz, $CDCl_3$)

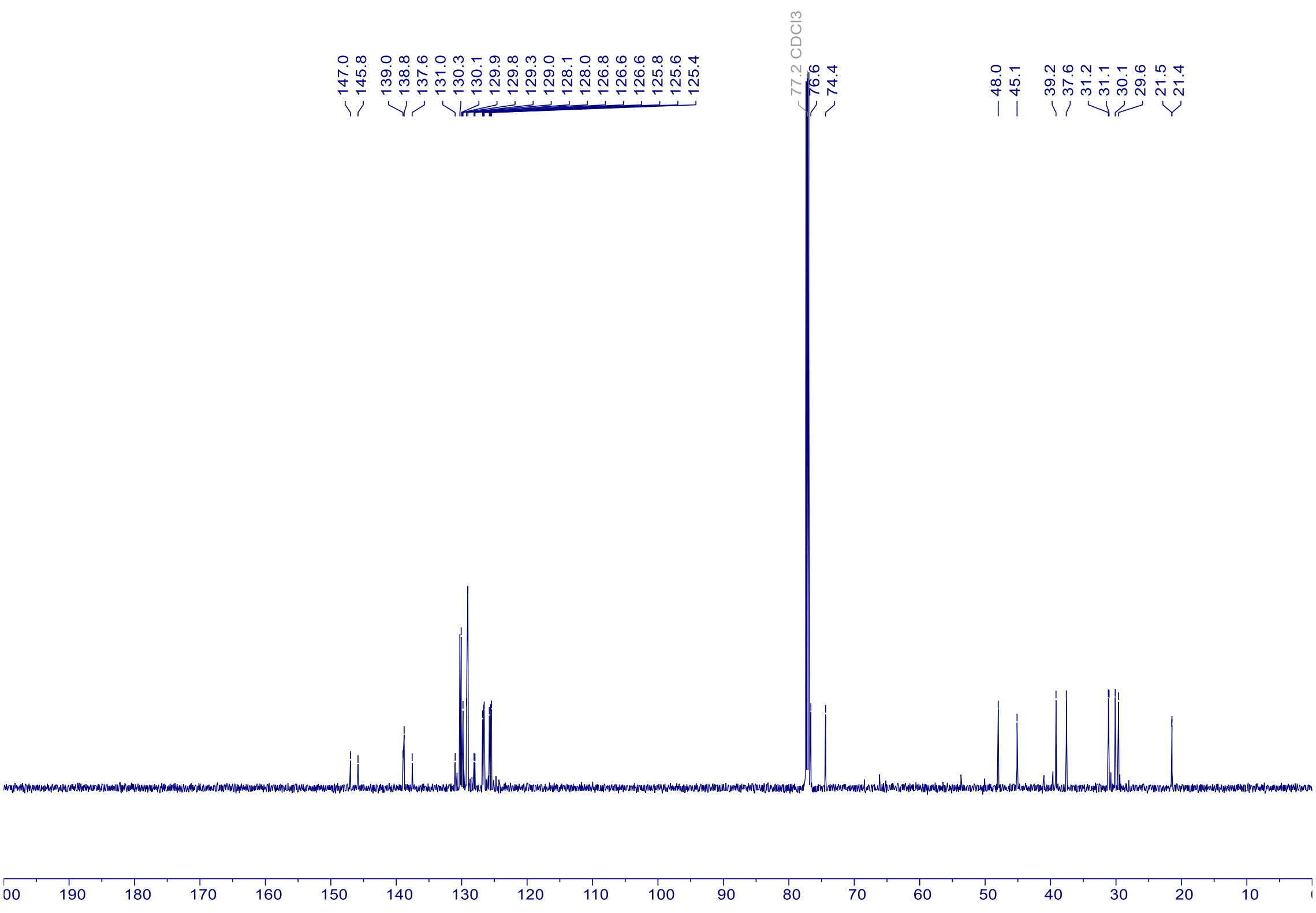

19a – ^{11}B NMR (192 MHz, $CDCl_3$)

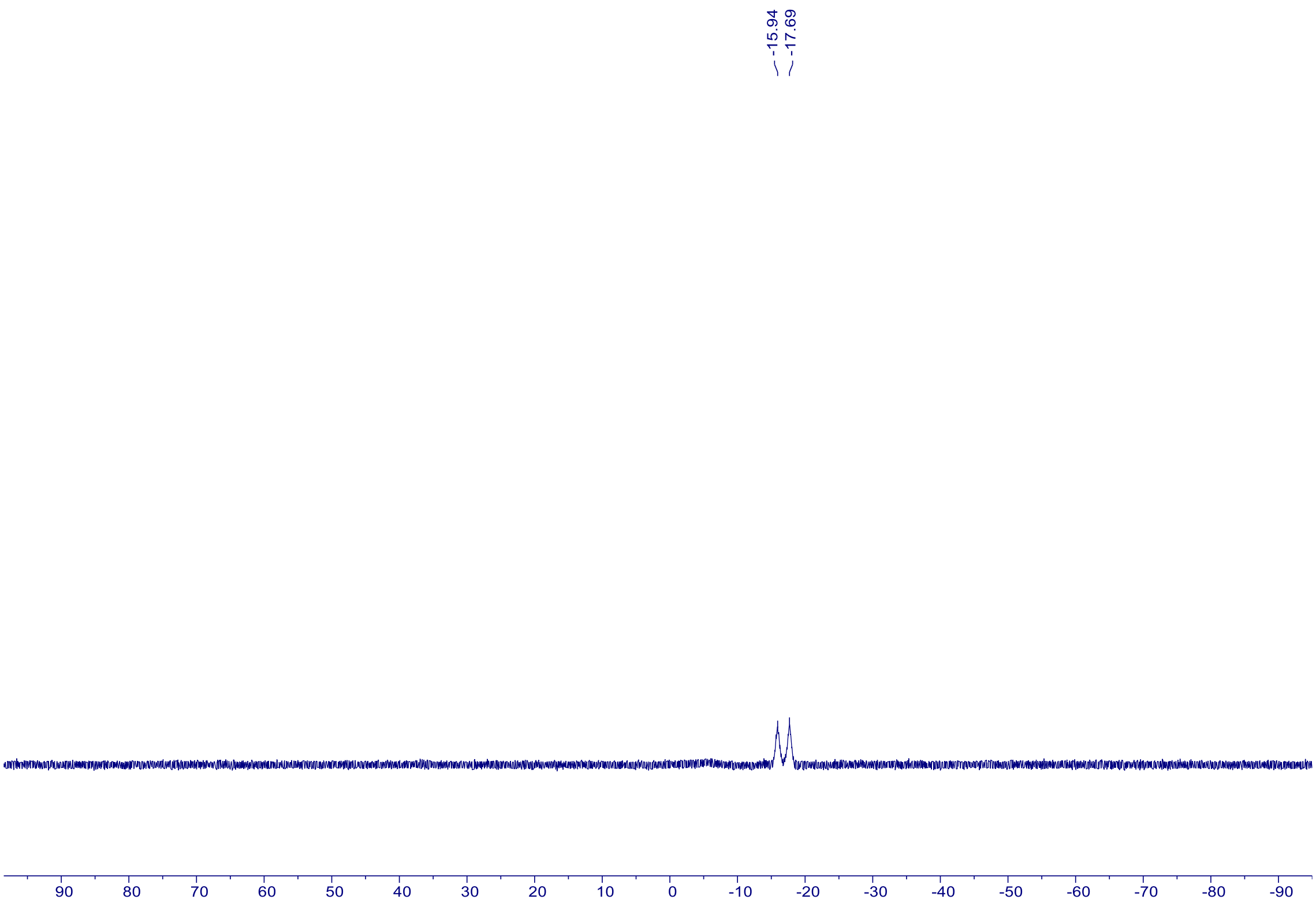

20a and 20a' – ^{1}H NMR (600 MHz, $CDCl_3$)

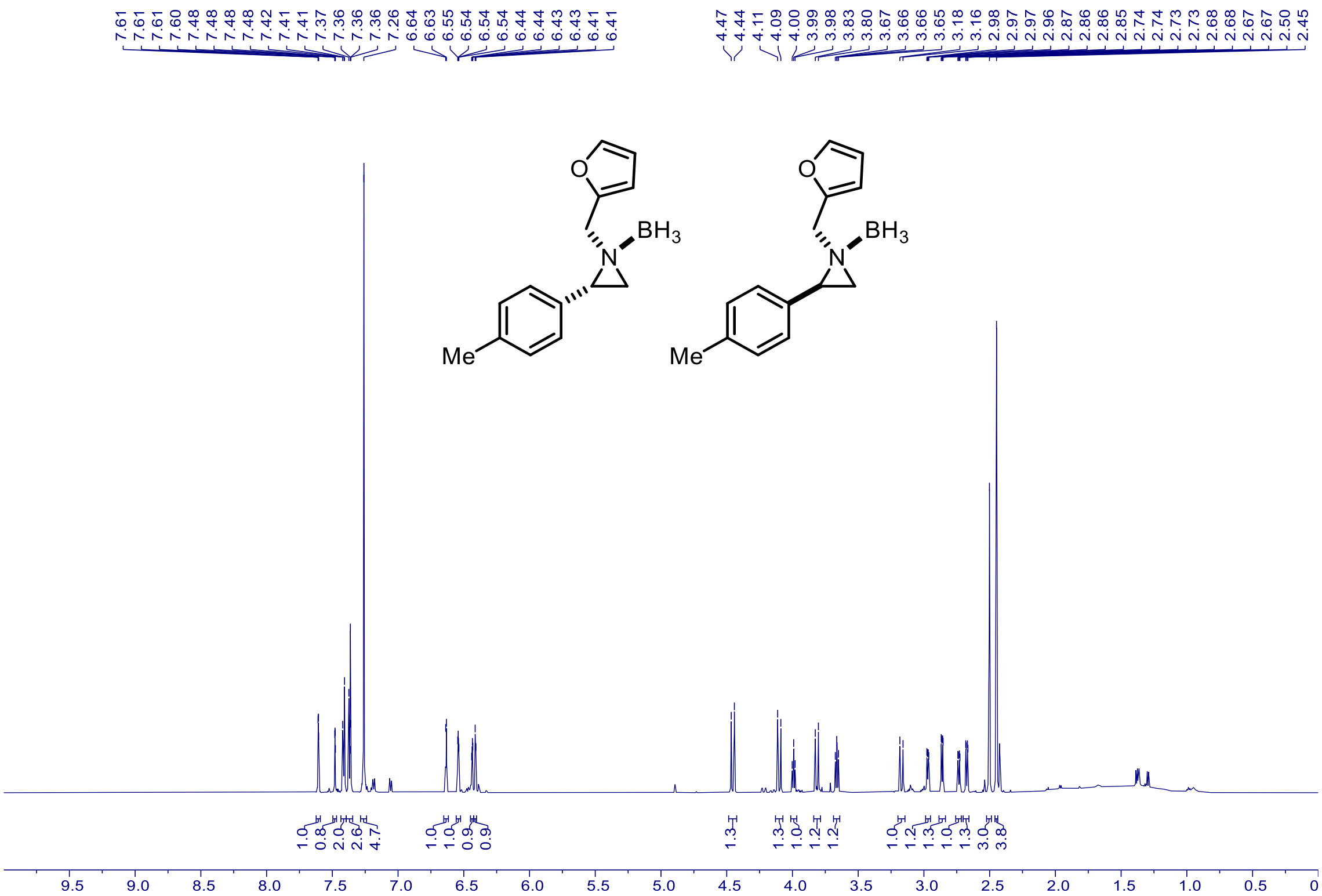

20a and 20a' – ^{13}C NMR (151 MHz, $CDCl_3$)

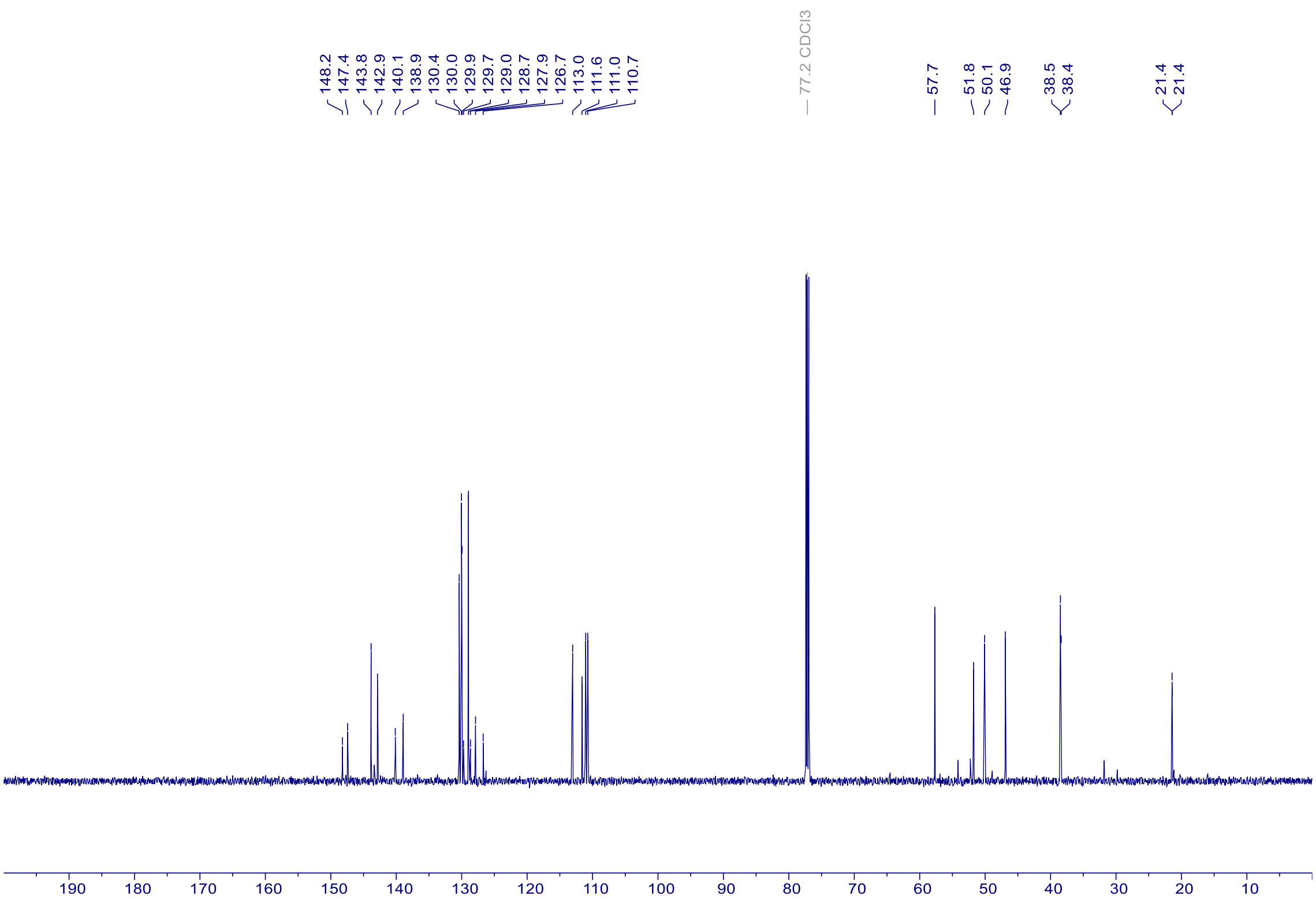

20a and 20a' – ^{11}B NMR (192 MHz, $CDCl_3$)

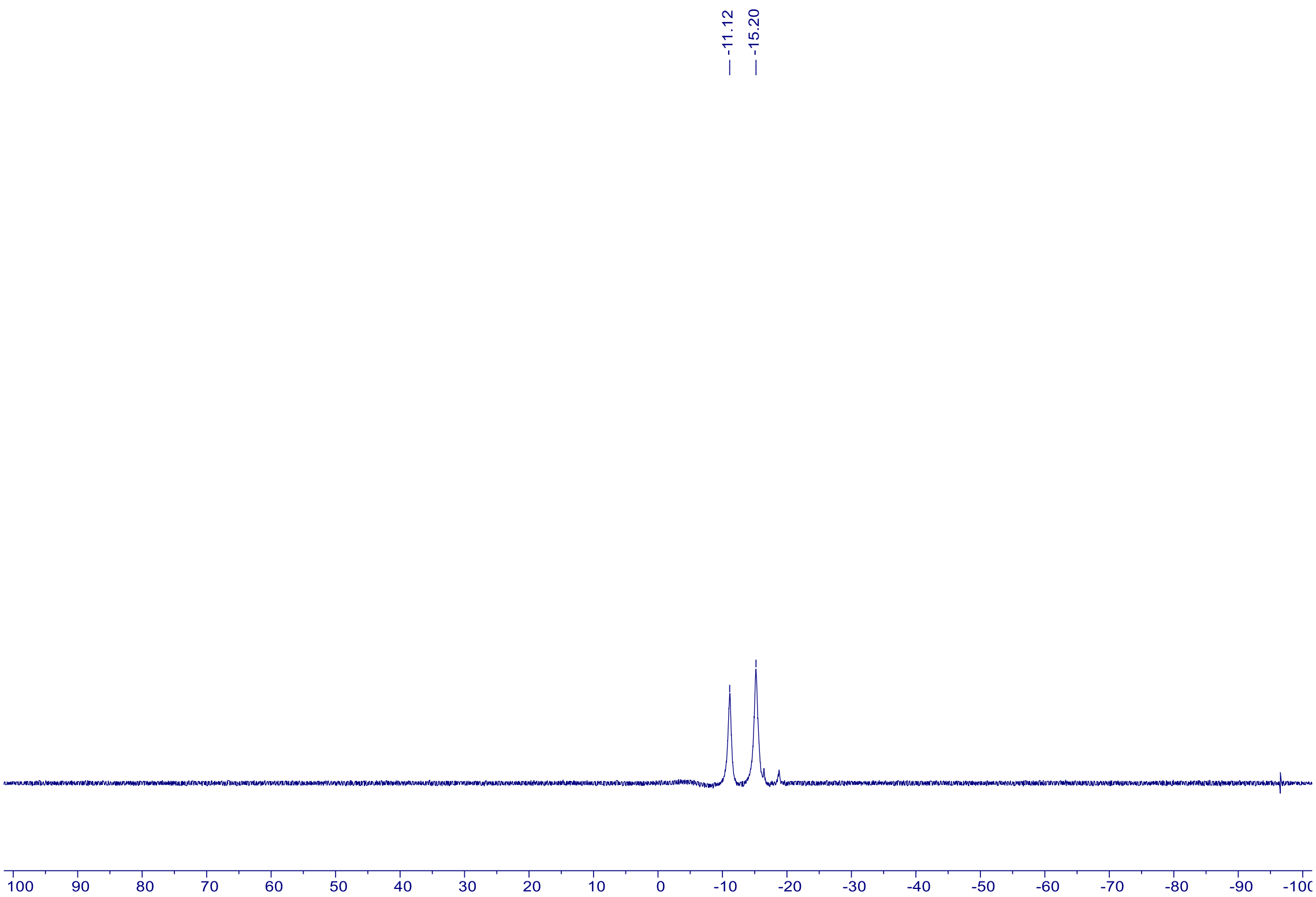

21a – ^{1}H NMR (400 MHz, $CDCl_3$)

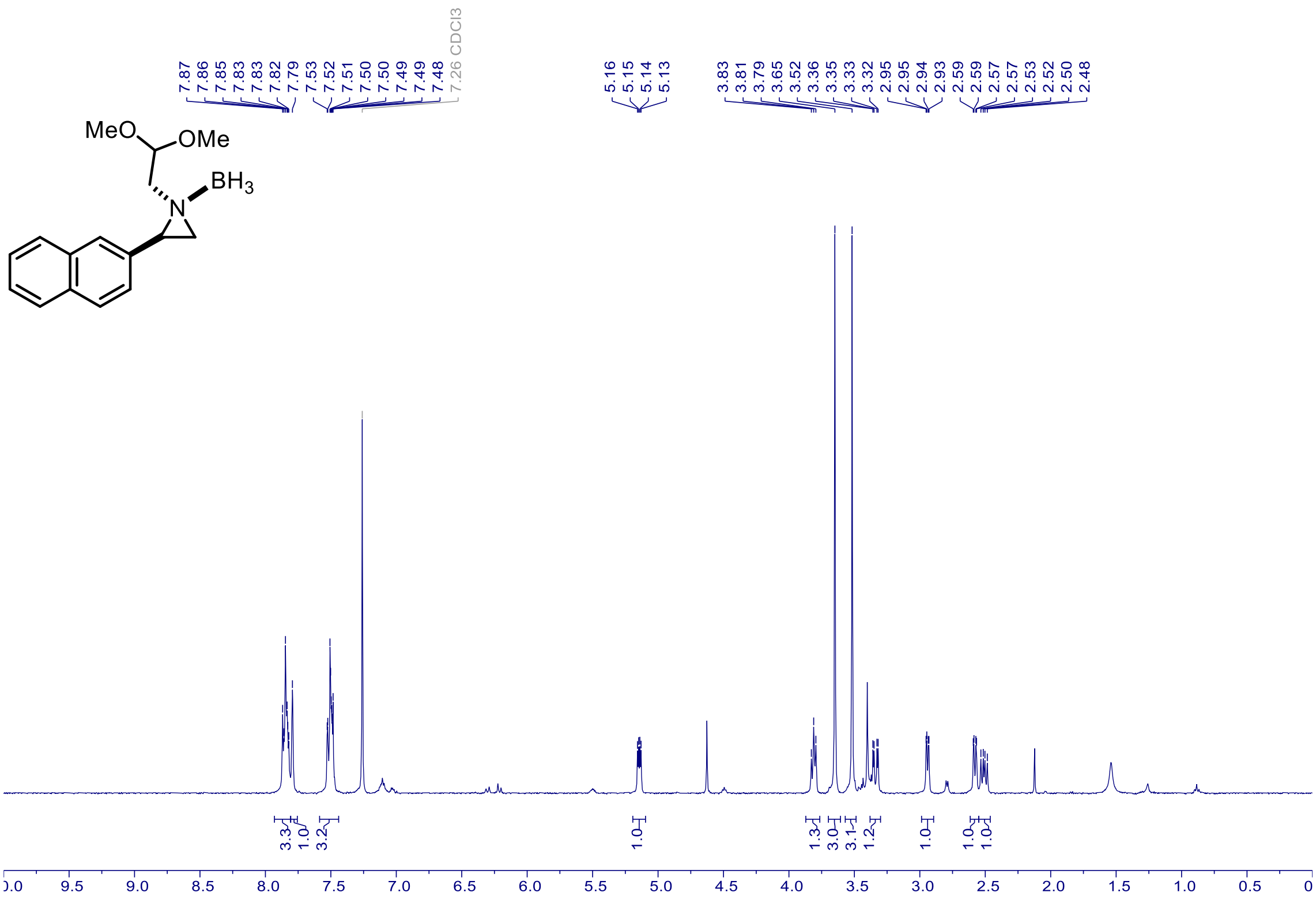

21a – ^{13}C NMR (101 MHz, $CDCl_3$)

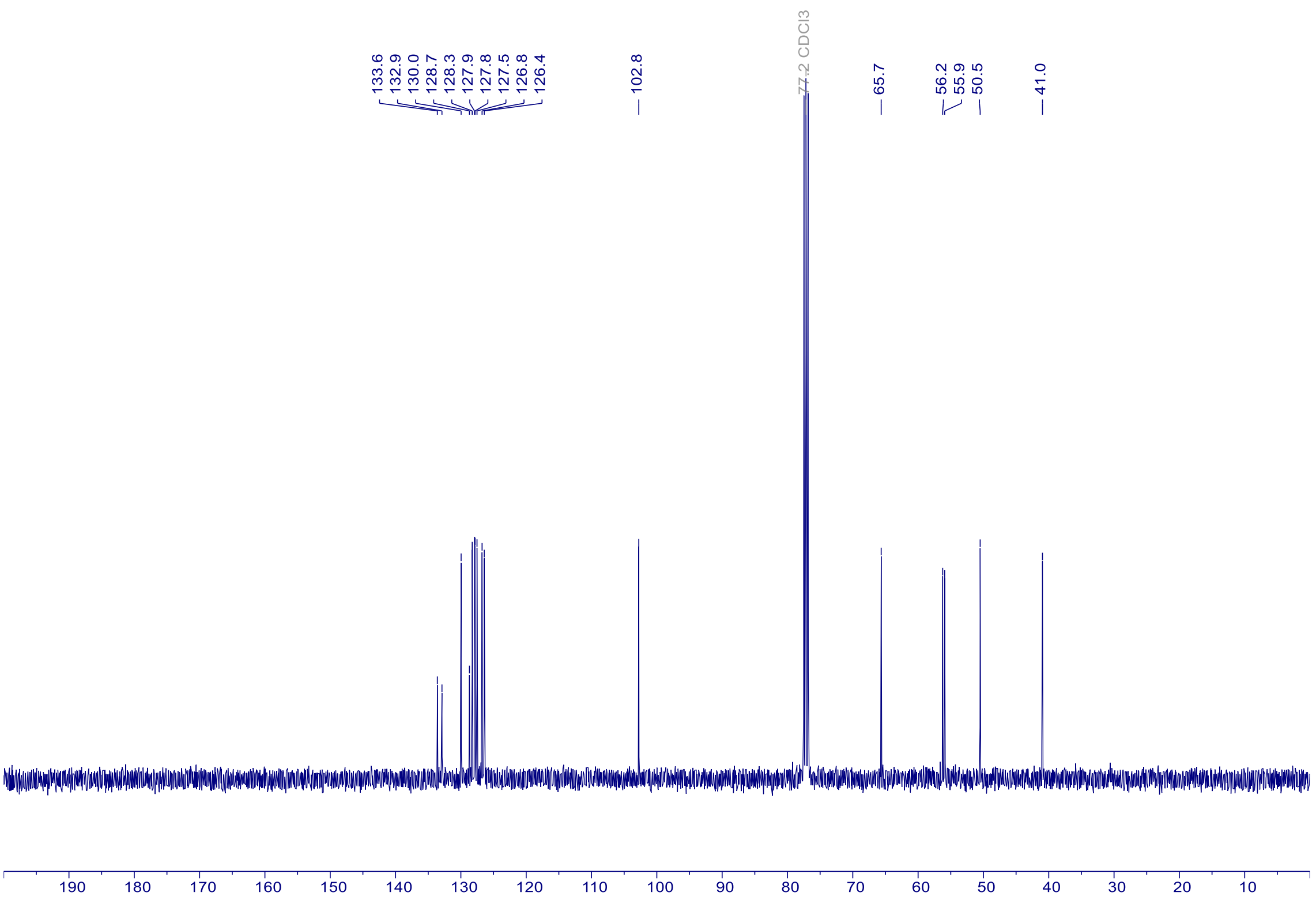

21a – ^{11}B NMR (192 MHz, $CDCl_3$)

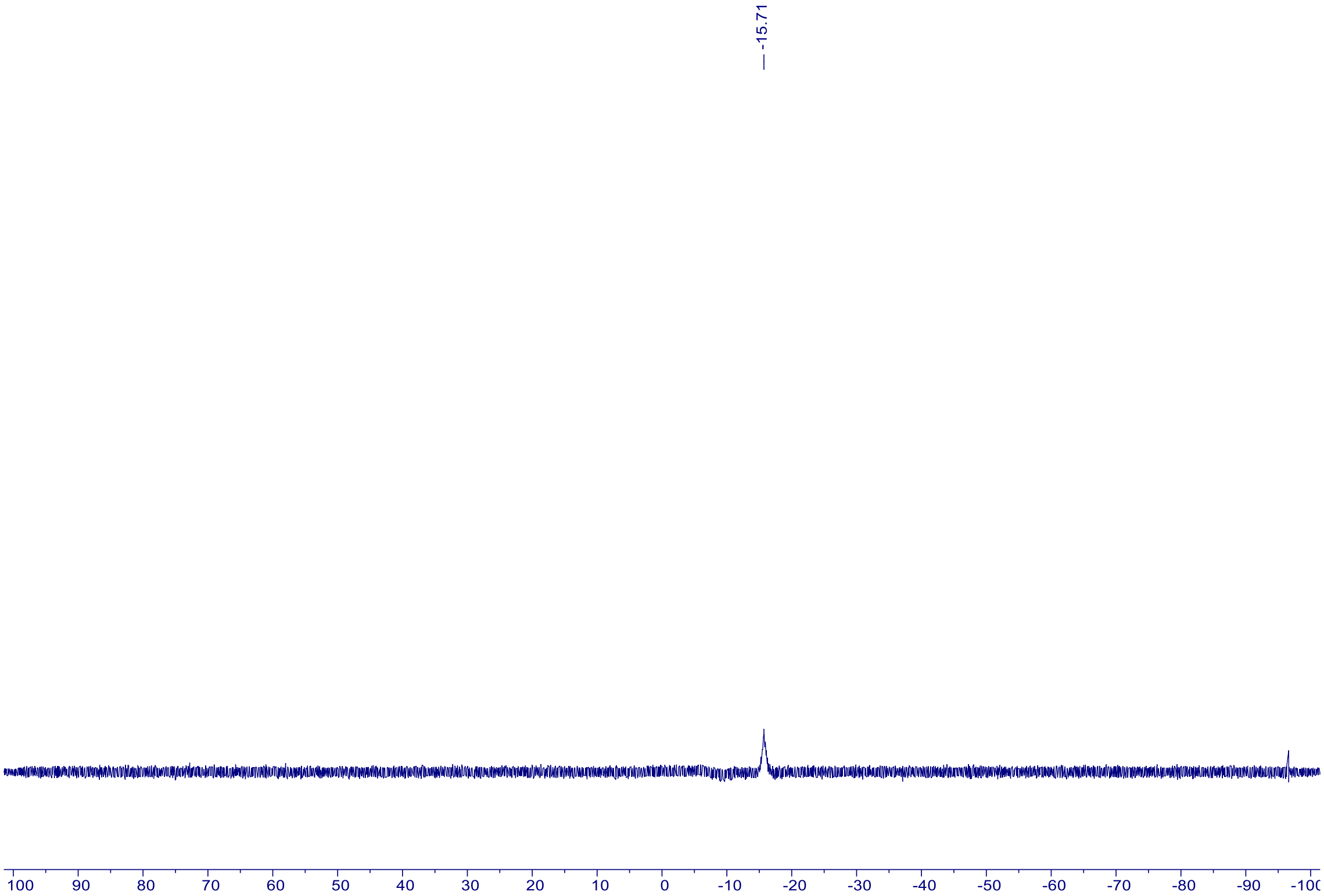

22a – ^{1}H NMR (600 MHz, $CDCl_3$)

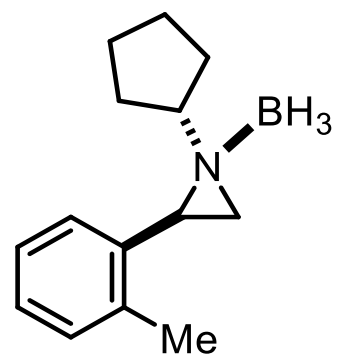

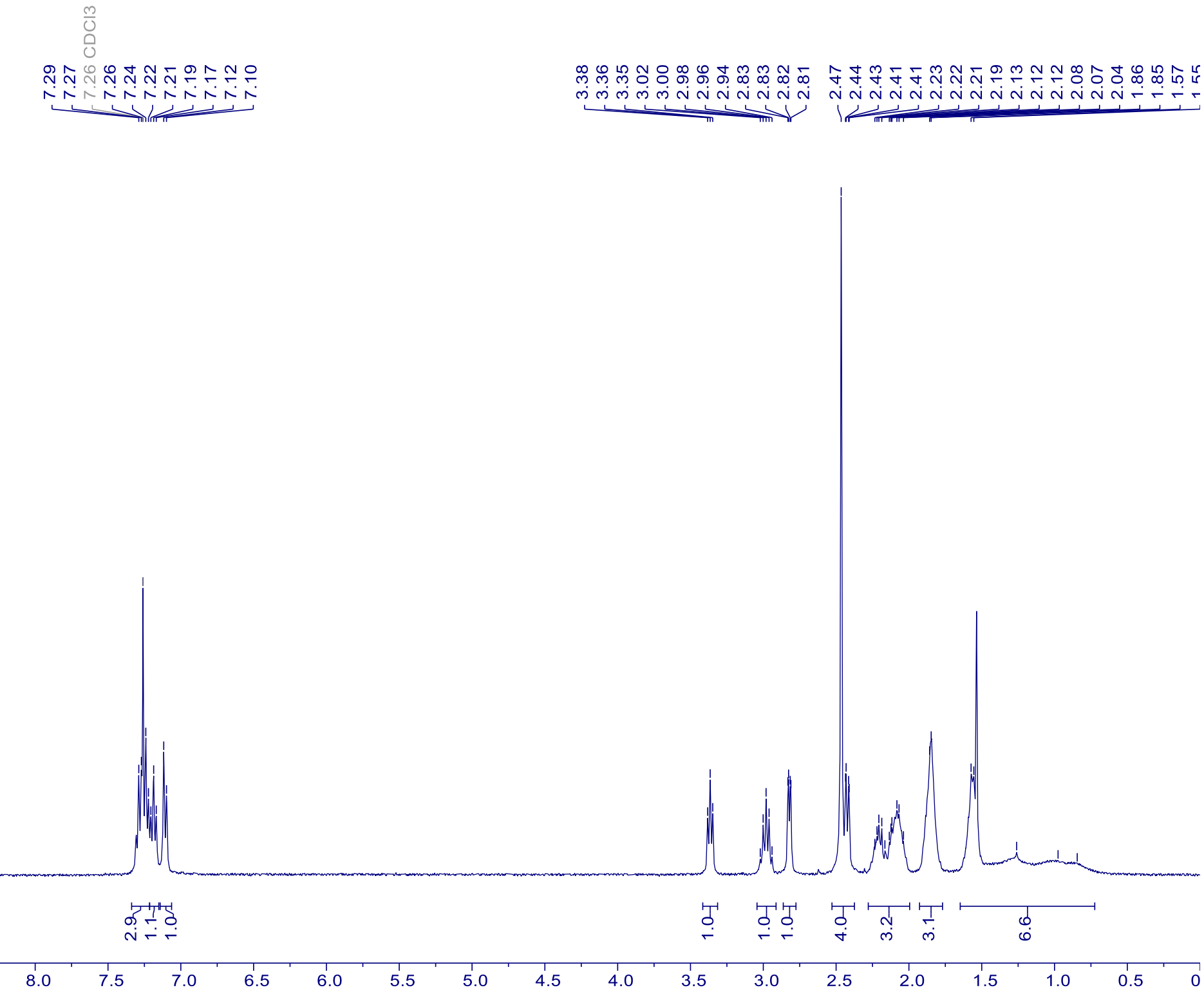

22a – ^{13}C NMR (101 MHz, $CDCl_3$)

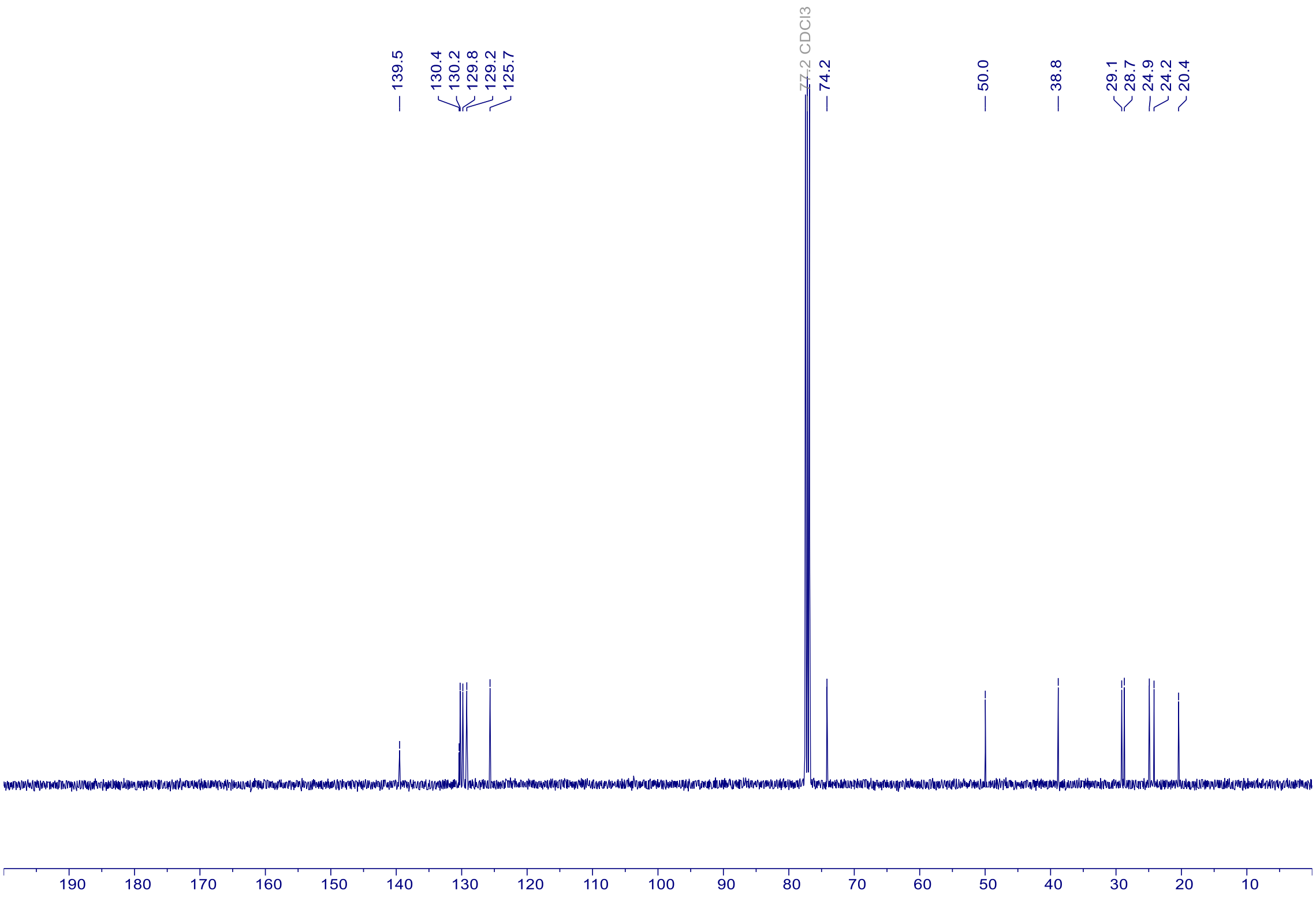

22a – ^{11}B NMR (128 MHz, $CDCl_3$)

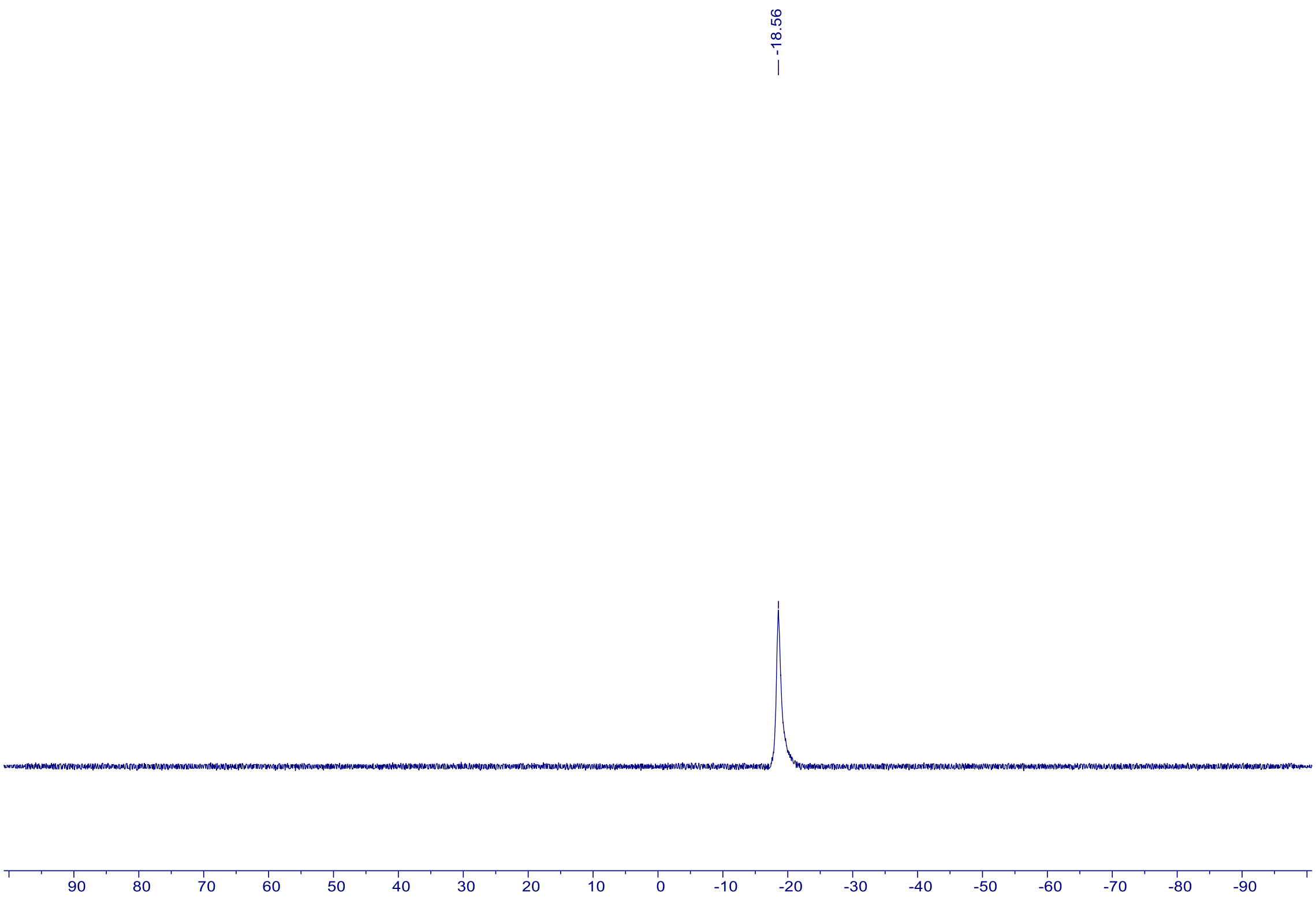

23a – ^{1}H NMR (600 MHz, $CDCl_3$)

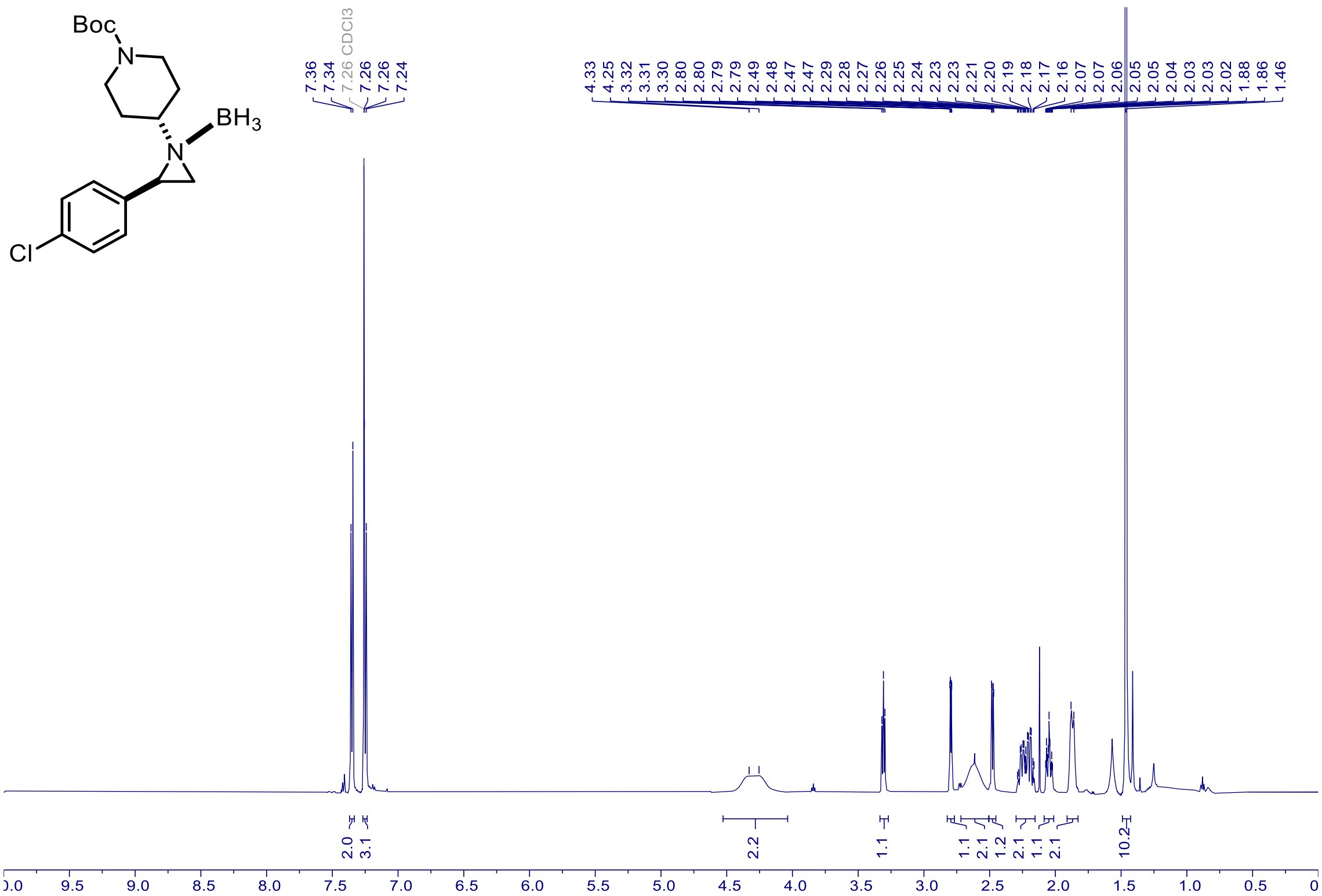

23a – ^{13}C NMR (151 MHz, $CDCl_3$)

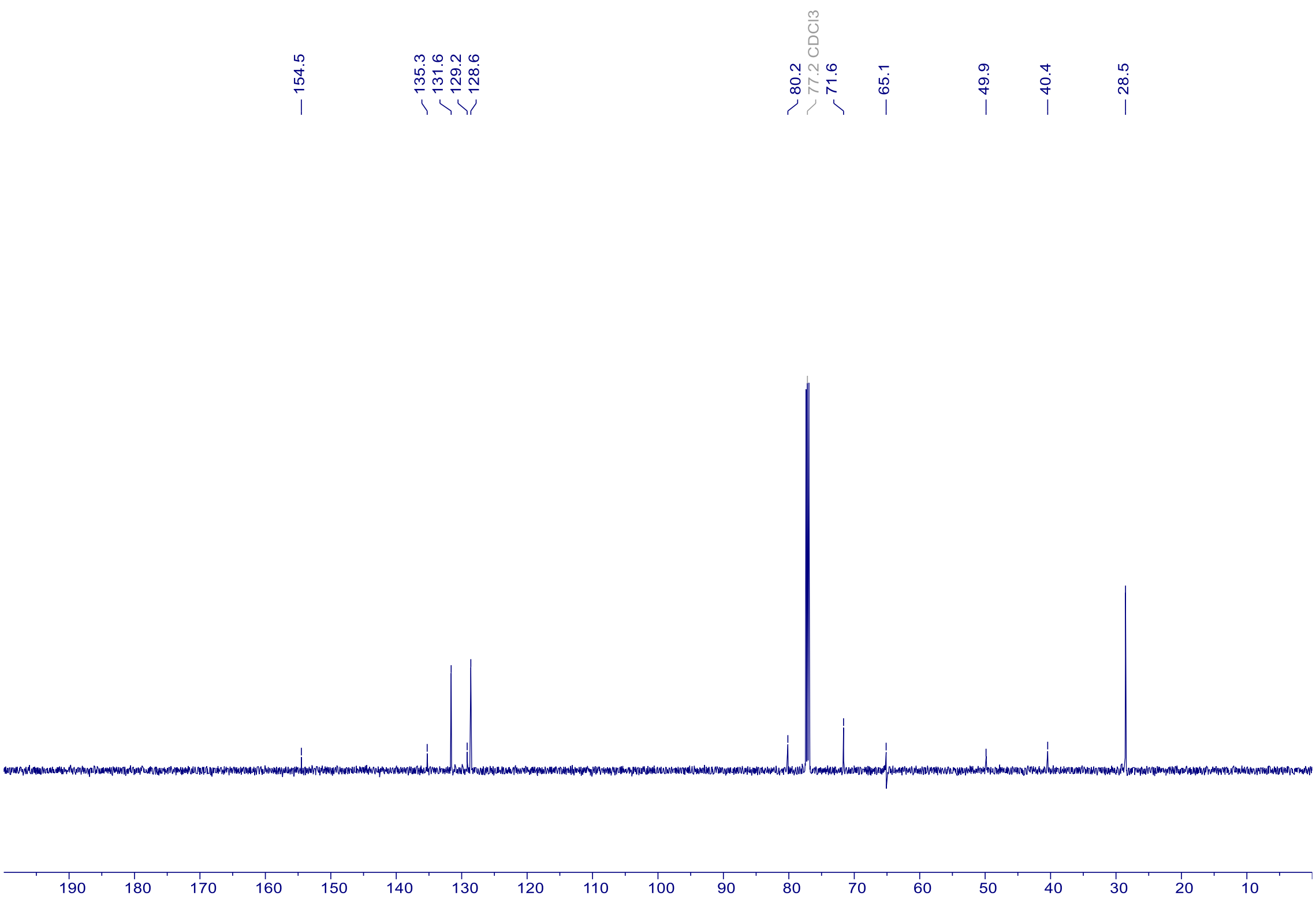

23a – ^{11}B NMR (192 MHz, $CDCl_3$)

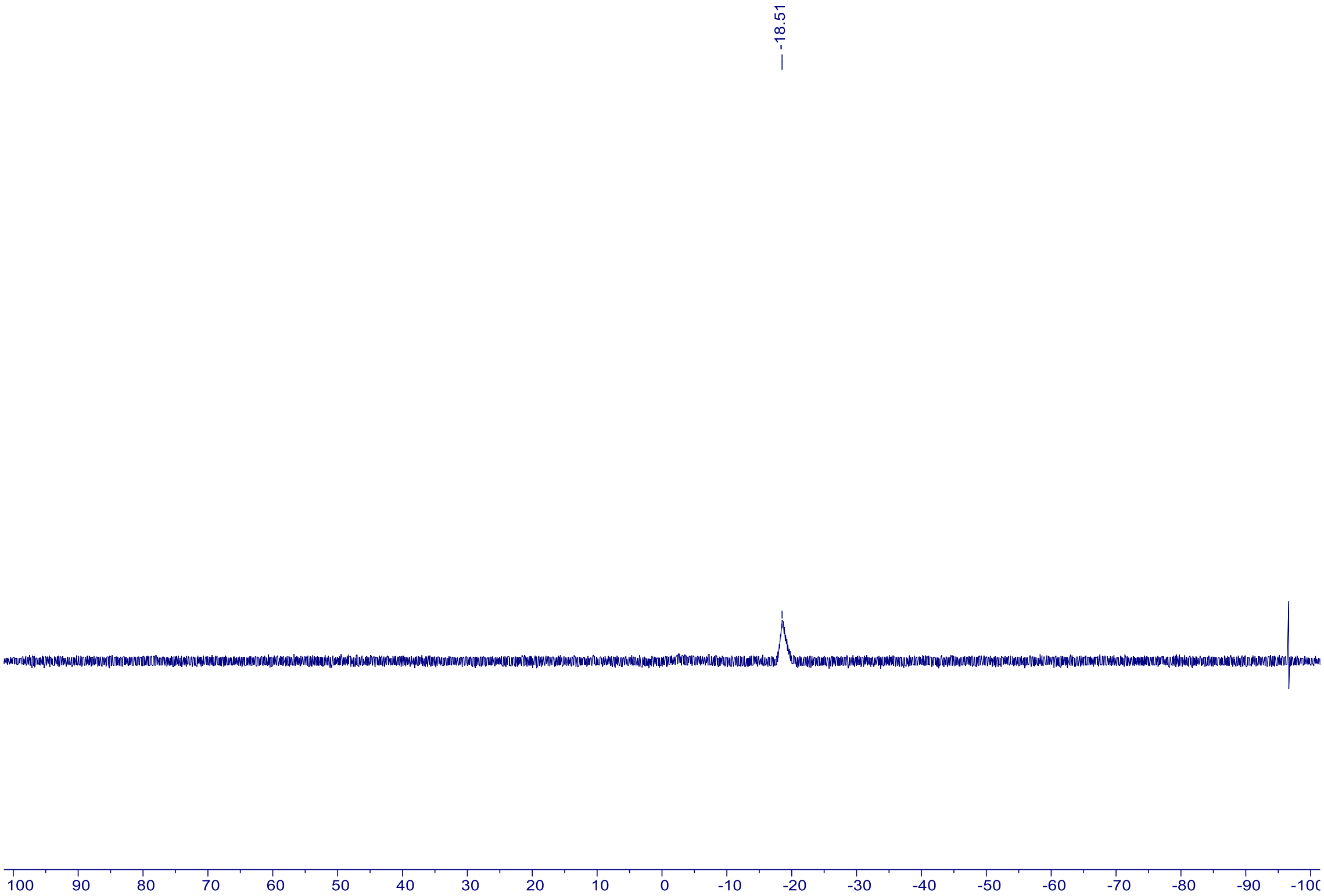

24a – ^{1}H NMR (600 MHz, $CDCl_3$)

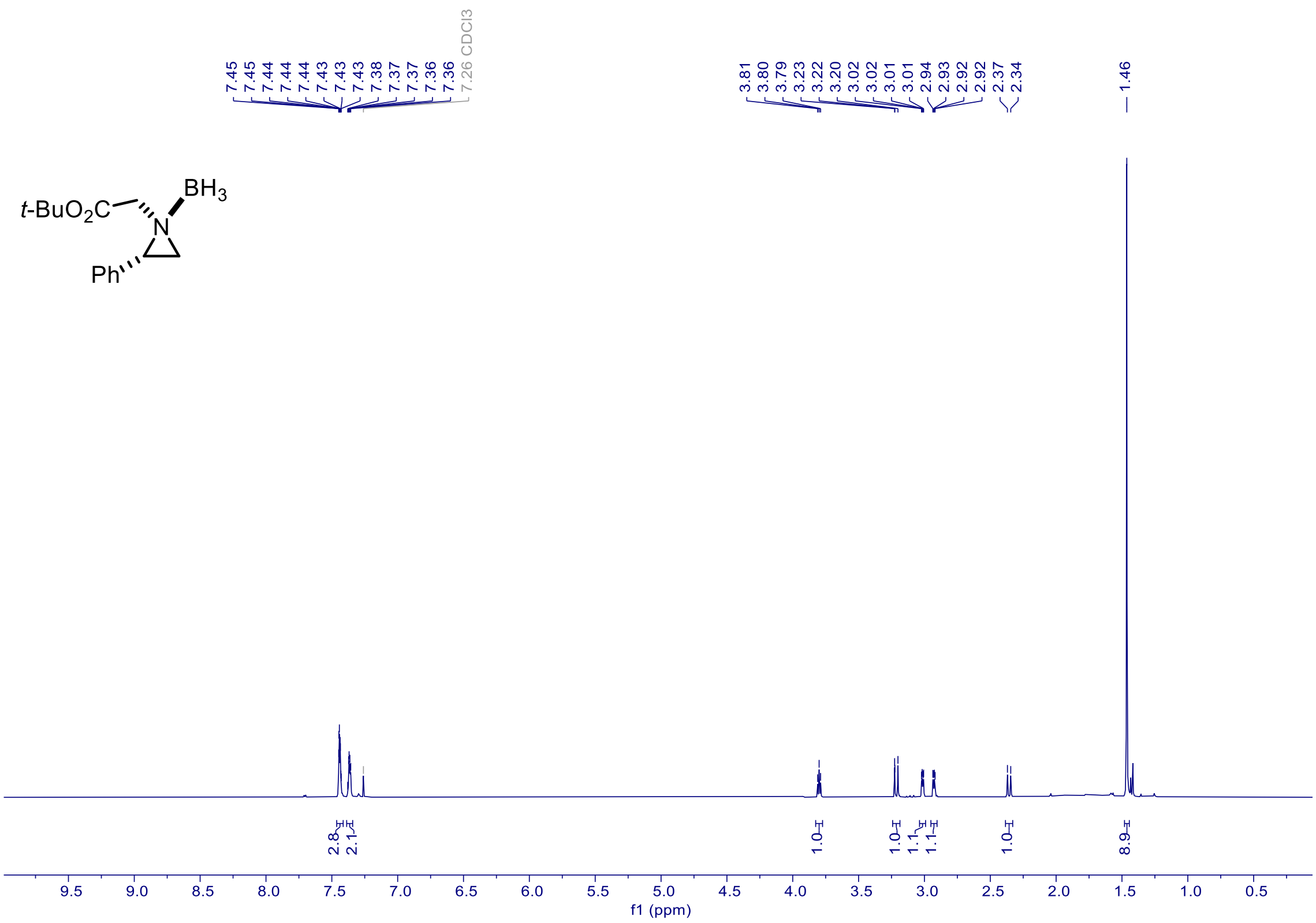

24a – ^{13}C NMR (151 MHz, $CDCl_3$)

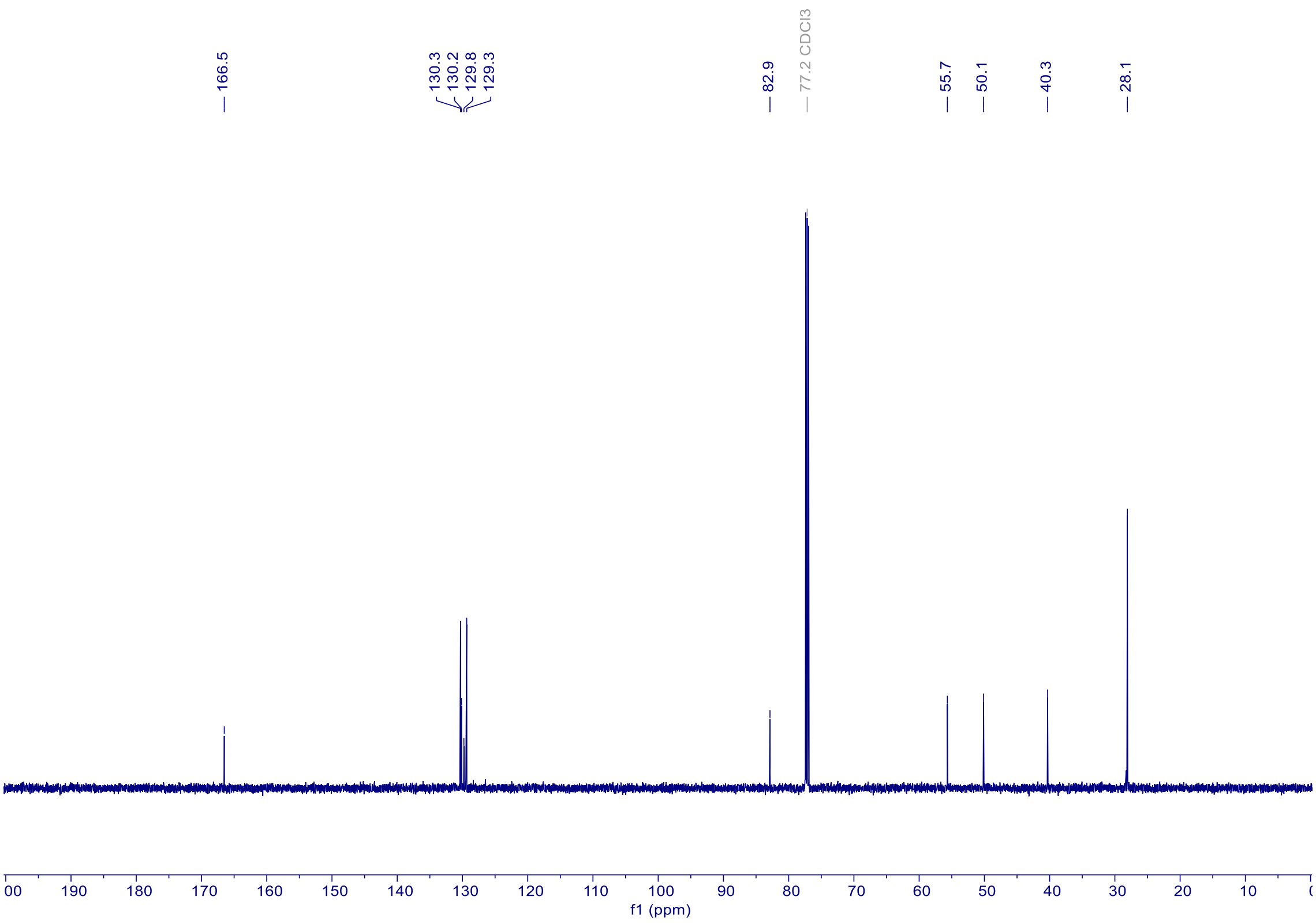

24a – ^{11}B NMR (193 MHz, $CDCl_3$)

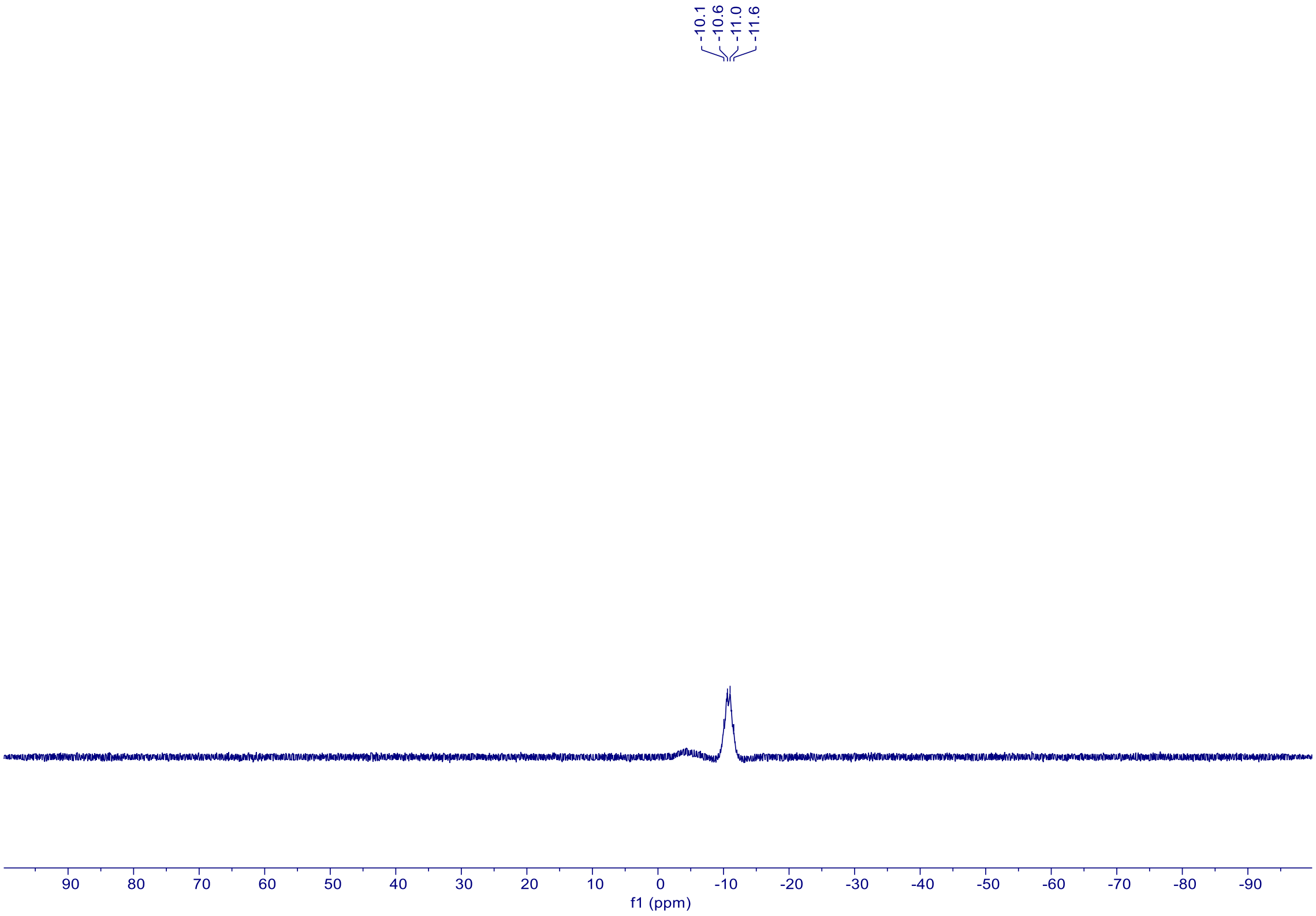

24a’ – ^{1}H NMR (600 MHz, $CDCl_3$)

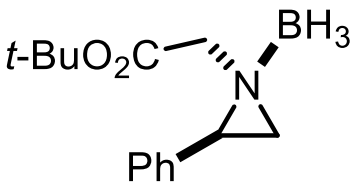

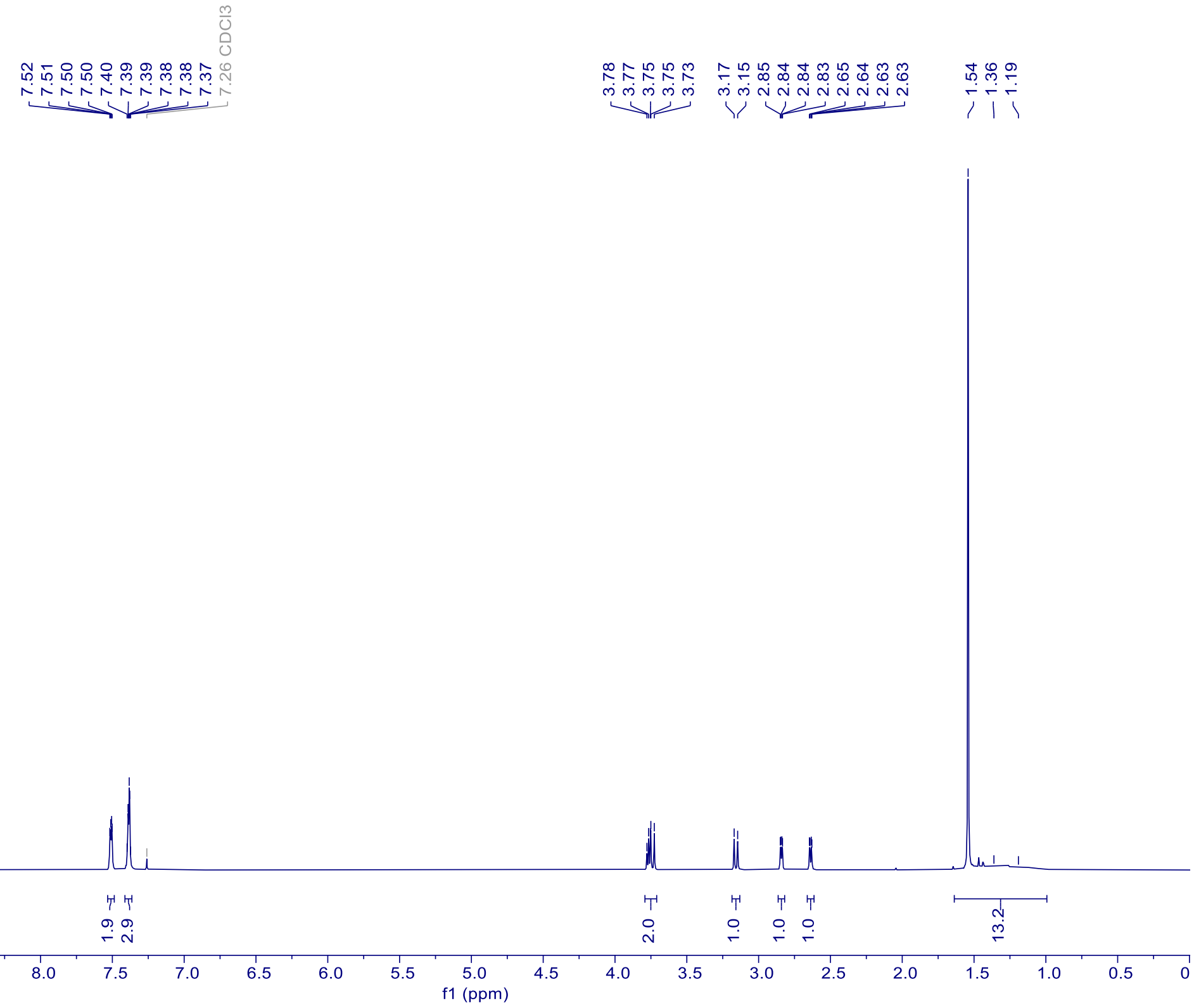

24a’ – ^{13}C NMR (151 MHz, $CDCl_3$)

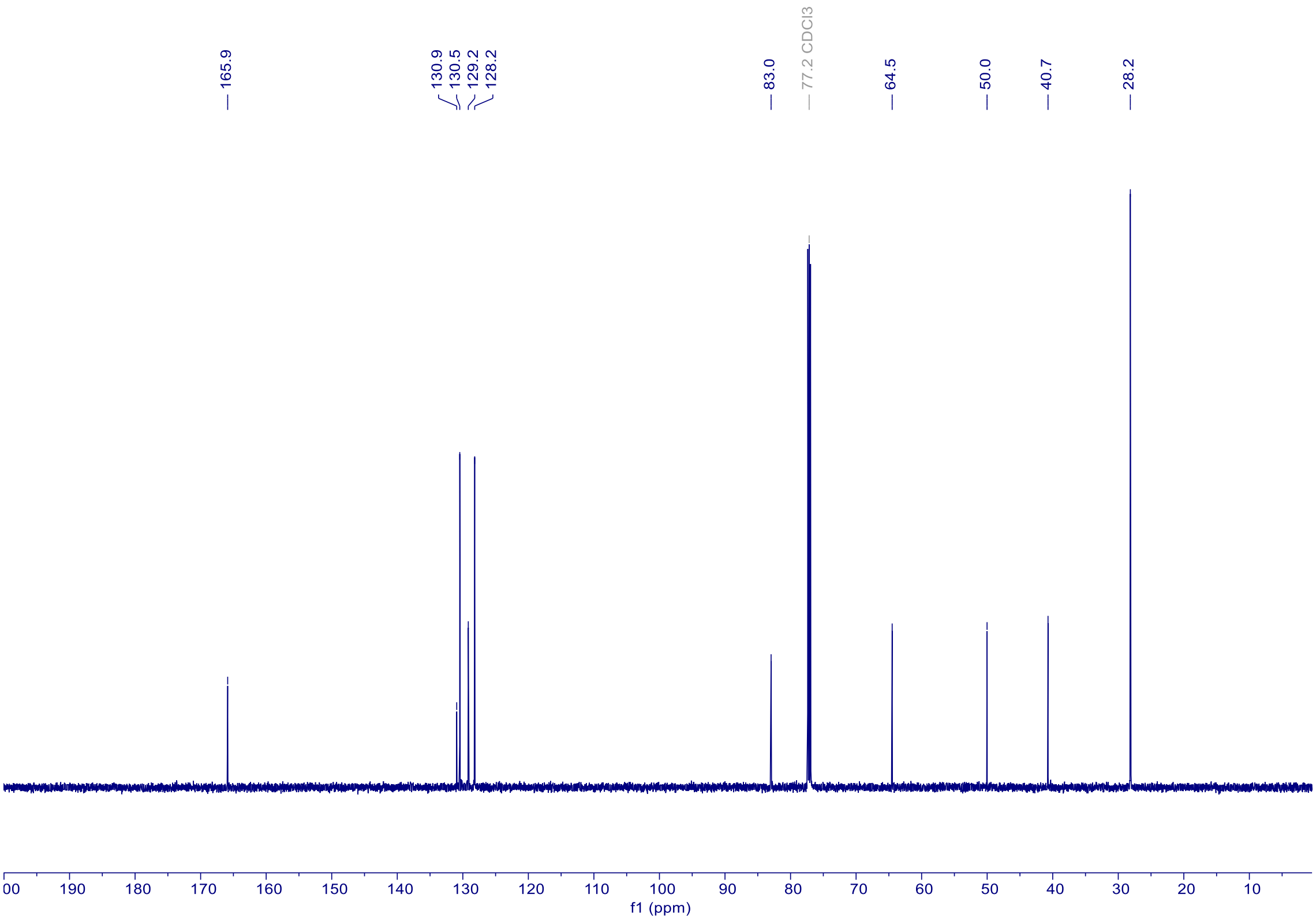

24a' – ^{11}B NMR (193 MHz, $CDCl_3$)

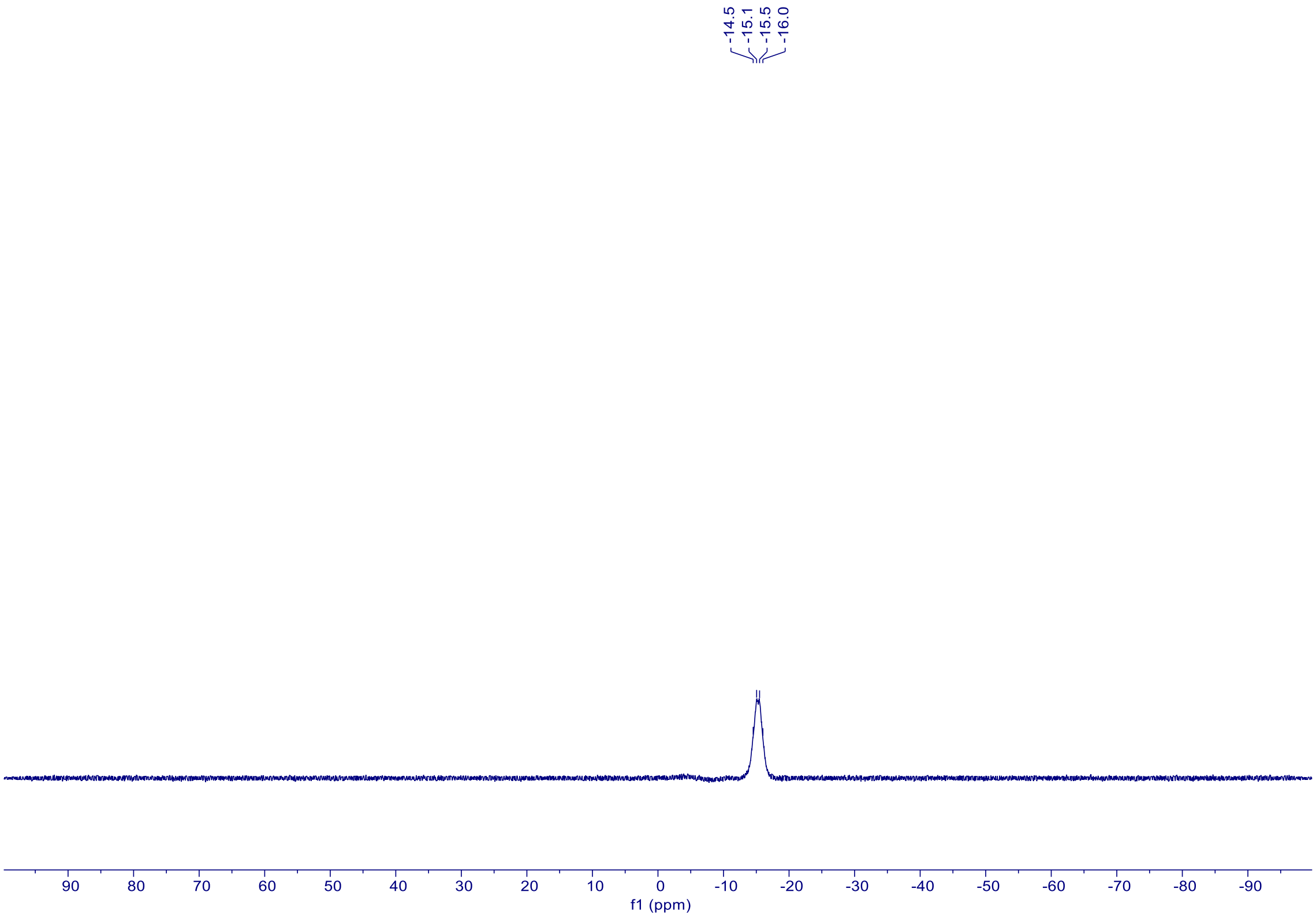

25a – ^{1}H NMR (600 MHz, $CDCl_3$)

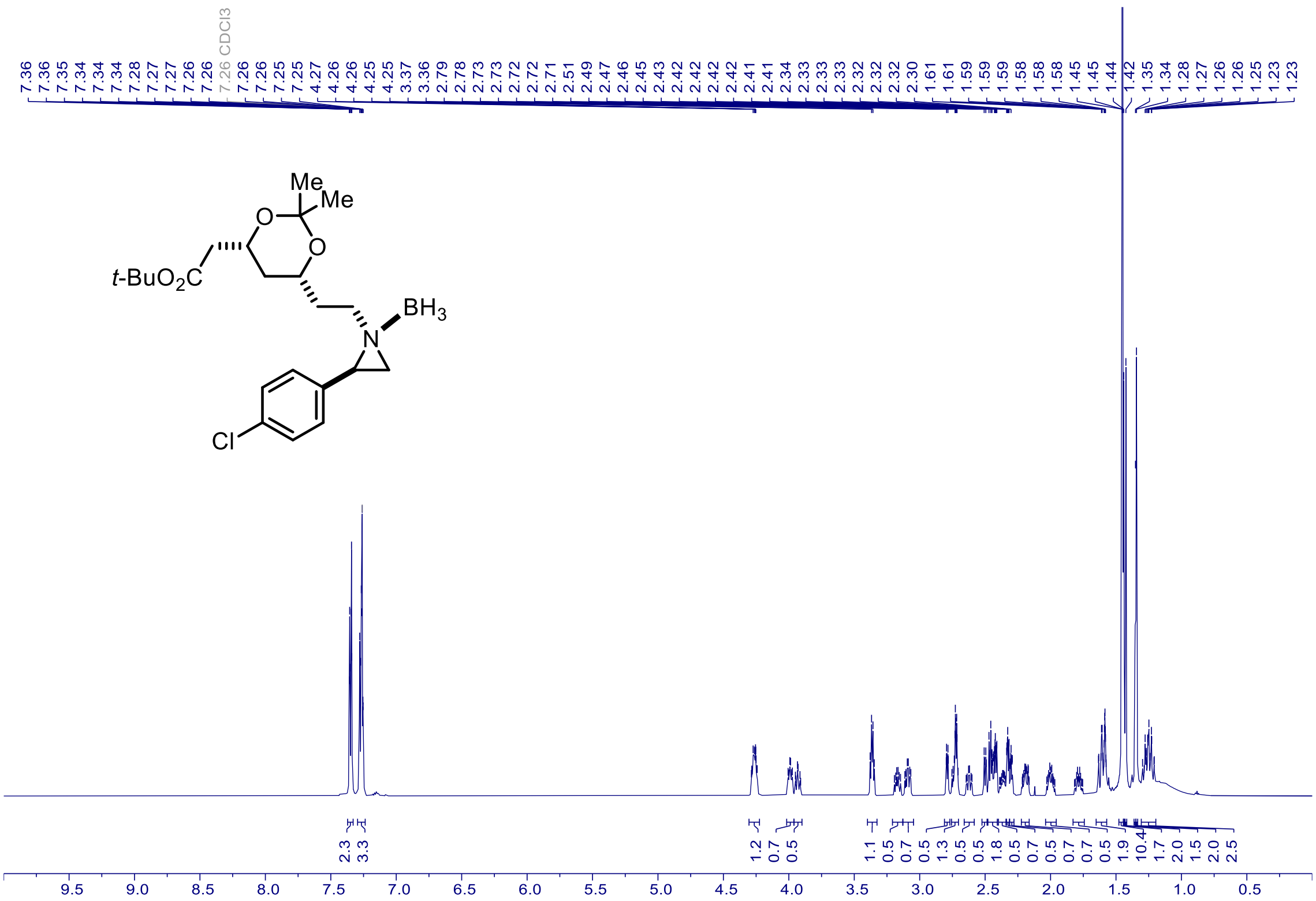

25a – ^{13}C NMR (151 MHz, $CDCl_3$)

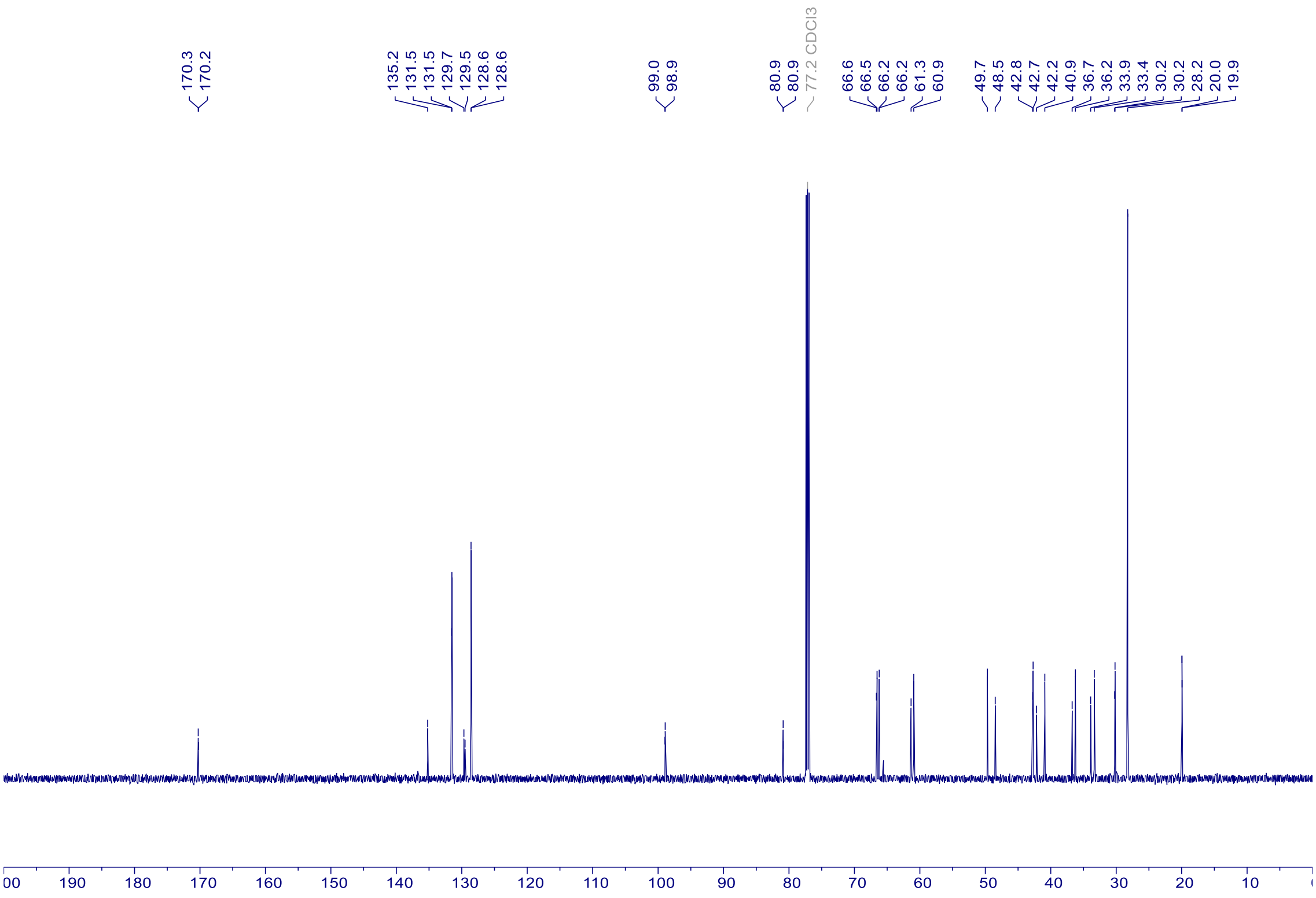

25a – ^{11}B NMR (192 MHz, $CDCl_3$)

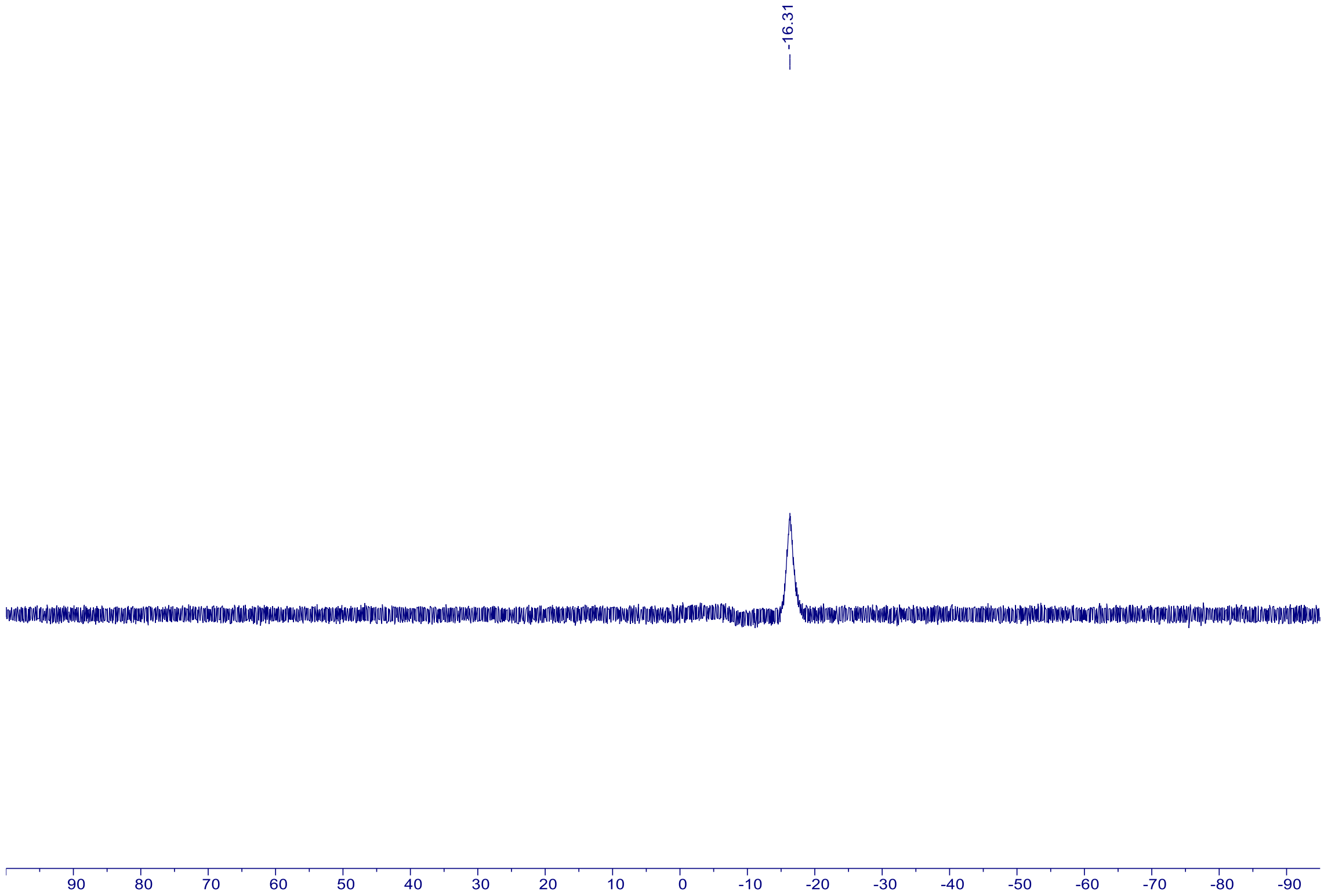

26a – ^{1}H NMR (600 MHz, $CDCl_3$)

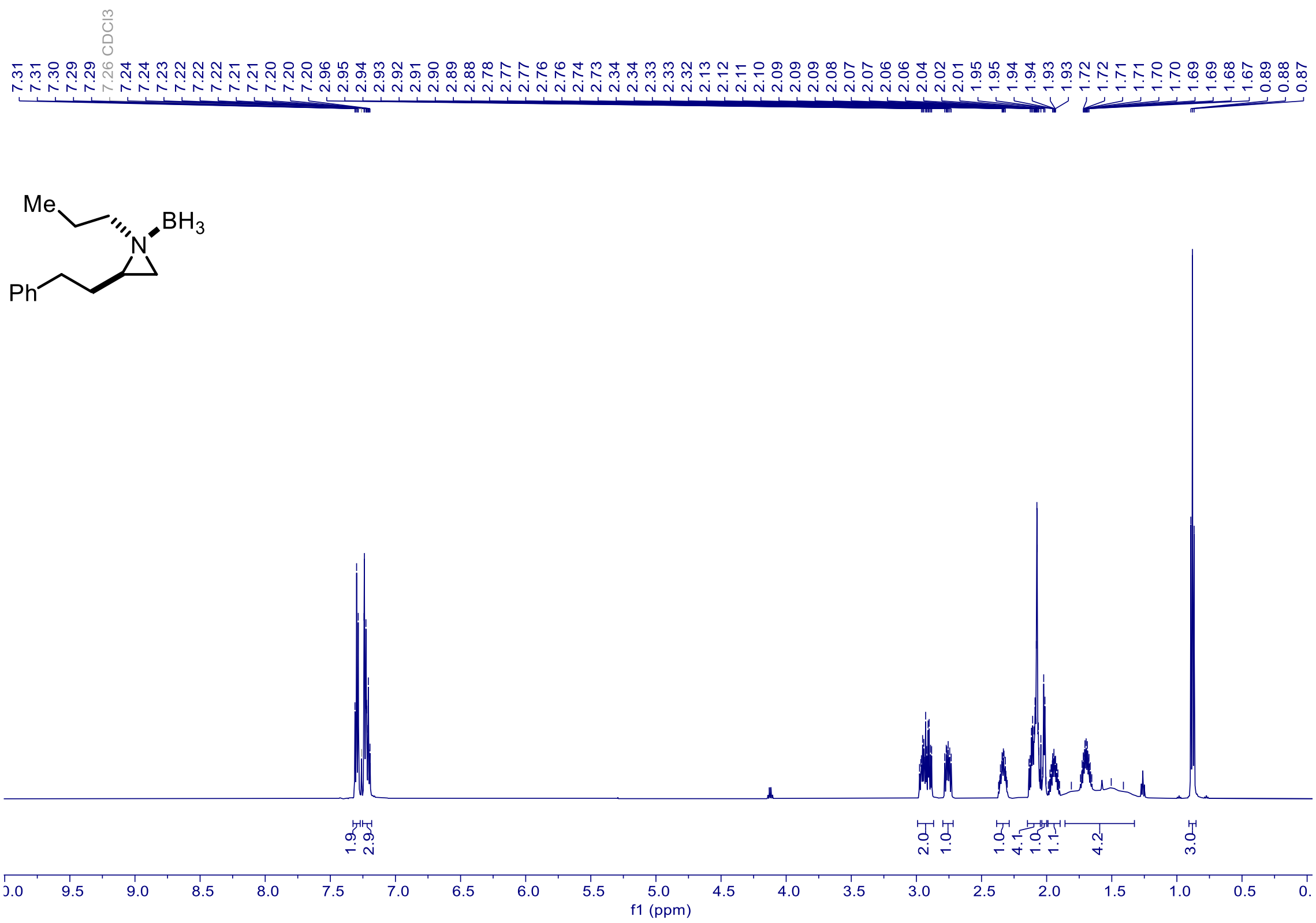

26a – ^{13}C NMR (151 MHz, $CDCl_3$)

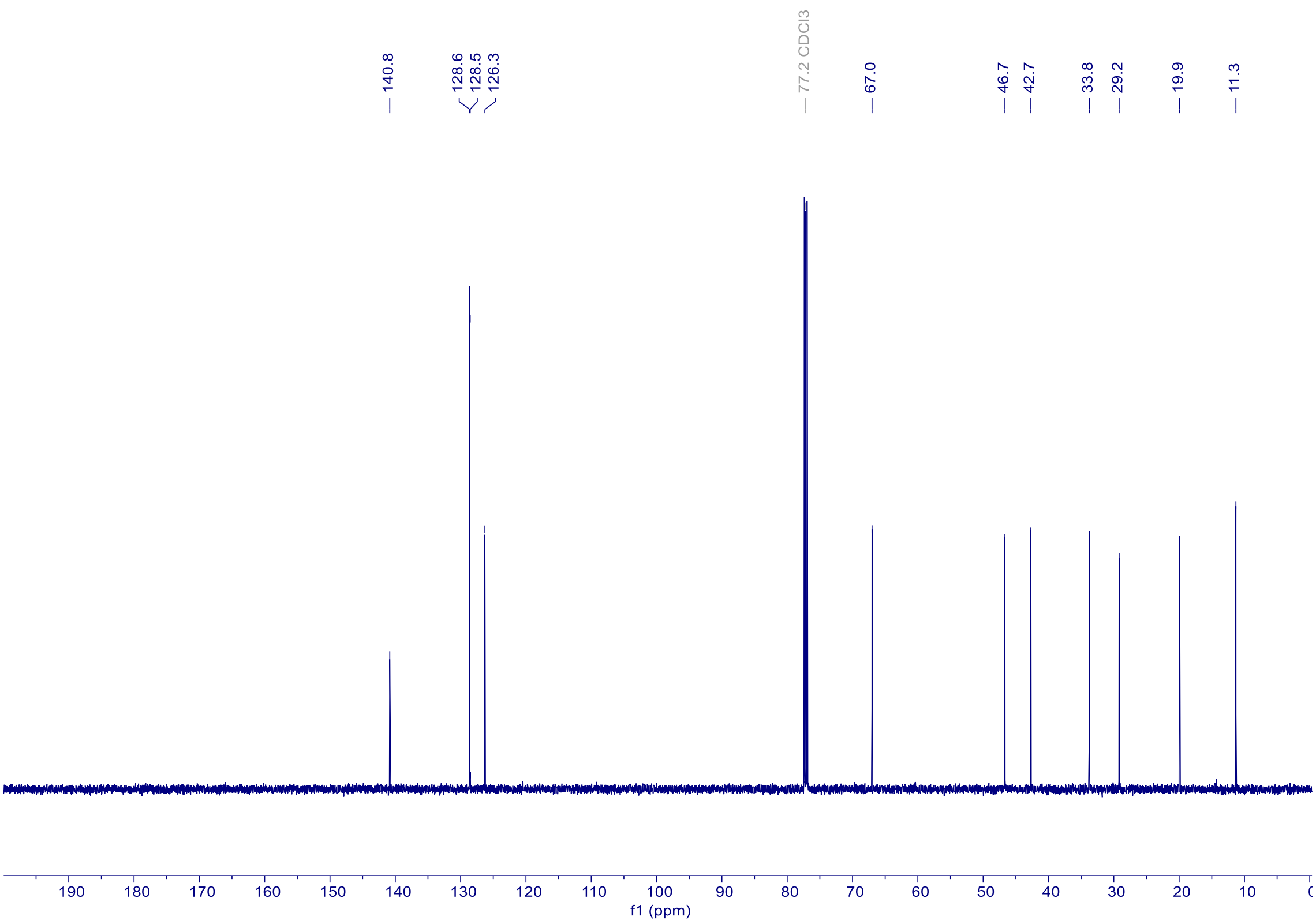

26a – ^{11}B NMR (193 MHz, $CDCl_3$)

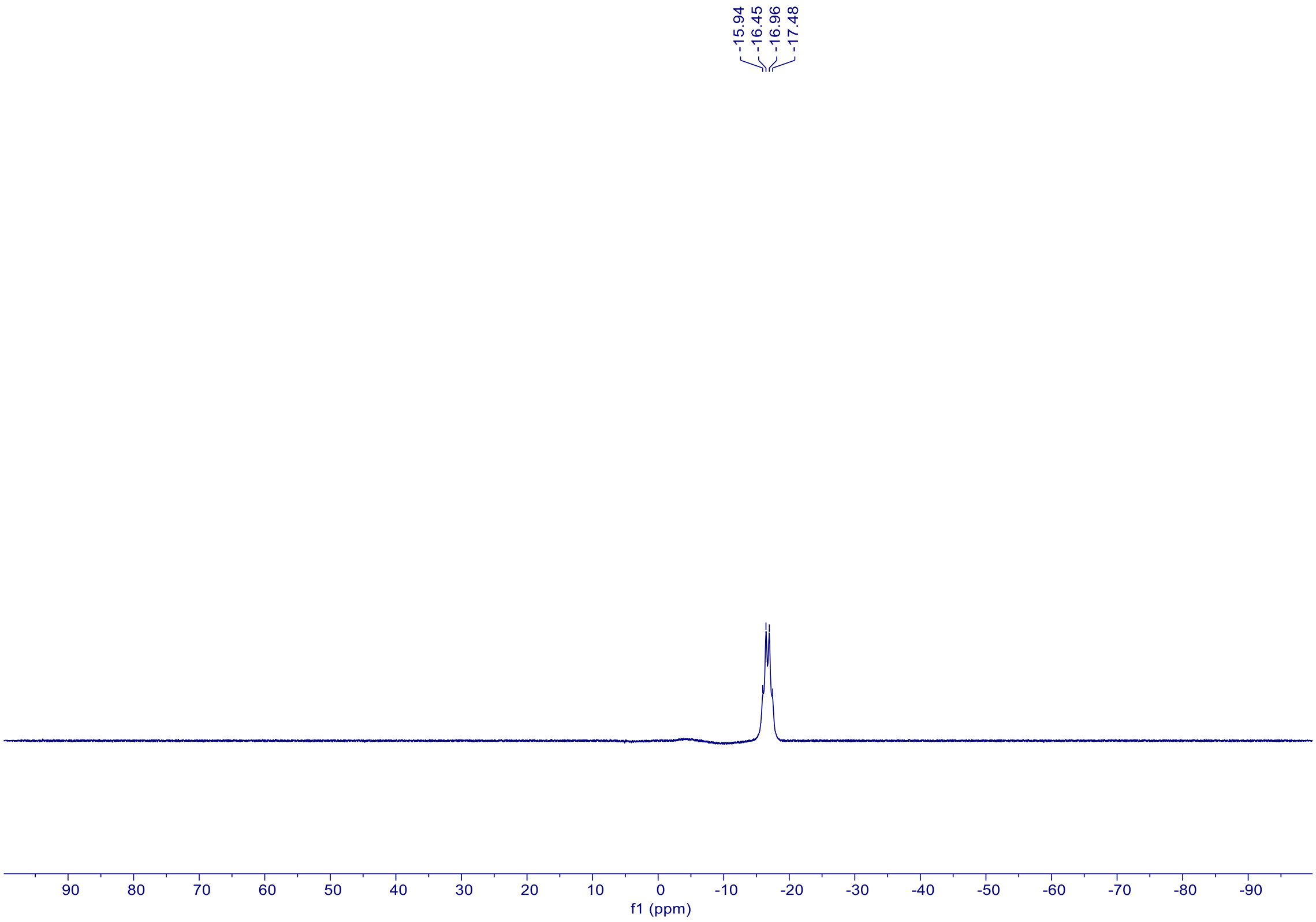

27a – ^{1}H NMR (600 MHz, $CDCl_3$)

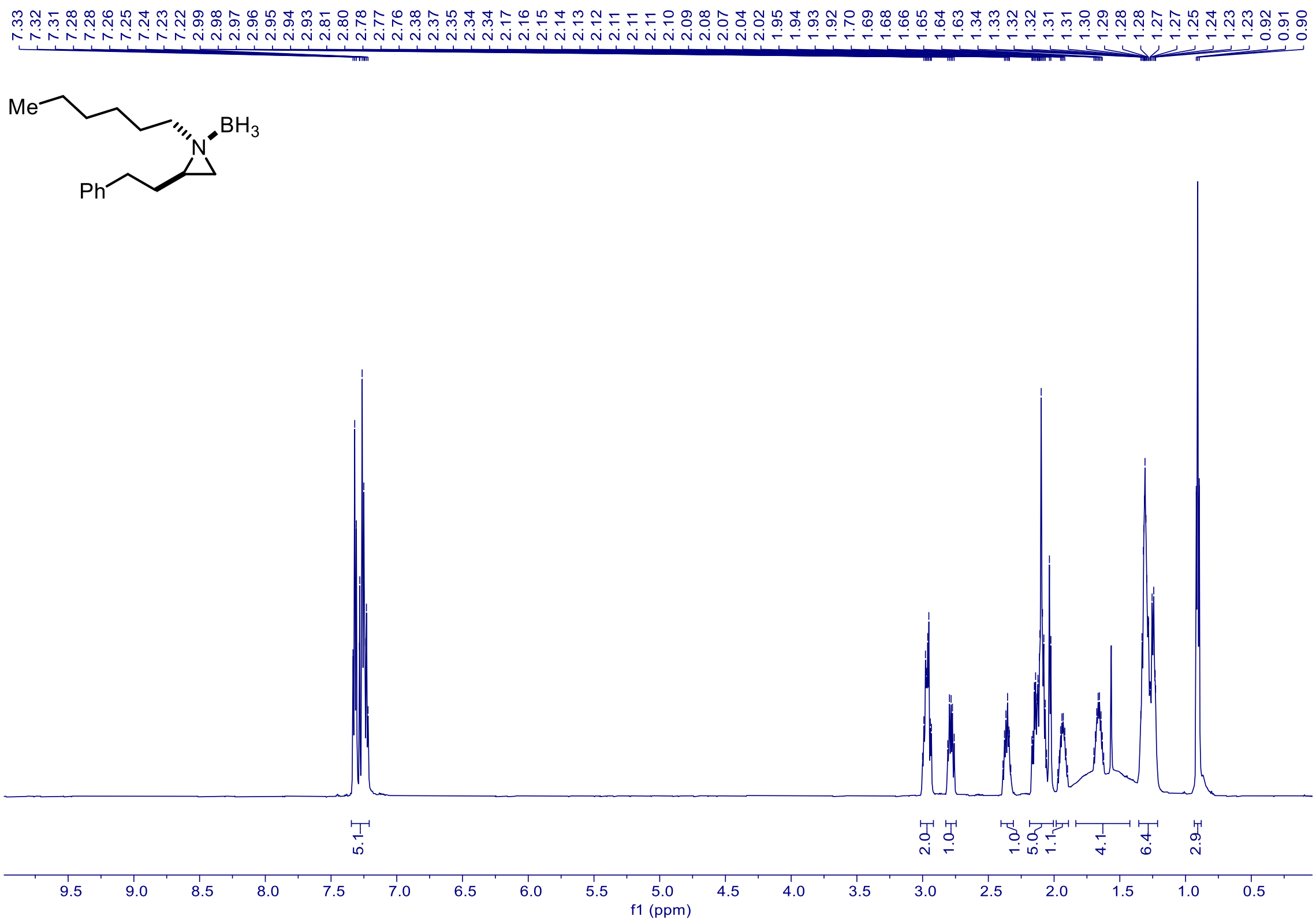

27a – ^{13}C NMR (151 MHz, $CDCl_3$)

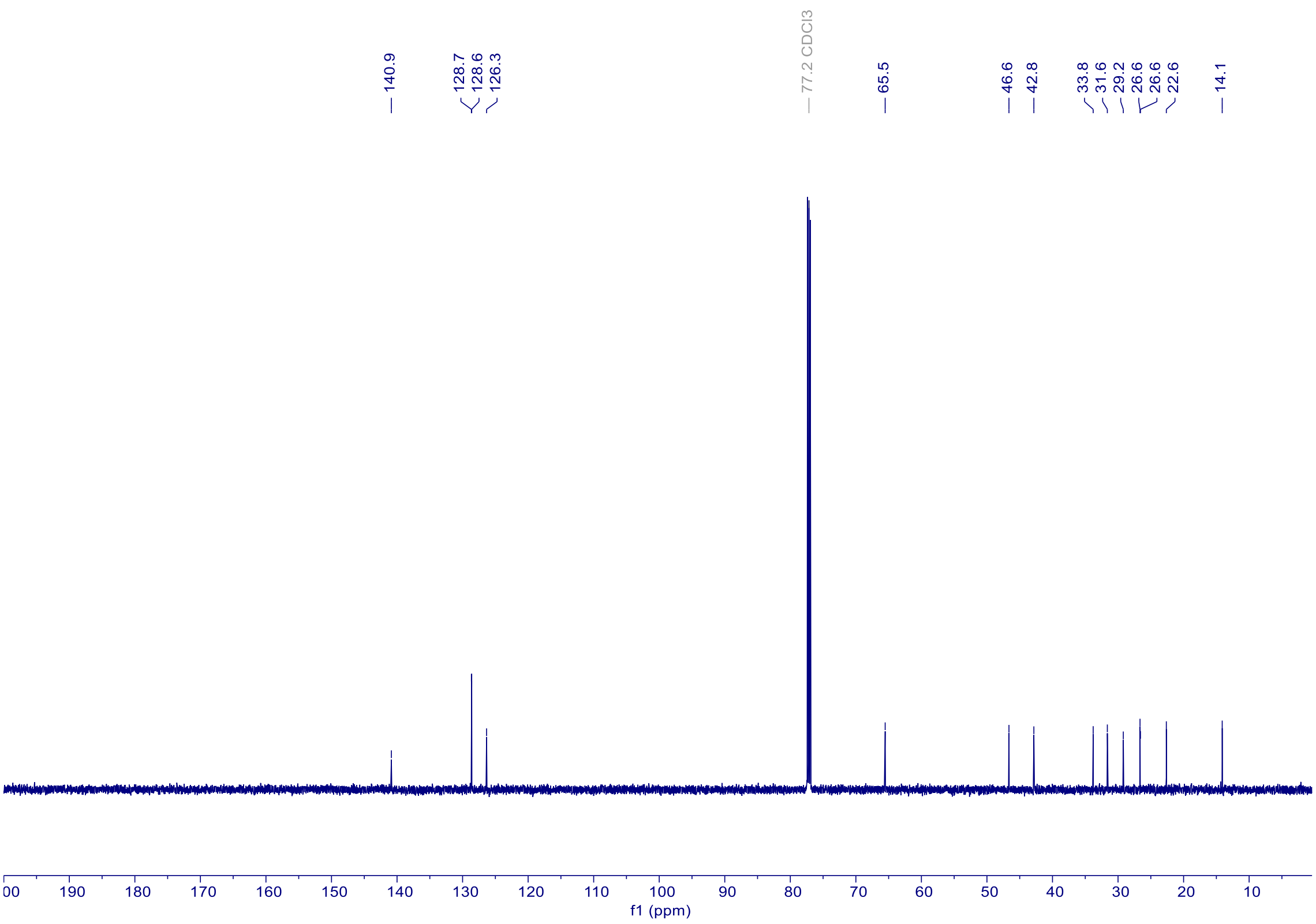

27a – ^{11}B NMR (193 MHz, $CDCl_3$)

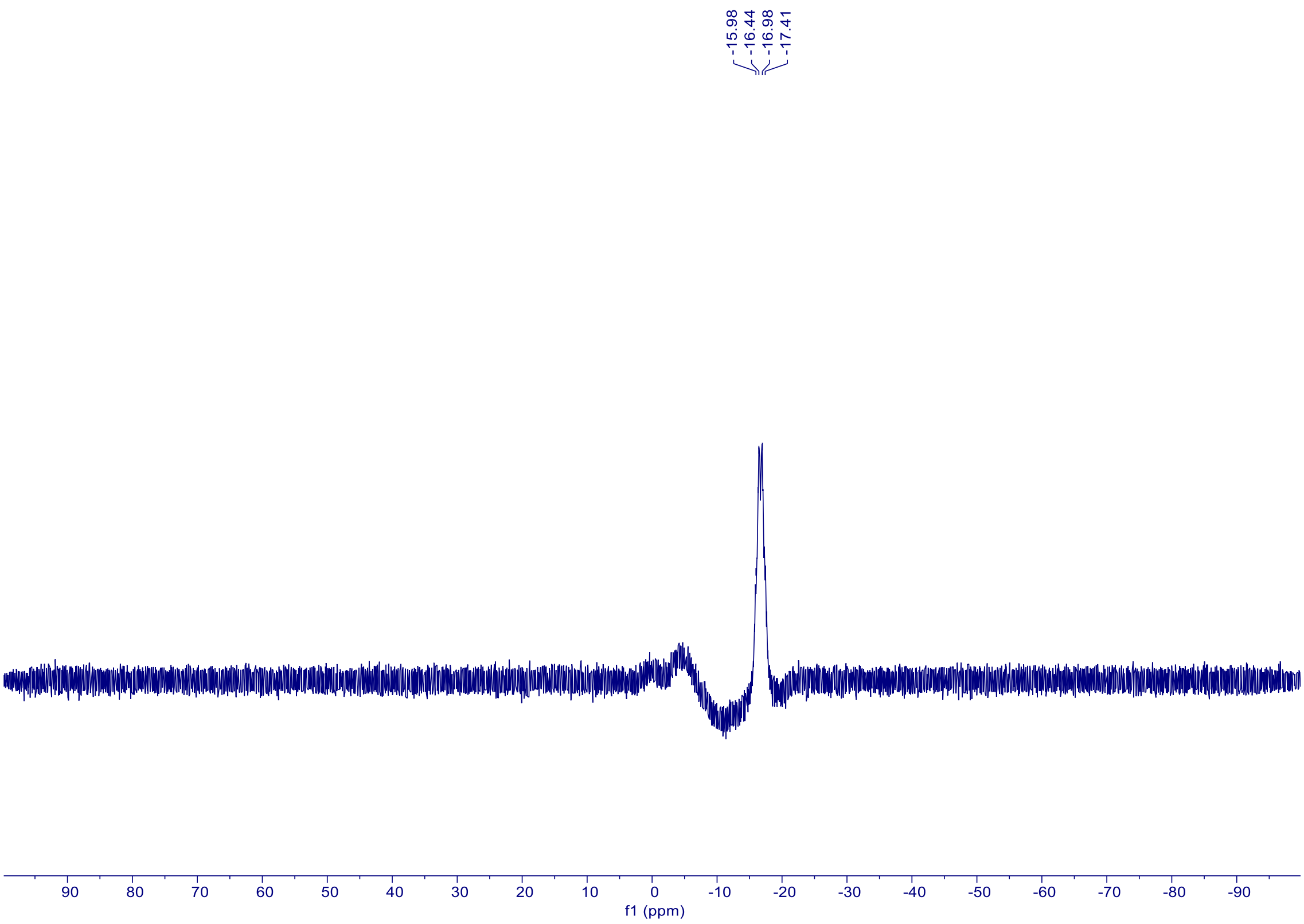

28a – ^{1}H NMR (600 MHz, $CDCl_3$)

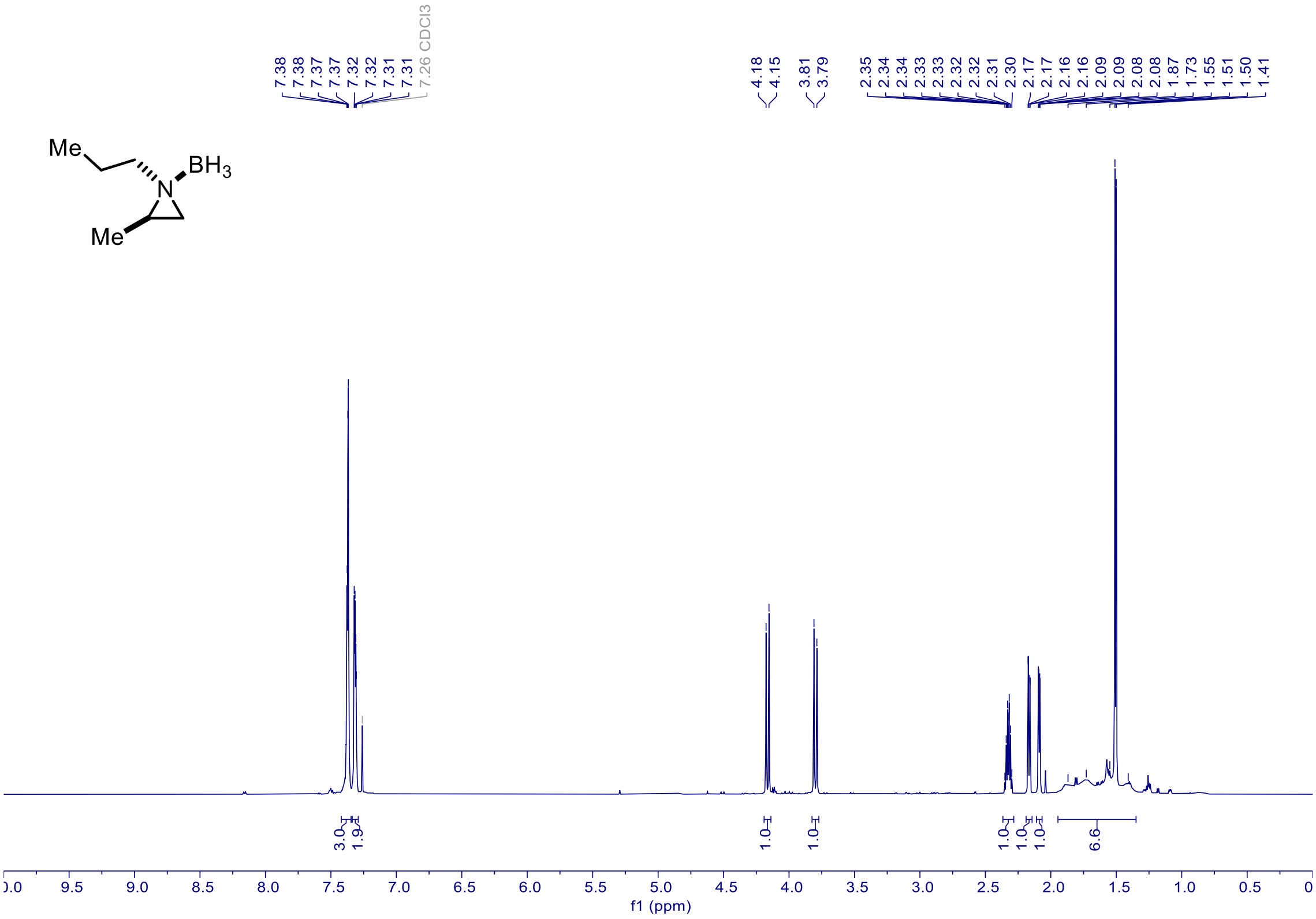

28a – ^{13}C NMR (151 MHz, $CDCl_3$)

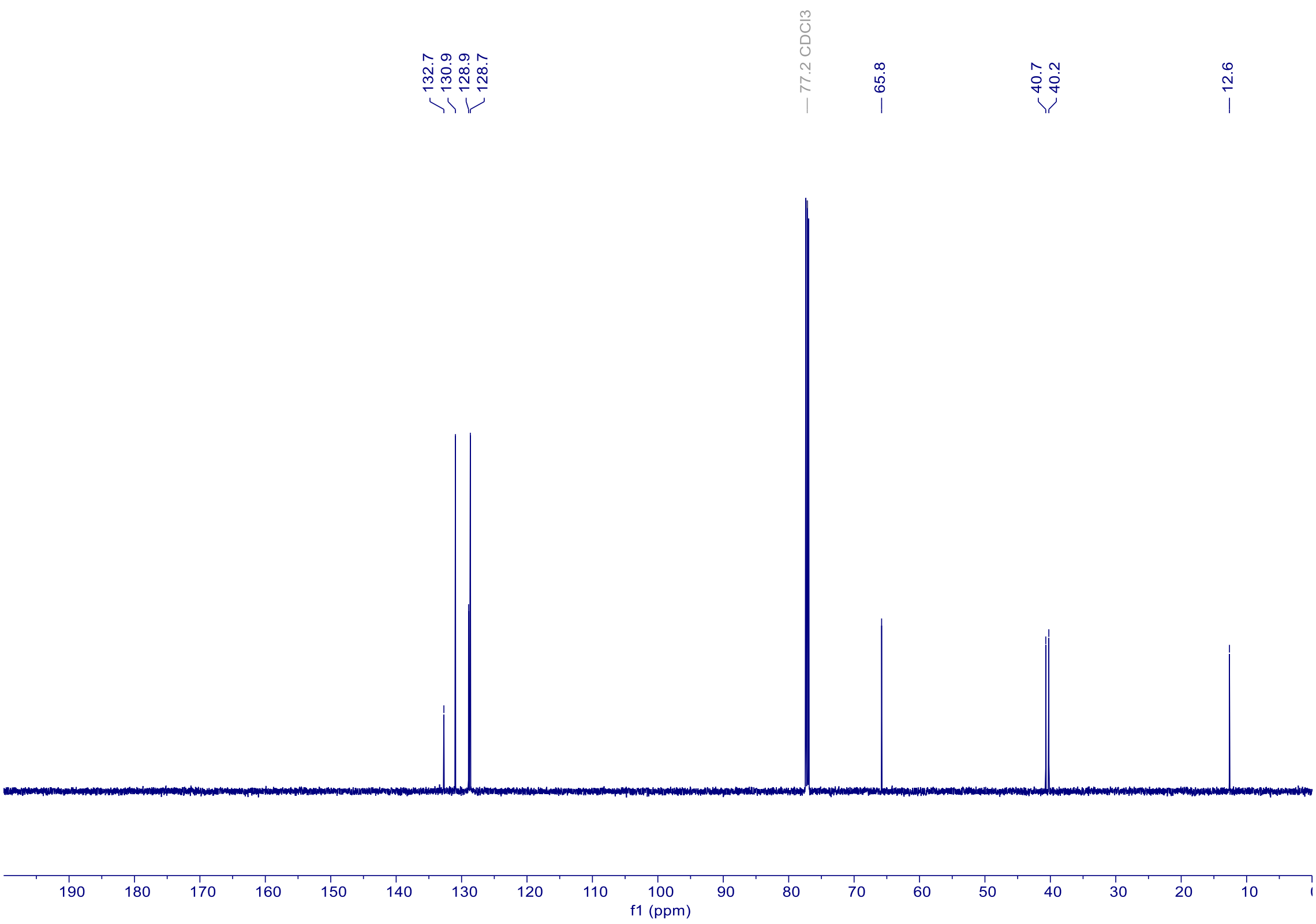

28a – ^{11}B NMR (193 MHz, $CDCl_3$)

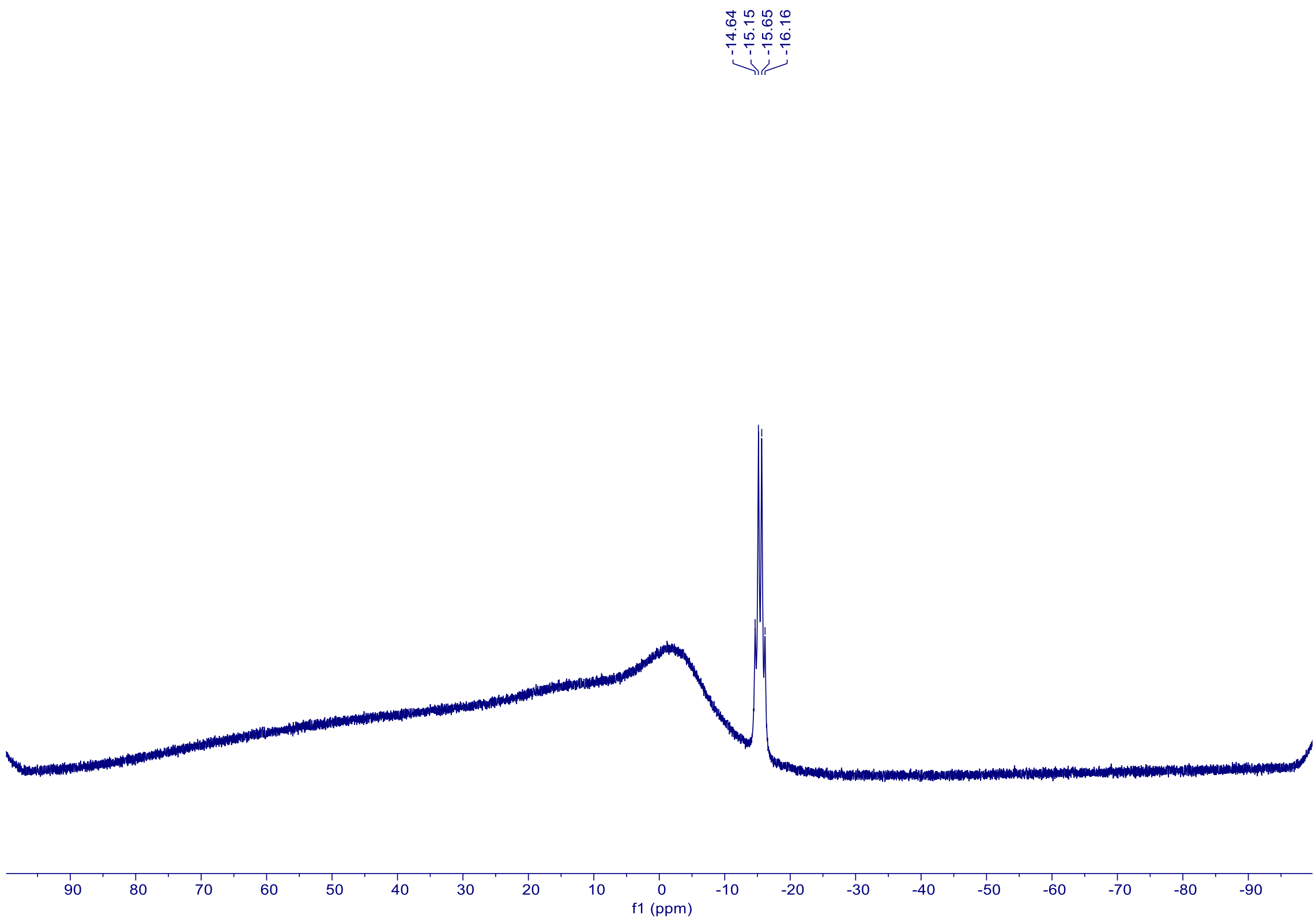

29a – ^{1}H NMR (600 MHz, $CDCl_3$)

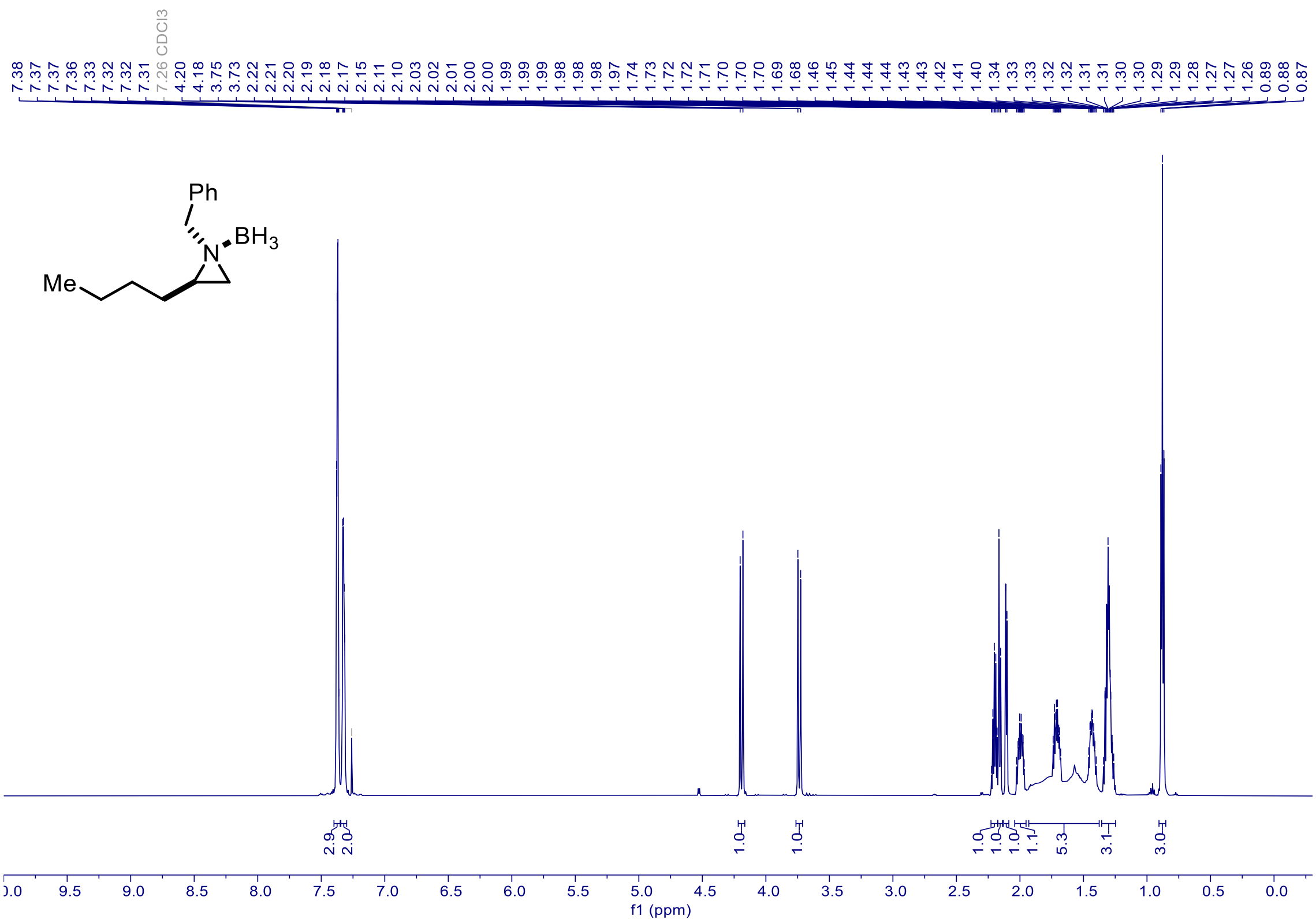

29a – ^{13}C NMR (151 MHz, $CDCl_3$)

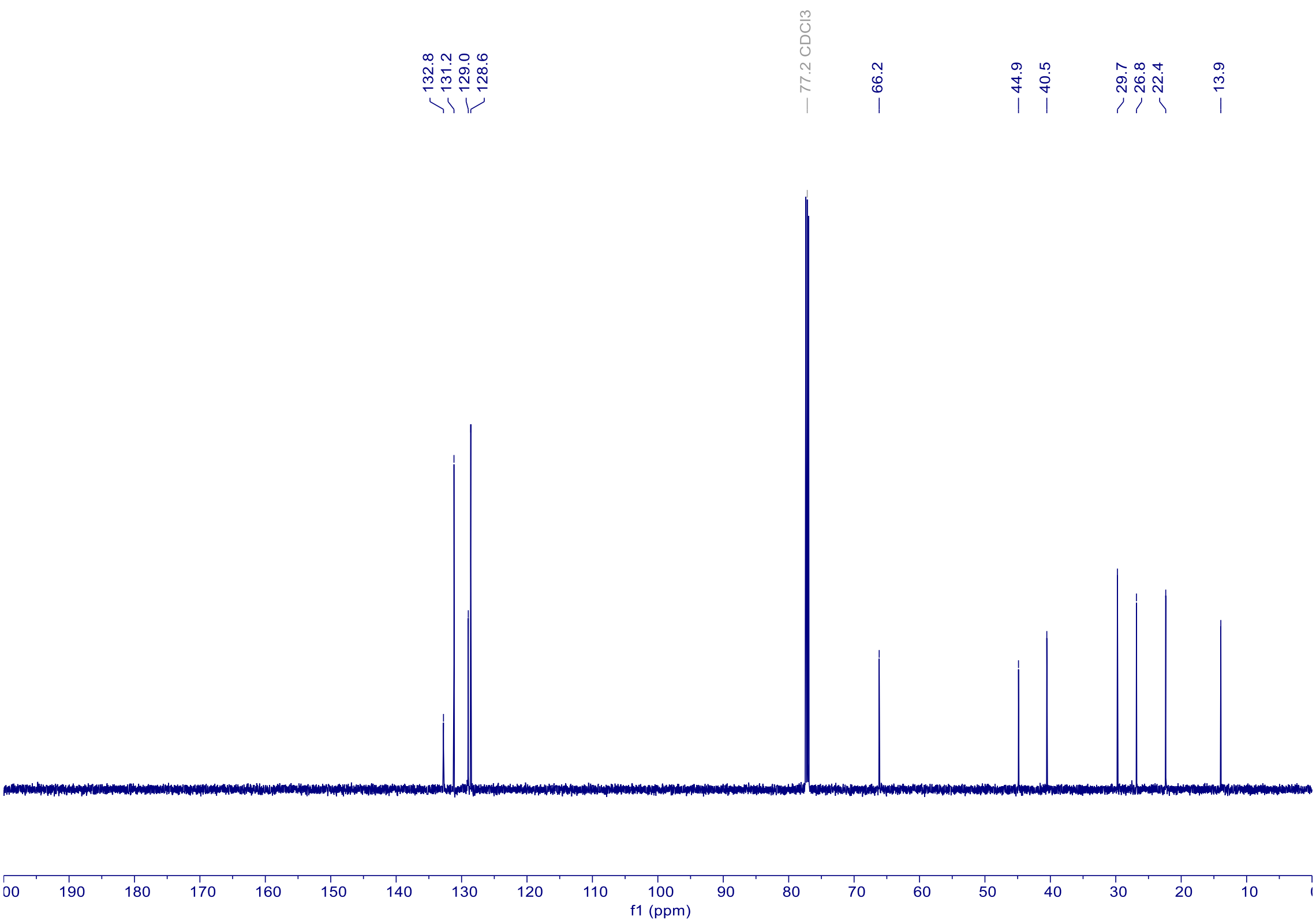

29a – ^{11}B NMR (193 MHz, $CDCl_3$)

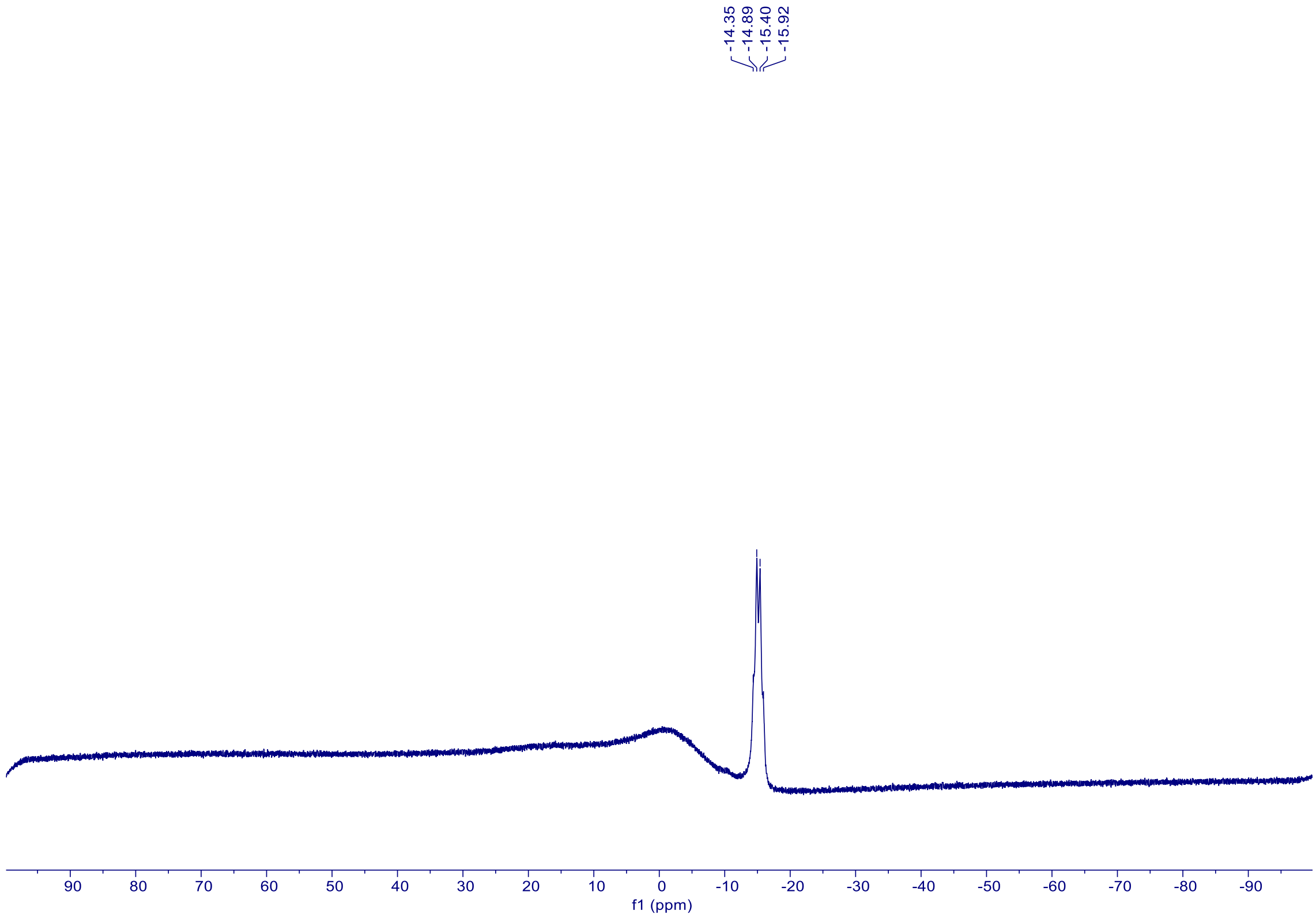

30a – ^{1}H NMR (600 MHz, $CDCl_3$)

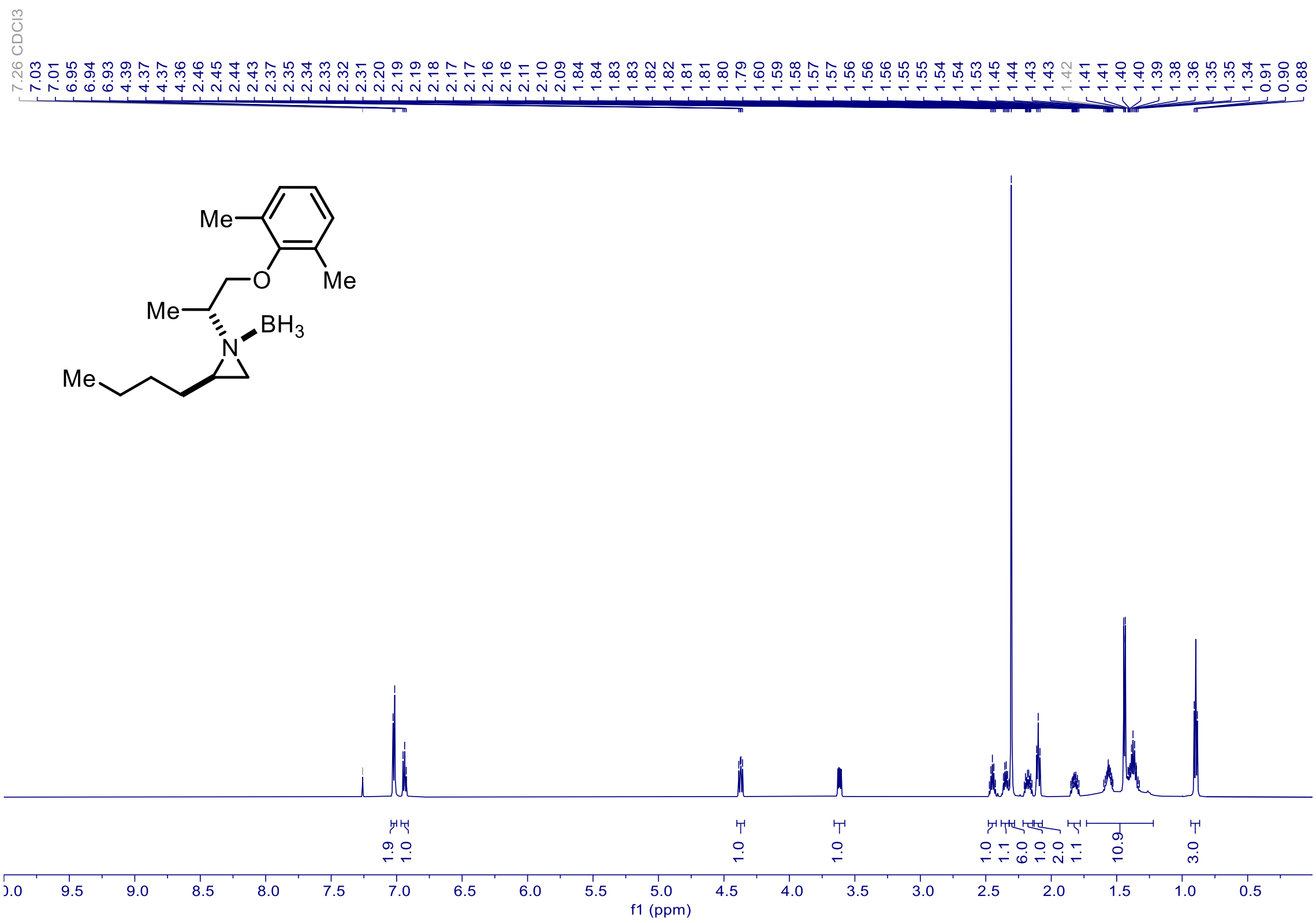

30a – ^{13}C NMR (151 MHz, $CDCl_3$)

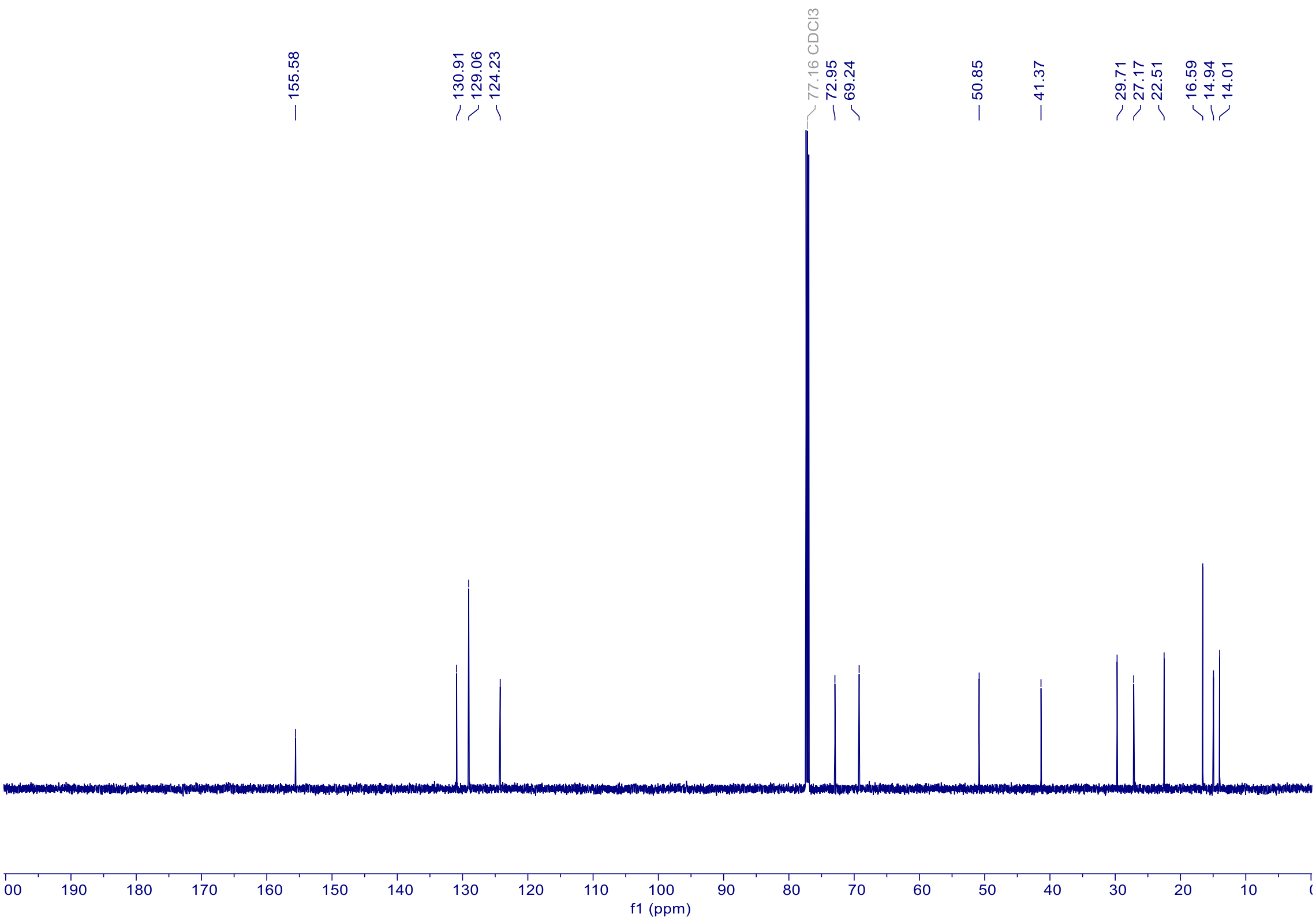

30a – ^{11}B NMR (193 MHz, $CDCl_3$)

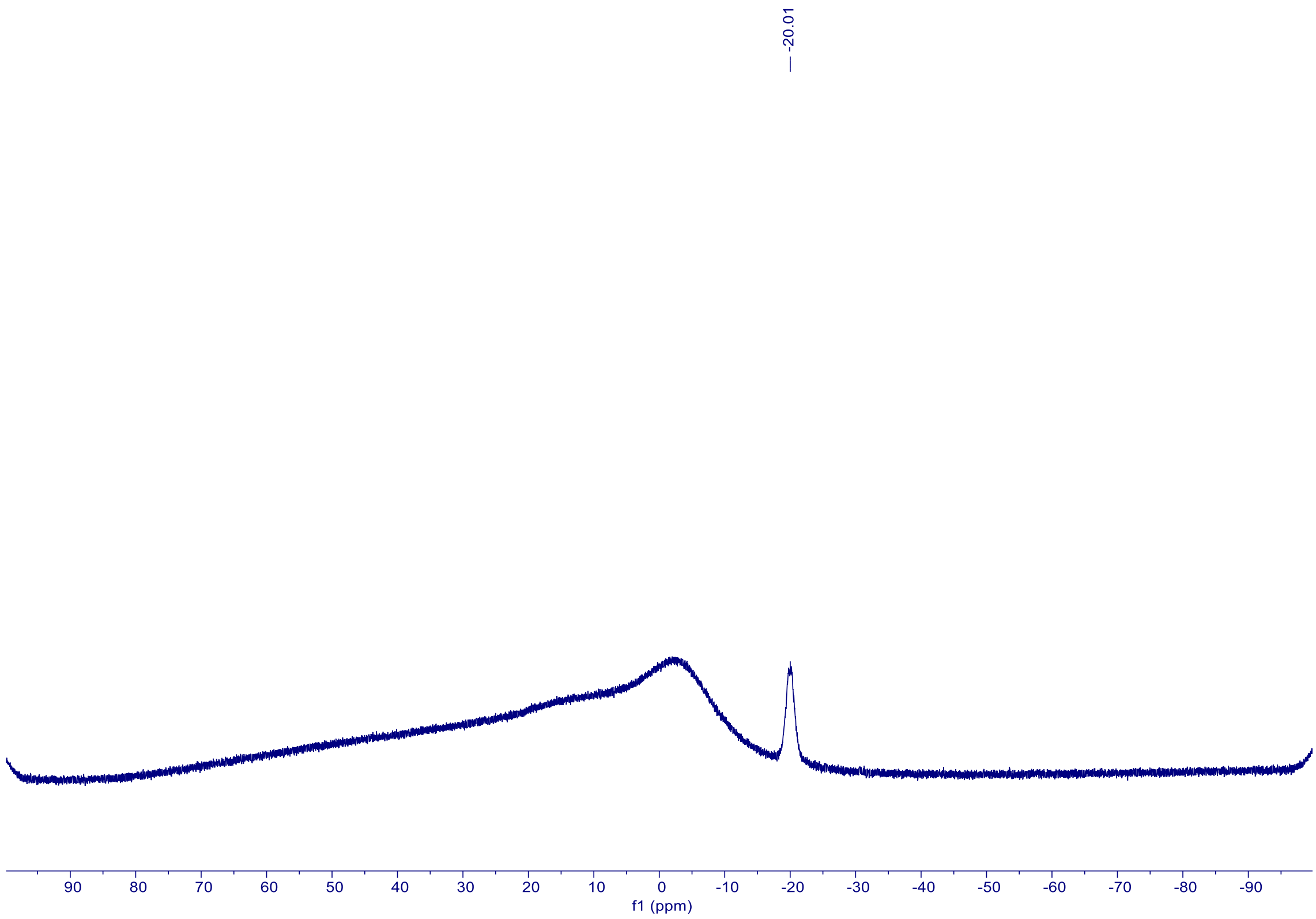

31a – ^{1}H NMR (600 MHz, $CDCl_3$)

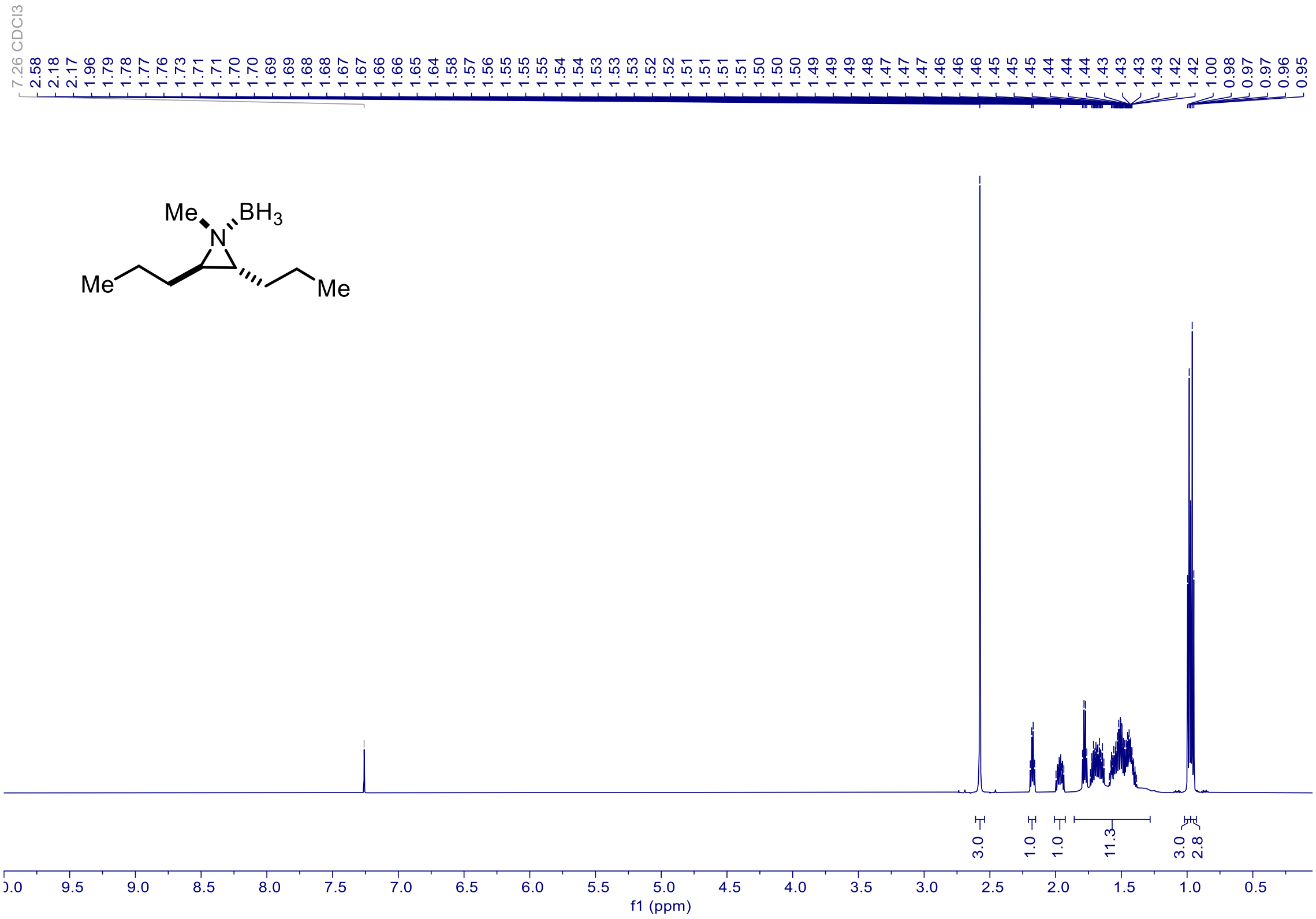

31a – ^{13}C NMR (151 MHz, $CDCl_3$)

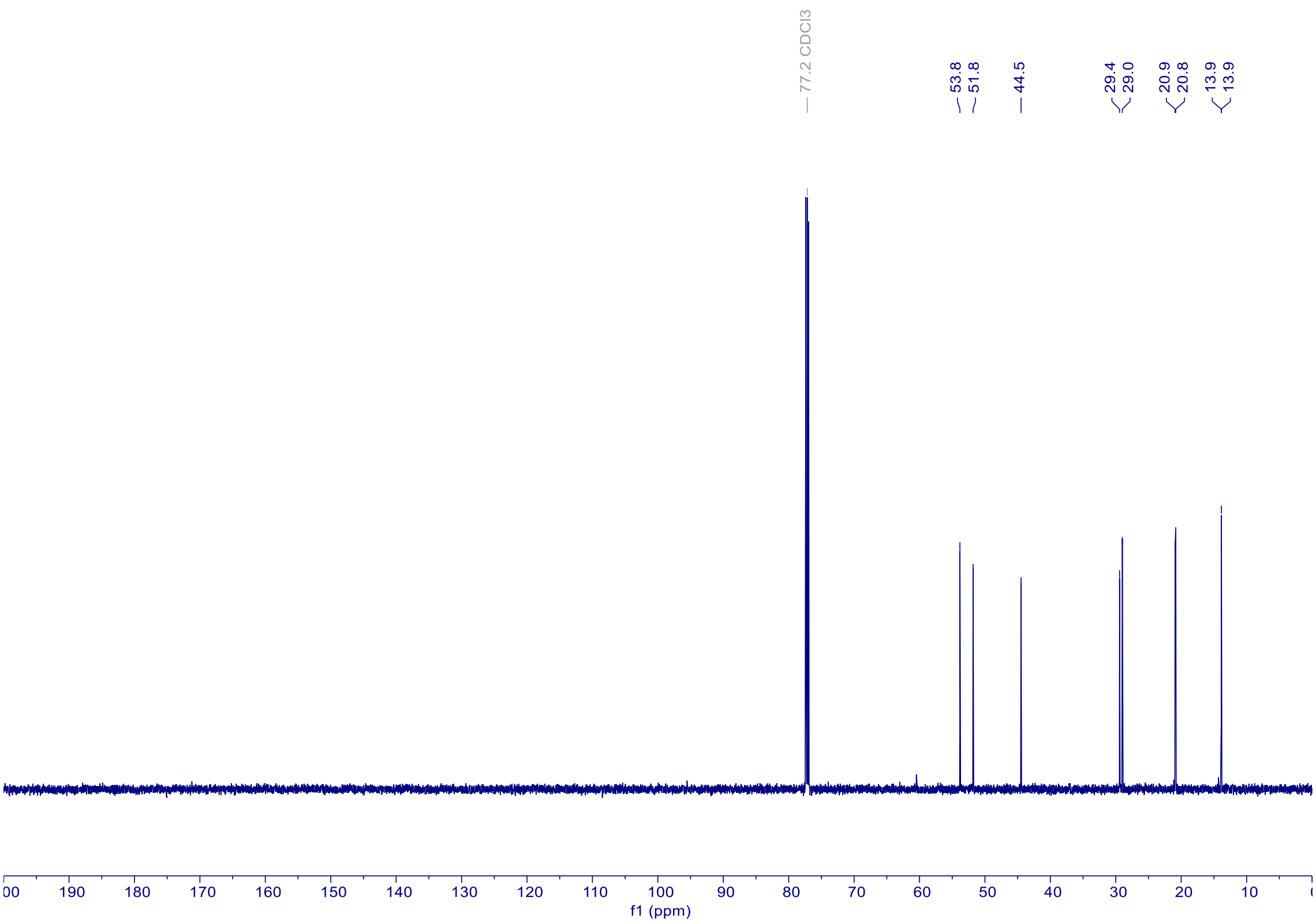

31a – ^{11}B NMR (193 MHz, $CDCl_3$)

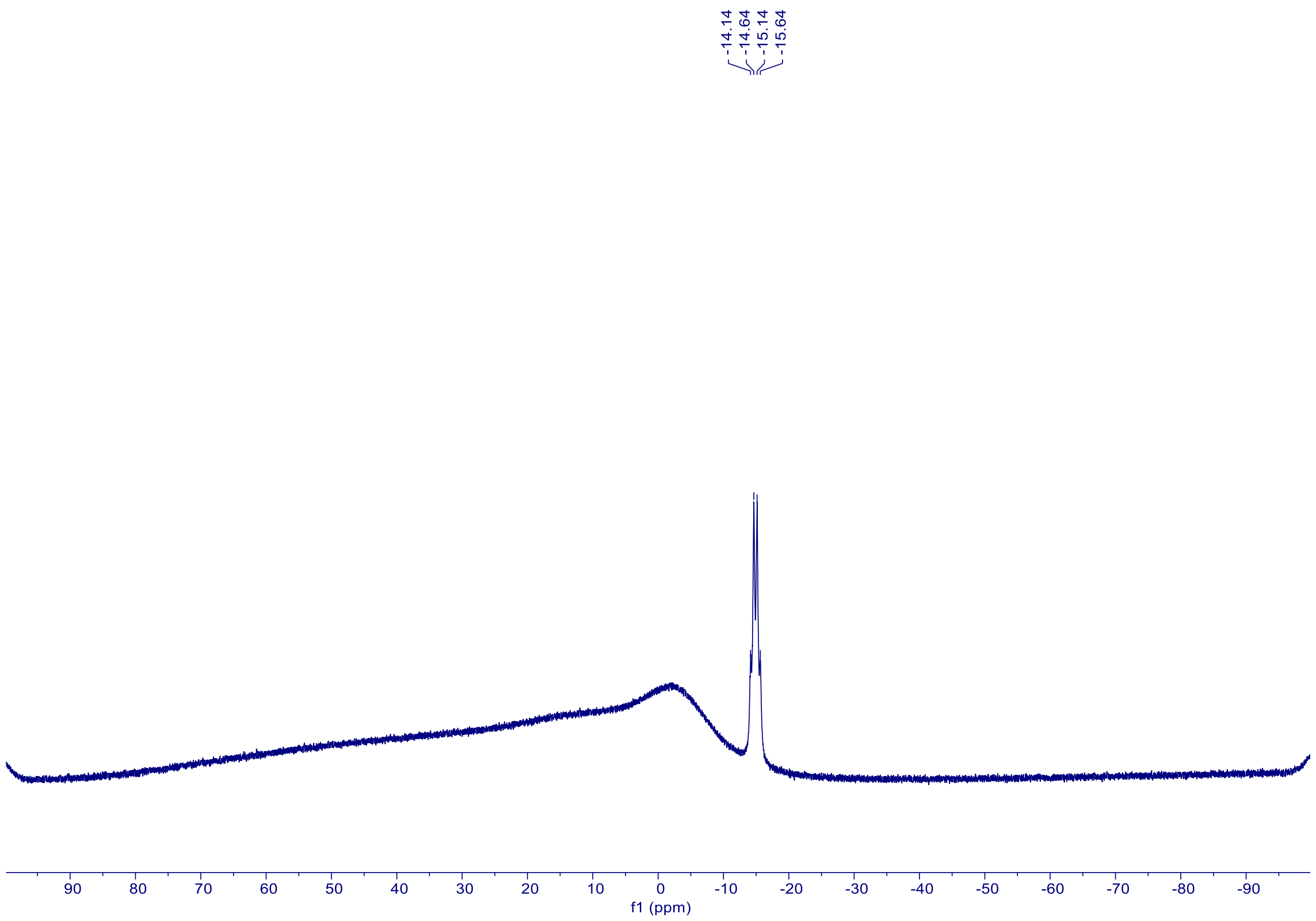

32a – ^{1}H NMR (400 MHz, $CDCl_3$)

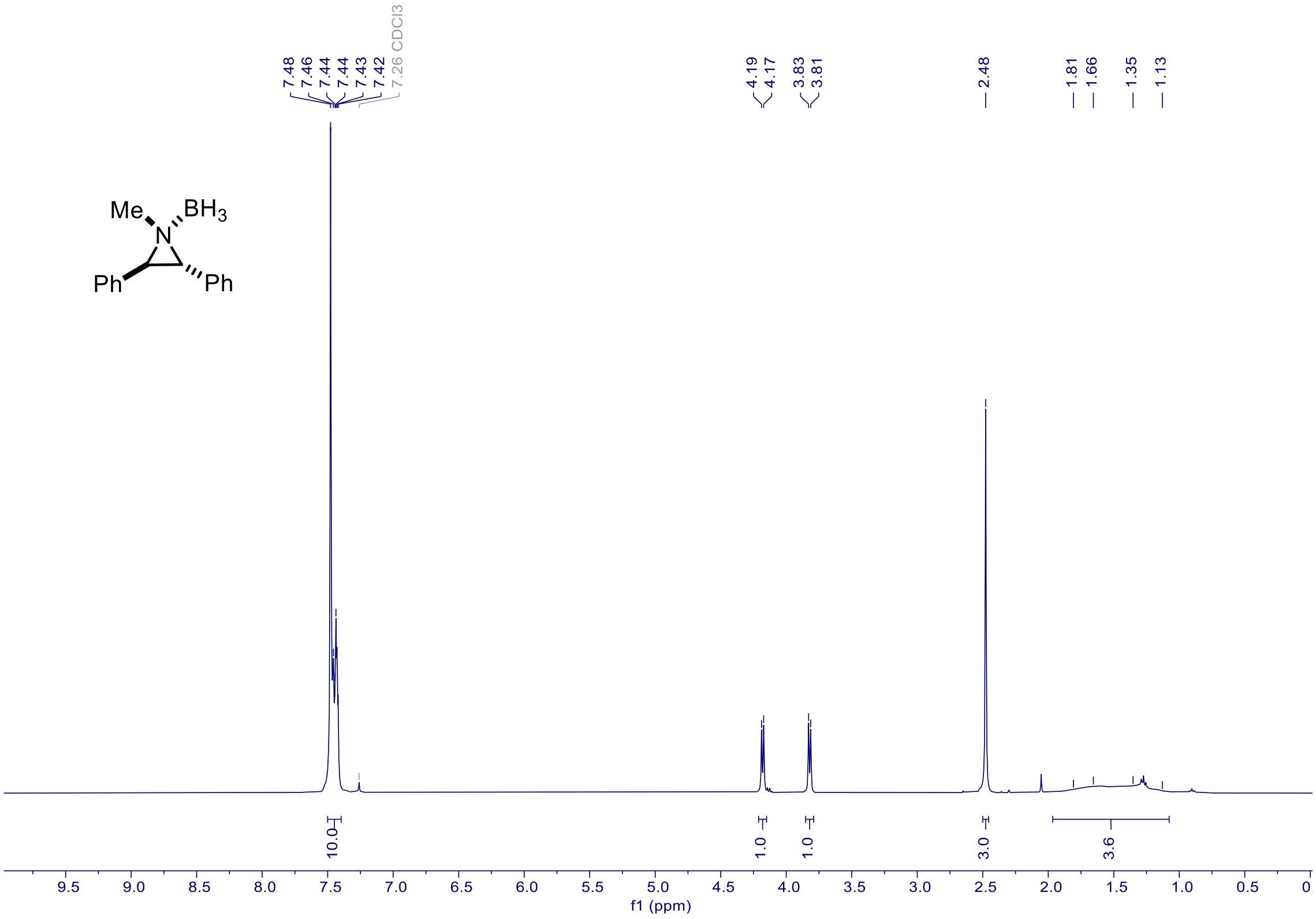

32a – ^{13}C NMR (101 MHz, $CDCl_3$)

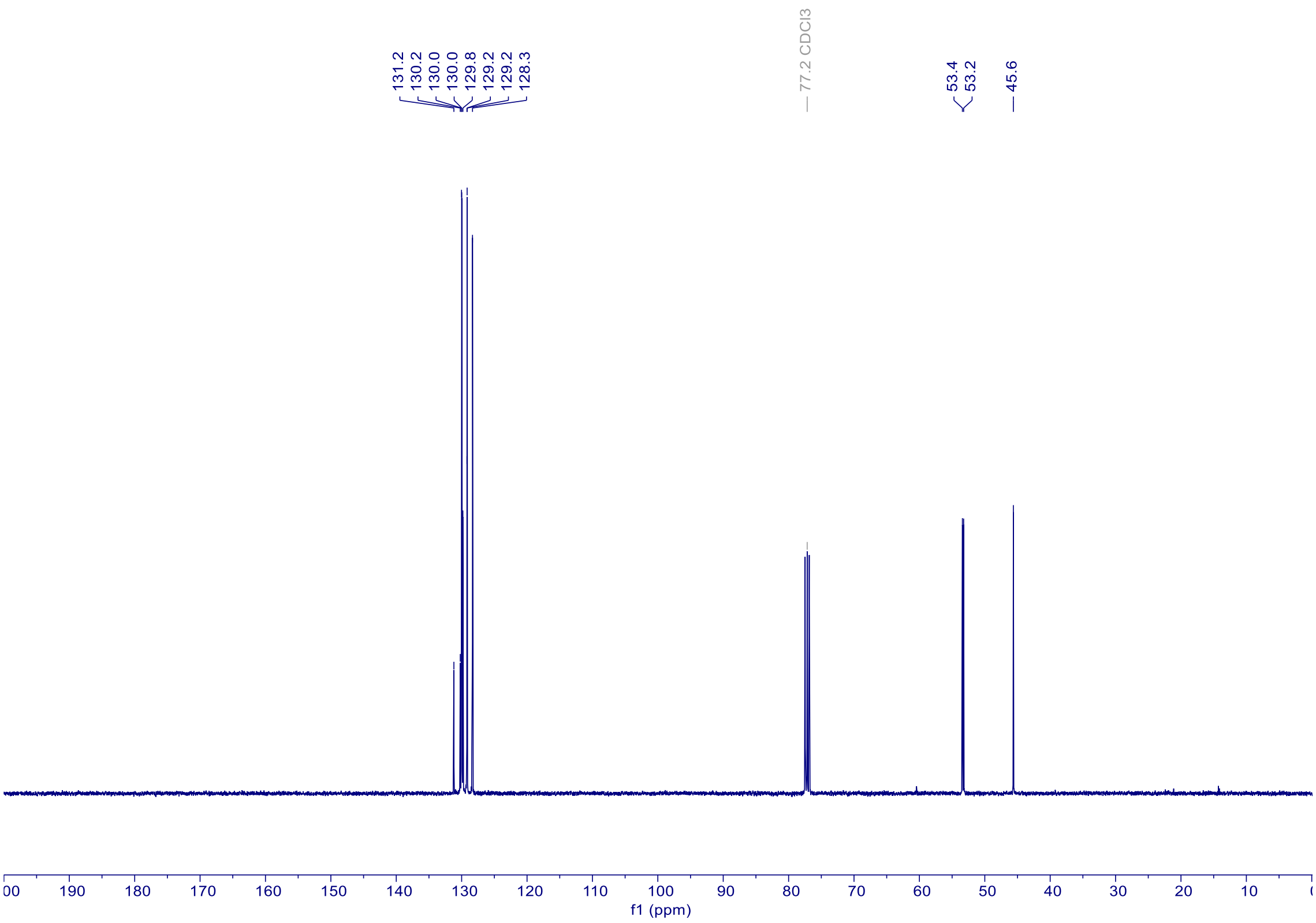

32a – ^{11}B NMR (128 MHz, $CDCl_3$)

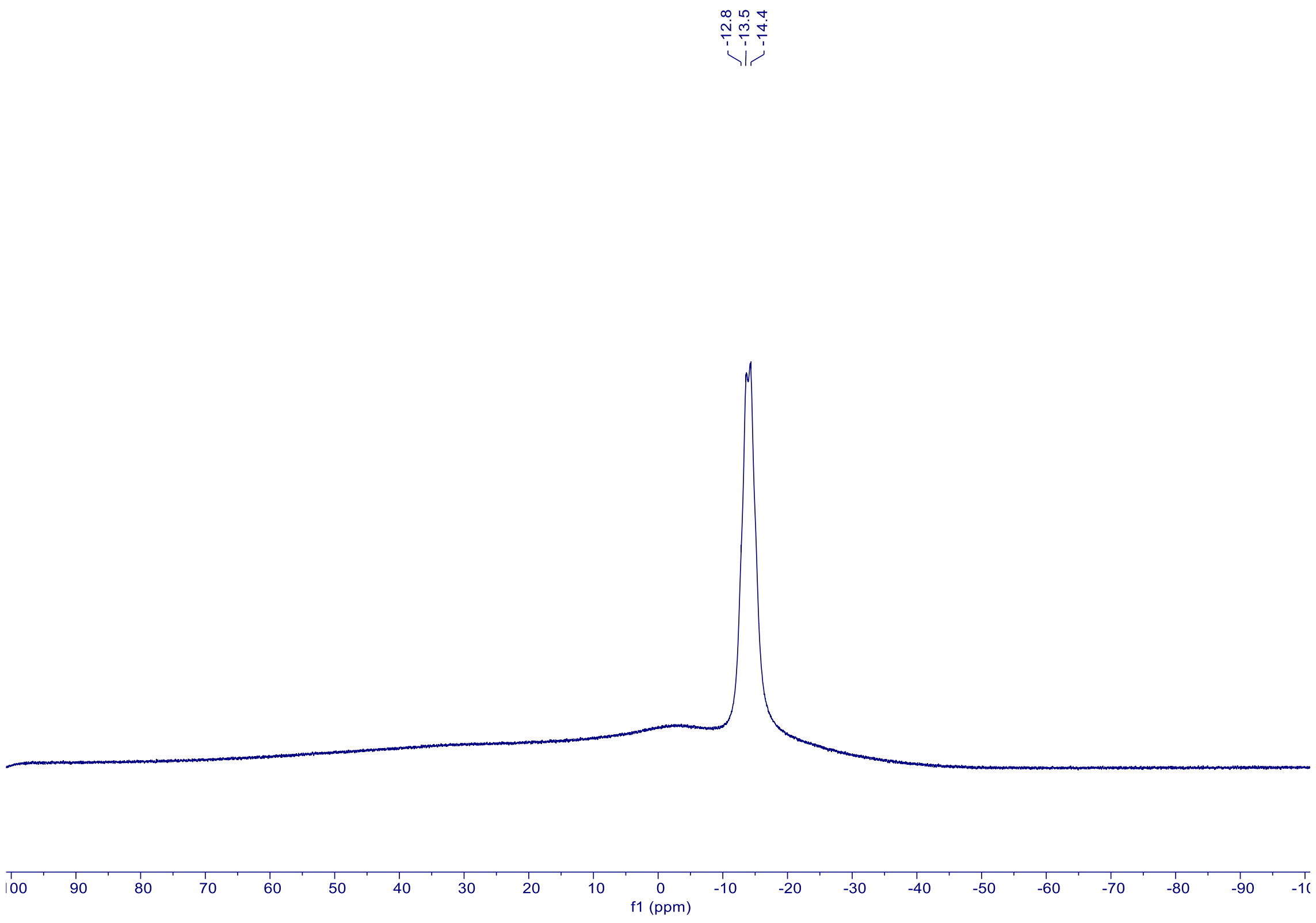

33a – 1H NMR (400 MHz, $CDCl_3$)

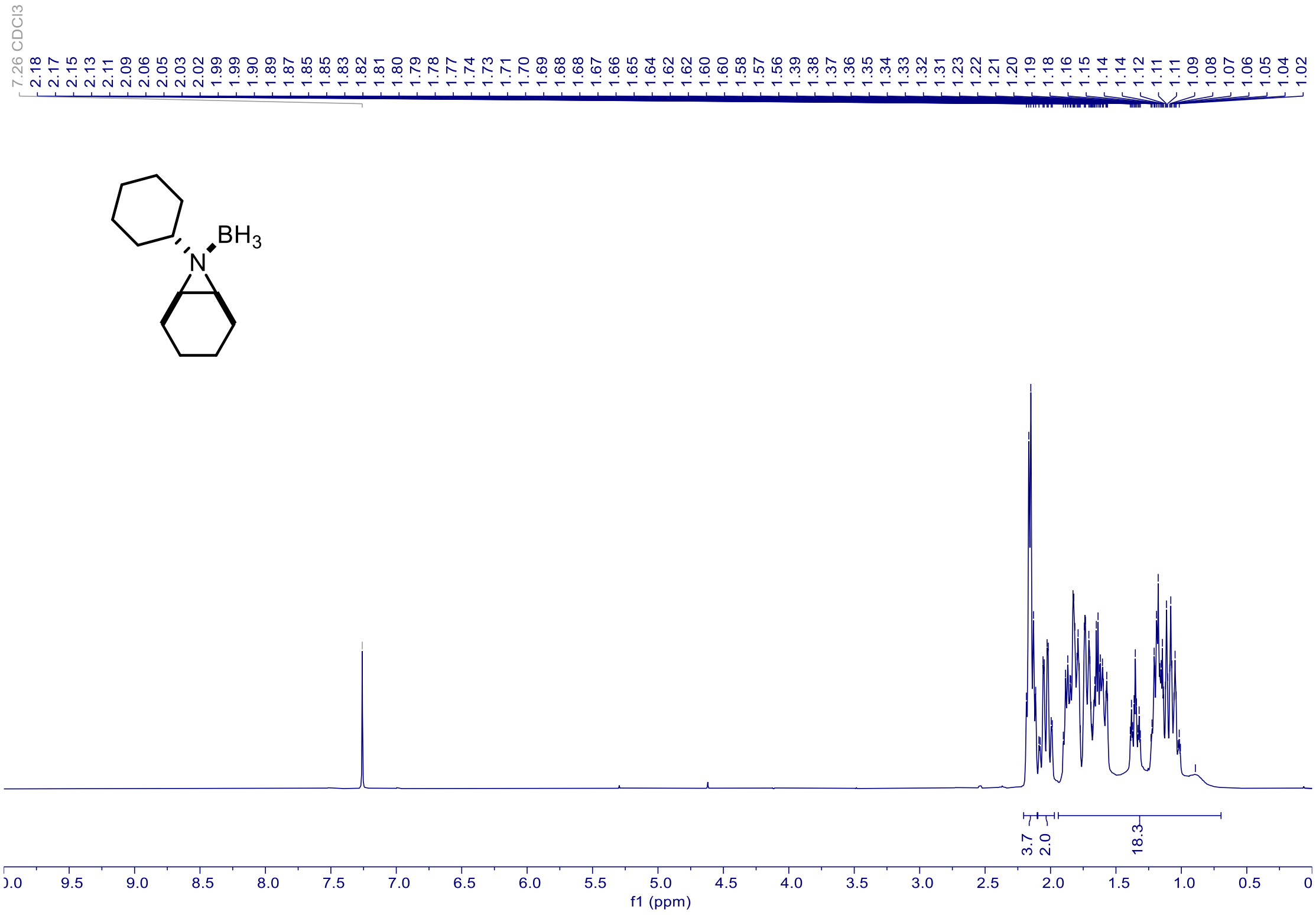

33a – 13C NMR (151 MHz, $CDCl_3$)

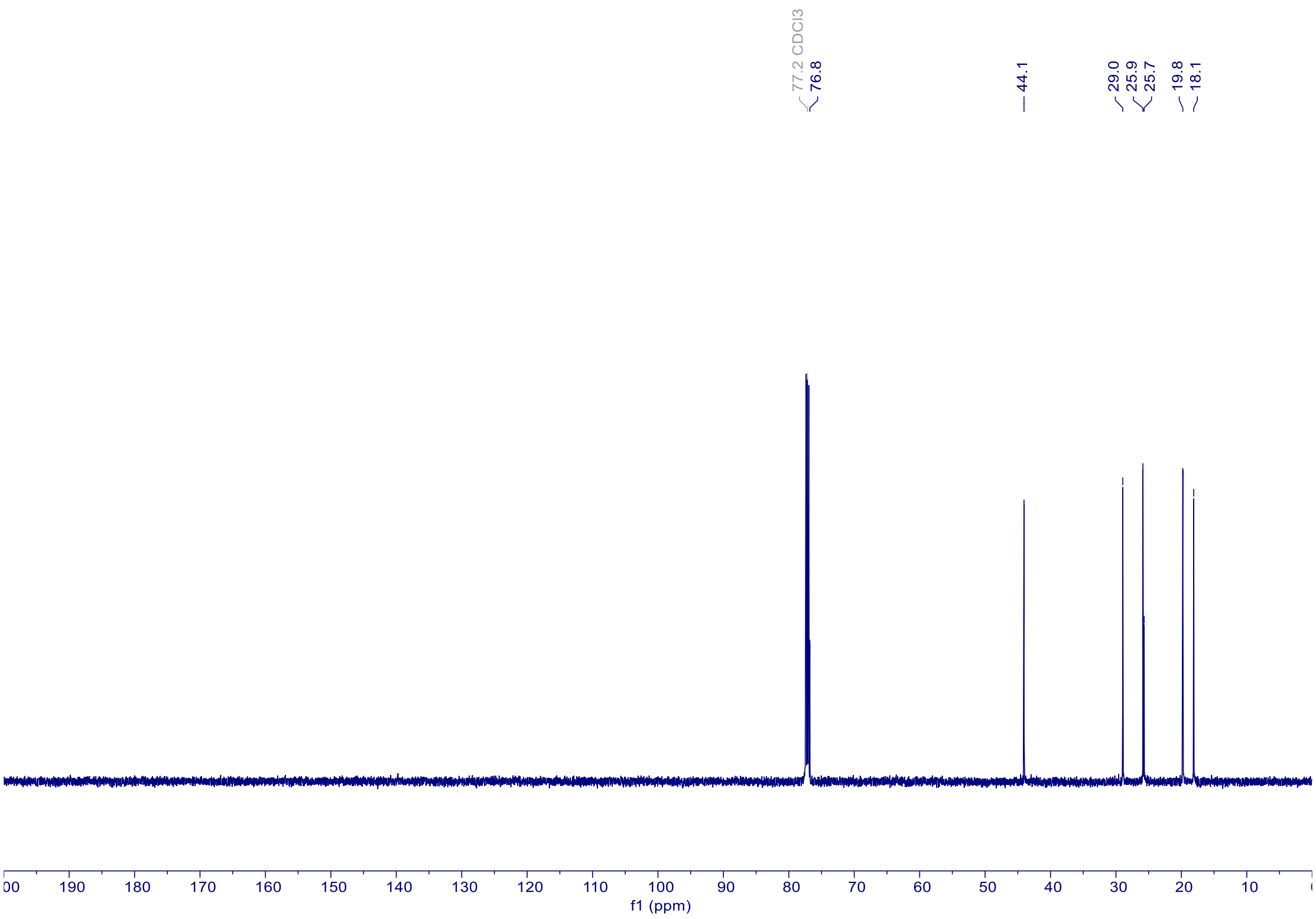

33a – ^{11}B NMR (193 MHz, $CDCl_3$)

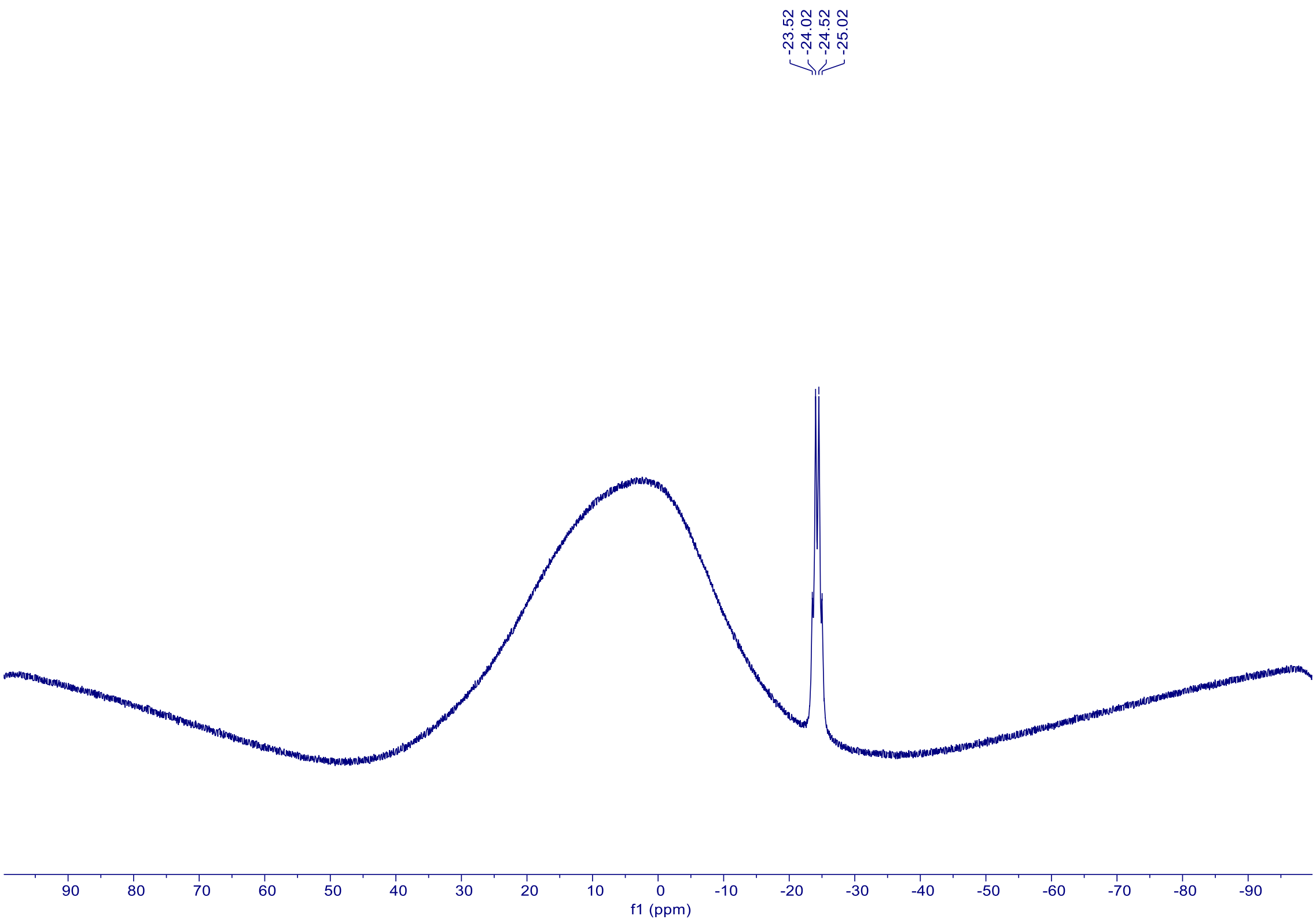

34a – ^{1}H NMR (600 MHz, $CDCl_3$)

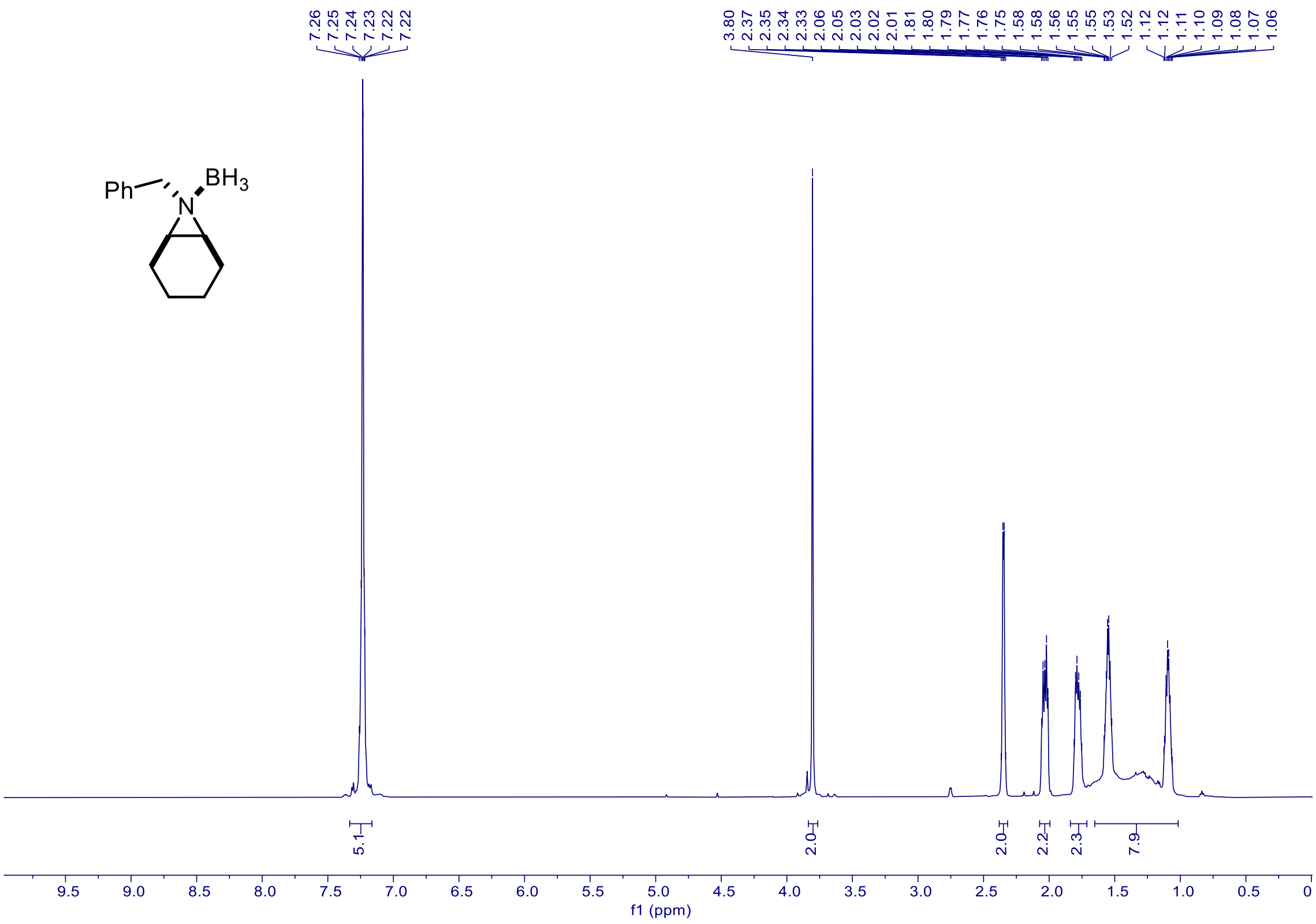

34a – ^{13}C NMR (151 MHz, $CDCl_3$)

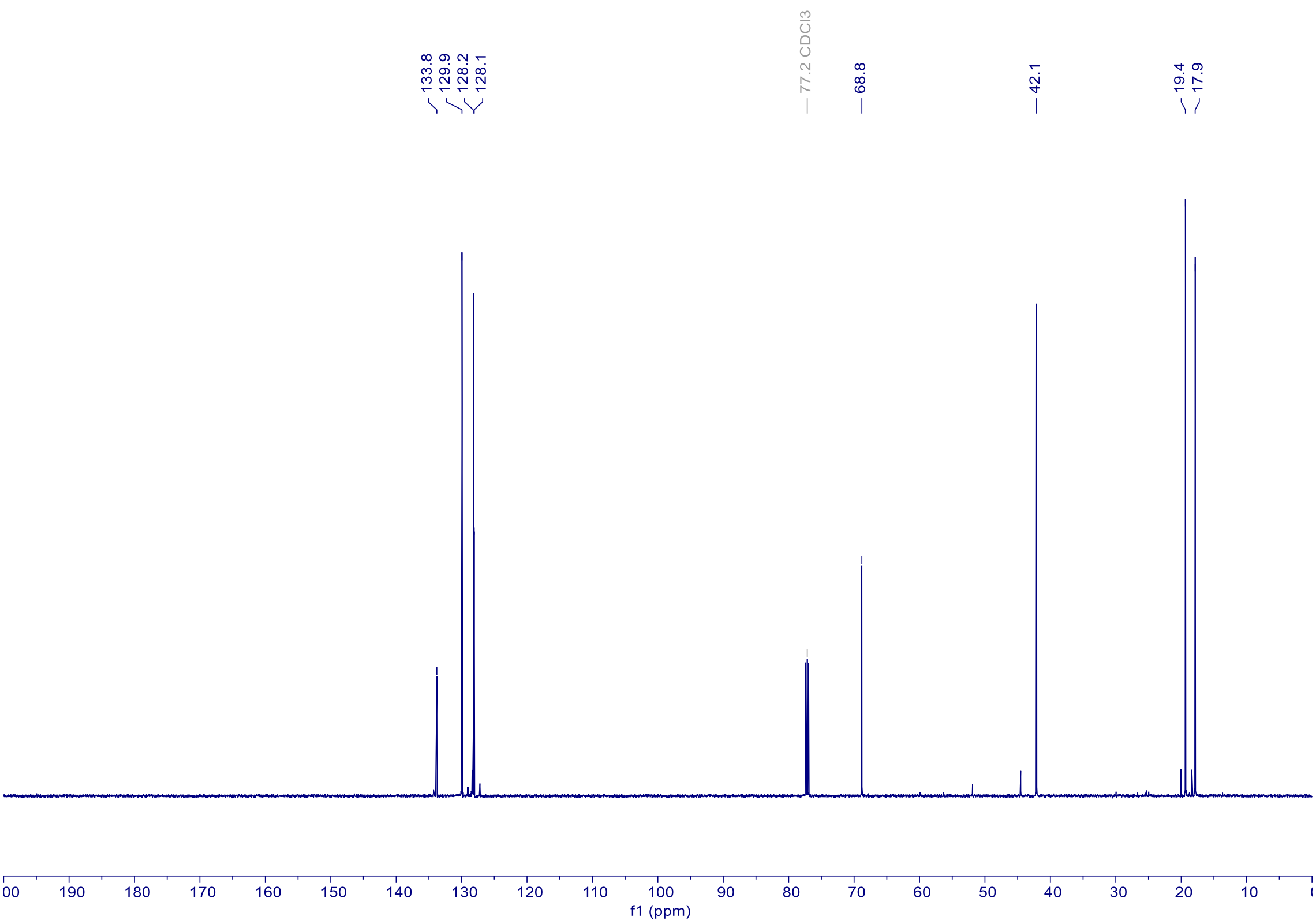

34a – ^{11}B NMR (193 MHz, $CDCl_3$)

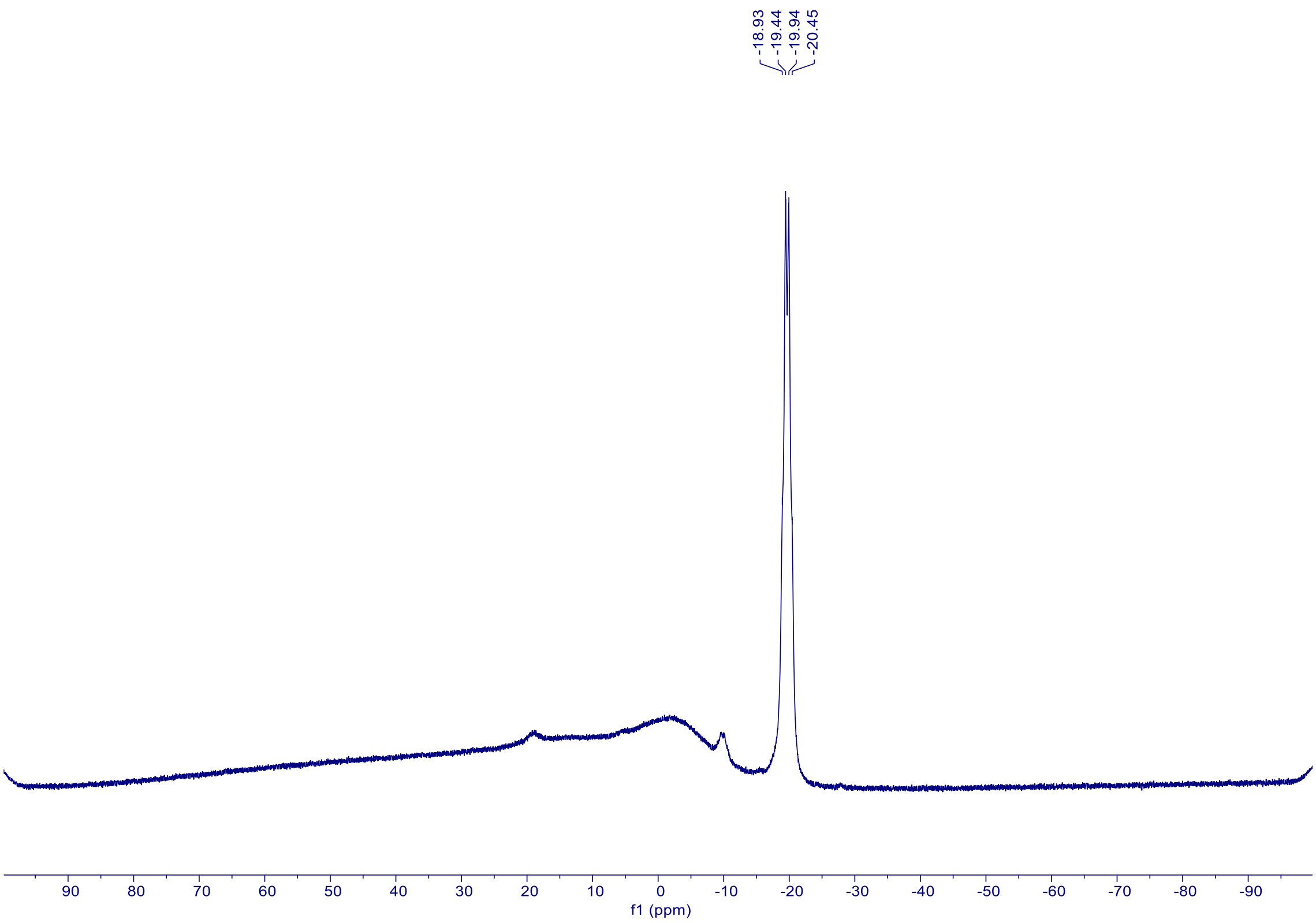

35a – ^{1}H NMR (600 MHz, $CDCl_3$)

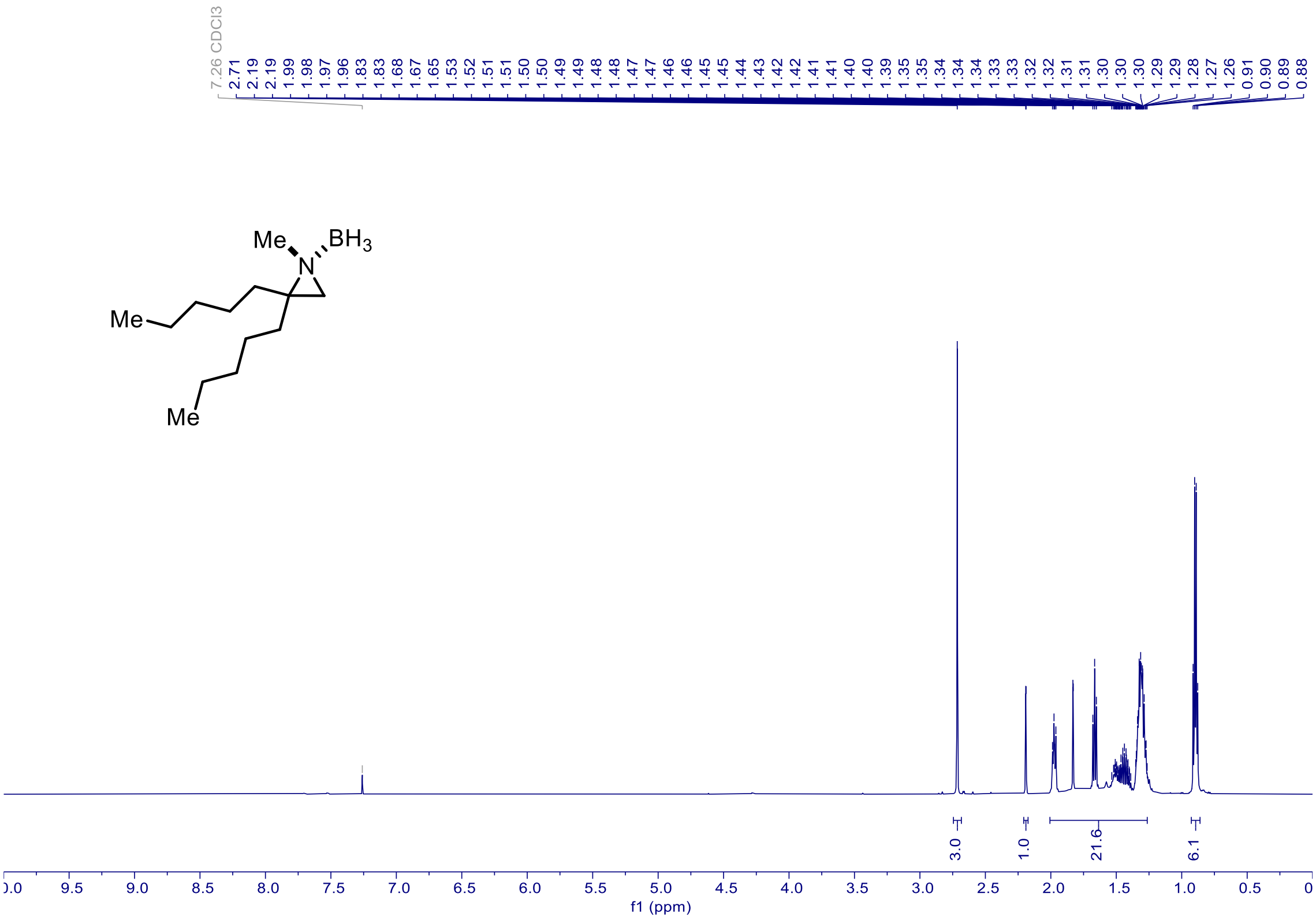

35a – ^{13}C NMR (151 MHz, $CDCl_3$)

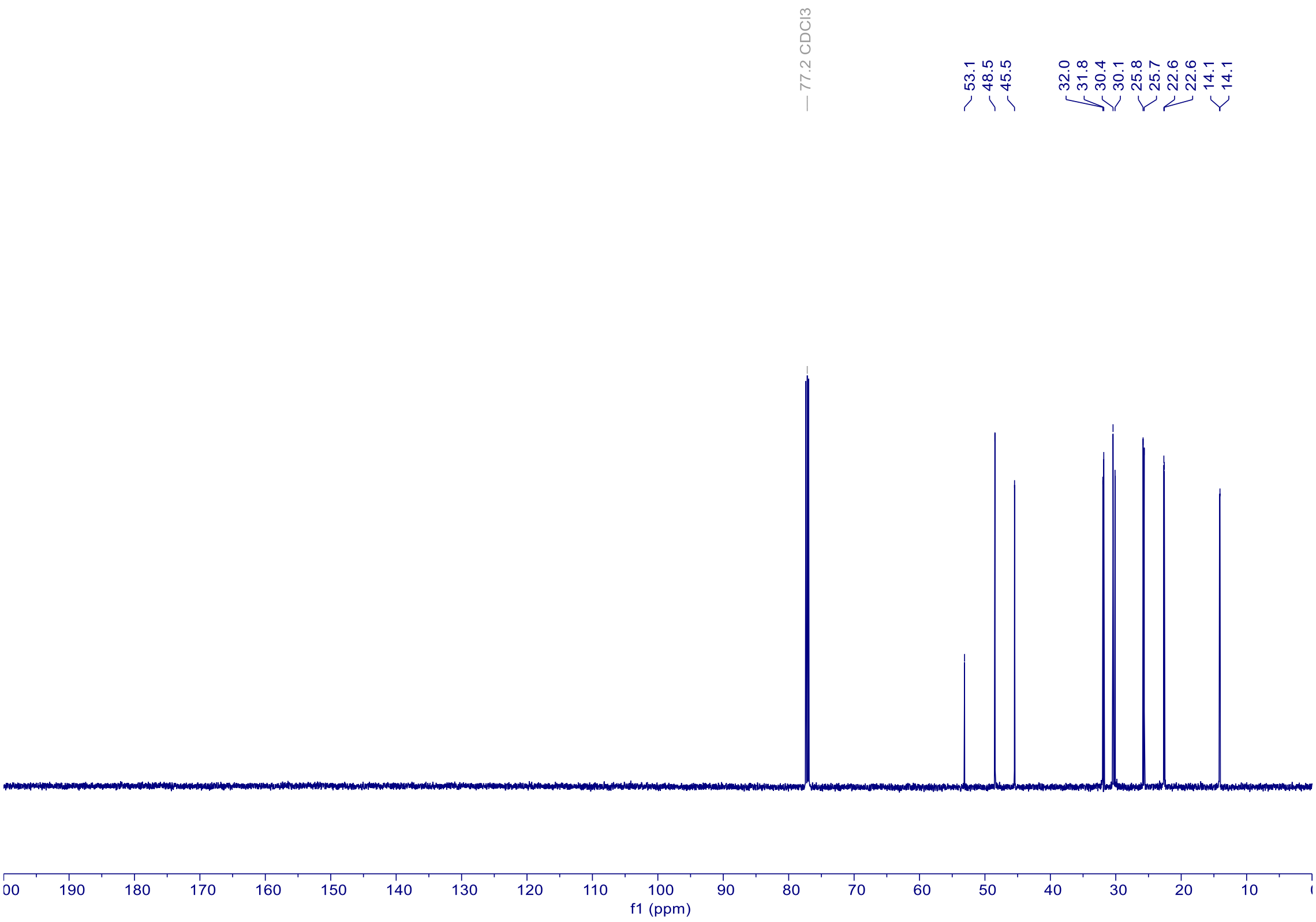

35a – ^{11}B NMR (193 MHz, $CDCl_3$)

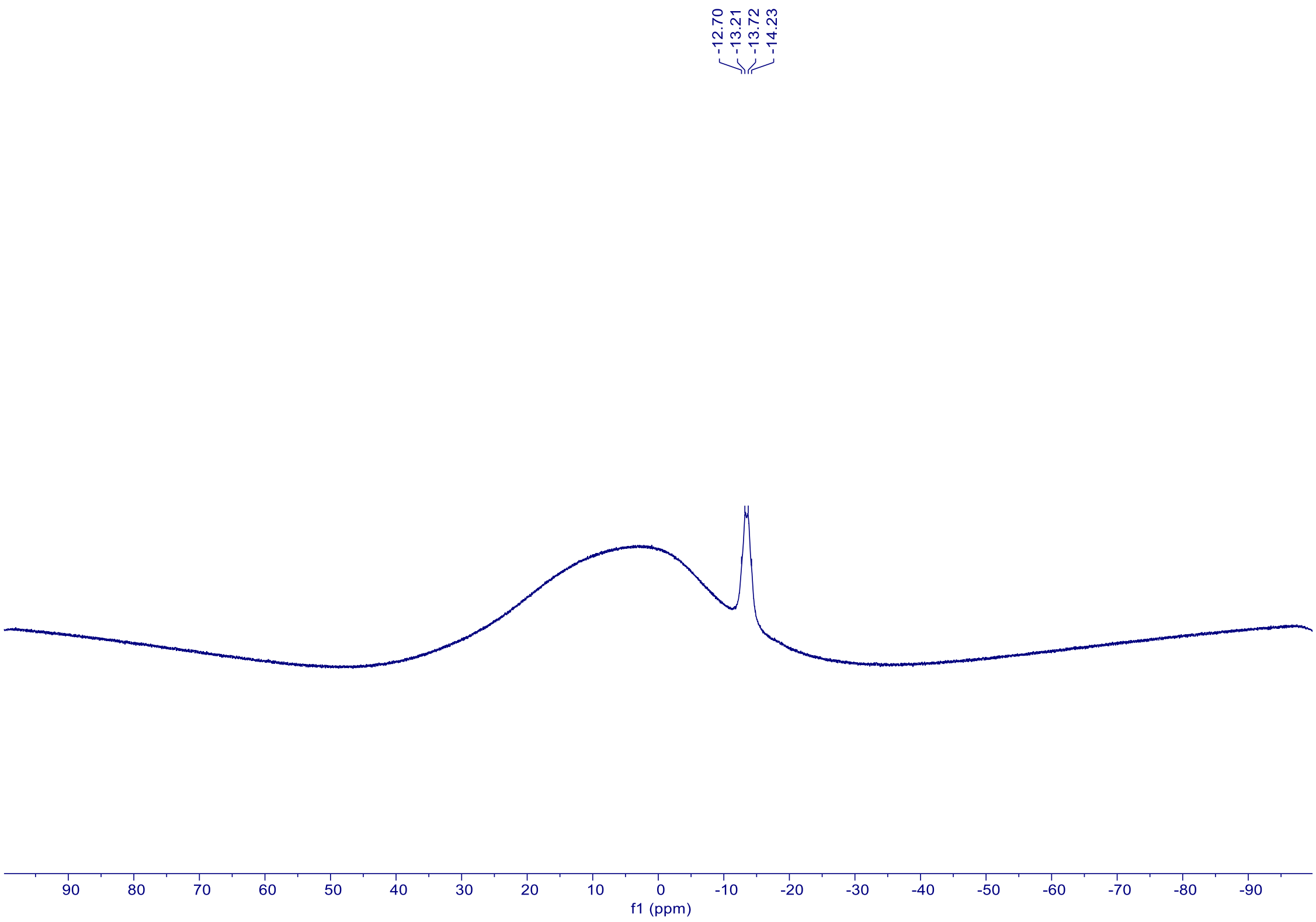

36a – ^{1}H NMR (600 MHz, $CDCl_3$)

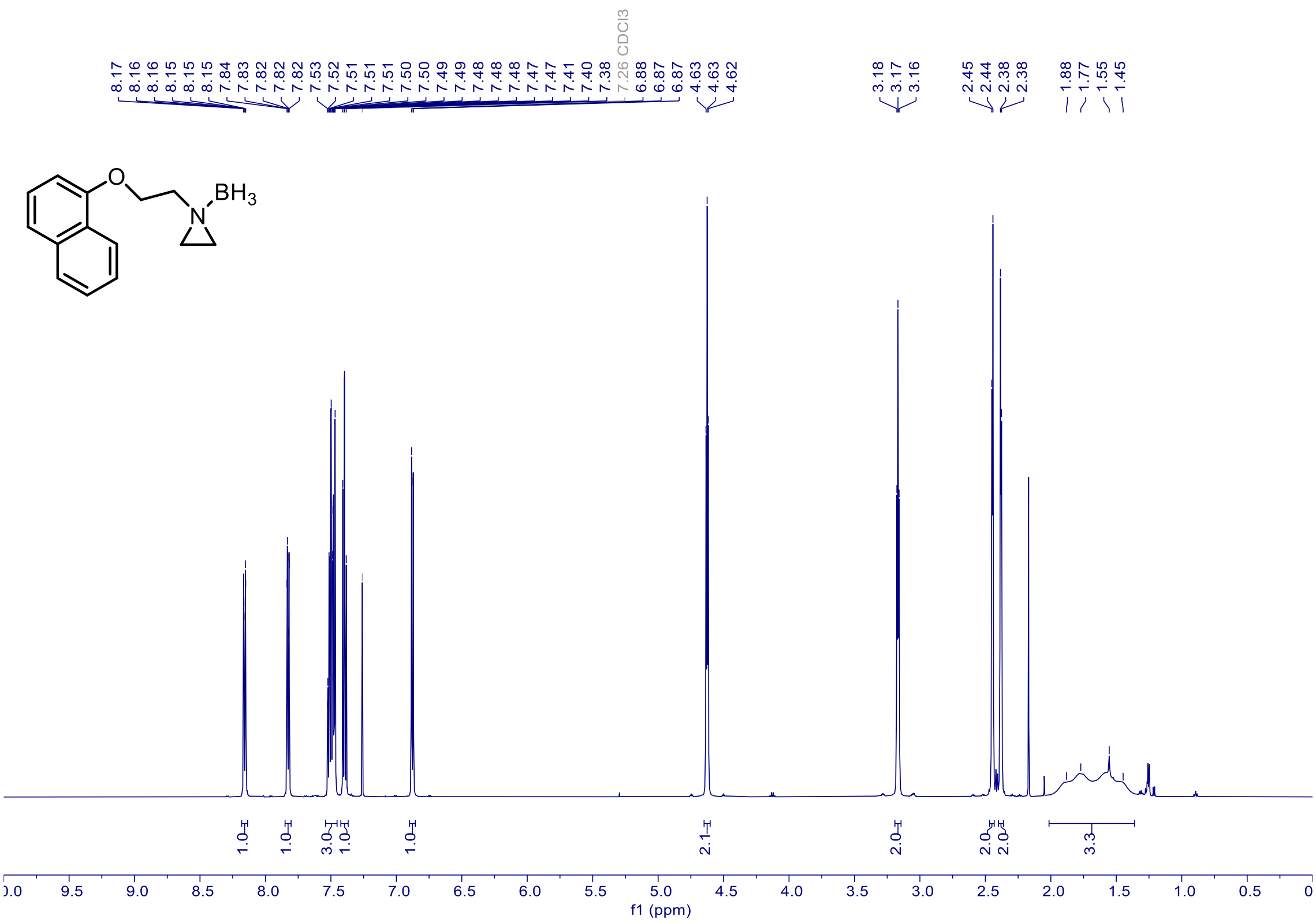

36a – ^{13}C NMR (151 MHz, $CDCl_3$)

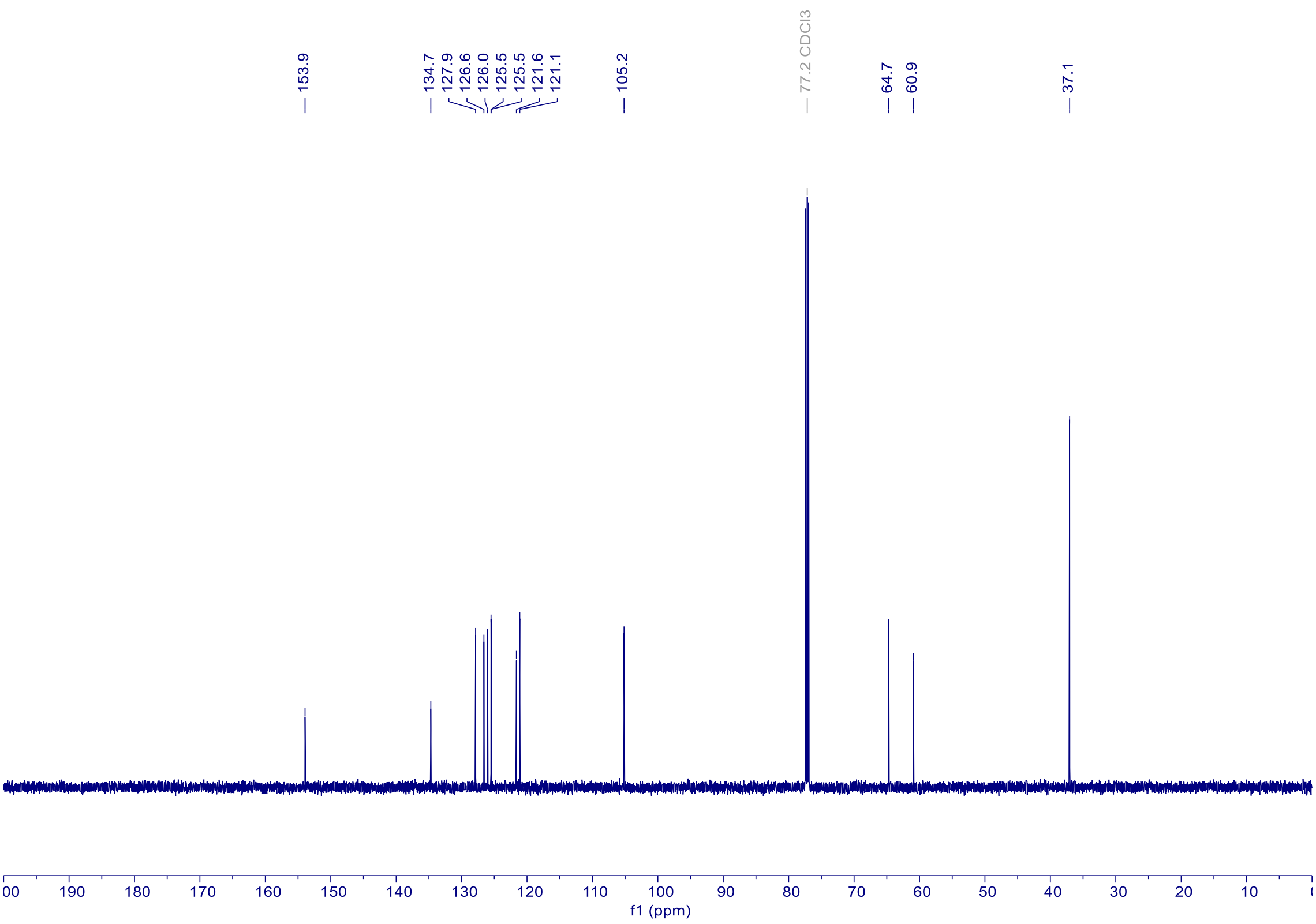

36a – ^{11}B NMR (193 MHz, $CDCl_3$)

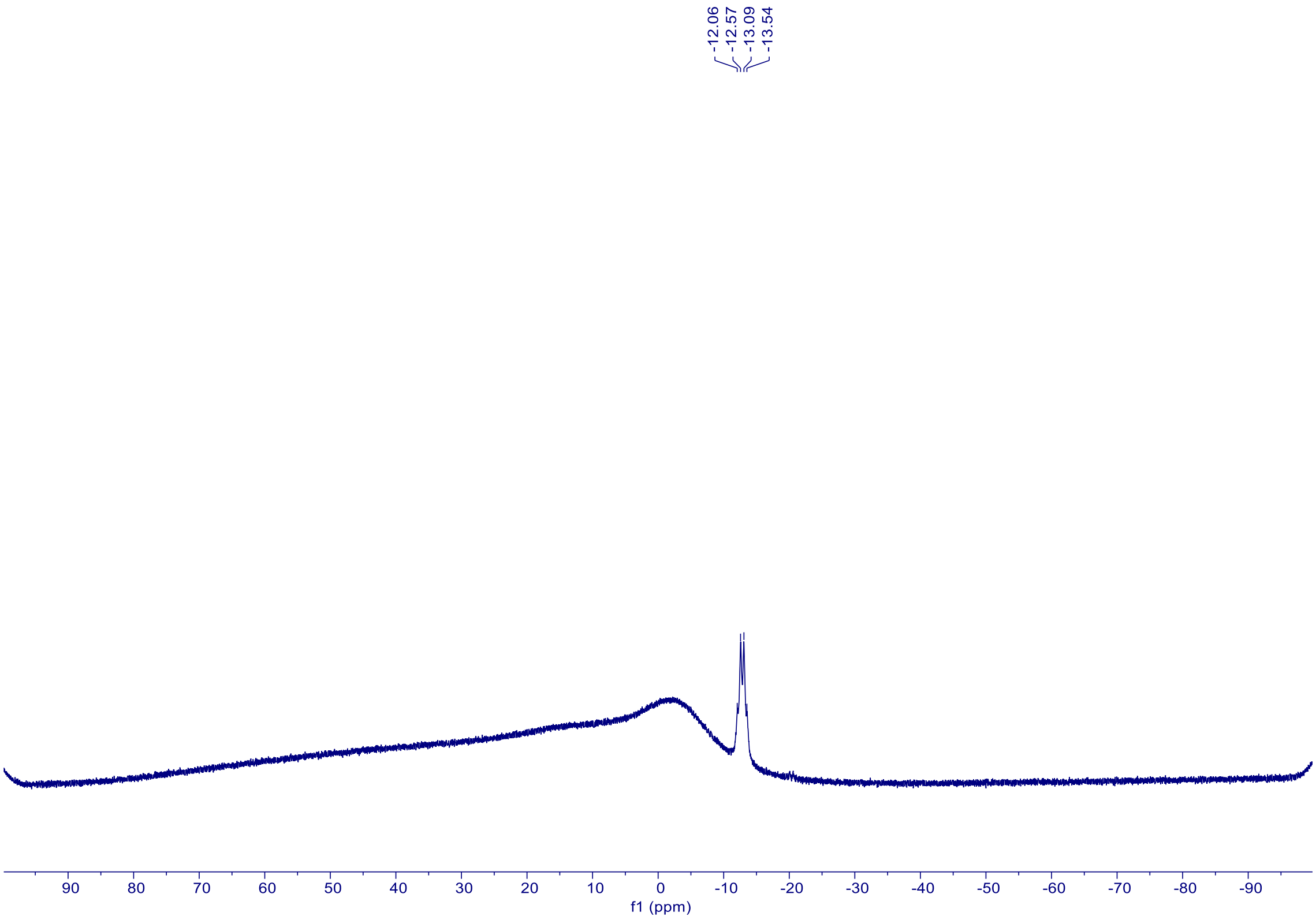

37a – ^{1}H NMR (600 MHz, $CDCl_3$)

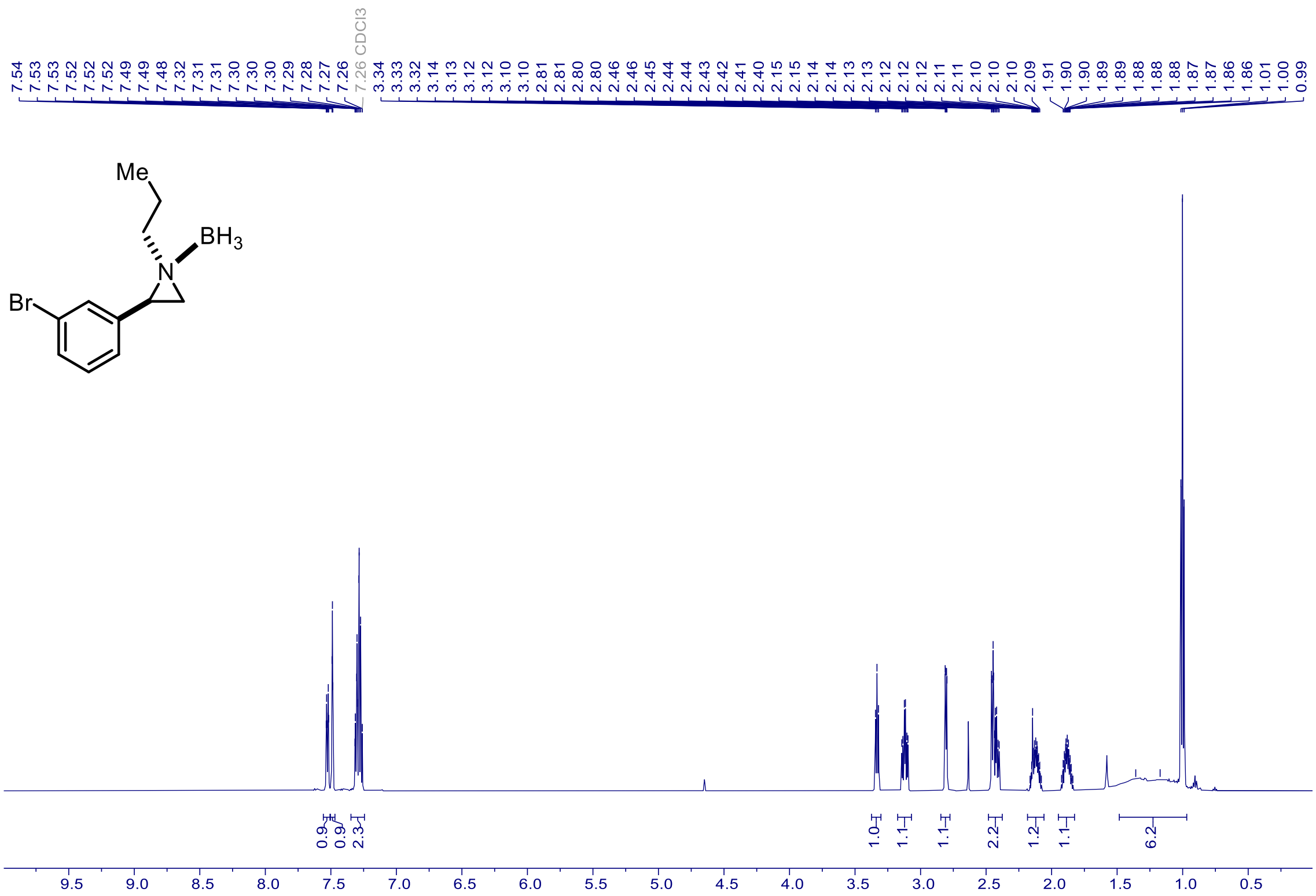

37a – ^{13}C NMR (151 MHz, $CDCl_3$)

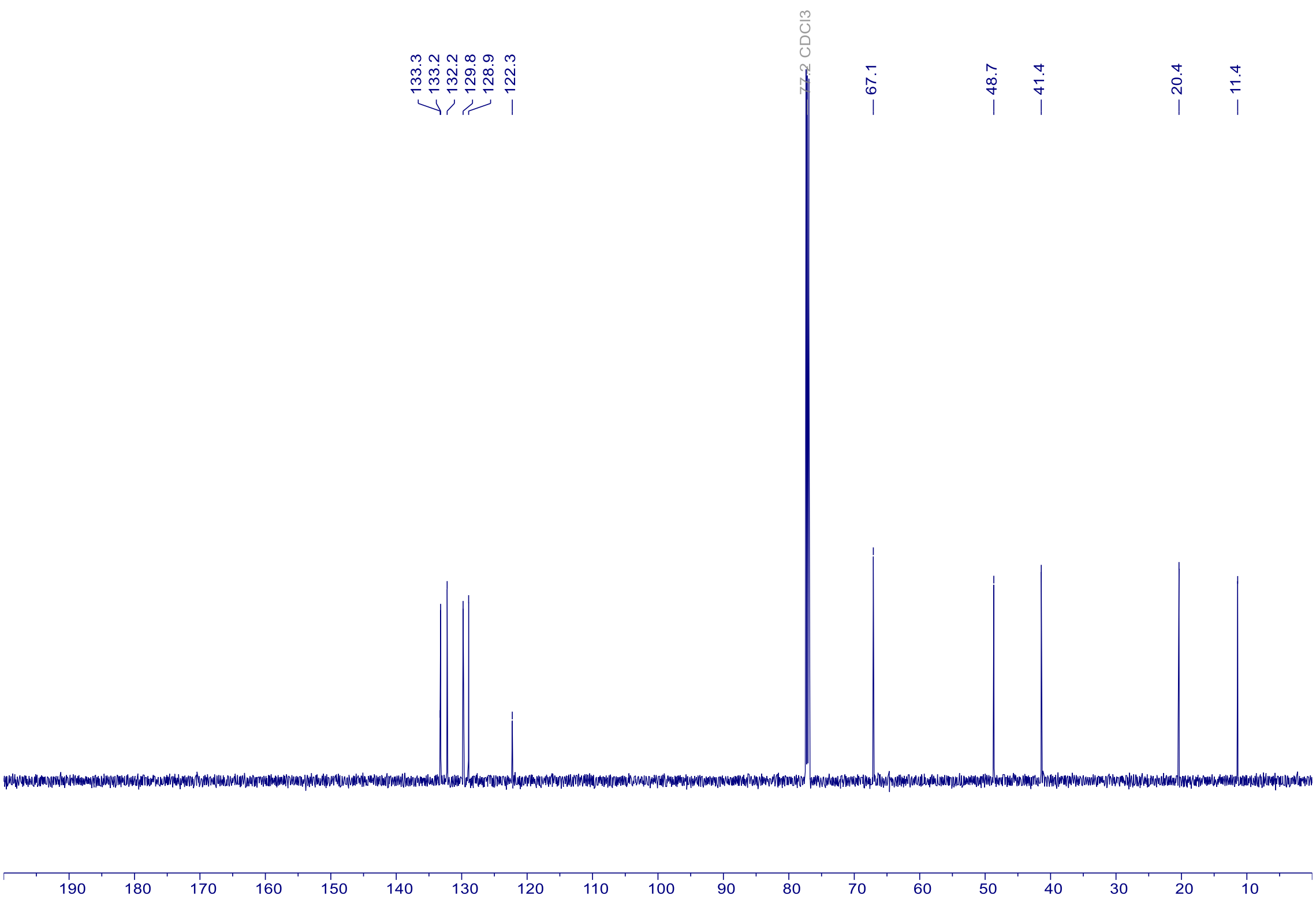

37a – ^{11}B NMR (192 MHz, $CDCl_3$)

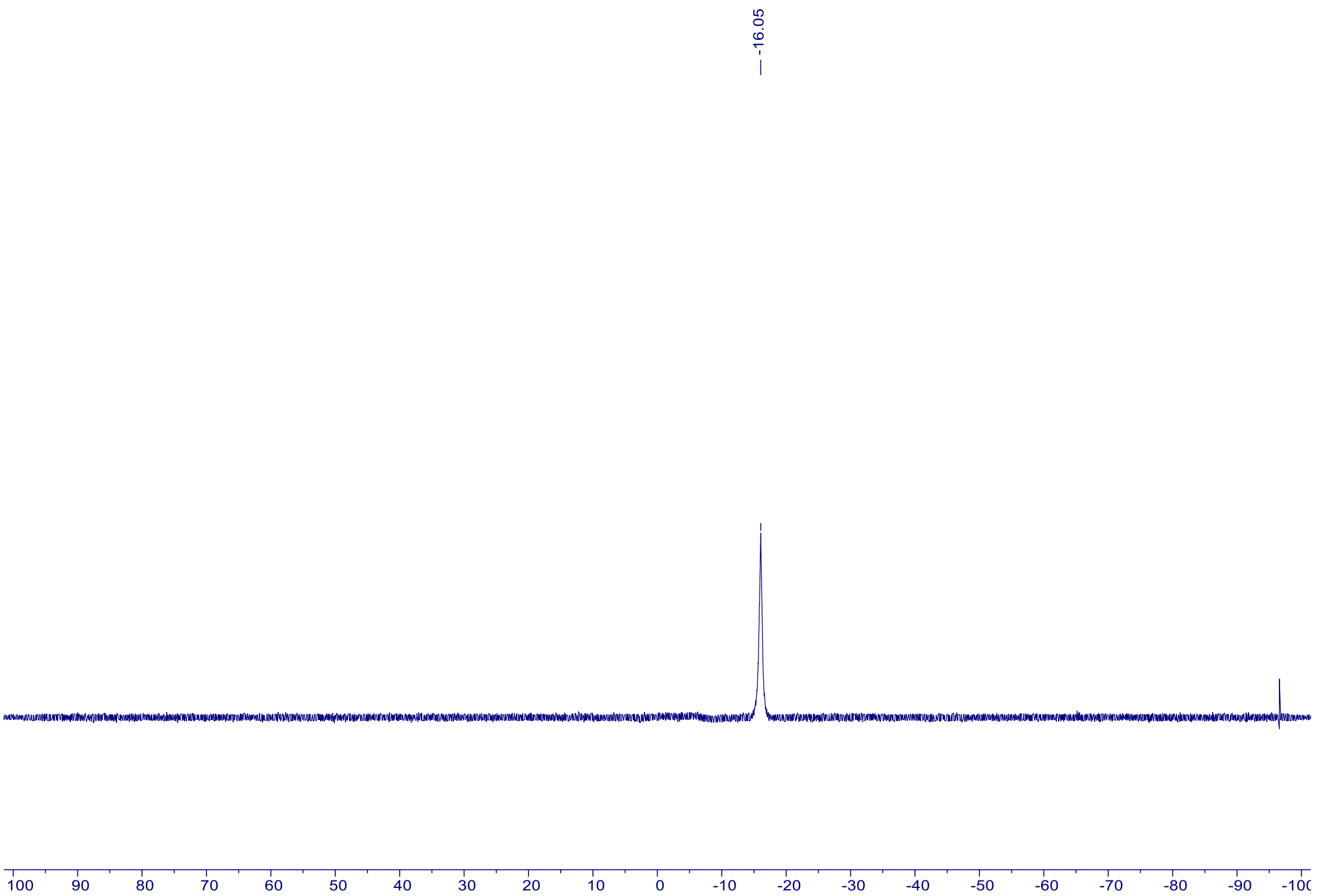

1b – ^{1}H NMR (600 MHz, $CDCl_3$)

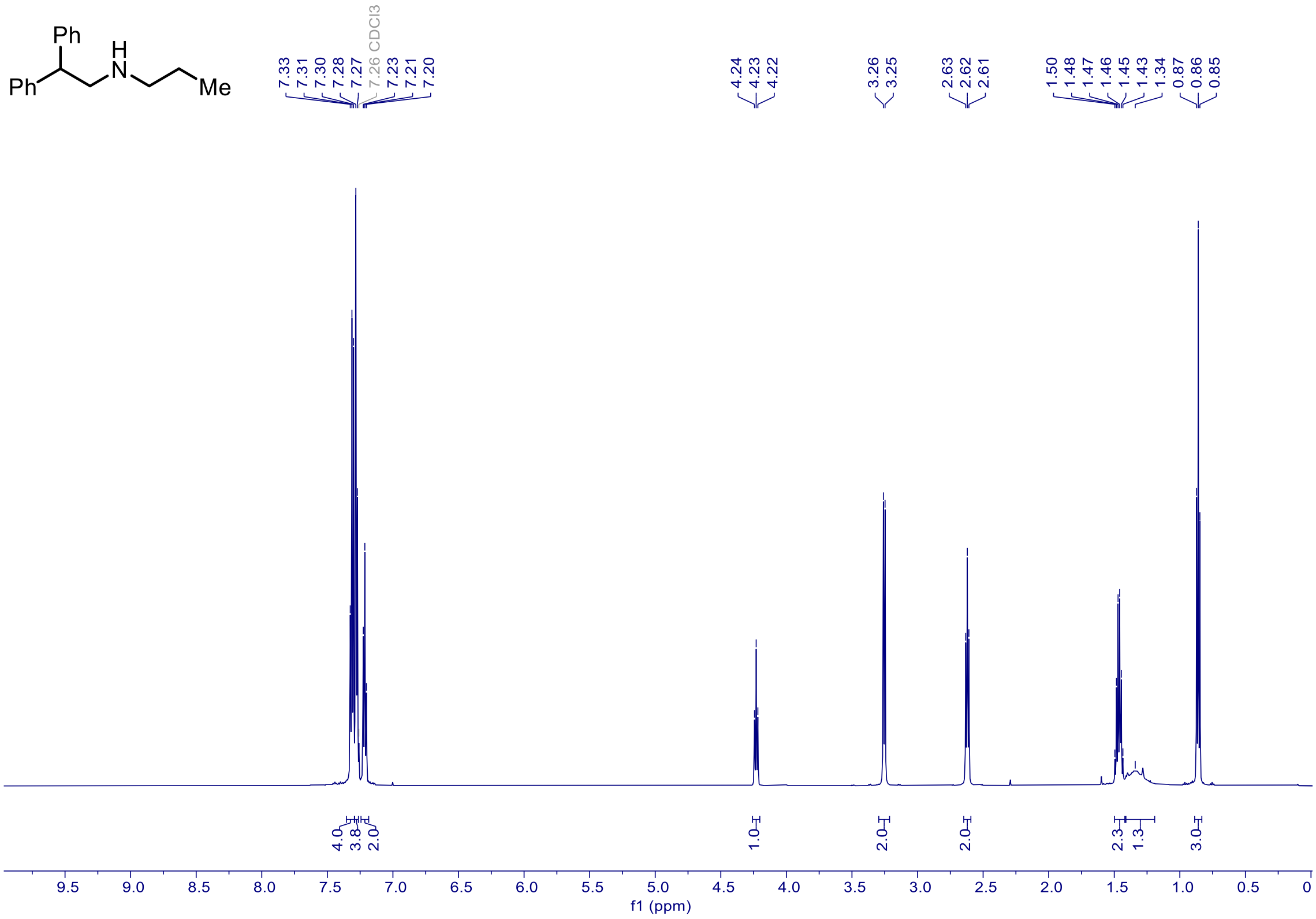

1b – ^{13}C NMR (151 MHz, $CDCl_3$)

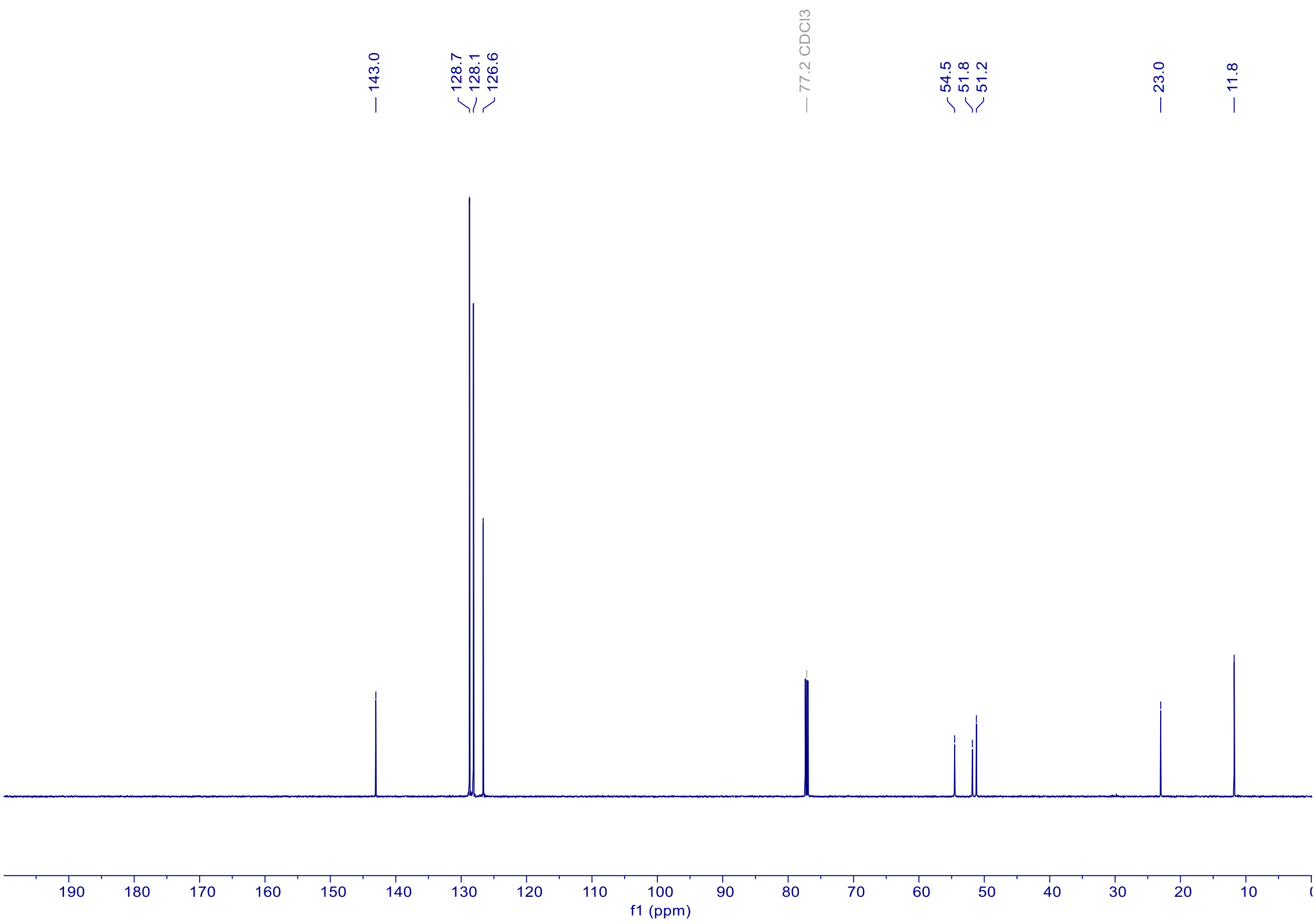

1c – ^{1}H NMR (600 MHz, $CDCl_3$)

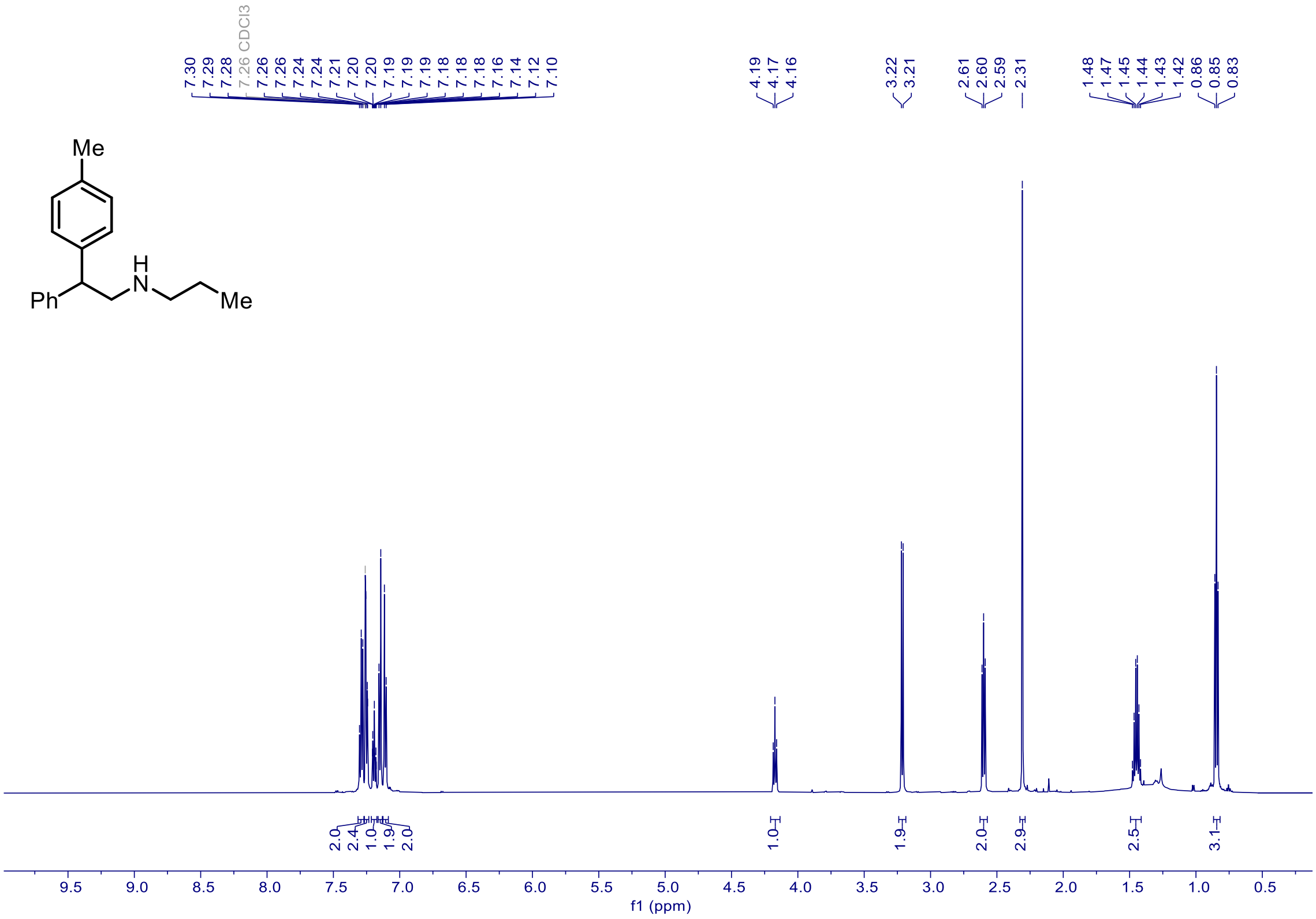

1c – ^{13}C NMR (151 MHz, $CDCl_3$)

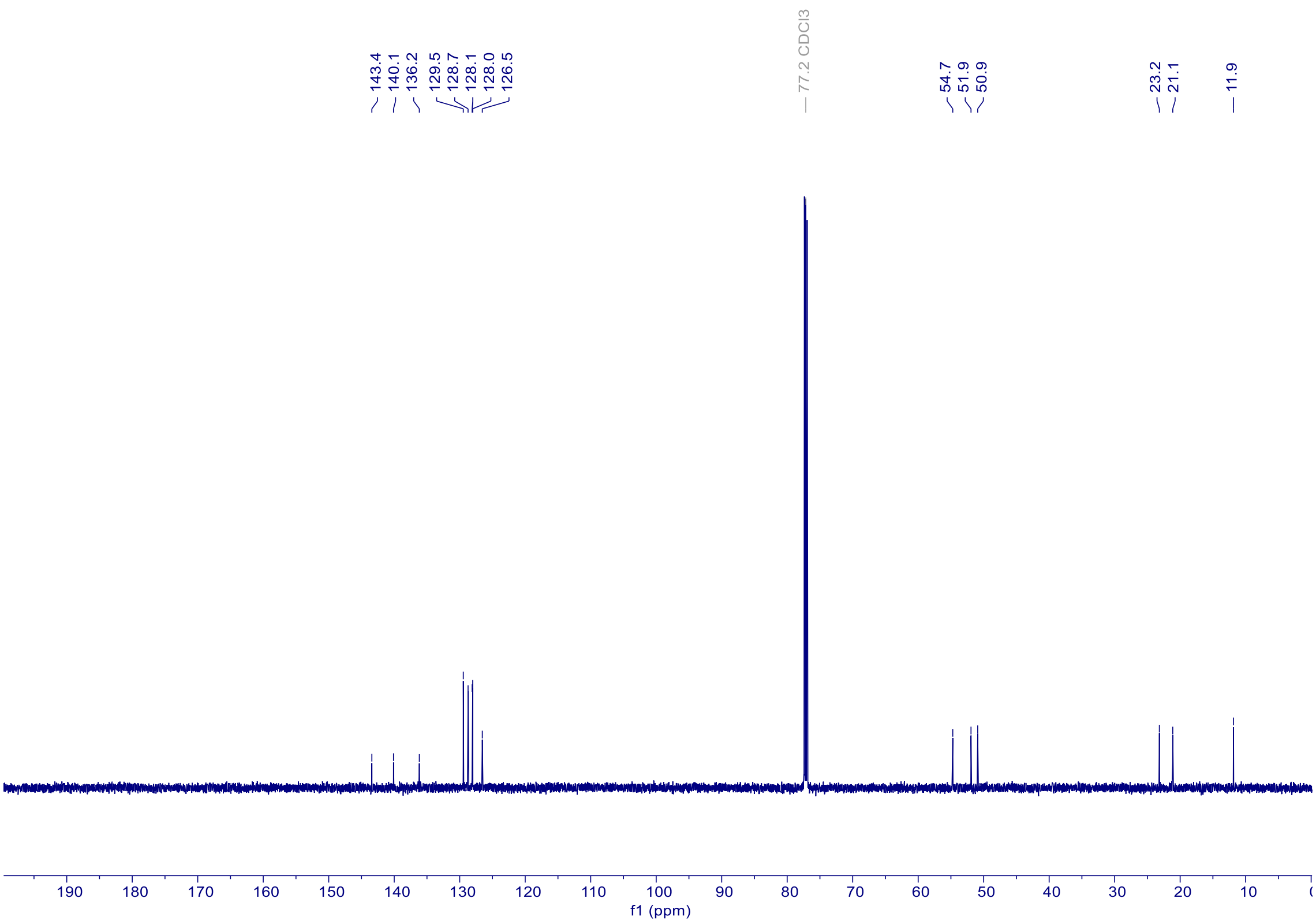

1d/3b – ^{1}H NMR (600 MHz, $CDCl_3$)

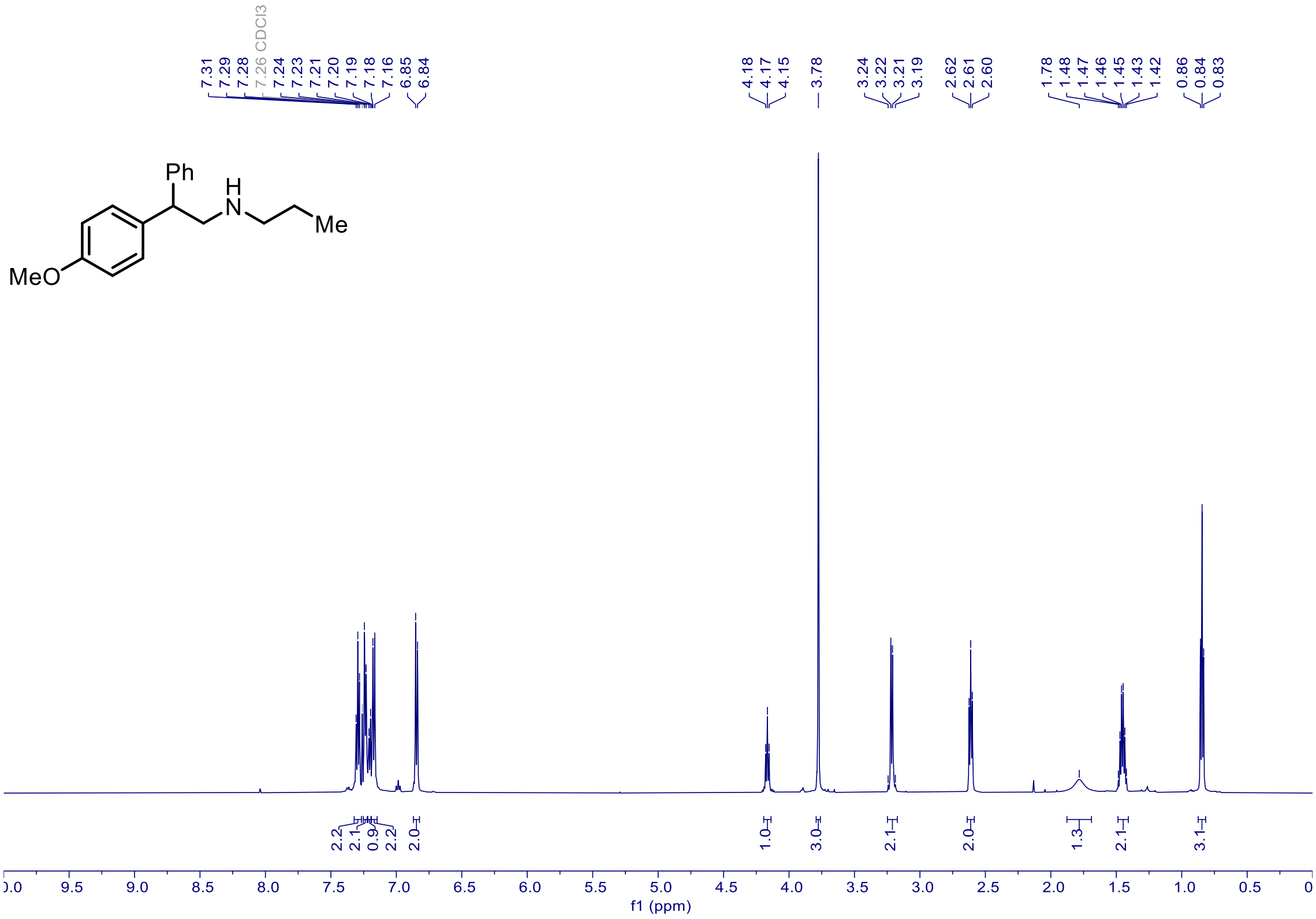

1d/3b – ^{13}C NMR (151 MHz, $CDCl_3$)

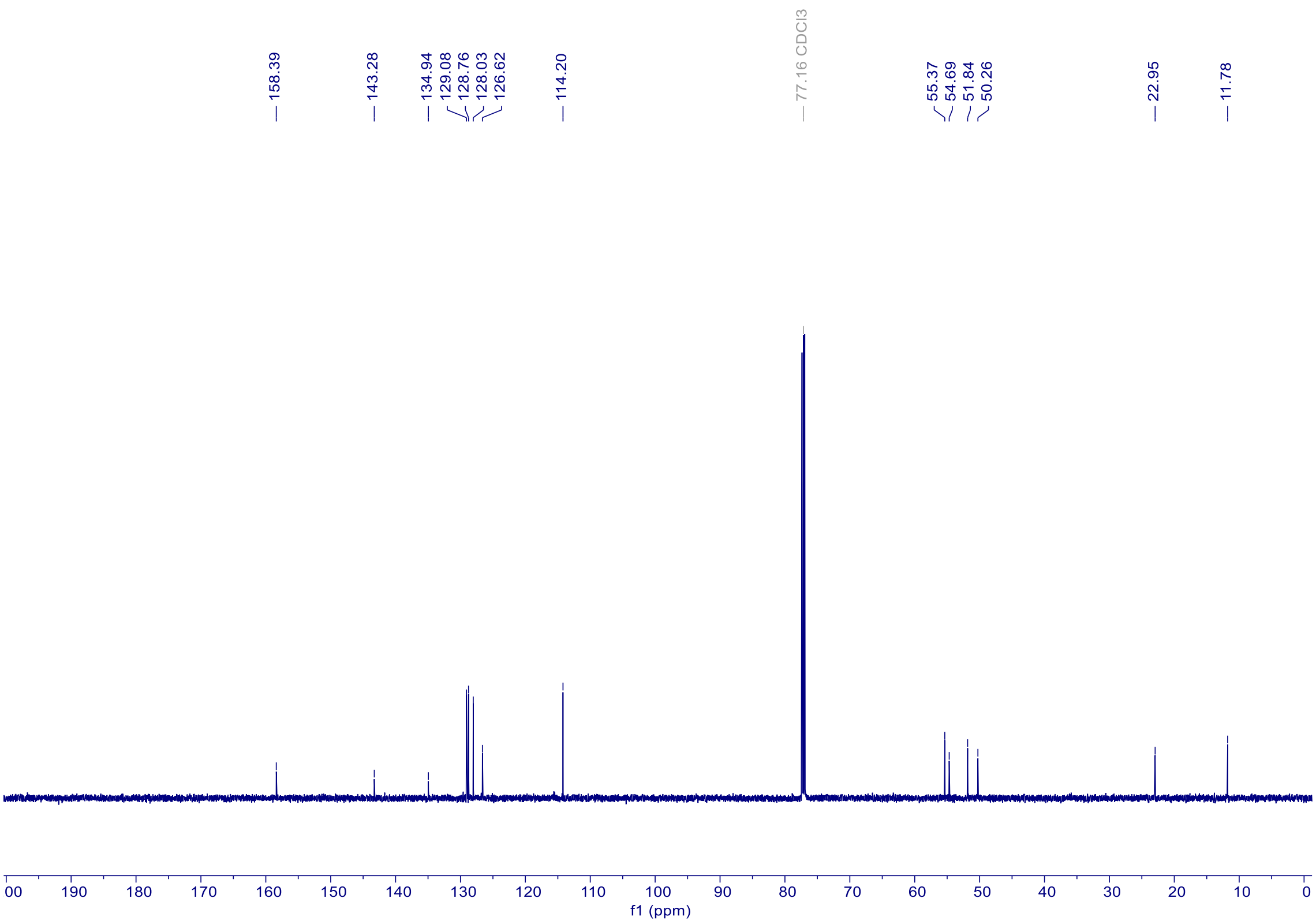

1e – ^{1}H NMR (600 MHz, $CDCl_3$)

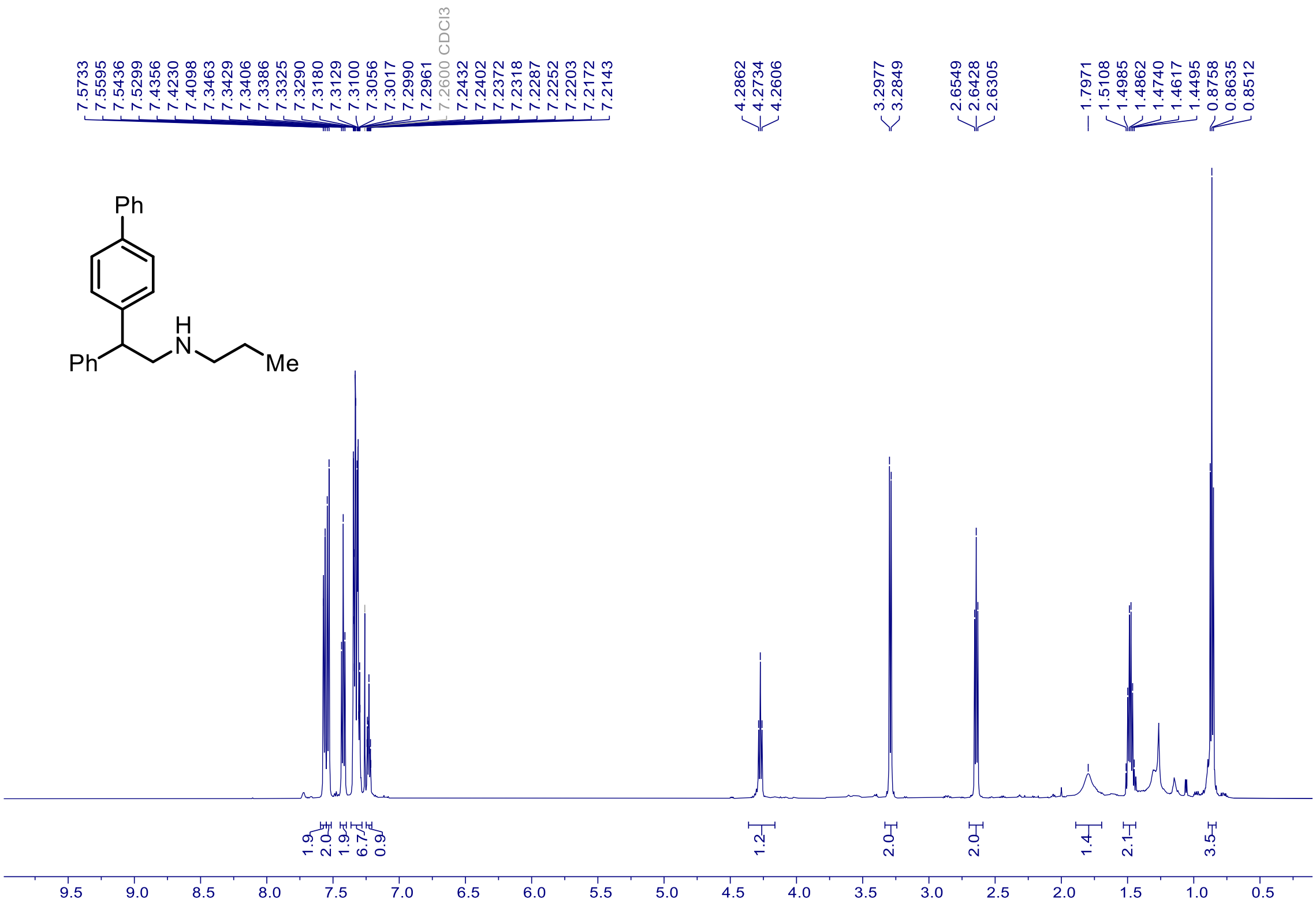

1e – ^{13}C NMR (151 MHz, $CDCl_3$)

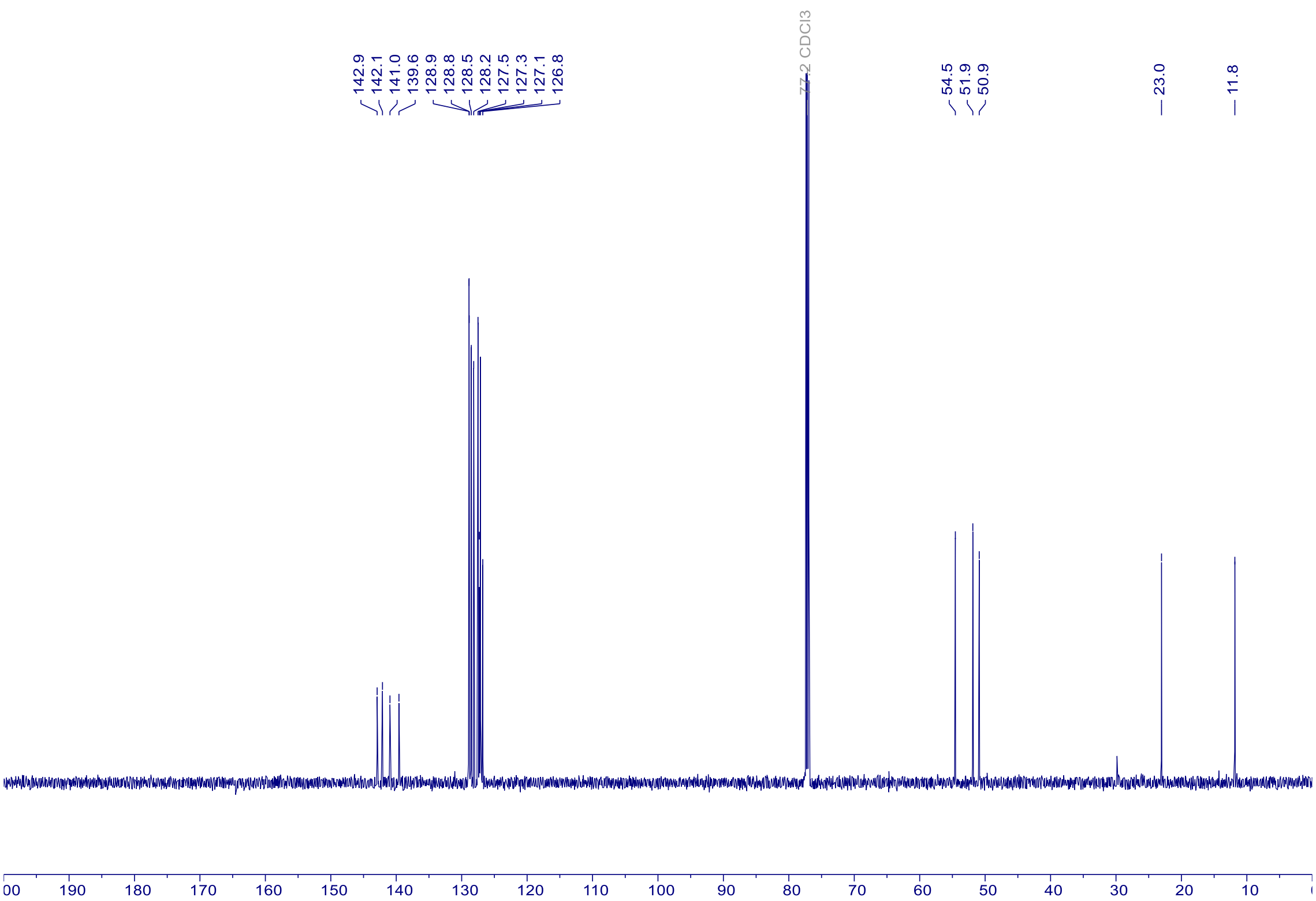

1f – ^{1}H NMR (600 MHz, $CDCl_3$)

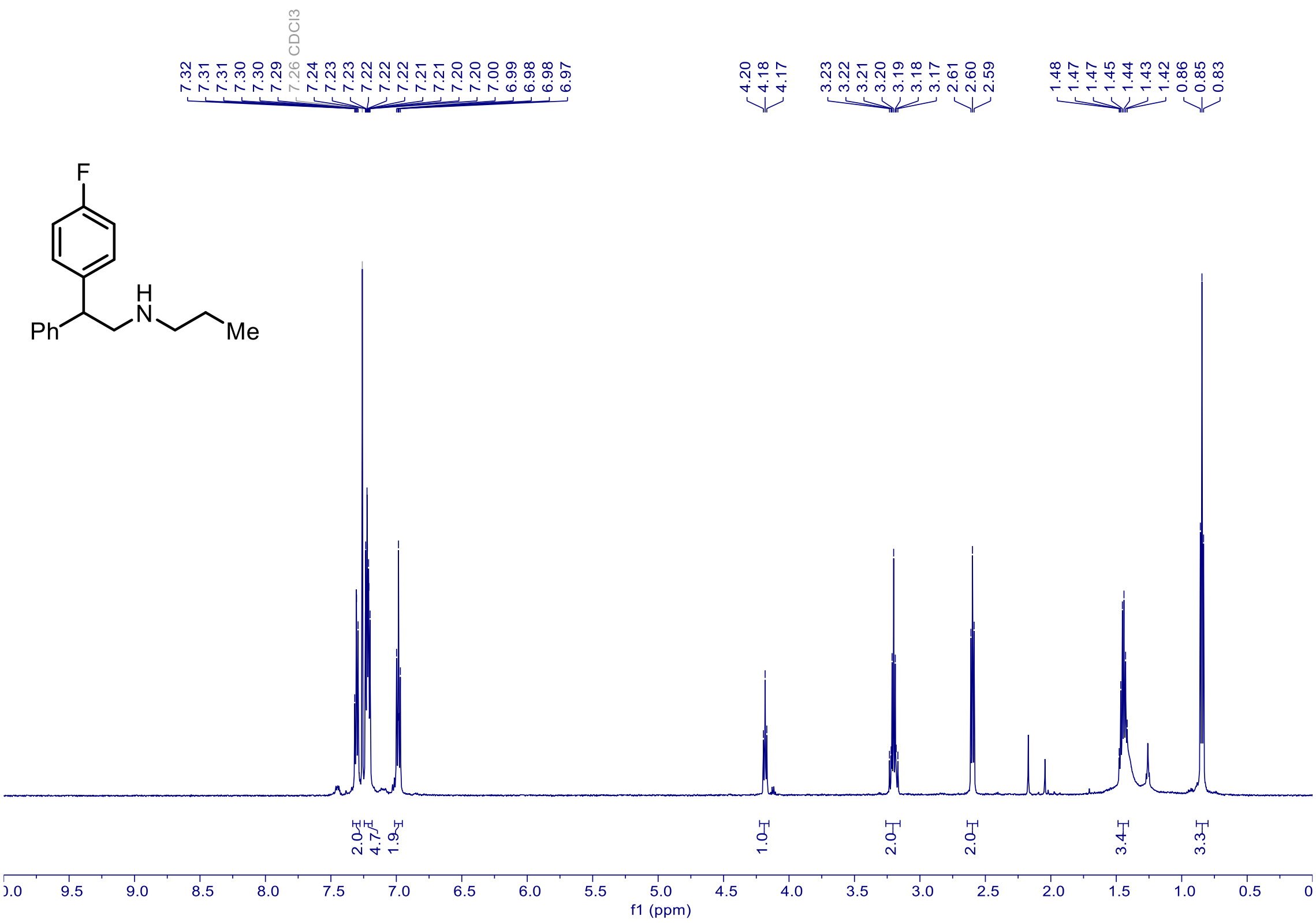

1f – ^{13}C NMR (151 MHz, $CDCl_3$)

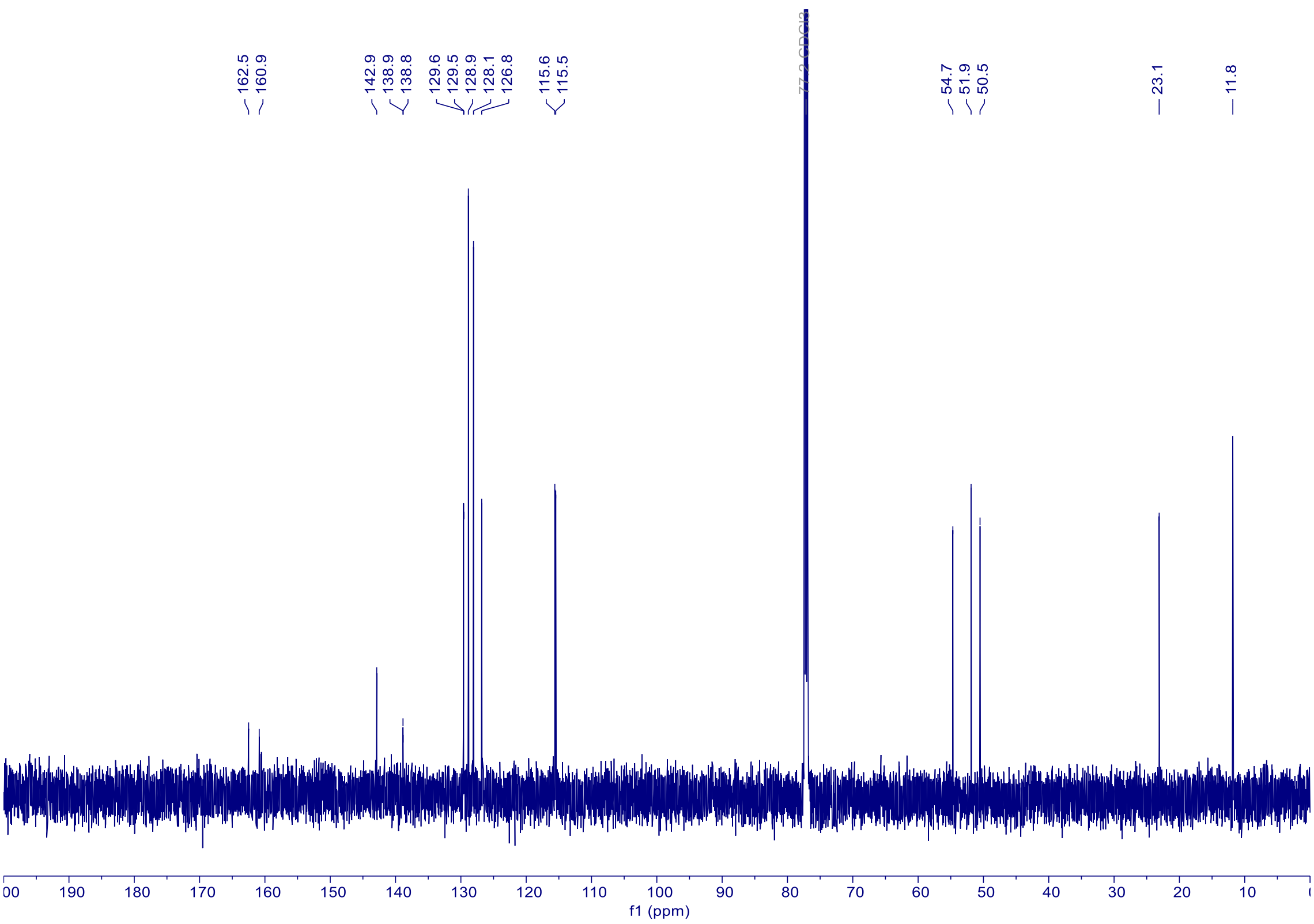

1f – ^{19}F NMR (565 MHz, $CDCl_3$)

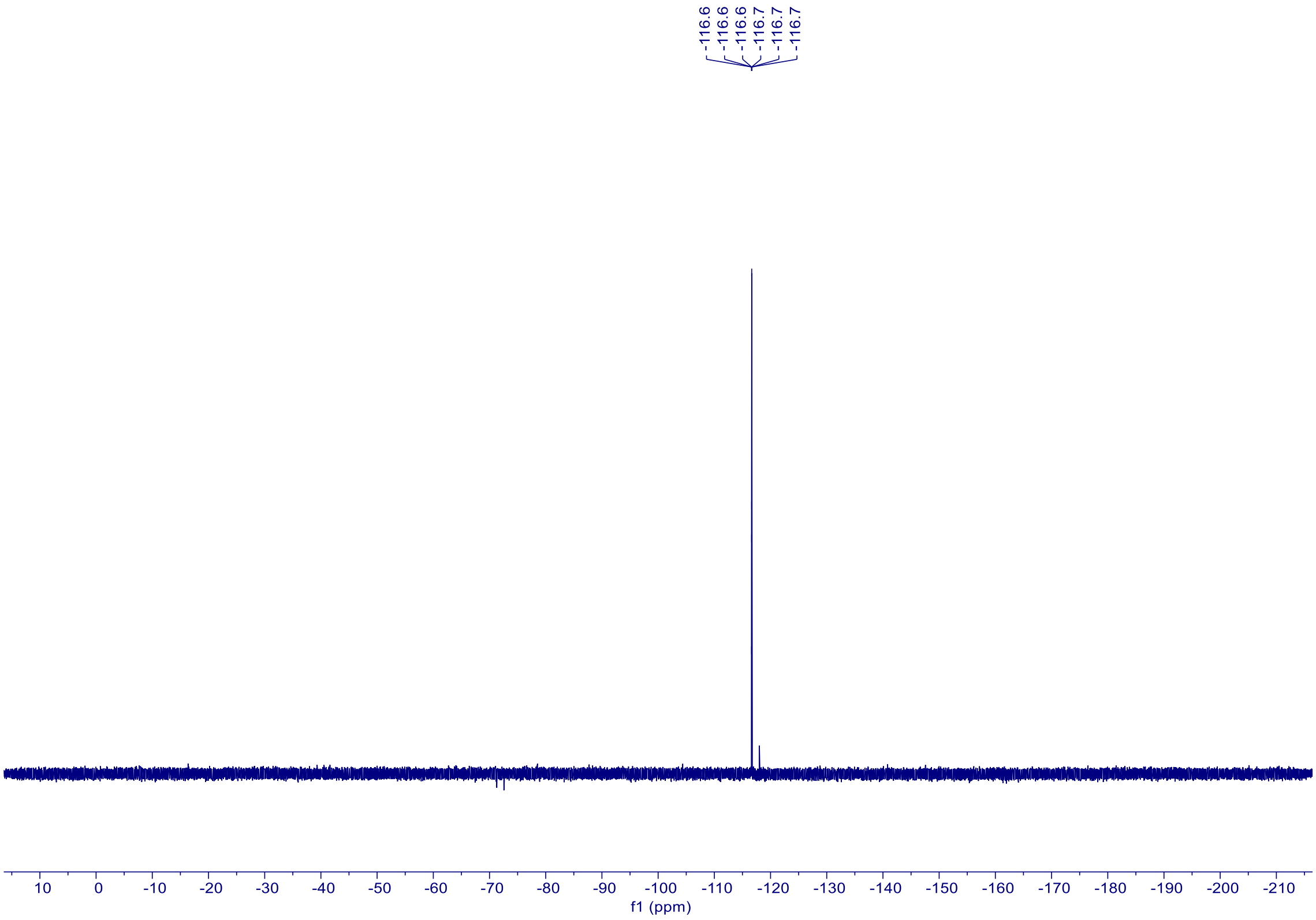

1g/5b – ^{1}H NMR (400 MHz, $CDCl_3$)

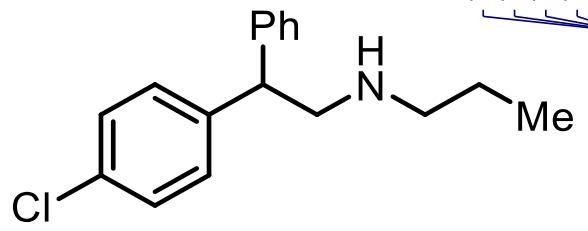

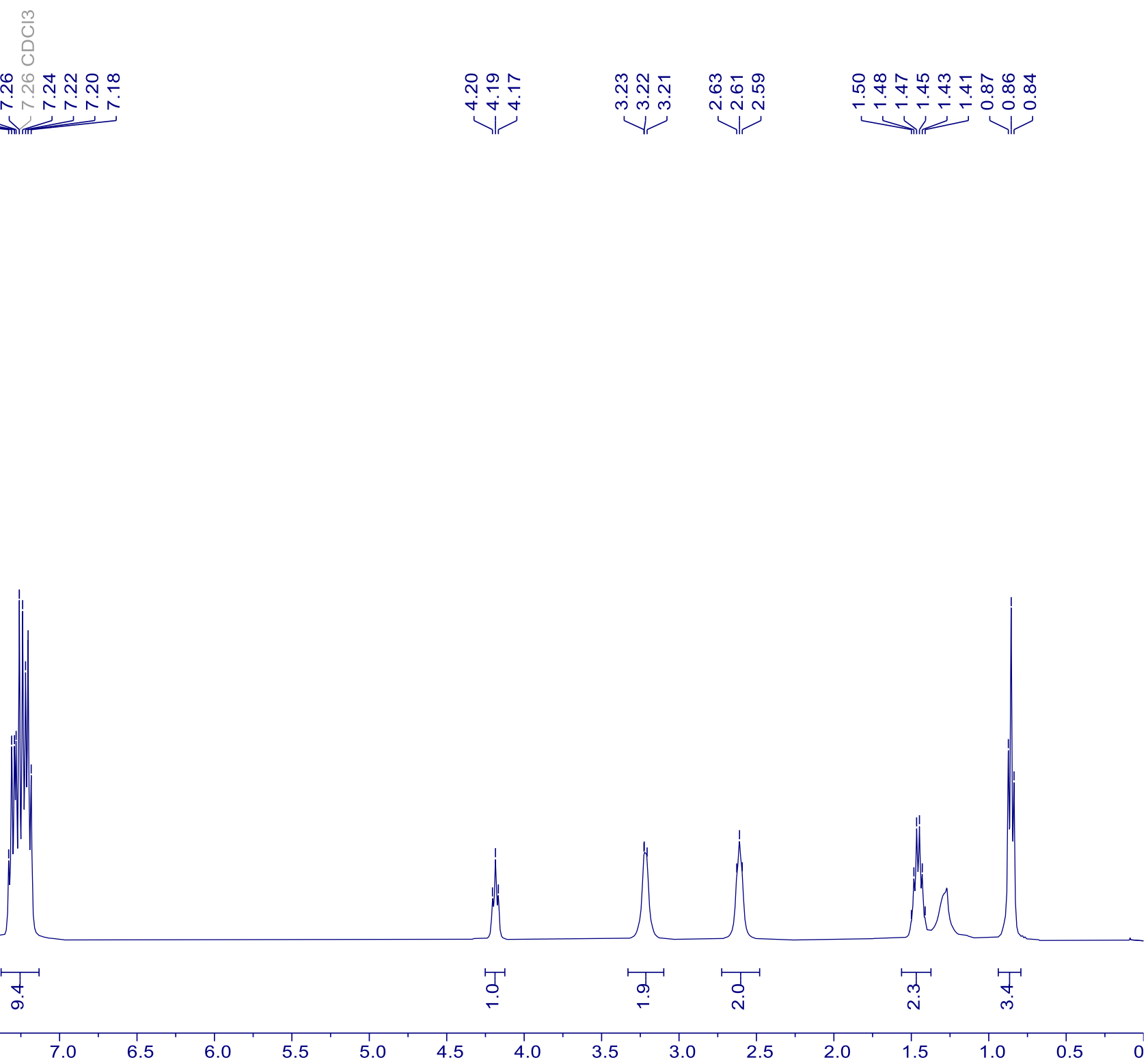

1g/5b – ^{13}C NMR (101 MHz, $CDCl_3$)

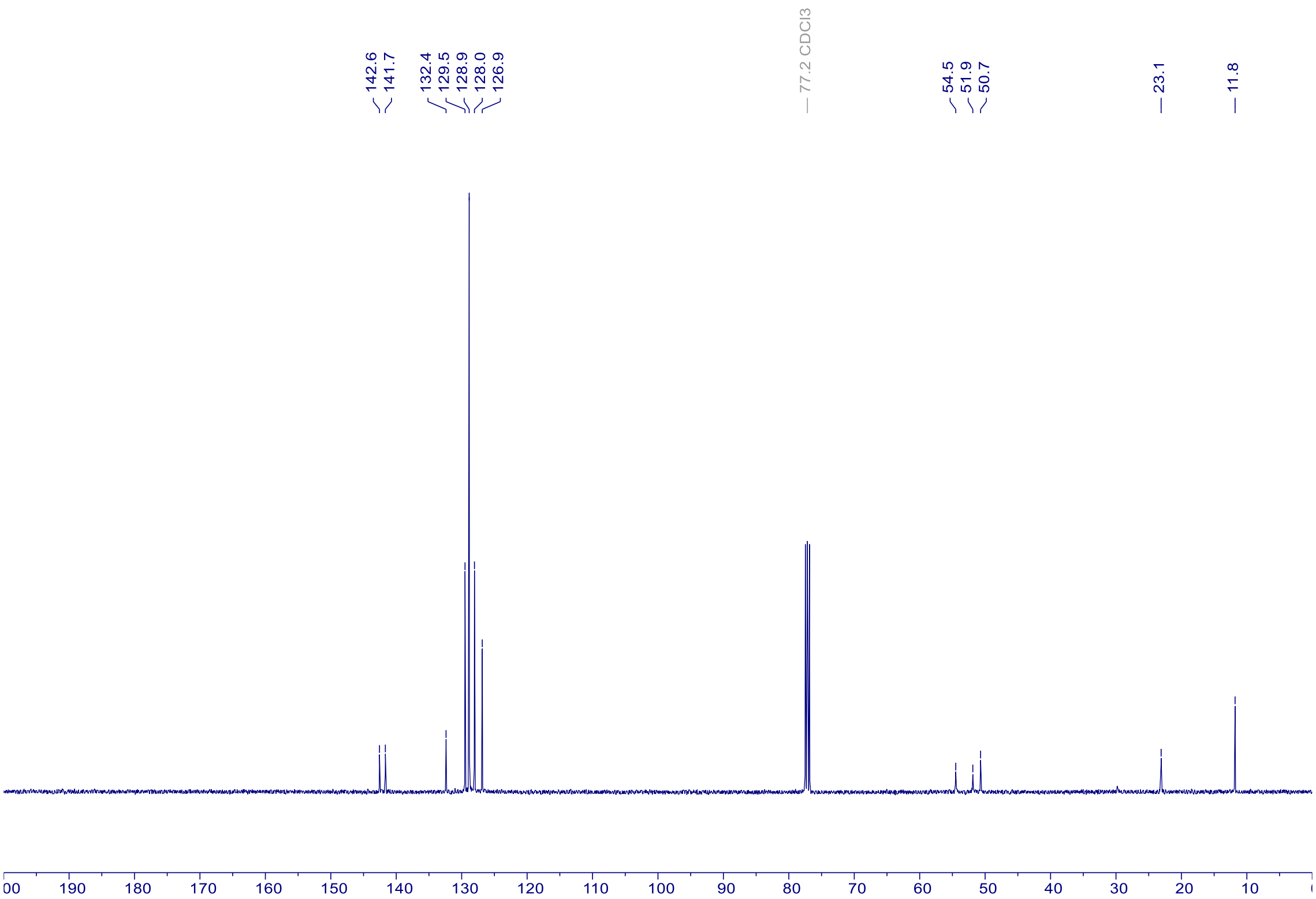

1h/6b – ^{1}H NMR (600 MHz, $CDCl_3$)

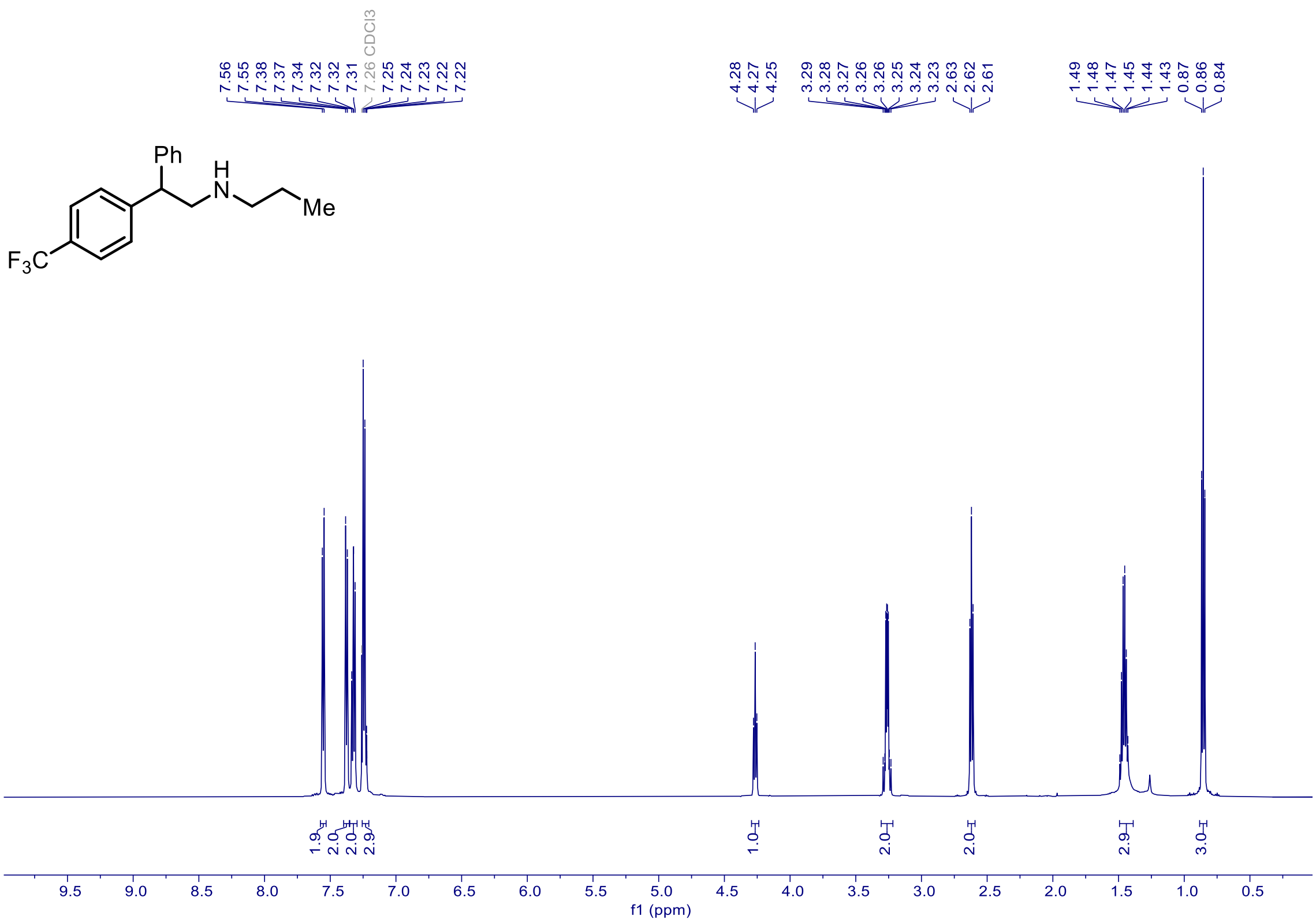

1h/6b – ^{13}C NMR (151 MHz, $CDCl_3$)

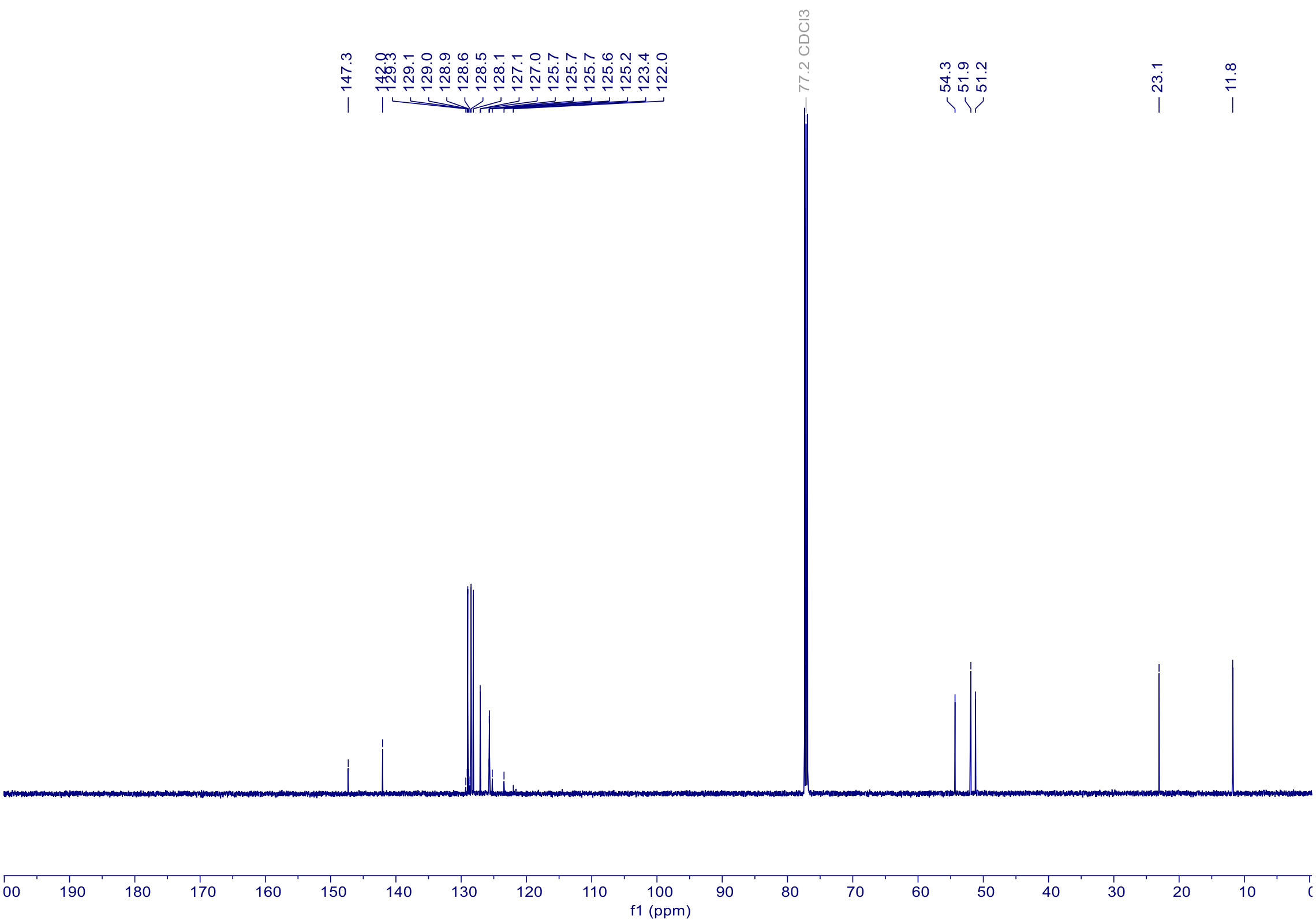

1h/6b – ^{19}F NMR (565 MHz, $CDCl_3$)

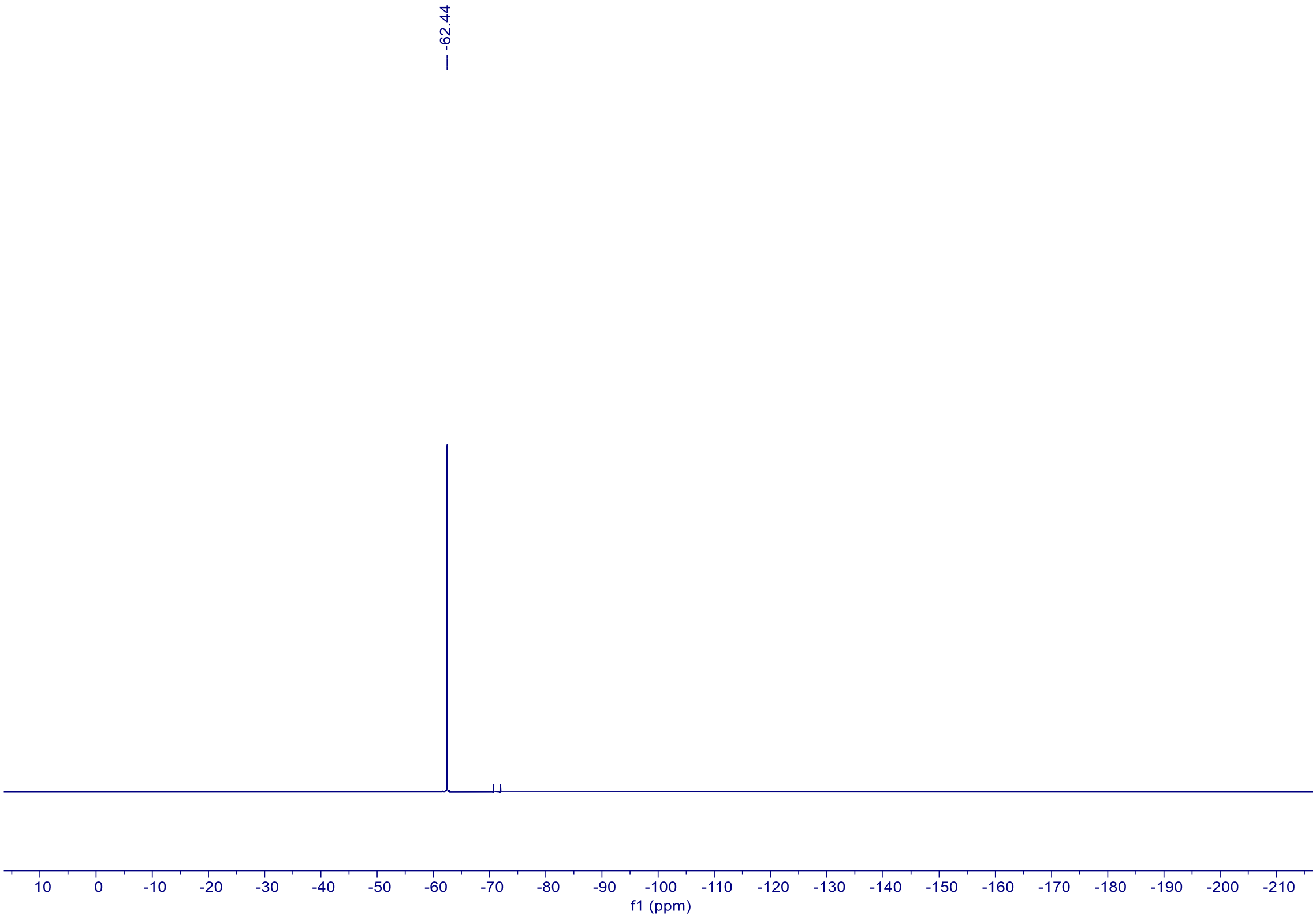

1i – ^{1}H NMR (600 MHz, CDCl$_3$)

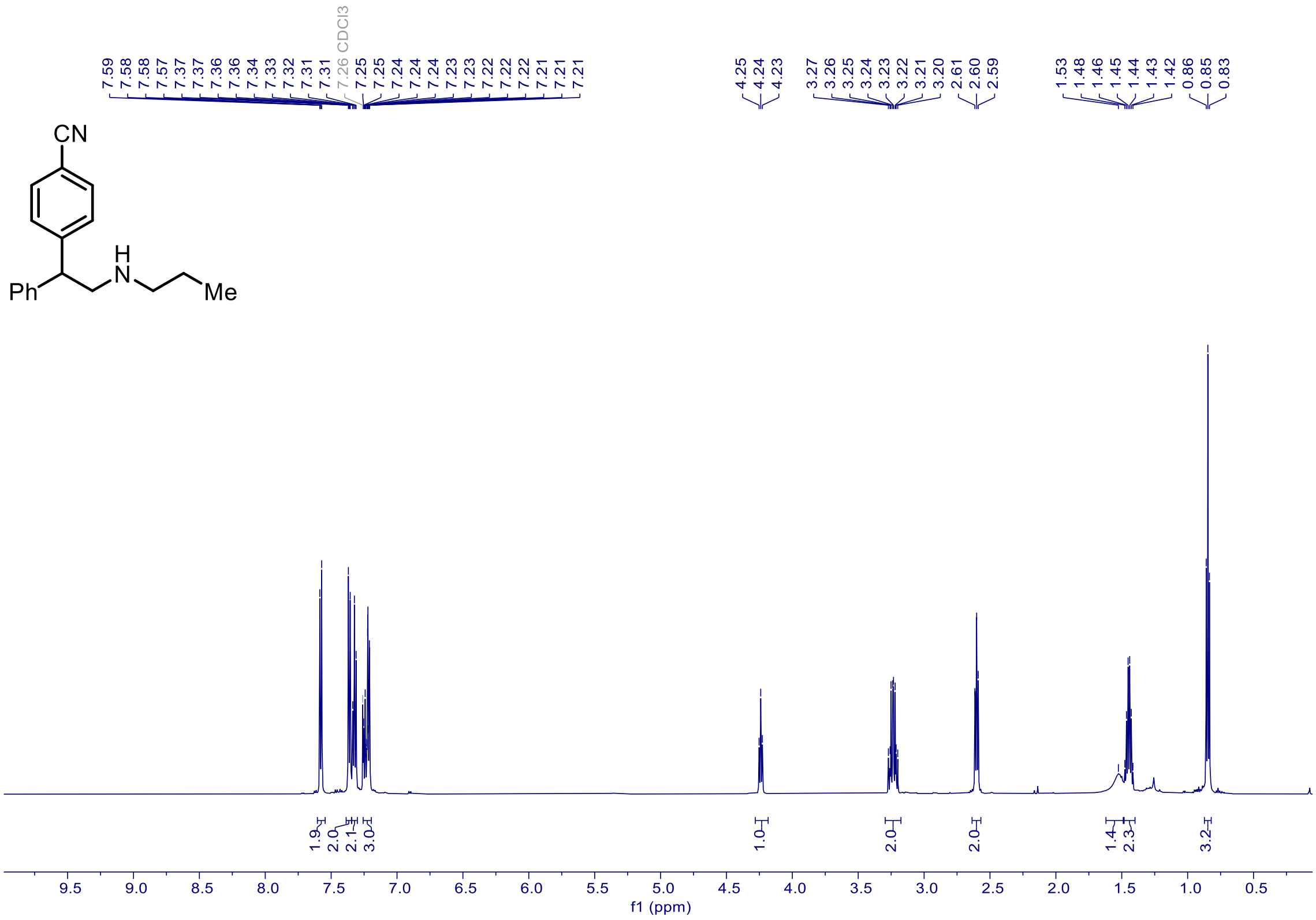

1i – ^{13}C NMR (151 MHz, CDCl$_3$)

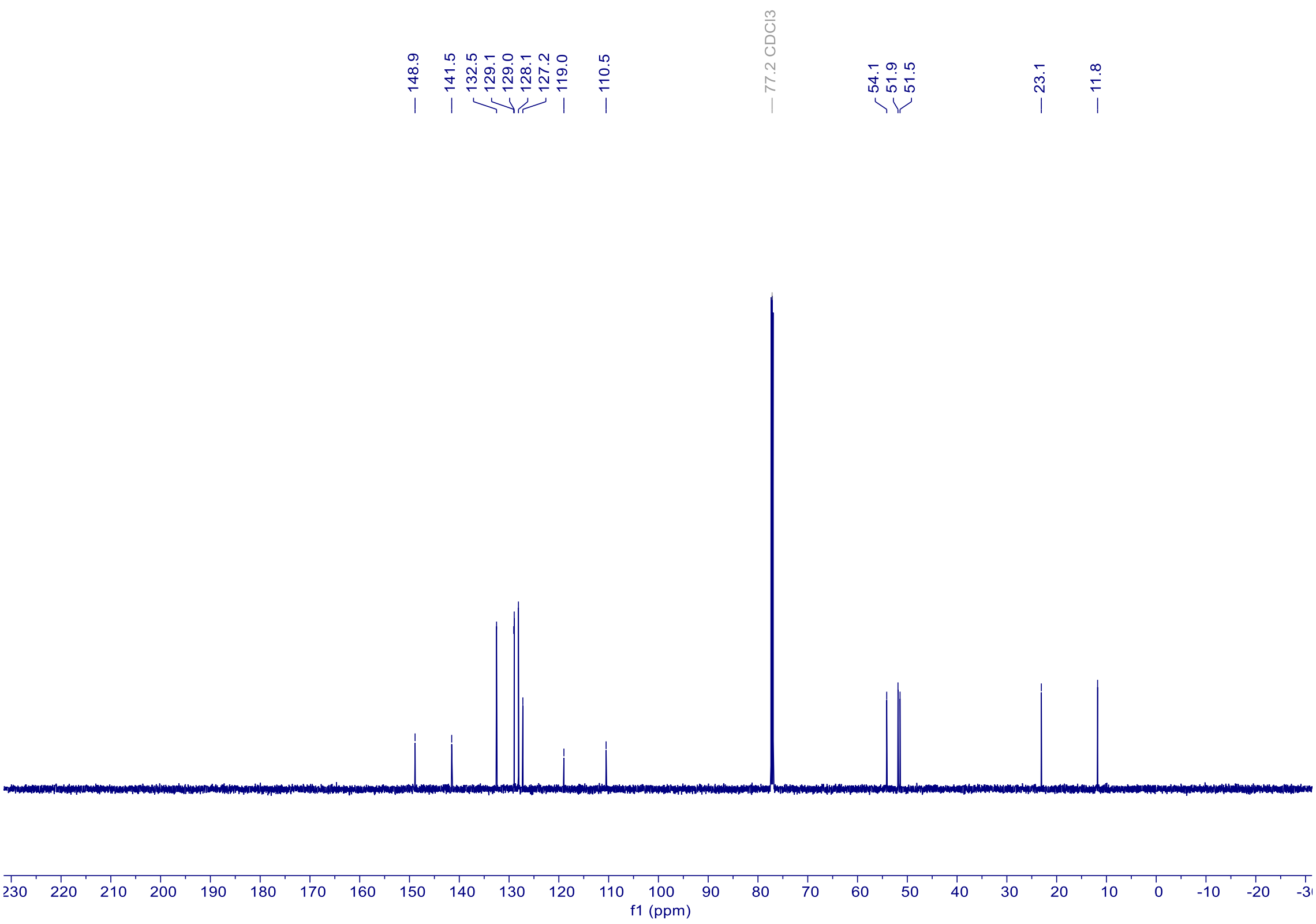

1j – ^{1}H NMR (600 MHz, $CDCl_3$)

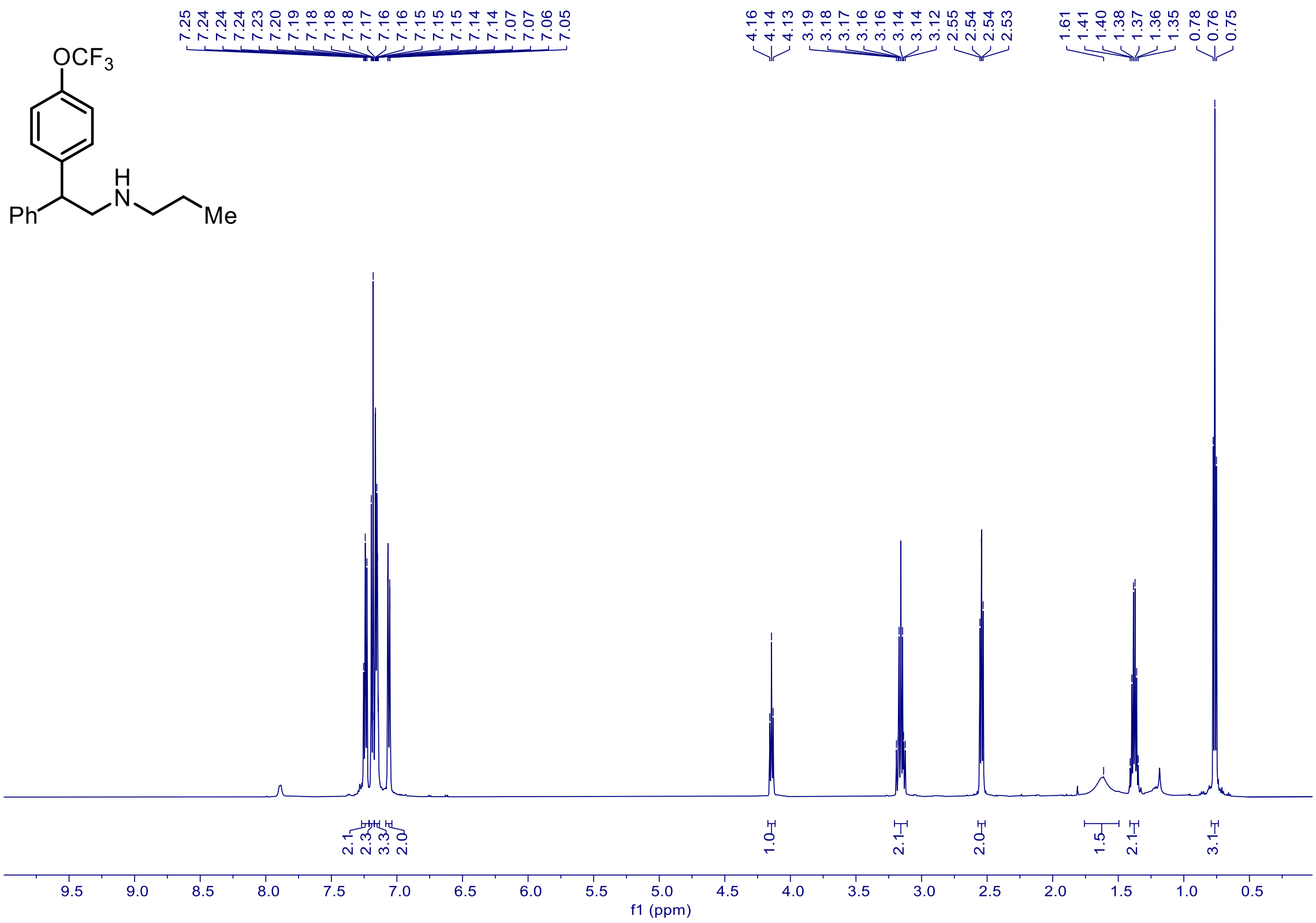

1j – ^{13}C NMR (151 MHz, $CDCl_3$)

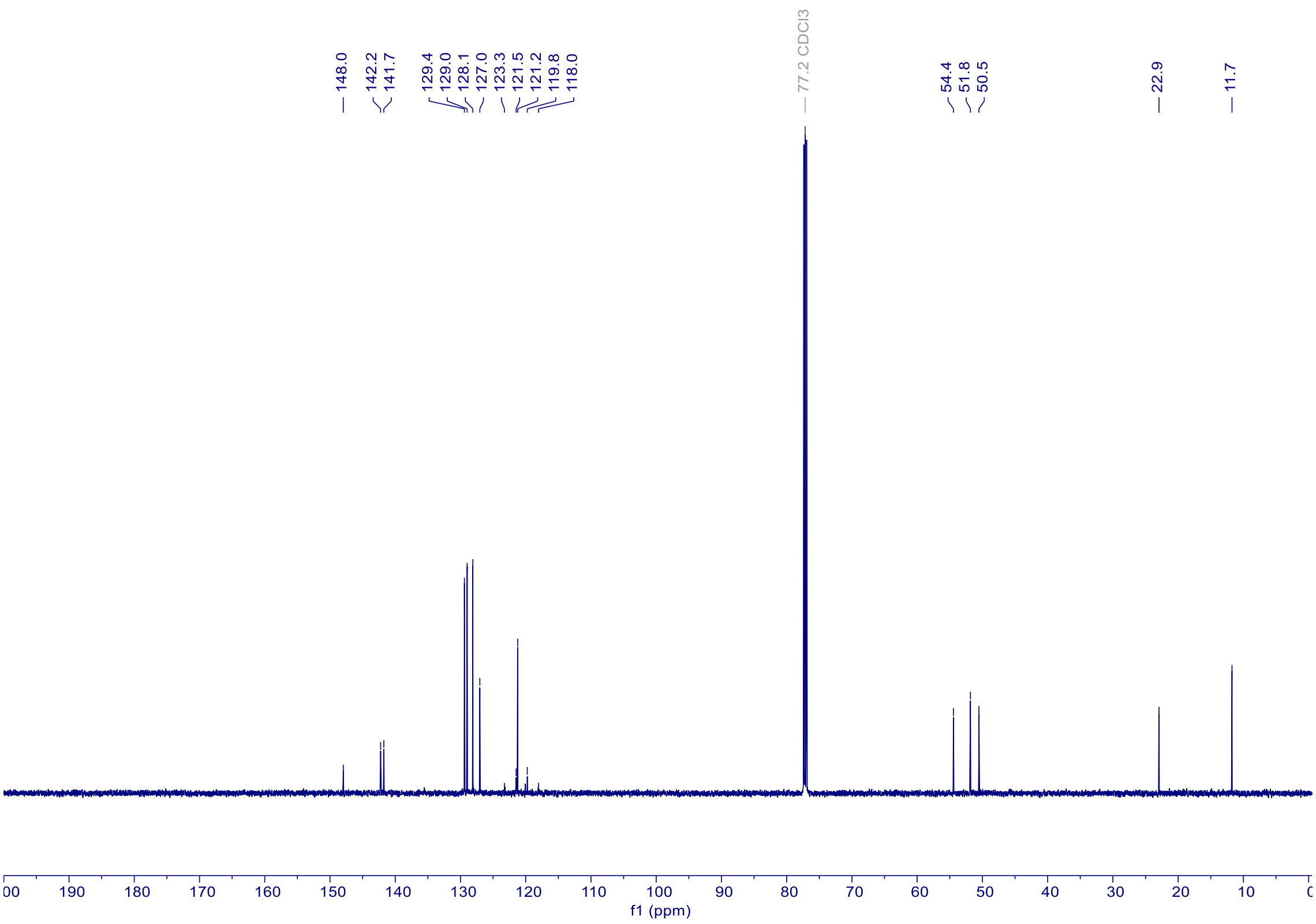

1j – ^{19}F NMR (565 MHz, $CDCl_3$)

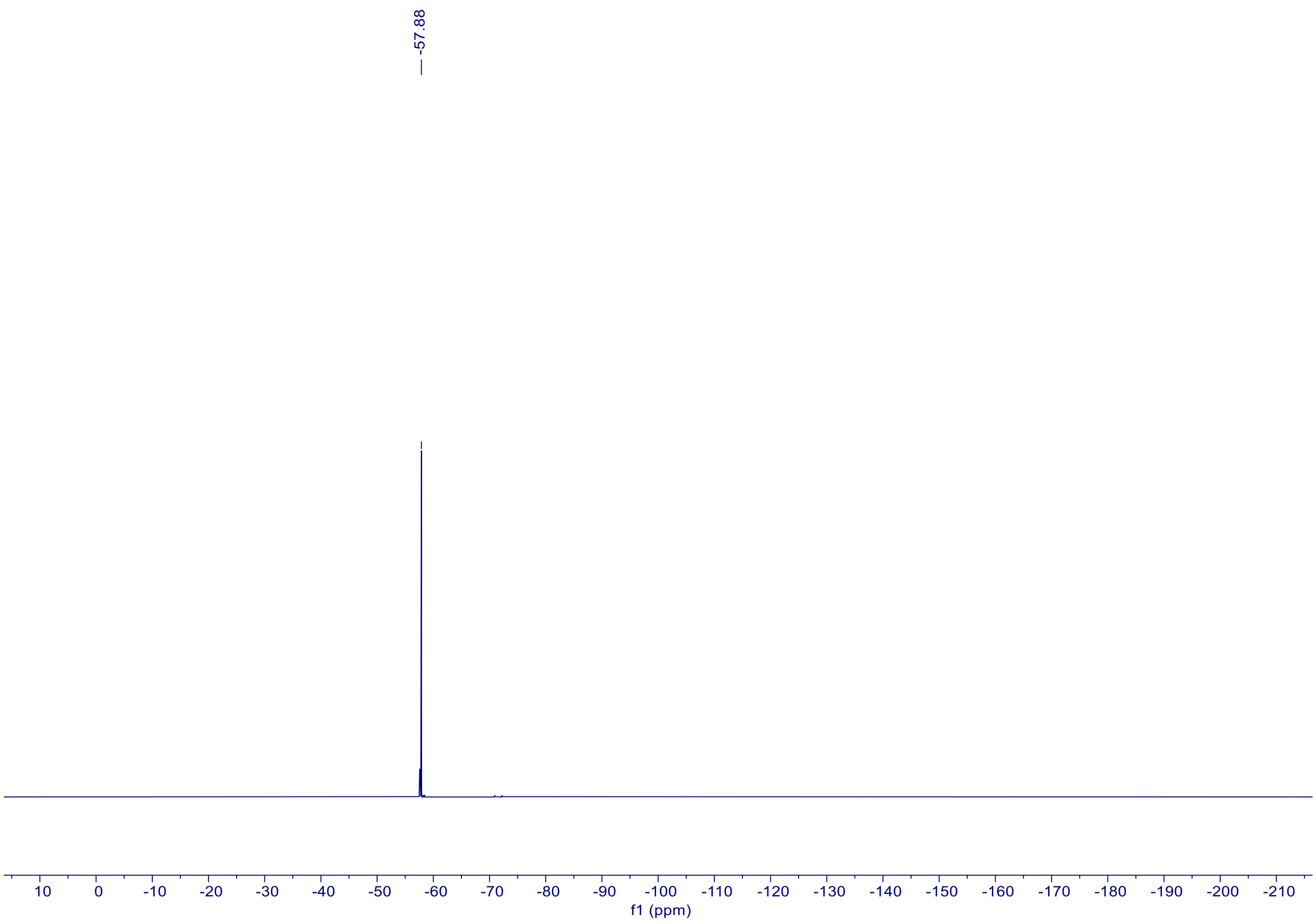

1k – ^{1}H NMR (600 MHz, $CDCl_3$)

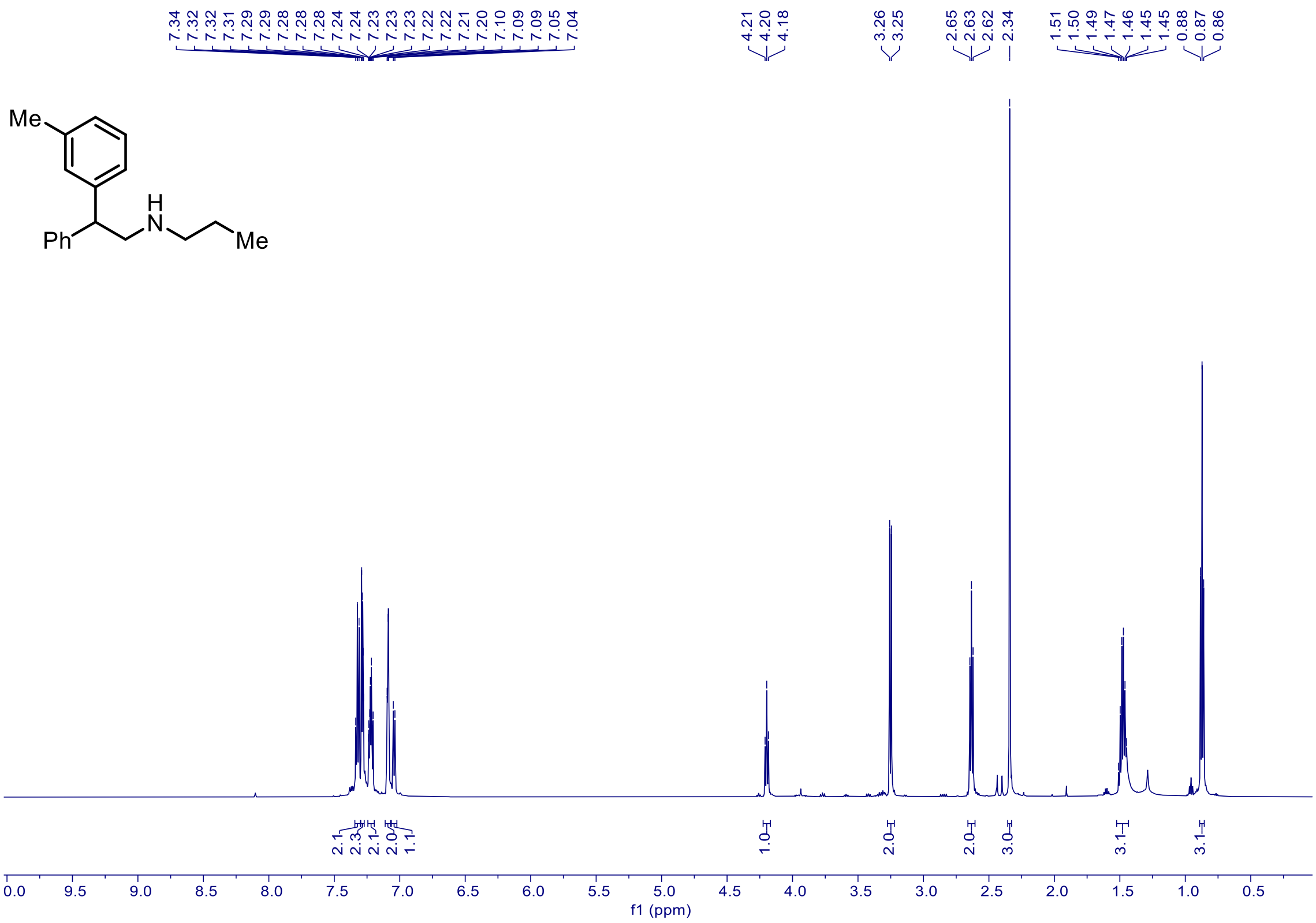

1k – ^{13}C NMR (151 MHz, $CDCl_3$)

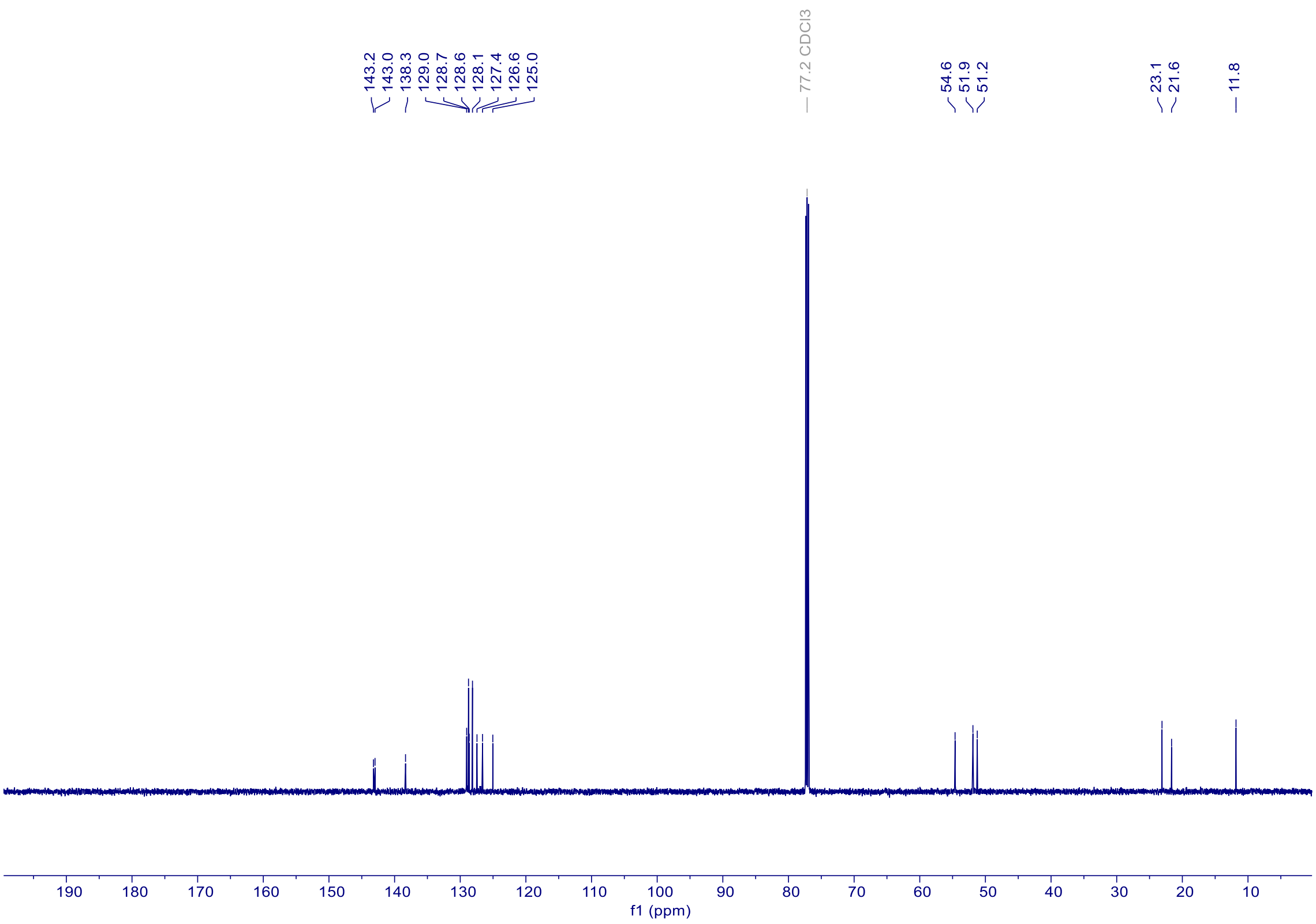

1l – ^{1}H NMR (400 MHz, $CDCl_3$)

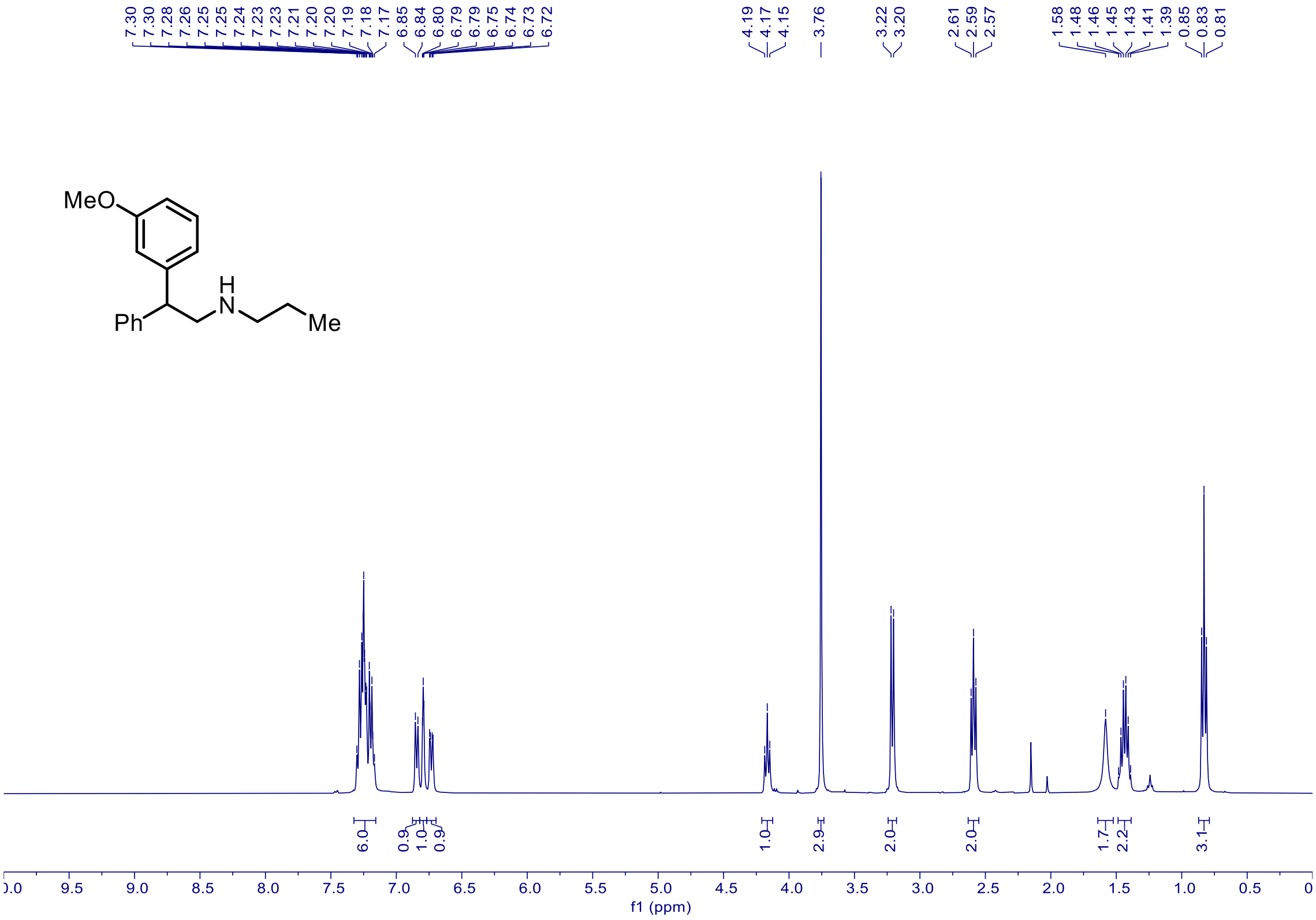

1l – ^{13}C NMR (101 MHz, $CDCl_3$)

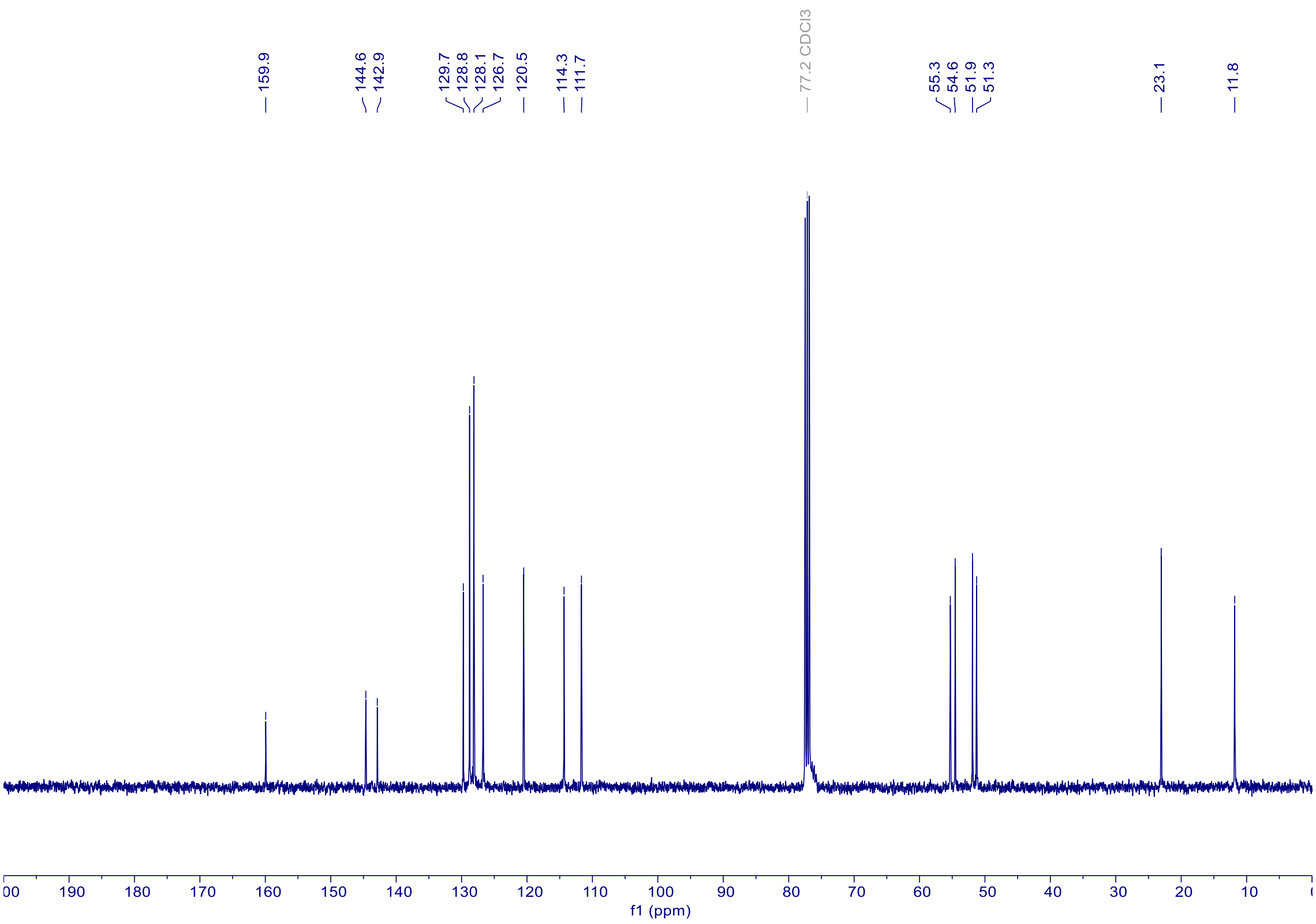

1m – ^{1}H NMR (600 MHz, $CDCl_3$)

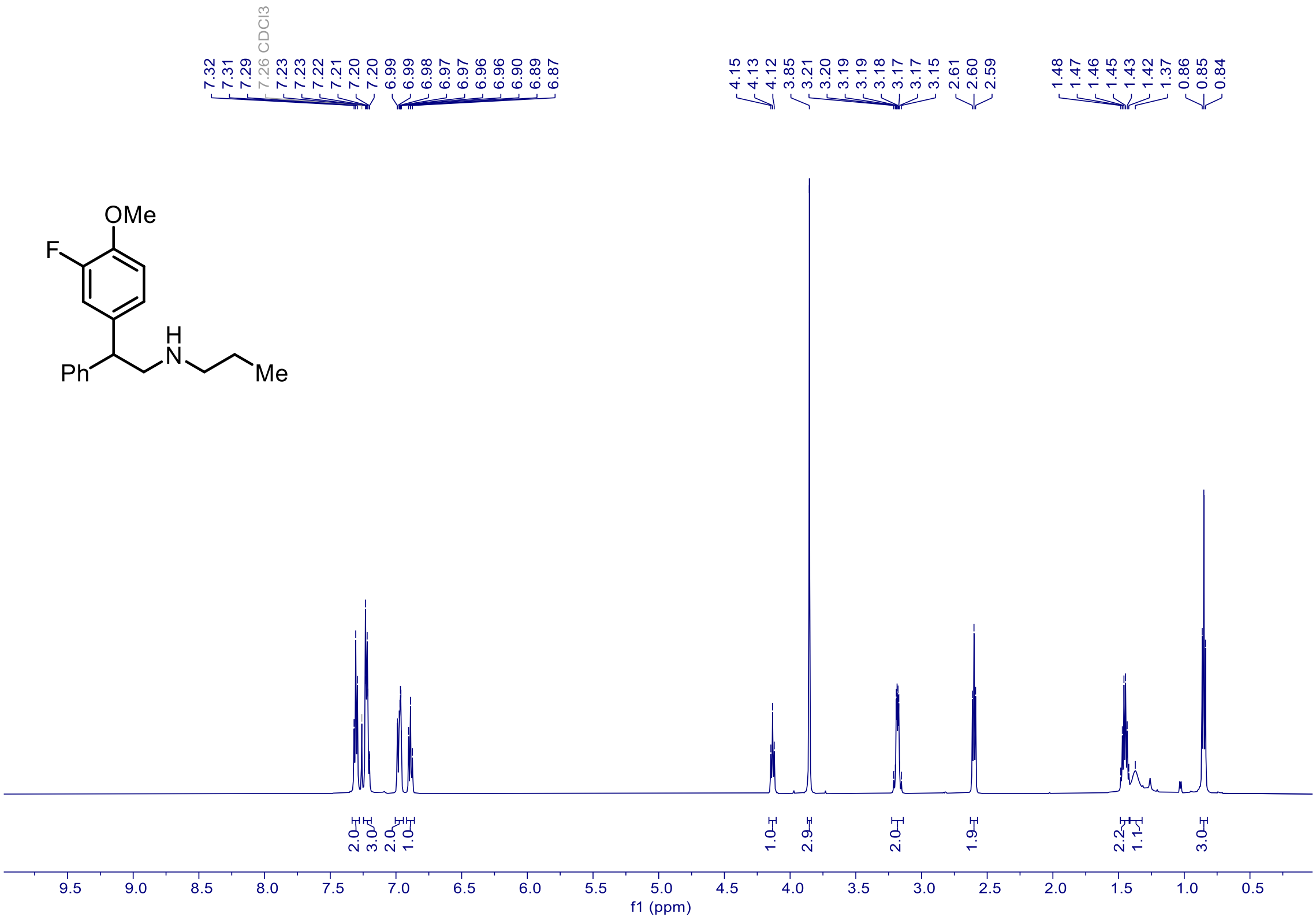

1m – ^{13}C NMR (101 MHz, $CDCl_3$)

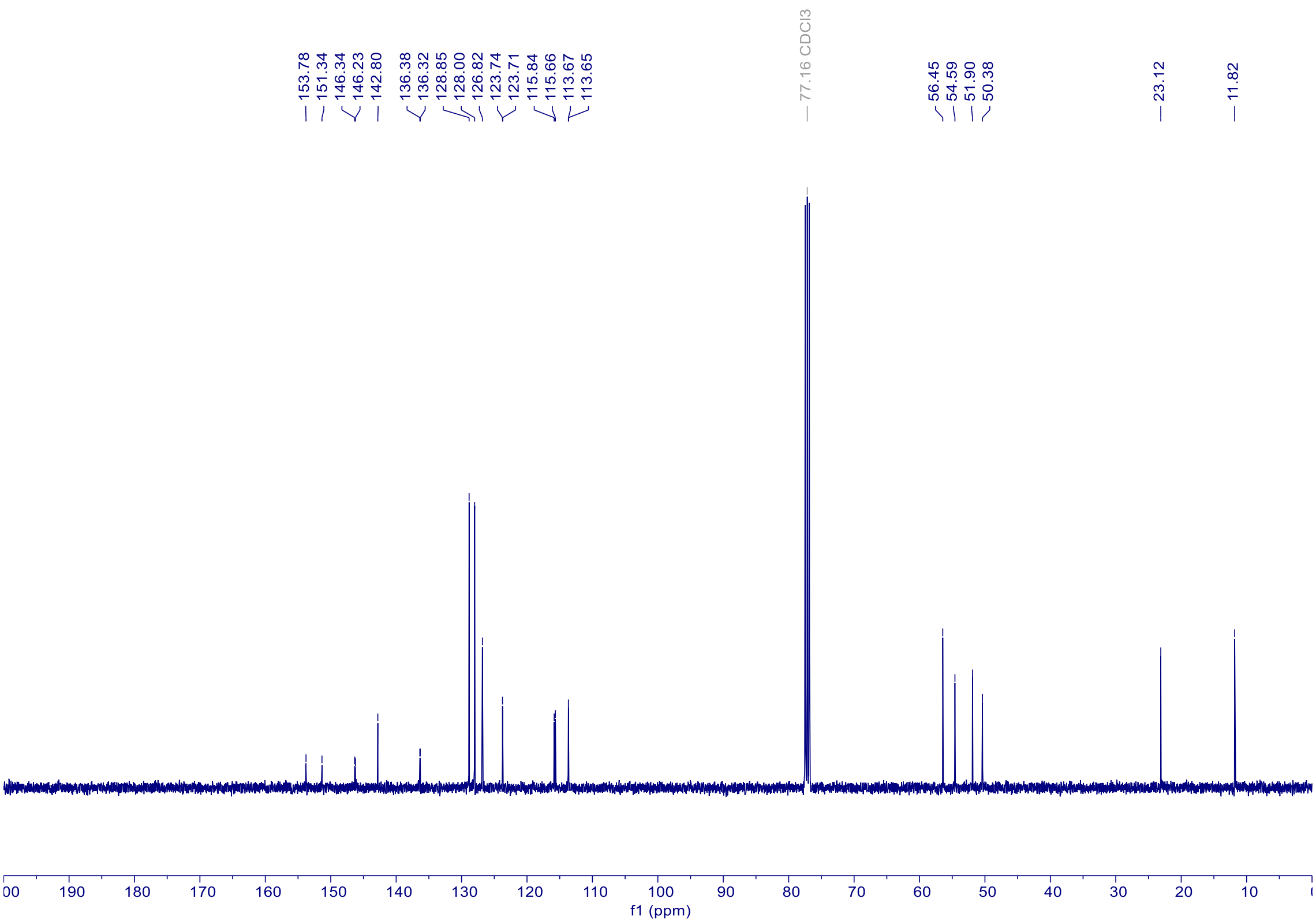

1m – ^{19}F NMR (565 MHz, $CDCl_3$)

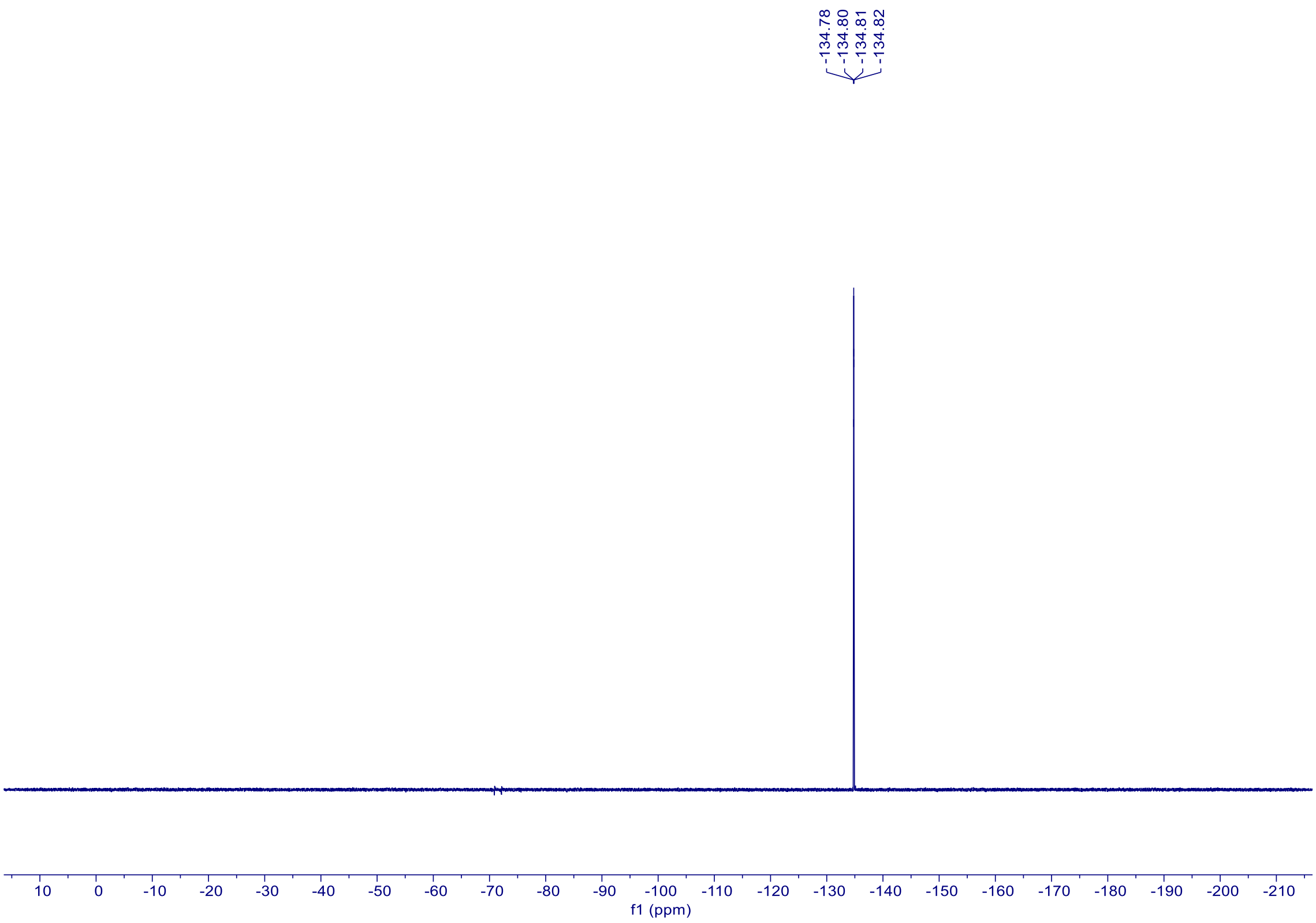

1n – ^{1}H NMR (600 MHz, $CDCl_3$)

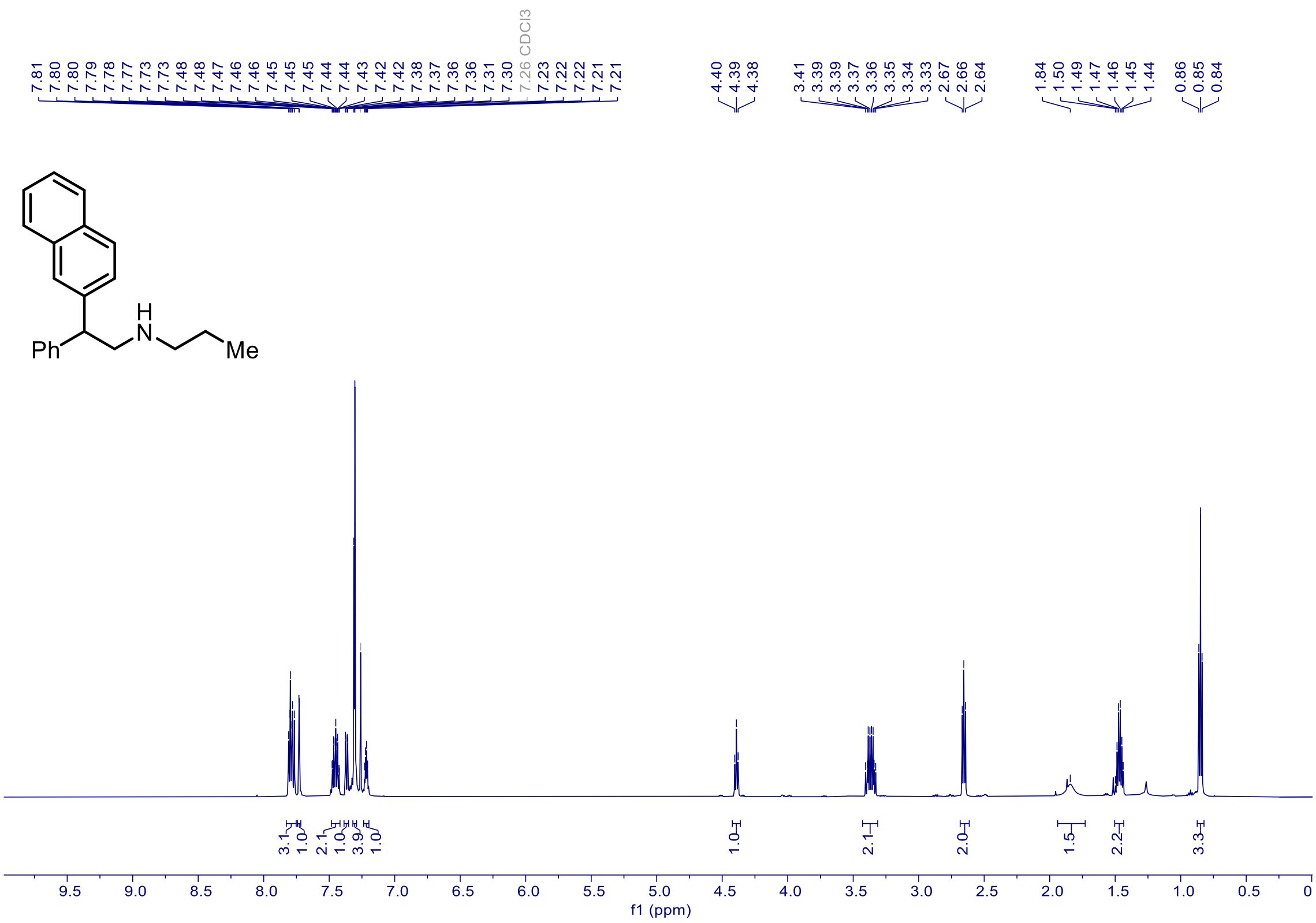

1n – ^{13}C NMR (151 MHz, $CDCl_3$)

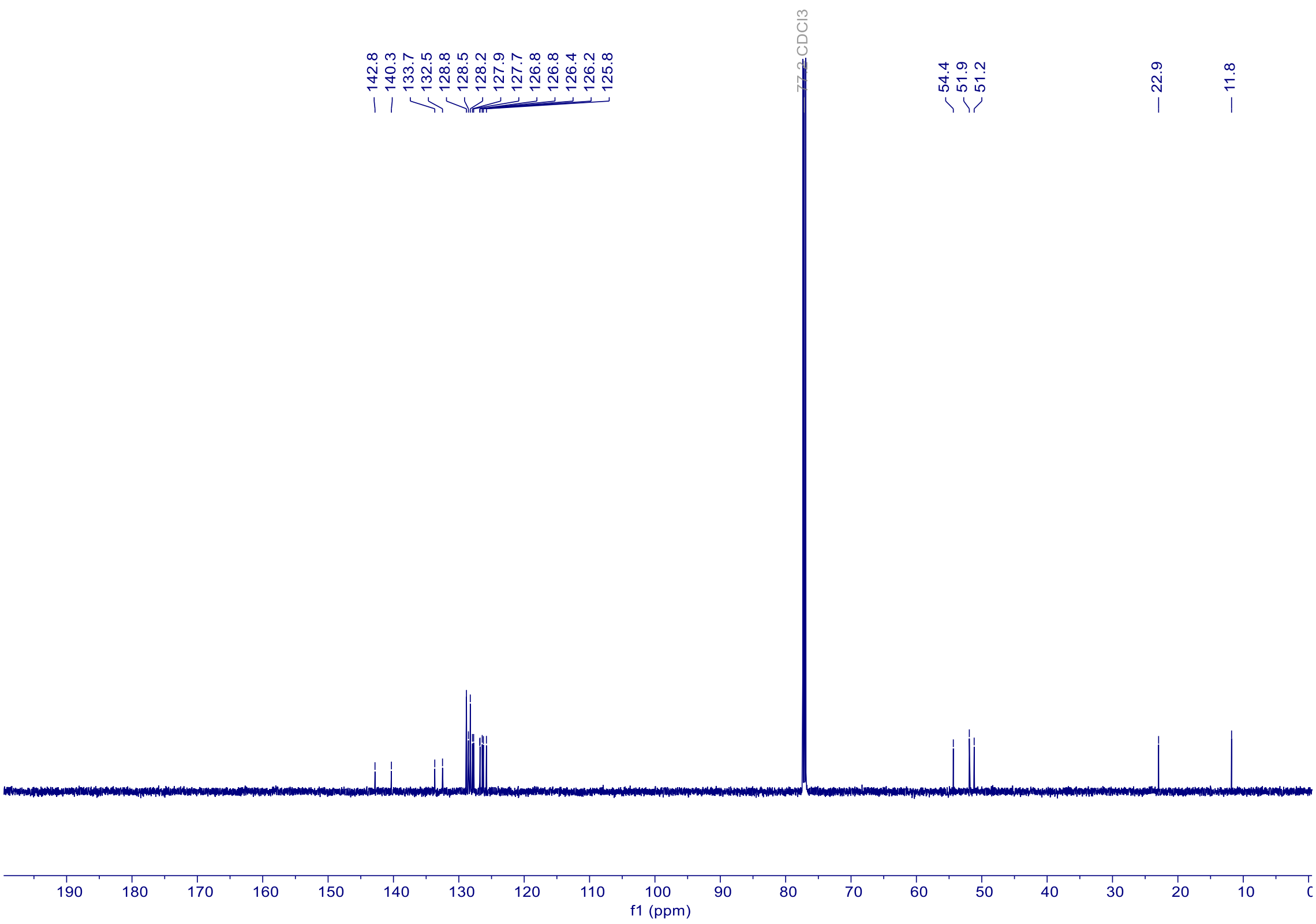

1o – ^{1}H NMR (600 MHz, $CDCl_3$)

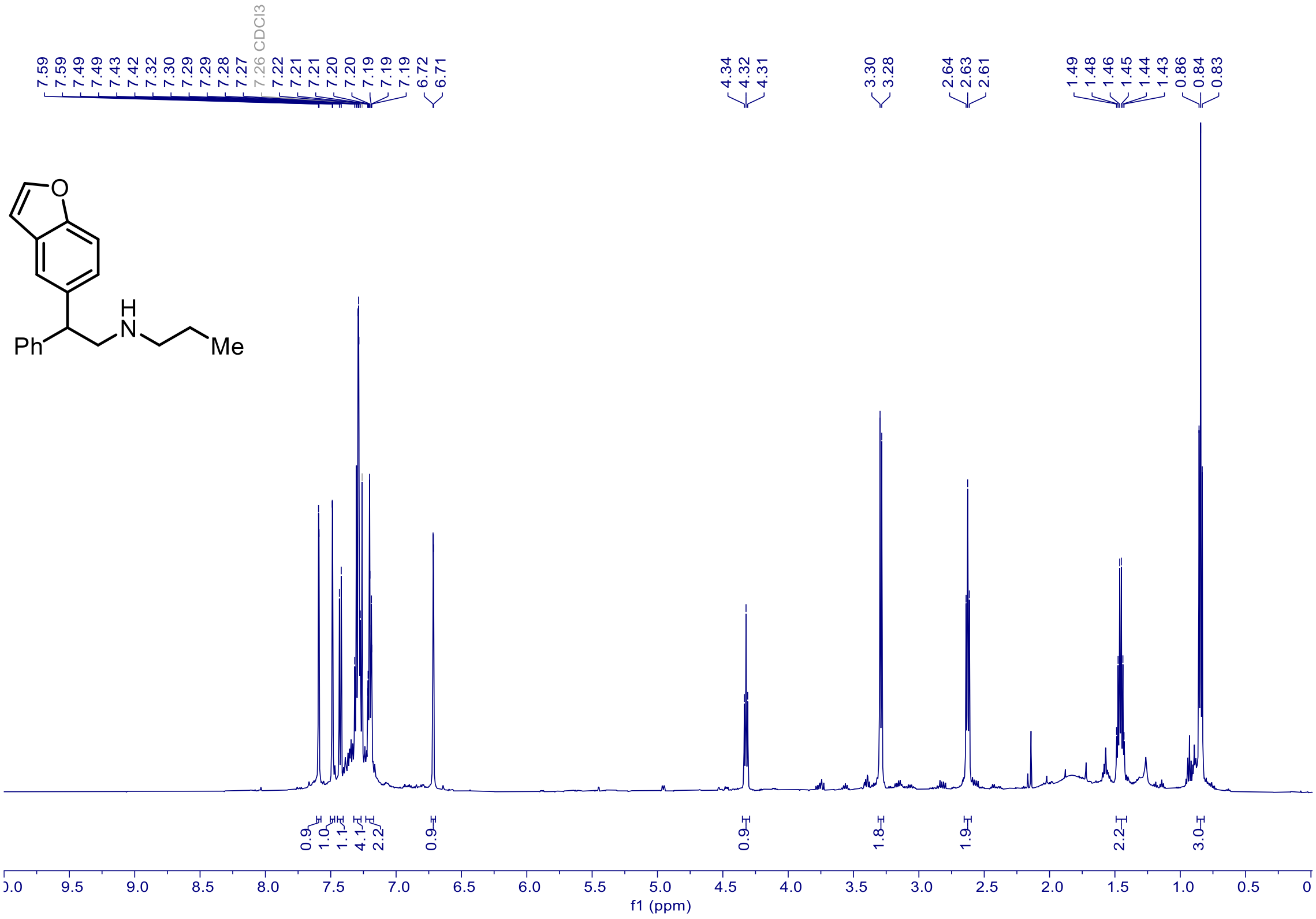

1o – ^{13}C NMR (151 MHz, $CDCl_3$)

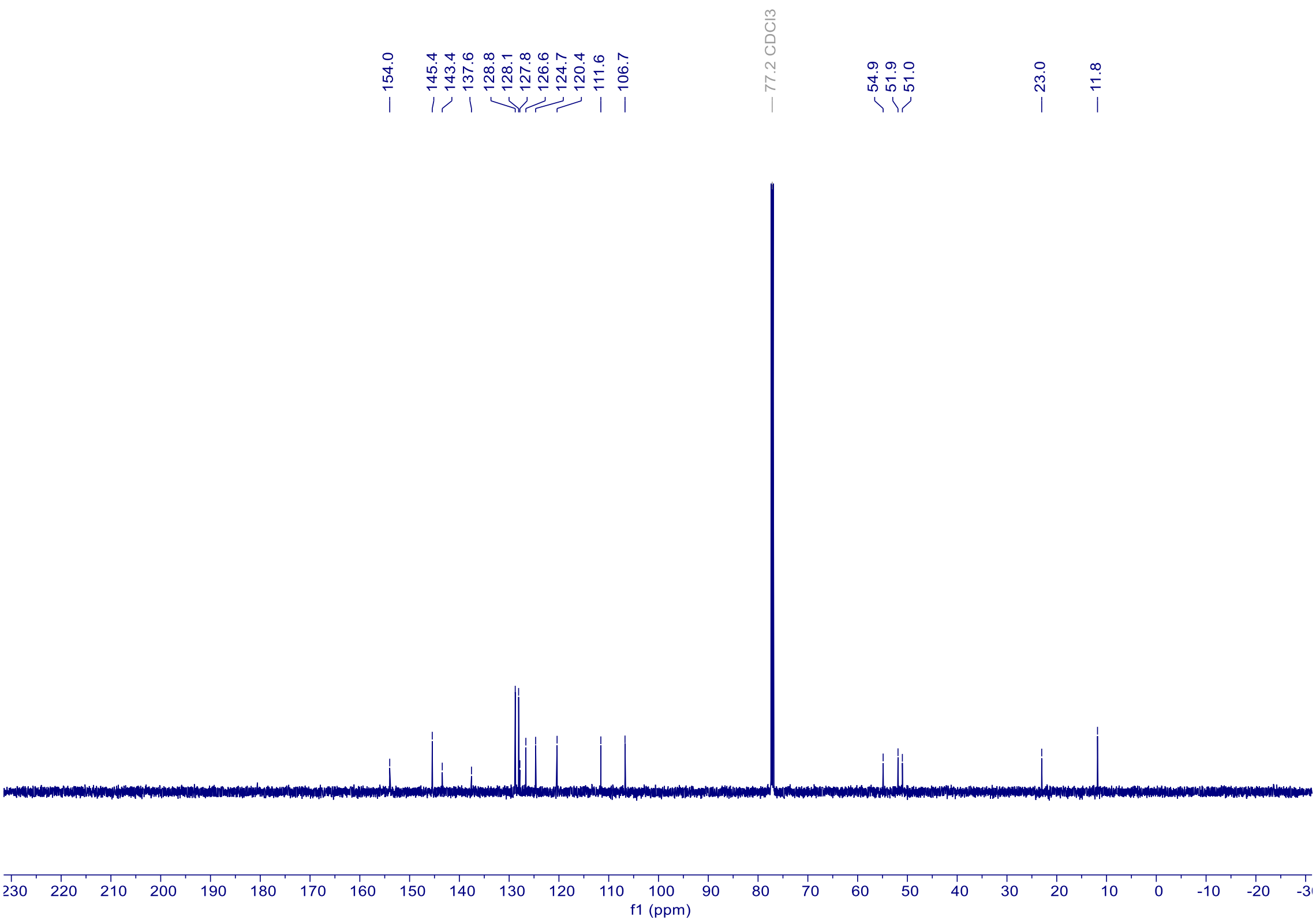

1p – ^{1}H NMR (600 MHz, $CDCl_3$)

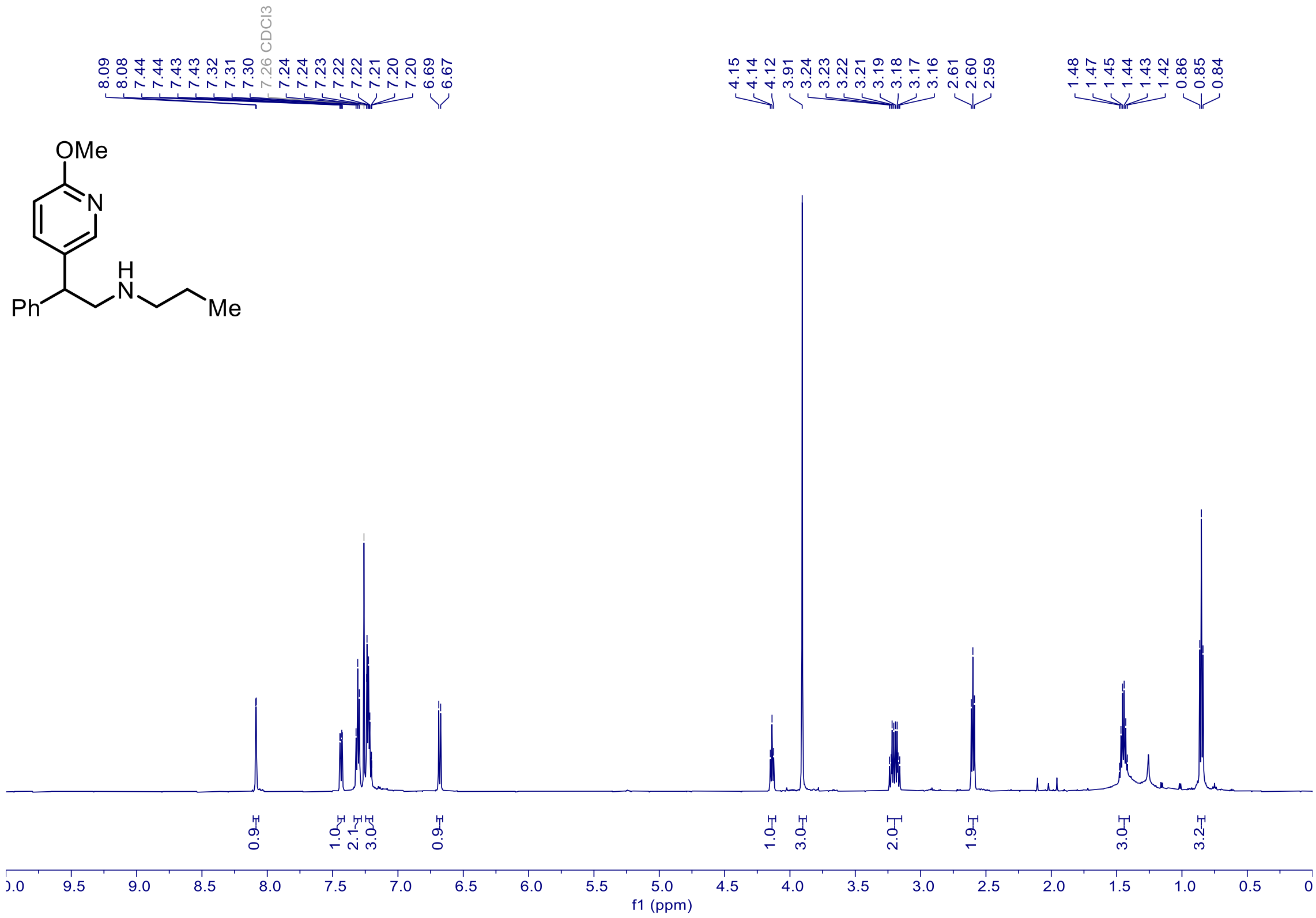

1p – ^{13}C NMR (151 MHz, $CDCl_3$)

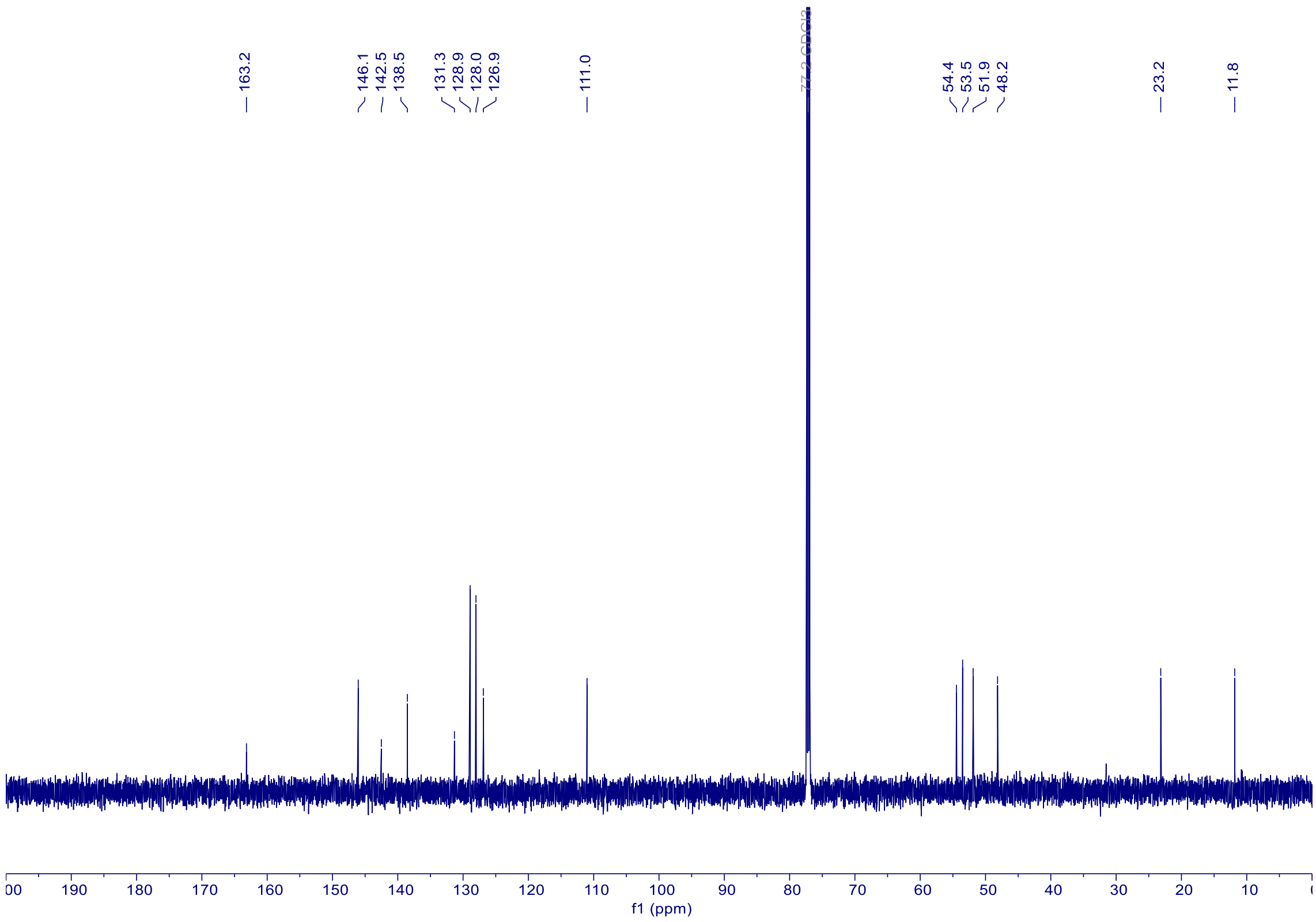

2b – ^{1}H NMR (600 MHz, $CDCl_3$)

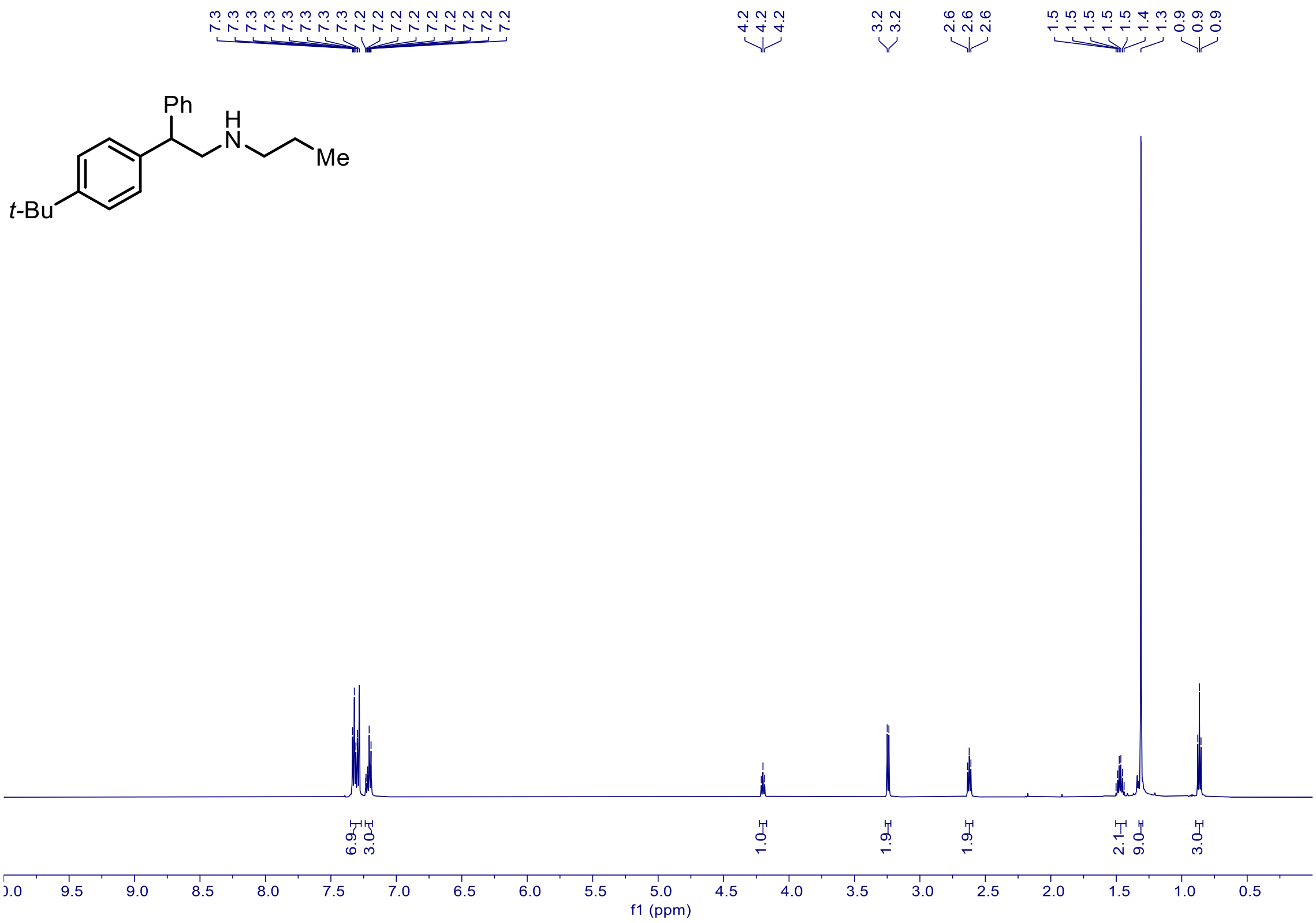

2b – ^{13}C NMR (151 MHz, $CDCl_3$)

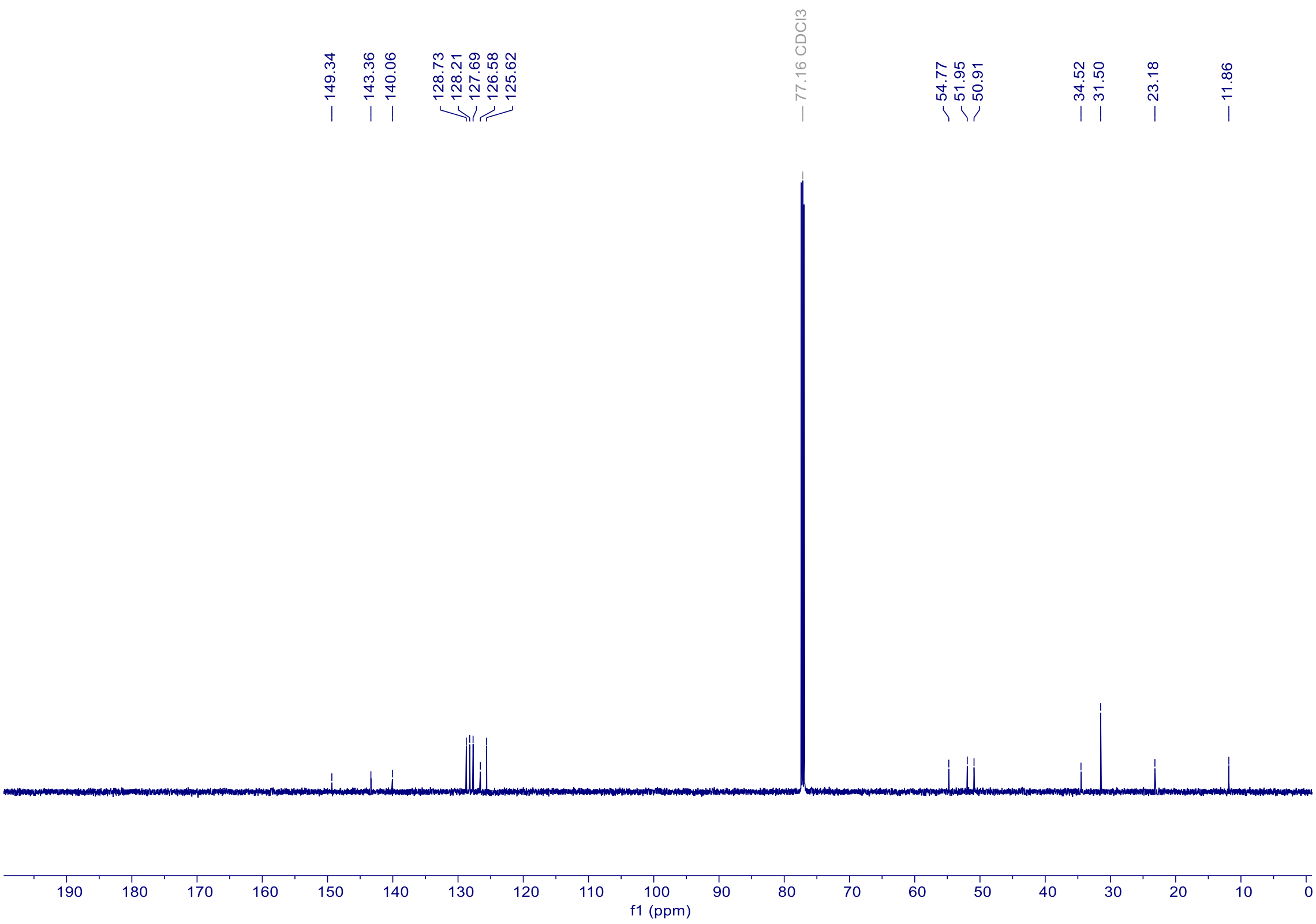

4b – ^{1}H NMR (400 MHz, $CDCl_3$)

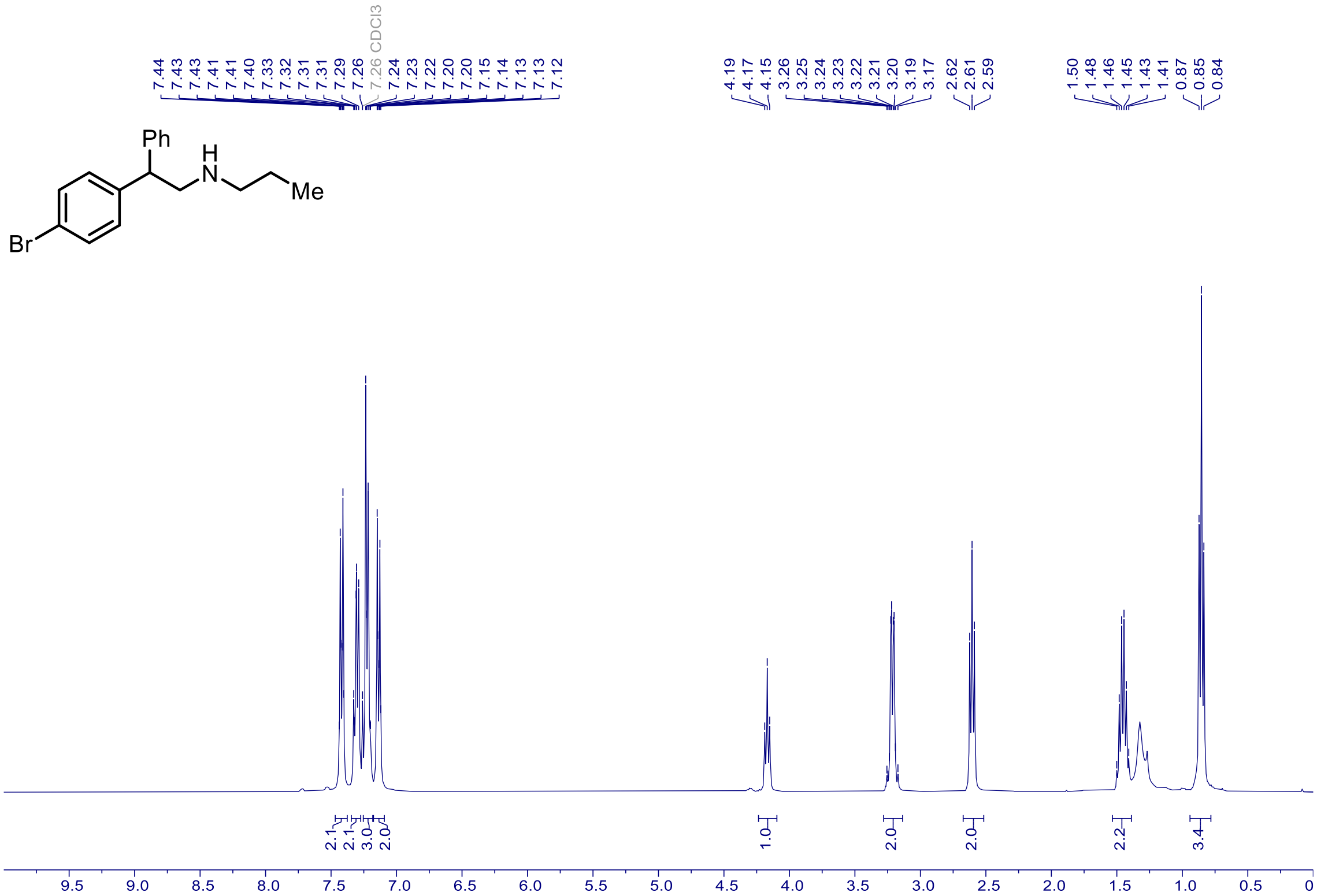

4b – ^{13}C NMR (101 MHz, $CDCl_3$)

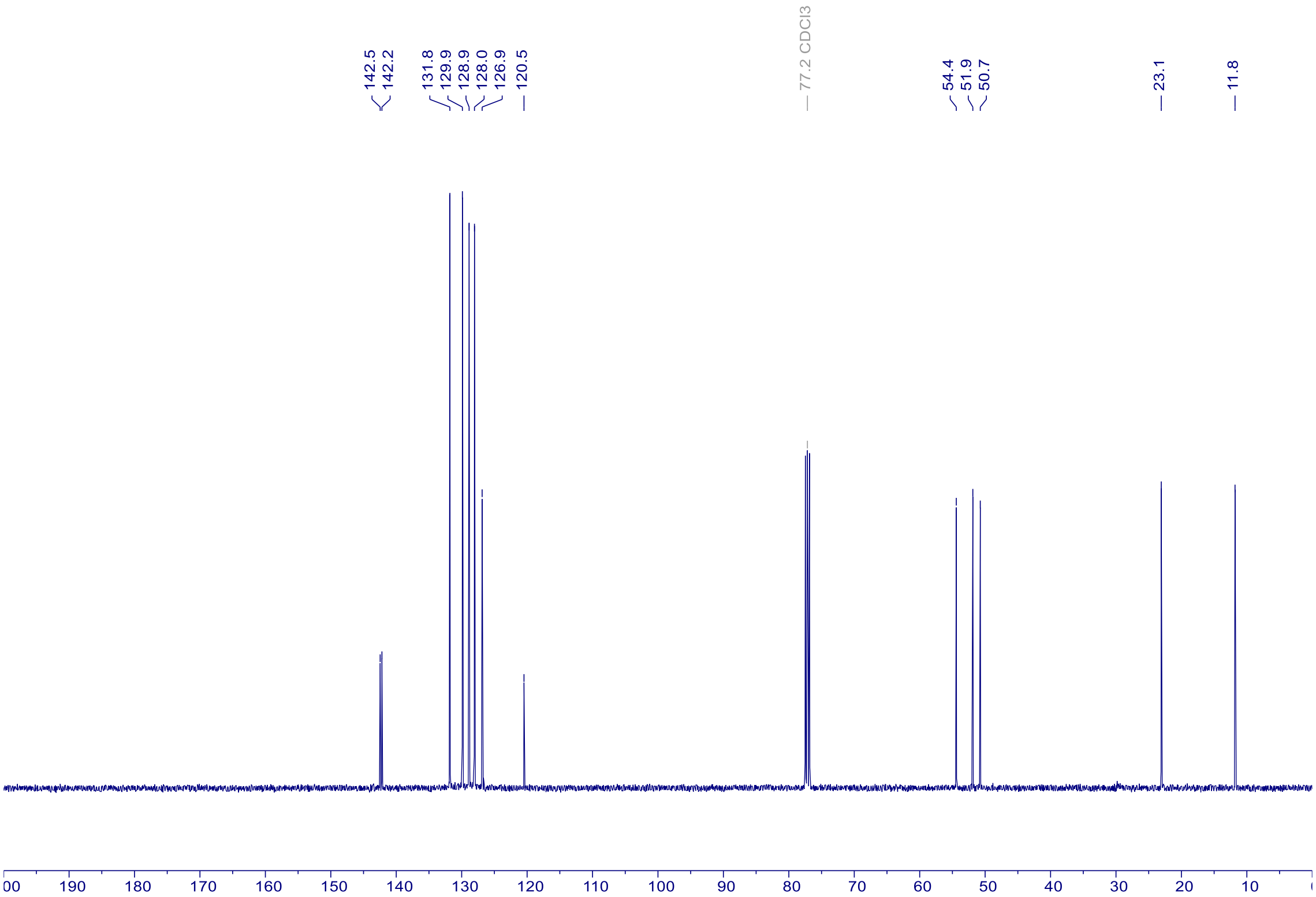

7b – ^{1}H NMR (400 MHz, $CDCl_3$)

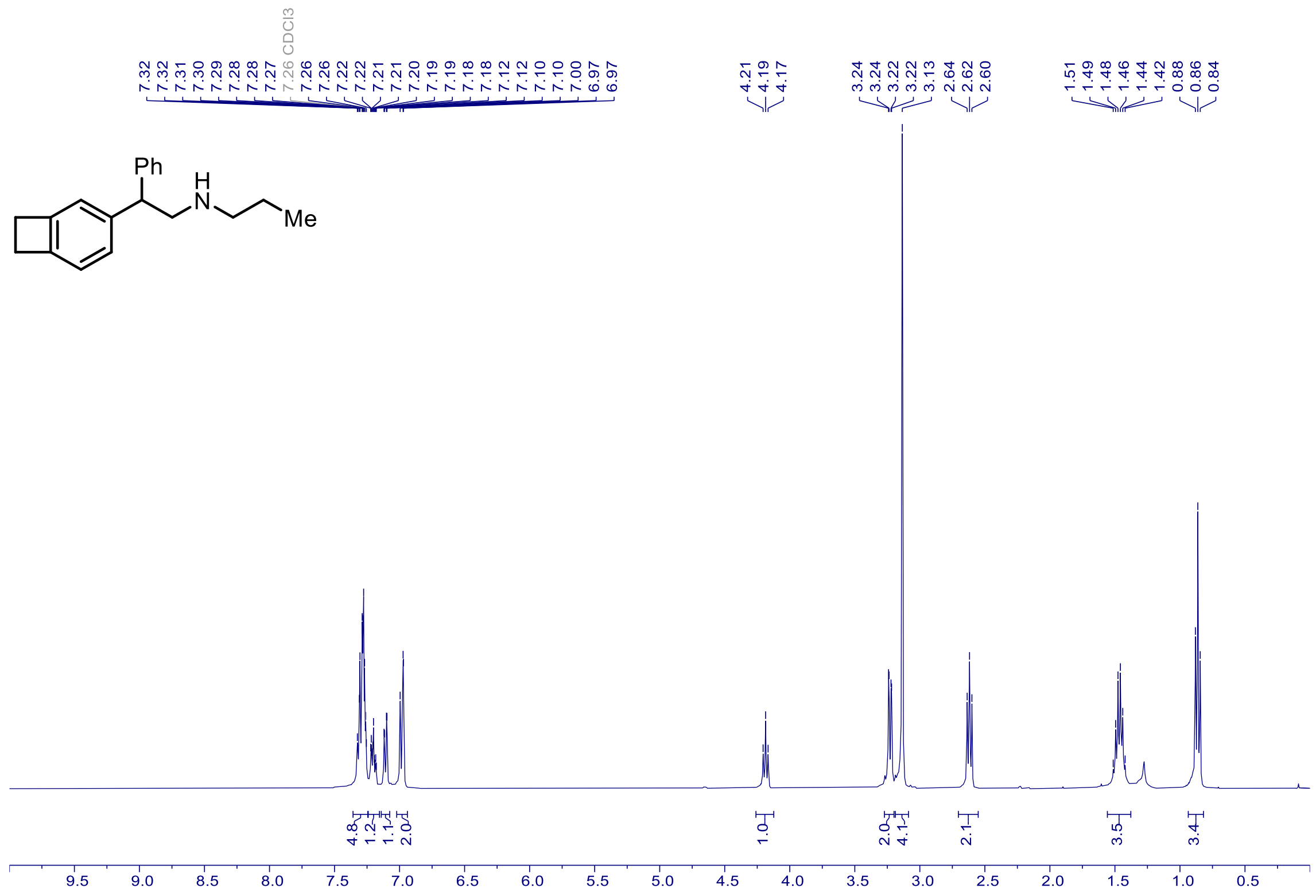

7b – ^{13}C NMR (151 MHz, $CDCl_3$)

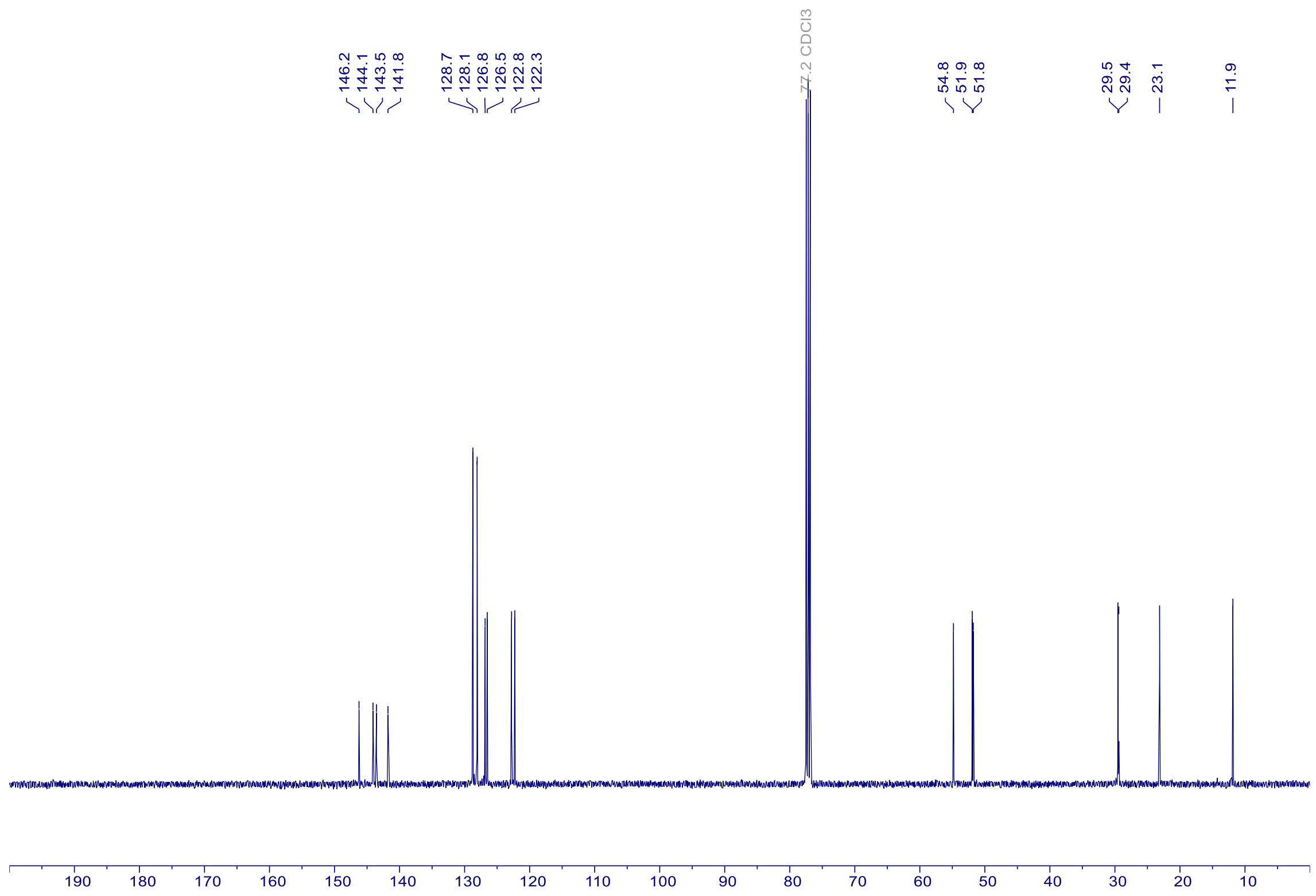

8b – ^{1}H NMR (600 MHz, CDCl$_3$)

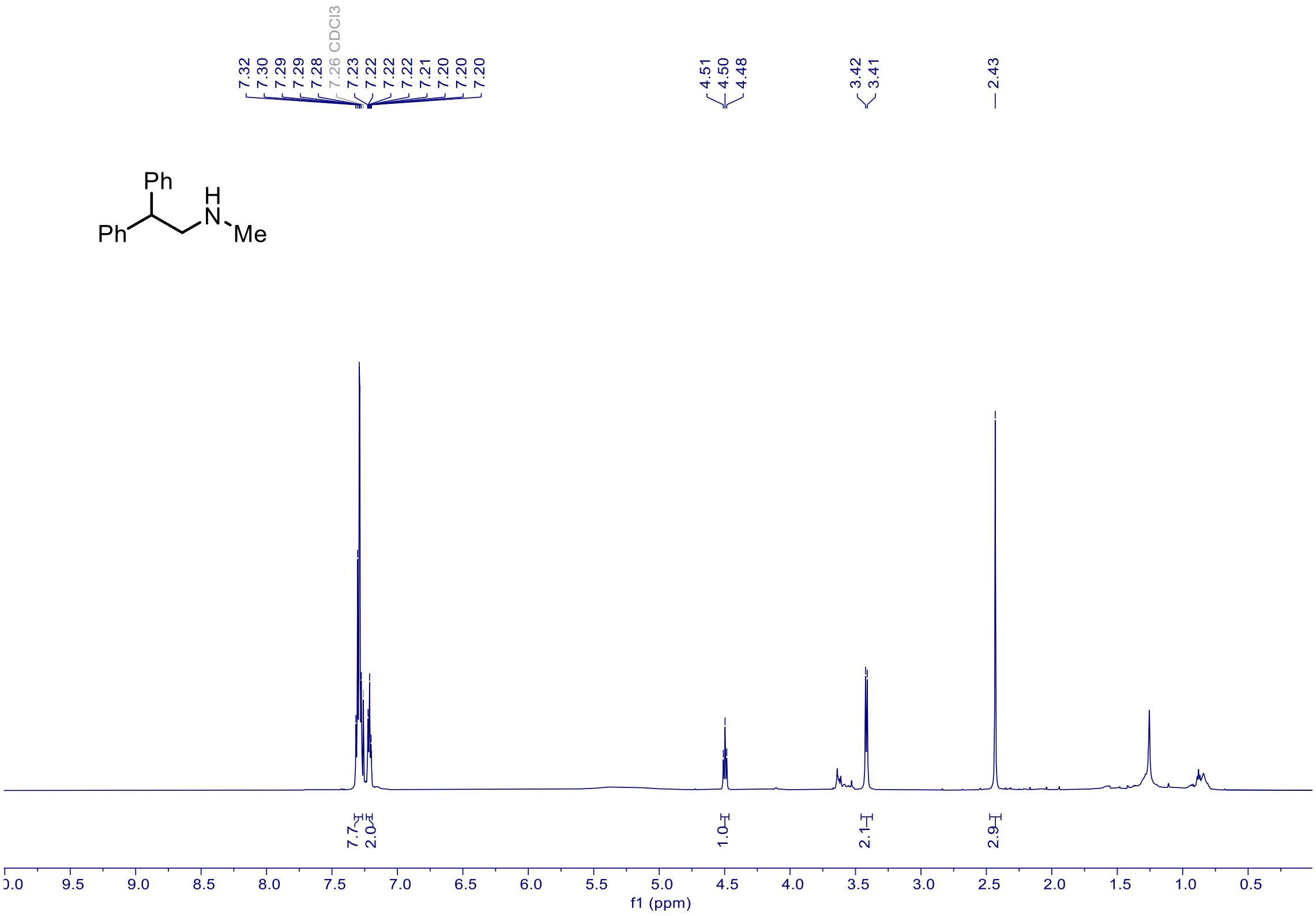

8b – ^{13}C NMR (151 MHz, CDCl$_3$)

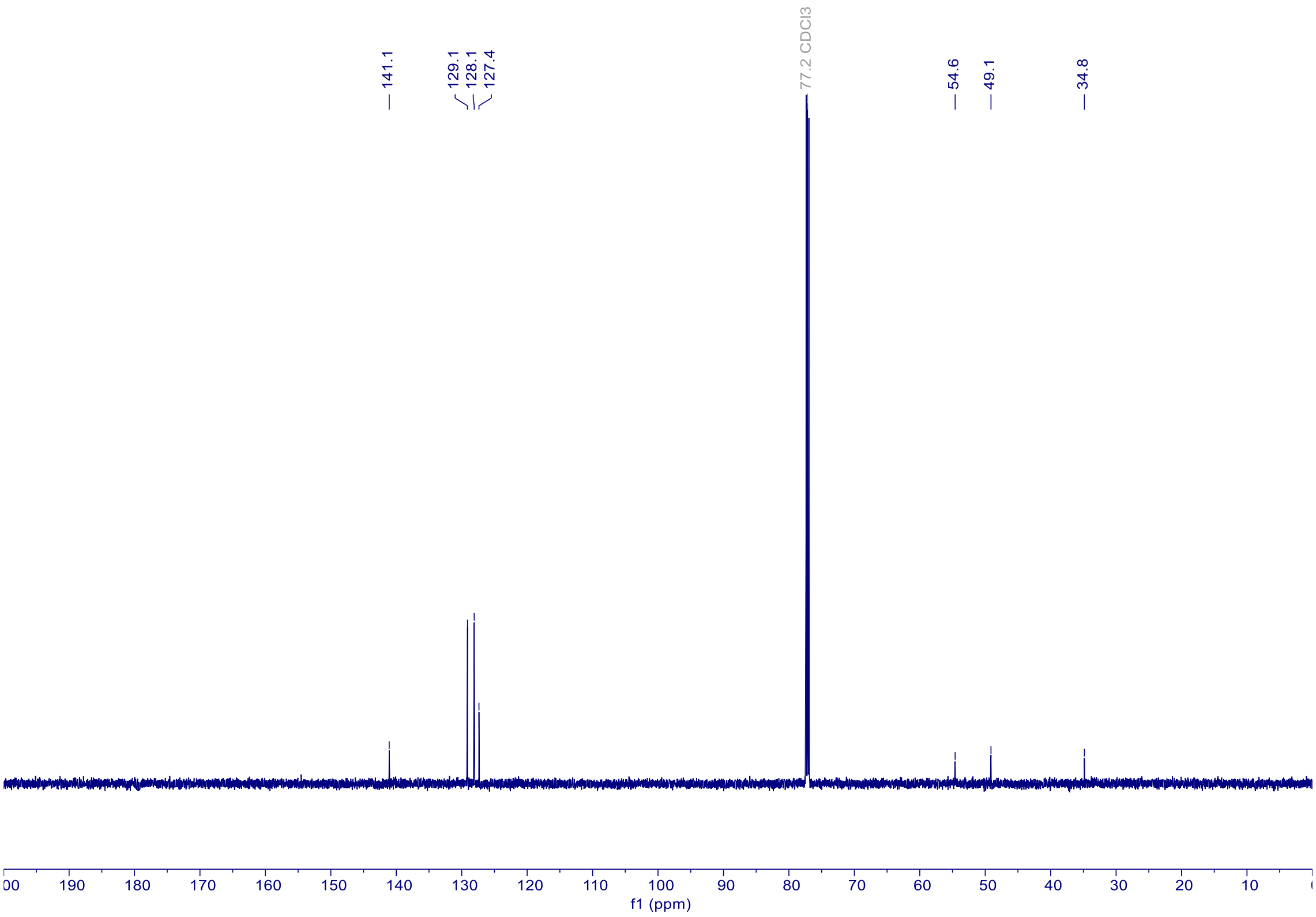

9b– ^{1}H NMR (400 MHz, $CDCl_3$)

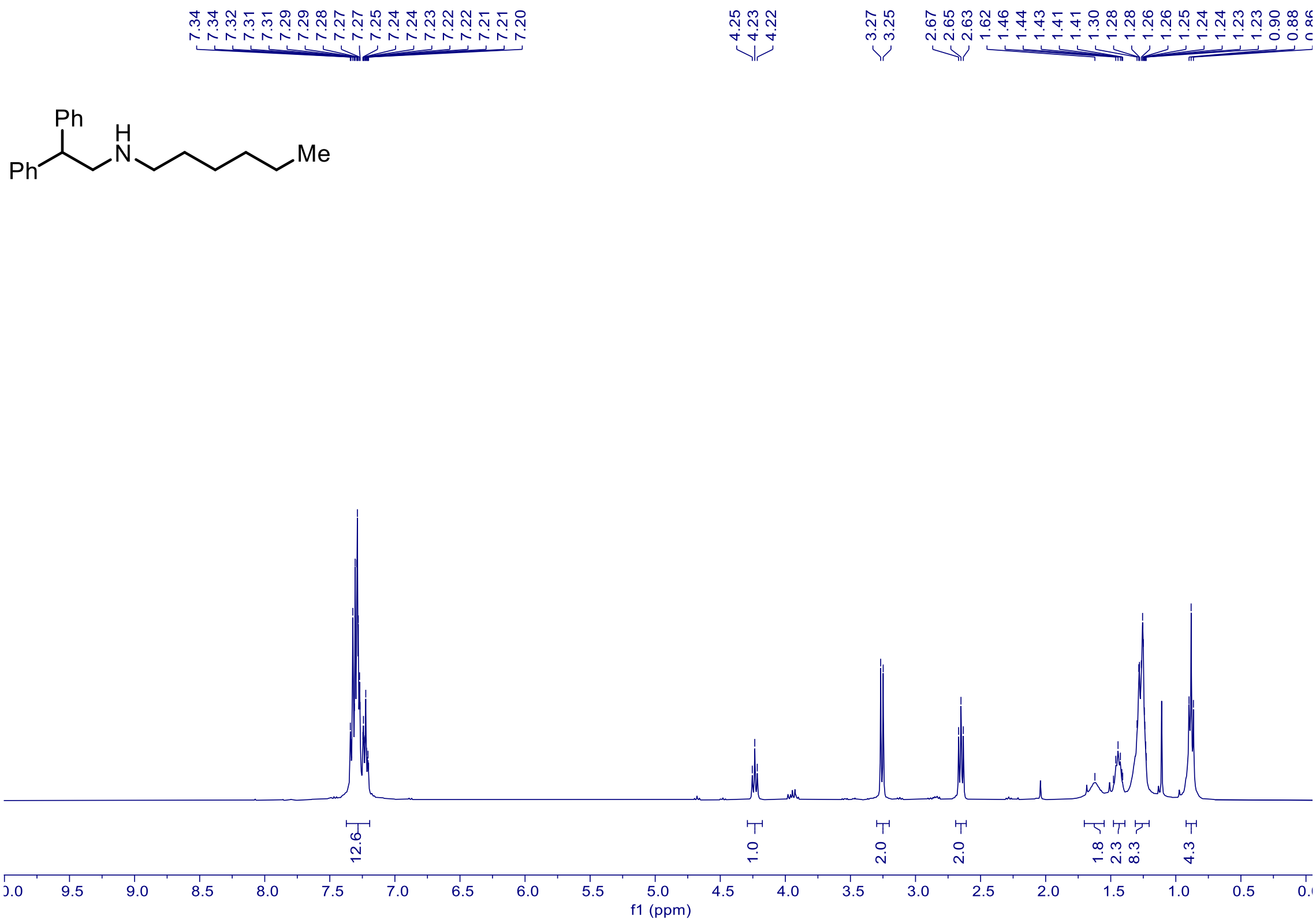

9b – ^{13}C NMR (101 MHz, $CDCl_3$)

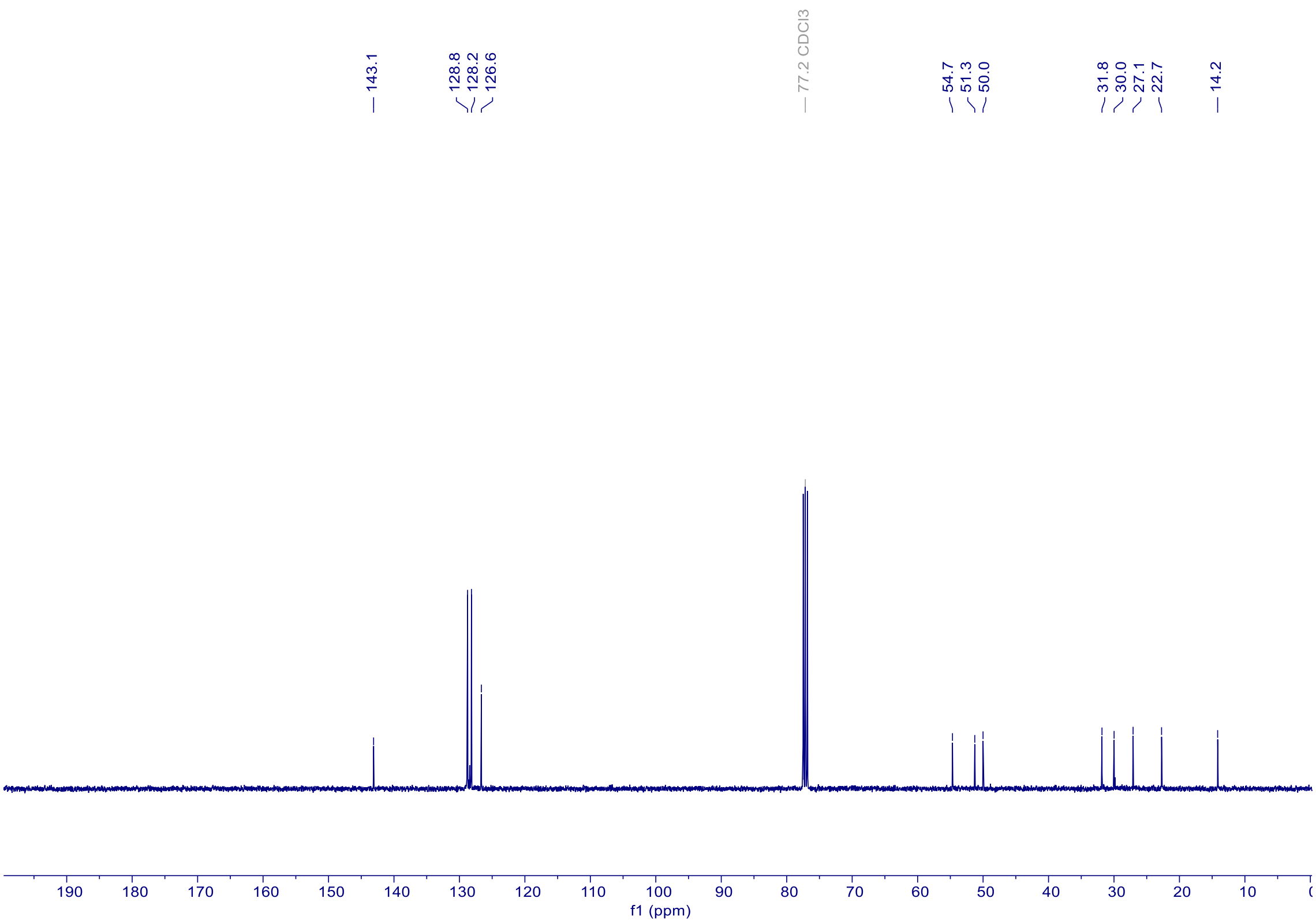

10b – ^{1}H NMR (400 MHz, $CDCl_3$)

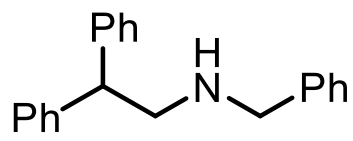

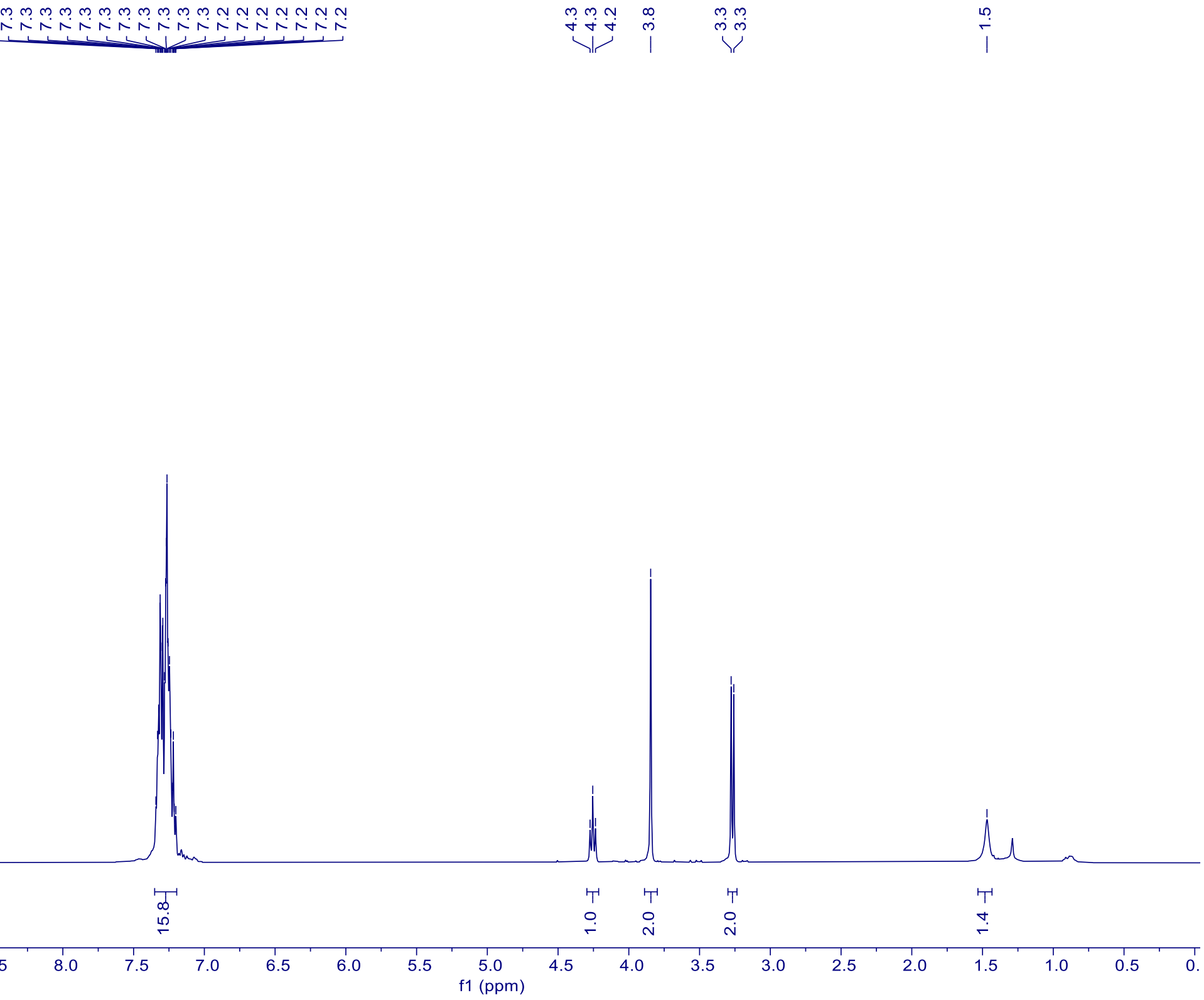

10b – ^{13}C NMR (101 MHz, $CDCl_3$)

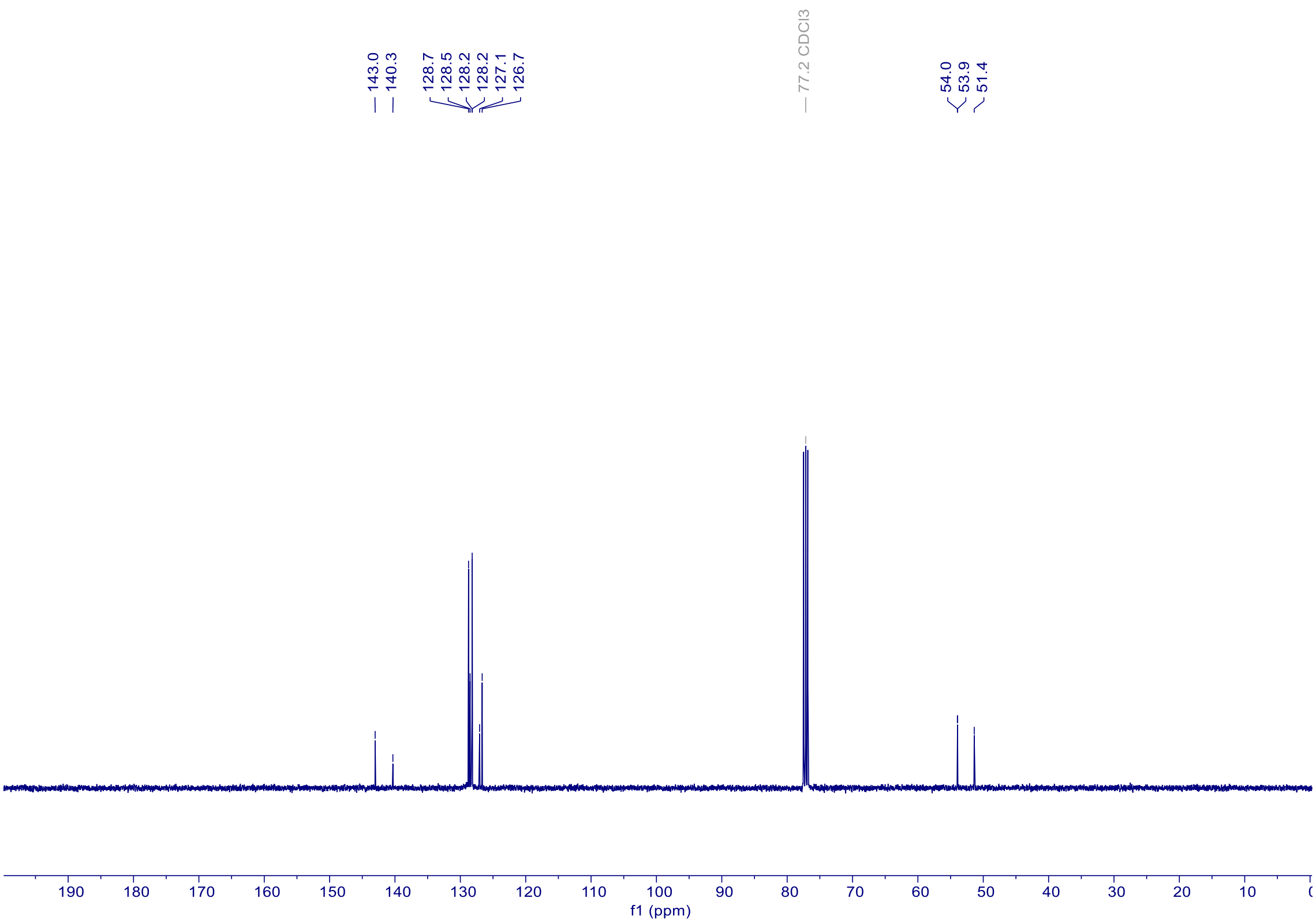

11b – ^{1}H NMR (600 MHz, $CDCl_3$)

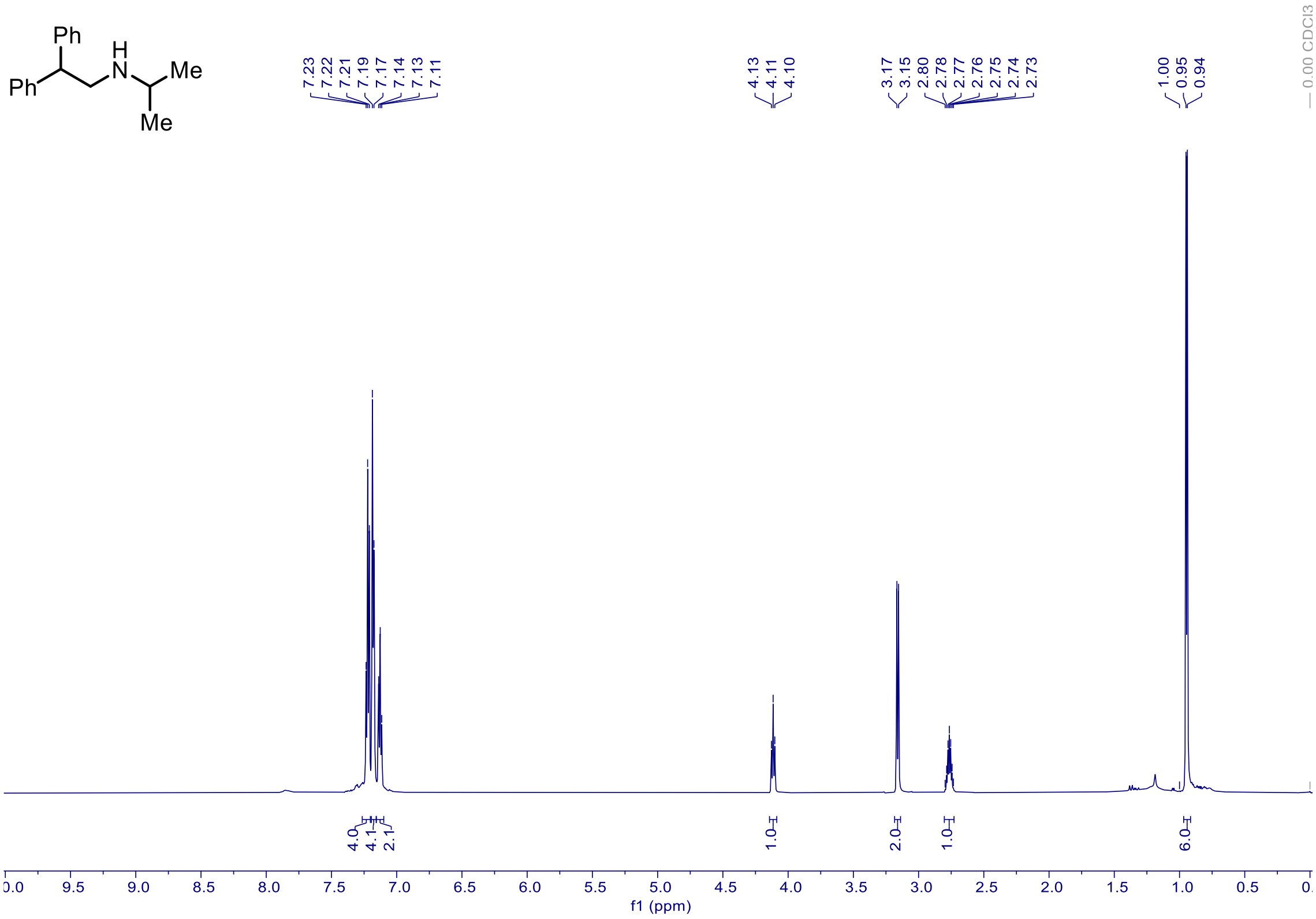

11b – ^{13}C NMR (151 MHz, $CDCl_3$)

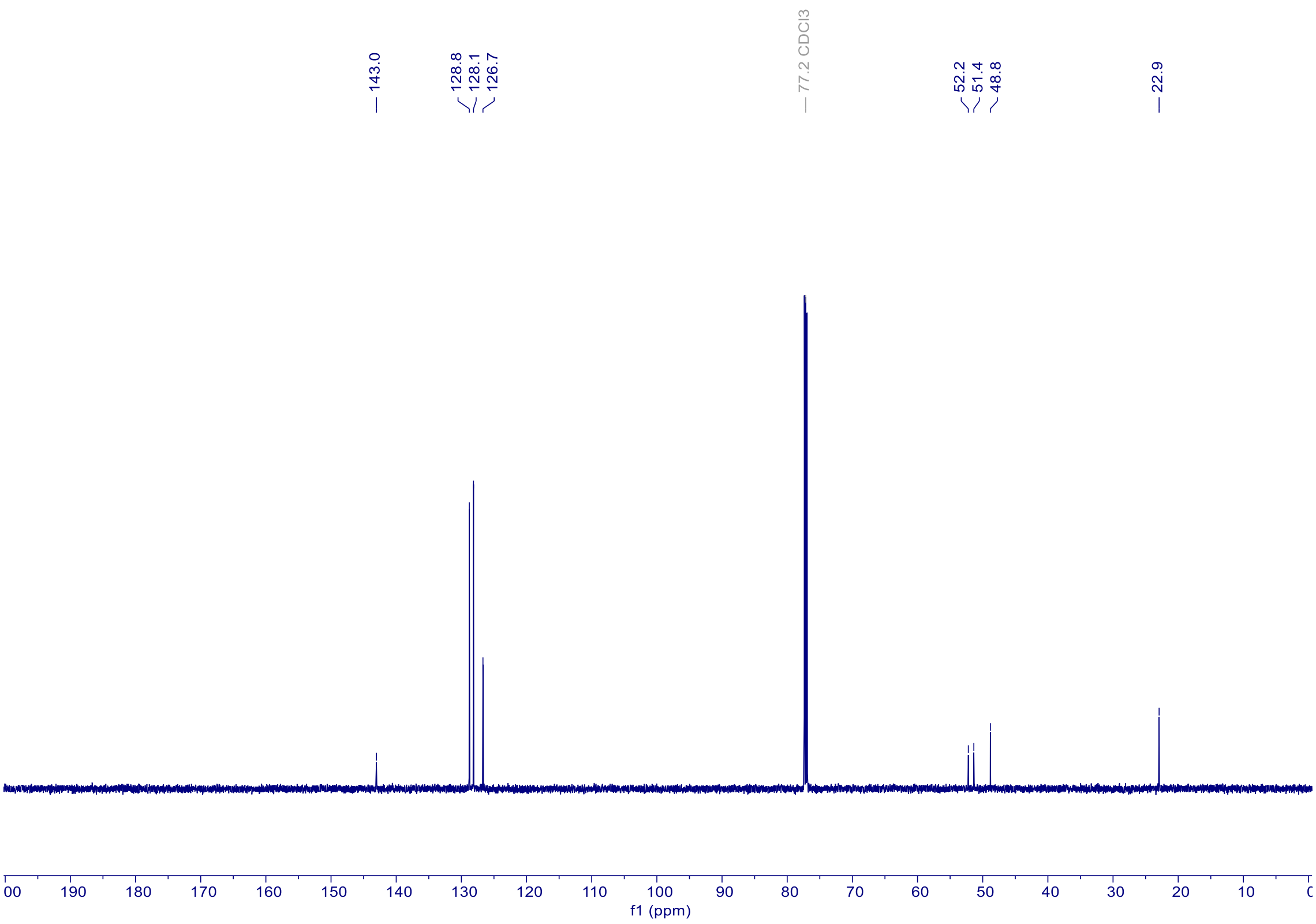

12b – ^{1}H NMR (600 MHz, $CDCl_3$)

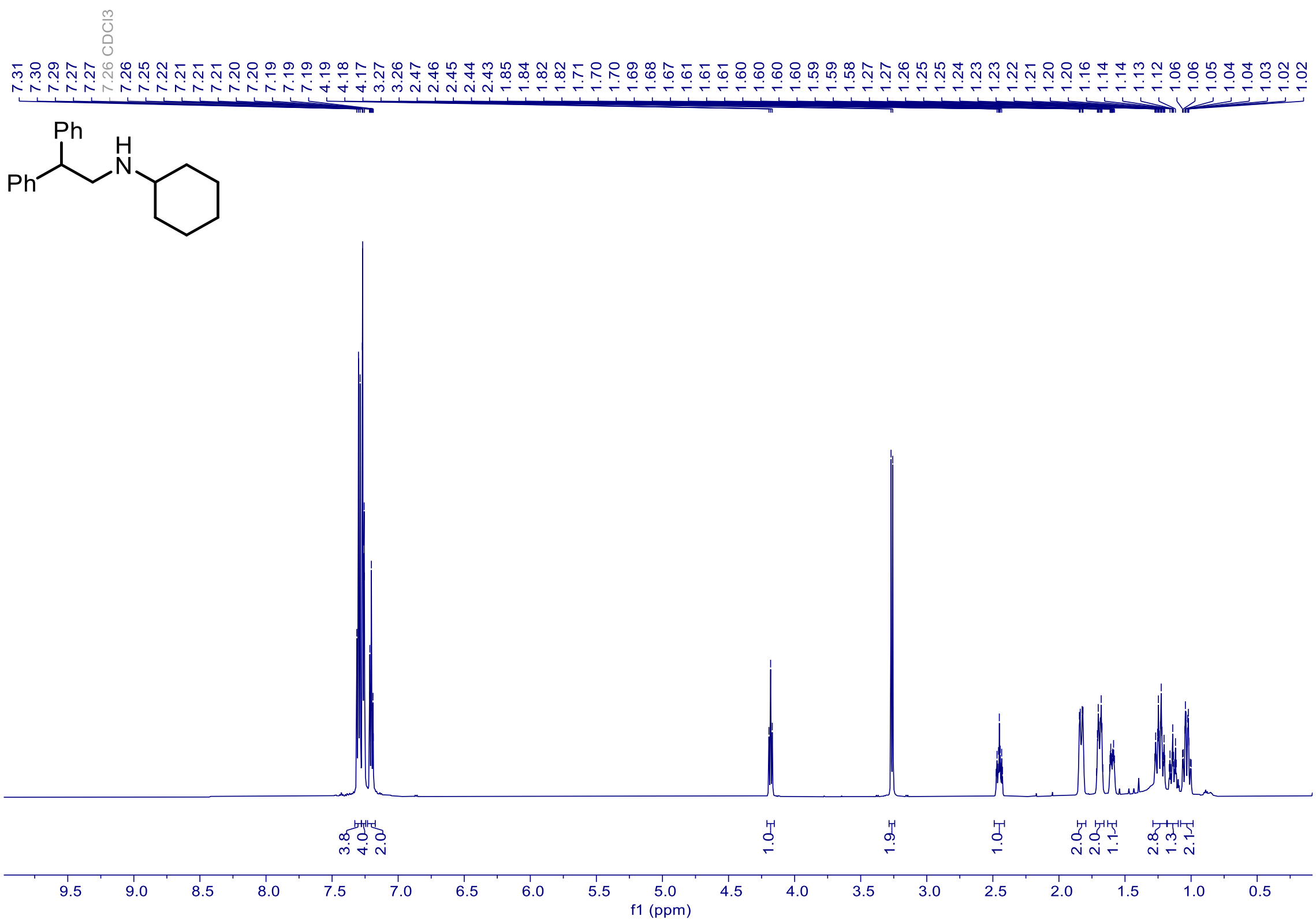

12b – ^{13}C NMR (151 MHz, $CDCl_3$)

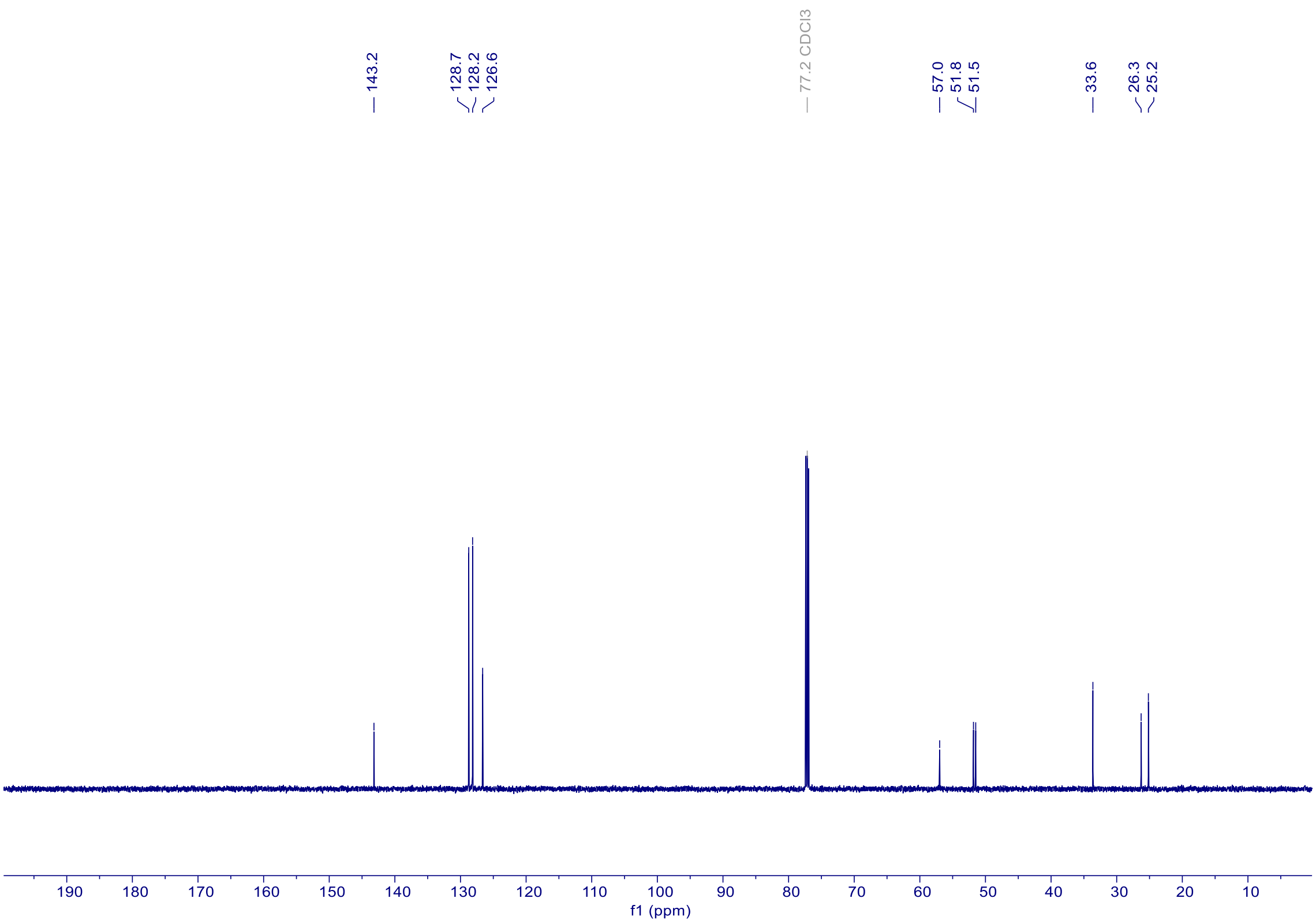

13b – ^{1}H NMR (600 MHz, $CDCl_3$)

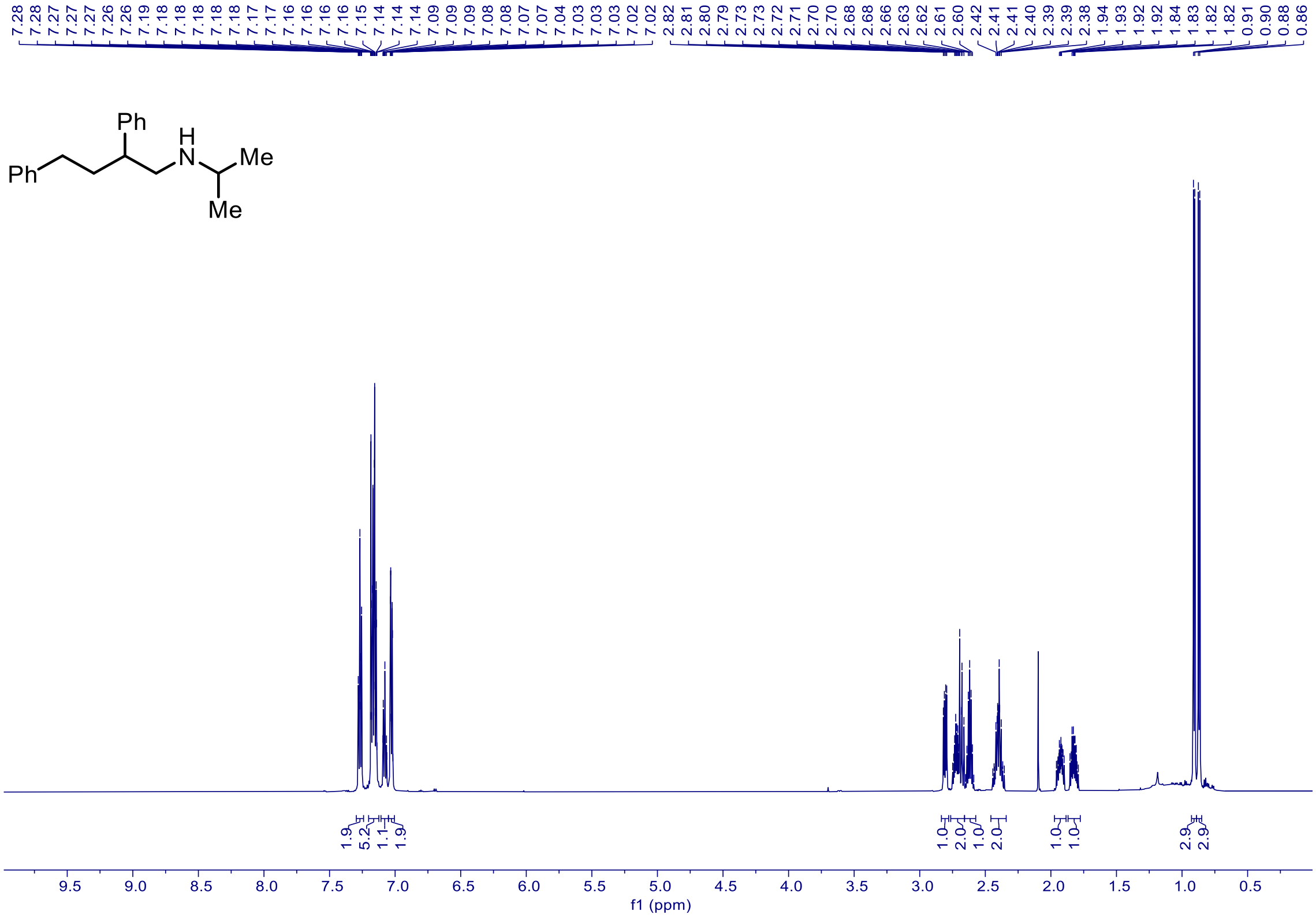

13b – ^{13}C NMR (151 MHz, $CDCl_3$)

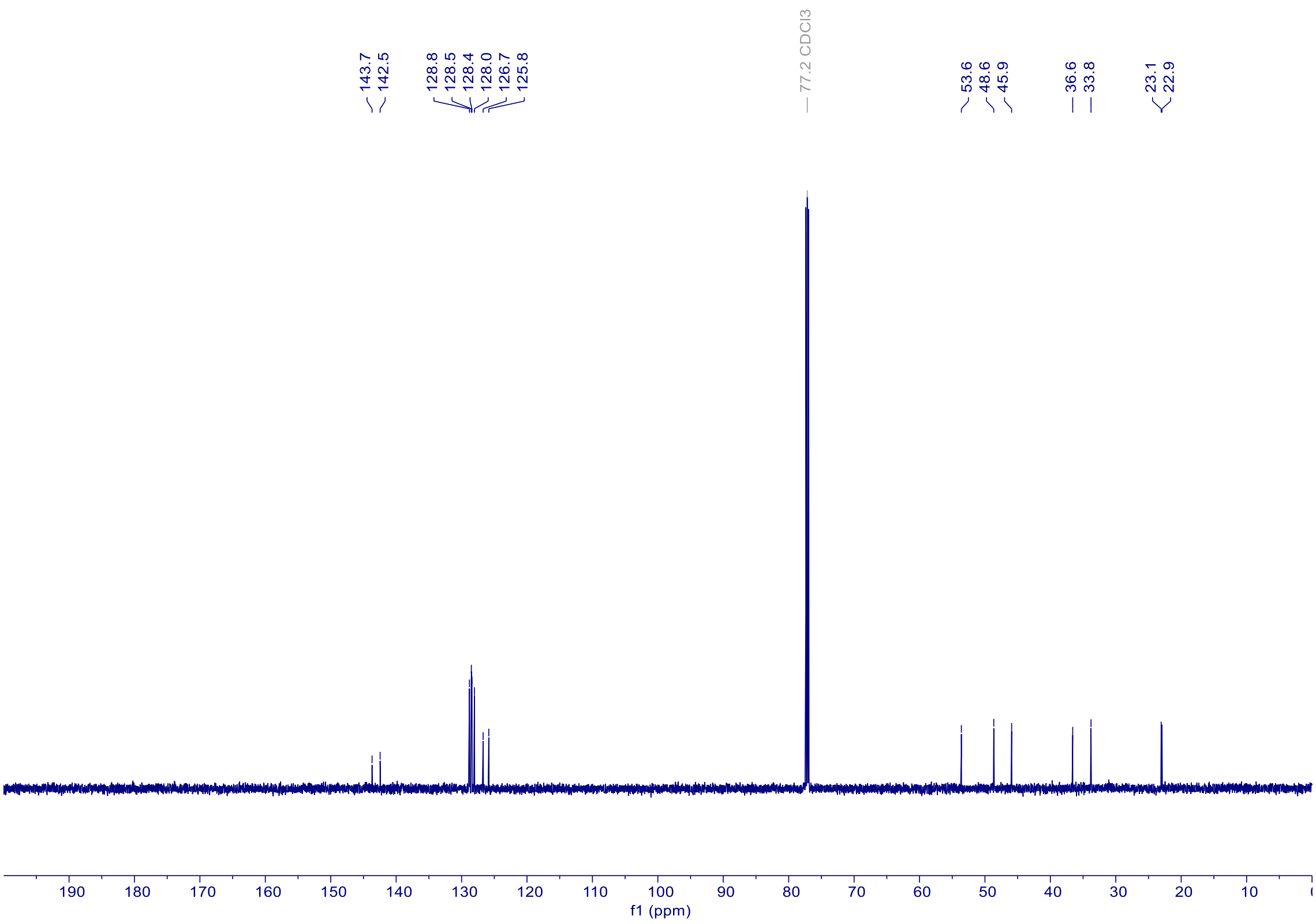

1q – ^{1}H NMR (600 MHz, $CDCl_3$)

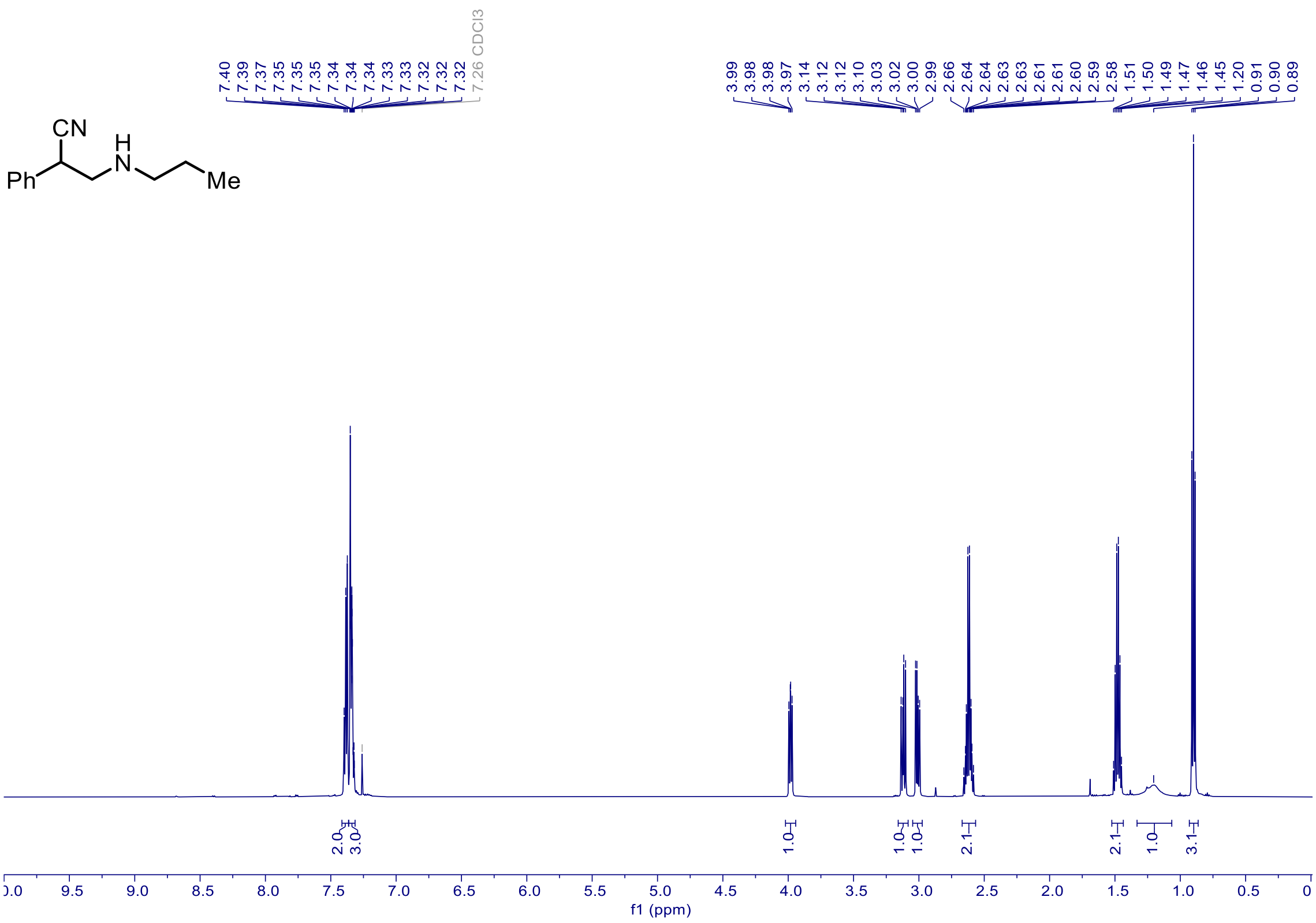

1q – ^{13}C NMR (151 MHz, $CDCl_3$)

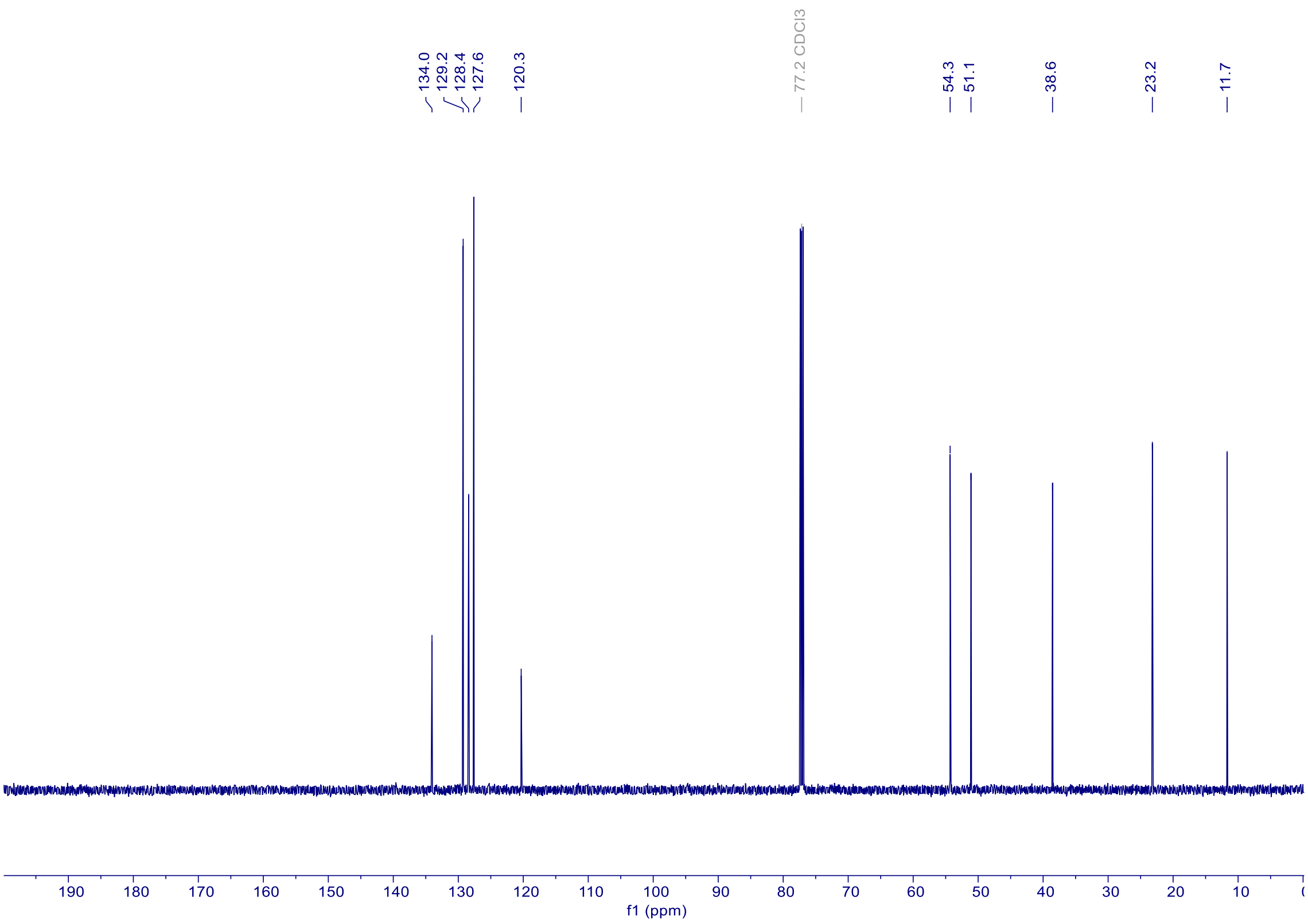

14b – ^{1}H NMR (600 MHz, $CDCl_3$)

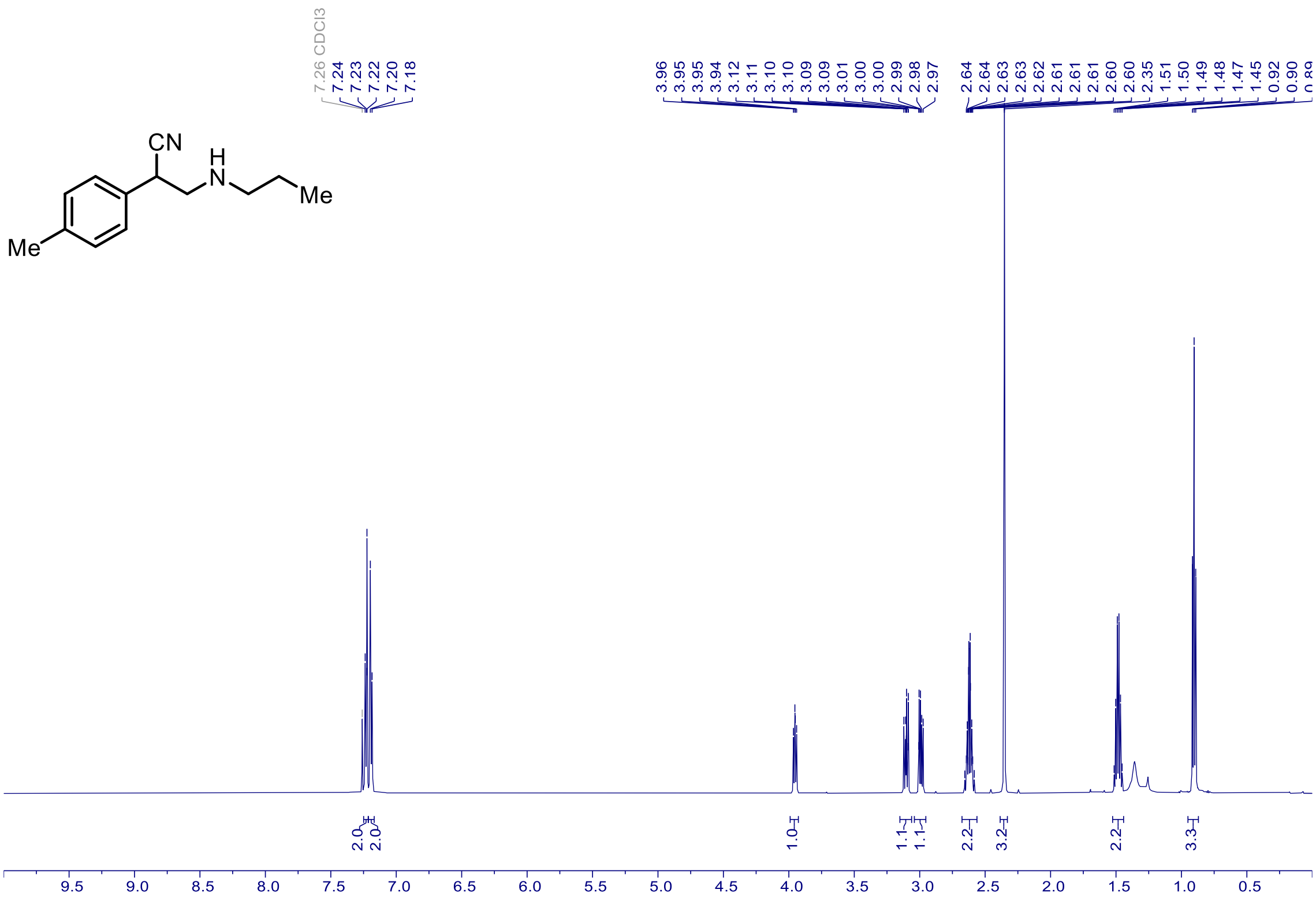

14b – ^{13}C NMR (151 MHz, $CDCl_3$)

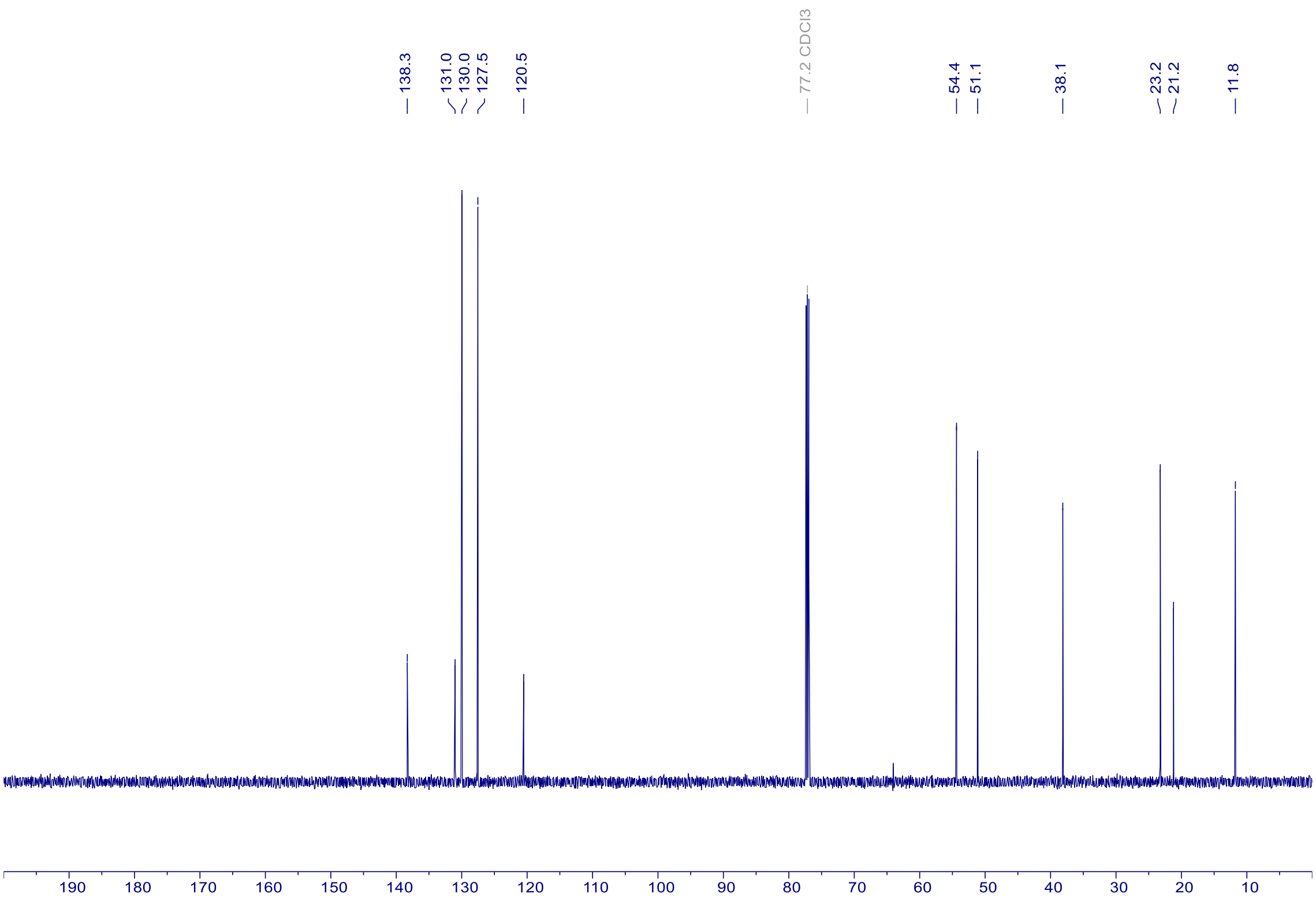

3c – ^{1}H NMR (400 MHz, $CDCl_3$)

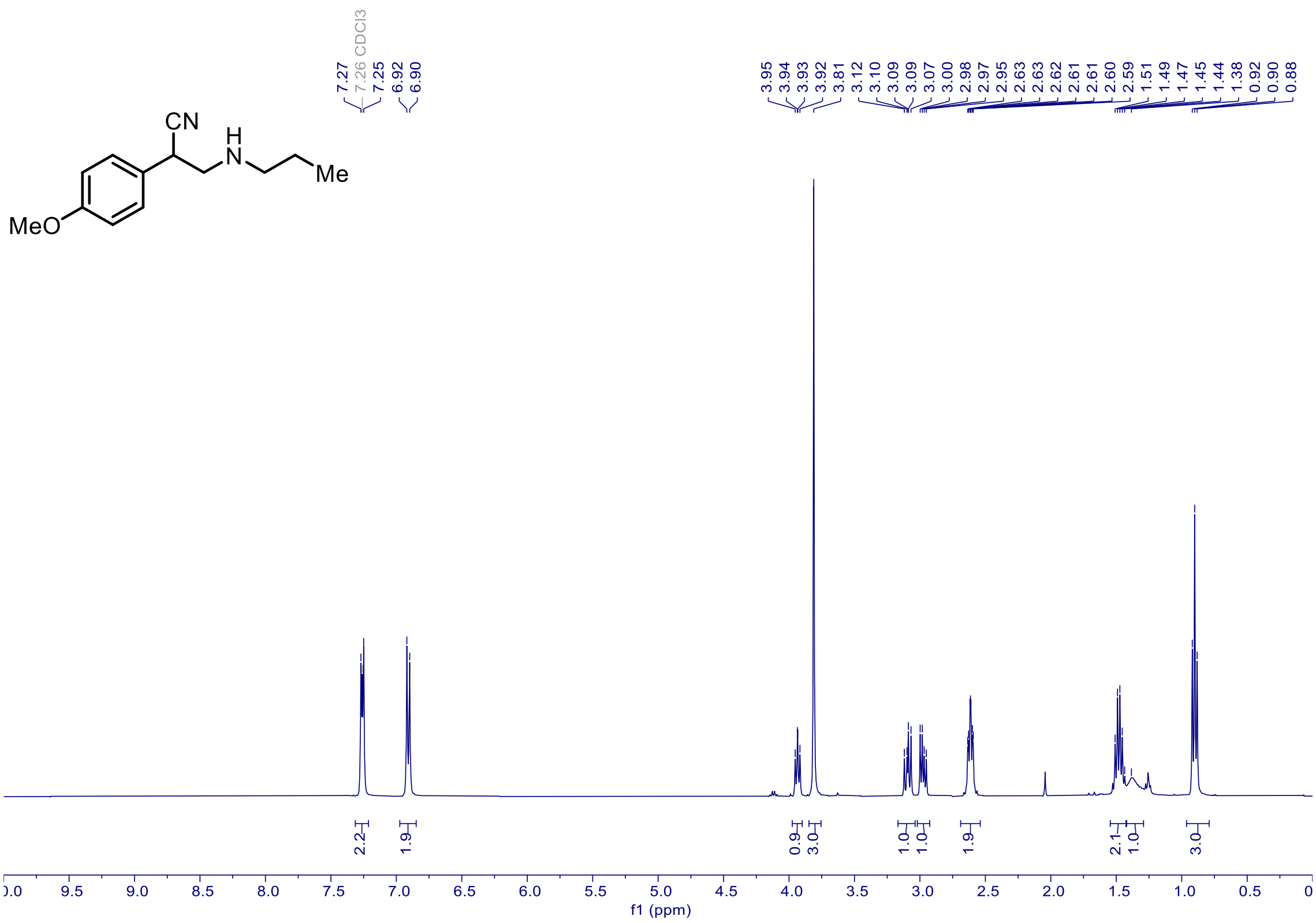

3c – ^{13}C NMR (101 MHz, $CDCl_3$)

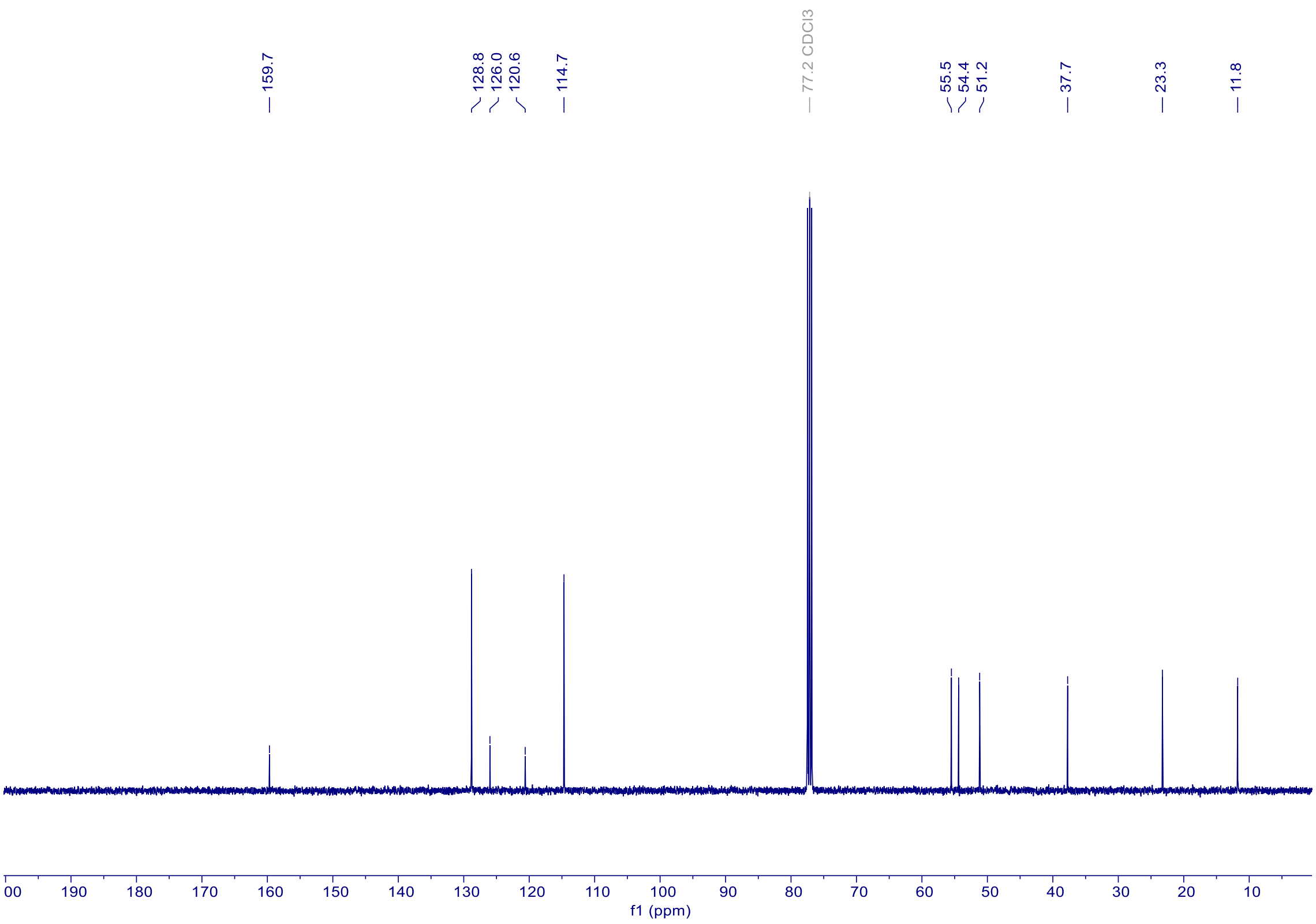

15b – ^{1}H NMR (600 MHz, $CDCl_3$)

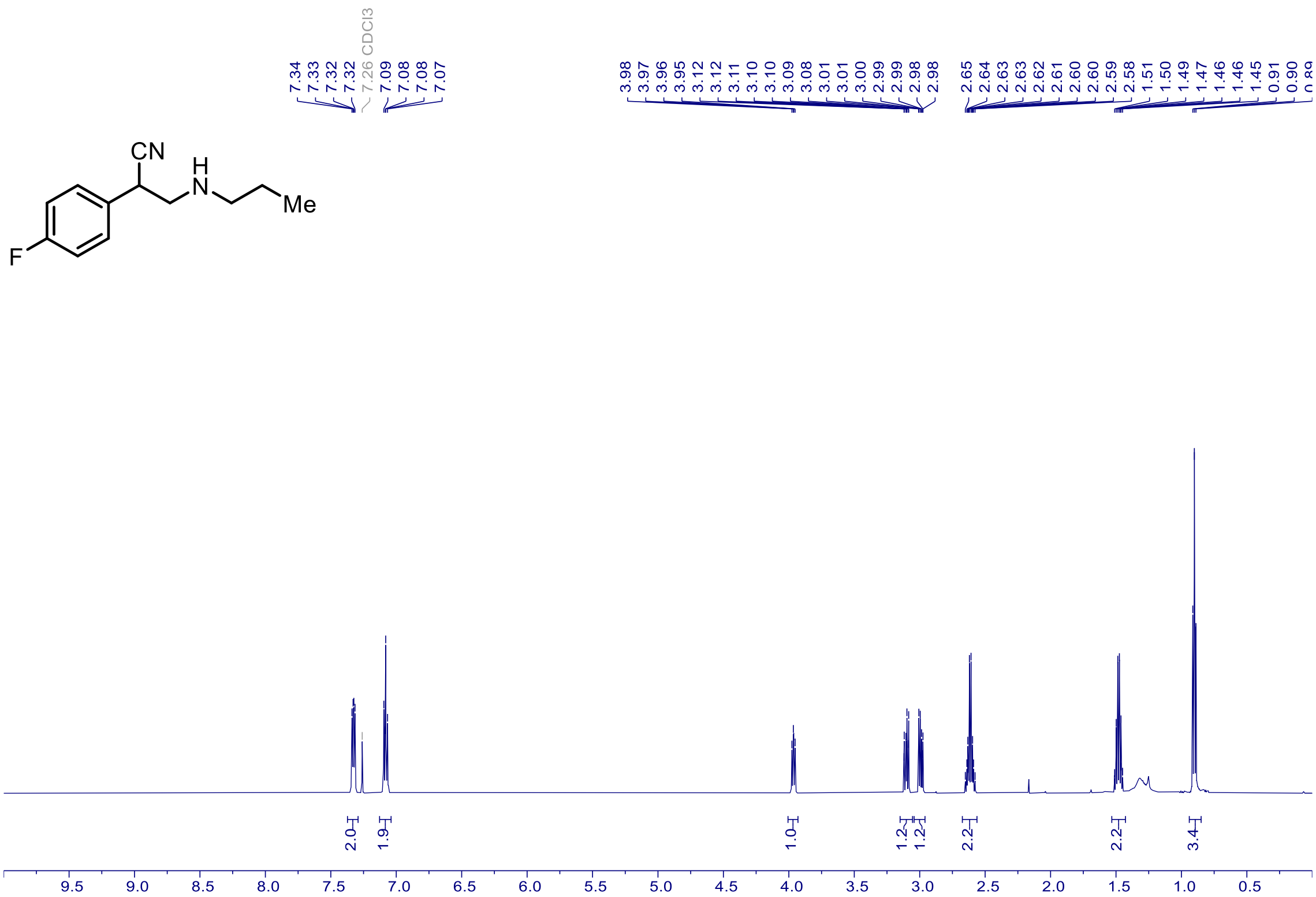

15b – ^{13}C NMR (151 MHz, $CDCl_3$)

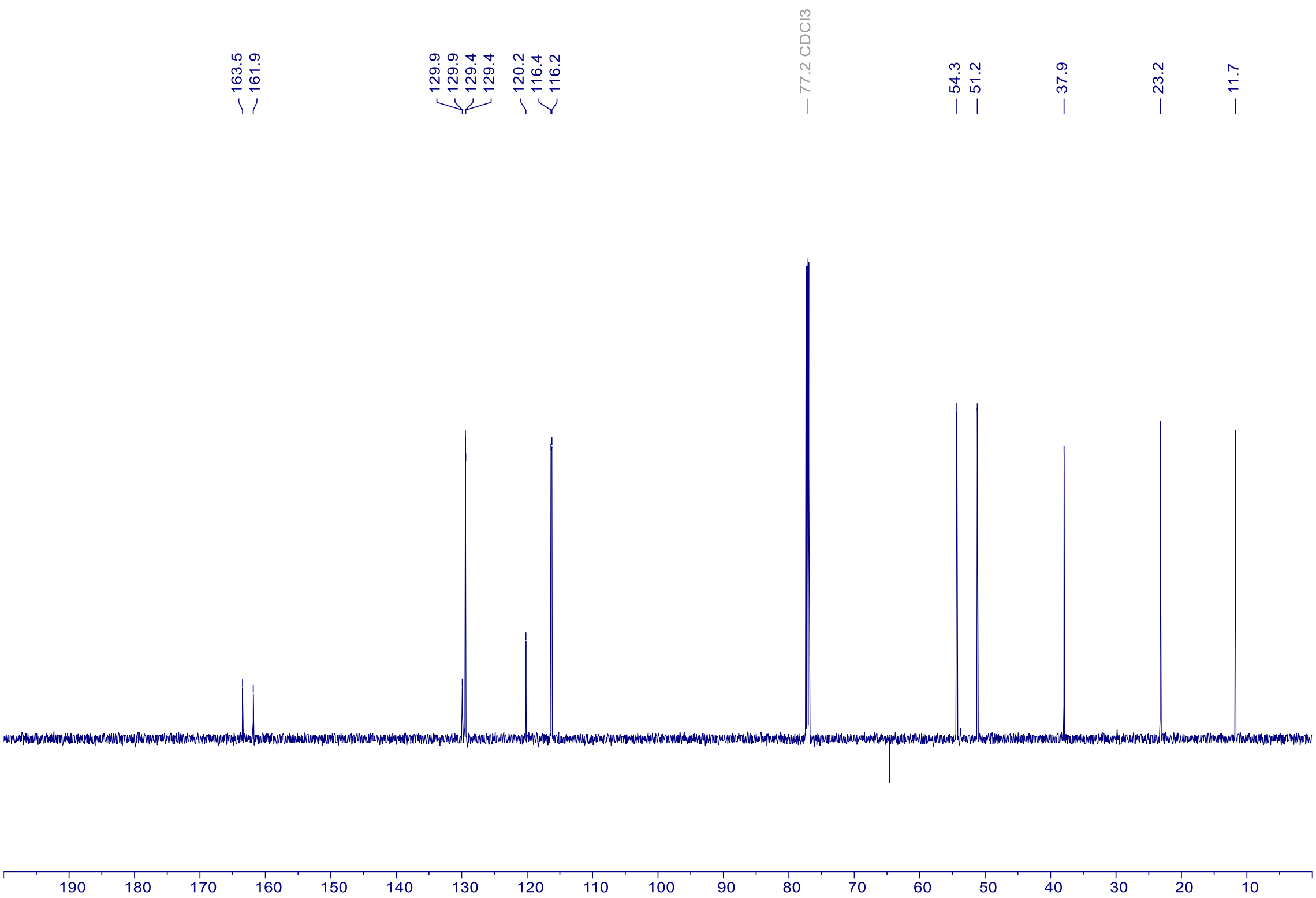

15b – ^{19}F NMR (564 MHz, $CDCl_3$)

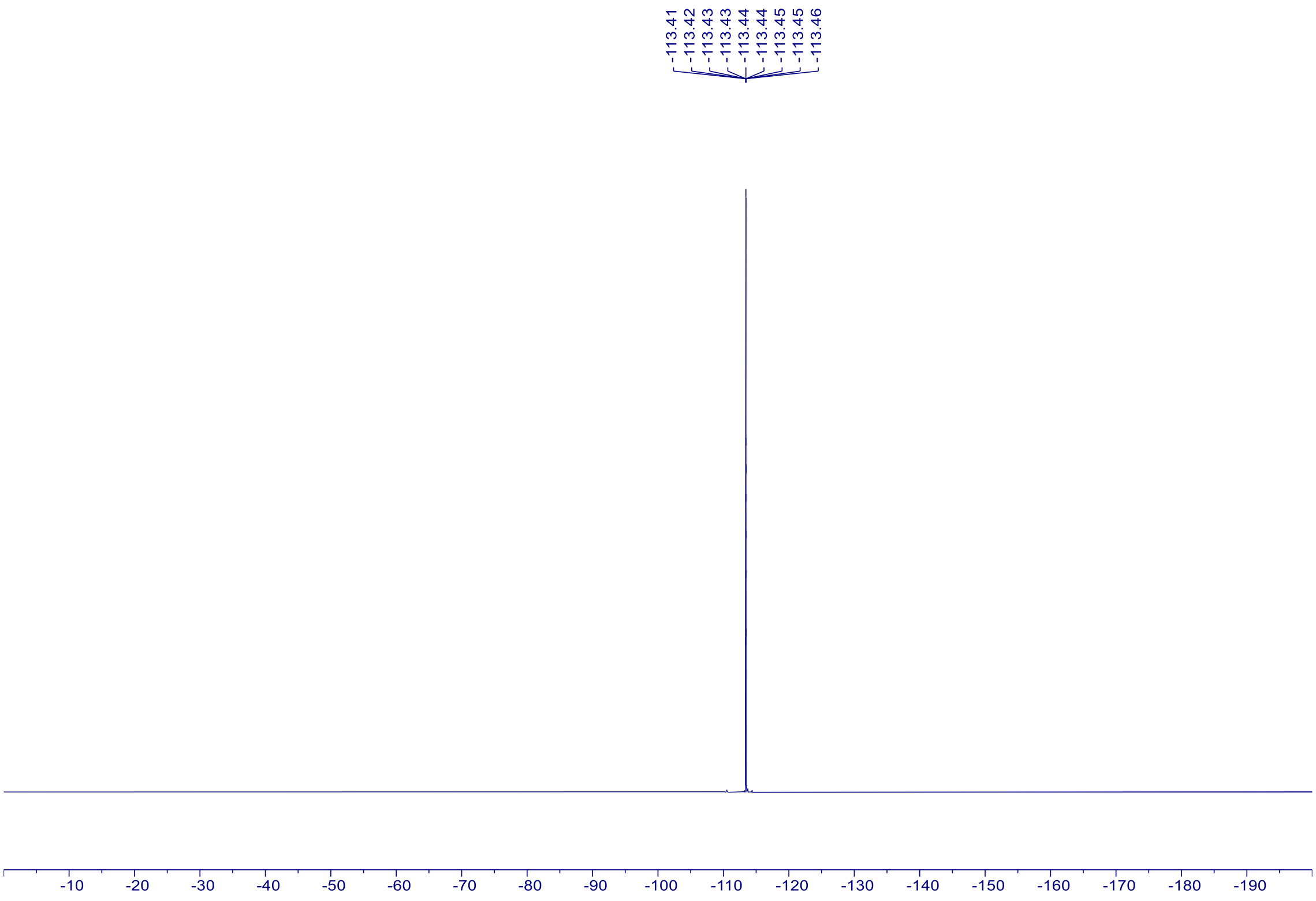

5c – ^{1}H NMR (600 MHz, $CDCl_3$)

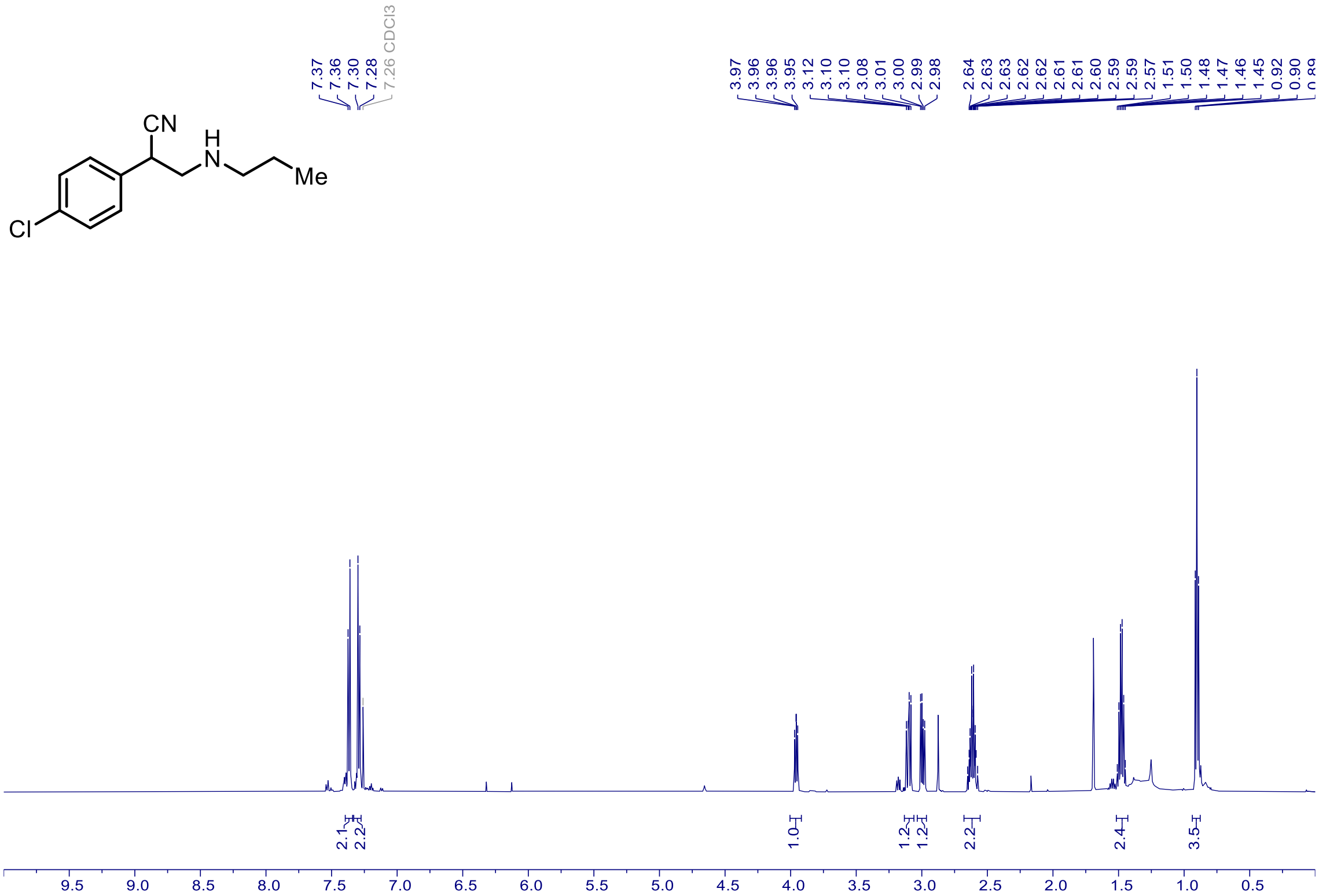

5c – ^{13}C NMR (151 MHz, $CDCl_3$)

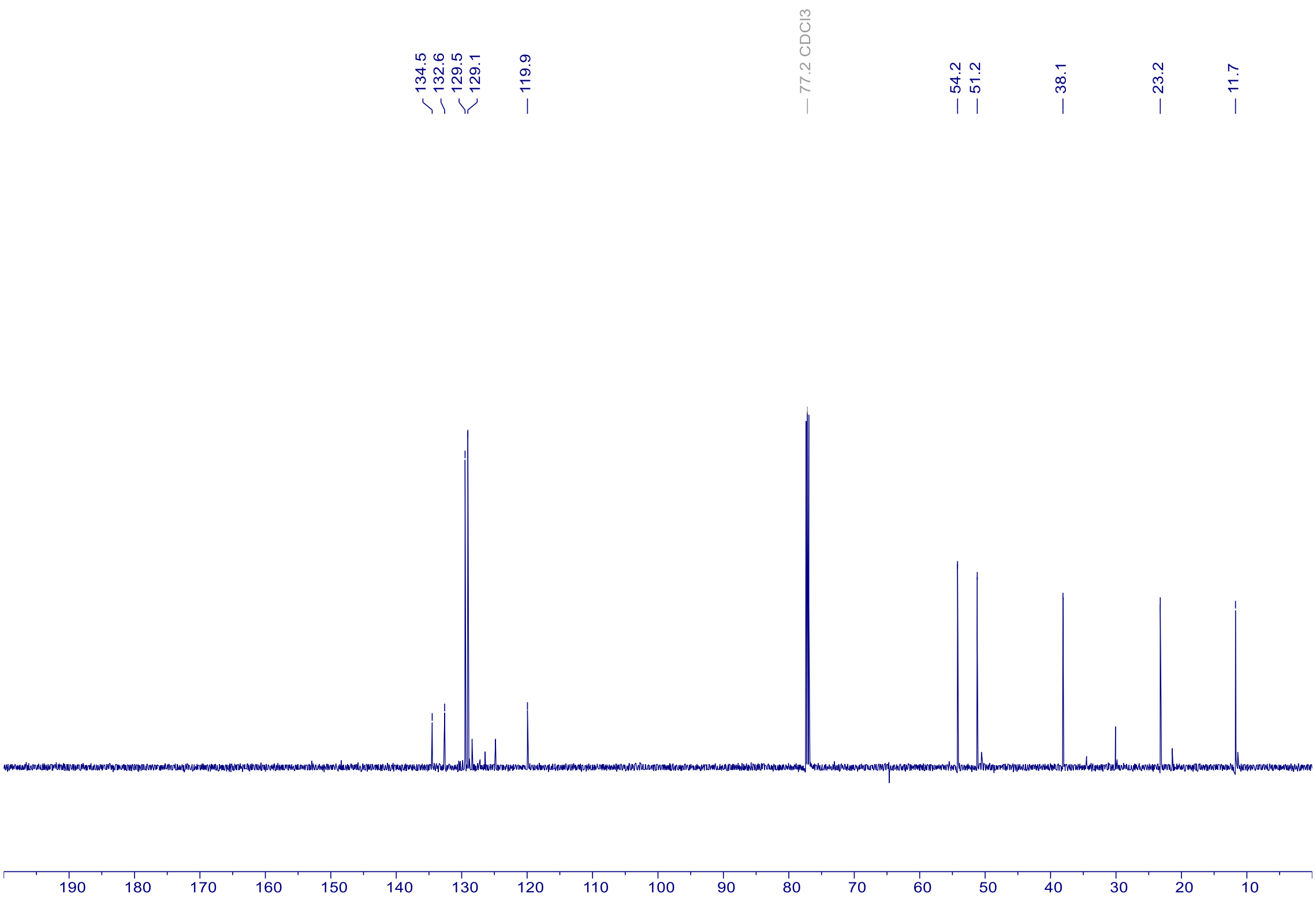

16b – ^{1}H NMR (400 MHz, $CDCl_3$)

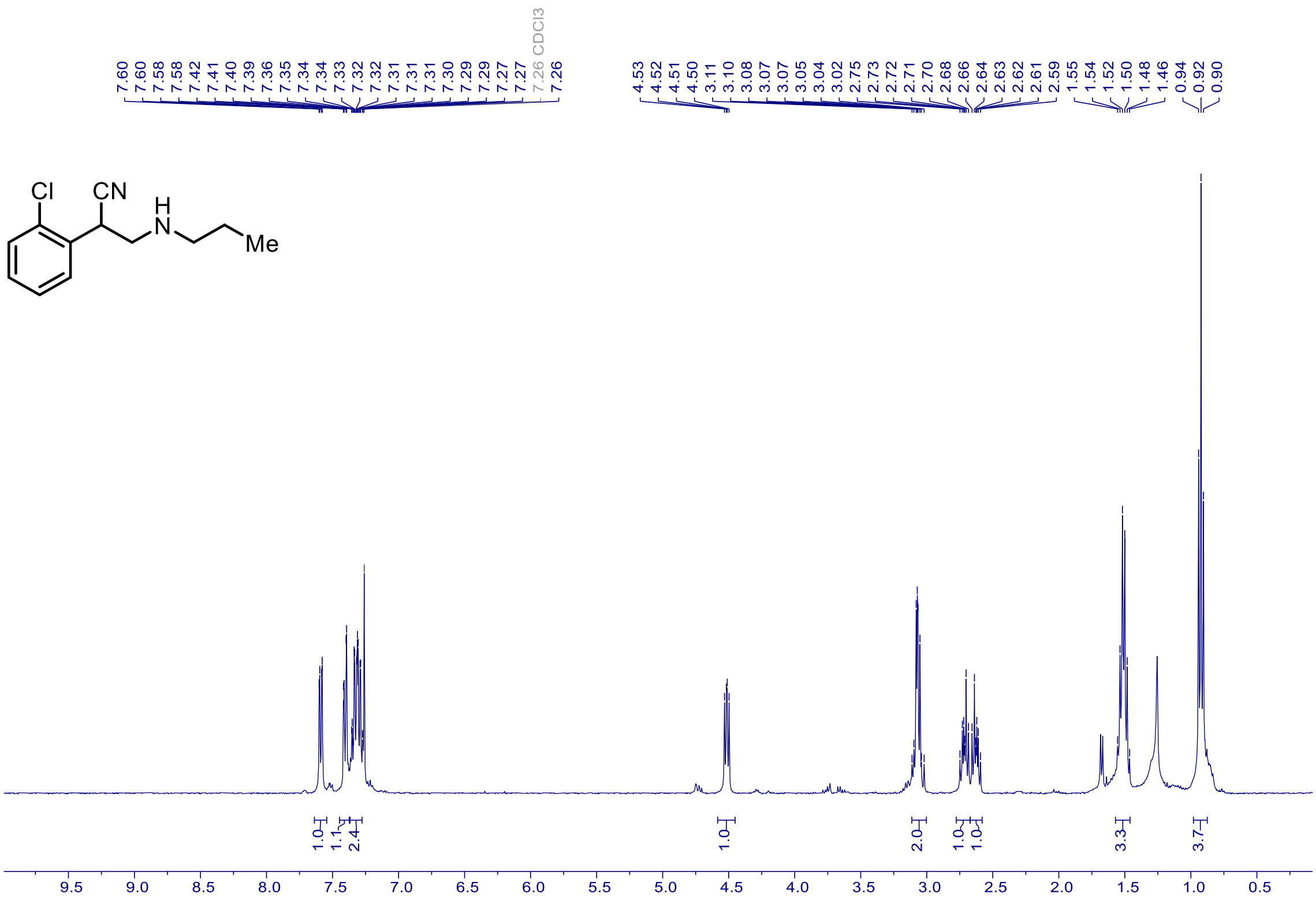

16b – ^{13}C NMR (101 MHz, $CDCl_3$)

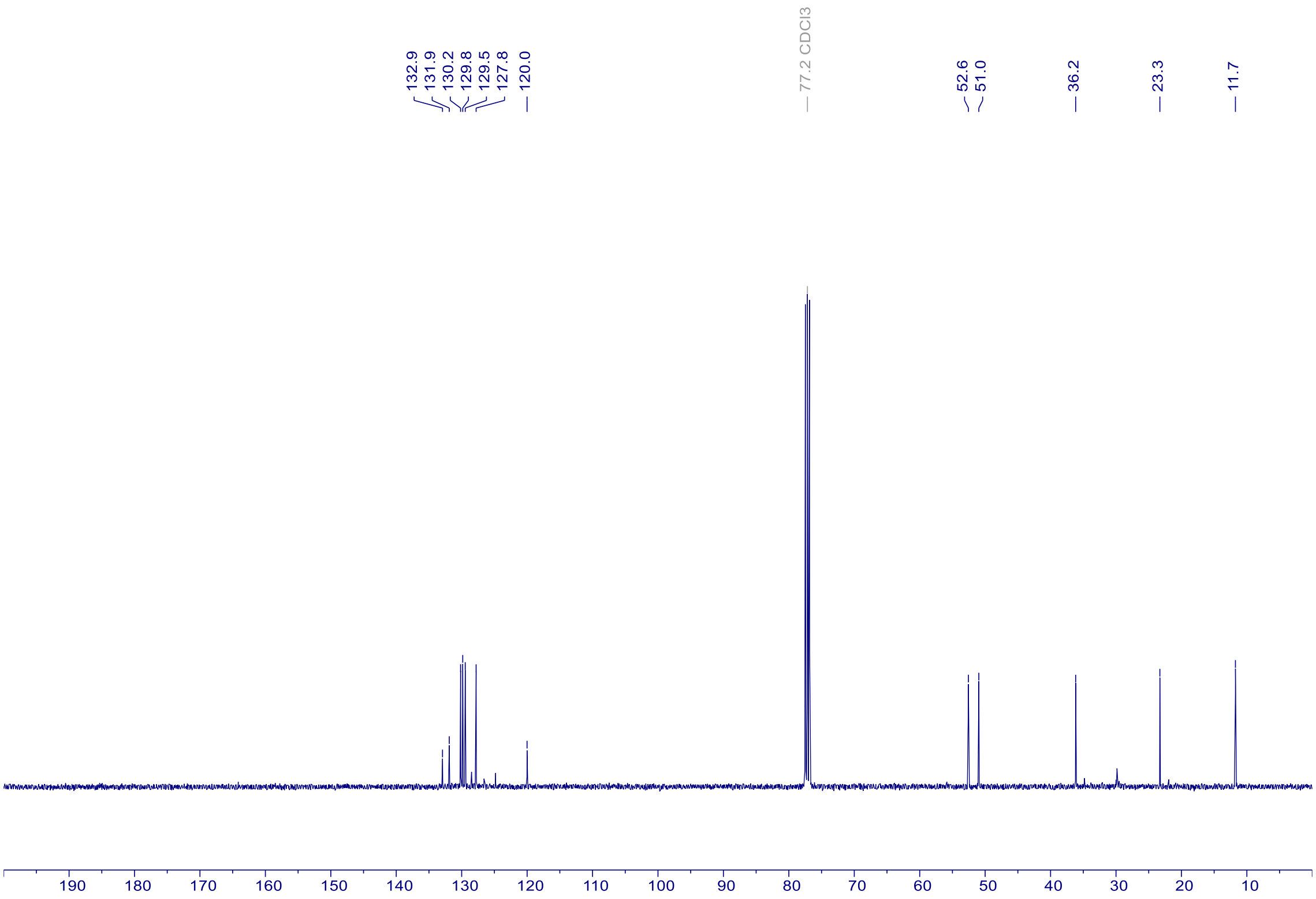

7c – ^{1}H NMR (600 MHz, $CDCl_3$)

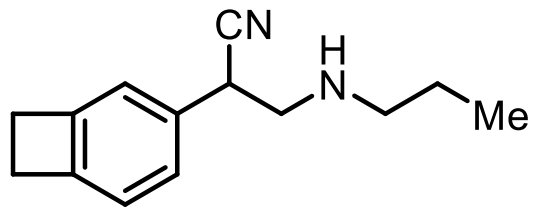

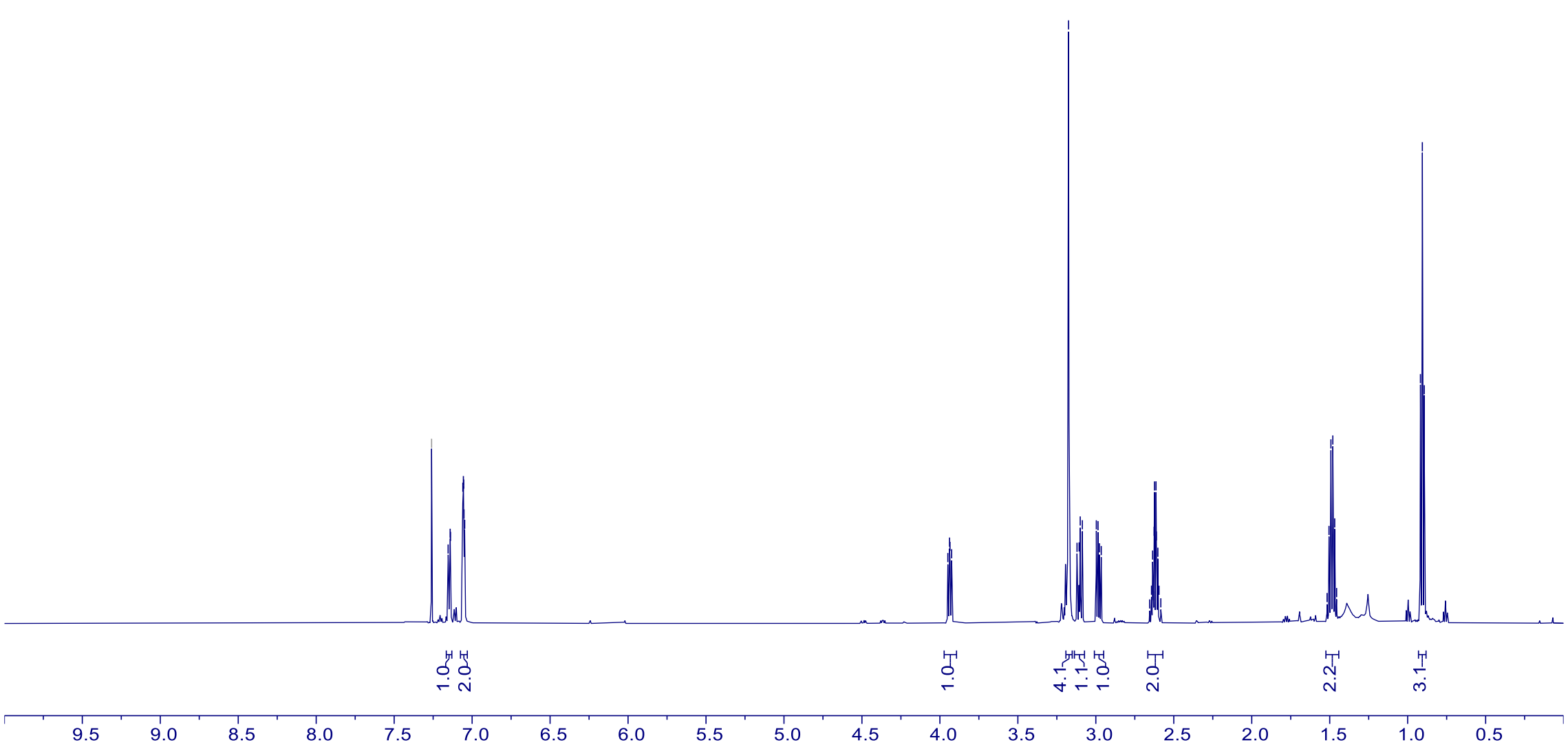

7c – ^{13}C NMR (151 MHz, $CDCl_3$)

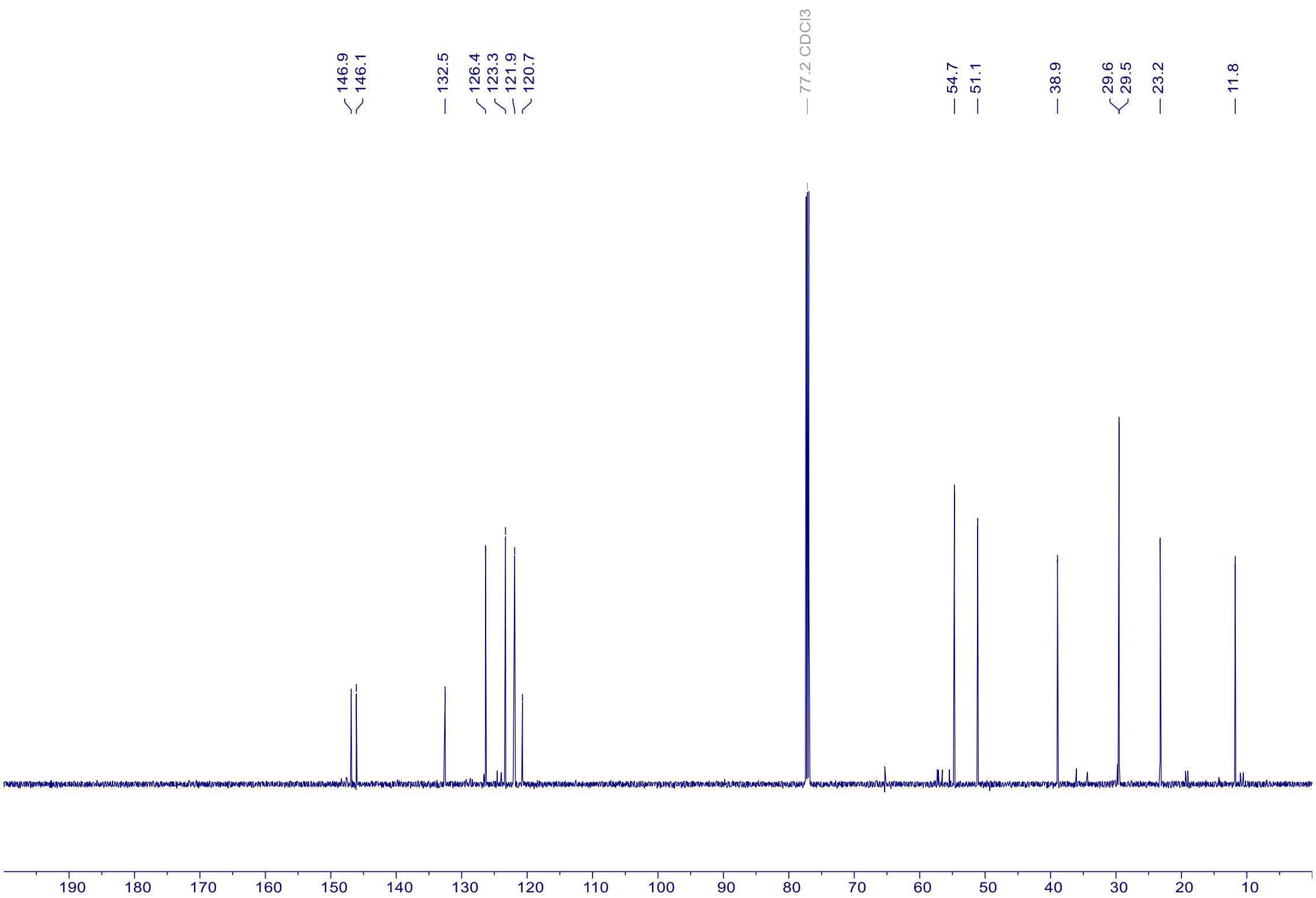

8c – ^{1}H NMR (600 MHz, CDCl$_3$)

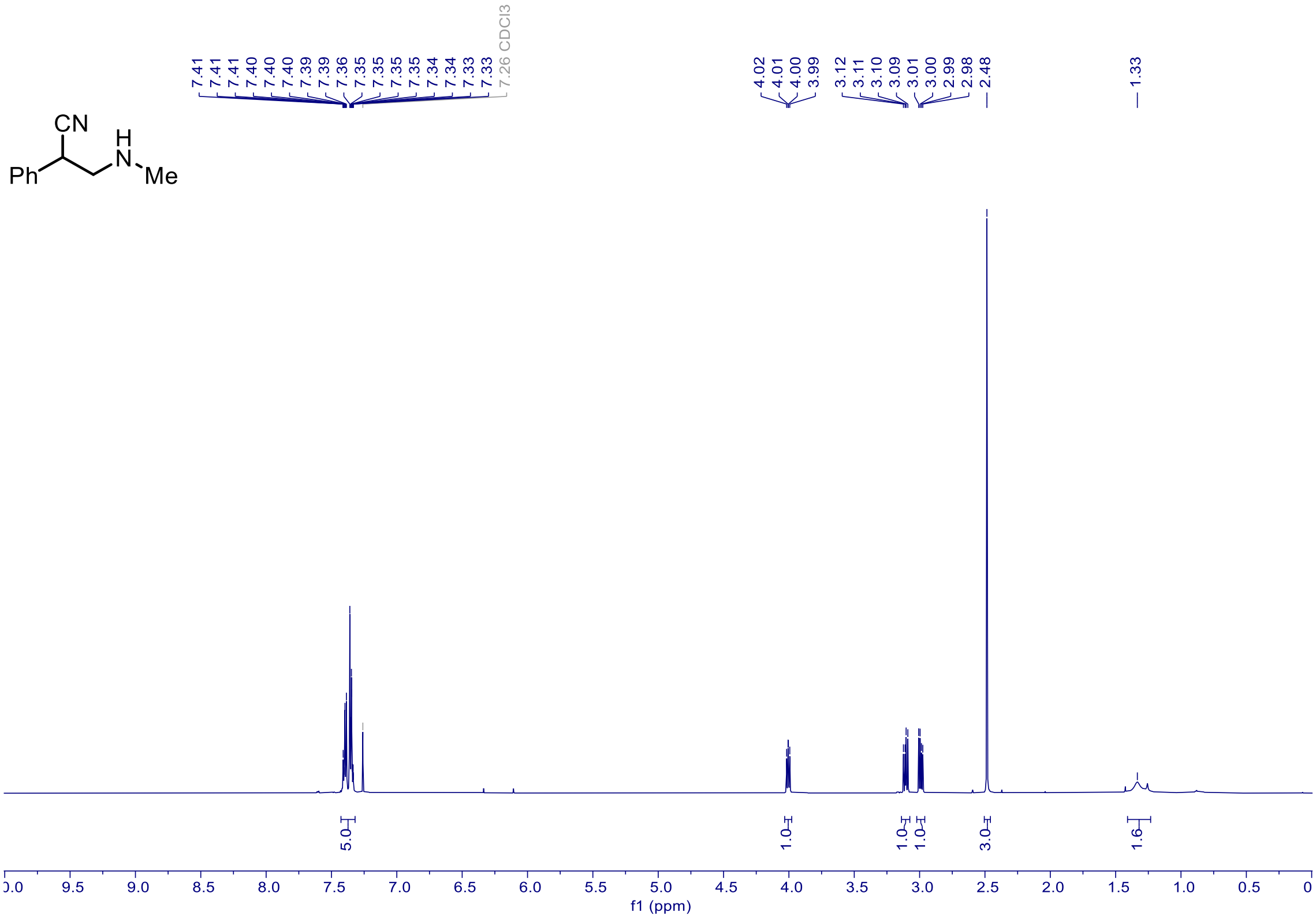

8c – ^{13}C NMR (151 MHz, CDCl$_3$)

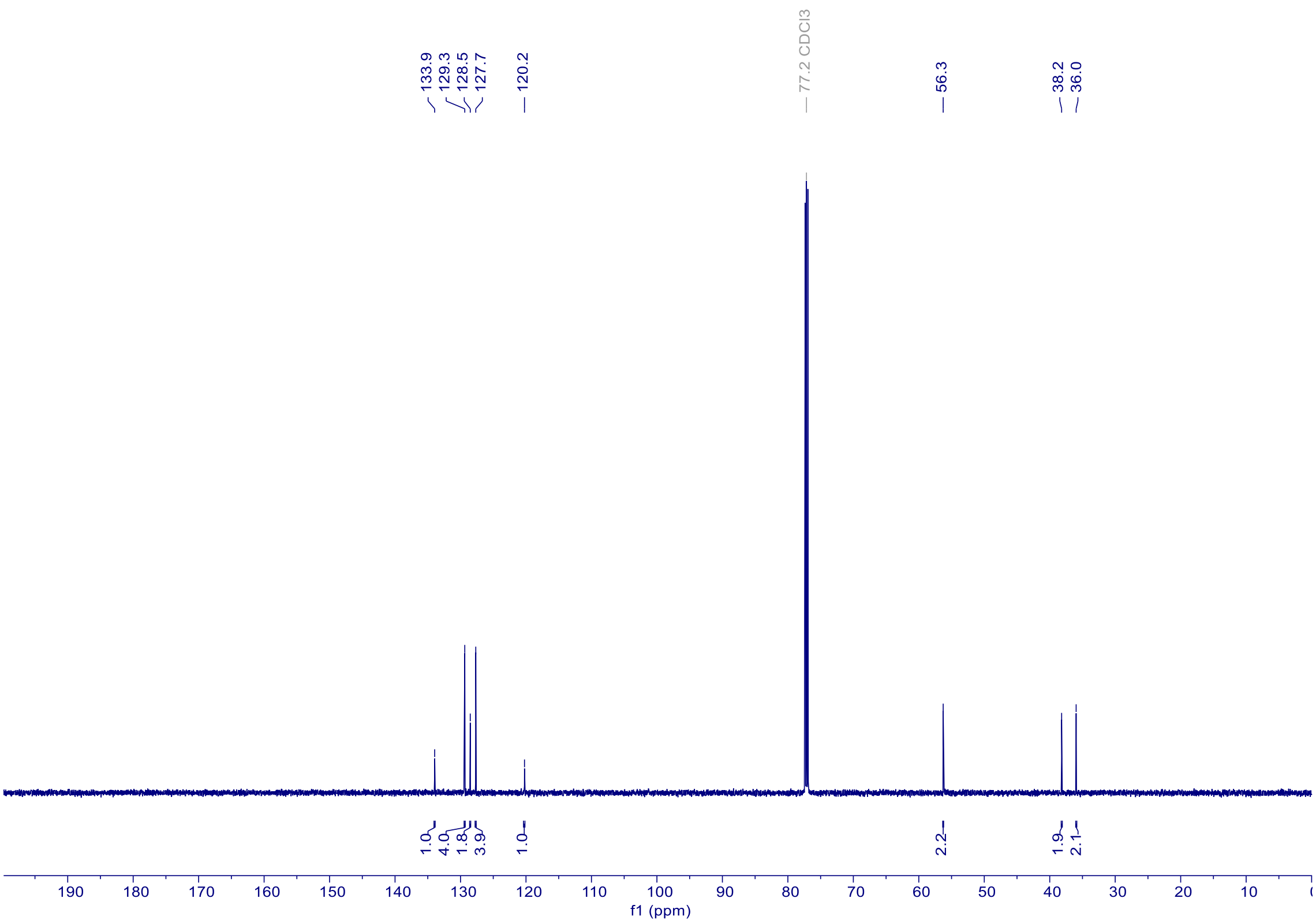

9c – ^{1}H NMR (600 MHz, $CDCl_3$)

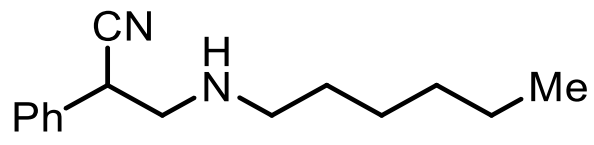

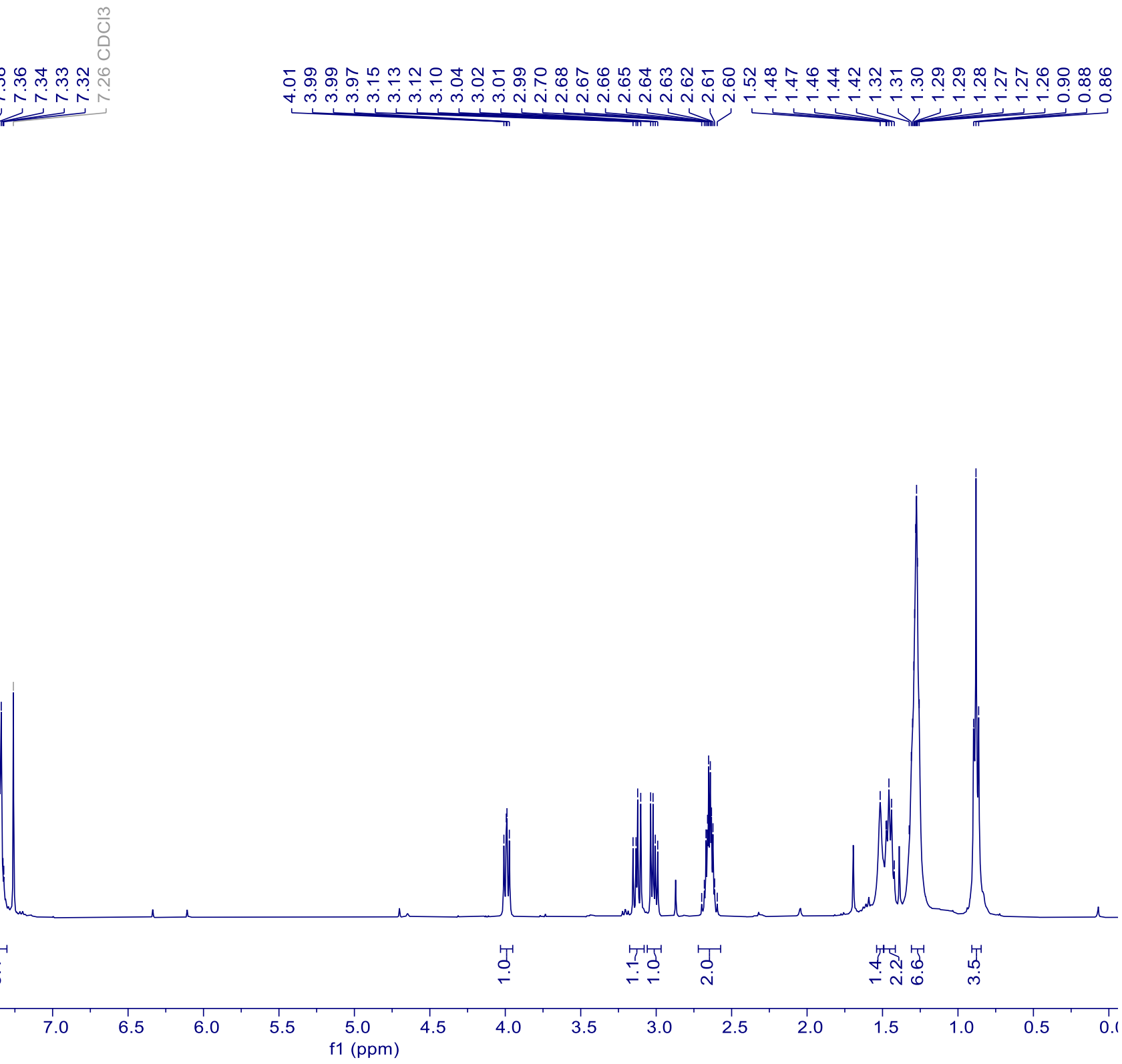

9c – ^{13}C NMR (151 MHz, $CDCl_3$)

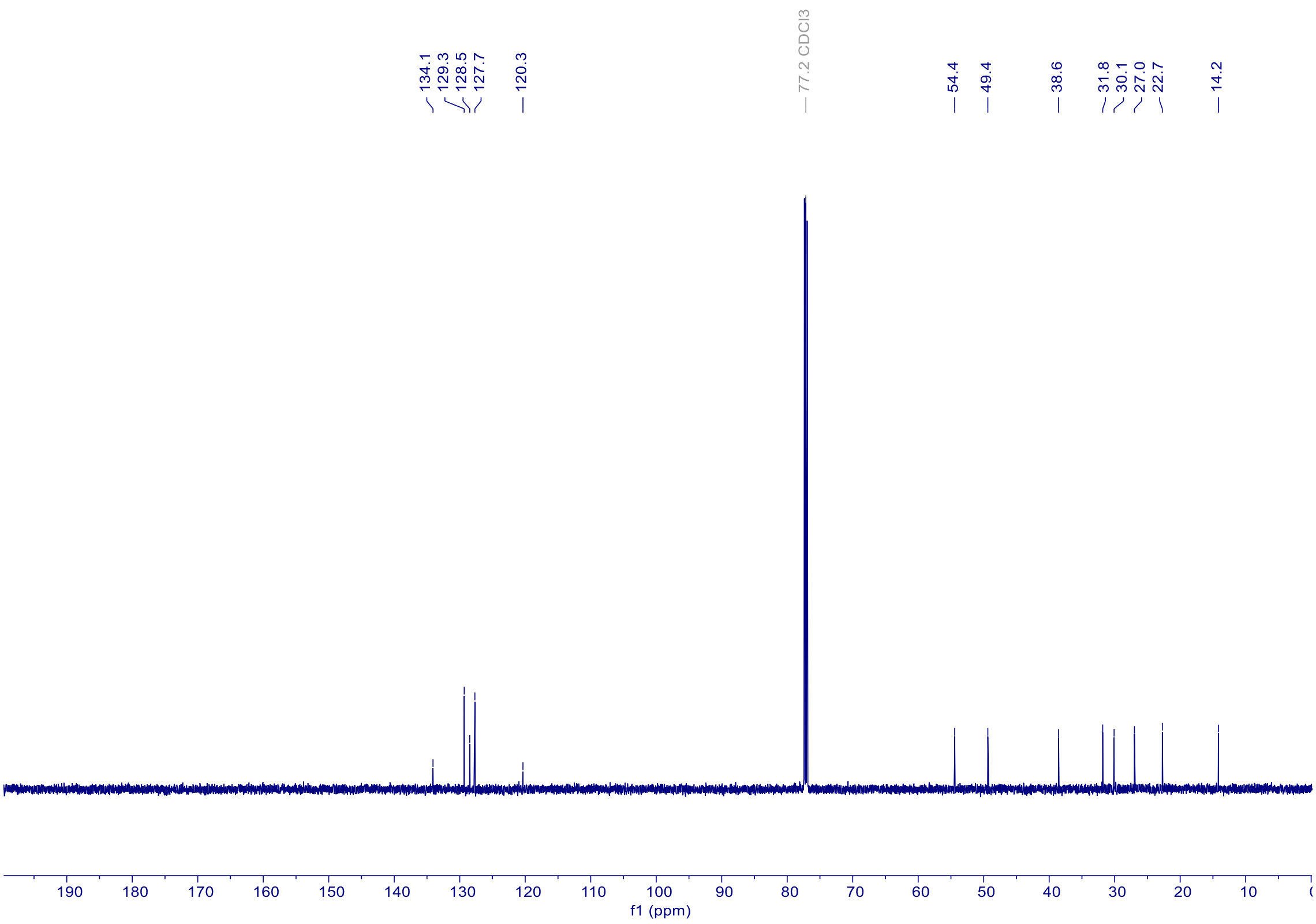

10c – ^{1}H NMR (400 MHz, $CDCl_3$)

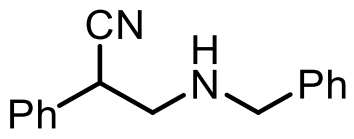

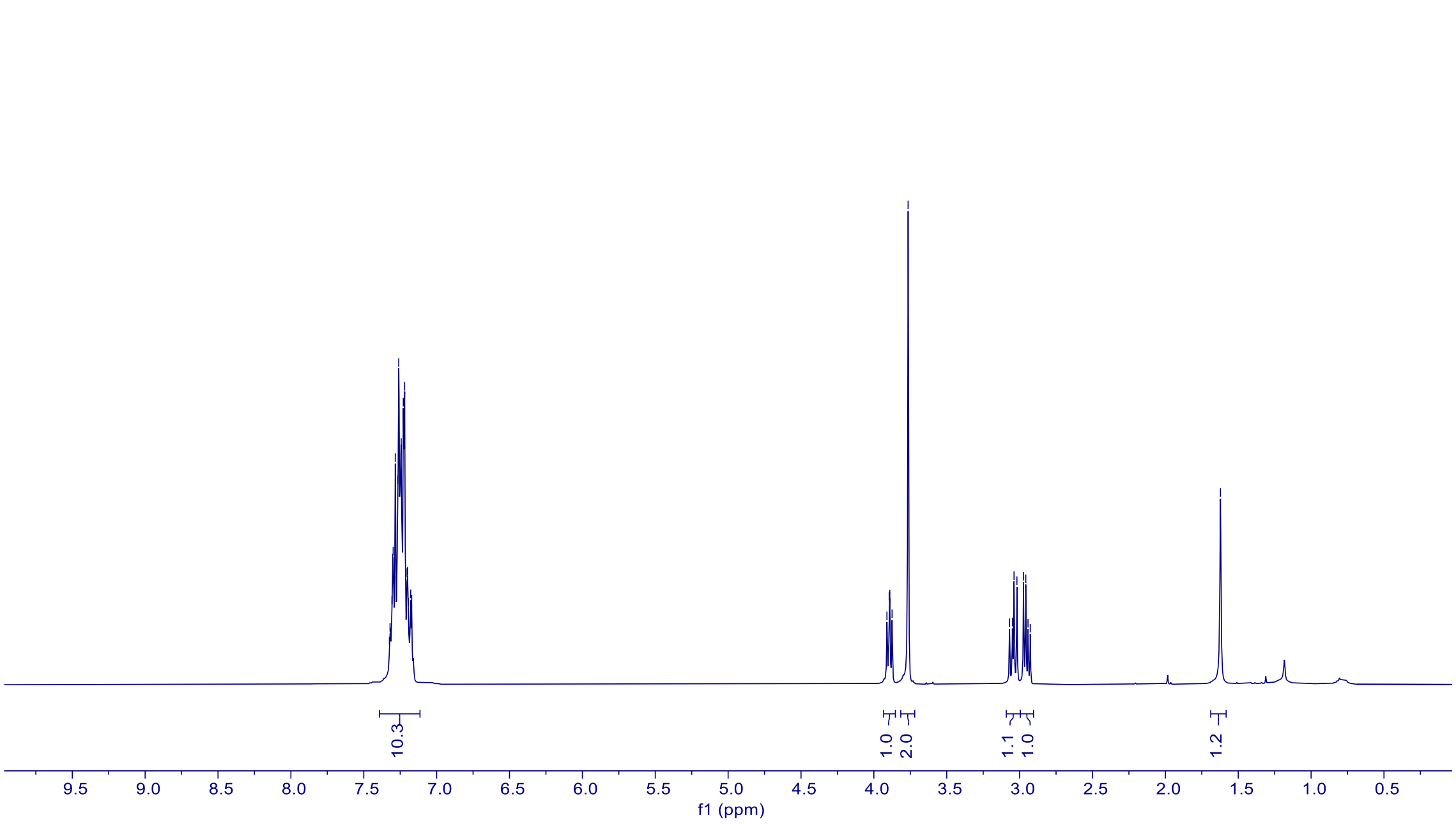

10c – ^{13}C NMR (151 MHz, $CDCl_3$)

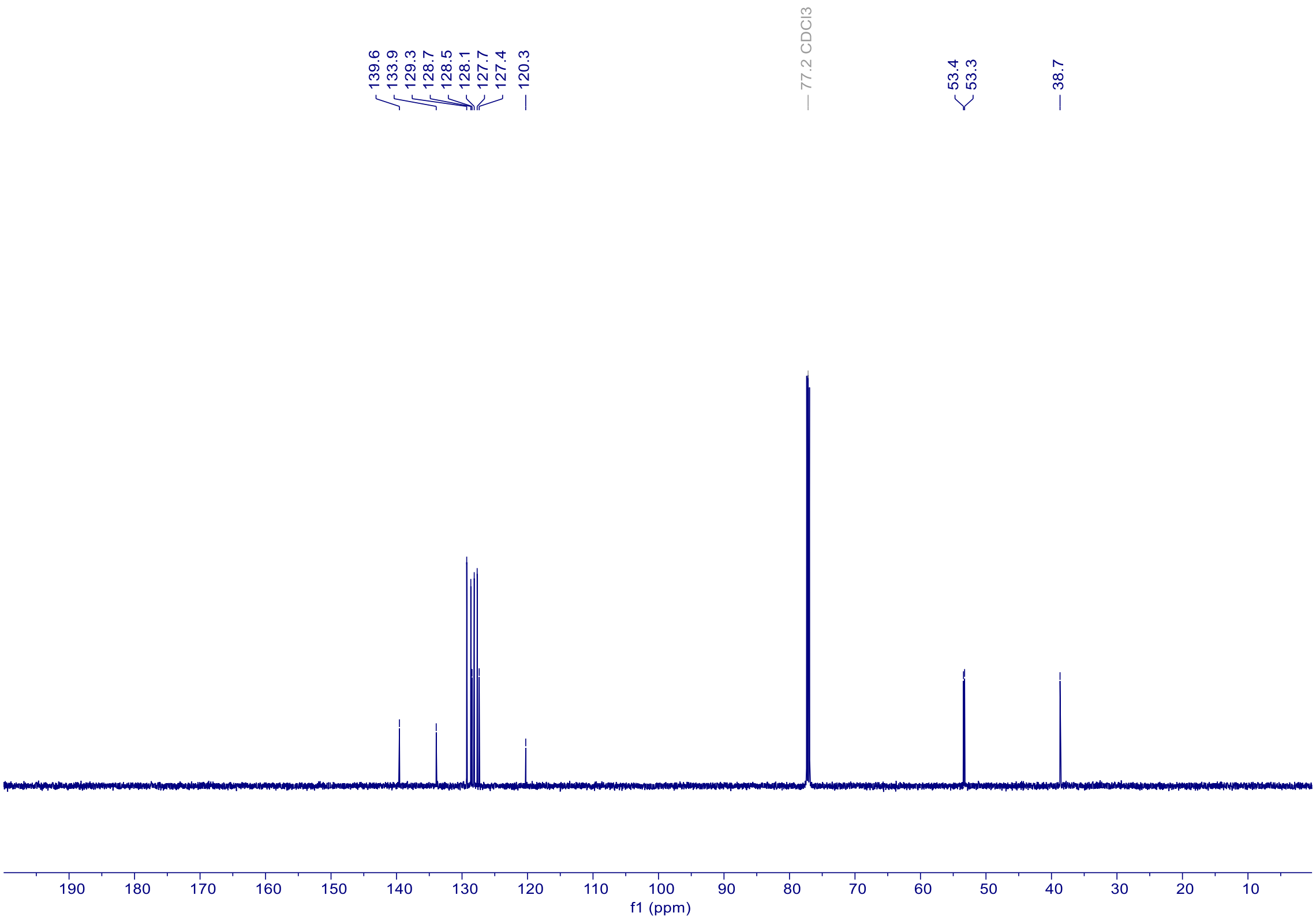

11c – ^{1}H NMR (400 MHz, $CDCl_3$)

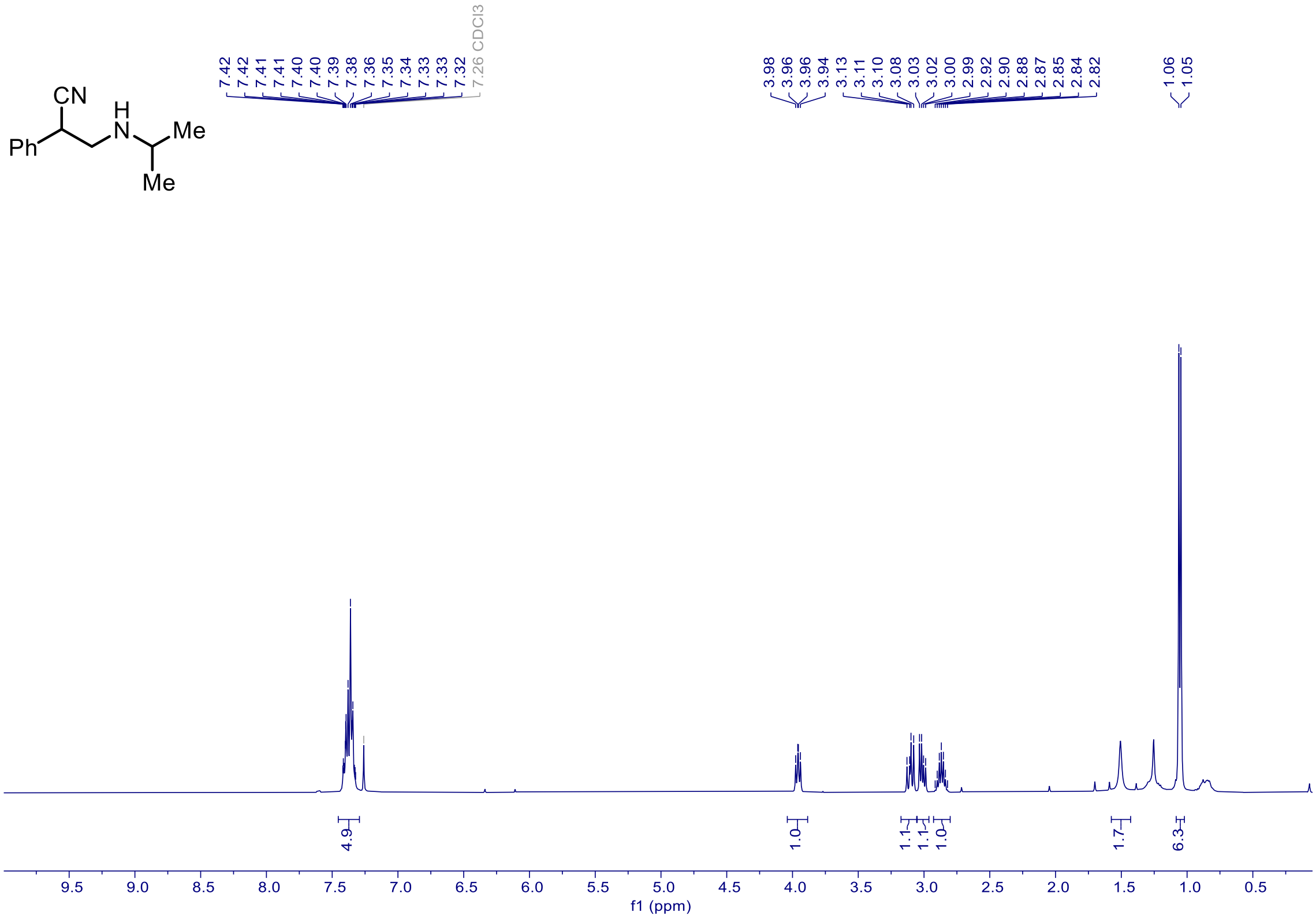

11c – ^{13}C NMR (151 MHz, $CDCl_3$)

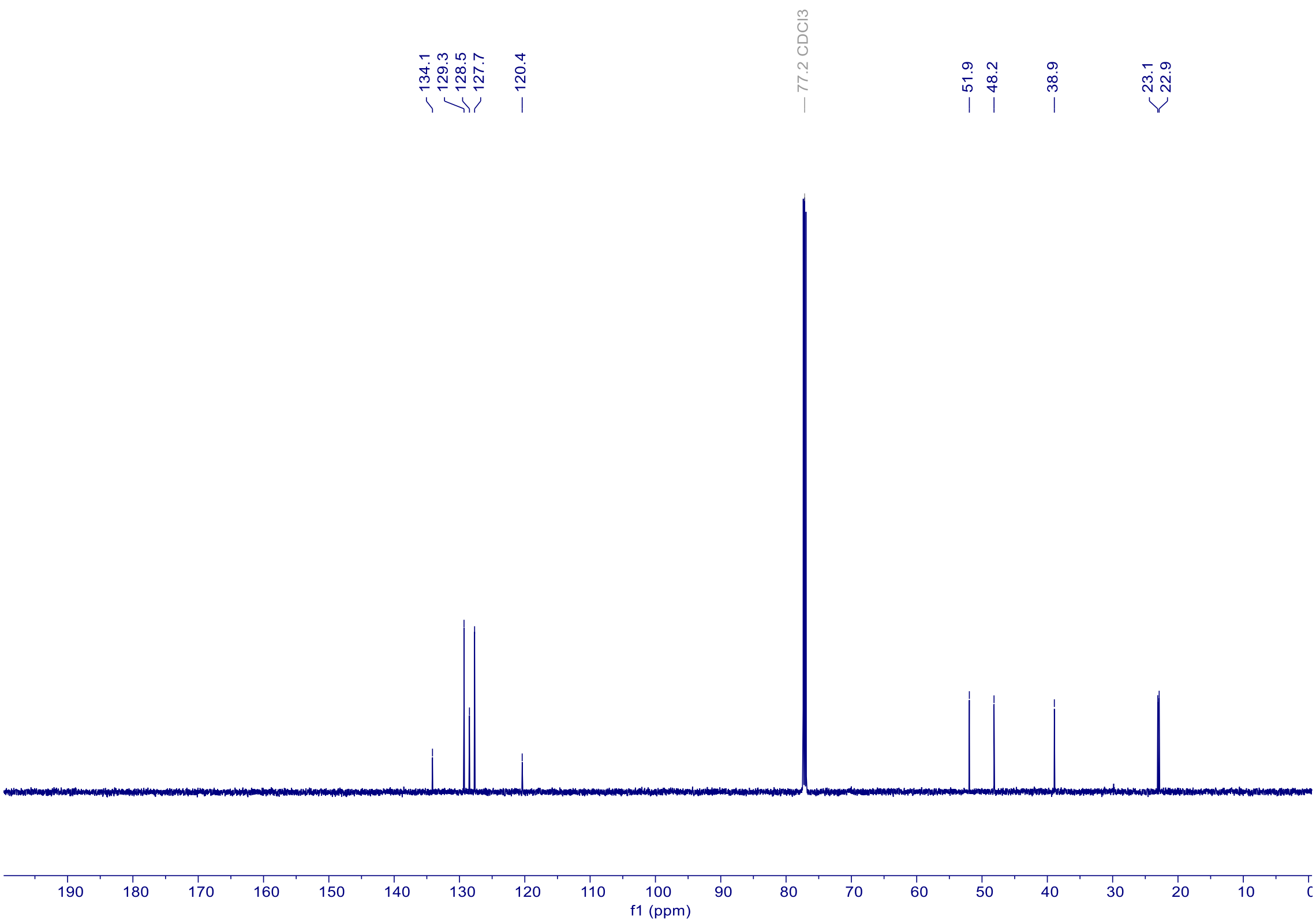

12c – ^{1}H NMR (600 MHz, CDCl$_3$)

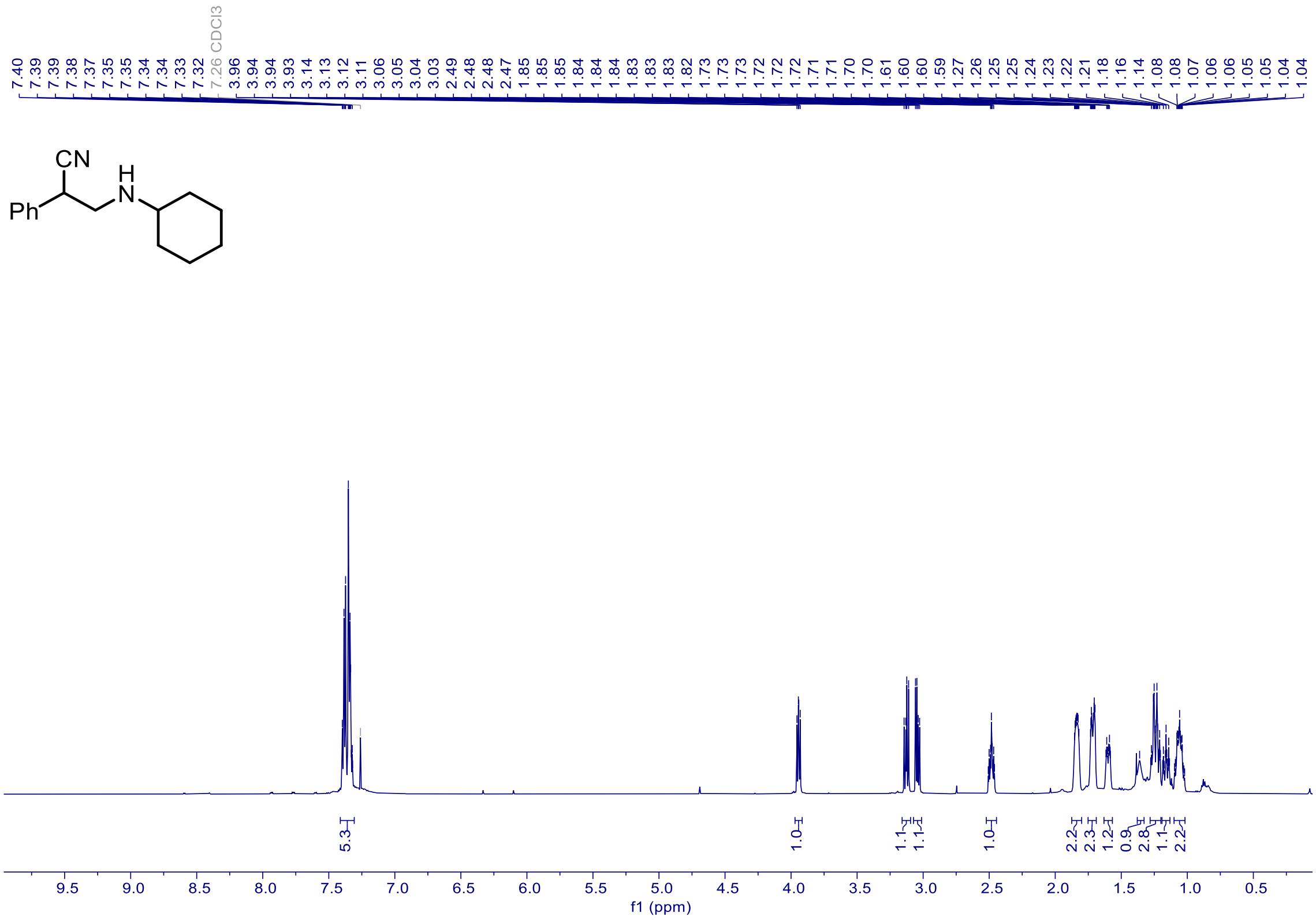

12c – ^{13}C NMR (151 MHz, CDCl$_3$)

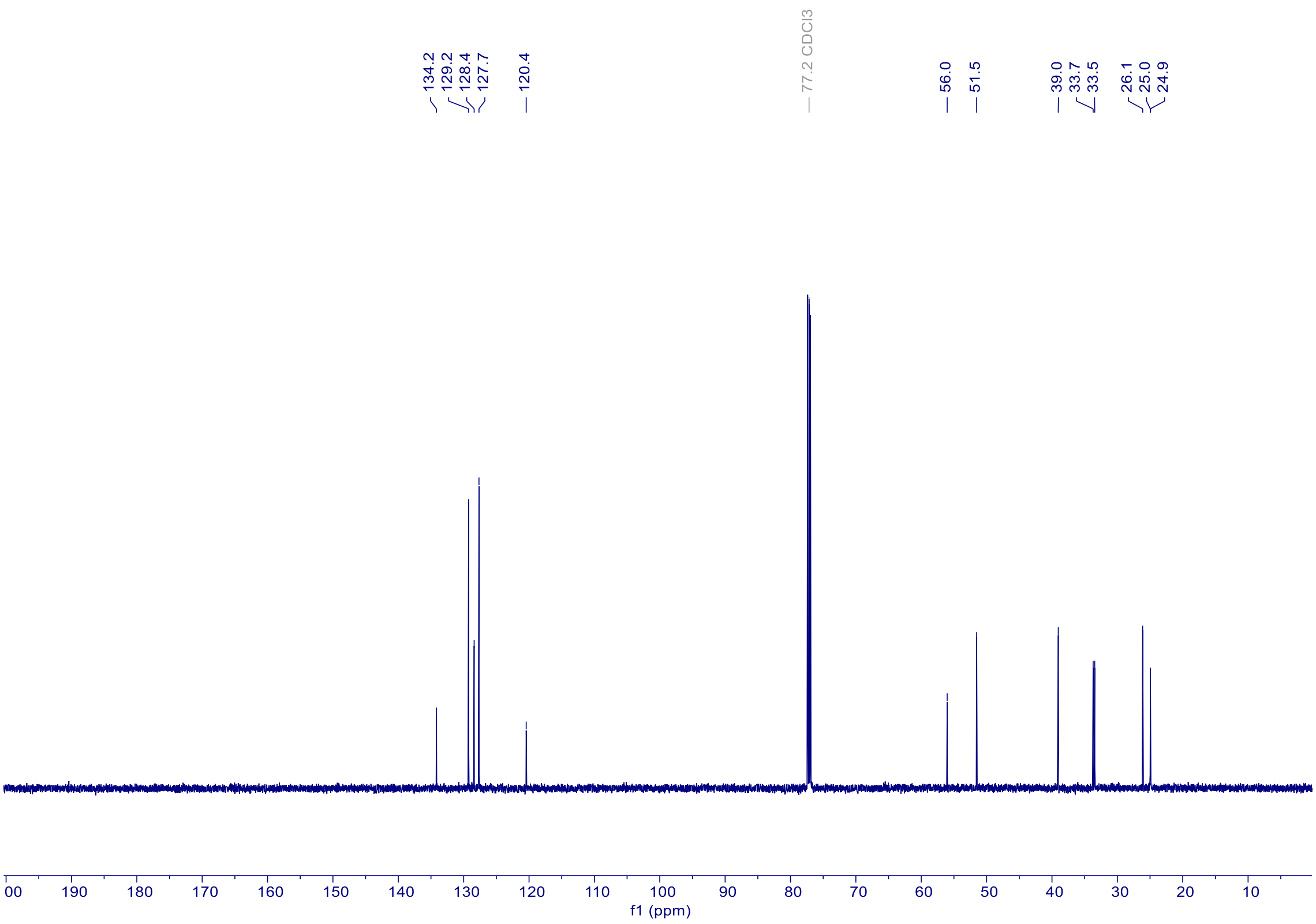

17b – ^{1}H NMR (600 MHz, $CDCl_3$)

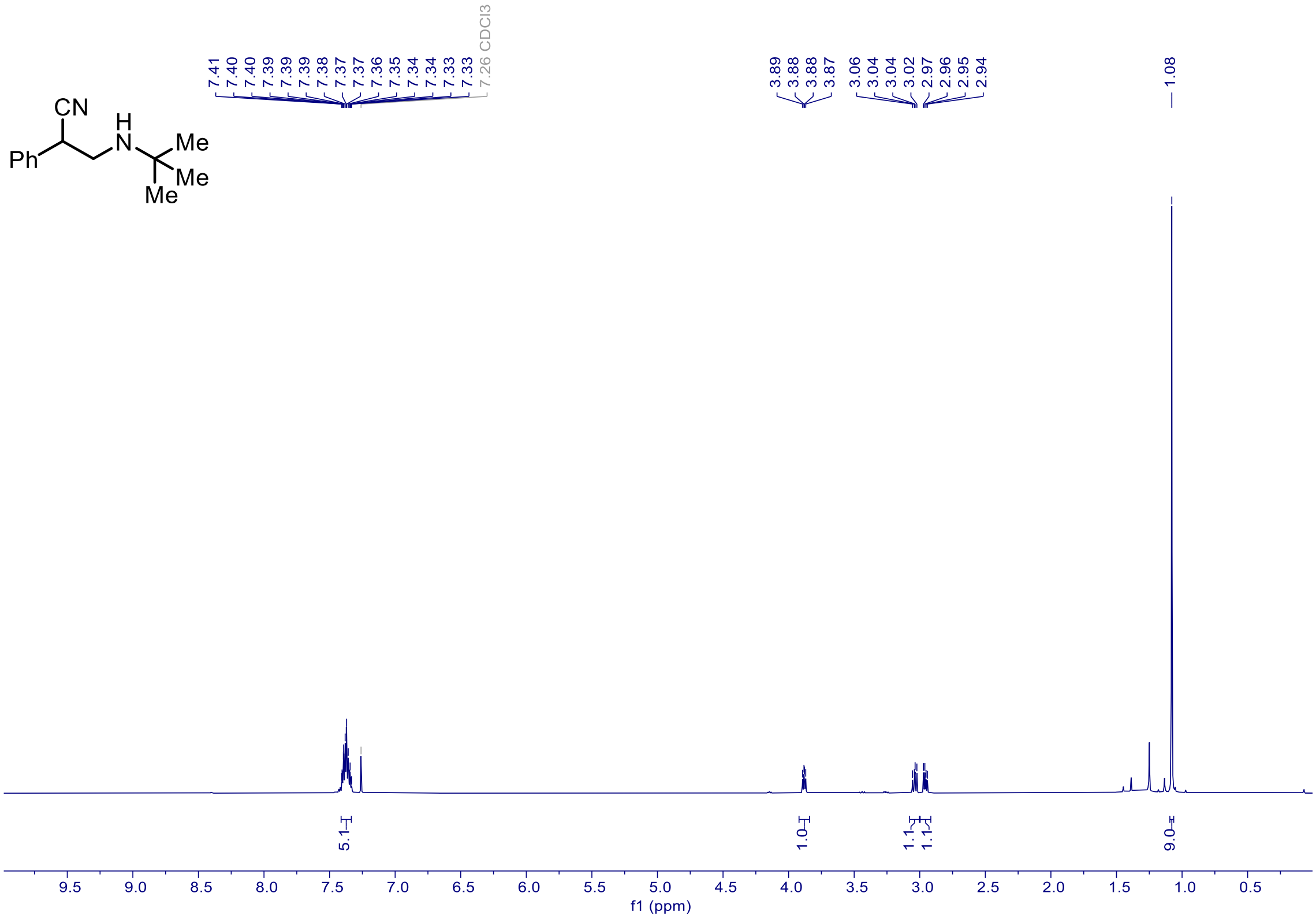

17b – ^{13}C NMR (151 MHz, $CDCl_3$)

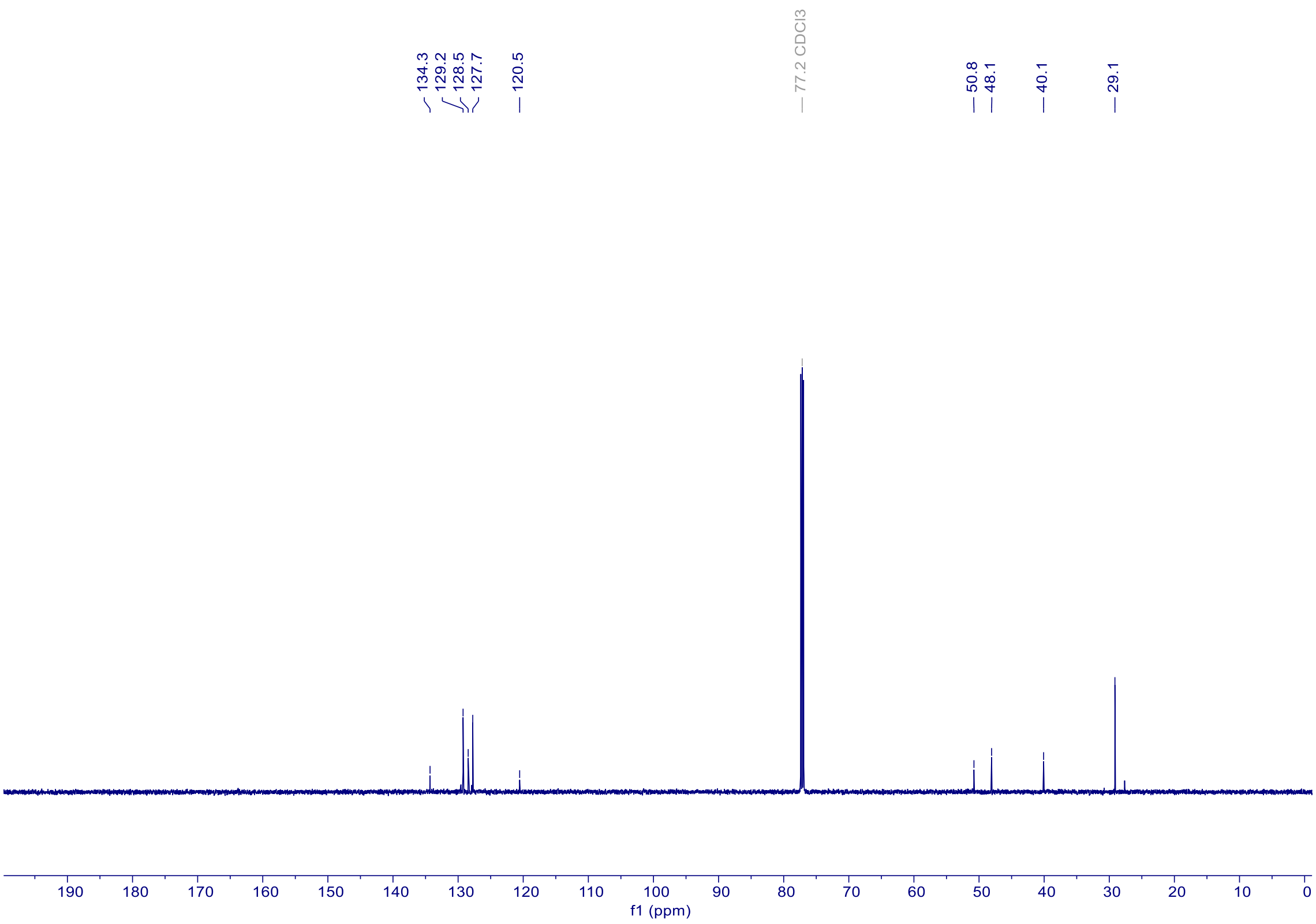

18b – ¹H NMR (600 MHz, $CDCl_3$)

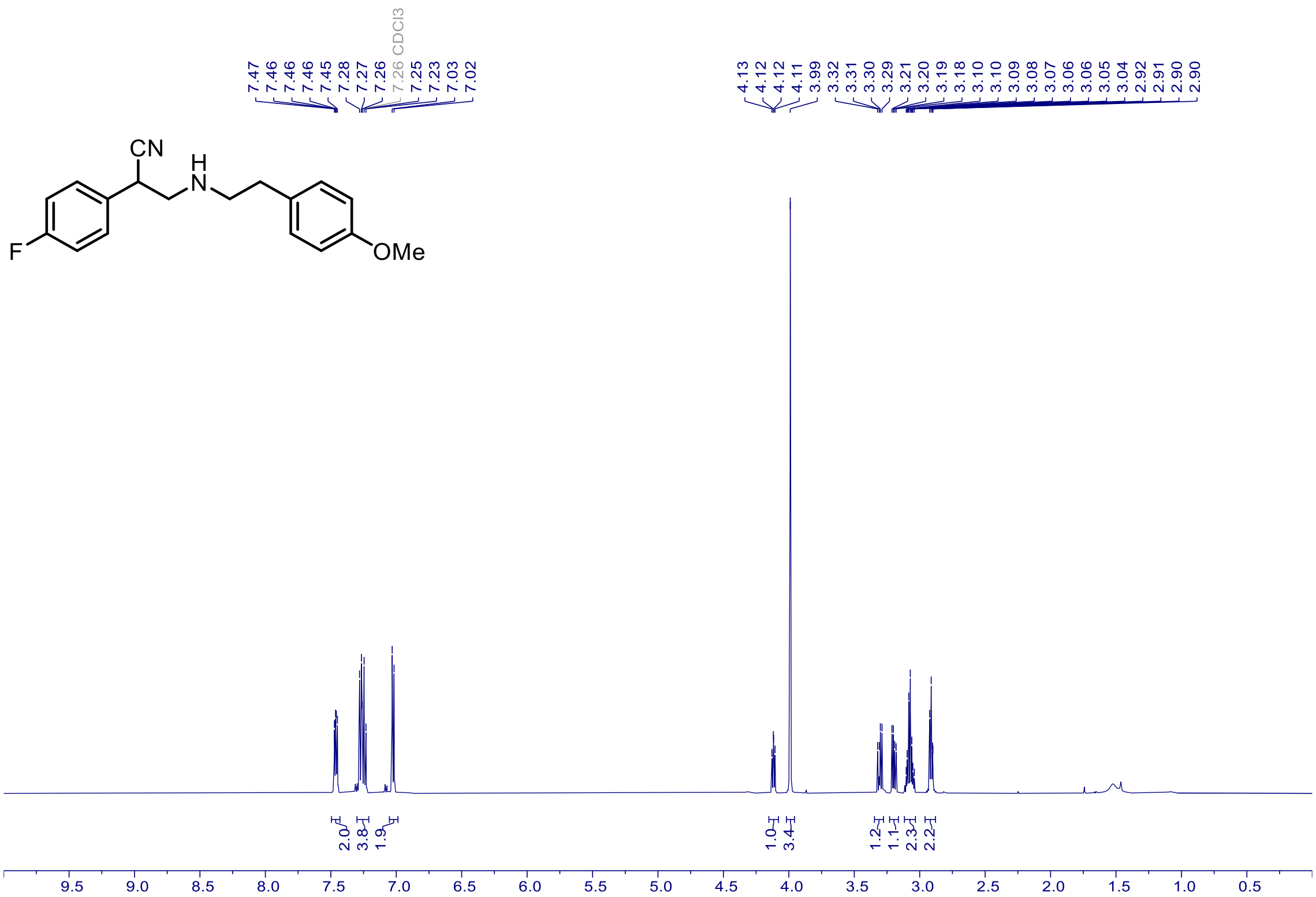

18b – ¹³C NMR (151 MHz, $CDCl_3$)

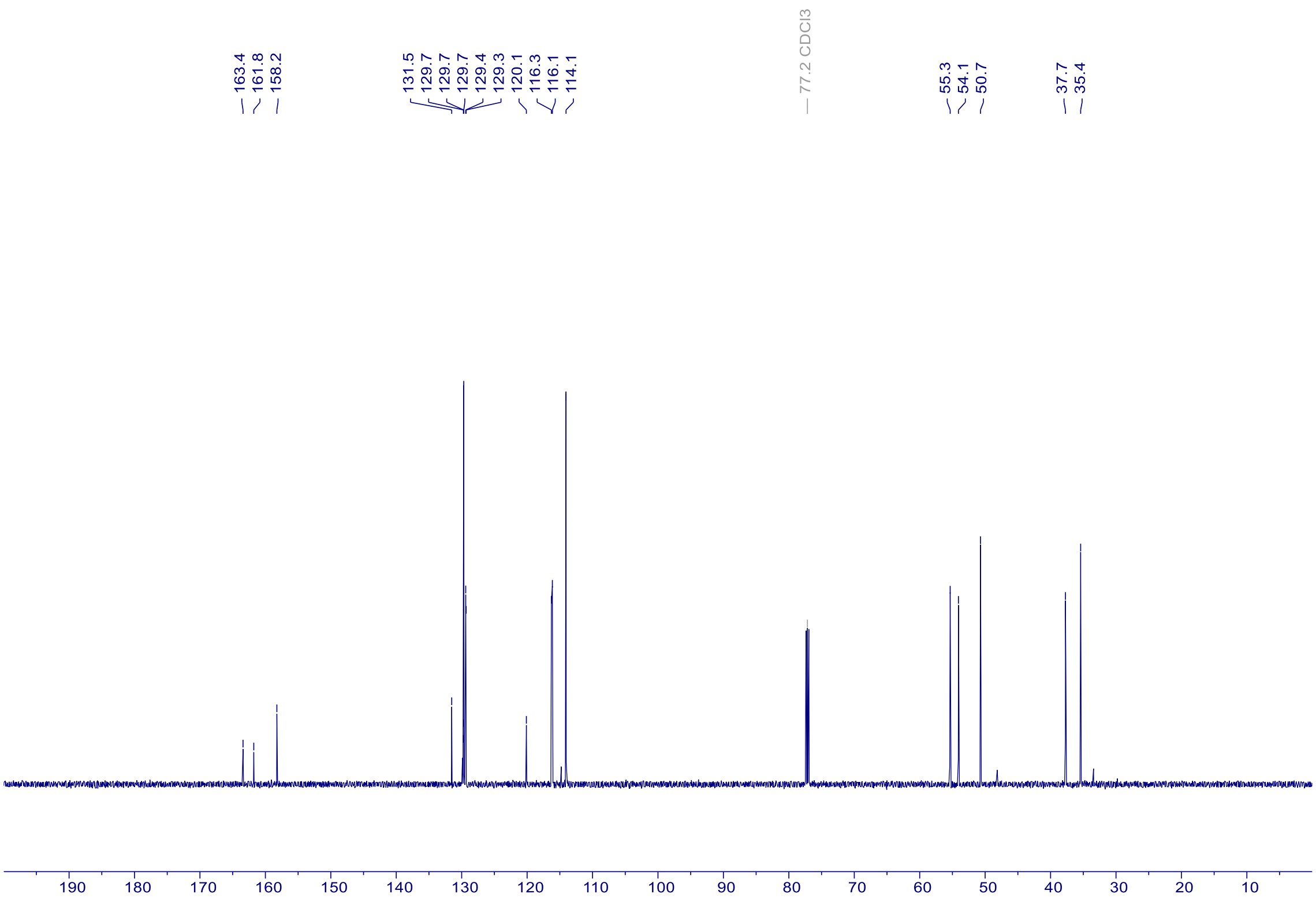

18b – ^{19}F NMR (564 MHz, $CDCl_3$)

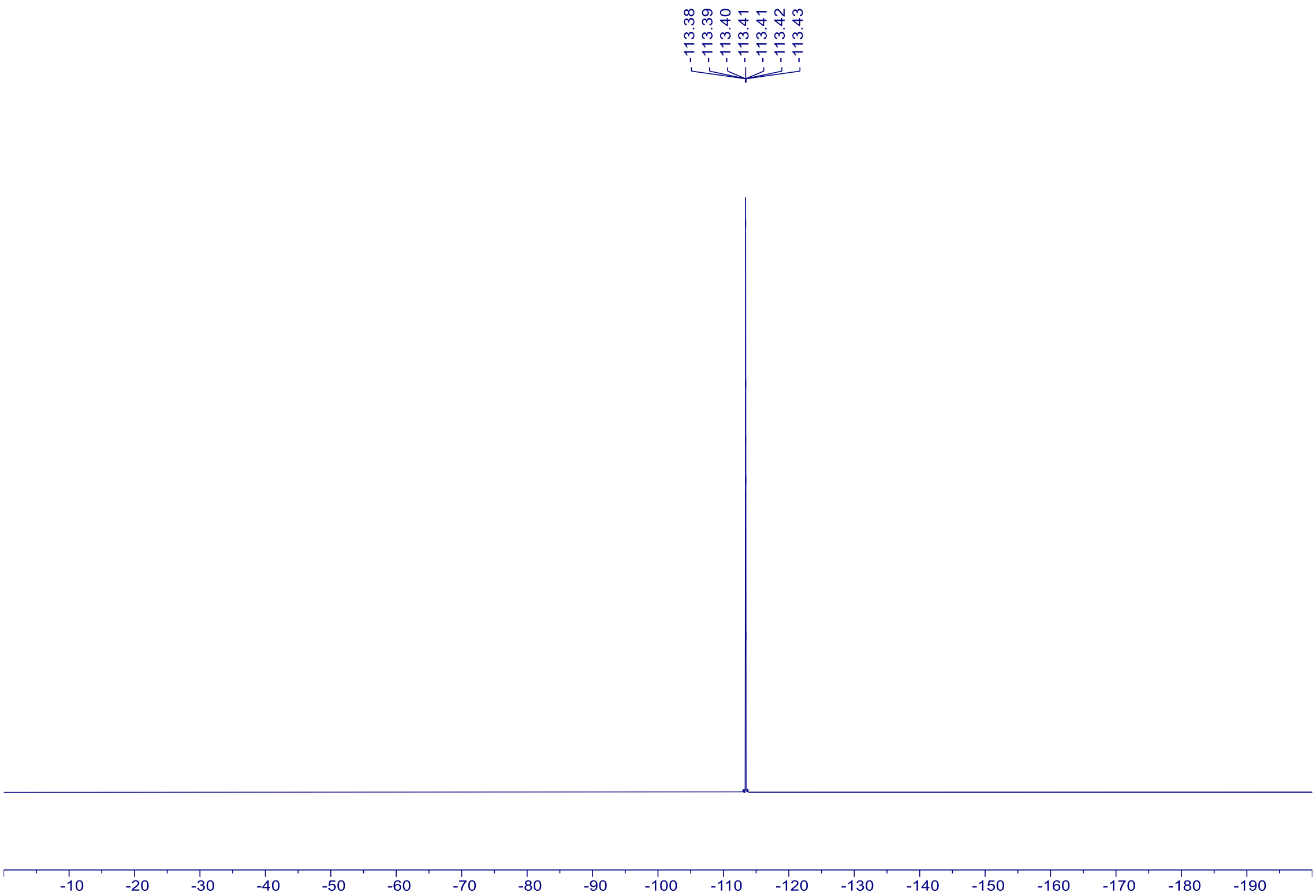

19b – ^{1}H NMR (600 MHz, $CDCl_3$)

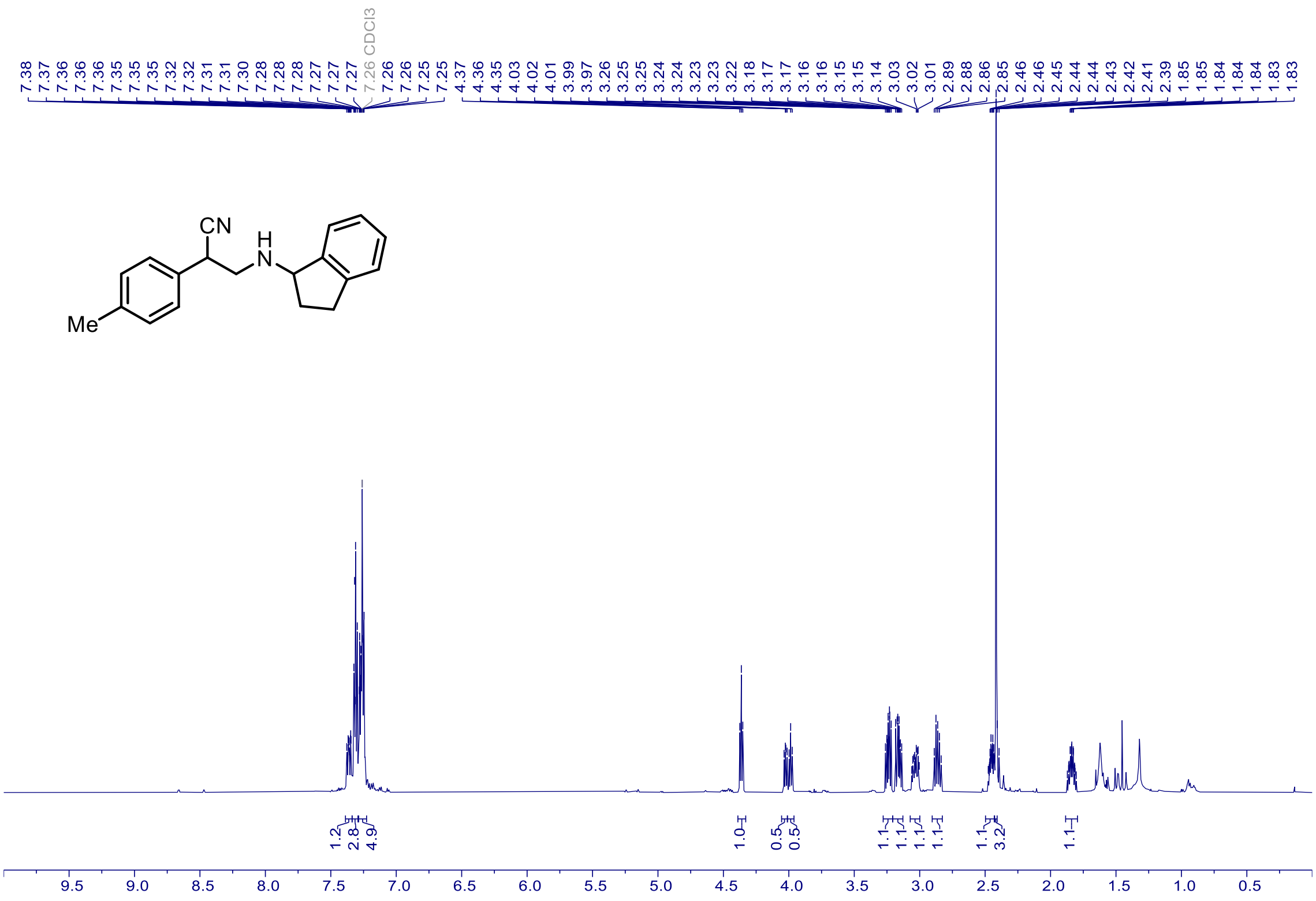

19b – ^{13}C NMR (151 MHz, $CDCl_3$)

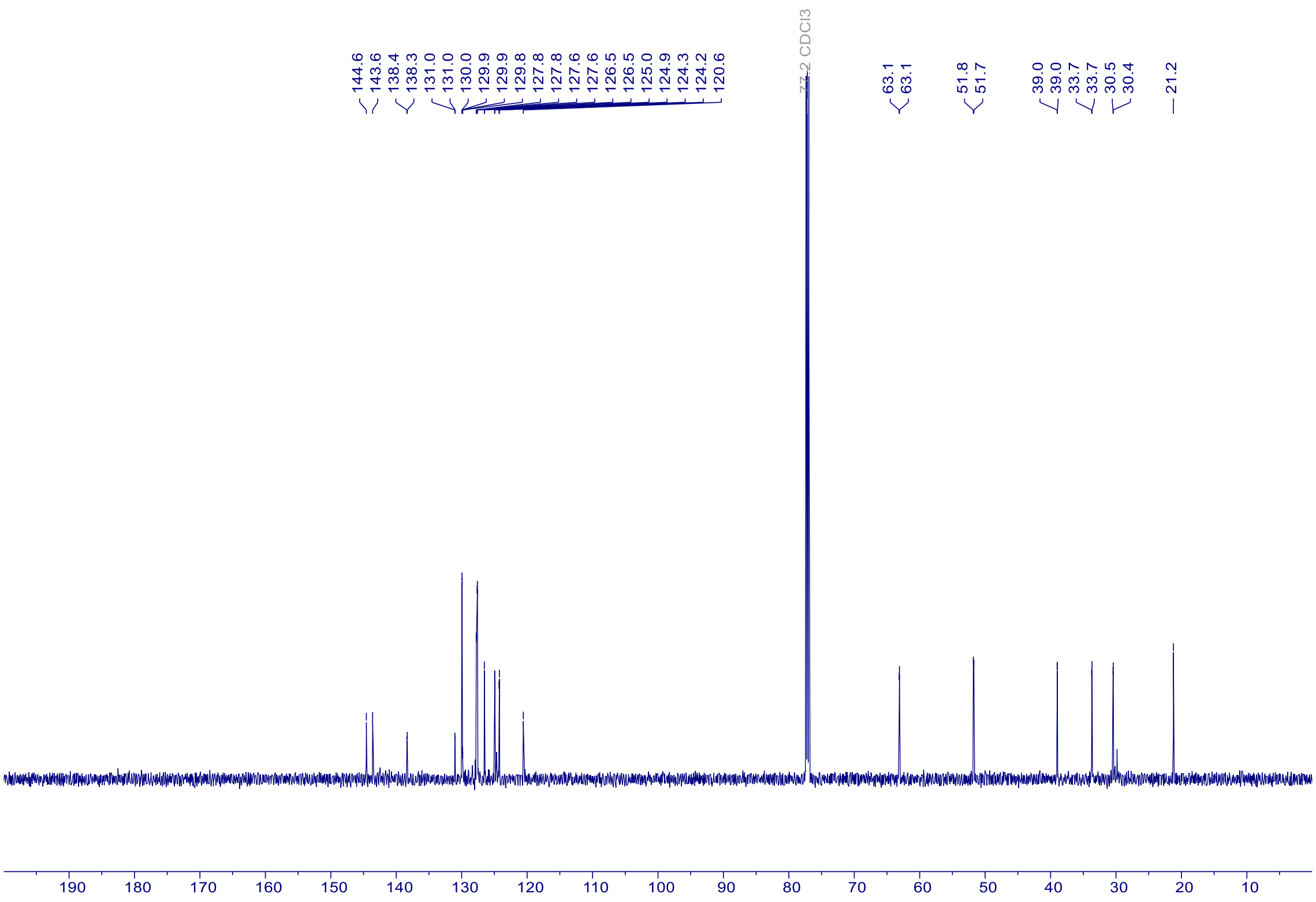

20b – ^{1}H NMR (600 MHz, $CDCl_3$)

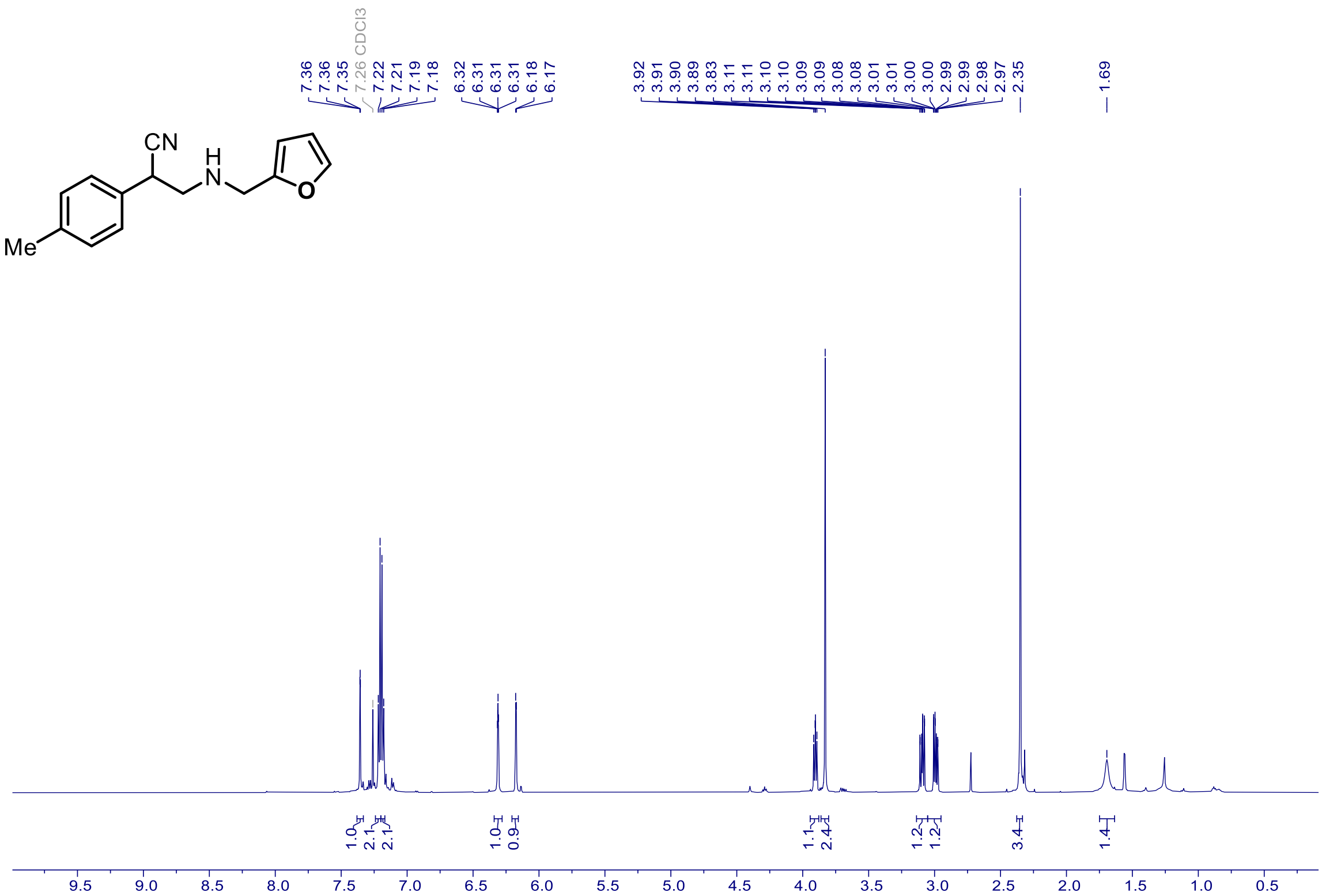

20b – ^{13}C NMR (151 MHz, $CDCl_3$)

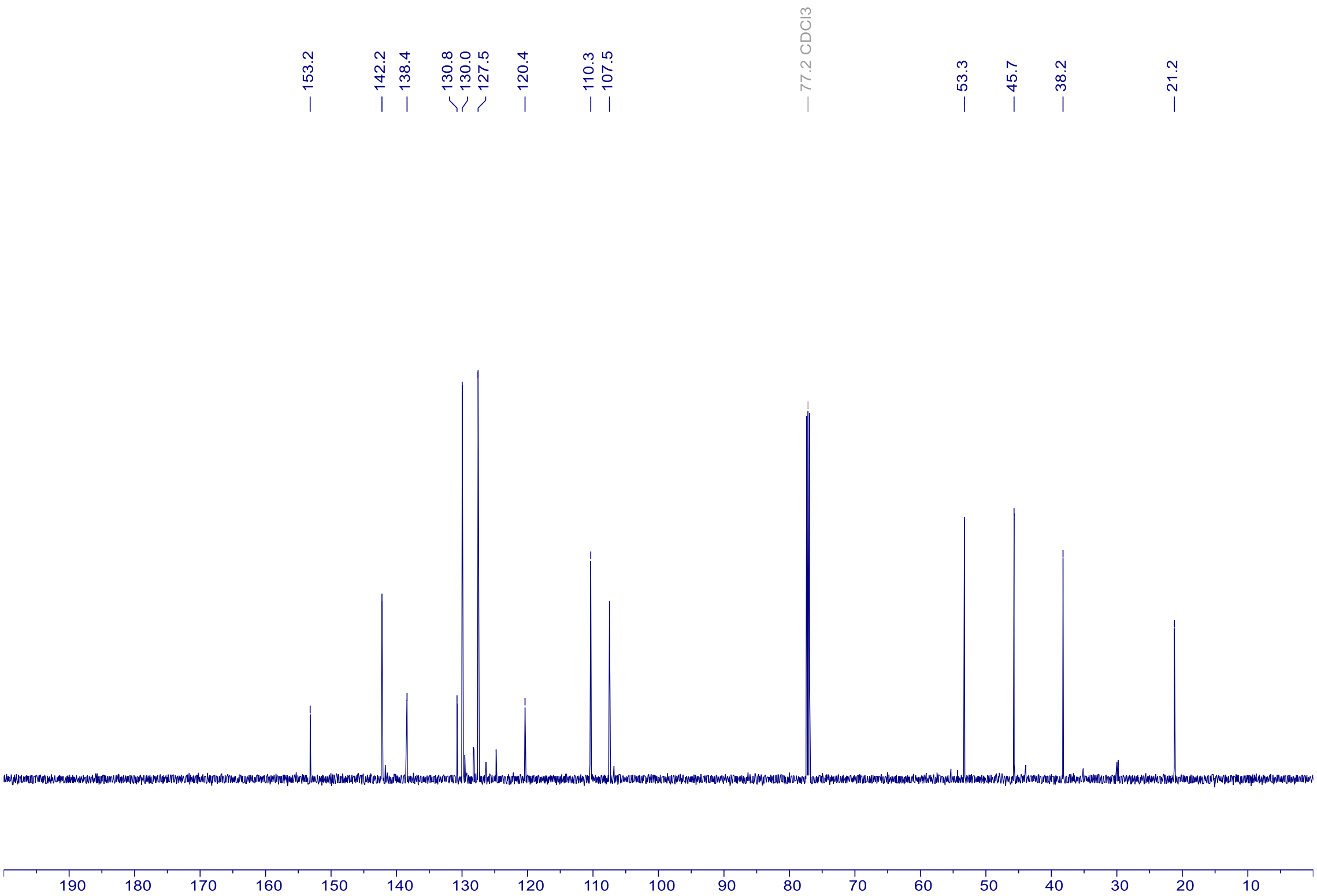

21b – ^{1}H NMR (600 MHz, $CDCl_3$)

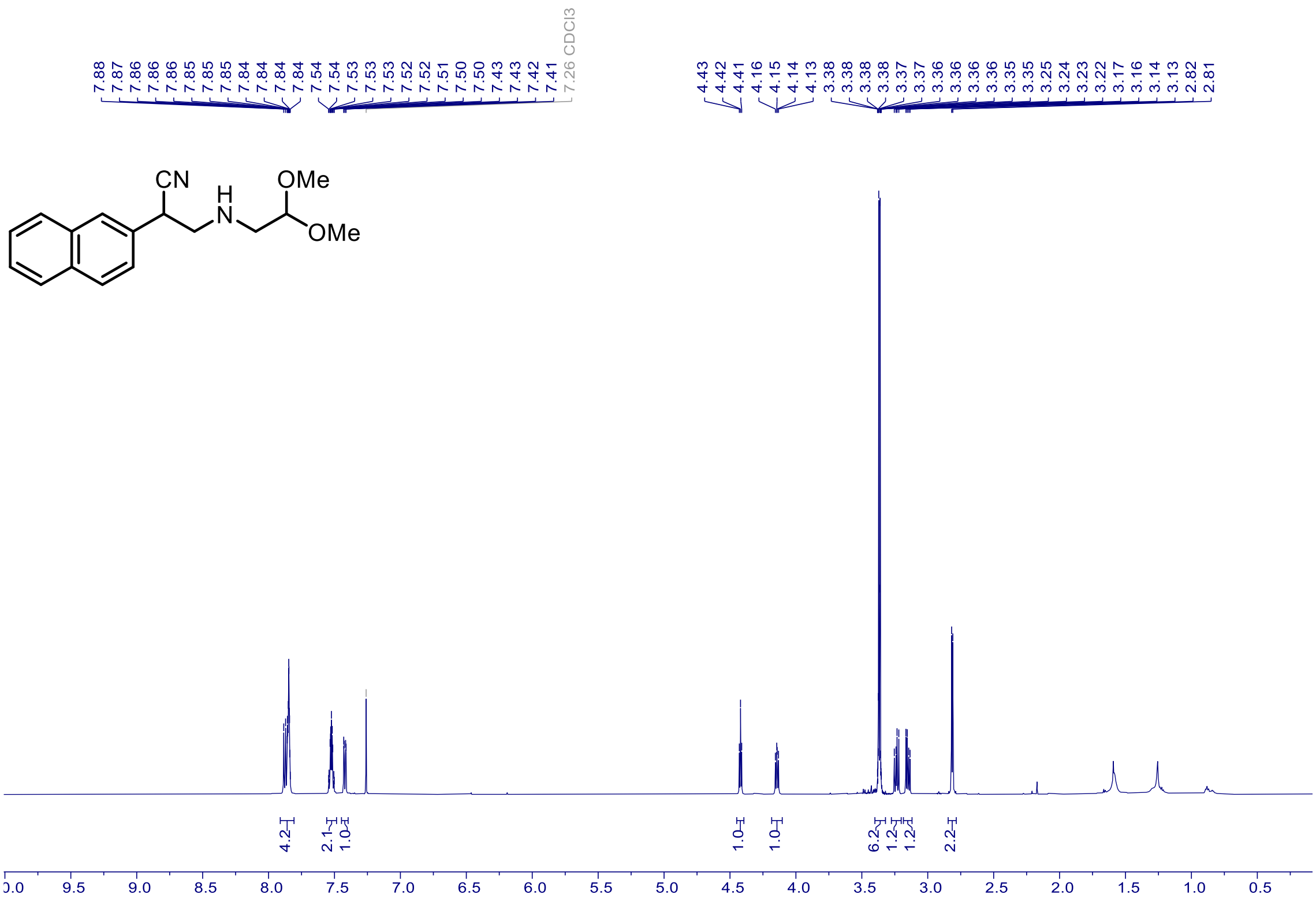

21b – ^{13}C NMR (151 MHz, $CDCl_3$)

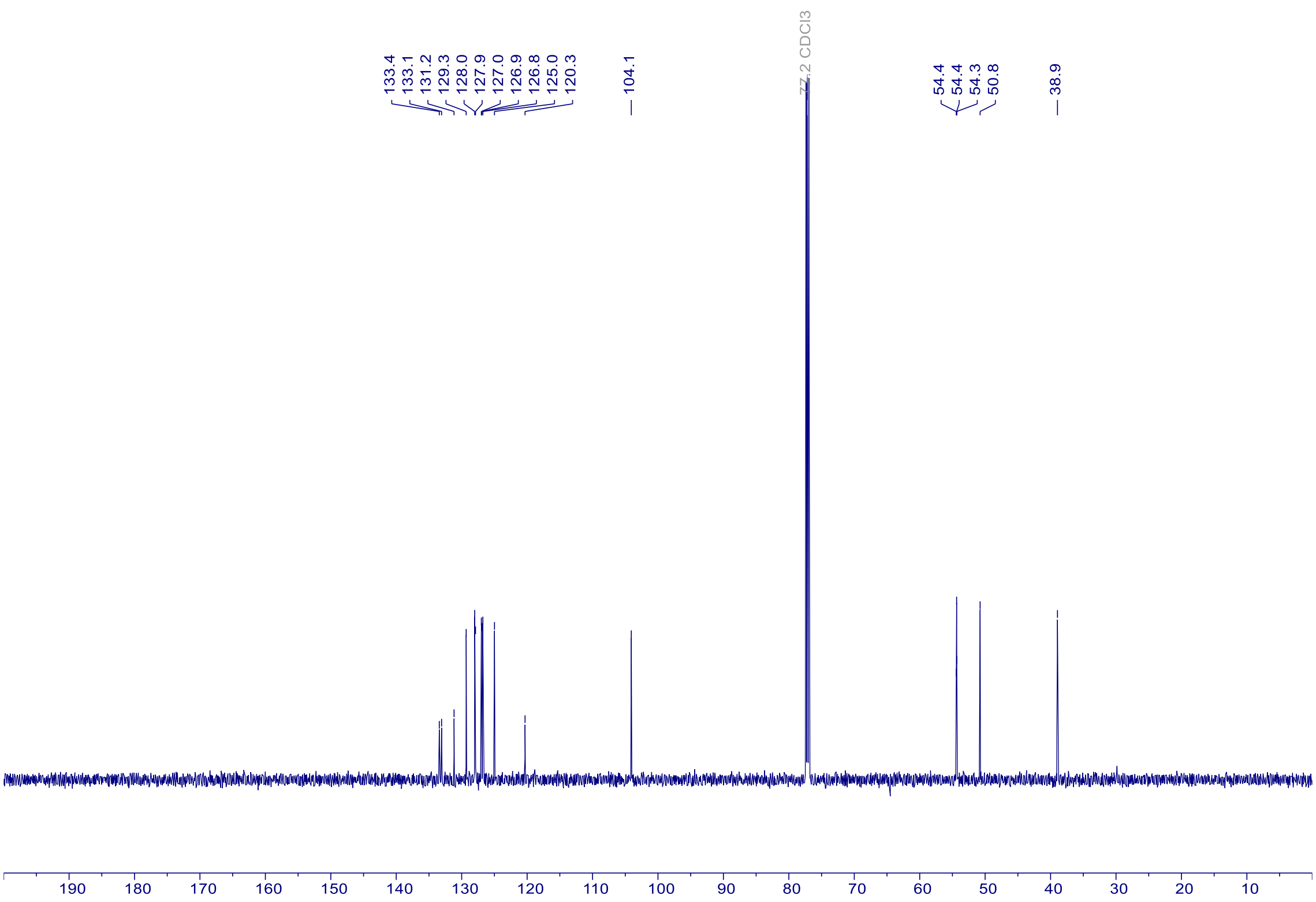

22b – ^{1}H NMR (600 MHz, $CDCl_3$)

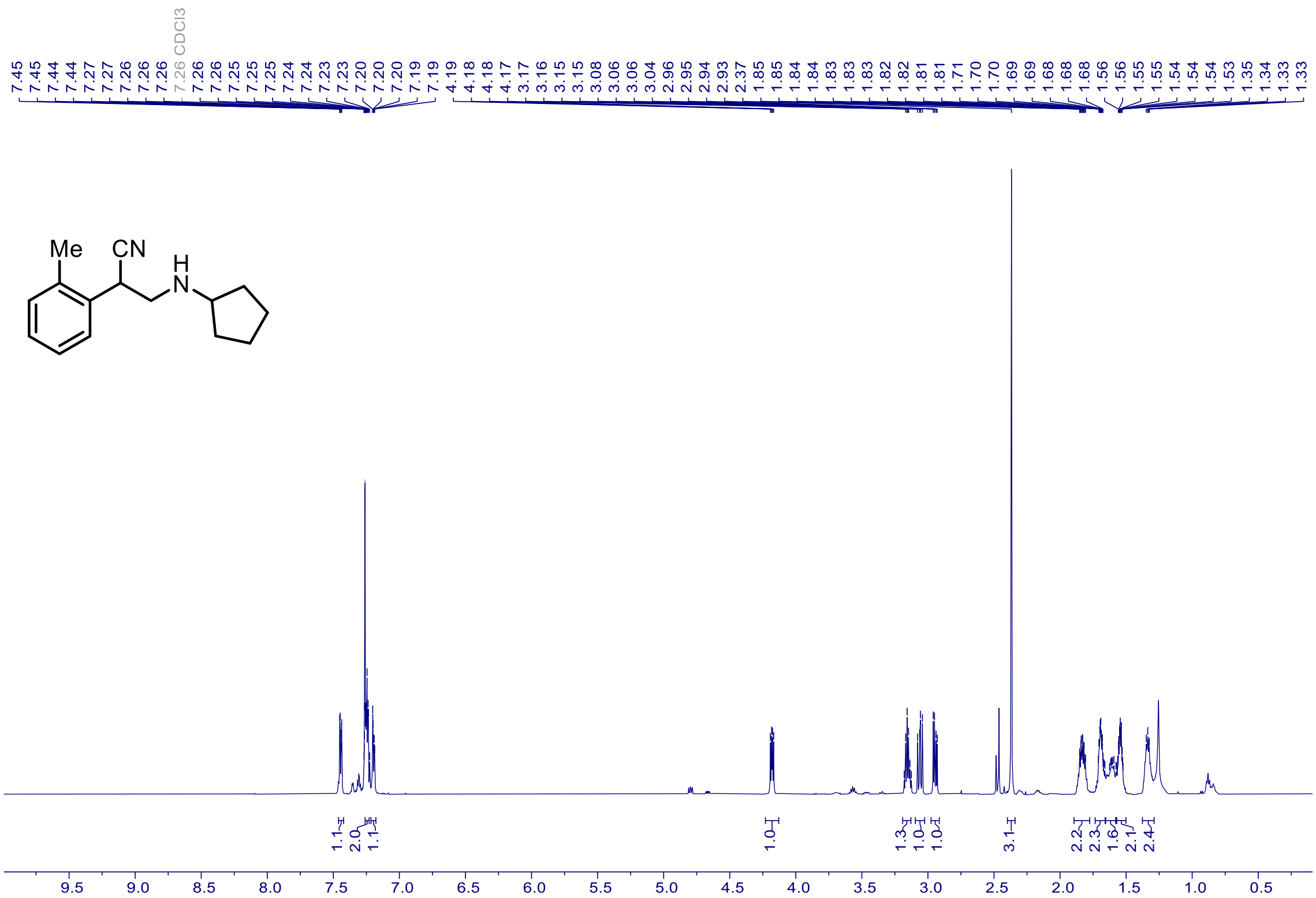

22b – ^{13}C NMR (151 MHz, $CDCl_3$)

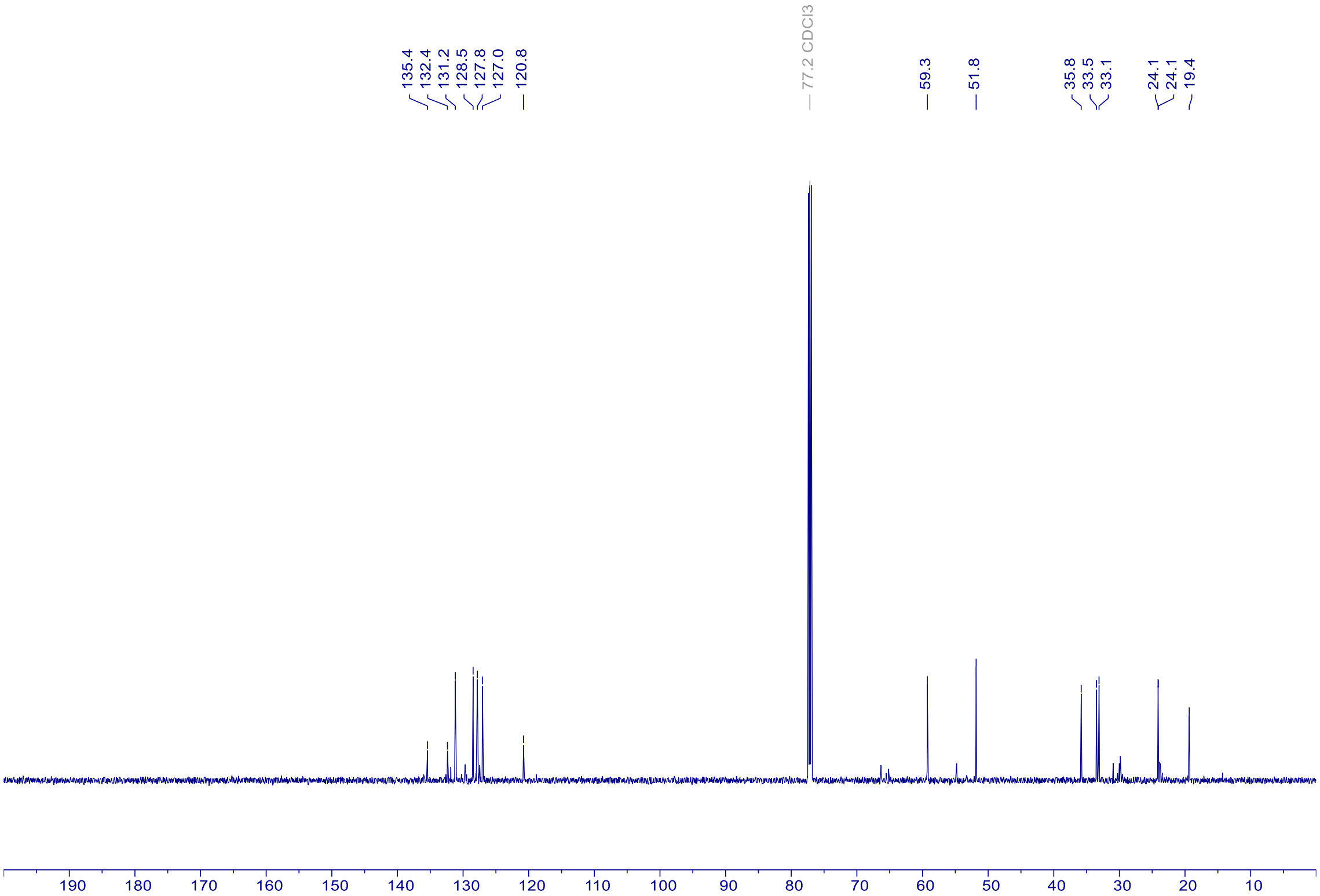

23b – ^{1}H NMR (600 MHz, $CDCl_3$)

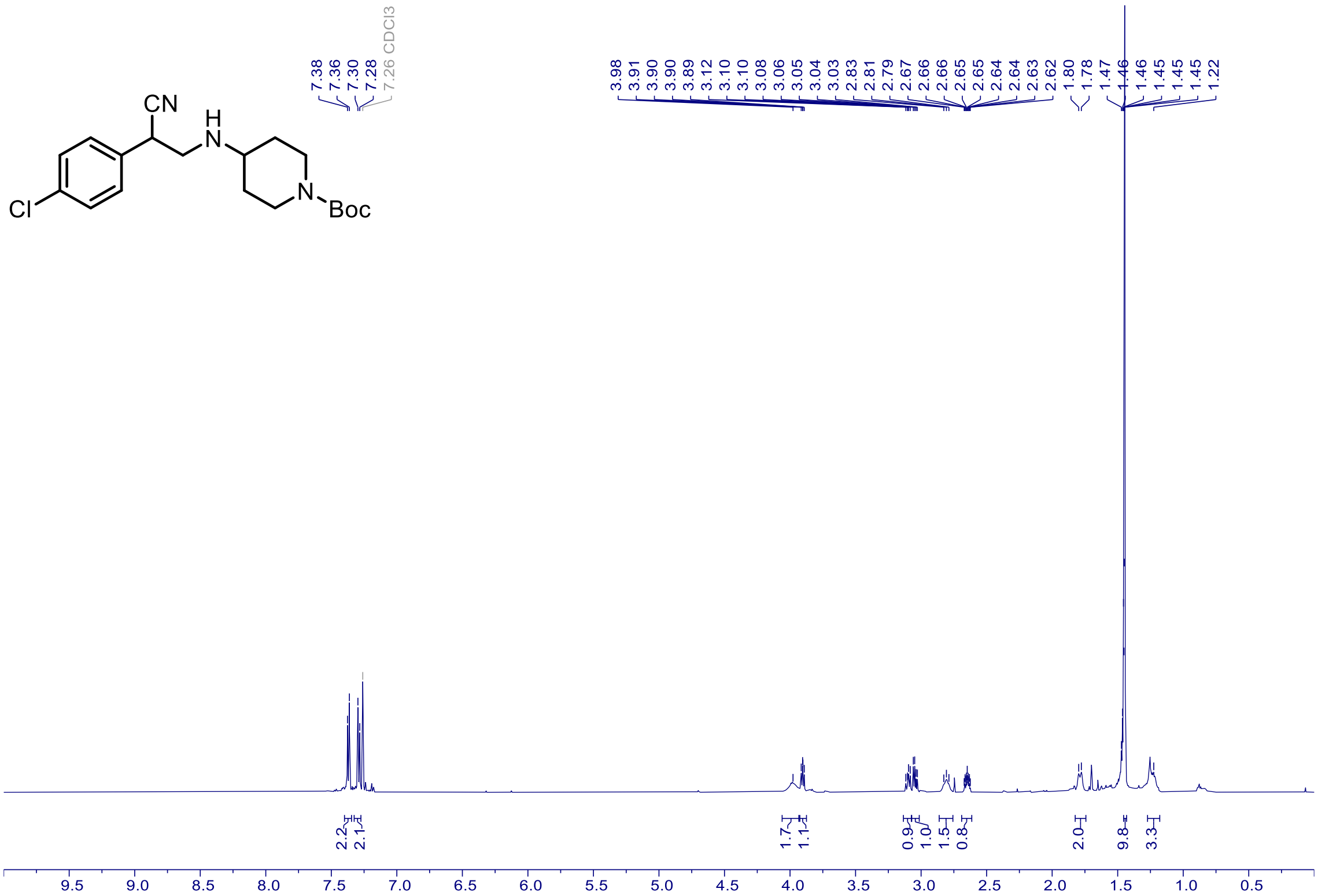

23b – ^{13}C NMR (101 MHz, $CDCl_3$)

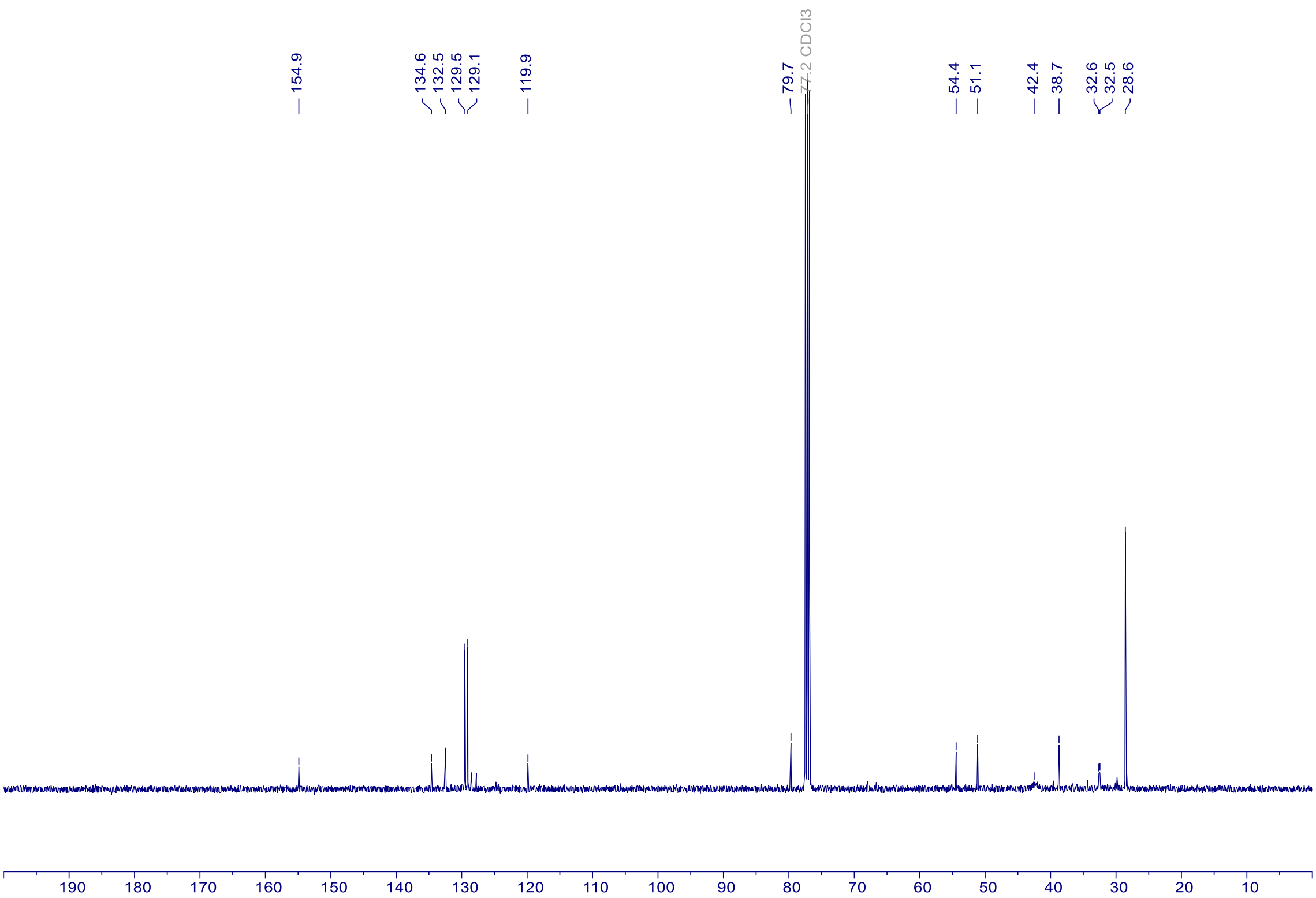

24b – ^{1}H NMR (600 MHz, $CDCl_3$)

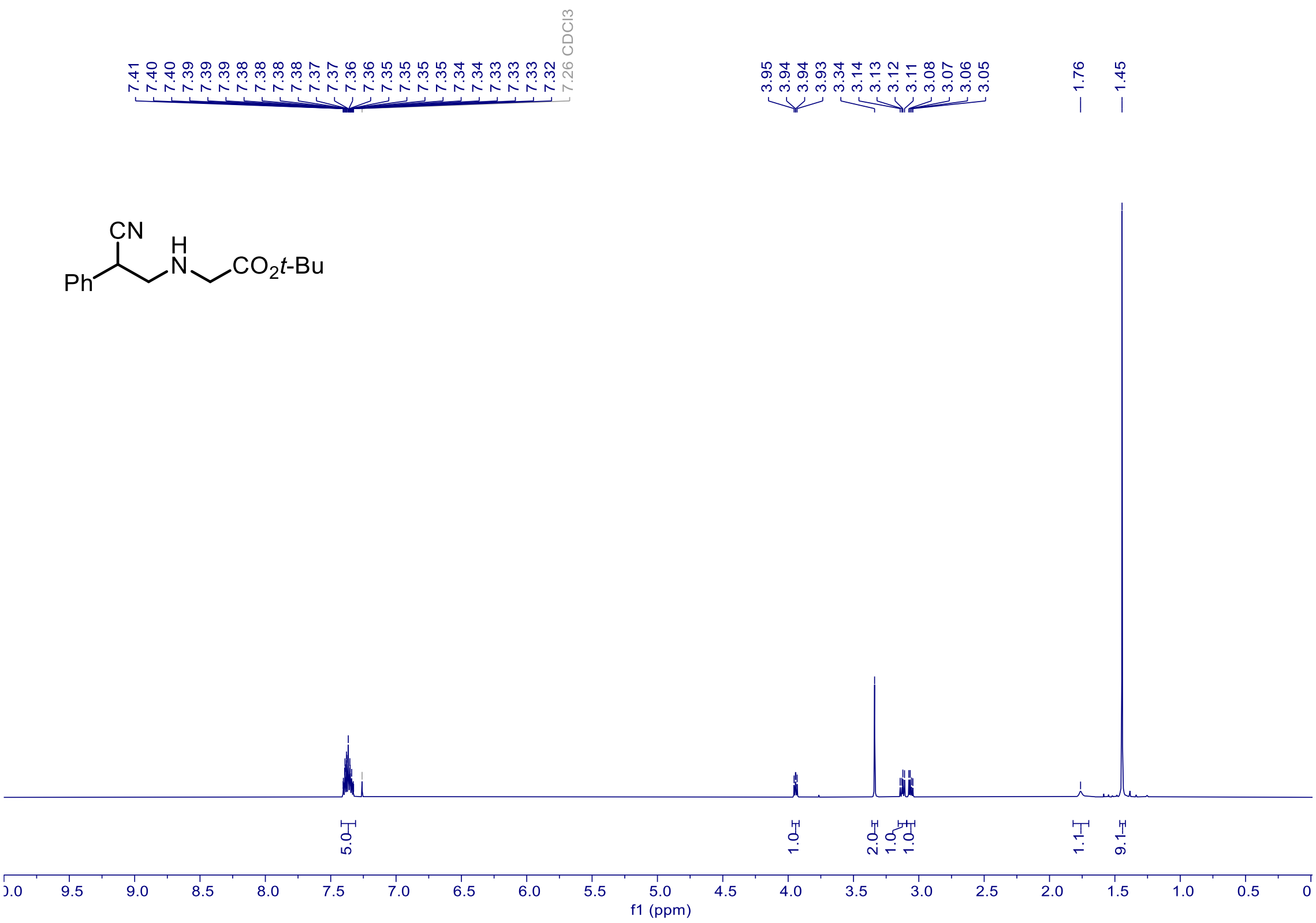

24b – ^{13}C NMR (151 MHz, $CDCl_3$)

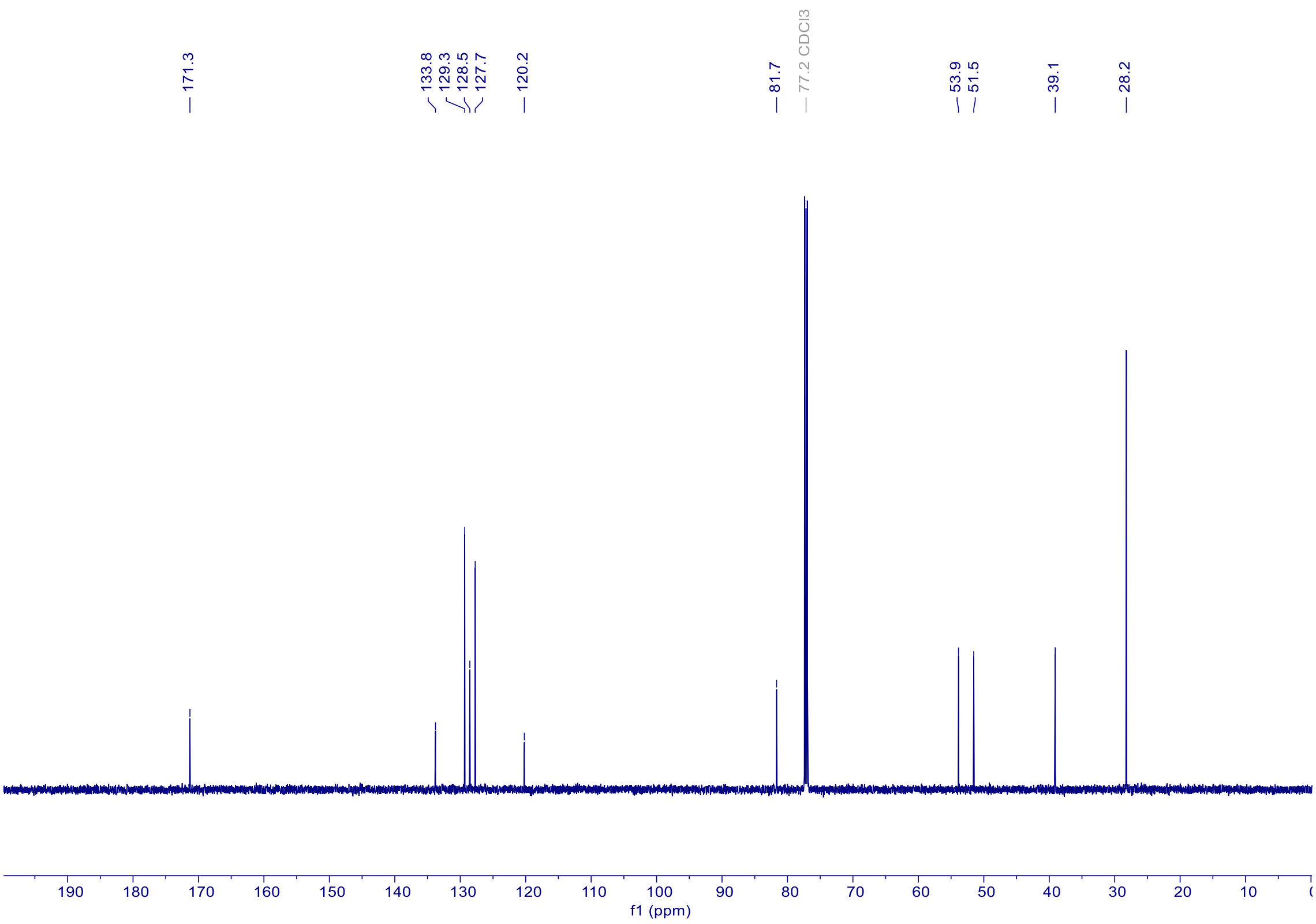

25b – ^{1}H NMR (600 MHz, $CDCl_3$)

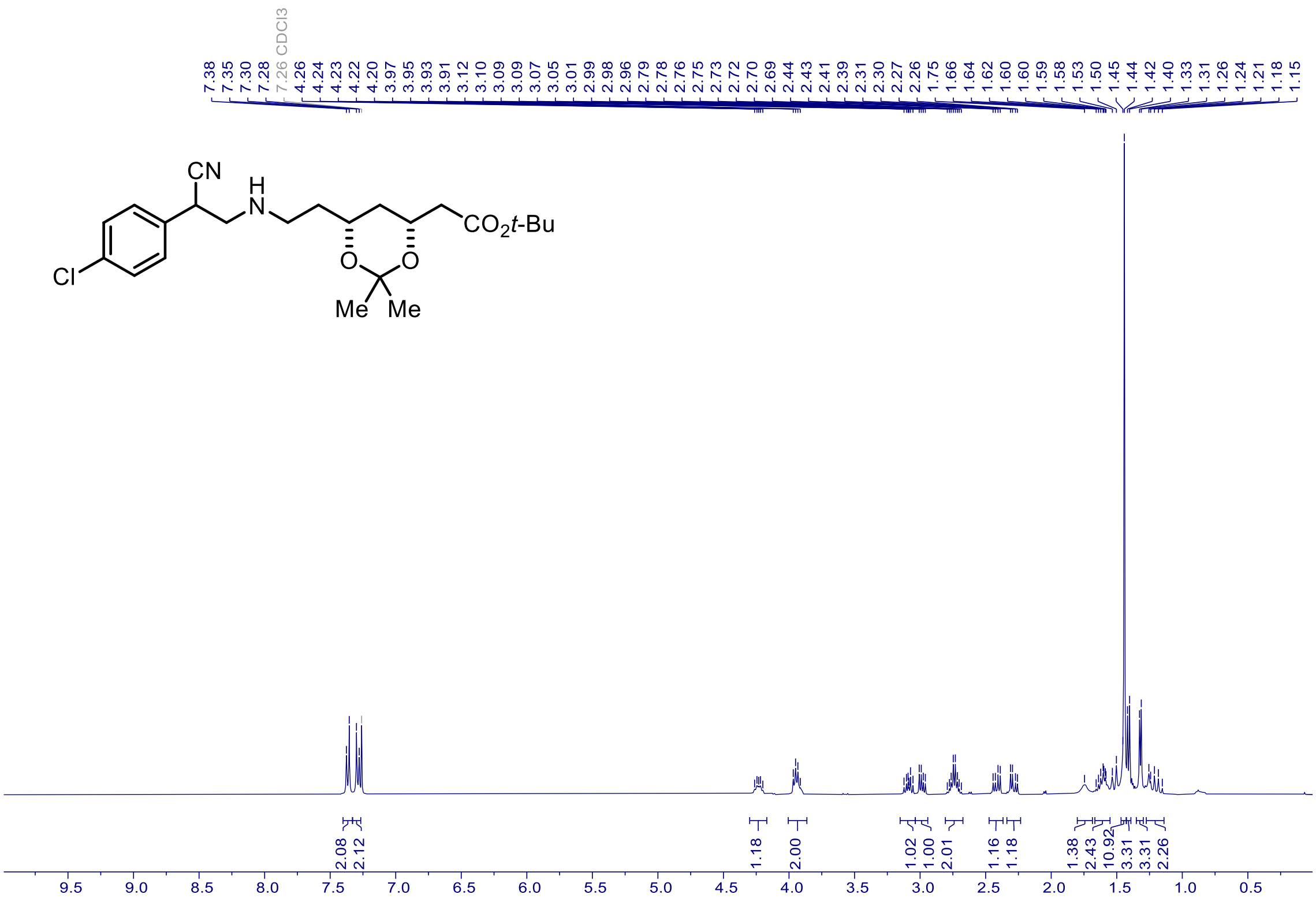

25b – ^{13}C NMR (151 MHz, $CDCl_3$)

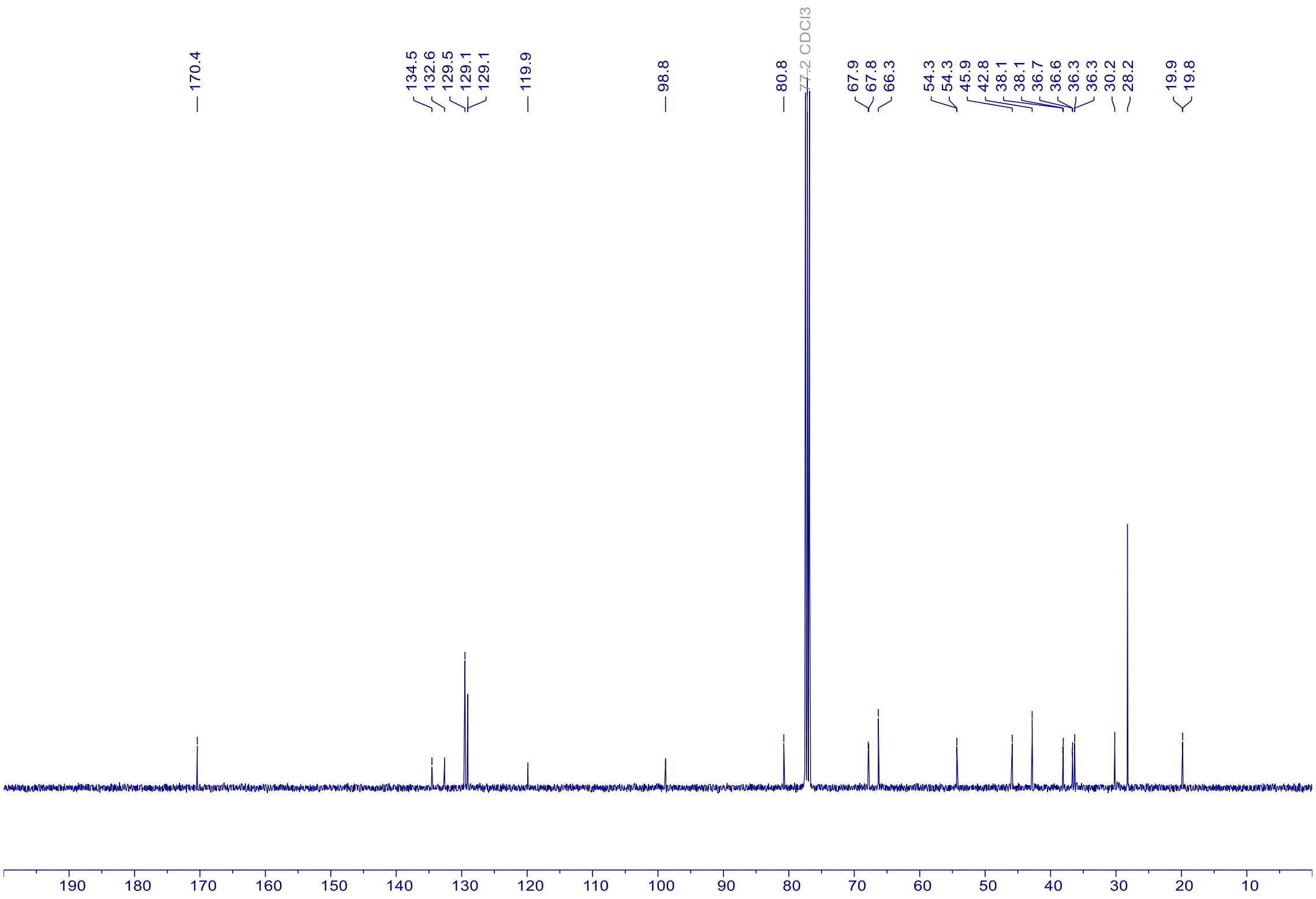

26b – ^{1}H NMR (600 MHz, $CDCl_3$)

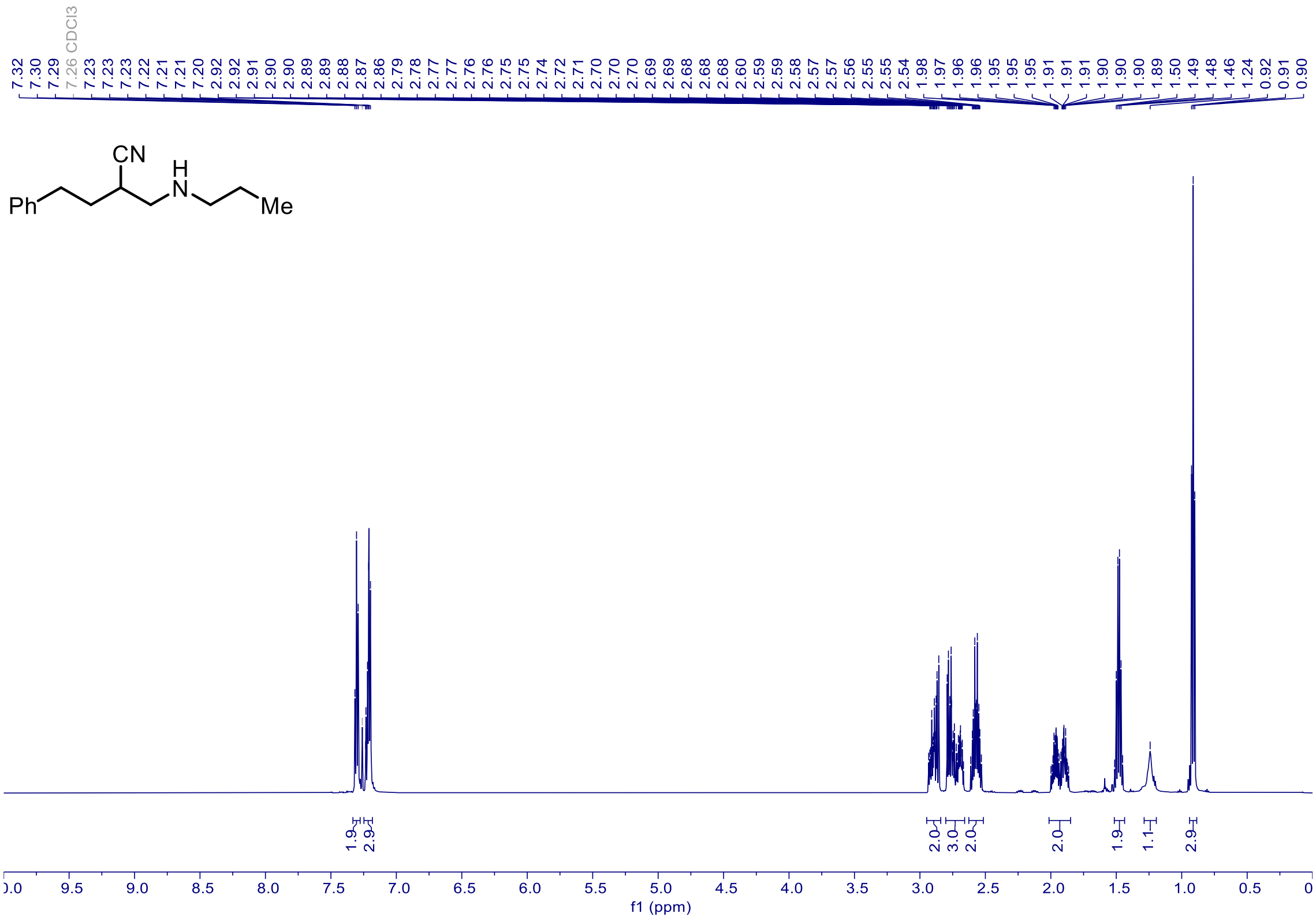

26b – ^{13}C NMR (151 MHz, $CDCl_3$)

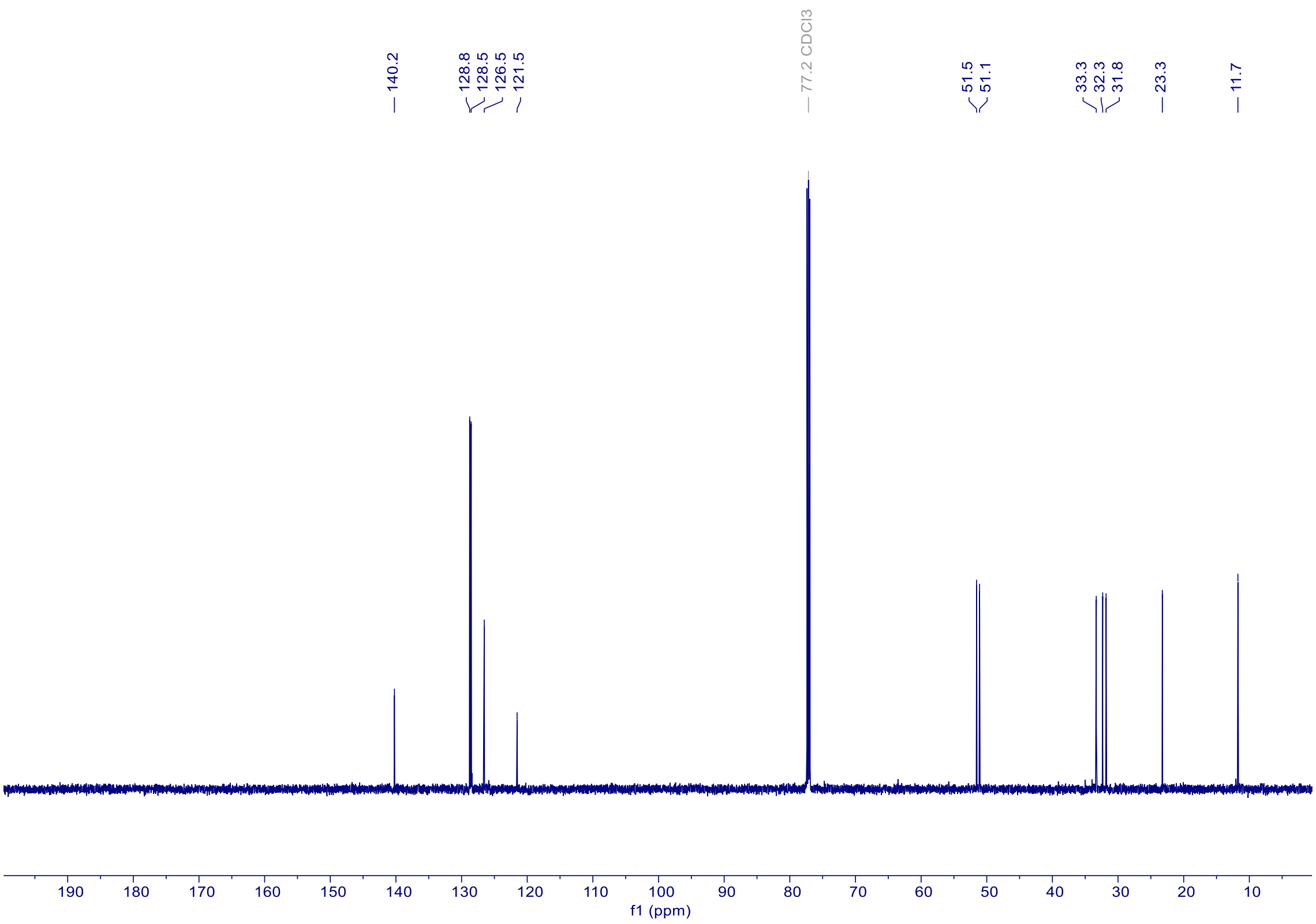

27b – ^{1}H NMR (400 MHz, $CDCl_3$)

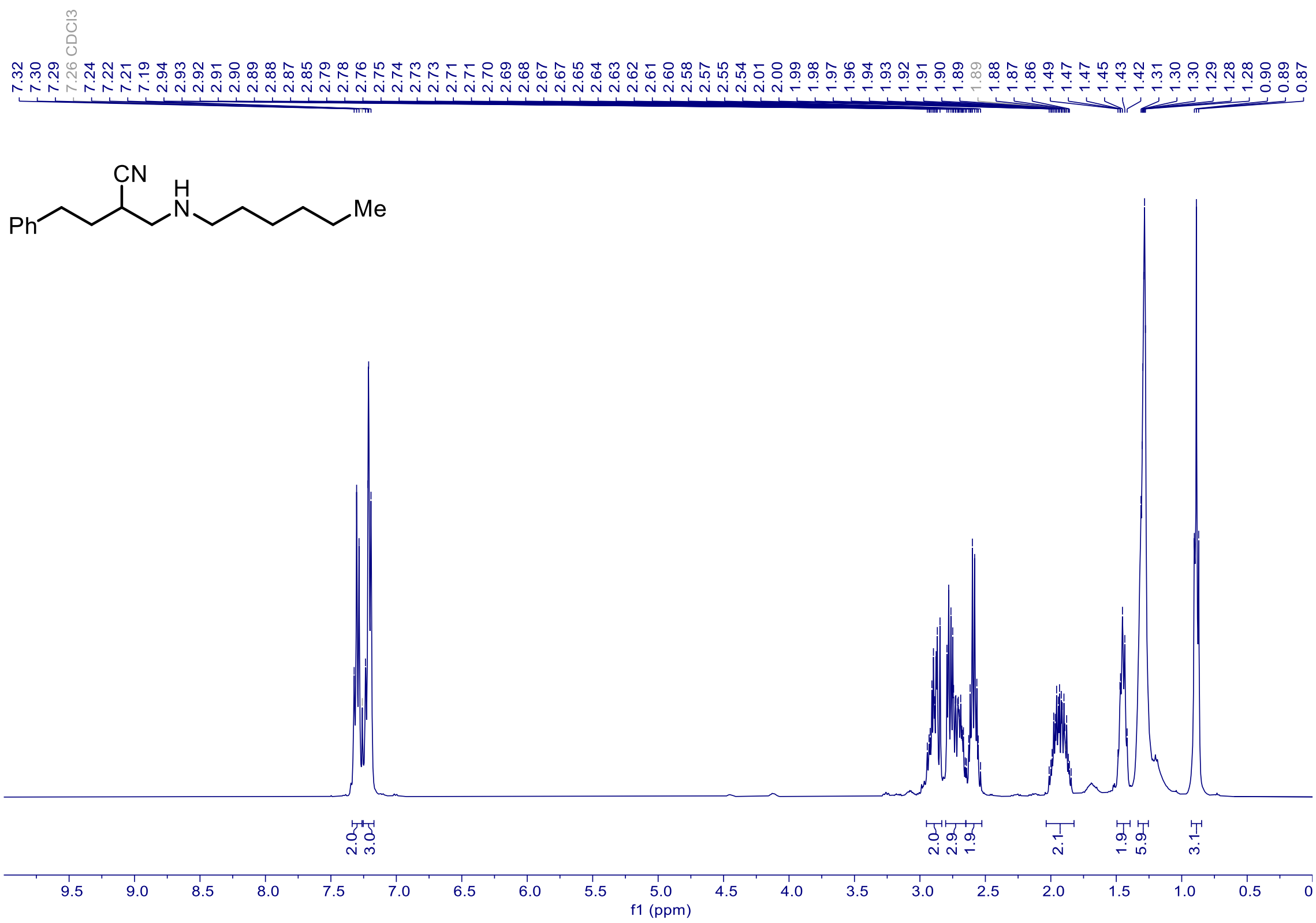

27b – ^{13}C NMR (101 MHz, $CDCl_3$)

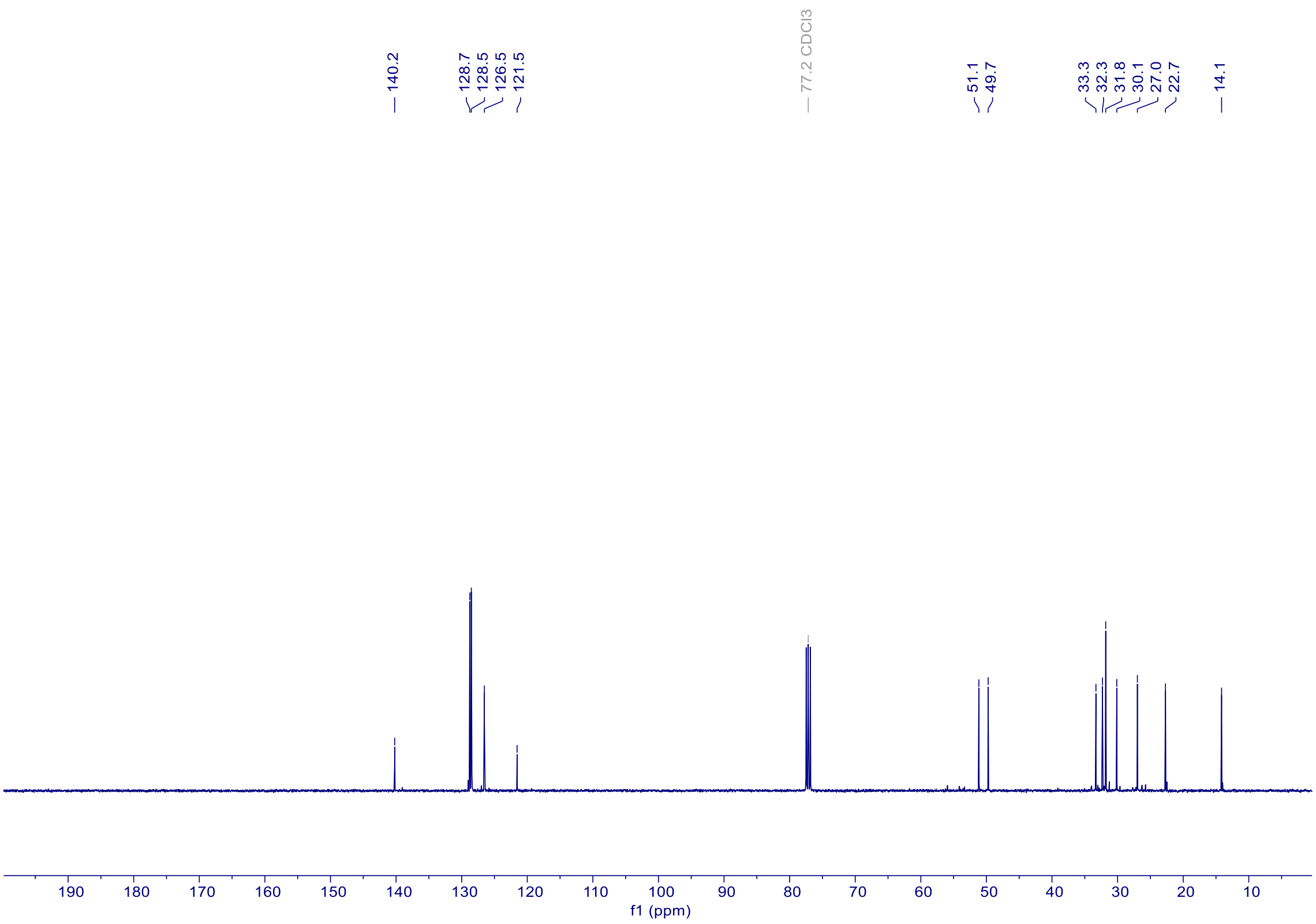

13c – ^{1}H NMR (600 MHz, $CDCl_3$)

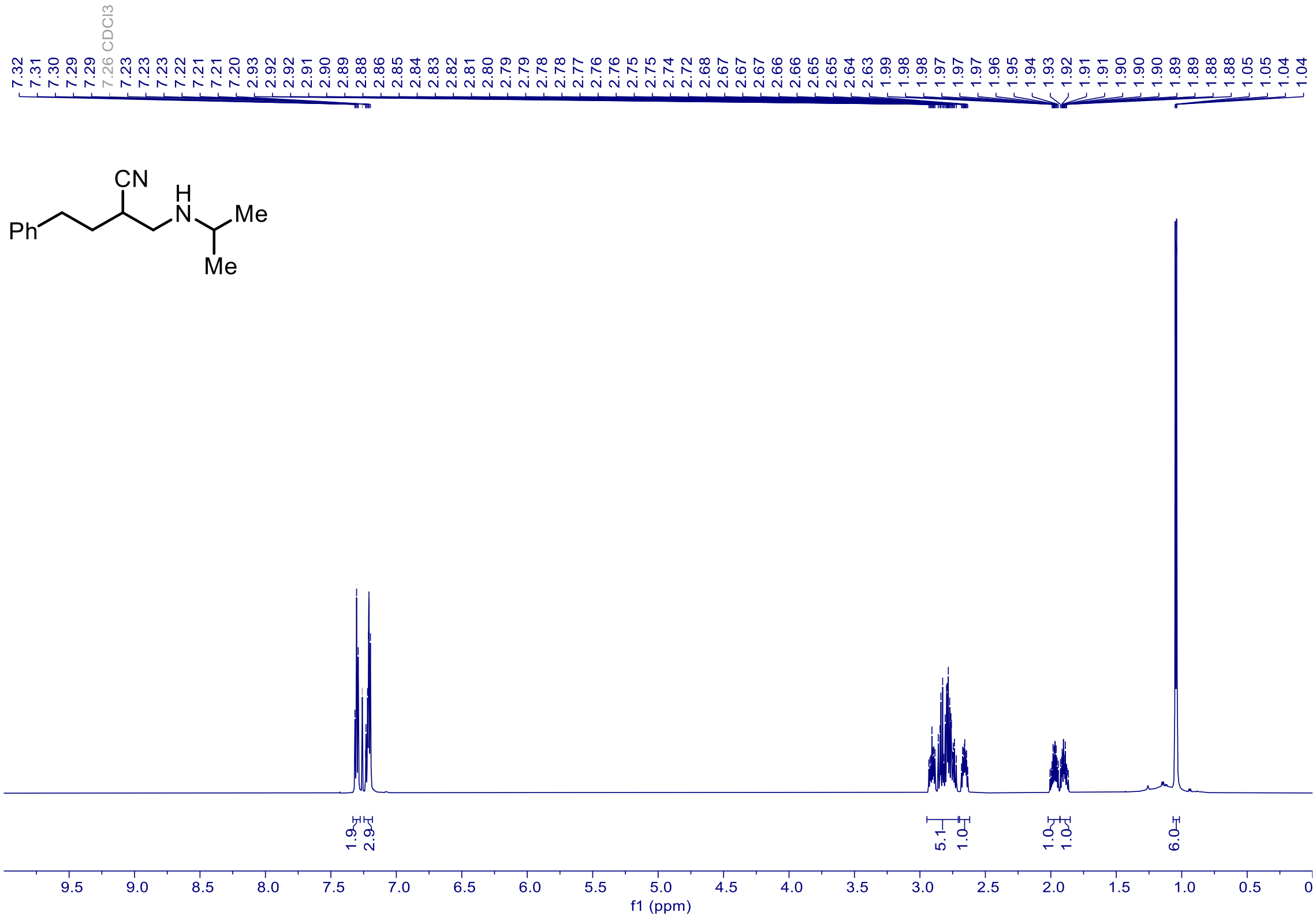

13c – ^{13}C NMR (151 MHz, $CDCl_3$)

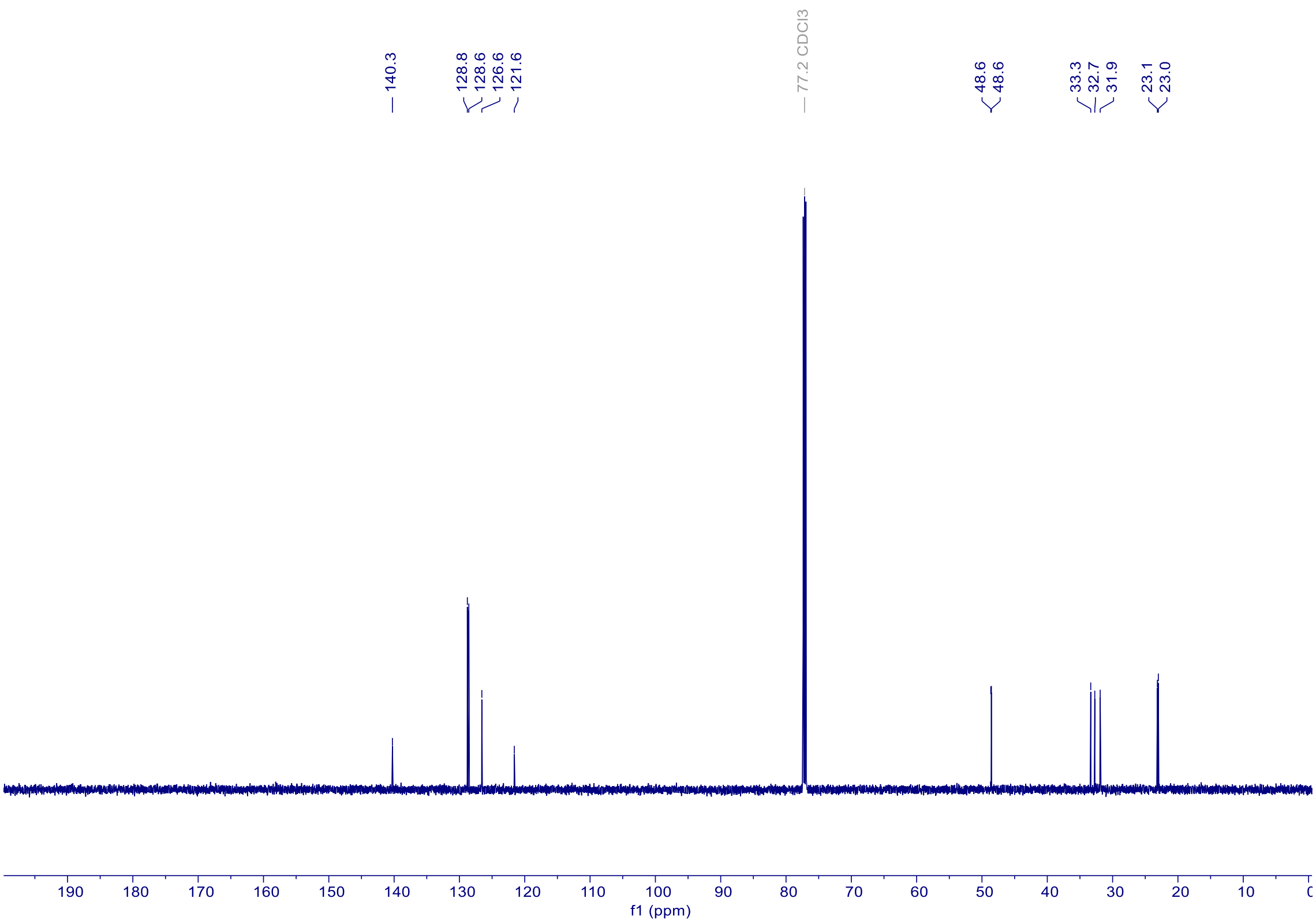

28b – ^{1}H NMR (600 MHz, $CDCl_3$)

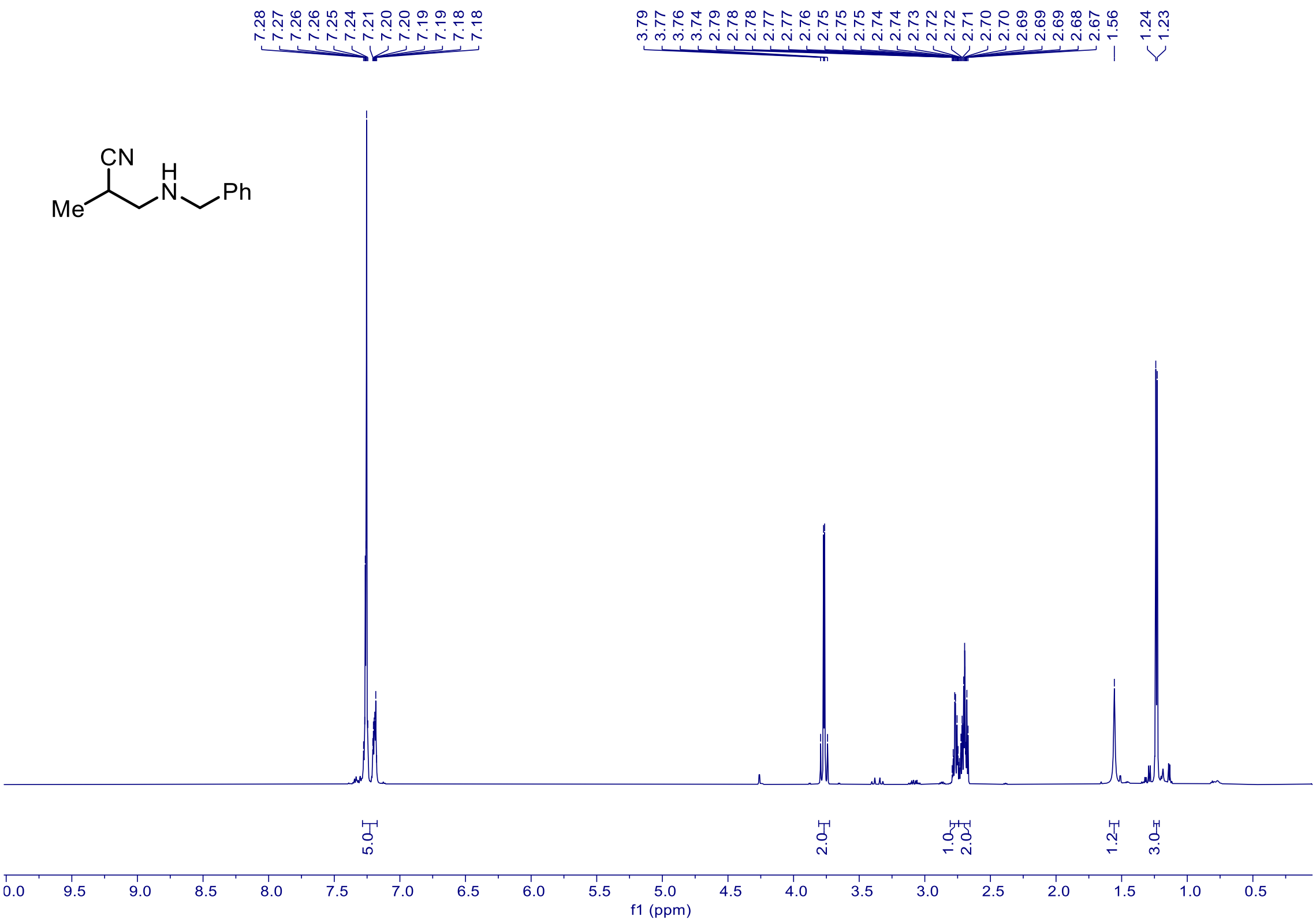

28b – ^{13}C NMR (151 MHz, $CDCl_3$)

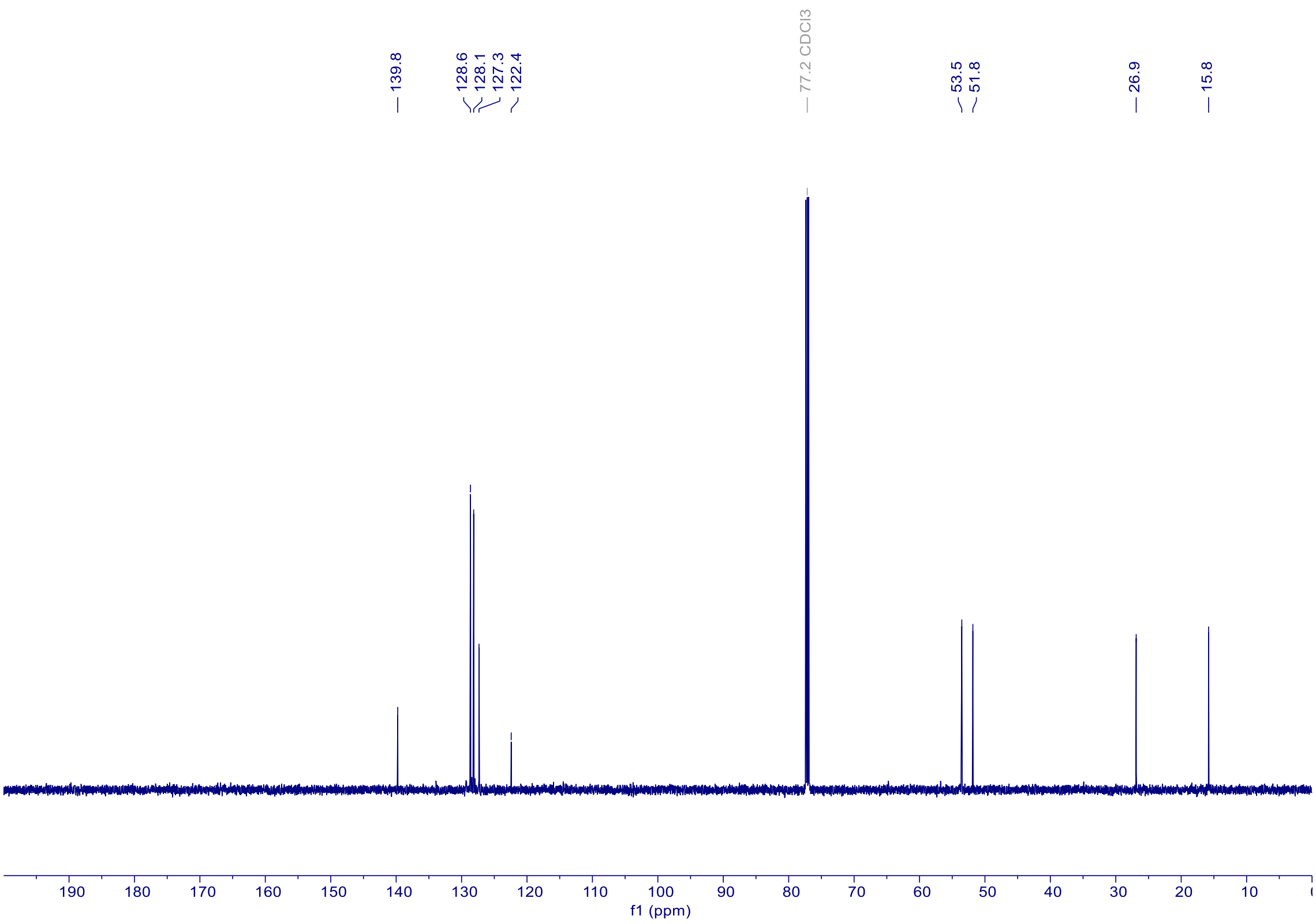

29b – ^{1}H NMR (600 MHz, $CDCl_3$)

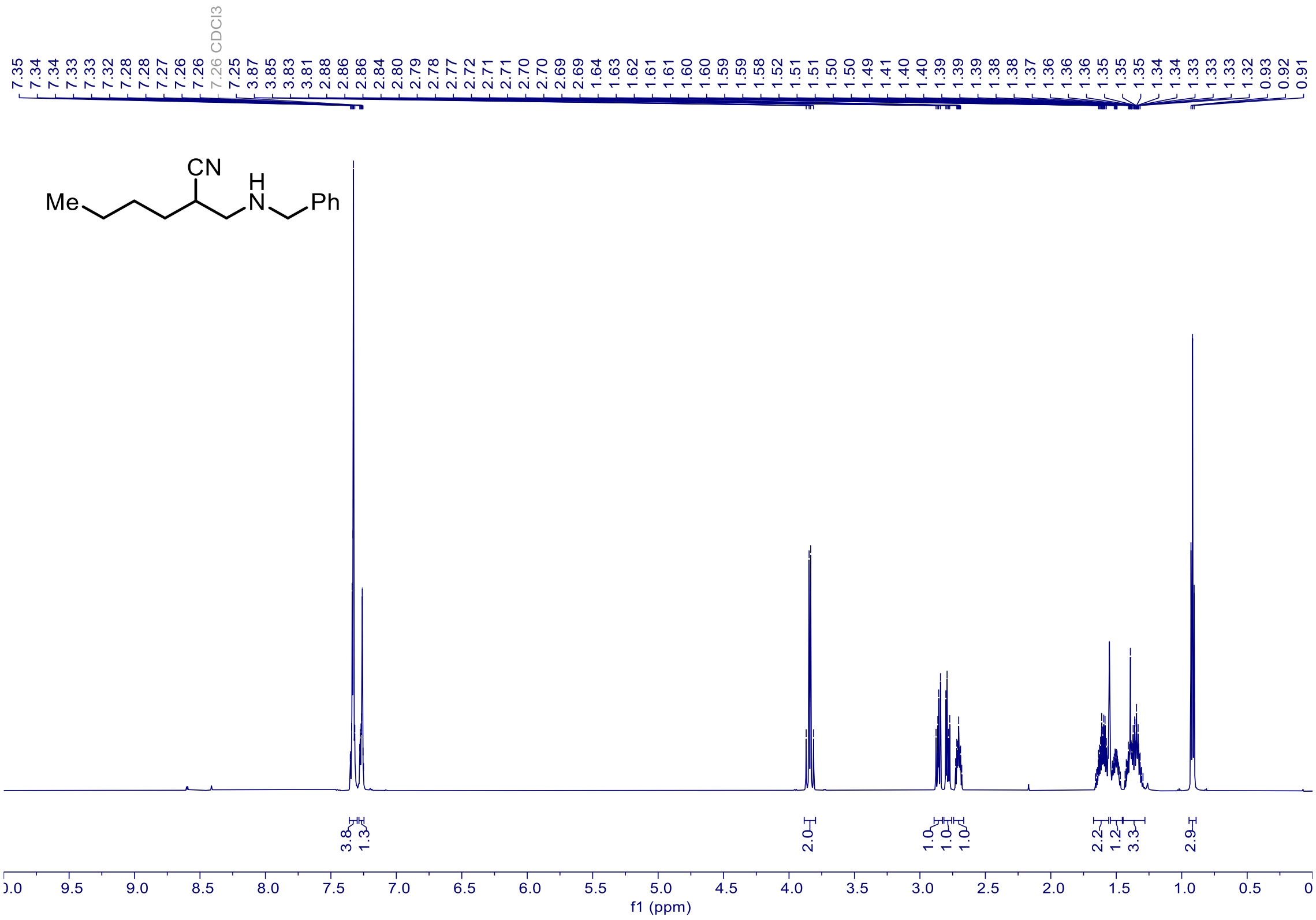

29b – ^{13}C NMR (151 MHz, $CDCl_3$)

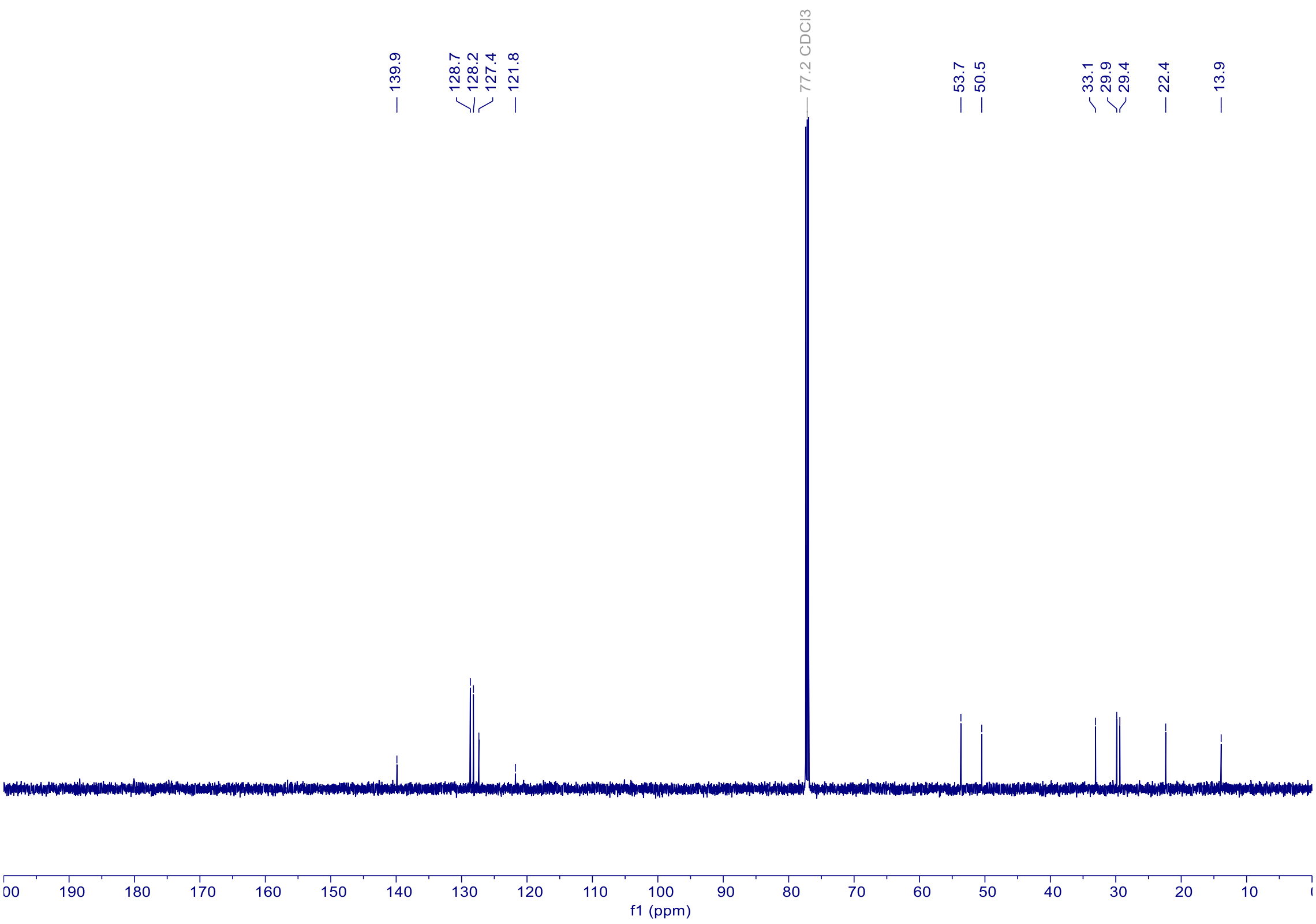

30b – ^{1}H NMR (600 MHz, $CDCl_3$)

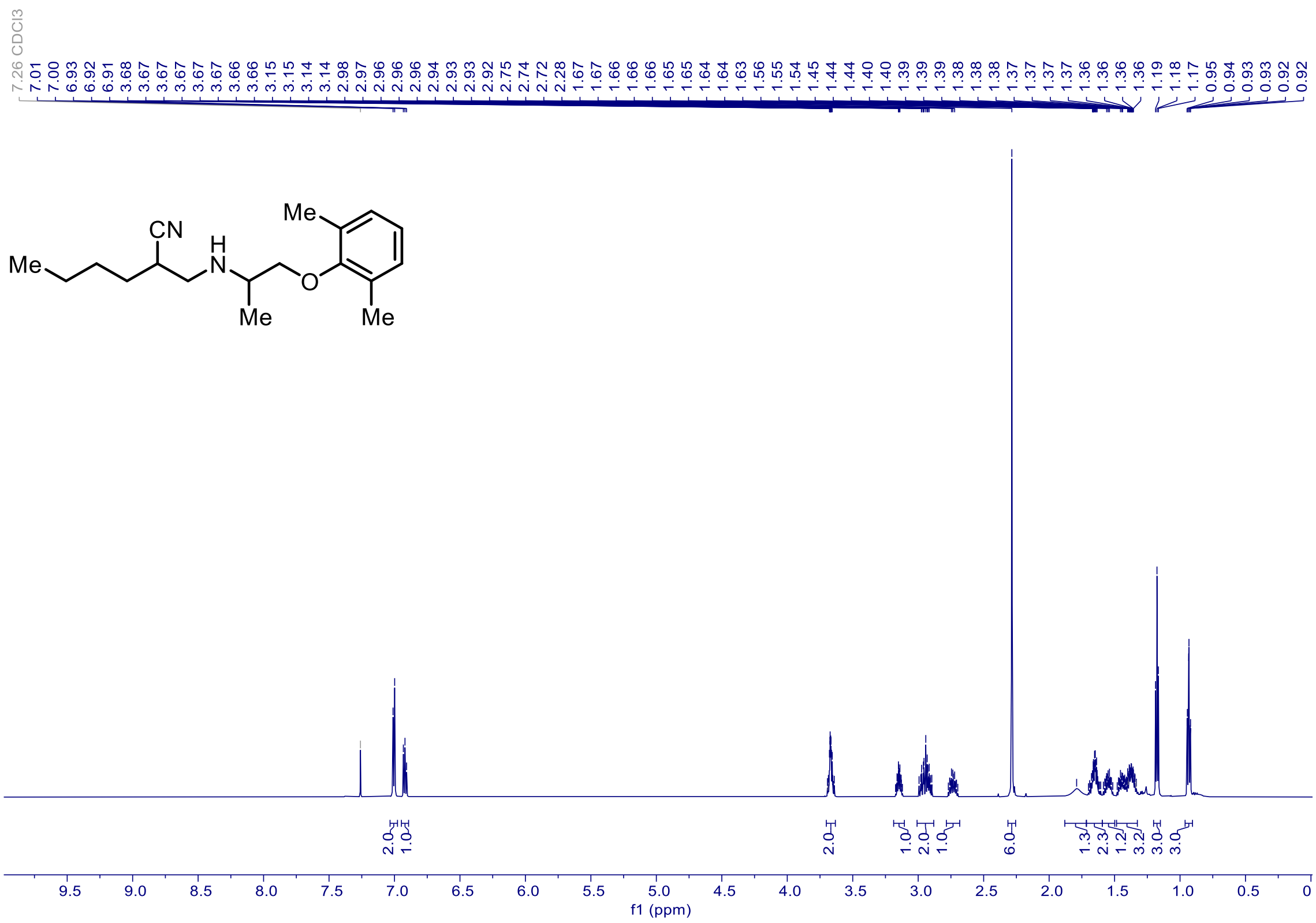

30b – ^{13}C NMR (151 MHz, $CDCl_3$)

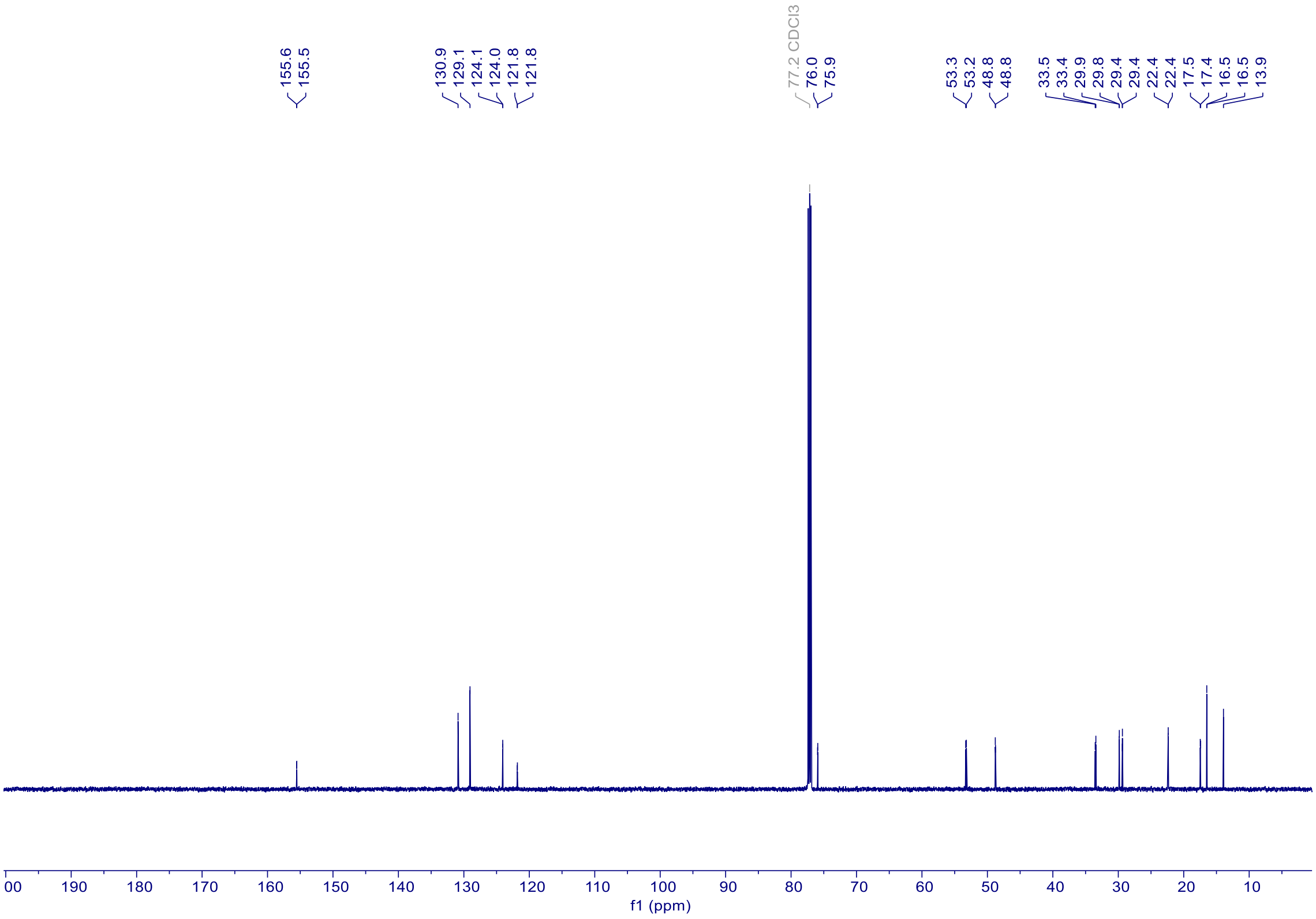

31b (*major diastereomer*) – ^{1}H NMR (600 MHz, $CDCl_3$)

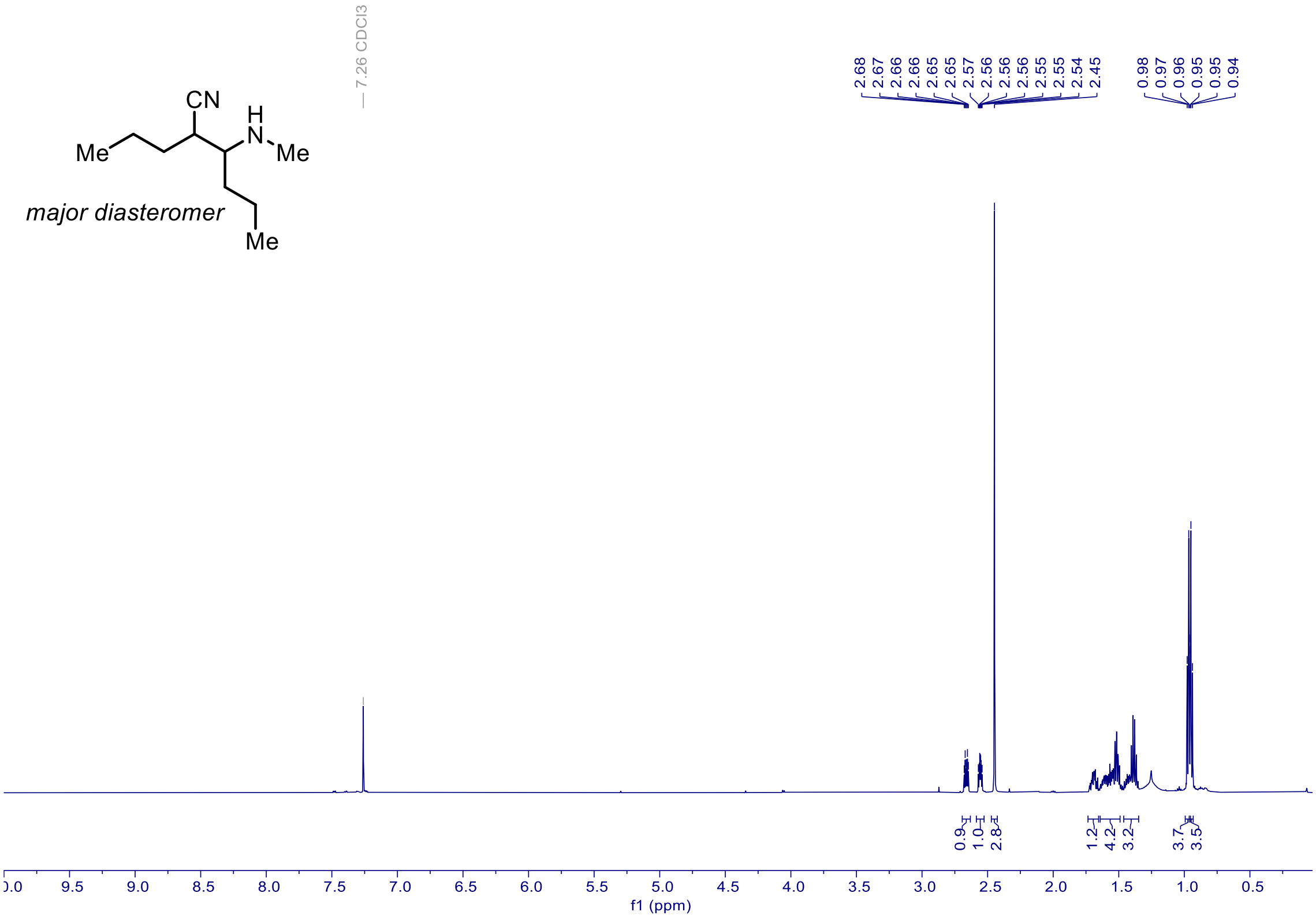

31b (*major diastereomer*) – ^{13}C NMR (151 MHz, $CDCl_3$)

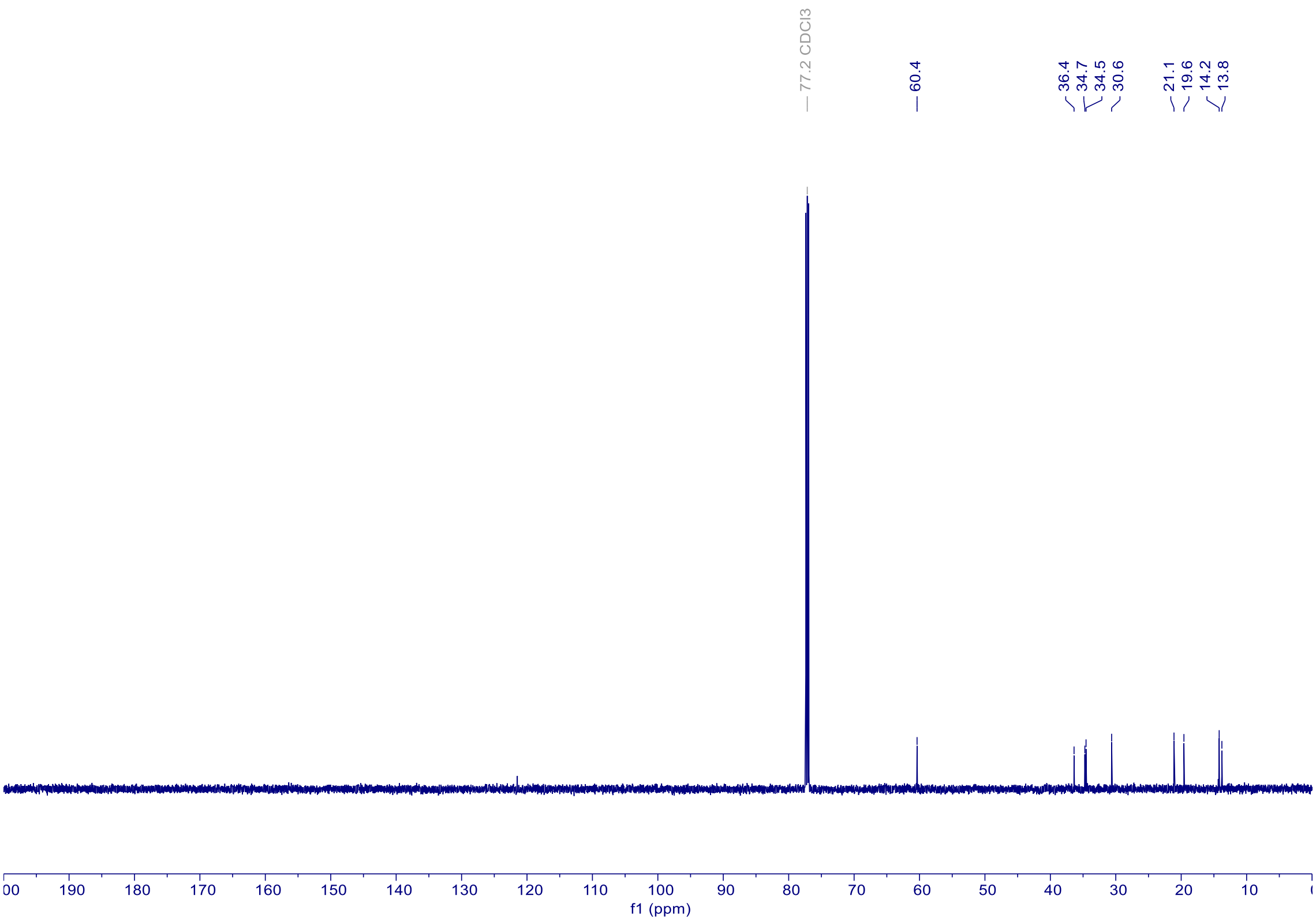

31b (*minor diastereomer*) – ^{1}H NMR (400 MHz, CDCl$_3$)

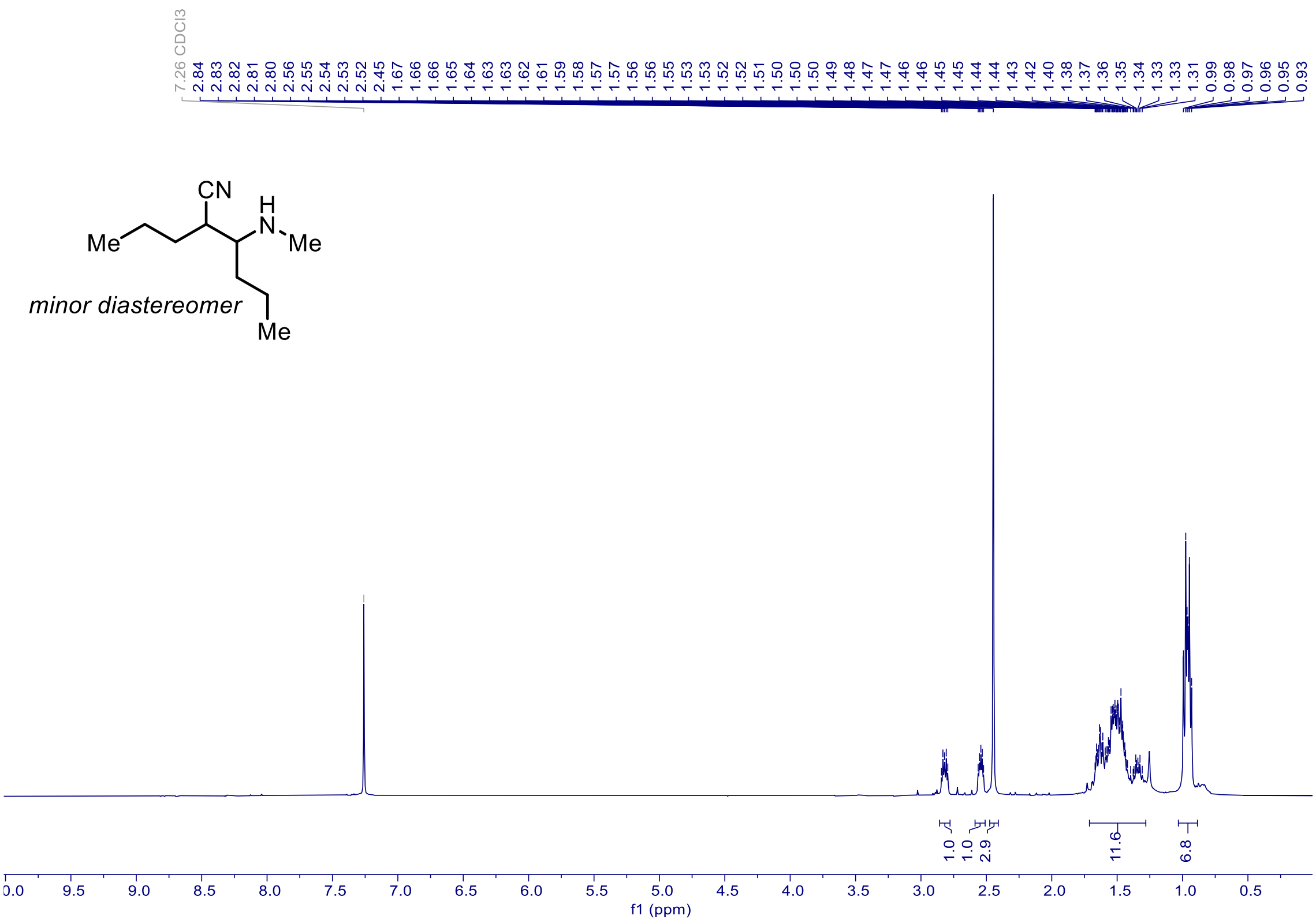

31b (*minor diastereomer*) – ^{13}C NMR (101 MHz, CDCl$_3$)

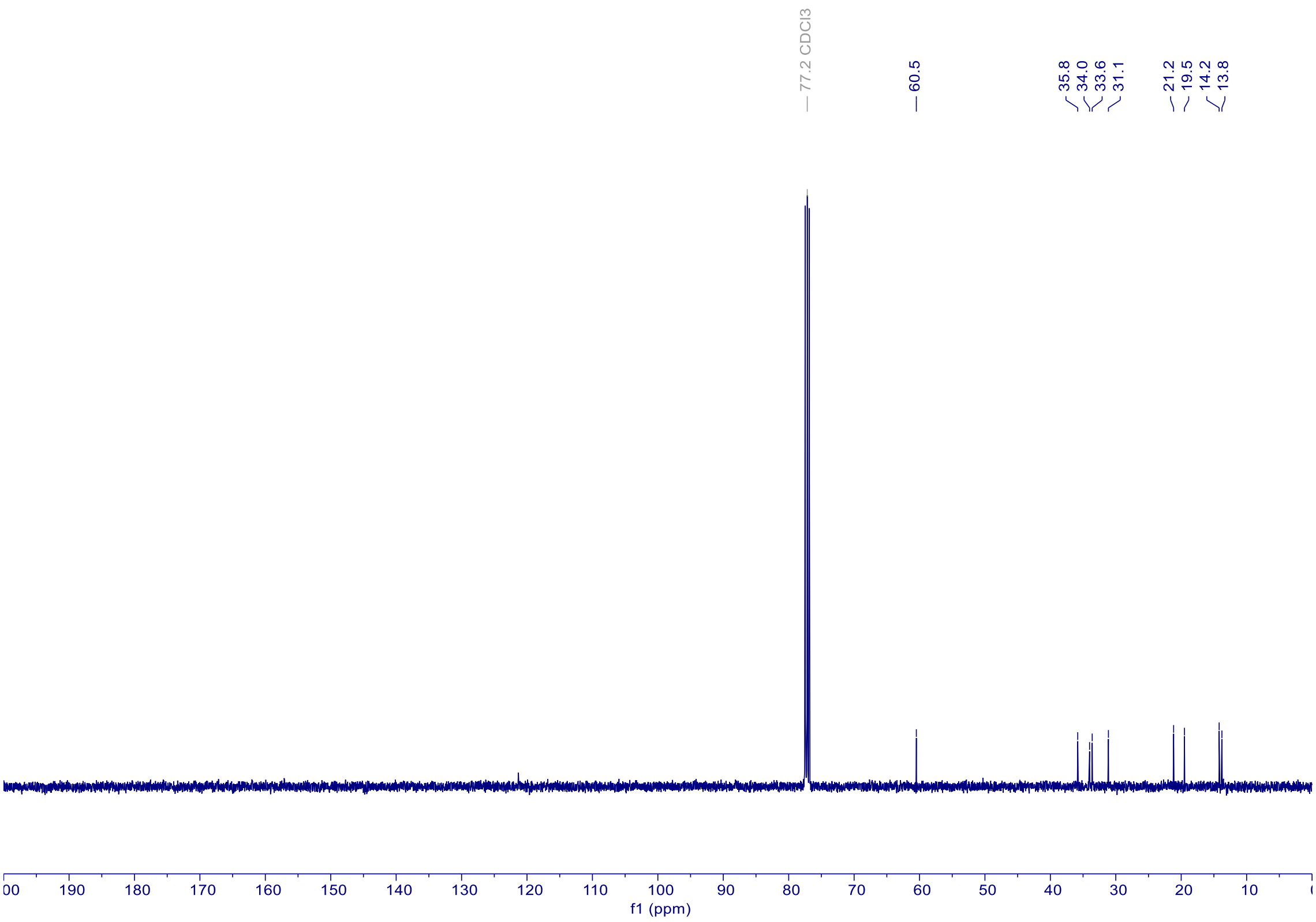

32b (*major diastereomer*) – ^{1}H NMR (600 MHz, $CDCl_3$)

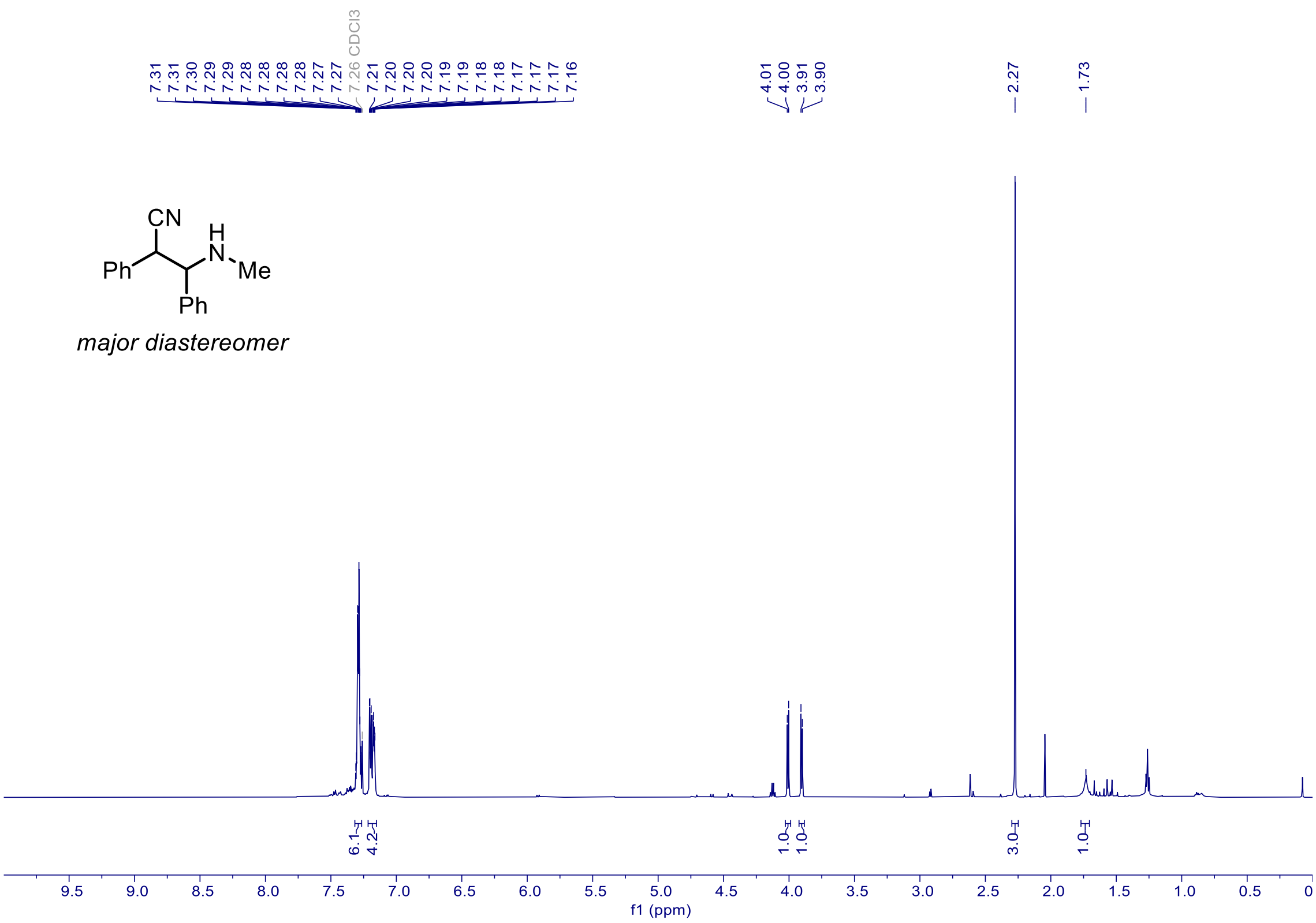

32b (*major diastereomer*) – ^{13}C NMR (151 MHz, $CDCl_3$)

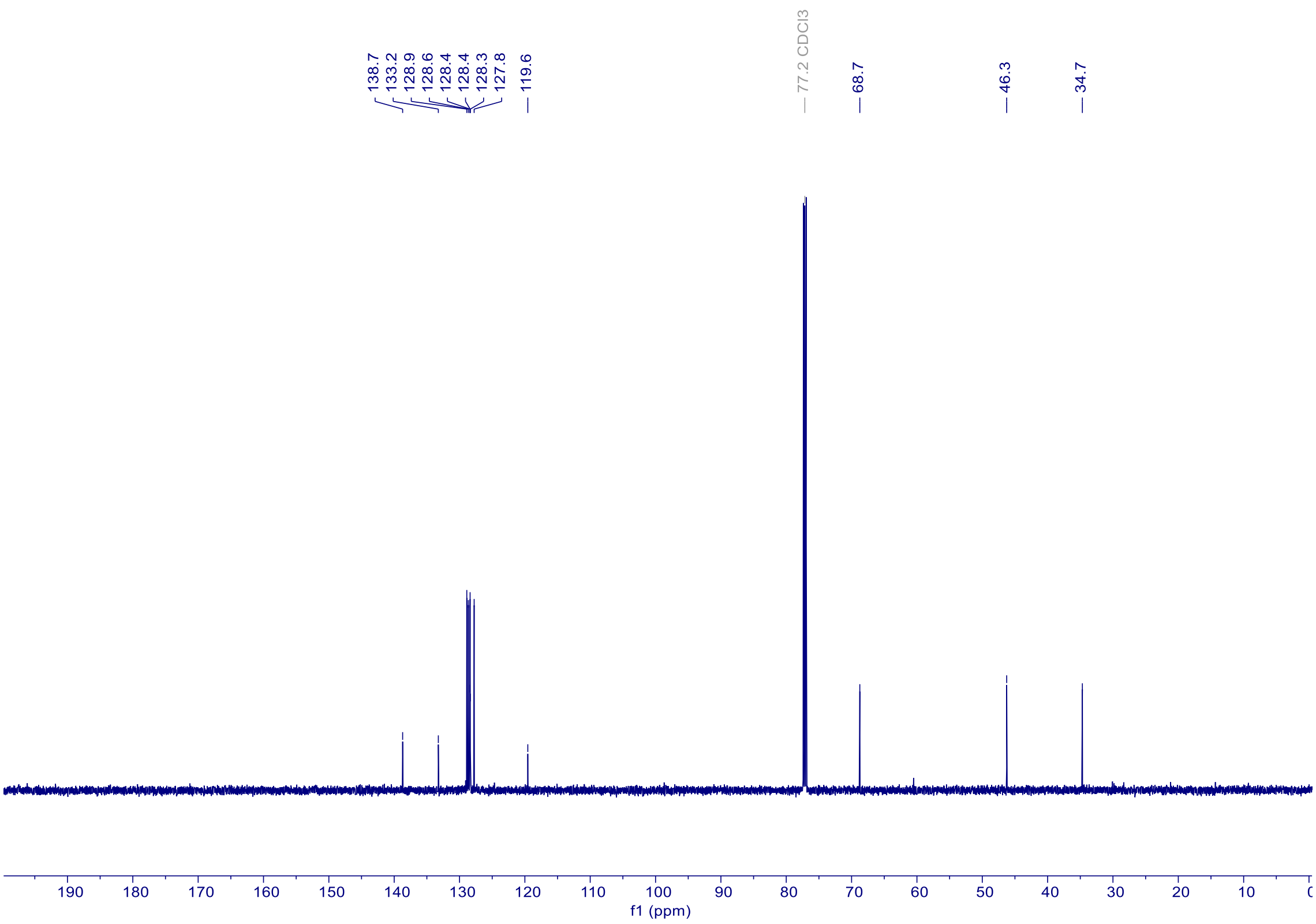

32b (*minor diastereomer*) – ^{1}H NMR (600 MHz, $CDCl_3$)

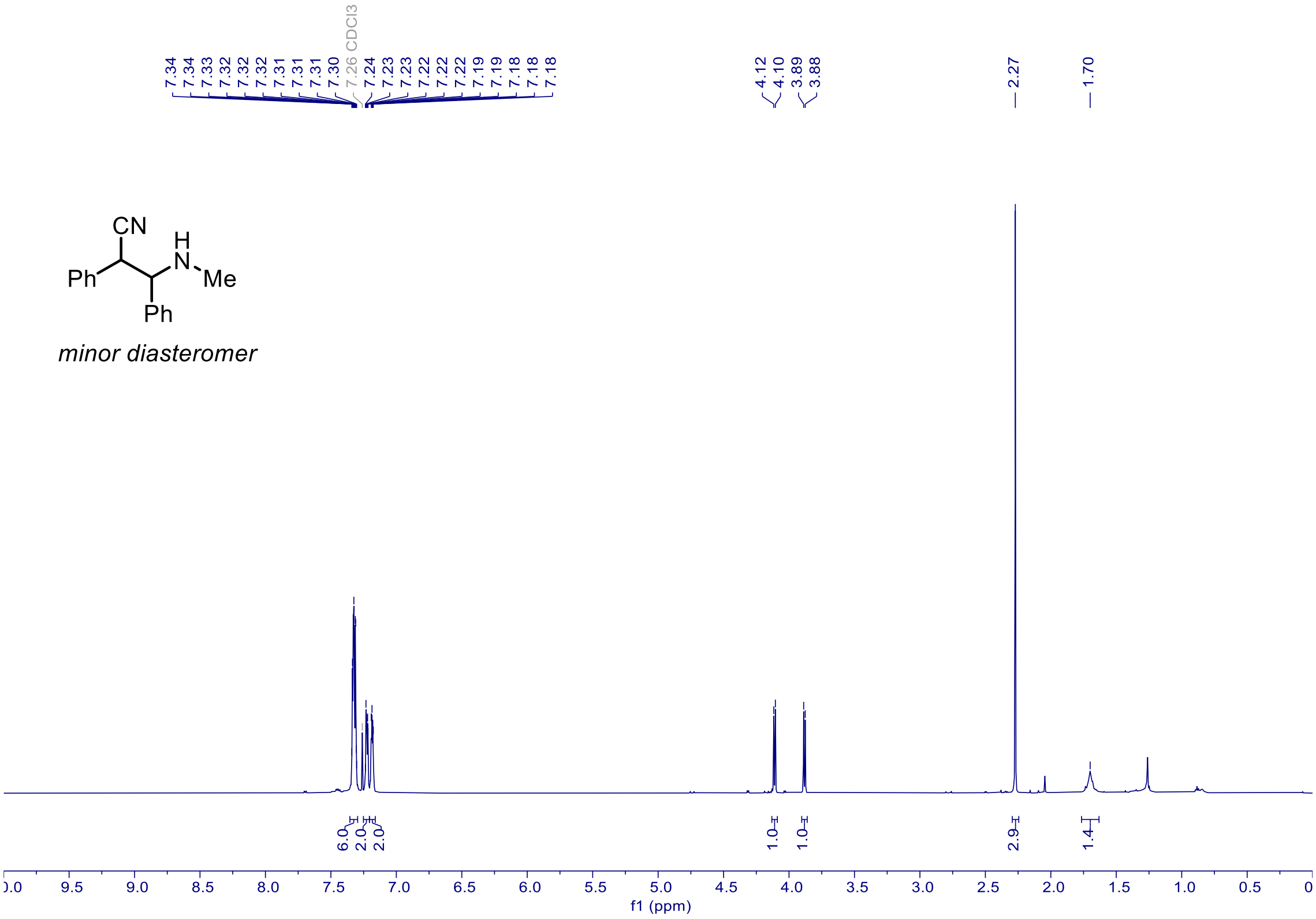

32b (*minor diastereomer*) – ^{13}C NMR (151 MHz, $CDCl_3$)

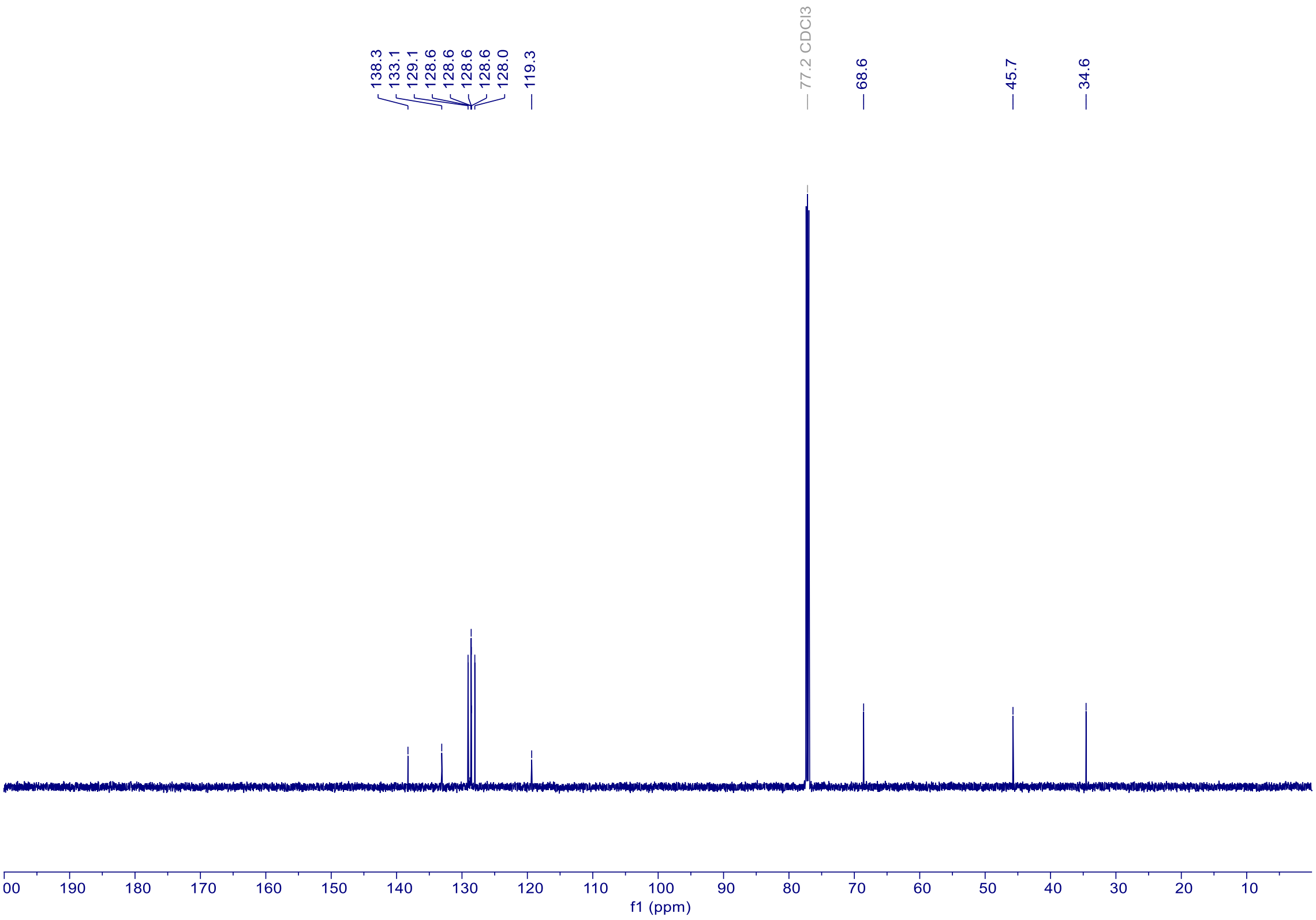

33b – ^{1}H NMR (400 MHz, $CDCl_3$)

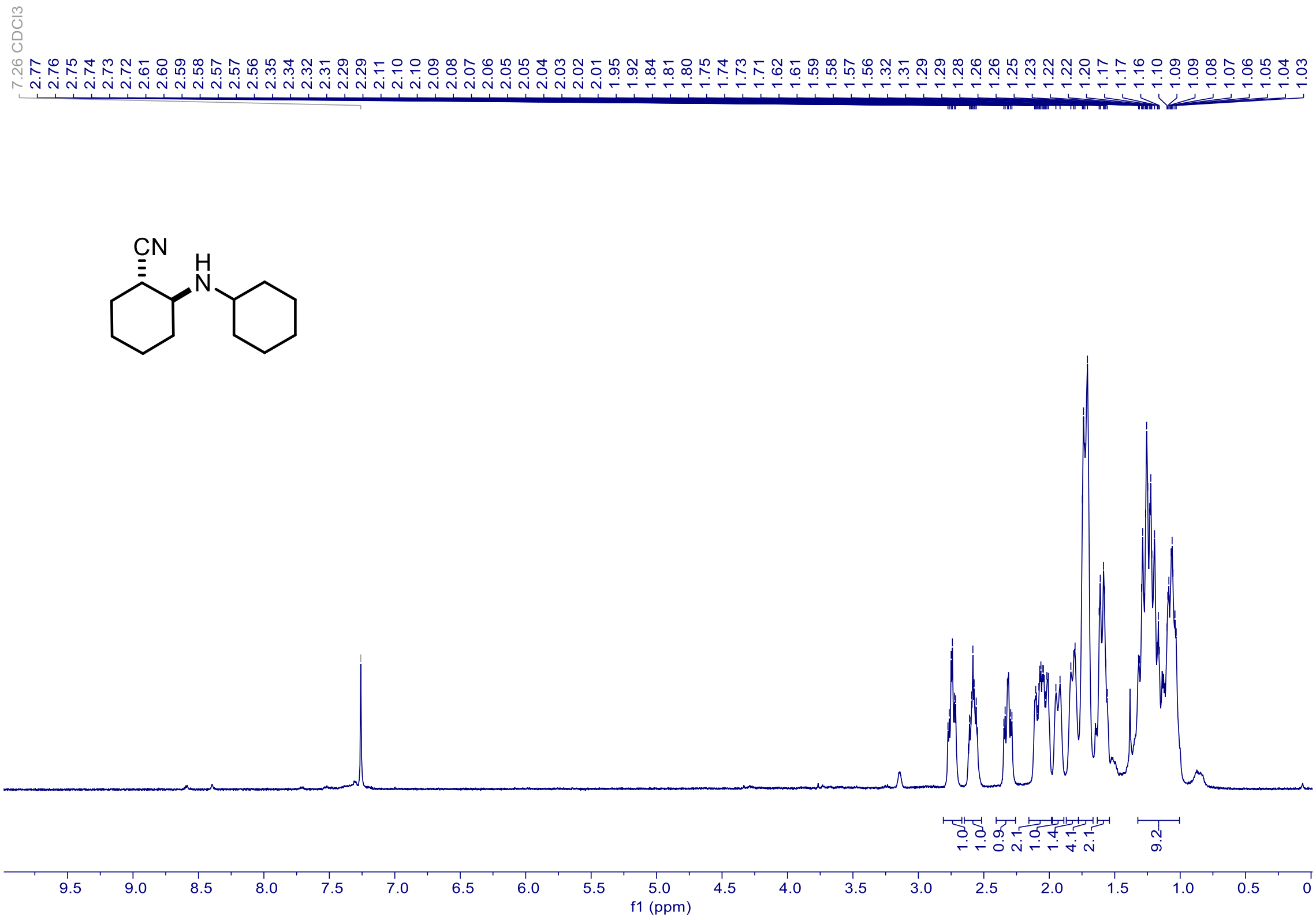

33b – ^{13}C NMR (151 MHz, $CDCl_3$)

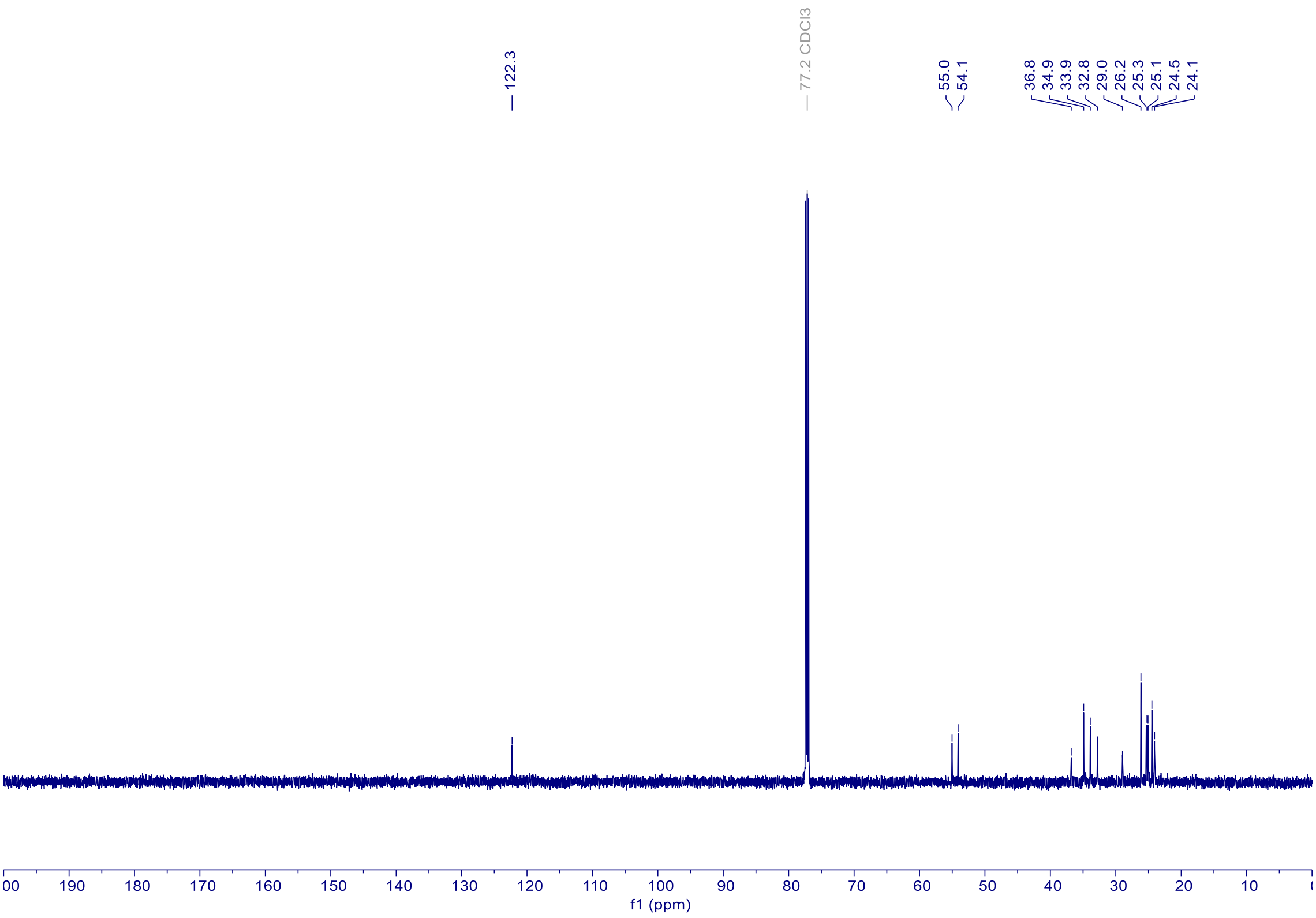

34b – ^{1}H NMR (400 MHz, $CDCl_3$)

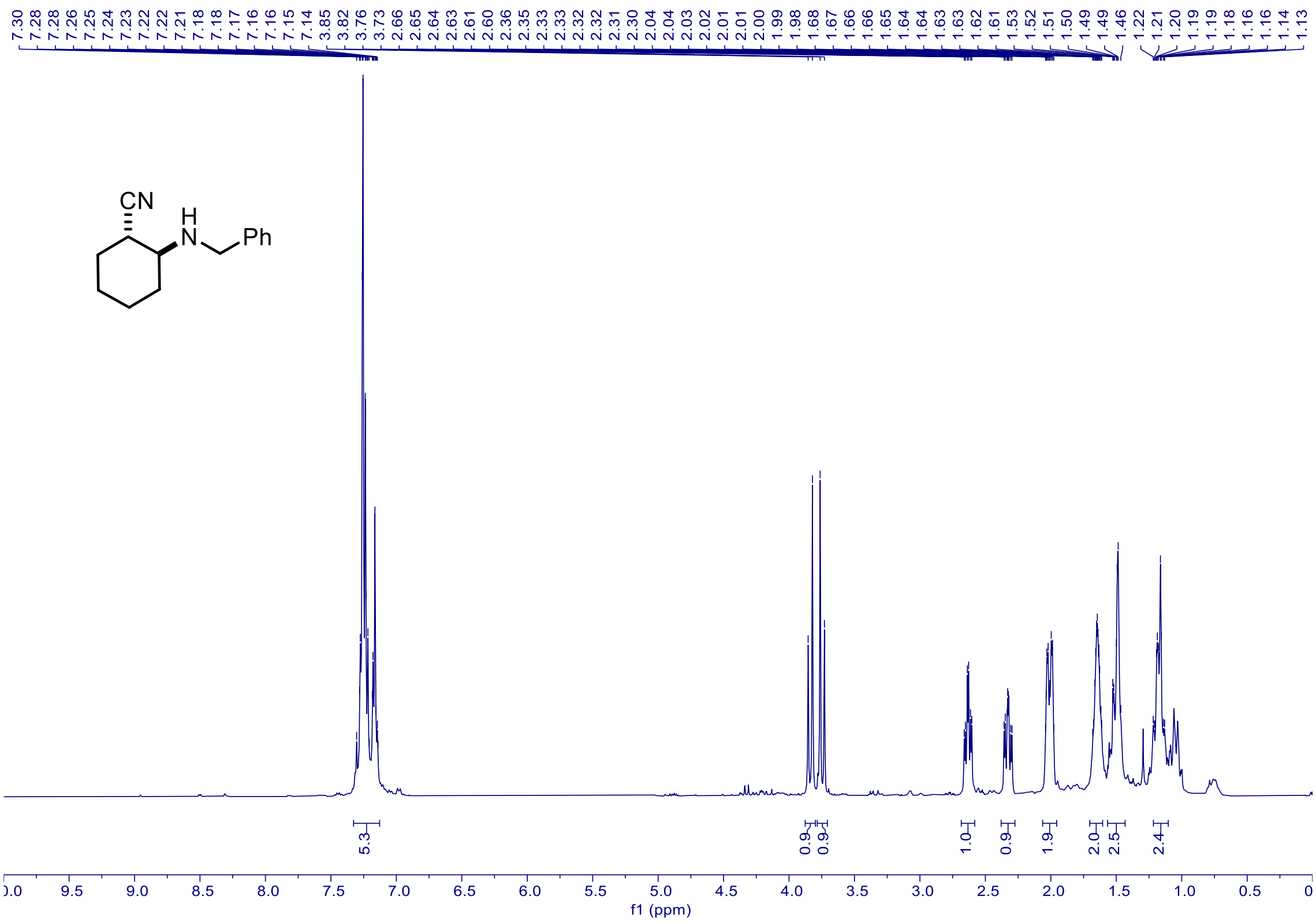

34b – ^{13}C NMR (101 MHz, $CDCl_3$)

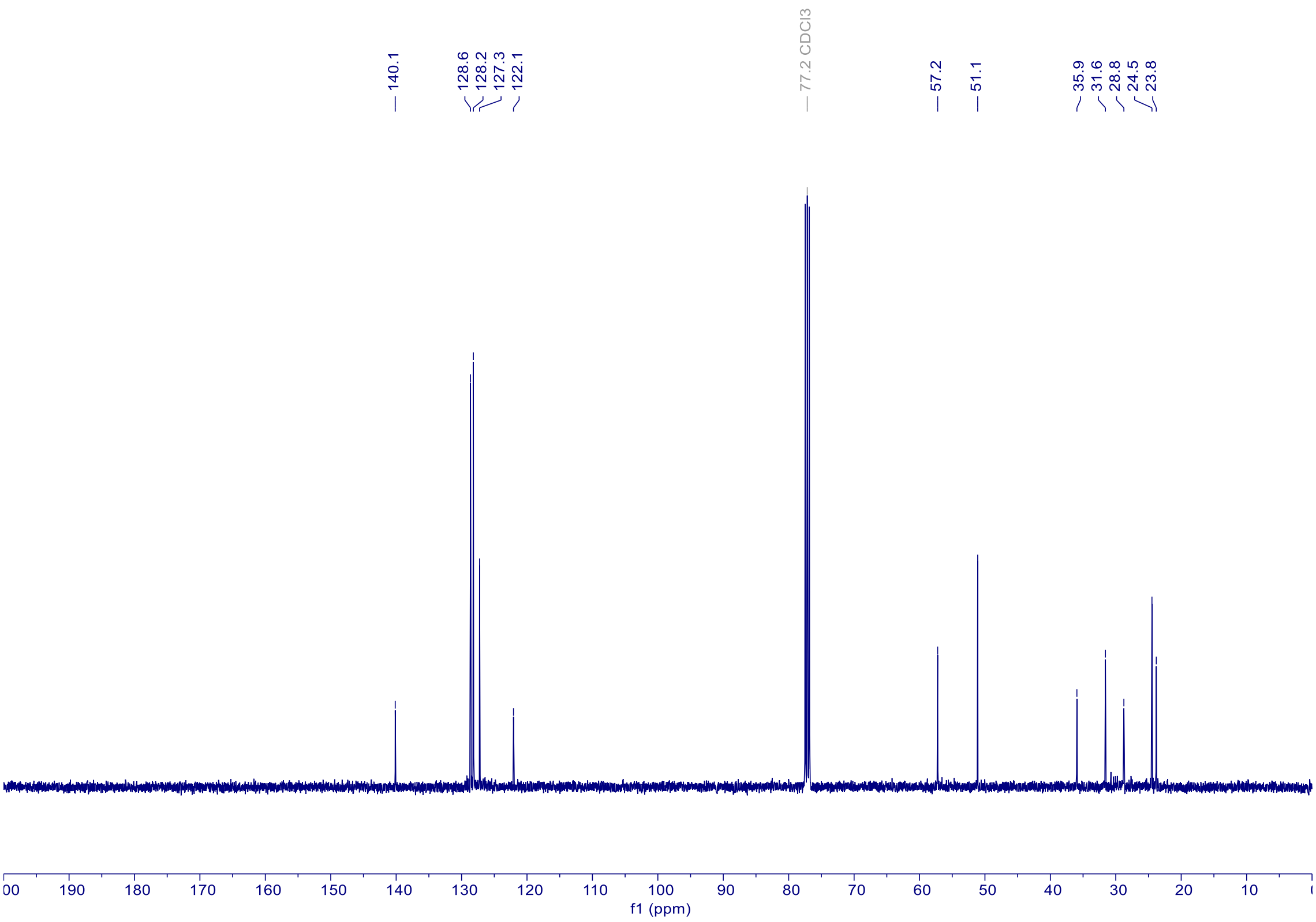

35b – ^{1}H NMR (400 MHz, CDCl$_3$)

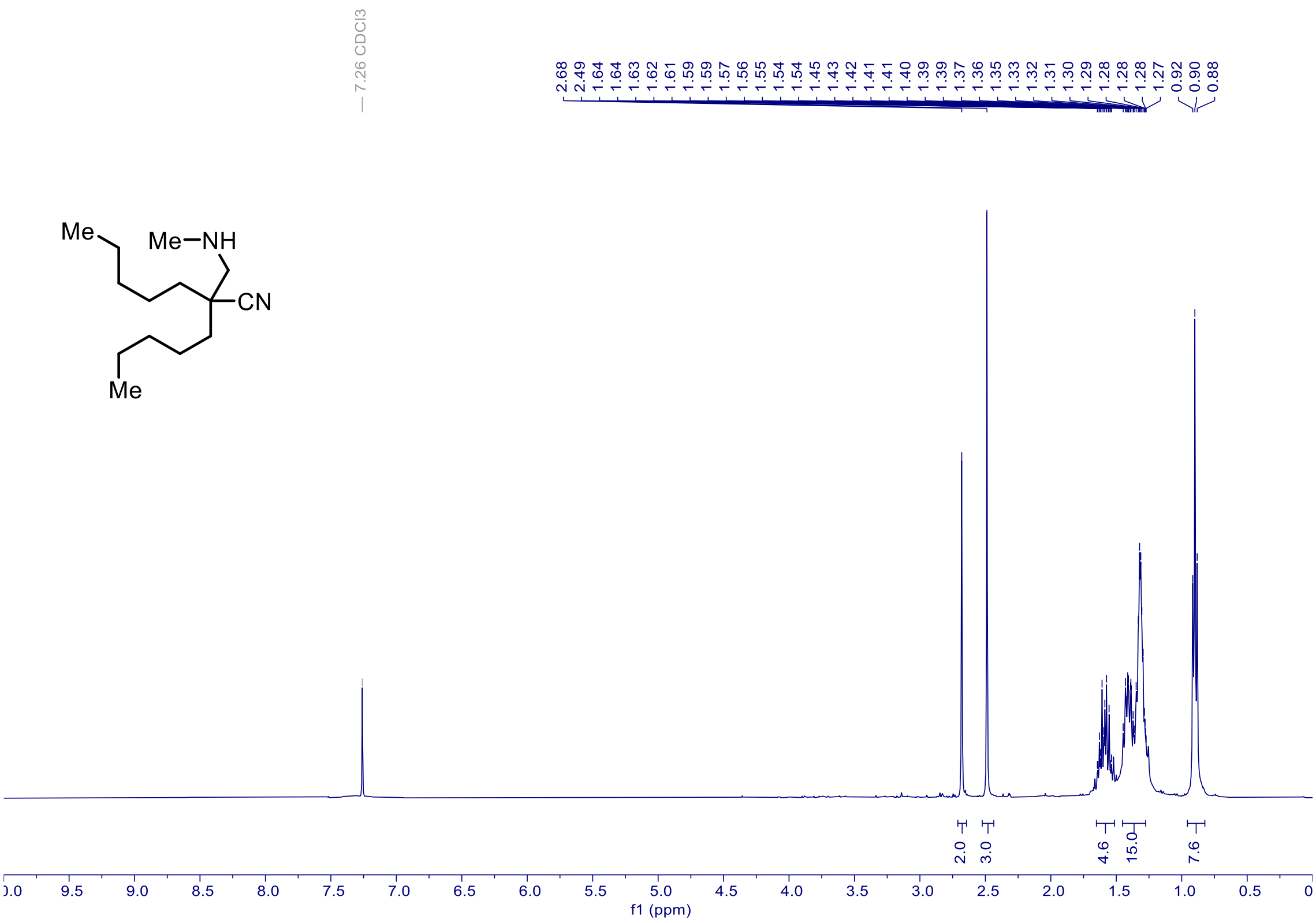

35b – ^{13}C NMR (101 MHz, CDCl$_3$)

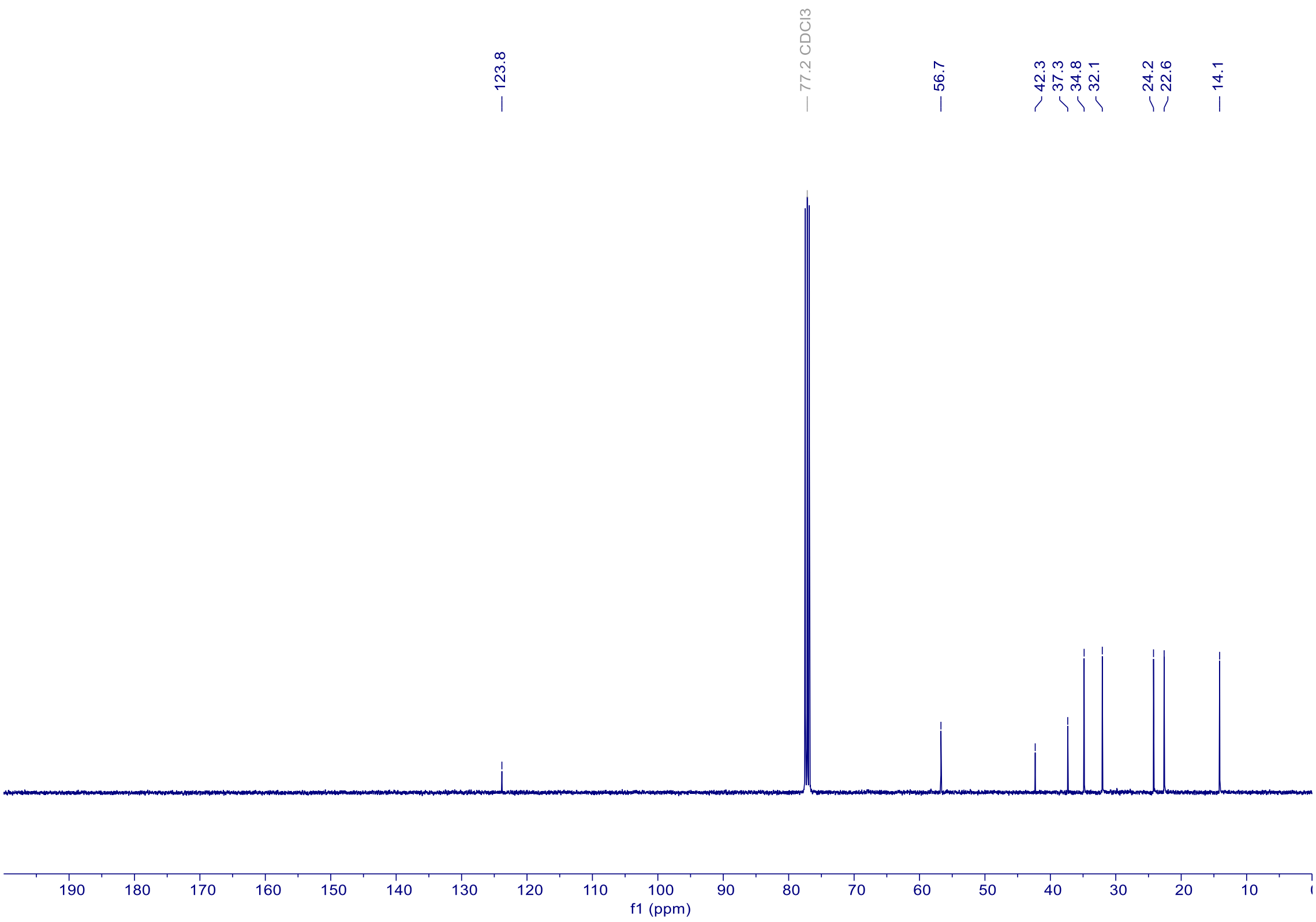

36b – ^{1}H NMR (400 MHz, $CDCl_3$)

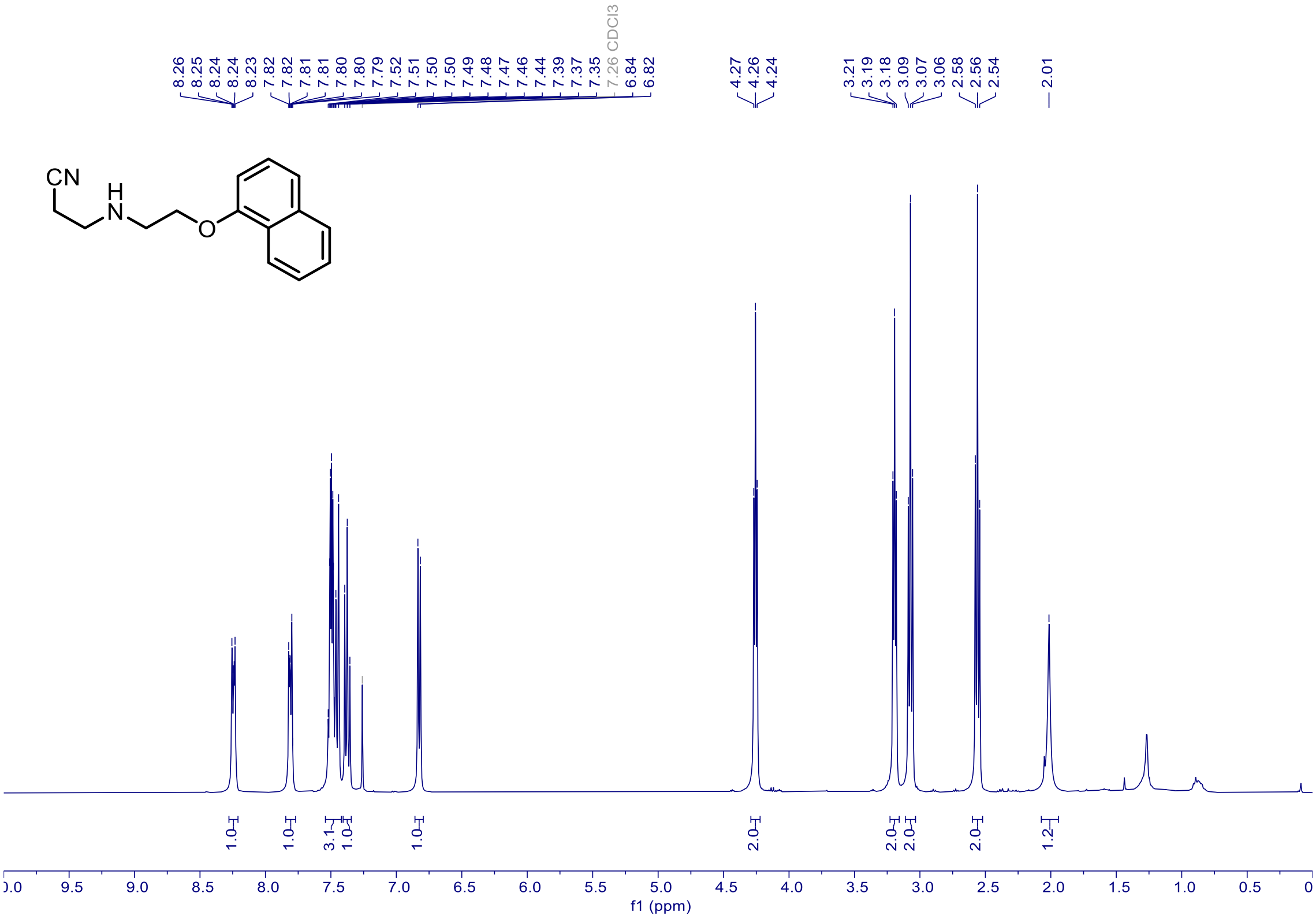

36b – ^{13}C NMR (101 MHz, $CDCl_3$)

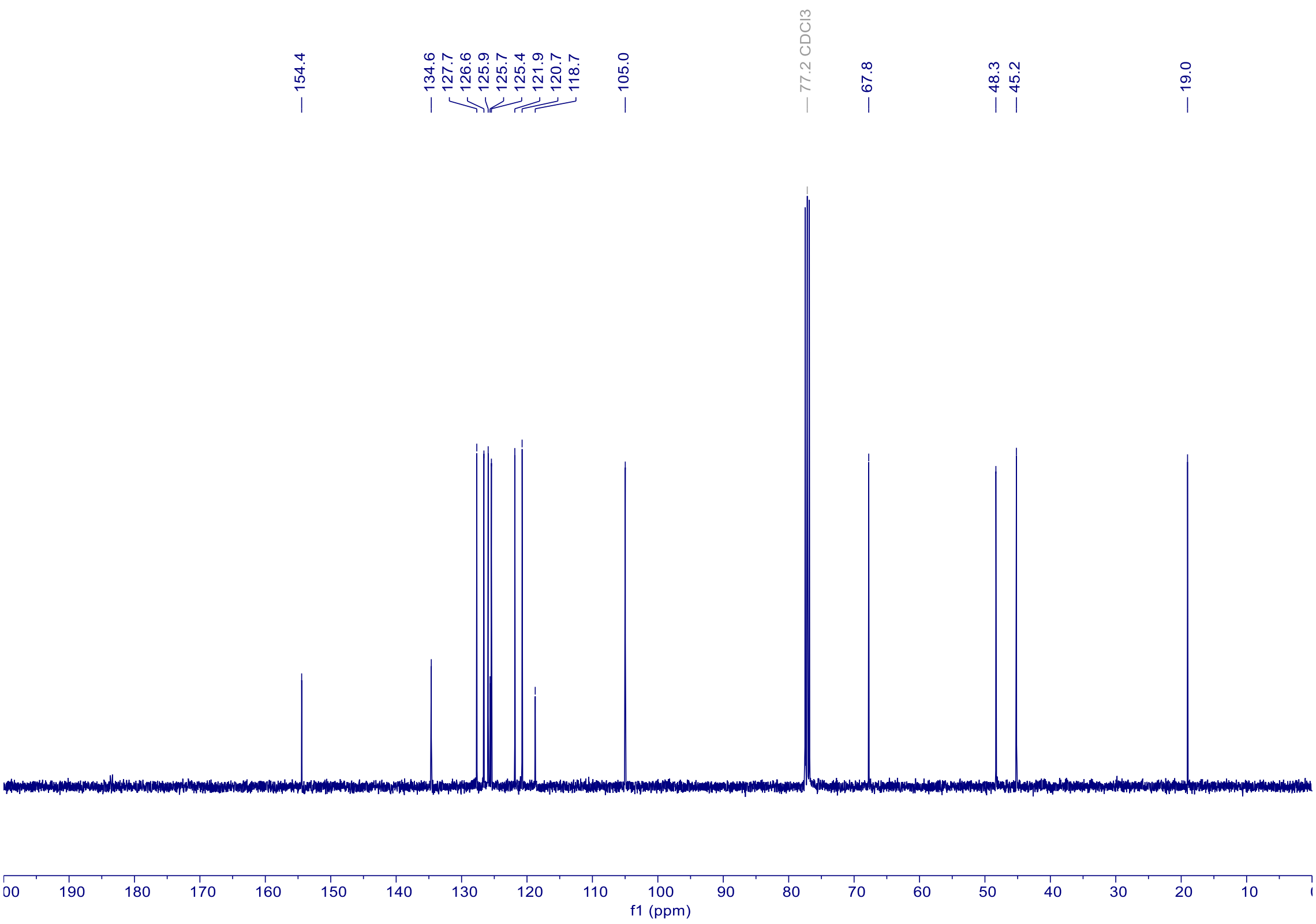

1r – ^{1}H NMR (600 MHz, $CDCl_3$)

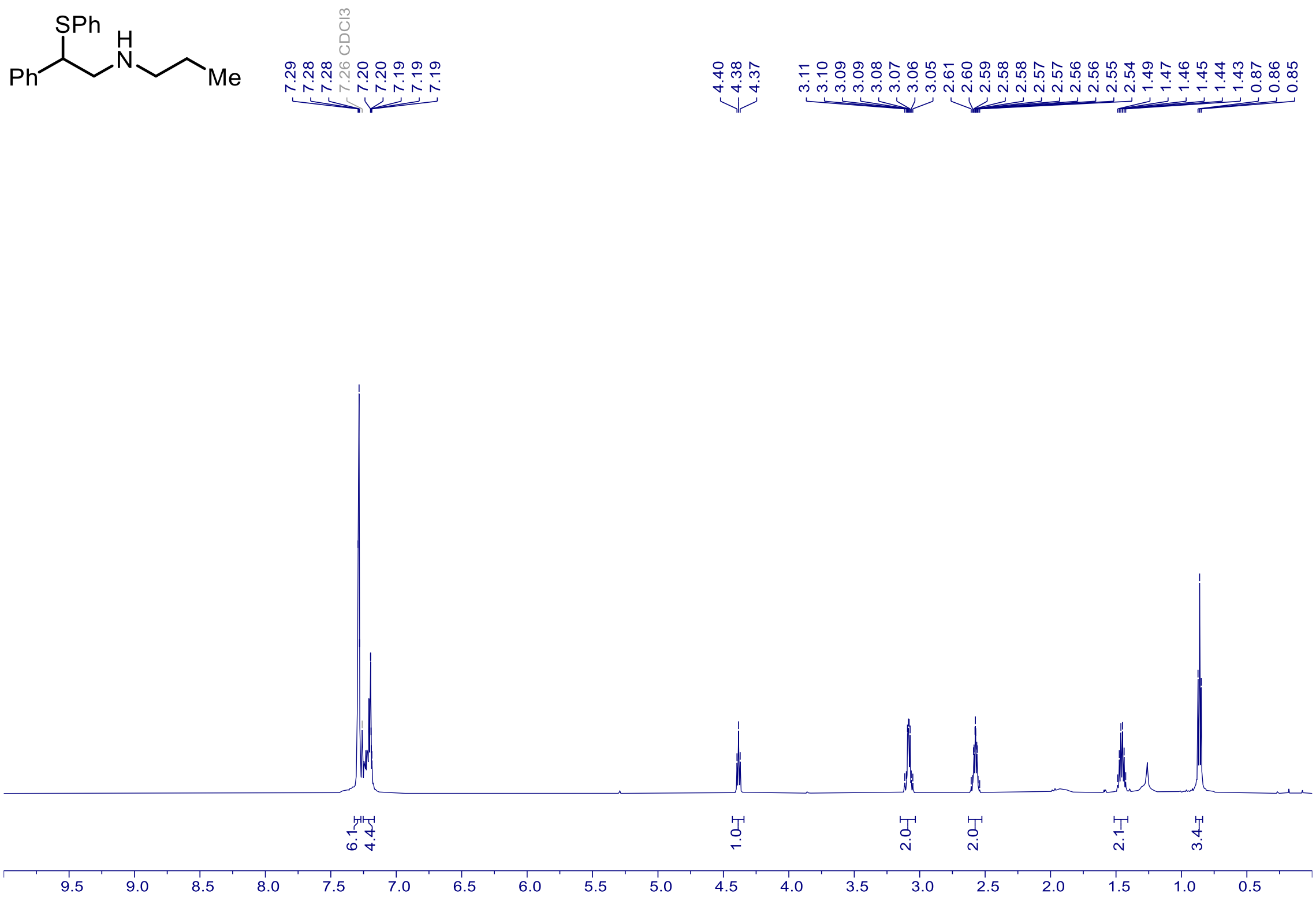

1r – ^{13}C NMR (151 MHz, $CDCl_3$)

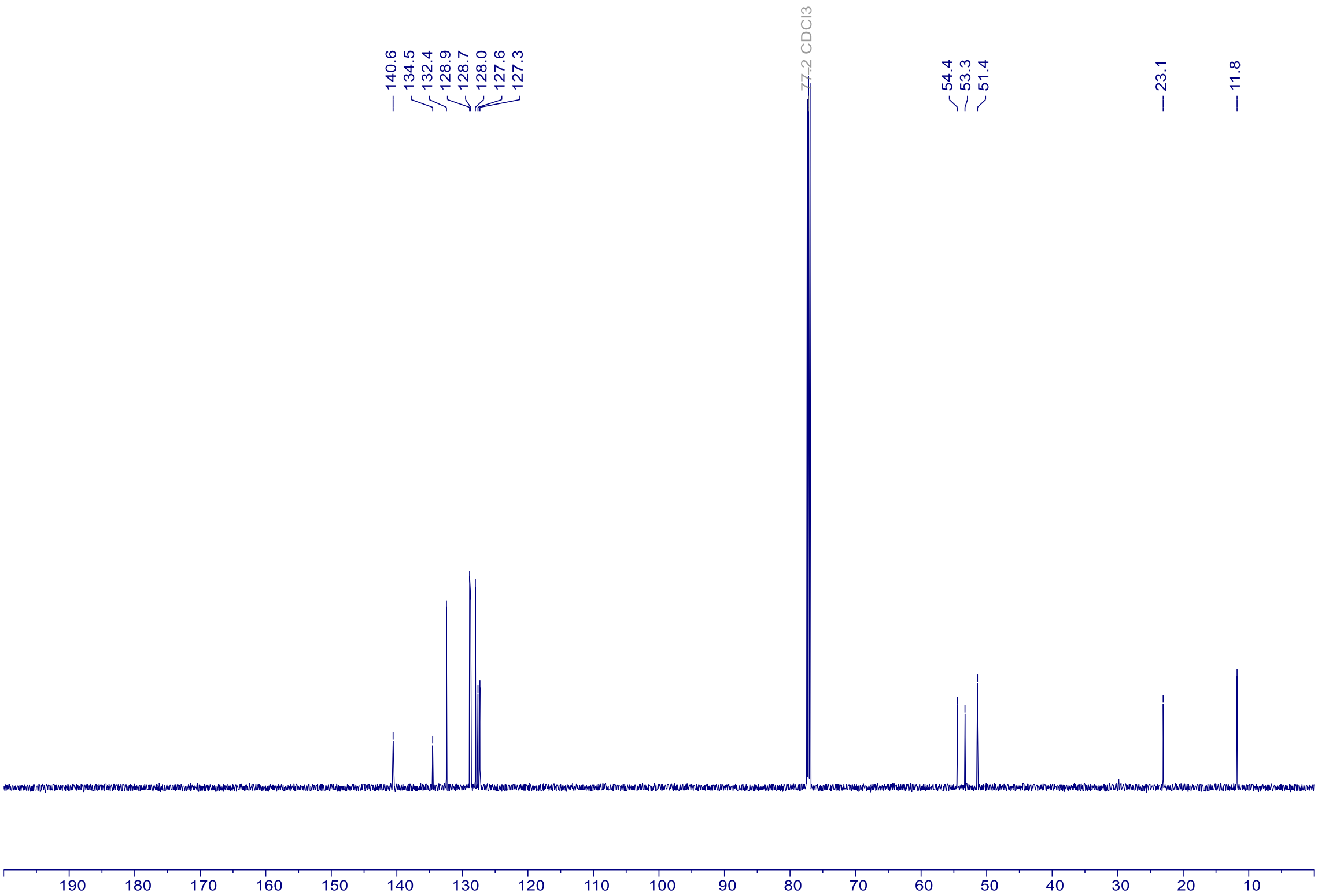

7d – ^{1}H NMR (400 MHz, CDCl$_3$)

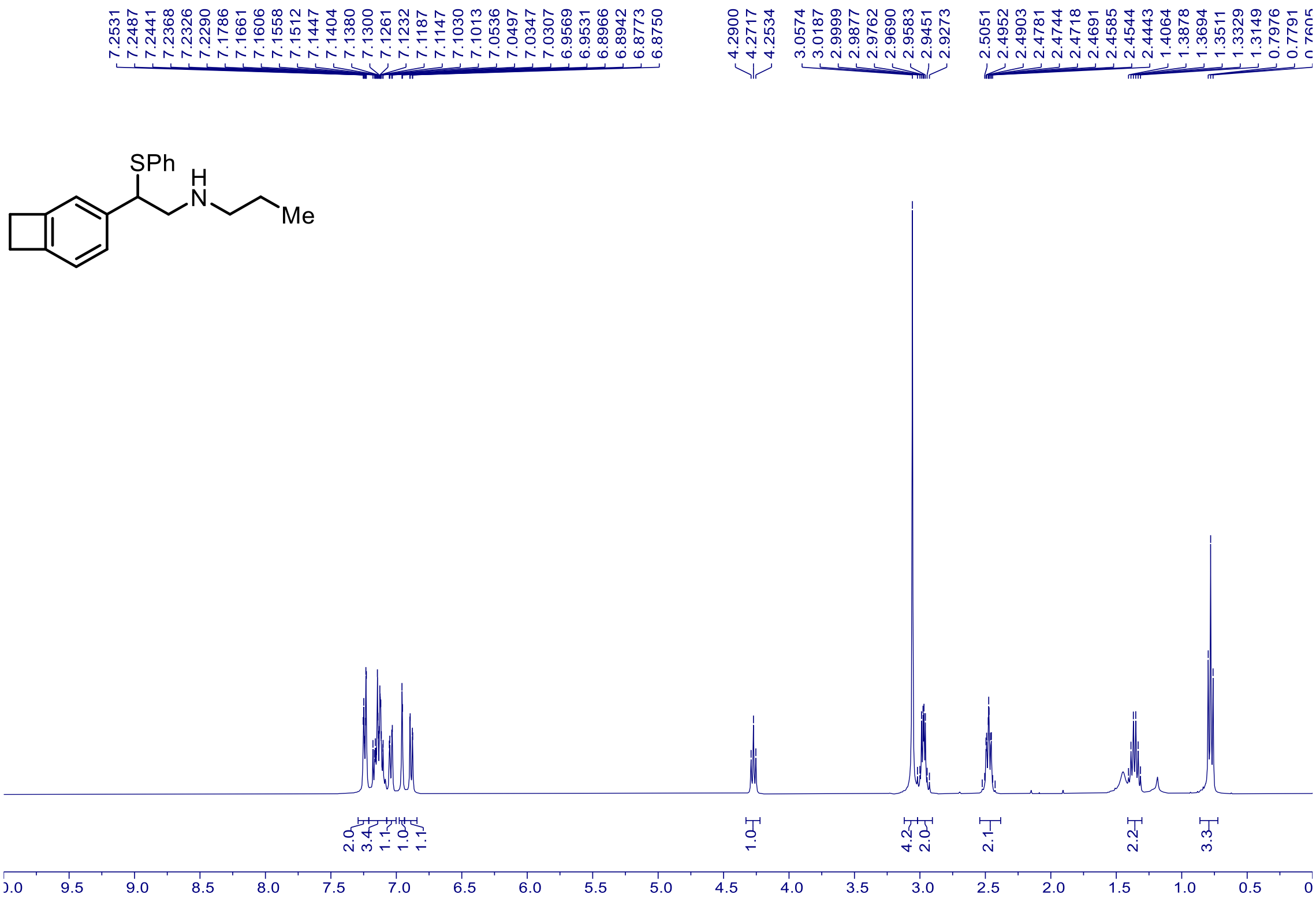

7d – ^{13}C NMR (101 MHz, CDCl$_3$)

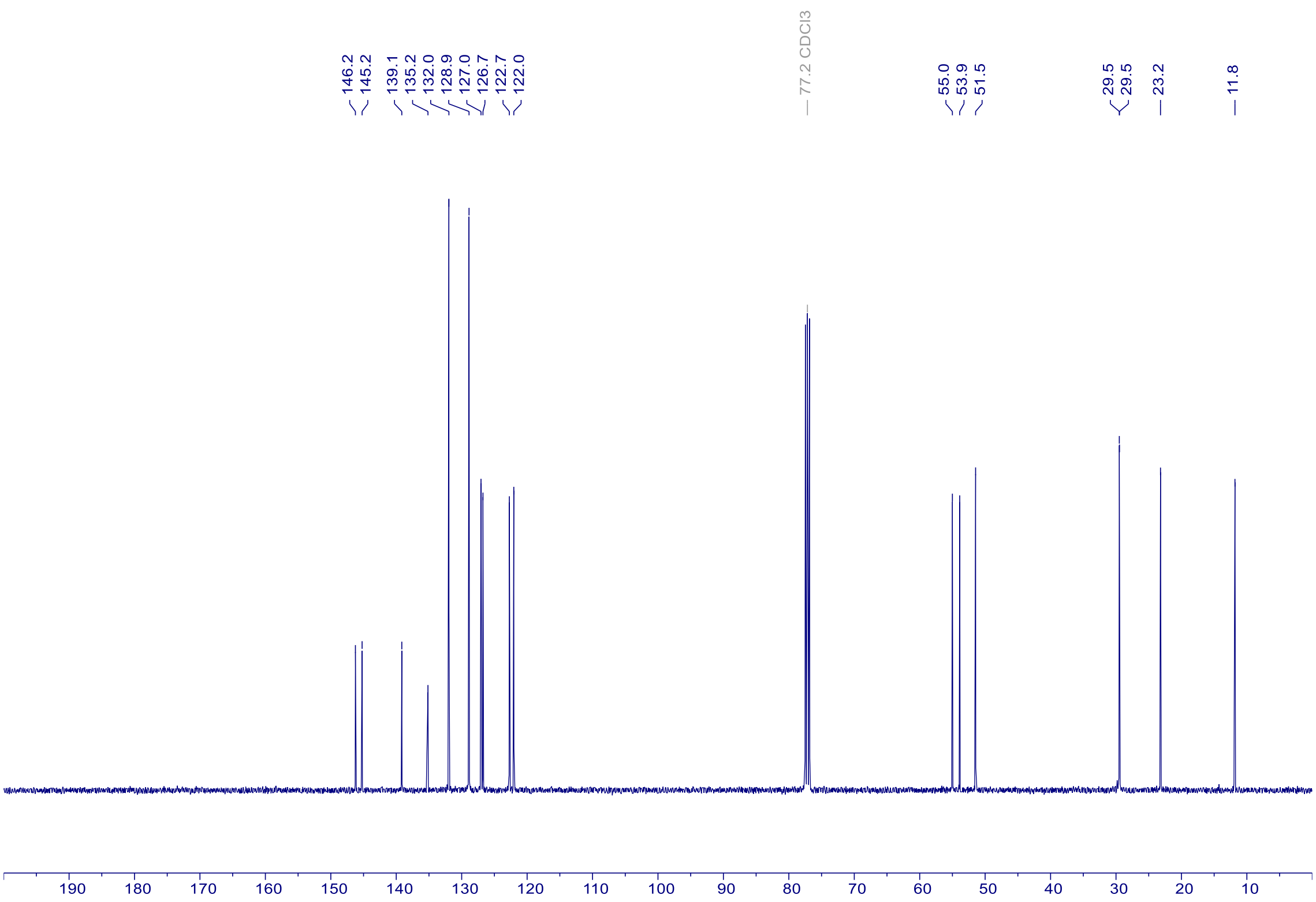

16c – ^{1}H NMR (400 MHz, $CDCl_3$)

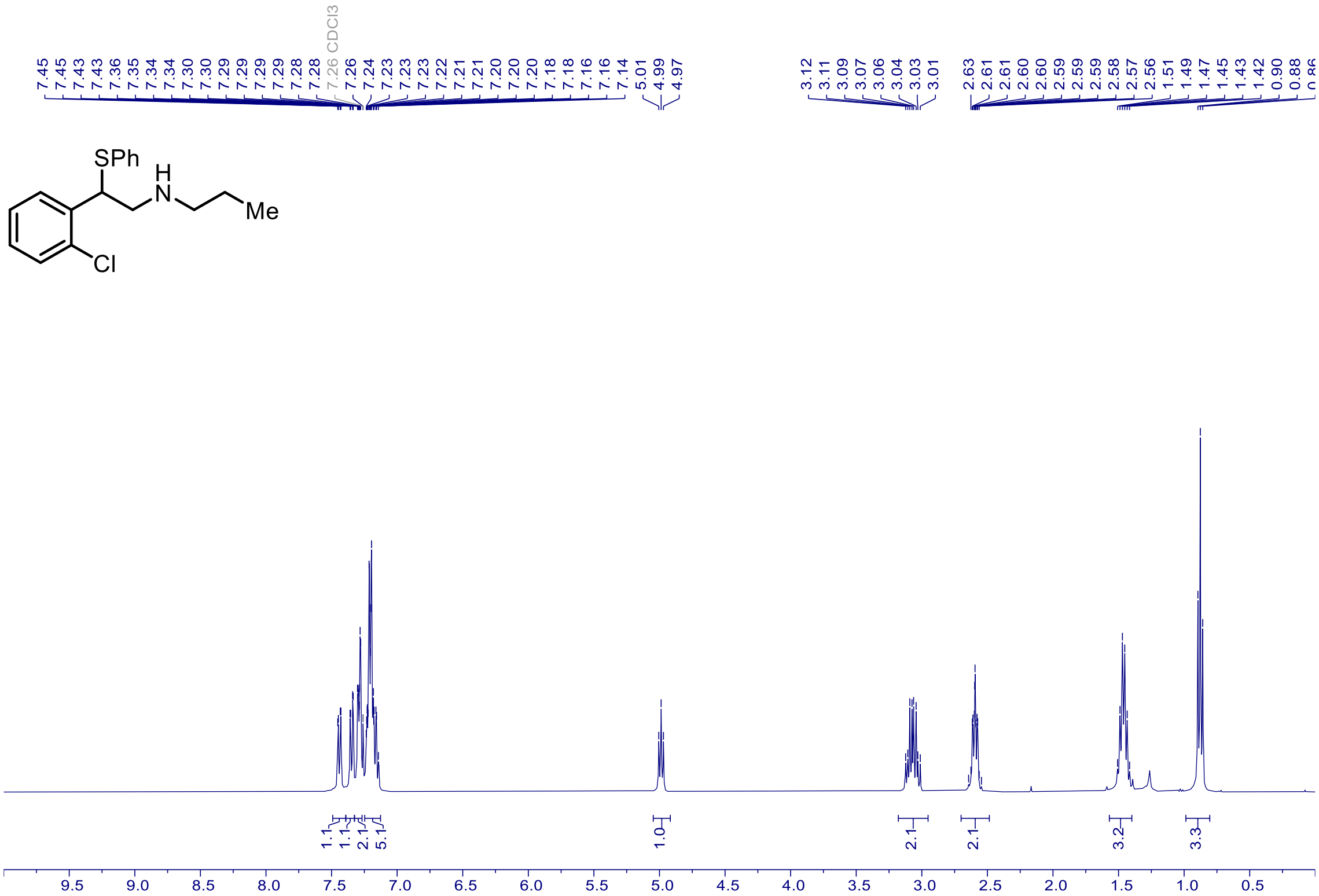

16c – ^{13}C NMR (101 MHz, $CDCl_3$)

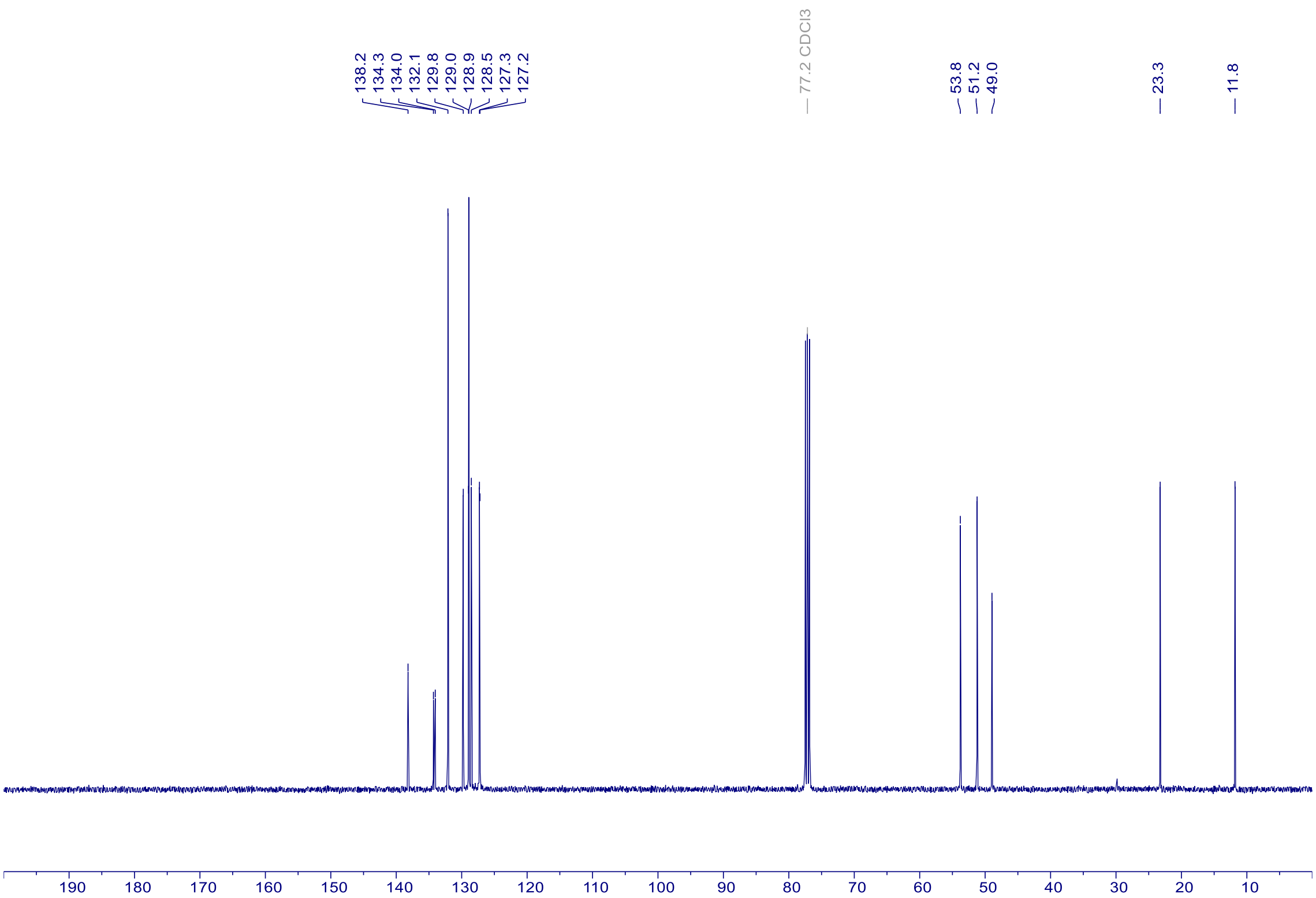

33b – ^{1}H NMR (600 MHz, CDCl$_3$)

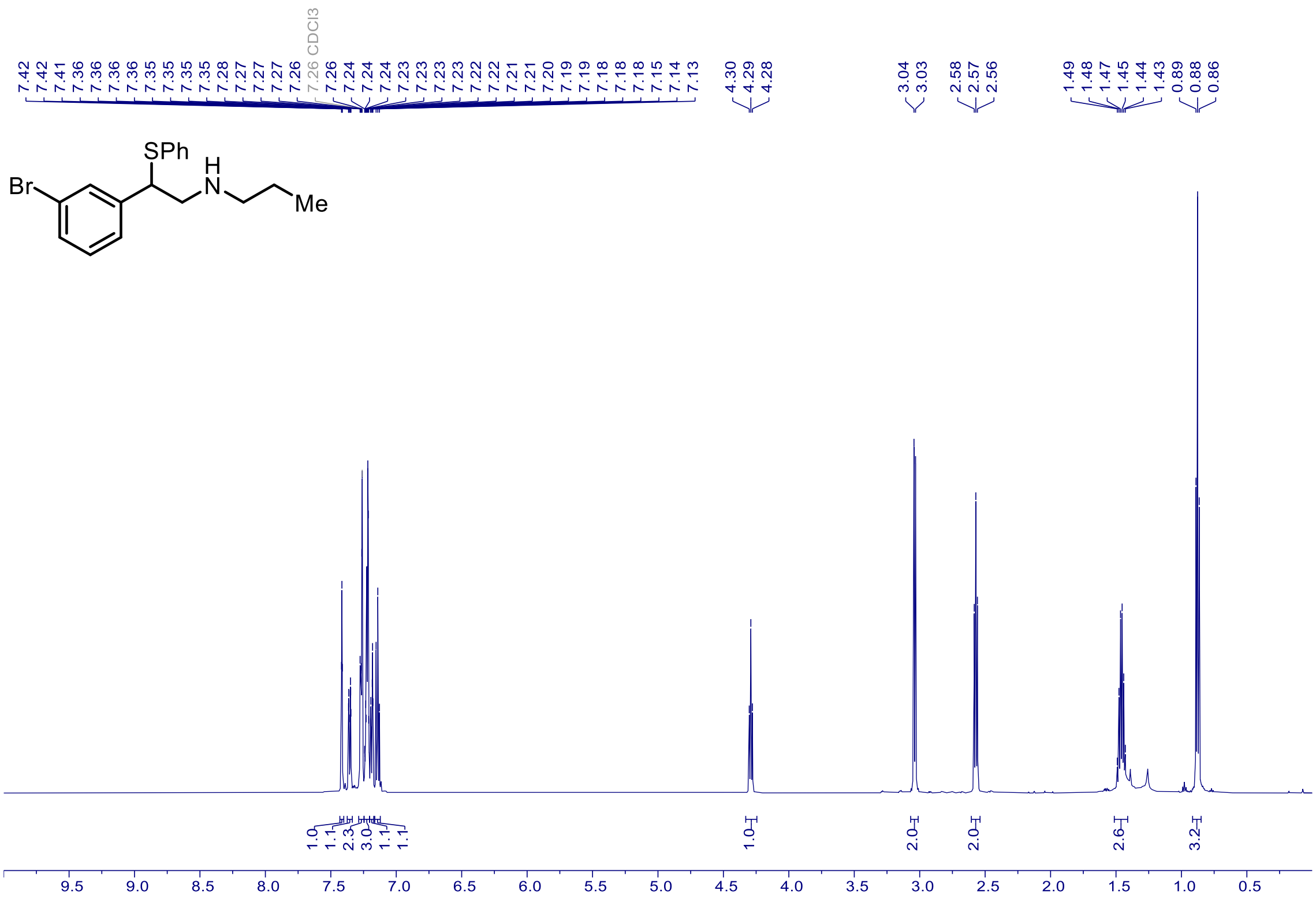

33b – ^{13}C NMR (151 MHz, CDCl$_3$)

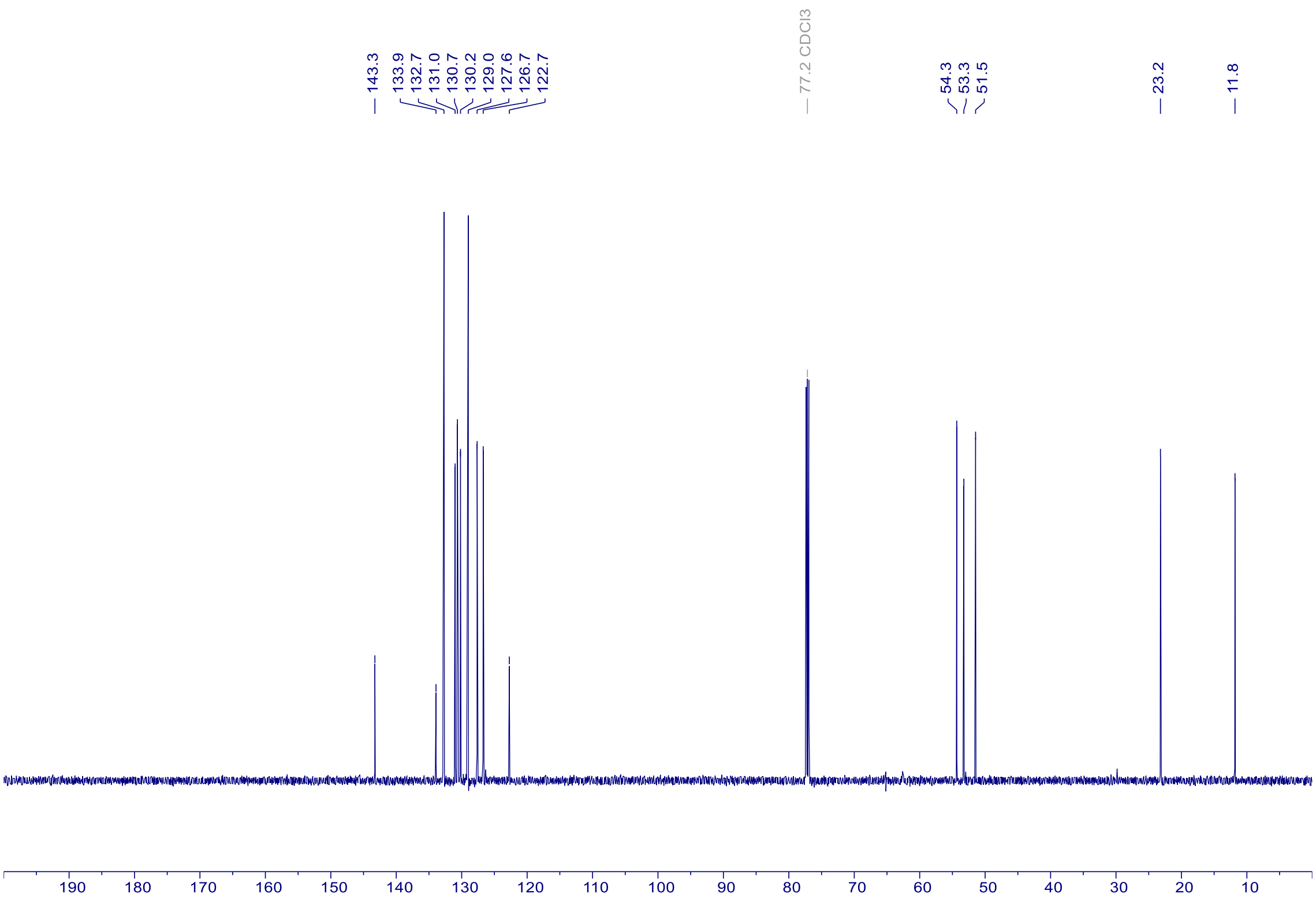

22c – ^{1}H NMR (600 MHz, CDCl$_3$)

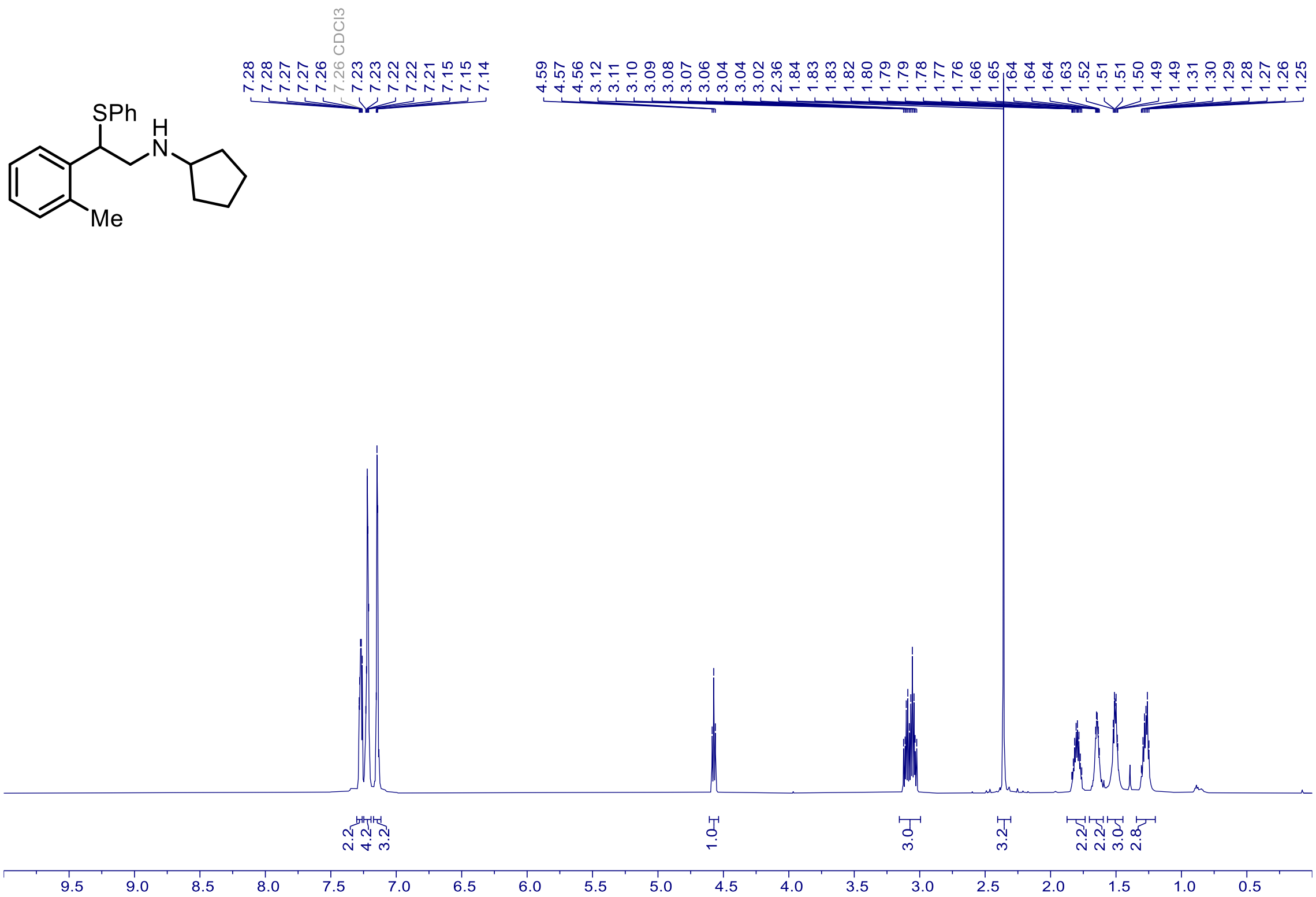

22c – ^{13}C NMR (151 MHz, CDCl$_3$)

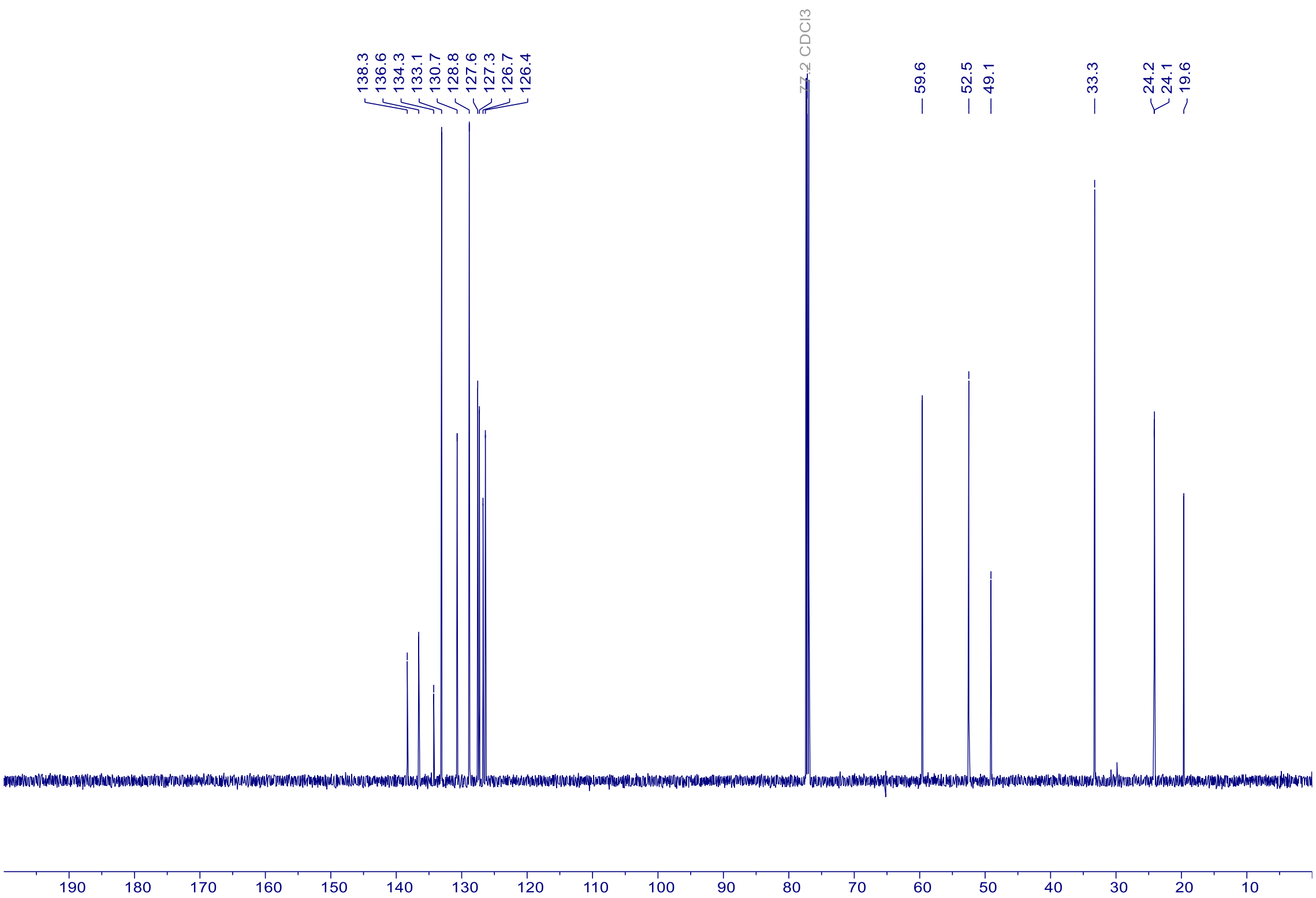

18c – ^{1}H NMR (600 MHz, $CDCl_3$)

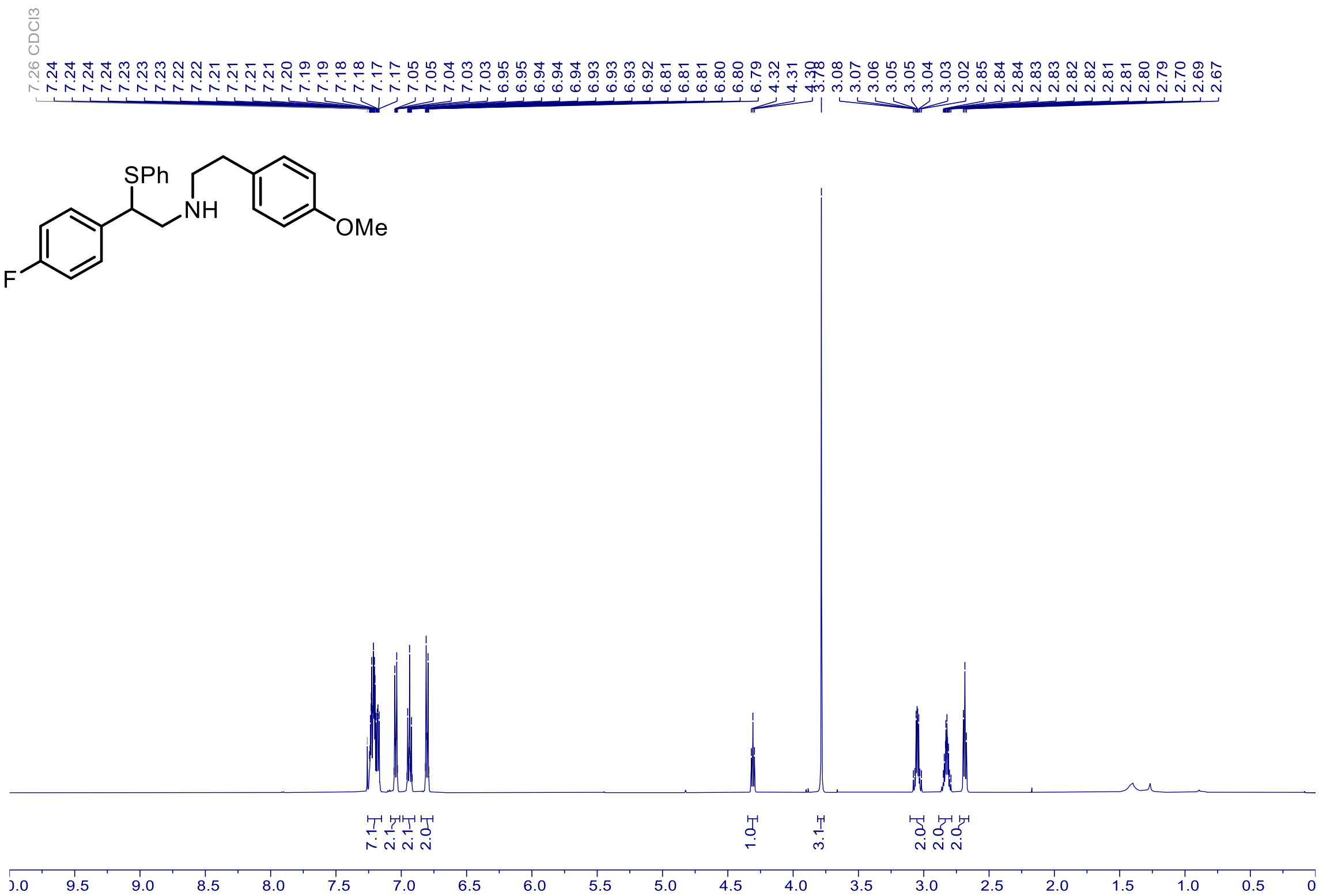

18c – ^{13}C NMR (151 MHz, $CDCl_3$)

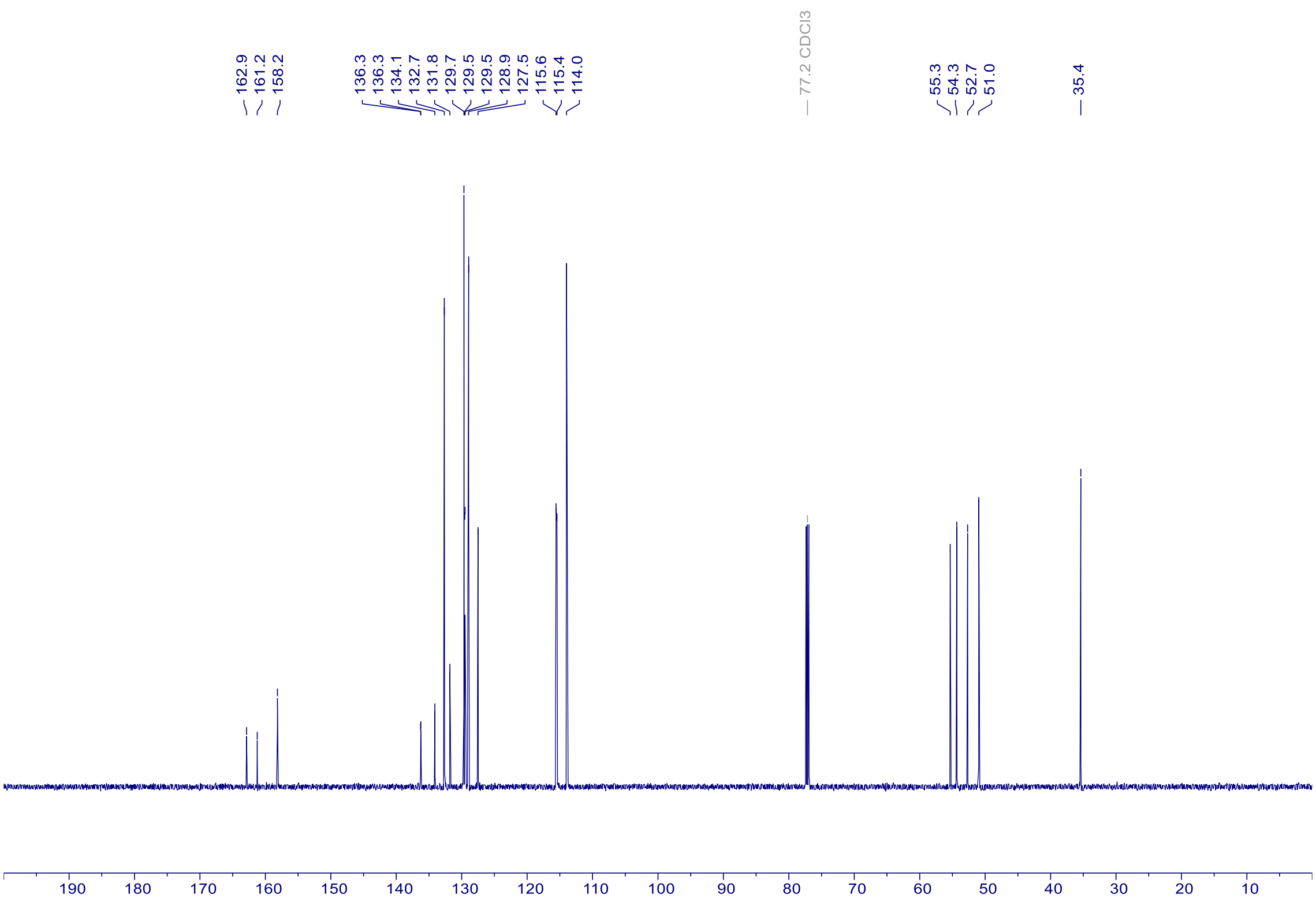

18c – ^{19}F NMR (565 MHz, $CDCl_3$)

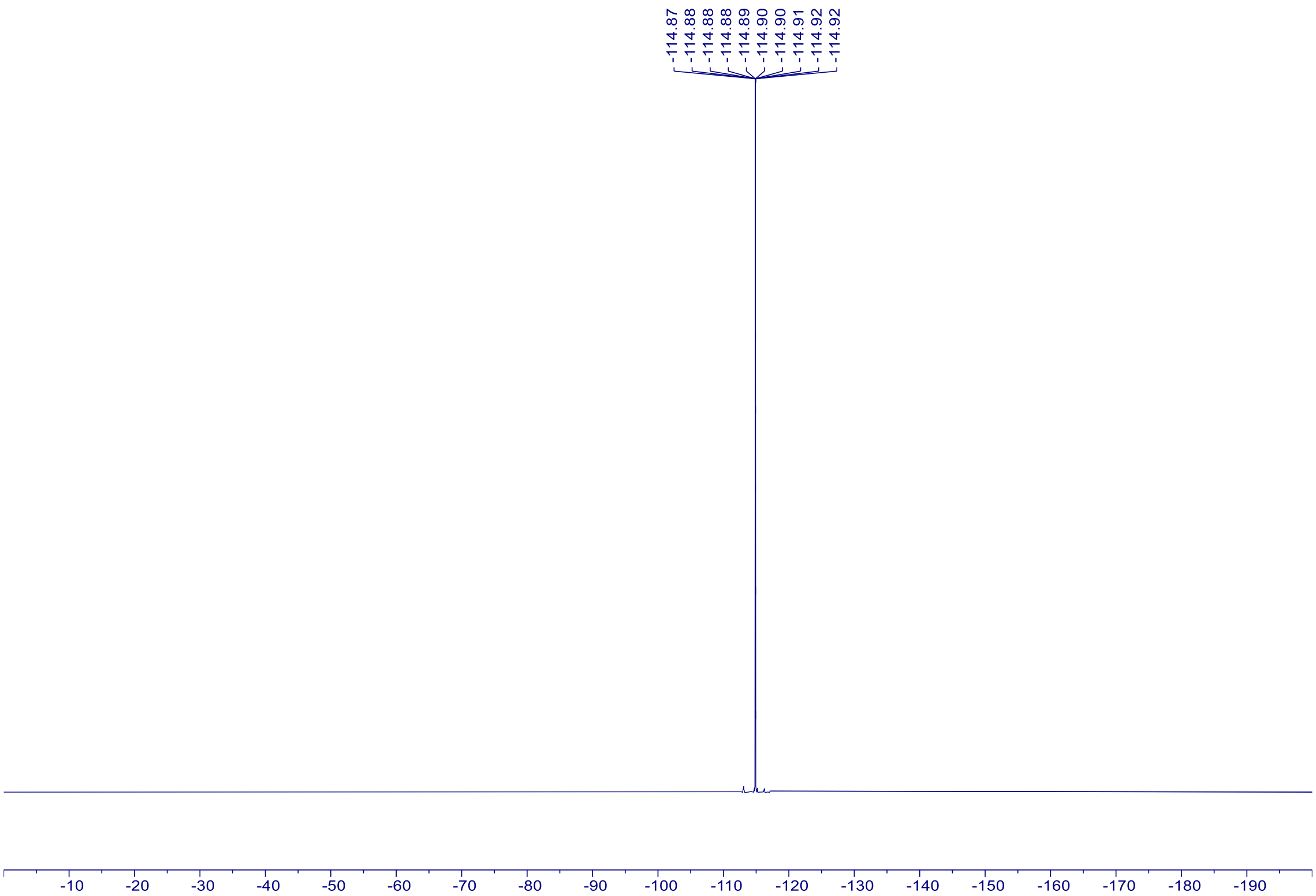

13d – ^{1}H NMR (400 MHz, $CDCl_3$)

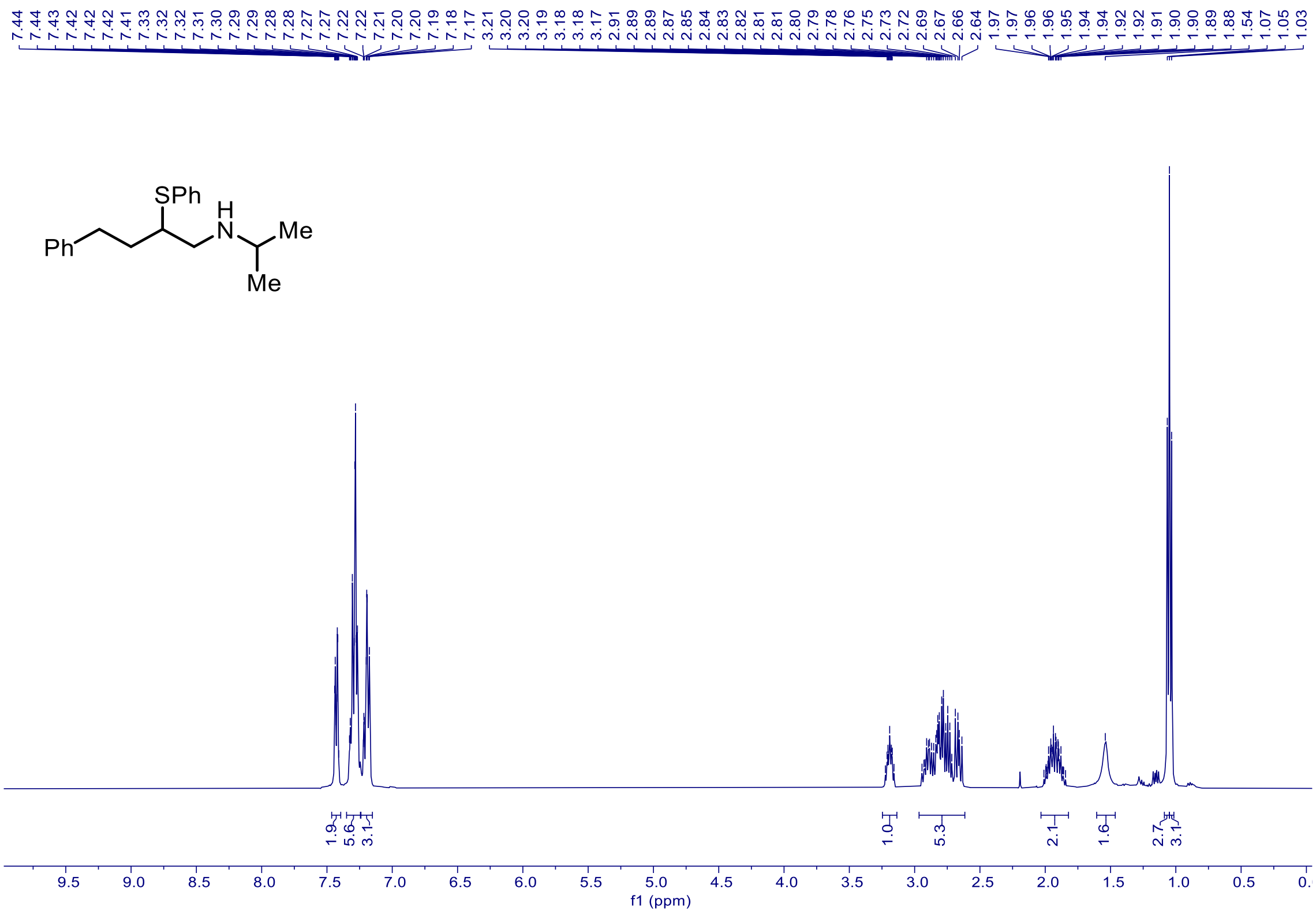

13d – ^{13}C NMR (101 MHz, $CDCl_3$)

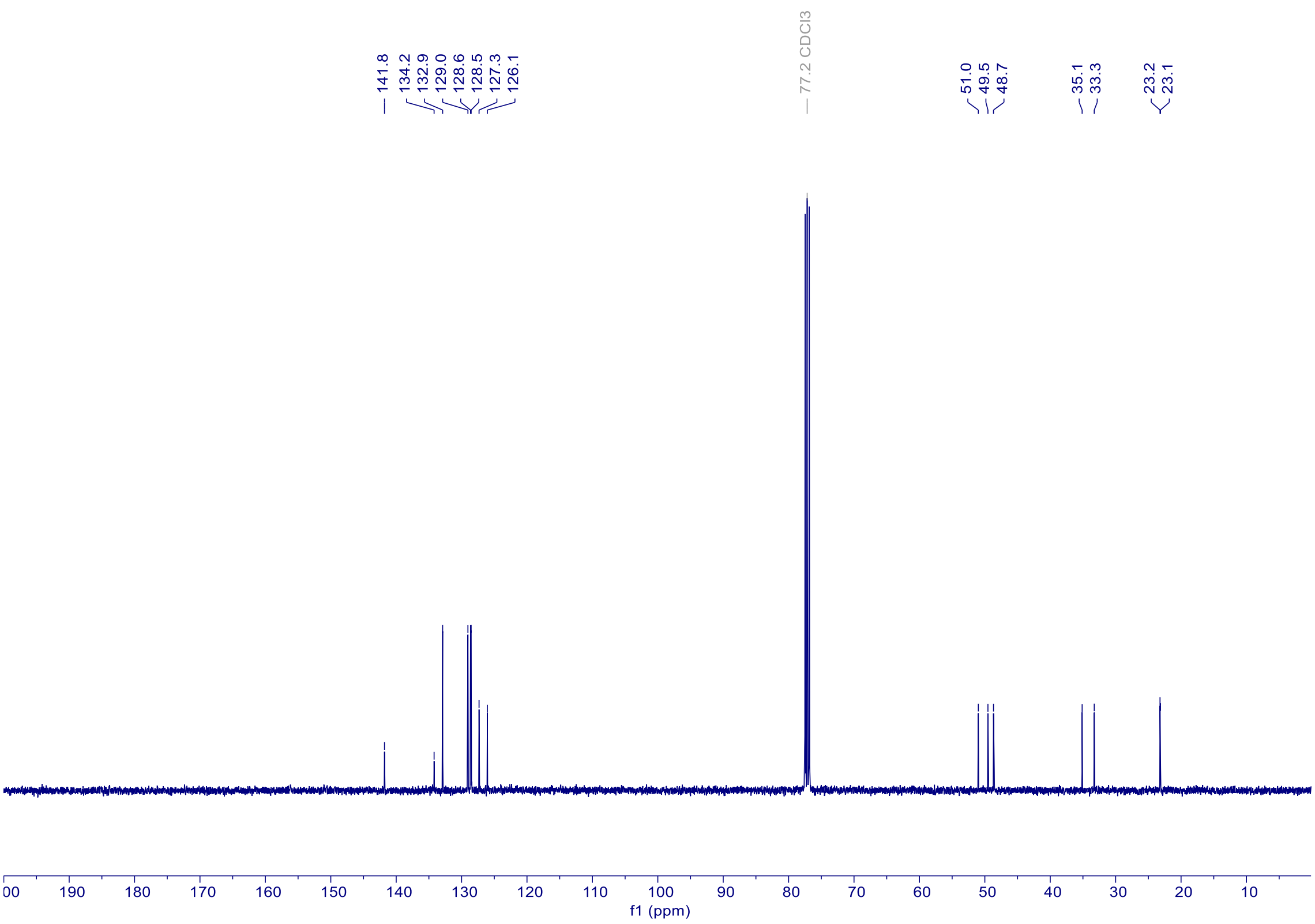

13e – ^{1}H NMR (400 MHz, $CDCl_3$)

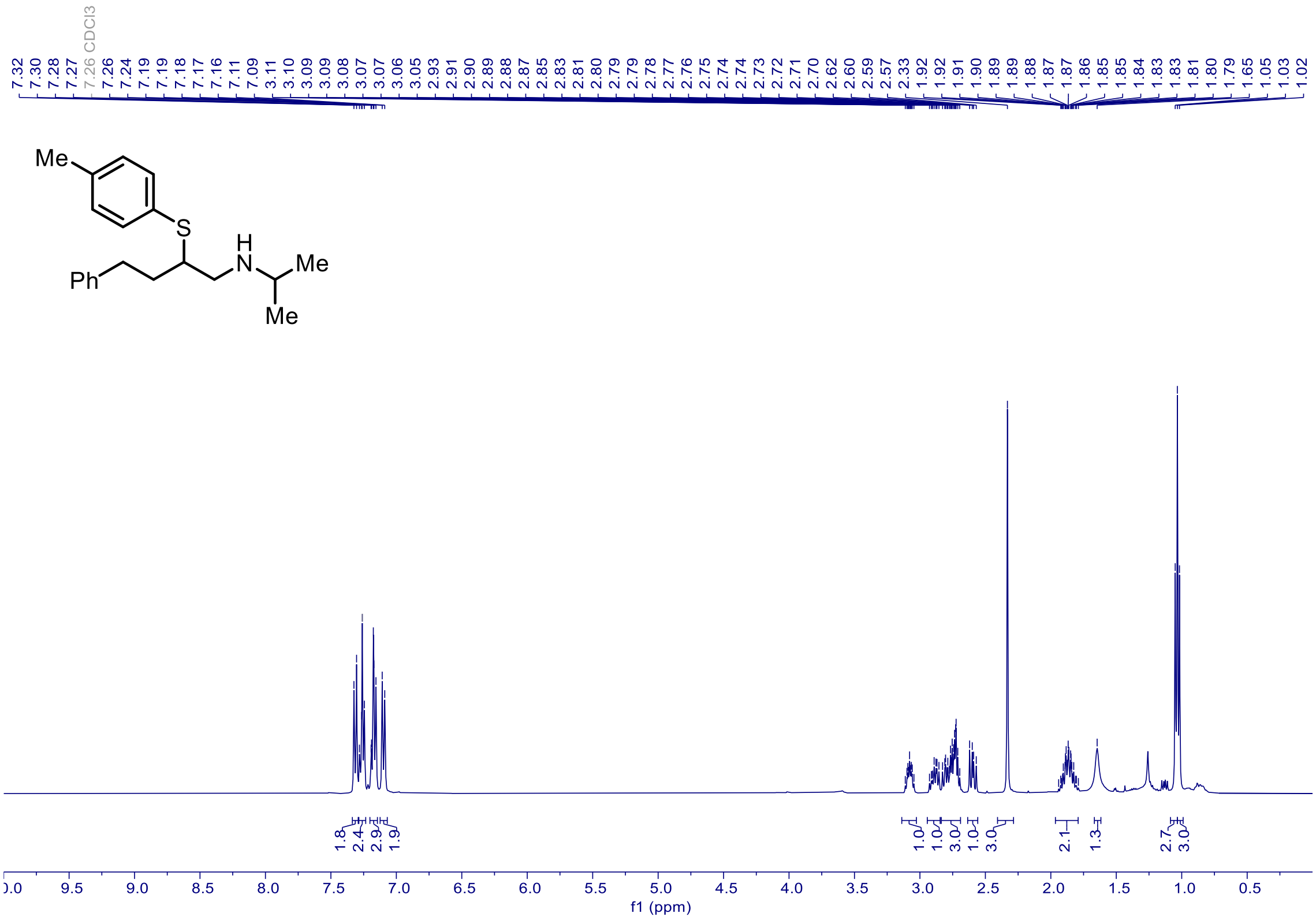

13e – ^{13}C NMR (101 MHz, $CDCl_3$)

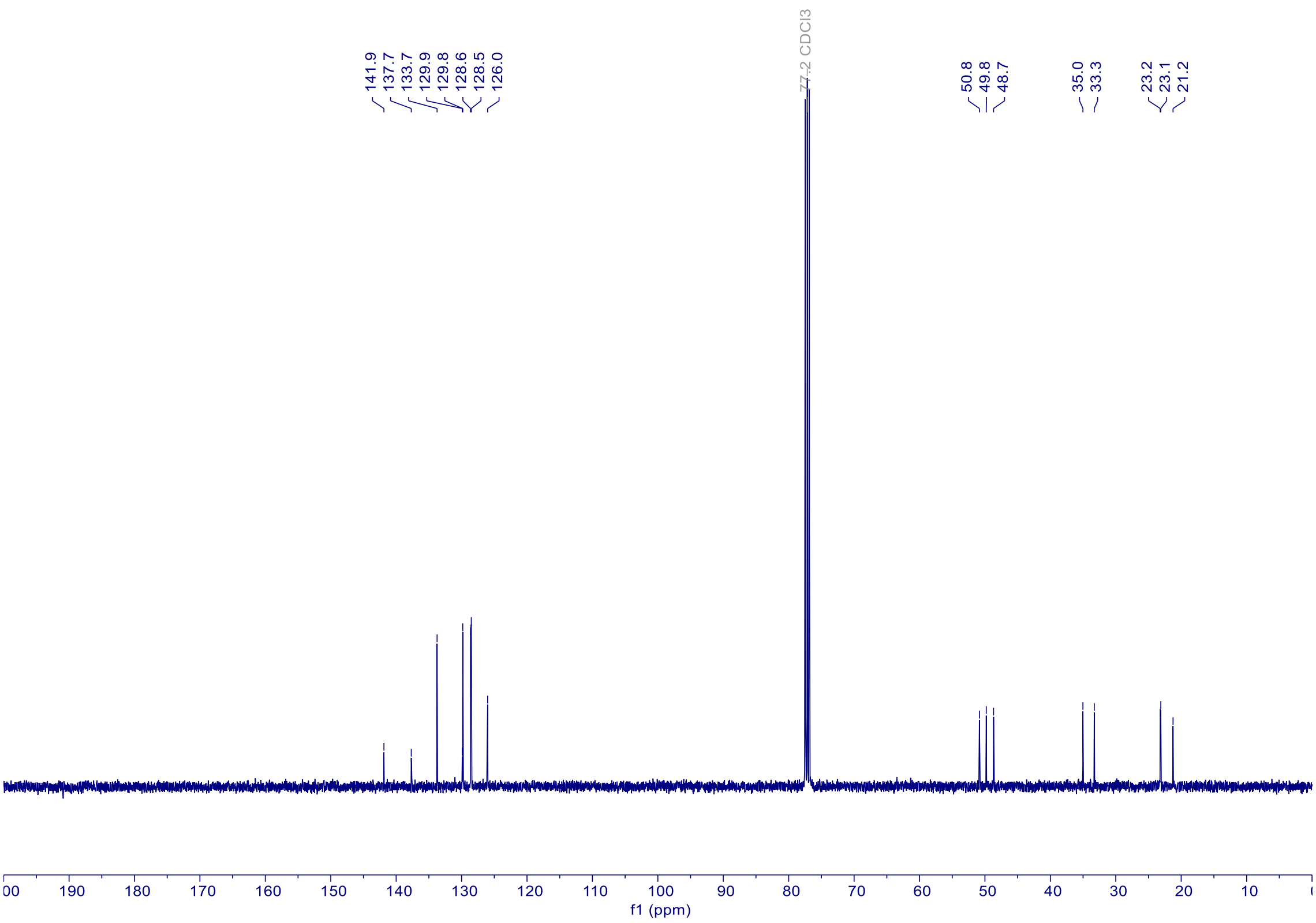

13f – ^{1}H NMR (600 MHz, $CDCl_3$)

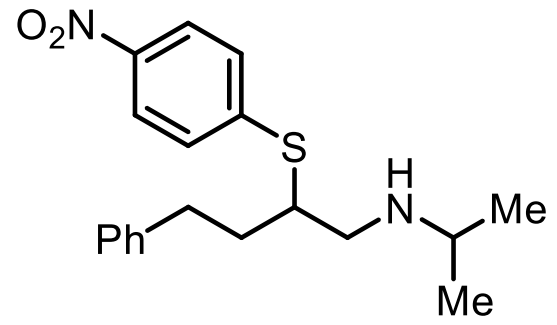

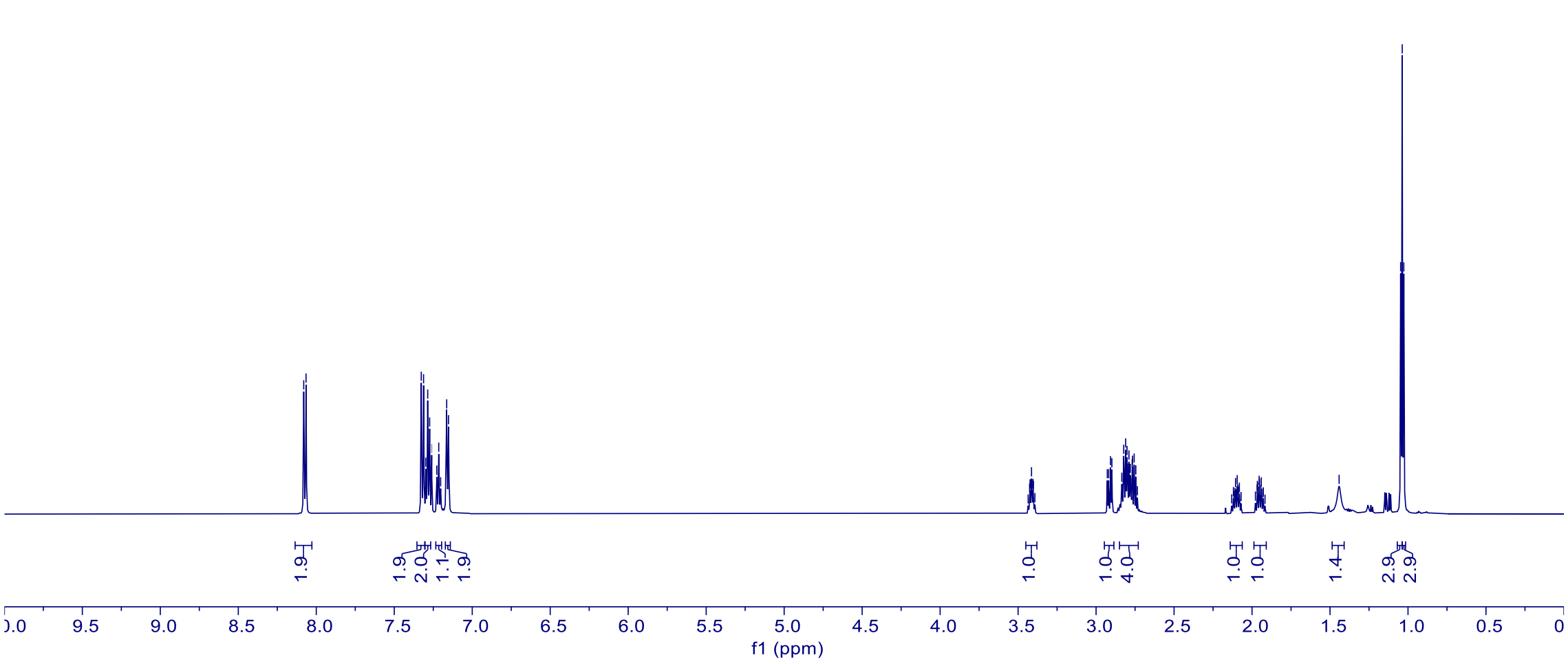

13f – ^{13}C NMR (101 MHz, $CDCl_3$)

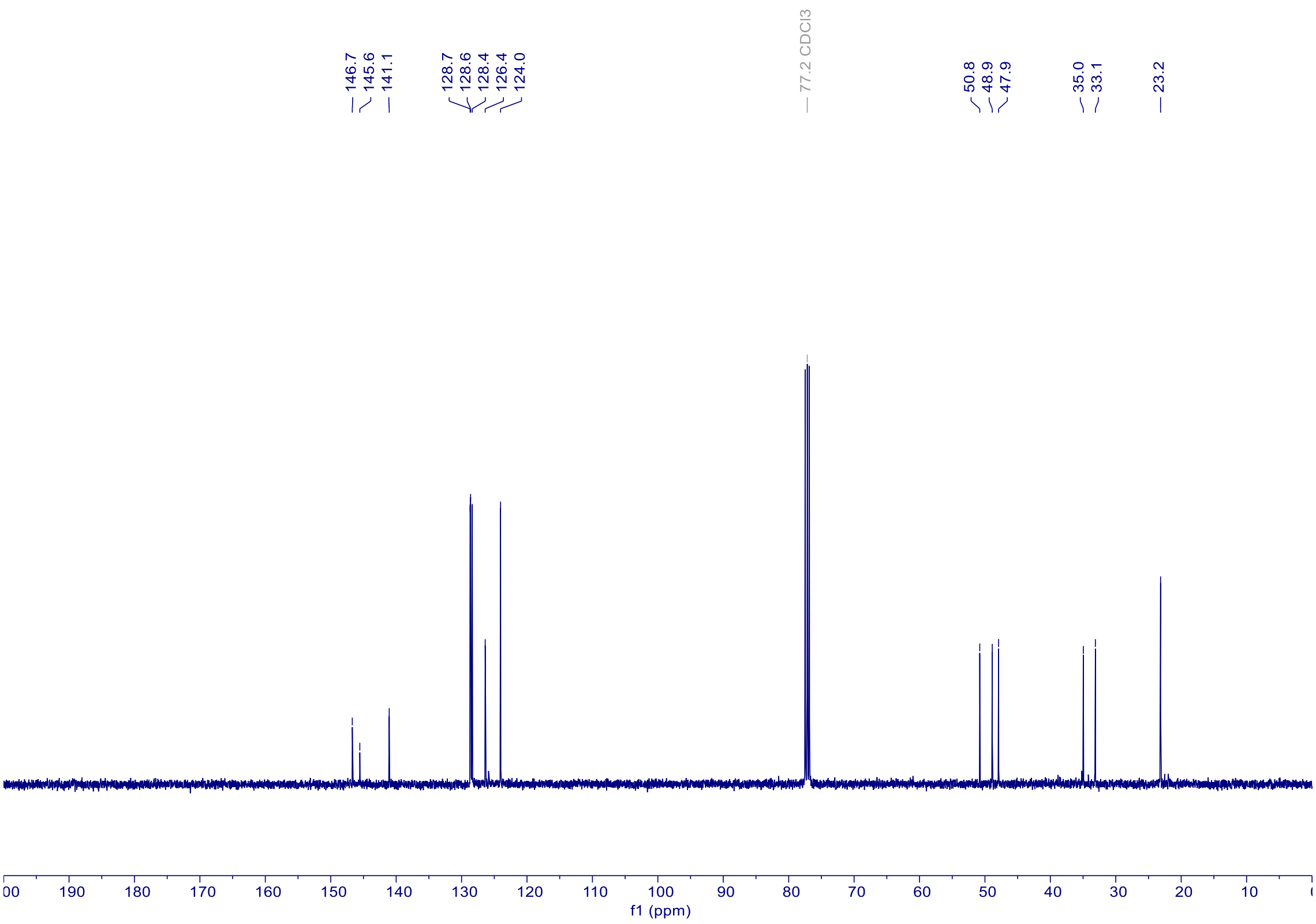

31c *(major diasteroisomer)* – ^{1}H NMR (400 MHz, $CDCl_3$)

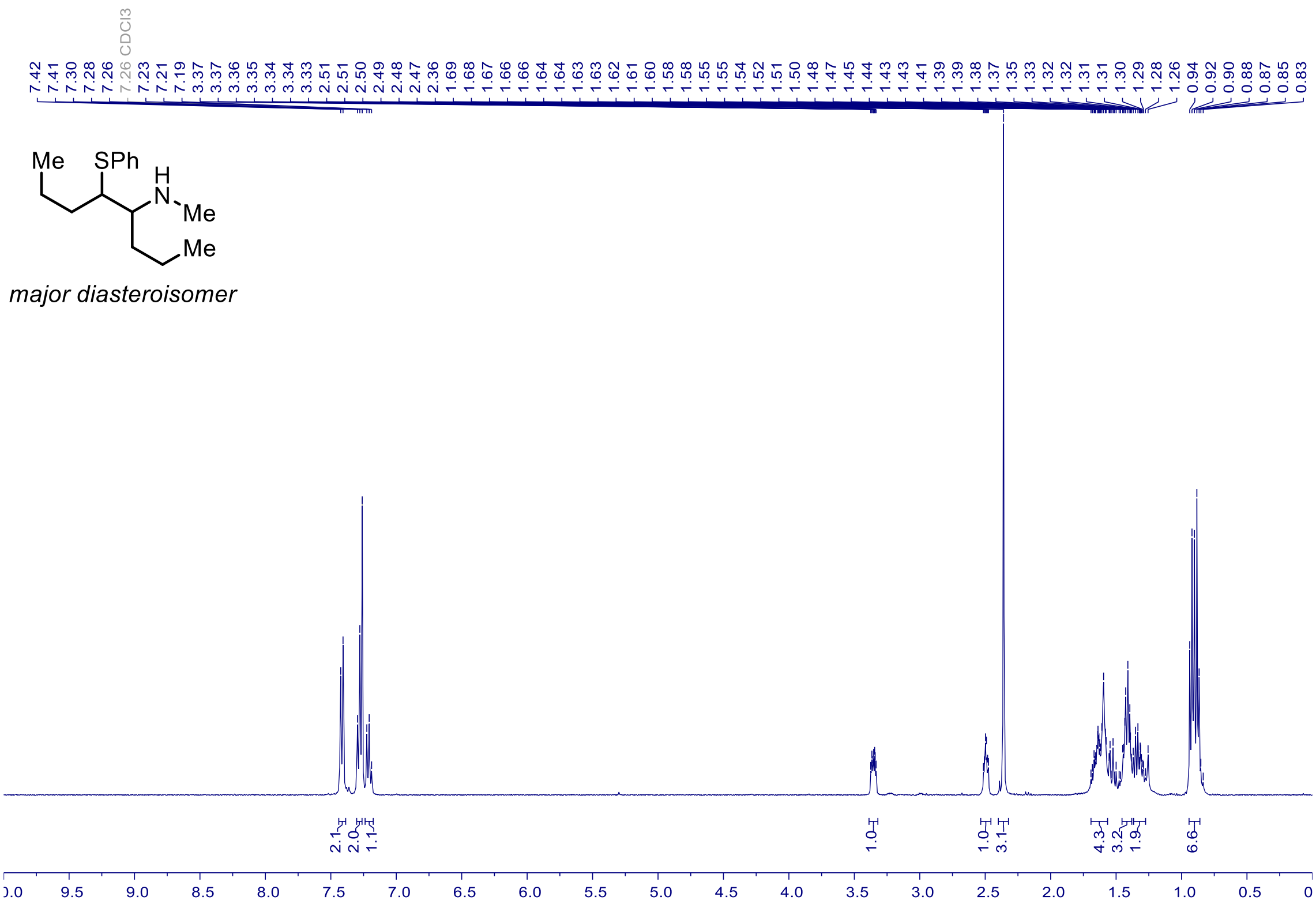

31c *(major diasteroisomer)* – ^{13}C NMR (101 MHz, $CDCl_3$)

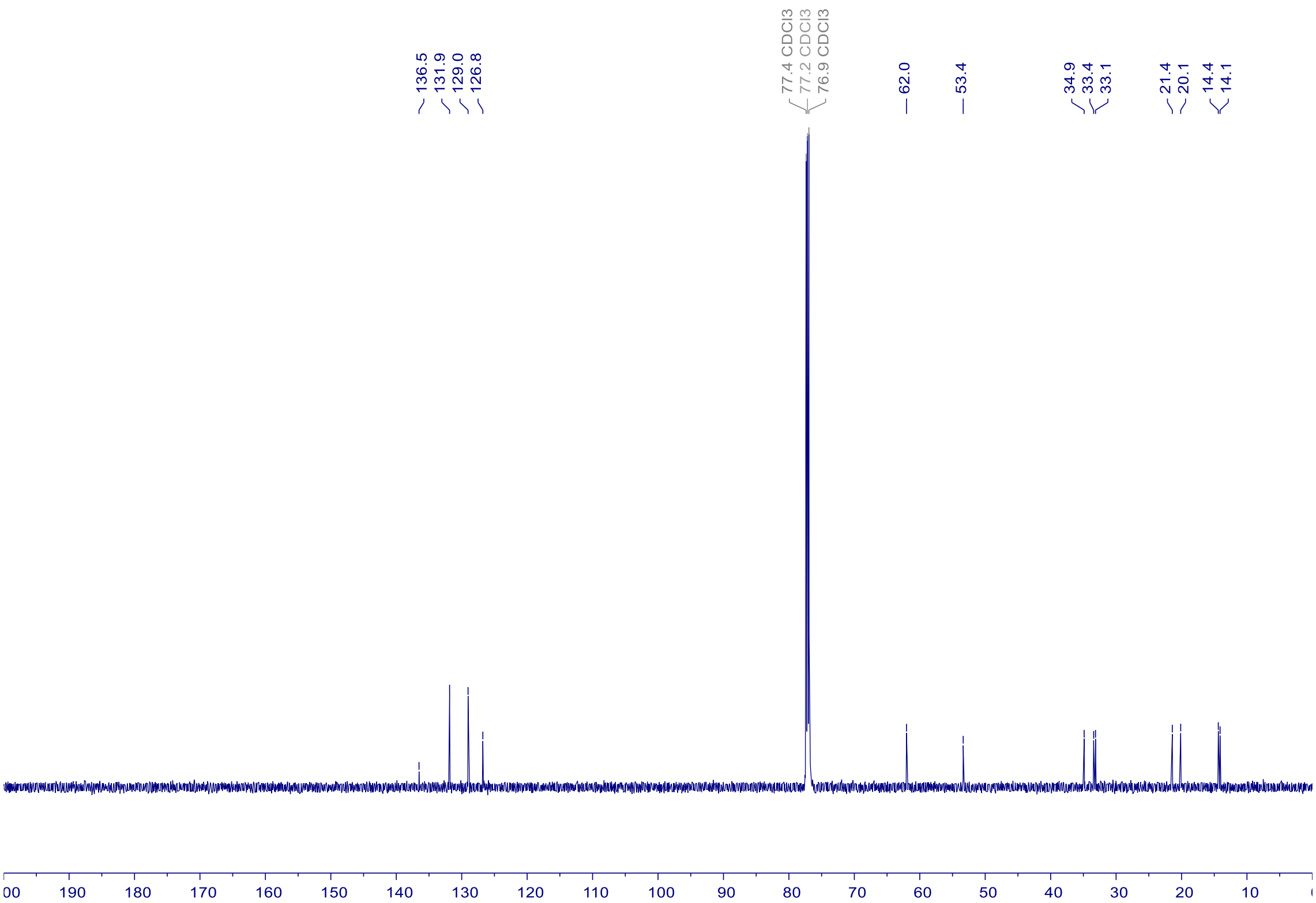

31c *(minor diasteroisomer)* – ^{1}H NMR (400 MHz, $CDCl_3$)

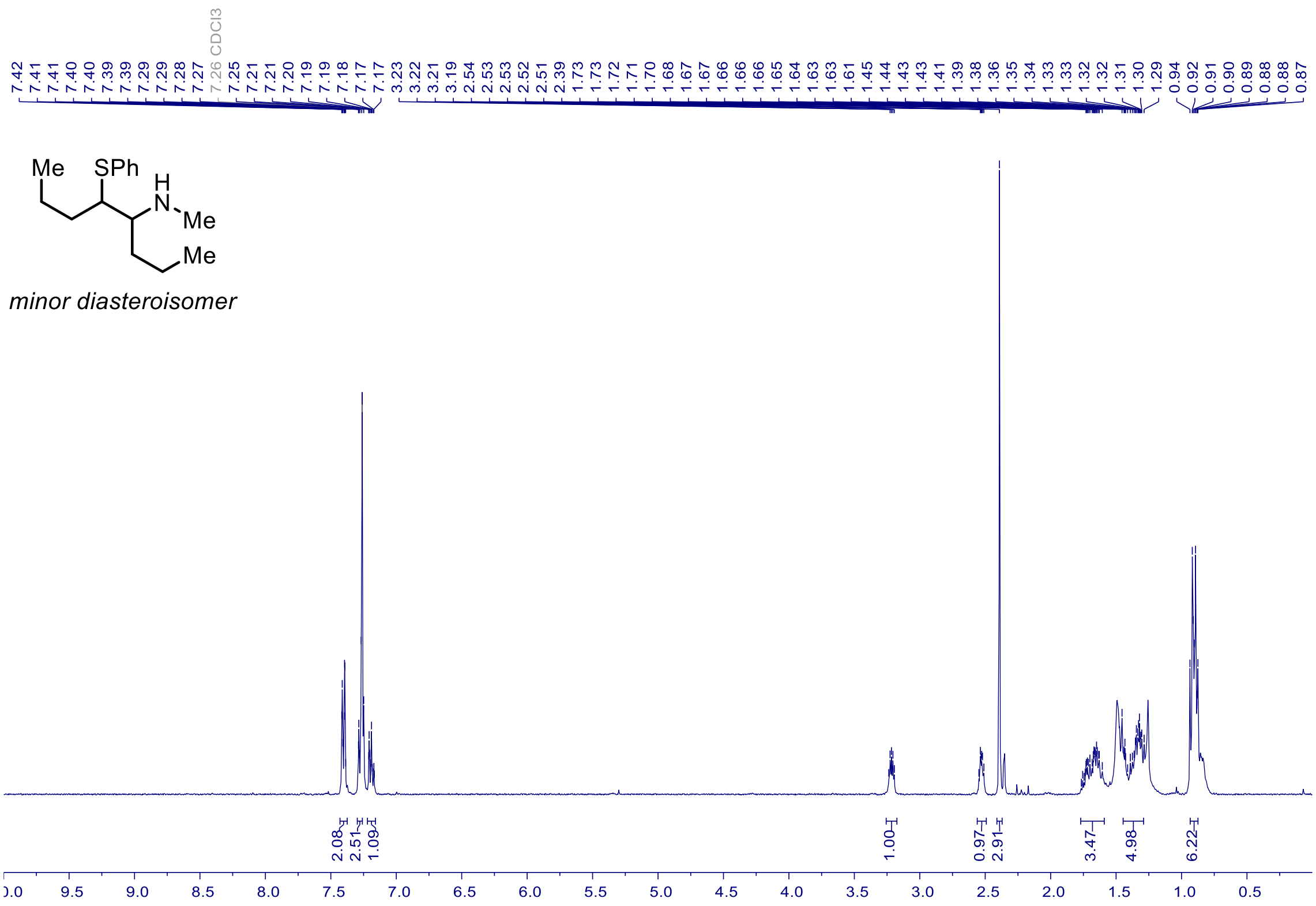

31c *(minor diasteroisomer)* – ^{13}C NMR (101 MHz, $CDCl_3$)

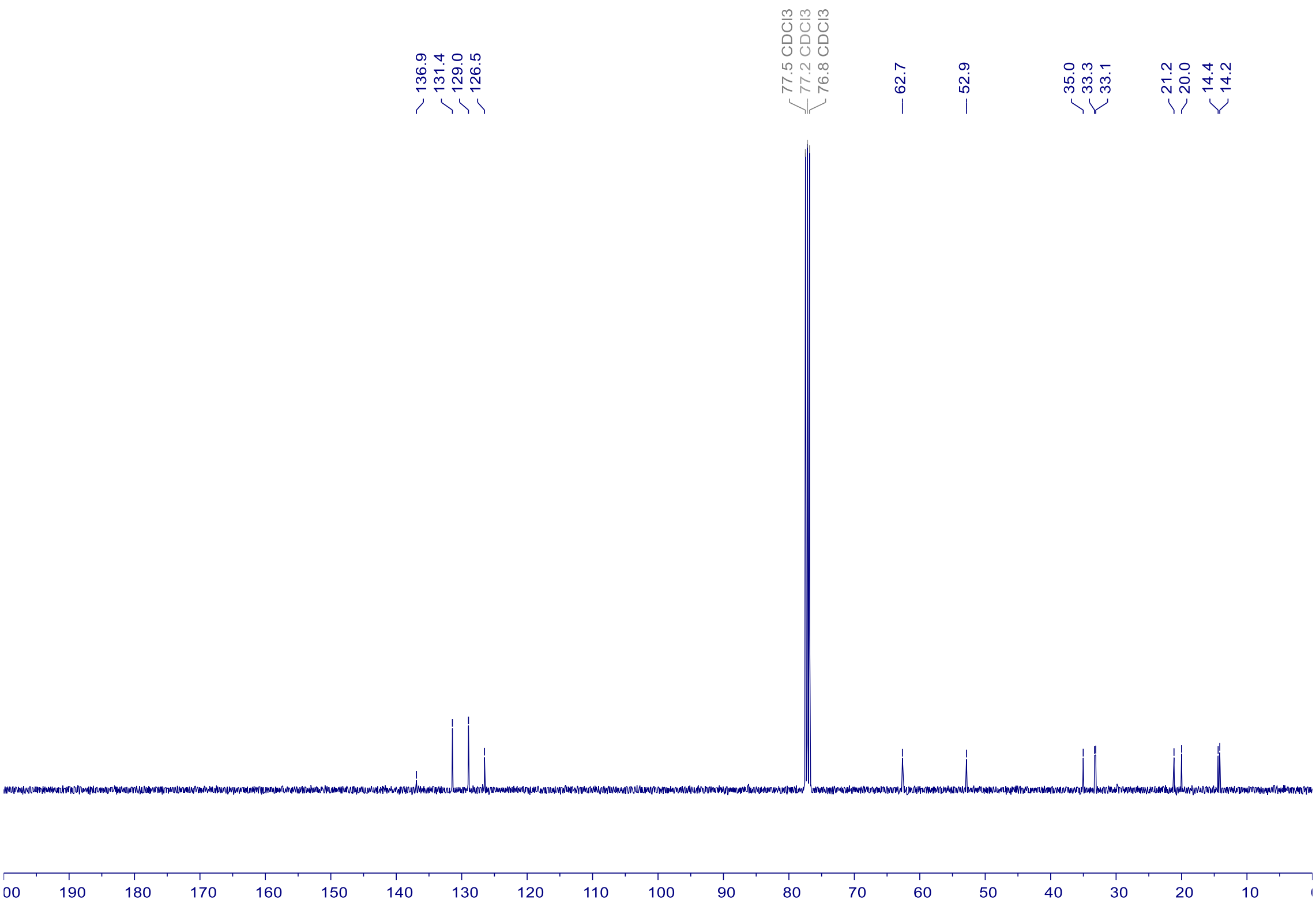

32c *(major diasteroisomer)* – ^{1}H NMR (400 MHz, $CDCl_3$)

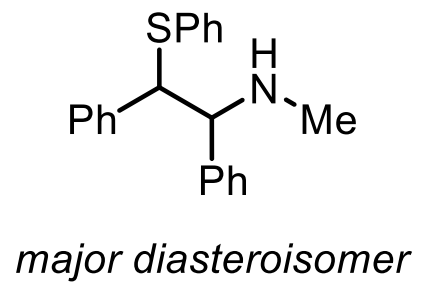

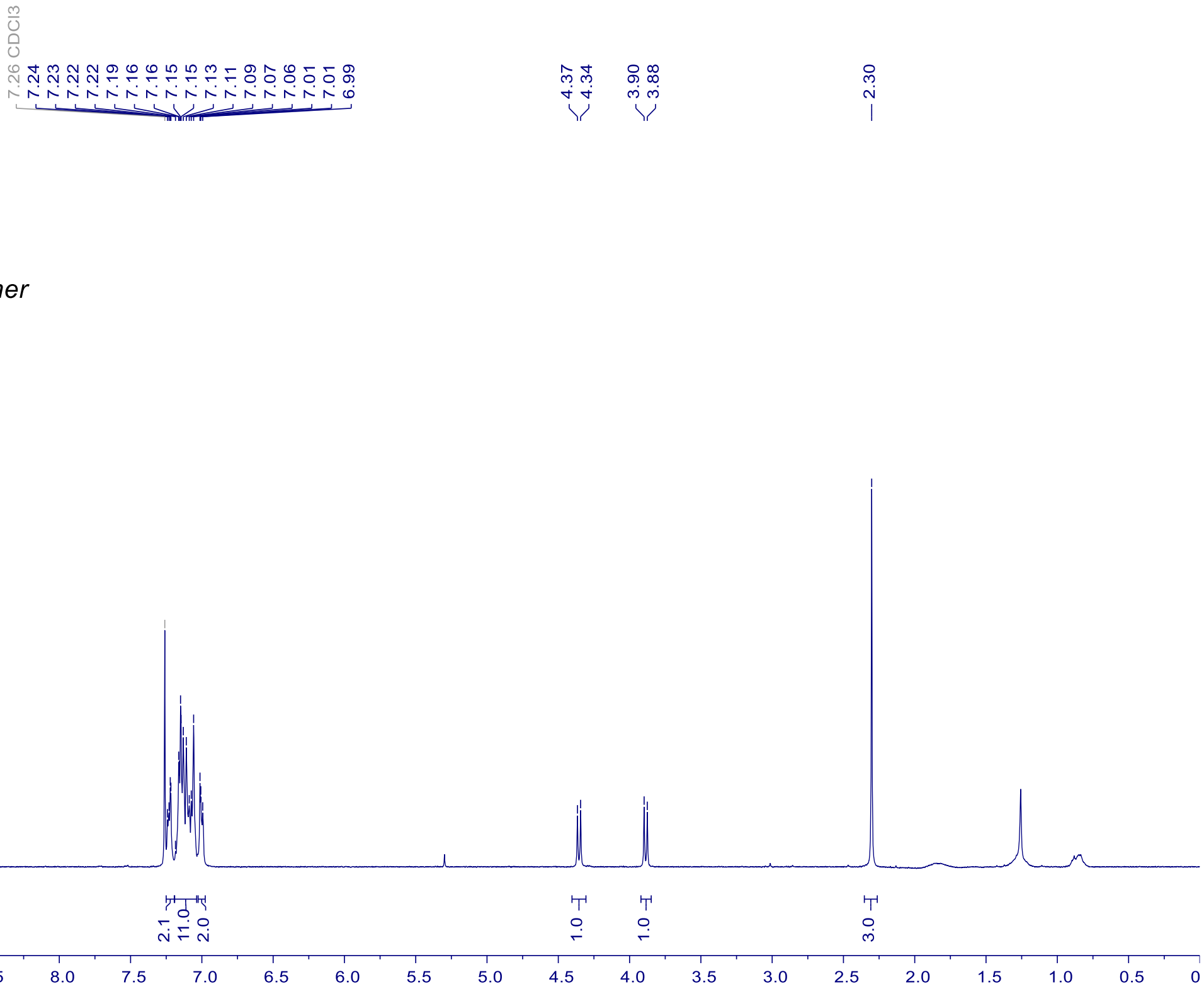

32c *(major diasteroisomer)* – ^{13}C NMR (101 MHz, $CDCl_3$)

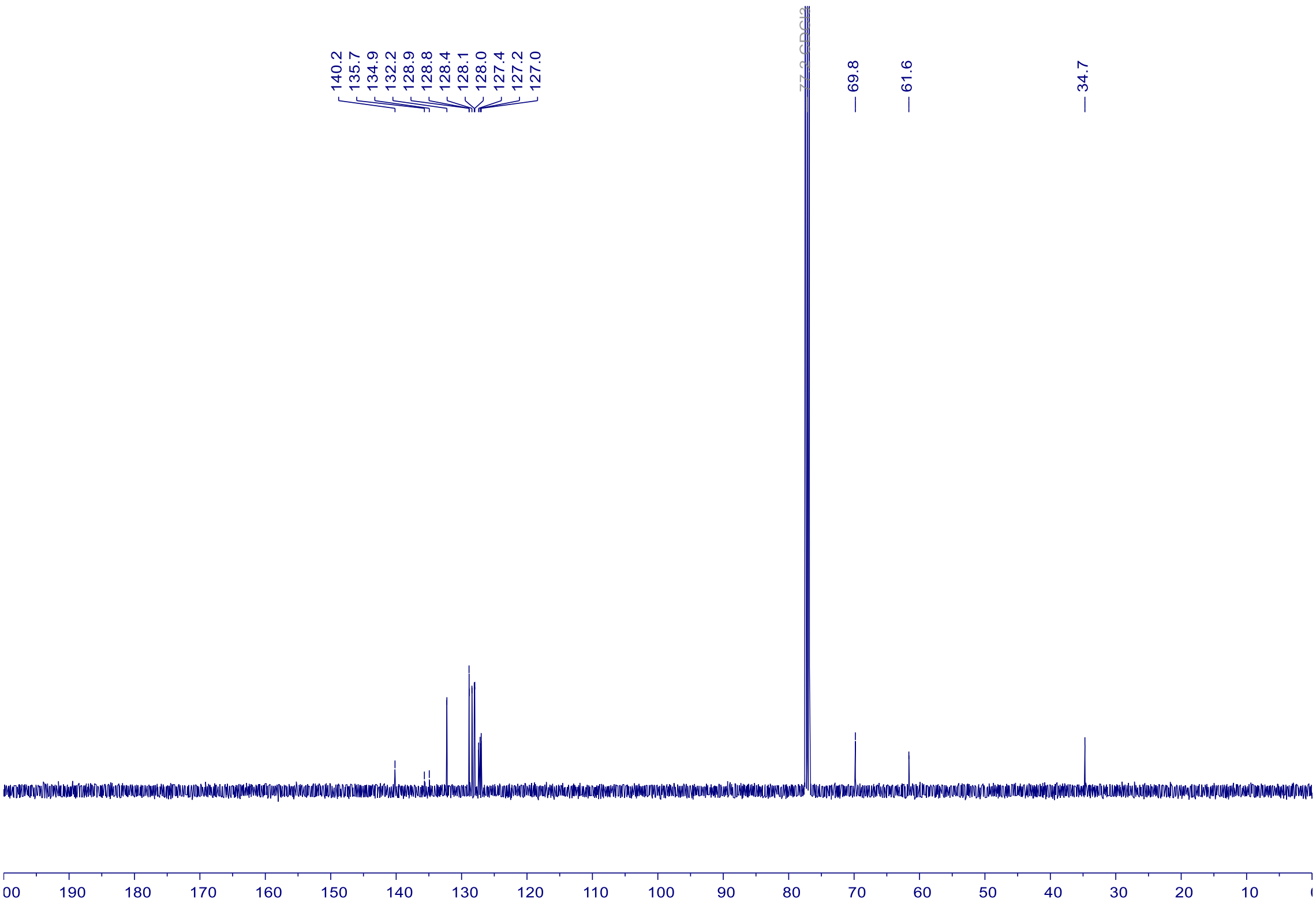

32c *(minor diasteroisomer)* – ^{1}H NMR (400 MHz, $CDCl_3$)

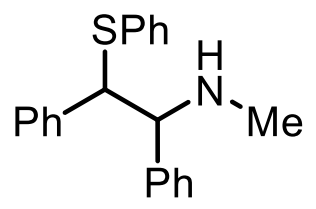

minor diasteroisomer

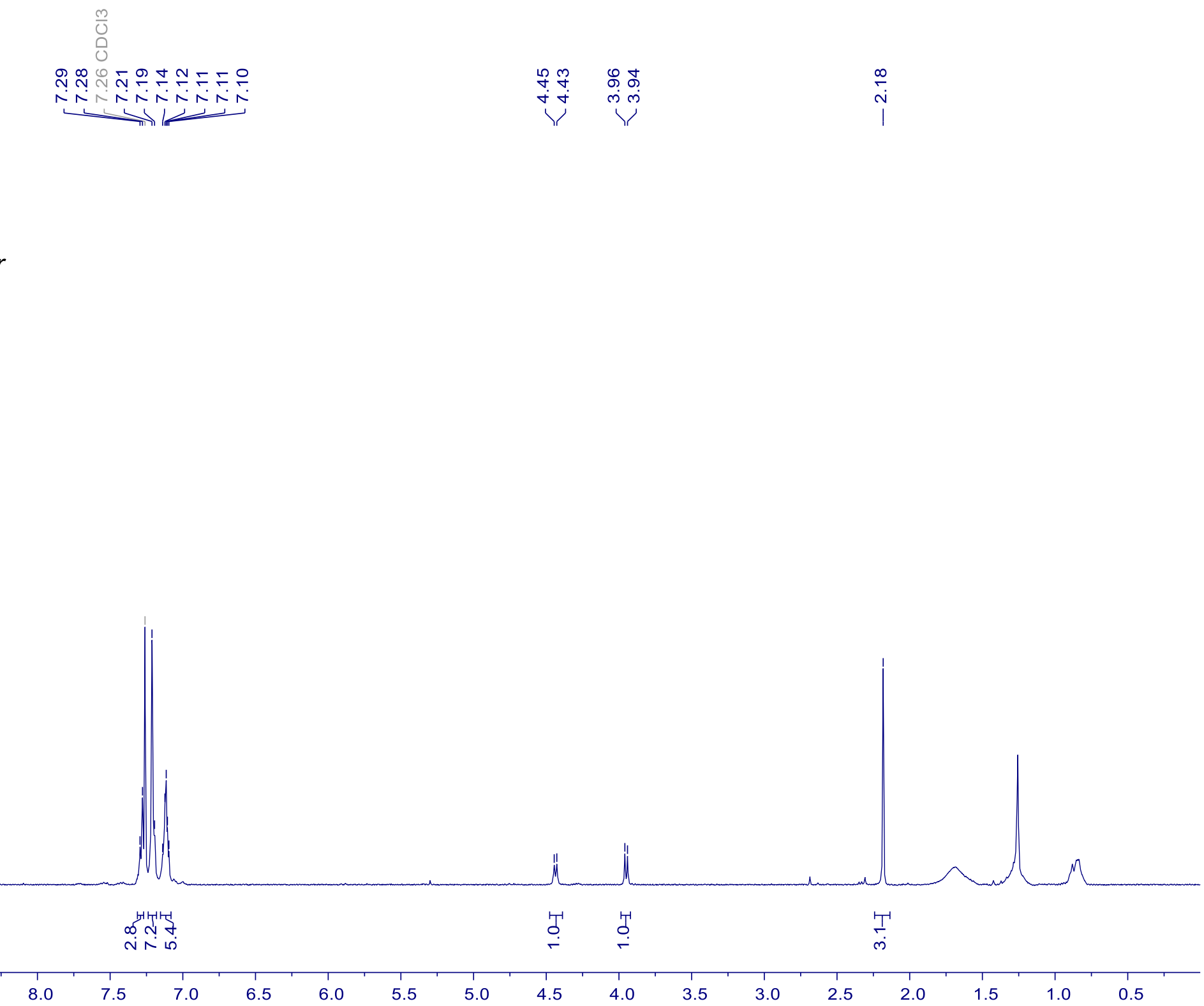

32c *(minor diasteroisomer)* – ^{13}C NMR (101 MHz, $CDCl_3$)

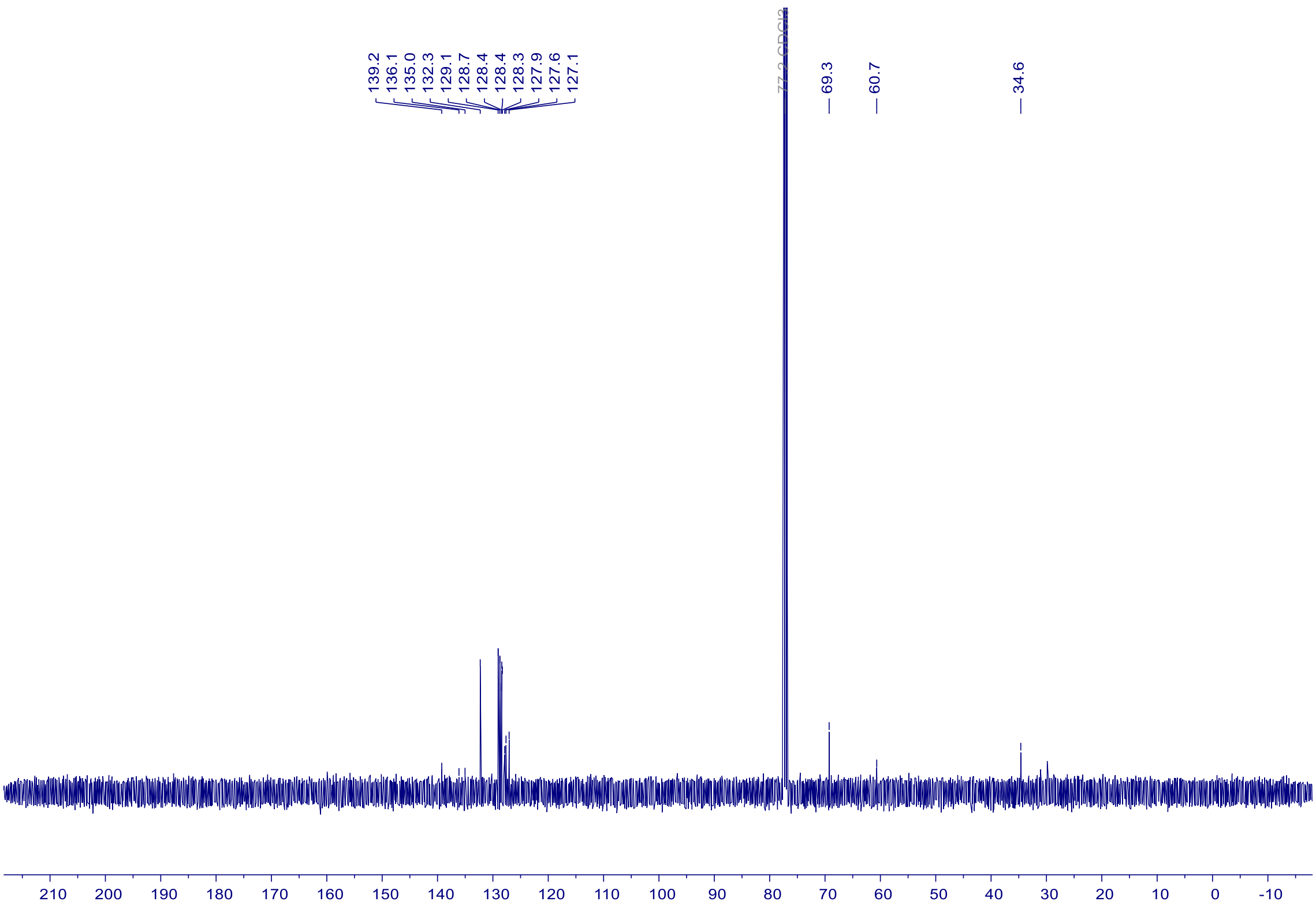

33c – 1H NMR (400 MHz, $CDCl_3$)

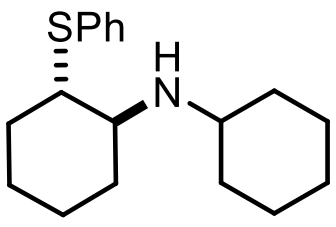

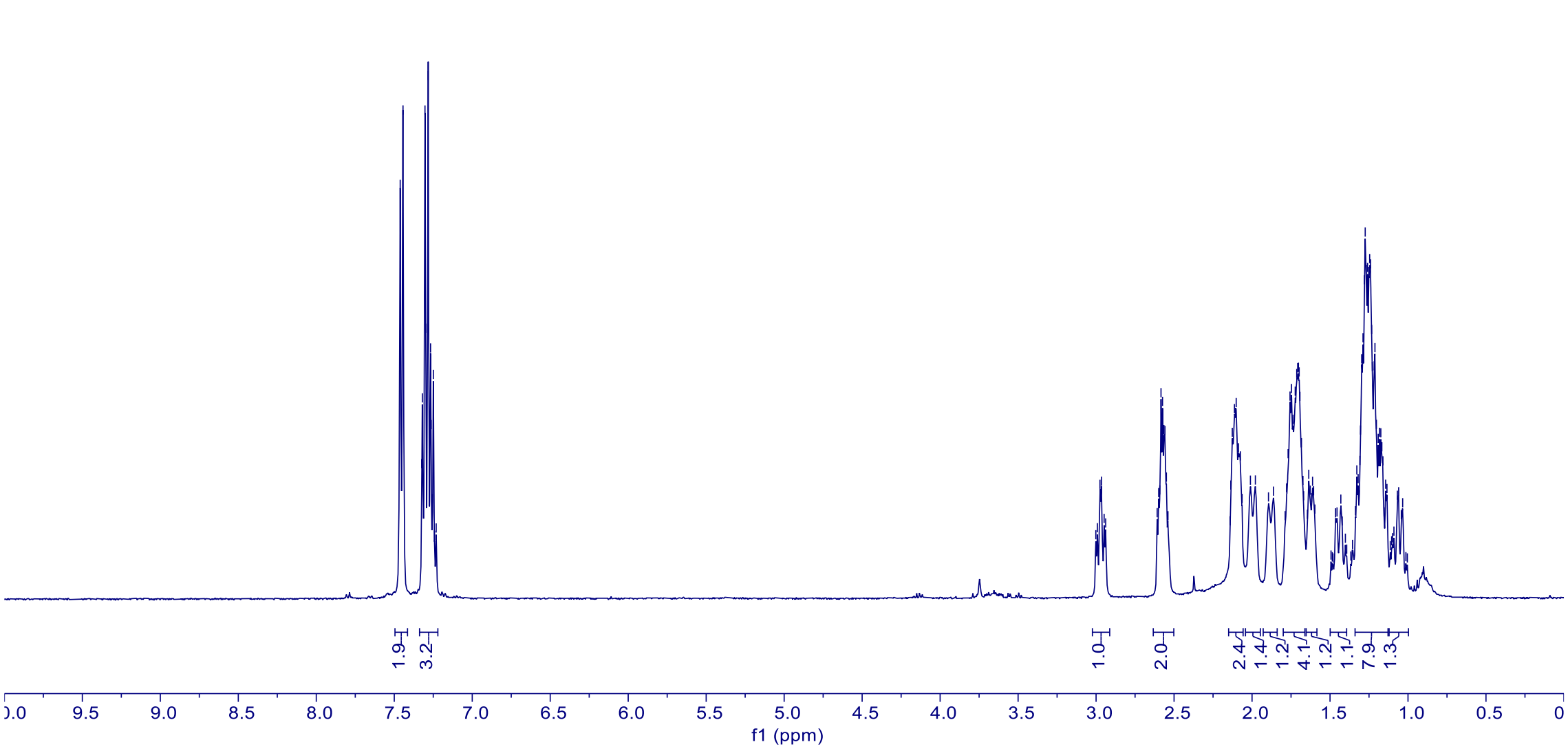

33c – 13C NMR (151 MHz, $CDCl_3$)

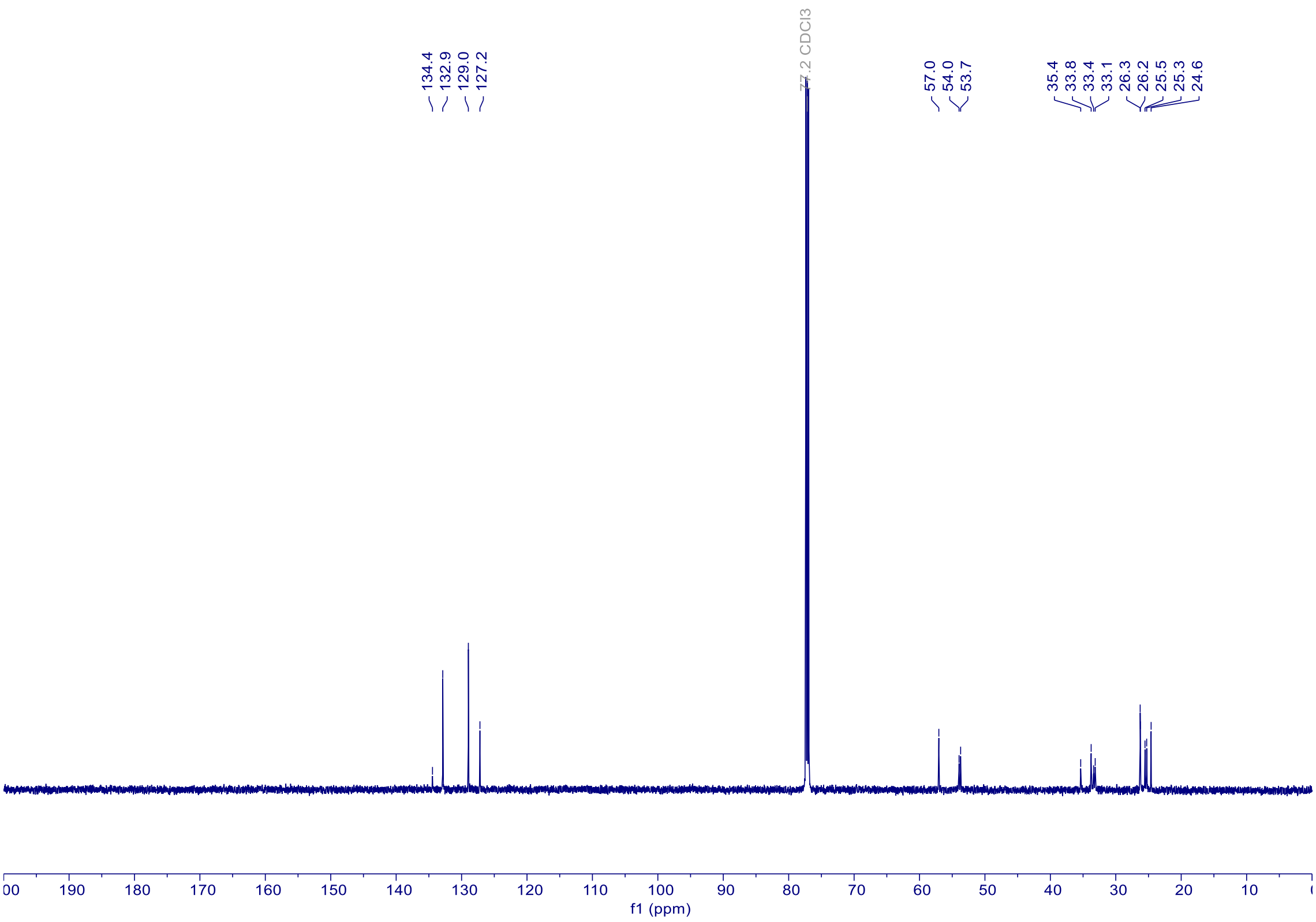

34c – ^{1}H NMR (400 MHz, $CDCl_3$)

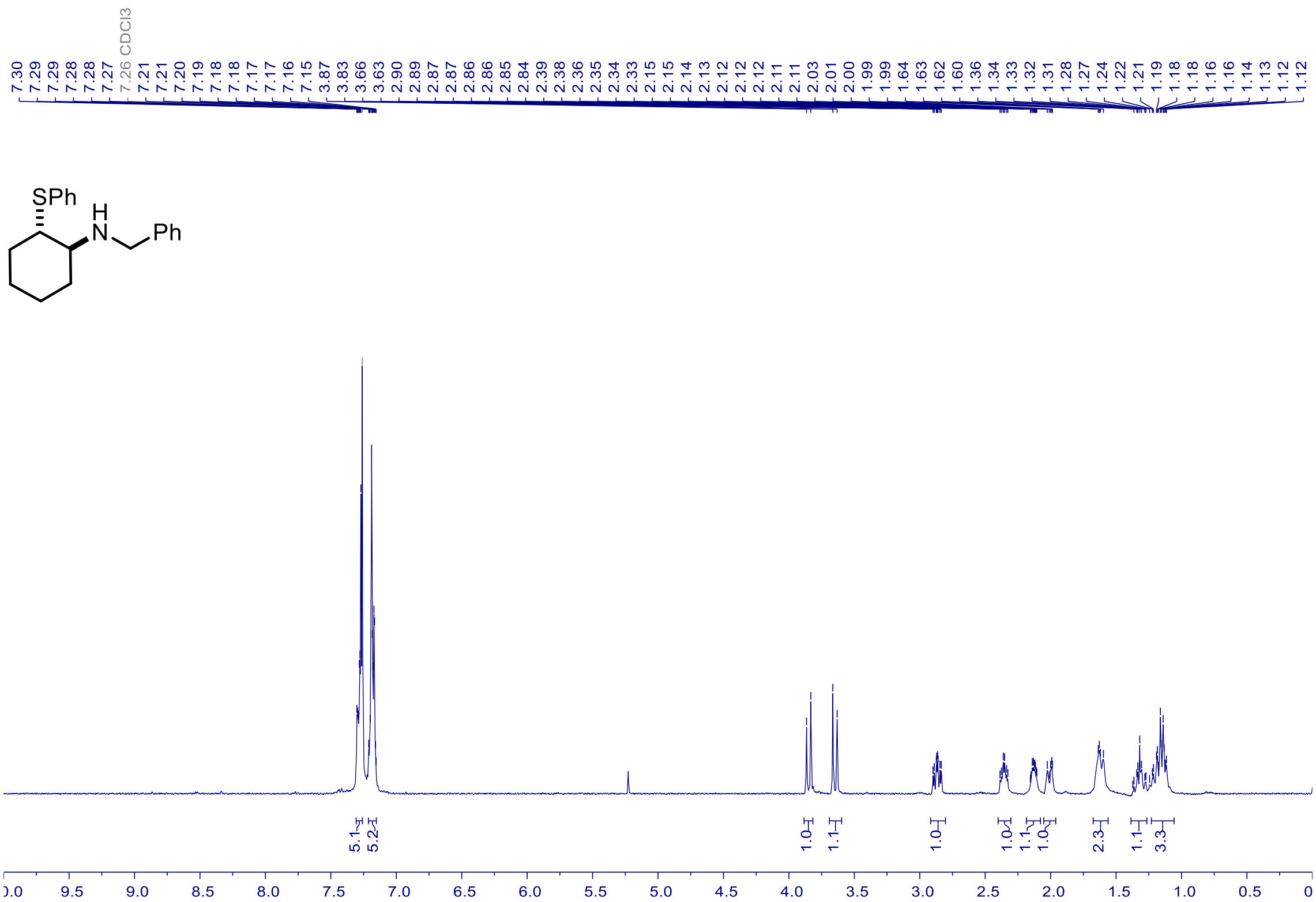

34c – ^{13}C NMR (101 MHz, $CDCl_3$)

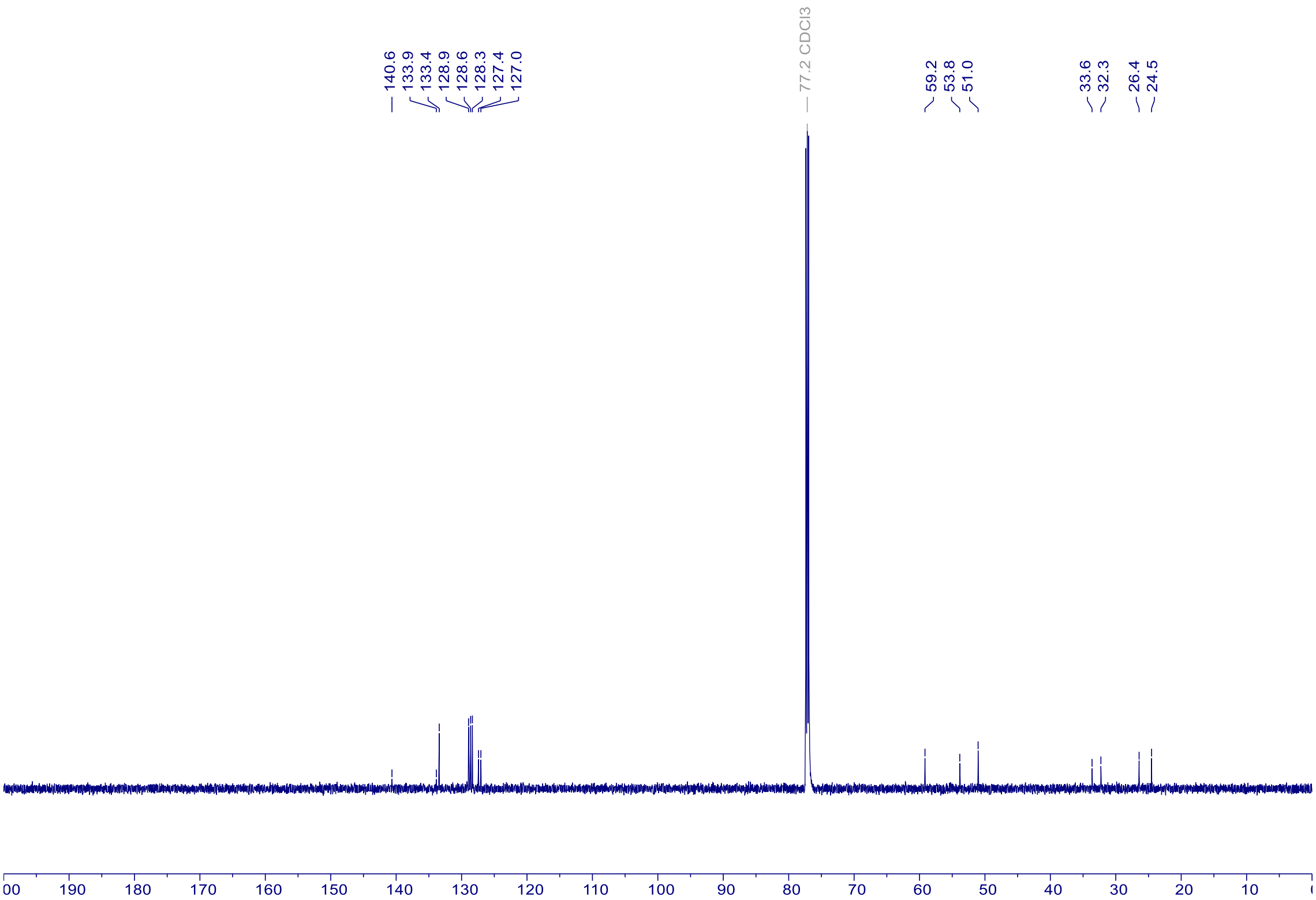

35c – ^{1}H NMR (600 MHz, $CDCl_3$)

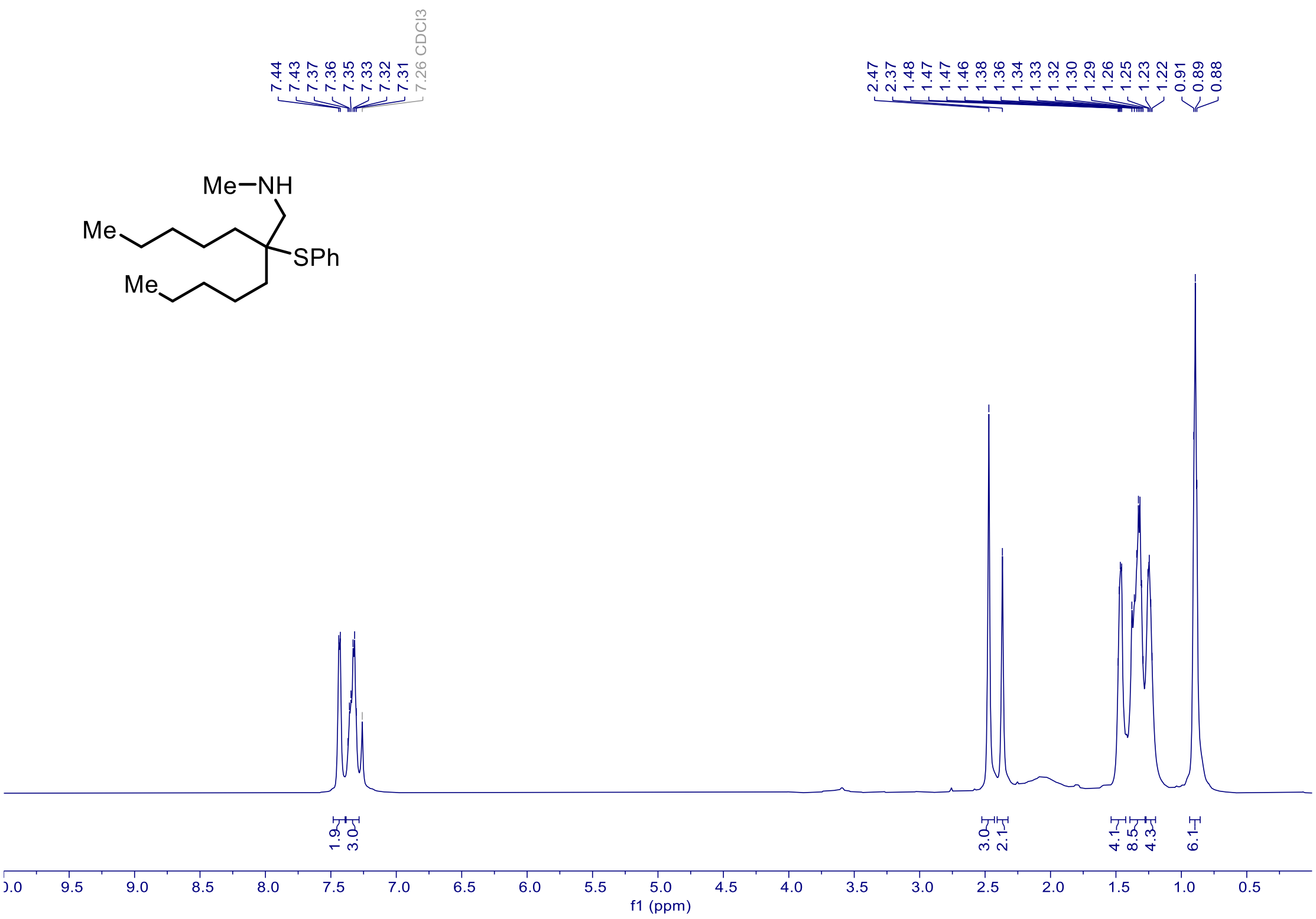

35c – ^{13}C NMR (151 MHz, $CDCl_3$)

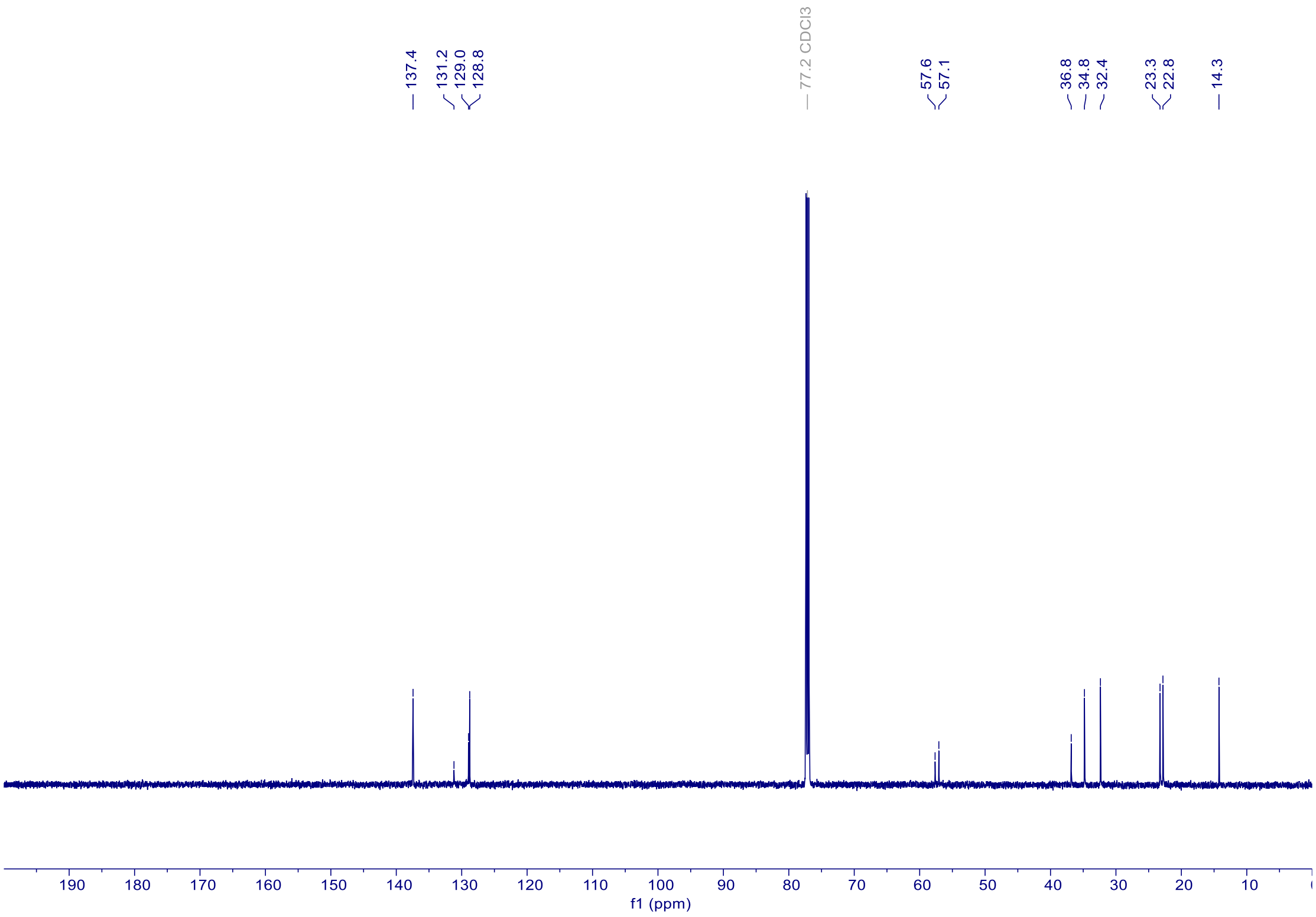

1s – ^{1}H NMR (400 MHz, $CDCl_3$)

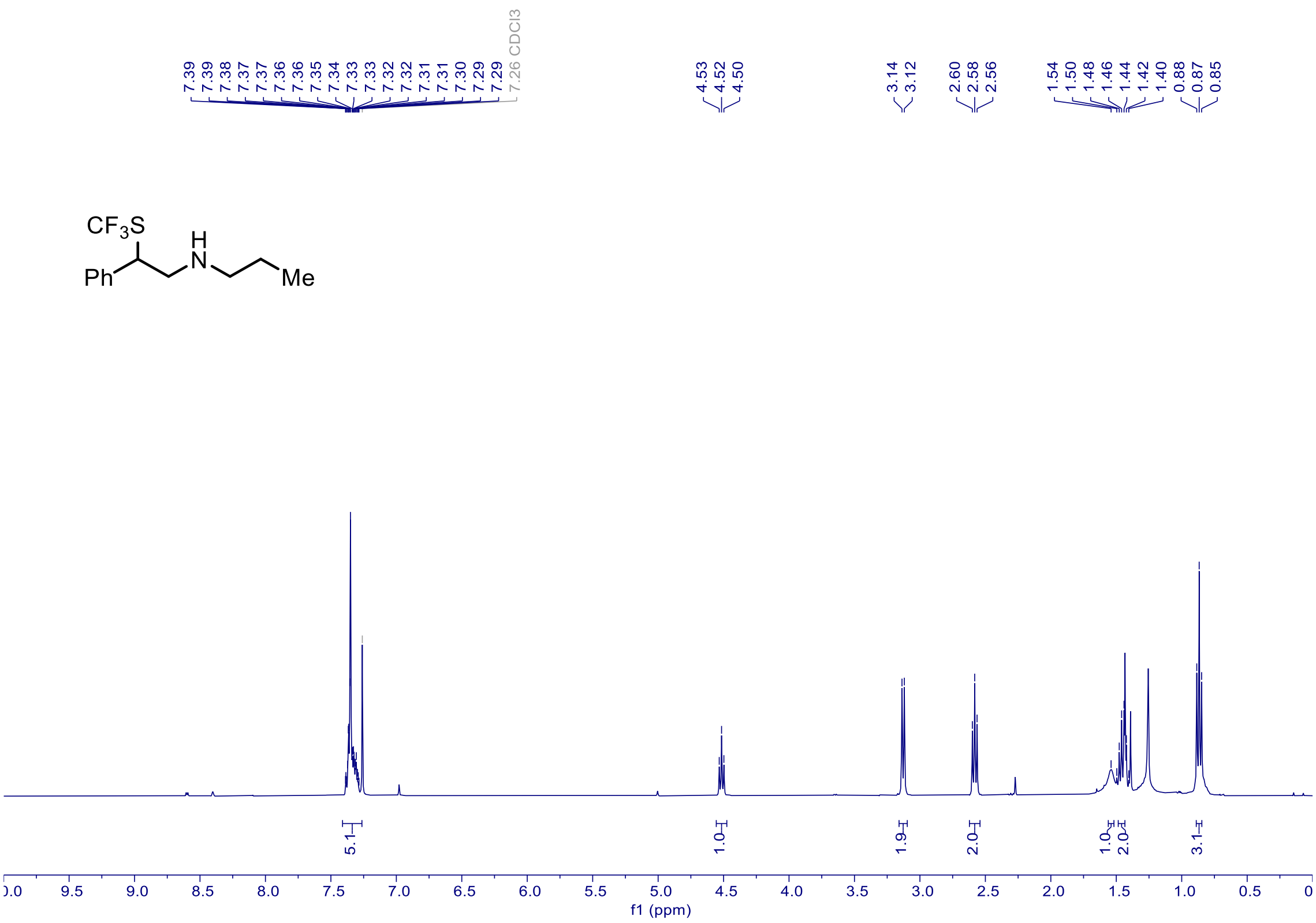

1s – ^{13}C NMR (101 MHz, $CDCl_3$)

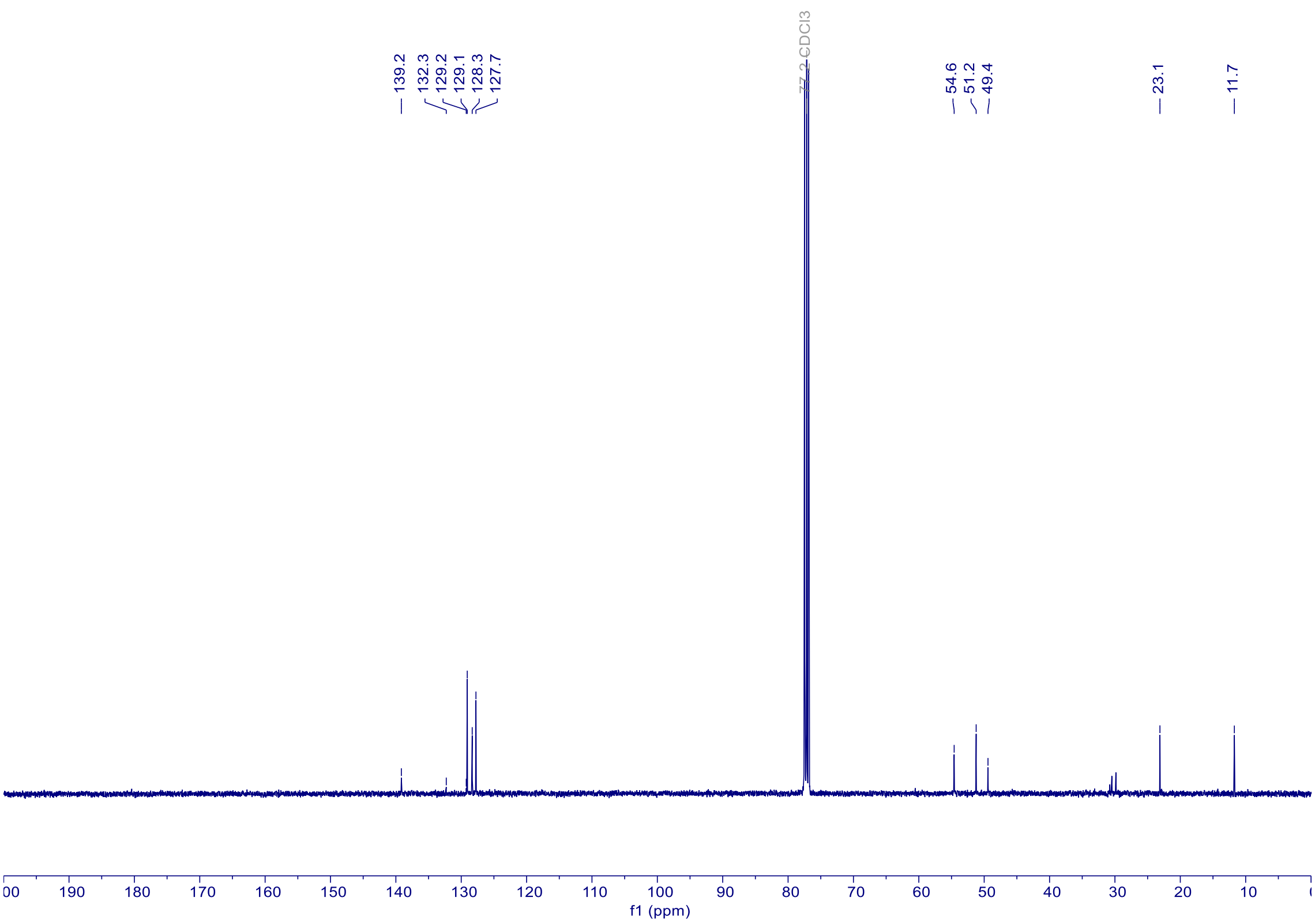

1s – ^{19}F NMR (376 MHz, $CDCl_3$)

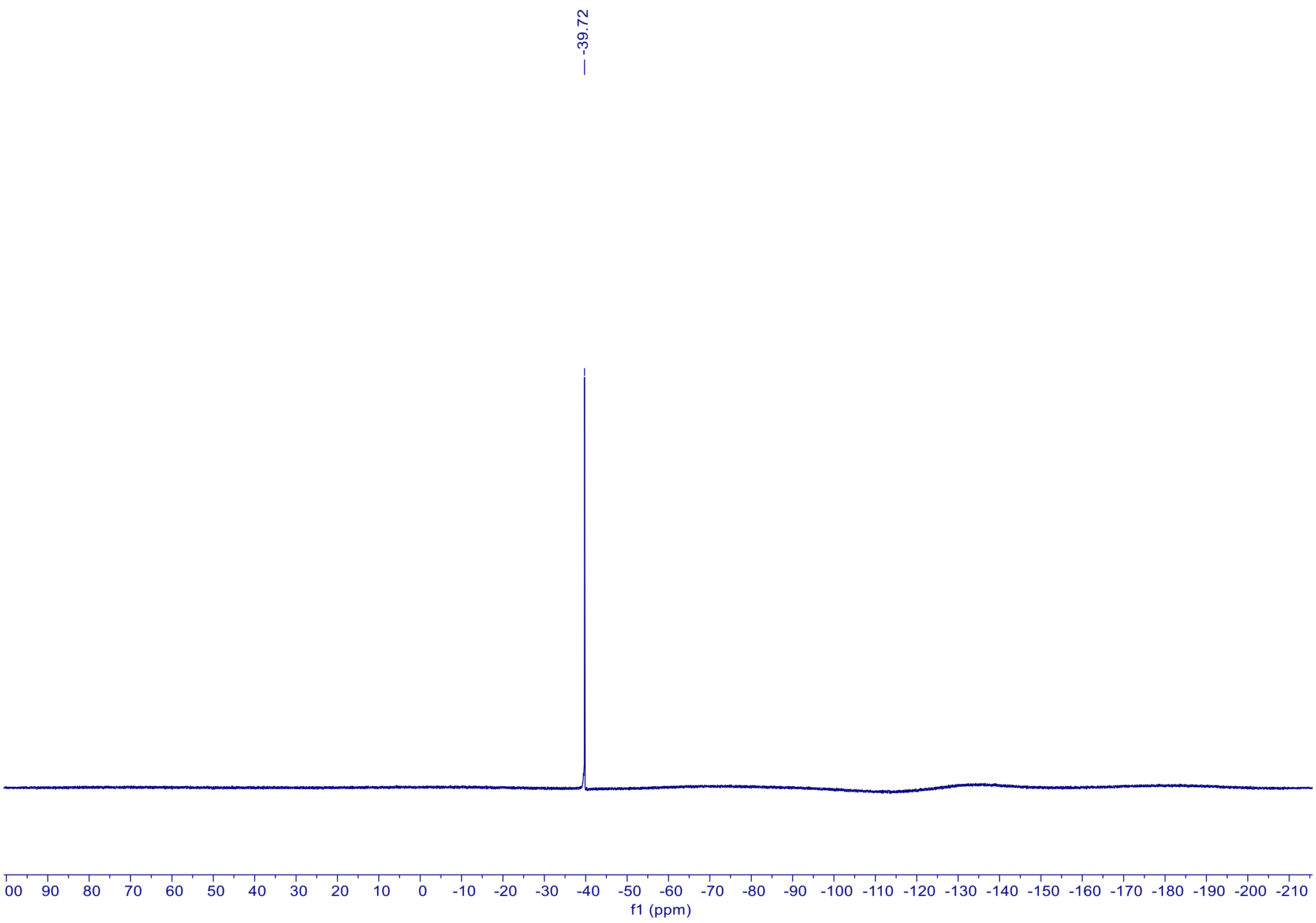

13g – ^{1}H NMR (600 MHz, $CDCl_3$)

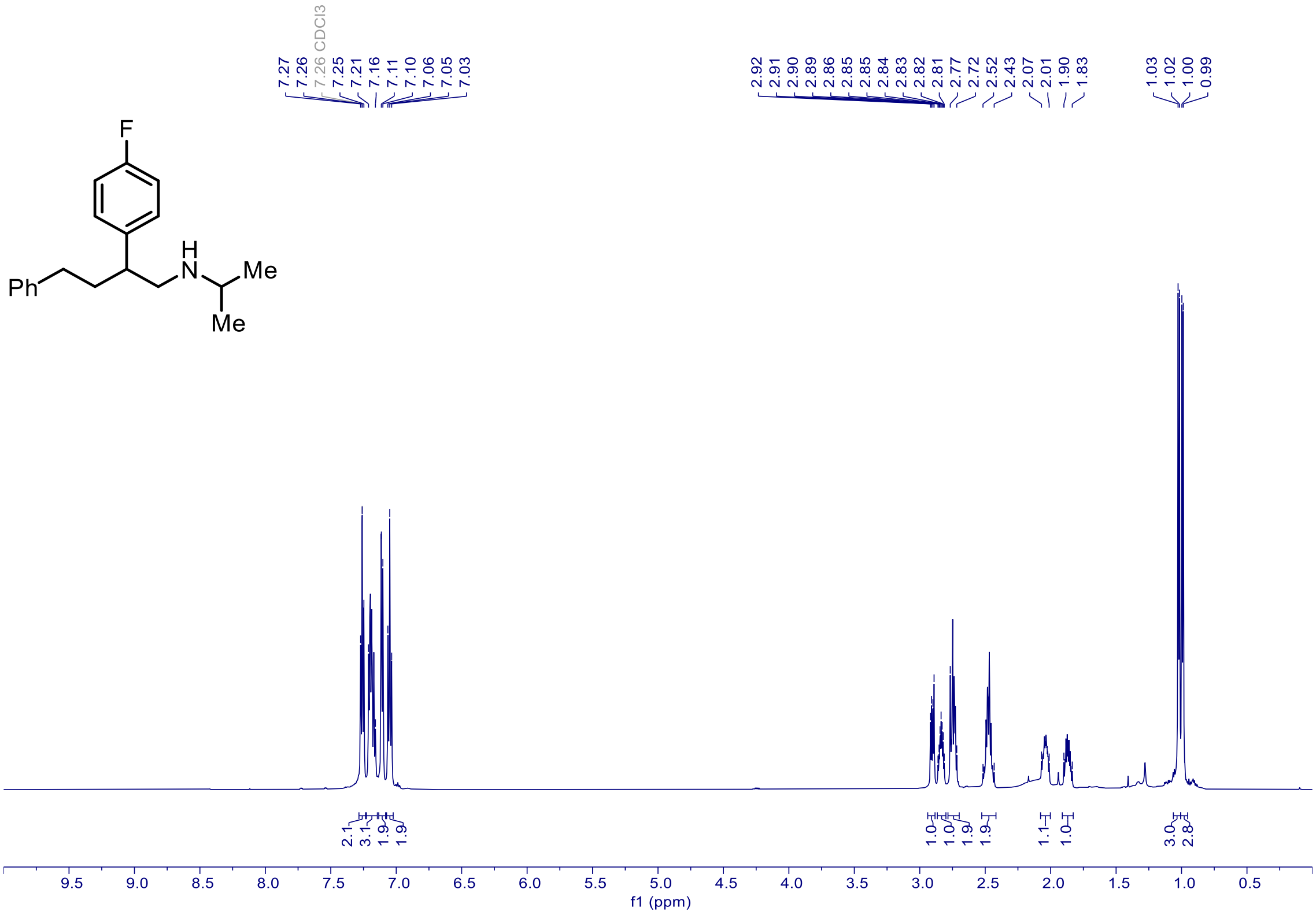

13g – ^{13}C NMR (151 MHz, $CDCl_3$)

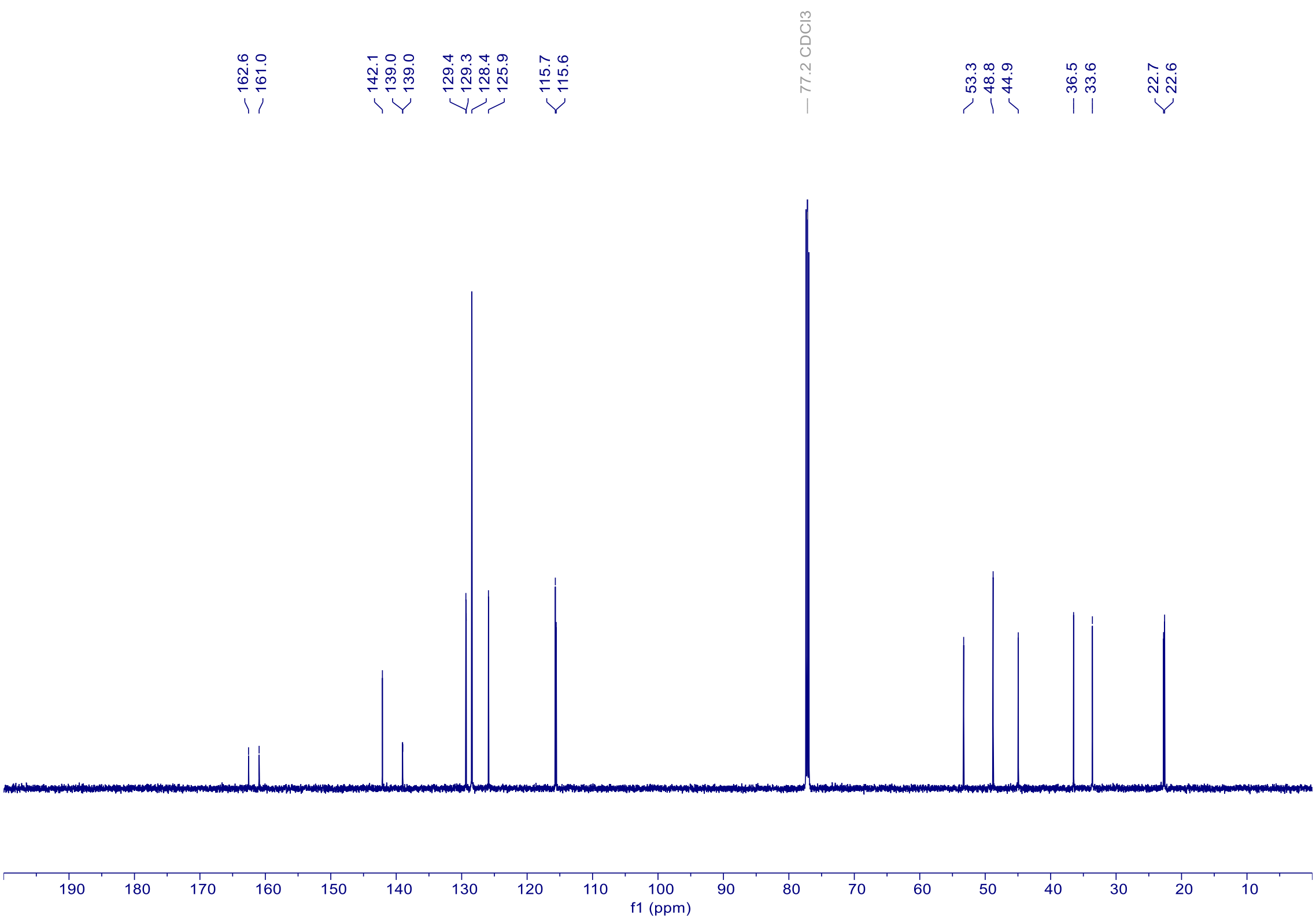

13g – ^{19}F NMR (564 MHz, $CDCl_3$)

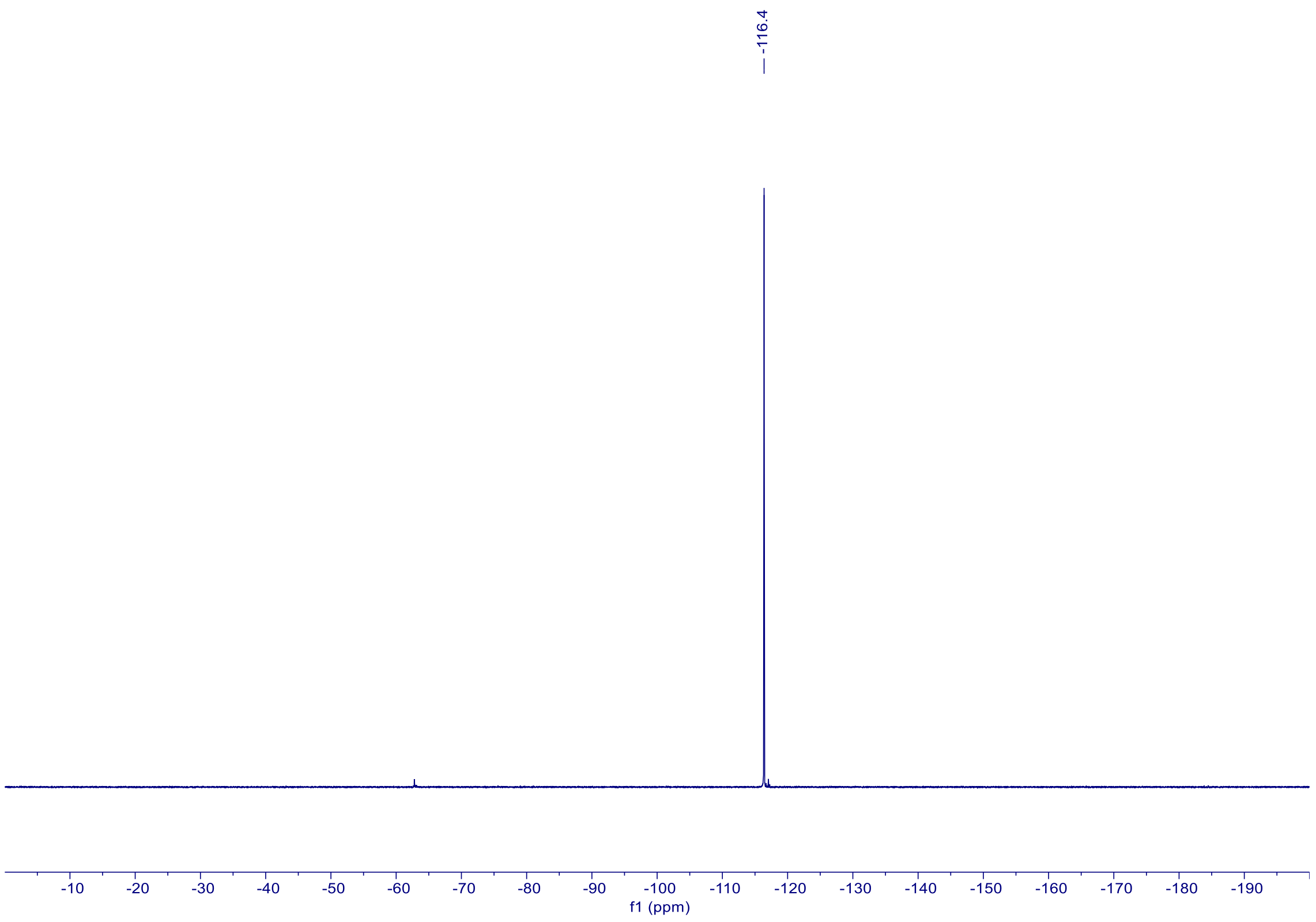

13h – ^{1}H NMR (600 MHz, $CDCl_3$)

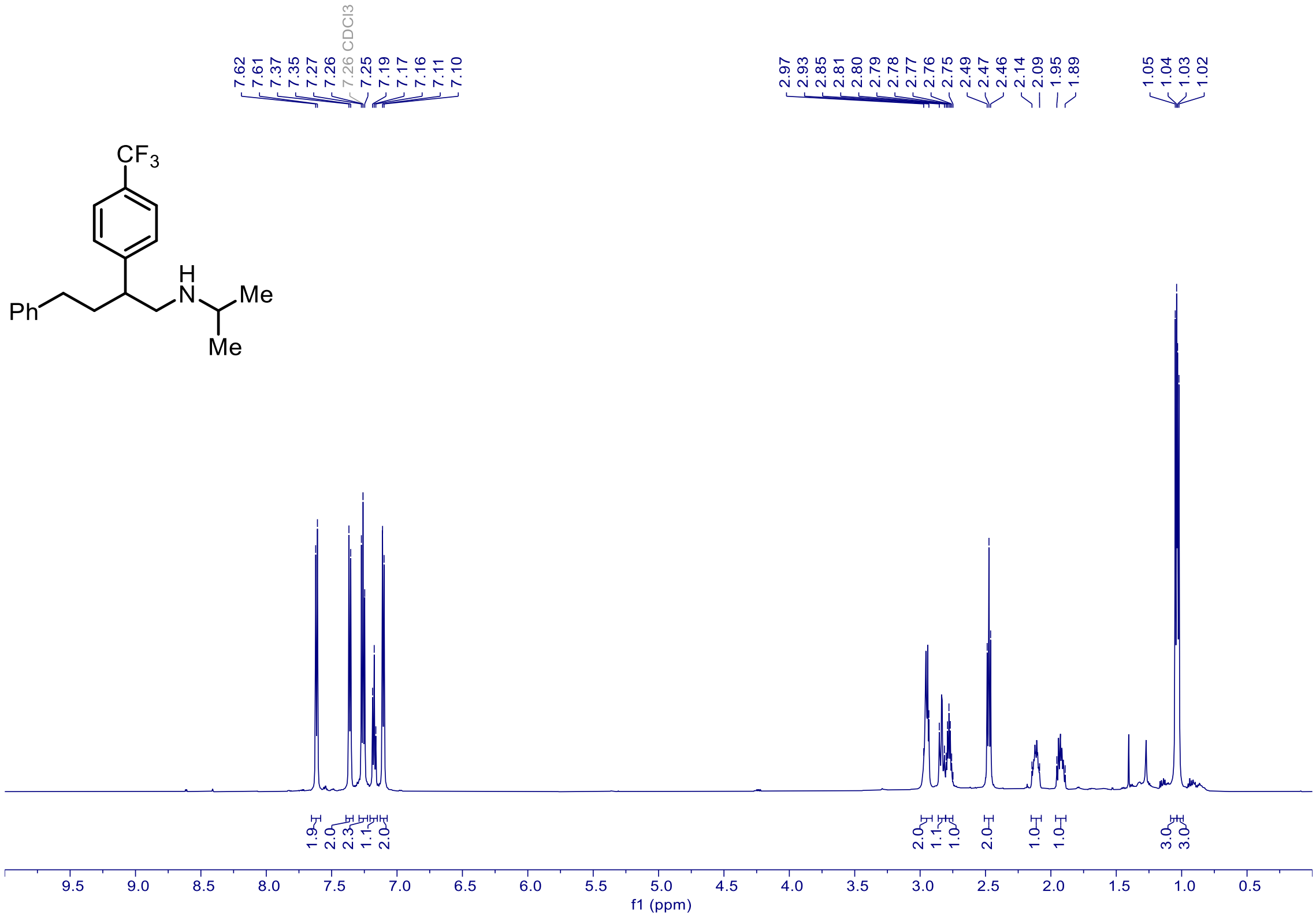

13h – ^{13}C NMR (151 MHz, $CDCl_3$)

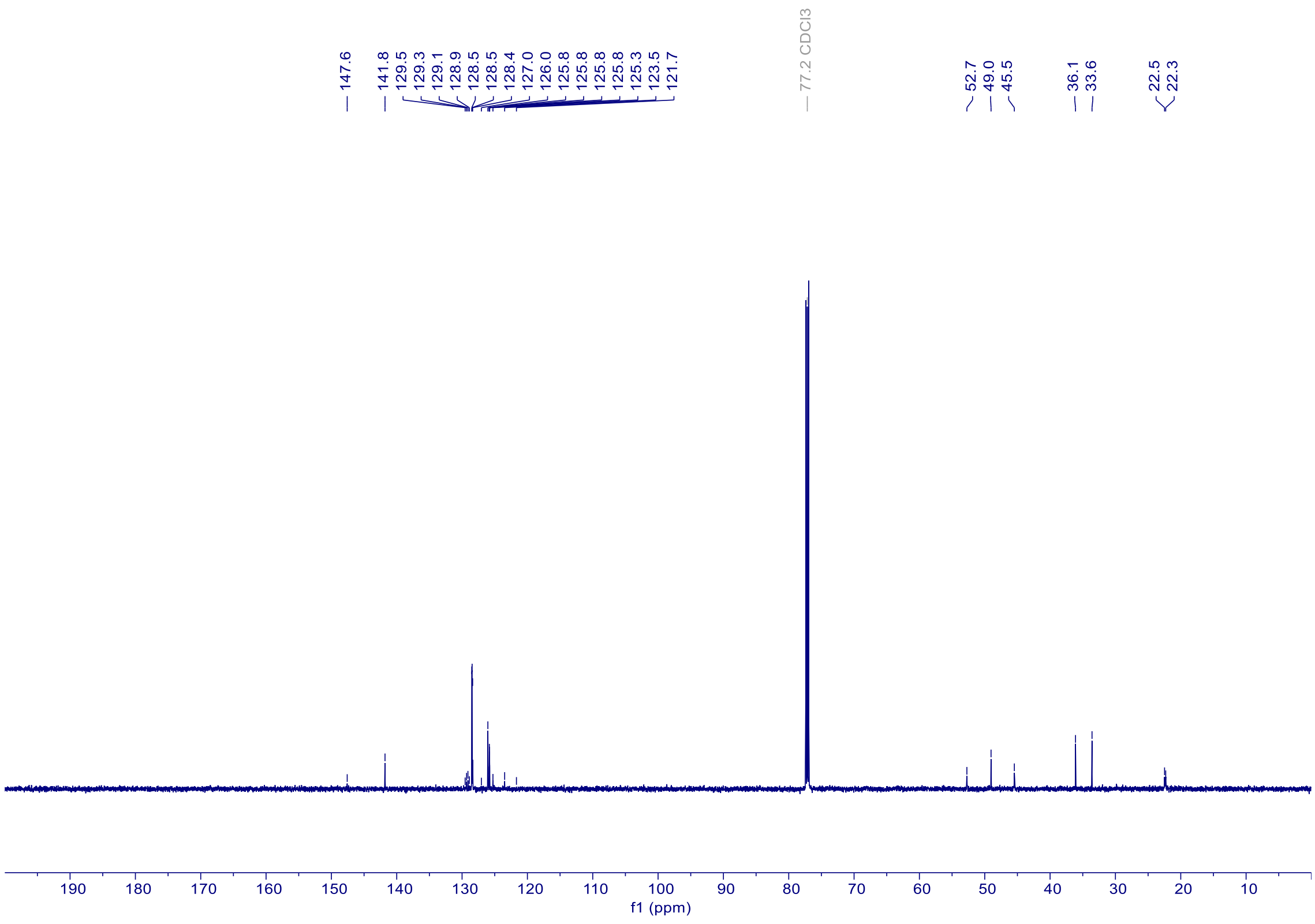

13h – ^{19}F NMR (564 MHz, $CDCl_3$)

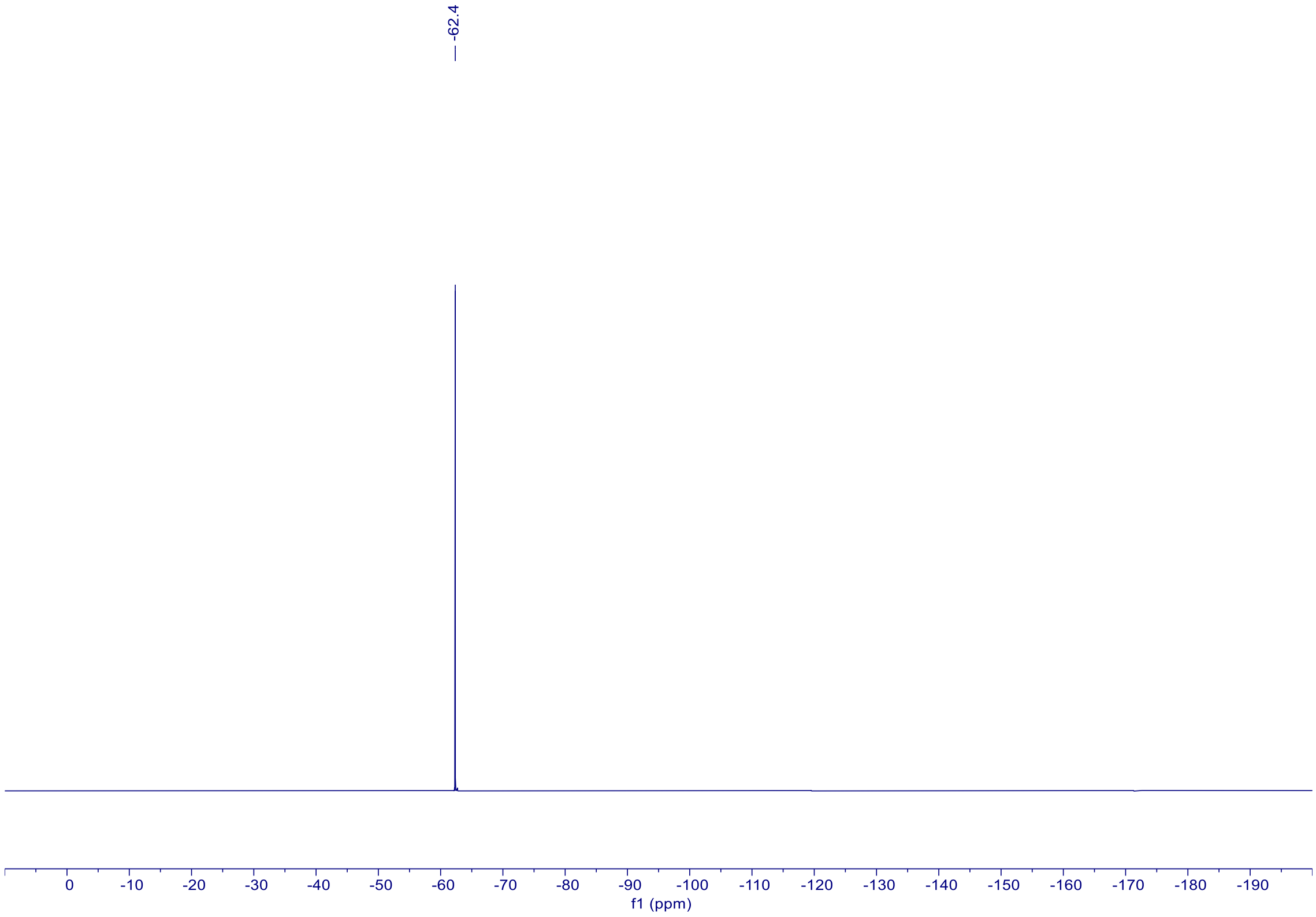

13i – ^{1}H NMR (600 MHz, $CDCl_3$)

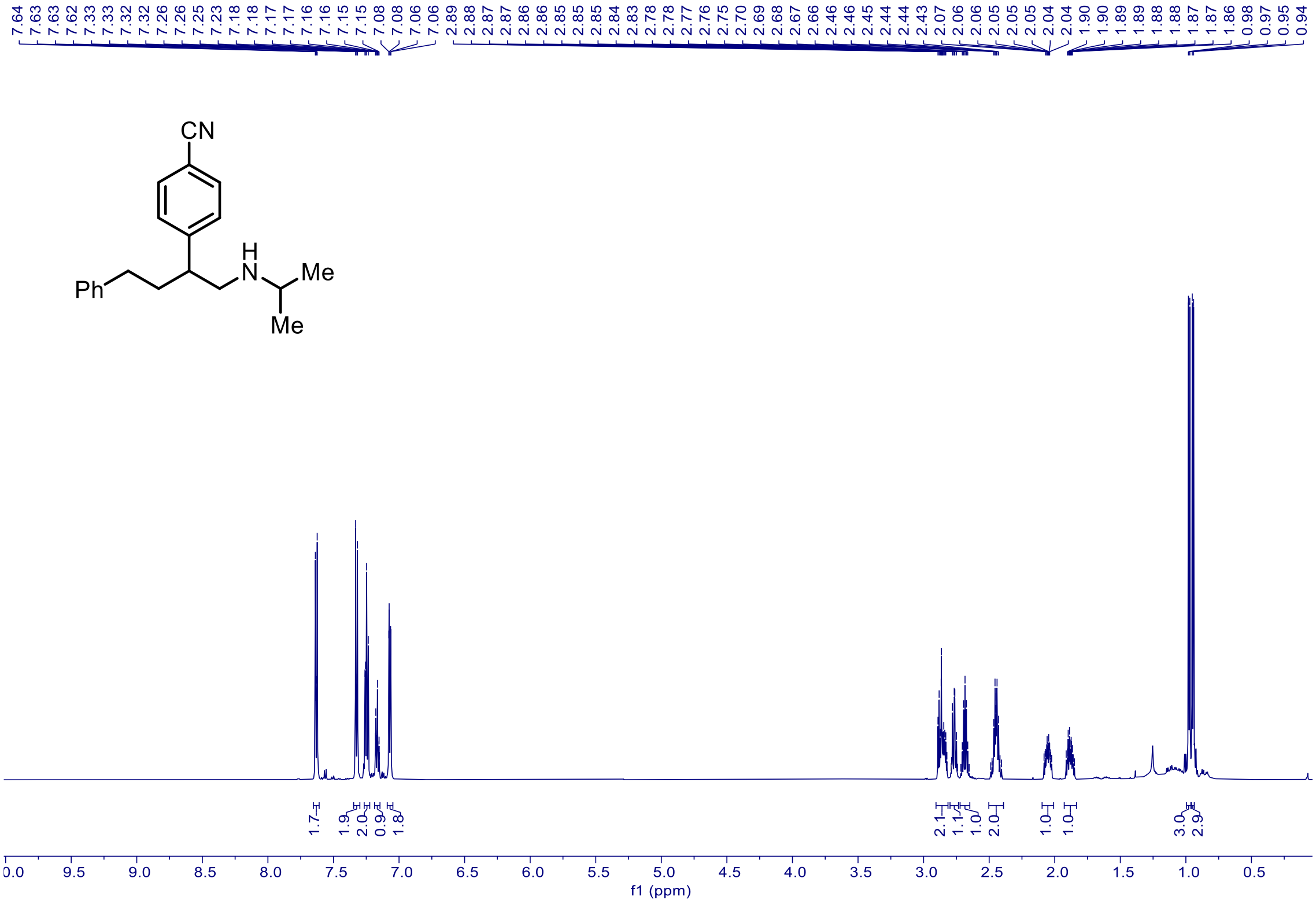

13i – ^{13}C NMR (151 MHz, $CDCl_3$)

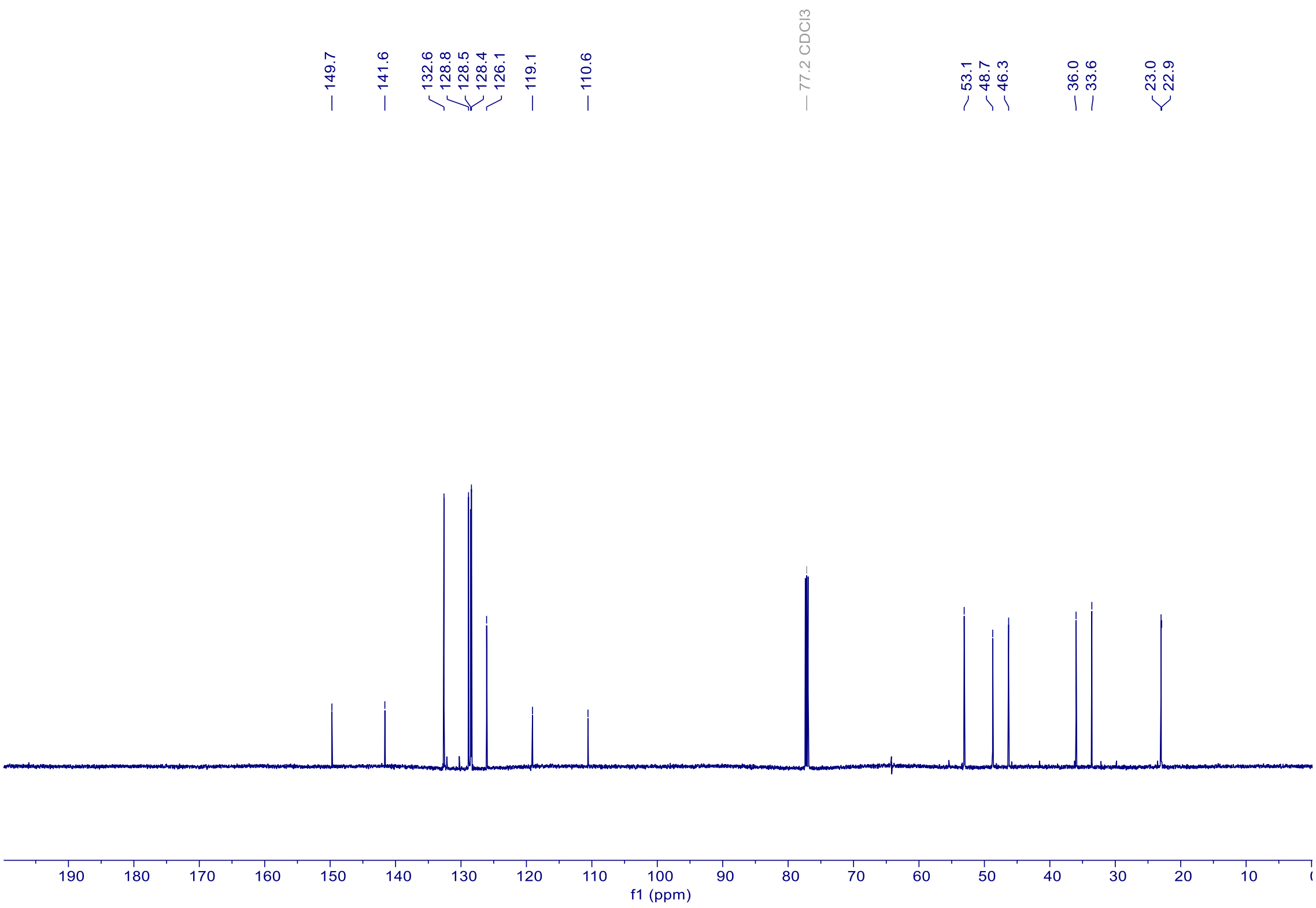

13j – ^{1}H NMR (600 MHz, $CDCl_3$)

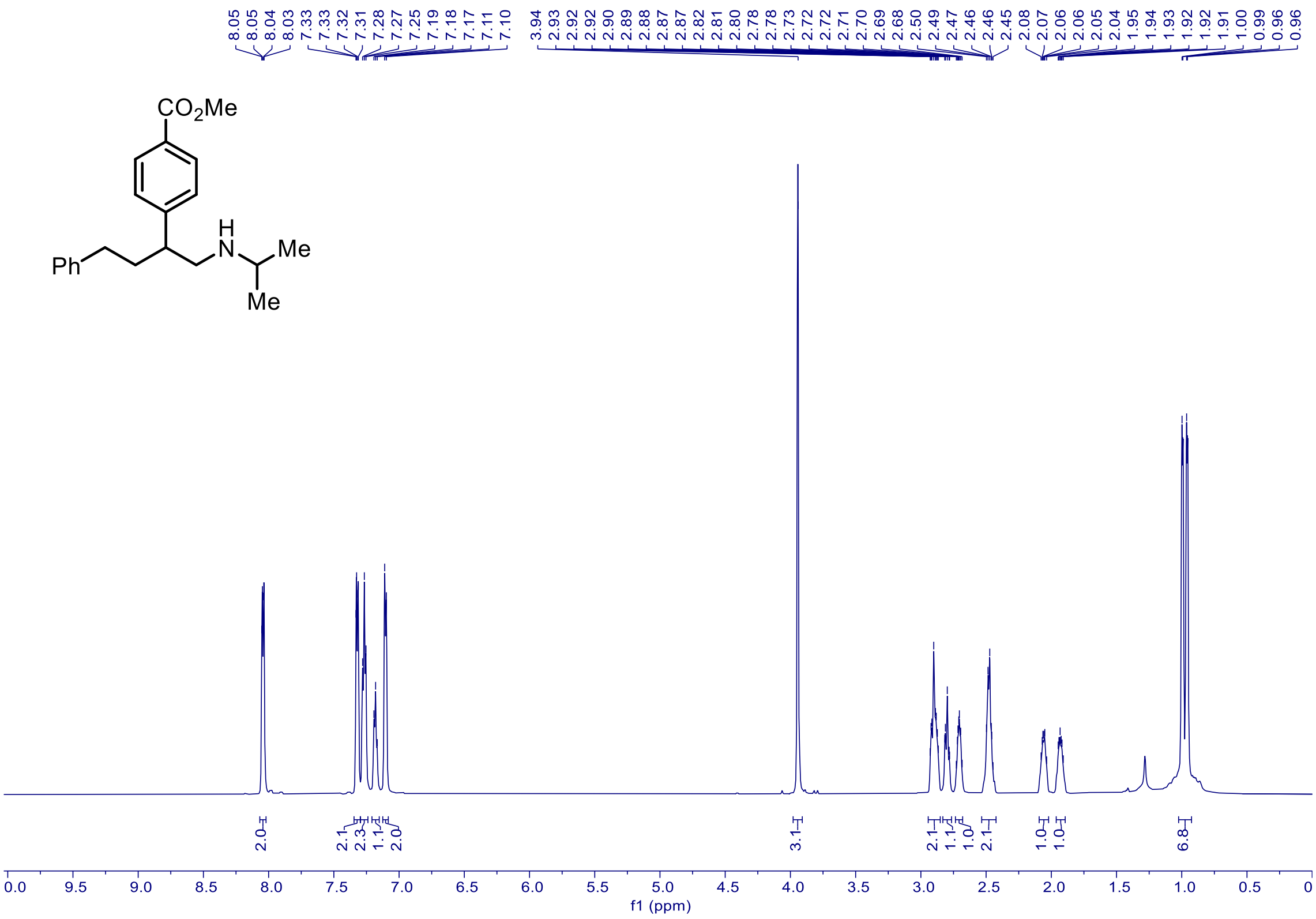

13j – ^{13}C NMR (151 MHz, $CDCl_3$)

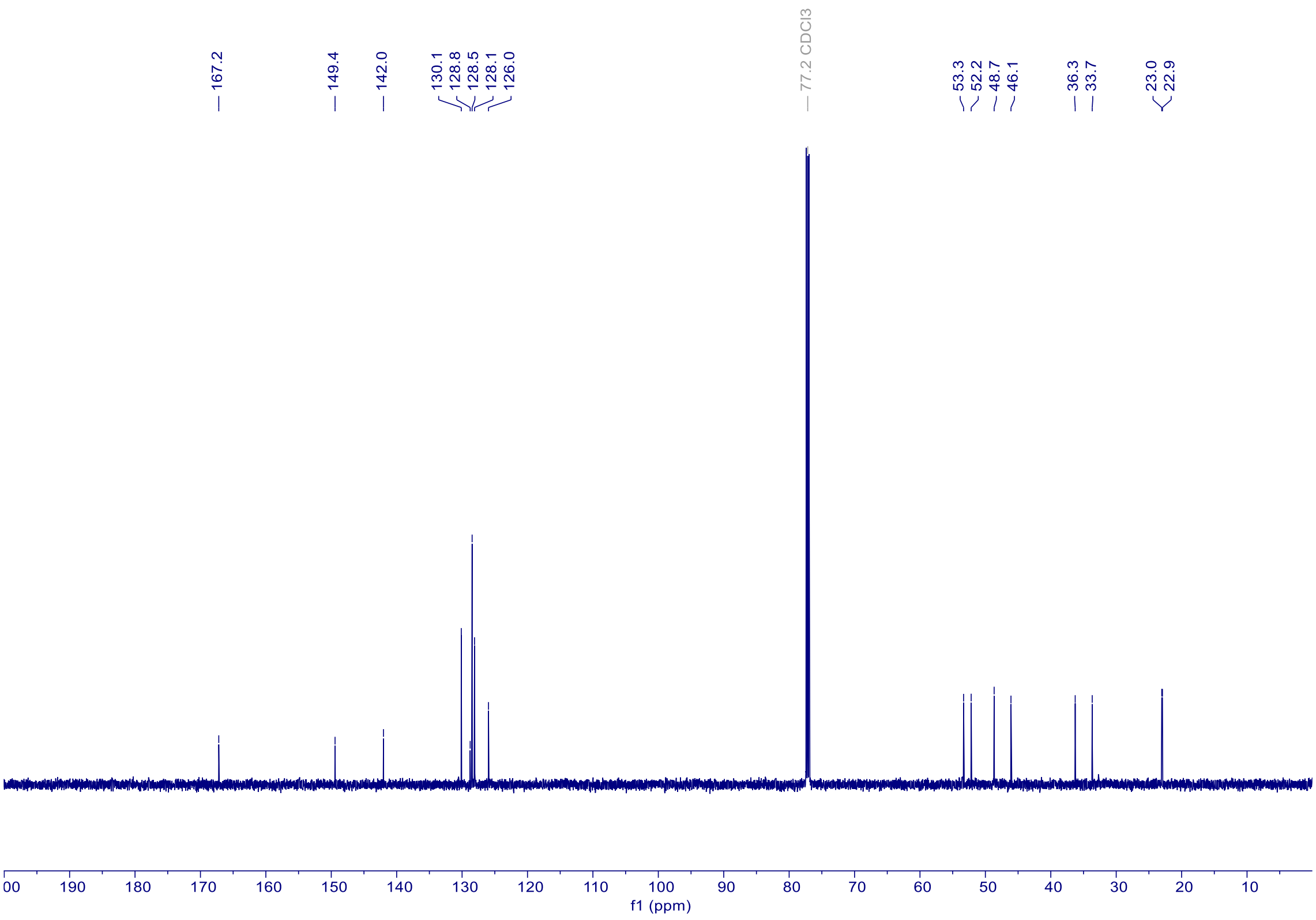

13k – ^{1}H NMR (600 MHz, $CDCl_3$)

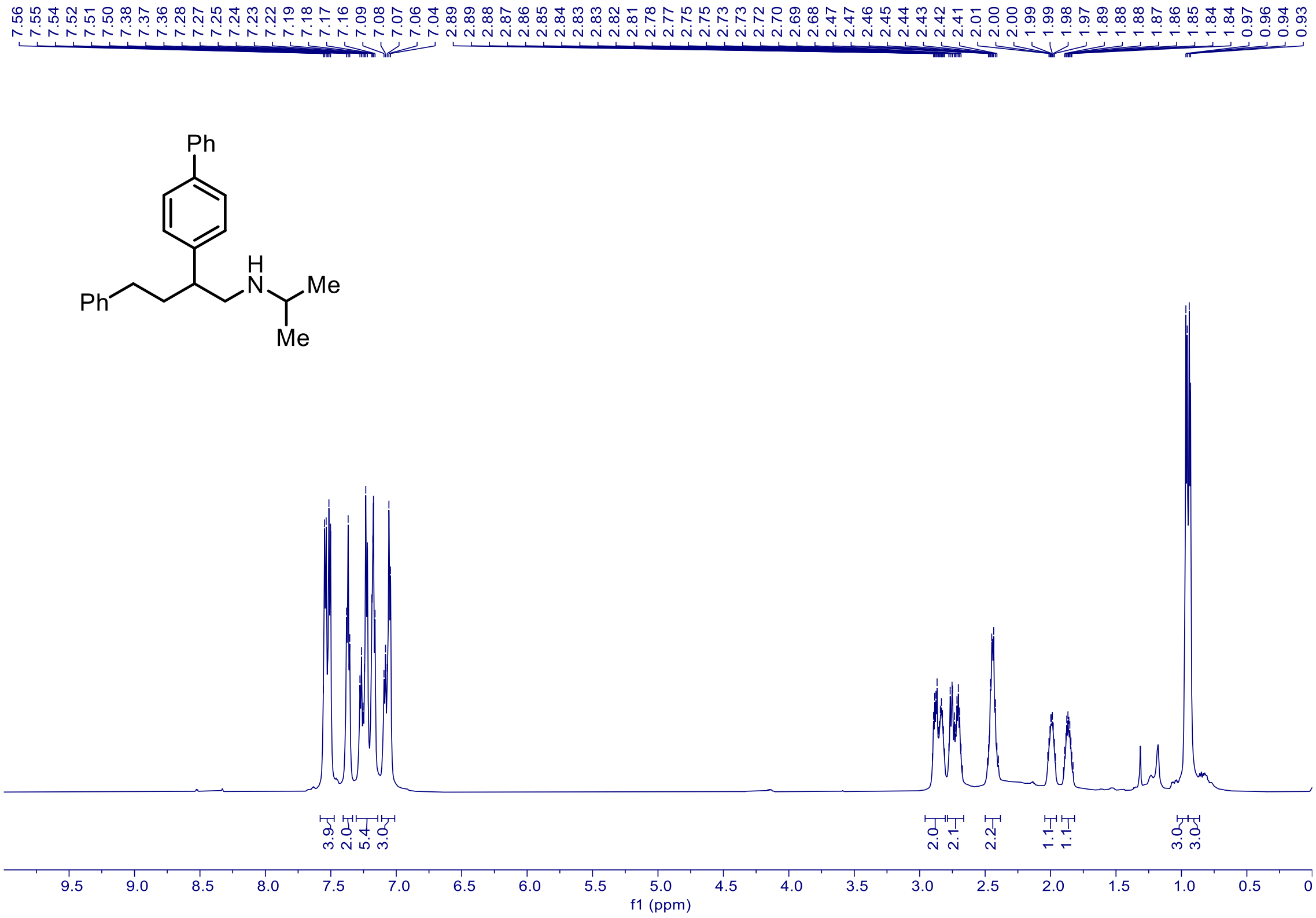

13k – ^{13}C NMR (151 MHz, $CDCl_3$)

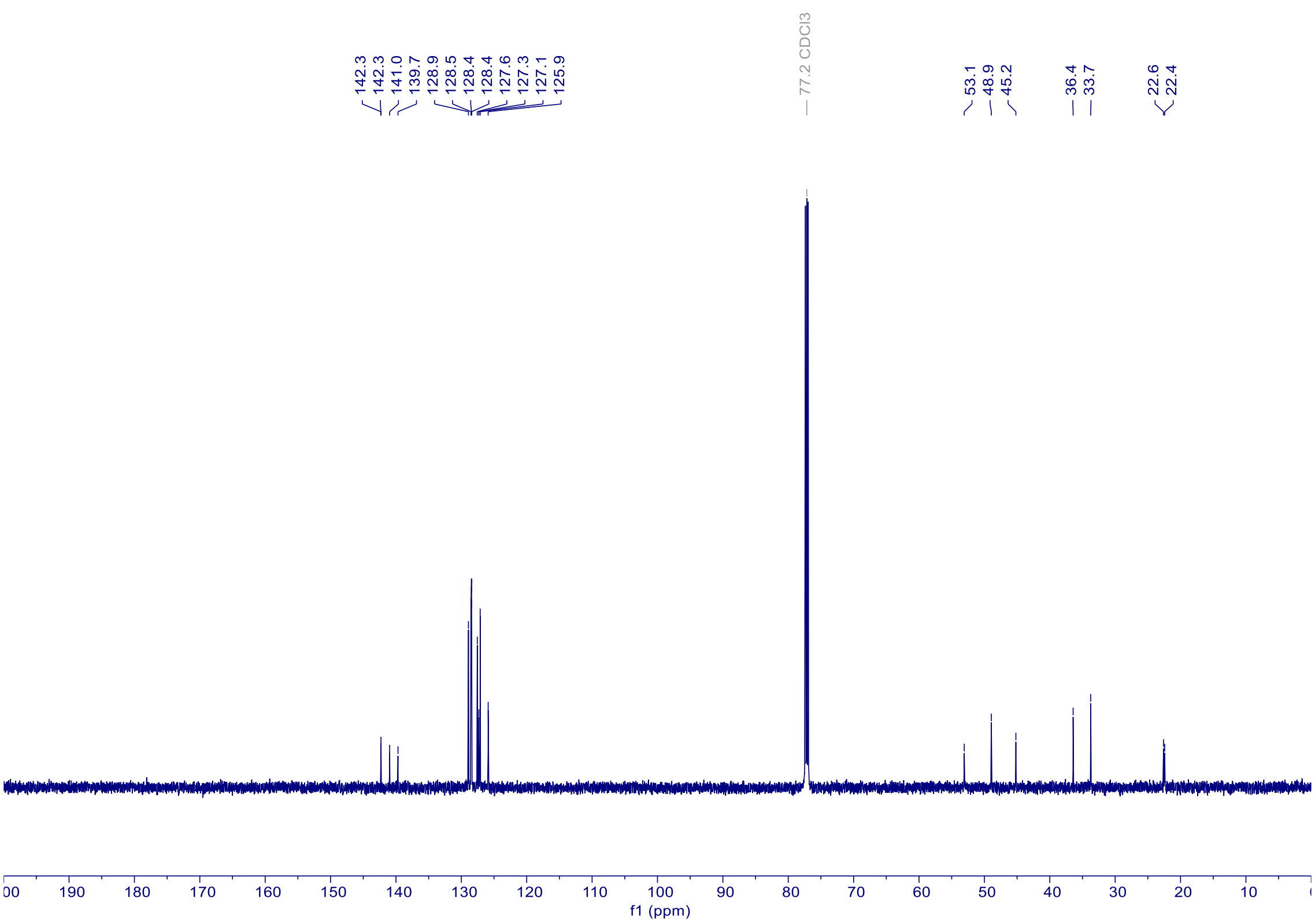

13l – ^{1}H NMR (400 MHz, $CDCl_3$)

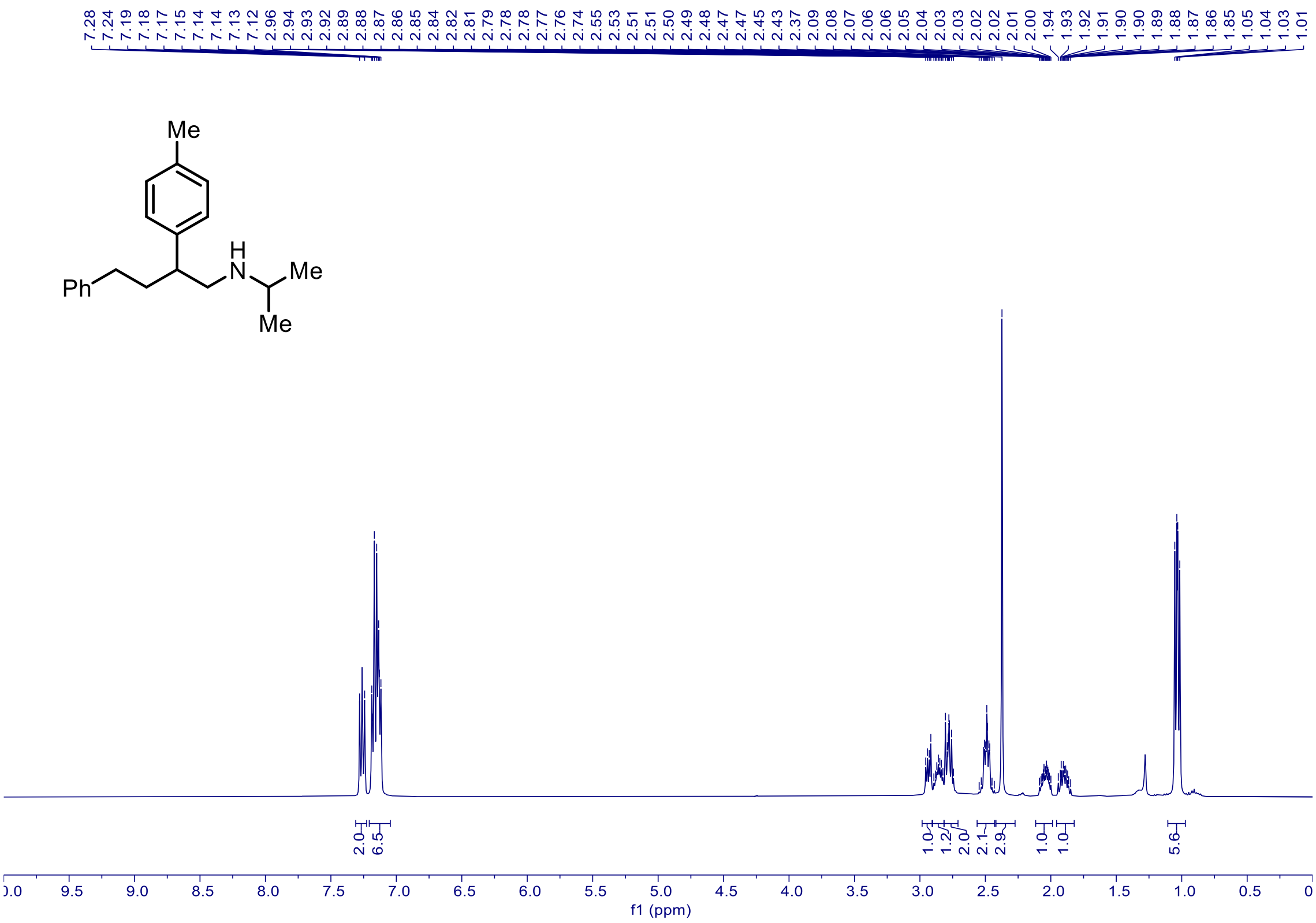

13l – ^{13}C NMR (101 MHz, $CDCl_3$)

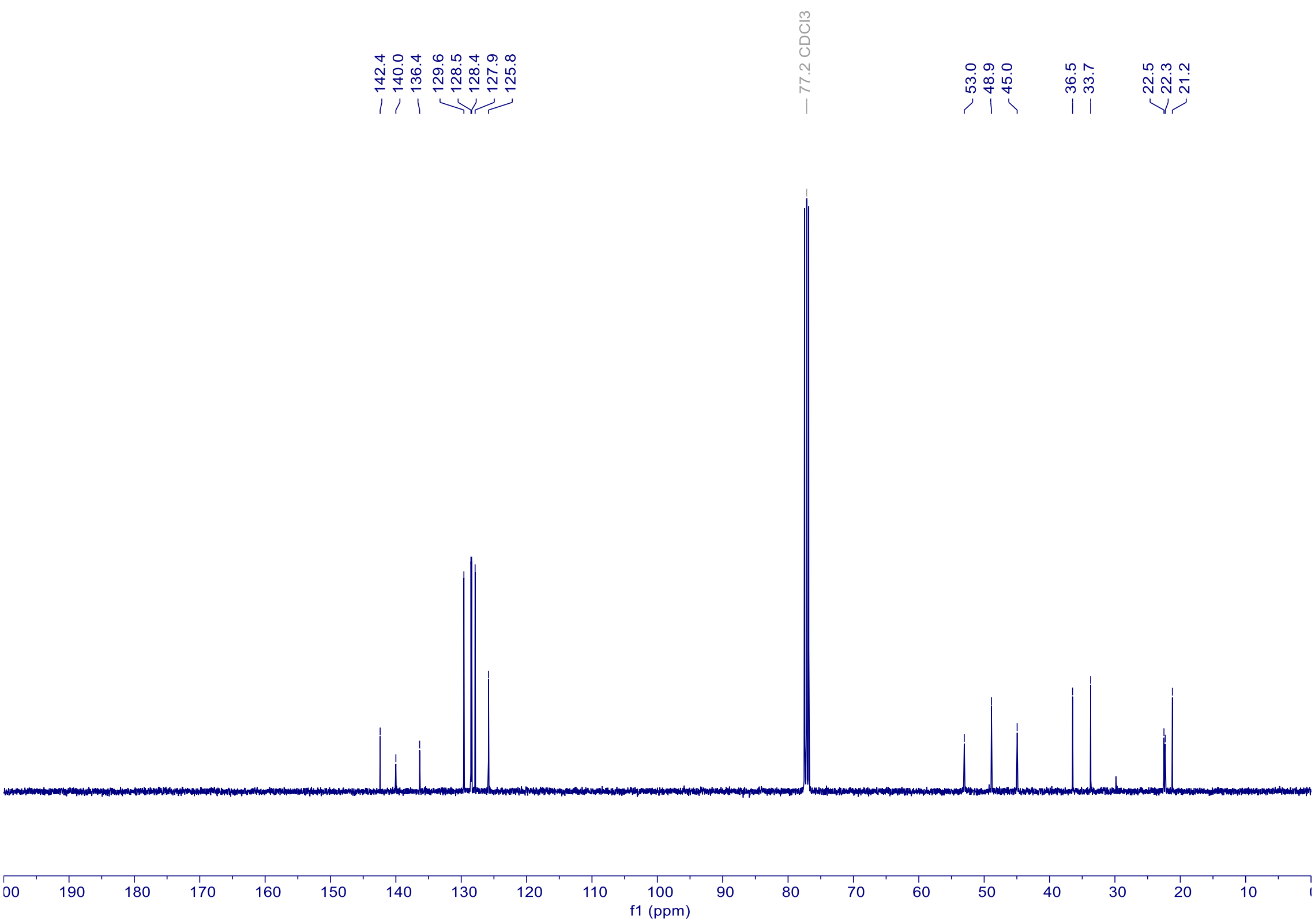

13m – ^{1}H NMR (600 MHz, $CDCl_3$)

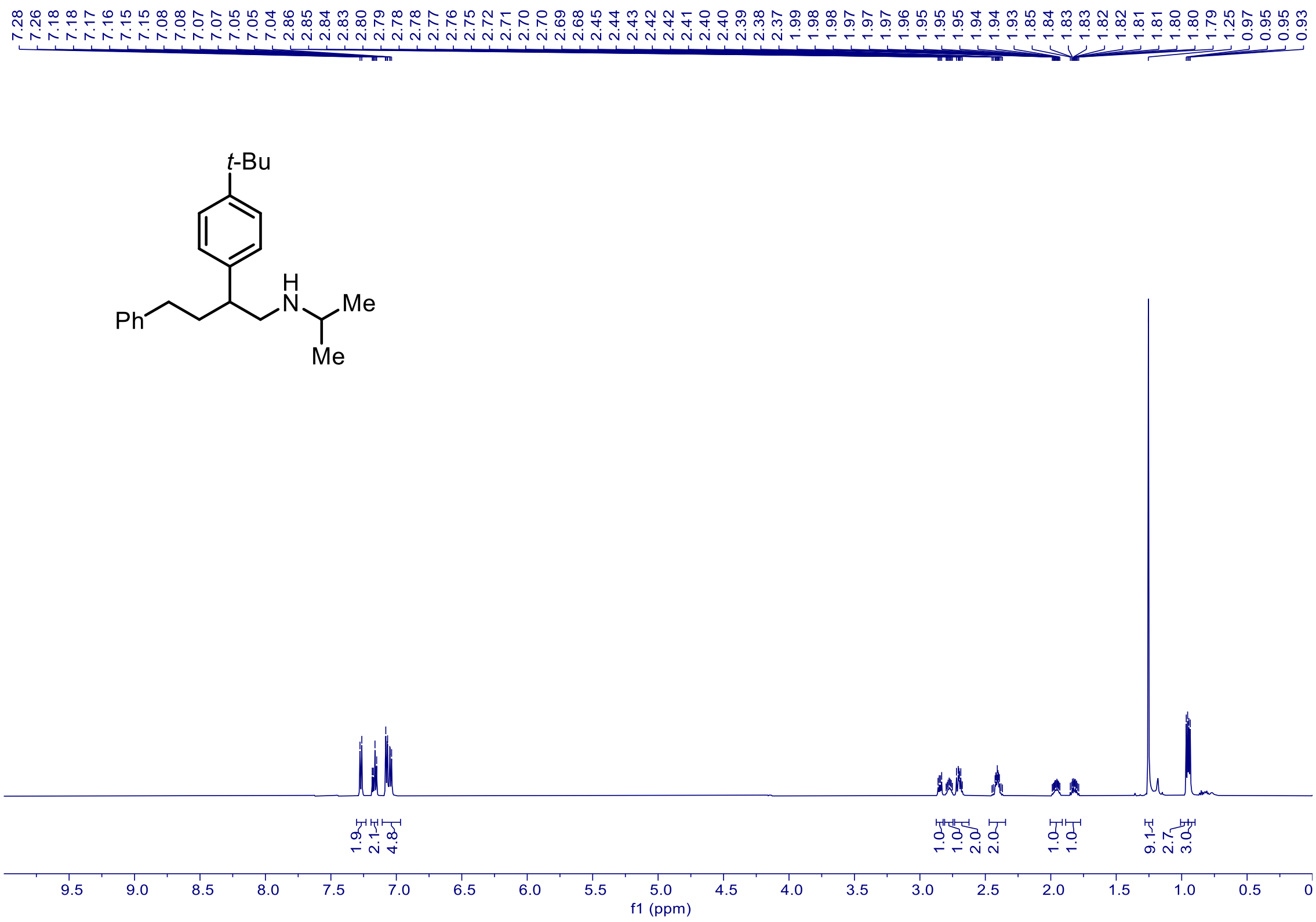

13m – ^{13}C NMR (151 MHz, $CDCl_3$)

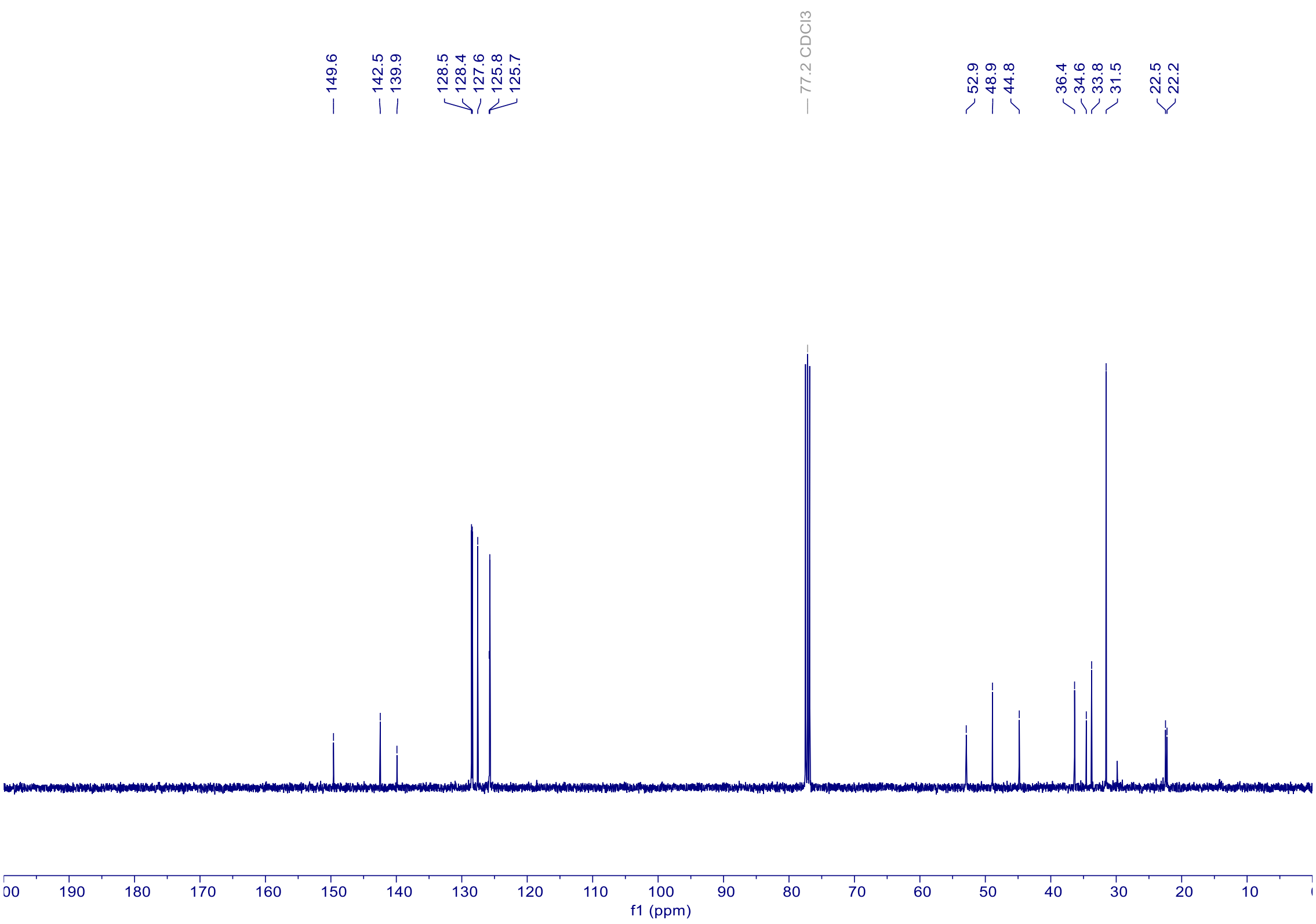

13o – ^{1}H NMR (600 MHz, $CDCl_3$)

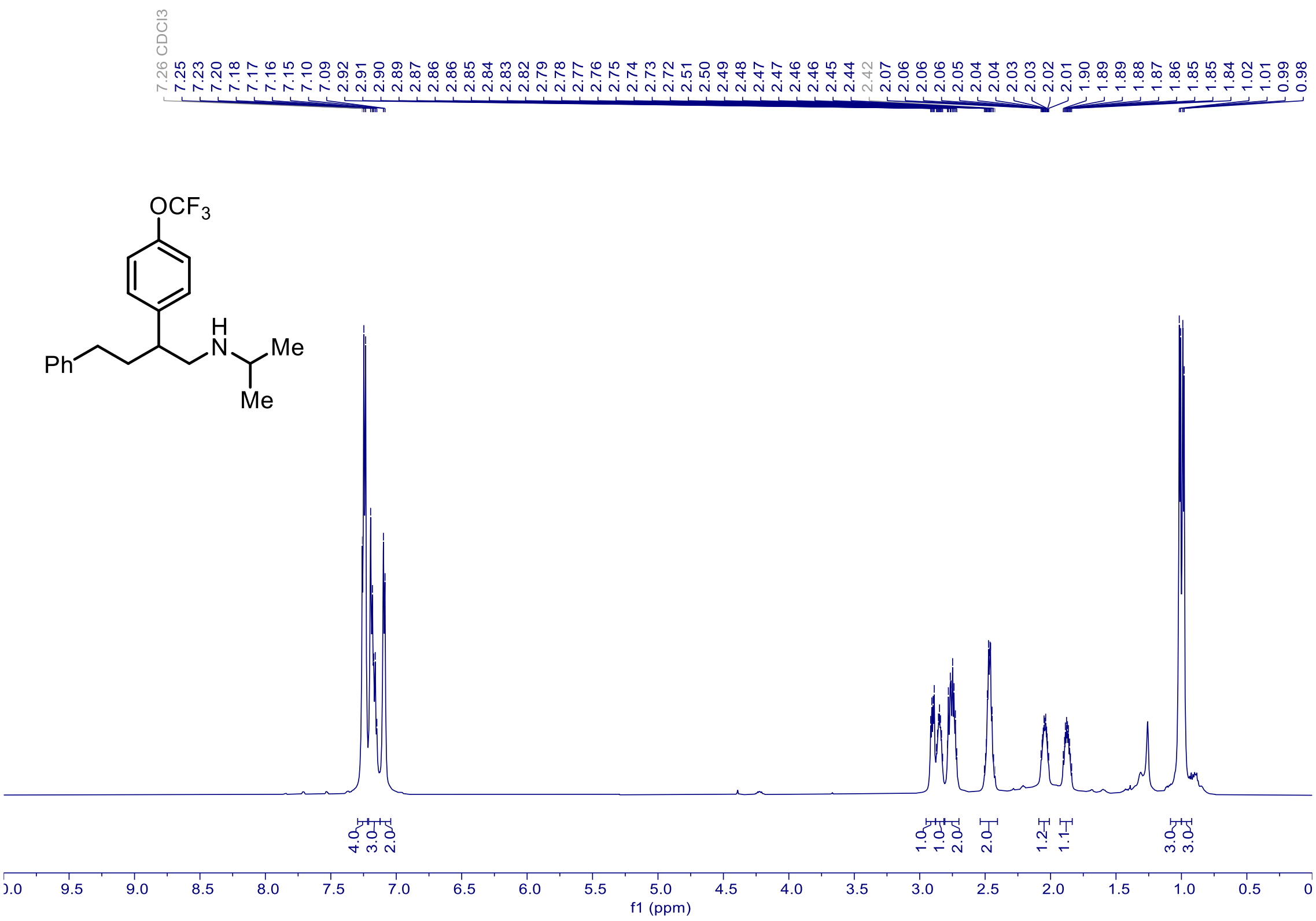

13o – ^{13}C NMR (151 MHz, $CDCl_3$)

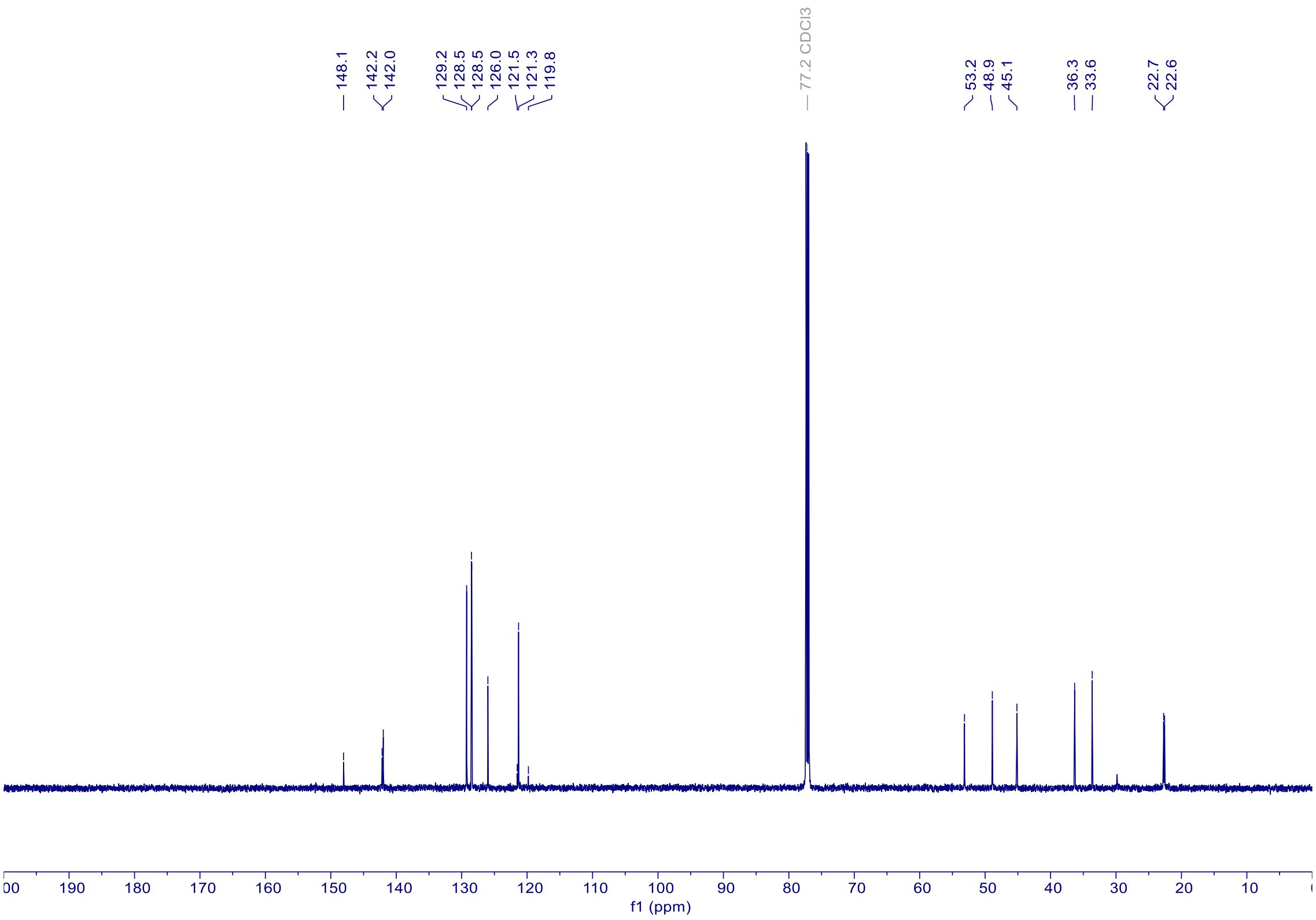

13o – ^{19}F NMR (564 MHz, $CDCl_3$)

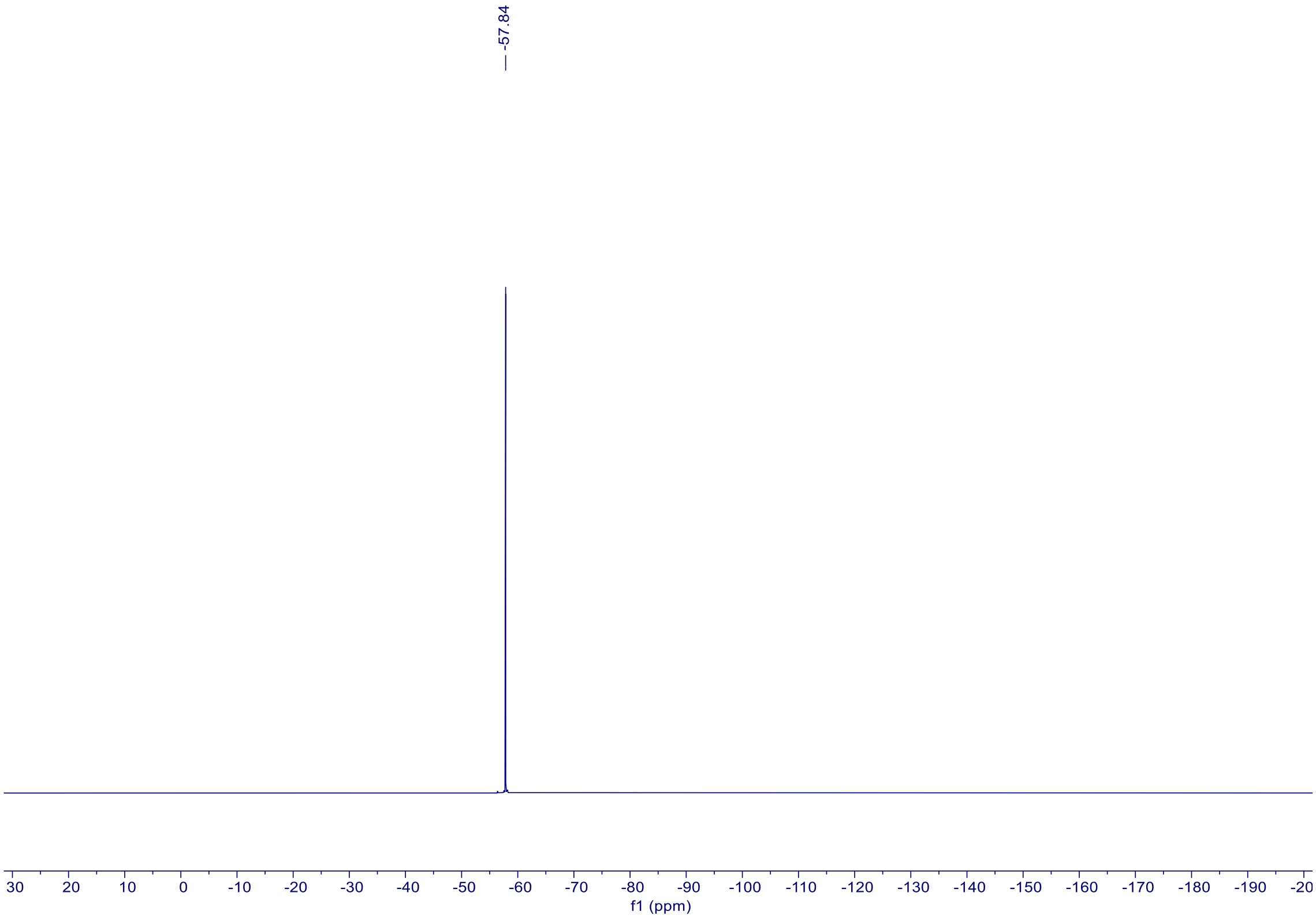

13p – ^{1}H NMR (600 MHz, $CDCl_3$)

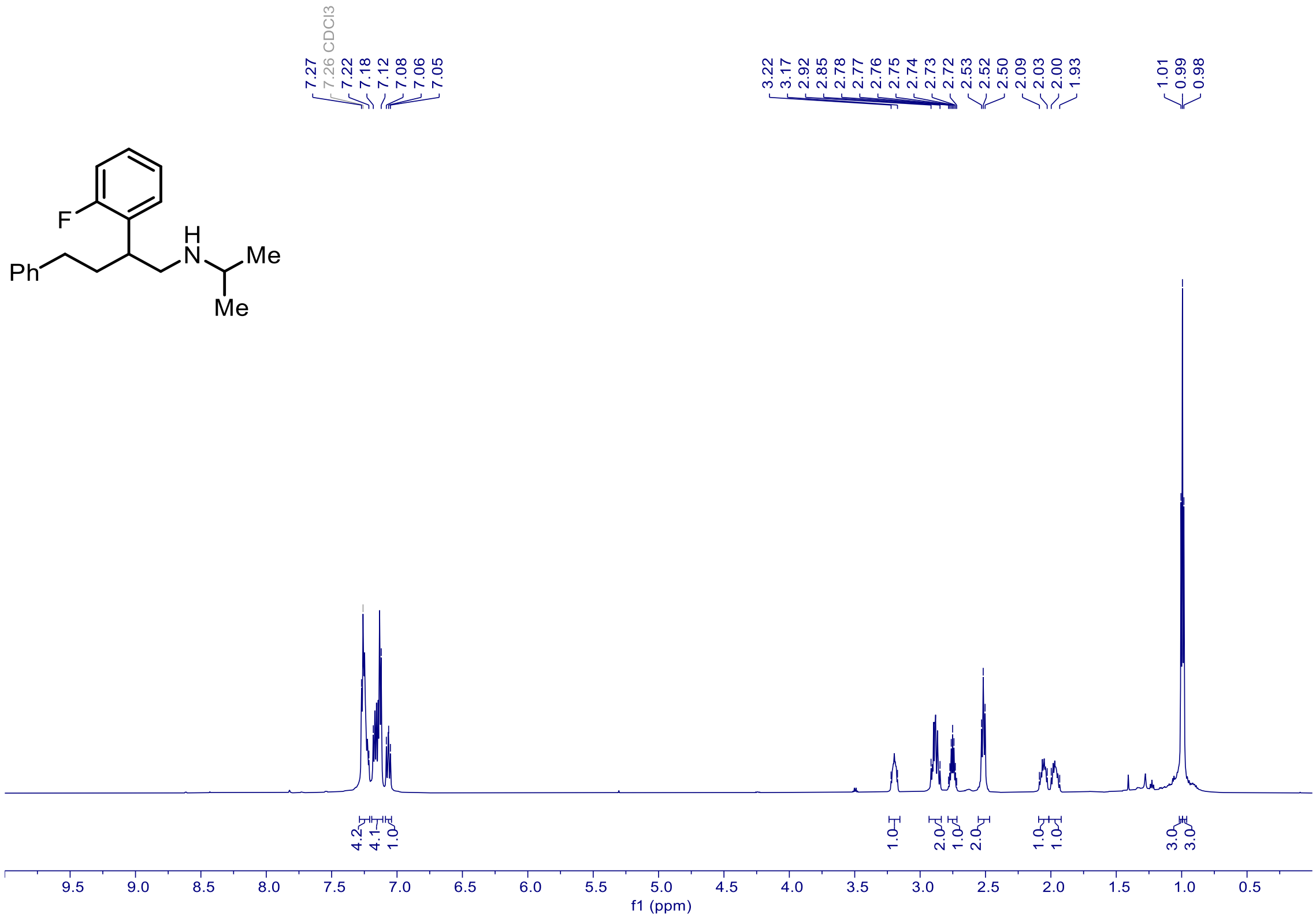

13p – ^{13}C NMR (151 MHz, $CDCl_3$)

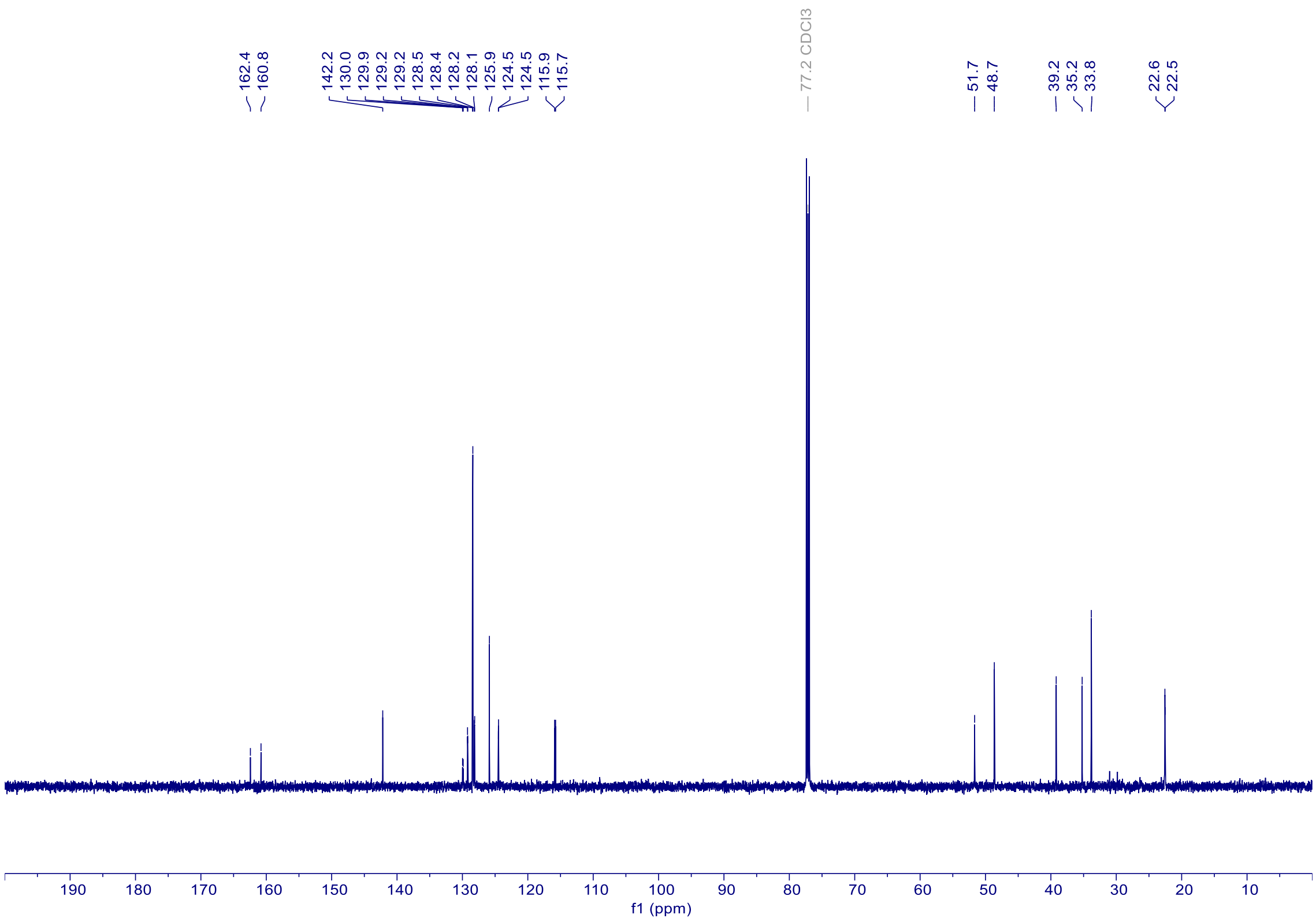

13p – ^{19}F NMR (564 MHz, $CDCl_3$)

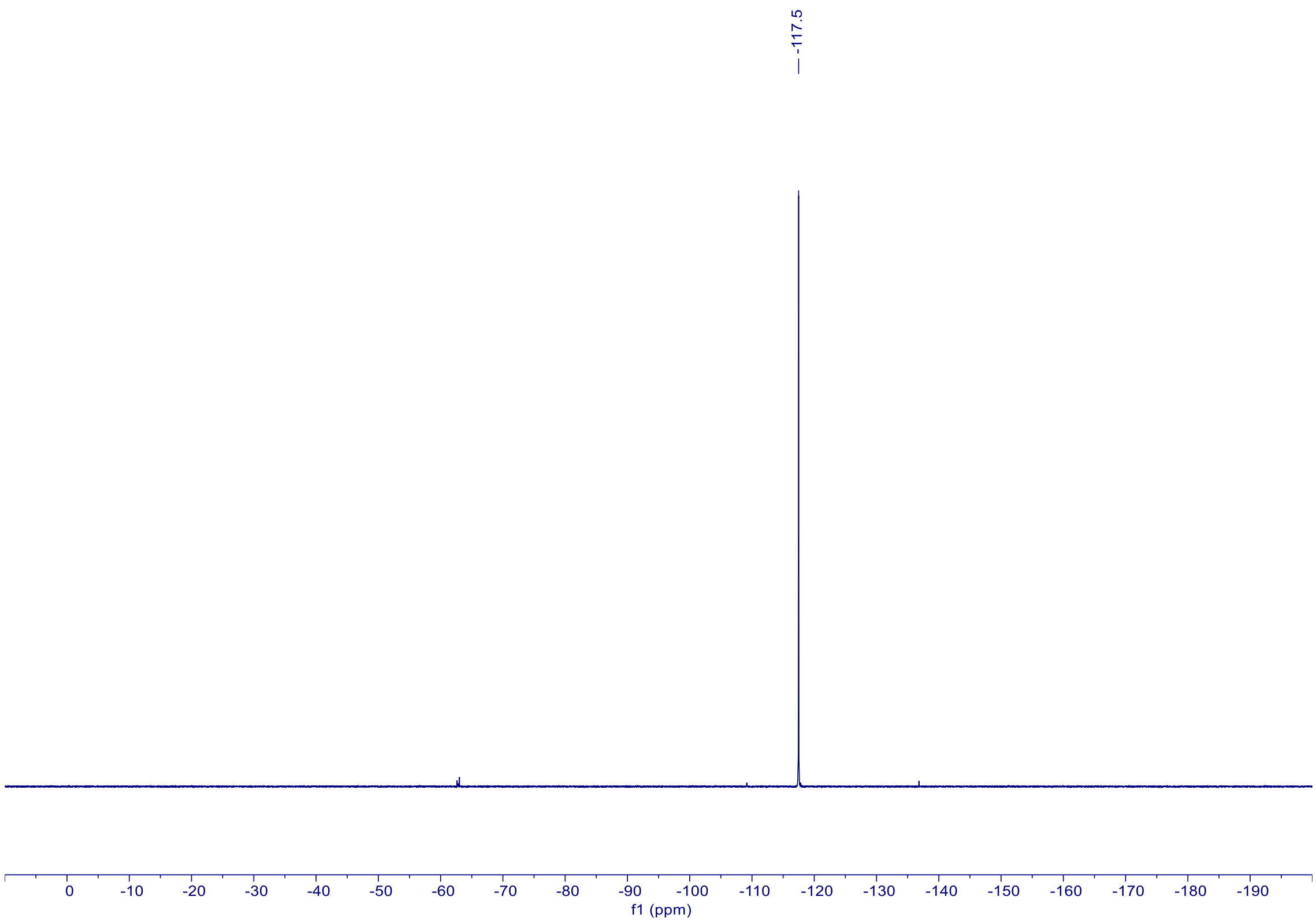

13q – ^{1}H NMR (600 MHz, CDCl$_3$)

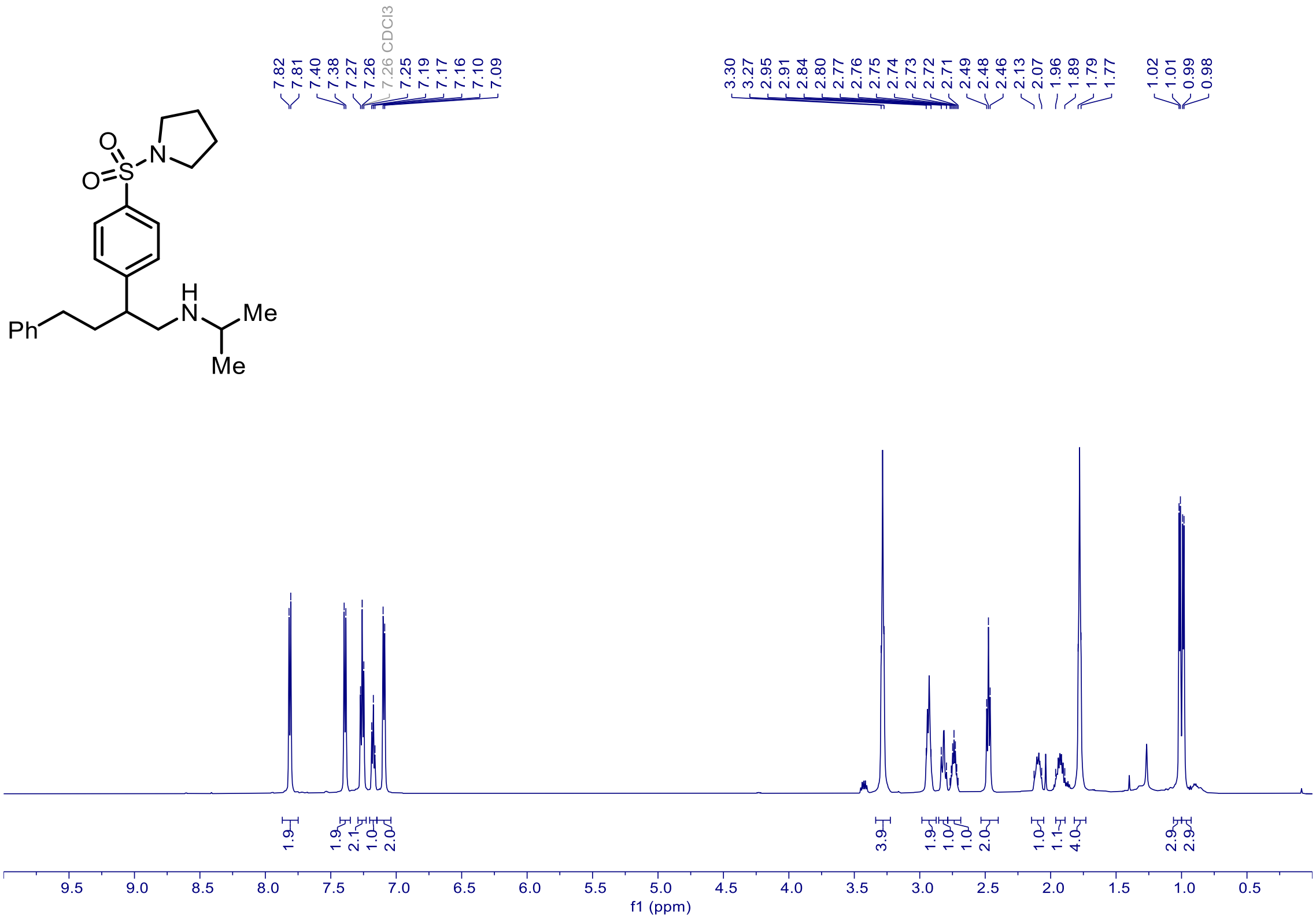

13q – ^{13}C NMR (151 MHz, CDCl$_3$)

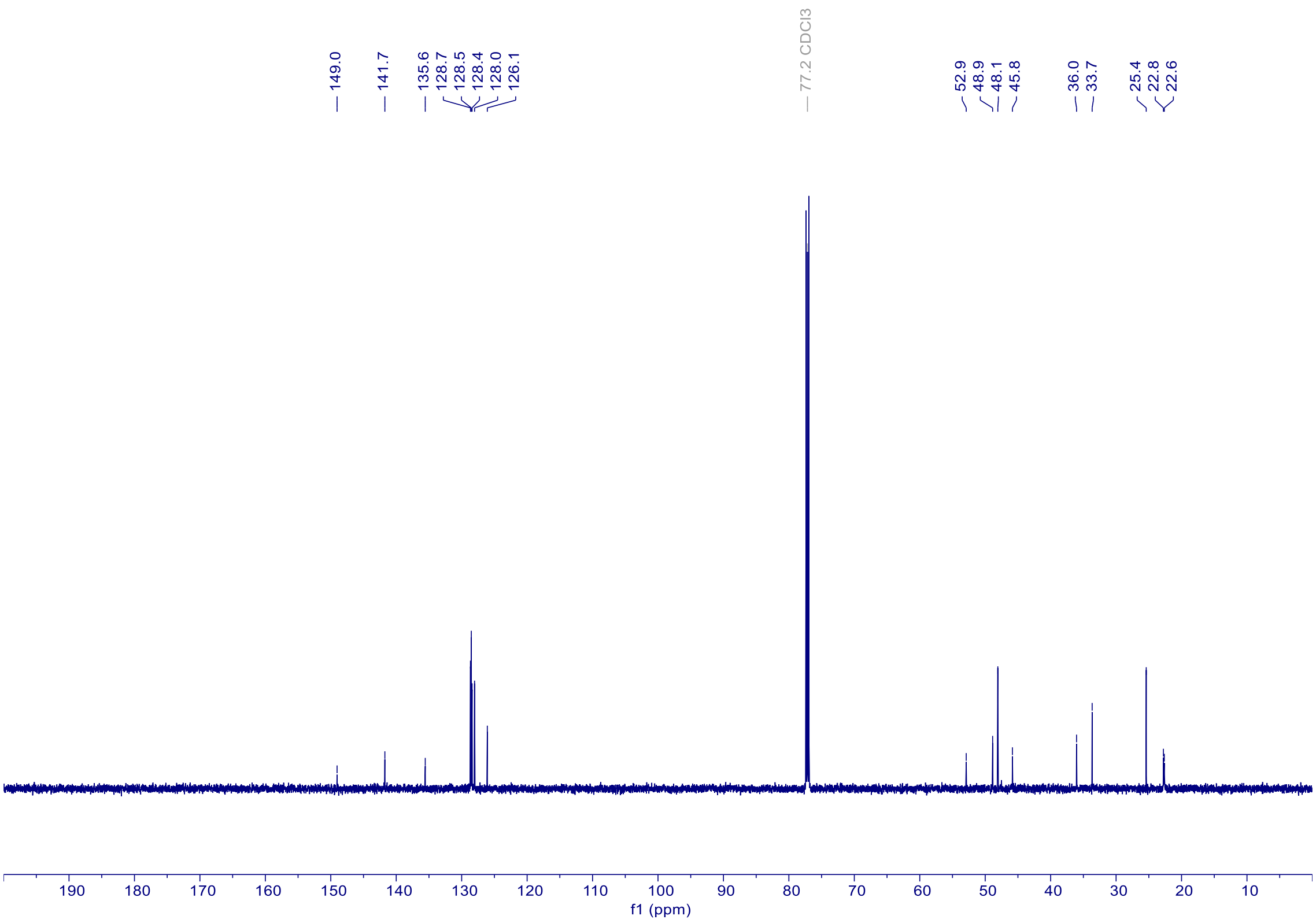

13r – ^{1}H NMR (600 MHz, $CDCl_3$)

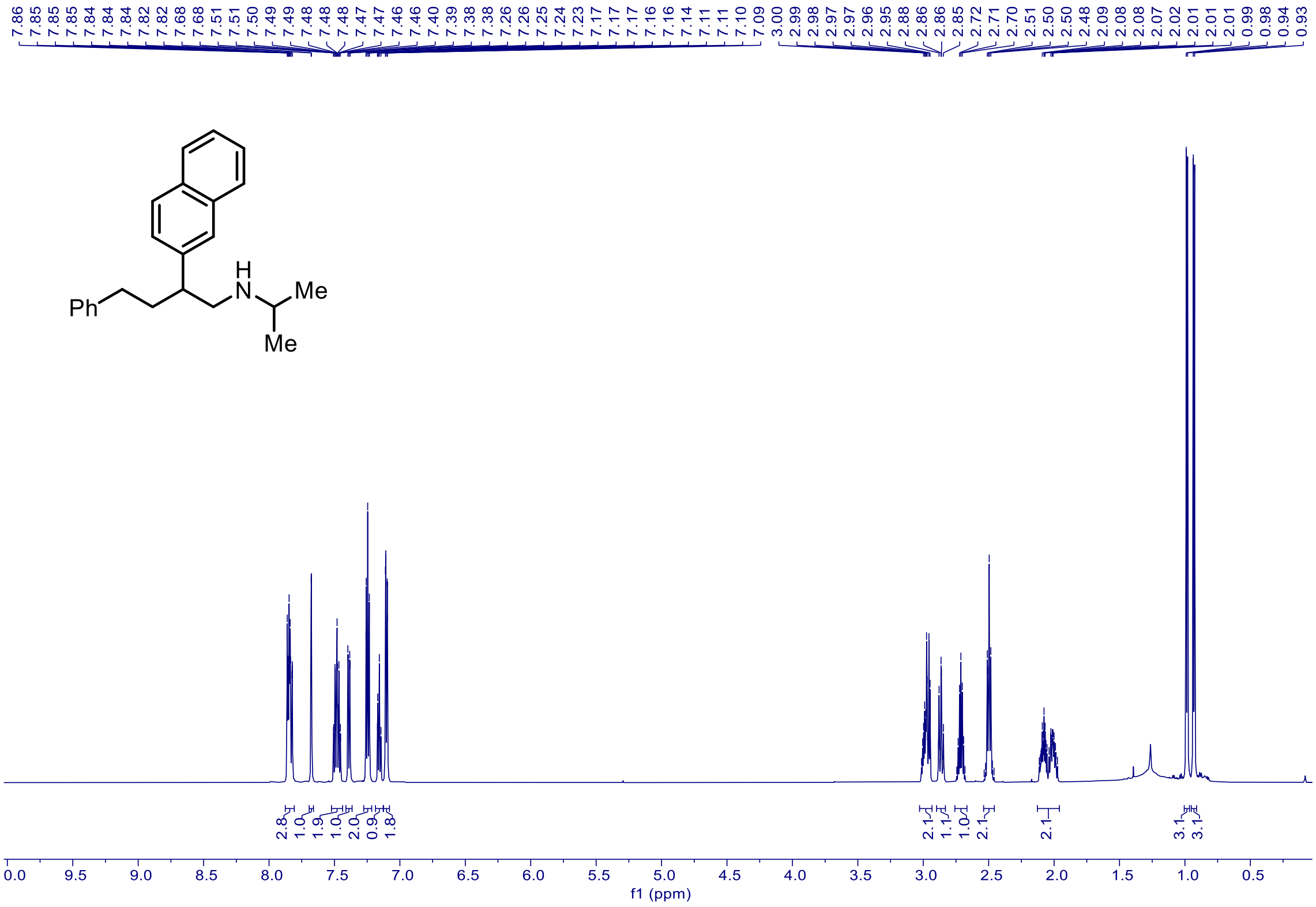

13r – ^{13}C NMR (151 MHz, $CDCl_3$)

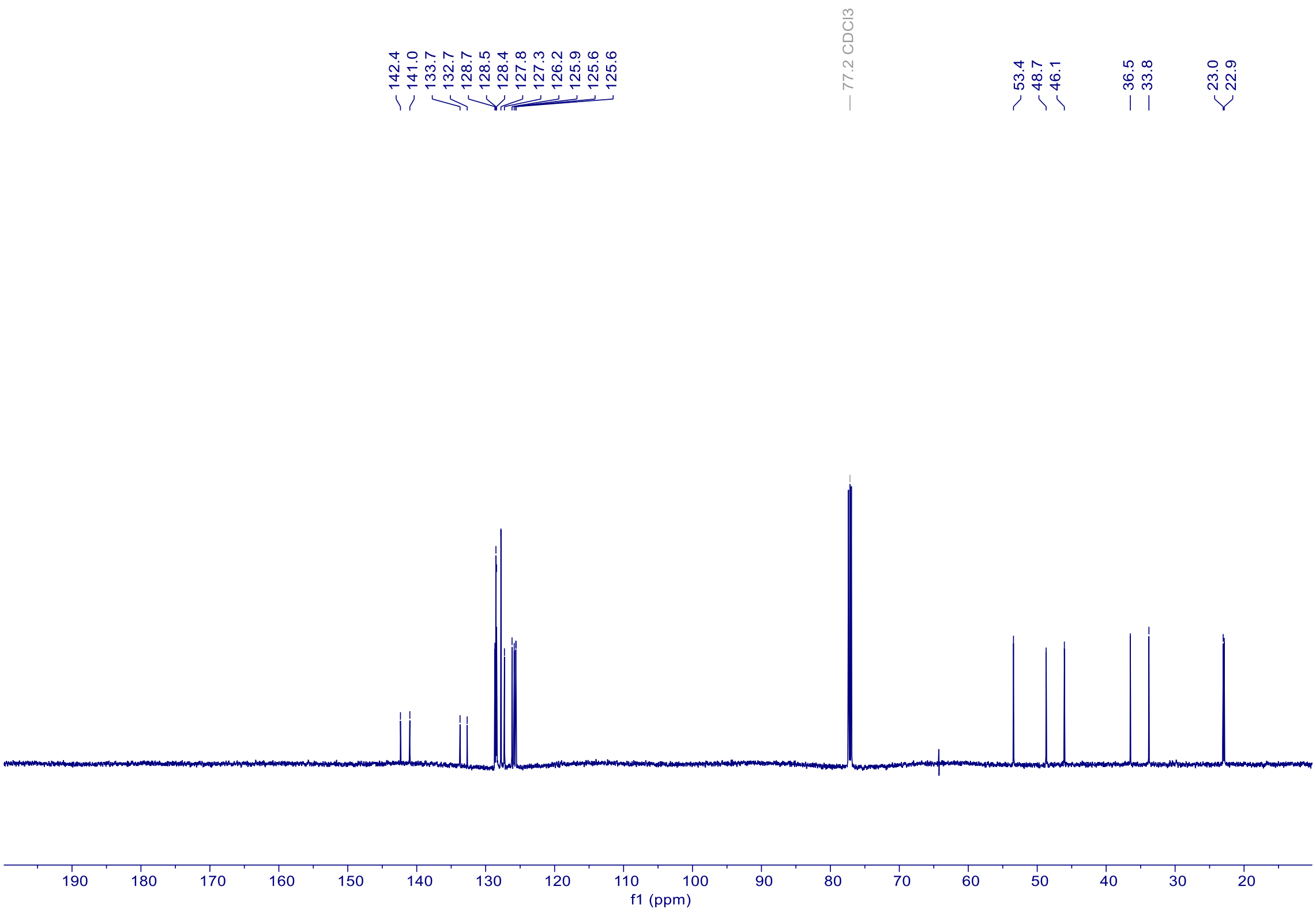

13s – ^{1}H NMR (600 MHz, CDCl$_3$)

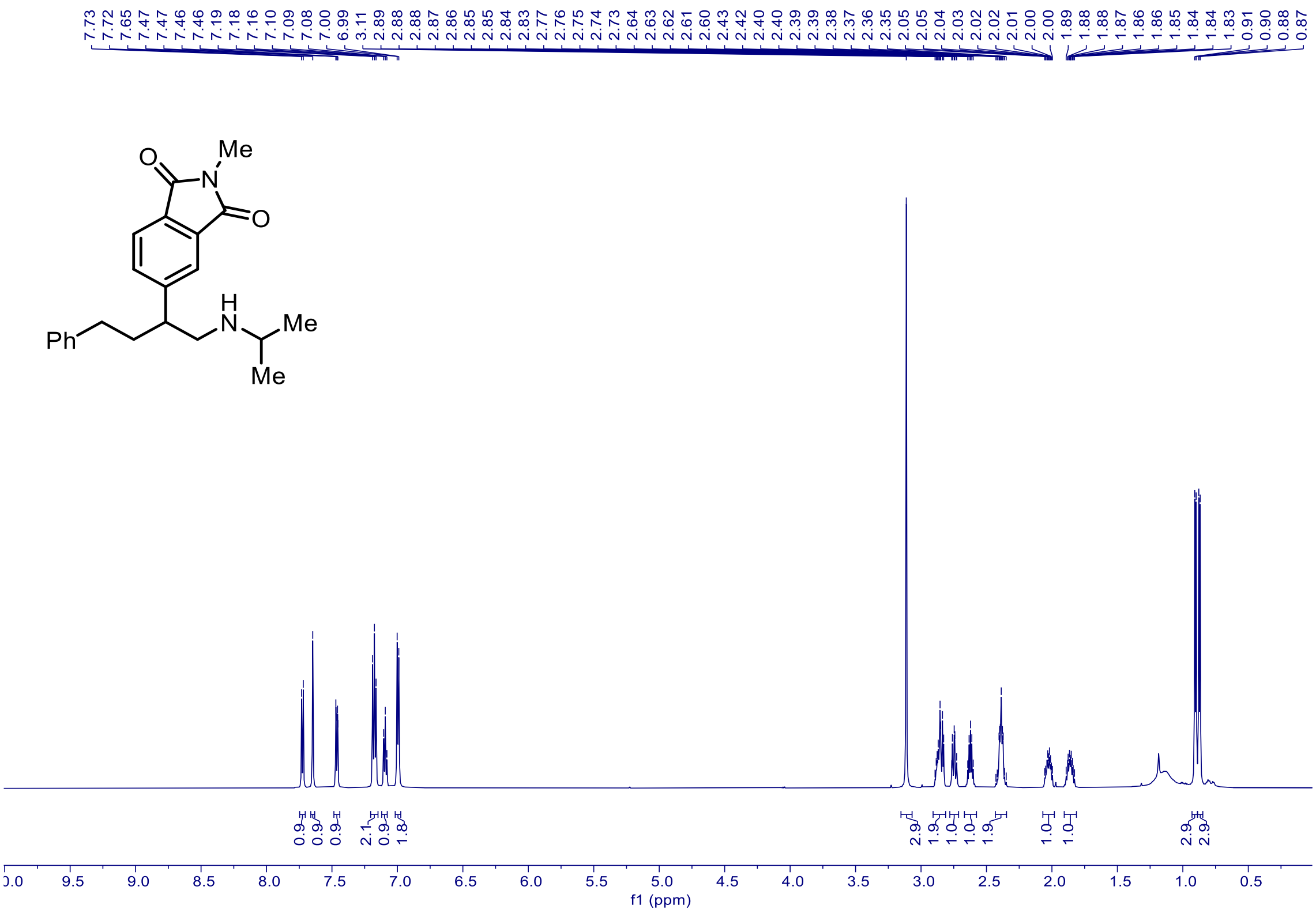

13s – ^{13}C NMR (151 MHz, CDCl$_3$)

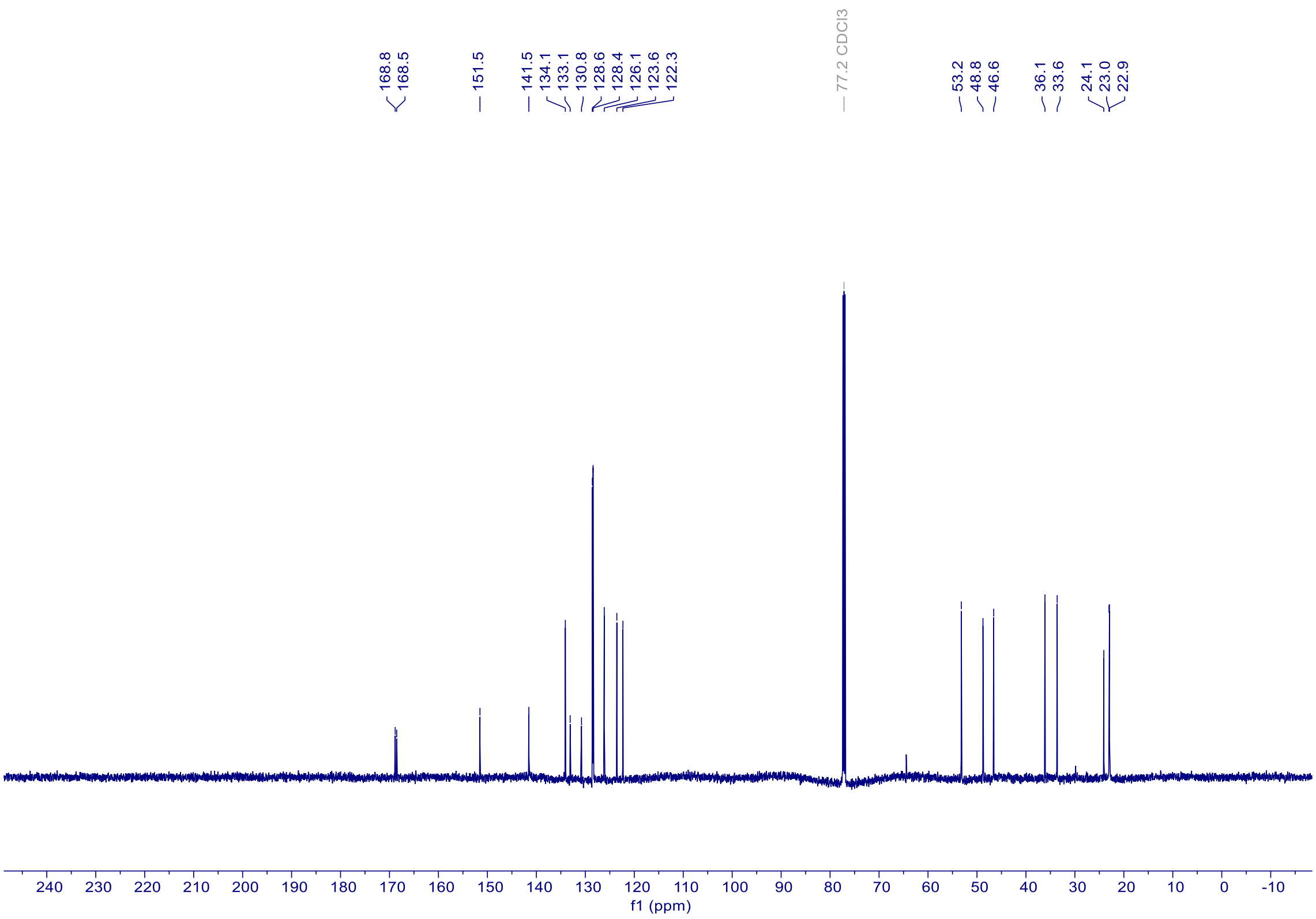

13t – ^{1}H NMR (600 MHz, $CDCl_3$)

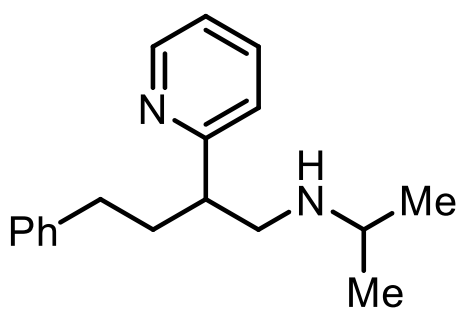

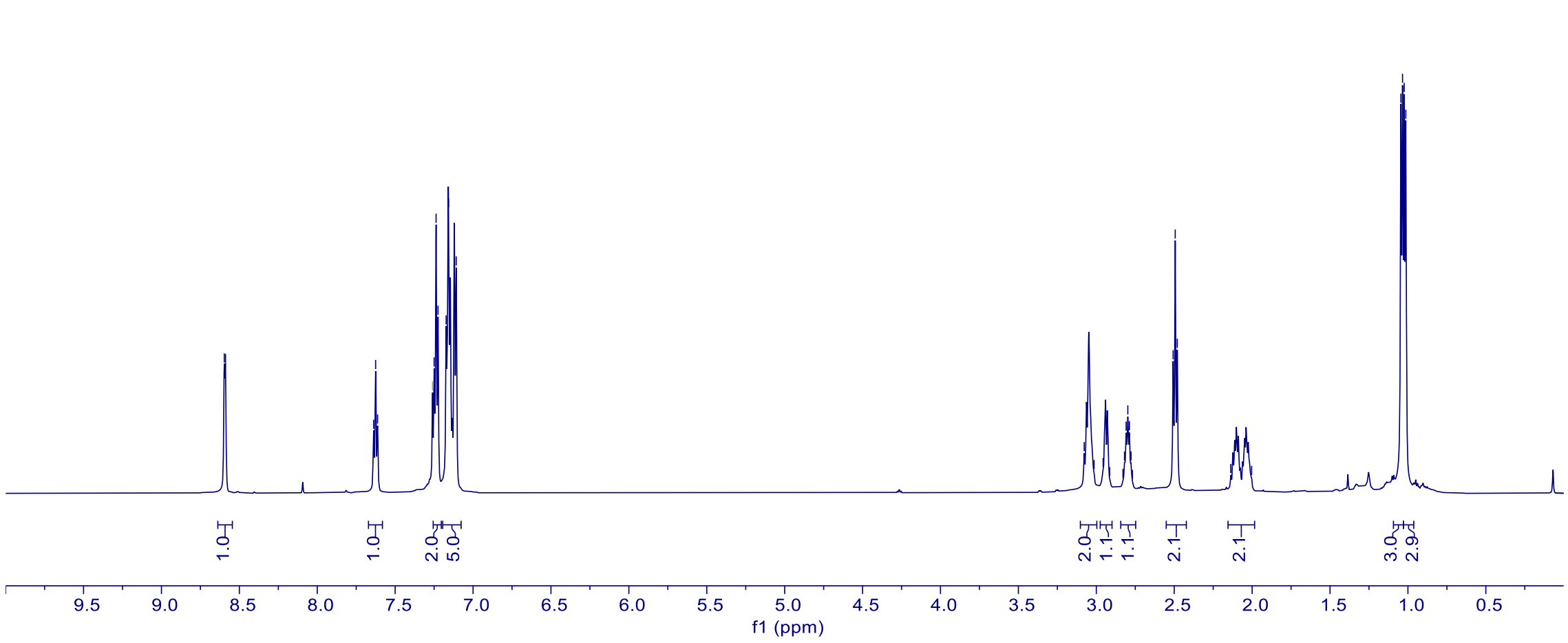

13t – ^{13}C NMR (151 MHz, $CDCl_3$)

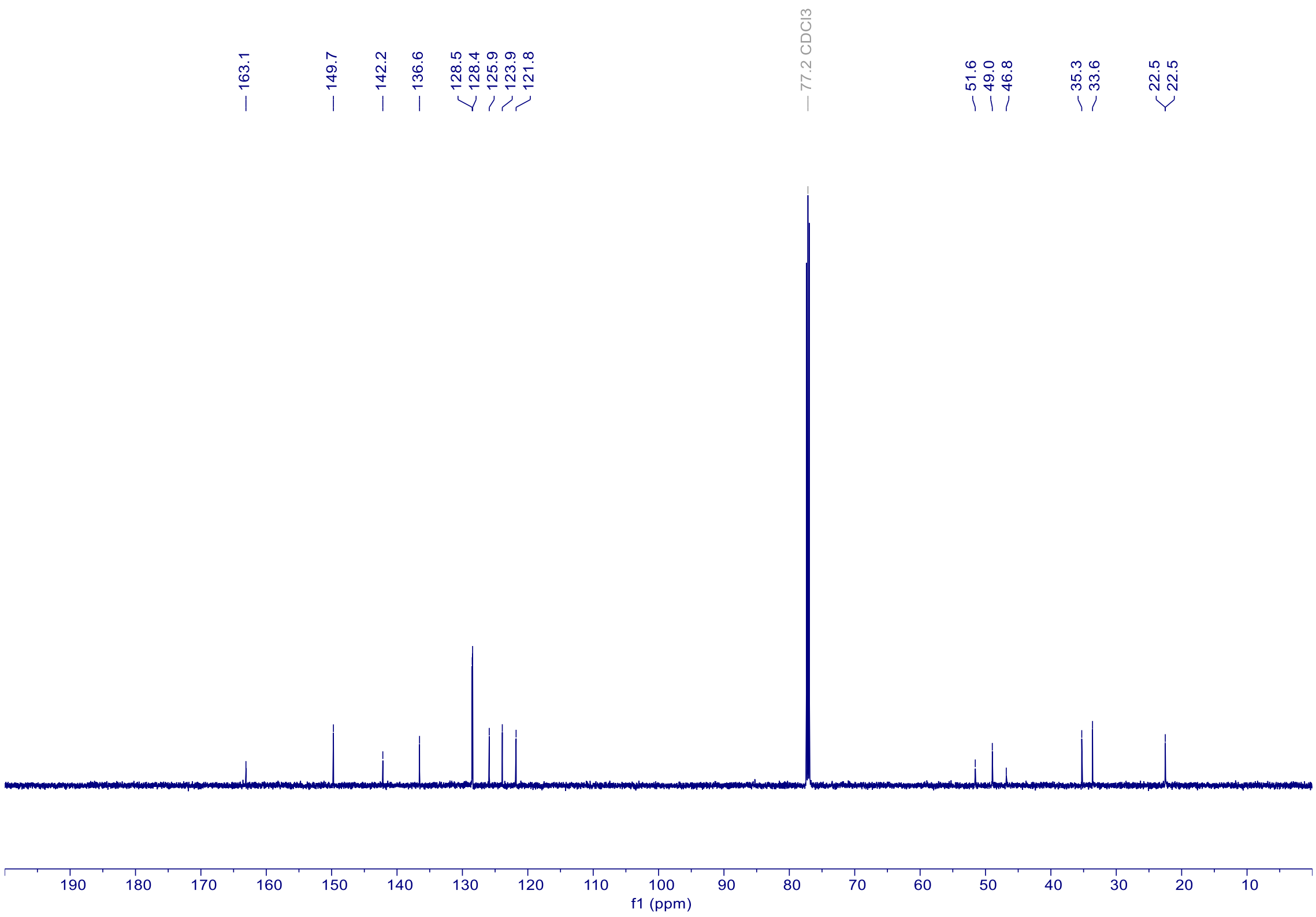

13u – ^{1}H NMR (600 MHz, $CDCl_3$)

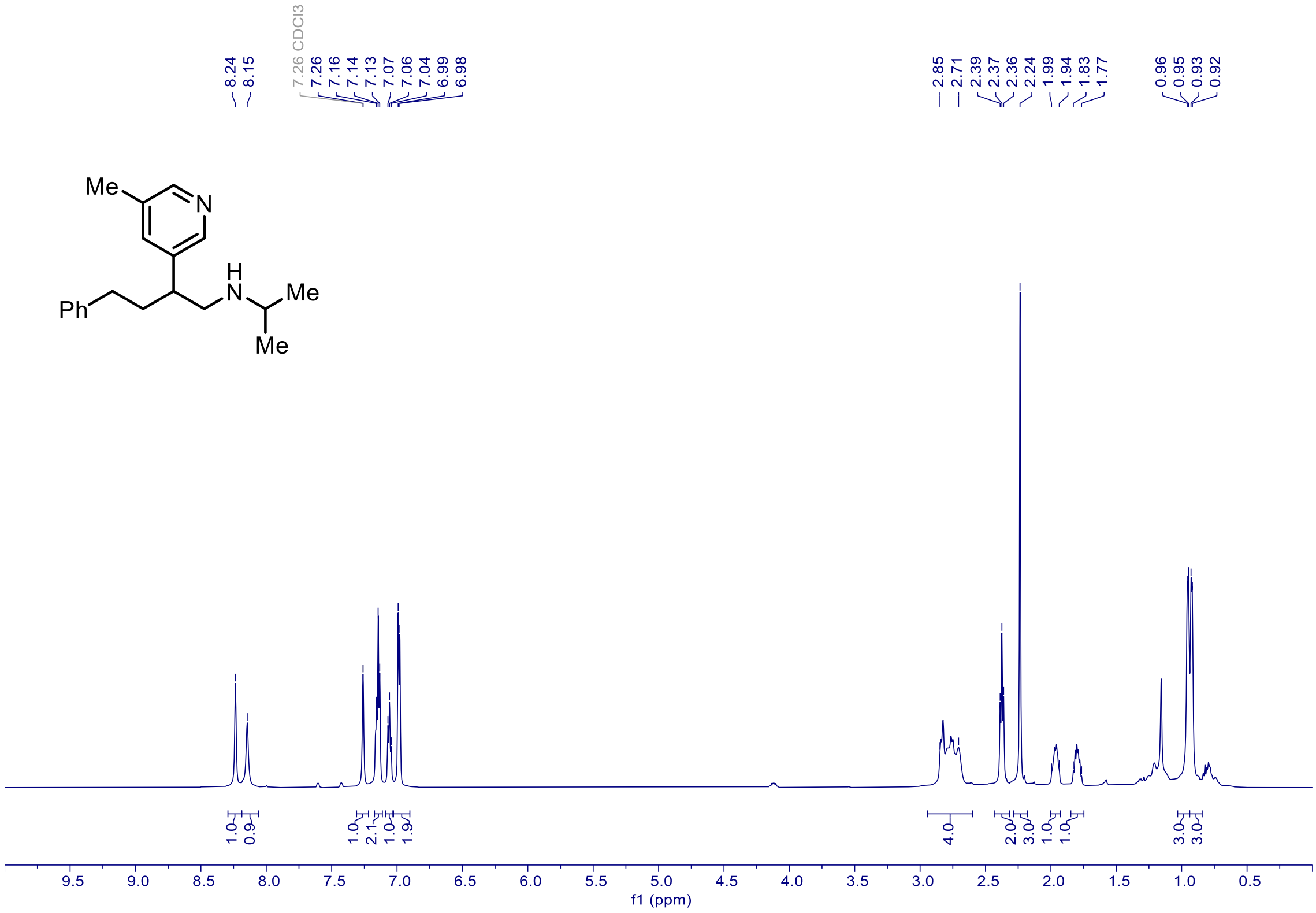

13u – ^{13}C NMR (151 MHz, $CDCl_3$)

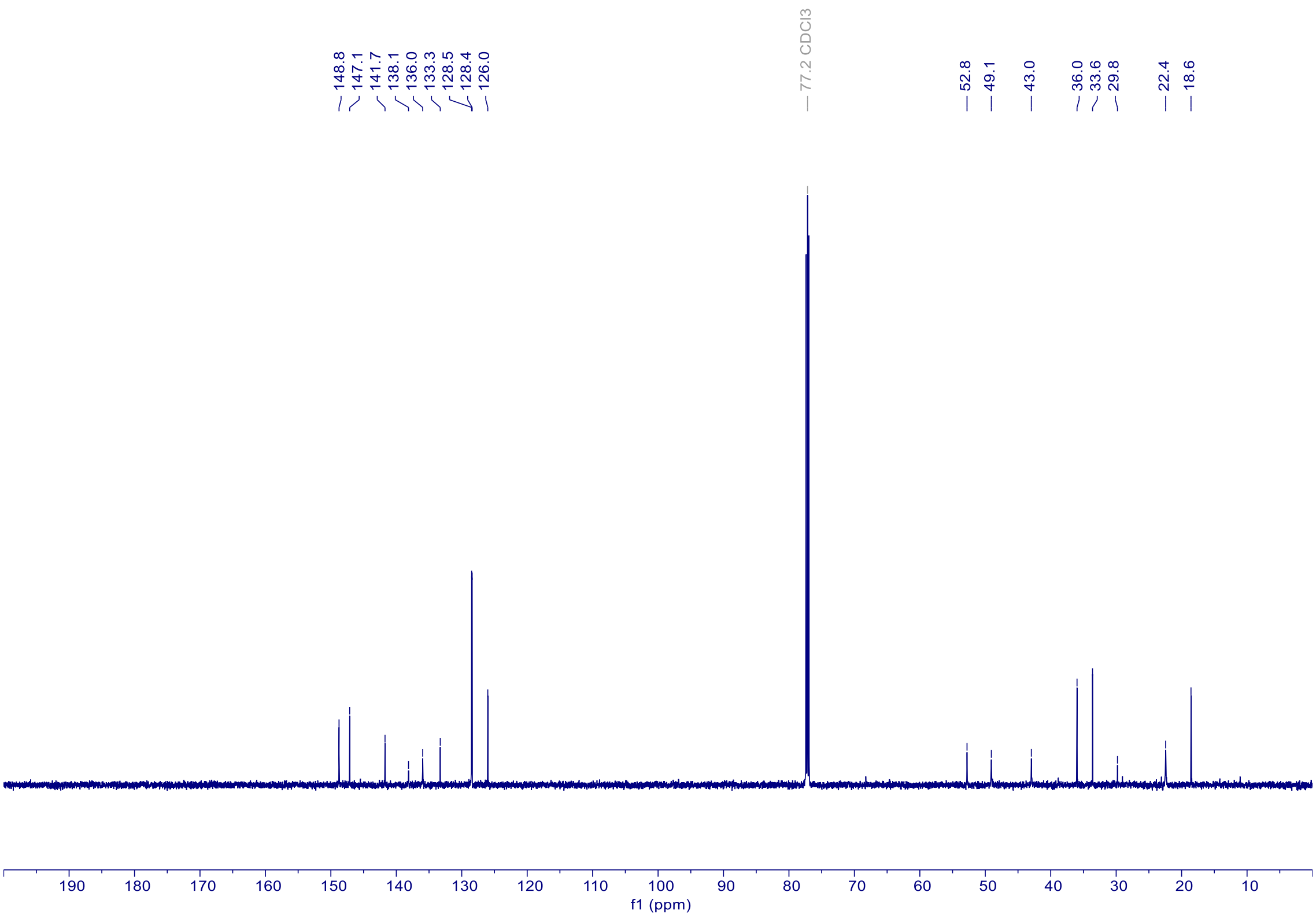

13v – ^{1}H NMR (600 MHz, CDCl$_3$)

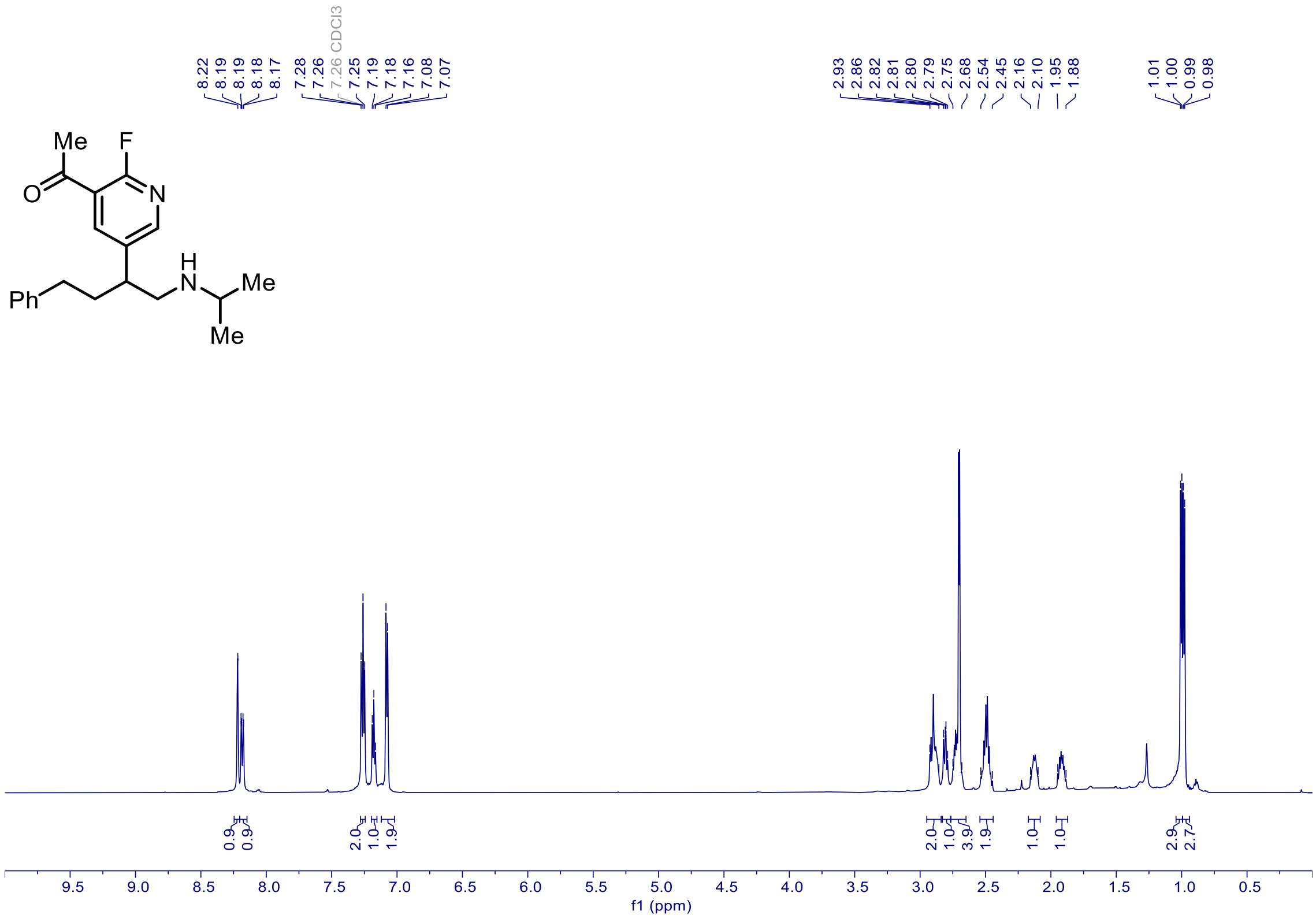

13v – ^{13}C NMR (151 MHz, CDCl$_3$)

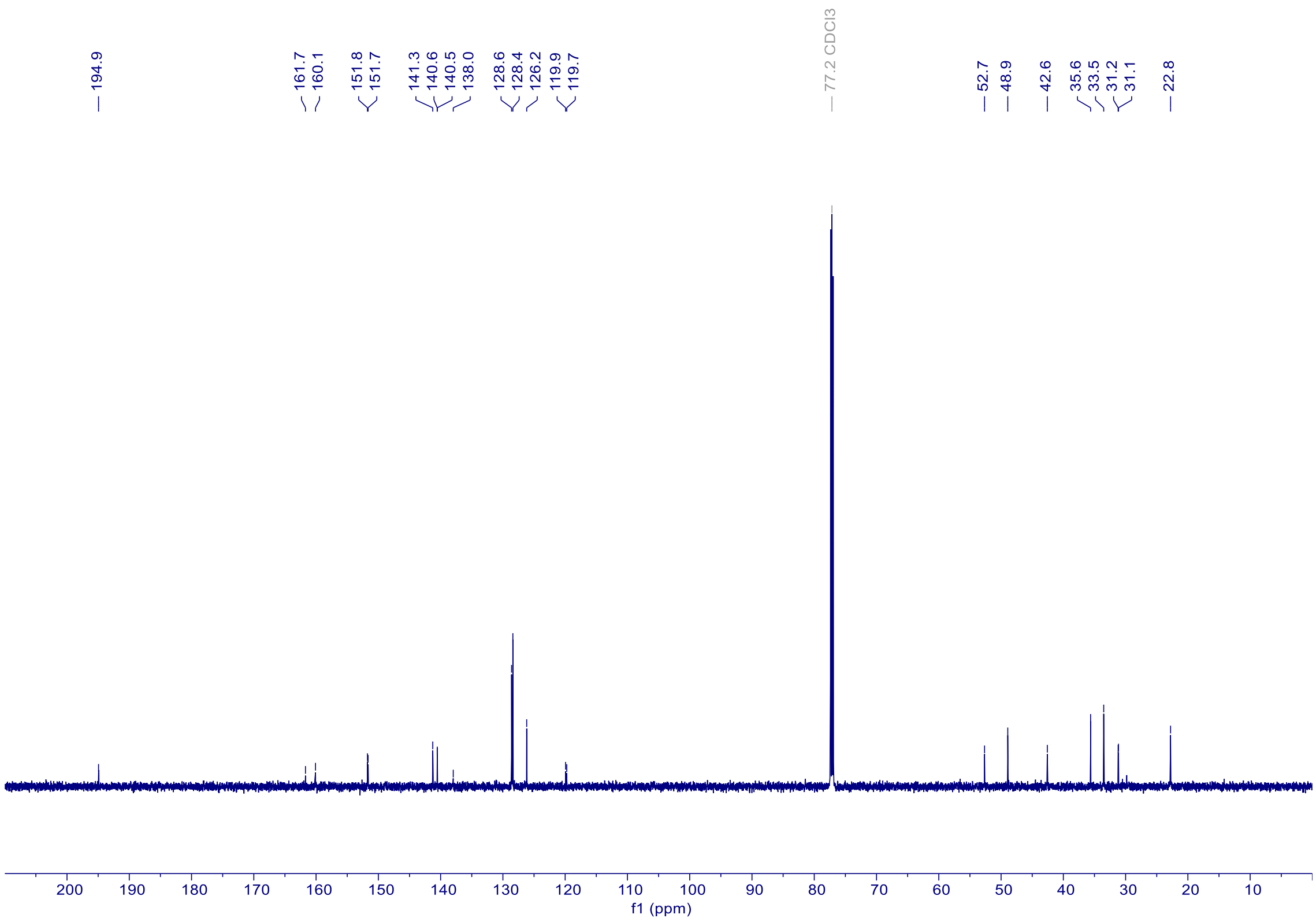

13v – ^{19}F NMR (564 MHz, $CDCl_3$)

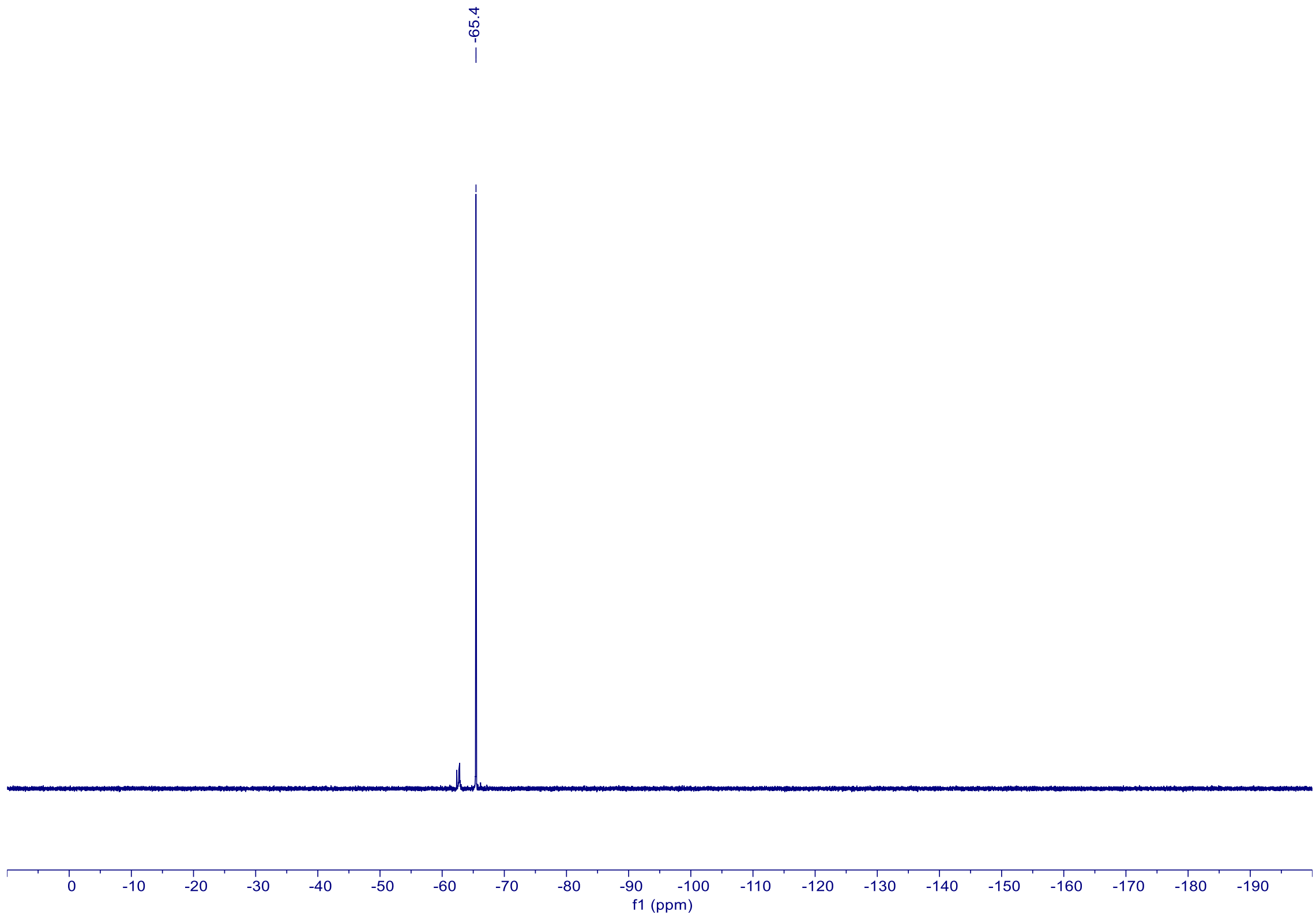

13w – ¹H NMR (600 MHz, $CDCl_3$)

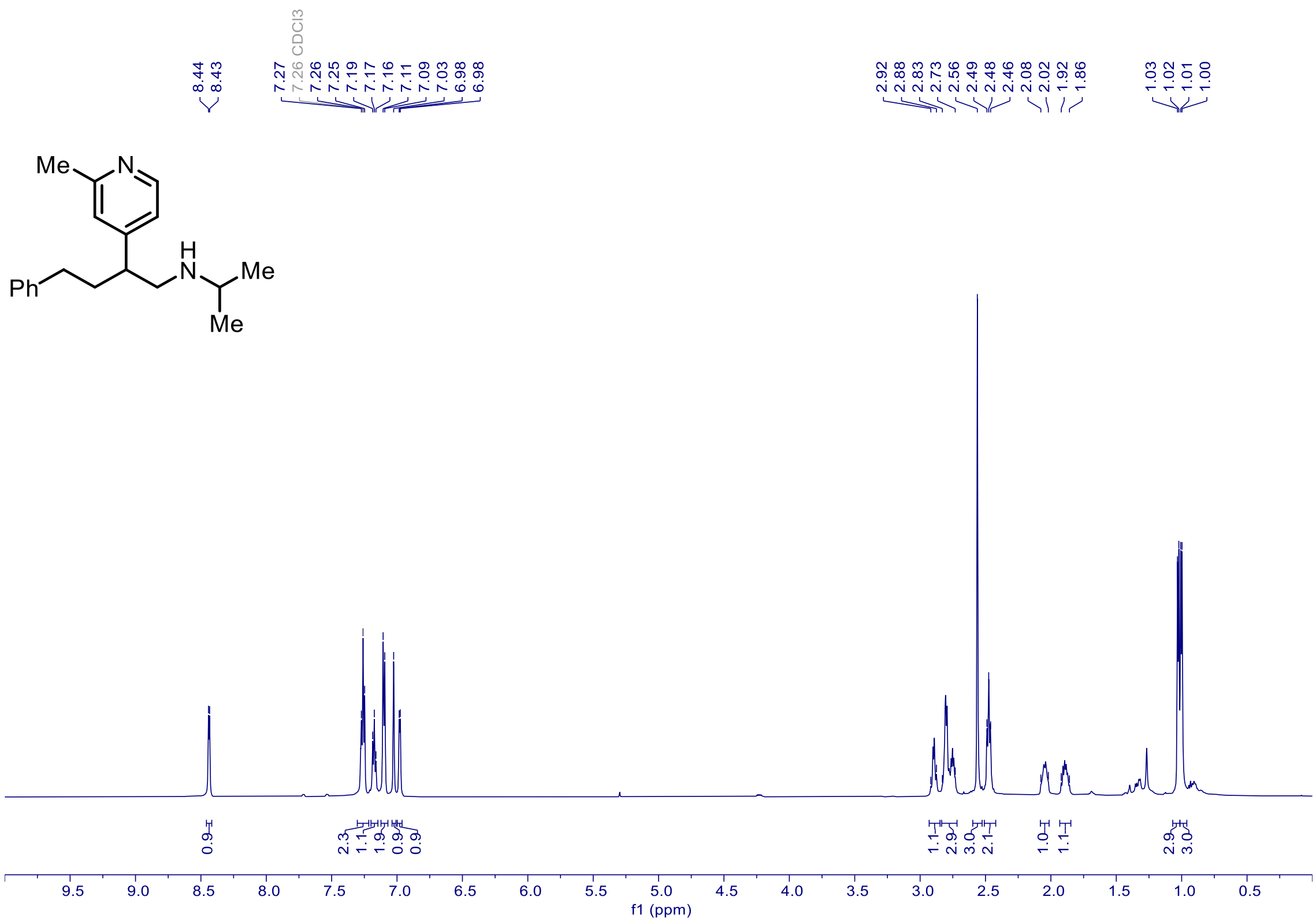

13w – ¹³C NMR (151 MHz, $CDCl_3$)

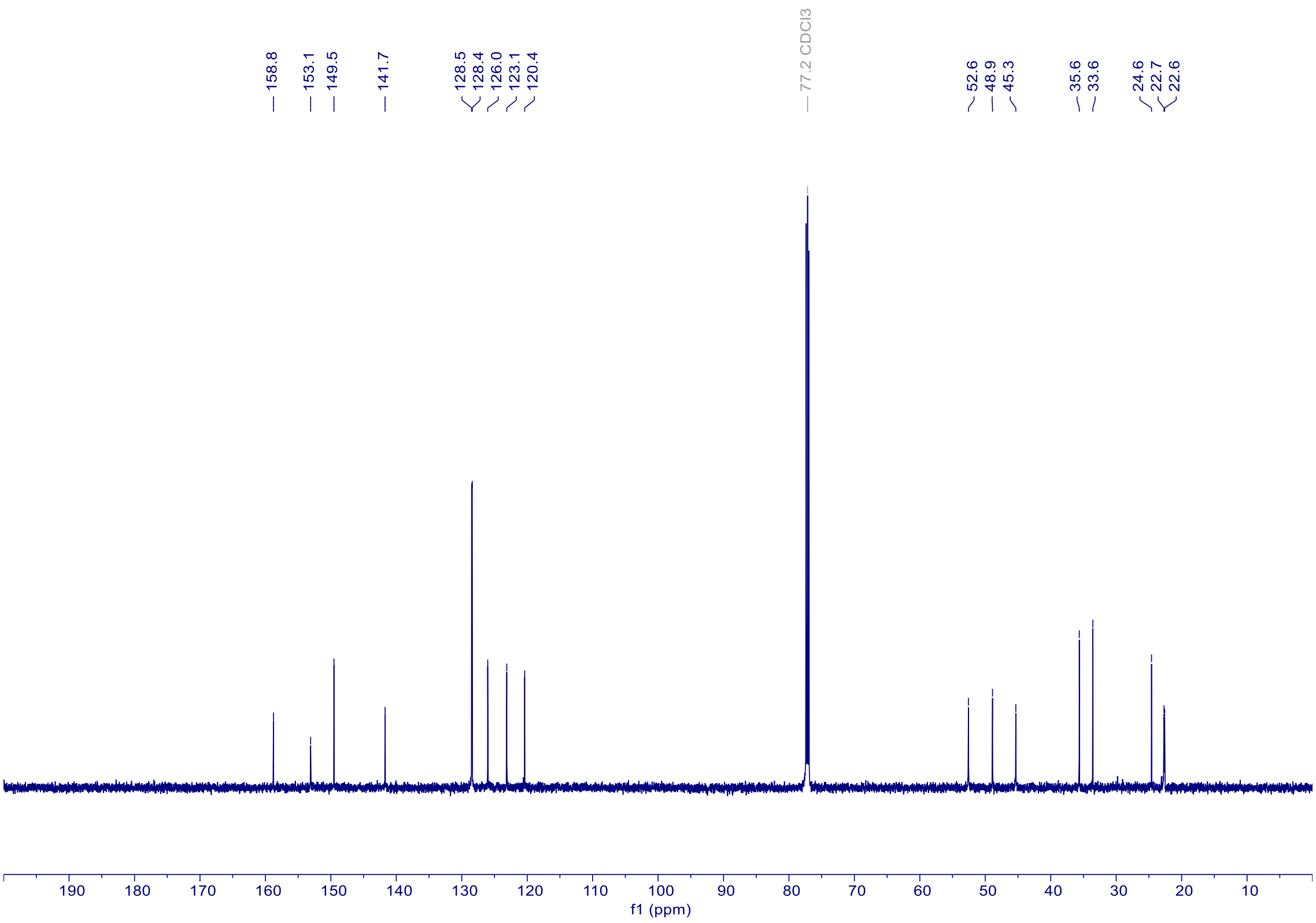

13x – ^{1}H NMR (600 MHz, $CDCl_3$)

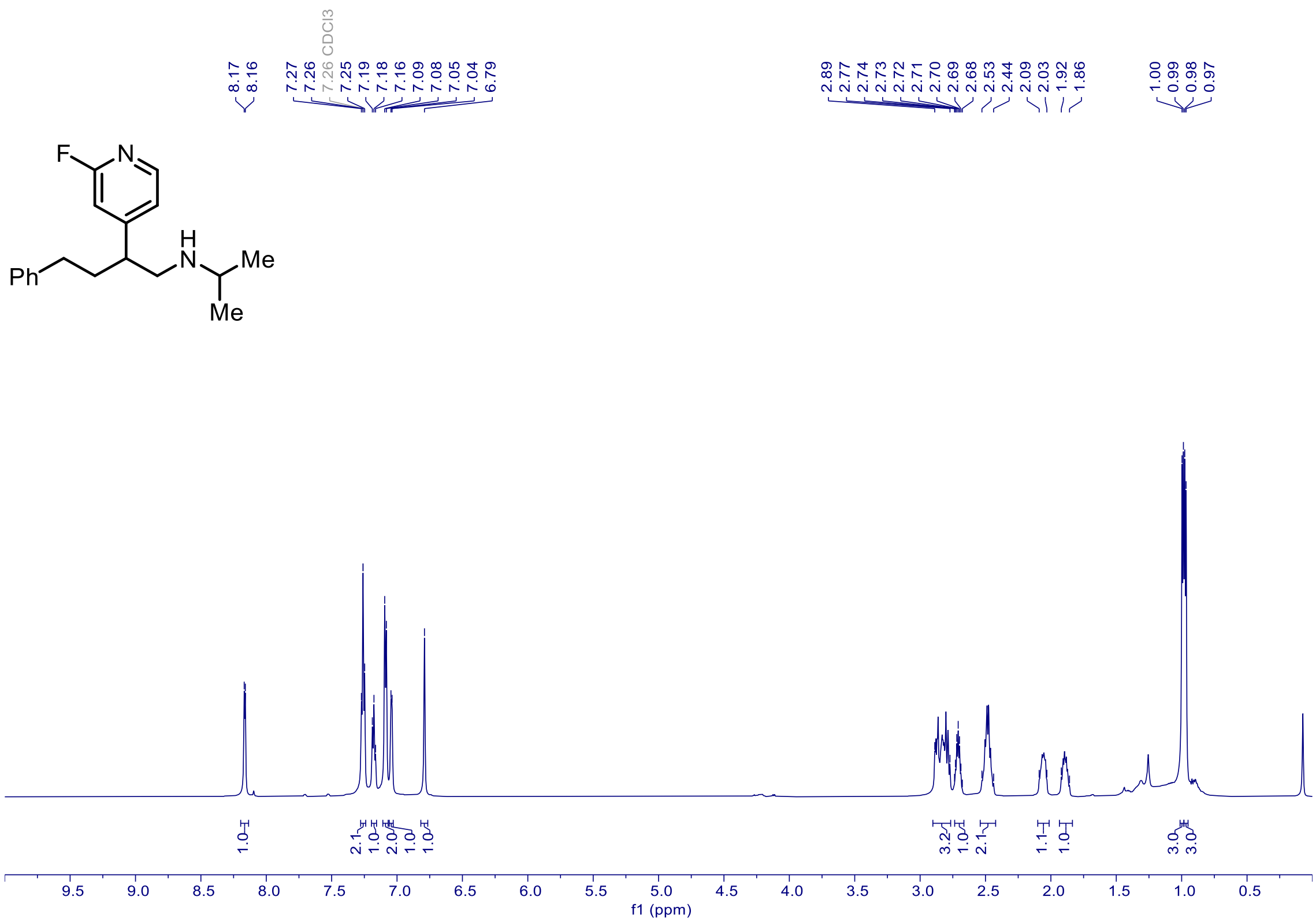

13x – ^{13}C NMR (151 MHz, $CDCl_3$)

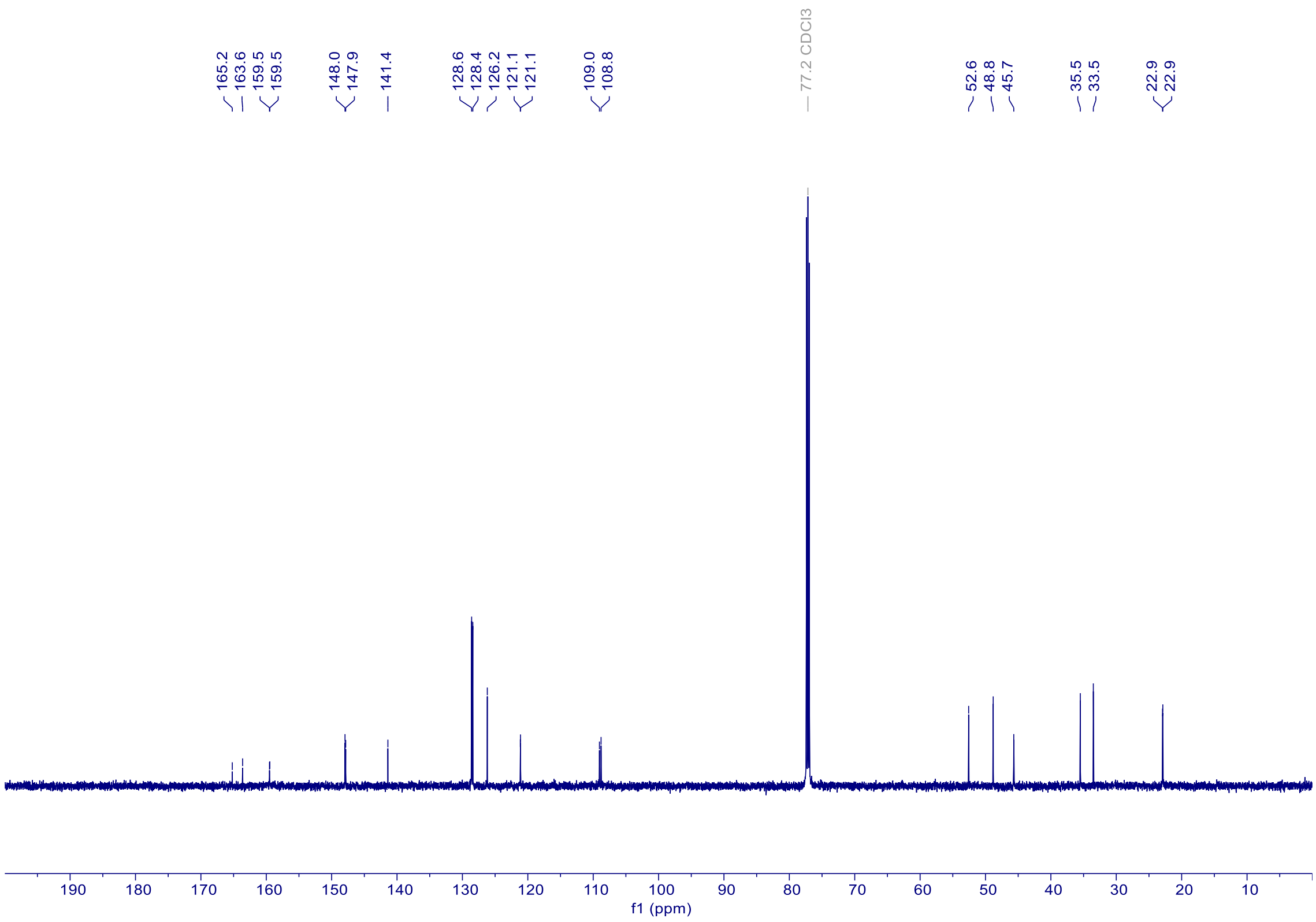

13x – ^{19}F NMR (564 MHz, $CDCl_3$)

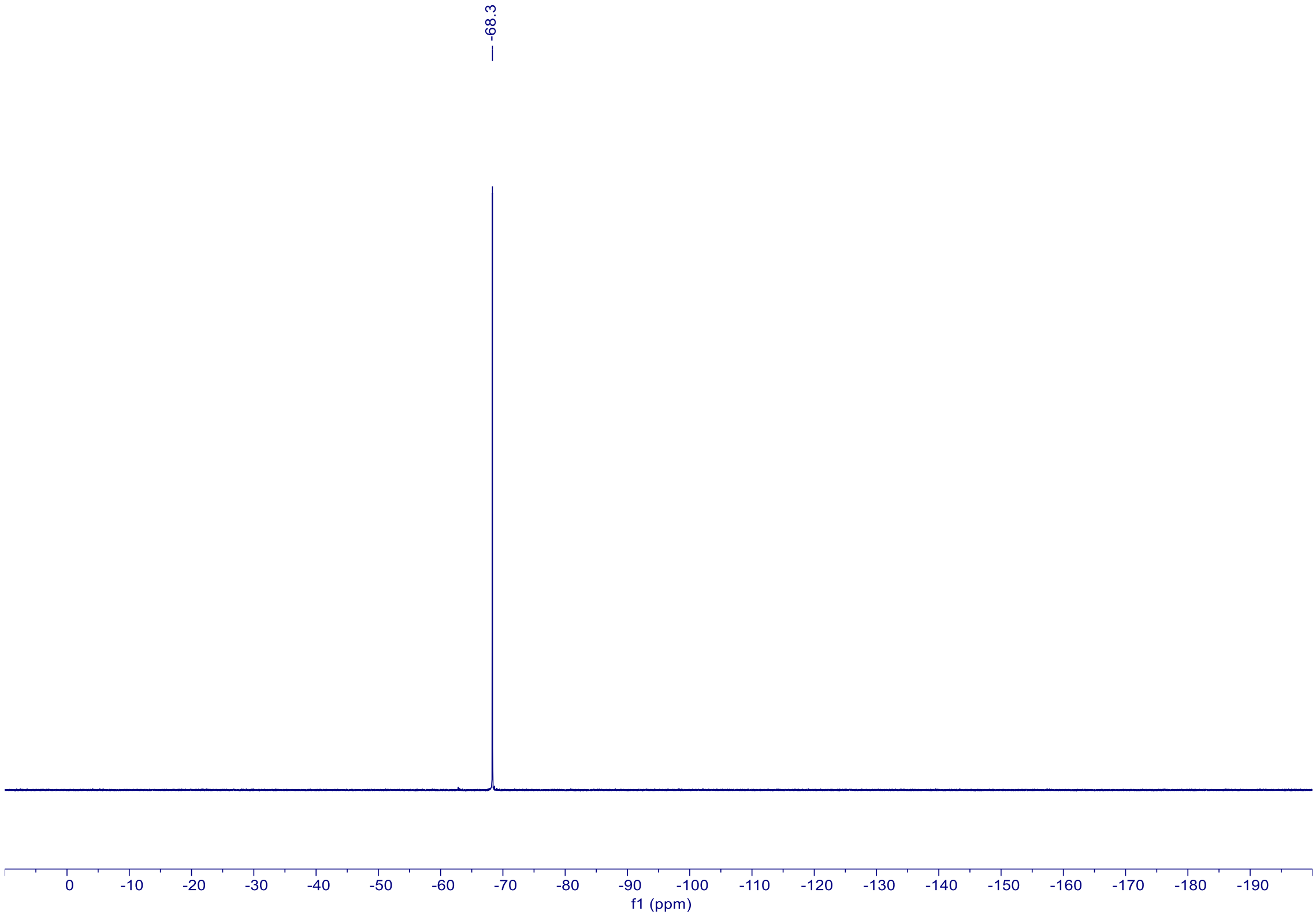

13y – ^{1}H NMR (600 MHz, $CDCl_3$)

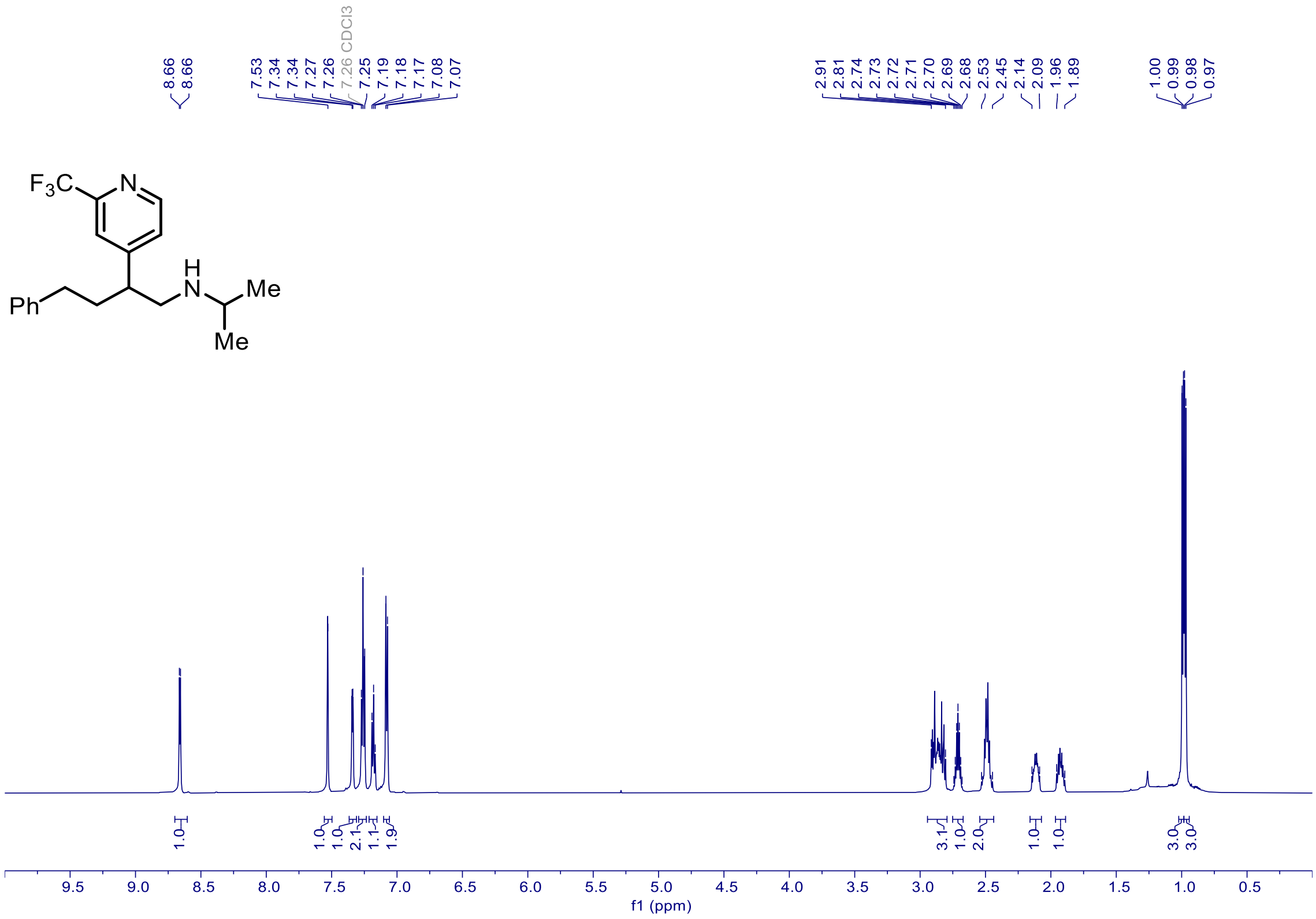

13y – ^{13}C NMR (151 MHz, $CDCl_3$)

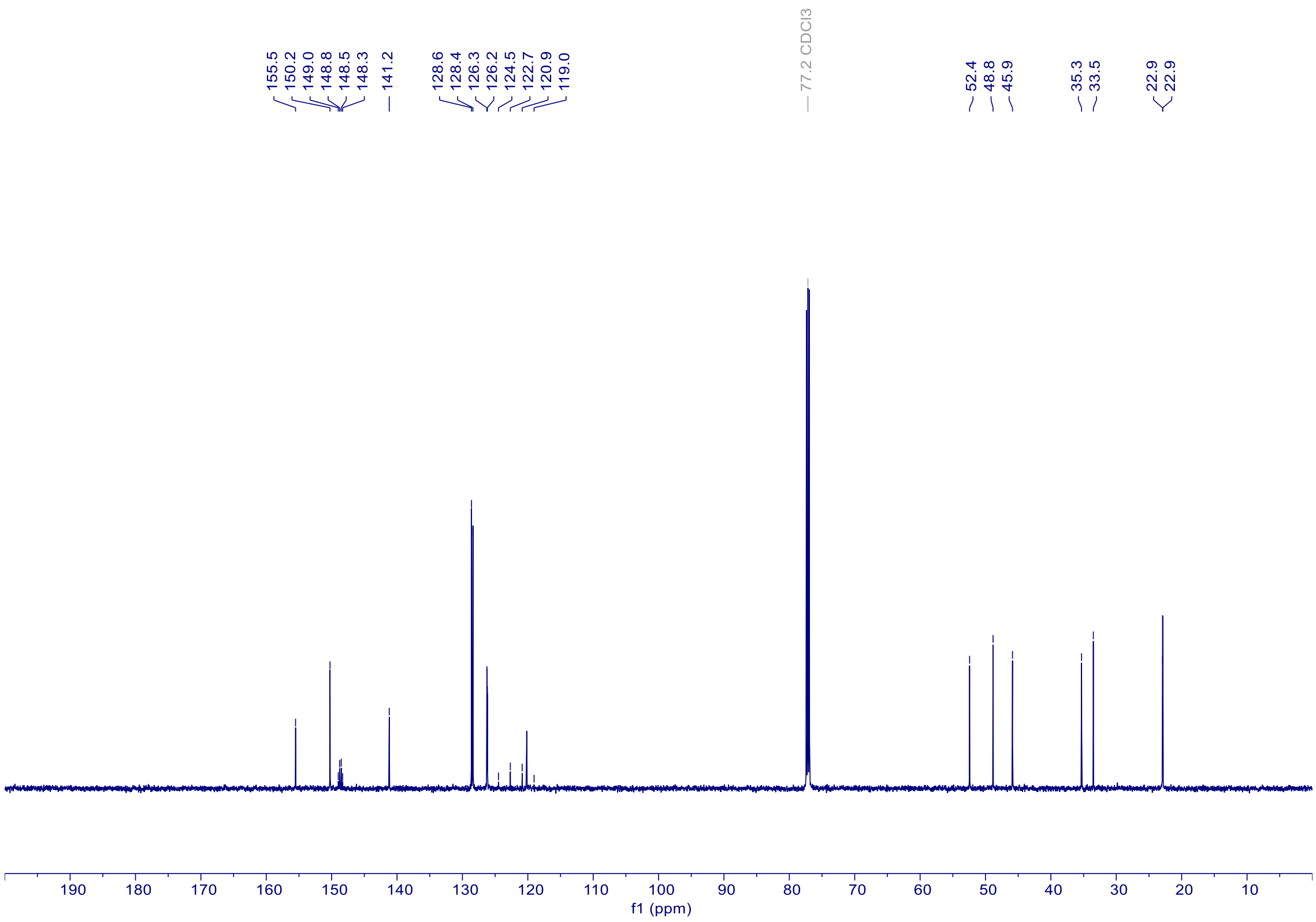

13y – ^{19}F NMR (564 MHz, $CDCl_3$)

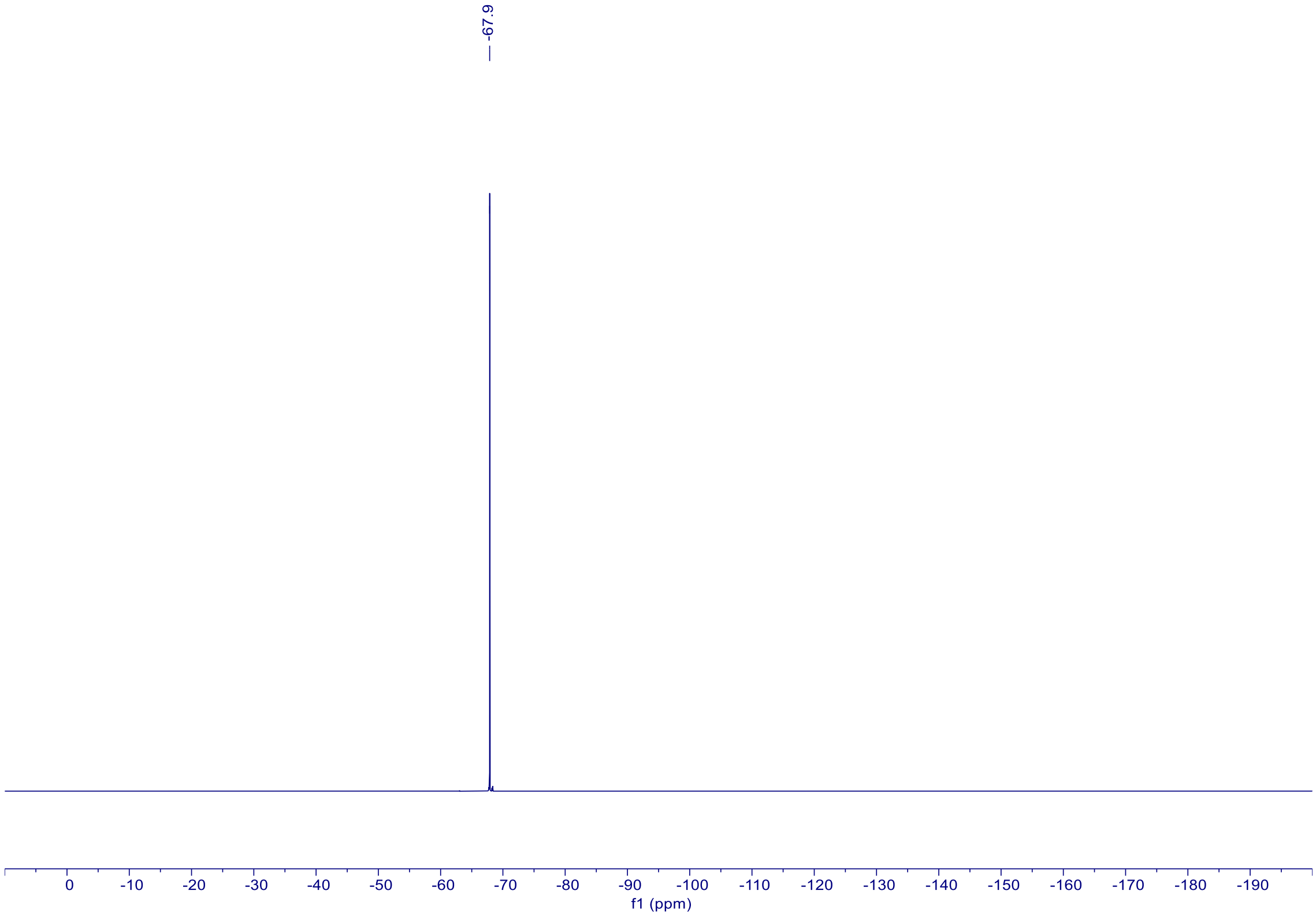

13z – ^{1}H NMR (600 MHz, $CDCl_3$)

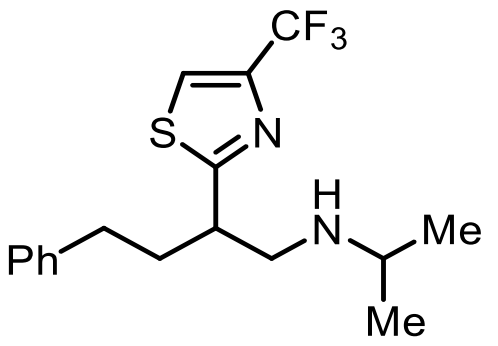

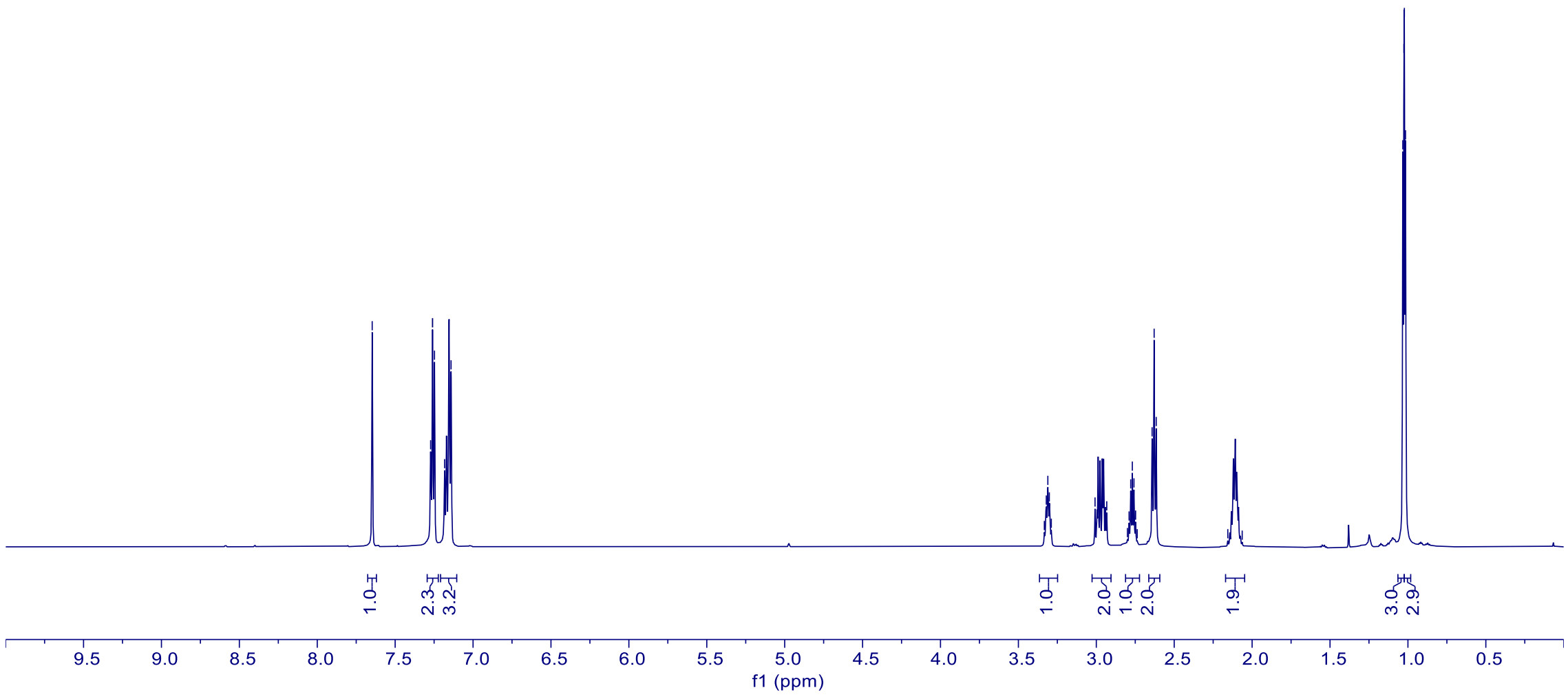

13z – ^{13}C NMR (151 MHz, $CDCl_3$)

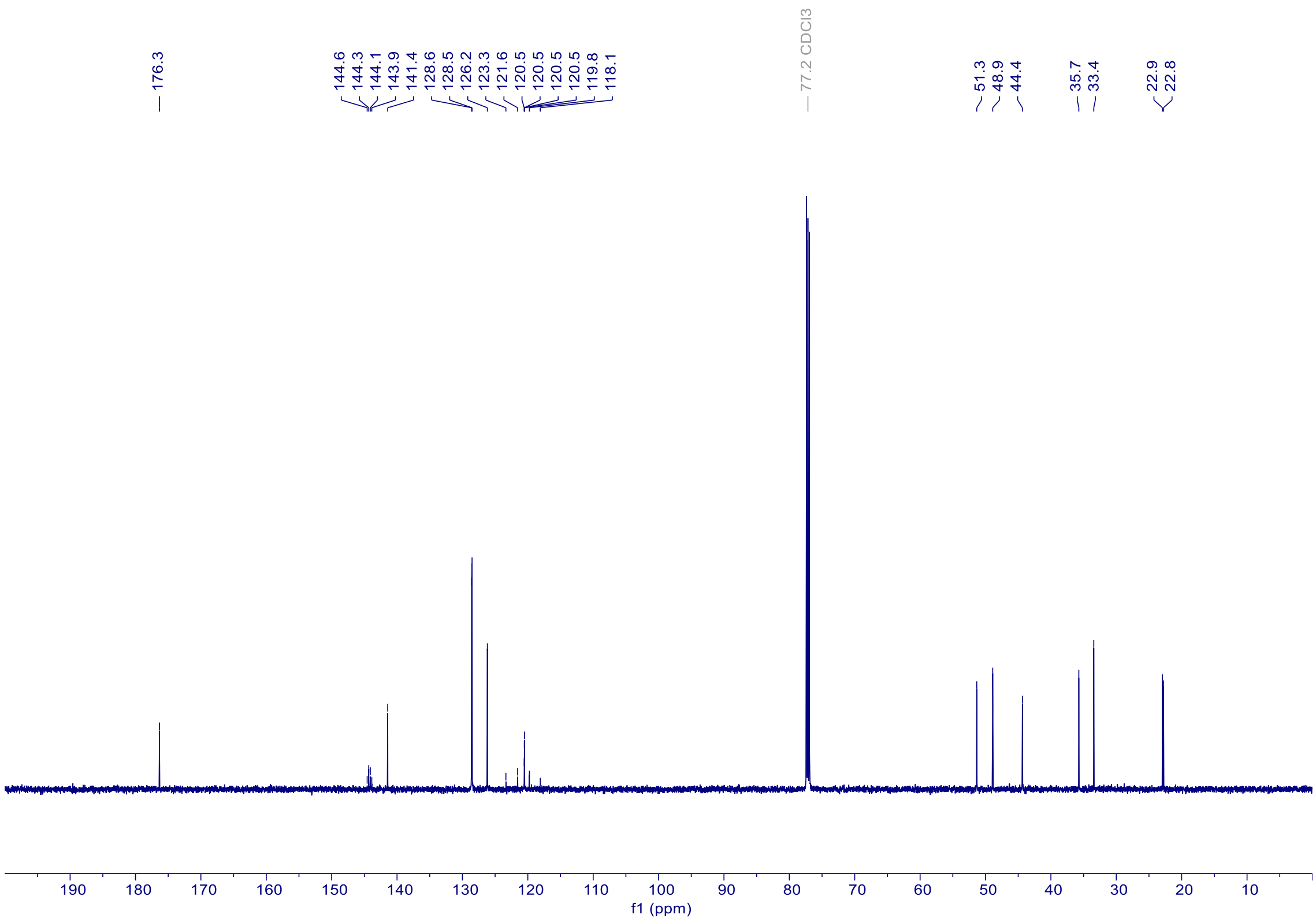

13z – ^{19}F NMR (564 MHz, $CDCl_3$)

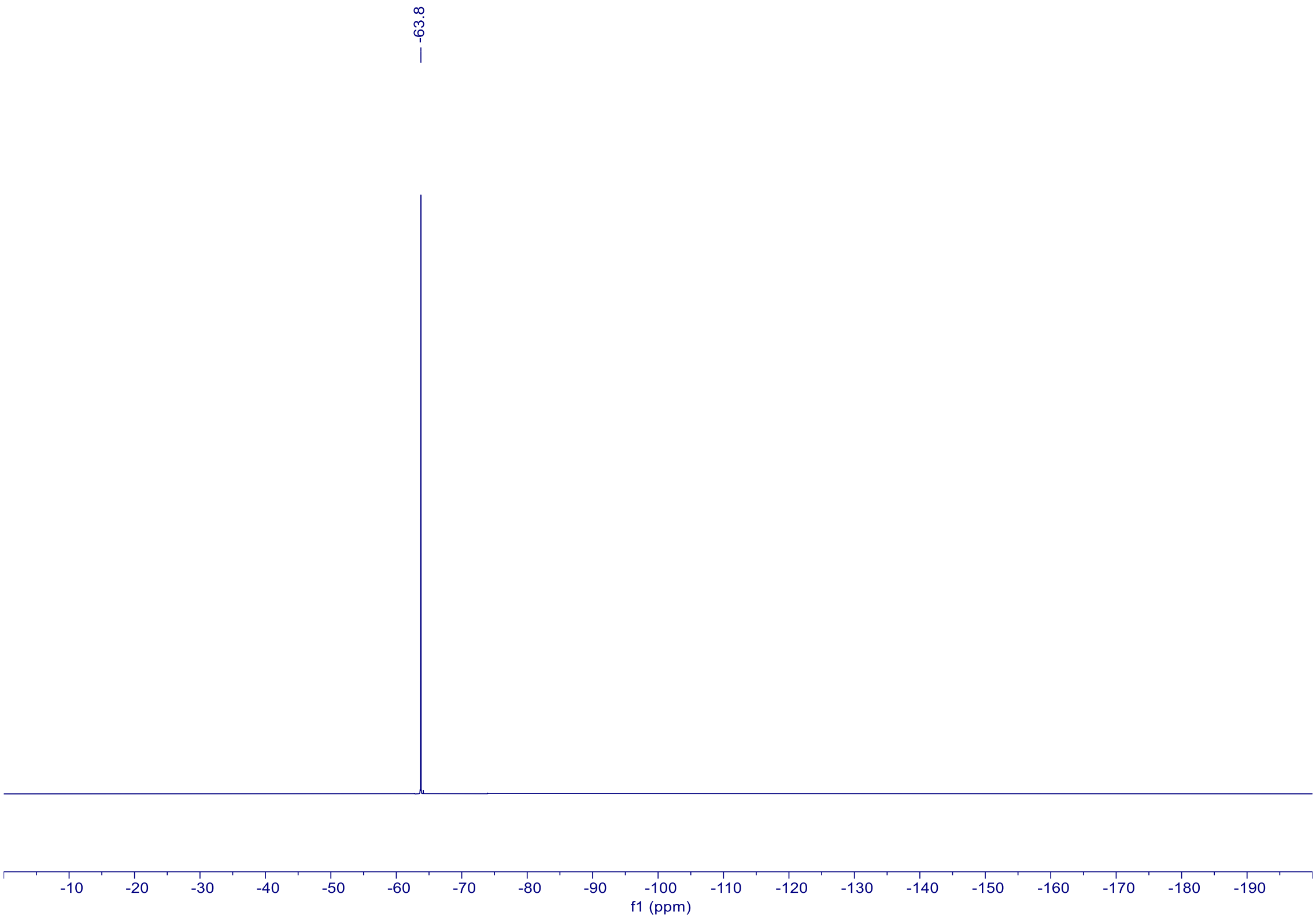

13ab – ^{1}H NMR (600 MHz, $CDCl_3$)

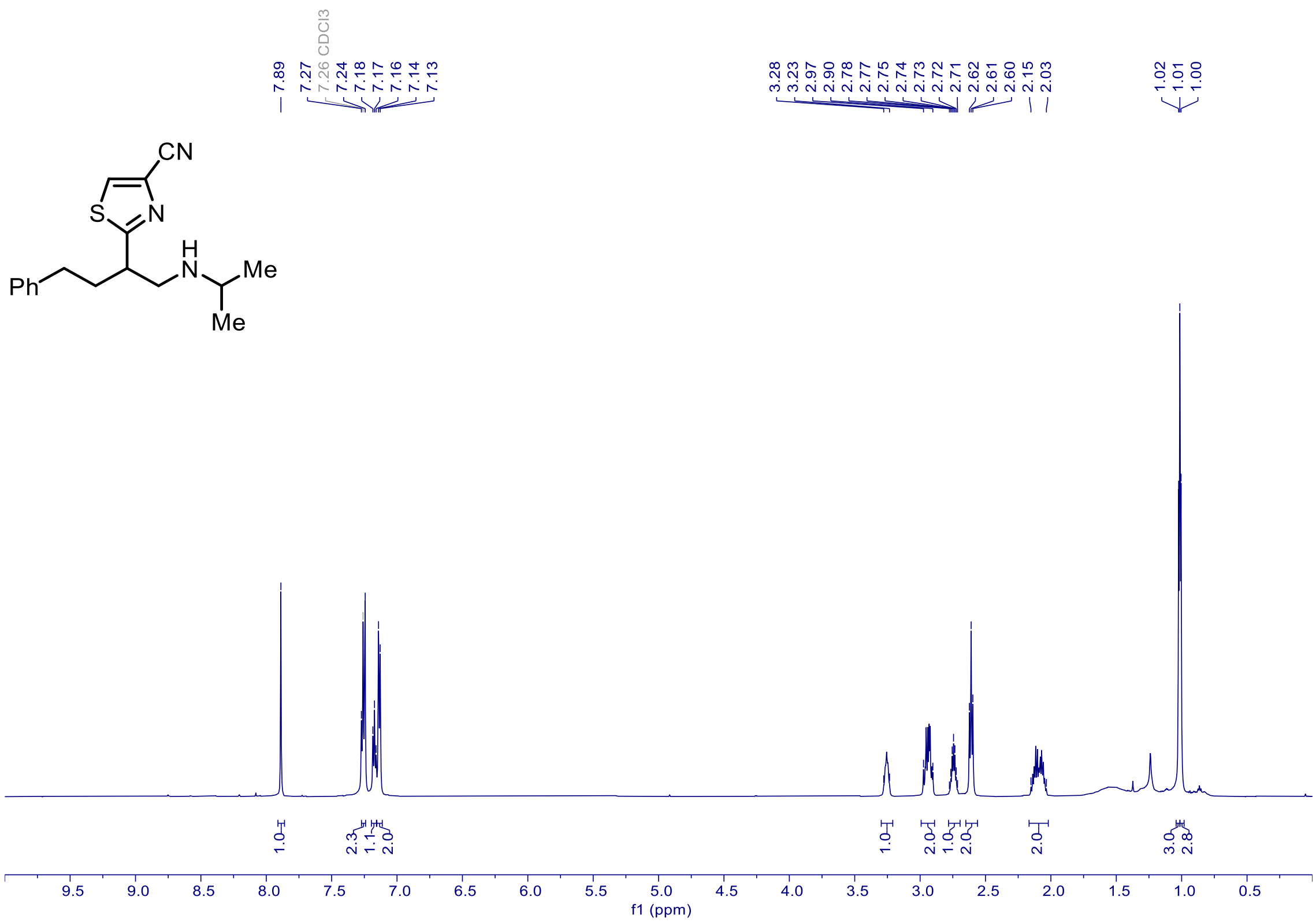

13ab – ^{13}C NMR (151 MHz, $CDCl_3$)

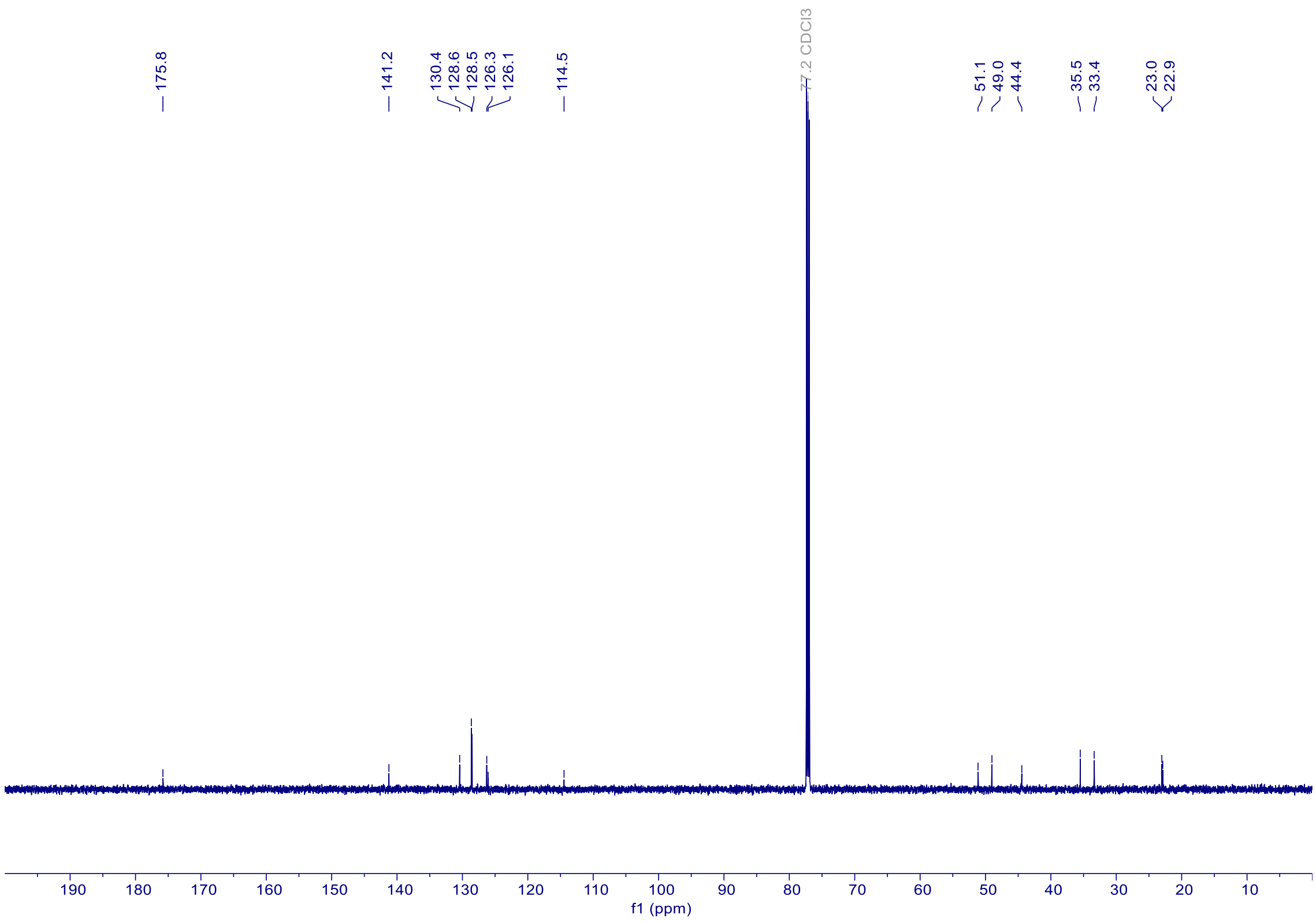

13ac – ^{1}H NMR (600 MHz, $CDCl_3$)

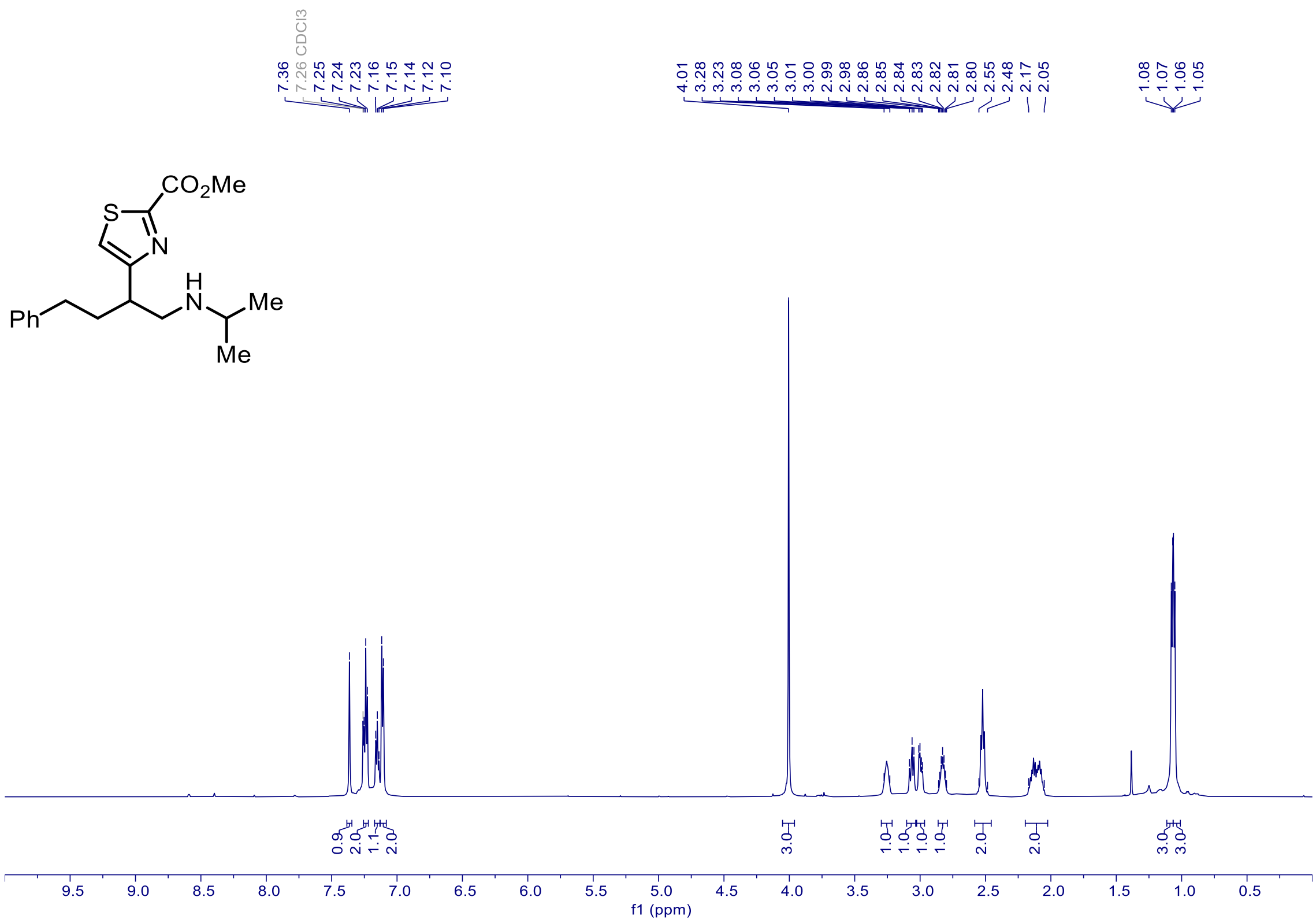

13ac – ^{13}C NMR (151 MHz, $CDCl_3$)

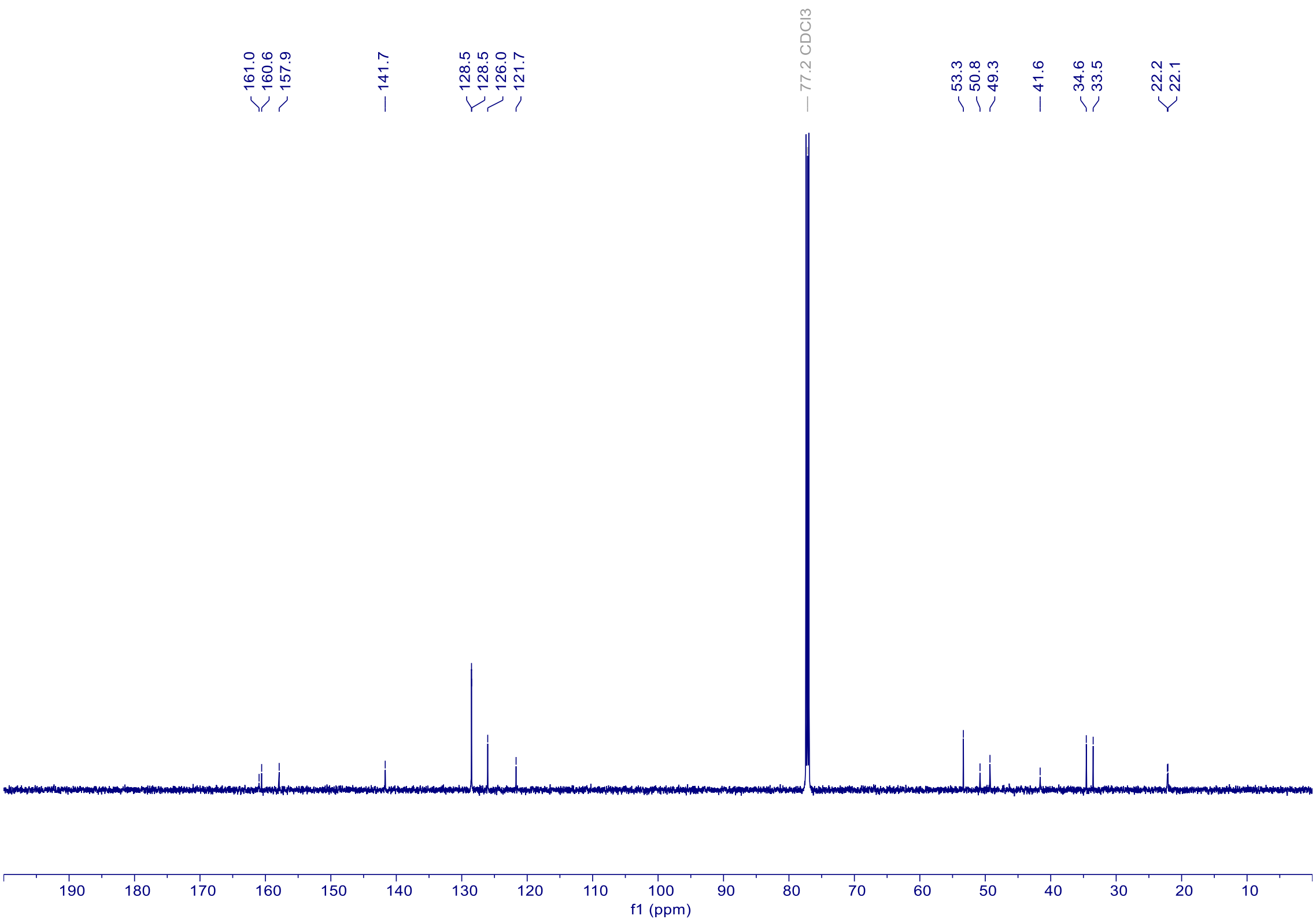

1t – ^{1}H NMR (600 MHz, CDCl$_3$)

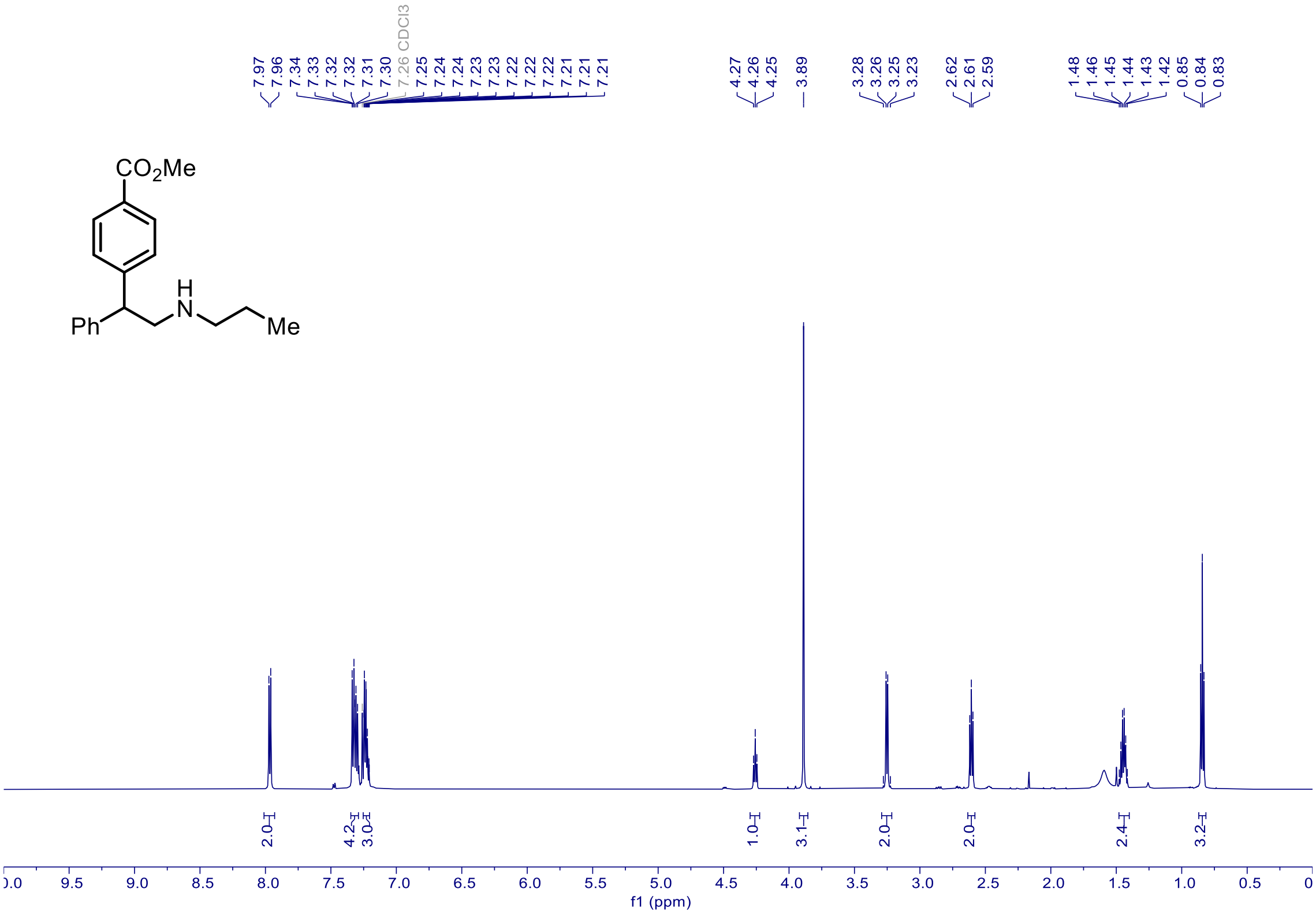

1t – ^{13}C NMR (151 MHz, CDCl$_3$)

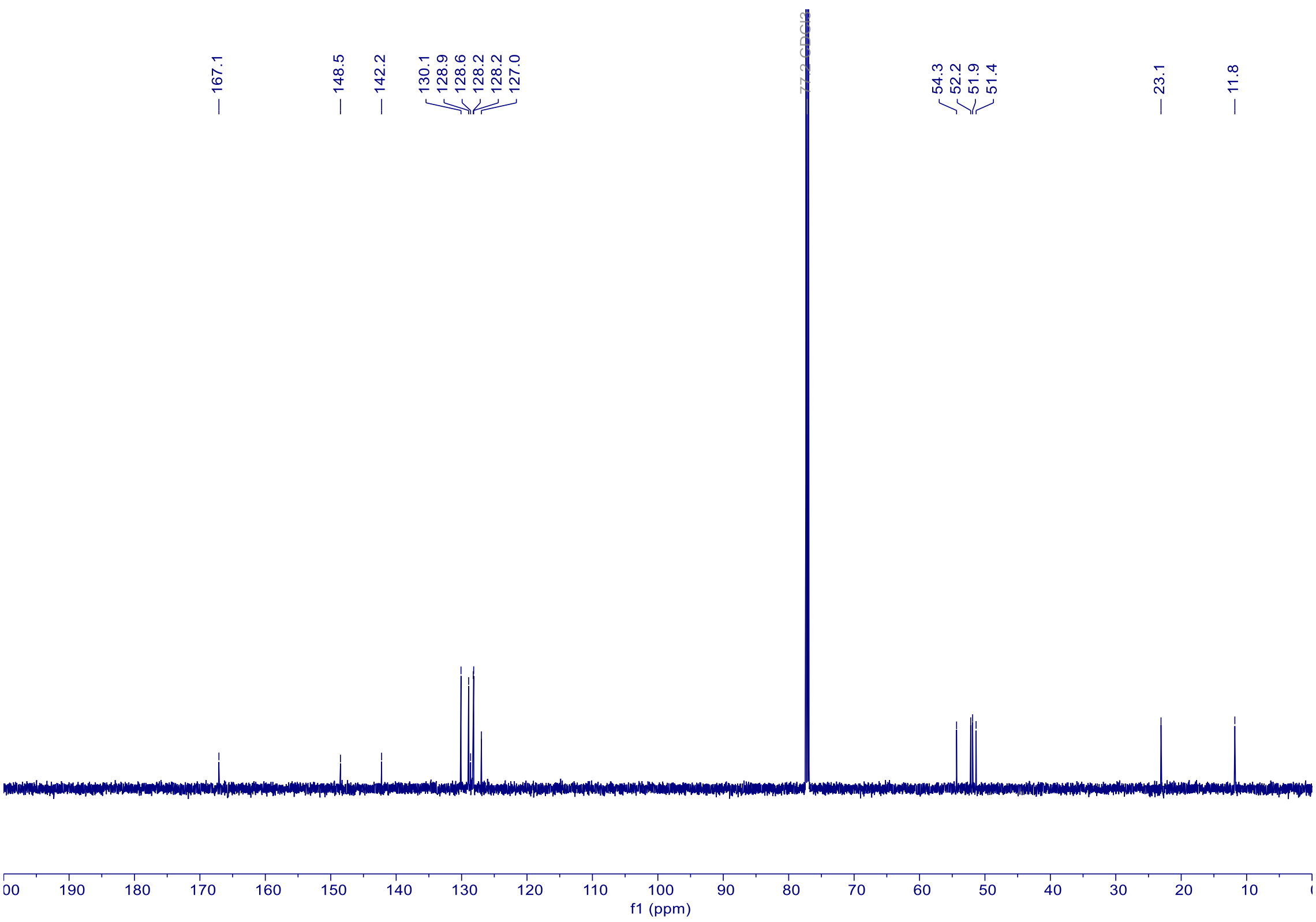

27c – ^{1}H NMR (600 MHz, $CDCl_3$)

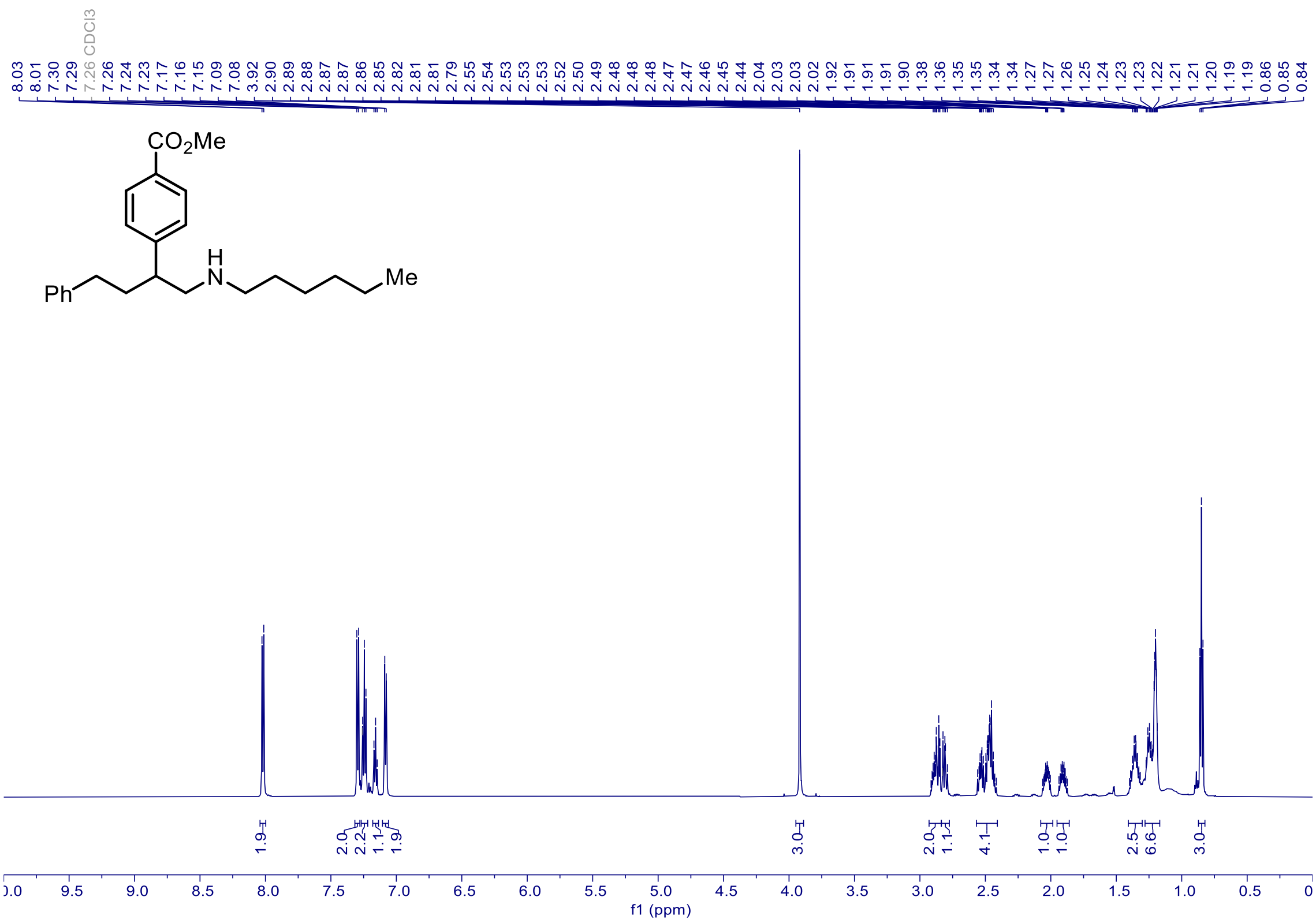

27c – ^{13}C NMR (151 MHz, $CDCl_3$)

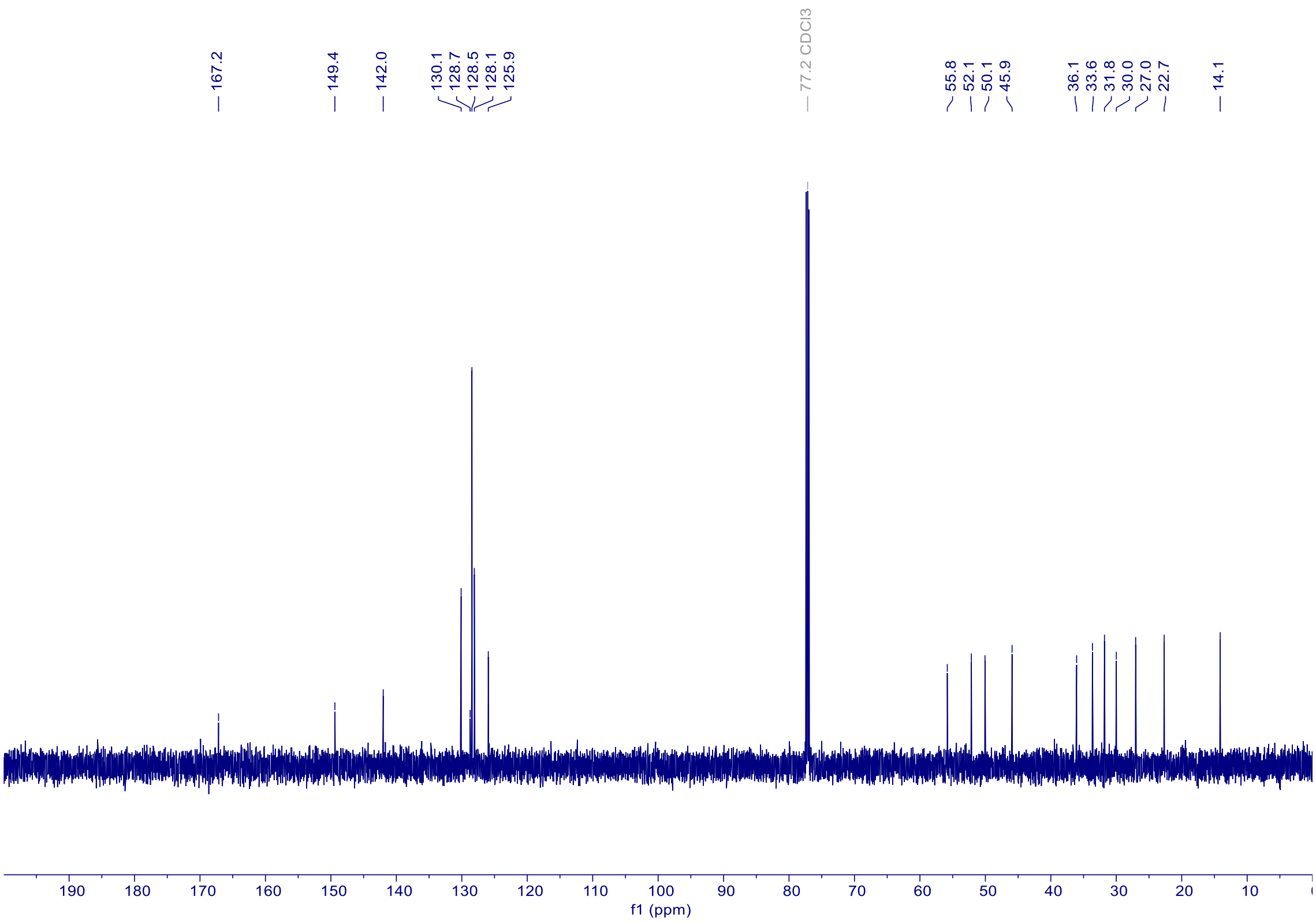

28c – ^{1}H NMR (600 MHz, $CDCl_3$)

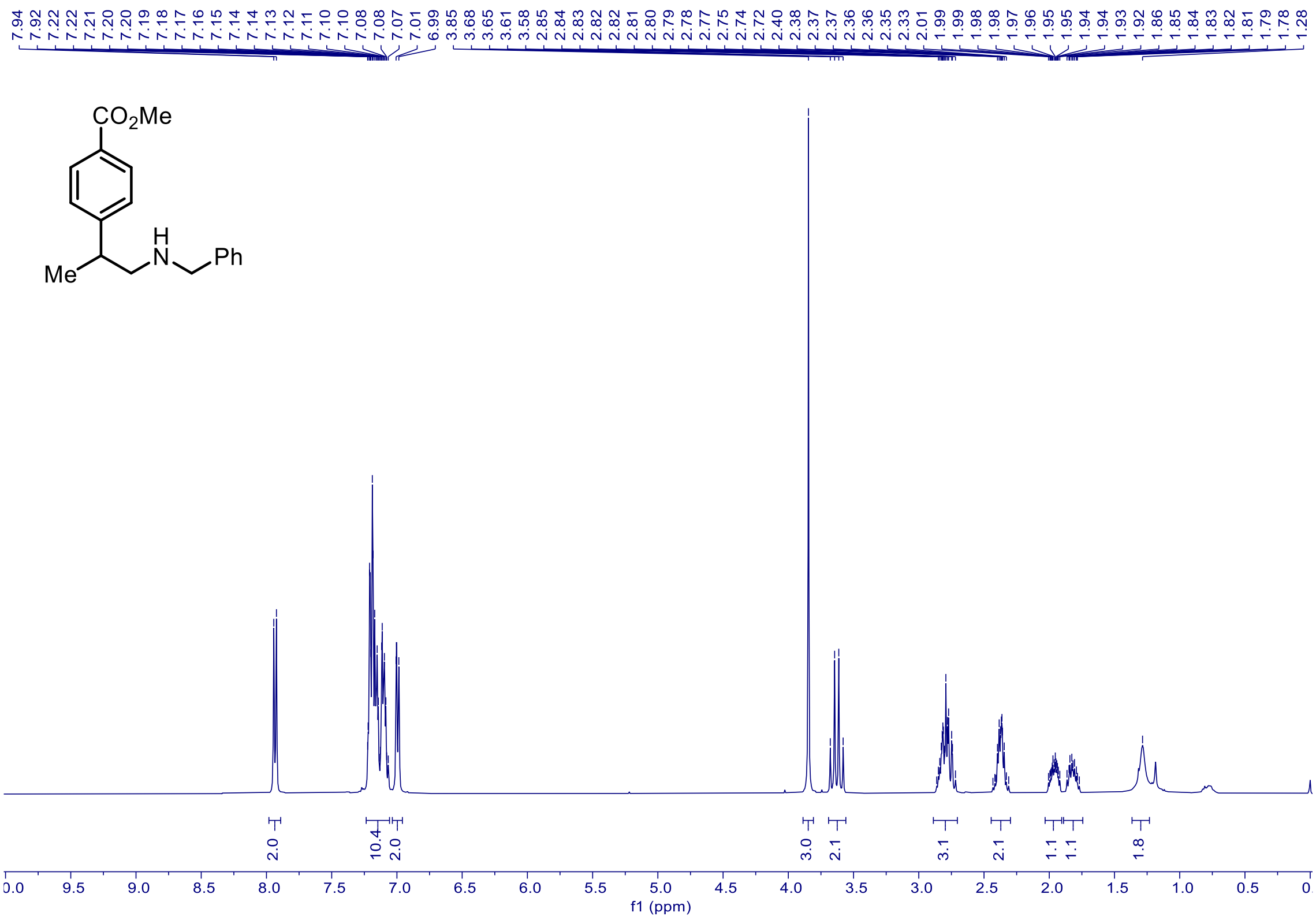

28c – ^{13}C NMR (151 MHz, $CDCl_3$)

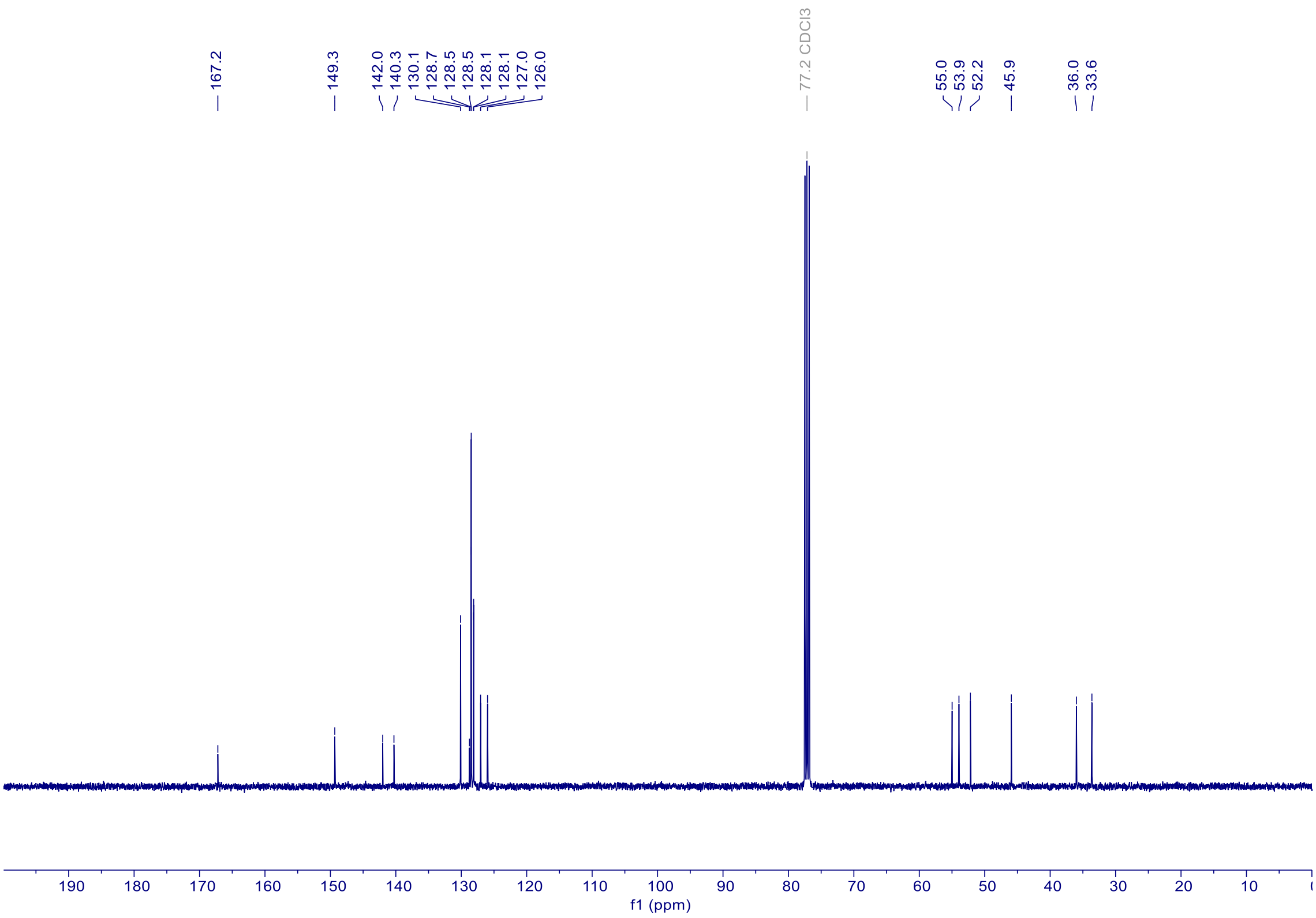

29c – ^{1}H NMR (600 MHz, $CDCl_3$)

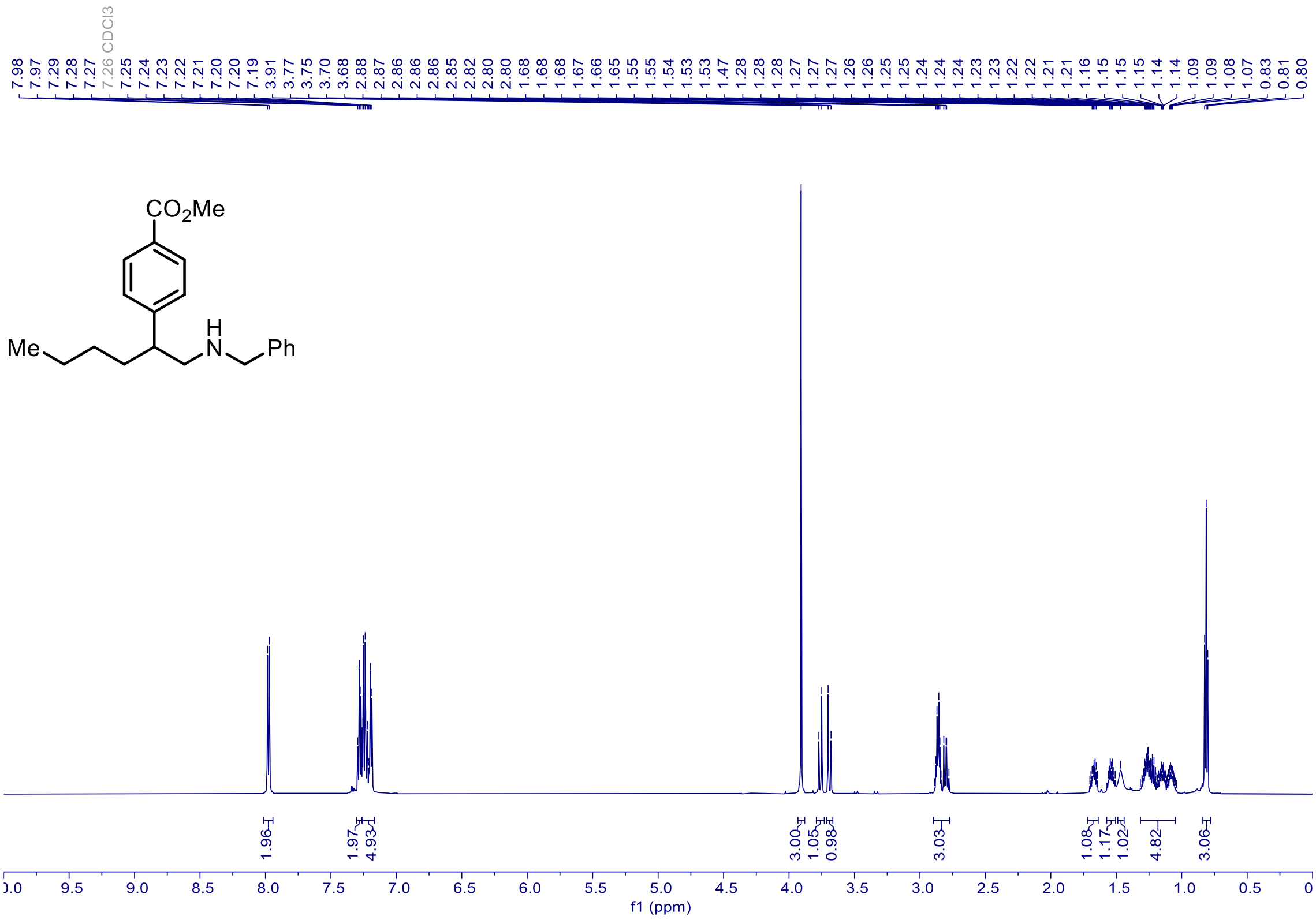

29c – ^{13}C NMR (151 MHz, $CDCl_3$)

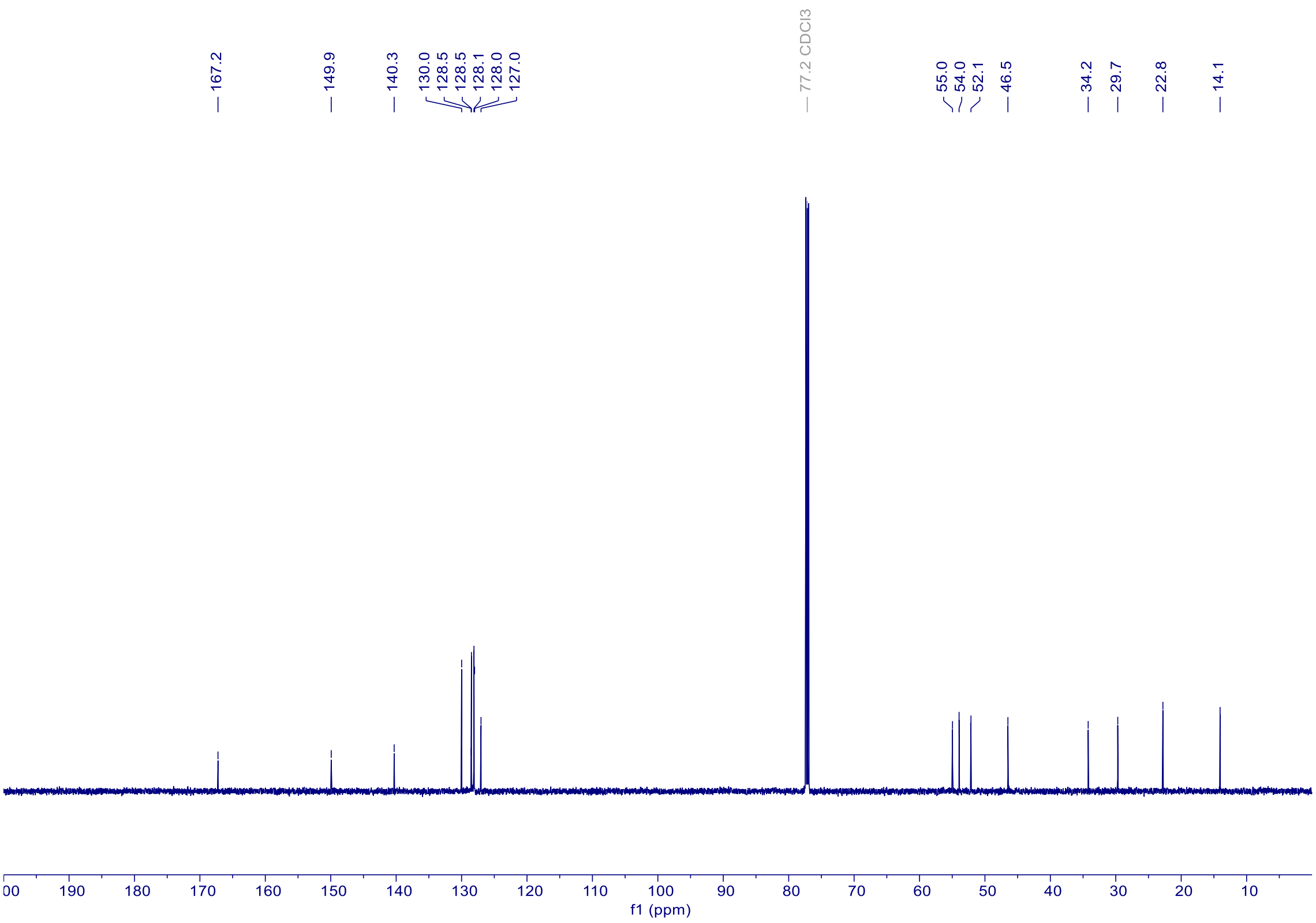

38b – ^{1}H NMR (600 MHz, $CDCl_3$)

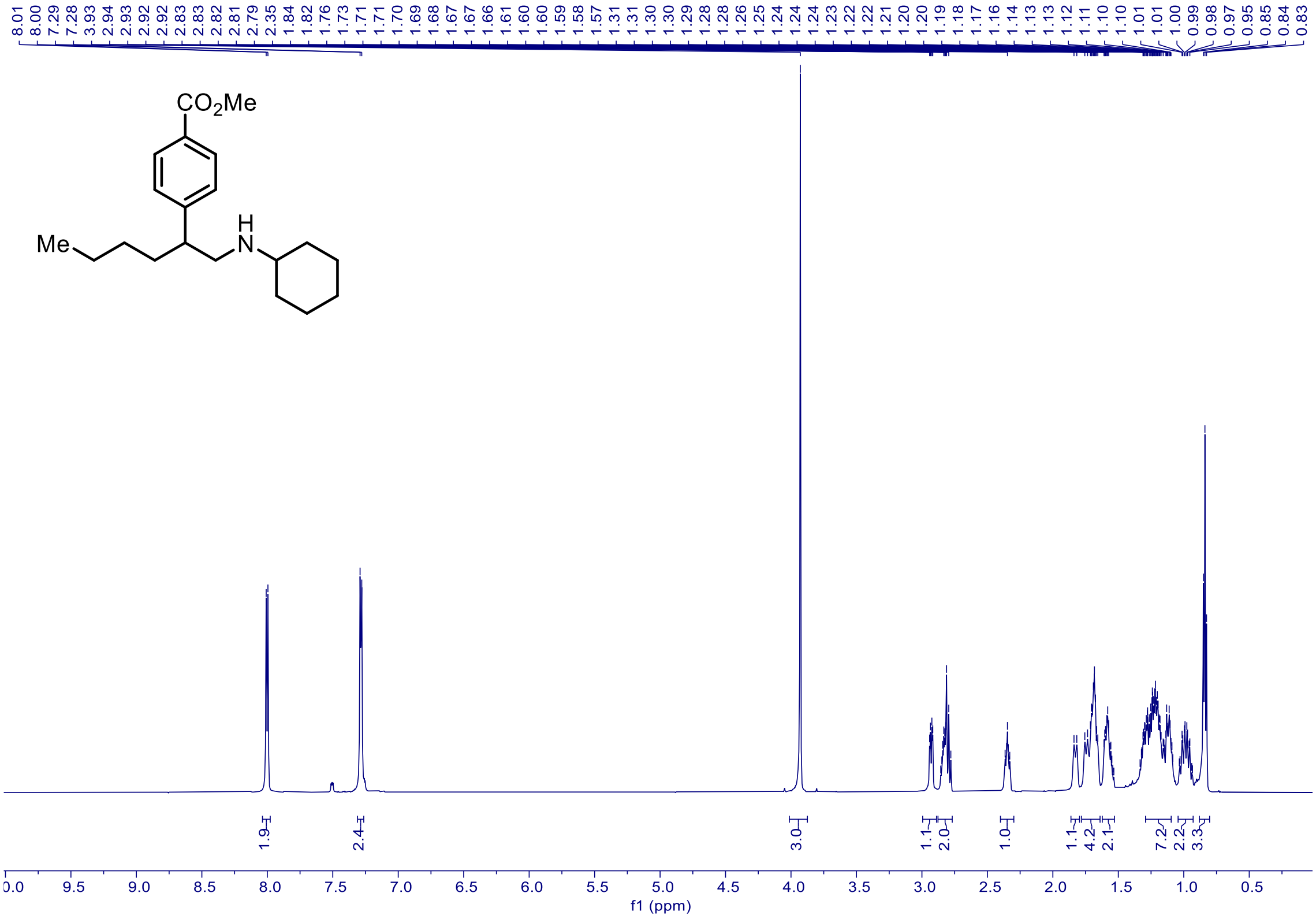

38b – ^{13}C NMR (151 MHz, $CDCl_3$)

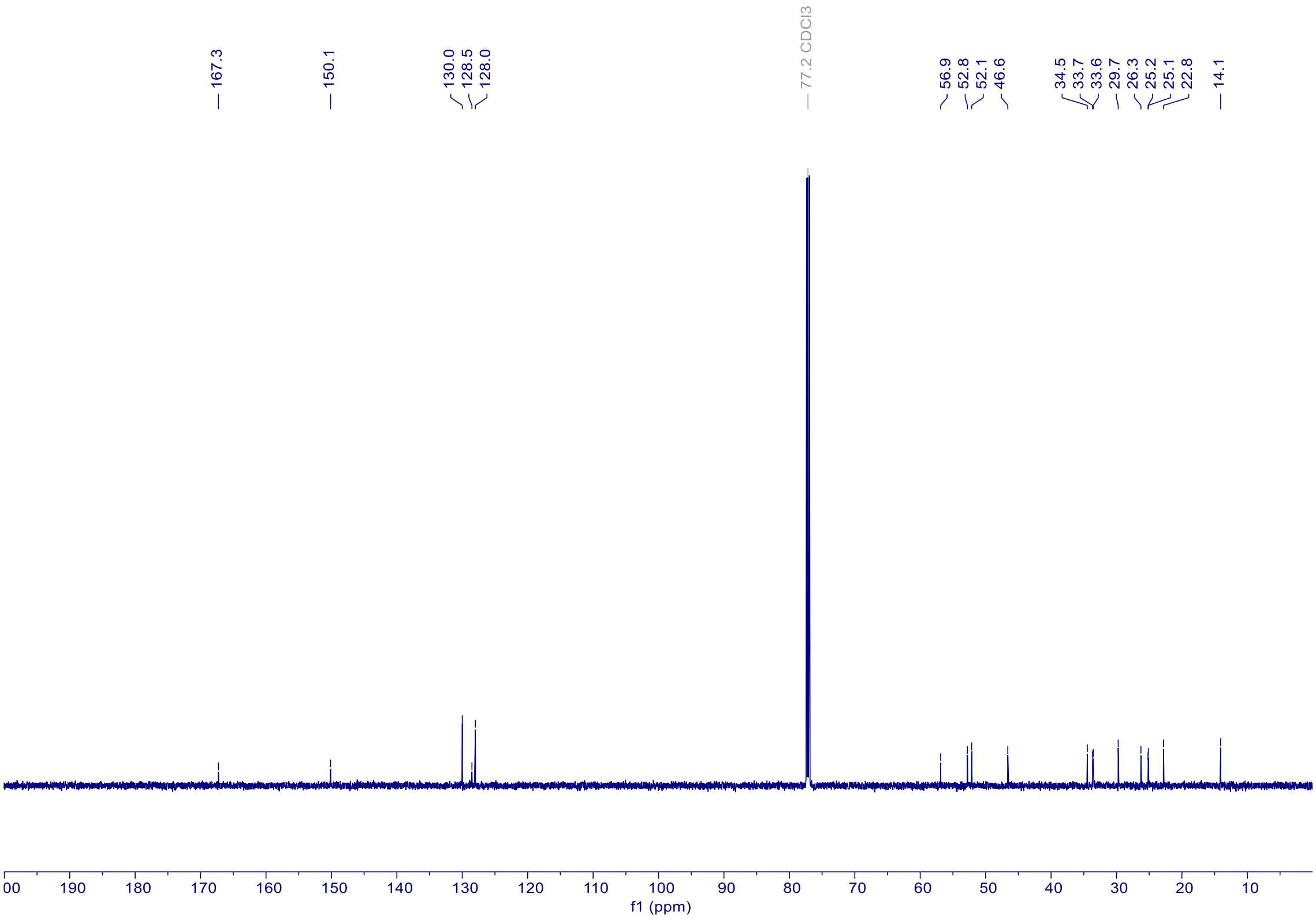

39b – ^{1}H NMR (600 MHz, $CDCl_3$)

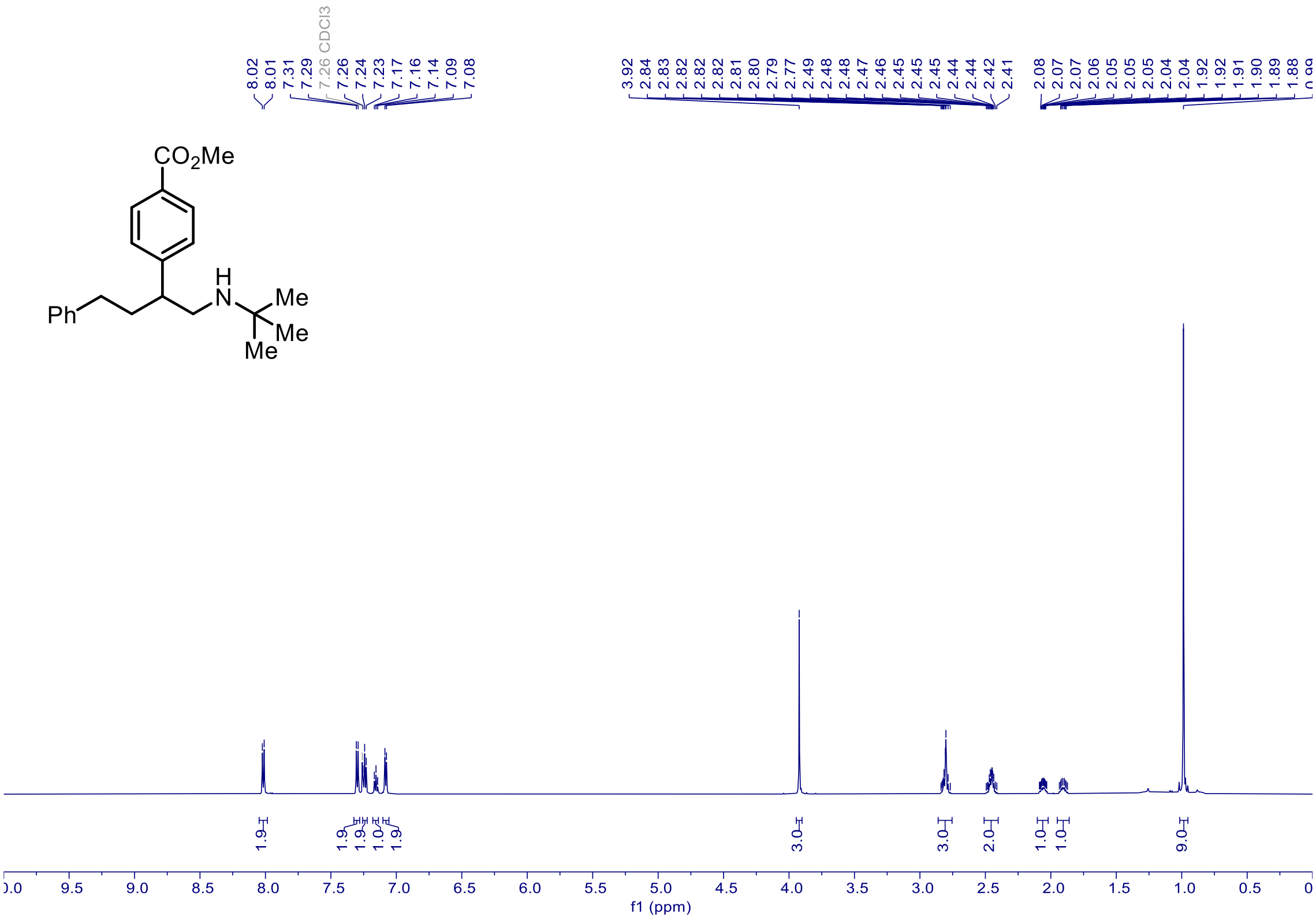

39b – ^{13}C NMR (151 MHz, $CDCl_3$)

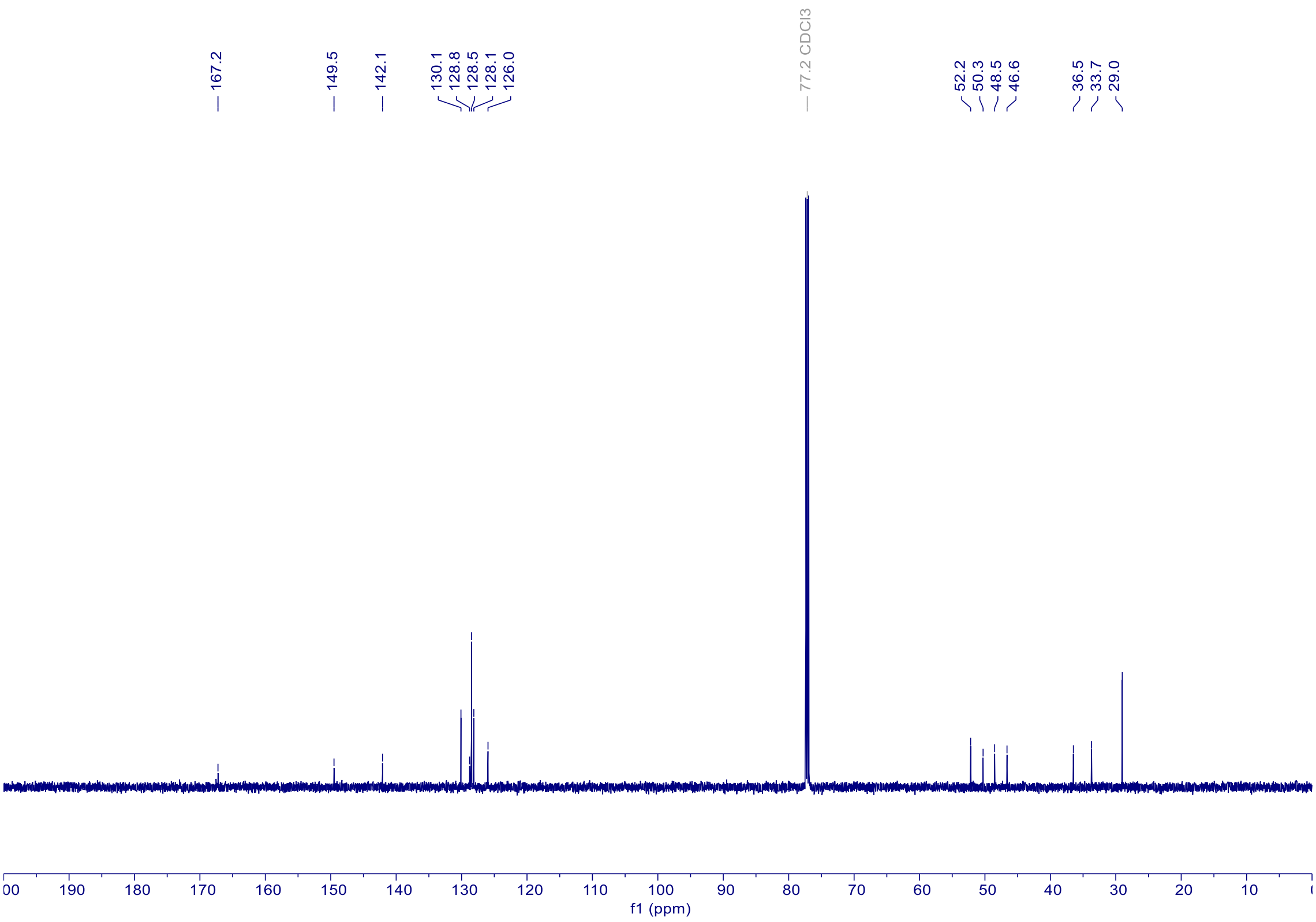

31d – ^{1}H NMR (400 MHz, $CDCl_3$)

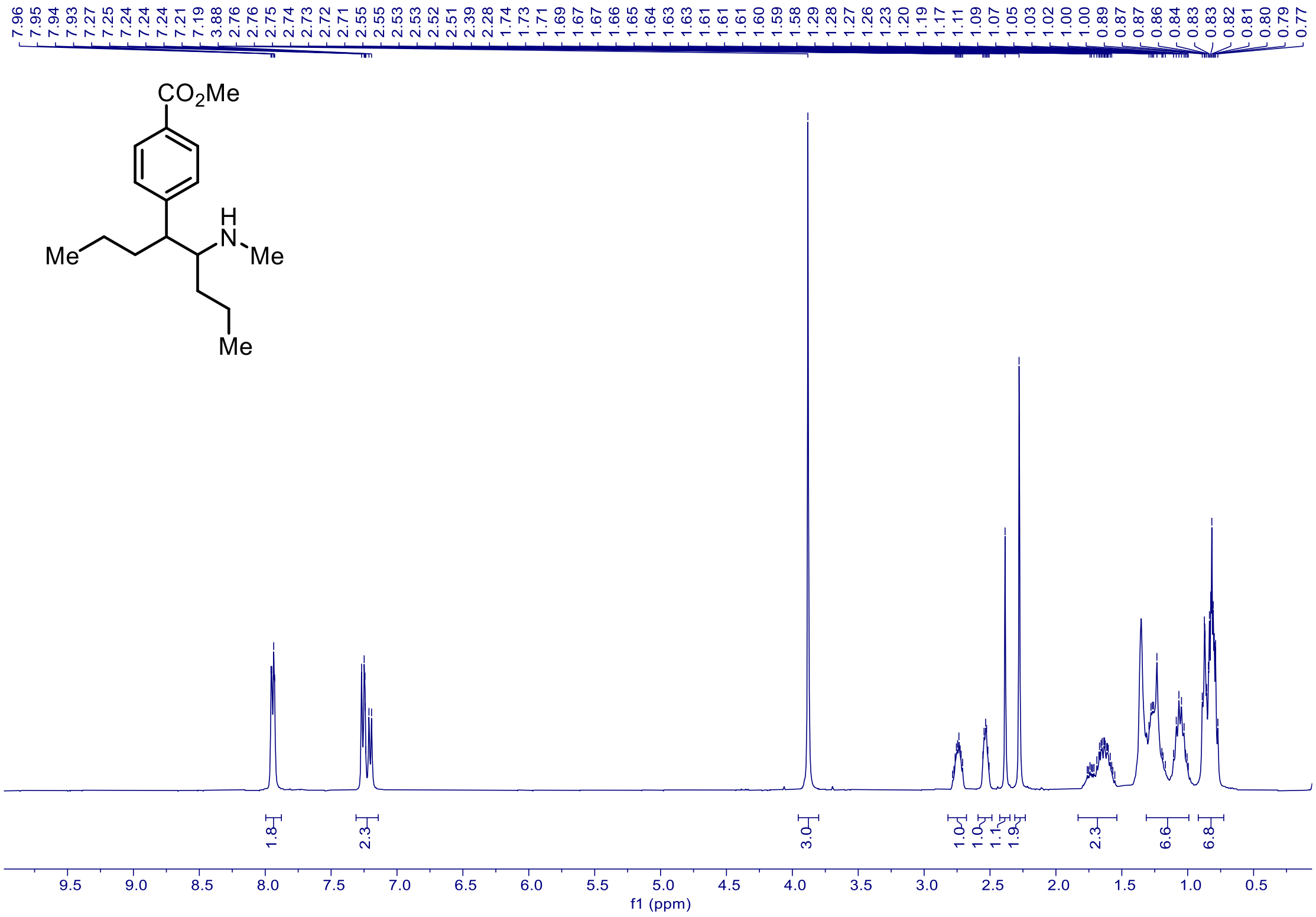

31d – ^{13}C NMR (151 MHz, $CDCl_3$)

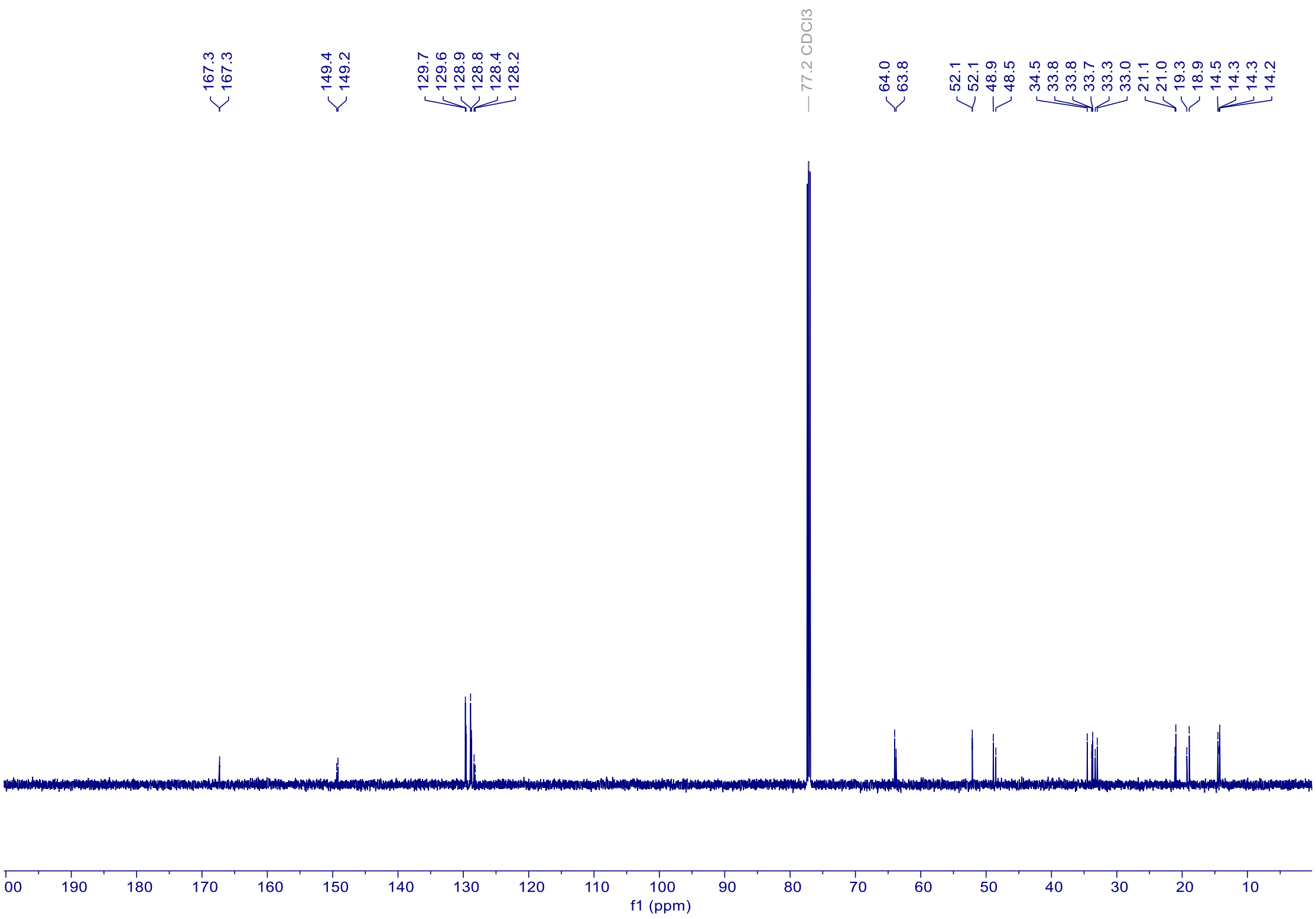

33d – ^{1}H NMR (400 MHz, $CDCl_3$)

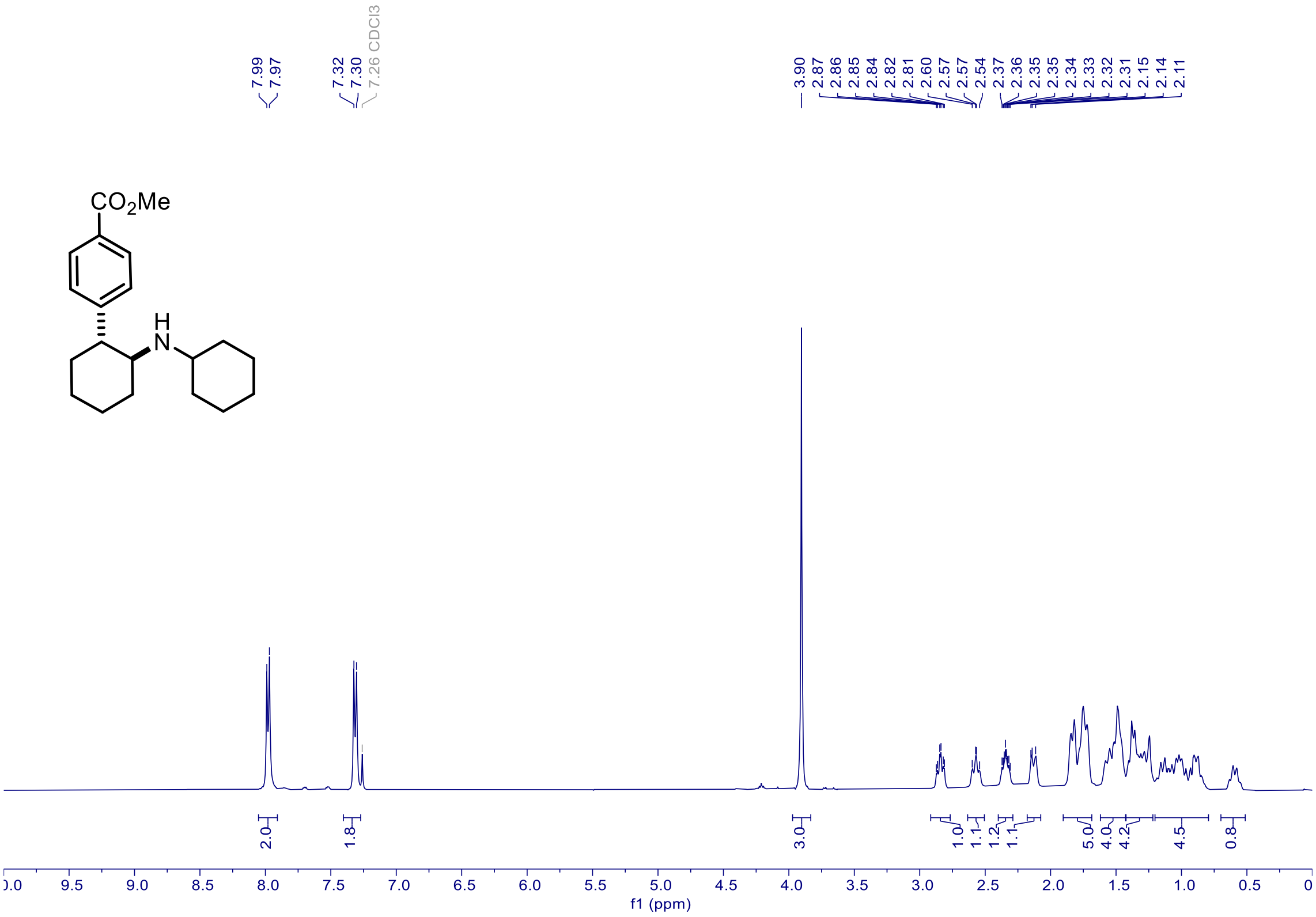

33d – ^{13}C NMR (151 MHz, $CDCl_3$)

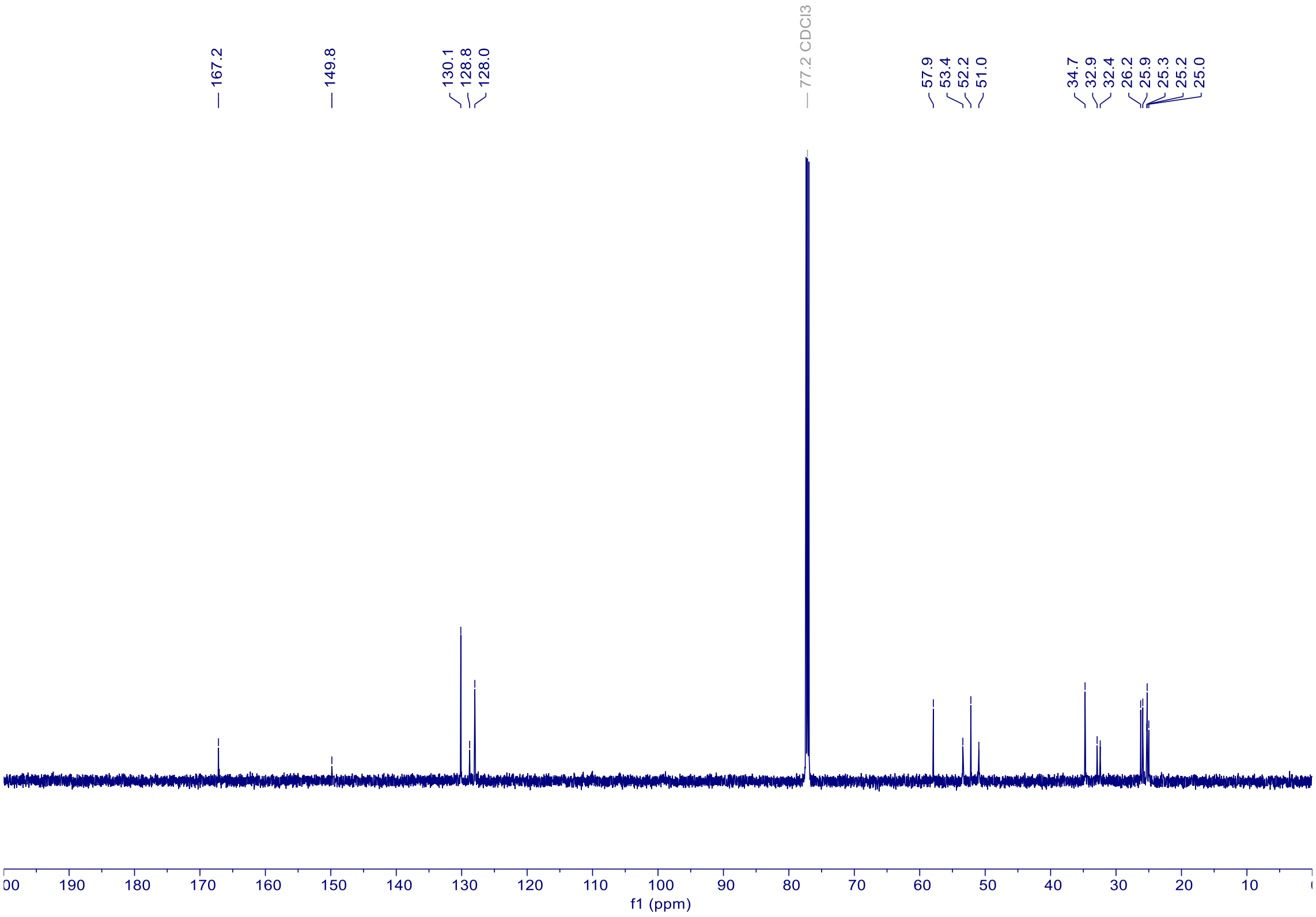

40b – ^{1}H NMR (600 MHz, $CDCl_3$)

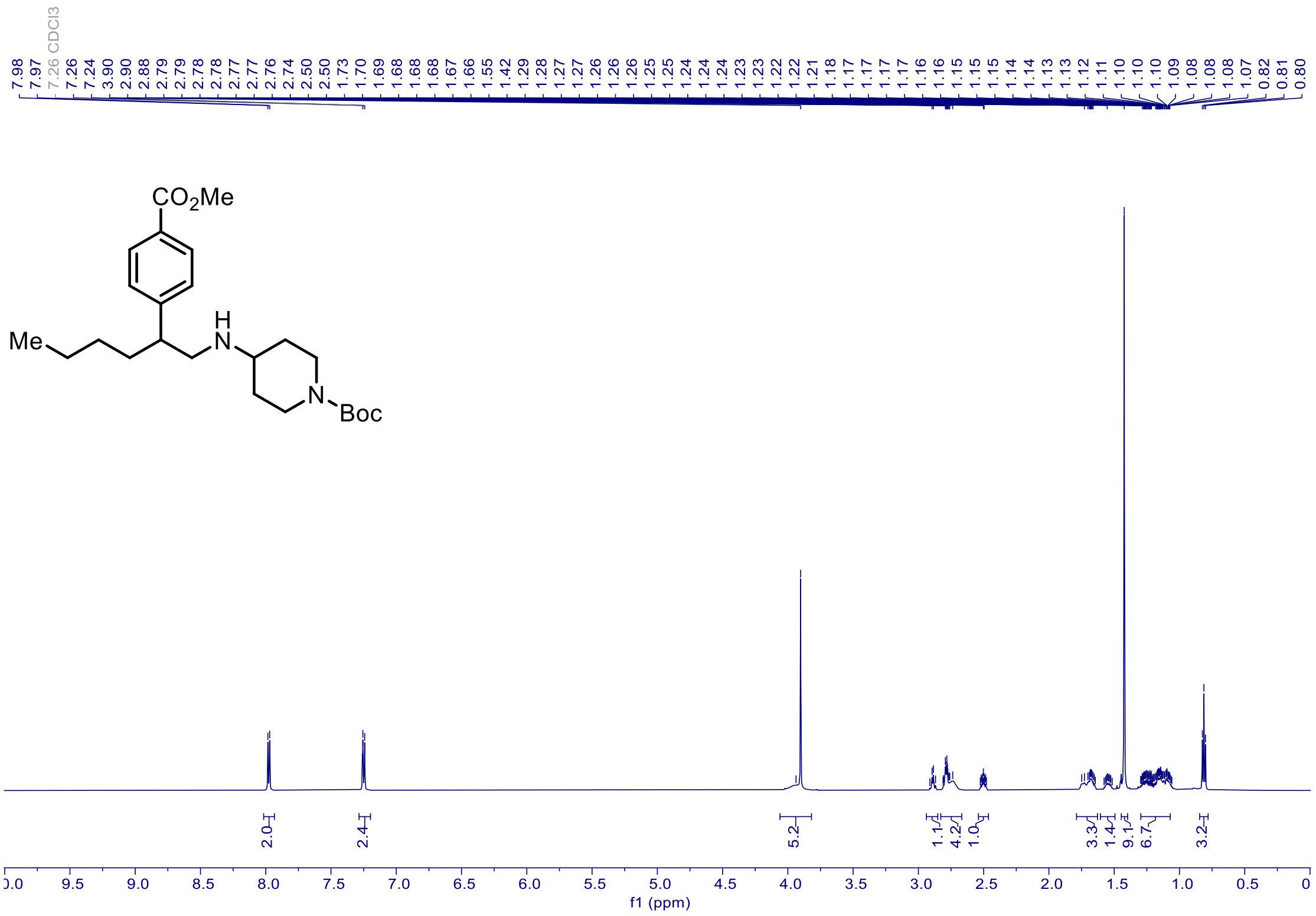

40b – ^{13}C NMR (151 MHz, $CDCl_3$)

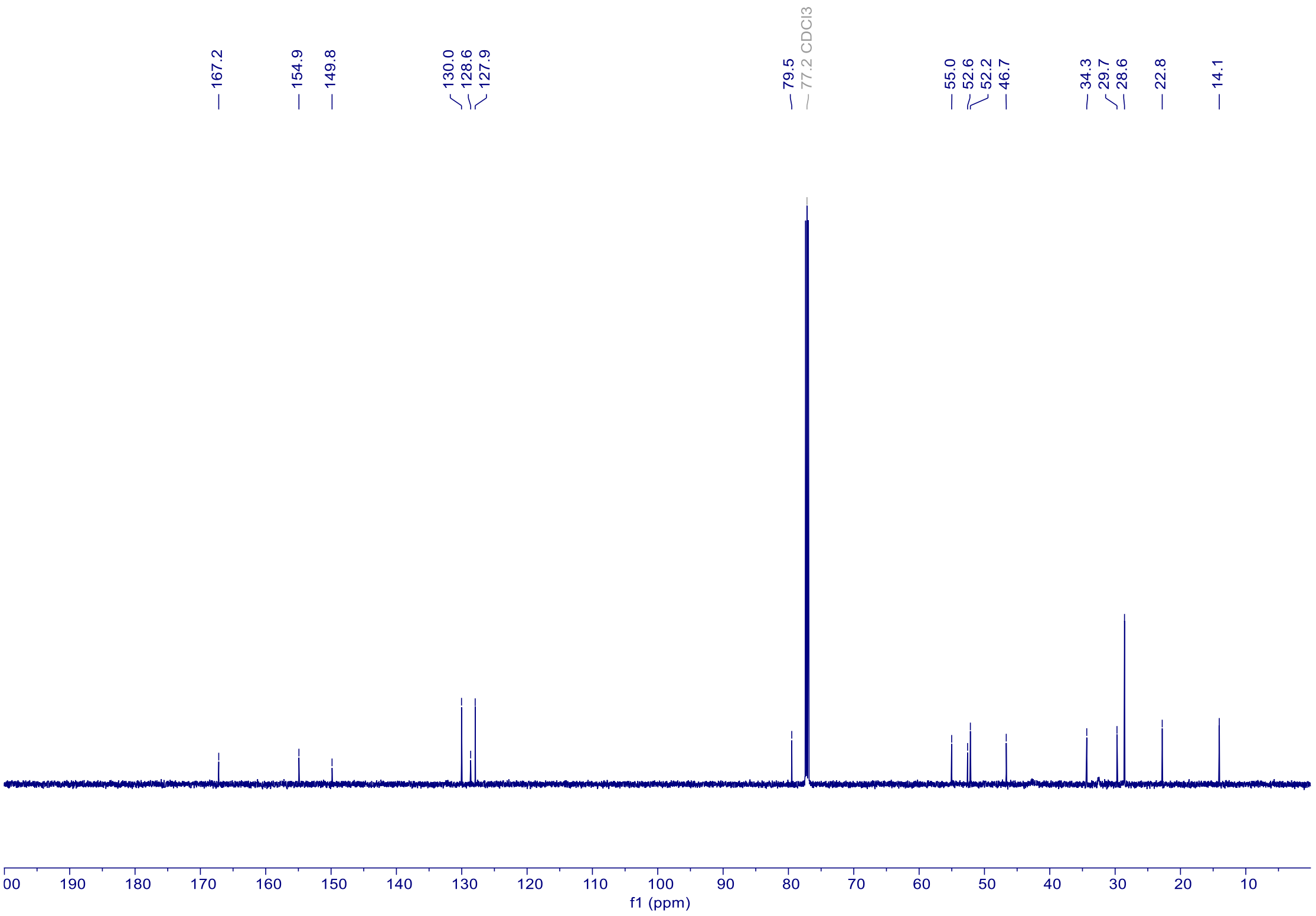

41b – ^{1}H NMR (600 MHz, $CDCl_3$)

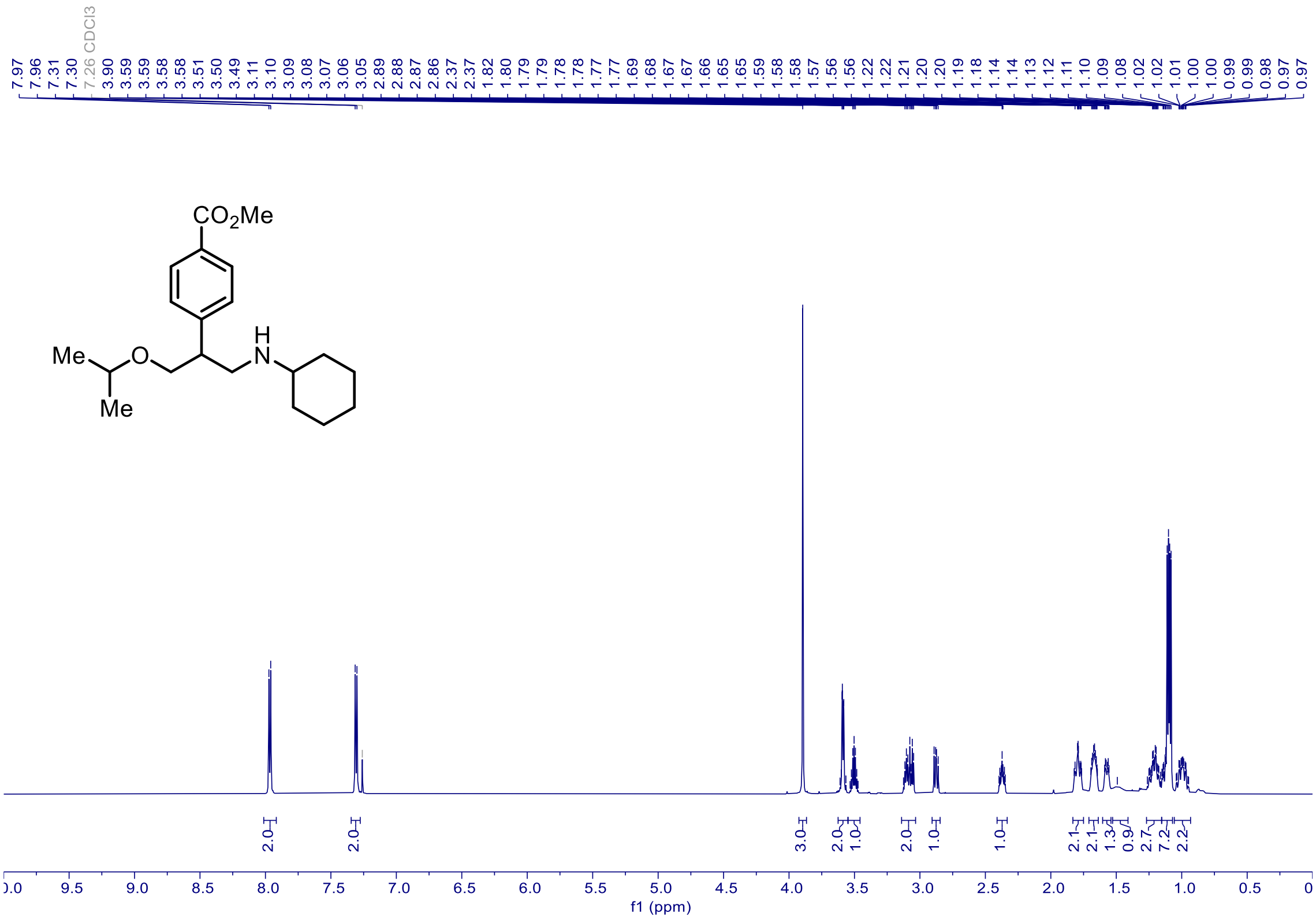

41b – ^{13}C NMR (151 MHz, $CDCl_3$)

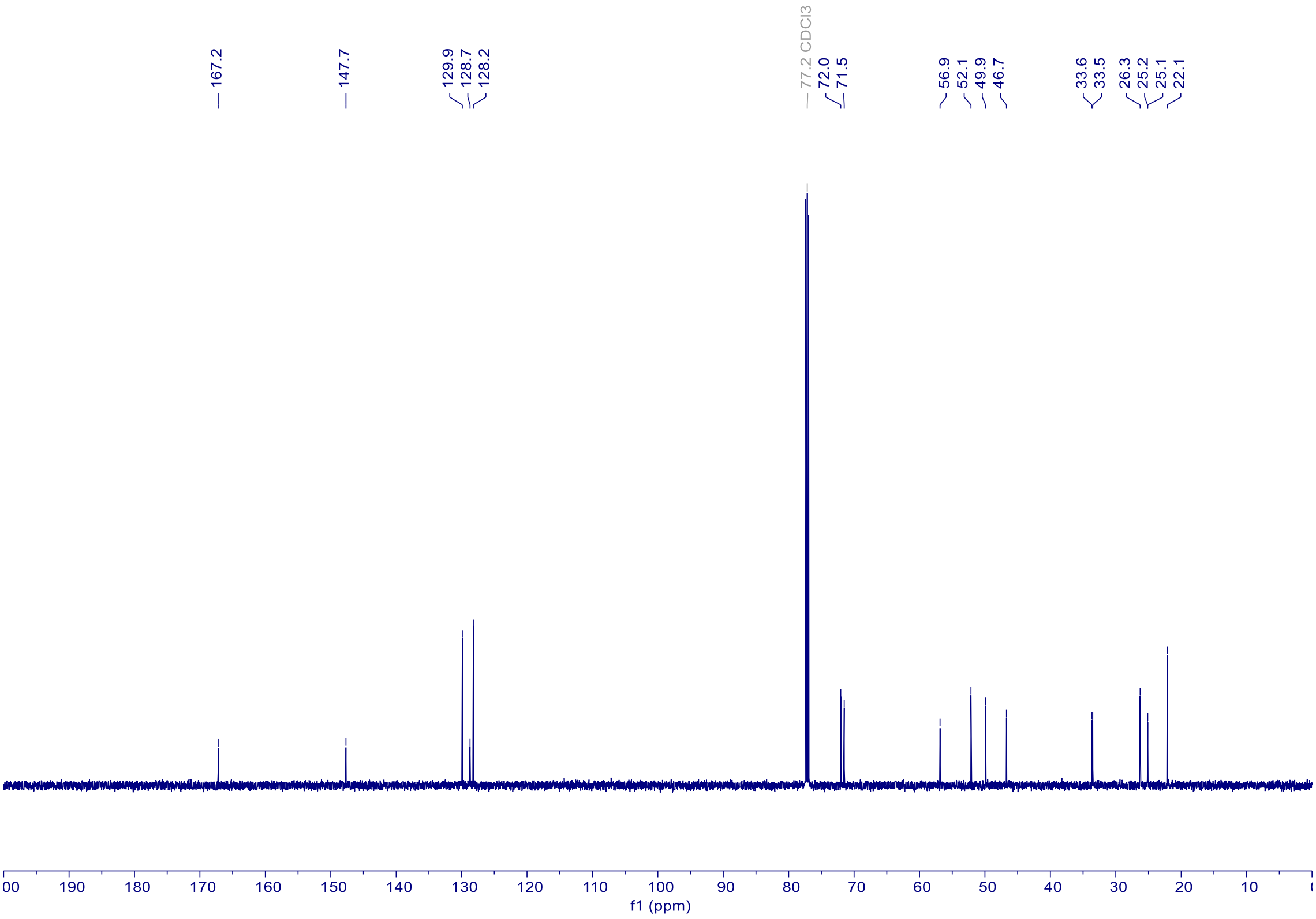

42b – ^{1}H NMR (400 MHz, $CDCl_3$)

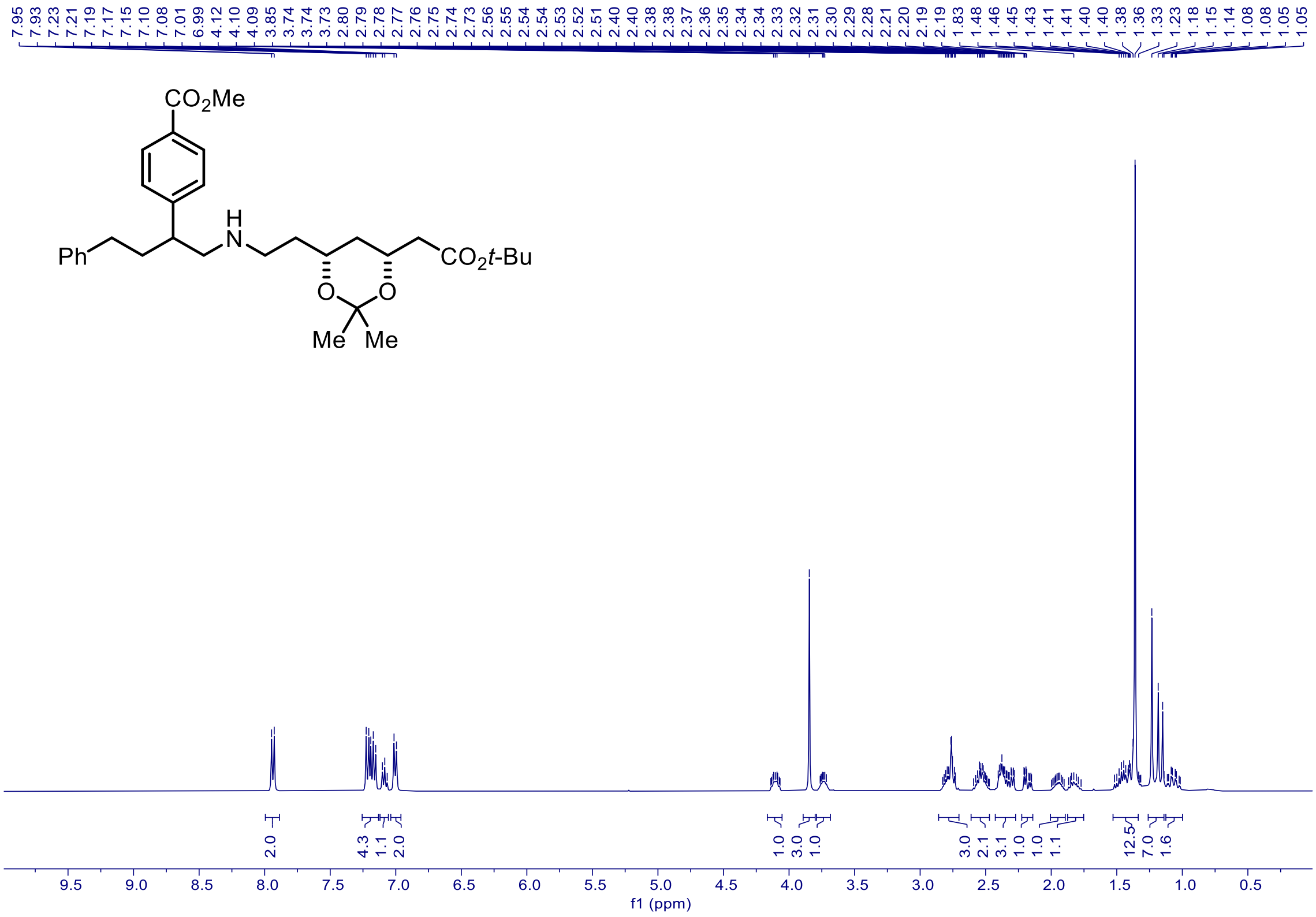

42b – ^{13}C NMR (101 MHz, $CDCl_3$)

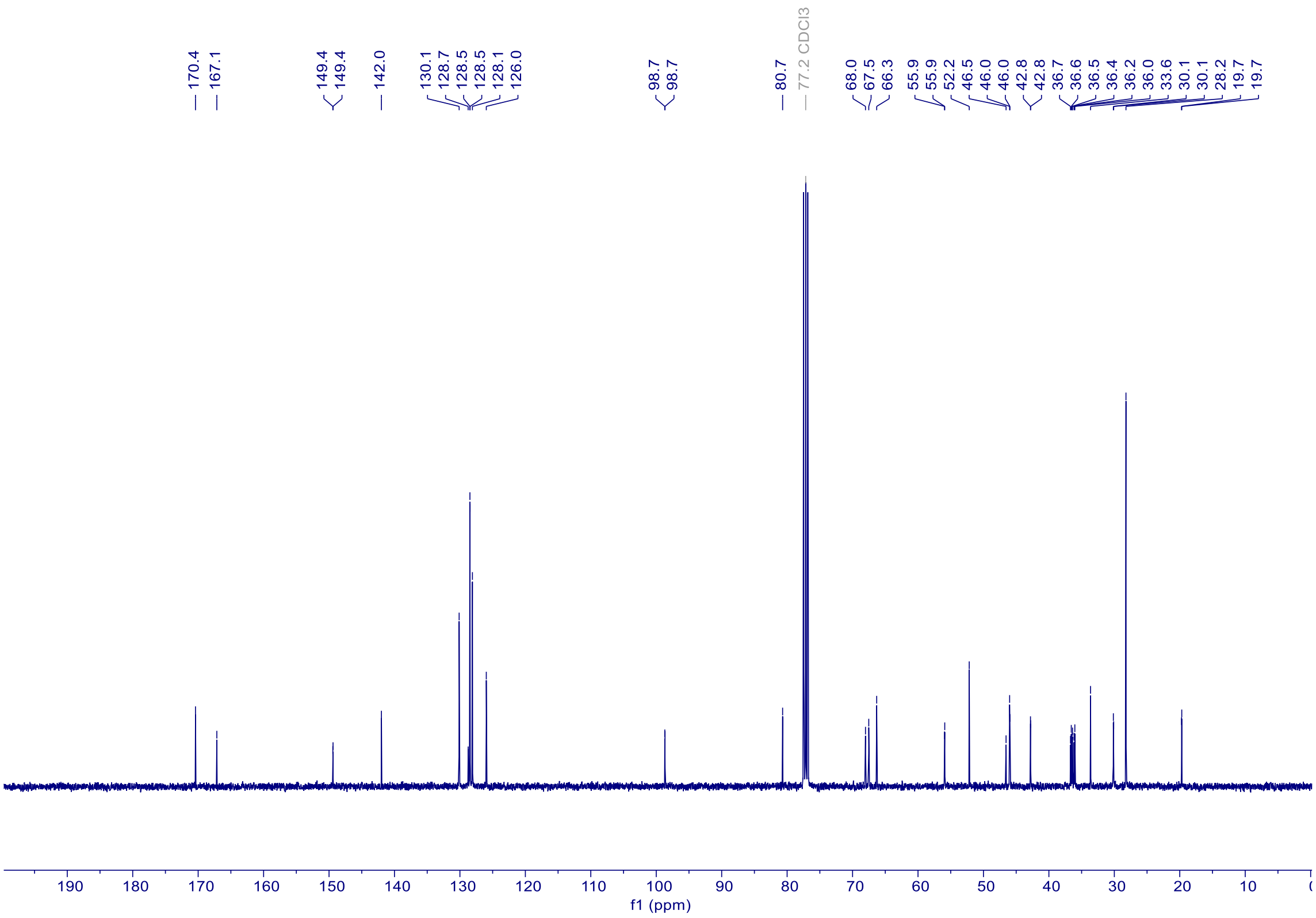

S14 – ^{1}H NMR (400 MHz, $CDCl_3$)

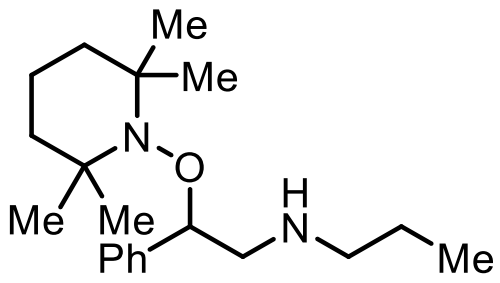

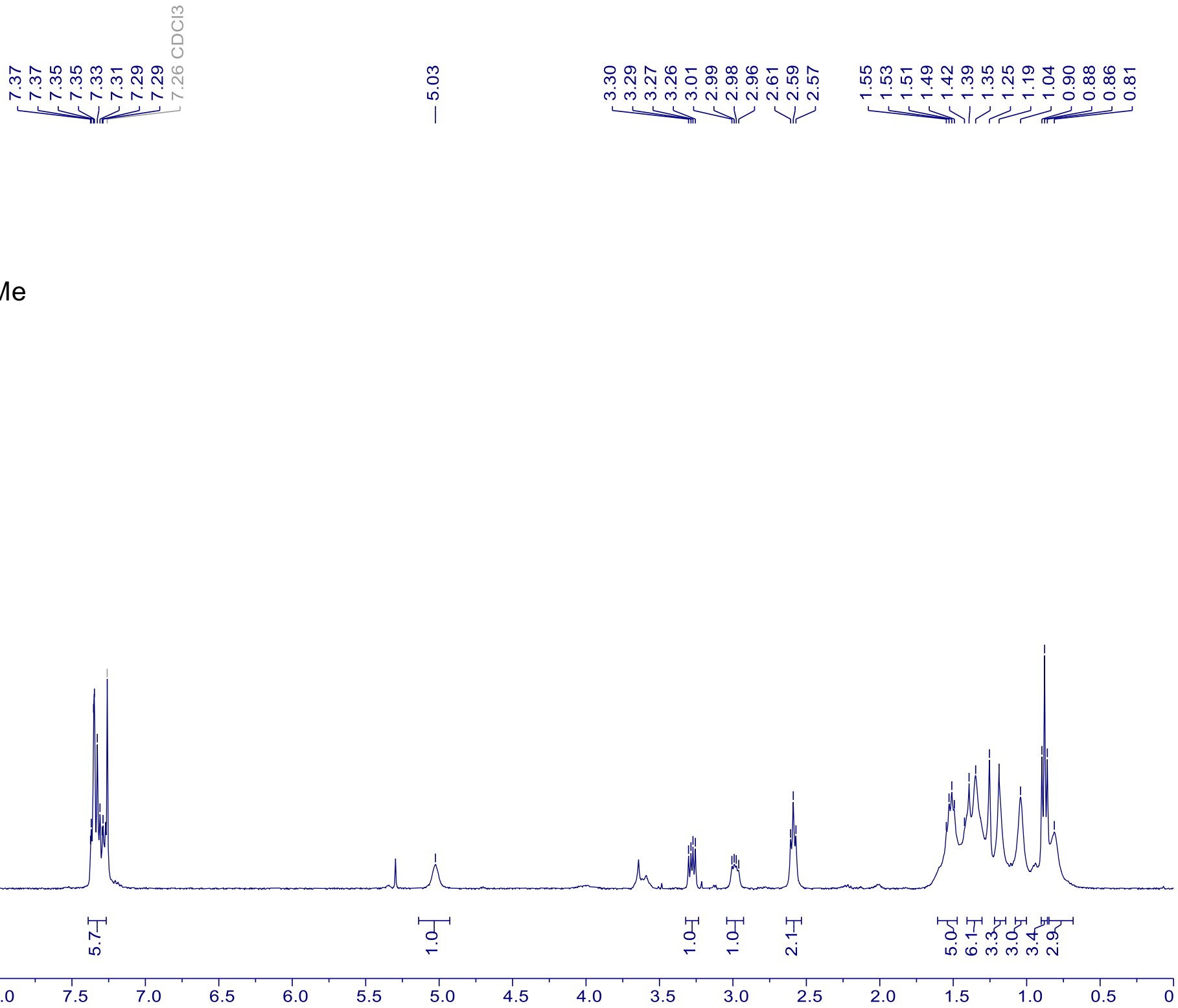

S14 – ^{13}C NMR (101 MHz, $CDCl_3$)

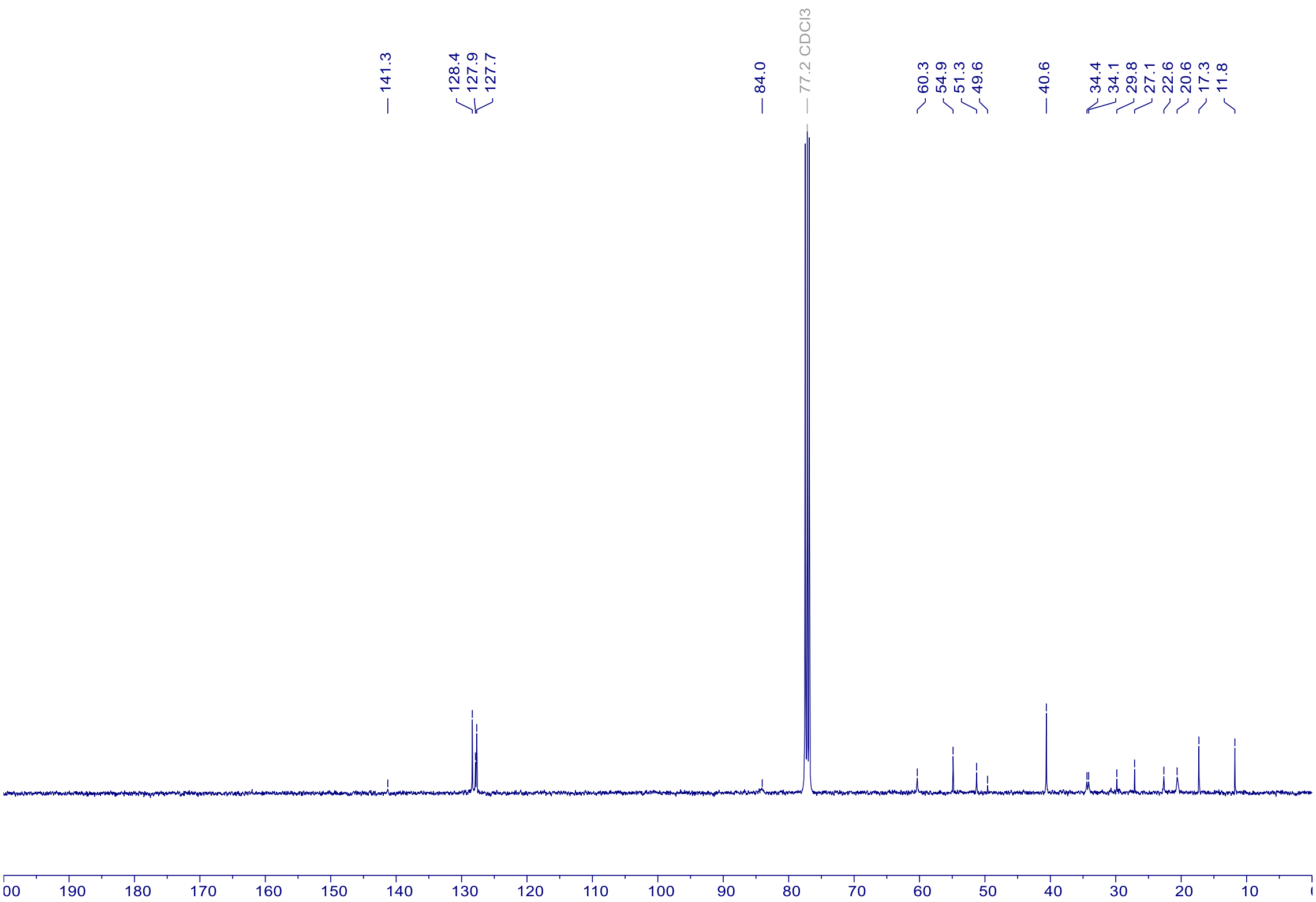

15 References

1 Matlock, J. V. *et al.* Synthesis of α-Substituted Vinylsulfonium Salts and Their Application as Annulation Reagents in the Formation of Epoxide- and Cyclopropane-Fused Heterocycles. *J. Org. Chem.* **79**, 10226-10239 (2014).

2 Lauder, K. *et al.* A facile and regioselective multicomponent synthesis of chiral aryl-1,2-mercaptoamines in water followed by monoamine oxidase (MAO-N) enzymatic resolution. *Org. Biomol. Chem.* **17**, 8982-8986 (2019).

3 Capriati, V., Florio, S., Luisi, R. & Musio, B. Directed Ortho Lithiation of N-Alkylphenylaziridines. *Org. Lett.* **7**, 3749-3752 (2005).

4 Liu, M.-S., Du, H.-W., Cui, J.-F. & Shu, W. Intermolecular Metal-Free Cyclopropanation and Aziridination of Alkenes with XH_2 (X=N, C) by Thianthrenation. *Angew. Chem. Int. Ed.* **61**, e202209929 (2022).

5 Cepanec, I., Litvić, M., Mikuldaš, H., Bartolinčić, A. & Vinković, V. Calcium trifluoromethanesulfonate-catalysed aminolysis of epoxides. *Tetrahedron* **59**, 2435-2439 (2003).

6 Wang, C. *et al.* Synthesis of 1,2-Diamines from Vinyl Sulfonium Salts and Arylamines. *Org. Lett.* **24**, 6455-6459 (2022).

7 Zhang, Z., Górski, B. & Leonori, D. Merging Halogen-Atom Transfer (XAT) and Copper Catalysis for the Modular Suzuki–Miyaura-Type Cross-Coupling of Alkyl Iodides and Organoborons. *J. Am. Chem. Soc.* **144**, 1986-1992 (2022).

8 Jüstel, P. M., Stan, A., Pignot, C. D. & Ofial, A. R. Inherent Reactivity of Spiro-Activated Electrophilic Cyclopropanes. *Chem. Eur. J.* **27**, 15928-15935 (2021).

9 Billacura, M. D. G., Lewis, R. D., Bricklebank, N., Hamilton, A. & Whiteoak, C. J. Ga-Catalyzed Temperature-Dependent Oxazolidinone/Piperazine Synthesis from Phenyl Aziridines Involving a Divergent Ligand-Assisted Mechanism. *Adv. Synth. Catal,* **365**, 3129-3137 (2023).

10 Yang, Z.-Z., He, L.-N., Peng, S.-Y. & Liu, A.-H. Lewis basic ionic liquids-catalyzed synthesis of 5-aryl-2-oxazolidinones from aziridines and CO_2 under solvent-free conditions. *Green Chem.* **12**, 1850-1854 (2010).

11 Tsuchiya, Y., Kumamoto, T. & Ishikawa, T. Guanidines as a Nitrogen Source for Direct Conversion of Epoxides to Aziridines. *J. Org. Chem.* **69**, 8504-8505 (2004).

12 Miniejew, C., Outurquin, F. & Pannecoucke, X. New phenylselanyl group activation: synthesis of aziridines and oxazolidin-2-ones. *Org. Biomol. Chem.* **2**, 1575-1576 (2004).

13 Piotti, M. E. & Alper, H. Inversion of Stereochemistry in the $Co_2(CO)_8$-Catalyzed Carbonylation of Aziridines to β-Lactams. The First Synthesis of Highly Strained trans-Bicyclic β-Lactams. *J. Am. Chem. Soc.* **118**, 111-116 (1996).

14 Watson, I. D. G. & Yudin, A. K. Ring-Opening Reactions of Nonactivated Aziridines Catalyzed by Tris(pentafluorophenyl)borane. *J. Org. Chem.* **68**, 5160-5167 (2003).

15 Azzena, U., Dettori, G., Pisano, L., Musio, B. & Luisi, R. BH_3-Promoted Stereoselective β-Lithiation of *N*-Alkyl-2-phenylaziridines. *J. Org. Chem.* **76**, 2291-2295 (2011).

16 Anderson, W. K. & Milowsky, A. S. Vinylogous carbinolamine tumor inhibitors. 19. Synthesis and antineoplastic activity of bis[[[(alkylamino)carbonyl]oxy]methyl]-substituted 3-pyrrolines as prodrugs of tumor inhibitory pyrrolebis(carbamates). *J. Med. Chem.* **29**, 2241-2249 (1986).

17 Tang, J.-T., Gan, Y., Li, X. & Ye, B. Regioselective reductive transamination of peptidic amides enabled by a dual Zr(IV)–H catalysis. *Chem* **9**, 869-880 (2023).

18 Teo, Y.-C. *et al.* NHC-Silyliumylidene Cation-Catalyzed Hydroboration of Isocyanates with Pinacolborane. *Inorg. Chem.* **62**, 16867-16873 (2023).

19 Yu, Z. *et al.* Ketoester methacrylate resin, secondary amine clean-up in the presence of primary amines. *J. Chem. Soc., Perkin Trans. 1*, 1947-1952 (2001).

20 El-Zoghbi, I., Kebdani, M., Whitehorne, T. J. J. & Schaper, F. Selective and Nonselective Aza-Michael Additions Catalyzed by a Chiral Zirconium Bis-Diketiminate Complex. *Organometallics* **32**, 6986-6995 (2013).

21 McAskill, N. & Sangster, D. Ultraviolet absorption spectra of the benzyl radical formed during pulse radiolysis. *Aust. J. Chem.* **30**, 2107-2113 (1977).

22 Becke, A. D. Density-functional exchange-energy approximation with correct asymptotic behavior. *Phys. Chem. A* **38**, 3098-3100 (1988).

23 Kohn, W., Becke, A. D. & Parr, R. G. Density Functional Theory of Electronic Structure. *J. Phys. Chem.* **100**, 12974-12980 (1996).

24 Neese, F. The ORCA program system. *WIREs Comput Mol Sci* **2**, 73-78 (2012).

25 Zhao, Y. & Truhlar, D. G. The M06 suite of density functionals for main group thermochemistry, thermochemical kinetics, noncovalent interactions, excited states, and transition elements: two new functionals and systematic testing of four M06-class functionals and 12 other functionals. *Theor. Chem. Acc.* **120**, 215-241 (2008).

26 Weigend, F. & Ahlrichs, R. Balanced basis sets of split valence, triple zeta valence and quadruple zeta valence quality for H to Rn: Design and assessment of accuracy. *Phys. Chem. Chem. Phys.* **7**, 3297-3305 (2005).

27 Weigend, F. Accurate Coulomb-fitting basis sets for H to Rn. *Phys. Chem. Chem. Phys.* **8**, 1057-1065 (2006).

28 Rivera-Chao, E., Olivier, W. J., Tilby, M. J. & Leonori, D. Photocatalytic hydrogenation of alkenes using ammonia-borane. *Chem*, 102711 (2025).

29 Zhang, Z. *et al.* Deaminative cross-coupling of amines by boryl radical β-scission. *Nature* **647**, 913-920 (2025).

30 Zhang, L. & Cao, Z. Formation and reactivity of NHC-boryl radicals: insight into substituent effect from theoretical calculations. *Phys. Chem. Chem. Phys.* **25**, 12072-12080 (2023).

31 Kuehn, L. *et al.* NHC induced radical formation via homolytic cleavage of B–B bonds and its role in organic reactions. *Chem. Sci.* **13**, 8321-8333 (2022).

32 Marenich, A. V., Cramer, C. J. & Truhlar, D. G. Universal Solvation Model Based on Solute Electron Density and on a Continuum Model of the Solvent Defined by the Bulk Dielectric Constant and Atomic Surface Tensions. *J. Phys. Chem. B* **113**, 6378-6396 (2009).

33 Gaussian 16 Rev. C.01, Frisch, M. J. *et al.* Gaussian, Inc., Wallingford, CT, 2016.

34 Glendening, E. D., Reed, A. E., Carpenter, J. E. & Weinhold, F. NBO Version 3.1 TCI, University of Wisconish, Madison 1998, 65.

35 Young, T. duartegroup/otherm: Major symmetry improvements. Zenodo: 2020. DOI: 10.5281/zenodo.4005686.

36 Baban, J. A. & Roberts, B. P. An e.s.r. study of the ring opening of 2-methylaziridine-boryl radicals. *J. Chem. Soc., Chem. Commun.*, 850-852 (1984).

37 Chai, Z. Catalytic Asymmetric Transformations of Racemic Aziridines. *Synthesis* **52**, 1738-1750 (2020).

38 Hu, X. E. Nucleophilic ring opening of aziridines. *Tetrahedron* **60**, 2701-2743 (2004).

39 Stanković, S. *et al.* Regioselectivity in the ring opening of non-activated aziridines. *Chem. Soc. Rev.* **41**, 643-665 (2012).

40 Petra, D. G. I., Kamer, P. C. J., Spek, A. L., Schoemaker, H. E. & van Leeuwen, P. W. N. M. Aminosulf(ox)ides as Ligands for Iridium(I)-Catalyzed Asymmetric Transfer Hydrogenation. *J. Org. Chem.* **65**, 3010-3017 (2000).

41 Aggarwal, V. K., Stenson, R. A., Jones, R. V. H., Fieldhouse, R. & Blacker, J. A novel procedure for the synthesis of aziridines: application of Simmons–Smith reagents to aziridination. *Tetrahedron Lett.* **42**, 1587-1589 (2001).

42 Bae, J. H., Shin, S.-H., Park, C. S. & Lee, W. K. Preparation of cysteinol derivatives by highly regioselective ring opening of nonactivated chiral aziridines by thiols. *Tetrahedron* **55**, 10041-10046 (1999).

43 Crousse, B., Narizuka, S., Bonnet-Delpon, D. & Bégué, J.-P. First Stereoselective Synthesis of *cis* 3-CF_3-Aziridine-2-carboxylates. A Route to New (Trifluoromethyl) α-Functionalised β-Amino Acids. *Synlett* **2001**, 0679-0681 (2001).

44 Wu, J., Hou, X.-L. & Dai, L.-X. An efficient procedure for cleavage of aziridines with various thiols promoted by $ZnCl_2$. *J. Chem. Soc., Perkin Trans. 1*, 1314-1317 (2001).

45 Chandrasekhar, M., Sekar, G. & Singh, V. K. An efficient method for opening nonactivated aziridines with TMS azide: application in the synthesis of chiral 1,2-diaminocyclohexane. *Tetrahedron Lett.* **41**, 10079-10083 (2000).

46 Wade, T. N. Preparation of fluoro amines by the reaction of aziridines with hydrogen fluoride in pyridine solution. *J. Org. Chem.* **45**, 5328-5333 (1980).

47 Srivastava, N. & Ha, H.-J. Regioselective ring opening of aziridine for synthesizing azaheterocycle. *Front. Chem.* **11**, :1280633 (2023).

48 Farràs, J. *et al.* β^3-Amino acids by nucleophilic ring-opening of *N*-nosyl aziridines. *Tetrahedron* **57**, 7665-7674 (2001).

49 Wu, J., Hou, X.-L. & Dai, L.-X. Effective Ring-Opening Reaction of Aziridines with Trimethylsilyl Compounds: A Facile Access to β-Amino Acids and 1,2-Diamine Derivatives. *J. Org. Chem.* **65**, 1344-1348 (2000).

50 Matsubara, S., Kodama, T. & Utimoto, K. $Yb(CN)_3$-catalyzed reaction of aziridines with cyanotrimethylsilane. A facile synthesis of optically pure β-amino nitriles. *Tetrahedron Lett.* **31**, 6379-6380 (1990).

51 Yadav, J. S., Subba Reddy, B. V., Parimala, G. & Venkatram Reddy, P. Lithium Perchlorate Catalyzed Regioselective Ring Opening of Aziridines with Sodium Azide and Sodium Cyanide. *Synthesis* **2002**, 2383-2386 (2002).

52 Hayashi, Y., Kumamoto, T., Kawahata, M., Yamaguchi, K. & Ishikawa, T. Ring-opening reaction of unactivated 3-arylaziridine-2-carboxylates with nitrile reagents. *Tetrahedron* **66**, 3836-3841 (2010).

53 Macha, L., Jala, R., Na, S.-Y. & Ha, H.-J. Atom Economical Multi-Substituted Pyrrole Synthesis from Aziridine. *Molecules* **27**, 6869 (2022).

54 Leeson, P. D., Carling, R. W., James, K. & Baker, R. Transannular reactions of 5-azido- and 5-nitronodibenzo[a,e]cyclooctatrienes and -dibenzo[a,d]cycloheptatrienes. Syntheses of pavine and homoisopavine analogs. *J. Org. Chem.* **55**, 2103-2108 (1990).

55 Kim, Y., Ha, H.-J., Yun, S. Y. & Lee, W. K. The preparation of stable aziridinium ions and their ring-openings. *Chem. Commun.*, 4363-4365 (2008).

56 D'Hooghe, M. *et al.* Systematic Study of Halide-Induced Ring Opening of 2-Substituted Aziridinium Salts and Theoretical Rationalization of the Reaction Pathways. *Eur. J. Org. Chem.* **2010**, 4920-4931 (2010).

57 Lee, J., Lee, J. E., Ha, H.-J., Son, S. I. & Lee, W. K. N-Methylative aziridine ring opening: asymmetric synthesis of hygroline, pseudohygroline, and hygrine. *Tetrahedron Lett.* **56**, 856-858 (2015).

58 Jung, J.-H., Kim, S., Eum, H., Lee, W. K. & Ha, H.-J. *N*-methylative aziridine ring opening and the synthesis of (*S*)-3-methylamino-3-[(*R*)-pyrrolidin-3-yl]propanenitrile. *Tetrahedron* **73**, 5993-5999 (2017).

59 Baldwin, J. E. *et al.* The ring opening of aziridine-2-carboxylate esters with organometallic reagents. *J. Chem. Soc., Chem. Commun.*, 1852-1854 (1989).

60 Kim, E., Baik, E., Shin, J., Hwang, J. & Dongbang, S. Aziridines as Versatile C(sp^3) Precursors in Transition-Metal-Catalyzed Cross-Coupling Strategies. *Synthesis* **57**, 1081-1101 (2024).

61 Calet, S., Urso, F. & Alper, H. Enantiospecific and stereospecific rhodium(I)-catalyzed carbonylation and ring expansion of aziridines. Asymmetric synthesis of .beta.-lactams and the kinetic resolution of aziridines. *J. Am. Chem. Soc.* **111**, 931-934 (1989).

62 Xu, S., Hirano, K. & Miura, M. Nickel-Catalyzed Regio- and Stereospecific C–H Coupling of Benzamides with Aziridines. *Org. Lett.* **23**, 5471-5475 (2021).
